# FAIM2 is a potential pan-cancer biomarker for prognosis and immune infiltration

**DOI:** 10.3389/fonc.2022.998336

**Published:** 2022-09-14

**Authors:** Jiayang Cai, Zhang Ye, Yuanyuan Hu, Yixuan Wang, Liguo Ye, Lun Gao, Qian sun, Shiao Tong, Zhiqiang Sun, Ji'an Yang, Qianxue Chen

**Affiliations:** ^1^Department of Neurosurgery, Renmin Hospital of Wuhan University, Wuhan, China; ^2^Central Laboratory, Renmin Hospital of Wuhan University, Wuhan, China; ^3^Department of Ophthalmology, Tongji Hospital, Tongji Medical College, Huazhong University of Science and Technology, Wuhan, China

**Keywords:** pan-cancer, FAIM2, prognosis, immune infiltration, glioma

## Abstract

Fas apoptosis inhibitory molecule 2 (FAIM2) is an important member of the transmembrane BAX inhibitor motif containing (TMBIM) family. However, the role of FAIM2 in tumor prognosis and immune infiltration has rarely been studied. Here, we conducted a pan-cancer analysis to explore the role of FAIM2 in various tumors and further verified the results in glioma through molecular biology experiment. FAIM2 expression and clinical stages in tumor samples and para-cancerous samples were analyzed by TIMER2 database, GEPIA database, and the TISIDB database. The role of FAIM2 on prognosis was analyzed *via* GEPIA2. We utilized the ESTIMATE algorithm to evaluate the ImmuneScore and StromalScore of various tumors. In addition, we explored the correlation between FAIM2 expression and tumor immune cell infiltration by the TIMER2 database. The immune checkpoint genes, tumor mutation burden (TMB), microsatellite instability (MSI), mismatch repair (MMR), and DNA methylation related to FAIM2 were analyzed based on the TCGA database. The correlation between FAIM2 expression with Copy number variations (CNV) and methylation is explored by GSCA database. Protein-Protein Interaction (PPI) analysis was obtained from the STRING database and the CellMiner database was used to explore the association between FAIM2 expression and drug response. FAIM2 co-expression genes were studied by the LinkedOmics database. Immunohistochemistry, Western Blotting Analysis, Cell Viability Assay, Colony Formation Assay, and Edu staining assay were used in the molecular biology experiments section. The FAIM2 expression was down-regulated in most tumors and highly expressed FAIM2 was associated with a better prognosis in several cancers. FAIM2 plays an essential role in the tumor microenvironment and is closely associated with immune Infiltration in various tumors. The expression of FAIM2 was closely correlated to TMB, MSI, MMR, CNV, and DNA methylation. Furthermore, FAIM2 related genes in the PPI network and its co-expression genes in glioma are involved in a large number of immune-related pathways. Molecular biology experiments verified a cancer suppressor role for FAIM2 in glioma. FAIM2 may serve as a potential pan-cancer biomarker for prognosis and immune infiltration, especially in glioma. Moreover, this study might provide a potential target for tumor immunotherapy.

## Introduction

FAIM2 (Fas apoptosis inhibitory molecule 2), also known as Lifeguard (LFG) and transmembrane BAX inhibitor motif containing 2 (TMBIM2), was a protein discovered by Somia NV et al. that can protect uniquely from cell death induced by Fas but not by the tumor necrosis factor-alpha (TNFα) (1). FAIM2 is mainly expressed in the nervous system-associated tissues and is located in the Golgi apparatus, endoplasmic reticulum (ER), and the plasma membrane (2). Studies have shown that FAIM2 plays an important role in maintaining calcium balance in the endoplasmic reticulum, which is one of the reasons for its resistance to FAS-mediated apoptosis (3). Then, FAIM2 anti-apoptotic activity has been found to have a role in certain types of cancer, such as non-small cell lung cancer, hepatocellular carcinoma and breast carcinoma (4–6). Because of these factors, FAIM2 was identified as an oncogenic role; however, Planells-Ferrer L et al. have revealed it as a tumor suppressor in neuroblastoma (7). To systematically investigate the role of FAIM2 in tumors, we conducted a pan-cancer analysis of it using large-scale RNA-sequencing (RNA-seq) data from various public databases.

Evading immune destruction is the emerging hallmark of cancer (8), which is becoming one of the targets of immunotherapies (9). In recent years, there are increasing research on the tumor immune microenvironment, and immunotherapy including immune checkpoint inhibitors has achieved great success in clinical cancer treatment (10, 11). Moreover, the great success of immunotherapy has endowed the search for novel cancer immune and prognostic biomarkers with a crucial role. Therefore, it is the right time to analyze the correlation and effect of a novel biomarker on cancer prognosis and immune infiltration.

In this study, the role of FAIM2 in prognosis and immune infiltration in human cancers was comprehensively analyzed. We analyzed the correlation between FAIM2 expression and patient prognosis and clinical grade. In addition, we further explored the potential association between FAIM2 expression and tumor infiltrating immune cells (TIICs), immune subtypes, molecular subtypes, and promising immune biomarkers in the tumor microenvironment (TME). Our results revealed that FAIM2 is downregulated in most tumors, influences the prognosis of various kinds of cancer patients, and is associated with various tumor grades. FAIM2 expression is associated with tumor immune infiltration and may be able to modulate tumor prognosis by interacting with tumor immune cell infiltration. Furthermore, we conducted molecular biology experiments in glioma further verified the tumor suppressor role of FAIM2. Taking these facts together, FAIM2 is a promising and prospective therapeutic target for cancer, as well as a marker for both clinical outcomes and immune infiltration.

## Material and methods

### Gene expression analysis of FAIM2

Tumor immune estimation resource, version 2 (TIMER2) database (http://timer.cistrome.org/) was used to explore the differences in FAIM2 expression levels between tumor and normal tissues in The Cancer Genome Atlas (TCGA). Expression difference between tumor and matched TCGA normal and GTEx data of FAIM2 was analyzed by the gene expression profiling interactive analysis (GEPIA, http://gepia2.cancer-pku.cn/) (12).

### Survival analysis and relationship with clinical stage

Survival Analysis was conducted by GEPIA “Survival” module. The box plots of the FAIM2 expression in different pathological stages and grades of 33 kinds of tumors were conducted by TISIDB database (http://cis.hku.hk/TISIDB/), a web portal for tumor and immune system interaction, which integrates multiple heterogeneous data types.

### Immune infiltration analysis of FAIM2

The relationship between FAIM2 expression and immune and stromal scores was assessed by R software (version: 4.2.0) packages “estimate” and “limma”. “Immune-Gene” module of TIMER2 database was used to explore the purity of tumors and the differences of immune cell Infiltration, algorithm including XCELL, TIMER, QUANTISEQ, MCPCOUNTER, EPIC, CIBERSORT-ABS, CIBERSORT, TIDE. Spearman’s rank correlation test was used to calculate p-values and partial correlation values. The relationships between FAIM2 expression and expression levels of the immune checkpoint markers including BLTA, CD200, TNFRSF14, NRP1, LAIR1, TNFSF4, CD244, LAG3, ICOS, CD40LG, CTLA4, CD48, CD28, CD200R1, HAVCR2, ADORA2A, CD276, KIR3DL1, CD80, PDCD1, LGALS9, CD160, TNFSF14, IDO2, ICOSLG, TMIGD2, VTCN1, IDO1, PDCD1LG2, HHLA2, TNFSF18, BTNL2, CD70, TNFSF9, TNFRSF8, CD27, TNFRSF25, VSIR, TNFRSF4, CD40, TNFRSF18, TNFSF15, TIGIT, CD274, CD86, CD44, and TNFRSF9 was evaluated by Spearman’s correlation analysis. Relations between the expression of FAIM2 and three kinds of immunomodulators including immunoinhibitory, immunostimulatory, and MHC molecules were analyzed by the TISIDE database. In addition, the TISIDB database was also used to explore the correlations between FAIM2 expression and immune or molecular subtypes of 33 cancer types.

The association between FAIM2 expression and tumor mutational burden (TMB) or microsatellite instability (MSI) in 33 kinds of tumors was analyzed by Spearman’s method. The somatic mutation dataset and MSI score were obtained from the TCGA database.

### Analysis of FAIM2 expression and MMR gene mutation and DNA methylation

The expression levels of mismatch repair (MMR) genes including EPCAM, PMS2, MSH6, MSH2, and MLH1 in 33 kinds of cancers were obtained from the TCGA database. Similarly, the expression levels of DNA methylation related genes including DNMT1, DNMT3L, DNMT3A, and DNMT3B were obtained from the TCGA database. Spearman’s correlation method was used to analyze the correlation of expression levels between FAIM2 and MMR genes and DNA methylation related genes.

### FAIM2 methylation profile and CNV profile in pan-cancer based on GSCA

Gene Set Cancer Analysis (GSCA) is an integrated platform (http://bioinfo.life.hust.edu.cn/GSCA/) (13) for genomic, pharmacogenomic, and immunogenomic gene set cancer analysis. The correlation between FAIM2 expression levels and Methylation and Copy number variation (CNV) in 33 kinds of tumors was analyzed by the GSCA platform.

### PPI network construction and gene enrichment analysis

STRING (Version: 11.5) (https://www.string-db.org/) database was used to construct the protein–protein interaction (PPI) network. Interactions with medium confidence of interaction scores over 0.7 (High confidence) were considered statistically significant. KEGG Orthology Based Annotation System (KOBAS) (http://kobas.cbi.pku.edu.cn) was used to conduct Kyoto Encyclopedia of Genes and Genomes (KEGG) and Gene Ontology (GO) analyses. FAIM2 high- and the low-expression groups in 33 kinds of cancers were used to explore the biological signaling pathway *via* Gene Set Enrichment Analysis (GSEA). The top 5 terms of KEGG analyses were exhibited. Gene sets with p < 0.05 were considered to be enrichment significant.

### Drug sensitivity of FAIM2 in pan-cancer

The data of RNA-seq expression profiles and DTP NCI-60 compound activity data were downloaded from CellMiner (http://discover.nci.nih.gov/cellminer/) to analyze the drug sensitivity of FAIM2 in 33 kinds of cancers. Then we chose drugs approved by FDA or clinical trials to further analyze the correlation between FAIM2 expression and drug sensitivity through R package “impute”, “limma”, “ggplot2”, and “ggpubr”.

### Database used to explore FAIM2 co-expression genes in glioma

The LinkedOmics database (http://www.linkedomics.org/) (14) was used to explore the FAIM2 correlated gene by using Spearman’s correlation coefficient. The FAIM2 co-expression genes were represented by volcano plot and heatmap. Then, we explored the Gene Ontology biological process (GO_BP), and KEGG pathways of FAIM2 and its co-expression genes by using the gene set enrichment analysis (GSEA) module in the LinkedOmics database.

### Human glioma and control brain tissues

All glioma (including GBM and LGG) and control brain tissues used in this study were obtained from the Department of Neurosurgery, Renmin Hospital of Wuhan University, China, after obtaining written patient informed consent from the patients. Tumor tissue specimens were collected from surgical resection and control brain tissues were collected from patients with traumatic brain injury during emergency surgeries. All glioma specimens had a confirmed pathological diagnosis by pathologists at Renmin Hospital of Wuhan University. Institutional Ethics Committee of the Faculty of Medicine, Renmin Hospital Affiliated to Wuhan University approved the procurement and rational use of specimens in this study (approval number: 2012LKSZ (010) H).

### Immunohistochemistry

The brain tissues were embedded in paraffin after being immobilized in formalin and cut into slices. After deparaffinized and hydrating the tissues, 10 mM sodium citrate (pH, 6.0) was used for antigen retrieval. The sections were incubated with 3% H_2_O_2_ for 10 min and blocked with serum for 1 h. The primary antibodies (anti-FAIM2, PH6298, Abmart) were incubated with the tissues at 4°C overnight and then the secondary antibody was incubated with them at room temperature for 1 h. Finally, the tissues were stained with DAB (G1212-200T, Servicebio), followed by hematoxylin counterstaining. We used an Olympus BX51 microscope (Olympus) to visualize the images.

### Cell culture

We purchased human glioma cell lines (U87 and U251) from the Cell Bank of the Shanghai Institute of Biochemistry and Cell Biology (Shanghai, China). High-glucose DMEM (GNM12800, Genom, Hangzhou, China) supplemented with 1% penicillin/streptomycin (BL505A, Biosharp, Anhui, China) and 10% foetal bovine serum (16140071, Thermo Fisher Scientific) were used to culture the cells in incubator with temperature at 37°C and 5% CO2.

### Stable cell lines establishment

The FAIM2 overexpression plasmids with GFP-tag were transfected with pMD2.G and psPAX2 into 293T cells to produce lentiviruses. And U87 and U251 cells were infected by lentivirus according to the manufacturer’s instruction. Twenty-four hours after infection, the virus-infected cells were cultured in the medium containing puromycin (2 ug/ml) for selection. The surviving cells were used in the subsequent experiments. The overexpression efficacies were verified by Western blot.

### Western blotting analysis

Lysis in RIPA buffer containing PMSF (ST506, Beyotime) for 20 min, cell samples were collected at 4°C. Polyacrylamide gel electrophoresis was used to separate the equal amounts of protein and the resulting proteins were transferred to PVDF membranes (IPFL00010, Millipore, Germany). Membranes were blocked and incubated with specific primary antibodies overnight at 4°C and then incubated in corresponding secondary antibodies for 1 h at room temperature. ChemiDoc Touch (Bio-Rad, USA) was used to scan the imaging. Finally, ImageJ software (Version: 1.53e, National Institutes of Health, Bethesda, MD, USA) was used to quantify the band intensity.

### Cell viability assay and colony formation assay

Following the manufacturer’s instructions (Topscience, Shanghai, China), a CCK-8 cell counting kit (C0005, CCK-8, Topscience) was used to detect the cell viability. For colony formation assay, we seeded 500 glioma cells per well in 6-well plates with complete medium, there is obvious single-cell colony formation after about two weeks. A camera was used to take a photo of each well and ImageJ software was used to count the cell colonies.

### Edu staining assay

Following the manufacturer’s instructions, an EdU Cell Proliferation Kit with Alexa Fluor 555 (MA0425, Meilun, Dalian, China) was used to detect the proliferating cells. A fluorescence microscope (Olympus BX51, Japan) was used to capture the picture of the cell.

### Statistical analysis

The statistical analysis of bioinformatic validation has been described above. For molecular biology verification, GraphPad Prism 8 was used for the statistical analysis. Two groups of means were compared using unpaired t-tests. All experimental results are reported as means ± SD and were repeated at least three times. *P <0.05, **P <0.01, and ***P <0.001 were regarded as significant.

## Results

### Analysis of gene expression in various normal and cancer tissues

**Figure 1:**
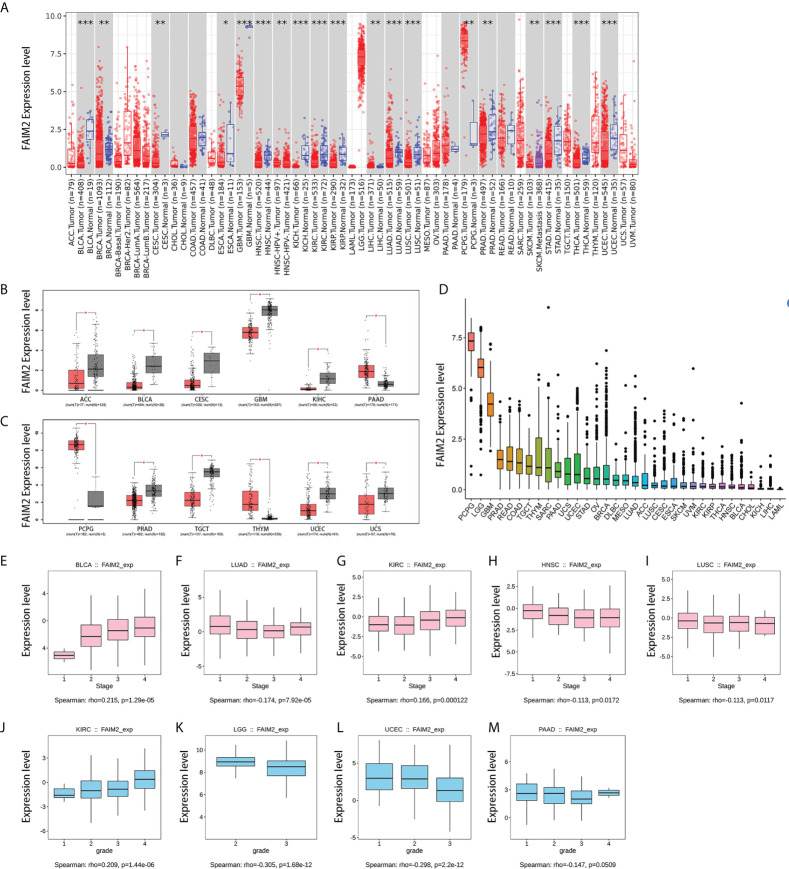
7The expression level of FAIM2 in different tumors and pathological stages or grades. **(A)** The expression level of the FAIM2 gene in different cancers or specific cancer subtypes was analyzed through TIMER2. **(B, C)** FAIM2 expression in several cancers and paired normal tissue in the GEPIA database. **(D)** FAIM2 Expression level in different tumors. **(E-M)** The correlation between FAIM2 expression and cancer stages or grades was analyzed by the TISIDE database. *p < 0.05; **p < 0.01; ***p < 0.001.

The expression levels of FAIM2 are lower in most tumors than in corresponding normal tissues through timer 2 database, including bladder urothelial carcinoma (BLCA), breast invasive carcinoma (BRCA), cervical squamous cell carcinoma and endocervical adenocarcinoma (CESC), esophageal carcinoma (ESCA), glioblastoma multiforme (GBM), head and neck squamous cell carcinoma (HNSC), kidney chromophobe (KICH), kidney renal clear cell carcinoma (KIRC), kidney renal papillary cell carcinoma (KIRP), lung adenocarcinoma (LUAD), lung squamous cell carcinoma (LUSC), prostate adenocarcinoma (PRAD), stomach adenocarcinoma (STAD), thyroid carcinoma (THCA), uterine corpus endometrial carcinoma (UCEC) (**Figure 1A**). On the contrary, the expression levels of FAIM2 are higher in liver hepatocellular carcinoma (LIHC) and pheochromocytoma and paraganglioma (PCPG) than in normal tissues (**Figure 1A**). The GEPIA platform was used to match the TCGA data with the normal tissue of the GTEx database, and the analysis showed that compared with the previous results, FAIM2 mRNA expression was also significantly low in adrenocortical carcinoma (ACC), testicular germ cell tumors (TGCT), and uterine varcinosarcoma (UCS) tumor tissues while thymoma (THYM) was high expression in tumor tissues (**Figure 1B, C**). The mRNA expression levels of FAIM2 in 33 kinds of cancers were shown in **Figure 1D** and PCPG, brain lower grade glioma (LGG), and GBM have a relatively high expression. We also showed that the expression levels of FAIM2 was related to the grade and stage of various tumors by multiple analysis methods (**Figures 1E-M** and **S1**).

### Analysis of survival prognosis

**Figure 2:**
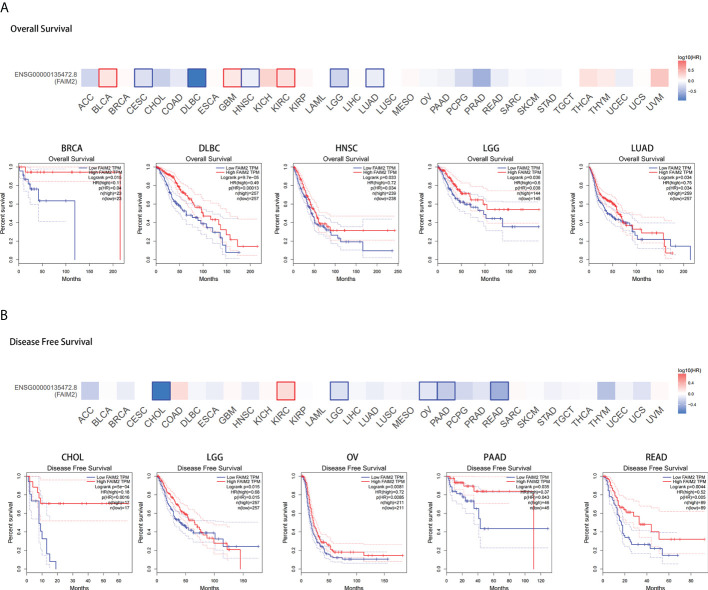
Correlation between FAIM2 expression and TCGA tumor survival prognosis. Overall survival **(A)** and disease-free survival **(B)** of different tumors in TCGA were analyzed by FAIM2 gene expression using GEPIA2 database. Survival plots and Kaplan-Meier curves for positive outcomes are given.

Overall survival analysis revealed that high expressed FAIM2 is linked to better prognosis for cancers including BRCA, lymphoid neoplasm diffuse large B-cell lymphoma (DLBC), HNSC, LGG, and LUAD of TCGA datasets, while there is an opposite trend in BLCA, GBM, and KIRC (**Figures 2A** and **S2A-C**). Disease free survival analysis shows high FAIM2 expression has a better prognosis in cholangio carcinoma (CHOL), LGG, ovarian serous cystadenocarcinoma (OV), pancreatic adenocarcinoma (PAAD), and rectum adenocarcinoma (READ) and has a poor prognosis in KIRC (**Figures 2B** and **S2D**). These results indicate that FAIM2 expression is associated with the prognosis of cases with different tumors.

### Analysis of immune infiltrating level in cancers

**Figure 3:**
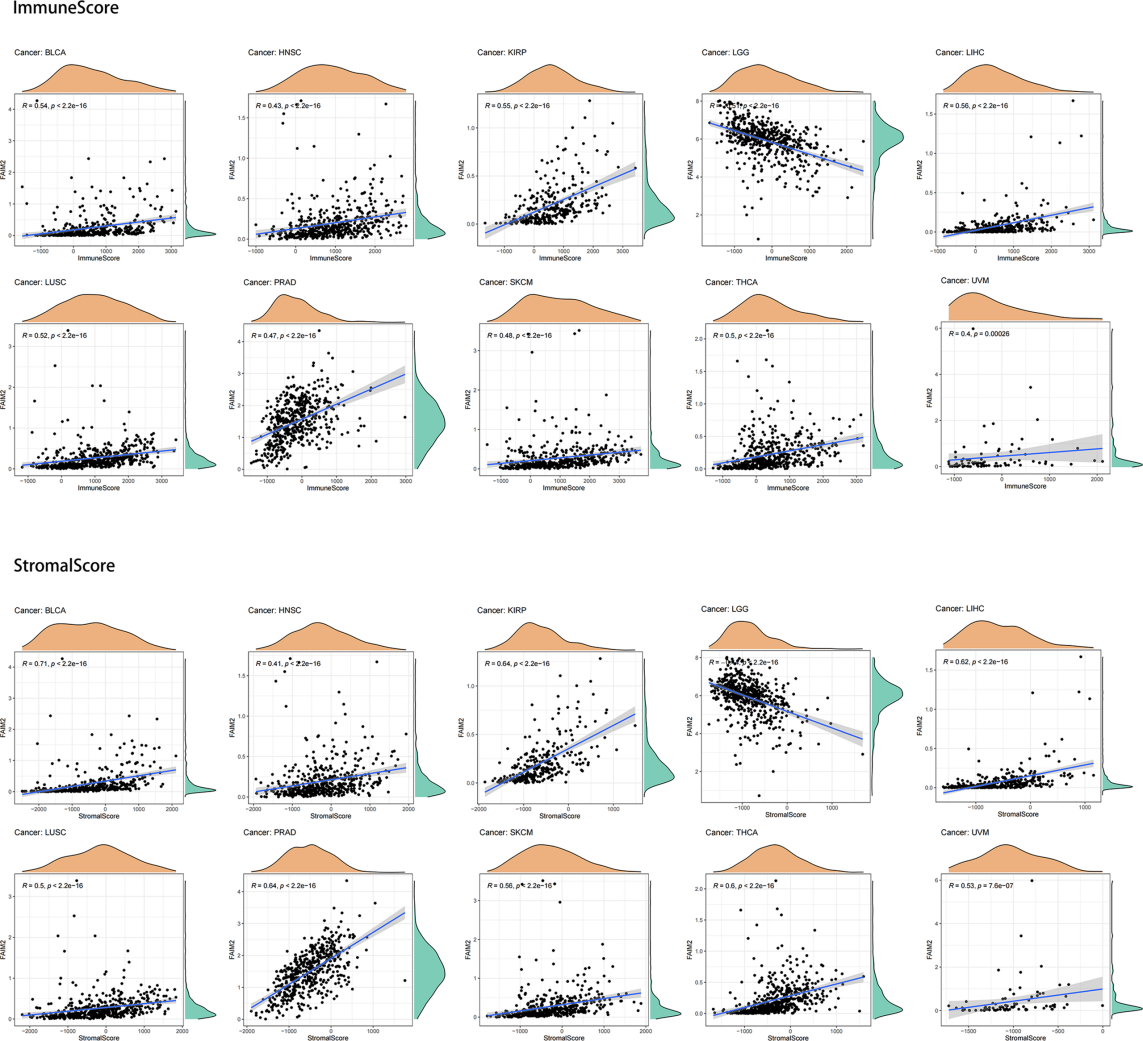
Correlation of FAIM2 expression with ImmuneScore and StromalScore in the top 10 cancers.

**Figure 4:**
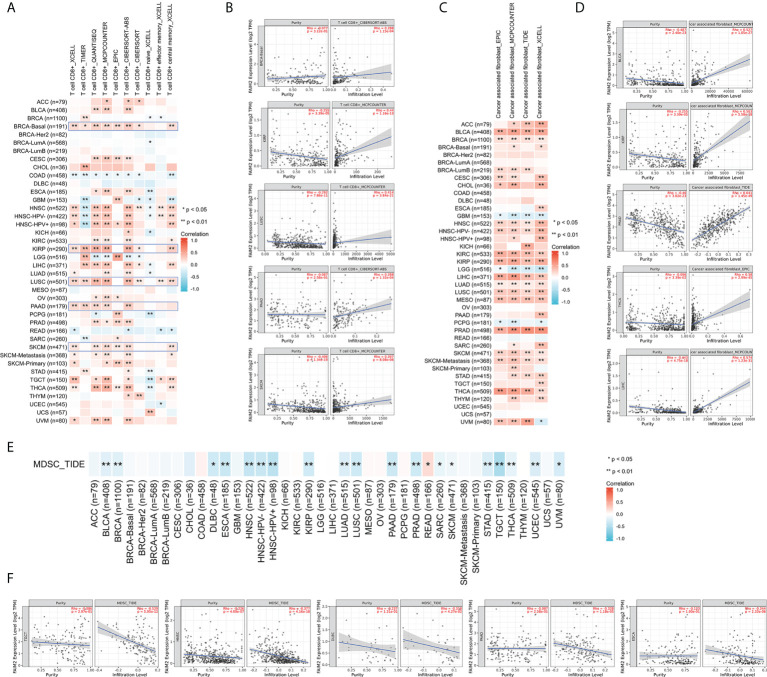
Different algorithms were used to explore potential correlations between FAIM2 expression levels and immune infiltration of CD8+ T cell **(A, B)**, cancer-associated fibroblasts **(C, D)**, and MDSC **(E, F)** infiltration levels in 33 types of cancer in TCGA. *p < 0.05; **p < 0.01.

First, we evaluated the expression and immune infiltration across the relationship between FAIM2 expression and ImmuneScore and StromalScore. The top 10 most significantly correlated with the expression of FAIM2 were shown in **Figure 3**. In various tumors including BLCA, HNSC, KIRP, LIHC, LUSC, PRAD, skin cutaneous melanoma (SKCM), THCA, and uveal melanoma (UVM), FAIM2 expression was positively correlated with ImmuneScore and StromalScore. While in LGG, FAIM2 expression was strongly negatively correlated with the Score. Furthermore, we explored the relationship between FAIM2 expression and immune cell infiltration through various algorithms of the TIMER2 database. The results showed that FAIM2 expression is statistically positively correlated with the infiltration level of CD8+ T cells in most tumors of TCGA (**Figure 4A**). For example, the FAIM2 expression level is positively correlated with the infiltration level of CD8+ T cells based on the CIBERSORT-ABS and MCPCOUNTER algorithm in BRCA-Basal, KIRP, LUSC, PAAD, and SKCM (**Figure 4B**). Interestingly, besides LGG, GBM, and PCPG, there is a positive connection exists between FAIM2 expression and cancer-associated fibroblast (CAF) in most tumors through 4 kinds of algorithms (**Figure 4C**), and the top 5 significantly correlated with expression of FAIM2 were shown in **Figure 4D**. On the contrary, the expression level of FAIM2 was negatively correlated with myeloid-derived suppressor cell (MDSC) in almost all tumors through TIDE algorithms (**Figure 4E**) and **Figure 4F** shows the five kinds of tumors with the strongest correlations. All these results indicated that FAIM2 expression was significantly correlated with the immune infiltration in cancers.

### Analysis the correlation of FAIM2 expression with immunomodulators and immune checkpoint genes

**Figure 5:**
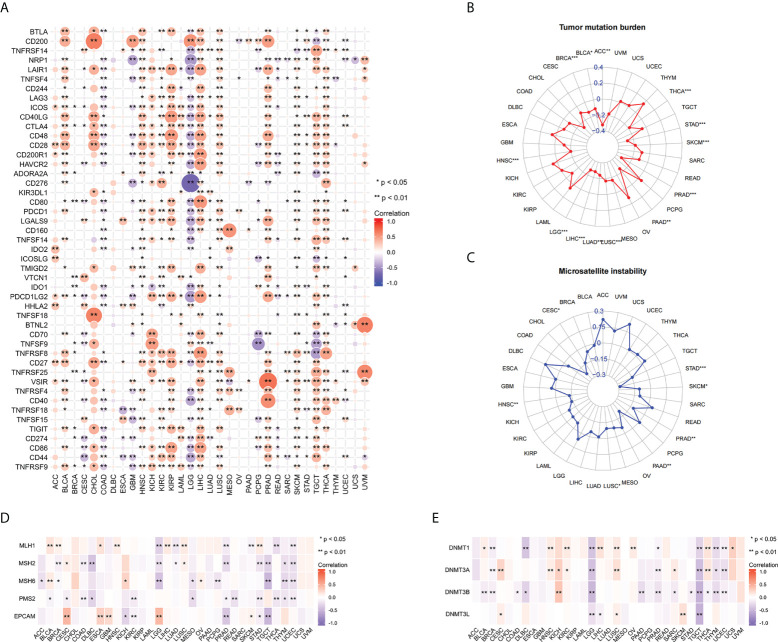
Correlations between FAIM2 expression and immune checkpoint genes, TMB, MSI, MMR, and DNA methyltransferase genes. Heat map of Correlations between FAIM2 expression and immune checkpoint genes **(A)**. Radar map of correlation between FAIM2 expression and TMB **(B)** and MSI **(C)**. The value in concentric circles reveals the range, and the curve reveals the correlation coefficient. *p < 0.05; **p < 0.01; ***p < 0.001. Correlation heat map of FAIM2 expression and MMR **(D)** and DNA methyltransferase genes **(E)**.

To further explore the relationship between FAIM2 and immunity, we explored the relations between three kinds of immunomodulators and the expression of FAIM2 *via* the TISIDE database. Our results found that there is a positive connection exists between the expression of FAIM2 and immunomodulators including Immunostimulator, Immunoinhibitor, and MHC molecules (**Figures S3A-C**). Next, the correlation between FAIM2 and immune checkpoint gene expression was analyzed. The results revealed that FAIM2 expression is positively correlated with most immune checkpoint genes in majority of 33 kinds of tumors (**Figure 5A**). Notably, FAIM2 expression is inversely correlated with most immune checkpoint genes in colon adenocarcinoma (COAD), GBM and LGG.

### Analysis the correlation of FAIM2 expression with the TMB, MSI, MMR gene, and DNA methylation

In all statistically significant results, FAIM2 expression levels were negatively correlated with both TMB and MSI. FAIM2 expression is negatively associated with TMB in ACC, THCA, SKCM, PRAD, PAAD, LUSC, LUAD, LIHC, LGG, HNSC, BRCA, and BLCA (**Figure 5B**). Similarly, there is a negative correlation exists between FAIM2 expression and MSI in STAD, SKCM, PRAD, PAAD, LUSC, HNSC, and CESC (**Figure 5C**). These results indicate that FAIM2 expression is widely associated with immunity in cancers. Next, we further explore the relationship between FAIM2 expression and carcinogenesis processes, particularly a link with MMR deficiencies and DNA Methylation. As a consequence, in most of 33 cancer types, FAIM2 expression negatively and strongly correlates with MMR gene (MLH1, MSH2, MSH6, PMS2 and EPCAM) expression especially MSH2 and PMS2 (**Figure 5D**). Meanwhile, FAIM2 expression is highly associated with these four DNA methyltransferases in multiple cancers, and especially in LGG and TGCT, FAIM2 expression was strongly negatively correlated with these four methyltransferases. (**Figure 5E**).

### Analysis the association of FAIM2 with molecular and immune subtypes

**Figure 6:**
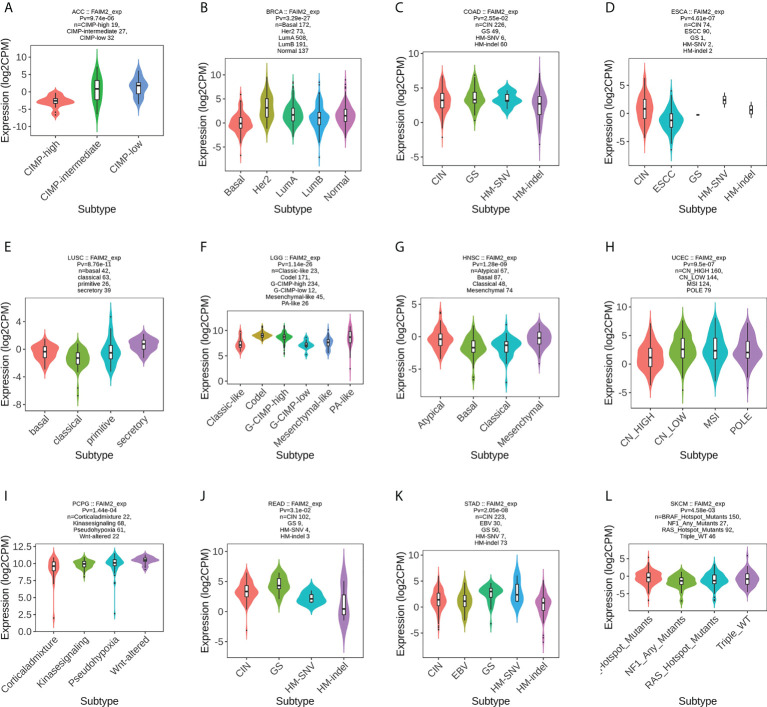
The relationship between FAIM2 expression and pan-cancer molecular subtypes. **(A)** in ACC, **(B)** in BRCA, **(C)** in COAD, **(D)** in ESCA, **(E)** in LUSC, **(F)** in LGG, **(G)** in HNSC, **(H)** in UCEC, **(I)** in PCPG, **(J)** in READ, **(K)** in STAD, **(L)** in SKCM.

**Figure 7:**
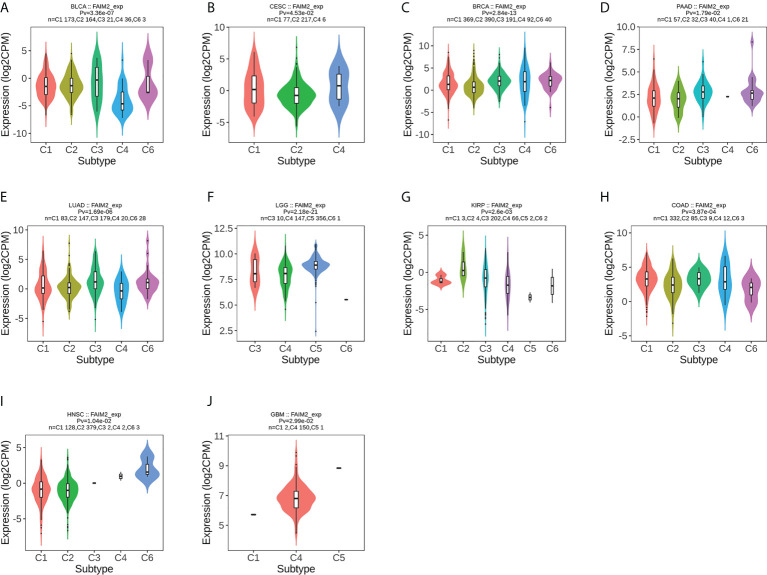
The relationship between FAIM2 expression and pan-cancer immune subtypes. **(A)** in BLCA, **(B)** in CESC, **(C)** in BRCA, **(D)** in PAAD, **(E)** in LUAD, **(F)** in LGG, **(G)** in KIRP, **(H)** in COAD, **(I)** inHNSC, **(J)** in GBM.

We used the TISIDB database to analyze the association of FAIM2 with immune and molecular subtypes. The results showed that there is a significant connection between FAIM2 expression and different molecular subtypes in ACC, BRCA, COAD, ESCA, LUSC, LGG, HNSC, UCEC, PCPG, READ, STAD, and SKCM (**Figure 6**). Immune subtypes were classified into six types, including C1 (wound healing), C2 (IFN-gamma dominant), C3 (inflammatory), C4 (lymphocyte depleted), C5(immunologically quiet), and C6 (TGF-b dominant). FAIM2 expression was related to different immune subtypes in BLCA, CESC, BRCA, PAAD, LUAD, LGG, KIRP, COAD, HNSC, and GBM (**Figure 7**). All these results indicated that FAIM2 expression differs in molecular subtypes and immune subtypes of various human cancer types.

### Analysis the FAIM2 CNV and methylation based on GSCA

**Figure 8:**
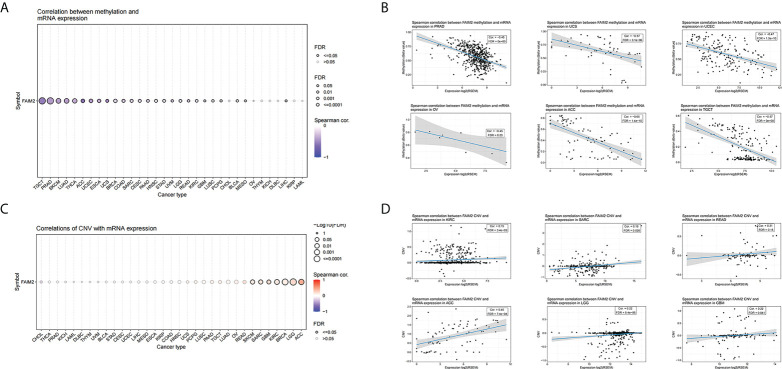
Correlations between FAIM2 mRNA expression and CNV and Methylation based on GSCA. **(A)** A Spearman association between FAIM2 methylation and mRNA expression was performed in pan-cancer. **(B)** The top six with the highest correlation scores between FAIM2 methylation and mRNA. **(C)** A Spearman association between FAIM2 CNV and mRNA expression was performed in pan-cancer. **(D)** The top six with the highest correlation scores between FAIM2 CNV and mRNA expression.

Based on GSCA, we conducted a Spearman correlation analysis between FAIM2 mRNA expression and methylation levels. Our results showed that FAIM2 methylation was shown to be strongly linked with FAIM2 mRNA expression in most cancer types (**Figure 8A**) and the top six with the highest correlation scores were shown in **Figure 8B**. Meanwhile, the association between FAIM2 CNV and mRNA was explored by Spearman correlation analysis. There is a statistically positive connection between FAIM2 CNV and mRNA expression in ACC, LGG, BRCA, KIRC, GBM, SARC, and SKCM (**Figure 8C**). **Figure 8D** show the top six with the highest correlation scores.

### Analysis the PPI network of FAIM2 and enrichment analysis

Through the STRING tool, a total of 10 FAIM2-binding proteins were obtained (**Figure S4A**). Next, we conduct a KEGG and GO enrichment analysis of them. KEGG analysis showed that these genes are mainly involved in Allograft rejection, p53 signaling pathway, TNF signaling pathway, Natural killer cell mediated cytotoxicity, and other pathways (**Figure S4B**). GO enrichment analysis indicates that these genes are mainly related to protein binding, negative regulation of extrinsic apoptotic signaling pathway *via* death domain receptors, melanocortin receptor activity, CD95 death-inducing signaling complex, regulation of stress-activated MAPK cascade, and tumor necrosis factor-activated receptor activity (**Figure S4C**). Furthermore, we conduct a GSEA enrichment analysis between the FAIM2 high expression group and FAIM2 low expression group in 33 kinds of cancers, and most results of them were involved in immunity (**Figure S5**).

### Analysis the FAIM2 expression and drug response

**Figure 9:**
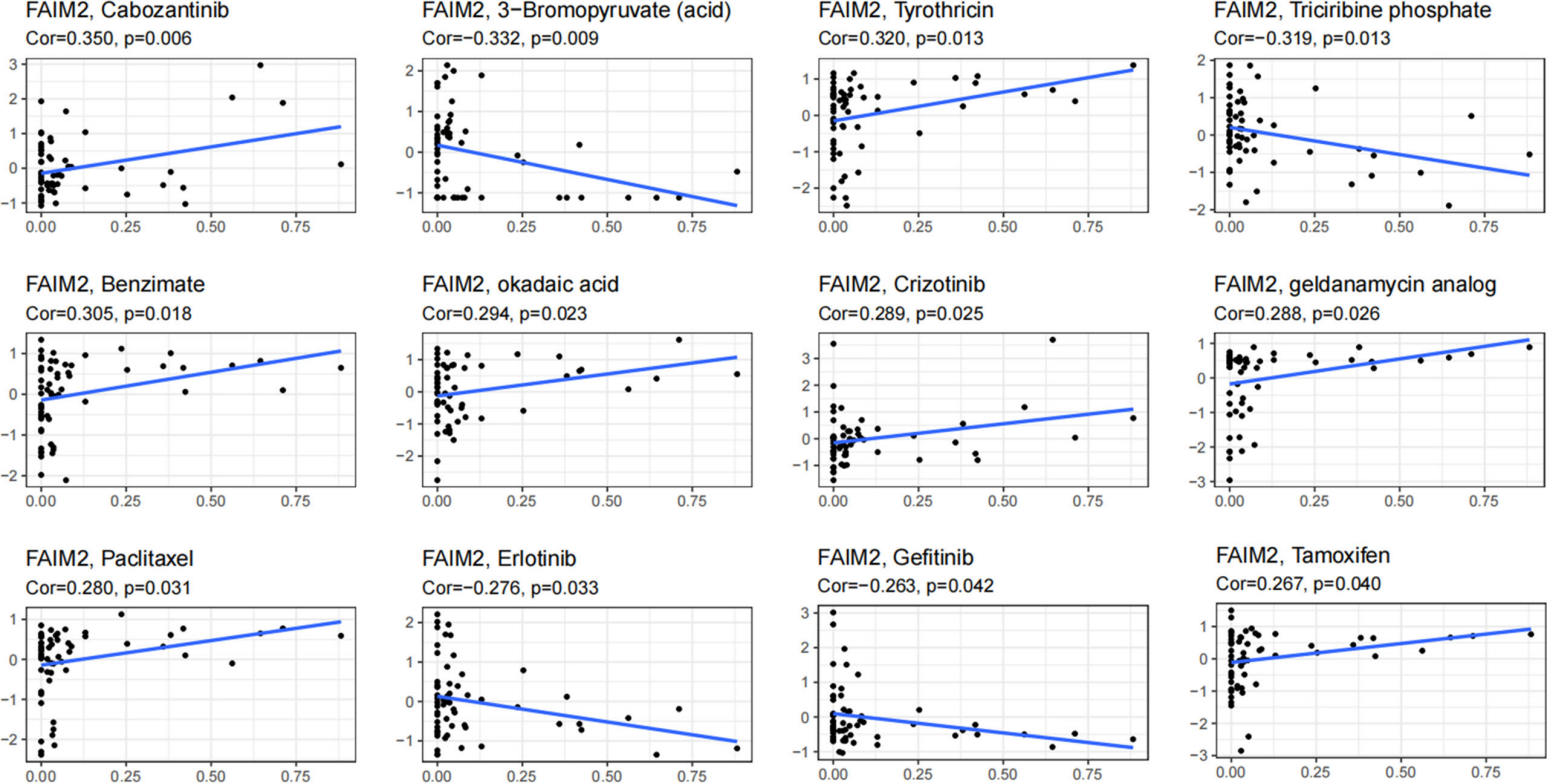
An illustration of the relationship between FAIM2 expression and expected medication response.

Through the CellMiner database, we found that FAIM2 expression was positively related to drug response in patients who received the treatment of Cabozantinib, Tyrothricin, Benzimate, okadaic acid, Crizotinib, geldanamycin analog, Paclitaxel, and Tamoxifen (**Figure 9**). On the contrary, there is a negative connection between FAIM2 expression and the anticancer drug including 3-Bromopyruvate (acid), Triciribine phosphate, Erlotinib, and Gefitinib (**Figure 9**). These results indicated that FAIM2 may be a marker for drug response in various cancer types.

### Analysis the FAIM2 co-expression genes in glioma

**Figure 10:**
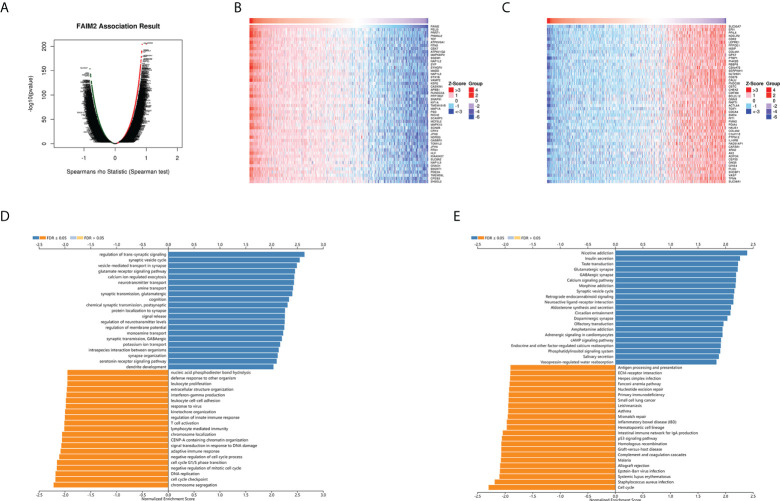
FAIM2 co-expression genes in glioma were analyzed by the LinkedOmics database. **(A)** A Volcano plot showing co-expression genes based on Spearman correlation coefficient in glioma cohort. The heat map of top 50 positive co-expression genes **(B)** and negative co-expression genes **(C)** of FAIM2 in glioma. Bar chart of FAIM2 GO analysis (biological process) **(D)** and KEGG pathways **(E)** in glioma cohort.

The above results identified that FAIM2 had a significant association with the prognosis and immunity of cancers. Next, we explored FAIM2 co-expression genes in Glioma using the LinkedOmics database to verify the potential function of FAIM2 in glioma. In glioma, the co-expression gene of FAIM2 were shown in a volcano plot (**Figure 10A**) and the top 50 genes positively and negatively correlated with FAIM2 were displayed in the heat map (**Figure 10B, C**). Next, we used the GSEA module of LinkedOmics database to explore the main GO (biological process) terms of FAIM2 co-expression genes. The results show these genes are mainly involved in cell cycle checkpoint, chromosome localization, adaptive immune response, T cell activation, regulation of innate immune response, lymphocyte mediated immunity, and leukocyte proliferation (**Figure 10D**). KEGG analysis results revealed that these genes main enriched in cell cycle, p53 signaling pathway, mismatch repair (MMR), primary immunodeficiency, and antigen processing and presentation (**Figure 10E**). All these results indicated that FAIM2 might play an essential role in regulating the immune response of the TME in glioma.

### Analysis the expression of FAIM2 in glioma

**Figure 11:**
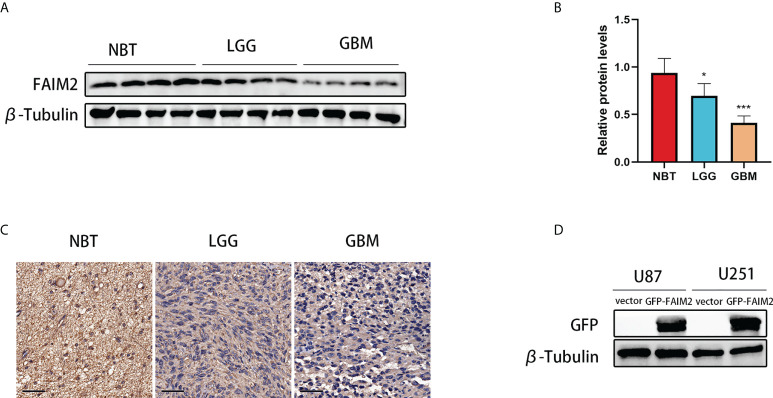
The expression level of FAIM2 in glioma. **(A, B)** Western blotting was used to detect FAIM2 protein levels in normal brain tissues (NBT) (n=4), LGG tissues (n=4), and GBM tissues (n=4). **(C)** IHC staining images for FAIM2 in clinical human normal brain, LGG and GBM tissues. Scale bars, 50 μm. NBT: n = 4, LGG: n=4, GBM: n=4. **(D)** The efficacy of GFP-FAIM2 overexpression was checked by western blot analysis. *p < 0.05; ***p < 0.001.

Glioma and control normal brain tissues were used to identify the expression level of FAIM2. Western blotting showed that the expression level of FAIM2 was down-regulated in glioma (including LGG and GBM) tissues (**Figures 11A, B**). Similarly, the results of IHC were consistent with Western blotting (**Figures 11C** and **S6**).

### Analysis the effect of FAIM2 on proliferation of glioma

**Figure 12:**
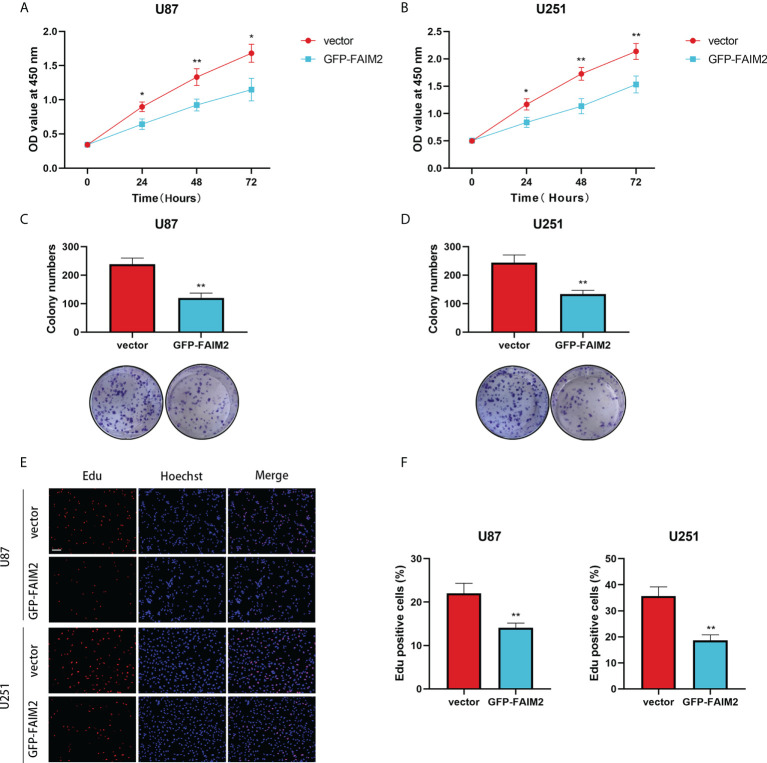
The Effect of FAIM2 on Proliferation of glioma. **(A, B)** CCK-8 assays were used to measure the viability of U87 and U251 cells. **(C, D)** FAIM2 overexpression inhibited U87 and U251 cell colony formation. **(E, F)** EdU assays were used to evaluate cell proliferation. Representative images are shown in **(E)** Scale bars: 50 μm. *p < 0.05; **p < 0.01.

To detect the effect of FAIM2 in glioma, we constructed stable FAIM2 glioma cell lines including U87 and U251. The overexpression efficacies were checked by western blot (**Figure 11D**). Cell viability assay showed that the overexpression of FAIM2 lead to lower cell viability in both U87 and U251 cells (**Figures 12A, B**). Colony formation assay revealed that the group of GFP-FAIM2 has significantly fewer and smaller colonies (**Figures 12C, D**). Similarly, in the Edu staining assay, the number of EdU-positive cells in the GFP-FAIM2 group was significantly less than that in the vector group (**Figures 12E, F**). All these results demonstrated that FAIM2 overexpression can inhibit the proliferation of glioma cells.

## Disscussion

FAIM2, also known as LFG and TMBIM2, is a member of the evolutionarily conserved TMBIM superfamily. All six members (TMBIM1-6) of the TMBIM family have reported antiapoptotic functions and are generally thought of as tumor promoters (15). Although these proteins were well documented in their role of inhibitors of cell death, the possibility of promoting cell death still exists (16). For example, TMBIM1 and FAIM2 (TMBIM2) are thought to be inhibitors of the external pathways of apoptosis, but a recent study suggests that TMBIM1 has a function as a pro-apoptotic component of the TMBIM family, initiating the cell death program in lysosomes (17). Similarly, the role of FAIM2 in tumors is still unclear. In the current study, FAIM2 acted as a tumor promoter in three tumor types and a tumor suppressor in one tumor type. To systematically explore the role of FAIM2 in tumors, we performed a pan-cancer analysis.

In this study, we conducted a comprehensive pan-cancer analysis and thoroughly investigated the role of FAIM2 in cancers. The results showed that low expression of FAIM2 is related to a poor prognosis in some cancer types. FAIM2 expression is closely associated with the immune infiltrating level, immune checkpoint genes, and molecular and immune subtypes. In addition, our result reveled that FAIM2 expression is negatively related to TMB, MSI, MMR, and DNA methylation in most cancer types. Furthermore, we explored the role of FAIM2 in intracellular signaling regulation and regulatory factor activity and verified the tumor suppressor effect of FAIM2 in glioma by molecular biology experiments.

Since 2011, evading immune destruction became one of the emerging hallmarks of cancer, scholars began to concentrate on immunotherapy research (8). Aran D et al. found that the low purity of the tumor indicated advanced stage and poor prognosis in LGG (18) and ESTIMATEScore represents the purity of the tumor (19). In our study, ESTIMATEScore including ImmuneScore and StromalScore have a positive connection with FAIM2 expression in most cancer types, but have a negative connection in LGG, which could explain why FAIM2 low expression has a connection with poor prognosis in LGG. Immunotherapy primarily recognizes and attacks cancer cells by harnessing immune cells inside and outside the tumor microenvironment (TME) (20), which has a higher specificity and lower side effects. However, the TME contains a repertoire of immune cells, stromal cells, endothelial cells, and cancer-associated fibroblasts (8) that include both anti-tumor and pro-tumor immune cells. In the TME, CD8+ T cells play as tumor-antagonizing immune cells while cancer-associated fibroblasts and MDSC play as tumor-promoting immune cells (21). Our study shows that FAIM2 expression is positively correlated with CD8+ T cell infiltration but negatively correlated with MDSC infiltration in most tumors, which indicated that FAIM2 mainly plays a role in anti-tumor immunity in various cancers. Interestingly, cancer-associated fibroblasts were negatively correlated with FAIM2 expression only in LGG, GBM, and PCPG, the correlation of FAIM2 and CAFs in other tumor types still needs further investigation.

Immune checkpoints are a set of inhibitory pathways built into the immune system that are critical for maintaining self-tolerance and regulating physiological immune responses and dysregulation of immune checkpoints is an important mechanism by which some tumors evade host immunity (22). Thus, blocking the immune checkpoint pathway is emerging as a promising approach for tumor immunotherapy (23). We explored the correlation between more than 40 frequent immune checkpoint genes and FAIM2 expression. Interestingly, in our results, almost all immune checkpoints were inversely correlated with FAIM2 expression in COAD, LGG, and GBM, but the complete opposite results were observed in other tumors, which suggests that FAIM2 may be a potential immunotherapeutic target for COAD, LGG, and GBM, while in other tumors, FAIM2 is positively correlated with checkpoint expression which might indicate that patients with high FAIM2 expression are more sensitive to immune checkpoint blockade therapy.

MMR genes play an important role in maintaining the stability of the genome and MSI is associated with increased cancer risk and has specific clinicopathological features, including increased TMB and lymphocyte entry into the tumor. In particular, TMB is a potential biomarker for predicting immune checkpoint blockade response (24). FAIM2 expression is closely related to TMB, MSI, and MMR, which proved that FAIM2 is closely related to the TME in human cancers. In addition, FAIM2 is significantly differentially expressed across immune subtypes and molecular subtypes of most cancer types, which may demonstrate that FAIM2 is a promising diagnostic pan-cancer biomarker and is involved in immune regulation. Furthermore, intracellular signaling regulation and regulatory factor activity are regarded to be important in cancer (25, 26). So, we investigated the connections between CNV, methylation, and FAIM2 expression in depth. We also constructed the PPI network of FAIM2 and carried out an enrichment analysis of FAIM2 and its related genes, and found that these genes are indeed related to immunity and apoptosis.

The results of the current study showed that FAIM2 is closely related to the regulation of immunity and play an anti-tumor role in glioma (GBM and LGG). So, we analyzed FAIM2 and its co-expressed genes in glioma through the LinkedOmics database and found that these genes are involved in a large number of immune-related pathways, including adaptive immune response, T cell activation, regulation of innate immune response, lymphocyte mediated immunity, leukocyte proliferation, primary immunodeficiency, and antigen processing and presentation. In addition, these genes are also related to MMR, p53 pathway, and cell cycle. Next, we validated the role of FAIM2 in Glioma by molecular biological methods. IHC and Western blotting confirmed that FAIM2 expression was downregulated in glioma tissues. EdU-DNA synthesis assay, cell viability assay, and colony formation assay confirmed that FAIM2 inhibits the proliferation of glioma cells. These results confirm the correctness and reliability of the results of the pan-cancer bioinformatics analysis in glioma, and we will conduct similar molecular biological validation in more cancers in the future.

However, even though we explored and integrated information from multiple databases, the present study still has several limitations. Firstly, although bioinformatics analysis provided us with some important insights into FAIM2 in malignancies, and we also validated the tumor suppressor role of FAIM2 in glioma by molecular biology methods, we are still required to validate our results further through biological experiments *in vitro* or *in vivo*. Secondly, whether FAIM2 affects clinical prognosis through the immune pathway is still unclear. Taken together, our first pan-cancer analysis of revealed the critical involvement of FAIM2 in tumorigenesis, clinical prognosis, and tumor immune infiltration. In the future, prospective research concentrating on FAIM2 expression and tumor immune infiltration would be useful in providing a conclusive answer, thereby developing immune-based anticancer therapies.

## Data availability statement

The datasets presented in this study can be found in online repositories. The names of the repository/repositories and accession number(s) can be found in the article/**Supplementary Material**.

## Ethics statement

The study was approved by the Institutional Ethics Committee of the Faculty of Medicine at Renmin Hospital of Wuhan University (2012LKSZ (010) H).

## Author contributions

JC, QC and JY designed the research. JC and ZY downloaded and analyzed the data. LY, ZY, YH, LG, ST, QS, ZS and YW wrote the paper. All authors contributed to the article and approved the submitted version.

## Funding

This work was supported by the National Natural Science Foundation of China (No.82072764) and Natural Science Foundation of Hubei Province (No.2020CFB256).

## Conflict of interest

The authors declare that the research was conducted in the absence of any commercial or financial relationships that could be construed as a potential conflict of interest.

## Publisher’s note

All claims expressed in this article are solely those of the authors and do not necessarily represent those of their affiliated organizations, or those of the publisher, the editors and the reviewers. Any product that may be evaluated in this article, or claim that may be made by its manufacturer, is not guaranteed or endorsed by the publisher.

## Supplementary material

Supplementary Figure 1 The correlation between FAIM2 expression and cancer stages was analyzed by the GEPIA database.

Supplementary Figure 2 Overall survival **(A-C)** and disease-free survival **(D)** of different tumors in TCGA were analyzed by FAIM2 gene expression using GEPIA2 database.

Supplementary Figure 3 Correlations between FAIM2 expression and MHC molecules **(A)**, Immunoinhibitor **(B) and** Immunostimulator (C).

Supplementary Figure 4 FAIM2 related genes enrichment analysis. **(A)** The protein-protein interaction (PPI) network using the STRING database. KEGG **(B)** and GO **(C)** enrichment analysis of the related genes. The outermost circle on the right represents the term in the right, and the inner circle on the right represents the p-value of the corresponding pathway.

Supplementary Figure 5 GSEA enrichment analysis between the FAIM2 high expression group and FAIM2 low expression group in various cancers.

Supplementary Figure 6 IHC score of FAIM2 in clinical tissues were quantified. NBT: n = 4, LGG: n=4, GBM: n=4. *p < 0.05.
