# Supplementary figures and images for: FAIM2 is a potential pan-cancer biomarker for prognosis and immune infiltration
Source: Front Oncol. 2022 Sep 14;12:998336. doi: 10.3389/fonc.2022.998336 (PMC9516132; doi:10.3389/fonc.2022.998336)

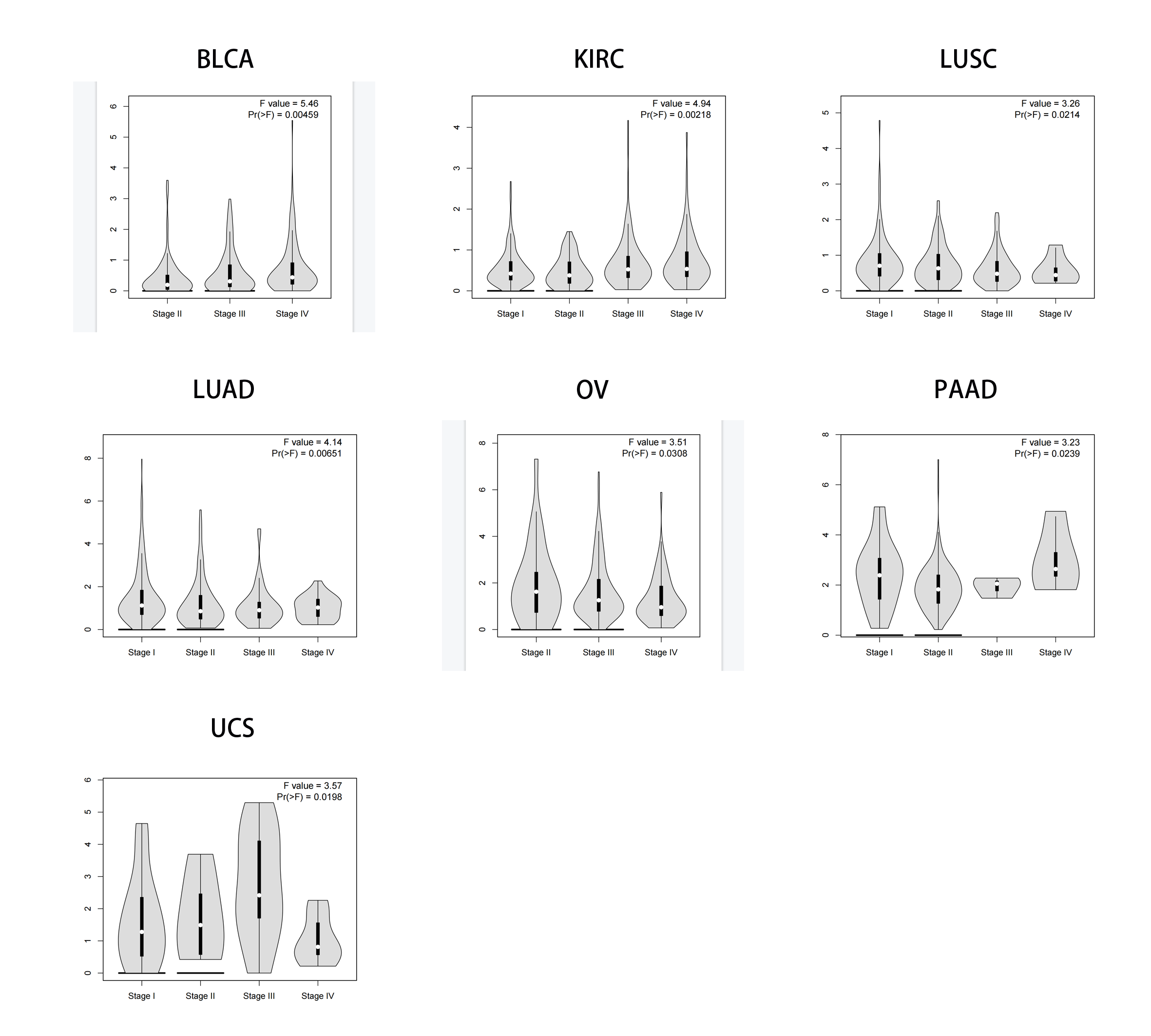

Supplement: Supplementary Figure 1 — The correlation between FAIM2 expression and cancer stages was analyzed by the GEPIA database. [file Image_1.tif]

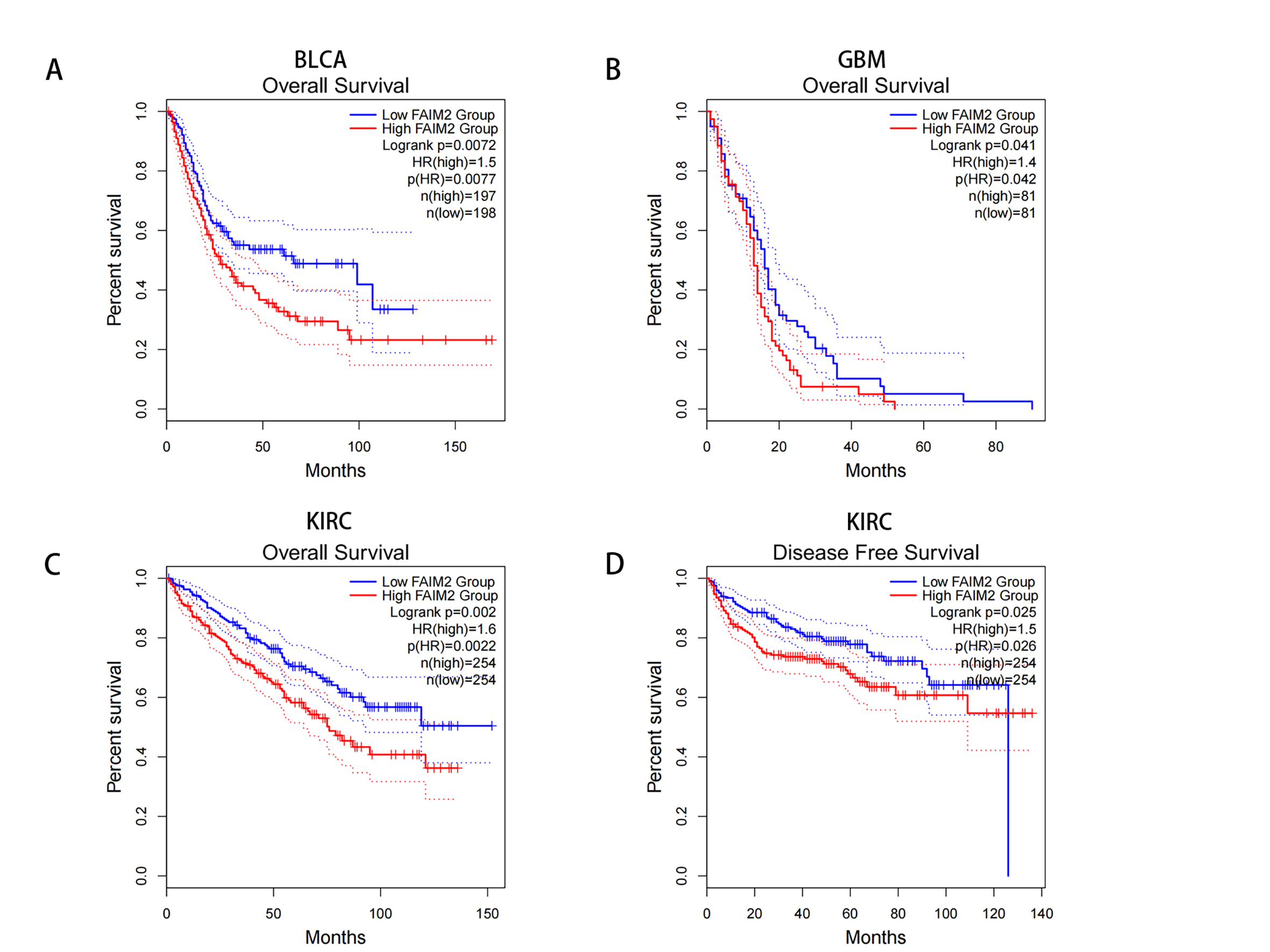

Supplement: Supplementary Figure 2 — Overall survival (A-C) and disease-free survival (D) of different tumors in TCGA were analyzed by FAIM2 gene expression using GEPIA2 database. [file Image_2.tif]

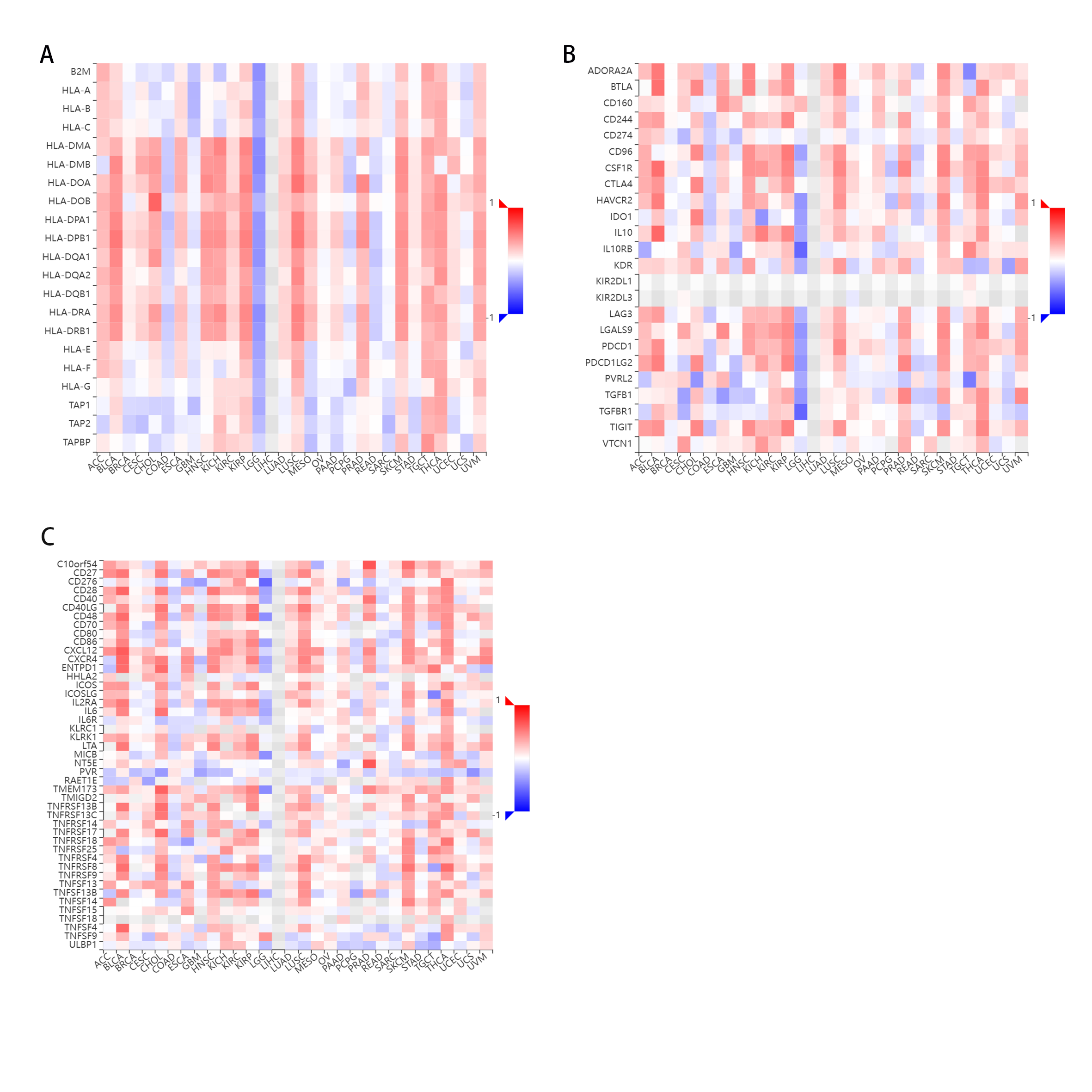

Supplement: Supplementary Figure 3 — Correlations between FAIM2 expression and MHC molecules (A), Immunoinhibitor (B) and Immunostimulator (C). [file Image_3.tif]

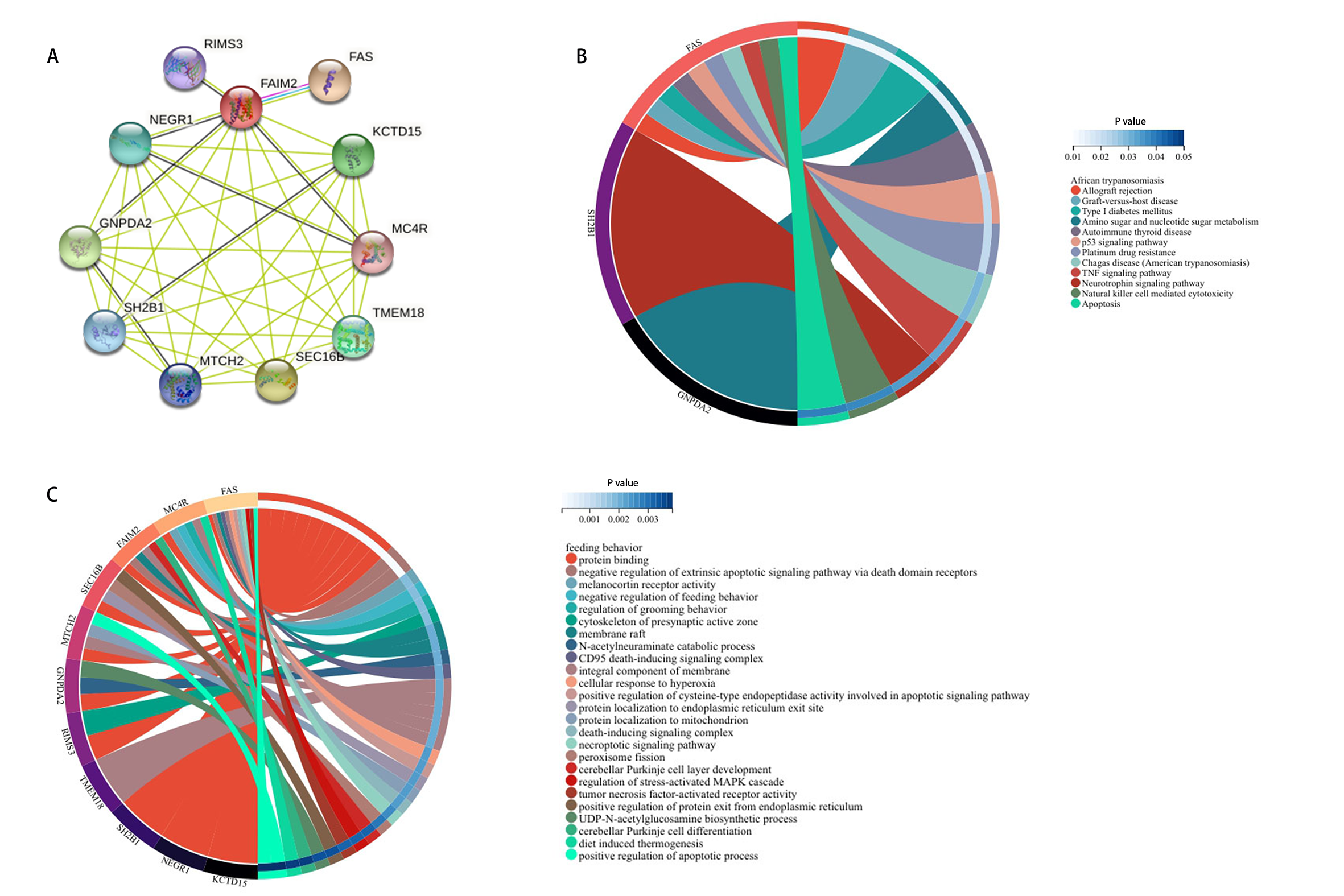

Supplement: Supplementary Figure 4 — FAIM2 related genes enrichment analysis. (A) The protein-protein interaction (PPI) network using the STRING database. KEGG (B) and GO (C) enrichment analysis of the related genes. The outermost circle on the right represents the term in the right, and the inner circle on the right represents the p-value of the corresponding pathway. [file Image_4.tif]

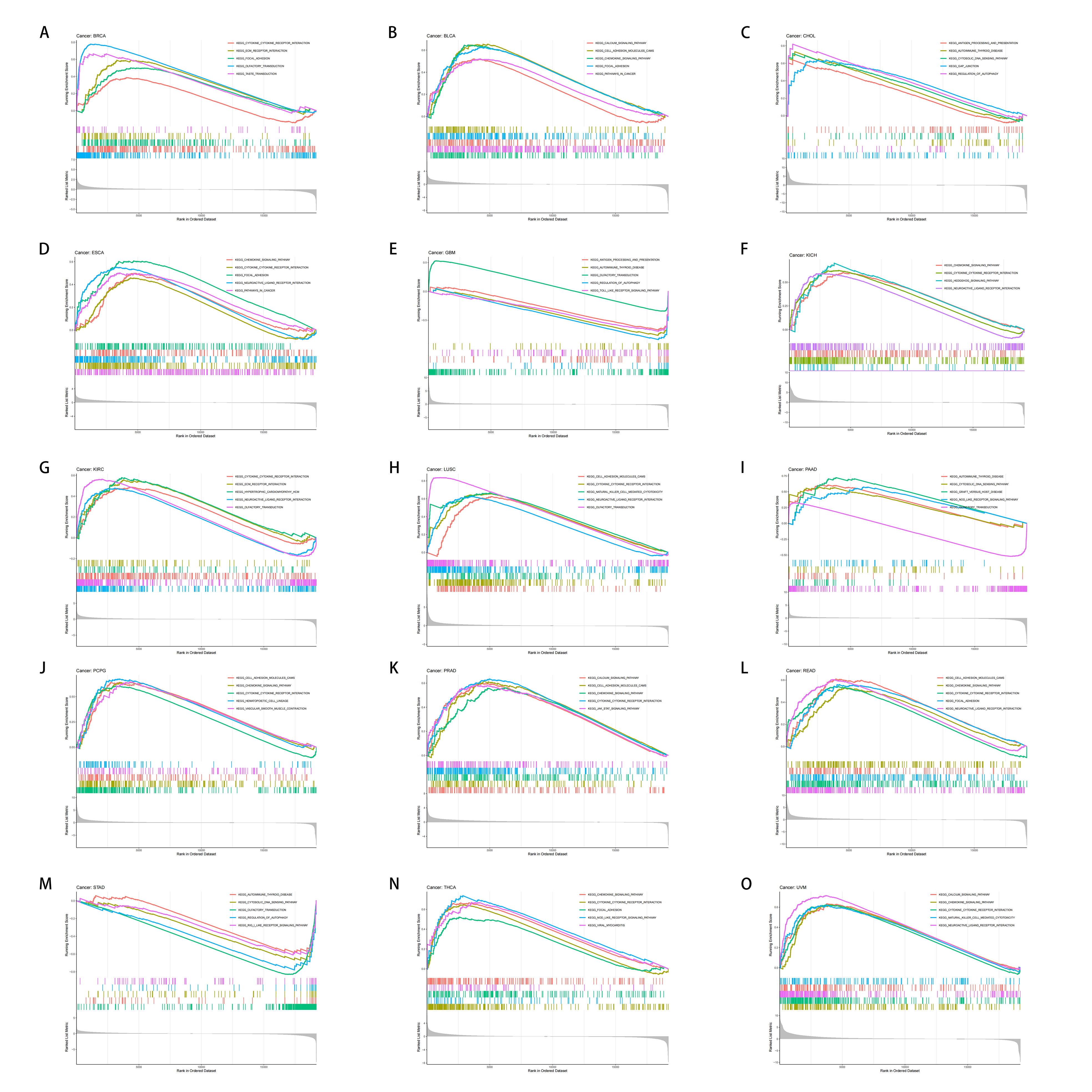

Supplement: Supplementary Figure 5 — GSEA enrichment analysis between the FAIM2 high expression group and FAIM2 low expression group in various cancers. [file Image_5.tif]

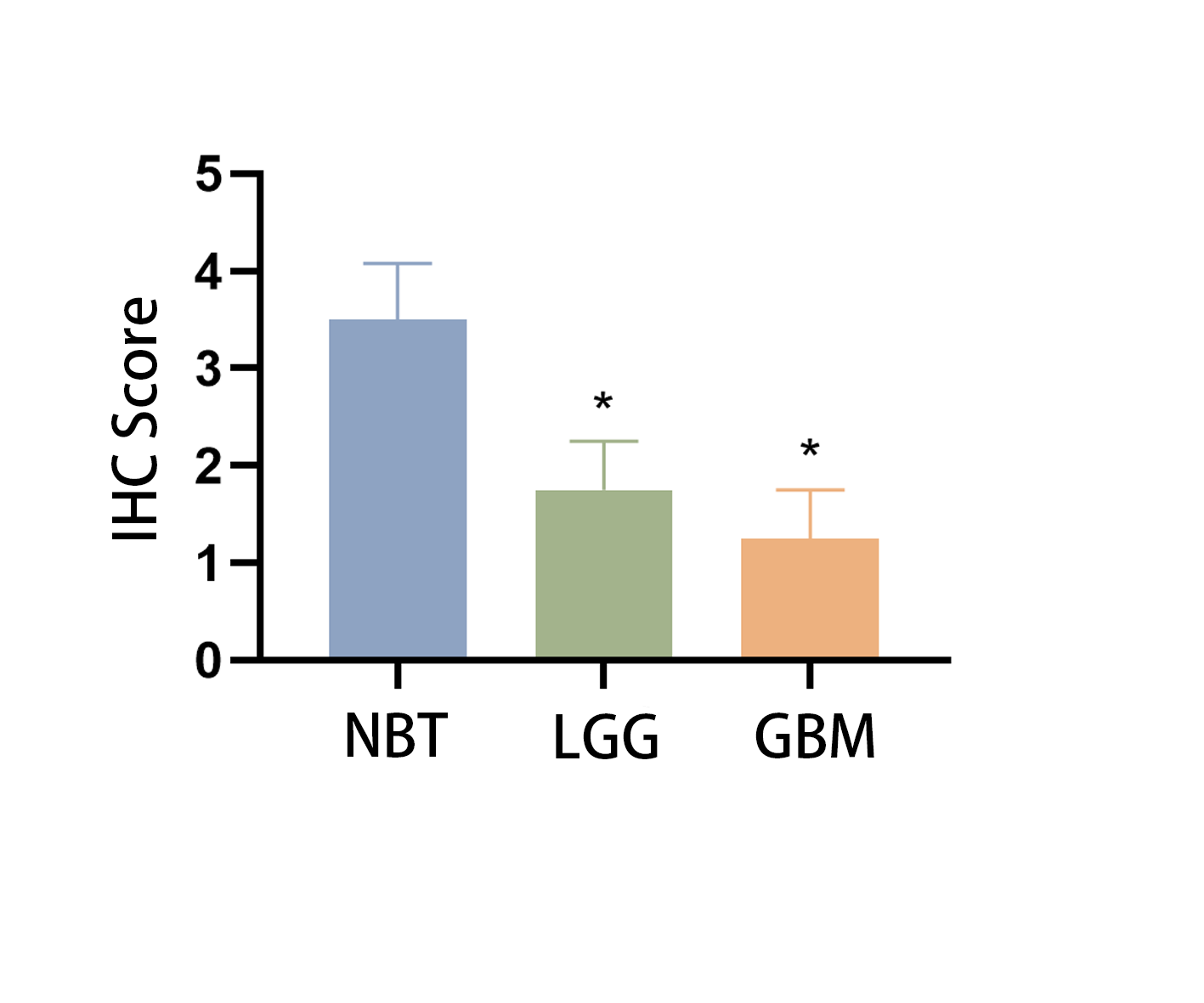

Supplement: Supplementary Figure 6 — IHC score of FAIM2 in clinical tissues were quantified. NBT: n = 4, LGG: n=4, GBM: n=4. *p < 0.05. [file Image_6.tif]

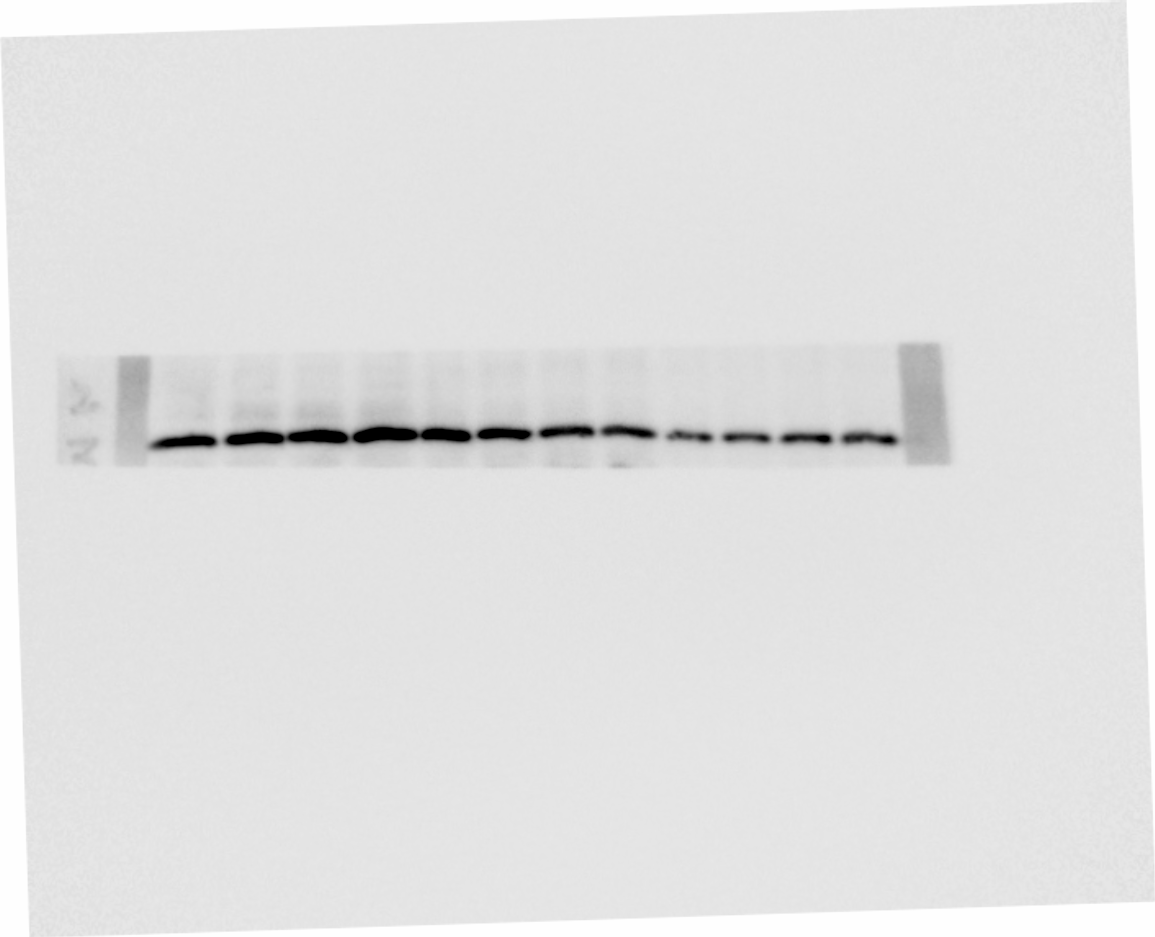

Supplement: Supplementary file 9 [file DataSheet_2.zip › Raw data 2/Figure 12/Figure 12A FAIM2.tif]

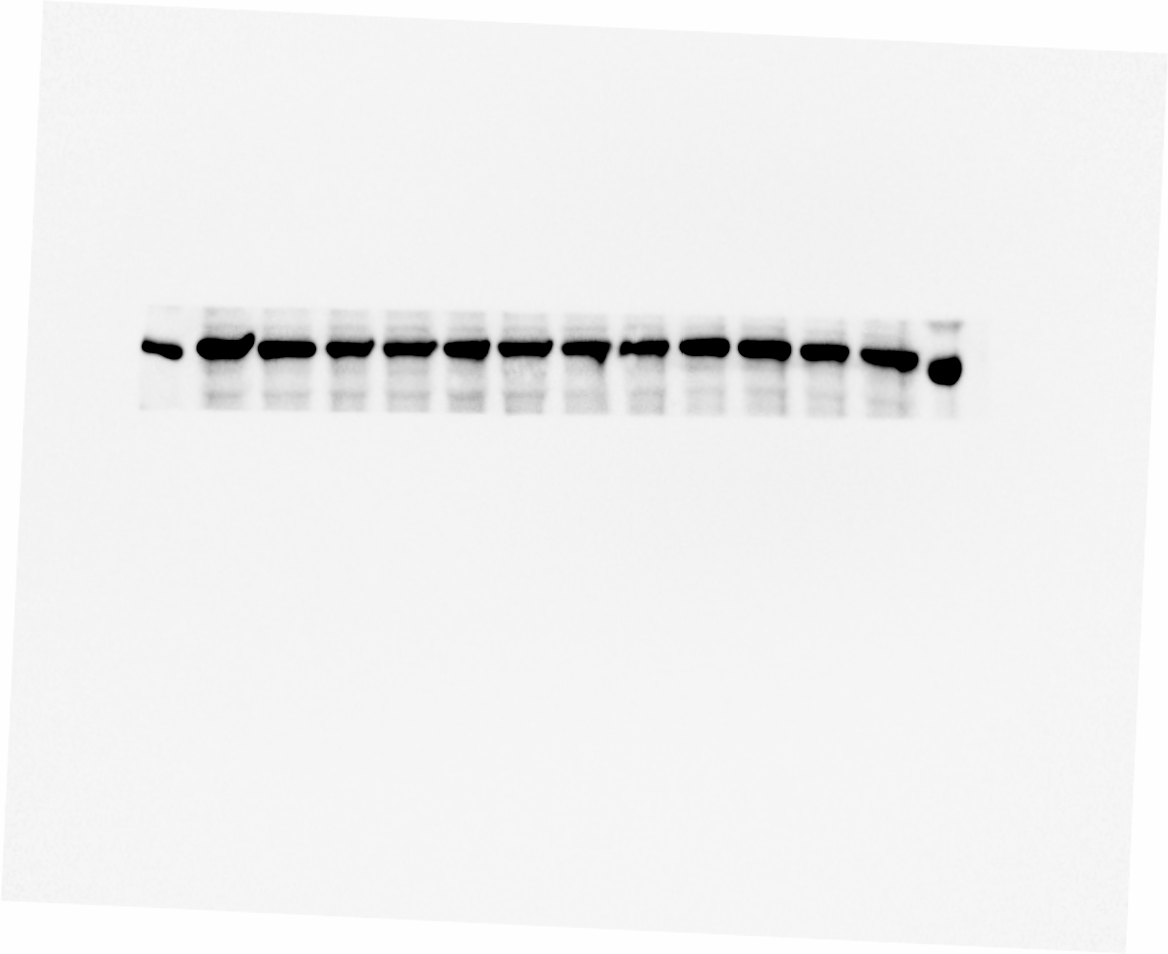

Supplement: Supplementary file 9 [file DataSheet_2.zip › Raw data 2/Figure 12/Figure 12A β-tubulin.tif]

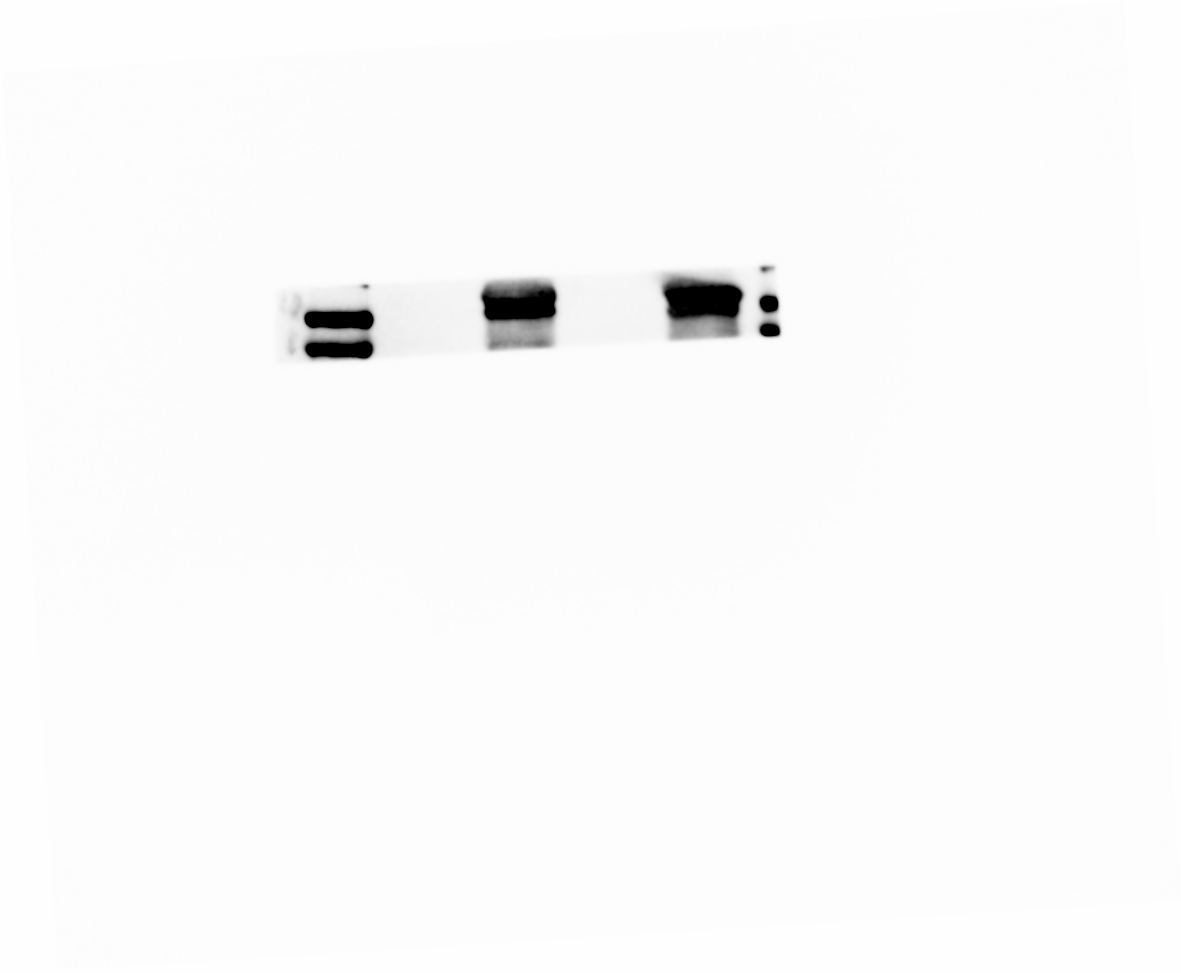

Supplement: Supplementary file 9 [file DataSheet_2.zip › Raw data 2/Figure 12/Figure 12D GFP.tif]

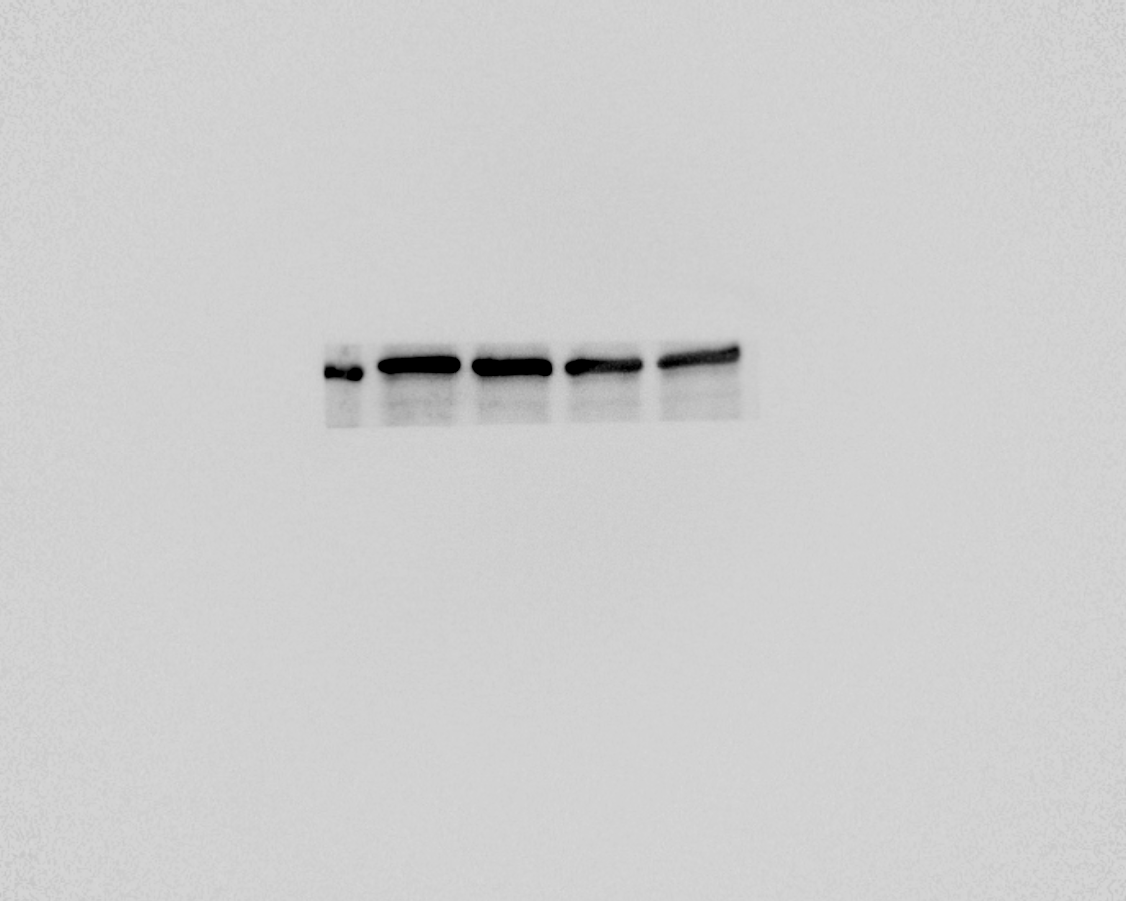

Supplement: Supplementary file 9 [file DataSheet_2.zip › Raw data 2/Figure 12/Figure 12D β-tubulin.tif]

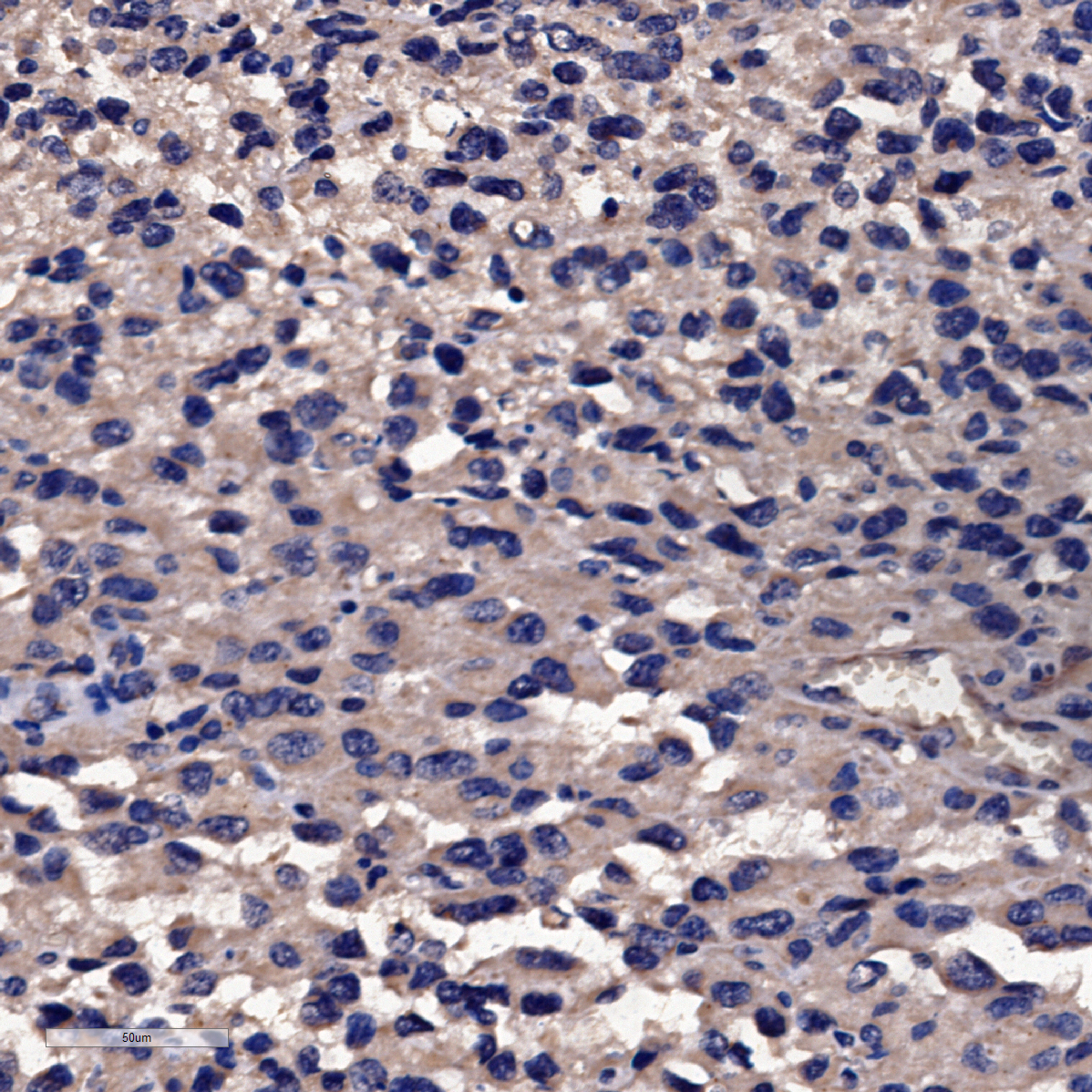

Supplement: Supplementary file 9 [file DataSheet_2.zip › Raw data 2/Figure 12/IHC/GBM.tif]

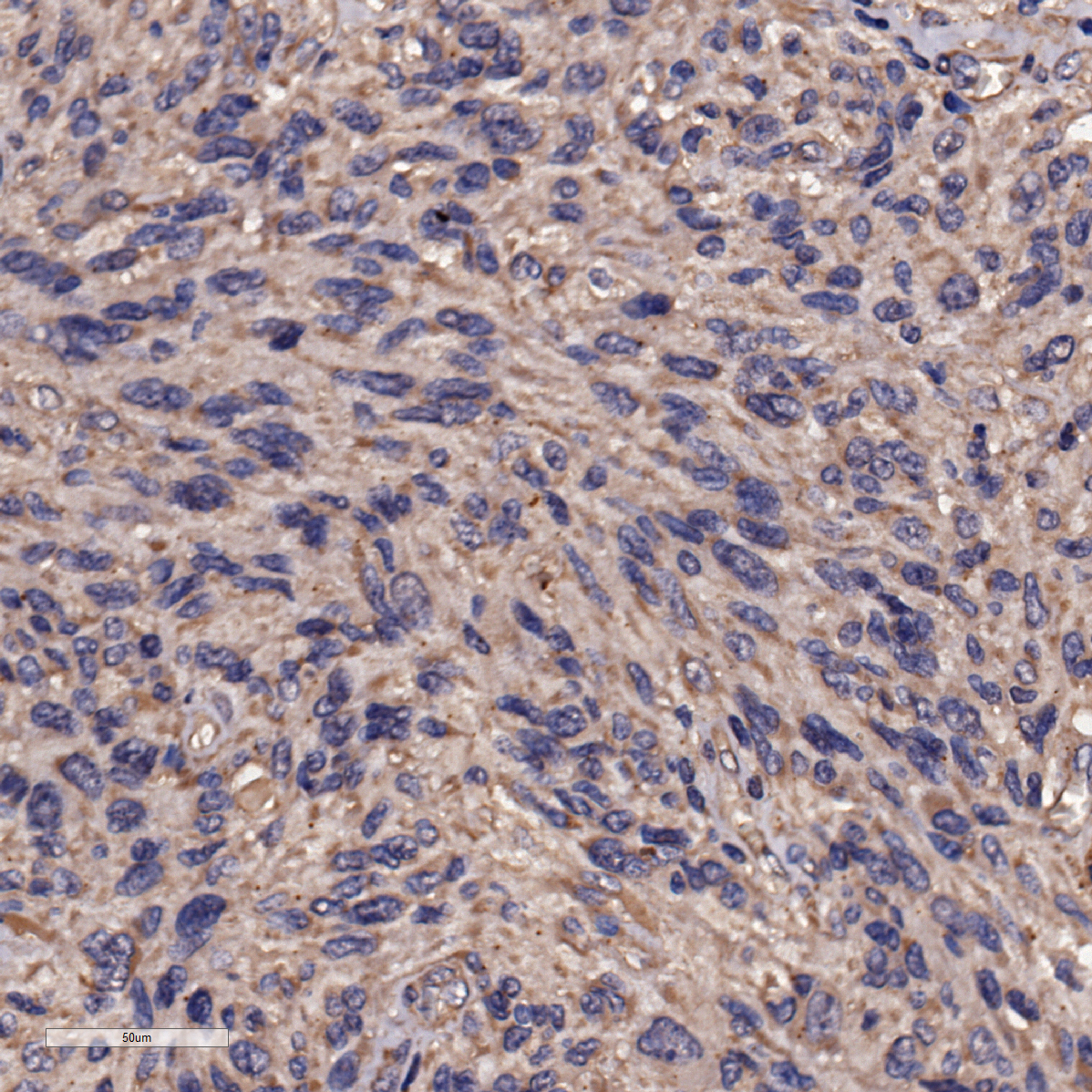

Supplement: Supplementary file 9 [file DataSheet_2.zip › Raw data 2/Figure 12/IHC/LGG.tif]

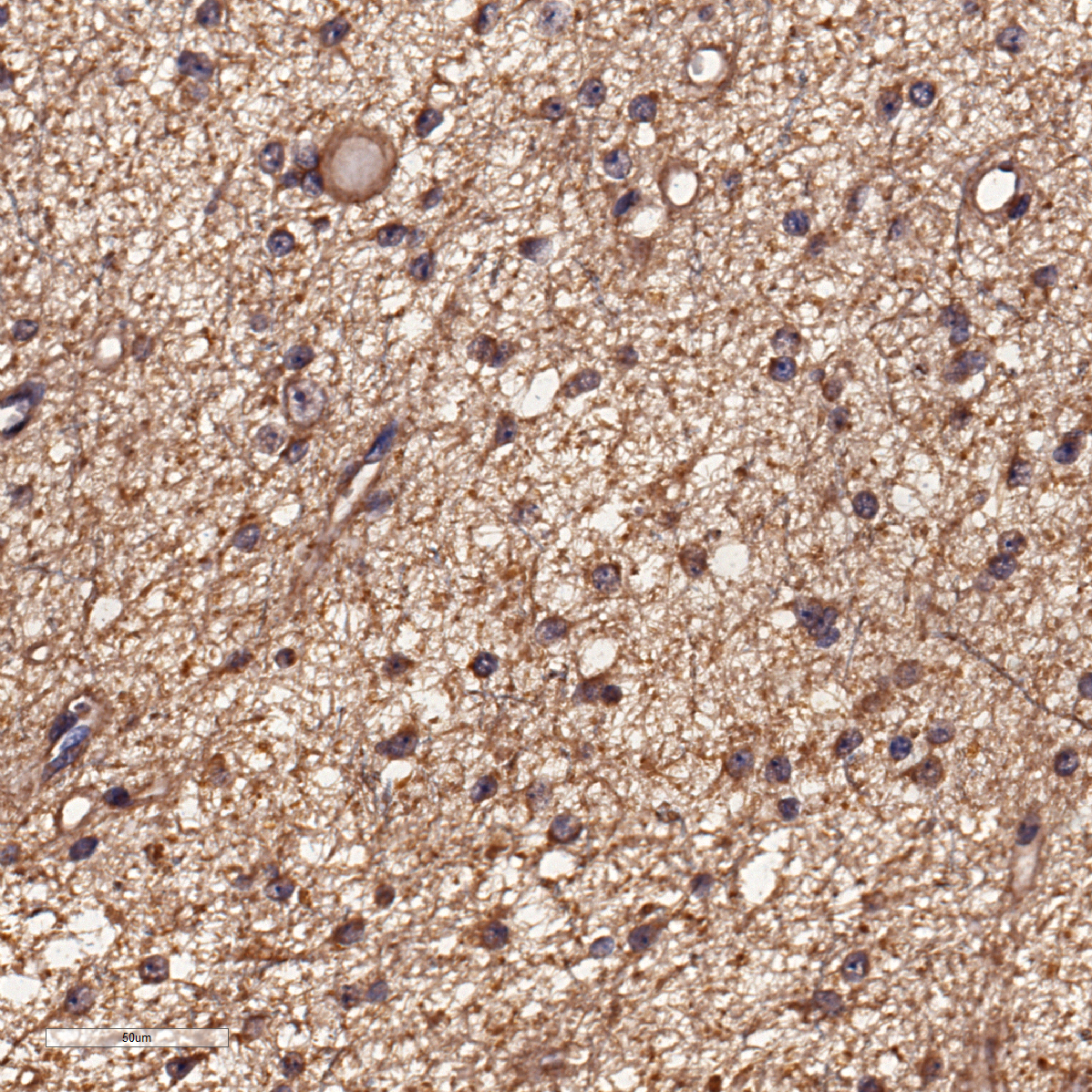

Supplement: Supplementary file 9 [file DataSheet_2.zip › Raw data 2/Figure 12/IHC/NBT.tif]

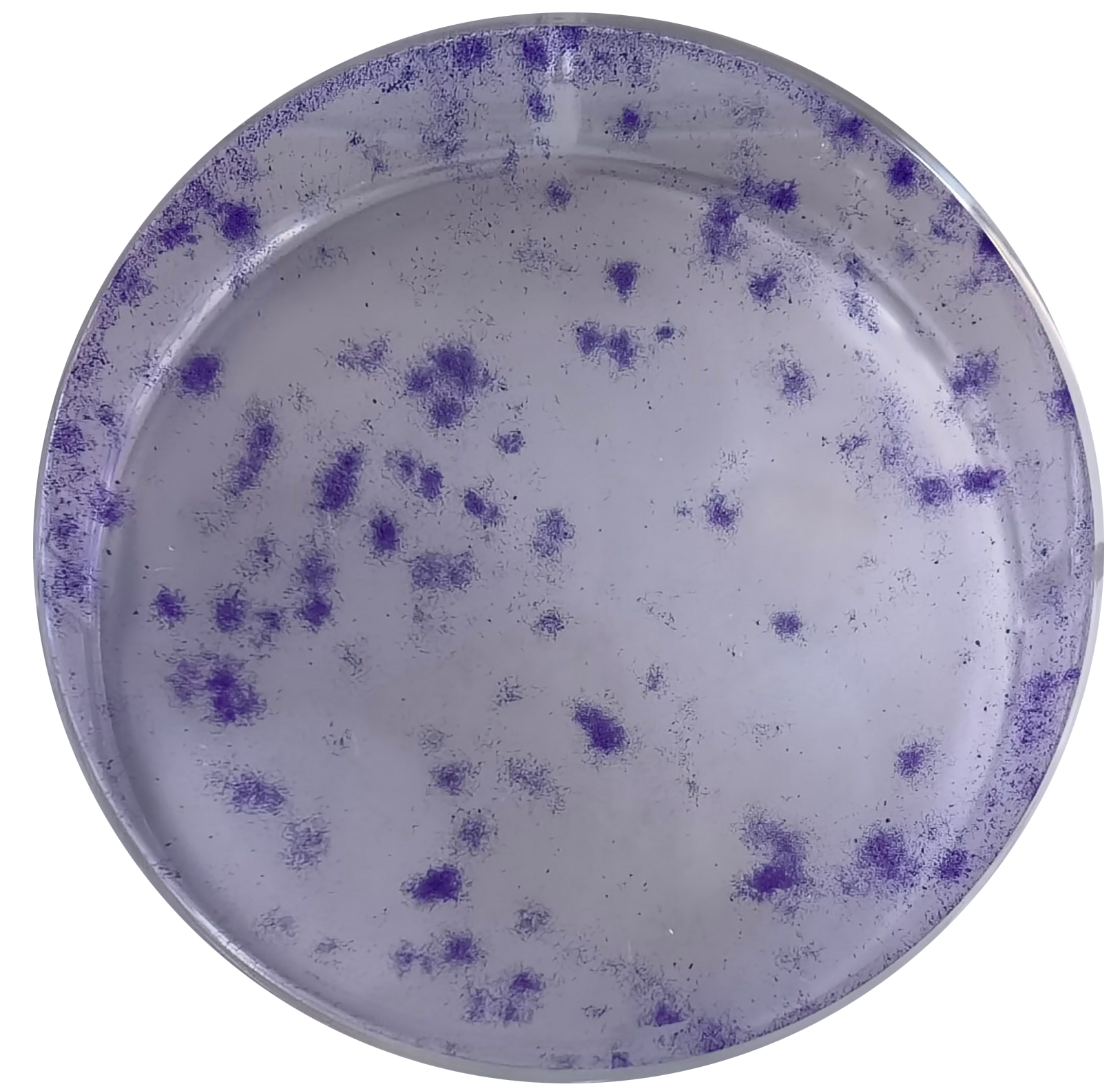

Supplement: Supplementary file 9 [file DataSheet_2.zip › Raw data 2/Figure 13/colony formation assay/5.png]

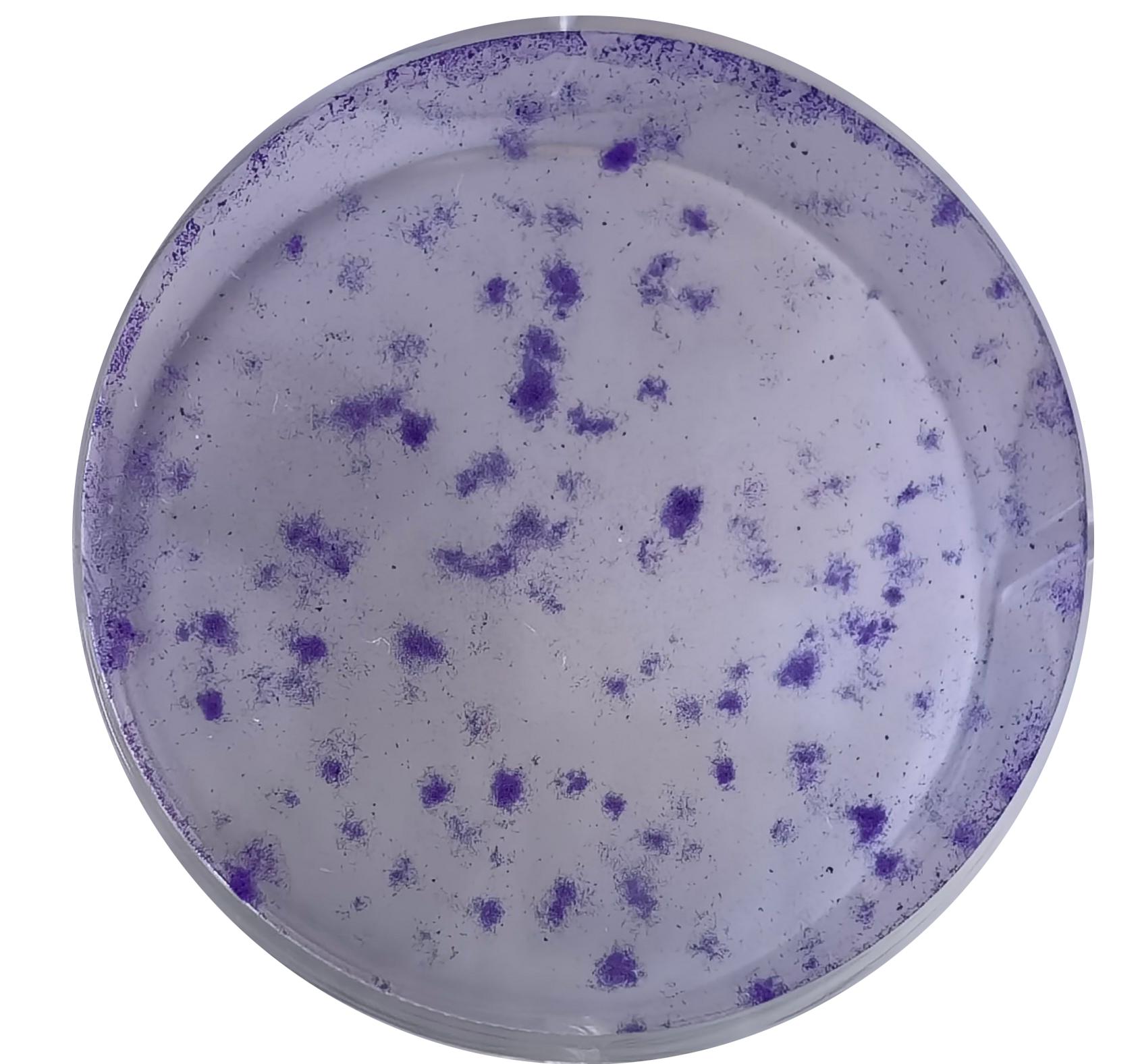

Supplement: Supplementary file 9 [file DataSheet_2.zip › Raw data 2/Figure 13/colony formation assay/6.png]

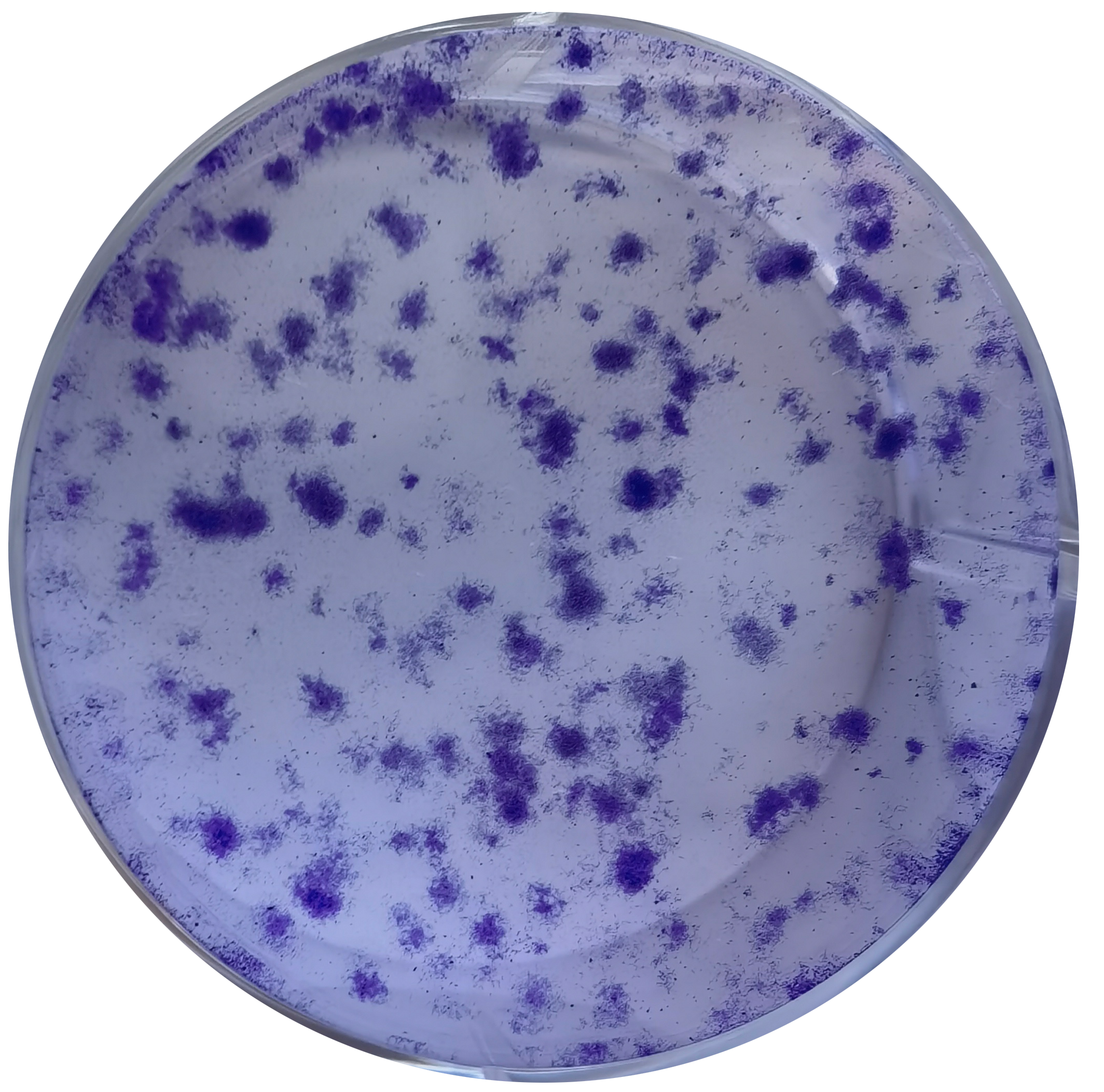

Supplement: Supplementary file 9 [file DataSheet_2.zip › Raw data 2/Figure 13/colony formation assay/C.png]

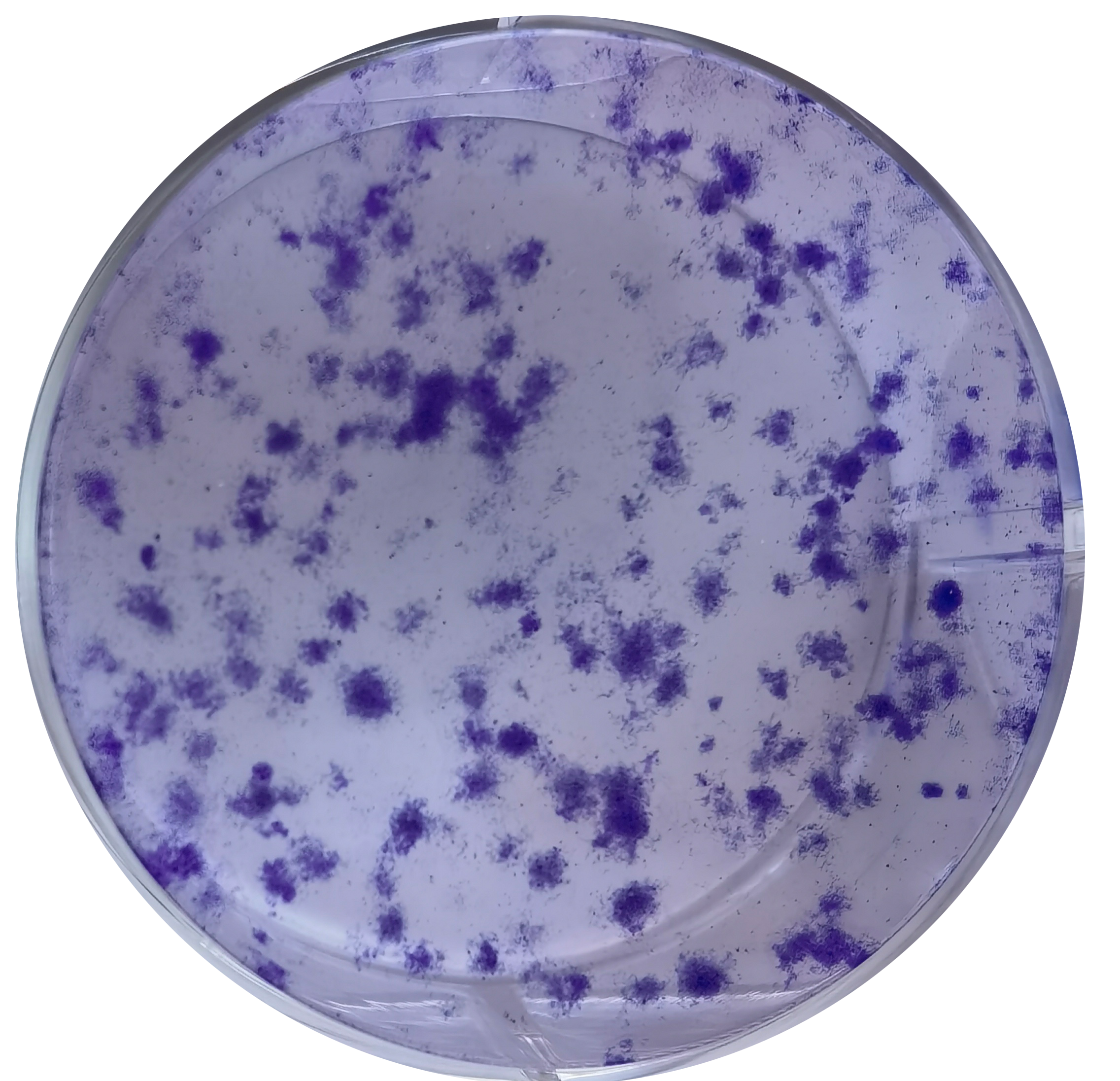

Supplement: Supplementary file 9 [file DataSheet_2.zip › Raw data 2/Figure 13/colony formation assay/D.png]

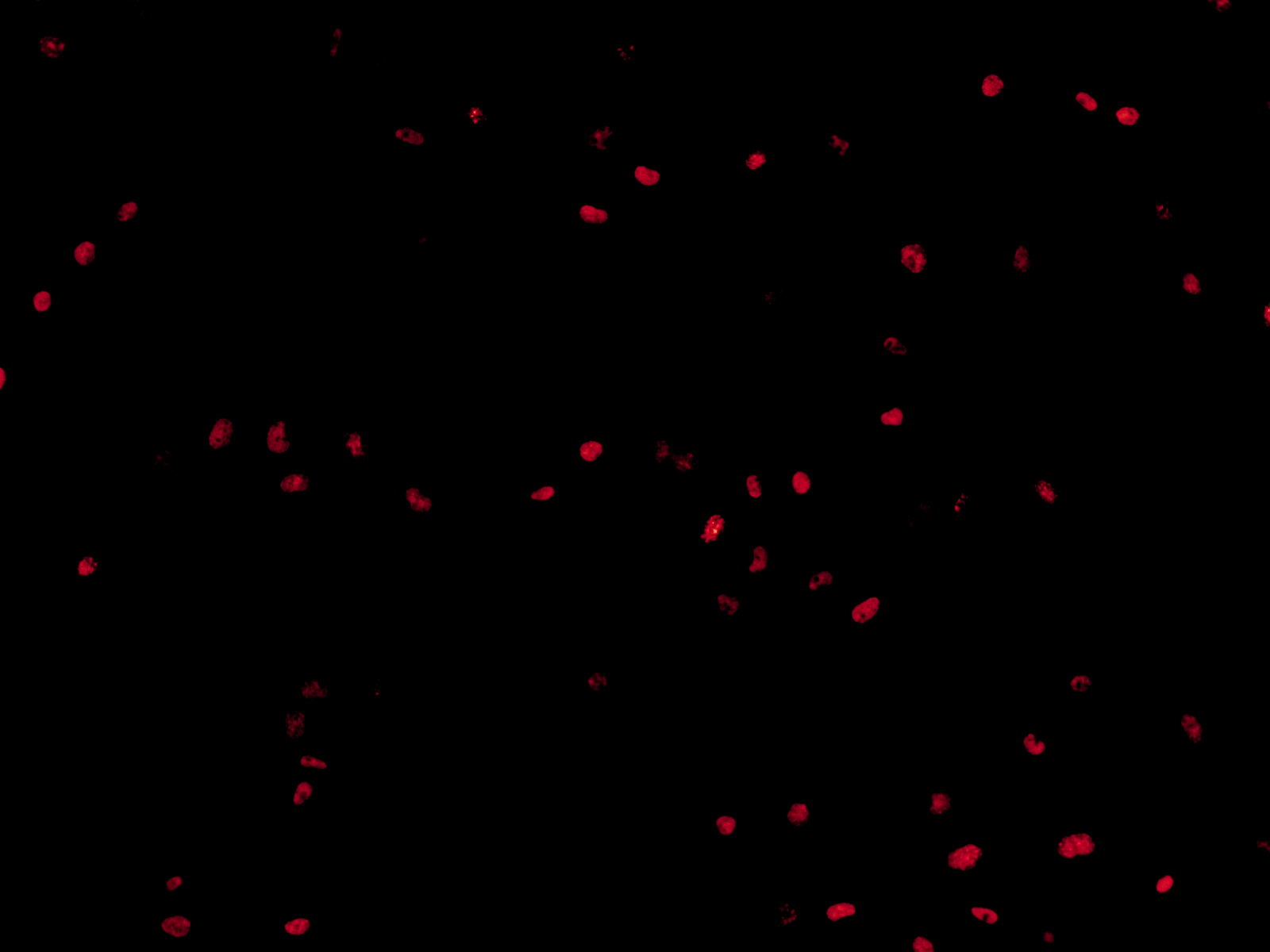

Supplement: Supplementary file 9 [file DataSheet_2.zip › Raw data 2/Figure 13/EDU/U251/U251-GFP-FAIM2 edu.tif]

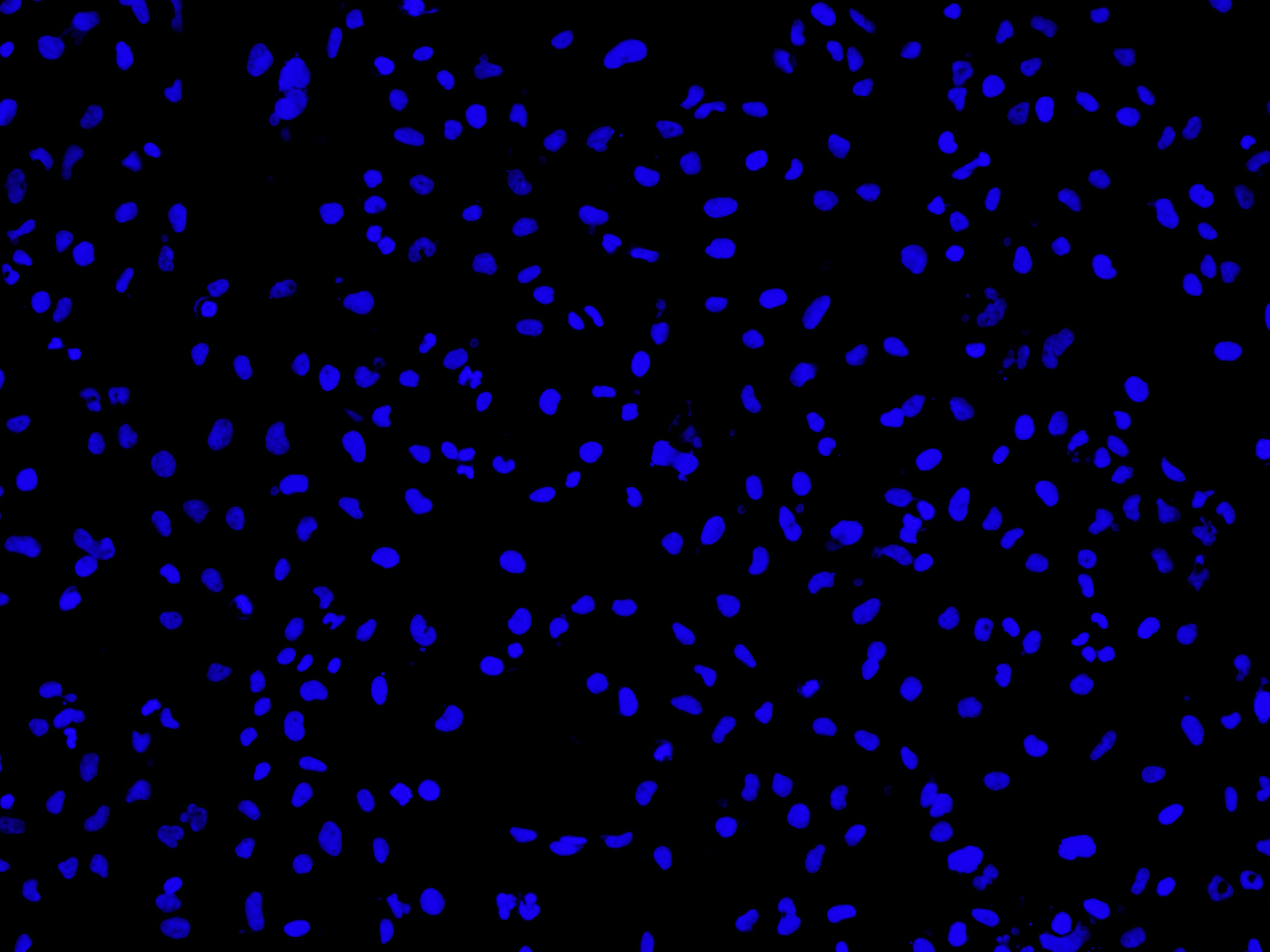

Supplement: Supplementary file 9 [file DataSheet_2.zip › Raw data 2/Figure 13/EDU/U251/U251-GFP-FAIM2 hochest.tif]

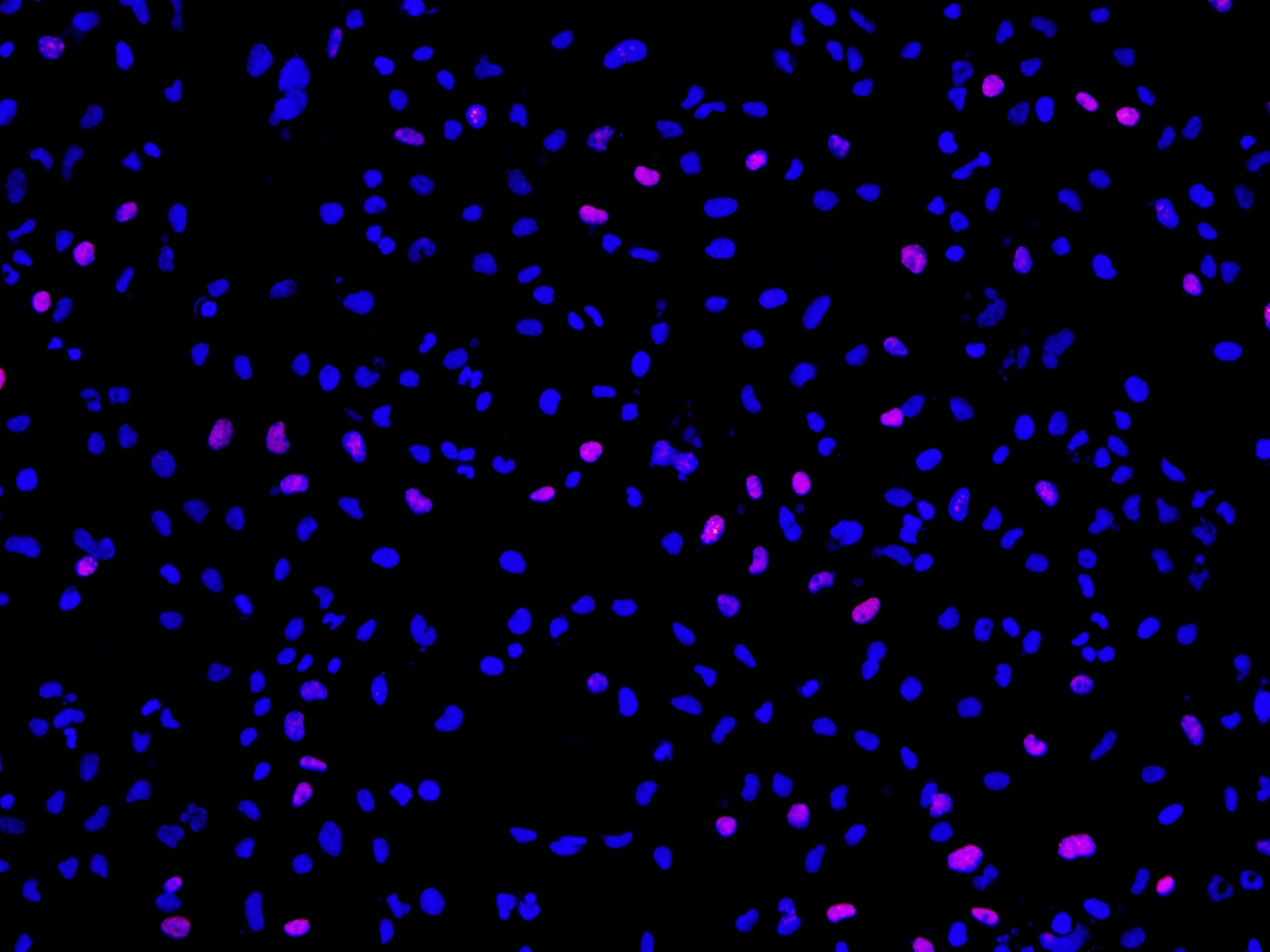

Supplement: Supplementary file 9 [file DataSheet_2.zip › Raw data 2/Figure 13/EDU/U251/U251-GFP-FAIM2 merge.tif]

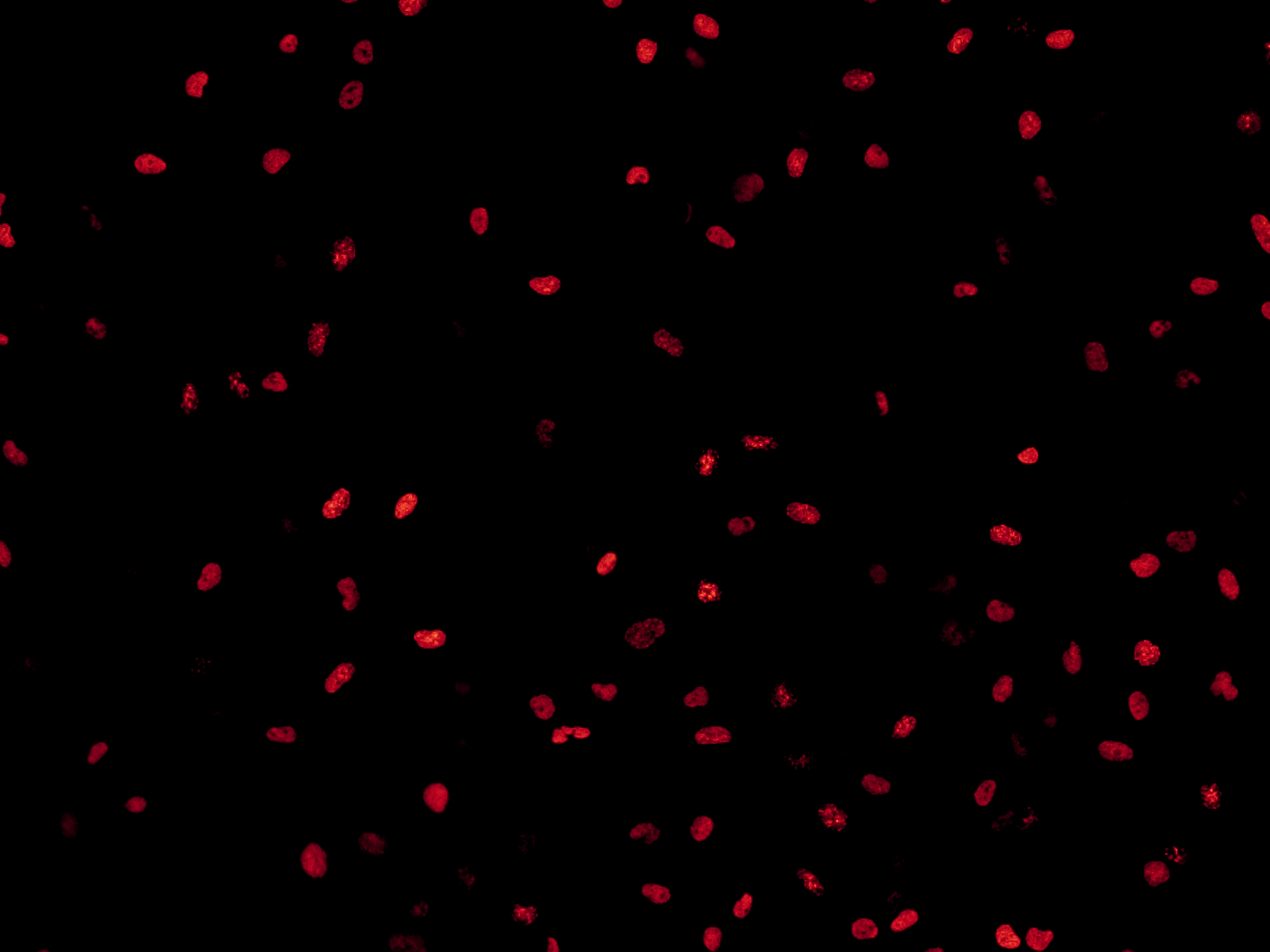

Supplement: Supplementary file 9 [file DataSheet_2.zip › Raw data 2/Figure 13/EDU/U251/U251-vector edu.tif]

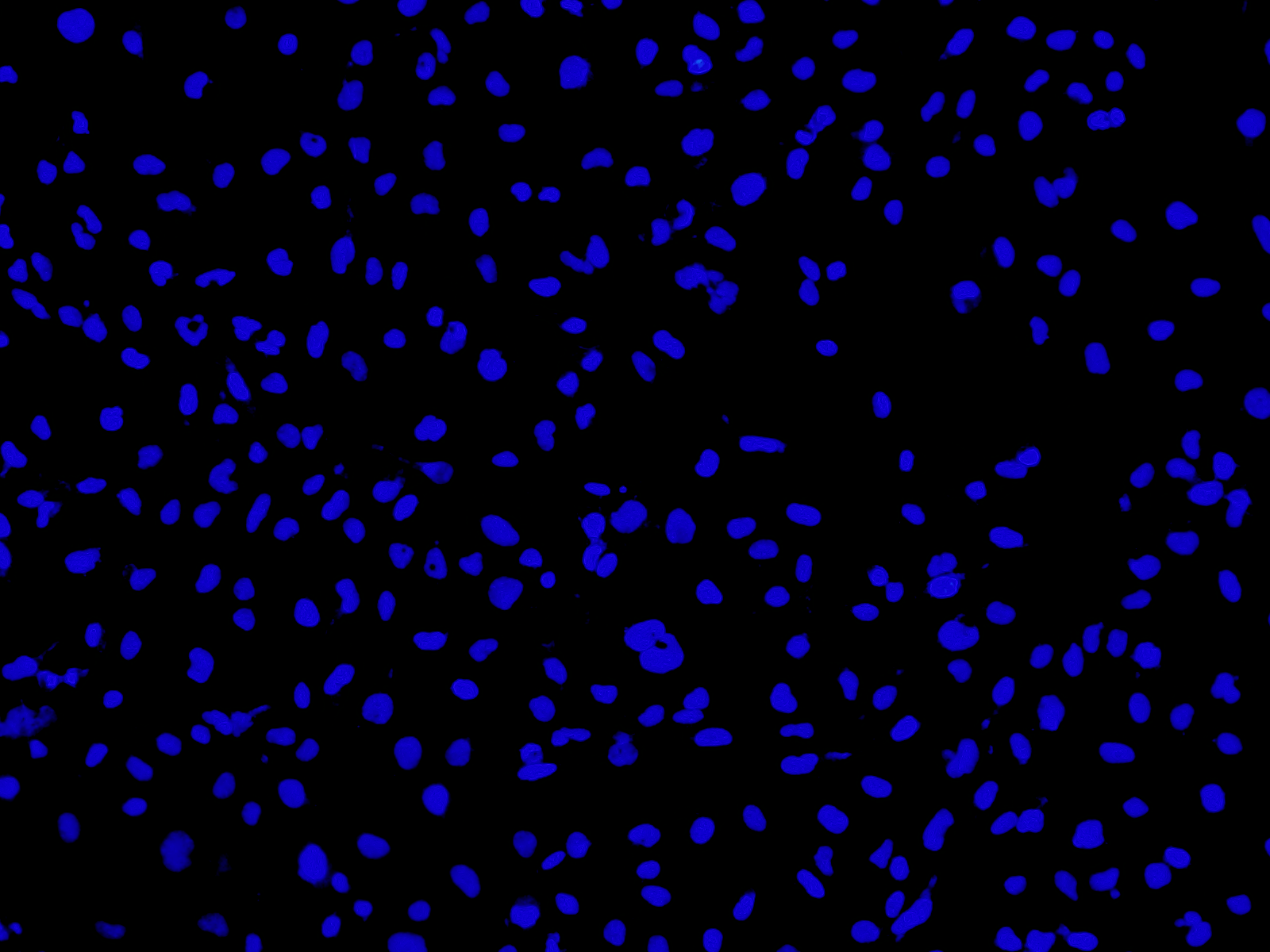

Supplement: Supplementary file 9 [file DataSheet_2.zip › Raw data 2/Figure 13/EDU/U251/U251-vector hochest.tif]

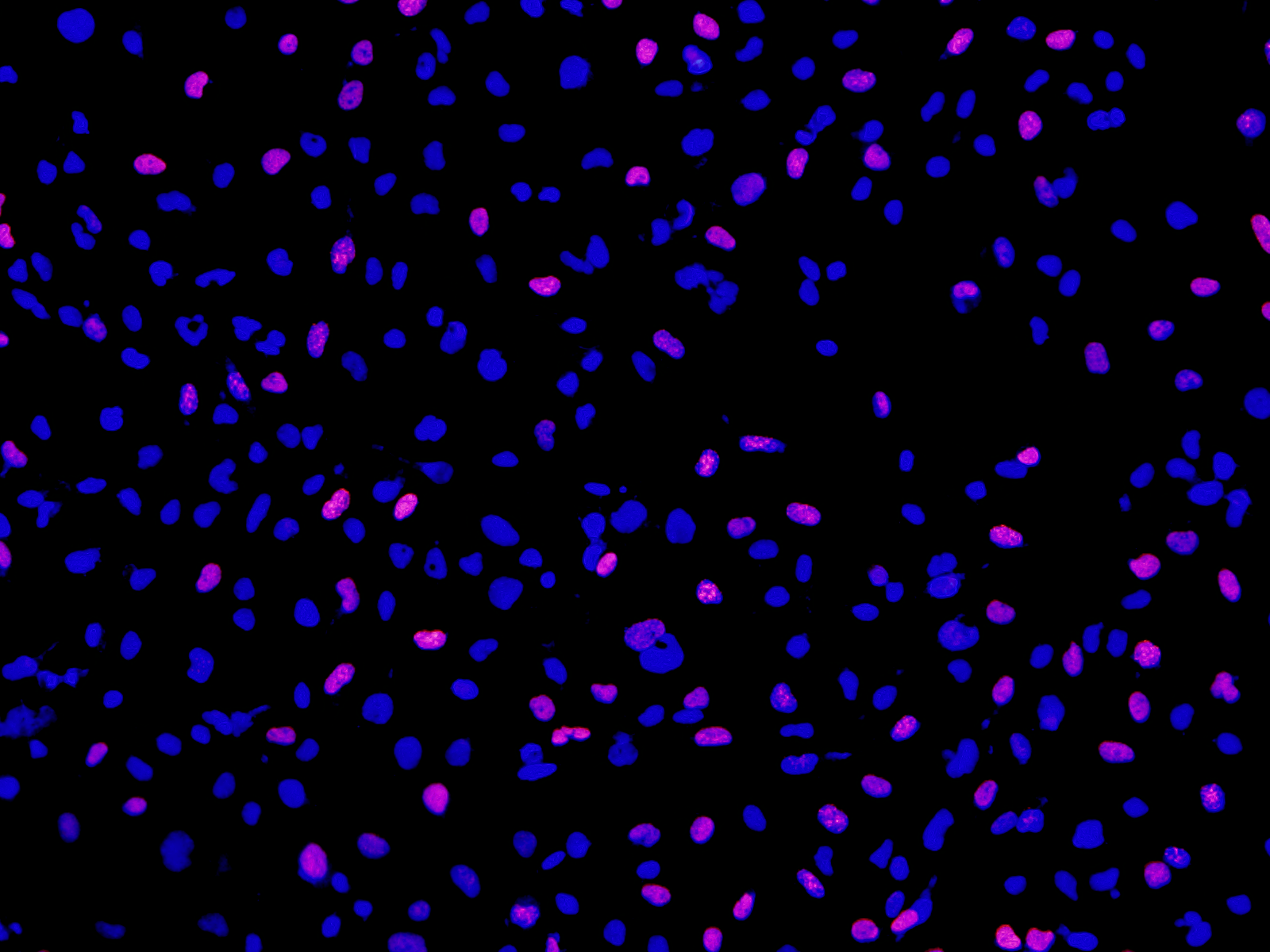

Supplement: Supplementary file 9 [file DataSheet_2.zip › Raw data 2/Figure 13/EDU/U251/U251-vector merge.tif]

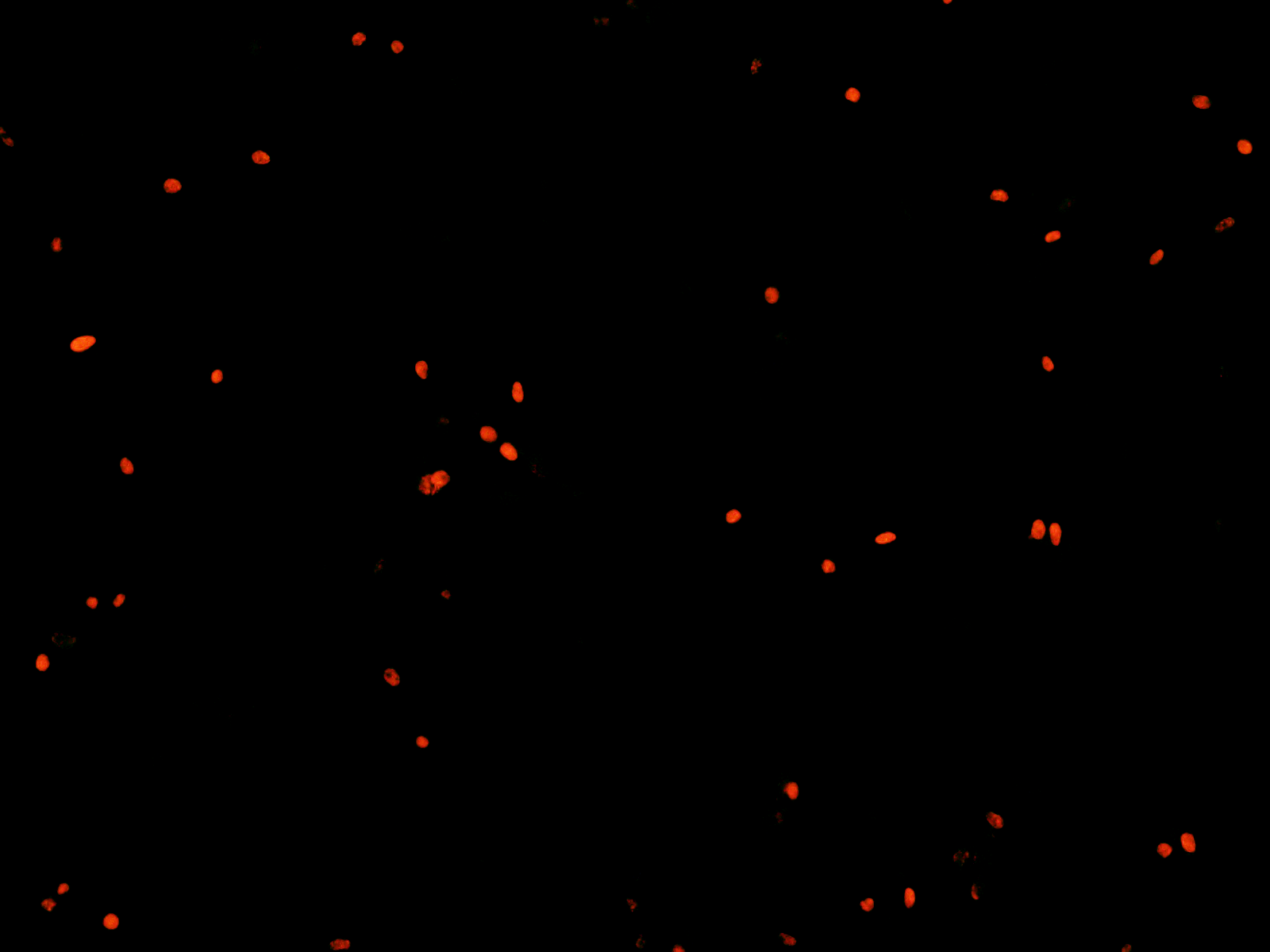

Supplement: Supplementary file 9 [file DataSheet_2.zip › Raw data 2/Figure 13/EDU/U87/U87-GFP-FAIM2 edu.tif]

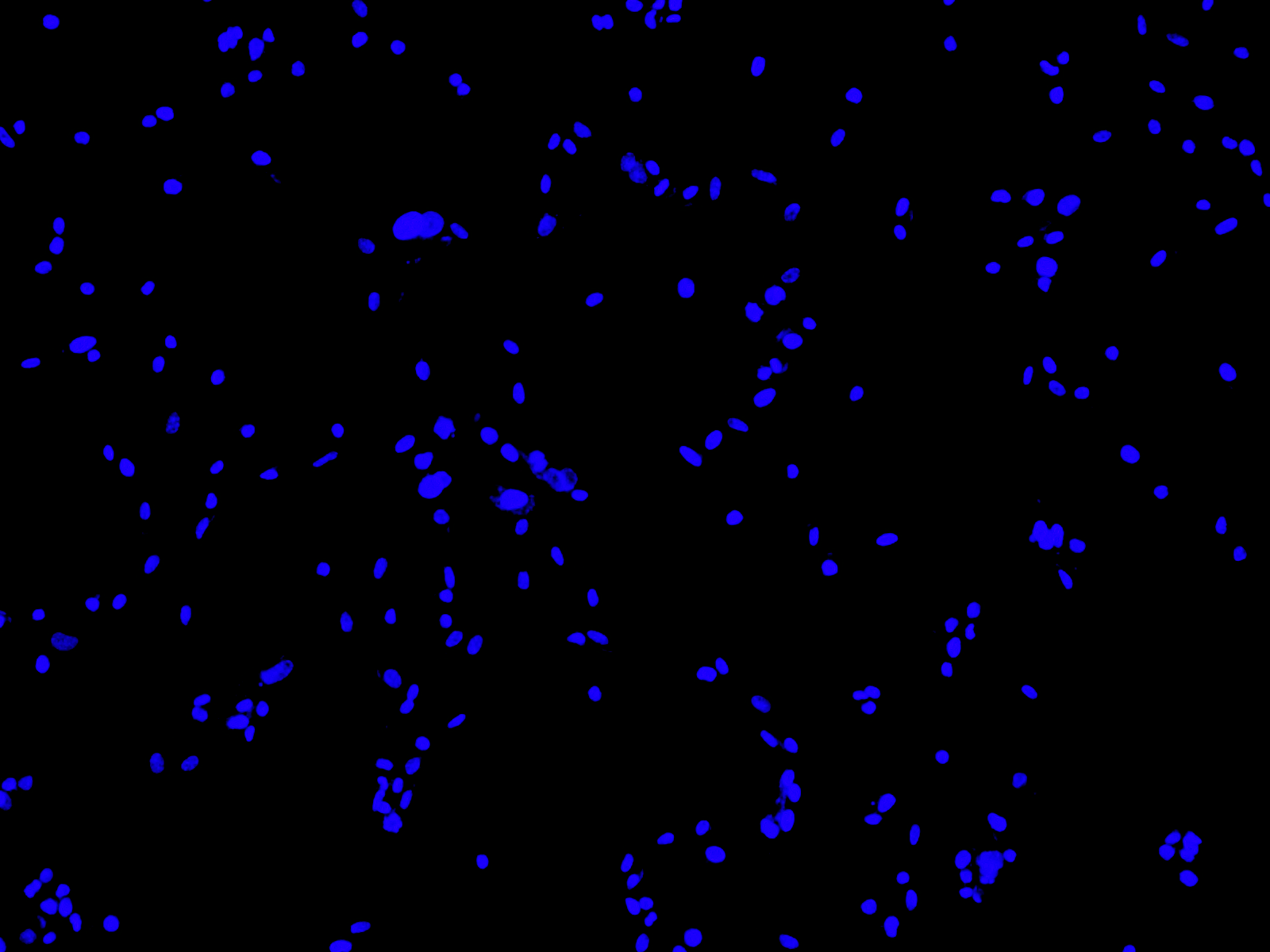

Supplement: Supplementary file 9 [file DataSheet_2.zip › Raw data 2/Figure 13/EDU/U87/U87-GFP-FAIM2 hochest.tif]

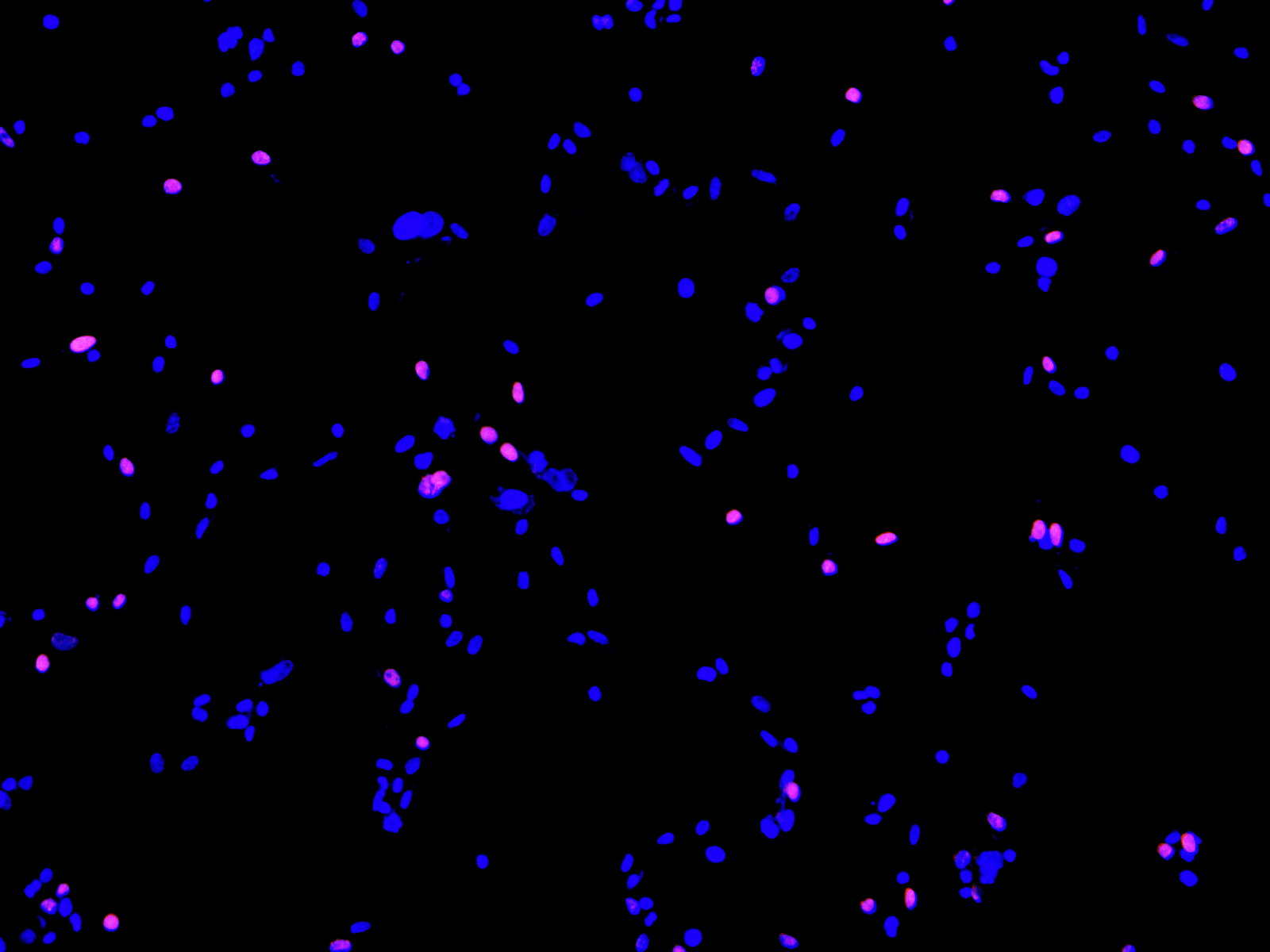

Supplement: Supplementary file 9 [file DataSheet_2.zip › Raw data 2/Figure 13/EDU/U87/U87-GFP-FAIM2 merge.tif]

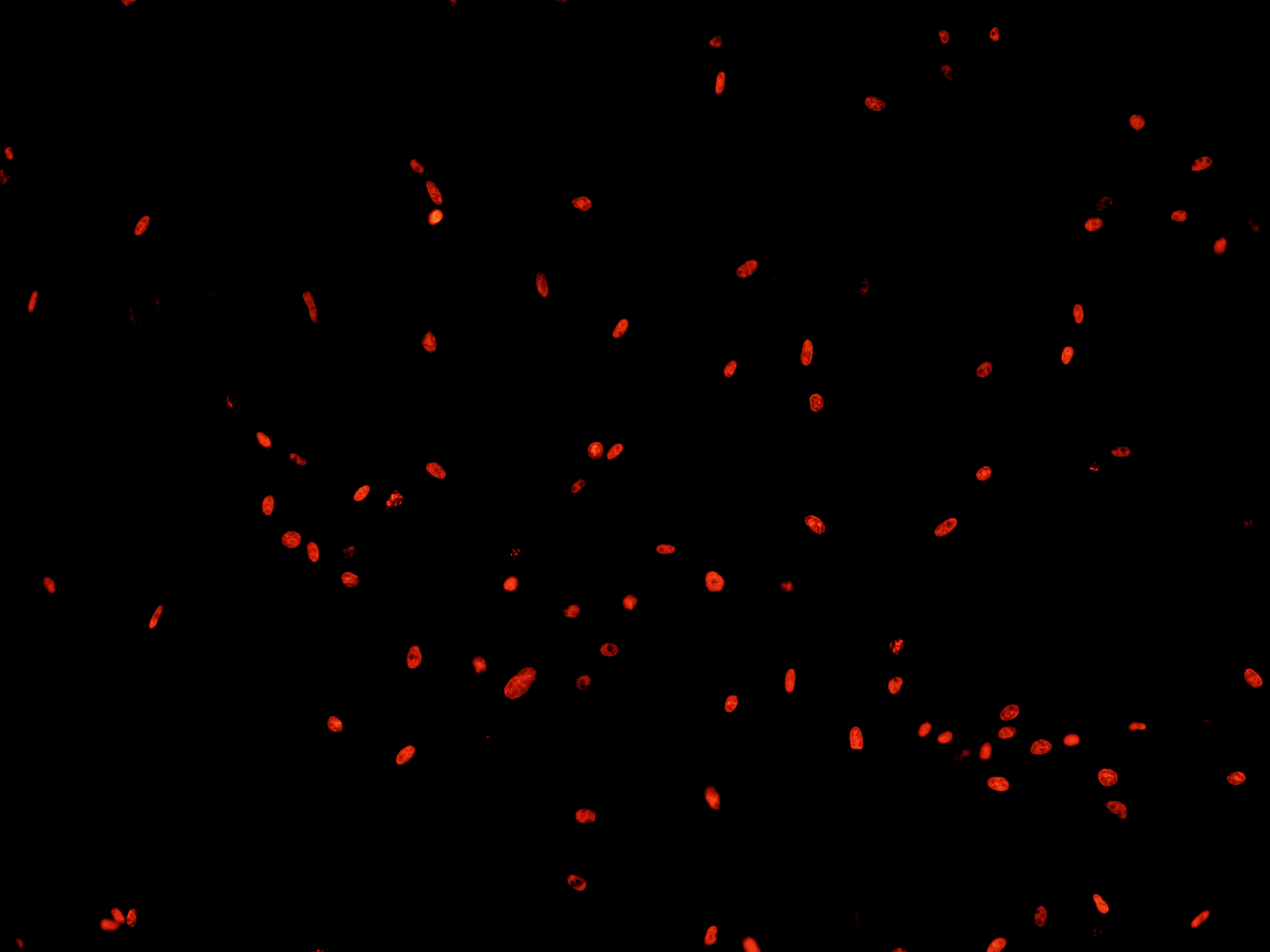

Supplement: Supplementary file 9 [file DataSheet_2.zip › Raw data 2/Figure 13/EDU/U87/U87-vector edu.tif]

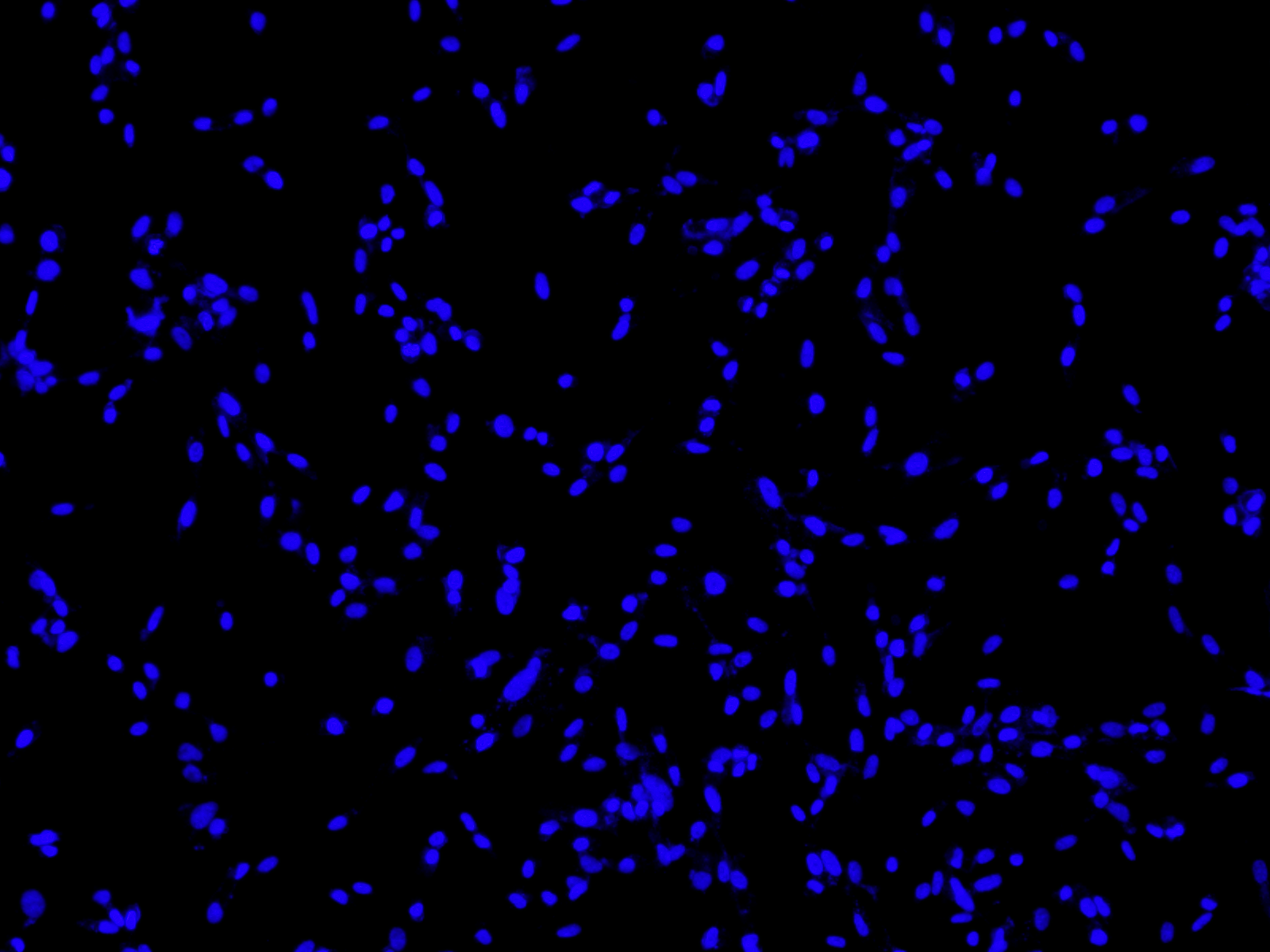

Supplement: Supplementary file 9 [file DataSheet_2.zip › Raw data 2/Figure 13/EDU/U87/U87-vector hochest.tif]

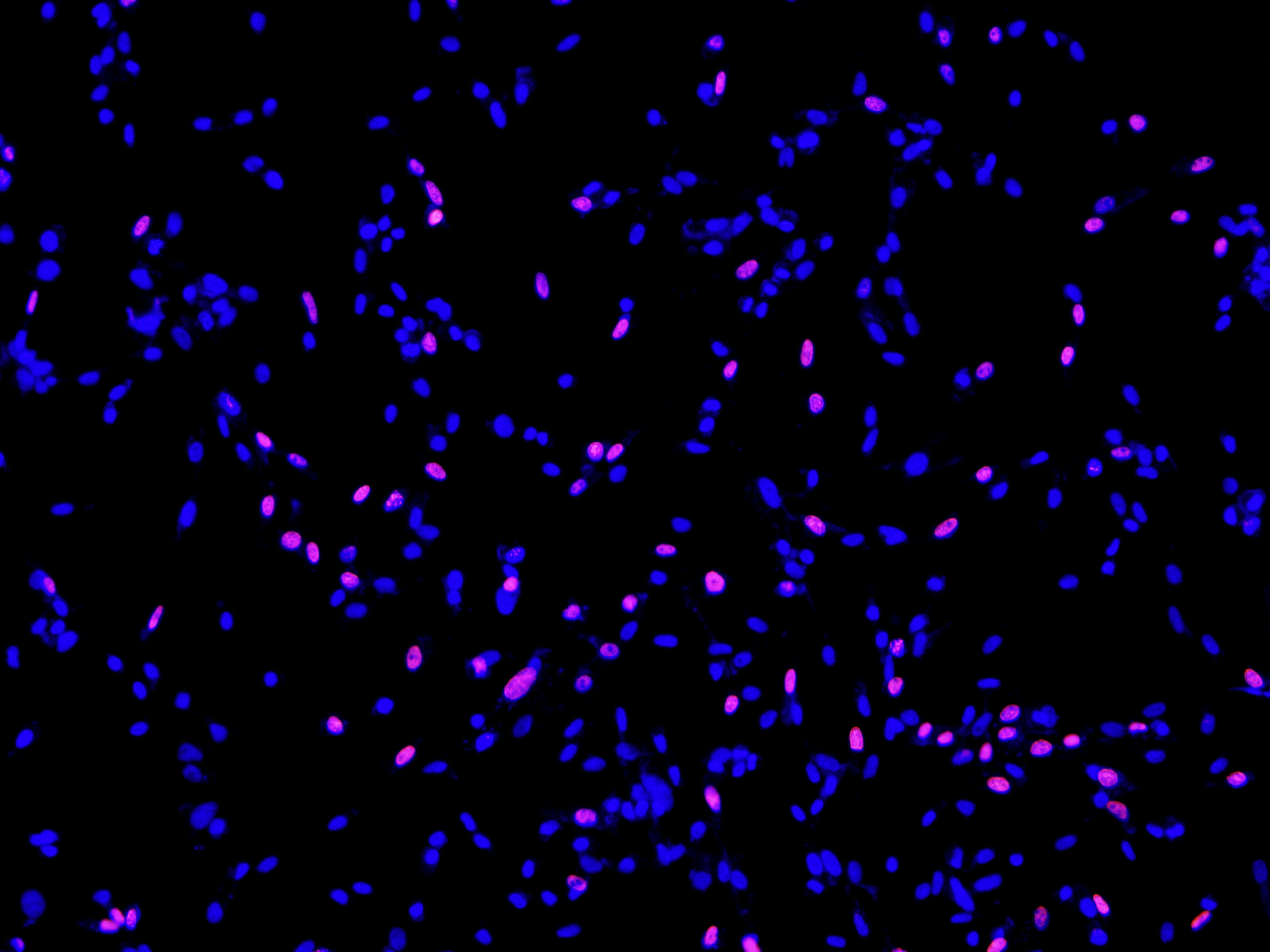

Supplement: Supplementary file 9 [file DataSheet_2.zip › Raw data 2/Figure 13/EDU/U87/U87-vector merge.tif]
