# Supplementary material for: FAIM2 is a potential pan-cancer biomarker for prognosis and immune infiltration
Source: Front Oncol. 2022 Sep 14;12:998336. doi: 10.3389/fonc.2022.998336 (PMC9516132; doi:10.3389/fonc.2022.998336)
Supplement: Supplementary file 8 [file DataSheet_1.zip › Raw data 1/other figures.doc]

Other figures were produced by the online databases, and the relevant information has been described in the Methods section of the manuscript.
