# Supplementary material for: FAIM2 is a potential pan-cancer biomarker for prognosis and immune infiltration
Source: Front Oncol. 2022 Sep 14;12:998336. doi: 10.3389/fonc.2022.998336 (PMC9516132; doi:10.3389/fonc.2022.998336)
Supplement: Supplementary file 8 [file DataSheet_1.zip › Raw data 1/Figure 10/nci60_RNA__RNA_seq_composite_expression.zip!/output/html/RNA__RNA_seq_composite_expression.html]

 
  RNA__RNA_seq_composite_expression 
 
 
 
 return to previous page  CellMiner Address:  https://discover.nci.nih.gov/cellminer/  
Download Normalized Data:  RNA: RNA-seq/composite expression  
CellMiner Database Version: 2.2 
Human Genome Version: HG-19 
Date: 04-02-2020 
Input data: All data 


 Table 1: RNA: RNA-seq/composite expression  

 Fragments per kilobase per million reads log2(FPKM + 1)  
  

  Gene name    Entrez gene id    Chromosome    Start    End    Cytoband   BR:MCF7  BR:MDA-MB-231  BR:HS 578T  BR:BT-549  BR:T-47D  CNS:SF-268  CNS:SF-295  CNS:SF-539  CNS:SNB-19  CNS:SNB-75  CNS:U251  CO:COLO 205  CO:HCC-2998  CO:HCT-116  CO:HCT-15  CO:HT29  CO:KM12  CO:SW-620  LE:CCRF-CEM  LE:HL-60(TB)  LE:K-562  LE:MOLT-4  LE:RPMI-8226  LE:SR  ME:LOX IMVI  ME:MALME-3M  ME:M14  ME:SK-MEL-2  ME:SK-MEL-28  ME:SK-MEL-5  ME:UACC-257  ME:UACC-62  ME:MDA-MB-435  ME:MDA-N  LC:A549/ATCC  LC:EKVX  LC:HOP-62  LC:HOP-92  LC:NCI-H226  LC:NCI-H23  LC:NCI-H322M  LC:NCI-H460  LC:NCI-H522  OV:IGROV1  OV:OVCAR-3  OV:OVCAR-4  OV:OVCAR-5  OV:OVCAR-8  OV:SK-OV-3  OV:NCI/ADR-RES  PR:PC-3  PR:DU-145  RE:786-0  RE:A498  RE:ACHN  RE:CAKI-1  RE:RXF 393  RE:SN12C  RE:TK-10  RE:UO-31  
  DDX11L1  100287102  1  11873  14409  1p36.33  0.199  0  0.381  0  0.079  0.154  0.142  0.04  0.344  0.162  0.131  0  0.13  0.196  0.181  0.306  0  0  0.605  0  0.163  0.538  1.12  0  0  0.107  0.219  0.087  0.317  0.08  0.106  0.118  0.259  0.437  0.16  0.21  0.109  0.088  0.065  0.333  0.162  0.396  0.246  0.118  0  0.056  0  0.102  0.105  0.264  0.11  0.131  0.057  0  0.164  0.136  0.184  1.092  0.176  0  
 
 WASH7P  653635  1  14361  29370  1p36.33  3.088  0.566  1.771  2.129  3.169  1.525  3.244  2.415  2.525  3.033  3.23  2.559  2.503  3.231  2.938  3.04  2.248  0.113  4.149  2.515  2.726  3.278  2.125  2.177  1.992  2.303  3.101  2.655  2.932  2.505  3.266  3.043  2.616  1.238  1.787  2.957  2.981  2.742  2.076  2.713  2.751  2.935  3.346  1.96  1.956  2.212  3.018  2.594  1.868  3.779  2.664  1.997  2.564  1.433  2.846  1.899  1.95  2.267  2.695  2.191  
 
 OR4F5  79501  1  69090  70008  1p36.33  0  0  0.147  0  0  0  0.12  0  0  0  0.1  0  0  0  0  0  0  0  0  0  0  0  0  0  0  0  0  0  0  0  0  0  0  0  0  0  0  0  0  0  0  0  0  0  0  0  0  0  0  0  0  0  0  0  0.263  0  0  0  0  0  
 
 HYDIN2  100288805  1  132568  407510  1q21.1  0  0.03  0.083  0  0.02  0.035  0.026  0.018  0.104  0.022  0.041  0.044  0  0.014  0  0.018  0  0.047  0.051  0.022  0  0.071  0.266  0  0  0.013  0.212  0.059  0  0.062  0.033  0.049  0.023  0.158  0.016  0  0.014  0  0.025  0.039  0.045  0.137  0.101  0  0.031  0.027  0.119  0.07  0  0.036  0.008  0.157  0.015  0.023  0.032  0.016  0.016  0.018  0.039  0  
 
 LOC729737  729737  1  134772  140566  1p36.33  1.303  0.542  0.262  0.457  2.124  0.24  0.458  0.31  0.49  0.611  0.558  0.74  1.077  1.622  1.315  2.265  1.035  0.042  0.492  0.96  1.981  1.063  0.747  0.131  0.205  0.456  0.907  1.021  0.78  0.356  0.573  0.455  1.162  0.389  0.3  2.059  1.695  1.647  0.396  1.236  1.605  0.584  1.413  0.681  0.481  0.316  0.201  1.373  0.531  1.551  0.623  1.688  0.464  0.88  1.132  0.43  0.846  0.697  0.67  0.599  
 
 LOC100132062  100132062  1  323891  328581  5q35.3  0.151  0  0.218  0.467  1.151  0.269  0  0.046  0.079  0  0.407  0.434  0.738  0.901  0.655  1.302  0.819  0  0.129  0  1.039  0.352  0.373  0.106  0.047  0.069  0.264  0.957  0.359  0.018  0  0.236  1.048  0.355  0  0.979  1.112  0.988  0  0.748  1.233  0.028  0.053  0.129  0  0.402  0  0.926  0.1  0.38  0.14  1.041  0.41  0.389  0.726  0.212  0.552  0.997  0.322  0.115  
 
 OR4F29  729759  1  367658  368597  1p36.33  0  0.129  0.224  0  0  0  0  0  0  0  0.122  0  0  0.175  0  0  0  0  0  0  0.28  0.15  0  0  0  0.125  0.346  0.108  0  0.439  0.178  0  0  0.39  0  0  0  0  0.078  0  0  0.362  0.468  0  0  0  0.155  0  0  0  0  0  0.15  0  0.198  0  0.123  0  0  0  
 
 CH17-408M7.1  102724558  1  429709  468683  1q21.1  0  0  0.112  0  0  0.182  0.11  0  0.173  0.052  0.092  0.13  0  0  0  0  0  0  0  0  0  0.108  0.376  0  0  0  0.623  0  0  0.132  0  0.153  0  0.185  0.077  0  0  0  0.058  0  0.244  0.148  0  0  0  0  0.417  0  0  0.136  0  0  0  0.052  0  0  0  0  0  0.12  
 
 LOC101928626  101928626  1  562759  564389  1p36.33  0  0  0.321  0  0  0  0  0.095  0  0  0  0.157  0  0  0  0  0  0  0  0  0  0  0.454  0  0  0  0  0  0  0  0  0  0  0.505  0  0  0  0  0  0  0  0.611  0  0  0  0  0  0  0  0  0  0  0  0  0  0  0  0.247  0  0  
 
 OR4F16  81399  1  621095  622034  1p36.33  0  1.85  0.192  0  0.089  0  0  0  0  0.075  0  0  0  0.244  0  0  0  0.15  0.12  0.158  0  0.171  0.523  0.405  0  0.23  0.298  0.153  0.992  0.366  0.166  0  0  0.374  0  0  0  0  0.172  0  0  0.394  0.337  0  0  0.084  1.966  0  0.219  0  0  0.215  0.15  0  0.224  0  1.079  0  0.143  0  
 
 LOC100133331  100133331  1  661138  665731  1p36.33  0.996  0.265  0.397  0.841  1.686  0.582  0.851  0.955  0.663  1.321  2.895  0.582  1.117  1.3  1.342  1.627  1.099  2.307  1.405  1.015  1.981  1.52  0.871  0.425  0.381  1.066  1.501  1.608  1.351  1.185  1.651  1.376  1.538  0.593  0.697  1.739  1.709  1.57  0.933  1.53  1.504  0.919  1.461  1.379  0.772  2.735  0.953  1.253  1.18  1.602  0.698  1.348  0.972  0.654  4.72  0.51  1.375  1.362  0.653  0.624  
 
 LOC100288069  100288069  1  700244  714068  1p36.33  1.268  0.381  0.513  0.656  1.568  1.291  0.647  0.787  0.403  1.166  0.962  0.487  1.271  1.431  1.08  1.316  0.97  0.278  0.498  1.146  1.814  1.449  0.578  0.812  0.36  1.093  1.132  1.411  0.991  1.009  1.454  1.05  0.938  0.445  0.998  1.359  1.482  1.179  0.496  1.279  1.438  0.788  0.854  1.081  1.012  0.644  0.43  1.344  1.365  0.459  0.817  1.276  1.157  0.826  1.297  0.816  1.406  1.352  0.656  0.853  
 
 FAM87B  400728  1  752750  755214  1p36.33  1.015  0.192  0.641  0.442  0.544  0.53  0.453  0.348  0.225  1.067  0.379  0.094  0.518  0.771  0.4  0.605  0.244  0.276  0.391  1.04  0.389  0.977  0.613  0.275  0.071  0.625  1.336  0.634  0.394  0.995  0.314  0.332  0.256  0.166  0.439  0.17  0.289  0.45  0.61  0.47  0.235  0.514  1.007  0.655  0.204  0.354  0.775  0.446  0.255  0.271  0.336  0.669  0.52  0.42  0.582  0.666  0.685  0.141  0.258  0.151  
 
 LINC00115  79854  1  761585  762902  1p36.33  0.648  0.517  0.514  0.853  0.929  0.888  0.609  0.75  0.246  0.857  0.503  0.222  0.424  0.941  0.548  0.871  0.56  0.575  0.945  1.289  1.044  0.682  0.802  0.476  0.241  0.496  0.78  0.869  0.503  1.008  0.806  0.531  0.772  0.383  0.418  0.374  0.53  0.549  0.673  0.39  0.43  0.773  0.902  0.763  0.727  0.531  0.983  0.948  0.694  1.466  0.343  0.662  0.508  0.476  0.787  0.452  0.759  0.355  0.348  0.27  
 
 LINC01128  643837  1  762970  794826  1p36.33  0.867  1.201  1.259  1.257  1.124  0.571  1.562  1.621  1.075  1.879  1.497  0.774  1.109  1.652  0.957  1.016  0.819  1.122  2.13  0.937  1.594  1.527  1.515  0.998  0.634  1.053  2.122  1.433  1.333  1.063  1.43  1.34  1.721  1.079  0.578  1.002  1.452  1.596  0.732  1.703  0.816  0.85  1.891  1.128  1.529  0.907  1.228  1.052  1.552  1.454  0.699  1.039  1.329  0.578  0.774  0.674  1.614  0.87  1.039  0.869  
 
 FAM41C  284593  1  803450  812182  1p36.33  0.128  0.135  0.307  0  0.258  0.137  0.09  0.075  0.205  0.127  0.109  0.084  0  0.125  0  0.138  0  0.358  0.122  0.177  0  0.165  0.438  0.073  0  0.101  0.243  0.054  0.079  0.261  0.138  0  0  0.212  0.088  0.173  0.086  0  0.164  0  0.093  0.277  0.249  0.154  0  0.122  0.091  0  0  0.179  0.107  0.345  0.094  0.077  0.187  0  0.141  0  0.104  0  
 
 LOC100130417  100130417  1  852197  855072  1p36.33  1.093  0.067  0.435  0.599  2.357  0.359  2.048  1.369  0.609  2.005  1.069  0  0.469  0.492  0.176  1.225  0.209  0  0.694  0.349  0  0.326  0.747  0.357  0.122  0.119  0.365  0.127  0.166  0.197  0.094  0.306  0.197  0.199  0.778  1.22  0.331  0.197  0.513  0.798  1.068  0.806  2.505  0.197  0.658  0.381  0.21  0.234  0.177  1.107  0.216  0.854  1.163  0.98  0.446  0.396  1.577  0  0.132  0.143  
 
 SAMD11  148398  1  861120  879961  1p36.33  1.394  0.512  0.861  0.568  1.595  0.269  3.821  4.806  0.66  3.415  2.238  0.119  0.066  0.72  0.156  0.307  0.176  0.497  0.27  0.275  0.147  0.667  0.948  0.32  0.951  0.133  0.543  0.222  0.12  0.353  0.061  0.442  0.182  0.361  2.183  1.947  0.251  0.163  0.795  1.377  0.6  2.061  2.172  0.132  0.501  0.365  1.267  0.366  0.38  0.503  0.13  1.086  1.466  1.394  0.423  0.41  2.514  0.193  0.342  0.105  
 
 NOC2L  26155  1  879582  894679  1p36.33  2.897  0.662  3.039  4.112  2.919  3.536  4.158  4.064  3.344  3.909  4.532  3.332  4.192  3.56  4.323  3.842  3.821  0.428  4.782  2.851  5.145  2.653  3.481  3.85  3.405  3.087  3.773  4.861  4.491  3.004  4.811  5.412  5.411  3.071  3.087  3.937  5.304  4.061  2.014  5.652  3.756  4.121  3.758  3.952  5.158  3.184  1.958  3.866  3.305  5.013  3.98  3.353  4.218  2.542  2.75  3.075  3.883  3.769  2.969  3.888  
 
 KLHL17  339451  1  895966  901099  1p36.33  2.055  0.453  1.881  2.479  1.895  1.402  2.331  2.091  1.857  2.535  1.937  1.047  2.379  2.774  2.729  2.555  1.525  0.254  3.525  2.219  2.182  2.266  1.533  1.056  1.328  1.079  3.031  1.751  2.359  1.932  2.625  2.455  2.301  0.962  1.393  2.206  2.539  2.17  1.497  2.883  1.782  2.227  2.463  1.313  1.625  0.993  1.674  1.711  1.254  2.842  1.823  2.008  2.019  0.933  2.675  1.227  2.346  1.686  1.543  1.128  
 
 PLEKHN1  84069  1  901876  910484  1p36.33  1.219  0.093  0.516  0.465  1.021  0.356  1.164  1.528  0.44  0.778  1.326  0.145  1.396  0.923  1.522  1.471  0.725  0.133  0  0.066  0.361  0.208  1.047  1.234  1.003  0.218  1.431  0.301  0.489  1.126  0.303  1.551  1.509  0.726  0.859  1.059  1.453  1.757  0.527  1.473  2.331  0.521  1.281  0.507  1.125  1.072  0.64  0.335  0.677  0.513  1.837  1.722  2.493  0.64  2.062  0.82  1.955  0.567  1.494  1.483  
 
 PERM1  84808  1  910578  917497  1p36.33  0.811  0.075  0.403  0.203  0.789  0.37  0.771  0.783  0.414  0.532  0.788  0.192  0.486  0.66  0.707  1.026  0.431  0.092  0  0.109  0.136  0.15  1.246  0.611  0.58  0.291  0.633  0.173  0.335  0.329  0.925  0.997  0.87  0.802  0.558  0.326  0.613  1.016  0.92  0.588  1.128  0.404  0.8  0.281  0.504  0.57  0.829  0.235  0.459  0.683  0.902  0.96  1.462  0.478  1.6  0.449  0.875  0.382  0.779  0.695  
 
 HES4  57801  1  934341  935552  1p36.33  1.662  0.304  3.401  5.878  3.334  2.824  0.28  2.557  3.328  1.975  1.663  0.415  1.777  2.185  1.598  3.281  1.673  0.321  2.019  0.489  0.736  1.318  2.141  1.324  1.258  0.132  1.293  0.57  0.892  0.773  1.836  0.791  1.403  2  1.737  2.075  0.913  0.332  2.44  1.027  2.052  3.348  3.671  1.414  3.332  2.306  2.248  2.999  0.662  4.411  1.167  2.341  1.988  1.502  2.915  1.692  1.681  2.004  1.786  1.304  
 
 ISG15  9636  1  948846  949919  1p36.33  2.571  3.116  3.891  10.06  2.021  4.359  6.308  6.144  3.966  4.712  4.739  3.104  5.39  6.486  3.986  10.322  3.73  1.387  7.202  2.705  2.707  3.744  5.13  7.867  6.571  2.101  3.643  2.46  2.945  1.373  2.83  2.876  4.253  4.561  1.522  3.043  3.875  6.177  6.282  3.729  5.945  4.045  5.44  4.668  4.818  4.689  5.684  3.412  1.418  5.56  4.947  4.352  3.604  5.184  4.1  3.212  6.794  4.702  4.946  3.197  
 
 AGRN  375790  1  955502  991499  1p36.33  2.962  0.176  3.586  4.481  4.335  2.428  3.659  4.778  4.712  4.229  4.176  1.504  3.611  1.857  3.871  5.356  2.336  0.206  1.078  0.618  2.147  0.575  1.221  1.95  2.989  2.72  3.334  2.833  3.101  2.109  3.767  3.658  3.776  2.355  2.965  4.45  5.06  4.664  3.507  4.758  4.914  3.767  2.413  4.186  5.4  4.081  2.883  3.791  3.67  4.513  3.724  4.224  5.242  3.283  4.875  3.943  5.046  3.373  4.216  4.937  
 
 RNF223  401934  1  1007125  1009687  1p36.33  1.257  0.102  0.44  0  1.011  0.25  0.715  0.261  0.227  0.599  0.25  0.158  0.681  0.333  0.485  1.629  0.6  0  0.138  0.285  0  0.319  0.703  0.29  0.158  0  0.435  0.163  0.24  0.391  0.166  0.161  0.185  0.332  0.247  0.141  0.116  0  0.31  0  1.743  0.322  0.166  0  0.791  0.459  0.082  0.172  0.206  0.211  0.321  1.426  0.285  0.56  0.323  0.381  0.204  0.288  0  0.14  
 
 C1orf159  54991  1  1017197  1051736  1p36.33  1.518  0.522  1.678  2.71  2.261  1.835  2.612  2.45  1.889  2.535  2.053  1.429  2.024  1.913  2.003  2.633  2.013  0.237  3.707  1.445  2.214  2.353  1.828  1.64  1.453  0.972  2.344  1.843  2.222  1.715  2.21  2.477  2.627  1.361  1.508  1.791  2.357  2.257  1.575  2.345  2.2  2.398  2.383  1.449  2.889  1.571  1.168  2.001  1.444  2.853  1.485  1.942  2.42  1.17  2.516  1.687  1.822  1.942  1.427  1.864  
 
 LINC01342  254099  1  1072396  1079434  1p36.33  0.616  0.205  0.191  0  0.454  2.191  0.948  0.305  0.265  0.462  0.618  1.872  0.643  0.554  0.419  1.191  0.761  0.602  0.415  1.07  0.423  0.697  0.806  0.369  0.326  0.197  3.388  0.271  0.368  2.991  1.338  1.851  0.57  0  0.705  0.205  0.197  0.404  0.591  0.343  0.207  1.603  0.912  0.342  1.088  1.676  1.307  0.726  0.348  0.099  0.331  1.209  0.538  1.073  0.624  1.137  0.521  0.948  0.369  0.475  
 
 TTLL10  254173  1  1109285  1133313  1p36.33  0.338  0.086  0.461  0.316  0.348  0.122  0.135  0.134  0.373  0.31  0.233  0.223  0.546  0.218  0.36  1.064  0.245  0.277  0.181  0.209  0.122  0.392  0.806  0.175  0.116  0.15  0.48  0.207  0.187  0.428  0.098  0.071  0.103  0.456  0.166  0.102  0.222  0  0.214  0.232  0.359  0.721  0.384  0.12  0.176  0.211  0.103  0.094  0  0.175  0.195  1.137  0.284  0.423  0.313  0.31  0.251  0.093  0.129  0.142  
 
 TNFRSF18  8784  1  1138887  1142089  1p36.3  1.273  0.112  0.249  0  1.154  0.274  0  0.142  0.258  0.19  0.144  0.182  0  3.491  0  0.454  0.13  0.195  0.119  0.156  0  0.327  5.759  4.454  0  0.136  0.286  0.104  0  0.266  0  0  0  0.232  0  0  0.433  0.459  0.323  0.807  0  0.505  0.127  0  0.438  0.224  0.499  0  0  0.37  0.36  1.322  0.161  0.459  0.181  0  0.192  0  0  0.44  
 
 TNFRSF4  7293  1  1146705  1149548  1p36  0  0  0.142  1.92  0.118  0.169  0.143  0.183  0.258  0.209  0.106  0.141  0  0.206  0.593  0.138  0.128  0.132  1.76  0.131  0  1.206  0.616  0  0  0.788  0.477  0.112  0  0.229  0.309  0.131  0.162  0.194  0.225  0  0.165  0.856  0.286  0  0.559  0.534  0.094  0.182  0  0.136  0.095  0  0  0.544  0  0.91  1.084  0.386  0.186  0.171  0.246  0  0  0  
 
 SDF4  51150  1  1152287  1167447  1p36.33  2.857  0.935  3.929  5.297  3.342  4.061  3.868  5.225  4.67  5.329  4.365  3.948  4.392  2.872  3.977  4.237  4.112  0.942  4.222  2.324  3.572  2.845  3.146  4.3  3.164  3.757  3.654  4.229  5.352  2.41  4.821  5.352  4.771  3.647  2.882  4.499  4.465  4.832  3.134  5.065  3.563  4.672  2.336  4.129  4.456  3.458  1.869  3.47  3.54  4.963  4.6  3.731  4.682  3.506  3.871  3.821  4.737  4.366  3.194  4.553  
 
 B3GALT6  126792  1  1167628  1170420  1p36.33  2.106  1.368  2.58  4.176  2.446  2.371  3.077  3.083  3.571  3.052  2.837  1.545  2.103  1.713  1.499  2.812  2.035  2.176  3.921  2.239  1.489  2.909  2.803  1.232  1.45  1.492  2.752  1.508  2.301  1.499  2.502  2.597  2.335  2.009  1.455  2.435  2.593  2.103  1.695  2.896  2.23  2.846  2.937  1.266  2.642  1.762  2.476  1.928  1.564  3.228  2.343  2.537  2.019  1.226  2.272  1.669  1.803  1.792  1.094  1.363  
 
 FAM132A  388581  1  1177825  1182102  1p36.33  0  0  0.214  0  0.348  0  0  0.244  0.272  0.261  0.314  0.178  0.432  0.318  0.141  0.501  0.336  0.417  0.516  0.203  0  0.621  0.499  0.137  0.232  0.159  0.35  0.334  0.243  0.309  0.165  0.201  0  0  0.106  0.149  0.239  0  0.353  0.328  0.283  0.766  0.337  0  0.998  0.426  0.115  0.204  0  0.476  0.256  0.762  0.268  0.429  0.376  0.241  0.274  0.211  0  0.232  
 
 UBE2J2  118424  1  1189291  1209234  1p36.33  2.749  1.982  3.084  3.845  2.881  2.948  3.911  3.68  3.945  3.251  3.75  2.33  2.802  3.366  2.568  2.931  2.67  0.841  4.732  2.326  2.998  3.453  3.435  2.331  2.616  1.921  3.032  2.685  2.859  1.802  3.568  3.674  3.35  2.547  2.132  2.555  3.705  3.113  2.677  3.429  2.809  3.695  3.365  2.214  3.77  2.177  1.321  2.923  2.24  4.757  3.04  2.401  3.333  1.835  3.342  2.407  2.961  3.847  2.194  3.299  
 
 SCNN1D  6339  1  1215815  1227409  1p36.3-p36.2  0.358  0.355  0.292  0.899  0.928  0.369  1.001  1.049  0.977  1.315  0.67  0.211  0.343  0.424  0.901  0.184  0.219  0.102  1.639  0.141  0.08  0.824  0.701  0.157  0.153  0.319  0.807  0.806  0.369  0.744  0.3  0.691  0.55  0.31  0.713  0.664  0.714  0.861  0.672  1.231  0.278  1.122  2.806  0.182  0.624  0.239  2.4  0.622  0.095  2.46  0.641  1.336  0.731  0.518  1.444  0.28  0.303  0.842  0.239  0.175  
 
 ACAP3  116983  1  1227763  1243269  -  1.579  0.131  1.635  1.876  2.158  1.255  2.414  2.995  2.182  3.316  2.409  1.558  2.965  1.316  1.933  2.147  1.753  0.21  4.011  0.779  1.362  2.151  2.279  2.705  0.971  1.633  2.599  2.406  2.413  1.325  3.006  2.793  2.813  1.396  1.071  1.929  2.149  2.389  0.773  1.803  1.584  3.039  3.383  1.162  1.867  1.426  1.154  1.65  0.936  2.598  2.104  1.759  2.14  1.401  1.925  1.058  2.42  1.155  0.965  1.525  
 
 PUSL1  126789  1  1243993  1247057  1p36.33  3.583  2.095  2.957  3.673  2.774  2.931  4.288  3.861  2.816  3.001  3.797  2.917  3.692  4.276  3.022  3.439  3.236  1.097  5.007  3.832  3.386  3.63  3.355  3.09  2.386  1.864  3.749  2.773  3.502  2.882  4.725  4.352  3.953  1.982  2.299  2.915  3.78  3.047  2.174  3.565  3.703  3.48  4.647  2.831  3.397  2.733  4.457  3.709  1.923  4.977  3.199  2.575  3.3  1.502  3.169  2.545  2.343  3.759  2.142  2.84  
 
 CPSF3L  54973  1  1246964  1260067  1p36.33  3.202  1.485  3.145  3.936  3.724  3.301  4.697  3.633  3.766  4.41  4.355  3.599  3.861  4.163  3.607  4.042  3.196  0.766  5.496  3.48  4.106  4.228  3.935  3.716  3.261  2.867  4.688  4.251  4.234  3.847  4.395  4.695  4.65  2.815  3.075  3.187  4.796  3.504  3.219  4.433  3.913  3.911  3.87  3.134  5.094  2.862  3.005  3.54  3.355  5.617  3.554  3.298  3.719  2.256  3.899  3.058  3.538  3.878  2.818  3.274  
 
 CPTP  80772  1  1260142  1264276  1p36.33  1.845  0.324  2.883  4.646  2.567  2.229  3.198  3.055  3.74  3.178  3.545  2.015  3.404  1.773  2.756  2.693  1.828  0.144  3.249  1.27  2.047  1.722  3.301  3.327  1.885  1.738  3.25  3.259  3.402  2.819  3.869  3.307  3.948  2.939  1.68  2.044  3.145  3.154  1.653  3.618  2.984  3.179  3.041  1.597  3.63  2.651  1.515  2.554  2.352  3.253  3.214  2.315  3.051  1.848  2.291  2.223  2.705  2.194  2.155  2.359  
 
 TAS1R3  83756  1  1266725  1269844  1p36.33  0.166  0.069  0.309  0.246  0.36  0.26  0.148  0.374  0.298  0.42  0.234  0.3  0.293  0.198  0.457  0.348  0.195  0.353  0.248  0.259  0.12  0.497  0.905  0.152  0.085  0.097  0.456  0.331  0.155  0.502  0.25  0.21  0.28  0.241  0.25  0.225  0.32  0.336  0.177  0.301  0.404  0.894  0.27  0.192  0.437  0.242  0.393  0.247  0  0.109  0.394  1.569  0.477  0.307  0.366  0.226  0.225  0.244  0.094  0.091  
 
 DVL1  1855  1  1270657  1284492  1p36  3.081  1.54  3.27  4.189  2.675  2.412  3.968  3.83  3.6  3.326  3.637  2.632  3.772  3.994  3.61  3.74  3.377  0.498  3.809  2.693  3.216  2.777  3.292  2.732  2.462  1.726  3.625  3.174  3.446  2.887  3.793  3.815  3.638  2.564  2.395  2.945  4.364  3.95  2.345  3.836  4.346  3.702  3.881  2.915  3.728  2.341  4.172  3.363  3.048  4.669  4.352  2.967  3.985  1.693  3.531  3.279  3.206  3.557  2.617  2.622  
 
 MXRA8  54587  1  1288068  1298921  1p36.33  0.391  0.145  4.918  2.706  4.651  0.445  0.445  4.749  0.484  0.793  0.693  0.29  0.878  0.457  0.911  0.532  0.309  0.579  0.288  0.262  1.401  0.734  0.983  0.332  0.175  0.593  1.024  0.577  0.833  0.722  0.544  0.508  1.14  0.502  0.308  0.688  1.299  4.687  0.302  1.044  1.08  1.072  0.501  0.255  0.951  0.426  0.361  2.079  0.494  3.72  1.232  1.825  0.598  0.788  0.689  0.817  4.71  3.302  3.944  0.179  
 
 AURKAIP1  54998  1  1309109  1310818  1p36.33  4.298  1.388  4.967  6.55  5.518  5.541  6.06  5.323  5.693  5.499  5.311  5.199  5.167  4.868  4.45  5.708  5.523  1.211  6.373  3.717  5.386  5.236  5.847  5.459  4.805  4.095  5.306  4.53  5.428  3.941  6.384  6.026  5.745  5.644  4.968  4.828  5.066  4.858  4.346  4.844  5.453  6.438  6.2  4.637  6.505  5.209  3.799  5.122  4.238  6.933  5.274  4.453  5.395  3.982  5.79  4.899  4.312  5.92  4.328  4.73  
 
 CCNL2  81669  1  1321090  1334718  1p36.33  3.456  2.646  2.871  3.513  3.866  3.098  3.842  4.34  3.995  4.764  4.252  2.154  2.999  4.468  3.553  4.394  3.068  3.377  5.418  3.202  3.409  3.948  4.095  2.884  2.254  2.706  4.374  2.859  4.008  2.665  4.909  4.041  3.481  2.235  2.458  3.799  3.781  4.213  3.222  3.752  3.458  3.717  4.775  3.389  3.439  3.079  3.344  3.074  2.86  5.48  3.704  2.497  3.537  2.251  4.14  2.436  4.105  3.076  2.622  2.798  
 
 LOC148413  148413  1  1334909  1337426  1p36.33  2.428  1.006  1.232  1.949  2.275  1.614  2.751  2.001  2.162  2.537  2.491  2.048  2.434  2.215  2.592  2.471  1.447  1.174  3.347  1.294  2.669  2.254  2.147  2.203  1.395  1.917  2.997  2.387  2.539  2.245  3.212  2.667  2.943  1.11  1.458  1.836  2.892  2.38  0.97  2.758  2.024  1.889  2.97  1.565  2.277  1.806  1.606  2.15  1.637  3.881  2.175  1.616  2.28  1.061  1.805  1.527  1.734  1.927  1.254  1.864  
 
 MRPL20  55052  1  1337275  1342693  1p36.3-p36.2  5.128  6.526  4.118  5.069  5.898  5.64  6.246  5.362  5.03  5.479  6.262  5.362  4.678  6.276  4.802  5.937  5.973  7.287  7.522  7.35  5.934  6.889  5.533  5.416  5.186  4.527  4.882  4.577  5.3  3.891  6.292  6.145  5.362  4.757  5.159  5.197  5.144  4.72  5.906  5.317  5.575  5.891  6.101  5.682  6.386  5.027  5.575  4.446  5.092  6.721  5.394  4.11  5.44  3.876  5.75  4.604  4.774  5.43  4.088  5.11  
 
 ANKRD65  441869  1  1353799  1356824  1p36.33  0  0.29  0.195  0.333  0.212  0.142  0.075  0.163  0.174  0.372  0.235  0.073  0.114  0.137  0.111  0.173  0.155  0.51  0.103  0.183  0  0.333  0.837  0.094  0  0.122  0.332  0.127  0.13  0.231  0  0.089  0.093  0.162  0.099  0.072  0.121  0  0.212  0.282  0.204  0.574  0.156  0.078  0.123  0.194  0.1  0  0  0.081  0.119  1.309  0.078  0.296  0.228  0.1  0.119  0  0  0  
 
 TMEM88B  643965  1  1361507  1363167  1p36.33  0.554  0  0.396  0  0.466  0.598  0  0.402  0  0.452  0.296  0.335  0  0.513  0  0.53  0  0.346  0  0.424  0.634  0.634  0  0.399  0.397  0  0.41  0.272  0  0.51  0  0  0  0  0.417  0.345  0  0  0.521  0  0  1.227  0.339  0  0.682  0.52  0  0  0  0  0.297  1.528  0.193  0.829  0.468  0.467  0  0  0  0  
 
 LOC102724312  102724312  1  1365536  1369953  1p36.33  0.541  0  0.31  0  1.744  0.187  0  0.504  0.142  0.298  0.632  0.148  0.793  0.643  0  1.222  0.176  0  0.115  0.182  0.256  0.209  0.607  0  0  0.111  0.274  0  0  0.23  0  0.139  0  0.197  0.145  0.169  0  0.346  0.068  0.226  0.449  0.507  0.12  0.181  0  0.216  1.276  0  0  0  0.143  1.033  0  0.318  0.146  0  0.211  0.471  0  0  
 
 VWA1  64856  1  1370902  1378262  1p36.33  1.675  0.14  1.128  0.966  3.41  0.473  0.642  3.395  1.351  2.669  2  1.401  2.767  1.644  1.402  3.723  1.083  0.229  0.058  0.189  0.331  0.336  0.839  0.13  0.107  1.595  0.521  0.126  1.521  0.646  0.507  0.216  0.053  0.268  1.344  1.335  0.305  2.249  0.28  1.931  1.928  2.223  1.044  1.799  0.916  0.719  2.762  0.631  0.427  0.967  0.32  1.655  0.111  0.984  0.662  0.896  1.437  1.632  0  0.742  
 
 ATAD3C  219293  1  1385068  1405538  1p36.33  0.591  0.033  0.443  1.074  0.626  0.238  0.441  0.642  0.522  0.761  0.763  0.482  0.792  0.356  0.848  0.73  0.611  0.167  1.053  0.388  0.511  0.559  2.402  1.91  0.369  0.355  1.049  0.832  1.252  0.495  1.004  1.021  1.186  0.492  0.133  0.371  1.164  0.702  0.181  1.231  0.378  0.957  0.583  0.459  0.712  0.397  0.21  0.533  0.232  0.844  0.666  0.664  1.074  0.401  0.423  0.406  0.724  0.247  0.254  0.447  
 
 ATAD3B  83858  1  1407163  1431582  1p36.33  1.564  0.127  1.586  3.51  1.68  2.305  2.484  3.49  1.514  3.009  3.159  2.445  3.81  2.232  3.604  3.144  2.564  0.182  4.806  1.193  3.382  2.163  1.896  3.192  1.733  2.054  2.501  3.28  3.928  2.369  4.27  4.288  4.007  1.65  0.691  3.027  3.895  3.788  0.666  4.99  2.718  3.567  2.889  2.383  3.525  2.608  0.416  2.777  1.475  4.064  2.767  1.986  3.738  1.174  2.306  1.553  3.559  2.91  2.077  2.8  
 
 ATAD3A  55210  1  1447522  1470067  1p36.33  2.693  0.192  2.412  3.73  2.121  2.817  3.682  3.923  2.749  3.879  3.778  3.201  3.713  3.503  3.462  3.393  3.413  0.174  5.273  2.6  3.642  3.136  3.269  3.619  2.868  2.413  3.744  3.961  4.012  2.615  4.959  4.819  4.46  2.151  2.068  3.071  4.312  3.742  1.316  4.217  3.086  3.928  3.383  2.619  3.845  2.676  0.972  3.76  1.931  4.725  3.361  2.495  4.081  1.63  3.157  2.629  3.625  3.587  2.638  3.3  
 
 TMEM240  339453  1  1470157  1475740  1p36.33  0.215  0  0.185  0.476  0.234  0  0.303  0.318  0.233  0.559  0.126  0.1  0.148  0.171  0.148  0.202  0.166  0  0.278  0.118  0  0.312  0.561  0.196  0.793  0.16  0.285  0.309  0.33  0.282  0.213  0  0.318  0.161  0.095  0  0.21  0.217  0.329  0.226  0  0.549  0.23  0  0.673  0.19  0.109  0.294  0  0.136  0.149  1.029  0.327  0.329  0.45  0  0.235  0.294  0  0  
 
 SSU72  29101  1  1477052  1510262  1p36.33  4.722  5.147  4.34  5.86  5.291  4.188  5.348  5.106  5.005  5.027  5.142  4.098  5.073  5.376  4.134  5.049  4.898  3.577  6.101  4.785  4.772  5.33  5.891  4.692  3.911  3.773  4.405  4.32  4.857  3.921  5.676  5.274  5.021  4.578  3.984  4.625  4.272  5.067  4.495  4.807  5.028  4.654  6.069  3.93  4.83  4.281  4.475  3.597  3.918  5.102  5.307  3.35  4.342  3.416  4.458  4.301  4.355  4.599  3.848  4.596  
 
 C1orf233  643988  1  1533387  1535476  1p36.33  3.828  0.281  1.438  4.648  0.118  1.007  0.623  2.567  0.193  4.06  0.059  0.79  3.93  1.719  0.469  0.12  1.971  0.202  1.908  2.057  1.029  1.09  3.702  2.775  0.087  2.118  3.957  1.971  2.901  2.627  3.209  2.603  0.121  0.362  1.435  3.207  1.572  1.646  1.576  4.294  2.637  2.96  3.706  2.092  3.05  2.549  1.035  3.549  2.828  4.789  0.266  2.831  0  0.361  3.015  2.029  1.044  3.165  0.218  1.512  
 
 MIB2  142678  1  1550794  1565990  1p36.33  0.862  0.085  1.322  2.119  0.692  0.619  0.904  2.16  2.452  2.269  1.014  0.511  1.888  1.421  1.482  0.961  1.901  0.121  1.69  0.377  0.473  1.382  1.912  1.275  0.287  0.628  1.741  1.688  1.518  0.729  1.035  1.448  1.772  0.942  0.449  0.946  2.102  1.881  0.547  1.887  0.888  1.521  1.287  0.416  1.643  1.052  0.283  1.593  0.788  1.7  1.391  1.485  1.632  0.799  0.68  0.722  1.679  1.013  0.438  0.794  
 
 MMP23B  8510  1  1567559  1570030  1p36.3  0  0  0  0  0.278  0.158  0.133  0.273  0.192  0.524  0.147  0.155  0  0.143  0  0.111  0.154  0.296  0.156  0  0  0.598  0.439  0.122  0.254  0.079  0.272  0.099  0  0.261  0  0  0  0  0  0  0.165  0  0.315  0.124  0  0.485  0.091  0  0.234  0.451  0.177  0.787  0  1.006  0.204  0.969  0.163  0.352  0.226  0  0.222  0.224  0  0  
 
 CDK11B  984  1  1570602  1655859  1p36.33  2.359  1.736  2.297  3.385  2.71  2.408  3.068  2.938  2.877  3.234  3.534  2.544  2.83  2.954  2.962  3.072  2.889  1.112  4.628  2.38  2.713  3.663  3.099  2.622  2.08  2.375  3.2  3.117  3.161  2.092  3.426  3.341  3.046  1.733  2.209  2.567  3.507  2.633  2.268  3.459  2.67  2.806  3.57  2.366  3.412  1.968  2.414  2.598  2.277  3.809  2.97  2.071  3.192  1.537  3.181  1.886  2.744  3.029  2.192  2.393  
 
 SLC35E2B  728661  1  1592938  1624243  1p36.33  2.395  1.508  1.803  2.586  2.818  1.532  2.102  2.702  2.92  2.918  2.904  1.397  2.621  1.953  3.354  2.61  1.835  3.175  4.372  2.116  2.199  2.988  2.197  1.675  1.842  1.611  2.452  2.124  2.556  1.488  2.748  1.933  2.152  1.48  1.08  2.091  2.752  2.403  1.402  2.835  1.915  1.612  3.268  1.767  2.451  1.361  1.058  1.724  1.792  2.819  2.363  1.599  2.166  1.134  2.247  1.888  2.393  1.783  1.019  2.231  
 
 MMP23A  8511  1  1631377  1633247  1p36.33  0  0.176  0.813  0  0.258  0.232  0.295  0.287  0.528  0.584  0.124  0.2  0.221  0.284  0  0.223  0.301  0.221  0.777  0.237  0  0.577  0.716  0.164  0.276  0.116  0.379  0.243  0.149  0.262  0  0  0.205  0  0  0  0.13  0  1.009  0  0.179  0.705  0.587  0  0  0.432  0.12  3.699  0  4.804  0.277  1.377  0.229  0.319  0.482  0  0.503  0.252  0  0.28  
 
 CDK11A  728642  1  1634169  1655791  1p36.33  1.597  1.75  1.608  2.261  2.055  1.996  2.298  2.175  1.946  2.287  2.533  1.698  2.136  2.348  2.115  2.39  2.096  1.263  3.886  1.73  2.357  2.846  2.364  1.711  1.399  1.428  2.029  2.134  2.284  1.384  2.668  2.57  2.154  1.117  1.671  1.917  2.379  1.801  1.729  2.422  2.055  2.16  2.508  1.692  2.712  1.466  1.211  1.836  1.552  2.878  1.813  1.607  2.287  1.033  2.466  1.22  2.138  2.295  1.484  1.664  
 
 SLC35E2  9906  1  1656276  1677438  1p36.33  2.309  1.891  1.604  2.23  2.855  1.261  1.854  2.079  2.567  2.448  2.55  1.186  2.325  1.739  2.78  2.448  1.406  2.965  3.686  2.245  1.86  2.701  2.129  1.274  1.419  1.466  2.29  1.715  2.419  1.437  2.325  1.7  2.051  1.571  1.004  2.202  2.469  2.247  1.402  2.334  2.169  1.569  2.817  1.532  2.402  1.508  1.441  1.384  1.823  2.176  1.824  1.424  1.743  1.261  2.152  1.641  2.205  1.554  0.727  1.906  
 
 NADK  65220  1  1682670  1711508  1p36.33  2.343  1.901  2.295  3.853  2.534  2.334  3.241  2.647  3.795  3.045  3.12  2.814  3.304  2.713  2.565  3.329  2.682  1.642  4.35  2.577  3.233  3.139  4.148  3.169  1.802  2.472  2.968  2.616  3.397  2.421  3.769  3.345  2.835  1.74  1.779  2.619  3.251  2.958  1.938  3.836  2.842  3.116  3.67  2.316  2.645  1.962  2.573  2.352  2.284  4.148  3.035  2.318  2.388  1.625  2.436  1.93  2.656  2.702  1.887  2.609  
 
 GNB1  2782  1  1716723  1822556  1p36.33  5.066  5.768  5.481  5.837  4.83  5.342  6.456  6.141  5.907  5.927  6.451  4.583  5.459  6.27  5.424  5.239  5.471  6.267  6.869  5.671  4.655  5.198  5.016  4.383  4.789  5.184  6.052  5.416  6.118  4.686  6.2  5.903  5.559  4.018  4.401  5.47  6.467  5.419  5.006  6.236  4.984  4.495  6.827  4.971  5.348  4.609  5.498  5.055  5.476  6.474  6.24  4.332  5.826  3.898  5.662  5.205  5.734  5.471  4.596  5.816  
 
 CALML6  163688  1  1846265  1848733  1p36.33  0  0  0  0  0.231  0.108  0  0.088  0  0.18  0.103  0  0.265  0.214  0.231  0  0.184  0  0  0.109  0  0.15  0.729  0.173  0  0.202  0.824  0.132  0.223  0.109  0  0.296  0  0.209  0  0.223  0  0  0.159  0.21  0  0.672  0.541  0  0  0.124  0.371  0  0  0.162  0  0.638  0  0.334  0.121  0  0  0  0  0  
 
 TMEM52  339456  1  1849028  1850740  1p36.33  2.283  0.377  0.432  0.964  1.459  0.616  0  0.282  0  0.09  0  0.236  2.465  4.025  2.201  1.514  1.973  0.175  0.485  0.453  2.597  0.358  3.792  3.467  0.204  0.484  0.882  0.76  0.463  0.278  0.358  1.024  0.267  0.26  0.609  0.605  1.193  1.18  0.341  2.263  0.267  1.817  2.63  2.542  1.92  1.406  1.917  1.766  0.699  3.337  1.355  1.545  0.394  0.742  1.061  1.047  0.43  1.212  1.286  0.738  
 
 CFAP74  85452  1  1853389  1935276  1p36.33  0.21  0.043  0.295  0.093  0.158  0.133  0.197  0.096  0.159  0.294  0.276  0.108  0.252  0.351  0.291  0.344  0.326  0.105  0.283  0.218  0  0.399  0.356  0.246  0.083  0.132  0.364  0.169  0.35  0.259  0.151  0.074  0.03  0.126  0.169  0.086  0.253  0.041  0.274  0.236  0.071  0.198  0.56  0.057  0.082  0.084  0.135  0.119  0.046  0.245  0.204  0.623  0.318  0.208  0.391  0.193  0.17  0.054  0.109  0.094  
 
 GABRD  2563  1  1950767  1962192  1p36.3  0.155  0.05  0.107  0  0.271  0.092  0.064  0.122  0.24  0.093  0.075  0.093  0  1.351  0  0.096  0.089  0.248  0  0.082  0  0.326  0.635  0  0.062  0.061  0.25  0.076  0.053  0.376  0  0.345  0  0.206  0.089  0.172  0.055  0  0.202  2.125  0.162  0.418  0.605  0.195  0.106  0.117  1.23  0  0  0.127  0.1  1.218  0.107  0.461  0.117  0.157  0.048  0  0  0  
 
 PRKCZ  5590  1  1981908  2116834  1p36.33-p36.2  2.547  0.44  0.233  0  1.953  0.249  0.659  0.194  0.559  1.418  0.898  1.546  2.921  2.939  2.941  2.098  1.968  0.628  0.13  0.285  0.189  0.361  0.653  0.98  0.253  2.267  3.258  2.451  2.725  1.666  2.358  2.776  0.912  0.535  0.966  1.699  2.4  0  0.737  2.527  2.034  0.822  2.186  2.704  2.639  1.676  0.585  2.068  2.208  3.392  2.495  1.766  1.593  1.004  1.953  0.995  0.197  2.234  1.431  2.39  
 
 FAAP20  199990  1  2115898  2144159  1p36.33  3.051  1.127  4.726  5.847  3.576  4.737  5.002  4.515  5.706  4.671  4.352  2.683  3.792  3.987  3.556  4.315  4.234  1.47  5.512  3.155  3.466  4.693  4.325  3.363  3.631  2.97  5.019  3.648  4.622  3.822  5.148  4.901  4.869  4.718  3.192  3.926  4.233  3.974  4.181  4.122  4.193  4.984  5.22  2.375  4.698  3.319  4.309  3.786  2.174  5.179  3.458  3.661  3.765  2.357  4.581  2.997  3.123  5.405  2.399  2.785  
 
 SKI  6497  1  2160133  2241652  1p36.33  1.955  1.565  2.818  3.601  1.801  2.38  2.755  4.111  3.409  3.628  3.281  2.033  2.61  2.068  3.345  2.487  1.917  1.041  2.383  1.899  2.607  1.196  1.271  1.722  2.179  2.935  3.207  2.686  3.84  1.856  3.55  3.558  3.412  2.092  1.581  2.982  3.321  2.967  1.895  3.239  2.544  2.713  3.013  2.842  3.949  2.669  1.185  2.842  2.822  3.04  3.139  2.391  2.655  1.281  2.166  2.226  3.105  2.646  1.571  2.869  
 
 MORN1  79906  1  2252691  2323190  1p36.33-p36.32  0.623  0.129  0.323  0.67  0.596  0.513  0.657  0.826  0.533  1.074  0.776  0.762  0.78  0.642  1.761  0.946  0.701  0.182  1.187  0.292  0.346  0.636  1.163  0.543  0.255  0.537  1.221  1.123  1.781  0.95  0.964  0.677  1.075  0.531  0.651  0.48  0.806  0.587  0.526  1.423  1.079  1.13  1.192  0.438  0.759  0.339  0.353  0.483  0.366  1.14  0.809  1.155  0.824  0.657  0.702  0.549  0.465  0.297  0.384  0.36  
 
 LOC100129534  100129534  1  2281852  2284100  1p36.33  0.37  0.084  0.233  0.327  0.586  0  0.406  0.382  0.466  0.742  0.392  0.724  0.323  0.423  1.085  0.76  0.316  0  0.382  0.351  0  0.521  0.724  0  0.161  0.301  0.698  0.72  0.746  0.334  0.194  0.276  0.229  0.35  0.352  0.078  0.337  0.457  0.381  0.959  0.447  0.381  0.76  0.122  0.589  0.146  0.236  0.264  0  0.775  0.357  0.355  0.231  0.544  0.403  0.145  0.261  0.091  0  0.1  
 
 RER1  11079  1  2323213  2336885  1p36  3.787  2.945  3.298  4.141  4.003  3.446  5.133  4.383  4.193  4.581  4.535  3.688  3.747  4.428  3.863  3.911  3.678  2.218  4.987  3.96  3.387  3.95  3.589  3.049  3.382  3.357  4.559  3.811  4.747  3.651  4.664  4.622  4.578  3.341  2.85  3.607  4.296  3.765  3.97  4.033  3.719  3.457  5.127  3.022  4.051  2.935  4.247  3.199  3.033  5.02  3.987  2.679  4.326  2.418  3.954  3.389  3.829  4.06  2.873  3.697  
 
 PEX10  5192  1  2336240  2344010  1p36.32  1.636  0.219  1.646  2.548  1.444  1.998  1.846  1.963  3.199  2.344  1.997  1.6  2.951  1.182  2.703  2.061  1.928  0.078  2.257  1  1.302  1.182  1.204  1.846  1.365  1.988  1.893  2.041  2.957  1.418  3.313  2.944  2.739  1.577  1.141  2.064  2.559  2.701  0.973  2.896  2.386  2.361  1.767  1.54  1.472  1.543  0.321  1.631  1.538  2.541  2.505  1.863  2.458  0.928  1.816  1.562  1.771  2.402  1.294  1.906  
 
 PLCH2  9651  1  2398897  2436969  1p36.32  0.224  0.048  0.245  0.126  0.116  0.193  0.16  0.183  0.238  0.274  0.125  0.158  0.348  0.2  0.261  0.205  0.132  0.175  0.198  0.098  0.117  0.281  0.741  0.09  0.065  0.081  0.422  0.146  0.113  0.244  0.186  0.05  0.076  0.231  0.183  0.059  0.207  0.045  0.142  0.411  0.225  0.205  0.247  0.095  0.161  0.105  0.071  0.105  0.056  0.171  0.281  1.062  0.105  0.315  0.151  0.152  0.066  0.084  0.072  0.058  
 
 PANK4  55229  1  2439969  2458067  1p36.32  2.107  1.465  1.633  2.349  1.467  1.607  2.028  2.061  1.763  2.214  1.518  1.078  2.136  2.223  2.506  1.812  1.548  1.248  2.828  1.671  2.109  2.546  1.422  1.072  1.318  1.176  2.427  1.764  2.292  1.858  2.583  2.296  2.329  1.14  1.079  1.571  2.26  1.74  1.421  2.143  1.807  1.824  2.577  1.695  1.966  1.232  1.797  2.03  1.811  3.088  2.064  1.869  1.75  0.704  1.679  0.993  1.396  1.87  0.928  1.542  
 
 HES5  388585  1  2460183  2461684  1p36.32  0  0  0.281  0  0.217  0  0.119  0.108  0.248  0.159  0.104  0.132  0.556  0.108  0.163  0.191  0.14  0.314  0.078  0.248  0.248  0.404  0.531  0.131  0  0.089  0.366  0.102  0.102  0.281  0  0.089  0  0.225  0.1  0.103  0.086  0  0.141  0.25  0  0.398  0.122  0  0.504  0.085  0.073  0.316  0  0.483  0.091  1.029  0.065  0.228  0.196  0.105  0  0.191  0  0.125  
 
 LOC115110  115110  1  2481358  2488450  1p36.32  0  0.04  0.169  0.078  0.033  0.03  0.024  0.052  0.225  0.065  0.048  0.193  1.661  0.035  0.691  0.427  0.122  0.062  0.298  0.075  0.063  0.198  0.823  0.284  0.036  1.185  1.863  1.141  1.777  1.141  2.101  1.156  2.157  0.713  0.022  0  0.105  0.108  0.061  0.058  0.023  0.171  0.063  0.195  0.038  0.046  0.211  0.032  0  0.031  0.059  0.464  0.034  0.193  0.065  0.051  0.114  0  0.17  0.125  
 
 TNFRSF14  8764  1  2487803  2497061  1p36.32  0.182  0.177  0.278  1.078  0.792  0.216  0.699  0.341  0.227  0.81  0.121  0.746  2.38  0.031  1.149  1.92  0.691  0.319  3.125  0.317  0  1.94  1.894  1.604  0.477  4.041  4.795  2.871  5.724  4.258  5.87  4.077  4.609  3.013  0.058  0  0.608  1.178  1.013  1.057  0  0.564  0.148  1.078  0.068  0.069  0.601  0.096  0  0.771  0.657  0.394  0.092  0.749  1.298  0.128  1.322  0.912  1.007  1.178  
 
 LOC100996583  100996583  1  2497973  2515973  -  0.278  0  0  0  0  0  0  0  0  0.104  0  0  1.311  0  0.262  0  0  0  0  0.177  0  0.179  0  0  0  0.324  1.093  0.161  0.385  0.141  0.48  0  0  0  0  0  0  0  0.083  0  0  0  0  0.424  0  0.165  0.098  0  0  0  0  0  0  0.194  0  0  0  0  0  0  
 
 FAM213B  127281  1  2517898  2522908  1p36.32  2.654  2.419  1.676  2.156  2.232  2.133  3.202  0.91  2.594  3.102  1.925  1.919  4.571  2.577  3.776  3.754  2.717  2.545  3.145  2.234  1.593  2.77  1.565  1.848  1.37  3.138  3.551  2.866  3.269  1.291  3.884  2.534  3.898  2.233  1.581  1.807  3.091  3.166  1.002  2.443  0.87  2.645  2.593  2.783  2.204  2.082  2.762  2.512  2.614  3.624  3.246  1.81  1.324  1.596  2.832  1.387  2.953  1.862  2.704  2.561  
 
 MMEL1  79258  1  2522080  2564481  1p36  0.238  0.117  0.127  0  0.386  0.112  0.165  0.189  0.145  0.16  0.121  0.204  0.148  0.165  0.145  0.318  0.107  0.284  0.065  0.209  0.157  0.315  0.391  0.133  0.147  0.208  0.509  0.156  0.097  0.253  0.222  0.207  0.154  0.171  0.169  0.149  0.136  0.119  0.09  0.166  0.169  0.509  0.147  0.133  0.269  0.258  0.126  0.2  0.143  0.132  0.162  0.745  0.158  0.248  0.155  0.217  0.079  0.179  0.158  0.163  
 
 TTC34  100287898  1  2572806  2706230  1p36.32  0.143  0  0.094  0.256  0.182  0.365  0.047  0.123  0.333  0.289  0.178  0.081  0.344  0.128  0.279  0.226  0.115  0  0.045  0.063  0  0.254  0.492  0  0  0.16  0.308  0.281  0.157  0.266  0.332  0.255  0.508  0.274  0.124  0.1  0.156  0.345  0.146  0.257  0.089  0.591  0.518  0.086  0.212  0.095  0.042  0.272  0  0.05  0.17  0.807  0.142  0.281  0.265  0.167  0.138  0.116  0  0  
 
 ACTRT2  140625  1  2938045  2939467  1p36.32  0  0  0.304  0  0.079  0  0  0.071  0.227  0.091  0.066  0  0  0.052  0  0.072  0  0.207  0.082  0.128  0  0.174  0.712  0  0  0  0.163  0.049  0.075  0.225  0  0  0  0.148  0  0  0  0  0.098  0  0  0.061  0.095  0  0  0  0.057  0  0  0  0  0.846  0.102  0.186  0.228  0  0  0  0  0  
 
 LINC00982  440556  1  2976180  2984289  1p36.32  0  0  0.138  0  0.071  0  0.468  0.621  1.078  0.92  0.241  0.087  0  0.356  0  0.146  0  0.239  0  0.137  0  0.627  1.059  0  0  0  0.273  0.07  0  0.306  0  0  0  0  0.1  0  0  0  0.52  0  0.221  0.422  1.071  0  0  0.123  0.045  0.418  0  1.219  0.079  1.509  0  0.384  0.105  0  0  0  0  0  
 
 PRDM16  63976  1  2985741  3355185  1p36.23-p33  0.198  0.037  0.192  0.111  0.091  0.106  0.452  0.362  0.878  0.805  0.439  0.113  0.619  0.601  0.299  0.142  0.256  0.636  0.039  0.103  0.025  0.625  0.513  0.061  0.047  0.113  0.217  0.09  0.062  0.195  0  0.076  0.02  0.271  0.116  0.051  0.07  0.049  0.475  0.28  0.048  0.297  0.891  0.115  0.056  0.083  0.135  0.487  0.029  0.696  0.074  0.664  0.055  0.22  0.115  0.119  0.079  0.023  0.029  0.053  
 
 ARHGEF16  27237  1  3371146  3397677  1p36.3  2.193  0  0.199  0.264  2.149  0.156  0.202  1.197  0.59  0.403  0.121  2.417  3.602  2.295  3.197  3.064  2.782  0.258  0.124  0.123  0.114  0.192  0.804  0.199  0  0.862  0.207  0.159  0.156  0.687  0.925  0.214  0  0.198  0.719  1.018  1.236  2.058  0.391  1.565  2.015  1.8  1.446  1.165  2.29  1.091  0.278  1.289  0.335  2.866  0.09  1.491  1.014  0.651  1.451  0.352  1.902  0.113  0.717  0.725  
 
 MEGF6  1953  1  3404505  3528059  1p36.3  1.052  0.414  1.941  3.499  0.832  0.289  1.814  3.657  0.224  0.803  0.324  0.316  0.953  1.062  1.295  0.509  0.88  1.16  2.002  0.689  0.073  0.3  0.612  0.202  0.038  0.79  0.84  0.428  0.393  0.283  0.495  0.088  0.035  0.18  0.698  1.06  0.651  2.834  2.192  0.609  0.574  0.94  2.044  1.192  0.304  0.941  1.624  1.225  1.959  2.235  0.157  1.002  2.415  1.146  2.331  1.513  2.715  0.123  1.617  2.02  
 
 TPRG1L  127262  1  3541555  3546694  1p36.32  2.255  1.155  2.812  3.019  3.204  1.727  3.157  2.41  2.89  3.042  2.552  3.085  3.253  2.871  2.301  3.707  2.807  0.549  3.243  1.793  1.031  2.275  3.286  1.738  1.808  2.485  3.485  3.193  3.673  1.845  3.163  3.004  3.344  2.932  1.408  2.351  2.521  2.266  2.495  2.94  2.315  1.578  2.473  2.458  3.366  2.325  1.618  2.37  2.701  3.751  3.592  1.794  2.797  1.642  2.336  2.152  2.668  2.218  1.807  2.708  
 
 WRAP73  49856  1  3547330  3566671  1p36.3  1.707  2.098  1.735  2.824  2.5  2.057  3.131  1.913  2.685  2.959  2.72  2.045  2.656  2.789  2.872  2.954  3.234  2.766  4.306  3.283  2.573  3.337  2.481  1.776  1.487  1.819  3.163  2.504  2.955  2.254  2.966  2.689  3.528  2.262  1.569  1.886  2.967  2.459  2.246  3.074  2.12  2.562  3.111  1.995  2.933  1.548  2.776  2.551  1.797  3.814  2.519  1.844  1.849  1.077  2.762  1.941  1.875  2.157  2.042  2.18  
 
 TP73  7161  1  3569128  3652765  1p36.3  1.473  0.367  0.154  0.308  1.664  1.054  0.853  0.175  1.199  1.749  1.302  0.49  0  1.913  2.352  1.754  1.146  0.524  1.267  0.454  0.562  1.108  0.497  0.095  0  0.167  1.075  0.124  2.06  0.173  2.153  0.429  0  0.163  0.289  0.064  1.024  0.093  0.273  1.995  1.415  1.215  2.375  0.179  0.551  0.422  1.332  1.336  0.103  1.059  0.179  0.756  0.102  0.174  0.63  0.148  0.669  0.449  1.305  0.154  
 
 TP73-AS1  57212  1  3652547  3663937  1p36.32  0  0.458  0.293  1.542  1.065  1.327  1.239  0.536  0.923  1.347  1.547  0.056  0  0.07  0.064  0.091  0.056  0.114  1.899  0.204  1.613  1.356  0.632  0  0  0.92  0.957  0.036  1.595  0.112  1.72  0.379  0  0.254  0.583  1.029  1.716  0.446  1.094  1.696  0.165  0.893  2.397  1.057  1.947  0.966  0.731  1.318  1.091  2.093  1.578  1.286  0.579  0.568  1.43  0.569  1.448  1.237  0.737  0.964  
 
 CCDC27  148870  1  3668964  3688209  1p36.32  0  0  0.096  0  0  0.073  0  0.044  0.112  0.029  0  0.053  0  0.054  0  0.05  0.063  0.099  0  0.063  0  0.127  0.407  0.086  0  0  0.113  0.053  0  0.12  0  0  0  0.126  0.066  0  0  0  0.063  0  0  0.232  0.084  0  0  0.059  0.041  0  0  0  0  0.433  0  0.121  0.053  0  0  0  0  0  
 
 SMIM1  388588  1  3689324  3692546  1p36.32  1.597  0  0.464  0.91  2.258  0  0  0.238  0.385  0  0.676  0.288  1.593  1.956  0  0.71  0.98  0.453  0  0  3.786  0.39  1.765  1.795  0  1.967  1.604  0.472  2.107  1.626  3.262  0.95  1.765  1.345  1.079  0.897  0.899  1.375  0.84  1.444  0.887  0.448  1.735  1.018  1.662  1.035  1.427  1.025  0  2.879  0.737  1.872  0  0.974  2.614  1.924  1.211  1.8  0.419  0.844  
 
 LRRC47  57470  1  3696783  3713068  1p36.32  3.129  2.605  2.845  4.09  3.265  3.034  4.143  3.609  3.582  3.793  3.886  2.715  3.518  3.724  3.265  3.055  3.324  2.214  4.873  4.298  3.416  4.219  3.604  2.896  2.622  2.672  3.947  3.578  4.062  2.753  3.914  4.119  4.215  3.15  2.736  3.315  3.65  3.332  3.251  4.178  2.913  2.794  4.66  2.693  3.701  2.642  4.009  2.618  2.785  4.404  3.505  2.271  3.646  2.252  3.107  2.643  3.376  3.003  2.44  3.234  
 
 CEP104  9731  1  3728644  3773797  1p36.32  1.114  0.833  1.166  1.387  1.241  0.86  1.775  1.698  1.442  1.719  1.738  0.919  1.366  1.49  1.645  1.154  1.061  0.877  2.173  0.804  1.413  1.514  1.578  1.13  0.742  1.166  1.605  1.462  1.641  0.889  1.708  1.994  1.591  0.955  0.718  1.562  1.85  1.436  0.842  2.017  0.937  1.142  1.872  1.04  1.777  1.102  0.777  1.233  1.64  1.668  2.068  0.553  1.413  0.773  1.064  1.108  1.603  1.04  0.862  1.634  
 
 DFFB  1677  1  3773830  3801993  1p36.3  0.786  0.053  0.64  0.992  0.427  0.686  1.149  0.698  0.749  1.156  1.103  0.749  1.528  0.958  1.266  0.831  1.037  0.289  2.146  1.434  1.191  1.03  1.467  0.793  0.472  0.797  1.182  0.767  1.197  0.556  1.263  0.936  1.522  0.692  0.378  0.501  0.736  0.757  0.352  0.73  0.239  0.573  1.11  0.847  0.981  0.424  0.398  0.499  0.804  1.101  1.067  0.788  0.627  0.368  0.424  0.477  0.611  0.246  0.281  0.638  
 
 C1orf174  339448  1  3805696  3816857  1p36.32  2.961  3.644  2.268  3.457  2.364  2.148  3.216  3.107  2.754  3.245  3.318  2.718  2.694  4.104  2.813  2.751  3.262  3.764  4.734  3.863  3.321  3.534  1.81  2.153  2.548  2.105  2.48  2.641  3.217  2.186  3.685  3.388  3.175  1.968  2.123  2.595  3.41  2.837  2.739  3.663  2.474  2.228  3.878  3.45  3.122  1.892  2.921  2.37  3.239  4.09  3.439  1.909  3.254  1.541  3.174  2.942  3.052  2.876  2.26  3.012  
 
 LINC01134  100133612  1  3816967  3832011  1p36.32  0  0  0.173  0  0.058  0  0.149  0  0.252  0.118  0.08  0.221  0.196  0.067  0.157  0.105  0  0.211  0.088  0.174  0  0.093  0.58  0.243  0  0  0.16  0.16  0.491  0.401  0.206  0.075  0.402  0.366  0.071  0.127  0.076  0.13  0.083  0.118  0.119  0.105  0.4  0  0.237  0.158  0.029  0  0  0  0.074  0.369  0.04  0.25  0.061  0.085  0.244  0.089  0.079  0  
 
 LINC01346  728716  1  4000671  4012643  1p36.32  0  0  0  0  0  0  0  0  0  0  0  0  0  0  0  0  0  0  0  0  0  0  0.542  0  0  0  0  0  0  0  0  0  0  0  0  0  0  0  0.088  0  0  0  0  0  0  0  0  0  0  0  0  0  0  0  0  0  0  0  0  0  
 
 LOC284661  284661  1  4472110  4484744  1p36.32  0  0.038  0.171  0  0  0.083  0  0.054  0.17  0.205  0  0  0  0.028  0  0.035  0  0  0  0.07  0  0.187  0.304  0  0  0  0.125  0.414  0.102  0.102  0.263  0  0  0.163  0  0  0  0  0  0  0  0.184  0.085  0  0  0.034  0.035  0  0  0  0.058  0.264  0  0.073  0  0  0  0  0  0  
 
 AJAP1  55966  1  4715104  4843851  1p36.32  0  0.116  0.137  0  0.063  0.138  1.767  0.079  0.188  1.818  0.151  0.182  0.065  0.07  0  0.082  0.111  0.095  0.265  0.143  0  0.472  0.608  0.137  4.049  0.042  0.337  0.079  0.111  0.255  0  0.054  0  0.177  0.118  0.936  0  0  0.09  0.41  0.08  0.358  0.118  0  0.108  4.061  0.141  0.633  0  1.719  0.073  0.823  1.519  0.276  0.263  0.12  0.374  0  0  2.962  
 
 NPHP4  261734  1  5922867  6052533  1p36  1.29  0.365  0.941  1.266  1.097  0.546  1.387  1.706  0.991  1.769  1.167  1.159  1.196  1.259  1.142  1.316  1.06  0.562  1.794  1.322  1.391  1.833  0.951  0.905  0.545  1.472  2.071  2.046  2.397  1.38  1.873  2.141  2.047  0.909  0.737  1.092  1.563  1.126  0.668  1.88  0.886  2.185  1.169  0.958  1.145  0.706  0.731  0.862  1.255  1.671  1.428  1.276  1.336  0.581  1.106  0.961  1.116  0.902  0.599  0.745  
 
 KCNAB2  8514  1  6052357  6161253  1p36.3  1.107  0.096  0.404  1.006  0.755  0.13  1.889  1.552  1.185  2.265  2.889  3.369  2.909  1.493  1.511  0.89  1.502  1.76  4.733  4.946  0.6  3.99  2.885  3.676  3.304  5.34  4.745  4.171  6.58  5.137  6.415  2.91  1.032  1.306  1.548  0.495  1.428  3.387  1.612  1.761  1.169  3.221  1.37  0.111  1.15  1.037  1.21  1.131  0  1.202  2.333  1.381  0.499  1.548  3.063  0.417  0.767  2.863  1.783  1.231  
 
 CHD5  26038  1  6161846  6240194  1p36.31  0.346  0.062  0.214  0.143  0.291  0.145  0.28  0.169  0.221  0.23  0.244  0.237  0.125  0.717  0.124  0.161  0.396  0.298  0.142  0.486  0.08  0.644  0.625  0.134  0.165  0.093  0.756  0.146  0.315  0.388  0.279  0.227  0.137  0.27  0.248  0.084  0.248  0.091  0.29  1.043  0.142  0.487  2.29  0.049  0.241  0.241  0.425  0.161  0.173  0.162  0.273  0.796  0.156  0.296  0.359  0.238  0.107  0.087  0.107  0.165  
 
 RPL22  6146  1  6245079  6259679  1p36.31  4.516  6.393  4.029  4.834  5.582  5.426  5.087  4.497  5.401  5.162  5.204  5.12  4.545  5.635  3.688  5.672  6.246  8.2  6.04  6.88  5.427  6.078  5.649  4.802  4.778  3.954  4.437  3.541  4.6  3.548  5.659  5.132  4.193  4.393  4.816  4.209  4.446  4.433  5.774  4.585  4.563  5.227  5.753  4.839  5.832  4.86  4.977  4.108  4.654  6.996  4.89  3.443  4.571  3.637  5.705  4.684  4.096  5.242  3.811  4.85  
 
 LOC102724450  102724450  1  6264899  6265840  1p36.31  0  0.439  0  0  1.236  0  0.574  0  0  0  0.641  0  0.476  0.968  0  0.703  0.738  0  0  0.862  1.436  0  2.256  2.007  0  1.278  1.03  0.764  2.96  0.156  3.292  2.693  2.493  1.383  0.837  2.199  0.496  0  0.66  0  0  0.584  0  0  0  0.768  1.764  0  0  1.557  0  0.759  0.281  0.226  0.691  0  0  1.118  0  0  
 
 RNF207  388591  1  6266188  6281359  1p36.31  1.097  0.489  0.73  0.3  1.502  0.14  0.367  0.302  0.107  0.239  0.993  0.137  1.068  2.253  1.878  0.891  0.979  0.733  0.1  0.508  0.771  0.156  1.175  1.113  0  0.62  1.911  1.013  2.168  0.702  2.132  1.934  2.04  0.811  0.967  2.515  1.218  0.664  1.532  1.532  2.246  1.489  3.263  0.976  0.735  1.344  2.542  1.405  0.974  3.457  1.301  1.435  2.086  0.9  1.821  1.045  2.28  1.043  0.835  0.608  
 
 ICMT  23463  1  6281252  6296044  1p36.21  3.105  2.491  3.329  3.245  3.526  3.25  4.402  3.932  3.176  4.128  4.071  3.073  3.445  3.814  3.063  3.619  3.315  1.413  3.488  3.176  2.601  3.611  2.807  2.451  2.488  3.204  4.085  2.99  4.22  2.957  4.312  3.688  3.732  2.155  2.499  3.422  3.884  3.744  3.215  4.043  3.44  2.619  4.288  2.955  3.695  3.075  3.763  2.998  3.596  4.114  4.18  2.806  3.6  1.875  3.595  2.832  3.427  2.537  2.661  3.593  
 
 LINC00337  148645  1  6296299  6299502  1p36.31  0.609  0.154  0.204  0.209  0.437  0  0.077  0.063  0.209  0.078  0.393  0  0.36  0.697  0.342  0.115  0.295  0.126  0.21  0  0  0.707  0.85  0  0.103  0.365  0.417  0.361  0.403  0.895  0.493  0.235  0.316  0.34  0.153  0.521  0.258  0.228  0.048  2.027  1.423  0.33  2.084  0.099  0.225  0.427  1.306  0.695  0.138  1.701  0.151  0.152  0.073  0.071  0.073  0.116  0.359  0  0.164  0.104  
 
 HES3  390992  1  6304251  6305638  1p36.31  0  0.173  0  0.552  0.164  0.495  0  0  0.599  0.196  0  0.249  0  0.296  0  0.161  0.178  0.856  0  0  0  0.473  1.063  0.246  0  0  0.424  0.183  0  0.346  0.262  0  0  0.411  0  0  0  0  0.283  0  0  0.907  0.307  0  0  0  0.092  0  0  0  0  1.323  0  0.292  0.12  0.347  0  0.346  0  0  
 
 GPR153  387509  1  6307405  6321035  1p36.31  1.067  0.102  0.776  0.883  1.113  0.458  1.311  1.356  1.769  2.726  1.039  1.096  1.678  0.873  0.498  0.922  0.707  0.209  0.724  0.137  0.19  0.766  0.702  0.262  0.873  2.66  1.442  1.068  2.763  1.518  2.86  2.342  2.231  1.417  0.335  1.097  0.783  1.988  0.888  2.317  0.841  1.337  3.601  0.521  0.752  0.683  0.515  0.79  1.082  1.829  4.83  1.388  0.137  0.71  1.42  0.72  0.739  0.916  0.398  0.372  
 
 ACOT7  11332  1  6324331  6453826  1p36  3.411  4.728  4.777  4.633  4.35  4.896  6.421  5.339  4.477  5.396  6.188  5.485  4.592  6.004  4.765  4.069  4.968  4.183  6.778  4.855  4.272  5.625  3.34  4.627  4.439  5.506  7.334  6.333  7.491  5.306  6.553  6.534  6.633  4.8  3.801  4.263  6.19  5.777  4.127  6.029  5.022  4.642  7.685  4.575  5.492  4.394  4.98  5.314  4.605  6.52  5.697  3.699  5.768  3.409  5.192  4.752  5.057  5.226  4.476  5.214  
 
 HES2  54626  1  6475293  6479979  1p36.31  0.326  0.076  0.203  0.124  0.183  0.033  0.045  0.045  0.206  0.068  0.297  0.563  0.165  1.049  0.165  0.043  0.064  0.49  0.022  0.076  0  0.146  0.793  0  0  0.411  0.168  0.088  0.072  0.408  0.232  0.047  0  0.266  0.069  0.113  0.038  0.209  0.235  0.425  0.545  0.265  1.649  0.213  0.704  0.639  0.158  0  1.447  0.113  1.296  0.465  0.038  0.211  0.191  0  0.038  0.028  0.067  0.301  
 
 ESPN  83715  1  6484847  6521004  1p36.31  1.362  0.067  0.109  0  3.087  0.077  0.064  0.072  0.397  0.127  0.163  0.811  0.85  2.225  0.277  0.238  0.145  0.494  0.039  0.144  0.162  0.365  0.734  0.418  0.049  0.292  0.255  0.285  0.069  0.227  0.085  0.139  0  0.204  0.104  0.142  0.104  0.539  0.167  0.96  0.188  0.372  0.135  0.104  1.728  0.672  0.58  0.141  0.991  0.5  1.16  1.053  0.045  0.323  0.17  0.204  0.117  0.081  0.089  0.1  
 
 TNFRSF25  8718  1  6521213  6526255  1p36.2  2.544  0.481  1.271  0.579  1.89  0.204  1.819  1.702  2.026  0.927  1.778  1.004  1.984  2.113  2.32  1.624  1.123  0.217  1.316  0.198  0  1.423  3.146  2.08  0.89  0.506  0.664  0.319  0  0.311  0.232  0.72  0.217  0.382  0.416  0.458  2.173  2.798  1.887  1.144  0.801  2.065  2.007  0.955  2.584  1.594  1.31  1.26  1.783  2.476  2.365  1.878  1.296  0.919  2.624  1.525  1.656  1.146  0.619  1.143  
 
 PLEKHG5  57449  1  6526151  6580121  1p36.31  1.563  0.055  0.904  0.259  1.201  0.436  1  1.67  1.024  0.539  1.576  0.795  1.857  1.507  2.245  1.029  1.023  0.263  0.171  0.129  0.094  0.644  0.7  0.382  1.338  0.742  0.511  0.264  0.56  0.4  0.41  0.658  0.494  0.398  0.399  0.495  1.691  2.303  0.7  0.955  1.064  1.14  0.762  1.155  2.416  1.42  0.941  1.3  1.643  2.244  2.573  1.606  1.028  0.713  1.737  1.253  1.519  1.34  0.485  1.584  
 
 NOL9  79707  1  6581406  6614658  1p36.31  1.429  1.943  1.48  1.414  1.701  1.904  2.168  2.1  1.378  1.992  2.477  1.293  1.754  2.383  2.232  1.743  1.431  2.436  2.065  1.754  2.847  2.206  1.769  1.614  1.72  1.585  1.704  1.676  2.335  1.34  2.408  2.326  1.986  1.092  1.373  1.884  2.476  1.936  1.303  2.571  1.468  1.274  2.741  1.988  2.971  1.675  1.461  1.641  1.844  2.697  2.145  1.295  2.342  0.893  1.722  1.607  1.954  1.783  1.877  2.267  
 
 TAS1R1  80835  1  6615337  6639817  1p36.23  0  0.082  0.203  0  0.077  0  0.056  0.028  0.18  0.139  0.064  0  0.064  0.082  0  0.035  0  0.122  0.039  0.122  0.089  0.268  0.687  0  0  0.062  0.179  0.059  0.077  0.088  0.103  0  0  0.344  0  0  0.082  0  0.086  0.125  0  0.112  0.245  0  0.07  0.056  0.065  0  0  0.076  0.063  0.709  0.03  0.112  0.107  0  0  0  0  0  
 
 ZBTB48  3104  1  6640050  6649340  1p36.3  1.334  0.15  0.939  1.905  1.421  0.91  2.069  1.705  1.893  1.963  2.332  1.1  1.395  1.891  1.906  1.745  1.615  0.067  2.432  2.053  1.885  2.05  1.936  1.895  0.893  1.102  1.848  2.09  1.946  1.387  1.937  2.529  1.982  0.892  1.006  1.456  2.276  1.955  1.028  2.621  1.545  1.99  3.076  1.341  2.568  1.51  1.33  1.532  1.329  3.041  1.601  1.35  1.681  0.572  1.486  0.911  1.289  1.178  1.178  1.097  
 
 KLHL21  9903  1  6650783  6662958  1p36.31  1.723  1.757  2.244  2.46  1.677  1.988  4.002  3.835  3.14  3.605  3.506  1.352  2.308  2.855  2.623  2.155  2.773  1.713  2.852  2.787  2.414  2.133  2.788  2.288  1.961  2.837  5.015  4.57  4.908  2.129  3.433  3.945  4.312  2.418  1.075  1.991  3.521  3.368  1.705  2.999  1.27  2.356  3.731  2.118  2.447  2.295  2.204  1.757  1.621  2.836  3.189  1.961  2.384  1.332  1.597  1.502  2.709  1.272  1.921  2.527  
 
 PHF13  148479  1  6673755  6684093  1p36.31  1.747  1.343  1.856  2.568  1.915  1.925  1.632  2.661  2.048  2.423  1.966  0.861  1.358  1.989  1.763  2.15  1.499  0.566  1.974  0.985  2.114  1.536  1.529  0.836  1.235  1.24  1.497  1.566  1.838  0.693  1.763  1.724  1.72  1.249  0.786  1.275  2.033  1.379  1.028  2.528  1.038  1.196  3.094  1.599  2.097  1.406  1.507  1.355  2.194  2.265  1.667  1.217  1.502  1.065  1.408  1.361  1.682  1.938  1.039  1.664  
 
 THAP3  90326  1  6684924  6695646  1p36.31  1.48  0.997  2.774  4.341  2.65  2.515  3.781  2.189  4.02  3.252  3.069  1.344  1.797  1.945  1.601  2.784  2.833  0.642  3.762  2.717  1.707  3.284  3.412  1.52  1.681  1.439  3.477  1.244  2.54  2.55  3.652  3.155  2.113  2.322  1.829  2.075  2.468  2.863  2.645  2.326  2.162  3.909  3.853  1.686  2.716  1.275  2.344  2.484  1.719  4.473  2.073  1.478  2.297  1.114  2.852  2.098  1.709  2.189  1.697  1.66  
 
 DNAJC11  55735  1  6694227  6761966  1p36.31  2.736  0.937  1.826  2.766  2.284  2.325  3.06  3.219  2.168  3.008  3.572  2.864  3.315  3.223  3.219  2.754  2.2  0.347  2.662  2.451  3.903  2.51  1.246  2.493  2.27  2.717  3.197  2.972  4.011  2.38  3.432  3.817  3.64  1.363  2.082  3.039  3.692  2.816  1.219  3.957  2.801  1.798  3.117  3.268  4.101  2.982  2.132  2.693  3.083  3.525  3.427  2.293  3.379  1.714  2.943  2.205  3.129  2.397  2.827  3.342  
 
 CAMTA1  23261  1  6845383  7829766  1p36.31-p36.23  3.633  5.856  3.551  3.501  3.75  4.306  4.346  4.225  3.82  4.603  4.088  3.429  2.813  4.403  2.714  4.069  4.509  6.886  5.357  4.225  3.65  4.139  3.664  3.415  2.836  3.372  3.207  2.539  3.955  2.123  4.493  4.044  3.495  3.254  3.174  3.479  3.575  2.911  3.513  3.691  2.805  3.456  3.944  3.107  4.257  4.065  4.028  3.029  4.388  5.387  2.875  2.648  3.349  2.005  3.87  3.158  3.236  4.001  3.455  3.446  
 
 VAMP3  9341  1  7831328  7841492  1p36.23  3.255  4.472  4.157  4.513  3.871  3.331  5.218  4.143  4.108  4.906  4.338  2.726  3.751  4.697  3.225  4.131  3.836  2.491  4.46  3.032  2.595  2.726  3.031  1.886  3.457  3.865  4.031  3.507  4.904  2.729  4.994  4.514  3.842  3.359  2.669  3.734  3.597  4.053  4.071  4.306  3.045  2.688  4.763  3.366  3.773  3.631  3.6  3.245  4.056  5.391  4.841  2.772  4.223  2.969  4.351  3.239  4.48  3.881  4.708  4.429  
 
 PER3  8863  1  7844488  7905240  1p36.23  2.008  1.248  1.007  1.624  1.631  0.789  1.556  2.883  1.902  3.263  1.343  0.991  1.018  0.888  0.446  0.634  0.285  0.455  0.829  0.301  1.515  1.862  0.291  0.036  0.202  0.76  1.973  0.937  2.511  1.678  2.045  0.654  2.262  1.109  0.468  0.371  1.294  1.175  0.567  1.23  1.737  0.559  1.352  0.359  1.272  1.031  0.611  0.582  2.461  0.755  1.419  0.383  1.637  0.512  0.594  1.003  1.879  0.8  1.365  0.444  
 
 UTS2  10911  1  7907671  7913551  1p36  0  0  0  0  0  0  0  0  0  0  0  0  0  0.961  0  0  0  0  0  0  0  0  0  0  0  0  0.36  0  0  0  0  0  0  0  0  0  0  0  0.099  0  0  0  0  0  0  0  0  0  0  0  0.437  0  0  0  0  0  0  0  0  0  
 
 TNFRSF9  3604  1  7975930  8003225  1p36  0  0.238  0.469  0.073  0  0  0.856  2.399  0.122  0.081  0.395  0.023  0  0.08  0  0.024  0.058  0.077  0.018  0.06  0  0.015  0.436  0  0.237  0  0.05  0  0  0.034  0  0  0  0.266  0.185  0.039  0.603  0.544  0.584  0.054  0.045  0.23  0.024  0.052  0.049  0.083  0.031  0.114  0.025  0.225  0.203  0.128  3.116  0.704  0.63  0.08  2.281  0.031  0.597  0.38  
 
 PARK7  11315  1  8021713  8045342  1p36.23  5.418  6.951  5.436  5.998  6.556  5.838  7.253  6.531  5.888  6.6  6.662  5.926  5.787  6.995  5.139  6.19  6.435  6.843  7.672  7.464  6.246  6.599  6.506  6.045  5.362  5.385  6.136  5.05  6.502  4.68  7.175  7.025  6.217  5.114  5.595  5.82  6.229  6.226  6.173  6.355  5.908  5.971  7.058  6.01  6.261  5.859  6.185  5.255  6.025  7.457  6.031  4.773  6.144  4.706  6.42  5.613  5.694  6.629  6.212  5.938  
 
 ERRFI1  54206  1  8071778  8086393  1p36  1.43  5.953  3.013  4.621  2.11  2.373  3.958  3.885  5.119  2.866  4.674  2.563  2.727  6.245  3.356  3.682  5.334  5.136  0.117  0.103  0.225  0.349  0.52  0.194  4.901  1.121  0.892  2.47  1.767  0.677  1.136  3.28  2.392  2.482  3  2.664  4.34  3.586  1.544  5.613  4.858  2.025  1.843  2.332  2.427  2.731  5.768  2.474  2.537  4.26  5.771  3.288  4.21  2.665  2.717  2.637  5.027  2.53  2.931  2.443  
 
 LOC102724539  102724539  1  8268731  8275268  1p36.23  0  0.294  0  0  0  0  0  0  0  0  0  0  0  0.303  0  0.253  0  0  0  0  0  0  0  0  0  0  0  0  0  0  0  0  0  0  0  0  0  0  0  0  0  0.228  0  0  0  0.188  0  0  0  0  0  0  0  0  0  0  0  0  0  0  
 
 SLC45A1  50651  1  8378144  8404227  1p36.23  0.135  0.043  0.394  0.912  0.172  0.355  1.163  0.298  1.406  0.871  0.618  0.067  0.102  1.411  0.371  0.165  0.232  0.359  0.064  0.189  0.079  0.35  0.53  0  0.047  0.125  0.269  0.078  0.048  0.186  0.058  0  0  0.304  0.883  0.436  0.495  1.07  0.186  2.911  0.047  0.799  0.245  0.149  0.248  0.167  0.333  0.076  0  0.034  0.577  1.287  0.168  0.158  0.219  0.234  0.3  0.647  0  0.221  
 
 RERE  473  1  8412463  8877699  1p36.23  3.313  1.672  3.12  3.652  3.045  2.64  3.538  3.87  3.591  4.148  3.656  2.473  3.044  2.745  3.001  3.213  2.508  2.251  2.17  2.151  2.738  2.181  2.799  3.255  2.411  3.33  4.167  3.473  4.606  3.282  4.162  4.446  4.36  2.499  2.367  3.045  4.036  3.429  2.323  3.922  3.193  2.303  5.504  2.469  4.129  2.832  2.494  2.815  2.527  2.771  3.126  2.59  3.368  1.889  3.132  2.692  3.757  2.607  2.931  3.32  
 
 LOC102724552  102724552  1  8484704  8495076  1p36.23  0  0  0  0  0  0  0  0.153  0  0.279  0.159  0  0  0.12  0  0.213  0  0  0  0  0  0.118  0  0  0  0  0.273  0.119  0.306  0  0  0  0  0  0  0  0.176  0  0.111  0  0.18  0  0  0  0  0.285  0.648  0  0  0.182  0  0  0  0  0.138  0  0  0  0  0  
 
 ENO1  2023  1  8921058  8939151  1p36.2  7.236  6.701  8.644  9.027  7.867  7.777  9.515  8.663  9.175  9.097  9.75  7.902  7.861  8.573  7.974  8.206  8.897  5.352  9.32  7.569  8.066  7.277  7.703  7.692  7.756  6.718  9.083  8.349  9.41  8.192  9.227  9.62  8.769  6.955  7.683  8.522  9.229  8.65  7.441  8.762  8.001  8.973  8.605  8.08  8.747  8.18  7.498  8.034  8.515  10.212  9.172  7.792  8.769  6.546  8.555  7.882  9.04  8.668  9.059  8.653  
 
 MIR6728  102465436  1  8926560  8926649  -  0  0  0  0  0  0  0  0  0  0  0  0  0  0  0  0  0  0  0  0  0  0  0  0  0  0  0  0  0  0  0  0  0  0  0  0  0  0  0  0  0  16.881  0  0  0  0  0  0  0  0  0  0  0  0  0  0  0  0  0  0  
 
 ENO1-AS1  100505975  1  8938893  8939943  1p36.2  0.537  0.505  0  0  1.421  0  0  0.24  0.381  0.504  1.005  0  0.383  0.831  0.445  0.659  0.609  0.934  1.008  0.493  0  0.485  1.727  0.902  0.572  0  0.15  0.172  0.708  0  1.319  0.553  0  0.605  0.281  0.799  0.434  0.452  0.166  0  0.675  0.289  0  0  0  0.334  0.678  0  0.554  1.118  0.262  0  0  0.352  0.43  0  0  0  0.606  0  
 
 CA6  765  1  9005892  9035148  1p36.2  0  0  0  0  0  0  0.181  0  0.575  0.121  0.126  0.227  0  0.067  0  0  0  0.344  0  0  0  0.143  0  0  0  0  0.196  0.094  0.132  0.189  0  0  0  0.215  0  0  0  0  0.146  0  0  0.496  0.266  0  0  0  0.061  0  0  0  0.101  0.402  0.081  0.106  0.094  0  0  0  0  0  
 
 SLC2A7  155184  1  9063358  9086404  1p36.2  0  0  0.126  0  0  0  0.054  0  0.117  0.106  0.099  0  0  0.082  0  0.087  0  0.085  0  0.084  0  0.12  0.158  0  0  0  0.268  0.047  0.088  0.164  0  0  0  0.231  0  0  0  0  0.138  0  0  0.108  0.191  0  0  0.049  0.077  0  0  0  0  0.524  0.05  0.211  0.097  0  0.063  0  0  0  
 
 SLC2A5  6518  1  9097004  9129887  1p36.2  0  0  0.947  0.918  0.053  0.176  0.15  0.859  0.32  2.556  0.136  0.099  0  0.202  0  0  0.086  0.167  0.045  3.144  0  0.23  0.577  0.832  0.106  0.091  0.302  0  0  0.264  0.175  0.099  0.12  0.308  0  0.673  0.159  0.147  0.105  0  0  0.259  0.194  0  0.125  0.143  0.087  0.119  0  0  0.093  0.444  0.309  0.268  0.118  0.165  0.269  0.135  0  0  
 
 GPR157  80045  1  9164475  9189229  1p36.23  0.719  0.172  0.514  0.647  0.896  0.186  0.154  1.057  0.344  1.006  0.821  1.057  0.414  0.542  1.019  0.525  0.25  0.398  0  0.249  0  0.684  0.518  0.374  0.231  0.406  0.714  1.107  1.902  0.828  0.811  0.559  1.292  0.443  0.231  1.378  1.008  0.64  0.305  0.512  0.452  0.769  0.279  0.142  1.369  0.473  0.305  0.411  0.435  0.122  0.786  1.547  1.141  0.593  0.388  0.207  0.897  0  0.593  0.275  
 
 H6PD  9563  1  9294862  9331394  1p36  1.15  0.584  1.112  2.168  1.188  1.04  1.356  2.646  2.009  3.65  1.444  2.537  2.345  0.913  2.11  1.375  1.139  0.277  1.962  0.868  0.214  0.979  1.194  1.023  0.969  1.745  1.443  1.786  2.193  1.163  1.836  1.852  0.933  1.034  1.179  2.245  2.104  3.191  0.766  2.775  2.067  1.343  1.638  1.139  0.756  1.142  0.801  0.612  1.603  0.91  2.056  1.551  1.459  1.086  1.749  1.013  2.02  0.441  1.112  1.634  
 
 SPSB1  80176  1  9352940  9429590  1p36.22  0.378  0.298  1.718  2.369  0.347  1.508  2.286  2.901  1.379  3.257  1.753  3.259  2.642  1.145  1.077  1.725  1.494  0.175  1.572  0.108  0.107  1.152  0.743  1.154  1.435  2.646  3.731  2.06  4.329  1.959  3.451  3.116  3.716  2.452  1.884  2.242  2.285  1.706  1.958  1.481  2.494  1.735  0.973  1.121  1.028  1.895  0.373  1.379  1.69  2.03  2.517  1.487  2.171  1.66  1.69  1.147  2.26  1.732  2.448  2.03  
 
 LOC100506022  100506022  1  9485152  9489055  -  0  0.06  0.308  0.279  0.073  0.112  0.512  0.17  0.519  0.597  0.114  0  0.059  0.174  0.105  0.14  0.079  0.219  0.167  0.038  0.085  0.184  0.592  0.119  0.065  0.236  0.706  0.268  0.587  0.151  0.497  0.49  0.404  0.505  0  0.084  0.225  0.099  0.071  0.184  0.252  0.273  0.05  0.26  0.135  0.086  0.103  0.063  0.103  0.118  0.062  0.25  0.098  0.06  0.117  0.118  0.12  0.123  0.112  0.099  
 
 SLC25A33  84275  1  9599527  9642831  1p36.22  1.935  2.687  0.252  3.105  2.784  1.735  2.424  2.169  2.961  2.317  2.878  2.557  2.723  3.558  1.908  2.065  2.31  3.358  4.24  2.468  2.947  2.758  2.221  1.702  1.53  1.946  2.132  1.99  2.715  1.845  3.702  3.078  2.395  1.596  0.899  1.833  2.537  1.335  1.352  2.328  2.023  1.737  3.875  2.154  2.949  2.433  0.874  1.407  1.816  3.409  2.061  1.079  0.335  0.622  1.548  1.215  1.222  2.075  2.407  1.641  
 
 TMEM201  199953  1  9648931  9674935  1p36.22  2.165  0.601  2.028  3.306  1.629  2.3  2.522  3.114  2.748  3.25  2.899  2.258  2.68  2.316  3.16  2.427  1.528  0.535  3.75  1.867  3.175  2.633  2.075  2.599  2.071  2.546  3.375  3.805  4.295  2.413  3.979  4.071  3.999  2.106  1.173  2.25  3.347  2.129  1.063  3.646  1.821  2.602  2.47  2.47  3.401  2.365  1.497  2.772  2.431  2.437  2.215  2.072  3.001  1.087  1.895  1.778  2.459  2.301  3.07  2.313  
 
 PIK3CD  5293  1  9711789  9789172  1p36.2  0.132  0.507  1.552  1.504  0.284  1.109  1.144  1.946  0.859  0.916  1.888  0.041  0.205  0.788  0.172  0.098  0.067  0.352  3.069  2.847  1.353  1.339  0.609  0.235  1.516  3.135  1.428  2.588  3.727  1.27  3.344  0.896  0.376  0.597  0.575  1.179  2.619  1.047  1.16  1.178  0.274  0.667  1.262  0.285  1.764  0.856  0.1  1.516  0.651  1.744  0.765  0.97  2.228  0.356  2.111  0.818  2.156  1.012  1.877  1.953  
 
 PIK3CD-AS1  644997  1  9712667  9714644  1p36.22  0  0  0.185  0  0  0  0  0.106  0.188  0.036  0  0  0  0.065  0  0  0.083  0  0  0.263  0  0.192  0.655  0  0  0  0.103  0  0  0.066  0  0  0  0.284  0  0.062  0  0  0.048  0  0  0.203  0.088  0  0  0.104  0.048  0.114  0  0  0  0.622  0  0.094  0  0  0  0  0  0  
 
 PIK3CD-AS2  101929074  1  9732483  9747632  1p36.22  0.887  0.698  0.472  3.628  0.296  2.465  0.828  1.578  3.127  0.895  2.012  0.884  0.56  3.383  1.058  0.524  0  3.309  2.545  0.615  3.451  1.186  2.678  2.976  1.153  1.867  0.665  2.292  3.795  2.833  4.943  1.638  1.884  2.783  2.818  3.34  0.753  0  0.224  3.162  0.321  1.488  3.014  1.346  3.179  0.51  2.02  0.836  0  1.864  0.711  0.753  0.909  0.266  2.455  0.893  0.764  1.534  0.939  1.358  
 
 CLSTN1  22883  1  9789078  9884584  1p36.22  3.56  1.441  4.281  4.5  3.431  3.097  4.439  4.925  4.895  4.932  4.764  2.983  3.32  3.739  4.372  4.305  4.049  0.494  4.188  2.727  1.539  3.883  2.258  2.727  3.162  3.884  3.97  4.18  5.314  3.103  4.179  4.081  3.792  2.205  2.466  4.228  4.994  4.636  3.265  5.297  3.959  3.091  3.813  4.899  4.697  3.826  4.187  4.144  3.936  4.709  4.337  2.985  4.024  2.311  4.673  4.393  4.784  3.556  4.827  4.76  
 
 CTNNBIP1  56998  1  9908333  9970316  1p36.22  1.647  1.959  1.785  2.444  3.024  1.717  1.704  3.284  3.413  3.017  1.795  3.982  1.279  1.807  2.163  2.609  2.443  2.04  3.82  2.091  1.253  2.231  1.734  1.146  0.922  2.08  3.636  1.948  3.138  2.21  2.522  2.382  2.937  2.117  0.846  2.179  3.127  3.461  1.34  3.095  3.38  1.55  4.437  1.113  1.582  2.765  2.553  1.175  1.45  2.193  2.731  1.638  2.665  0.924  1.374  1.236  2.045  2.45  2.53  2.192  
 
 LZIC  84328  1  9989775  10002826  1p36.22  1.431  2.333  1.72  1.46  2.119  1.791  1.672  2.034  1.649  2.118  2.286  1.735  2.265  2.666  1.65  1.722  2.089  2.792  3.754  1.591  2.61  2.298  1.52  1.248  1.1  1.162  1.63  1.84  2.039  1.34  2.921  2.596  1.884  1.263  1.008  1.912  1.901  1.6  0.829  2.46  2.492  1.985  2.519  1.797  1.813  1.978  0.462  0.684  2.009  2.725  2.607  0.905  1.807  0.678  1.358  1.542  2.356  2.378  1.846  1.802  
 
 NMNAT1  64802  1  10002980  10045556  1p36.22  0.846  1.253  0.911  1.032  0.71  0.717  1.279  1.114  1.059  1.464  0.972  0.798  1.234  1.16  0.81  0.535  1.287  1.238  1.693  1.194  1.577  1.02  1.485  0.474  0.255  0.885  1.357  0.255  1.657  0.33  0.874  1.013  0.657  1.052  0.329  0.766  1.032  0.575  0.94  1.006  0.675  0.832  0.871  0.506  0.696  0.788  1.199  0.629  0.491  1.566  1.55  0.649  1.009  0.318  0.749  0.73  0.959  0.105  1.255  1.046  
 
 RBP7  116362  1  10057254  10076078  1p36.22  0  0.42  0  0  4.213  1.333  0  0.361  0  0  0  0  0  4.892  0.385  0  0  0.26  0  0  0  0.164  0  0  0  0  0.122  0  0  0.1  0  0  0  0  0.54  0  1.623  0  0.17  2.226  0.872  0  2.518  0.659  3.163  2.292  0.244  3.379  1.325  4.726  0.798  2.097  0  0  3.459  1.808  0  4.231  1.796  0.442  
 
 UBE4B  10277  1  10093040  10241296  1p36.3  2.427  1.466  1.909  1.703  1.933  1.699  2.628  3.007  1.983  2.852  3.081  2.174  2.527  2.855  2.659  2.336  2.301  1.904  2.247  1.584  2.937  1.827  2.116  2.191  1.567  2.138  2.883  2.619  3.343  1.697  3.044  2.998  2.895  1.586  1.439  2.779  2.871  2.387  1.203  3.615  2.492  1.479  3.284  1.794  2.952  2.584  1.757  2.247  2.454  2.725  2.795  1.632  2.196  1.462  2.294  1.9  2.791  2.256  3.017  2.683  
 
 KIF1B  23095  1  10270763  10441661  1p36.2  1.114  1.516  1.87  2.265  1.446  1.471  2.609  3.122  2.156  2.75  3.01  1.064  2.181  1.965  2.337  1.505  1.906  1.323  1.256  0.694  1.456  0.973  1.318  0.769  1.499  2.132  2.308  1.792  2.666  1.602  2.252  2.494  2.051  1.214  1.357  2.394  2.414  2.201  1.814  2.869  1.74  1.442  3.133  2.321  2.868  2.057  1.635  2.12  2.241  2.748  2.581  1.099  1.912  1.058  1.575  1.599  2.716  1.553  2.451  2.449  
 
 PGD  5226  1  10459048  10480568  1p36.22  4.457  3.228  3.412  5.596  4.558  3.717  6.313  5.045  4.957  4.963  5.326  4.429  6.425  5.09  4.765  4.711  4.847  2.994  6.07  5.392  5.207  5.184  4.791  4.912  3.481  3.27  5.213  4.292  4.682  4.232  4.984  4.823  4.678  3.257  6.673  4.909  6.304  4.63  5.005  4.363  6.145  5.942  6.758  4.885  5.466  4.722  4.904  4.117  4.646  5.585  4.582  3.78  4.739  4.615  4.317  4.247  4.553  5.252  5.307  4.707  
 
 APITD1  378708  1  10490158  10502872  1p36.22  2.418  3.91  1.433  1.828  3.003  1.997  2.482  1.72  2.031  2.381  2.973  2.305  2.204  3  1.536  2.412  1.863  4.882  4.046  2.884  2.158  2.227  2.545  2.086  1.609  1.557  1.508  1.797  2.242  1.375  2.855  2.567  2.025  1.477  2.035  1.847  2.584  2.091  1.752  2.044  2.307  2.596  2.737  1.506  2.305  1.432  1.082  1.255  2.29  3.192  2.097  1.62  2.208  1.261  2.222  1.845  1.507  2.097  2.782  2.14  
 
 APITD1-CORT  100526739  1  10490158  10512060  1p  0.397  0.444  0.495  0.807  0.668  1.012  1.074  0.767  0.721  0.735  1.226  0.441  0.455  1.005  0.314  0.618  0.88  0  2.107  0.869  0.516  1.183  0.389  0.248  0.315  0.32  1.283  0.663  0.814  0.095  1.007  0.872  0.832  0.59  1.176  0.436  0.597  0.303  0.429  0.555  0.868  0.94  0.912  0.304  0.779  0.531  0.572  0.627  0.784  1.465  0.71  1.002  0.847  0  0.699  0.323  0.46  0.247  0.716  0.367  
 
 CORT  1325  1  10509775  10512060  1p36.22  0.305  0.331  0.151  0.441  0.415  0.212  0.251  0.222  0.341  0.366  0.443  0.183  0.598  0.554  0.386  0.297  0.189  0.969  0.898  0.725  0  0.973  0.77  0.207  0  0  0.206  0.104  0.212  0.057  0.387  0.224  0.258  0.357  0.188  0  0.354  0  0.487  0  0.347  0.336  0  0.28  0.45  0.12  0.127  0.212  0  0.31  0.236  0.192  0.325  0.271  0.189  0.158  0.234  0.542  0.298  0  
 
 DFFA  1676  1  10520602  10532613  1p36.3-p36.2  2.892  2.38  2.897  4.257  2.707  2.639  3.808  3.873  2.591  3.478  3.858  2.916  3.803  3.706  3.68  2.979  3.656  1.07  3.835  3.038  4.087  3.24  2.926  3.061  3.783  2.574  3.668  3.845  4.25  3.354  4.463  4.044  4.154  2.637  2.626  3.055  4.176  3.454  1.938  5.34  3.366  2.257  5.103  4.227  4.217  3.621  2.072  4.485  3.457  5.031  3.855  2.795  3.661  1.967  3.537  3.441  3.391  3.902  3.852  3.878  
 
 PEX14  5195  1  10535002  10690815  1p36.22  1.635  0.782  1.905  3.256  2.193  2.319  3.086  2.977  2.86  2.953  3.016  2.305  2.869  2.183  2.83  2.725  2.726  0.645  3.448  1.963  3.016  2.03  1.993  2.047  1.939  1.803  2.767  2.719  3.408  2.331  3.252  2.92  3.054  1.715  1.927  2.463  3.465  2.898  1.555  3.256  2.791  2.721  2.843  1.94  2.929  2.221  2.096  2.967  2.355  4  2.864  2.132  3.194  1.537  2.659  2.229  2.407  2.742  2.995  2.698  
 
 CASZ1  54897  1  10696665  10856733  1p36.22  2.379  0.351  0.469  1.917  2.371  0.988  0.614  1.296  0.522  0.875  1.083  2.035  2.014  0.809  1.319  1.731  1.13  1.051  0.606  0.295  0.2  0.876  0.677  0.134  0.137  0.441  0.552  0.127  0.267  0.727  0.17  0.306  0.564  0.41  0.415  1.567  0.792  0.608  0.668  1.472  0.801  0.967  1.322  0.515  1.353  1.326  0.857  1.299  1.538  1.247  0.645  1.854  1.792  0.918  1.002  0.745  1.758  1.739  2.36  1.914  
 
 C1orf127  148345  1  11006529  11042094  1p36.22  0  0.031  0.109  0  0.048  0  0.264  0.114  0.183  0.128  0.03  0.068  0.157  0.027  0  0  0.064  0.076  0.209  0  0.082  0.104  0.448  0  0  0.044  0.117  0.189  0  0.113  0  0  0  0.437  0.066  0  0.048  0  0.062  0  0.06  0.061  0.046  0  0.081  0.541  0.044  0  0  0  0.026  0.362  0.026  0.092  0.058  0.052  0  0  0  0  
 
 TARDBP  23435  1  11072678  11085549  1p36.22  3.589  5.408  2.737  3.156  3.664  3.09  4.333  3.608  3.067  3.797  4.393  3.017  3.719  4.262  3.749  3.745  3.502  5.638  5.446  4.27  3.553  4.102  3.011  2.726  3.221  3.052  3.131  3.357  3.272  2.577  3.782  3.787  3.549  2.517  2.97  3.407  3.907  3.131  3.532  3.902  3.397  3.094  4.16  3.544  3.168  3.07  3.958  3.115  3.277  4.952  3.711  2.272  3.677  2.611  3.628  3.172  3.632  3.53  3.78  3.802  
 
 MASP2  10747  1  11086579  11107296  1p36.3-p36.2  0.329  0.226  0.24  0  0.346  0  0.238  0.37  0.161  0.529  0.609  0.154  0.179  0.635  0.765  0.3  0.101  0.392  0.188  0.401  0  0.987  0.509  0.249  0  0.214  0.513  0.358  0.615  0.153  0.45  0.345  0.383  0.355  0  0.199  0.176  0.398  0.275  0.357  0.339  0.441  0.744  0.318  0.39  0.269  0  0.29  0.418  0.211  0.312  0.405  0.407  0.117  0.241  0  0.51  0  0.56  0.204  
 
 SRM  6723  1  11114648  11120091  1p36-p22  5.463  4.528  6.324  6.44  5.46  6.094  7.685  6.375  5.795  6.256  6.543  4.509  6.118  5.451  5.952  5.703  4.922  3.281  7.761  6.868  6.827  6.689  6.71  7.466  5.339  5.635  7.052  7.094  7.298  6.353  7.441  7.901  7.422  6.074  5.474  6.159  7.317  6.79  5.647  7.165  5.489  6.552  7.322  5.521  5.834  5.773  5.79  6.067  4.502  7.327  6.712  5.245  6.833  4.368  5.427  5.533  5.823  7.087  6.855  5.858  
 
 EXOSC10  5394  1  11126669  11159967  1p36.22  2.594  4.007  1.871  2.734  3.019  2.708  2.923  3.386  1.993  3.491  3.638  2.511  2.967  3.446  3.265  3.13  3.054  4.008  4.324  3.171  3.619  3.415  1.801  2.427  2.326  2.79  3.111  3.191  3.843  2.296  3.898  3.616  3.575  1.808  2.321  3.147  3.633  3.26  2.654  4.141  2.861  2.134  3.209  2.978  3.261  2.723  1.544  3.071  3.251  4.492  3.187  2.173  3.128  1.74  3.163  2.459  3.656  3.16  3.902  3.224  
 
 MTOR  2475  1  11166587  11322608  1p36.2  2.543  1.764  1.784  1.791  2.054  1.776  2.833  2.816  1.895  2.824  2.935  1.533  2.123  2.974  3.248  2.128  2.16  1.5  3.03  1.961  2.972  2.444  1.447  2.098  1.409  2.366  2.993  2.889  3.734  2.316  3.217  2.913  3.29  1.384  1.391  2.817  3.352  2.532  1.59  3.988  2.356  1.231  3.374  2.42  2.914  2.558  2.041  2.389  2.711  2.721  2.825  2.01  2.729  1.112  2.255  1.95  2.617  2.36  3.257  2.733  
 
 MTOR-AS1  100873935  1  11203954  11209595  -  0  0  0.261  0.638  0.323  0  0.531  0.345  0.356  0.422  0.513  0.215  0.244  0.167  0.444  0.3  0.394  0  0  0.368  0.35  0.254  0.681  0.193  0  0.272  1.063  0.22  0.977  0.081  0.456  0.422  0.585  0.406  0  0.254  0.576  0.423  0.202  0.454  0.275  0.209  0.614  0  0  0.15  0  0  0  0  0.274  0.566  0.531  0  0.243  0  0.363  0.436  0.597  0  
 
 ANGPTL7  10218  1  11249345  11256038  1p36  0.216  0.087  0.217  0  0.053  0.109  0.056  0.157  0.082  0.165  0.181  0.063  0.155  0.221  0.369  0.13  0.059  0.082  0.043  0.06  0.202  0.135  0.461  0.056  0.066  0.161  0.234  1.091  0.361  0.155  0.14  0.336  0.213  0.369  0  0.058  0.142  0.201  0.067  0.086  0.117  0.3  0.079  0.186  0.235  0.232  0.09  0.181  0.21  0.059  0.115  0.298  0.226  0.14  0.196  0  0.25  0  0.137  0  
 
 UBIAD1  29914  1  11333254  11348491  1p36.22  2.232  1.529  1.755  2.453  2.407  1.688  2.945  2.388  2.742  2.295  2.453  1.94  2.055  2.413  2.577  2.025  1.961  0.49  3.467  2.892  2.598  2.496  2.848  2.738  1.839  1.983  2.949  2.657  3.105  2.738  2.592  2.827  3.353  2.489  1.803  2.481  3.143  2.12  1.853  3.137  2.029  1.861  2.966  2.013  2.934  1.952  1.399  1.877  1.936  2.625  2.238  1.961  2.518  1.07  1.89  1.616  1.982  1.751  3.07  2.136  
 
 PTCHD2  57540  1  11539294  11597640  1p36.22  0.204  0.031  0.102  0  0.159  0.073  0.095  0.064  0.161  0.118  0.06  0.108  0.03  0.243  0.165  0.08  0.079  0.147  0.772  0.075  0  0.298  0.452  0.344  0.043  0.053  0.205  0.047  0.075  0.209  0.051  0.051  0.06  0.262  0.067  0.092  0.034  0  0.124  0.301  0.036  0.223  0.364  0.039  0.066  0.052  0.101  0.071  0.033  0.059  0.074  0.756  0.064  0.2  0.116  0.092  0.041  0  0.127  0.026  
 
 LOC101929181  101929181  1  11669587  11673411  -  0  0  0  0  0  0  0  0  0.182  0  0  0  0  0.069  0  0  0  0.36  0  0  0  0.065  0.497  0  0  0  0.169  0.063  0  0.101  0  0  0.112  0.139  0  0  0  0  0.073  0  0.088  0.268  0.582  0  0  0.116  0  0  0  0  0  0.206  0  0.063  0.051  0  0  0  0  0  
 
 FBXO2  26232  1  11708417  11714888  1p36.22  0.699  0.089  0.294  0.955  2.735  0.123  0.221  0.067  0.552  0.288  0.279  0.373  2.339  4.599  2.46  0.146  0.141  0.568  0.14  0.271  0.216  0.427  0.882  0.731  0.424  1.274  1.644  0.306  1.924  0.598  0.917  0.702  0.576  0.821  0.549  3.552  0.859  2.865  3.677  3.731  0.86  0.696  3.966  0.276  1.688  2.886  2.74  0.487  0.335  1.784  0.423  1.9  0.137  0.37  0.7  0.222  0.252  0.43  0.565  0.594  
 
 FBXO44  93611  1  11714431  11723384  1p36.22  1.435  0.193  1.399  1.885  1.913  1.22  1.616  1.318  2.77  2.648  1.923  0.435  1.481  2.686  2.185  0.464  0.403  0.06  0.223  0.603  1.153  1.906  1.214  0.86  1.471  1.851  2.383  1.641  3.228  1.776  3.317  2.788  2.636  1.54  1.52  2.431  1.234  2.097  1.999  3.04  1.278  1.918  3.199  1.486  1.876  1.589  1.126  1.063  1.541  2.608  1.525  1.019  0.565  1.006  1.618  1.445  1.305  1.581  2.026  1.71  
 
 FBXO6  26270  1  11724149  11734409  1p36.22  1.444  0.482  0.582  2.639  2.218  0.769  0.781  1.414  0.679  0.641  1.656  0.601  1.299  3.139  1.155  0.264  0.78  0.147  1.037  1.47  3.044  0.477  0.498  0.394  0.75  1.365  1.541  1.378  2.787  0.458  2.307  2.913  0.528  0.758  1.394  2.268  0.988  1.873  1.322  2.856  1.655  1.239  4.591  1.277  1.596  1.475  0.047  1.083  1.964  2.621  2.2  0.866  0.398  0.681  0.736  1.678  1.768  1.216  1.095  1.067  
 
 MAD2L2  10459  1  11734536  11751678  1p36  3.763  2.213  3.926  5.152  3.908  4.404  5.592  4.911  3.619  5.126  4.879  2.894  3.218  5.222  3.967  3.554  3.967  0.836  7.421  4.402  5.272  5.568  4.471  4.016  3.844  3.88  4.529  3.942  4.981  3.39  5.268  5.15  4.708  3.129  3.094  4.157  4.833  4.763  3.518  5.508  3.734  4.148  5.489  3.777  4.218  4.05  3.482  4.536  4.182  6.11  4.432  3.683  4.436  2.44  3.848  3.732  4.523  5.477  4.918  3.7  
 
 DRAXIN  374946  1  11751780  11780336  1p36.22  0  0.065  0.1  0.961  0.051  0  0.132  0.6  0.142  0.243  0.749  0.081  0.078  0.386  0  0.077  0.098  0.123  1.36  0.059  0  0.314  0.44  0  0  0.271  0.192  0.122  0.198  0.119  0  0  0  0.27  0.072  0  0.052  0  0.127  0.174  0  0.455  0.834  0  0.122  0.084  0.089  0  0  0  0  0.459  0  0.137  0.097  0  0  0  0  0.086  
 
 AGTRAP  57085  1  11796141  11810828  1p36.22  4.101  1.203  3.258  5.437  4.566  2.491  5.648  4.451  3.391  4.33  3.637  3.48  4.13  5.105  3.717  4.211  4.149  0.326  4.466  4.743  4.387  2.561  4.022  4.33  4.05  3.939  4.761  4.32  5.072  4.909  5.086  5.058  4.452  3.666  3.922  4.495  4.637  4.724  3.656  5.519  3.465  4.236  5.422  3.235  4.54  4.2  4.019  3.902  3.213  5.584  4.251  3.311  2.607  3.486  4.585  4.568  3.346  4.836  5.333  3.894  
 
 C1orf167  284498  1  11822249  11849642  1p36.22  0  0.015  0.103  0  0.064  0.068  0  0.021  0.14  0.052  0.019  0.054  0  0.042  0  0.043  0.077  0.026  0.016  0.028  0.134  0.079  0.443  0  0  0.028  0.123  0.046  0.027  0.118  0.034  0.026  0  0.139  0.022  0  0.038  0  0.035  0  0.025  0.389  0.051  0.038  0  0.053  0.019  0  0  0.03  0.035  0.585  0.023  0.088  0.047  0.042  0.026  0.031  0  0  
 
 LOC102724659  102724659  1  11837133  11839676  1p36.22  0  0  0.098  0  0.053  0  0  0  0.137  0.058  0  0  0  0  0  0  0  0.085  0  0  0  0.065  0.653  0  0  0  0.065  0  0  0.047  0  0  0  0.248  0  0  0  0  0  0  0  0.046  0.038  0  0  0.045  0  0  0  0  0  0.166  0  0.046  0.047  0  0  0  0  0  
 
 MTHFR  4524  1  11845786  11866160  1p36.3  0.589  0.492  0.376  1.49  1.404  0.339  0.55  0.793  1.289  1.183  1.202  1.006  1.593  0.651  1.366  1.071  1.037  0.428  1.515  1.03  3.093  1.568  1.657  1.358  0.387  1.139  1.298  1.164  1.387  1.266  2.06  1.328  1.497  0.905  0.487  1.459  0.742  0.94  0.509  2.134  1.591  0.717  1.962  1.004  1.702  1.026  0.563  0.731  0.695  1.31  0.718  0.784  1.103  0.743  1.263  0.718  1.636  0.714  1.433  0.915  
 
 CLCN6  1185  1  11866152  11903201  1p36  1.164  0.822  1.304  2.23  1.425  0.769  1.346  1.652  1.31  2.113  1.853  0.482  1.109  1.247  1.342  1.329  0.902  0.706  1.925  1.086  1.268  1.684  1.036  1.253  1.208  1.678  1.935  1.79  2.208  1.369  2.464  1.657  1.838  0.881  0.804  1.713  2.112  1.6  1.354  1.806  1.483  0.852  2.23  1.265  1.313  1.11  0.723  1.618  1.239  2.027  1.669  1.023  1.705  0.443  1.791  1.277  2.005  1.306  1.847  1.883  
 
 NPPA-AS1  100379251  1  11900375  11907673  1p36.21  0  0.149  0.229  0.334  0.354  0.246  0.222  0.218  0.116  0.313  0.279  0  0.272  0.441  0.127  0.223  0.131  0  0.212  0.064  0.247  0.534  0.316  0.14  0  0.175  0.133  0.315  0.44  0.089  1.761  0.528  0.41  0.317  0  0.151  0.303  0.249  0.095  0.187  0.461  0.449  0  0.292  0.149  0.22  0  0.157  0.275  0.553  0.14  0.115  0.337  0.203  0.29  0.104  0.342  0.18  0.262  0.126  
 
 NPPA  4878  1  11905766  11907840  1p36.21  0.285  0.211  0.168  0  0.421  0  0.399  0  0.264  0.358  0.333  0  0.241  0.6  0.193  0.141  0.291  0.261  0.41  0.274  0.299  0.766  0.585  0.442  0  0.366  0.283  0.532  0.791  0.604  1.587  0.632  0.528  0.216  0.403  0.165  0.624  0.291  0.114  0.45  0.384  0.243  0.843  0.358  0  0.209  0  0.286  0.259  0.802  0  0.683  0.452  0.096  0.32  0  0.485  0.372  0.436  0  
 
 NPPB  4879  1  11917520  11918992  1p36.2  0.328  0.894  6.083  0  0  7.411  0  0.148  0  0.089  0.548  0  0  0  0  0  0  0  0  0.451  0  0.157  0.58  0  0  0  0.121  0.316  0  0.065  0  0  0  0  0.195  0  3.134  0  7.579  0  0  0  0.555  0.39  0  5.275  0  0.556  0  0  0  0  0  0  1.41  0.276  0.22  0  0  3.562  
 
 KIAA2013  90231  1  11979644  11986485  1p36.22  3.076  1.825  3.52  4.694  2.979  2.604  4.658  4.356  4.058  4.374  4.836  3.912  4.051  3.633  4.024  3.485  3.668  0.83  4.275  2.979  3.013  2.706  3.83  3.678  2.402  3.11  4.372  4.217  5.04  4.24  4.082  4.653  4.421  2.86  2.324  3.288  4.536  4.231  2.5  4.417  3.258  3.807  3.851  3.275  3.528  3.526  2.36  3.171  3.234  4.325  3.862  2.599  3.785  2.124  3.167  2.903  3.774  2.857  3.855  3.221  
 
 PLOD1  5351  1  11994723  12035599  1p36.22  3.06  1.531  5.804  5.592  3.559  3.543  5.728  6.149  5.767  7.026  6.103  2.833  3.667  5.257  4.005  2.694  4.095  0.326  0.216  2.059  3.775  1.915  3.19  3.394  3.264  4.97  5.215  5.216  6.391  4.122  5.069  5.95  5.441  3.554  2.383  4.397  5.31  5.557  2.988  5.617  2.776  3.924  4.326  5.381  3.895  4.338  5.069  3.505  4.253  4.678  5.399  2.285  4.77  3.74  3.354  4.669  5.998  3.124  4.465  4.488  
 
 MFN2  9927  1  12040237  12073572  1p36.22  2.725  2.049  2.734  3.345  2.626  2.671  3.932  3.497  3.323  3.518  3.916  2.416  3.741  3.16  3.715  2.961  2.812  2.43  3.727  2.217  3.71  2.492  2.333  2.388  2.923  3.386  3.532  3.761  4.429  3.289  4.079  4.116  3.807  1.976  1.918  3.243  4.52  3.548  2.355  4.377  2.879  2.665  3.817  3.235  3.888  3.293  1.183  2.965  3.214  4.086  4.11  2.49  3.643  1.89  2.943  2.572  3.31  2.913  3.597  3.718  
 
 MIIP  60672  1  12079298  12092106  1p36.22  1.552  0.079  2.332  4.145  2.204  2.153  2.612  2.509  2.612  3.009  2.743  1.96  2.894  2.379  2.6  2.596  2.162  0  2.779  2.436  3.41  2.979  2.948  2.799  2.083  2.027  2.788  1.832  3.155  1.925  3.681  3.415  2.866  1.541  1.639  2.232  3.176  2.934  2.164  3.065  2.137  2.653  3.589  2.203  3.336  2.795  1.172  2.75  1.584  4.25  2.272  1.987  2.698  1.478  2.669  1.995  2.474  3.438  3.711  1.805  
 
 TNFRSF8  943  1  12123433  12204264  1p36  0  0.036  0.395  0.104  0.099  0.142  0.166  0.037  0.164  0.107  0.267  0.073  0  0.097  0  0.195  0.036  0.123  1.816  0.092  2.65  0.978  1.106  1.814  0.037  0.07  0.203  0.064  0.42  0.378  0  0.627  0  0.271  0.046  0.048  0.499  1.702  0.228  0.578  0  0.711  0.142  0.116  0.289  0.088  0.039  0.652  0.051  1.213  0.102  0.408  0.054  0.098  0.169  0.094  0.285  0.039  0  0  
 
 TNFRSF1B  7133  1  12227059  12269277  1p36.22  0.068  0.819  0.18  1.764  0.099  0.029  2.049  0.511  0.147  0.672  0.073  3.61  1.15  0.06  0.367  0.437  2.088  0.849  0.684  2.723  3.1  0.089  1.922  2.86  0  1.502  0.215  1.258  0.047  0.63  0.073  0.418  0  0.426  0.107  0.061  2.728  0.876  0.577  1.207  0.031  0.676  0.04  0.13  0.121  0.102  0.696  0.044  0  0.04  0.303  0.529  0.038  0.219  0.042  0.118  0.079  1.189  0.377  0.176  
 
 VPS13D  55187  1  12290095  12572098  1p36.22  1.276  0.497  0.79  0.967  1.698  0.743  1.244  1.369  1.142  1.642  1.553  0.533  1.294  1.367  1.207  0.765  0.775  0.371  1.343  0.727  1.487  0.996  0.523  0.513  0.753  1.118  1.559  1.718  1.424  1.197  1.428  1.309  1.645  0.679  0.559  1.395  1.538  1.369  0.832  1.66  1.247  0.388  1.241  1.134  1.226  1.12  0.444  0.835  1.379  1.026  1.828  0.705  1.21  0.63  0.834  0.76  1.243  0.982  1.314  1.222  
 
 DHRS3  9249  1  12627938  12677737  1p36.1  0.89  0.532  1.822  4.655  1.674  0.374  6.603  1.856  4.182  4.14  4.846  4.127  2.482  4.506  1.07  4.195  3.073  2.267  0.604  0.304  3.383  1.502  3.31  1.983  3.422  2.896  6.62  2.649  1.152  0.472  0.219  0.18  2.643  2.105  3.208  3.202  3.126  2.523  4.895  4.371  5.45  2.66  1.42  1.363  2.412  1.675  5.091  0.191  0.912  1.953  4.608  2.293  2.313  4.356  3.775  5.346  1.084  0.434  0  2.288  
 
 AADACL4  343066  1  12704565  12727097  1p36.21  0  0  0.125  0  0  0  0  0  0.154  0  0  0  0  0.108  0  0  0  0  0  0  0  0.074  0.294  0  0  0  0.064  0  0  0.062  0  0  0  0.245  0  0  0  0  0.09  0  0  0.06  0.033  0  0  0.075  0.041  0  0  0  0  0.31  0  0.1  0  0  0  0  0  0  
 
 AADACL3  126767  1  12776117  12788726  1p36.21  0  0  0.149  0  0  0  0  0.018  0.145  0.017  0  0.031  0  0.011  0  0  0  0.029  0  0.026  0  0.032  0.38  0  0  0  0.049  0  0  0.036  0  0  0  0.345  0  0  0  0  0.025  0  0  0.02  0.03  0  0  0.024  0.029  0  0  0  0  0.136  0  0.054  0.018  0  0  0  0  0  
 
 C1orf158  93190  1  12806133  12821102  1p36.21  0  0  0.156  0  0.108  0  0  0  0  0  0  0  0  0  0  0  0  0  0  0  0  0.063  0  0  0  0  0.131  0  0  0.07  0  0  0  0.341  0  0  0  0  0.103  0  0  0.173  0  0  0  0  0  0  0  0  0  0  0  0  0  0  0  0  0  0  
 
 PRAMEF12  390999  1  12834990  12838048  1p36.21  0  0  0.171  0  0  0  0  0  0.138  0  0  0  0  0  0  0  0  0.085  0  0  0  0.103  0.418  0  0  0  0.051  0  0  0.053  0  0  0  0.249  0  0  0  0  0  0  0  0.253  0.045  0  0  0  0  0  0  0  0  0.229  0  0.032  0  0  0  0  0  0  
 
 PRAMEF1  65121  1  12851545  12856777  1p36.21  0  0  0.249  0  0  0  0  0  0.259  0.045  0  0  0  0  0  0  0  0  0  0  0  0.05  0.812  0  0  0  0.068  0  0  0.047  0  0  0  0.495  0  0  0  0  0  0  0  0.056  0  0  0  0  0  0  0  0  0  0.182  0  0  0  0  0  0  0  0  
 
 PRAMEF11  440560  1  12884617  12891264  1p36.21  0  0  0.23  0  0  0  0  0  0.087  0  0  0  0  0  0  0  0  0  0  0  0  0.036  0.571  0  0  0  0  0  0  0  0  0  0  0.345  0  0  0  0  0  0  0  0.056  0  0  0  0  0  0  0  0  0  0.111  0  0  0  0  0  0  0  0  
 
 LOC101060301  101060301  1  12907229  12908157  -  0.916  1.094  0.854  1.71  0.61  0.186  0.808  0.307  1.37  0.605  0.451  0.731  0.334  1.455  0.781  1.038  1.186  0  1.253  0.296  0.349  1.428  1.796  0.682  0.789  0.371  0.711  0.43  0.639  0.149  0.503  0.552  0.422  0.318  1.055  0.234  0.64  0.166  0.601  0.459  0.396  0.869  0.899  0.442  0.237  0.294  0.822  0.395  0.513  0.827  0.59  0.558  1.003  0.333  1.369  0.453  0.246  0.26  0.454  0.392  
 
 HNRNPCL3  649330  1  12907229  12908235  -  0  0  0  0  0  0  0  0  0  0  0.313  0  0.576  0  0  0  0  0.451  0  0  0  0  0  0  0  0  0  0  0  0.081  0  0  0  0.918  0  0  0  0  0  0  0  0.276  0  0  0  0  0  0  0  0.623  0  0  0  0  0  0  0  0  0  0  
 
 HNRNPCL1  343069  1  12907229  12908609  1p36.21  0  0  0.276  0  0  0  0  0  0  0  0  0  0  0.059  0  0  0  0  0  0  0  0  0  0  0  0  0.075  0  0  0  0  0  0  0  0  0  0  0  0  0  0  0  0  0  0  0  0  0  0  0  0  0  0  0  0  0  0  0  0  0  
 
 PRAMEF2  65122  1  12916940  12921764  1p36.21  0  0  0.244  0.167  0  0  0  0  0.227  0  0  0  0  0  0  0  0  0  0  0  0  0.048  0.666  0  0  0  0.064  0  0  0.032  0  0  0  0.374  0  0  0  0  0  0  0  0.066  0  0  0  0  0  0  0  0  0  0.244  0  0.063  0  0  0  0  0  0  
 
 PRAMEF4  400735  1  12939032  12946025  1p36.21  0  0  0.128  0  0  0  0  0  0.189  0  0  0  0  0  0  0  0  0  0  0  0  0  0.703  0  0  0  0  0  0  0  0  0  0  0.325  0  0  0  0  0  0  0  0.131  0  0  0  0  0  0  0  0  0  0  0  0  0  0  0  0  0  0  
 
 PRAMEF7  441871  1  12976466  12980300  1p36.21  0  0  0.119  0  0  0  0  0  0.205  0  0  0  0  0  0  0  0  0  0  0  0  0.046  0.341  0  0  0  0.026  0  0  0.019  0  0  0  0.174  0  0  0  0  0  0  0  0.093  0  0  0  0  0  0  0  0  0  0.18  0  0  0  0  0  0  0  0  
 
 PRAMEF6  440561  1  12998301  13007406  1p36.21  0  0  0.151  0  0  0  0  0  0.114  0  0  0  0  0  0  0  0  0  0  0  0  0  0.603  0  0  0  0  0  0  0  0  0  0  0.403  0  0  0  0  0  0  0  0  0  0  0  0  0  0  0  0  0  0.093  0  0  0  0  0  0  0  0  
 
 PRAMEF9  343070  1  12998504  13002353  1p36.21  0  0  0  0  0  0  0  0  0  0  0  0  0  0  0  0  0  0  0  0  0  0  0.397  0  0  0  0  0  0  0  0  0  0  0  0  0  0  0  0  0  0  0.091  0  0  0  0  0  0  0  0  0  0  0  0  0  0  0  0  0  0  
 
 PRAMEF25  441873  1  13052239  13112702  1p36.21  0  0  0.145  0  0  0  0  0  0.093  0  0  0  0  0  0  0  0  0  0  0  0  0  0.202  0  0  0  0  0  0  0  0  0  0  0.196  0  0  0  0  0  0  0  0  0  0  0  0  0  0  0  0  0  0  0  0  0  0  0  0  0  0  
 
 LOC645359  645359  1  13052393  13112702  1p36.21  0  0  0  0  0  0  0  0  0  0  0  0  0  0  0  0  0  0  0  0  0  0  0.594  0  0  0  0  0  0  0  0  0  0  0  0  0  0  0  0  0  0  0.046  0  0  0  0  0  0  0  0  0  0  0  0  0  0  0  0  0  0  
 
 PRAMEF5  343068  1  13108513  13117751  1p36.21  0  0  0.106  0  0  0  0  0  0  0  0  0  0  0  0  0  0  0  0  0  0  0  0  0  0  0  0  0  0  0  0  0  0  0.202  0  0  0  0  0  0  0  0  0  0  0  0  0  0  0  0  0  0  0  0  0  0  0  0  0  0  
 
 HNRNPCL2  440563  1  13182959  13184326  1p36.21  0  0.089  0.239  0.324  0  0  0  0  0.376  0.085  0.158  0.16  0.175  0.147  0.325  0.177  0.132  0.147  0.168  0  0  0.139  0.596  0  0  0.165  0.161  0.088  0.103  0.074  0  0  0  0.331  0.154  0  0.157  0  0.044  0.166  0  0.41  0.083  0  0  0  0  0  0  0.183  0.087  1.408  0.134  0  0.157  0  0  0  0  0  
 
 PRAMEF18  391003  1  13328195  13698405  1p36.21  0  0  0  0  0  0  0  0  0.074  0  0  0  0  0  0  0  0  0  0  0  0  0  0.269  0  0  0  0.024  0  0  0  0  0  0  0  0  0  0  0  0  0  0  0.127  0  0  0  0  0.033  0  0  0  0  0  0  0  0  0  0  0  0  0  
 
 PRAMEF15  653619  1  13421175  13428190  1p36.21  0  0  0.132  0  0  0  0  0  0.128  0  0  0  0  0  0  0  0  0  0  0  0  0  0.435  0  0  0  0.024  0  0  0  0  0  0  0.18  0  0  0  0  0  0  0  0  0  0  0  0  0  0  0  0  0  0  0  0  0  0  0  0  0  0  
 
 PRAMEF19  645414  1  13474052  13477569  1p36.21  0  0  0  0  0  0  0  0  0.069  0.029  0  0  0  0  0  0  0  0  0.03  0  0  0  0.208  0  0  0  0.082  0  0  0.078  0  0  0  0.15  0  0  0  0  0.024  0  0  0.072  0.028  0  0  0  0.074  0  0  0  0  0.137  0.026  0.028  0  0  0  0  0  0  
 
 PRAMEF16  654348  1  13495253  13498259  1p36.21  0  0  0  0  0  0  0  0  0  0  0  0  0  0  0  0  0  0  0  0  0  0  0.36  0  0  0  0  0  0  0  0  0  0  0  0  0  0  0  0  0  0  0  0  0  0  0  0  0  0  0  0  0  0  0  0  0  0  0  0  0  
 
 PRAMEF14  729528  1  13668268  13673511  1p36.21  0  0  0.139  0  0  0  0  0  0.072  0  0  0  0  0  0  0  0  0  0  0  0  0.031  0.332  0  0  0  0.032  0  0  0.035  0  0  0  0.165  0  0  0  0  0  0  0  0  0  0  0  0  0  0  0  0  0  0.085  0  0  0  0  0  0  0  0  
 
 PRAMEF17  391004  1  13716091  13719089  1p36.21  0  0  0.12  0.169  0.052  0  0.274  0  0.167  0  0.072  0.113  0  0.077  0  0.135  0.112  0  0.079  0.069  0.19  0.172  0.253  0  0  0  0.226  0.045  0  0.162  0  0  0  0.258  0.135  0  0  0  0.063  0  0  0.053  0.107  0.186  0  0.046  0.166  0  0.15  0.061  0.073  0.131  0  0.091  0.081  0  0  0  0  0  
 
 PRAMEF20  645425  1  13736906  13747803  1p36.21  0  0  0.12  0  0  0  0  0  0  0  0  0  0  0  0  0  0  0  0  0  0  0.054  0.26  0  0  0  0.055  0  0  0.037  0  0  0  0.144  0  0  0  0  0.035  0  0  0  0  0  0  0  0  0  0  0  0  0.113  0  0  0  0  0  0  0  0  
 
 LRRC38  126755  1  13801444  13840242  1p36.21  0  0  0.157  0  0.094  0.092  0.671  0.078  0.102  0.262  0.063  0.113  0  0.034  0  0.065  0.186  0.292  3.579  0.143  0  3.017  1.313  0.771  0  0.059  0.163  0.043  0  0.142  0  0  0  0.275  0  0  0  0  0.411  0.117  0  0.876  0.083  0.128  0  0  0.087  0.076  0  0.237  0.232  1.199  0  0.107  0.159  0  0  0  0  0.063  
 
 PDPN  10630  1  13910251  13944452  1p36.21  0  0  0.224  0  0  0.515  2.261  0.898  0.134  4.096  0.175  0.046  0  0.028  0  0.031  0  0.08  0.075  0.067  0  0.117  0.206  0  0  0  0.115  0  0  0.077  0  0  0  0.369  0.049  0  0.278  0  3.87  0  0  0.391  0.041  0.128  0  0.776  0.073  1.164  0  2.752  0.042  0.244  0.492  0.111  0.038  0  1.566  0  0  3.932  
 
 PRDM2  7799  1  14026734  14151574  1p36.21  1.997  1.118  1.854  2.062  2.374  1.671  2.459  3.198  2.295  2.882  2.644  1.5  2.213  2.078  2.666  1.842  1.732  1.082  2.871  2.214  2.006  2.75  2.256  2.035  1.682  2.435  2.81  2.47  3.462  1.472  3.153  2.734  2.689  1.412  1.21  2.079  2.537  1.976  1.549  2.842  2.124  1.156  2.902  2.122  2.746  2.469  1.005  1.631  2.047  1.9  2.628  1.512  1.764  1.188  1.741  1.249  2.589  1.129  2.14  2.146  
 
 KAZN  23254  1  14925212  15444544  1p36.21  1.499  2.052  1.702  2.541  0.829  1.875  0.765  2.444  2.321  2.924  2.335  0.422  1.417  1.952  1.188  0.457  0.489  0.403  0.396  1.886  2.04  0.423  1.512  1.622  0.233  2.785  3.628  2.808  2.529  2.036  3.457  2.92  0.07  0.524  0.729  0.321  1.341  0  2.127  2.395  0.782  1.699  1.569  2.097  3.355  3.059  1.57  2.192  3.452  2.282  0.27  1.199  3.39  0.557  3.567  2.249  3.38  0.159  3.737  2.971  
 
 TMEM51-AS1  200197  1  15438310  15478960  1p36.21  0.035  0.021  0.112  0  0.019  0.023  0.017  0.024  0.173  0.041  0.057  0.452  0.4  0.074  0.456  0.174  0.251  0.073  0.013  0.028  0  0.031  0.469  0  0  0.051  0.069  0.297  0.531  0.212  0.556  0.165  0.041  0.248  0.055  0.154  0.013  0  0.077  0.168  0.121  0.026  0.208  0.037  0.13  0.208  0.013  0.038  0  0.058  0.023  0.311  0.166  0.15  0.17  0.023  0.152  0  0.363  0.077  
 
 TMEM51  55092  1  15479027  15546974  1p36.21  1.609  1.191  2.096  1.913  0.529  0.549  2.965  2.517  1.682  2.243  2.409  3.312  2.779  2.23  2.938  3.019  2.843  0.905  0.091  0.154  0.417  0.421  2.26  0.642  1.768  2.518  3.15  2.463  4.219  2.281  3.946  3.175  2.751  2.032  2.501  2.312  2.706  1.973  2.51  2.093  2.115  1.885  2.52  1.599  2.169  2.266  4.159  2.064  2.384  3.306  1.7  2.34  3.688  1.807  4.169  2.424  3.019  2.256  4.225  3.16  
 
 C1orf195  727684  1  15490691  15498120  1p36.21  0  0  0  0  0  0  0  0.376  0  0.32  0.333  0  0  0.331  1.35  0.801  0.62  0  0  0  0  0.191  0  0  0  0.714  0.354  0.437  1.364  0.669  0.715  0.476  0.597  0  0.527  0.412  0.41  0.512  0  0  0.481  0.191  0  0  0  0.629  0  0.519  0  0  0  0.856  0.835  0  0.471  0.818  0.648  0  1.19  0  
 
 FHAD1  114827  1  15573767  15724622  1p36.21  0  0.025  0.513  0  0.032  0.028  0.123  0.105  0.124  1.395  0.143  0.14  0.08  0.403  0.095  0.159  0.113  0.111  0.046  0.081  0  0.088  0.747  0.036  1.059  0.283  0.167  0.963  0.124  0.08  0.036  0.082  0.076  0.193  0.063  0.021  0.114  0.107  0.104  0.088  0.066  0.725  0.057  0.025  0.195  0.086  0.184  0.113  0.058  0.07  0.129  0.429  0.201  0.222  0.185  0.137  0.303  0.073  0.811  0.037  
 
 EFHD2  79180  1  15736390  15756839  1p36.21  2.398  4.307  3.585  4.23  2.393  3.195  5.379  4.652  4.848  4.198  5.655  5.766  3.807  3.876  3.829  5.271  4.278  3.412  5.684  4.516  3.085  3.523  3.728  3.355  5.536  3.881  4.501  4.129  4.564  2.764  4.797  5.094  5.043  4.139  4.282  2.873  5.526  5.368  4.513  3.945  3.185  6.599  3.565  2.983  3.277  3.834  5.403  3.336  3.67  4.32  5.669  6.072  5.975  4.735  5.473  5.337  5.629  4.935  4.078  4.728  
 
 CTRC  11330  1  15764937  15773153  1p36.21  0  0  0  0  0  0  0.119  0.153  0  0.165  0.143  0  0  0.099  0  0  0  0.124  0.338  0.203  0  0.215  0  0  0  0  0.061  0  0  0.123  0  0  0  0  0  0  0  0  0.158  0  0  0.729  0  0  0  0  0.054  0  0  0  0  0.982  0.111  0.329  0  0  0  0  0  0  
 
 CELA2A  63036  1  15783222  15798586  1p36.21  0  0  0  0  0  0.157  0  0  0  0  0  0  0  0  0  0  0  0.21  0  0  0  0.144  0  0  0  0  0.111  0  0  0.076  0  0  0  0  0  0  0  0  0  0  0  0  0.06  0  0  0  0.058  0  0  0  0  0.447  0  0.149  0  0  0  0  0  0  
 
 CELA2B  51032  1  15802595  15817895  1p36.21  0  0  0  0  0  0  0  0  0  0.084  0  0  0  0  0  0  0  0  0  0  0  0.066  0  0  0  0  0.119  0  0  0.091  0  0  0  0  0  0  0  0  0.061  0  0  0.136  0  0  0  0  0  0  0  0  0  0.277  0  0  0  0  0  0  0  0  
 
 CASP9  842  1  15817895  15851285  1p36.21  0.669  0.377  1.049  1.031  1.304  0.661  2.734  1.558  1.7  1.732  2.31  1.215  1.316  1.848  1.25  0.982  1.557  0.858  1.492  1.065  1.895  1.135  1.542  1.108  0.566  1.398  2.149  1.793  1.699  2.333  3.176  1.54  1.307  0.841  1.042  1.886  2.036  1.258  0.718  1.817  1.35  1.573  3.609  0.953  1.322  1.343  2.899  0.837  1.224  1.448  1.538  1.071  1.488  0.589  1.424  0.957  1.532  1.458  0.827  1.153  
 
 DNAJC16  23341  1  15853307  15898228  1p36.1  1.128  1.837  0.886  1.274  1.226  0.758  1.662  1.422  1.567  1.71  1.635  1.119  1.788  2.153  1.51  1.381  1.378  1.713  1.324  1.188  1.282  1.32  1.342  0.781  0.795  1.278  1.035  1.3  1.878  0.673  1.483  1.611  1.261  0.944  0.798  1.487  1.71  1.351  1.131  1.911  1.171  0.655  1.603  1.15  1.722  1.169  0.893  0.769  1.524  1.313  1.794  0.678  1.525  0.56  1.221  1.041  1.551  1.028  1.025  1.653  
 
 AGMAT  79814  1  15898193  15911605  1p36.21  0.652  0.754  0.393  0.365  0.274  0.061  0.382  0.083  0.127  0.545  0.448  2.276  2.432  0.068  0.813  1.678  1.577  2.073  0.275  0.368  0  0.165  1.21  1.453  0.034  0.135  0.158  0.095  0.06  0.099  0.146  0.044  0  0.305  1.183  0.324  0.965  0.181  0.141  0.392  0.223  0.801  0.929  0.067  1.299  0.383  0.085  0.683  0.072  1.376  0.538  0.472  0.2  0.126  0.232  0.061  0.334  0.052  0  0.352  
 
 DDI2  84301  1  15943952  15987552  1p36.21  1.773  0.401  0.787  1.218  1.301  1.137  1.016  2.151  0.649  1.692  1.992  2.079  2.339  1.68  2.024  1.659  1.569  0.348  0.741  1.141  2.504  1.633  0.422  1.434  1.725  2.039  1.895  1.83  2.529  1.194  2.656  2.296  2.281  0.511  1.029  1.644  2.317  1.666  0.721  2.832  2.009  0.603  1.924  1.869  2.81  1.753  0.312  1.037  1.922  0.494  1.789  1.102  1.806  1.029  1.264  1.28  1.939  1.183  1.182  2.042  
 
 RSC1A1  6248  1  15986363  15988217  1p36.1  2.19  1.661  1.136  1.427  2.242  0.497  1.567  1.86  1.009  1.718  2.302  2.012  2.319  2.193  1.944  2.177  2.088  1.048  1.698  1.702  2.159  2.465  1.534  1.615  1.755  1.859  2.125  1.499  2.891  0.85  2.596  2.236  1.941  1.143  0.952  1.412  1.995  1.644  1.063  2.459  1.742  0.785  3.133  1.72  2.597  1.488  0.163  0.924  1.728  1.128  1.514  0.702  1.772  0.942  1.692  1.134  1.87  0.945  1.333  2.231  
 
 PLEKHM2  23207  1  16010826  16061264  1p36.21  2.188  0.354  3.047  3.811  1.984  2.557  3.556  3.752  3.462  3.894  3.168  1.451  2.7  2.471  2.643  2.251  2.15  0.163  3.708  1.42  3.054  2.821  2.459  2.317  2.285  2.727  3.786  3.603  4.086  3.051  4.031  3.832  4.271  2.414  1.756  2.912  3.763  3.224  2.489  3.349  2.419  2.77  3.094  2.724  3.779  3.288  0.622  2.69  3.543  3.702  3.073  2.429  3.193  2.178  2.957  3.024  3.296  3.544  3.045  3.058  
 
 SLC25A34  284723  1  16062808  16067887  1p36.21  0.22  0.046  0.433  0.295  0.452  0.368  0.779  0.116  0.239  0.687  0.34  0.138  0.091  0.317  0.172  0.257  0.302  0  0.427  0.413  0.091  0.661  0.659  0.073  0.062  0.286  0.798  0.697  0.278  0.32  0.124  0.322  0.826  0.353  0.277  0.162  0.572  0.183  0.77  0.083  0.096  0.359  0.248  0.113  0.595  0.432  0.146  0.337  0.125  0.38  0.088  0.554  0.302  0.274  0.403  0.192  0.426  0.27  0.173  0.1  
 
 TMEM82  388595  1  16068986  16074292  1p36.21  0.514  0.29  0.591  0.349  0.26  0.294  0.264  0.231  0.63  0.337  0.242  0.226  0.265  0.333  0.342  0.59  0.505  0.188  0.444  0.559  0  1.105  1.163  0.157  0.411  0.132  0.609  0.201  0.415  0.162  0.513  0.331  0.244  0.421  0.535  0.217  0.306  0.34  0.598  0.167  0.235  1.065  0.309  0  0.72  0.241  0.487  0.309  0.15  0.44  0.239  0.845  0.392  0.296  0.629  0.259  0.261  0.504  0.377  0.16  
 
 FBLIM1  54751  1  16085254  16113084  1p36.21  0.781  0.842  5.587  3.072  1.485  1.355  0.17  4.196  3.061  3.255  3.589  3.017  3.662  0.801  0.576  1.028  2.83  1.951  0.191  0.245  0.112  0.512  0.932  0.08  0.064  2.343  0.172  0.551  0.107  0.416  0.169  0.077  2.005  2.251  1.598  3.029  4.312  2.091  4.355  2.005  2.119  1.115  2.44  2.506  3.549  4.54  0.831  1.935  2.128  3.054  3.704  2.036  0.104  0.197  4.097  0.069  0.325  1.329  0.066  0.86  
 
 UQCRHL  440567  1  16133656  16134194  1p36.21  4.858  5.799  4.16  5.244  4.396  4.292  4.718  3.787  4.689  4.347  4.431  4.868  4.561  5.864  3.698  5.107  5.922  5.869  5.618  7.565  4.635  5.999  6.35  5.47  4.768  3.034  5.095  3.513  4.958  3.88  5.956  5.144  4.885  5.066  4.586  4.038  3.744  4.074  5.96  3.555  4.181  4.439  6.266  4.197  4.505  0.783  6.526  4.266  0  5.519  3.903  2.743  4.549  3.959  4.789  4.822  2.452  4.839  4.961  0  
 
 FLJ37453  729614  1  16160709  16174642  1p36.21  1.228  0.725  0.735  0.677  1.657  1.199  0.905  0.74  1.189  1.252  0.875  0.499  0.702  0.613  0.561  1.293  1.058  1.999  3.124  1.329  0.932  1.711  1.57  0.851  0.389  0.311  1.068  0.774  0.509  0.401  1.373  0.774  1.114  1.188  0.82  1.129  0.959  0.512  0.613  0.645  1.088  1.046  2.205  0.412  0.998  0.73  0.968  0.612  0.532  1.483  0.708  1.023  0.573  0.412  1.151  0.428  0.609  0.947  0.664  0.577  
 
 SPEN  23013  1  16174358  16266950  1p36  3.18  2.081  2.227  2.881  2.036  2.031  3.204  3.863  2.381  3.276  2.902  2.327  3.296  3.467  3.342  2.881  2.673  2.251  2.893  3.175  3.053  3.633  2.199  2.737  2.601  2.423  3.432  2.873  3.379  2.294  3.208  2.953  3.408  1.502  2.072  2.597  3.363  2.882  2.243  3.796  3.152  2.069  3.343  2.642  3.288  2.816  2.144  3.057  2.881  2.356  3.081  2.078  3.156  2.005  3.043  2.312  3.388  2.359  2.885  2.776  
 
 ZBTB17  7709  1  16268363  16302627  1p36.13  1.576  0.363  1.607  1.985  1.708  1.41  3.146  2.577  1.811  2.717  2.087  1.081  1.741  1.798  1.923  1.453  1.412  0.269  3.078  1.131  2.144  2.502  1.403  1.322  1.384  1.086  2.584  2.636  2.64  2.207  2.468  2.924  2.632  1.137  1.02  1.541  2.63  1.996  1.466  2.556  1.67  1.795  3.332  1.332  2.472  1.447  0.904  2  1.472  2.977  1.861  1.907  2.08  0.952  1.97  1.398  2.131  2.102  1.348  1.556  
 
 C1orf64  149563  1  16330730  16333190  1p36.13  0  0  0  0  3.323  0  0  0.094  0.136  0  0  0  0  0.059  0  0  0  0.215  0  0  0  0.051  0.619  0  0  0  0.126  0  0  0.099  0  0  0  0  0  0  0  0  0.056  0  0  0.381  0  0  0  0  0  0  0  0  0  0.522  0  0  0  0  0  0  0  0  
 
 HSPB7  27129  1  16340522  16345285  1p36.23-p34.3  0.171  0  2.061  0.15  0.079  0.111  0.036  6.309  0.238  2.973  0.157  0.09  0  0.069  0  0.07  0.049  0.076  0  0.062  0.136  0.179  0.491  0.147  0  0.127  0.212  0.052  0.113  0.082  0.155  0.07  0.056  0.188  0  0.299  0  0.702  0.094  0  0  0.148  0.049  0.063  0.451  0.071  0.028  0.076  0  0.044  0.055  0.379  0.058  0.186  0.127  0  0.183  0  0  0  
 
 CLCNKA  1187  1  16348485  16360545  1p36  0.112  0  0.061  0  0.151  0.145  0  0.358  0.091  0.092  0.169  0  0  0.085  0  0  0  0  0  0  0  0.133  0.379  0.068  0  0  0.076  0.035  0  0.112  0.097  0  0  0.13  0  0.469  0  0  0.039  0  0  0.232  0.063  0  0.252  0.127  0.04  0  0  0.352  0  0.493  0  0.065  0.111  0  0  0  0  0  
 
 CLCNKB  1188  1  16370230  16383821  1p36  0.113  0  0.093  0  0.1  0  0  0.076  0.129  0.067  0.06  0  0  0.203  0  0.069  0  0  0.047  0  0.297  0.142  0.5  0  0  0  0.112  0.097  0  0.119  0  0  0  0.139  0  0.577  0.068  0  0.048  0.149  0  0.369  0.065  0.248  0.431  0.201  0.042  0.471  0  1.828  0.047  0.585  0  0.078  0.309  0.119  0  0  0.205  0  
 
 FAM131C  348487  1  16384263  16400127  1p36.13  1.067  0  0.848  0.764  0.238  0.409  1.246  0.797  0.766  0.494  0.644  0.085  0.948  2.162  0.545  0.23  0.935  0.089  0  0.071  0  0.112  1.049  0.379  0.359  0.07  0.139  0.1  0.06  1.544  0  0.526  0  0.162  1.876  1.619  0.531  0.704  0.49  1.694  0.602  1.254  1.189  0.126  1.715  0.166  0.584  1.19  0.426  2.674  1.083  1.664  0.831  0.332  2.123  0.757  0.483  1.315  1.262  0.784  
 
 EPHA2  1969  1  16450831  16482582  1p36  0.719  1.883  3.276  5.095  0.774  3.8  5.456  5.779  4.16  3.201  4.94  1.812  4.487  4.04  3.588  4.569  4.359  1.002  0.196  0.156  0.088  0.539  0.639  0.287  3.562  2.27  0.491  3.707  0.747  0.959  3.518  2.57  1.21  0.993  3.056  4.01  6.291  4.489  3.144  4.743  4.479  2.731  4.195  2.564  5.879  3.867  3.933  4.285  4.709  4.887  5.713  3.596  4.852  2.902  4.251  4.571  3.645  4.633  4.165  4.649  
 
 ARHGEF19  128272  1  16524598  16539104  1p36.13  2.192  0.112  1.003  1.188  2.48  0.391  0.653  1.052  0.336  0.901  0.251  1.576  1.846  2.716  0.819  2.426  1.08  0.111  1.717  0.347  0.662  1.276  1.324  0.714  0.505  0.657  1.399  1.122  0.966  0.527  0.911  0.934  1.198  0.629  0.515  1.076  2.091  1.678  0.904  1.99  0.549  1.255  1.088  0.615  1.787  1.795  1.012  1.389  0.369  3.368  0.812  1.522  0.676  0.796  1.865  0.912  1.695  1.74  1.703  0.87  
 
 RSG1  79363  1  16558181  16563659  1p36.13  0.753  0.166  0.656  0.768  0.674  0.435  0.746  0.459  0.617  0.707  0.551  0.845  1.115  0.577  0.711  0.759  0.608  0.219  0.494  0.21  0.973  0.43  1.113  0.866  0.427  0.482  0.999  0.956  0.97  0.403  1.128  0.863  1.065  0.566  0.223  0.776  1.064  0.865  0.346  1.071  0.634  0.678  0.51  0.682  0.598  0.66  0.22  0.467  0.527  1.069  0.788  0.925  0.422  0.461  0.478  0.829  0.275  0.477  0.251  0.693  
 
 FBXO42  54455  1  16573334  16678986  1p36.23-p36.11  1.706  1.172  1.56  1.743  1.553  1.217  2.288  1.925  1.911  2.061  2.153  1.14  1.82  2.081  1.772  1.524  1.606  0.807  2.614  1.536  1.556  1.987  1.383  1.106  0.764  1.185  1.877  1.708  2.213  1.298  2.055  1.917  1.78  0.844  0.858  1.467  2.074  1.708  1.113  2.417  1.432  0.926  2.684  1.879  1.488  1.828  1.871  1.379  1.768  2.26  1.876  0.92  1.458  0.61  1.152  1.16  2.091  1.294  1.309  1.486  
 
 SZRD1  26099  1  16693524  16724643  1p36.13  3.98  2.128  3.884  4.289  3.728  4.336  4.989  4.892  4.056  4.925  5.381  4.095  4.301  4.373  4.232  4.322  4.053  1.093  4.941  3.567  3.972  3.971  3.36  4.034  4.335  3.668  4.524  4.366  4.809  3.694  4.875  4.77  5.097  3.367  3.639  4.105  5.087  4.749  3.448  5.359  4.169  3.631  4.675  4.225  4.031  4.358  3.53  3.975  4.58  4.72  4.996  3.152  4.654  3.122  4.302  4.065  4.627  4.729  4.135  4.803  
 
 SPATA21  374955  1  16725137  16763919  1p36.13  0  0  0.179  0  0.056  0.296  0.049  0.049  0.196  0.039  0.067  0  0  0.054  0  0.062  0.062  0  0.049  0.074  0  0.085  0.6  0  0  0.051  0.104  0  0.073  0.065  0  0  0  0  0  0  0  0  0.086  0  0  0.091  0.205  0  0.094  0.059  0.259  0.11  0  0  0.042  0.388  0.052  0.061  0.09  0  0.061  0  0  0  
 
 NECAP2  55707  1  16767166  16786584  1p36.13  2.178  2.56  3.087  3.511  2.555  2.208  3.889  3.473  3.595  3.412  3.392  2.224  2.749  2.893  2.225  3.04  2.935  2.499  4.658  2.674  3.318  3.158  4.06  2.531  2.32  1.723  3.386  2.574  2.844  1.709  2.846  2.9  3.255  2.804  1.668  2.416  3.516  2.784  2.704  3.437  2.343  2.514  3.637  2.389  2.34  2.391  3.389  2.685  2.633  4.638  3.459  1.912  3.324  2.045  3.222  2.655  3.32  2.657  3.052  3.336  
 
 CROCCP3  114819  1  16793930  16819196  1p36.13  0.426  0.234  0.341  0.267  0.216  0.18  0.273  0.47  0.48  0.882  0.506  0.13  0.289  0.637  0.754  0.16  0.206  0.273  0.706  0.092  0.178  0.42  0.613  0.131  0.089  0.313  0.815  0.369  0.543  0.254  0.521  0.189  0.6  0.299  0.254  0.456  0.566  0.678  0.281  0.391  0.222  0.384  0.949  0.292  0.166  0.23  0.224  0.47  0.4  0.54  0.321  0.557  0.508  0.192  0.43  0.331  0.704  0.35  0.286  0.145  
 
 LOC100133301  100133301  1  16865218  16866530  1p36.13  0.199  0  0.56  0  0.304  0  0.285  0  0.416  0.137  0.166  0.205  0.328  0.109  0.311  0.197  0.174  0  0.246  0.121  0.391  0.312  1.139  0.43  0.395  0.334  0.164  0.243  0  0.143  0  0  0  0.86  0.236  0.273  0.346  0  0.063  0.296  0  0.078  0.122  0.153  0  0  0.161  0  0.217  0.217  0.364  0.328  0.25  0.161  0.079  0.164  0.18  0  0.43  0.385  
 
 NBPF1  55672  1  16888921  16940100  1p36.13  2.12  1.865  1.209  1.207  2.336  1.509  2.075  1.406  1.322  2.494  1.746  0.471  1.502  1.594  1.698  0.765  1.33  0.739  2.966  0.26  0.969  1.845  0.999  0.487  0.402  0.963  1.658  1.129  1.18  1.31  1.962  1.181  1.499  0.867  1.217  2.057  1.675  2.592  0.756  2.373  0.759  0.746  2.801  1.34  0.436  0.92  1.237  1.954  2.334  3.115  3.784  1.238  1.479  0.678  1.15  1.074  1.865  1.571  1.234  0.944  
 
 CROCCP2  84809  1  16944750  16957401  1p36.13  2.3  2.659  1.515  1.815  2.422  0.852  1.92  1.63  2.018  2.719  1.844  1.292  2.104  2.824  2.313  1.995  2.767  4.095  4.762  2.817  2.322  3.024  2.743  1.887  1.08  1.679  2.742  1.892  2.831  2.111  2.758  2  2.341  1.664  1.306  1.692  1.708  2.031  1.655  2.283  1.601  1.53  3.87  1.558  1.231  1.042  2.122  2.15  1.328  4.058  1.965  1.252  1.509  0.74  2.084  1.263  1.273  1.887  1.704  1.152  
 
 MST1P2  11209  1  16972068  16976915  1p36.2  0.681  0.071  0.381  0.222  0.787  0.166  0.156  0.151  0.546  0.55  0.368  0.08  0.346  1.229  0.531  0.183  0.219  0.21  1.255  0.188  0.161  0.522  1.104  0.138  0.057  0.341  0.969  0.361  0.479  0.227  0.721  0.41  0.261  0.522  0.194  0.632  0.198  0.205  0.333  0.53  0.292  0.397  0.631  0.675  0  0.151  0.651  0.224  0.157  1.048  0.461  0.686  0.103  0.177  0.478  0.221  0.387  0.141  0  0.199  
 
 ESPNP  284729  1  17017712  17046652  1p36.13  0.304  0.066  0.162  0  0.777  0.11  0  0.076  0.258  0.177  0.14  0.181  0.083  0.302  0.07  0.188  0.26  0.39  0.041  0.158  0  0.399  0.563  0.17  0  0.053  0.212  0.385  0.077  0.205  0  0.086  0  0.278  0.107  0.066  0.091  0  0.161  0.095  0  0.755  0.137  0  0  0.121  0.066  0  0  0.044  0.148  1.395  0.059  0.282  0.184  0.248  0.06  0  0  0  
 
 LOC729574  729574  1  17060446  17060956  1p36.13  0  0  0  0  0  0  0.24  0  0  0  0  0  0  0  0  0  0  0  0  0  0  0  0  0  0  0  0.202  0  0  0  0  0  0  0  0  0  0  0  0  0  0  0.655  0  0  0  0  0  0  0  0  0  0  0  0  0  0  0  0  0  0  
 
 MST1L  11223  1  17081128  17090975  1p36.13  0.264  0  0.303  0  0.544  0.041  0.037  0.041  0.377  0.176  0.28  0.035  0.197  0.703  0.229  0.062  0.118  0.099  0.272  0.074  0  0.329  0.692  0.114  0.048  0.179  0.455  0.204  0.341  0.109  0.343  0.19  0.084  0.552  0.076  0.127  0.089  0.208  0.106  0.189  0.092  0.359  0.603  0.263  0  0.058  0.033  0.062  0.095  0.33  0.15  0.411  0.089  0.117  0.274  0.143  0.218  0.116  0.043  0.078  
 
 CROCC  9696  1  17248444  17299474  1p36.13  0.833  0.079  0.456  0.432  0.868  0.496  0.638  0.991  0.726  0.933  0.586  0.185  0.725  1.045  1.18  0.478  0.493  0.199  1.7  0.107  0.172  0.776  0.916  0.661  0.178  0.921  1.337  1.194  1.665  1.068  1.685  0.667  0.845  0.37  0.403  0.378  0.825  0.606  0.252  0.903  0.36  0.911  1.241  0.439  0.315  0.273  0.202  0.524  0.509  0.949  0.455  1.048  0.629  0.412  0.635  0.349  0.879  0.387  0.62  0.29  
 
 MFAP2  4237  1  17300998  17308081  1p36.1-p35  2.129  0.507  4.781  4.746  1.856  1.759  0.237  2.405  0.26  3.283  1.164  0  0.871  4.422  0  0.419  0.704  0.295  0.099  0.134  0  0.167  1.492  0.494  3.911  3.198  0.82  1.426  0.202  0.096  0.455  0.498  0  0.229  0  2.414  0.844  0.498  3.19  3.002  0  0.78  1.294  2.561  3.085  1.988  0.119  1.953  0.33  4.167  0.521  1.032  0  0.086  0.09  0  1.611  0.551  0  0.936  
 
 ATP13A2  23400  1  17312452  17338467  1p36  1.859  0.359  1.976  1.897  2.744  1.866  2.88  3.91  4.241  3.031  3.381  2.327  3.846  2.635  3.33  2.996  3.182  0.193  3.457  1.547  2.349  2.411  2.013  2.627  2.527  3.149  4.064  3.431  4.093  2.441  4.1  4.181  3.593  1.942  1.454  2.948  3.678  2.213  2.107  3.65  2.756  2.531  3.244  2.635  1.931  2.141  2.047  2.237  2.634  3.001  2.731  2.023  2.419  1.748  2.625  2.014  2.65  1.546  2.853  3.588  
 
 SDHB  6390  1  17345224  17380665  1p36.1-p35  4.986  5.693  3.96  4.016  5.208  4.42  6.47  5.54  4.338  4.902  6.294  5.12  5.707  6.657  4.839  4.959  5.83  6.535  6.87  6.34  5.834  5.053  4.46  4.985  4.904  4.753  6.016  4.894  6.218  5.593  6.813  6.788  6.054  4.282  4.471  4.554  5.495  4.933  4.846  5.815  5.025  4.066  6.395  5.04  4.563  5.289  5.675  4.808  4.53  6.878  5.645  3.47  5.225  3.508  5.251  4.793  4.949  5.769  4.758  5.192  
 
 PADI2  11240  1  17393255  17445948  1p36.13  1.157  0.026  0.134  0.297  1.237  0.036  0.083  0.037  0.109  0.167  5.578  0.087  0.11  0.318  0.125  1.313  0.046  0.208  0.136  0.464  0  0.469  0.468  0.139  0.032  0.06  0.241  0.068  0  0.348  0.052  0.024  0  0.178  0.462  0.622  0.038  0.043  0.096  0.645  2.015  0.167  3.42  2.141  0.322  1.737  0.305  0.044  0.851  0.195  0.037  0.755  0.018  0.147  2.041  4.012  0.087  0  0.151  4.196  
 
 PADI1  29943  1  17531620  17572501  1p36.13  0  0.044  0.199  0.176  0.041  0.932  0.193  0.21  0.207  0.093  1.025  2.983  0.1  0.024  0  3.091  0.224  0.045  0.065  0.038  0.051  0.058  0.429  0  0  0  0.067  0.032  0  0.073  0.189  0  0  0.207  0.288  0.156  0.784  0.096  0.199  0.069  1.057  0.153  0.575  0.982  0.082  0.143  0.045  0  2.946  0.177  0.078  0.362  0.365  0.284  1.554  0.75  0.111  0  0.104  2.437  
 
 PADI3  51702  1  17575592  17610727  1p36.13  0.578  0  0.107  0.336  2.369  0  0.841  0.128  0.3  0.106  5.402  0.033  0  0.277  0  0.061  0  0  0  0.037  0  0.096  0.456  0  0  0  0.088  0.497  0.304  0.064  0.11  0.17  0  0.171  0.284  0.132  0.102  0  0.078  0.668  0.247  0.146  0.058  0.099  0  0.071  0.037  0  0.099  0  0.023  0.426  0.051  0.393  7.445  3.007  0  0  0  0.468  
 
 PADI4  23569  1  17634689  17690495  1p36.13  0.104  0  0.179  0  0.211  0  0.174  0.141  0.096  0.274  1.076  0.201  0.311  0.043  0  0.308  0.272  0.272  0.403  0.484  0  0.127  2.608  0.248  0  0.071  0.13  0.223  0.178  0.187  0.22  0.119  0  0.215  0  0.069  0.191  0  0.169  0.095  0.218  0.202  0.272  0  0.09  0  0.089  0  0  0  0.068  0.412  0.04  0.035  0.905  0.079  0.083  0  0.084  0.094  
 
 PADI6  353238  1  17698690  17728195  1p36.13  0  0  0.068  0  0.056  0  0  0  0.097  0.035  0  0  0  0.07  0  0.07  0  0  0.029  0.045  0  0.039  0.294  0  0  0  0.1  0.028  0  0.101  0  0  0  0.107  0  0  0  0  0.033  0  0  0.031  0.044  0  0  0  0.054  0  0  0  0.042  0.236  0  0.055  0.035  0  0  0  0  0  
 
 RCC2  55920  1  17733250  17766250  1p36.13  4.197  1.723  3.231  4.153  3.948  2.925  4.267  4.345  4.273  4.703  5.37  4.339  4.367  4.178  4.109  4.719  4.482  1.691  6.251  3.863  4.789  4.86  4.861  3.755  3.183  3.536  4.754  3.925  4.21  2.825  4.953  5.085  4.792  2.733  3.093  3.508  4.59  2.996  3.142  4.132  4.07  3.623  4.663  4.431  3.767  4.228  2.018  3.028  4.441  4.553  3.445  2.681  4.174  2.943  3.839  3.67  4.097  3.864  3.633  4.071  
 
 ARHGEF10L  55160  1  17866329  18024370  1p36.13  2.055  0.255  1.147  0  1.368  0.515  1.116  1.991  1.48  2.028  1.571  2.625  2.378  0.897  1.658  1.925  1.383  1.042  0.03  0.091  0  0.181  0.958  0.243  0.035  0.691  0.726  0.867  0.282  0.765  1.136  1.082  1.264  0.593  0.62  1.329  0.95  0.987  0.709  0.836  2.032  1.034  1.151  0.692  0.578  1.299  1.024  0.642  1.095  1.309  0.037  0.937  1.26  1.131  1.321  1.036  1.834  0.983  1.423  1.166  
 
 ACTL8  81569  1  18081807  18153558  1p36.2-p35  0.278  0.056  0.167  0.147  0.131  0  0.224  0.075  0.139  0.159  0.066  0.101  1.385  0.096  0.13  0.065  0.077  0  0.282  0.817  0.391  0.142  5.347  3.081  0  0  0.211  2.337  0.087  0.125  0.072  0.685  0.852  0.671  0.205  0  0  0.08  0.066  0.49  0.081  0.557  0.054  0.114  0.193  0.676  0.927  0  0  0.324  0  0.868  0  0.617  0.132  0  0  0.079  0  0  
 
 LOC101927876  101927876  1  18392150  18400905  1p36.13  0  0  0.119  0  0  0  0  0  0.164  0  0  0  0  0  0  0  0  0.067  0  0  0  0.055  0.455  0  0  0  0.065  0  0  0.04  0  0  0  0.194  0  0  0  0  0.024  0  0  0.184  0.029  0  0  0  0.023  0  0  0  0  0.127  0  0.035  0  0  0  0  0  0  
 
 IGSF21  84966  1  18434239  18704977  1p36.13  0  0  0.107  0  0.101  0  0  0.043  0.16  0.09  0.049  0.102  0  0.063  0  0.084  0  0.159  0  0.149  0  0.223  0.433  0  0  0  0.251  0.046  0.055  0.198  0  0  0  0.315  0.105  0  0  0  0.134  0  0  0.375  0.116  0.07  0  0.091  0.065  0  0  0  0.073  0.853  0.056  0.186  0.104  0.08  0  0  0  0  
 
 KLHDC7A  127707  1  18807423  18812480  1p36.13  0.053  0.018  0.146  0  1.26  0.114  0.062  0.044  0.551  0.077  0.044  0.103  0.031  0.098  0.339  1.594  0.076  0.228  0.031  0.087  0.042  0.275  0.568  0.062  0.032  0.041  0.133  0.032  0.037  0.132  0  0  0  0.325  0.718  0.849  0.025  0  0.314  0  0.195  0.277  0.077  1.936  0  0.564  0.776  0  1.165  0.024  0.038  0.677  0.778  0.827  1.201  0.147  0.532  0  0.905  1.304  
 
 PAX7  5081  1  18957499  19075360  1p36.13  0  0.042  0.23  0  0.053  0.085  0.085  0.095  2.122  1.964  0.394  0.093  0  0.074  0  0.111  0.07  0.1  0.061  0.146  0  0.305  0.622  0  0  0.058  0.247  0.061  0  0.177  0.05  0  0  0.397  0.36  0  0.03  0  0.106  0  0  0.695  0.104  0  0.067  0.121  0.076  0  0  0.036  0.044  0.732  0.075  0.124  0.104  0.135  0.044  0  0  0  
 
 TAS1R2  80834  1  19166092  19186155  1p36.13  0  0  0.182  0  0.044  0.077  0  0.029  0.142  0.062  0.031  0.056  0  0.041  0  0.041  0.067  0.367  0  0.109  0  0.249  0.451  0  0  0  0.125  0.031  0  0.139  0  0.09  0  0.28  0.049  0  0  0  0.101  0  0  0.252  0.095  0  0  0.051  0.028  0  0  0  0.039  0.931  0.033  0.228  0.093  0  0  0  0  0  
 
 ALDH4A1  8659  1  19197923  19229293  1p36  2.988  0.093  1.677  1.635  3.187  1.099  1.664  1.534  1.21  2.189  2.006  3.461  2.167  1.522  2.448  2.481  1.951  0.191  2.837  0.783  2.085  1.121  1.465  1.111  0.606  3.358  2.598  1.963  2.028  1.448  2.918  2.316  1.96  1.048  1.098  2.272  2.187  1.338  0.828  2.388  1.552  1.703  2.132  1.917  0.963  1.986  0.667  1.726  0.958  3.37  2.051  1.44  1.959  0.612  1.829  1.667  1.514  2.129  2.762  2.481  
 
 IFFO2  126917  1  19230773  19282826  1p36.13  0.584  0.707  1.328  0.966  0.614  0.801  1.382  3.605  1.428  2.137  3.32  2.18  0.548  1.083  1.07  1.907  1.299  0.181  1.203  0.267  0.182  0.549  0.8  0.976  0.898  1.257  1.366  1.595  1.027  0.499  1.473  1.41  1.06  0.647  1.814  1.948  1.476  1.669  0.54  0.797  1.162  1.248  1.557  1.102  0.929  1.311  1.151  0.783  2.123  1.628  1.768  1.575  1.881  0.762  1.528  2.524  1.955  1.775  2.183  1.744  
 
 UBR4  23352  1  19400999  19536746  1p36.13  2.88  2.136  2.13  2.102  2.253  2.427  3.524  3.753  1.724  3.526  3.91  2.632  3.355  3.224  3.585  3.009  2.938  0.812  2.659  2.369  3.894  2.593  1.786  2.41  2.097  2.554  3.23  4.091  3.973  2.84  3.846  3.705  3.528  1.304  2.329  3.453  4.299  3.42  2.531  4.523  3.028  1.857  3.801  3.081  2.555  3.107  2.719  3.552  3.55  3.317  3.622  2.524  3.102  2.003  3.274  2.892  3.685  3.074  3.149  3.199  
 
 EMC1  23065  1  19542157  19578053  1p36.13  2.26  1.881  2.411  2.509  1.982  2.01  3.125  3.606  2.322  3.426  3.373  1.867  2.746  3.046  2.623  2.116  2.056  1.483  2.428  2.002  2.544  1.599  1.976  2.143  1.967  2.416  2.688  3.156  3.69  1.759  2.934  3.605  2.872  1.388  1.298  2.523  3.728  3.174  1.675  3.786  2.404  1.789  2.889  2.75  2.18  2.245  2.215  2.097  2.313  2.96  3.205  1.437  3.058  1.472  2.03  2.023  3.169  2.662  2.118  3.103  
 
 MRTO4  51154  1  19578074  19586622  1p36.13  3.034  3.396  2.517  3.308  3.288  3.683  4.128  3.836  2.812  3.074  4.387  3.567  3.535  4.1  3.347  3.968  3.327  2.244  5.017  4.076  4.364  3.809  3.153  3.518  3.548  2.867  2.585  3.439  3.607  2.142  4.433  4.731  3.937  2.741  3.157  3.211  4.436  3.519  2.728  4.117  3.536  2.938  3.697  3.824  3.287  3.415  2.724  3.419  3.569  4.897  3.78  2.728  4.068  1.99  3.618  2.949  3.734  4.307  3.562  3.803  
 
 AKR7L  246181  1  19592475  19600568  1p36.13|1p35-p36.1  0.381  0.346  0.214  0.165  0.547  0.271  0.43  0.378  0.264  0.529  0.456  0.516  0.519  0.481  0.378  0.939  0.345  0.892  1.151  0.366  0  0.39  0.519  0.11  0.073  0.191  0.611  0.117  0.301  0.266  0.468  0.273  0.29  0.334  0.127  0.138  0.207  0.227  0.189  0.29  0.258  0.275  0.801  0.187  0  0.276  0.144  0.131  0.392  0.269  0.252  0.336  0.322  0.085  0.253  0.175  0.212  0.282  0.147  0.093  
 
 AKR7A3  22977  1  19609056  19615280  1p36.13  0.437  0.231  0.255  0  1.126  0.363  0.454  0.529  0.181  0.695  0.718  3.066  1.711  0.61  0.536  2.213  1.508  0.603  1.433  0.705  0.197  0.404  0.383  0.142  0  0.326  0.929  0.499  0.445  0.299  0.686  0.398  0.238  0.198  0  0.241  0.321  0.431  0.225  0.357  0.322  0.382  0.424  0.298  0  0.597  0.084  0.19  0.237  0.323  0.36  0.542  0.369  0.254  0.379  0.16  0.338  0.532  0.272  0  
 
 LOC100506730  100506730  1  19619740  19622230  1p36  0.964  0.407  0.383  0.713  1.286  0.745  1.212  1.544  0.823  1.729  1.852  0.836  1.365  1.295  1.329  0.732  0.803  1.168  1.976  0.862  0.811  0.945  0.772  0.656  0.235  0.955  0.728  0.364  0.478  0.127  1.166  0.781  0.59  0.564  0.37  0.767  0.841  0.826  0.567  0.821  0.693  0.45  1.442  0.723  0.754  1.168  0.275  0.575  1.201  0.78  1.361  0.663  0.712  0.214  0.576  0.531  0.795  1.28  0.451  0.569  
 
 AKR7A2  8574  1  19629201  19638640  1p36.13  3.359  2.418  3.077  2.376  4.119  2.445  4.26  3.853  3.321  4.079  3.644  3.097  3.433  4.517  2.87  3.564  3.425  1.951  5.047  4.215  2.597  3.154  3.105  3.033  2.255  3.451  3.884  3.313  4.193  2.943  4.545  4.49  3.548  2.441  2.504  3.191  3.471  3.33  3.086  3.533  3.214  2.953  4.664  2.783  2.832  2.972  3.452  2.505  2.575  4.056  3.242  2.458  3.184  1.585  3.542  2.862  3.079  3.452  2.418  3.385  
 
 PQLC2  54896  1  19638739  19658922  1p36.13  1.763  1.198  2.027  2.719  2.269  1.976  3.344  3.163  2.654  2.79  3.095  1.173  2.475  1.878  1.962  2.011  1.715  0.506  2.085  1.31  2.281  1.342  2.125  0.984  1.461  2.711  3.238  2.8  3.56  2.749  4.005  3.623  2.999  1.894  1.589  2.337  3.024  3.108  1.836  3.268  2.086  1.441  3.901  1.797  1.85  2.13  2.061  1.825  1.437  3.006  2.242  2.01  2.552  1.477  2.84  2.161  2.516  2.213  1.967  2.31  
 
 CAPZB  832  1  19665266  19812135  1p36.1  4.487  3.896  5.483  4.893  5.327  4.507  5.859  6.407  5.108  6.201  5.776  4.661  4.836  4.989  4.645  5.019  5.232  3.515  6.738  4.497  4.804  4.736  4.335  4.251  4.553  4.403  5.037  4.78  5.612  3.678  5.872  5.913  5.567  4.274  4.194  5.36  5.899  5.067  4.591  5.675  4.876  4.611  5.68  4.989  5.047  5.389  3.348  4.536  5.49  6.245  5.308  4.033  5.704  3.996  4.988  4.781  5.767  5.761  5.153  5.767  
 
 MINOS1  440574  1  19923470  19956315  1p36.13  2.597  5.348  2.313  2.394  3.561  3.46  3.882  3.123  2.444  3.674  3.636  3.414  2.511  3.148  1.745  3.47  3.671  6.481  4.29  4.932  3.435  4.073  2.964  3.157  2.335  2.156  2.763  1.468  2.964  1.775  4.188  3.575  3.068  2.455  2.953  2.703  2.441  2.601  4.053  2.56  2.664  2.574  3.202  2.645  3.312  3.547  3.96  1.964  2.842  3.615  2.709  1.641  2.831  2.537  3.184  2.482  2.326  2.644  2.329  2.832  
 
 MINOS1-NBL1  100532736  1  19923470  19984949  1p36.13  0.696  0  0.62  1.441  0.414  0  0.671  0.374  0.643  0.527  0.334  0  0.227  0.063  0.381  2.771  2.446  0  0.182  0  0.779  0  0  0  0  0  2.404  0.353  0.505  0.785  1.076  0.285  0  0.596  0  0  0.089  0.901  0  0.311  0  0.076  0  0  2.31  1.216  0  0.407  0  0  0  0.931  0.425  0.125  2.018  0  0.248  1.445  0.36  0  
 
 RPS14P3  644068  1  19934300  19935138  1p36.13  3.673  2.187  2.237  2.962  4.017  2.629  3.679  2.05  3.133  2.884  2.411  2.513  2.418  3.699  1.969  3.758  4.355  2.422  3.957  5.252  2.503  4.848  3.331  3.123  2.847  1.131  3.546  2.157  2.58  3.666  2.539  2.636  1.986  2.802  3.274  1.706  2.129  1.861  5.438  1.69  1.43  3.645  4.447  2.468  2.295  2.819  2.135  2.224  1.854  4.375  1.866  2.222  3.133  2.701  4.01  2.873  1.718  3.008  2.536  1.374  
 
 NBL1  4681  1  19969722  19984949  1p36.13  0.567  0.09  3.654  3.495  4.022  2.667  3.714  6.379  0.941  5.989  1.817  4.464  4.766  3.732  4.08  3.42  4.488  0.094  2.304  0.348  0  0.431  0.974  0.871  1.687  4.167  5.597  3.672  6.722  5.469  7.407  6.783  5.122  3.572  2.723  2.946  4.746  4.588  1.835  3.34  2.995  3.233  3.465  2.594  5.379  3.534  2.735  3.422  4.659  4.369  4.267  2.237  4.895  3.75  3.224  4.502  4.223  3.706  4.39  5.257  
 
 HTR6  3362  1  19991779  20007459  1p36-p35  0.302  0.039  0.174  0  0.082  0.077  0.083  0.087  0.161  0.264  0.037  0.075  0.288  0.213  0.116  0.09  0.065  0.12  0.046  0.162  0  0.297  0.601  0.133  0.06  0.043  0.202  0.037  0.176  0.233  0.2  0.265  0.079  0.293  0.223  0.231  0  0.072  0.154  0.335  0.061  0.759  0.484  0.043  0.354  0.133  0.044  0.072  0.055  0.032  0.081  1.014  0.055  0.372  0.17  0.088  0.065  0.105  0  0  
 
 TMCO4  255104  1  20008705  20126410  1p36.13  2.115  1.622  0.944  0.289  1.74  1.507  3.246  2.202  1.377  1.504  1.436  1.958  1.694  2.134  1.845  1.739  1.466  0.953  1.111  0.9  0.194  0.117  1.638  1.55  0.838  1.47  2.868  1.411  1.756  2.089  2.598  1.598  2.904  1.743  0.694  1.9  1.619  1.901  0.529  1.95  1.848  1.087  1.634  0.956  1.516  1.845  2.64  0.89  0.634  1.728  1.926  1.077  1.654  1.22  1.818  1.25  2.101  1.408  1.662  1.623  
 
 RNF186  54546  1  20140521  20141771  1p36.13  0  0  0.15  0  0  0  0.145  0  0.225  0  0  0.202  0  0  0  0.072  0  0  0  0.076  0  0.181  0.716  0.111  0  0  0.137  0  0  0.078  0  0  0  0.392  0  0.112  0  0  0.054  0  0  0.097  0.046  0  0.144  0  0.039  0  0  0  0.062  0.772  0  0.108  0  0  0  0  0  0  
 
 OTUD3  23252  1  20208887  20239437  1p36.13  0.697  0.935  0.701  0.437  0.809  0.896  0.985  1.148  0.768  0.975  1.567  1.239  0.656  1.559  1.064  0.767  1.058  1.631  1.94  0.84  0.694  1  0.623  0.365  0.452  0.627  0.879  0.852  1.357  0.34  1.449  1.452  0.892  0.587  0.403  1.129  1.28  0.568  0.475  1.206  0.746  0.501  1.619  1.143  0.801  0.829  0.824  0.572  1.257  1.425  1.408  0.767  0.905  0.273  0.596  0.427  0.85  0.976  0.622  0.433  
 
 PLA2G2E  30814  1  20246799  20250110  1p36.13  0  0  0  0  0  0  0  0  0  0  0  0  0  0  0  0  0  0  0  0  0  0.145  0  0  0  0  0.079  0  0  0  0  0  0  0  0  0  0  0  0  0  0  0  0  0  0  0  0  0  0  0  0  0  0  0  0  0  0  0  0  0  
 
 PLA2G2A  5320  1  20301923  20306932  1p35  0  0  0.517  0  0  0  0  0  0  0.099  0  10.517  0  0.09  0  0.219  0.424  0  0  0  0  0  0  0  0  0  0.113  0  0  0.105  0  0  0  0  1.327  0.83  0  0  0  0  0  0  0  0  0  0  0  0  0  0  0  0.477  0  0  0  0  0  0  0  0  
 
 PLA2G5  5322  1  20396700  20418394  1p36-p34  0  0  0.083  0  0  0  0  0  0.141  0.081  0  0.049  0  0  0  0  0  0  0  0  0  0.109  0.404  0  0  0  0.191  0  0  0.051  0  0  0  0.187  0  0  0.063  0  0  0  0  0  0.038  0  0  0  0.057  0  0  0  0  0.193  0  0.043  0  0  0  0  0  0  
 
 PLA2G2D  26279  1  20439142  20446059  1p36.12  0  0  0.184  0  0  0  0.096  0  0.163  0  0  0  0  0.03  0  0  0  0  0  0  0  0.044  1.05  0.274  0  0  0.213  0  0  0.04  0  0  0  0.328  0  0  0  0  0  0  0  0.096  0.025  0  0  0.046  0.023  0  0  0  0  0.207  0  0.052  0.039  0  0  0  0  0  
 
 PLA2G2F  64600  1  20465822  20476879  1p35  0  0  0.117  0  0.021  0  0  0  0.215  0.03  0.025  2.368  0  0.041  0  0.109  0  0.063  0  0  0  0.089  0.366  0  0  0  0.066  0  0  0.066  0  0  0  0.255  0  0  0  0  0.037  0  0  0  0.039  0  0  0  0.021  0  0  0  0  0.265  0  0.054  0  0  0  0  0  0  
 
 PLA2G2C  391013  1  20490483  20501687  1p36.12  0  0  0  0  0  0  0  0  0  0  0  0  0  0  0  0  0  0  0  0.347  0  0  0  0  0  0  0  0  0  0  0  0  0  0  0  0  0  0  0  0  0  0  0  0  0  0  0  0  0  0  0  0  0  0  0  0  0  0  0  0  
 
 UBXN10-AS1  101928017  1  20510735  20512979  1p36.12  0  0  0  0  0  0  0  0  0  0  0  0.793  0  0  0  0  0  0  0.661  0  1.05  1.286  0  0  0  0.328  0  0  0  0.087  0  0  0  0  0  0  0  0  0  0  0  0  0  0  0  0  0  0  0  0  0  0  0  1.238  0  0  0  0  0  0  
 
 UBXN10  127733  1  20512570  20522541  1p36.12  0  0  0.102  0  0.133  0.039  0.026  0  0.114  0.019  0  2.688  0.043  0.018  0  0.156  0  0.082  0.022  0.041  1.009  0.084  0.442  0  0  0.26  0.06  0.018  0  0.041  0.018  0  0  0.262  0.032  0.213  0.075  0.081  0.028  0  0  0.07  0.026  0.096  0.081  0.067  0.029  0  0.053  0  0.022  0.303  0.01  0.252  0  0  0.016  0  0  0  
 
 VWA5B1  127731  1  20617411  20681387  1p36.12  0  0.02  0.146  0  0.047  0  0.059  0.017  0.096  0.05  0.029  0.031  0  0.045  0  0.046  0.031  0.123  0.035  0.069  0  0.128  0.41  0  0  0  0.176  0.029  0.023  0.112  0.03  0  0  0.215  0.062  0.049  0  0  0.066  0  0  0.143  0.063  0.036  0.064  0.053  0.025  0  0  0  0.041  0.561  0.058  0.104  0.048  0.055  0  0  0  0.035  
 
 LINC01141  339505  1  20686293  20755287  1p36.12  0  0.054  0.099  0  0  0  0  0.019  0.115  0.045  0  0  0  0.044  0  0.022  0  0  0  0.041  0  0.057  0.241  0  0  0  0.066  0  0  0.07  0  0  0  0.237  0  0  0  0  0.033  0  0  0.021  0.054  0  0  0  0.034  0  0  0  0.025  0.223  0  0.083  0.045  0  0  0  0  0  
 
 CAMK2N1  55450  1  20808883  20812728  1p36.12  2.082  2.095  3.385  0.709  4.373  2.398  3.908  0.216  4.74  4.248  0.839  3.138  2.218  2.728  1.806  3.875  4.325  3.664  0.306  0.064  0.308  0.431  0.407  0.071  2.405  1.421  2.159  0.781  1.83  1.177  2.587  2.572  2.492  3.258  3.088  3.643  1.873  3.43  1.517  0.514  2.594  1.894  1.209  0.175  0.615  2.272  3.266  0.507  1.917  1.748  0.498  2.264  2.22  3.093  1.495  1.957  2.355  2.53  0.437  3.615  
 
 MUL1  79594  1  20825940  20834674  1p36.12  1.821  1.564  2.412  2.984  2.81  1.841  4.024  3.335  3.734  3.285  3.302  2.147  2.76  2.524  2.287  2.409  2.536  0.419  3.207  2.559  2.464  2.311  2.708  2.232  2.441  2.574  3.221  2.925  3.543  3.051  3.487  3.893  3.871  2.677  1.98  3.027  3.309  2.883  2.51  3.111  2.592  2.486  2.632  2.061  2.225  2.568  2.305  2.212  2.666  3.618  3.015  1.837  3.183  1.773  2.487  1.798  2.657  2.224  2.312  2.786  
 
 FAM43B  163933  1  20878931  20881513  1p36.12  0.069  0.077  0.203  0  0.118  0.12  0.243  0.183  0.258  0.182  0.087  0.182  0  0.112  0  0.114  0.161  0.183  0.033  0.248  0  0.534  0.54  0.064  0.09  0.107  0.242  0.101  0.09  0.267  0  0.065  0.155  0.572  0.39  0  0.106  1.246  0.21  1.082  0.126  0.8  0.632  0.184  0  0.129  0.086  0.355  0  0.909  0.248  1.448  0.067  0.33  0.271  0.151  0.404  1.442  0.062  0.055  
 
 CDA  978  1  20915443  20945400  1p36.2-p35  0  1.288  0.443  0  0.103  0.908  1.047  0.794  0.192  0  0.098  4.419  1.603  3.147  0.505  2.158  1.026  0.297  0.485  1.145  0  0.05  0.842  0.783  4.218  0  0.051  0.489  0  0.1  0  0.308  0  0.358  2.737  0.919  4.28  5.911  1.481  0.369  0.985  1.188  0  0  0.624  2.441  1.219  5.063  4.671  6.612  6.547  4.072  1.437  0.173  0.106  1.054  1.212  7.481  0.575  4.475  
 
 PINK1  65018  1  20959947  20978004  1p36  1.499  2.789  3.366  1.037  2.128  2.451  4.206  3.298  4.086  3.236  2.061  2.071  1.447  2.318  2.545  1.323  2.133  0.57  2.281  0.451  1.219  1.258  1.934  1.064  2.303  2.017  3.321  2.341  2.668  2.02  2.808  3.139  3.062  1.938  1.36  2.233  3.59  4.132  3.032  2.641  1.817  1.796  3.08  1.218  1.425  2.834  0.2  3.034  3.615  4.683  4.271  1.893  3.475  1.546  1.641  1.58  2.659  4.477  1.972  2.819  
 
 MIR6084  102464833  1  20960171  20960281  -  0  0  0  0  0  4.173  0  0  0  0  0  0  0  0  0  0  0  0  0  0  0  0  0  0  0  0  0  0  0  0  0  0  0  0  0  0  0  0  0  0  0  0  0  0  0  0  0  0  0  0  0  0  0  0  0  0  0  0  0  0  
 
 PINK1-AS  100861548  1  20969149  20978686  1p36.12  0.878  1.267  1.611  0.537  0.475  1.071  1.827  1.115  1.792  1.251  1.038  0.779  1.382  1.558  2.142  0.483  1.261  0.771  1.099  1.602  0.462  1.549  1.158  0.593  0.597  0.756  1.312  0.741  0.901  0.512  0.537  0.87  1.169  1.065  0.628  0.488  0.896  0.761  1.315  0.986  0.635  0.564  0.571  1.351  0.724  0.931  1.488  0.694  1.122  0.878  1.614  0.648  1.268  0.721  0.837  0.413  1.064  1.236  0.589  1.266  
 
 DDOST  1650  1  20978259  20988037  1p36.1  4.672  3.308  4.917  5.472  5.105  4.81  6.54  5.929  5.61  6.186  5.989  5.422  5.415  6.015  5.096  5.118  5.667  2.465  5.982  5.823  4.621  4.394  5.663  5.553  5.116  5.373  5.529  5.752  6.247  4.666  6.18  6.344  6.009  4.287  4.14  5.088  6.155  5.831  4.475  6.073  5.045  4.664  5.45  5.356  5.016  5.208  5.25  4.501  4.774  6.344  5.581  4.217  5.285  4.185  4.928  4.81  5.455  5.523  4.938  5.641  
 
 KIF17  57576  1  20990506  21044510  1p36.12  0.056  0.113  0.206  0.76  0.085  0.62  0.18  0.118  0.307  0.161  0.149  0.084  0.07  0.234  0.135  0.148  0.063  0.119  0.128  0.555  0  0.324  0.696  0.044  0.086  0.124  0.305  0.221  0.101  0.169  1.912  0.425  0  0.208  0.142  0.164  0.213  0.163  0.358  0.564  0.079  0.423  0.6  0.169  0.153  0.217  0.083  1.275  0.051  1.414  0.354  0.923  0.112  0.399  0.71  0.358  0.107  0.296  0.154  0.495  
 
 SH2D5  400745  1  21046224  21059133  1p36.12  0.291  1.121  0.688  1.838  0.114  1.104  0.527  0.383  0.386  0.143  0.249  0.063  0.68  1.125  0.087  0.107  0.051  0.125  0.134  0.102  0  0.266  0.605  0.173  1.133  0.521  0.787  1.175  0.742  0.455  0.447  0.763  1.028  0.668  0.609  0.385  1.869  1.258  2.402  0.462  0.322  0.219  1.831  0.956  0.177  0.546  1.487  1.458  0.087  2.573  1.495  1.806  1.429  0.824  2.39  1.044  0.833  1.542  2.151  2.363  
 
 HP1BP3  50809  1  21069170  21113181  1p36.12  3.311  4.465  2.971  3.386  3.66  3.303  3.723  3.818  3.879  4.608  4.727  2.934  3.062  4.436  3.372  4.08  3.99  5.078  5.759  3.491  3.241  4.57  3.514  2.953  2.934  3.471  3.502  2.832  3.877  2.169  4.448  3.419  3.785  3.139  3.12  3.762  3.826  3.781  3.713  4.148  3.427  2.905  4.329  3.502  2.827  3.794  2.782  3.241  3.751  4.917  4.246  2.584  3.315  2.359  3.747  2.898  4.158  3.699  3.276  3.89  
 
 EIF4G3  8672  1  21132784  21503381  1p36.12  2.739  3.01  2.216  2.076  2.538  2.553  3.539  3.988  2.174  3.888  3.62  2.266  3.644  2.876  4.07  3.07  2.388  2.572  3.12  2.846  2.595  2.715  1.279  2.319  3.087  2.708  3.526  3.431  4.159  2.499  3.689  3.403  3.561  1.506  2.493  3.141  3.758  3.186  2.791  3.983  3.317  1.825  4.305  2.65  2.813  2.928  1.8  3.447  3.386  2.978  3.566  1.694  3.105  1.818  3.015  2.438  3.465  3.618  2.355  3.318  
 
 ECE1  1889  1  21543739  21672034  1p36.1  2.226  2.054  4.203  3.522  2.657  3.099  4.735  3.056  4.279  4.768  4.904  3.445  2.942  3.219  2.792  3.886  2.509  0.433  3.38  0.67  2.113  1.681  1.989  2.305  2.633  2.906  2.382  3.105  3.814  2.054  3.072  2.964  3.029  1.541  2.18  3.862  4.149  4.856  2.553  4.617  3.86  4.035  2.494  3.989  3.435  3.521  2.241  3.188  3.872  4.222  4.604  2.884  4.513  3.633  4.09  3.526  4.307  3.482  4.378  4.847  
 
 LOC100506801  100506801  1  21619782  21626362  1p36.1  0.091  0  0.31  0  0.077  0.081  0.13  0.074  0.155  0.138  0.254  0.207  0.095  0.183  0.157  0.145  0.044  0  0.105  0.035  0  0.101  0.609  0.354  0  0.108  0.095  0.078  0.117  0.064  0.089  0.049  0.087  0.368  0.059  0.103  0.123  0.22  0.118  0.263  0.083  0.341  0.044  0.165  0.109  0.177  0.052  0.145  0.195  0.121  0.233  0.287  0.17  0.068  0.465  0.114  0.183  0.088  0.249  0.073  
 
 NBPF3  84224  1  21766582  21811393  1p36.12  1.452  0.853  1.151  1.143  1.18  1.261  1.251  0.941  0.736  1.324  1.292  0.815  0.873  1.106  0.406  0.576  0.92  0.465  2.003  0.26  0.411  0.378  0.745  0.434  0.42  0.547  1.007  0.485  1.168  1.21  0.99  1.132  0.903  0.614  0.625  0.643  0.76  1.307  0.34  1.606  0.657  0.334  1.891  0.532  0.789  0.963  0.296  0.871  0.775  1.768  3.525  0.671  0.475  0.259  0.525  0.466  0.524  0.863  0.609  0.736  
 
 ALPL  249  1  21835857  21904905  1p36.12  0.214  0.054  3.717  0  0.13  0.231  0.603  1.158  0.166  1.472  0.057  0.067  0  0.134  0  0.077  0.139  0.104  0  0.075  0  0.158  0.491  0  0  0  0.133  0.03  0  0.15  0.058  0.094  0  0.284  0.067  0.078  0  0  1.324  0.724  0.929  0.372  0.476  0.44  0.805  0.897  0.029  0.634  0.094  1.792  0.129  0.671  0  0.131  0.094  0.147  0.143  0  0  0  
 
 RAP1GAP  5909  1  21922707  21995856  1p36.1-p35  1.684  0.029  0.324  0.169  0.669  0.196  0.408  0.188  0.478  0.835  0.098  1.319  2.795  1.41  0.582  2.304  0.804  0.138  2.931  0.161  2.427  0.299  0.367  0.098  0  2.43  0.755  0.22  0.939  1.431  4.292  1.712  0.671  0.253  2.118  1.113  0.28  0.194  0.319  1.27  2.43  0.802  0.872  1.083  1.109  1.649  0.871  0.444  0.551  1.327  1.011  0.871  0.302  0.801  1.263  0.138  0.727  0.061  0.573  0.073  
 
 USP48  84196  1  22004791  22109688  1p36.12  1.888  3.363  1.672  1.597  1.994  1.495  2.238  2.76  1.782  2.922  3.386  1.52  2.354  3.177  2.467  2.266  2.499  3.773  3.326  2.704  2.999  2.496  1.957  1.667  2.125  2.54  2.992  2.68  3.425  2.361  3.531  3.072  2.924  1.959  1.25  2.307  2.261  2.077  2.136  2.359  1.99  1.64  3.062  2.442  1.939  2.297  1.376  1.6  2.484  2.876  2.856  1.409  2.122  1.301  2.183  1.89  2.761  1.688  1.891  2.498  
 
 LDLRAD2  401944  1  22138757  22151714  1p36.12  0.35  0.14  2.989  1.107  0.744  0.306  0.782  1.299  1.397  3.239  0.358  0.393  0.603  0.301  0.59  2.72  1.234  0.079  0.053  0.875  0.245  0.252  0.613  0.26  0.243  0.891  2.696  1.146  1.534  0.358  1.167  0.94  0.753  1.507  1.264  1.084  1.431  0.718  2.462  0.156  0.668  2.992  1.148  0.53  0.468  1.443  4.077  0.472  0.953  1.521  1.062  0.834  2.083  0.726  2.528  1.244  1.156  0.222  1.383  1.091  
 
 HSPG2  3339  1  22148724  22263790  1p36.1-p34  1.67  0.297  3.539  0.529  1.366  1.562  2.123  5.011  0.785  5.82  1.763  1.533  3.127  1.584  3.007  3.808  1.569  0.141  0.03  0.343  2.922  0.249  0.364  0.677  2.004  3.334  4.765  4.961  4.206  1.266  3.437  3.61  4.041  1.24  2.238  4.375  5.004  3.728  1.922  1.983  3.66  2.005  2.203  2.849  2.951  4.442  2.666  2.766  3.917  0.676  3.901  2.745  5.017  2.298  3.615  3.846  4.036  3.017  4.334  4.55  
 
 CELA3B  23436  1  22303417  22315847  1p36.12  0  0  0  0  0  0  0  0  0  0  0  0  0  0  0  0  0.172  0.298  0  0  0  0.216  0  0  0  0  0.074  0  0  0.088  0  0  0  0  0  0  0  0  0  0  0  0.366  0  0  0  0  0  0  0  0  0  0.433  0  0.113  0  0  0  0  0  0  
 
 CELA3A  10136  1  22328148  22339035  1p36.12  0  0  0  0  0  0  0  0  0  0  0  0  0  0  0  0  0.31  0  0  0  0  2.464  0  0  0  0  0.07  0  0  0.09  0  0  0  0  0  0  0  0  0.067  0  0  0.081  0  0.149  0  0  0  0  0  0  0  0.227  0  0  0  0  0  0  0  0  
 
 LOC101928043  101928043  1  22350486  22352541  -  0  0  0  0  0.147  0.491  0  0  0  0  0.181  0  0  0.088  0  0  1.103  0.332  0.337  0  0  0.213  0.935  0.391  0.265  0.208  0.16  0.191  0.223  0.079  0.508  0.228  0  0.455  0  0  0.147  0  0  0  0  0  0  0  0  0  0  0  0  0  0.156  0.243  0.148  0.146  0  0  0  0.362  0  0  
 
 LINC00339  29092  1  22351683  22357717  1p36.12  1.703  1.646  1.839  2.117  2.001  1.798  1.396  1.893  1.979  2.73  2.001  0.683  1.514  3.15  1.429  1.534  2.514  2.162  4.354  0.867  1.454  2.132  3.693  1.76  1.526  2.037  2.736  1.908  2.457  0.616  3.68  3.299  2.009  1.773  1.653  1.675  2.844  1.372  0.599  2.007  1.858  1.357  4.084  1.893  1.112  0.888  0.413  0.782  2.269  2.991  2.305  1.575  1.32  0.85  1.786  1.718  1.834  1.976  1.138  1.713  
 
 CDC42  998  1  22379119  22419436  1p36.1  3.422  4.769  4.332  4.2  4.151  4.819  5.018  5.2  4.397  4.893  5.826  4.335  4.66  5.231  3.796  4.675  4.908  4.365  5.555  4.273  4.438  3.596  4.07  3.477  4.994  3.757  3.965  3.722  4.389  2.375  4.741  4.657  3.935  2.586  3.484  4.244  4.64  4.359  4.005  4.586  4.434  3.847  4.882  5.046  3.931  3.886  3.345  3.46  5.182  5.242  5.168  2.72  4.77  2.971  3.715  4.404  5.067  4.73  4.524  4.768  
 
 WNT4  54361  1  22443797  22469519  1p36.23-p35.1  0.618  0.045  0.593  0.247  0.657  0.101  0.14  0.381  0.205  0.526  0.326  0.033  0.16  0.306  0.584  0.328  0.275  0.079  0.133  0.274  0.148  0.233  0.703  0.11  0.075  0.212  0.35  0.217  0.276  0.34  0.197  0.192  0.18  0.395  0.152  1.642  0.561  0.469  0.522  0.417  0.818  0.518  0.055  0.196  0.228  0.301  0.088  0.28  0.394  0.189  0.24  0.555  0.338  0.153  0.279  0.11  0.48  0.21  0.289  0.222  
 
 ZBTB40  9923  1  22778343  22857650  1p36  1.802  0.83  0.896  0.958  2.021  0.751  1.631  1.815  0.995  1.547  1.787  0.975  1.891  1.598  1.607  1.026  1.044  0.32  1.955  1.328  1.947  1.652  0.888  1.185  1.098  1.3  1.515  1.952  1.94  1.115  1.842  1.753  1.64  0.645  0.777  1.502  1.886  1.926  0.775  1.985  1.204  0.94  2.558  1.399  1.044  1.123  0.533  1.512  1.285  1.824  1.564  0.68  1.362  0.698  1.349  1.023  2.046  0.975  1.18  1.423  
 
 EPHA8  2046  1  22890003  22930087  1p36.12  0.083  0  0.215  0  0.772  0.149  0.054  0.066  0.185  0.101  0.059  0.268  0  0.151  0  0.052  0.104  0.177  0.041  0.132  0  0.326  0.721  0.074  0.068  0.299  0.175  0.073  0.054  0.558  0  0.287  0  0.329  0.113  0  0  0.076  0.131  0.219  0.055  1.191  0.158  0.133  0.104  0.223  0.091  0  0  0.029  0.036  1.145  0.056  0.187  0.114  0.115  0.04  0  0  0  
 
 C1QA  712  1  22963117  22966175  1p36.12  0  0  0  0  0  0.134  0  0  0.142  0  0  0  0  0.052  0  0  0  0  0  0.238  0  0.231  0.987  0.206  0  0  0.098  0  0  0.071  0  0  0  0.24  0  0  0  0  0.076  0  0  0.757  0.052  0  0  0  0  0  0  0  0  0.706  0  0.15  0.105  0  0  0  0  0  
 
 C1QC  714  1  22970117  22974603  1p36.11  0  0.118  0.183  0  0  0  0  0  0.185  0  0  0  0  0.069  0  0  0  0.117  0  0  0  0.135  0.589  0  0  0  0.118  0  0  0.106  0  0  0  0.33  0  0  0  0  0.073  0  0  0.067  0.072  0  0  0.086  0  0  0  0  0  0.55  0  0.146  0  0  0  0  0  0  
 
 C1QB  713  1  22979681  22988029  1p36.12  0  0  0  0  0.082  0  0  0  0.208  0  0  0.149  0  0  0  0  0  0.123  0  0.166  0  0.193  0.535  0  0  0  0.079  0  0  0.099  0  0  0  0.268  0.116  0  0  0  0.051  0  0  0.423  0.093  0  0  0  0  0  0  0  0  0.91  0  0.181  0  0.169  0  0  0  0  
 
 EPHB2  2048  1  23037330  23247993  1p36.1-p35  0.881  0.25  0.949  0.064  0.092  0.48  3.895  3.44  0.958  2.364  2.598  5.114  2.813  1.143  1.33  0.669  0.659  0.293  0.04  0.055  0  0.213  0.804  0.528  1.511  0.021  0.192  0.063  0.037  0.157  0.042  0.303  0.034  0.32  1.552  3.01  3.239  2.011  1.804  0.759  2.504  0.718  1.294  0.298  1.312  0.679  1.192  1.67  2.134  1.999  1.675  0.626  2.401  1.477  2.636  2.22  2.487  0.737  2.661  4.294  
 
 LACTBL1  646262  1  23279535  23291923  1p36.12  0  0.098  0  0  0.141  0.241  0.131  0.121  0.136  0.154  0.061  0.177  0  0.129  0  0.187  0.135  0.242  0.067  0.173  0  0.743  0.417  0.072  0  0  0.178  0.042  0  0.152  0  0.134  0  0.416  0.144  0.097  0  0  0.267  0  0.079  0.887  0.108  0.092  0.134  0.064  0.078  0  0  0  0.09  1.985  0.072  0.285  0.278  0.111  0.068  0.148  0  0.083  
 
 C1orf234  729059  1  23337326  23342343  1p36.12  0  0  0  0  0  0  0  0  0  0.16  0  0  0  0  0  0  0  0  0  0  0  0.175  0  0  0  0  0.141  0  0  0.135  0  0  0  0  0  0  0  0  0  0  0  0  0  0  0  0  0.128  0  0  0.199  0  0  0  0  0  0  0  0  0  0  
 
 KDM1A  23028  1  23345940  23410184  1p36.12  3.572  3.307  3.049  3.8  3.159  2.869  4.243  3.844  3.203  3.837  4.743  3.04  3.162  4.755  3.937  4.302  4.245  3.386  5.968  4.186  3.894  4.987  2.792  2.647  3.299  2.902  3.624  3.007  4.026  2.5  4.942  4.143  3.468  2.228  2.692  3.271  4.151  3.422  2.815  4.195  3.056  2.845  5.663  3.909  3.271  3.164  3.747  3.269  4.124  5.268  4.497  2.501  3.662  2.004  3.342  3.263  3.666  3.294  3.01  3.967  
 
 LUZP1  7798  1  23410515  23495351  1p36  1.002  1.577  2.232  1.717  1.504  1.375  2.605  3.371  1.344  2.584  2.678  1.432  2.29  2.188  1.992  1.793  2.034  0.741  1.882  1.583  1.402  1.45  0.706  0.732  2.486  1.972  1.927  2.247  2.827  1.705  2.746  2.449  2.73  1.565  1.287  2.202  3.034  2.176  2.672  2.484  1.635  1.019  3.226  2.634  2.236  1.863  1.723  2.056  2.643  2.269  2.694  1.681  2.542  1.69  2.167  1.925  2.959  1.702  2.174  3.042  
 
 HTR1D  3352  1  23518387  23521222  1p36.3-p34.3  0.471  0.426  0.301  0.44  0.368  0.267  0.358  0.608  0.217  0.129  1.039  2.196  1.583  1.514  0.636  0.41  1.421  0.067  0  0.119  0  0.112  0.479  0.172  0.123  0  0.343  0.126  0.18  0.243  0  0  0  0.518  2.02  1.257  2.511  0.304  2.573  0.687  0.651  0.217  0.344  0.334  0.18  0.046  0.775  0.083  1.309  0.511  0.246  0.916  0.091  0.246  0.689  1.276  0.266  1.199  0.06  2.291  
 
 LOC100996511  100996511  1  23607801  23612011  -  0.319  0.648  0.271  0.206  0.626  0.29  0.337  0.474  0.421  1.005  0.82  0.181  0.356  0.962  0.758  0.977  0.232  1.878  2.174  0.806  0.316  0.501  0.549  0.228  0.245  0.417  0.327  0.197  0.647  0.127  1.061  0.504  0.175  0.261  0.352  0.484  0.771  0.705  0.482  0.163  0.536  0.286  0.907  0.456  0.154  0.148  0.448  0.578  0.563  1.592  0.548  0.206  0.484  0.114  0.74  0.285  0.799  0.374  0.665  0.252  
 
 HNRNPR  10236  1  23631180  23670857  1p36.12  3.36  4.427  2.413  2.662  3.245  2.598  3.647  3.441  2.826  3.384  4.316  2.885  3.585  4.271  3.407  3.636  3.526  4.736  5.236  3.557  3.528  3.755  2.756  2.724  3.348  2.68  3.231  2.863  3.356  2.072  3.205  3.408  3.759  2.568  2.712  3.318  3.476  3.062  3.021  3.257  3.208  2.789  3.94  3.24  2.656  2.632  1.757  2.826  3.244  4.163  3.37  2.328  3.361  2.115  3.218  2.994  3.326  3.473  2.873  3.725  
 
 ZNF436  80818  1  23685940  23696357  1p36  1.101  0.739  0.814  1.245  1.032  0.205  1.356  1.557  3.232  2.482  2.834  0.478  1.273  1.359  1.153  1.333  1.071  1.015  1.38  0.869  0.613  0.937  0.973  0.32  0.511  0.826  1.134  0.721  1.049  0.229  1.189  1.218  1.036  0.729  0.419  0.866  1.121  1.237  0.808  1.802  1.367  0.786  2.262  0.526  1.535  0.75  1.117  0.607  1.182  1.408  1.437  0.53  1.158  0.694  0.906  0.859  1.922  0.763  0.609  1.232  
 
 ZNF436-AS1  148898  1  23695463  23698330  1p36.12  0.376  1.093  0.318  0.569  0.75  0.581  0.503  0.613  0.688  1.631  1.188  0.232  0.462  1.387  0.492  1.267  1.007  1.717  1.38  1.34  0.593  0.439  1.036  1.249  0.191  0.213  1.05  0.379  0.785  0.117  0.674  0.53  0.778  1.001  0.567  0.63  0.417  0.9  1.474  0.265  0.621  1.779  1.444  0.296  0.191  0.89  1.818  0.247  0.919  0.588  0.602  0.743  1.033  0.902  1.539  0.749  0.726  0.769  0.427  0.569  
 
 TCEA3  6920  1  23707401  23751272  1p36.12  3.096  1.148  0.302  0.312  3.519  0.147  0.509  0.749  0.358  0.842  0.339  0.341  0.233  1.195  0.259  1.213  0.483  1.515  0.735  0.58  0  0.179  0.442  0.063  0.088  0.949  0.104  0.147  0.1  0.107  0  0.188  0  0.2  0.072  0.659  0.887  2.728  0.196  1.89  0.291  0.189  0.844  0  1.524  0.356  0.036  0.907  0  2.075  1.493  0.287  0.202  0.509  0.226  0.518  2.813  0.249  2.472  1.626  
 
 ASAP3  55616  1  23755055  23810750  1p36.12  1.347  0.386  0.891  1.576  2.485  0.353  1.398  2.476  1.429  2.878  1.43  0.045  0.599  1.278  1.046  0.22  1.079  0.117  0.075  0.108  1.168  0.227  0.372  0.037  0.379  0.905  1.189  1.024  1.053  0.458  1.313  1.425  0.805  0.593  0.447  1.029  1.995  1.377  0.685  1.303  0.78  1.271  2.495  0.702  1.656  0.956  0.351  1.147  1.02  2.184  1.604  0.919  0.887  0.88  0.493  0.526  1.927  1.096  0.676  0.983  
 
 E2F2  1870  1  23832919  23857712  1p36  1.96  0.259  0.663  1.343  1.535  0.516  1.115  0.757  0.452  1.422  1.716  1.453  1.795  2.09  1.767  0.875  1.372  0.159  4.582  1.506  1.515  2.289  2.36  2.412  0.709  1.339  1.333  1.683  1.799  1.353  1.852  1.082  2.346  1.092  1.015  1.583  1.137  0.532  0.33  2.76  1.13  1.665  2.674  1.5  1.44  1.09  0.979  0.817  1.118  1.497  0.655  1.166  0.855  0.543  0.855  0.576  0.868  1.113  1.744  0.706  
 
 LOC101928163  101928163  1  23848421  23854911  -  0.371  0  0.286  0  0.141  0.153  0.085  0  0  0.131  0.149  0.121  0.165  0.168  0.252  0.092  0  0  0.288  0.132  0  0.246  0.589  0.312  0  0  0.152  0.186  0.172  0.092  0.169  0  0.218  0  0.189  0.135  0.124  0  0  0.268  0  0.136  0.307  0.174  0  0  0  0.115  0.258  0  0.07  0.354  0  0.185  0.063  0  0.112  0  0.168  0  
 
 ID3  3399  1  23884420  23886285  1p36.13-p36.12  4.168  3.558  5.009  5.714  5.068  4.429  7.115  7.051  3.99  6.703  6.29  1.644  5.486  5.953  3.812  0.336  5.286  0.709  5.406  0.58  1.648  4.627  3.603  2.113  6.114  2.5  4.611  3.573  5.512  1.919  4.604  4.562  3.723  1.164  5.938  6.424  4.231  2.099  6.325  6.811  3.808  5.704  8.634  3.042  4.633  4.054  3.335  2.932  2.817  3.873  2.707  4.346  5.02  4.873  2.465  3.843  3.627  2.87  5.113  3.498  
 
 MDS2  259283  1  23953823  23967056  1p36  0  0  0  0  0  0  0.112  0  0  0  0  0  0  0.266  0  0  0.15  0  0  0.135  0  0.087  0.43  0.112  0  0  0.182  0  0  0.086  0  0  0  0  0  0  0  0  0.071  0  0  0  0.174  0  0.162  0  0.087  0  0  0  0  0  0.078  0.072  0  0  0  0  0  0  
 
 RPL11  6135  1  24018268  24022915  1p36.1-p35  8.512  10.539  6.991  6.876  9.016  8.118  9.466  8.671  7.092  9.045  9.284  8.395  8.319  9.889  7.71  9.104  9.366  10.408  10.832  10.667  9.037  9.488  8.411  9.128  8.934  7.188  8.89  7.712  8.918  7.585  9.558  9.221  8.837  6.811  8.183  7.854  8.742  8.567  8.697  8.429  8.217  7.615  9.91  8.954  8.23  8.385  9.313  8.073  7.977  10.477  7.984  6.989  8.63  6.989  9.144  8.146  8.313  9.452  8.412  8.959  
 
 TCEB3  6924  1  24069855  24088549  1p36.1  2.394  2.424  2.217  2.561  2.572  1.76  3.588  3.501  2.716  3.253  3.832  1.83  3.206  2.777  2.844  2.843  1.945  1.711  3.554  2.238  2.981  2.321  2.381  2.017  3.401  2.7  2.892  3.279  3.72  2.156  3.792  3.968  4.013  2.49  1.544  3.226  3.465  2.971  2.782  3.609  2.677  1.985  3.891  3.102  2.83  2.575  1.624  2.371  2.648  3.302  3.416  1.936  3.068  1.543  2.247  2.178  2.862  2.426  2.416  2.94  
 
 TCEB3-AS1  100506963  1  24086871  24104787  -  0.767  0.111  0  0.914  0.83  0  1.042  0.528  0.75  0.785  0.476  0.384  0.502  1.296  0.235  0.263  1.463  0  1.503  0  1.205  1.388  2.426  0.724  0.272  0.596  0.831  0.496  0.778  0.218  1.082  0.935  0.835  0.318  0.332  0.27  0.373  0.876  0.13  0.47  0.529  0.346  1.294  0.272  0  0.466  0.053  0  0  1.086  1.227  0.32  0.835  0.323  1.197  0  0.462  0  0  0.681  
 
 PITHD1  57095  1  24104875  24114722  1p36.11  3.309  3.781  2.558  3.273  3.593  2.498  4.871  3.41  3.792  3.771  4.758  3.036  3.348  4.441  2.843  3.756  3.856  3.402  5.337  4.851  4.277  4.264  4.265  2.843  2.722  2.901  3.285  2.684  3.536  2.251  3.584  3.454  3.378  2.288  2.044  2.311  3.104  2.841  3.979  3.027  2.786  3.059  4.645  2.878  2.72  3.021  4.056  2  3.329  4.497  4.149  2.033  3.476  2.014  3.143  2.771  3.634  3.422  2.902  3.109  
 
 LYPLA2  11313  1  24117645  24122029  1p36.11  2.768  0.618  2.645  4.027  3.856  2.767  4.444  4.256  3.619  4.13  4.562  4.347  4.742  3.689  3.757  4.769  4.179  0.3  5.206  2.768  4.263  3.495  3.57  3.849  4.475  3.5  4.174  4.729  4.867  3.487  5.105  5.09  5.774  4.088  2.642  4.204  4.519  4.14  2.803  4.56  4.323  3.869  4.704  3.796  4.406  4.657  2.722  3.852  3.322  5.579  3.906  2.975  3.537  2.907  3.826  3.527  3.753  3.886  4.242  4.544  
 
 GALE  2582  1  24122088  24127294  1p36-p35  3.111  2.268  1.963  2.433  3.495  2.22  5.005  3.92  2.324  3.242  4.794  3.84  4.908  5.077  3.416  4.367  4.711  1.517  4.129  3.03  3.869  2.986  2.198  2.165  3.489  3.864  4.206  4.176  5.234  3.775  6.292  5.005  4.954  2.984  3.295  4.026  4.998  4.905  3.085  4.259  4.645  3.465  4.815  3.095  1.617  3.897  4.74  3.055  3.534  5.531  4.286  2.709  4.322  2.641  5.13  4.26  3.961  4.395  3.97  4.403  
 
 HMGCL  3155  1  24128366  24151949  1p36.1-p35  2.107  1.169  2.856  3.251  3.027  1.752  4.215  2.894  3.195  3.462  3.264  3.03  3.221  3.016  2.738  3.368  4.218  1.407  3.496  1.908  1.968  2.069  3.171  1.161  1.239  2.587  3.265  1.881  3.221  2.511  3.25  2.587  1.292  1.482  0.954  2.479  3.54  2.627  2.484  2.928  2.887  2.506  1.534  2.127  1.43  2.313  3.009  1.794  2.296  3.894  3.202  1.479  2.673  1.176  3.605  2.269  2.55  1.701  2.123  3.4  
 
 FUCA1  2517  1  24171571  24194859  1p34  2.17  2.991  0.591  1.016  2.312  1.135  3.196  1.714  2.902  2.724  2.283  1.476  2.917  3.355  1.837  3.91  2.299  2.808  1.41  2.983  1.23  1.443  2.32  1.305  0.09  2.719  2.619  1.503  3.99  1.394  3.109  1.791  1.696  1.404  0.837  2.91  1.883  3.28  2.192  2.215  3.081  1.428  1.538  1.684  0.975  2.527  3.086  1.134  0.987  2.781  2.417  1.193  1.603  1.505  2.399  1.285  2.437  0.66  2.53  2.935  
 
 CNR2  1269  1  24200459  24239817  1p36.11  0.152  0  0.296  0  0.045  0.169  0.278  0.083  0.208  0.11  0.044  0  0  0.057  0.099  0  0  0.14  0.261  0.413  0  0.165  0.768  0  0  0  0.155  0  0  0.086  0  0  0  0.383  0  0  0  0  0.062  0  0.122  0.129  0.06  0  0  0.136  0.155  0.165  0  0.117  0.117  0.348  0.065  0.082  0.054  0.207  0.054  0  0  0  
 
 PNRC2  55629  1  24286300  24289949  1p36.11  3.78  5.107  2.961  3.713  3.243  3.031  3.284  3.317  3.004  3.728  3.975  3.146  3.908  4.767  3.874  4.297  4.128  6.229  5.148  4.031  4.102  3.721  3.505  2.997  3.902  2.918  3.006  2.881  3.457  1.801  4.167  3.74  3.45  2.767  1.971  3.64  3.829  3.641  3.323  4.086  2.807  2.628  4.938  4.064  2.296  3.321  3.195  2.832  3.136  4.873  3.652  1.964  3.209  1.879  3.181  2.634  3.816  2.817  3.473  3.888  
 
 SRSF10  10772  1  24290836  24306953  1p36.11  1.859  3.485  1.116  1.543  2.056  1.522  2.671  1.866  1.495  2.034  2.577  1.659  2.172  2.986  1.898  2.258  2.007  3.683  3.693  2.743  2.086  2.731  1.493  1.586  1.701  1.531  1.609  1.412  1.853  1.161  2.45  2.321  1.874  0.927  1.337  2.007  2.21  1.566  1.646  2.274  1.735  1.485  2.497  1.878  1.36  1.726  2.083  1.756  1.64  2.893  1.806  1.042  1.901  1.097  1.703  1.486  2.139  1.985  1.888  2.248  
 
 MYOM3  127294  1  24382530  24438665  1p36.11  0.056  0.02  0.112  0  0.04  0.034  0.116  0.123  0.138  0.091  0.159  0.922  2.291  0.574  0.068  0.755  0.042  0.075  0.061  0.093  0  0.163  0.368  0  0.057  0.062  0.115  0.036  0.033  0.09  0.035  0.029  0.05  0.257  0.42  0.128  0.226  0.608  0.114  0.416  0.034  0.489  0.041  0  0  0.064  0.127  0.371  0.115  0.898  0.303  0.421  0.181  0.796  1.714  0.084  1.071  0  1.228  1.438  
 
 IL22RA1  58985  1  24446260  24469775  1p36.11  0.087  0.132  0.124  0.243  0.296  0.393  0.661  0.439  0.074  0.223  0.592  2.456  2.443  0.976  1.395  2.536  2.442  0.135  0.043  0.081  0  0.261  0.461  0.092  0.173  0.878  0.119  0.268  0  0.241  0.056  0.177  0.526  0.415  0.54  0.553  0.762  0.607  0.226  1.891  0.036  0.663  0.047  0  0.38  0.835  0.915  0.242  0.087  0.645  0.47  0.557  0.151  0.747  0.635  0.3  0.482  0.746  0.832  0.509  
 
 IFNLR1  163702  1  24480646  24513765  1p36.11  0.222  0.135  0.171  0  0.489  0.06  0.092  0.048  0.329  0.027  0.659  0.406  1.014  1.047  1.388  0.407  0.773  0.165  0.044  0.055  0  0.132  1.17  0.486  0  0.685  0.172  0.029  0  0.057  0  0  0  0.247  0.133  0.606  1.004  0.255  0.091  0.833  0.778  0.4  0.208  0.393  0.342  0.695  0.387  0.257  0  0.697  0.99  0.422  0.172  0.088  0.547  0.274  0.94  0.106  0.627  0.374  
 
 LOC284632  284632  1  24526729  24538180  1p36.11  0  0  0.199  0  0  0  0  0.045  0.209  0  0  0  0  0  0  0  0  0  0  0  0  0.08  0.464  0  0  0  0.059  0  0  0.234  0  0  0  0.3  0  0  0  0  0.044  0  0  0.087  0.028  0  0  0  0.026  0  0  0  0  0.142  0  0.054  0.045  0  0  0  0  0  
 
 GRHL3  57822  1  24645811  24690970  1p36.11  0.277  0.095  0.191  0  1.409  0.113  0.16  0.044  0.165  0.072  0.206  0.061  0.598  0.081  1.4  0.303  0.332  0.133  0  0.066  0  0.089  0.446  0.141  0.064  0  0.169  0.07  0  0.116  0  0.083  0  0.184  0.063  0.095  0.109  0.076  0.163  4.252  3.273  0.927  1.66  0.668  0.559  0.699  0.085  0  0  0  0.125  0.502  0.049  0.159  0.08  0  0.109  0  0  0.086  
 
 STPG1  90529  1  24683488  24741587  1p36.11  0.673  0.877  1.297  0.407  1.294  0.971  0.747  1.046  0.818  0.608  1.542  1.069  1.442  1.259  1.063  0.862  1.234  0.244  0.509  0.741  0.59  0.103  1.029  0.141  1.01  1.039  2.407  0.828  1.414  0.902  1.596  1.551  1.461  0.909  0.673  1.138  1.937  1.195  1.169  0.931  1.26  1.161  1.698  0.655  0.824  1.216  0.999  1.175  1.509  2.013  2.449  0.532  1.207  1.007  0.723  0.66  1.334  0.759  0.958  0.42  
 
 NIPAL3  57185  1  24742244  24799473  1p36.12-p35.1  1.015  1.092  1.886  0.279  2.469  1.097  2.801  1.789  1.854  2.62  2.434  1.378  1.958  1.175  2.006  1.649  1.782  0.513  1.813  1.319  1.204  1.068  2.154  1.425  1.644  1.401  3.253  2.038  2.817  0.89  1.74  2.797  3.525  2.523  0.509  0.792  1.672  1.856  2.161  1.079  1.527  1.313  1.33  1.122  1.499  2.394  0.566  1.79  2.173  2.438  3.291  0.968  0.73  1.459  0.383  0.557  2.215  1.655  1.009  0.669  
 
 RCAN3AS  100750325  1  24822822  24828850  1p36.11  0  0  0  0  0.804  0  0.549  0  0  0.332  0.416  0  0  0  0  0.316  0.775  0  0.857  0  0  0.317  0.885  0  0  0  0.623  0  0  0  0  0.664  0  0.752  0  0  0  0  0.288  0  0  0.368  1.622  0  0  0  0  0  0  0.562  0  0  0  0  0.326  0  0  0  0  0  
 
 RCAN3  11123  1  24828840  24863510  1p35.3-p33  1.328  2.306  1.297  0  2.101  1.689  1.916  1.563  1.155  2.389  2.956  1.524  1.769  2.401  1.703  2.47  2.465  1.477  2.038  0.952  0.181  2.079  1.598  0.262  1.395  1.438  2.855  2.091  2.97  1.308  2.228  2.772  2.418  1.612  1.542  1.353  1.915  2.188  0.998  1.996  1.325  1.875  2.314  0.684  1.541  1.693  1.361  1.52  1.589  1.963  2.827  1.295  1.678  1.101  0.743  1.017  2.492  1.32  1.038  0.191  
 
 LOC100506985  100506985  1  24865763  24882515  -  0.231  0.376  0.38  0  0.185  0.384  0.827  0.47  0.825  1.315  1.385  0.773  0.659  1.129  0.428  1.009  0.908  0  1.472  0  0  0.304  0.783  0.12  0.651  0.716  1.942  0.666  1.193  1.236  1.405  1.37  0.894  0.648  0.218  0.653  0.796  0.82  0.192  0.554  0.448  0.661  1.044  0  0.383  0.494  0  0.667  0  1.212  1.695  0.454  0.334  0.162  0.088  0.211  1.159  0.274  0.164  0  
 
 NCMAP  400746  1  24882566  24935818  1p36.11  0.186  0.052  0.319  0.088  0.219  0.094  0.12  0.021  0.105  0.903  0.151  0.089  0.198  0.363  0.257  0.608  0.525  0.104  0.074  0.21  0  0.663  0.62  0.145  0.06  0.039  0.118  0.121  0.3  0.11  0.049  0.066  0  0.256  0.179  0.333  0.125  0  0.323  0.08  0.056  0.067  0.115  0.041  0.084  0.124  0.928  0.058  0.046  0.058  0.072  0.154  0.212  0.169  0.354  0.126  0.184  0.041  0.155  0.05  
 
 SRRM1  10250  1  24969593  24999772  1p36.11  3.439  4.326  2.797  3.798  4.02  3.819  4.023  4.154  3.136  4.373  5.049  3.371  3.949  4.935  4.985  4.586  4.6  4.125  4.834  4.221  4.782  5.019  2.908  3.767  3.987  3.747  3.53  3.802  4.277  2.639  4.349  4.467  3.905  2.379  3.454  4.474  4.531  3.628  3.589  4.781  3.931  3.474  4.856  4.92  3.746  4.64  2.341  4.076  4.666  3.988  4.308  3.377  4.37  3.064  4.2  4.048  4.533  4.236  4.431  4.64  
 
 CLIC4  25932  1  25071759  25170815  1p36.11  1.444  4.265  4.906  3.532  2.162  4.026  3.814  4.749  4.252  5.131  4.681  1.423  0.261  3.099  2.284  2.117  2.91  2.559  3.176  1.913  2.189  1.841  2.93  1.663  3.165  3.337  3.027  2.94  3.443  1.688  3.247  3.801  2.742  2.573  1.439  3.594  4.23  3.212  5.216  2.233  1.712  2.41  3.218  3.101  3.027  3.345  1.655  4.52  3.445  6.135  4.655  1.805  3.989  3.048  2.282  4.34  4.468  6.318  4.279  4.252  
 
 RUNX3  864  1  25226001  25291501  1p36  0.086  0.122  0.223  0.159  0.07  0.158  0.125  2.582  0.182  0.227  1.129  1.378  0.839  2.686  0.043  0.08  0.101  1.742  2.819  4.122  0.11  0.947  3.667  3.209  1.928  4.296  5.078  3.617  5.298  4.02  4.739  1.578  0  0.24  1.167  3.102  2.895  2.085  0.124  2.44  1.916  0.164  2.779  0.076  1.204  0.761  0.091  2.047  0.598  2.163  0.103  1.898  0.046  0.178  0.195  0.091  0.205  2.141  0  0.032  
 
 SYF2  25949  1  25548766  25559013  1p36.11  2.336  4.414  1.791  2.488  2.715  2.073  3.089  2.298  2.744  2.991  3.109  2.583  2.196  3.914  1.878  2.903  3.622  5.915  4.38  4.305  2.113  3.504  3.349  1.948  3.128  2.15  2.591  1.683  2.029  1.948  3.099  3.005  2.057  2.022  1.768  1.895  1.802  2.202  3.713  1.931  2.011  2.516  2.294  2.62  1.749  2.071  3.211  1.469  1.798  3.739  2.29  1.402  1.442  1.602  3.13  1.961  2.515  2.636  1.911  2.496  
 
 RSRP1  57035  1  25568739  25573985  1p36.13-p35.1  2.643  3.039  1.46  2.178  2.027  1.651  2.655  2.526  2.654  3.472  2.631  1.865  1.836  3.23  2.503  2.731  2.36  5.876  4.541  4.358  2.52  2.737  2.917  2.218  1.609  1.893  2.745  1.764  2.008  0.819  2.45  2.163  2.031  1.743  1.306  1.78  2.246  2.742  3.011  1.755  1.19  2.551  2.422  1.652  1.265  2.358  2.757  2.109  2.2  3.781  2.069  1.502  2.09  1.42  2.739  2.121  2.713  1.554  2.029  1.611  
 
 RHD  6007  1  25598976  25656936  1p36.11  0  0  0.202  0.357  0.072  0.186  0.123  0.191  0.293  0.315  0.184  0.166  0.102  0.157  0.234  0.119  0.143  0.124  0.206  0.101  1.032  0.169  0.485  0  0  0.058  0.349  0.105  0.096  0.331  0  0  0.093  0.424  0.061  0.077  0.285  0.224  0.132  0.151  0  0.57  0.052  0  0.124  0.11  0.36  0  0  0.068  0.11  0.238  0.115  0.069  0.235  0  0.158  0.177  0.195  0.07  
 
 TMEM50A  23585  1  25664788  25688852  1p36.11  2.696  4.733  3.444  3.97  3.399  3.224  4.521  4.375  3.633  4.285  3.976  3.285  3.265  3.946  2.77  3.889  3.576  4.72  5.037  3.762  3.195  3.156  3.911  3.1  4.261  3.123  3.133  2.793  3.084  1.881  3.937  3.755  2.501  1.906  2.518  3.375  3.264  3.66  4.123  3.471  2.97  2.502  3.637  2.81  2.204  3.007  3.343  3.036  2.761  5.093  3.656  2.067  3.358  2.246  4.044  3.352  3.872  3.62  3.204  4.65  
 
 RHCE  6006  1  25688739  25747363  1p36.11  0.18  0.348  0.367  0  0.2  0.137  0.862  0.357  0.247  0.884  0.135  0  0.124  0.21  0.255  0.348  0.362  0.759  0.25  0.569  1.414  0.582  0.713  0.315  0.153  0.125  0.188  0.098  0.203  0.138  0.339  0.452  0  0.225  0.13  0  0.176  0  1.189  0.44  0.278  0.105  0.048  0  0.302  0.583  0.175  0  0  0.165  0.431  0.369  0.34  0.154  0.691  0.229  0.172  0.214  0.113  0.123  
 
 TMEM57  55219  1  25757348  25826698  1p36.11|1p36.11  0.894  1.455  1.255  2.247  1.668  0.849  2.025  2.411  1.963  2.313  2.008  1.38  1.527  1.305  1.286  1.264  1.533  1.382  2.798  1.411  1.387  2.17  2.326  1.315  1.786  1.315  0.974  1.215  1.214  0.666  1.704  1.779  1.034  0.835  0.503  1.503  1.428  1.532  1.413  0.925  0.861  0.881  1.355  1.584  0.914  1.413  0.959  1.071  2.024  1.997  1.646  0.633  1.074  0.707  0.959  1.2  1.889  0.614  1.003  1.45  
 
 LDLRAP1  26119  1  25870075  25895377  1p36-p35  1.455  0.555  2.15  1.693  2.571  1.865  2.572  3.321  1.82  3.517  3.404  1.869  2.123  2.273  2.822  1.951  1.303  0.253  0.71  1.425  2.316  0.312  2.798  2.573  1.338  2.343  3.12  3.201  2.815  1.664  3.695  3.859  2.542  1.18  1.447  2.199  2.79  2.674  1.422  3.059  1.941  2.225  1.902  1.66  1.523  1.975  1.78  1.139  2.708  2.153  1.902  1.246  2.158  1.338  1.257  1.175  2.404  1.788  1.633  1.458  
 
 MAN1C1  57134  1  25943958  26112698  1p35  0.194  0.188  0.177  0.069  0.783  0.352  0.287  0.597  0.558  1.559  0.199  0.08  0.09  0.207  0.076  0.069  0.066  0.046  0.144  0.887  0  0.167  0.484  0.088  0.047  1.157  0.414  0.748  0.248  0.186  0.905  1.436  0.091  0.314  0.097  0.731  0  0  0.257  0.127  0.133  0.162  0.264  0.051  0.081  0.164  0.268  0.218  0.102  0.398  0.076  0.453  0.076  0.147  0.103  0.066  0.09  0.155  0  0.058  
 
 SEPN1  57190  1  26126666  26144713  1p36.13  3.111  1.408  3.504  4.348  4.091  3.754  4.929  5.253  4.856  5.728  5.096  2.082  4.396  4.739  3.897  1.725  2.609  0.284  2.411  4.136  2.679  0.848  1.354  1.531  4.228  5.916  6.027  4.847  5.218  4.357  5.117  6.08  4.861  3.352  3.678  4.809  4.212  5.055  3.897  5.064  4.282  3.99  4.343  3.26  2.537  2.797  2.959  3.224  4.406  3.952  4.578  3.339  3.531  3.16  3.564  3.128  4.629  4.402  3.186  4.081  
 
 MTFR1L  56181  1  26146396  26159433  1p36.11  2.871  2.384  2.782  3.359  2.966  2.468  3.962  3.404  4.007  3.768  3.862  2.306  2.794  3.708  2.762  2.051  2.59  1.365  3.953  3.366  2.927  2.871  2.059  1.508  2.851  3.127  3.854  3.171  3.291  3.003  4.221  3.97  3.492  2.468  1.88  3.423  3.427  3.268  2.457  3.41  2.619  2.407  3.894  2.89  1.83  2.543  3.545  2.451  2.479  4.137  3.547  1.664  2.522  1.335  2.424  2.491  2.757  2.527  1.369  2.712  
 
 LOC646471  646471  1  26146444  26150097  1p36.11  1.066  0.036  0.523  0.448  0.488  0.415  0.272  0.569  0.569  0.819  0.731  0.257  0.754  0.518  1.081  0.25  0.22  0.109  0.25  0.396  0.709  0.606  0.613  0.146  0.164  0.643  1.002  0.774  0.536  0.307  0.584  0.553  0.806  0.381  0.158  0.529  0.678  0.677  0.212  0.64  0.552  0.525  0.522  0.666  0.197  0.622  0.201  0.518  0.623  0.152  0.571  0.631  0.5  0.236  0.328  0.229  0.695  0.435  0.121  0.199  
 
 AUNIP  79000  1  26158403  26185949  1p36.11  1.169  1.598  1.197  1.992  1.321  1.025  2.082  1.123  0.856  1.4  2.073  1.215  1.593  2.416  1.82  1.794  1.597  1.779  2.687  2.151  2.127  1.463  1.773  0.837  0.968  1.13  1.689  1.007  1.629  0.901  1.945  1.863  1.202  0.829  1.306  1.201  1.436  1.01  0.876  1.875  1.771  1.214  2.303  1.874  1.329  1.6  0.838  1.088  1.729  2.565  1.269  0.964  1.391  0.641  1.162  1.132  1.458  1.651  1.215  1.113  
 
 PAQR7  164091  1  26187974  26197744  1p36.11  0.969  0.39  1.281  1.252  1.308  1.263  1.464  1.604  0.6  1.291  1.228  0.164  0.828  1.566  1.354  1.197  0.459  0.082  0.278  0.299  0.836  0.346  0.837  0.485  1.68  1.246  0.792  0.915  1.127  0.22  0.876  0.324  0.469  0.394  0.875  2.19  1.943  1.692  1.054  3.233  2.322  0.554  1.661  2.565  1.449  1.549  1.065  1.661  0.56  2.294  1.705  1.808  0.826  0.188  1.683  1.976  1.307  2.759  0.434  1.882  
 
 STMN1  3925  1  26210676  26233368  1p36.11  5.242  8.107  5.233  6.407  6.069  6.39  6.128  5.709  4.853  6.746  6.947  4.316  4.538  7.554  5.275  5.488  6.061  7.777  9.532  7.831  6.09  8.143  6.058  5.296  5.961  4.941  4.942  5.272  4.613  3.956  6.462  5.445  5.55  5.246  4.677  4.946  5.666  5.965  5.796  5.633  5.218  5.689  7.133  6.199  4.91  5.956  6.419  5.712  5.802  8.314  5.957  4.305  5.173  3.362  6.148  5.928  5.483  7.478  5.891  6.098  
 
 PAFAH2  5051  1  26286257  26324648  1p36  1.215  0.509  0.876  1.363  1.601  0.407  1.735  0.658  1.237  1.319  1.544  1.937  1.608  1.501  1.034  1.16  1.564  0.587  2.174  0.464  1.04  1.073  1.216  0.62  0.734  1.215  1.129  1.188  0.95  0.745  1.419  1.441  1.286  0.894  0.825  1.493  1.49  1.428  0.698  1.728  1.589  0.489  1.406  1.293  0.656  1.282  0.56  0.53  1.044  1.147  1.2  1.23  1.085  0.443  1.319  0.959  0.984  0.807  0.716  1.467  
 
 EXTL1  2134  1  26348270  26362954  1p36.1  0.093  0.027  0.223  0.136  0.259  0.069  0.047  0.053  0.243  3.6  0.077  0.088  0.046  0.098  0.045  0.115  0.067  0.161  0.023  0.088  0.128  0.187  0.499  0  0.043  3.167  3.686  3.534  3.152  0.78  1.015  3.05  1.07  0.323  0.119  0.059  0.17  0  0.115  0.196  0.878  0.253  0.149  0.044  0.093  0.127  0.121  0.105  0  0.092  0.1  0.494  0.07  0.17  0.139  0.058  0.044  0.166  0.042  0.044  
 
 SLC30A2  7780  1  26364512  26372629  1p35.3  0  0  0.125  0  0.045  0  0  0  0.11  0.382  0.029  0.057  0.258  0.048  0  0.137  0  0  0  0.081  0  0.14  0.357  0  0  0.175  0.506  0.377  0.137  0.144  0  0.084  0.072  0.192  0  0  0.136  0  0.075  0  0.097  0.091  0.046  0  0.264  0.37  0.075  0  0  0.039  0.052  0.468  0.031  0.095  0.086  0  0  0  0  0  
 
 TRIM63  84676  1  26377795  26394125  1p34-p33  0  0.229  0.125  0  0  0  0  0  0.087  0  0  0  0  0.037  0  0.05  0  0  0  0.102  0  0.108  0.32  0  0  2.225  1.391  1.147  2.848  2.915  4.97  1.997  0.849  0.651  0  0  0  0  0.055  0  0  0.154  0.044  0  0  0.054  0.056  0  0  0  0.044  0.402  0  0  0  0  0  0  0  0  
 
 PDIK1L  149420  1  26437655  26452039  1p36.11  0.978  1.031  0.48  0.551  0.714  0.25  0.902  0.352  1.218  0.804  1.389  0.792  0.979  1.479  0.938  1.129  1.214  1.182  1.869  0.96  1.412  1.204  1.43  0.679  0.356  0.5  0.678  0.466  0.453  0.262  0.95  0.615  0.549  0.425  0.264  0.629  0.758  0.719  0.515  0.671  0.827  0.439  0.732  0.61  0.343  0.561  0.155  0.264  0.562  0.786  1.063  0.522  0.463  0.291  0.433  0.534  0.656  0.835  0.237  0.453  
 
 FAM110D  79927  1  26485510  26489119  1p36.11  0  0  0.221  0  0.152  0.075  0.301  0.175  0.357  0.223  0.169  0.153  0  0.306  0.143  0.114  0.173  0.235  0.275  0.254  0.403  0.605  0.519  0.108  0.207  0.173  0.456  0.156  0.212  0.367  0.087  0.158  0  0.198  0.222  0.352  0.098  0  0.182  0.128  0.149  0.357  0.231  0.317  0.16  0.179  0.479  0  0.254  0.403  0.195  1.21  0.145  0.247  0.25  0.245  0  0  0.094  0.107  
 
 ZNF593  51042  1  26496387  26497364  1p36.11  4.262  2.548  4.112  5.676  4.682  4.858  6.084  4.462  4.104  3.966  4.728  4.506  4.827  4.972  3.601  4.798  4.608  1.632  5.078  6.483  5.018  4.91  4.89  4.846  4.703  3.295  5.237  3.784  4.46  4.332  5.814  6.009  4.287  3.399  4.309  4.053  4.401  4.672  4.167  4.505  4.724  4.587  5.018  4.143  3.914  4.502  5.538  4.238  3.053  5.861  4.124  3.241  4.748  3.219  4.843  4.232  3.112  5.504  4.029  3.589  
 
 CNKSR1  10256  1  26503980  26516376  1p36.11  1.851  0  0.202  0  2.91  0.138  0.435  0.131  0.248  0.138  0.578  1.72  2.483  2.721  2.632  2.498  2.248  0  0.293  0.047  0.291  0.377  0.435  0  0.123  0.075  0.162  0.393  0.093  0.195  0  0.137  0.067  0.147  0.094  0.8  1.501  0.869  0.184  1.757  2.681  0.324  0.405  2.236  1.868  2.326  2.122  0.25  0.588  0.394  1.355  1.681  0.263  0.136  0.083  0.084  0.247  0.099  0.113  0.086  
 
 CATSPER4  378807  1  26517118  26529033  1p35.3  0.468  0  1.194  0.723  0.212  0.469  1.226  0.289  0.444  1.095  0.542  0.601  0.509  0.4  0.356  1.278  0.753  0.089  0.969  1.028  0.237  1.056  0.674  0.247  1.094  0.553  1.16  0.387  0.914  0.287  0.346  0.422  0.177  0.353  0.275  0.245  0.752  0.595  1.636  0.35  0.216  0.567  0.855  0.312  0.21  0.247  1.375  0.14  0.175  0.529  1.447  0.774  1.247  0.583  1.186  0.566  0.613  0.586  0.318  0.267  
 
 LOC101928303  101928303  1  26551810  26556331  -  0  0  0.237  0  0.058  0  0.39  0.075  0.335  0  0  0.139  0.301  0.077  0  0.165  0.256  0  0.159  0  0.681  0.315  0.457  0  0  0.157  0.313  0  0  0.272  0  0.139  0  0.306  0.133  0.15  0.195  0  0.111  0.395  0  0.281  0.155  0.489  0  0.294  0.237  0  0.537  0  0.212  0.605  0.072  0.178  0.182  0.221  0.167  0  0.304  0.369  
 
 CEP85  64793  1  26560643  26605529  1p36.11  1.205  1.133  1.726  2.726  1.91  1.453  2.329  2.128  1.929  2.94  3.297  1.639  2.675  2.014  2.115  2.384  2.165  0.621  4.339  2.767  3.344  2.947  2.358  1.886  1.421  2.082  2.543  2.104  2.382  1.312  2.884  2.716  2.105  1.165  1.442  1.82  2.937  2.777  1.656  2.922  2.137  2.578  2.677  2.089  1.267  2.465  2.332  2.146  2.43  3.611  3.077  1.472  2.202  1  1.907  1.414  2.286  1.867  1.934  1.675  
 
 SH3BGRL3  83442  1  26606212  26608013  1p36.11  5.148  5.964  7.965  7.095  4.937  7.397  8.643  7.92  5.486  7.99  6.756  6.44  6.72  6.261  5.51  7.785  7.112  2.351  8.503  6.273  5.552  6.198  5.344  5.745  8.178  7.638  7.732  6.221  6.986  4.462  7.019  8.073  6.318  5.674  5.156  6.233  7.837  8.677  7.844  6.272  6.309  5.208  6.839  6.762  5.265  6.959  5.323  6.878  6.805  7.96  9.364  5.71  7.373  6.137  7.102  6.592  7.343  8.669  6.697  7.276  
 
 UBXN11  91544  1  26608772  26644756  1p36.11  1.463  0.298  1.12  2.227  2.52  1.806  2.73  1.952  1.667  2.613  2.248  1.547  2.173  1.634  2.263  2.648  2.227  0.145  3.893  1.803  1.49  1.636  1.706  1.753  1.316  1.606  3.37  2.038  2.677  1.835  3.112  3.113  2.178  0.841  1.409  1.901  2.481  2.397  1.158  2.183  2.362  2.266  2.223  1.549  1.303  2.024  3.1  1.953  2.233  3.709  1.618  1.392  2.219  0.788  2.459  0.674  1.901  2.495  1.954  1.59  
 
 CD52  1043  1  26644410  26647014  1p36  0  0  0  0  0  0  0  0  0  0  0  0  0  0  0  0.591  0  0  6.522  1.513  3.318  0.788  0  0  0  0.256  0.085  0  0  0  0  0  0  0  0  0  0.336  0  0  0  0  0.203  0  0  0  0  0  0  0  0.345  0.196  0.412  0  0  0  0  0  0  0  0  
 
 AIM1L  55057  1  26648349  26680621  1p36.11  0.261  0.053  0.202  0.079  1.091  0.055  0.198  0.056  0.189  0.108  0.19  1.046  2.483  0.58  2.328  1.078  0.666  0.15  0.202  0.323  0.141  0.278  0.653  0.166  0.15  0.127  0.246  0.103  0.083  0.277  0.207  0  0.027  0.107  0.506  0.15  0.966  0.326  0.439  0.262  1.755  0.329  0.124  2.027  0.39  1.802  0.283  0.225  1.172  0.342  0.189  0.443  0.186  0.135  0.322  0.161  0.112  0.237  0.38  1.321  
 
 ZNF683  257101  1  26688124  26699285  1p36.11  0  0  0.125  0  0  0  0  0  0.186  0  0  0  0  0  0  0  0  0  0.996  0  0  0.1  0.41  0  0  0  0.095  0  0  0.056  0  0  0  0.171  0  0  0  0  0  0  0  0  0.056  0  0  0  0  0  0  0  0  0.489  0  0.124  0.053  0  0  0  0  0  
 
 LIN28A  79727  1  26737268  26756219  1p36.11  0.097  0.036  0.164  0.08  1.874  0.075  0.031  0.035  0.155  0.032  0.027  0  0.047  0.086  0  0.042  0  0.043  0.039  0.029  0.515  0.068  0.508  0  0  0  0.169  0.037  0  0.055  0.045  0.036  0  0.215  0.046  0  0.399  0.044  0.129  0.103  0.046  0.103  0.181  4.77  0  0.102  0.042  0.053  0.06  0  0.047  0.204  0.044  0.059  0.054  0.047  0.034  0.065  0  0  
 
 DHDDS  79947  1  26758772  26797795  1p36.11  1.862  1.059  2.533  3.363  2.259  2.147  2.694  2.678  2.138  2.954  3.122  2.273  2.583  2.787  2.567  2.284  2.071  0.468  3.017  1.707  2.633  1.982  1.99  1.734  2.079  2.384  2.652  2.189  2.608  1.994  3.214  3.08  2.505  1.287  1.502  2.066  2.98  2.552  1.972  2.732  2.448  1.582  2.066  2.668  1.645  2.602  1.711  1.986  2.416  2.797  3.036  1.833  2.6  1.436  2.503  2.275  2.898  2.53  2.171  3.079  
 
 LOC101928324  101928324  1  26789247  26794028  1p36.11  0  0  0.3  0  0  0  0  0.191  0  0.441  0.257  0.225  0.505  0.192  0.36  0.237  0  0  0.38  0.298  0  0.204  0.425  0.557  0  0.221  0.162  0.199  0  0.061  0.37  0.216  0  0  0.417  0  0.27  0.256  0.362  0  0.509  0  0.066  0  0  0.303  0  0  0.467  0.369  0.195  0.308  0.303  0  0  0.246  0.504  0.268  0  0  
 
 HMGN2  3151  1  26798901  26803133  1p36.1  6.396  7.672  5.029  5.508  6.334  5.688  6.904  6.842  4.277  6.782  7.637  5.582  5.309  6.949  5.809  6.576  5.889  7.119  9.396  7.126  5.962  6.792  5.085  4.74  5.76  5.517  5.592  4.482  4.465  3.622  7.016  6.229  5.481  3.859  3.989  5.158  5.913  5.744  5.686  5.831  5.861  5.553  5.948  5.987  4.987  5.14  5.594  4.878  6.008  7.684  6.489  3.794  5.649  3.187  5.313  5.017  5.965  6.096  5.51  6.259  
 
 RPS6KA1  6195  1  26856248  26901520  1p  2.493  1.122  0.869  1.809  2.468  1.12  3.163  1.516  1.742  2.936  3.252  2.56  4.205  3.551  3.582  3.176  3.666  0.708  4.407  4.57  2.59  1.743  2.576  2.226  1.363  2.555  3.518  2.767  2.212  2.687  3.015  2.589  3.487  2.032  1.392  2.706  2.631  1.792  1.306  2.6  2.527  2.399  2.526  3.443  2.623  3.056  1.507  2.508  3.503  3.868  3.929  2.074  2.933  1.67  3.238  2.442  2.472  1.986  3.186  3.363  
 
 ARID1A  8289  1  27022521  27108601  1p35.3  4.23  2.513  2.904  4.439  2.368  3.25  4.225  4.287  3.16  4.054  4.31  3.165  4.199  4.219  4.741  3.711  3.858  2.916  4.843  3.866  3.974  4.822  3.052  3.578  2.881  3.431  4.073  3.827  3.538  3.708  4.167  4.144  3.632  1.764  2.922  4.21  4.558  4.084  2.642  5.143  4.396  2.973  5.352  3.724  3.697  3.667  3.616  3.965  3.302  3.826  4.074  3.101  4.129  2.377  3.605  2.723  4.208  4.195  3.401  3.839  
 
 PIGV  55650  1  27114453  27124894  1p36.11  2.066  1.899  1.242  1.405  2.098  0.85  2.368  1.519  1.453  1.425  1.13  1.492  2.099  2.275  1.479  2.151  2.121  1.905  2.184  1.498  1.407  1.217  2.715  1.477  1.22  1.152  1.857  1.44  1.842  1.642  2.03  1.877  1.36  1.106  0.965  1.837  1.877  1.87  1.042  2.25  2.136  1.156  2.211  0.879  0.699  1.094  2.184  1.434  1.164  3.039  1.982  1.001  1.193  0.681  1.653  1.367  1.532  1.203  1.378  1.718  
 
 ZDHHC18  84243  1  27153200  27182211  1p36.11  1.958  0.268  1.875  2.249  1.609  2.106  2.735  2.909  2.43  2.595  3.22  1.36  2.231  1.837  2.572  2.684  1.545  0.063  3.407  0.694  2.117  2.325  2.161  1.915  1.675  2.018  2.17  2.052  2.16  1.248  2.604  2.033  2.11  1.155  1.873  1.928  2.949  2.494  1.412  3.229  2.54  2.127  1.976  2.164  1.78  2.022  1.354  2.802  3.056  3.379  2.355  2.763  2.139  1.721  2.364  2.046  2.579  2.705  2.458  2.08  
 
 SFN  2810  1  27189632  27190947  1p36.11  5.013  4.152  4.038  0.558  5.445  0.923  0.94  1.041  0.918  0.836  3.095  5.543  6.474  5.639  6.801  5.633  6.517  1.542  1.739  0.774  1.504  1.417  3.021  3.285  0.593  0.629  0.595  0.542  0.597  0.694  0.58  0.582  0.815  0.691  5.302  3.998  2.394  4.747  5.271  5.499  6.196  5.82  0.966  5.655  2.729  5.185  6.318  6.93  5.946  8.395  6.54  6.132  3.001  3.518  2.137  1.59  1.129  3.441  6.527  5.465  
 
 GPN2  54707  1  27205872  27216869  1p36.11  2.934  2.21  2.251  2.537  2.86  1.943  3.791  3.283  2.29  2.977  3.355  2.358  2.876  3.08  2.536  2.358  2.736  0.979  3.387  3.158  3.059  2.774  2.672  2.094  2.267  2.009  3.546  2.94  2.865  2.693  3.727  3.96  3.093  2.085  1.737  1.989  3.425  2.801  2.247  2.687  2.795  2.487  3.37  2.307  2.485  2.735  3.347  2.512  2.824  3.787  2.638  2.225  3.174  1.539  3.04  2.336  2.475  2.869  2.353  2.751  
 
 GPATCH3  63906  1  27216978  27226962  1p35.3-p35.1  0.739  0.137  1.21  2.315  1.517  1.323  1.587  1.614  1.49  1.889  2.16  1.497  1.652  1.213  1.32  1.74  1.561  0.105  2.768  0.829  1.772  1.622  1.797  1.302  1.68  1.221  1.24  1.38  1.474  1.246  2.478  2.167  1.191  0.401  0.885  1.301  1.822  1.653  0.853  1.881  1.537  1.52  1.785  1.657  0.946  1.441  1.025  1.543  1.3  3.111  1.573  1.251  1.512  0.804  1.351  0.968  1.434  1.389  1.54  1.153  
 
 NR0B2  8431  1  27237974  27240567  1p36.1  0  0  0.17  0.212  0.086  0  0  0  0.121  0.082  0.075  0.141  1.711  0.113  0.134  0  0  0  0.09  0  0  0.082  0.545  0  0  0  0.239  0.058  0  0.114  0  0  0  0.351  0.086  0.109  0.153  0  0.063  0  0  0.251  0.045  0.097  0  0  0  0  0.159  0.218  0.078  0.393  0.06  0.098  0  0  0.092  0  0  0.299  
 
 NUDC  10726  1  27248212  27273362  1p35-p34  4.047  2.855  4.158  5.238  4.543  4.433  6.002  5.373  4.433  5.032  5.581  4.611  5.018  5.883  4.122  4.832  5.106  1.275  6.82  5.84  5.212  5.59  4.708  4.736  5.568  3.627  4.999  4.806  4.95  4.122  5.846  5.777  4.829  3.59  4.29  4.366  5.089  4.697  5.089  5.202  4.639  4.818  5.015  4.499  4.207  4.551  5.472  5.423  4.219  7.23  5.41  3.669  4.955  3.699  5.233  4.143  4.636  5.334  4.559  5.555  
 
 KDF1  126695  1  27276046  27286901  1p36.11  2.355  0  0.102  0  2.838  0  0  0.097  0.13  0.065  0.044  3.182  3.525  2.945  3.066  3.308  3.092  0.266  0.087  0.25  0.497  0.188  2.075  1.212  0  0  0.154  0.046  0.093  0.298  0.145  0  0  0.179  0.069  0.476  0.317  0  0.056  0.411  2.983  0.129  0.066  0.512  2.318  2.864  0.151  0  0.18  0  0.296  1.242  0  0.081  0.132  0  0  0  0.69  0.348  
 
 TRNP1  388610  1  27320194  27327377  1p36.11  0.43  0.564  2.3  1.001  0.664  1.515  1.311  0.642  1.753  1.702  1.186  2.149  3.287  3.028  0.9  4.23  3.174  2.24  0.062  0.219  0.138  0.334  0.994  0.299  0.723  1.278  1.75  1.899  0.985  1.655  2.134  2.007  1.728  1.593  3.85  3.008  3.291  1.932  3.45  1.025  0.699  1.078  1.989  2.148  0.742  2.325  3.814  1.443  3.204  2.329  4.609  4.957  3.369  2.232  4.055  4.091  2.861  0.255  3.76  4.183  
 
 FAM46B  115572  1  27331510  27339333  1p36.11  1.657  0.088  2.416  1.16  2.845  1.361  0.167  1.232  0.367  1.002  2.398  0.13  0.476  2.217  0.757  0.233  0.343  0.043  0.074  0.097  0.101  0.264  0.834  0.168  0.109  0.251  1.97  2.515  0.089  0.16  0.075  0.262  2.04  0.843  0.335  1.182  1.694  0.972  1.131  0.746  1.053  0.784  1.635  1.785  1.648  2.642  1.022  2.363  1.16  3.358  1.089  1.498  0.274  1.037  1.14  1.091  0.908  1.802  0  0.43  
 
 SLC9A1  6548  1  27425299  27481621  1p36.1-p35  2.449  0.509  2.413  2.324  3.183  1.67  3.366  3.744  2.012  3.257  3.41  2.263  2.953  2.01  2.882  2.009  2.69  0.357  2.353  1.272  1.82  2.282  1.27  1.253  2.828  2.468  2.77  2.573  2.673  2.151  3.195  3.34  2.565  1.26  1.403  2.616  3.508  2.741  2.959  2.901  3.23  2.638  1.708  2.748  1.779  2.641  1.865  3.157  2.747  3.22  3.944  1.747  2.765  1.947  2.229  1.965  2.654  1.962  1.919  3.548  
 
 WDTC1  23038  1  27561006  27635124  1p36.11  2.135  0.359  2.351  3.028  2.393  1.791  2.716  2.971  2.686  3.374  2.669  2.345  2.646  2.03  3.143  2.049  2.432  0.366  2.552  1.87  2.466  1.443  2.534  2.212  2.336  2.14  2.661  2.514  2.552  1.649  2.568  2.152  2.651  1.449  0.98  1.853  3.191  3.043  1.206  2.643  1.857  1.471  1.733  0.811  1.8  2.933  0.571  2.77  2.44  3.18  3.483  1.89  1.918  1.279  1.751  1.819  2.165  2.196  1.612  2.44  
 
 TMEM222  84065  1  27648635  27662891  1p36.11  3.623  0.835  3.119  4.312  4.178  3.318  5.201  4.832  4.9  4.56  4.761  3.893  3.949  4.072  3.515  4.17  3.863  0.273  5.409  3.361  3.51  3.973  3.462  2.978  4.15  3.006  5.019  3.51  4.294  3.601  4.552  4.49  4.105  2.595  2.768  3.717  4.151  3.758  2.831  4.735  3.748  3.581  3.075  2.96  3.126  3.475  4.164  4.391  3.52  5.693  3.863  2.64  4.342  2.436  3.541  2.846  3.669  4.301  4.346  3.873  
 
 ACTG1P20  644961  1  27650364  27653016  1p36.11  1.468  0.726  3.331  2.917  1.577  1.4  3.408  2.46  3.47  2.293  2.592  1.642  2.09  1.805  1.427  1.573  2.378  0.337  2.759  1.321  0.924  2.668  1.827  0.724  1.924  2.375  3.252  1.575  2.796  3.896  1.907  1.22  1.632  2.761  1.951  1.439  2.766  1.577  1.588  2.195  0.832  3.15  3.957  1.552  0.765  1.521  2.085  1.299  1.582  2.165  2.403  1.213  3.027  1.496  3.129  3.781  1.619  1.098  2.318  1.482  
 
 SYTL1  84958  1  27668482  27680423  1p36.11  2.363  0  0.19  0  3.282  0.191  0.073  0.187  0.67  0.226  0.773  1.649  1.999  3.017  2.282  2.754  1.815  0.145  4.347  3.933  0.46  2.937  3.36  2.054  0  0.182  0.205  0.14  0.115  0.134  0.151  0  0.178  0  0.208  0.246  0.309  1.196  0.208  0.27  3.2  0.548  1.603  1.733  1.369  2.404  2.033  1.323  0.393  3.152  1.259  1.137  0.231  0.167  1.029  0.1  0.225  0.142  0.644  0  
 
 MAP3K6  9064  1  27681669  27693357  1p36.11  1.641  0.429  1.7  1.834  1.232  0.922  1.826  2.96  1.082  2.883  2.033  0.61  1.811  1.878  1.938  1.452  0.877  0.17  1.425  0.595  0.914  0.428  0.58  0.642  1.199  2.347  2.904  2.937  2.645  1.072  2.938  2.92  3.054  1.062  0.681  1.638  1.396  1.551  0.89  2.551  1.658  1.372  1.934  1.79  1.104  2.032  0.329  2.529  2.91  3.189  1.932  1.659  1.864  0.946  1.546  1.431  2.526  1.388  1.577  0.895  
 
 FCN3  8547  1  27695600  27701315  1p36.11  0  0  0  0  0.163  0  0  0  0  0.069  0  0  0  0.149  0  0  0  0  0  0  0  0.196  0.558  0  0  0  0.126  0.137  0  0.264  0.154  0.21  0  0.215  0  0  0  0  0.059  0.295  0  0.443  1.532  0  0  0  0  0.22  0  0  0  0  0.15  0.131  0.145  0  0  0  0  0  
 
 CD164L2  388611  1  27705589  27709823  1p36.11  0.206  0  0.126  0  0.847  0  0  0  0.139  0  0  0  0.257  1.388  0  0.1  0.105  0  0  0  0.134  0.052  0.691  0  0  0  0.153  0  0  0.06  0  0  0  0.187  0  0.265  0  0  0.041  0.24  0  0.29  0.057  0.113  0.408  0.457  0.069  0  0  0.074  0.087  0.47  0  0.105  0.063  0  0  0  0  0  
 
 GPR3  2827  1  27719147  27722317  1p36.1-p35  0  0.204  0.85  1.093  0.262  1.462  1.219  1.8  1.581  1.206  1.004  0.176  0.738  1.479  1.212  0.625  1.535  0.165  0.626  0.508  2.053  0.604  0.877  0.891  1.35  1.927  1.918  2.46  2.442  1.328  3.039  2.764  2.474  1.344  0.478  1.5  2.04  1.966  0.408  2.555  0.495  1.462  1.615  0.997  0.184  0.326  1.177  2.118  1.115  2.771  1.672  1.02  0.633  0.209  0.736  0.641  0.729  1.149  0.503  0.511  
 
 WASF2  10163  1  27730729  27816698  1p36.11  3.495  2.458  4.292  4.845  3.834  3.379  4.734  5.213  4.19  5.199  4.877  3.966  3.635  4.178  4.297  4.314  4.752  1.615  4.883  4.204  4.415  4.471  4.15  3.646  4.558  3.9  4.89  4.25  4.513  3.781  5.279  5.003  3.899  2.65  2.987  4.26  5.178  4.944  3.622  4.928  4.529  3.361  4.394  4.166  3.418  4.257  3.82  4.402  4.912  5.411  4.956  3.118  4.774  3.096  3.982  4.268  5.296  4.151  4.522  4.712  
 
 AHDC1  27245  1  27860755  27930005  1p36.13  1.326  0.427  2.171  2.47  1.413  1.011  2.08  3.542  2.684  3.668  2.243  1.322  2.005  1.87  2.312  1.896  1.849  0.333  1.612  0.961  1.988  1.743  0.645  0.757  1.674  2.009  2.463  2.721  2.411  0.785  2.683  2.591  2.506  1.159  1.07  1.861  2.629  3.295  1.788  2.216  2.421  1.591  1.372  1.357  2.075  2.143  1.544  3.335  1.542  2.942  2.487  2.176  3.096  1.831  1.254  1.747  2.815  2.357  1.367  1.956  
 
 FGR  2268  1  27938800  27961727  1p36.2-p36.1  0  0  0.172  0.318  0.037  0  0.049  0.179  0.327  0.173  0.104  0  0  0.027  0  0.172  0  0.195  0.216  2.038  0  0.688  0.501  0.104  0  0.122  0.118  0.047  0  0.094  0.172  0  0  0.162  0  0  0  0  0.181  0  0  0.285  0.047  0  0  0.067  0.069  0  0  0.051  0  0.389  0  0.104  0.054  0  0  0  0  0  
 
 IFI6  2537  1  27992571  27998724  1p35  0.794  2.759  3.316  10.087  3.737  3.452  5.017  5.714  3.355  5.551  4.016  3.601  6.318  3.839  2.581  9.488  3.664  4.465  3.883  3.167  0  5.48  3.065  8.391  7.26  3.795  5.596  4.657  5.215  2.755  5.102  4.649  3.602  3.701  1.535  0.972  1.554  3.095  9.707  2.584  5.13  3.503  2.896  1.218  1.348  2.484  4.975  2.626  1.445  4.663  3.796  2.097  3.031  5.627  4.017  2.458  7.506  4.495  2.631  2.238  
 
 FAM76A  199870  1  28052489  28089423  1p35.3  0.612  0.796  0.78  1.513  0.822  0.593  1.047  1.22  1.049  1.228  1.008  0.749  1.032  1.561  0.856  1.048  1.003  0.711  1.992  0.69  0.739  1.136  1.025  0.718  0.555  0.638  0.904  0.575  0.607  0.188  1.055  0.756  0.496  0.524  0.37  0.695  0.8  0.803  0.473  0.835  0.649  0.553  0.987  0.664  0.399  0.83  0.694  0.855  1.047  1.489  1.062  0.433  0.654  0.204  0.506  0.433  1.204  0.969  0.831  0.76  
 
 STX12  23673  1  28099693  28150963  1p35.3  1.267  1.718  1.91  3.884  1.825  1.11  2.618  2.614  2.606  2.586  2.239  1.027  1.997  2.162  1.475  2.025  1.845  1.474  2.762  1.923  1.251  1.23  1.768  1.028  1.852  2.07  1.542  1.49  1.758  1.447  2.497  2.029  1.597  1.678  0.586  1.619  1.659  1.831  2.71  1.861  1.348  1.378  2.541  1.38  0.969  1.977  0.988  1.305  1.222  3.106  2.428  0.459  1.401  1.095  1.503  1.412  1.975  1.5  1.102  2.188  
 
 PPP1R8  5511  1  28157251  28178183  1p35.3  2.537  2.681  2.16  2.893  2.706  2.209  3.127  2.847  2.482  2.997  3.66  2.388  2.993  3.88  2.56  2.722  2.734  2.661  4.453  3.141  3.176  3.018  3.115  2.4  2.395  2.521  2.424  2.544  2.532  2.183  3.033  2.972  2.565  1.589  1.82  2.24  2.766  2.534  2.299  3.194  2.505  2.099  3.226  2.888  2.07  2.353  2.174  2.531  2.565  4.211  2.887  1.577  2.838  1.68  2.535  2.131  2.842  2.623  2.582  2.728  
 
 SCARNA1  677774  1  28160911  28161077  1p35.3  0  0  0  0  0  0  0  0  0  0  0  0  0  0  0  0  0  0  0  0  0  0.778  0  0  0  0  0  0  0  0  0  0  0  0  0  0  0  0  0  0  0  0  0  0  0  0  0  0  0  0  0  0  0  0  0  0  0  0  0  0  
 
 THEMIS2  9473  1  28199053  28213196  1p35.3  0.841  0.621  1.097  2.813  0.171  0.751  0.734  1.104  0.217  0.761  0.18  0  0.616  2.314  0.549  0  0.179  0.349  2.053  3.199  1.156  0.275  3.33  2.617  0.687  2.231  1.514  0.769  1.6  1.854  3.358  1.475  0.695  0.568  0.136  0.141  1  0.598  1.352  1.268  0  0.331  1.125  0.241  1.329  1.956  0.039  0.502  0.136  1.153  0.647  0.646  0.303  0.132  0.174  0.855  1.917  0.631  0  0.089  
 
 RPA2  6118  1  28218035  28241308  1p35  2.445  3.581  2.264  3.241  3.438  3.38  4.229  2.903  2.917  4.057  4.76  3.156  3.573  3.815  2.965  3.725  3.793  2.315  5.592  4.268  3.963  4.437  3.563  2.641  2.911  3.125  3.393  3.431  3.23  2.072  4.392  3.737  3.616  2.45  2.594  2.936  3.461  3.474  2.856  4.409  2.898  3.079  4.618  2.955  2.459  3.074  2.583  3.546  2.943  6.077  4.17  2.285  3.242  1.86  3.391  2.439  3.17  3.569  2.113  3.1  
 
 SMPDL3B  27293  1  28261465  28285668  1p35.3  2.834  0.091  0.182  0  3.165  0  0.233  0.107  0.486  0.11  1.035  1.116  2.574  2.58  2.935  2.535  1.484  0.476  0.361  0.892  0.325  0.233  3.315  2.66  0  0.132  0.097  0.087  0.152  0.415  0  0.087  0.132  0.262  0.405  1.311  0.189  0.358  0.074  1.836  0.384  0.359  1.639  0.919  2.543  2.747  1.645  1.67  0  3.782  0.924  1.705  0.117  0.35  2.287  0.414  0.144  0.293  2.283  0.821  
 
 XKR8  55113  1  28285972  28294607  1p35.3  0.848  1.167  0.644  0.76  1.228  0.992  1.02  1.412  1.533  2.841  2.644  0.904  1.555  1.803  1.303  1.49  0.942  0.491  2.516  1.08  0.618  1.707  1.516  1.022  1.547  1.707  0.311  1.36  2.103  0.129  0.863  0.608  0.093  0.207  0.883  0.91  1.684  2.597  1.252  1.836  1.429  1.183  2.463  1.235  0.903  0.769  0.76  1.596  0.643  2.876  1.466  1.419  1.689  1.295  1.456  0.079  1.843  0.52  2.066  0.993  
 
 EYA3  2140  1  28296854  28415148  1p36  1.932  1.185  1.443  1.191  1.141  1.147  2.304  1.936  1.286  2.011  2.467  1.723  1.938  2.623  2.226  1.497  1.779  1.02  3.05  2.499  2.45  1.979  1.697  1.886  1.842  1.869  1.7  2.308  2.275  1.485  2.765  2.176  2.506  1.434  0.9  1.321  2.309  1.919  1.09  2.176  1.313  0.777  1.78  3.495  1.682  1.681  0.806  2.169  2.358  2.387  2.209  0.906  2.154  0.961  1.703  1.463  2.791  2.009  2.044  2.129  
 
 PTAFR  5724  1  28473676  28520447  1p35-p34.3  0.154  0.031  0.233  0.116  0.125  0  0.206  0.039  0.246  0.096  0.174  1.1  0.335  0.371  0.244  0.306  0.143  0.129  0.146  0.178  0.197  0.126  0.623  0  0.058  0.087  0.1  0.103  0.11  0.092  0.114  0.155  0  0.333  0.125  0.04  0.259  0.085  0.176  0.297  1.205  0.428  0.03  0.954  0.76  1.048  0.231  0.097  0.075  0.182  0.088  0.464  0.092  0.053  0.09  0.082  0.038  0.113  0.192  0.056  
 
 DNAJC8  22826  1  28526789  28559542  1p35.3  4.429  4.569  4.095  4.736  4.793  4.005  5.746  4.746  4.29  4.819  5.751  3.948  4.119  5.606  4.213  4.347  4.619  4.221  6.747  6.727  4.531  5.861  4.508  3.776  4.625  4.024  5.34  3.859  4.625  4.492  5.575  5.173  4.037  2.864  3.409  3.927  4.521  4.422  5.19  4.687  3.666  3.826  5.263  4.357  3.08  3.941  6.57  4.267  3.81  6.434  4.911  3.158  4.925  3.188  5.594  4.216  4.599  4.729  4.426  4.665  
 
 ATPIF1  93974  1  28562601  28564616  1p35.3  5.796  8.051  5.013  5.598  7.501  6.377  6.762  5.915  4.609  6.267  6.654  6.973  6.236  7.028  5.597  6.811  7.68  9.868  8.415  7.786  7.088  7.097  6.736  6.621  5.441  4.605  6.091  3.898  5.242  3.161  6.515  5.931  4.772  4.239  5.761  5.602  4.538  5.405  6.859  4.971  6.093  5.861  5.938  6.469  5.288  6.981  7.295  5.621  5.805  8.276  5.56  4.813  6.351  5.496  6.592  5.691  5.755  6.374  5.376  5.444  
 
 SESN2  83667  1  28585962  28609002  1p35.3  1.245  0.384  2.814  4.158  1.991  0.923  2.344  0.997  3.541  2.182  2.307  0.95  1.261  2.06  1.662  1.579  3.256  0.643  2.204  0.784  3.102  1.428  3.803  1.25  2.788  1.691  0.972  1.057  0.648  2.705  2.22  2.782  0.748  0.978  0.726  1.057  2.042  2.183  3.544  2.109  1.65  2.121  2.323  2.004  1.305  3.134  0.461  1.805  1.25  2.815  2.156  1.642  2.254  1.904  2.739  2.441  2.066  1.628  3.142  2.72  
 
 MED18  54797  1  28655512  28662478  1p35.3  0.725  0.455  0.718  1.496  0.754  0.918  0.511  1.671  1.028  1.805  2.478  1.786  1.767  0.821  1.923  1.562  1.659  0.17  3.298  0.602  1.651  1.015  0.785  1.189  1.892  1.704  0.478  1.675  1.875  0.206  2.227  1.996  1.209  1.038  1.087  1.62  2.007  1.62  0.584  2.092  1.886  1.131  0.273  1.373  0.769  1.622  0.032  1.516  1.064  3.199  1.749  0.968  2.014  1.202  1.097  1.417  1.706  1.268  0.64  2.046  
 
 PHACTR4  65979  1  28696092  28826881  1p35.3  2.332  2.125  1.94  2.877  2.768  2.391  2.997  2.89  2.083  2.834  3.501  2.331  2.808  2.938  2.927  2.72  2.7  2.204  2.853  1.867  2.377  3.084  1.877  2.202  2.575  2.019  2.951  2.542  2.58  2.003  2.748  2.369  2.528  1.427  2.239  2.521  2.675  2.71  2.656  3.13  2.627  1.823  3.299  2.852  2.07  3.846  1.517  3.417  3.245  3.979  2.895  1.879  3.059  2.084  3.244  2.722  3.154  2.399  2.904  2.938  
 
 SNHG3  8420  1  28832454  28837404  1p36.1  2.704  4.185  2.639  3.489  3.297  3.497  3.241  3.063  2.354  2.972  4.421  1.824  2.934  4.026  3.055  3.959  4.348  3.882  6.234  4.862  4.883  4.2  4.508  3.796  2.772  2.385  3.133  2.84  3.306  1.961  4.032  3.586  3.394  2.804  3.053  2.825  2.885  2.491  3.778  2.806  2.399  3.773  3.496  2.56  1.939  3.41  2.862  3.422  2.633  5.829  2.219  2.737  2.76  2.019  3.565  2.559  3.765  3.745  3.613  1.563  
 
 RCC1  1104  1  28832454  28865708  1p36.1  4.299  2.622  2.623  3.09  2.886  3.09  4.68  3.513  1.721  3.732  4.584  3.579  4.964  5.235  5.111  4.351  4.131  1.633  5.331  3.225  4.424  3.84  2.764  3.645  3.799  2.599  4.195  3.837  3.757  3.322  4.524  4.53  4.296  1.491  2.956  3.156  4.296  3.25  2.024  4.78  4.342  3.297  4.335  3.929  3.328  3.87  3.196  4.388  4.011  5.487  3.861  2.825  4.108  2.377  3.256  2.951  3.599  3.997  3.442  3.492  
 
 RNU105A  26768  1  28835069  28835227  1p35.3  8.217  2.943  8.912  5.807  4.698  12.119  5.231  7.74  5.206  6.817  6.538  10.95  7.953  7.971  4.194  9.554  8.998  0  4.698  6.89  8.521  7.333  4.283  10.002  9.222  3.857  5.656  5.007  7.402  5.646  8.769  7.762  5.303  6.546  8.643  6.259  4.074  7.249  6.819  4.759  7.771  11.213  3.65  6.912  8.291  8.637  0  7.989  4.889  4.806  3.69  7.505  7.656  8.326  5.754  9.326  6.205  9.549  8.282  4.129  
 
 TRNAU1AP  54952  1  28879528  28905057  1p35.3  2.248  3.332  2.037  2.257  2.545  2.064  3.495  2.89  1.784  2.862  3.434  1.795  2.184  3.07  1.903  2.798  1.92  3.113  4.191  2.924  2.194  3.195  2.091  1.619  2.812  1.75  3.309  1.952  2.453  1.759  3.186  2.858  2.353  0.942  1.521  1.941  2.743  2.167  2.436  2.662  3.044  1.564  4.504  1.808  1.54  2.037  3.346  2.682  2.554  4.446  2.291  1.686  3.053  1.326  2.438  1.699  2.896  3.718  2.318  2.218  
 
 SNHG12  85028  1  28905049  28908366  1p35.3  2.518  3.361  1.822  2.941  2.887  2.097  2.691  2.128  1.225  2.427  3.045  1.219  2.377  4.546  2.041  3.318  3.847  4.027  4.425  3.904  3.121  3.114  2.331  2.161  2.289  2.066  2.491  2.051  1.752  1.439  3.196  2.662  2.424  0.822  2.426  1.876  2.792  2.51  3.486  2.163  1.967  3.082  3.394  2.79  1.906  2.83  1.43  3.083  1.809  4.879  1.849  1.518  2.942  1.875  3.716  2.78  2.887  2.808  2.466  2.09  
 
 SNORA61  677838  1  28906275  28906405  1p35.3  3.207  3.44  0  0  2.647  4.018  4.039  2.534  0  3.055  5.045  0  3.768  5.93  4.247  4.348  3.561  0  5.857  3.809  0  3.813  0  0  0  3.344  3.48  3.568  3.024  2.116  3.934  3.398  4.471  0  0  3.496  3.78  0  2.3  0  0  5.195  4.55  0  3.182  4.854  4.604  4.12  0  5.622  3.663  0  4.297  2.691  4.4  3.034  5.229  0  3.73  0  
 
 SNORA44  677825  1  28906892  28907024  1p35.3  0  0  0  0  0  0  0  0  0  0  0  0  0  3.37  0  2.09  0  0  3.416  2.359  0  1.258  0  0  0  0  2.793  2.056  0  0  0  0  0  0  0  0  2.334  0  1.307  0  0  4.597  0  3.902  0  0  1.803  0  0  3.041  0  0  0  0  1.958  0  3.206  0  0  0  
 
 SNORA16A  692073  1  28907431  28907565  1p35.3  0  0  0  0  0  0  0  0  0  1.814  2.269  0  0  0  0  2.025  0  0  0  0  0  0  0  0  0  0  0.855  0  0  0  0  0  0  0  0  0  0  0  1.248  0  0  2.264  0  0  0  0  0  0  0  0  0  0  0  2.164  0  3.278  0  0  0  0  
 
 RAB42  115273  1  28918711  28921088  1p35.3  0  0.18  0.189  0  0.192  0  0.325  0.485  0.198  0.181  0.775  0  0.372  0.123  0.237  0.278  0  0  0  0.205  0.279  0.366  0.755  0  0.136  0.23  0.248  0.062  0  0.137  0.153  0.22  0.14  0.253  0  1.187  0.551  0.581  0.165  0.184  0.14  0.42  0.126  0  0.956  1.501  0.107  1.12  0.23  1.189  0.148  0.805  1.393  1.429  1.547  2.471  3.447  1.149  1.758  1.234  
 
 TAF12  6883  1  28929608  28969604  1p35.3  2.972  2.542  2.072  3.146  2.948  2.554  4.148  3.347  2.627  3.608  3.872  2.795  3.146  4.595  2.234  3.151  3.193  4.856  4.229  2.958  3.233  3.103  3.126  2.197  2.62  2.473  2.194  1.957  2.204  1.235  3.359  3.025  1.968  2.067  2.467  2.761  3.147  2.272  2.702  3.656  2.339  2.496  3.745  2.987  2.246  3.325  2.839  2.414  2.313  4.695  3.463  1.521  2.768  1.898  3.041  2.58  2.405  2.14  2.572  2.629  
 
 RNU11  26824  1  28975111  28975246  1p35  0  0  2.906  0  1.959  4.05  0  0  0  1.65  0  0  0  1.709  0  3.163  3.47  3.701  0  0  0  3.216  0  4.858  0  0  0  0  0  0  4.101  3.254  0  0  0  0  0  0  1.528  0  0  6.836  0  0  3.755  2.156  0  0  0  0  0  0  1.881  0  0  2.983  0  0  0  0  
 
 GMEB1  10691  1  28995239  29042115  1p35.3  1.614  0.958  1.018  1.793  1.061  0.922  1.965  1.869  1.261  1.63  2.04  1.267  1.694  1.836  1.822  1.179  1.091  0.593  2.11  1.317  1.463  1.262  1.36  1.305  1.384  0.983  2.045  1.301  1.026  1.149  1.959  1.48  1.452  0.543  0.925  1.379  1.956  1.056  0.922  2.105  1.246  0.782  2.314  1.237  0.987  0.967  1.363  1.169  1.465  1.873  1.417  1.004  1.415  0.486  0.881  0.807  1.592  0.854  1.323  1.243  
 
 YTHDF2  51441  1  29063132  29096287  1p35  4.036  4.611  3.113  4.536  3.782  2.64  4.257  4.146  4.228  3.966  4.747  3.289  4.332  5.398  4.197  3.949  3.528  5.438  5.657  4.399  4.159  4.237  3.892  3.309  3.635  4.083  4.193  4.378  4.382  3.891  4.721  4.669  3.901  3.002  2.494  3.732  4.118  3.374  3.428  4.558  3.416  3.07  4.446  4.018  3.305  3.64  2.488  3.353  3.686  5.174  3.729  2.419  3.822  2.371  3.598  3.204  3.977  3.193  3.68  4.086  
 
 OPRD1  4985  1  29138653  29190208  1p36.1-p34.3  0  0.175  0.2  0  0.128  0.086  0.209  0.306  0.211  1.513  0.051  0.208  0  0.094  0  0.182  0.171  0.357  0.046  0.207  0  0.576  0.437  0.121  0  1.123  0.503  2.316  0.531  0.182  0.108  0.127  0  0.173  0.215  0.062  0.147  0  0.258  0.135  0  1.089  0.069  0  0  0.183  0.158  0  0  0  0.096  1.946  0.046  0.239  0.119  0.149  0  0  0.119  0  
 
 EPB41  2035  1  29213602  29446558  1p33-p32  2.05  1.079  0.471  0.248  1.275  1.537  1.246  1.751  1.755  1.685  2.573  1.817  2.756  2.55  2.591  2.041  2.053  2.333  3.128  2.214  3.636  3.036  2.179  2.848  0.498  1.996  1.398  2.001  2.154  1.177  2.611  1.207  1.954  1.066  0.871  1.342  1.792  1.521  1.073  1.241  1.303  1.04  1.872  1.177  2.228  1.518  0.275  1.214  0.305  1.611  2.379  1.06  0.858  1.481  1.527  1.751  1.755  1.034  1.409  1.805  
 
 TMEM200B  399474  1  29445936  29450421  1p35  0.114  0.697  1.544  0.559  0.276  0.341  0.104  1.272  0.976  1.825  1.045  0.363  0.092  1.237  1.15  0.364  0.275  1.623  1.064  0.469  0.193  1.492  2.147  0.139  0.643  1.785  1.515  0.178  0.114  0.171  0.173  0.083  0  0.493  0.22  0.639  1.253  1.974  1.919  1.614  0.125  1.292  1.984  0.12  0.4  0.152  0.612  1.582  0  2.266  0.608  1.081  1.324  1.28  2.217  0.412  2.118  2.213  1.762  1.91  
 
 SRSF4  6429  1  29474249  29508637  1p35.3  2.711  3.674  3.444  4.28  3.06  3.424  4.292  3.94  4.162  4.224  4.745  2.956  4.08  3.912  4.219  3.818  3.731  3.266  5.606  3.304  3.98  4.129  3.803  2.883  3.134  3.06  4.021  2.978  3.376  2.838  4.248  3.807  3.35  2.54  2.727  3.511  4.061  3.141  3.402  3.569  3.134  3.368  4.042  3.773  2.771  3.389  3.612  3.635  3.665  5.132  4.058  2.492  3.732  2.493  3.37  3.577  3.9  3.657  3.085  3.086  
 
 MECR  51102  1  29519168  29557470  1p35.3  1.838  0.791  1.563  2.399  1.521  1.51  2.549  1.983  2.207  2.265  2.155  1.681  2.333  2.639  1.419  2.299  2.33  0.805  3.461  2.96  2.031  2.111  1.87  1.88  2.005  1.795  2.233  1.662  1.787  2.009  2.769  2.452  1.819  1.289  1.421  1.538  2.274  2.161  1.663  2.666  2.113  1.979  2.482  1.737  1.503  2.334  2.313  2.29  1.134  4.098  2.252  1.326  1.993  0.776  2.313  1.393  1.646  2.195  1.685  2.229  
 
 PTPRU  10076  1  29563027  29653325  1p35.3  1.594  0.05  0.305  0.073  1.424  0.15  1.248  1.171  1.251  3.076  1.473  0.091  1.228  1.849  3.544  0.687  0.376  0.127  0.785  0.093  0.202  0.647  0.509  0.432  0.698  1.181  1.636  1.684  1.483  1.142  3.698  1.409  0.259  0.195  1.176  2.787  0.368  0.219  0.292  0.795  3.117  1.219  1.212  2.122  1.112  1.439  0.775  1.06  1.659  1.059  0.735  0.94  1.366  0.658  0.675  0.199  2.562  0.379  0.169  0.655  
 
 LOC101928460  101928460  1  29656131  29676626  -  0.085  0  0.171  0  0.09  0  0  0  0.169  0.106  0  0  0  0.082  0.078  0.035  0  0  0  0  0  0.072  0.6  0  0  0  0.059  0  0  0.065  0.178  0  0  0.202  0  0.141  0  0  0  0  0  0  0.031  0  0  0  0.042  0  0  0  0.042  0.088  0.047  0.041  0.052  0  0.138  0  0  0  
 
 LOC101929406  101929406  1  30486798  30510459  -  0  0  0.179  0  0  0  0  0  0.102  0  0  0  0  0  0  0  0  0  0  0.077  0  0.037  0.372  0  0  0  0.079  0  0  0.039  0  0  0  0.16  0  0  0  0  0  0  0  0.321  0.052  0  0.119  0  0.525  0  0  0  0  0.246  0  0  0  0  0  0  0  0  
 
 MATN1  4146  1  31184123  31196432  1p35  0.157  0.086  0.159  0.092  0.059  0.078  0.207  0.132  0.193  0.142  0.263  0.07  0.298  0.054  0.134  0.103  0.074  0.194  0.101  0.098  0.25  0.244  0.464  0.07  0.129  0.214  0.544  0.171  0.188  0.146  0.119  0.106  0.057  0.248  0.113  0.092  0.202  0.054  0.128  0.274  0.064  0.326  0.261  0.121  0.046  0.1  1.419  0.071  0.088  0.216  0.122  0.416  0.124  0.105  0.087  0.173  0.112  0.071  0.085  0.073  
 
 MATN1-AS1  100129196  1  31191618  31199593  1p35.2  0.788  0.18  0.462  0.778  0.682  0.429  0.55  0.33  0.539  0.633  1.103  0.37  0.63  0.559  0.449  0.568  0.496  0.434  0.134  0.295  0.98  0.28  0.856  0.37  0.198  0.557  1.151  1.023  0.562  0.192  1.088  0.88  0.222  0.561  0.437  0.59  0.255  0.732  0.361  0.704  0.697  1.455  1.586  0.38  0.148  0.2  0.826  0.31  0.25  0.462  0.655  1.085  0.465  0.51  0.516  0.21  0.579  0.591  0.086  0.16  
 
 LAPTM5  7805  1  31205314  31230683  1p34  0  1.477  0.131  0  0.153  0  1.534  0.911  1.107  1.501  0.502  0.307  0  0.05  0  0.844  0  0.928  7.252  6.24  1.864  4.512  5.686  4.891  0  3.248  0.122  2.295  0.378  0.099  0.073  0.067  0  0.158  0  0  4.622  3.055  0.164  0.415  0.352  0.696  0.029  0.225  0  0.101  0.407  0  0  0.496  0.065  0.257  0.564  0.071  0.144  0.08  0.863  0.315  0  0.145  
 
 SDC3  9672  1  31342312  31381480  1pter-p22.3  1.303  1.051  2.994  2.854  1.271  2.202  3.681  1.424  4.63  5.385  3.749  1.663  3.254  2.827  2.488  1.046  1.184  1.79  0.119  0.057  2.65  0.425  0.845  0.567  2.869  5.632  6.303  5.203  7.178  4.089  5.818  5.792  4.774  4.132  1.519  2.674  3.808  5.173  2.208  4.24  1.489  3.674  3.499  3.374  2.55  3.741  2.942  2.665  3.805  2.212  2.493  2.25  3.515  2.257  3.264  2.21  4.249  3.146  2.074  4.06  
 
 PUM1  9698  1  31404352  31538564  1p35.2  3.27  3.584  2.451  3.559  3.319  2.228  3.842  3.658  3.134  3.837  4.207  2.874  3.857  4.183  4.103  3.533  3.819  5.046  4.14  3.523  3.21  3.677  2.605  2.82  3.2  3.051  3.83  3.344  3.355  3.157  3.729  3.67  3.146  1.852  2.391  3.452  3.994  3.442  3.181  4.274  3.545  2.091  4.932  3.727  3.319  3.327  3.607  3.257  3.357  4.021  3.624  2.228  3.508  2.031  3.358  3.74  3.751  2.969  3.331  4.071  
 
 NKAIN1  79570  1  31652591  31712734  1p35.2  0.87  0  0.116  0.564  2.596  0  0.152  0.244  0.429  0.157  0.151  0.05  0.081  0.621  0.24  0.052  0.157  0.282  0.107  0.23  0  0.243  0.511  0.041  0  0.205  0.501  0.051  0.09  0.794  0.245  1.284  0.104  0.288  0.432  0.616  0.086  0  0.202  1.266  0.194  0.785  3.785  0.06  0.365  0.282  0.055  0.489  0.4  0.513  0.1  0.626  0  0.136  0.154  0.19  0.387  0.132  0  0.074  
 
 SNRNP40  9410  1  31732414  31769644  1p35.2  3.659  3.366  2.78  3.299  3.382  3.222  4.686  4.057  3.054  4.16  5.049  3.217  3.994  5.544  3.599  4.022  4.249  1.876  5.928  3.617  4.221  3.991  3.022  2.864  3.816  3.046  3.779  2.628  3.283  2.581  4.416  4.285  3.272  1.767  2.568  3.531  4.446  3.596  3.367  4.821  3.568  3.083  4.871  3.975  3.021  3.058  1.348  3.011  3.442  4.926  4.148  2.766  3.717  1.571  3.667  3.452  3.822  3.881  3.388  3.754  
 
 ZCCHC17  51538  1  31769828  31837800  1p35.2  3.33  4.853  3.691  3.457  3.53  2.775  5.217  3.745  3.701  4.837  4.822  3.168  3.349  4.484  2.925  3.731  3.498  5.244  5.117  4.631  3.675  4.088  3.458  2.743  3.349  4.514  5.356  4.459  4.747  2.559  4.892  5.393  4.568  3.569  2.597  3.058  4.115  3.544  3.589  3.686  2.944  2.789  5.178  3.6  2.703  3.61  3.273  3.258  3.646  4.961  4.497  2.055  3.883  2.001  3.742  3.921  4.03  3.802  3.057  3.881  
 
 FABP3  2170  1  31838099  31845923  1p33-p32  0.694  0.538  1.4  0.531  0.561  0.476  1.022  0.564  0.526  0.819  0.711  0.349  0.379  0.791  0.602  0.355  0.288  0  0.646  1.049  0.364  0.553  0.457  0.134  0.357  2.358  2.197  0.743  0.771  0.477  1.124  1.001  0.67  0.24  0.393  0.42  1.269  0.61  0.565  0.349  0.296  0.249  1.501  1.518  0.527  1.451  1.69  0.568  0.46  1.603  0.609  0.259  0.411  0.135  5.178  0.458  2.324  0  0.187  0.441  
 
 SERINC2  347735  1  31882411  31907527  1p35.1  2.005  1.383  2.02  0  6.27  2.418  4.673  4.067  2.153  2.108  2.787  2.899  4.827  4.634  3.91  6.489  5.42  0.141  1.952  0.472  0.617  1.342  3.403  2.809  0.202  3.273  2.395  0.506  1.884  3.219  3.357  3.544  0.466  0.61  2.129  4.671  7.53  1.935  2.124  2.519  5.525  2.406  1.957  2.208  2.728  4.671  3.024  2.175  2.817  2.902  4.85  3.206  4.837  2.853  2.748  1.075  4.303  3.334  4.246  6.013  
 
 LINC01225  149086  1  31971838  31974167  1p35.2  0  0  0.177  0  0.091  0  0  0  0.26  0  0  0.22  0  0  0  0  0  0.434  1.2  0  0  1.165  0.36  0.135  0  0  0.052  0  0  0.107  0  0  0  0.332  0  0  0.178  0  0  0  0  0  0.042  0  0  0  0  0  0  0  0  0.285  0  0.065  0  0  0  0  0  0  
 
 LINC01226  284551  1  31984035  31989846  1p35.2  0  0  0.147  0  0  0  0.056  0.03  0.117  0.028  0  0.05  0  0.016  0  0  0  0.303  1.458  0  0  0.674  0.418  0  0  0  0.073  0  0  0.072  0  0  0  0.181  0  0  0.106  0  0.016  0  0  0.093  0.059  0  0  0.067  0  0  0  0  0  0.067  0  0.032  0  0  0.046  0  0  0  
 
 TINAGL1  64129  1  32042085  32053287  1p35.2  0  1.076  0.248  0.231  1.571  0.203  0.106  0.368  1.566  0.214  4.738  4.649  3.384  2.832  1.785  5.924  6.012  0.355  1.012  0.202  0  0.32  0.522  0.073  0  0.349  0.427  1.719  0.599  0.151  0.215  0.069  0  0.143  0.209  2.861  6.006  3.961  3.804  0.226  4.033  1.391  0.095  0.91  4.86  5.596  3.395  3.595  3.585  3.6  2.275  3.168  4.7  0.328  6.688  4.809  3.068  0.208  3.822  6.059  
 
 HCRTR1  3061  1  32083300  32092919  1p33  0  0  0.201  0  0  0.165  0  0.055  0.153  0.09  0.08  0  0  0.102  0  0.052  0  0  0  0.071  0  0.149  0.449  0  0  0  0.219  0.034  0  0.118  0  0  0  0.104  0.065  0.062  0.141  0  0.086  0  0.061  0.614  0.081  0  0.096  0.036  0  0  0  0.049  0.057  0.574  0.043  0.111  0  0  0  0.094  0  0  
 
 PEF1  553115  1  32095462  32110838  1p34  3.785  1.921  3.938  4.232  3.982  3.297  5.624  4.336  4.092  4.644  5.071  3.683  3.995  4.151  3.833  4.085  4.425  1.326  5.792  3.715  3.714  4.169  4.138  3.507  3.355  3.58  4.908  4.096  4.041  3.208  4.533  4.636  3.957  2.941  3.098  3.475  4.871  3.892  3.649  4.349  3.616  3.874  4.633  3.799  3.339  4.03  3.947  4.022  4.295  5.816  4.654  3.187  4.656  2.944  4.008  4.035  4.569  3.86  3.941  4.312  
 
 COL16A1  1307  1  32117847  32169768  1p35-p34  0.069  0.089  1.717  0.916  0.08  0.254  0.256  3.501  0.166  1.54  0.503  0.033  0.153  0.146  0.052  1.672  0.103  0.315  0.024  0.024  0  0.1  0.618  0.155  0.188  0.762  0.26  2.073  0.219  0.132  1.354  1.77  1.711  0.302  0.119  2.281  1.053  0.637  0.502  0.133  0.061  0.241  0.074  0.054  0.32  0.316  0.035  0.102  0.027  0.922  0.132  0.305  0.313  0.395  0.22  0.385  2.815  0.12  0.114  0.678  
 
 ADGRB2  576  1  32192705  32229664  1p35  0.506  0.035  0.924  2.102  1.847  0.615  1.404  3.177  1.513  2.538  1.433  0.125  0.102  0.855  0.278  0.15  0.103  0.107  0.891  0.076  0  1.35  0.479  0.328  0.51  1.013  0.448  0.225  1.144  1.146  1.341  1.018  0.097  0.221  0.659  2.391  1.654  0.727  0.603  1.749  0.326  1.278  1.79  0.161  0.866  0.527  0.158  0.976  0.31  1.354  1.014  1.055  0.89  0.483  0.632  1.011  1.351  1.276  0.057  1.457  
 
 SPOCD1  90853  1  32256022  32281652  1p35.2  0.18  0.256  2.587  0  1.874  0.565  3.932  3.49  0.21  4.219  1.344  0  0.078  0.335  0.278  0.055  0.038  0.092  0.702  0.036  0.079  0.544  0.44  0  3.607  1.666  1.67  0.079  0.257  0.192  0.324  0.943  0.618  0.21  0  0.638  2.888  2.612  1.052  0.607  0.215  0.286  0.071  0  0  0.894  0.189  0.305  0  0.65  0.79  1.606  1.698  1.292  1.929  0.268  2.228  0.217  0.322  1.441  
 
 PTP4A2  8073  1  32372021  32403988  1p35  3.852  5.888  4.326  5.349  5.721  3.789  5.553  4.954  4.639  5.222  5.286  4.144  3.984  5.566  3.896  4.627  5.013  5.661  7.115  4.662  4.794  6.184  4.299  3.7  4.933  4.127  4.286  3.993  3.945  3.545  4.809  4.583  3.552  2.935  3.607  4.576  5.333  4.941  4.319  5.344  3.611  4.056  5.344  3.683  3.227  4.065  5.65  3.421  4.45  5.11  5.322  3.603  4.489  2.846  4.23  4.534  4.791  4.57  4.583  4.558  
 
 KHDRBS1  10657  1  32479294  32526460  1p32  3.847  5.257  3.699  4.662  4.507  4.21  5.287  5.077  3.96  4.573  5.52  4.084  4.719  4.876  4.103  4.852  4.642  5.96  6.213  4.784  4.551  5.051  4.271  3.893  4.74  3.864  4.079  3.982  3.875  4.007  4.698  5.026  4.338  3.184  3.751  4.353  4.616  4.165  3.967  4.996  4.366  4.255  5.328  4.418  3.973  4.379  3.861  4.421  4.162  5.801  4.802  3.408  4.863  3.259  4.485  4.66  4.506  4.677  4.465  5.189  
 
 TMEM39B  55116  1  32538502  32568467  1p35.1  2.68  0.911  2.368  3.059  2.011  1.788  3.737  3.227  2.718  3.416  3.073  1.902  1.813  2.976  2.352  2.6  2.409  0.522  4.374  2.058  2.062  3.287  3.082  2.177  2.437  1.688  3.675  2.087  2.49  1.925  2.697  2.954  3.006  1.685  1.605  1.572  3.76  2.252  1.954  3.307  2.133  2.607  3.499  1.849  2.064  2.428  2.985  2.311  2.026  3.556  2.455  1.945  2.606  1.441  2.479  2.457  2.451  2.401  2.288  2.368  
 
 KPNA6  23633  1  32573643  32642168  1p35.1  3.032  2.716  3.04  3.719  2.728  2.681  4.061  4.01  3.453  3.858  4.218  2.264  3.592  3.789  3.549  2.962  3.02  1.788  3.915  2.646  2.898  2.847  2.664  2.482  3.194  3.102  3.123  3.34  3.185  2.344  3.715  3.901  2.919  1.787  2.256  3.123  3.958  3.675  2.767  4.147  3.301  2.224  4.131  3.48  2.752  3.393  2.648  3.017  3.519  3.932  3.978  2.232  3.747  2.102  3.142  3.436  3.746  3.266  3.209  3.828  
 
 TXLNA  200081  1  32645344  32663886  1p35.1  2.137  1.118  2.965  3.995  2.933  3.179  4.259  4.205  3.539  4.541  4.738  2.706  3.709  2.344  3.65  3.413  2.911  0.499  4.464  2.286  2.998  3.171  2.782  2.828  3.418  3.616  4.492  3.98  4.31  2.965  4.697  4.38  4.022  2.204  2.455  3.353  4.197  3.874  2.876  4.451  3.083  2.939  4.273  3.29  2.871  3.006  1.844  3.413  3.677  4.448  3.797  2.477  3.259  2.532  2.967  3.612  3.964  3.003  3.591  3.68  
 
 CCDC28B  79140  1  32665986  32670991  1p36.11-p34.2  1.415  0.201  2.477  2.99  2.442  1.116  3.698  3.226  2.314  2.86  1.859  0.322  1.204  1.11  0.704  2.139  2.275  0.284  4.76  0.718  3.098  3.259  1.962  1.638  1.941  1.317  1.978  1.46  0.949  0.785  3.233  2.088  2.192  1.447  0.577  2.077  2.023  1.41  0.913  0.926  0.413  1.508  3.93  2.295  1.707  1.763  1.217  1.139  0.469  2.808  1.025  0.799  1.292  0.356  1.542  1.94  1.824  3.176  1.464  1.748  
 
 IQCC  55721  1  32671235  32674288  1p36.11-p34.2  0.983  0.218  0.781  1.384  1.741  0.93  1.658  1.082  0.894  1.708  1.628  0.858  1.24  1.079  1.69  1.411  1.338  0.261  2.725  0.79  1.878  1.799  1.402  0.722  0.939  1.021  1.26  0.97  1.009  0.696  1.543  1.43  0.916  0.433  0.692  1.26  1.516  1.431  0.651  1.794  1.405  0.53  2.196  1.181  0.852  1.094  1.001  1.066  1.066  2.199  1.727  1.141  1.578  0.548  1.456  1.094  0.989  1.381  0.937  0.801  
 
 DCDC2B  149069  1  32674694  32681797  1p35.1  0.183  0  0.364  0.388  0.328  0.409  0.233  0.446  0.346  0.691  0.611  0.183  0.334  0.252  0.726  0.596  0.349  0  0.287  0.103  0  0.171  0.446  0  0  0.518  0.453  0.51  0.529  0.385  0.327  0.457  0.296  0  0.411  0.36  0.545  0.528  0.122  0.391  0.506  0.188  0.665  0.185  0.211  0.405  0  0.277  0.266  0.442  0.464  0  0.75  0.124  0.536  0.228  0.606  0  0.227  0.298  
 
 TMEM234  56063  1  32681797  32687926  1p36.11-p34.2  1.23  0.094  1.918  2.54  1.255  1.103  2.281  2.085  1.965  1.872  2.202  1.188  1.761  1.392  1.42  2.027  1.697  0  2.469  1.411  1.256  1.67  1.777  1.19  2.047  1.488  1.481  1.542  1.917  0.794  2.698  2.152  1.574  0.893  1.096  2.172  1.631  2.191  1.309  1.594  1.658  2.043  2.461  2.009  0.974  1.623  0.57  1.433  2.019  2.938  1.944  0.907  2.35  0.783  1.98  1.91  1.967  1.849  1.517  1.907  
 
 EIF3I  8668  1  32687970  32697205  1p34.1  5.301  5.992  4.988  5.38  5.442  4.94  7.289  6.057  5.507  6.024  6.945  5.775  5.643  7.107  5.249  5.724  6.101  5.628  7.163  5.918  6.571  5.62  5.612  5.468  5.942  5.158  5.333  6.287  5.598  5.386  6.659  7.316  6.149  4.661  4.598  5.482  6.558  6.09  5.18  6.589  5.577  4.816  6.8  5.67  5.855  5.78  4.415  5.032  5.669  6.951  6.458  4.519  5.849  3.974  5.742  5.803  5.645  5.966  5.492  6.12  
 
 MTMR9LP  339483  1  32697260  32707311  1p35.1  0.205  0.053  0.291  0.709  0.534  0.275  0.908  0.882  0.161  0.915  0.37  0.185  0.087  1.214  0.739  0.355  0.643  0.629  0.173  0.151  0.085  0.382  0.866  0.131  0.111  2.098  2.738  0.843  2.625  0.641  2.551  0.389  0.528  0.456  0.406  0.992  0.474  0.154  0.093  0.414  0.418  0.34  0.537  0.211  0.084  0.081  0.607  0.562  0.39  1.447  0.843  0.98  0.321  0.269  0.521  0.08  0.384  0.31  0.311  0.158  
 
 FAM167B  84734  1  32712817  32714461  1p35.1  0  0  0.429  0.998  0.21  0  0.596  0.279  0.286  0.898  0.305  0  0  0.27  0.194  0.109  0.307  0  0.42  0  0  0.564  0.996  0.122  0  2.071  6.492  2.749  4.169  2.02  4.803  4.058  1.909  3.117  0  0.333  0.209  0  0.494  0  0.146  0.211  0.859  0  0.214  0.162  0  0.256  0  0.664  1.07  0.835  0  0.11  0.274  0  0.253  0  0  0.138  
 
 LCK  3932  1  32716839  32751768  1p34.3  0  0  0.183  0  0.098  0  0.535  0  0.099  0.205  0.081  6.389  0.136  0.132  0  0.075  3.8  1.685  7.536  0  0  6.329  0.271  0.089  0.067  0  0.109  0  0  0.097  0  0  0  0.198  0.081  0  0.089  0  0.085  0  0.144  0.518  0.086  0  0.467  0.328  0.211  0  0  0.674  0.482  0.585  0.158  0.083  0.062  0.072  0  0  0  0  
 
 HDAC1  3065  1  32757707  32799224  1p34  4.438  3.508  3.007  3.309  4.296  2.972  4.444  4.386  3.443  3.977  5.203  4.838  4.751  5.624  4.373  5.047  5.678  3.991  6.092  4.802  4.326  5.344  3.358  2.923  3.968  3.156  4.099  3.603  3.77  2.494  3.793  4.118  3.539  2.461  3.09  3.421  4.419  4.076  3.328  4.763  3.896  3.095  4.638  4.271  3.14  3.981  4.254  3.366  3.996  5.069  4.697  2.268  4.087  2.553  4.05  3.642  4.288  4.033  3.313  3.832  
 
 MARCKSL1  65108  1  32799429  32801840  1p35.1  5.905  2.786  4.396  6.122  5.122  4.561  5.13  4.942  6.278  5.775  6.4  6.395  6.209  6.437  4.358  6.851  6.844  4.505  7.925  2.326  6.27  6.754  3.336  3.567  4.091  6.578  7.547  5.583  6.41  5.496  7.184  7.522  7.272  6.011  4.291  5.183  5.463  4.192  2.806  5.449  2.269  4.02  8.36  5.877  5.204  5.518  4.296  4.986  1.537  7.458  6.352  2.523  4.479  2.094  4.197  5.219  4.554  4.216  4.427  5.462  
 
 FAM229A  100128071  1  32826870  32827844  1p35.1  0.445  0.491  0.564  1.089  0.913  0  0.546  1.19  1.778  1.476  1.274  0.462  0.262  1.134  0.44  0.895  0.779  0.307  2.209  0.691  0.886  1.536  2.203  0.48  0.323  0.268  1.751  0.428  0.853  0.377  1.08  0.82  0.54  0.41  0.22  0.373  0.207  0.447  1.949  0  0.304  2.468  0.98  0.384  0.618  0.79  0.412  1.086  0  2.015  0.293  1.275  0.64  0.44  1.08  0.656  0.474  1.013  0.378  0  
 
 TSSK3  81629  1  32827861  32829924  1p35-p34  0.477  0.156  0.537  0.732  1.269  0.524  0.863  1.629  1.121  2.24  2.466  0.266  0.648  1.05  1.638  0.68  0.965  0.356  1.798  0.681  1.359  1.408  0.859  0.992  0.607  0.996  2.326  1.451  1.692  0.993  1.392  0.958  1.245  0.479  0.513  0.981  1.09  1.113  1.388  1.07  0.87  1.335  1.657  0.849  0.292  0.97  0.079  0.55  1.002  0.427  0.69  1.177  1.59  0.533  0.765  0.458  1.25  1.296  1.214  0.22  
 
 BSDC1  55108  1  32830233  32860062  1p35.1  1.921  0.734  2.015  2.678  2.663  2.099  2.968  3.146  3.083  3.318  3.379  2.19  2.606  2.355  2.804  2.678  2.56  0.881  3.025  0.839  3.002  2.091  2.129  2.09  2.199  2.275  2.853  2.287  2.725  1.731  2.836  2.823  2.317  1.05  1.393  1.92  2.65  2.861  3.122  2.794  2.039  1.79  3.367  2.225  2.125  2.613  1.247  2.301  2.538  3.607  3.235  1.471  2.267  1.722  2.333  2.333  3.071  2.592  2.441  2.577  
 
 ZBTB8B  728116  1  32930657  32953459  1p35.1  0.121  0.064  0.157  0.1  0.042  0.167  0.053  0.105  0.159  0.051  0.115  0.039  0.034  0.231  0.049  0.052  0  0.12  0.037  0.045  0.047  0.104  0.417  0  0.048  0.084  0.121  0.093  0.18  0.258  0.587  0.186  0.078  0.253  0.068  0.042  0.101  0.089  0.046  0.266  0.118  0.135  1.426  0.233  0.214  0.202  0.027  0.291  0.054  0.338  0.194  0.343  0.045  0.062  0.054  0.12  0  0.259  0.034  0.063  
 
 ZBTB8A  653121  1  33004745  33071551  1p35.1  0.768  1.142  0.628  0.865  0.804  0.527  0.739  1.561  0.732  1.072  0.651  0.323  0.536  1.267  0.808  0.43  0.737  1.165  0.991  0.216  0  0.682  0.642  0.219  0.506  0.727  0.645  0.308  0.475  0.477  0.748  0.593  0.252  0.364  0.362  0.695  0.564  0.494  0.833  0.615  0.607  0.36  1.25  0.808  0.376  0.585  0.703  0.564  1.059  1.075  0.74  0.356  0.646  0.31  0.616  0.538  0.793  0.54  0.643  0.692  
 
 ZBTB8OS  339487  1  33086816  33116191  1p35.1  1.918  3.865  1.702  2.004  2.162  2.692  3.379  2.678  1.961  2.632  3.16  2.572  1.918  3.345  1.323  2.362  2.638  5.491  3.652  2.553  2.537  2.514  2.295  2.494  2.279  1.698  2.331  1.666  2.61  1.111  3.399  3.049  2.241  1.192  1.904  2.271  2.17  2.329  3.391  2.282  2.694  1.712  3.047  1.949  1.988  2.568  3.081  1.39  2.233  3.245  2.355  1.489  2.492  1.84  2.743  2.071  2.526  3.232  2.921  2.293  
 
 RBBP4  5928  1  33116748  33151812  1p35.1  2.489  2.056  1.558  2.825  2.397  2.02  2.679  3.009  2.72  3.24  3.883  2.608  2.919  3.981  2.375  3.127  3.139  2.823  5.498  2.566  3.18  3.402  2.84  2.741  2.53  2.106  2.241  2.072  2.641  1.033  3.723  2.81  2.579  1.376  1.834  2.53  3.243  2.627  2.333  3.28  2.081  1.893  3.604  3.22  1.911  2.638  1.932  1.938  2.622  3.674  2.932  1.857  2.464  1.465  2.551  2.657  2.936  2.51  2.626  2.95  
 
 SYNC  81493  1  33145506  33168361  1p35.1  0  1.958  2.968  2.645  1.09  1.976  1.673  3.144  1.066  3.028  1.472  0.065  0.34  0.766  0.165  0.067  0.088  3.231  0  2.72  0.154  1.074  1.629  0  0.248  1.715  0.603  0.419  0.29  0.119  0.866  0.123  0  0.596  0.38  1.317  0.606  0.411  4.103  0.139  0  1.128  0.351  0.351  0.529  1.38  0.081  0.673  1.589  1.259  1.503  0.606  1.579  1.339  1.529  1.443  1.947  0  0.373  1.554  
 
 KIAA1522  57648  1  33207511  33240571  1p35.1  4.217  0.603  2.729  3.15  5.164  1.493  2.848  3.934  3.039  3.529  4.352  3.966  5.268  3.363  4.383  5.383  4.091  0.934  0.28  0.278  2.583  0.47  2.396  2.153  0.157  2.635  3.246  1.853  2.771  2.92  3.453  1.865  1.68  0.794  2.783  3.938  3.332  3.115  4.074  2.883  5.057  2.786  3.777  3.758  3.993  4.889  2.352  3.244  4.887  4.734  4.19  2.984  4.072  3.267  4.152  3.969  4.397  2.767  4.223  3.987  
 
 YARS  8565  1  33240839  33283633  1p35.1  2.833  2.582  4.528  5.178  3.15  3.222  4.894  4.648  4.734  4.283  4.889  4.234  4.025  4.877  3.999  4.256  4.613  2.298  5.986  4.779  4.988  4.384  4.952  3.57  4.129  2.923  4.25  3.485  3.823  4.213  4.417  4.935  3.703  2.916  3.401  3.452  4.382  3.674  5.581  4.468  3.713  3.164  4.65  3.713  3.204  3.937  3.412  3.335  3.406  5.371  4.227  2.852  3.898  3.091  4.166  4.317  4.239  3.977  3.568  4.031  
 
 S100PBP  64766  1  33283042  33324480  1p35.1  1.379  1.611  1.233  1.959  1.319  0.607  1.681  1.909  1.45  1.939  1.895  0.806  1.283  2.204  1.572  1.578  1.433  1.745  3.702  1.488  1.626  1.823  1.777  1.458  0.993  1.307  1.685  1.331  1.299  0.837  1.973  1.494  1.402  1.021  0.873  1.473  2.054  1.648  2.236  2.009  1.304  0.87  2.503  1.316  0.681  1.41  0.997  0.854  1.352  1.877  1.693  0.818  1.272  0.742  1.355  1.502  1.576  1.284  0.993  1.099  
 
 FNDC5  252995  1  33327868  33338093  1p35.1  0.274  0  1.737  0.71  0.285  0.251  0.276  0.842  0.307  0.689  0.659  0.05  0.17  0.213  0.421  0.144  0.123  0.086  0.181  0.184  0.203  0.274  0.592  0.081  0.069  0.141  0.317  0.111  0.068  0.381  0.17  0.229  0.158  0.226  0.19  1.193  0.273  0.242  0.292  0.237  0.132  0.164  0.753  0.204  0.386  0.892  0.824  0.167  0.58  0.103  0.176  0.453  0.107  0.097  0.913  0.257  0.269  0  0.085  0.095  
 
 HPCA  3208  1  33352097  33360247  1p35-p34.2  0.534  0.131  0.245  0.268  0.052  0  0.142  0.159  0.367  0.311  0.299  0.101  0.203  0.26  0.304  0.683  0.158  0.127  0.102  0  0  0.149  0.508  0.073  0  0.313  0.251  0.289  0.125  0.132  0.239  0  0  0.142  0.157  0.326  0.395  0.165  0.158  0.443  0  0.523  1.547  0  0.202  0.067  0.467  0.408  0  1.484  0.412  0.75  0.225  0.637  0.339  0.207  0.505  0.248  0  0  
 
 TMEM54  113452  1  33360195  33366953  1p35-p34  5.4  1.495  4.326  4.285  5.452  4.258  5.659  3.929  4.608  4.128  3.007  6.306  6.5  4.971  5.733  7.005  5.875  0  0.708  0.161  1.435  0.493  0.669  0.669  4.064  4.544  4.65  4.404  3.921  5.033  5.689  3.432  1.023  0.91  3.22  4.687  6.029  5.531  4.126  4.765  5.864  2.603  6.341  1.485  4.129  4.627  3.687  5.494  1.822  6.816  4.039  4.163  5.937  3.853  5.981  5.867  4.972  5.736  6.154  5.532  
 
 RNF19B  127544  1  33402046  33430414  1p35.1  1.281  1.169  1.472  3.728  1.302  1.266  2.335  2.11  2.058  1.867  2.736  1.861  2.696  2.164  2.19  3.005  3.351  1.059  2.283  0.501  1.233  1.209  1.567  1.086  1.526  2.411  2.971  2.637  2.987  2.865  3.984  3.063  2.234  1.448  1.012  2.341  2.856  2.237  2.534  2.628  1.914  1.639  2.932  1.544  1.189  1.853  0.987  1.398  1.817  2.296  2.821  1.561  1.929  1.411  1.762  2.547  1.896  1.592  1.957  2.213  
 
 AK2  204  1  33473540  33502512  1p34  3.525  4.556  3.708  4.575  4.052  3.356  4.47  3.944  3.701  4.116  4.741  4.312  4.506  4.809  4.222  4.602  4.628  5.499  5.604  5.729  4.943  4.101  4.444  4.127  4.254  3.224  4.613  3.767  4.626  3.416  5.253  4.962  4.383  3.323  3.205  3.555  4.172  3.715  4.98  4.307  3.723  3.235  4.925  3.825  3.902  4.386  4.559  3.575  3.604  5.268  4.209  2.856  4.036  2.755  4.027  3.977  3.96  4.418  3.716  4.041  
 
 AZIN2  113451  1  33546713  33586132  1p35.1  0.379  0.447  0.601  2.206  0.327  0.413  1.416  0.92  1.812  1.131  1.452  0.118  0.405  1.14  0.895  0.39  0.537  0.137  1.152  0.296  0  0.503  0.702  0  0.485  0.539  1.178  1.224  0.975  0.955  2.477  1.29  0.735  0.421  0.367  0.607  1.907  1.918  1.106  0.657  0.504  0.636  1.131  0.234  0.414  0.674  0.911  0.503  0.373  1.073  0.774  0.847  0.831  0.61  1.687  1.183  0.695  1.684  0.78  0.562  
 
 TRIM62  55223  1  33611002  33647671  1p35.1  2.043  0.269  1.812  1.851  1.722  0.592  1.956  1.69  1.42  1.915  2.113  0.553  1.212  1.702  1.643  0.82  1.039  0.358  1.855  0.647  0.508  1.48  0.896  0.994  1.116  1.836  1.883  2.486  2.33  1.162  3.385  2.109  1.691  0.681  0.331  0.839  1.681  1.937  1.321  1.682  1.624  0.86  1.595  0.798  0.969  1.374  0.282  1.052  1.258  1.163  1.614  1.632  2.001  0.758  1.464  0.868  1.477  1.704  1.016  1.596  
 
 ZNF362  149076  1  33722173  33766320  1p35.1  2.045  0.325  2.097  2.54  1.689  1.967  2.108  3.348  2.847  3.013  2.658  0.696  0.881  1.198  1.859  0.544  0.723  0.298  3.385  1.323  0.133  2.64  0.967  0.682  1.628  2.183  2.703  2.257  2.106  1.858  3.578  2.808  2.531  1.575  1.781  2.697  2.001  0.861  2.445  2.465  3.523  1.512  4.052  1.94  1.213  2.147  1.681  1.278  1.976  2.096  2.049  1.711  2.053  0.592  2.403  0.961  1.776  1.445  1.64  2.268  
 
 A3GALT2  127550  1  33772366  33786699  1p35.1  0  0.093  0  0  0.205  0.257  0.101  0.117  0.145  0.168  0  0.186  0  0.063  0  0.14  0.118  0.257  0  0.215  0  0.669  0.412  0  0  0  0.235  0.086  0  0.258  0.193  0  0  0.282  0  0.163  0  0  0.265  0  0  1.629  0.457  0  0  0.139  0.092  0  0  0  0  2.073  0  0.291  0.332  0.136  0  0  0  0  
 
 PHC2  1912  1  33789223  33841194  1p34.3  1.929  1.965  3.578  5.134  2.479  3.528  4.864  4.708  4.758  5.126  5.04  4.421  3.836  4.106  3.272  4.463  5.698  0.807  4.81  2.442  3.083  3.82  2.814  2.729  4.97  4.008  4.912  4.5  4.291  3.278  5.513  5.337  4.486  3.197  2.502  3.697  5.157  5.288  4.741  3.686  3.262  3.146  4.388  2.985  2.689  3.427  4.732  2.82  3.756  4.81  4.795  3.315  4.026  2.264  4.152  4.13  4.15  4.632  4.142  4.72  
 
 LOC101929464  101929464  1  33815952  33828846  1p35.1  0  0.755  0  0  0  0  0  0  0  0.191  0.29  0  0.456  0.419  0  0  0  0  0  0  0  0  0  0  0  0.225  0.646  0.301  0.246  0  0.64  0.535  0  0  0  0  0.322  0.551  0  0.598  0.332  0  0.176  0  0  0  0  0  0  0  0.349  0  0  0.31  0  0  0  0  0  0  
 
 ZSCAN20  7579  1  33938231  33961995  1p34.3  0.296  0.291  0.51  0.609  0.275  0.226  0.688  0.613  0.615  0.784  0.962  0.251  0.663  0.781  0.854  0.428  0.364  0.4  0.361  0.47  0.246  0.264  0.506  0.163  0.378  0.454  0.65  0.443  0.637  0.282  0.929  0.516  0.539  0.333  0.169  0.366  0.828  0.627  0.599  0.793  0.336  0.243  0.95  0.433  0.654  0.418  1.279  0.375  0.382  0.415  0.418  0.342  0.555  0.219  0.419  0.361  0.67  0.242  0.431  0.546  
 
 CSMD2  114784  1  33979598  34631443  1p34.3  0.037  0.02  0.123  0.038  0.091  0.112  1.043  0.03  0.11  0.048  0.444  0.19  0.013  0.061  0.012  0.031  0.042  0.085  0.015  0.067  0.019  0.145  0.354  0.02  0.015  0.026  0.133  0.025  0.019  0.09  0.014  0.019  0  0.138  0.015  0.014  0  0  1.636  0.048  0.014  0.308  0.069  0.014  0.035  0.042  0.127  0.022  0  0.011  1.877  0.449  0.021  0.082  0.052  0.047  0.021  0.014  0.011  0.01  
 
 HMGB4  127540  1  34326075  34330392  1p35.1  0  0  0  0  0  0  0  0  0.251  0  0  0  0  0  0  0  0  0  0  0  0  0.112  0.322  0  0  0  0.072  0  0  0.105  0  0  0  0.218  0  0  0  0  0.136  0  0  0  0  0  0  0  0  0  0  0  0.142  0.965  0  0.176  0  0  0  0  0  0  
 
 CSMD2-AS1  402779  1  34334556  34351059  -  0  0  0  0  0  0  0  0  0  0  0  0  0  0  0  0  0  0  0  0  0  0.082  0  0  0  0  0.025  0  0  0.039  0  0  0  0  0  0  0  0  0.04  0  0  0  0  0  0  0  0  0  0  0  0.085  0  0  0  0  0  0  0  0  0  
 
 C1orf94  84970  1  34632483  34684731  1p34.3  0  0  0.173  0  0  0.082  1.117  0.035  0.145  0  0.183  0.124  0  0.037  0  0.057  0  0.079  0.058  0.059  0  0.194  0.53  0  0  0  0.143  0  0  0.123  0  0  0  0.198  0  0  0  0  0.103  0  0  0.438  0.054  0  0.078  0.096  0.058  0  0  0  0.827  0.499  0  0.118  0.04  0  0  0  0  0  
 
 GJB5  2709  1  35220647  35224113  1p35.1  0  0  0.175  0  0.828  0  0  0  0.107  0.143  0.084  1.002  0.27  1.901  0.106  0  0.854  0.221  0  0  0  0.24  0.458  0  0  0  0.103  0  0  0.115  0  0  0  0.18  0  0  0  0  0.126  0  2.63  0.284  0.031  0.322  0.21  0.14  0  0  0.217  0  0.763  0.505  0  0.188  0.103  0  0  0  0.166  0  
 
 GJB4  127534  1  35225341  35229325  1p34.3  0  0.053  0.142  0.132  0.072  0  0  0.025  0.166  0.152  0  1.293  0.176  0.304  0.077  0.112  1.091  0.069  0.045  0.058  0  0.189  0.6  0  0  0  0.103  0.034  0  0.096  0  0.046  0  0.239  0  0  0  0  0.066  0  0.45  0.341  0.038  0.584  0.077  0.067  0.018  0  0.344  0.081  0.099  0.562  0.032  0.122  0.109  0  0  0  0.084  0  
 
 GJB3  2707  1  35246789  35251967  1p34  0.328  0.073  0.149  0.17  0.229  0  0  0.11  0.206  0.367  0.043  3.938  2.529  2.919  2.651  3.337  4.355  0  0.06  0.111  0  0.243  0.454  0.105  0  0  0.124  0  0  0.108  0.272  0  0  0.174  0.087  1.093  0.123  0.134  0.2  0.977  4.463  0.482  0.086  1.363  1.554  2.242  0.903  1.685  2.598  3.061  3.416  1.754  0  0.188  0.722  0.108  0.136  0.085  2.405  0.535  
 
 GJA4  2701  1  35258598  35261348  1p35.1  0  0  0.197  0  0.059  0.097  0  0.04  0.16  0.475  0.043  0.059  0.086  0.113  0.222  0.095  0.163  0.216  0  0.162  0  0.233  0.699  0  0  0  0.267  0  0.163  0.221  0  0  0  0.16  0.16  0  0  0  0.212  0  0.145  0.396  0.118  0  0  0.898  0.085  0.12  0.122  0  0.117  0.723  0.096  0.245  0.081  0  0.092  0  0  0  
 
 SMIM12  113444  1  35315962  35325417  1p34.3  1.99  2.139  1.724  2.083  1.983  1.391  2.774  2.142  1.934  2.346  2.427  1.716  2.199  2.425  1.579  1.871  1.961  1.422  3.202  2.783  2.133  2.019  2.337  1.86  1.947  1.741  2.395  1.755  2.036  1.797  2.667  2.679  2  1.302  1.485  1.675  1.958  2.013  2.857  1.869  1.468  1.528  3.029  1.554  2.214  1.925  1.474  1.779  2.123  3.029  2.356  1.211  2.327  1.462  2.206  1.676  1.94  2.041  2.499  1.998  
 
 DLGAP3  58512  1  35331036  35395186  1p35.3-p34.1  0.447  0.08  0.148  0.194  0.122  0.098  0.034  0.084  0.142  0.202  0.068  0.146  0.075  0.101  0.063  0.09  0.199  0.261  0.152  0.21  0  0.255  0.493  0.075  0.048  0.058  0.376  0.046  0.087  0.316  0.128  0.041  0.043  0.212  0.19  0.044  0.09  0  0.141  0.941  0.064  0.513  0.457  0.155  0.314  0.598  0.122  0.037  0.08  0.069  0.087  1.095  0.068  0.403  0.161  0.088  0.054  0.213  0.439  0  
 
 LOC653160  653160  1  35441299  35444307  1p34.3  0.285  0.29  0.355  0.314  0.302  0.206  0.568  0.905  0.627  1.091  0.779  0.325  0.651  0.937  0.687  0.688  0.239  0.446  1.093  0.282  0.185  0.815  0.621  0.231  0.35  0.565  0.618  0.251  0.294  0.151  0.665  0.401  0.235  0.18  0.287  0.476  0.519  0.567  0.907  0.218  0.397  1.19  0.855  0.16  0.188  0.179  0.693  0.254  0.353  0.408  0.393  1.208  0.659  0.433  1.028  0.51  0.577  0.267  1.214  0.257  
 
 ZMYM6NB  100506144  1  35447126  35450948  1p34.3  2.344  5.156  2.335  2.344  2.425  2.256  3.817  3.402  3.006  3.919  3.013  3.448  3.085  3.974  2.681  3.6  3.411  4.885  5.046  3.369  2.845  3.216  2.141  1.272  4.327  2.831  2.733  1.944  2.462  1.562  3.983  3.525  2.543  2.927  2.501  3.216  3.361  3.371  5.487  2.701  2.878  2.04  4.671  3.209  2.301  2.449  4.05  2.05  2.887  4.291  2.7  2.686  3.606  3.18  4.531  3.952  3.752  3.67  4.578  4.049  
 
 ZMYM6  9204  1  35451766  35497569  1p34.2  0.542  1.013  0.569  0.738  0.887  0.563  0.929  0.957  0.947  1.124  1.191  0.535  0.898  0.991  0.652  0.909  1.117  1.349  1.695  1.134  0.905  1.028  0.889  0.485  0.58  0.775  0.689  0.593  0.8  0.248  1.179  0.991  0.487  0.385  0.574  0.973  0.825  0.896  1.223  1.003  0.638  0.576  1.325  0.7  0.795  0.833  0.368  0.534  0.837  1.44  1.366  0.407  0.654  0.595  0.91  0.852  1.044  0.577  0.941  0.878  
 
 ZMYM1  79830  1  35525386  35581459  1p34.3  1.187  2.314  0.614  1.217  0.847  0.77  1.012  1.069  0.661  1.348  1.873  0.633  1.025  2.131  1.257  1.1  1.041  1.641  1.55  0.641  0.939  0.954  0.141  0.084  1.191  0.868  0.813  0.851  1.107  0.696  1.556  1.338  0.609  0.431  0.515  1.234  1.602  1.339  1.58  1.941  0.714  0.495  2.359  1.14  1.041  1.274  1.016  0.857  1.269  1.315  1.534  0.743  1.163  0.389  0.725  1.043  1.138  0.931  1.146  1.312  
 
 SFPQ  6421  1  35649200  35658743  1p34.3  4.319  3.884  3.269  3.837  3.965  4.141  4.487  4.898  3.954  4.415  5.085  3.896  4.325  4.879  3.995  4.293  4.05  4.57  5.937  4.326  4.882  4.857  3.651  3.884  4.452  3.658  3.823  4.151  3.999  3.338  4.817  4.89  4.32  3.325  4.078  4.455  4.4  3.885  4.271  4.706  3.992  4.429  4.646  4.495  4.234  4.165  3.176  4.274  4.256  5.487  4.205  3.754  4.477  3.562  4.299  4.29  4.295  4.196  4.486  4.971  
 
 ZMYM4  9202  1  35734567  35887545  1p32-p34  1.597  2.065  1.464  1.634  1.482  1.118  1.909  2.189  1.786  1.919  2.679  0.906  1.997  2.548  2.052  1.669  1.56  2.117  2.718  1.842  2.303  2.008  1.124  1.108  1.672  1.617  1.138  1.783  1.596  1.109  2.166  2.063  1.25  0.68  1.237  2.262  2.103  2.039  2.69  2.696  1.895  0.953  2.87  1.983  1.534  1.496  0.873  1.496  1.907  2.473  2.315  0.906  1.557  1.205  1.861  1.968  2.263  1.707  1.927  2.527  
 
 KIAA0319L  79932  1  35899090  36023037  1p34.2  2.259  1.935  1.658  2.718  3.024  1.675  3.092  3.339  3.218  3.333  3.13  2.53  2.561  2.508  2.293  2.614  2.509  1.612  2.982  1.762  2.296  1.743  1.96  1.645  2.013  2.975  2.715  3.314  2.871  2.218  3.557  2.787  2.7  1.618  1.629  2.827  3.129  3.541  3.319  3.149  2.799  1.777  3.113  2.406  1.971  2.536  2.165  1.697  2.16  2.982  2.961  1.473  2.784  1.724  3.012  2.918  3.005  1.971  2.989  3.187  
 
 NCDN  23154  1  36023392  36032380  1p34.3  2.493  0.315  2.654  2.554  2.513  1.963  3.751  3.412  2.599  3.488  2.887  2.234  2.368  2.038  2.858  2.491  1.935  0.41  3.45  2.404  2.415  2.448  2.174  2.544  3.114  2.563  3.576  3.645  3.329  2.542  3.664  3.832  3.671  2.374  1.729  1.98  3.185  2.798  3.136  3.313  2.388  2.101  3.897  1.472  2.489  3.091  3.993  2.62  1.574  3.42  3.174  2.182  3.014  2.348  1.93  2.651  2.353  2.458  2.782  2.375  
 
 TFAP2E  339488  1  36038970  36060927  1p34.3  0.157  0.245  0.195  0.172  0.193  0.136  0.281  0.436  0.284  0.559  0.509  0.062  0.164  0.616  0.262  0.176  0.092  0.187  1.277  0.259  0  0.404  0.228  0.146  0.163  0.179  0.966  0.292  0.196  0.111  0.144  0.23  0.329  0.167  0.362  0.136  0.246  0.205  0.465  0.271  0.208  0.588  0.584  0.128  0.25  0.255  0.519  0.196  0.201  0.35  0.245  0.866  0.193  0.194  0.211  0.079  0.215  0.069  0.185  0.123  
 
 PSMB2  5690  1  36065142  36107445  1p34.2  2.962  4.05  2.867  3.418  3.49  3.752  5.194  4.271  2.771  4.13  5.251  3.913  4.068  4.824  3.269  4.423  4.089  4.184  5.883  4.737  4.546  4.74  3.253  3.58  3.892  2.886  3.991  3.677  3.602  2.491  4.523  4.248  3.103  1.991  3.23  3.252  4.328  3.777  5.404  4.374  3.724  3.201  4.509  3.655  3.326  4.119  3.523  3.394  3.925  5.067  4.119  2.968  4.549  3.76  4.033  4.303  4.192  4.395  4.472  4.212  
 
 C1orf216  127703  1  36179476  36184790  1p34.3  0.987  0.226  1.641  1.59  0.944  1.34  2.69  2.533  2.418  2.414  2.39  1.114  1.753  1.634  2.736  1.736  0.567  0.135  2.782  1.121  1.669  0.872  1.328  1.127  1.972  2.604  2.932  3.211  2.711  2.571  3.025  2.709  2.618  1.286  0.903  1.605  2.597  2.279  2.097  2.448  1.188  1.251  2.652  1.202  0.887  0.998  0.66  1.944  1.043  3.405  1.778  1.015  1.769  0.965  1.2  1.23  1.509  1.268  2.094  1.586  
 
 CLSPN  63967  1  36197712  36235551  1p34.2  1.265  3.012  1.095  1.488  1.17  1.868  1.94  2.027  0.516  2.181  2.462  1.154  1.771  2.176  1.823  1.709  1.278  2.771  2.768  1.474  2.32  2.31  1.127  1.092  1.742  1.508  1.559  1.798  1.505  1.207  2.536  1.924  2.062  0.92  1.324  1.414  2.418  1.111  1.963  2.401  1.75  1.282  2.572  1.713  1.282  1.879  1.406  1.751  2.289  1.746  2.008  1.244  1.999  0.715  1.668  1.368  2.105  1.774  2.331  1.917  
 
 AGO4  192670  1  36273827  36323490  1p34  0.454  0.687  0.508  1.114  0.585  0.54  0.587  0.965  0.73  0.925  0.841  0.399  0.612  0.9  0.855  0.881  0.764  0.712  1.819  1.053  0.574  0.967  0.705  0.192  0.618  0.915  0.852  0.675  0.949  0.614  1.203  0.683  0.59  0.517  0.286  0.832  0.774  1.224  0.901  0.66  0.526  0.546  1.669  0.561  0.218  0.588  0.48  0.381  1.022  0.804  1.125  0.693  0.58  0.335  0.69  0.57  0.994  0.655  0.745  0.748  
 
 AGO1  26523  1  36348809  36389899  1p34.3  1.977  1.184  1.621  1.005  1.789  1.68  2.685  2.404  2.292  2.595  3.499  1.724  2.312  2.406  2.932  1.801  1.586  1.614  2.326  1.823  2.96  2.417  0.986  1.401  2.058  2.165  3.36  2.485  2.756  2.045  3.298  2.735  3.018  1.229  1.371  2.383  2.904  2.251  2.336  3.085  2.206  1.2  3.109  1.924  2.008  1.841  0.957  1.711  2.374  1.462  2.721  1.463  2.223  1.104  1.446  2.096  2.357  2.313  2.301  2.461  
 
 AGO3  192669  1  36396682  36522063  1p34  1.608  2.029  1.137  0.873  1.439  0.925  2.296  2.442  1.095  1.91  2.718  0.814  1.783  2.844  1.873  1.364  1.169  1.869  2.248  1.857  1.492  1.793  1.085  1.218  1.234  0.986  2.843  1.49  1.373  0.8  1.997  2.125  1.944  0.649  0.895  1.788  2.007  1.84  2.516  1.892  1.529  0.714  3.188  1.361  1.009  1.599  1.163  1.333  1.591  1.3  2.12  0.98  1.798  0.726  1.281  1.546  1.972  1.354  2.531  1.494  
 
 TEKT2  27285  1  36549675  36553876  1p34.3  0  0  0.106  0.282  0.061  0  0  0.044  0.192  0  0.16  0  0  0.045  0  0  0  0  0.068  0.078  0.139  0.119  0.25  0  0  0  0.141  0  0  0.069  0  0  0  0.132  0  0  0.098  0  0.093  0  0  0.072  0.135  0  0.231  0.117  0  0  0  0.615  0  0.454  0  0.055  0.065  0  0.061  0  0  0  
 
 ADPRHL2  54936  1  36554452  36559533  1p34.3  2.299  1.389  2.877  3.813  3.134  2.538  3.816  3.603  3.165  3.287  4.189  2.649  3.288  2.907  2.778  4.19  3.332  0.404  4.802  2.567  2.793  3.124  2.988  2.869  2.619  2.406  3.928  3.166  3.075  2.471  3.566  3.249  3.464  2.185  2.027  2.333  3.54  2.75  3.8  3.388  2.791  3.102  4.15  2.654  2.558  3.195  1.729  2.61  3.207  4.283  3.552  2.213  3.665  2.353  3.418  2.975  4.119  3.253  4.347  3.31  
 
 COL8A2  1296  1  36560836  36590685  1p34.2  0.125  0.033  0.865  0.116  0.106  0.088  0.121  1.312  0.294  0.415  0.216  0.094  0.103  0.121  0.145  0.116  0.136  0.11  0.093  0.102  0.071  0.218  0.636  0.047  0.093  0.225  0.371  0.109  0.099  0.271  0.07  0.098  0.086  0.204  0.126  0.077  0.198  0.389  0.291  0.43  0.069  0.448  0.168  0.083  0.148  0.339  0.055  0.196  0.134  0.082  0.144  0.586  0.154  0.19  0.356  0.191  0.242  0.068  0.194  0.106  
 
 TRAPPC3  27095  1  36602169  36621654  1p34.3  3.523  4.13  3.727  4.394  3.613  3.685  4.643  4.387  3.822  4.479  5.023  3.665  3.917  4.582  3.359  4.257  3.777  3.376  5.466  5.059  3.722  4.836  4.079  3.571  3.964  2.93  4.185  3.007  3.891  2.947  4.494  4.409  3.214  2.372  3.546  3.37  3.912  3.927  6.461  3.839  3.158  3.551  5.242  3.38  3.019  3.775  4.267  2.972  3.625  4.935  4.093  2.505  4.082  3.764  4.488  3.943  3.784  3.891  5.087  3.953  
 
 MAP7D1  55700  1  36621565  36646451  1p34.3  1.714  1.2  3.591  4.146  2.562  3.703  5.381  5.26  3.702  4.646  5.19  2.139  3.445  3.2  2.431  2.787  1.998  0.272  5.033  2.932  2.962  3.748  2.558  2.672  4.458  3.02  4.326  3.294  3.707  3.17  4.53  4.609  3.838  2.068  3.194  3.205  4.719  4.062  5.061  4.598  3.642  3.715  5.502  3.204  3.077  3.826  3.808  4.125  3.354  4.981  3.615  3.191  4.38  3.753  4.036  3.983  4.07  4.299  4.959  4.424  
 
 THRAP3  9967  1  36690016  36770957  1p34.3  3.67  3.423  2.66  3.017  3.4  2.998  4.16  4.02  3.174  4.05  4.674  2.948  3.462  5.078  4.036  4.018  3.498  2.409  4.027  3.942  3.849  4.26  3.053  3.127  3.525  3.301  3.519  3.18  3.525  3.22  3.792  3.821  3.562  2.313  3.097  3.358  3.749  3.185  3.719  4.133  3.737  3.265  3.909  4.415  3.091  3.447  3.72  3.361  3.892  3.569  3.7  3.075  3.879  3.04  3.811  3.719  3.902  3.38  4.38  4.183  
 
 SH3D21  79729  1  36771993  36786948  1p34.3  0.606  0.102  0.867  0.584  0.333  0.646  0.195  0.801  0.23  0.765  1.346  1.319  1.654  0.48  0.856  0.769  0.963  0.08  0.59  0.139  0.404  0.461  0.459  0.129  0.445  1.062  0.687  0.355  1.44  0.26  1.707  0.616  0.53  0.105  0.221  0.321  0.749  0.988  1.592  1.005  0.58  0.659  0.875  1.682  0.946  1.137  0.595  0.432  0.67  0.16  1.439  0.987  0.416  0.366  1.512  1.327  2.275  1.325  2.247  0.919  
 
 EVA1B  55194  1  36787630  36789755  1p34.3  1.136  0.161  3.403  4.113  0.373  2.528  2.226  3.519  3.691  1.613  1.961  0.854  1.139  2.551  1.068  1.494  0.7  0.59  1.301  1.265  1.497  0.918  1.9  0.88  3.187  2.206  2.921  0.65  1.903  0.587  3.294  2.159  1.453  1.345  1.889  0.521  2.592  4.322  4.667  2.188  1.204  2.548  2.823  2.713  1.621  1.914  1.377  1.573  1.46  2.58  2.022  2.029  2.505  2.035  3.828  3.602  2.601  4.448  2.759  2.289  
 
 STK40  83931  1  36805219  36851528  1p34.3  1.943  2.11  2.12  3.145  1.956  2.23  2.571  3.506  3.462  2.764  3.734  3.101  3.443  1.875  2.911  2.223  2.672  1.214  4.121  1.859  4.029  2.601  2.997  2.731  2.622  2.369  3.419  2.857  2.977  2.185  3.42  3.31  3.678  1.87  1.069  2.313  3.08  3.596  3.883  3.898  3.756  2.251  2.454  2.702  1.573  2.502  3.052  2.382  2.393  3.028  2.35  1.264  2.783  2.005  3.501  2.839  3.152  4.085  3.548  3  
 
 LSM10  84967  1  36859020  36863560  1p34.3  3.596  3.042  3.79  5.065  3.895  3.853  5.813  4.458  4.715  4.681  5.269  3.506  3.607  4.856  3.367  3.762  3.573  1.854  5.713  4.095  4.332  4.283  4.97  4.166  4.518  3.521  4.443  3.466  4.201  3.791  5.251  5.42  3.84  3.214  3.445  4.288  4.692  4.089  6.333  4.319  3.698  4.301  5.565  3.606  2.903  3.64  3.734  3.358  4.384  5.417  4.417  2.822  4.383  3.362  4.371  4.251  3.107  4.956  4.318  3.802  
 
 OSCP1  127700  1  36883506  36916086  1p34.3  1.253  1.368  1.155  1.054  1.922  0.695  1.67  1.375  1.549  1.735  1.825  0.78  1.112  1.557  0.919  1.062  0.858  0.574  0.88  0.266  0  0.871  0.78  0  0.661  0.818  0.778  0.679  0.88  0.561  1.415  2.378  0.395  0.343  0.672  1.188  1.216  1.016  2.179  1.357  1.311  1.251  1.777  0.848  0.417  0.875  0.892  0.786  1.064  1.372  1.151  0.59  1.153  0.766  1.356  1.09  0.425  1.08  1.235  1.418  
 
 MRPS15  64960  1  36921361  36930040  1p34.3  4.156  5.391  3.819  4.754  4.953  4.575  5.128  5.153  4.186  4.867  5.628  4.651  4.712  5.633  4.138  5.221  5.323  4.891  6.664  6.018  5.391  5.489  4.57  4.58  5.119  3.562  3.832  3.422  4.11  3.222  5.641  5.614  3.786  2.702  3.991  4.544  4.766  4.131  6.815  4.455  5.03  4.703  5.438  4.74  4.005  4.84  4.303  4.242  5.044  6.52  4.668  4.196  4.692  3.869  5.318  4.518  4.263  5.642  6.099  4.771  
 
 CSF3R  1441  1  36931643  36948915  1p35-p34.3  0  0  0.131  0  0.029  0  0  0  0.066  0.028  0  0.035  0  0.029  0  0.034  0  0.05  0.039  1.882  0  0.25  0.346  0  0  0.058  0.124  0.197  0  0.077  0.067  0  0  0.152  0  0  0  0  0.042  0  0.085  0.297  0.048  0.065  0  0.026  0.049  0  0  0  0.032  0.277  0.033  0.05  0.03  0  0  0  0  0  
 
 GRIK3  2899  1  37261127  37499844  1p34.3  0.292  0.042  0.169  0.052  1.837  0.033  0.027  0.047  0.137  2.707  0.029  0.054  0.026  0.034  0  0.036  0.038  0.069  0.011  0.037  0  0.128  0.481  0.016  0.024  0.018  0.149  0.023  0.021  0.105  0  0.06  0  0.143  0.021  0.025  0.011  0  0.055  0.459  0.015  0.235  0.189  0.014  0.042  0.03  0.049  0.015  0  0.013  0.025  0.421  0.022  0.096  0.045  0.041  0  0.025  0  0.022  
 
 LINC01137  728431  1  37920479  37940044  1p34.3  0.317  0.143  0.823  0.713  1.72  0.388  2.41  1.805  1.052  1.165  1.501  0.815  0.761  0.155  0.398  1.038  0.56  0.221  1.22  0.285  0  0.473  1.032  0.167  0.979  0.48  0.814  0.343  0.448  0.713  0.486  0.691  0.362  0.375  1.786  1.379  0.926  0.297  1.055  0.52  0.999  1.372  0.449  0.193  0  0.46  0.983  0.147  1  0.695  0.684  0.666  0.662  0.992  1.067  0.37  0.772  0.487  0.284  1.249  
 
 ZC3H12A  80149  1  37940118  37949978  1p34.3  1.831  1.073  1.36  1.511  2.579  1.208  2.71  3.008  0.884  1.642  2.493  2.561  2.198  2.14  1.764  2.806  1.861  0.956  2.018  3.421  1.491  2.011  1.103  1.225  3.117  1.504  2.179  1.511  1.497  2.286  1.693  1.739  1.589  0.439  2.477  1.8  3.256  2.166  3.242  1.843  2.196  2.09  2.239  1.588  1.341  2.591  3.029  0.848  1.602  2.02  1.635  1.454  2.269  1.723  2.245  1.794  2.997  2.169  3.627  2.192  
 
 MEAF6  64769  1  37955560  37980420  1p35.3-p33  1.249  1.809  1.237  1.772  2.294  1.273  1.88  1.966  1.592  1.958  2.136  0.98  1.539  1.989  1.234  1.665  1.945  2.101  3.621  1.785  1.816  2.177  2.074  1.087  1.656  1.512  1.736  1.157  1.412  0.862  2.476  1.772  1.441  1.089  0.891  1.698  1.598  1.243  2.811  1.914  1.595  1.114  2.535  1.462  0.632  1.467  0.867  1.084  1.387  2.948  2.131  0.832  1.284  1.188  1.518  1.499  1.609  2.031  2.421  1.967  
 
 SNIP1  79753  1  38000049  38019945  1p34.3  1.049  1.599  0.845  1.234  1.141  0.904  1.419  1.623  1.248  1.43  1.807  0.792  0.959  1.586  1.089  1.339  1.259  1.367  2.213  1.311  1.124  1.273  1.156  0.857  1.179  0.88  1.15  1.124  1.043  0.263  1.302  1.363  0.958  0.926  0.713  0.876  1.159  1.039  1.895  1.325  0.888  0.782  2.081  0.892  0.957  0.93  0.996  0.802  0.916  1.665  1.513  0.837  1.301  0.694  0.997  1.242  1.115  1.316  1.888  1.211  
 
 DNALI1  7802  1  38022519  38032458  1p35.1  0.275  0.05  0.129  0  3.466  0.286  1.649  0.082  0.153  0.134  0.233  0.062  0.088  0.14  0.098  0.112  0.045  0.131  0.095  0.063  0.104  0.124  0.357  0.043  0.04  0.135  0.121  0.08  0.064  0.062  0.047  0.078  0.107  0.239  0  0.555  0.279  0.077  0.118  0.119  0  0.031  0.194  0.152  0.139  0.091  0.612  0.943  0.191  1.876  0.063  0.207  0.04  0.042  0.424  0.449  0.098  0.122  0.24  0.349  
 
 GNL2  29889  1  38032412  38061586  1p34.3  2.621  3.443  2.407  2.802  2.743  2.754  3.133  3.604  2.537  3.328  3.878  2.264  2.536  3.478  2.636  3.137  2.849  3.875  4.351  3.321  3.93  2.942  2.76  2.734  3.02  2.678  2.596  2.924  2.759  1.962  3.878  3.633  2.836  1.892  2.115  2.923  3.19  2.733  3.717  3.747  2.771  2.046  3.783  3.07  2.821  3.086  1.037  2.294  2.788  3.99  3.517  1.879  2.852  2.059  2.872  2.631  3.206  2.345  3.623  2.849  
 
 RSPO1  284654  1  38076950  38100595  1p34.3  1.001  1.152  1.312  2.24  1.525  0.266  0.568  0.252  0.798  0.565  0.51  0.272  0.525  0.943  0.345  1.212  0.624  1.164  0.859  1.938  0.226  1.823  1.348  0.932  0.214  0.768  1.814  0.685  0.994  1.622  0.451  0.535  0.357  1.108  1.226  0.376  0.34  0.529  1.608  0.579  0.264  1.261  1.795  0.351  0.507  0.481  2.463  0.237  0.155  0.314  0.265  0.859  0.869  0.641  1.352  0.533  0.859  0.327  0.781  0.184  
 
 C1orf109  54955  1  38147241  38156267  1p34.3  1.618  1.855  1.259  2.144  1.73  1.51  2.288  1.103  2.093  1.935  2.628  1.689  2.595  2.251  2.229  1.875  2.28  2.369  3.185  1.992  2.328  1.873  2.847  1.546  1.936  1.591  2.344  1.425  1.575  1.777  2.842  2.202  1.711  1.143  1.228  1.793  2.674  2.158  2.87  2.383  1.455  1.334  3.363  1.784  1.206  1.947  2.002  1.547  1.148  3.246  2.554  1.061  1.866  0.909  1.342  1.355  1.671  1.897  2.393  1.422  
 
 CDCA8  55143  1  38158072  38175391  1p34.3  3.374  2.611  2.979  3.748  3.202  3.623  4.769  3.92  1.343  4.385  5.072  2.999  3.621  4.113  3.84  3.581  4.119  1.493  5.783  3.037  3.99  3.642  2.919  2.905  3.408  2.734  4.083  3.299  3.549  3.117  4.342  4.032  3.624  2.127  2.874  3.104  4.481  4.314  3.716  4.782  3.375  2.967  4.62  3.812  2.773  3.421  3.325  3.787  4.143  5.131  4.199  2.908  4.372  2.636  3.785  3.373  4.235  3.418  5.121  4.098  
 
 EPHA10  284656  1  38181645  38230824  1p34.3  0.413  0.134  0.191  0.173  0.594  0.173  0.171  0.096  0.279  0.107  0.059  0.195  0.544  0.783  1.078  0.326  0.762  0.217  0.077  0.26  0  0.428  0.738  0.125  0.033  0.061  0.433  0.106  0.054  0.438  0.11  0.099  0  0.269  0.27  0.242  0.08  0  0.311  0.653  0.42  0.539  1.221  0.078  0.119  0.122  1.037  0.342  1.009  0.625  0.127  1.063  0.137  0.228  0.592  0.131  0.075  0.2  1.281  0.064  
 
 MANEAL  149175  1  38259773  38267278  1p34.3  2.298  1.62  2.268  0.484  3.232  2.317  3.132  2.363  3.4  2.737  2.211  0.709  3.437  2.735  3.545  2.924  2.805  1.575  4.073  0.285  0.527  2.406  2.946  3.107  1.653  0.731  3.686  1.28  2.7  2.988  4.231  4.147  3.549  2.329  1.997  3.267  0.663  0.906  2.698  3.514  3.507  1.769  4.27  0.946  0.587  0.866  2.218  2.257  3.055  3.544  2.678  1.785  3.006  0.509  2.507  2.126  1.834  2.892  4.045  2.442  
 
 YRDC  79693  1  38268613  38273865  1p34.3  2.437  2.854  2.629  3.185  2.208  2.193  4.096  3.017  3.314  2.628  3.753  2.17  3.054  3.927  2.576  2.82  2.9  2.764  4.719  3.012  3.39  3.303  3.519  2.616  3.285  2.495  3.545  3.281  3.016  2.468  4.344  4.398  3.234  2.21  1.848  2.707  3.915  2.356  4.033  3.301  2.54  2.523  4.723  1.803  2.06  2.073  1.677  2.076  1.832  4.101  3.231  1.665  2.624  1.323  2.146  2.406  2.269  2.329  3.669  2.415  
 
 C1orf122  127687  1  38273472  38275126  1p34.3  3.906  3.3  4.257  4.693  5.465  4.231  5.1  4.836  5.036  5.094  4.861  3.459  4.228  4.226  3.079  4.063  4.658  0.688  5.272  3.563  3.307  4.581  4.499  3.844  3.836  3.624  3.857  3.902  3.672  2.632  5.061  4.939  4.362  3.94  3.716  4.257  3.822  3.998  6.593  4.394  3.766  4.437  5.552  3.468  3.243  4.516  4.275  3.184  3.627  5.384  4.119  3.187  4.273  3.975  4.252  4.183  3.645  4.043  4.552  3.655  
 
 MTF1  4520  1  38275238  38325292  1p33  1.365  0.764  1.071  1.611  1.249  0.775  2.19  1.909  1.479  1.618  1.98  0.849  1.7  1.731  1.532  1.468  1.108  0.514  1.567  1.25  0.943  1.201  1.134  0.818  1.136  1.185  1.565  1.997  1.293  0.697  1.555  1.716  1.333  0.703  0.755  1.192  1.716  1.543  1.775  2.215  1.05  0.678  1.714  1.103  1.466  1.124  1.086  1.06  1.353  1.143  1.503  0.662  1.674  1.004  1.121  1.31  1.652  0.922  2.186  1.333  
 
 INPP5B  3633  1  38326368  38412729  1p34  0.762  0.418  0.78  0.379  0.911  0.941  0.621  1.525  1.508  1.91  1.743  0.78  1.176  1.171  1.503  1.322  1.247  0.521  2.615  1.376  1.539  1.276  1.037  0.877  1.126  1.342  1.026  1.024  1.345  0.988  1.944  1.529  0.854  0.487  0.757  0.953  1.272  0.911  1.309  1.293  0.967  0.73  1.192  1.148  0.973  0.783  0.487  1.066  1.059  2.071  1.072  1.112  1.11  1.015  1.349  1.257  1.248  1.373  2.004  1.592  
 
 SF3A3  10946  1  38422651  38455761  1p34.3  3.395  4.377  2.747  3.586  3.388  3.284  4.406  4.217  3.063  3.999  4.614  3.032  3.788  4.61  3.805  3.736  3.968  4.386  5.727  5.343  4.827  4.262  3.522  3.26  4.349  2.943  3.897  3.722  3.708  3.013  4.82  4.894  4.119  2.685  2.798  3.411  4.131  3.461  4.844  4.46  3.257  2.663  4.881  3.711  2.882  3.508  4.02  3.31  3.347  5.078  4.204  2.547  3.949  2.823  3.869  3.62  4.235  3.887  4.616  4.374  
 
 FHL3  2275  1  38462441  38471187  1p34  1.347  0.774  3.17  2.564  1.842  2.096  3.467  4.813  3.09  4.194  3.588  1.905  2.841  2.984  2.57  2.921  2.717  0.448  2.815  3.096  5.013  1.066  0.742  0.424  2.682  2.617  2.843  2.125  2.636  1.571  3.458  4.626  3.206  2.215  1.693  3.588  3.681  2.997  1.909  2.625  2.518  1.428  3.123  2.522  2.068  3.791  1.403  3.143  2.883  4.481  2.705  1.479  2.986  2.66  2.683  3.27  3.762  2.232  3.388  3.068  
 
 UTP11L  51118  1  38478383  38490497  1p34.3  1.91  4.156  1.817  2.687  2.836  2.243  2.897  3.07  2.189  2.839  3.784  2.184  2.608  3.676  1.982  2.72  3.089  6.246  4.777  3.724  3.811  3.021  2.436  2.239  2.905  2.728  1.633  2.103  2.211  1.233  3.788  3.544  2.376  1.561  1.89  2.535  2.31  1.938  4.089  2.727  2.364  1.904  2.983  2.864  2.258  2.533  1.946  1.985  2.459  4.208  2.978  1.483  2.18  2.042  2.586  2.465  2.806  2.565  3.209  2.76  
 
 POU3F1  5453  1  38509522  38512450  1p34.1  0  0.094  0.126  0  0.162  0.25  0.09  0.273  0.099  0.329  0.093  0.354  0  0.159  0  0.358  0.143  0.193  0.048  0.175  0.076  0.528  0.46  0.115  0.077  0.519  0.422  0.168  0.373  0.549  2.563  0.584  0  0.153  0.16  0.053  0.04  0  0.224  0.151  0  0.574  0.18  0.103  0.116  0.245  0.233  0.09  0.148  0.047  0.097  1.479  0.091  0.357  0.205  0.143  0.106  0.081  0.059  0.074  
 
 LINC01343  339442  1  38674705  38680439  1p34.3  0  0  0.113  0  0  0  0  0.028  0.178  0.038  0  0  0  0.059  0  0  0  0  0  0  0  0.101  0.501  0  0  0  0.119  0  0  0.042  0.104  0  0  0.244  0  0  0  0  0.056  0  0  0.59  0  0  0  0.034  0.03  0  0  0  0  0.242  0  0.08  0.09  0  0  0  0  0  
 
 RRAGC  64121  1  39303868  39325495  1p34  2.191  4.037  2.547  3.772  2.659  2.236  3.556  2.534  3.318  3.42  3.475  1.638  2.585  3.02  1.708  2.358  1.785  3.411  3.399  3.183  1.726  2.62  2.701  1.387  2.383  2.694  3.525  1.894  2.585  2.524  3.979  2.979  2.232  1.811  1.247  3.071  2.615  2.601  4.39  2.234  2.217  1.986  4.032  1.722  1.734  2.158  2.739  1.549  2.116  3.2  3.226  1.244  2.469  1.622  2.592  2.431  2.941  2.337  3.329  2.505  
 
 MYCBP  26292  1  39328161  39339050  1p33-p32.2  2.965  4.019  1.442  1.272  2.917  1.394  3.024  2.504  1.26  2.518  3.146  2.993  3.127  3.921  2.72  2.743  2.272  4.496  3.983  3.794  2.973  2.211  2.632  2.441  2.66  1.515  1.921  2.113  2.023  1.343  2.775  3.006  2.01  1.277  1.65  3.455  2.371  2.809  2.615  2.299  2.036  1.69  3.528  2.497  1.635  2.584  1.797  1.575  2.558  3.396  3.081  1.17  2.677  1.516  2.734  2.532  2.156  2.571  3.43  2.807  
 
 GJA9-MYCBP  100527950  1  39328161  39347298  1p  0.197  0.228  0.153  0.533  0.17  0.077  1.324  0.205  0.361  0.314  0.22  0.157  0.667  0.208  0.117  0.332  0.358  0.228  0.13  0.204  0.124  0.472  0.238  0.117  0.069  0.43  0.985  0.193  0.237  0.21  0.307  0.117  0.347  0.226  0.151  0.405  0.168  0.26  0.291  0.125  0.266  0.451  0.473  0.065  0.18  0.152  1.145  0.25  0  0.214  0.233  0  0.716  0.068  0.195  0.099  0.197  0.142  0.215  0.113  
 
 GJA9  81025  1  39339738  39347298  1p34  0  0  0  0  0  0  0  0  0  0  0  0  0  0  0  0.121  0  0  0  0.071  0  0  0.147  0  0  0  0  0  0.09  0  0  0.106  0  0  0  0  0  0  0  0  0  0  0.101  0  0  0.102  0  0  0  0  0  0.18  0  0  0  0  0  0.186  0  0  
 
 RHBDL2  54933  1  39351477  39407502  1p34.3  0  0.862  0.157  0  0.701  0.479  0.691  0.689  0.289  0.556  0.247  0.094  0.296  0.048  0.134  2.015  0.068  0  0.087  0.106  0  0.093  0.501  0.072  0  0.101  0.315  0.096  0.067  0.075  0.2  0.305  0.113  0.25  0.198  0.218  0.267  0.203  1.393  0  0.274  0.408  0.057  0  0.449  0.353  0.217  0.423  0  1.403  0.583  0.21  0.207  0.55  0.785  0.778  0.788  0.633  1.012  0.677  
 
 AKIRIN1  79647  1  39456915  39471737  1p34.3  2.087  3.049  2.9  3.543  3.013  2.407  3.577  4.175  4.123  3.375  4.323  2.244  3.391  3.521  2.878  3.832  3.44  3.632  4.189  3.756  3.646  2.718  2.994  2.229  3.594  3.227  3.174  3.352  3.567  2.83  4.367  4.106  2.773  2.313  1.971  4.879  3.814  3.009  4.604  4.858  3.295  2.323  4.955  3.25  3.097  3.248  2.264  2.701  2.51  4.779  4.023  1.87  3.106  2.083  3.1  3.148  3.474  2.571  3.866  3.443  
 
 NDUFS5  4725  1  39491966  39500308  1p34.2-p33  7.538  9.349  6.554  7.401  7.859  7.412  8.285  7.141  6.912  7.315  7.985  6.709  6.674  8.505  5.632  7.498  7.203  10.259  8.903  9.767  7.635  8.28  7.001  7.45  7.495  5.404  7.267  6.064  6.679  6.607  8.227  7.815  6.242  5.389  7.078  8.583  6.896  6.926  9.027  6.883  6.864  6.991  9.144  7.463  7.087  7.219  7.837  6.201  6.713  8.137  7.201  4.759  7.411  6.914  7.868  7.361  6.212  7.314  8.186  6.706  
 
 MACF1  23499  1  39549838  39952810  1p32-p31  1.602  2.441  2.196  1.992  0.842  1.932  2.244  3.089  2.309  3.274  3.549  2.016  1.448  2.354  2.054  1.596  1.675  1.578  3.016  1.373  2.337  1.175  1.038  0.732  2.086  1.993  2.181  2.26  2.397  1.584  2.375  2.741  2.541  1.047  1.443  4.269  3.197  2.546  3.651  2.149  2.495  1.32  4.786  2.187  1.219  2.245  2.132  2.02  2.907  2.26  3.099  1.639  2.739  2.002  3.398  2.512  2.961  2.806  3.857  3.616  
 
 KIAA0754  643314  1  39875175  39882154  1p34.3  1.616  1.002  1.931  2.194  0.553  1.86  1.861  2.597  1.171  2.162  2.789  2.724  1.683  2.571  2.091  1.756  1.28  0.397  0.755  0.483  1.377  1.223  0.465  0.666  1.944  1.672  1.926  2.476  2.398  1.492  2.146  1.968  2.501  0.777  1.149  4.213  3.384  2.777  2.161  1.845  2.466  0.56  3.103  2.808  1.305  2.318  1.747  2.293  3.194  0.638  2.366  1.312  3.215  1.636  2.917  3.023  2.553  2.428  4.088  3.578  
 
 BMP8A  353500  1  39957317  39995541  1p34.3  0.289  0.036  0.194  0.088  0.411  0.061  0.121  0.171  0.181  0.971  0.177  0.11  0.363  0.854  0.49  0.209  0.28  0.076  0.207  1.269  0.037  0.236  1.387  0.402  0.063  0.631  0.282  0.524  0.504  0.286  0.359  0.297  0.095  0.19  0.404  0.733  0.236  0.109  0.185  0.425  0.288  0.311  0.308  0.409  0.095  0.319  0.047  0.217  0.66  0.222  0.162  0.693  0.198  0.182  0.309  0.108  0.324  0.102  0.415  0.083  
 
 PPIEL  728448  1  39987951  40025370  1p34.3  0  0.065  0.253  0.171  1.178  0.149  0.104  0.122  0.251  1.176  0.247  0.043  0.453  0.986  0.454  0.324  0.088  0.246  0.552  3.275  0.15  0.374  0.412  0.147  0.109  0.759  0.188  0.355  0.87  0.414  0.682  0.388  0.103  0.241  0.731  1.033  0.235  0.138  0.649  0.358  0.22  0.232  0.188  0.161  0.118  0.299  0.071  0.2  0.7  0.224  0.152  0.506  0.101  0.125  0.944  0  0.611  0.095  0.5  0.08  
 
 PABPC4  8761  1  40026484  40042521  1p34.2  3.354  4.019  3.376  3.476  3.783  2.815  4.34  3.942  3.242  4.608  4.258  2.607  3.368  5.558  4.028  4.051  3.331  3.634  5.756  1.149  4.517  3.343  3.666  3.722  3.916  4.025  3.738  4.134  4.527  4.019  5.262  5.461  3.88  2.76  2.683  5.082  4.718  4.573  3.562  5.051  3.576  3.667  4.215  4.701  3.286  4.189  0.885  3.042  3.985  5.239  4.094  2.869  4.254  3.014  4.038  4.238  4.444  3.556  5.254  4.779  
 
 LOC101929516  101929516  1  40030741  40038875  1p34.3  0.742  0.266  1.114  1.649  0.904  0.633  0.636  0.836  1.444  1.023  0.706  0.944  0.867  1.041  1.074  1.209  0.662  0.237  0.267  0.92  0.913  1.871  0.654  0  0.578  0.624  0.675  0.956  1.34  0  0.864  0.824  0.45  1.025  0.948  0.493  1.045  0.446  1.058  1.073  0.568  0.702  1.032  1.078  0.486  0.571  0.366  0.582  0.824  0.606  0.571  0.637  1.199  0.445  1.154  1.212  0.973  0  1.161  0.349  
 
 SNORA55  677834  1  40033045  40033182  1p34.3  0  0  0  0  0  0  0  0  0  0  0  0  0  0  0  0  0  0  0  0  0  0  0  0  0  0  0  1.671  0  0  0  0  0  0  0  0  0  0  1.028  0  0  0  0  0  0  2.462  0  0  0  0  0  0  0  0  0  0  0  0  0  0  
 
 HEYL  26508  1  40089102  40105348  1p34.3  0.556  0.095  0.184  0.103  0.049  0  0.039  0.481  0.169  0.089  0.071  0.039  0.136  1.756  0.139  0.075  0.032  0.101  0.113  0.106  0.134  0.113  0.903  0.091  0  0.055  0.08  0.055  0.054  0.079  0.068  0.075  0  0.186  0.942  1.621  0.582  0.103  0.069  1.277  0.402  0.285  1.09  0  0.749  0.206  0.035  0.677  0.132  1.18  0.031  1.147  0.053  0.183  1.112  0.054  0.058  0.634  0.076  0.063  
 
 NT5C1A  84618  1  40124792  40137710  1p34.3-p33  0  0  0.159  0  0  0  0  0.072  0.175  0.059  0  0.123  0  0  0  0  0  0.113  0  0.168  0  0.295  0.711  0  0  0.152  0.135  0  0  0.156  0  0  0  0.281  0  0  0  0  0.075  0.153  0  0.22  0.055  0  0  0  0  0  0  0.165  0  0.734  0  0.13  0.119  0  0  0  0  0  
 
 HPCAL4  51440  1  40144319  40157382  1p34.2  0  0.031  0.18  0  0.061  0.134  0  0.079  0.189  0.057  0.094  0.033  0.042  0.951  0.093  0.051  0.036  0.107  0.041  0.055  0  0.199  0.491  0.029  0  0.105  0.129  0.141  0.158  0.154  0.338  0.205  0.1  0.286  0.059  0.054  0.03  0.05  0.13  0.496  0  0.294  0.053  0  0.171  0.12  0.034  0.094  0  0.624  0.032  0.655  0.024  0.134  0.063  0.04  0.034  0  0.089  0  
 
 PPIE  10450  1  40204516  40229586  1p32  2.351  1.523  2.132  2.564  3.289  1.736  2.297  2.729  2.714  3.136  3.531  2.548  2.6  3.871  2.496  2.864  2.405  1.063  3.963  2.919  2.853  2.578  2.87  1.824  2.31  2.287  2.951  2.468  2.427  2.224  3.517  3.414  2.779  1.693  1.875  3.834  2.268  2.374  3.04  2.835  2.548  2.2  3.528  2.302  1.87  2.466  2.959  2.123  2.35  4.322  3.246  2.172  1.546  1.488  3.115  1.863  2.428  3.143  2.643  2.319  
 
 BMP8B  656  1  40223902  40254533  1p35-p32  0.74  0.115  0.205  0.103  0.653  0.29  0.259  0.366  0.373  1.927  0.538  0.561  0.864  1.178  1.516  0.728  0.837  0.218  0.222  0.712  0.198  0.579  0.871  0.833  0.171  2.81  0.658  2.05  1.991  0.723  0.683  0.501  0.296  0.235  0.968  1.681  0.197  0.241  0.378  1.497  0.752  0.494  0.328  0.664  0.225  0.578  0.874  0.723  0.586  1.091  0.658  1.298  0.379  0.548  0.92  0.22  0.36  0.72  1.601  0.236  
 
 OXCT2  64064  1  40235196  40237020  1p34  2.054  0.157  1.651  1.258  1.618  0.628  2.015  0.268  1.885  1.525  1.593  1.064  0.888  1.939  1.203  1.9  2.675  0.381  1.631  1.952  0.507  2.579  1.832  1.098  1.327  0.801  1.855  1.925  1.398  1.638  0.615  0.637  0.413  1.402  1.457  0.898  0.967  0.291  1.438  0.854  0.373  1.999  1.505  0.699  0.745  0.426  1.983  0.699  0.31  2.138  0.628  2.328  1.627  1.059  2.753  1.05  0.657  0.558  1.613  0.837  
 
 TRIT1  54802  1  40306705  40349177  1p34.2  1.692  2.407  1.459  2.194  1.837  1.411  1.955  1.924  1.607  1.75  2.571  1.437  1.882  3.22  1.813  2.207  2.269  4.683  3.419  2.172  2.582  1.793  2.246  1.338  2.019  1.317  1.434  1.684  1.446  1.393  2.269  2.696  1.472  0.982  1.255  2.579  1.936  1.733  2.118  2.551  1.501  1.553  3.214  1.509  1.238  1.698  1.235  1.153  1.411  3.086  1.911  1.1  1.733  0.803  2.185  1.506  1.653  1.591  2.675  1.56  
 
 MYCL  4610  1  40361095  40367687  1p34.2  0.329  0.22  0.407  1.005  0.773  0.253  0.697  0.307  0.828  0.826  0.754  1.19  0.561  1.123  0.91  0.506  0.807  0.365  0.296  0.991  0.784  0.633  1.218  0.454  0.421  0.252  0.33  0.259  0.535  0.878  0.573  0.616  0.354  0.399  0.276  1.322  0.19  0.173  1.244  2.81  0.399  0.79  1.882  0.858  0.464  0.565  0.2  0.392  0.481  1.121  0.468  0.919  0.498  0.502  0.76  0.358  0.552  0.414  0.972  0.213  
 
 MFSD2A  84879  1  40420783  40435640  1p34.2  0.287  0.655  0.811  1.225  1.028  1.515  3.237  2.726  4.866  2.851  4.526  0.927  3.012  0.973  1.506  1.225  0.979  0.366  2.406  1.207  0  1.728  1.564  1.475  1.122  1.14  1.758  0.053  0  0.492  1.935  2.308  0.561  0.211  0.475  1.14  2.194  1.976  1.016  1.553  0.824  1.967  1.108  1.117  0.908  1.265  0.569  0.989  0.172  1.773  1.21  0.756  1.187  0.843  0.749  1.498  0.775  0.593  1.4  0.785  
 
 CAP1  10487  1  40506254  40538321  1p34.2  3.935  6.667  6.072  5.794  5.024  4.703  5.746  6.065  5.033  6.124  6.186  4.775  5.018  5.902  4.397  5.126  5.194  5.622  6.433  6.539  4.491  4.986  4.749  3.553  6.074  5.428  5.235  5.356  6.106  4.262  6.364  6.17  5.457  4.255  4.123  6.668  5.661  5.442  6.933  5.826  4.875  3.972  5.774  4.69  4.259  5.556  4.742  4.831  5.35  6.516  5.773  4.044  5.682  4.82  5.886  5.447  6.33  4.922  7.046  6.037  
 
 PPT1  5538  1  40538381  40563142  1p32  4.409  4.056  3.304  4.711  5.021  2.66  4.528  4.499  4.654  4.602  4.688  3.687  3.76  4.138  4.28  4.233  4.296  4.113  5.296  4.752  3.696  3.596  4.294  3.675  4.17  4.074  4.211  4.102  4.41  3.243  5.331  5.155  3.894  3.021  2.433  4.684  4.166  3.133  3.682  4.77  4.361  4.056  4.659  3.746  3.185  3.365  3.647  3.428  2.926  5.338  3.952  2.676  3.658  2.622  4.451  3.815  3.586  3.816  4.731  4.076  
 
 RLF  6018  1  40627040  40706593  1p32  1.245  1.234  1.096  0.947  1.104  0.786  1.31  2.01  1.015  1.634  2.28  0.629  1.459  1.765  1.486  1.09  1.714  1.289  1.066  0.8  1.589  0.908  0.603  0.674  1.082  0.918  1.122  1.042  1.475  0.763  1.549  1.404  1.013  0.481  0.656  2.195  1.35  1.182  1.177  1.468  1.381  0.532  1.236  1.597  0.999  1.141  0.78  0.919  1.457  0.992  1.783  0.649  1.221  0.627  0.955  1.145  1.975  1.079  1.812  1.191  
 
 ZMPSTE24  10269  1  40723721  40759856  1p34  2.608  3.958  1.986  2.669  2.746  1.928  3.467  2.963  3.043  3.259  3.616  2.186  3.266  3.734  2.553  2.738  3.006  3.631  4.037  2.832  2.632  1.752  2.557  1.72  2.556  2.304  2.227  2.129  2.367  1.45  3.118  3.138  2.22  1.739  1.661  4.409  2.923  2.383  3.181  2.792  2.69  2.469  2.699  2.296  1.844  2.565  1.736  1.575  2.509  3.233  3.293  1.261  2.488  2.485  1.449  2.027  2.825  1.909  3.561  2.534  
 
 COL9A2  1298  1  40766162  40782939  1p33-p32  1.318  0.048  0.136  0.191  1.91  0.175  0.141  0.205  0.857  0.731  0.746  0.094  0  0.633  0.097  0.077  0.073  0.068  0.693  0.499  0  0.238  1.652  0.619  0.049  0.508  0.336  1.514  0.212  0.228  0.473  1.027  0  0.142  0  0.125  1.017  0.26  0.128  0.899  0.862  0.452  1.181  0  1.004  2.181  0.095  0.961  0.088  2.151  0.111  0.201  0.109  0.146  0.047  0.094  1.527  0.12  0.074  0.063  
 
 SMAP2  64744  1  40839377  40888998  1p35.3-p34.1  2.163  1.231  3.102  3.082  2.594  2.412  4.744  4.006  2.455  3.75  3.681  3.207  2.773  2.419  2.086  2.033  2.839  1.143  4.915  3.222  4.586  3.375  4.518  3.365  2.951  2.379  3.689  3.292  3.707  3.015  3.449  4.762  3.822  2.285  1.26  2.892  2.689  3.146  1.769  2.907  2.051  1.806  3.679  1.822  2.063  2.312  1.818  2.116  2.219  3.361  5.385  1.361  2.392  1.625  1.213  1.883  2.713  1.828  2.312  1.896  
 
 ZFP69B  65243  1  40916336  40929390  1p34.2  0.744  1.045  0.878  0.908  0.569  0.356  0.864  1.036  0.246  0.798  0.78  0.195  0.635  0.084  0.592  0.218  0.513  1.156  1.122  0.233  0.862  0.818  0.54  0.194  1.117  0.262  0.354  0.685  0.337  0.171  0.804  1.119  0.986  0.63  0.304  1.592  0.611  0.756  0.212  1.197  0.648  0.424  2.038  0.718  0.264  0.446  0.197  0.305  0.511  0.448  0.565  0.483  0.716  0.364  0.378  0.776  0.808  0.736  0.199  0.659  
 
 ZFP69  339559  1  40943301  40962015  1p34.2  1.2  1.58  0.618  1.044  0.078  0  0.897  1.269  0  0.726  0.059  0.693  0.924  0.043  0.761  0.266  0.938  2.771  2.339  0.646  0.528  0.6  0.81  0.498  0.775  0.538  0.766  0.674  0.669  0.463  1.319  0.613  1.082  0.756  0  1.532  1.085  0.809  0.47  1.096  0.126  0.291  2.287  0.371  0.411  0.365  0.33  0.372  0.791  0.972  0  0.344  0.142  0.638  0.073  0.741  0.734  0.601  0.459  0.537  
 
 EXO5  64789  1  40974432  40982214  1p34.2  1.66  2.447  0.964  1.609  1.636  0  1.497  2.038  0.694  1.522  1.156  0.382  0.076  0.303  0  1.383  0.736  2.227  3.151  1.719  0.333  2  1.176  0.608  0.184  0.073  1.143  0.936  1.04  0.189  1.874  0.829  0.913  0.545  0.418  2.218  2.026  1.273  1.448  1.594  1.452  0.681  2.939  1.271  0.302  0.917  1.991  1.304  1.51  2.455  0.357  0.817  1.592  0.82  1.387  0.21  1.697  1.281  1.349  1.1  
 
 ZNF684  127396  1  40997232  41013841  1p34.2  0.472  1.028  0.425  0.827  0.473  0.211  0.792  0.651  0.4  0.725  1.066  0.228  0.333  0.983  0.186  0.327  0.887  1.169  1.796  0.652  0.247  0.573  0.525  0.337  0.381  0.497  0.72  0.334  0.472  0.247  0.968  0.596  0.695  0.579  0.203  1.105  0.414  0.216  0.398  0.636  0.313  0.392  1.399  0.31  0.407  0.469  0.143  0.296  0.416  0.933  0.594  0.147  0.409  0.308  0.488  0.33  0.544  0.175  0.648  0.383  
 
 RIMS3  9783  1  41086351  41131324  1pter-p22.2  0.408  0.086  0.788  0.773  0.191  0.183  0.152  1.325  1.385  0.727  0.614  0.331  0.529  0.926  0.639  0.031  0.128  0.095  2.37  0.097  0.192  1.051  1.237  1.071  0.239  0.386  0.318  0.147  0.272  0.35  0.14  0.128  0.261  0.244  0.291  0.759  0.135  0.408  0.27  0.76  0.075  0.297  0.921  0.223  0.269  0.258  0.22  0.643  0.41  1.07  0.091  0.667  0.09  0.54  0.902  0.069  0.388  0.629  2.005  0.143  
 
 NFYC-AS1  100130557  1  41154751  41157933  1p34.2  0.422  0.283  0.22  0.437  0.507  0.547  0.232  0.252  0.28  0.565  0.487  0.14  0.357  0.276  0.393  0.557  0.301  0.657  0.905  0.427  0.452  0.471  0.389  0.267  0.213  0.251  0.404  0.304  0.413  0.276  0.63  0.425  0.409  0.38  0.37  0.535  0.298  0.484  0.452  0.212  0.292  0.245  0.047  0.311  0.361  0.375  0.795  0.473  0.25  0.822  0.266  0.379  0.27  0.177  0.535  0.234  0.378  0.284  0.539  0.164  
 
 NFYC  4802  1  41157241  41237276  1p32  2.818  2.409  1.853  3.048  2.953  2.55  4.277  3.378  3.028  3.402  3.87  3.044  3.251  3.715  3.391  3.111  2.978  1.87  5.466  3.092  3.13  3.905  3.416  3.135  3.308  2.579  4.655  3.557  3.783  3.6  4.204  4.065  3.817  2.094  2.663  5.136  3.717  2.802  3.264  4.784  3.728  2.794  4.869  3.007  2.824  3.466  2.415  3.076  2.547  4.27  3.335  2.203  3.129  2.443  3.203  2.805  3.012  3.491  4.021  3.486  
 
 KCNQ4  9132  1  41249683  41306124  1p34  0.99  0.138  0.202  1.116  0.78  0.172  0.046  0.109  0.162  0.087  0.088  0.148  1.044  1.198  1.337  1.268  0.232  0.293  0.682  1.116  0.793  1.069  0.677  0.516  0.102  0.683  0.282  0.091  0.175  0.148  0.181  0.1  0  0.161  0.147  0.535  0.229  1.388  0.227  0.413  0.107  0.677  0.66  1.432  0.096  0.305  0.755  0.365  0.057  0.514  0.069  0.869  0.08  0.176  0.771  0.114  0.193  0.043  1.432  0.386  
 
 CITED4  163732  1  41326727  41328018  1p34.2|1p35-p34  1.185  0.155  0.51  5.423  4.352  1.158  0.547  0.4  1.74  0.584  1.227  0.895  0.548  1.21  0.593  1.298  0.33  0.375  0.214  2.377  0.275  0.846  0.696  0.089  0.196  0.172  4.124  0.479  0.208  0.543  0.265  1.729  3.339  4.645  2.716  3.758  0.9  0.257  1.226  1.089  4.187  3.923  0.623  1.34  0.825  2.818  3.061  1.594  1.112  3.241  0.743  2.233  0.366  0.816  2.998  3.609  0.428  2.895  2.475  2.233  
 
 CTPS1  1503  1  41444970  41478237  1p34.1  3.337  4.134  2.973  3.492  2.417  2.968  4.401  3.556  2.504  3.561  4.041  2.509  3.548  4.259  3.291  3.438  2.776  3.392  5.838  3.868  3.479  4.15  3.427  2.747  3.641  2.904  3.981  3.005  3.336  2.579  4.572  4.491  3.411  1.739  3.35  5.804  4.71  3.136  3.625  3.922  2.863  3.043  4.777  3.043  2.312  3.533  4.029  3.595  3.024  5.584  4.04  1.842  3.394  1.862  3.318  2.956  3.207  4.379  3.958  3.453  
 
 SLFNL1-AS1  100507178  1  41480261  41509562  1p34  0.294  0.144  0.421  0.179  0.39  0.417  0.331  0.808  0.292  0.813  0.897  0.13  0.468  0.557  1.137  0.37  0.142  0.111  0.461  0.383  0.691  0.732  0.59  0.662  0.104  0.374  0.819  0.768  0.507  0.418  0.662  0.525  0.792  0.301  0.766  1.933  1.109  0.84  0.255  0.754  0.701  0.418  0.253  0.621  0.586  0.98  0.283  1.489  0.723  0.335  0.673  0.815  0.716  0.292  0.336  0.265  0.725  1.389  1.142  0.156  
 
 SLFNL1  200172  1  41481268  41487427  1p34.2  0.36  0  0.252  0  0.136  0.077  0.281  0.585  0.106  0.474  0.601  0.186  0.379  0.446  0.772  0.13  0.125  0  0.324  0.217  0.207  0.399  0.252  0.463  0.125  0.166  0.445  0.502  0.325  0.097  0.231  0.37  0.346  0.137  0.668  1.16  0.954  0.468  0.111  0.403  0.419  0.329  1.135  0.289  0.4  0.719  0  1.332  0.392  0.151  0.376  0.789  0.471  0.143  0.218  0.464  0.382  0.299  0.613  0.145  
 
 SCMH1  22955  1  41492870  41707815  1p34  1.816  1.575  2.205  3.083  2.194  2.118  3.305  3.263  2.857  3.617  2.956  1.502  2.351  2.71  2.867  2.98  2.048  0.58  4.515  2.825  2.501  3.302  1.566  2.125  2.318  2.043  3.123  2.573  1.926  2.511  3.184  2.769  2.942  1.424  1.521  4.283  3.047  3.191  2.775  2.875  2.37  2.467  3.466  2.197  1.694  1.905  1.153  2.077  2.649  3.106  2.803  1.906  2.881  2.193  2.77  2.161  2.902  2.14  3.755  2.21  
 
 FOXO6  100132074  1  41827602  41849263  1p34.2  0.731  0.194  0.366  0  1.32  0.204  0.142  1.298  1.683  0.335  0.507  0.209  0.075  0.808  0.568  0.793  0.194  0.437  0.25  0.223  0.108  1.233  0.616  0.933  0.117  0.21  0.301  0.497  0.13  0.228  1.283  0.135  0  0.087  0.282  0.904  0.922  0.936  0.399  1.249  0.363  0.763  0.596  0.37  0.611  0.716  0.135  1.278  0.406  1.48  0.146  1.46  0.16  0.721  0.413  0.334  0.264  0.729  0.635  0.577  
 
 EDN2  1907  1  41944445  41950354  1p34  0.582  0.16  0.28  0  0.699  0  0  0.274  0  0  1.017  0  0  0.188  0.153  0.087  0.418  0  0  0  0  0.182  0.832  1.137  0  0  0.098  0.069  0  0.104  0  0  0  0  1.479  0.516  0.312  0.894  0.345  0  0  0.253  0.071  2.129  3.027  6.034  0  0  2.013  1.516  0.102  0.729  3.858  2.382  5.795  1.452  0.539  0  1.336  2.198  
 
 HIVEP3  59269  1  41972035  42501596  1p34  2.142  1.904  1.225  0.452  2.581  1.433  2.346  3.462  3.185  2.738  3.771  1.17  0.228  2.1  0.299  1.373  1.575  0.565  1.421  1.39  0.177  2.397  1.108  1.607  1.866  1.55  3.049  2  1.345  0.341  1.632  2.642  2.234  1.031  0.639  2.458  1.906  3.249  1.672  1.656  1.491  0.969  1.097  2.911  0.663  1.235  1.36  1.162  1.657  1.524  2.826  0.838  1.505  0.721  0.975  1.411  2.424  0.845  1.044  1.48  
 
 GUCA2B  2981  1  42619091  42621495  1p34-p33  0  0  0  0  0  0  0  0  0  0  0  0  0  0  0  0  0  0  0  0  0  0.168  0  0  0  0  0.1  0  0  0.104  0  0  0  0  0  0.37  0  0  0  0  0  0  0  0  0  0  0  0  0  0  0  0.319  0  0  0  0  0  0  0  0  
 
 GUCA2A  2980  1  42628361  42630395  1p35-p34  0  0  0  0  0  0  0  0  0.237  0  0  0.744  0  0  0  0.238  0  0  0  0  0  0.155  0  0  0  0  0  0  0  0  0  0  0  0  0  0.627  0  0  0  0  0  0.456  0  0  0  0.266  0  0  0  0  0  0  0  0  0  0  0  0  0  0  
 
 FOXJ3  22887  1  42642209  42801548  1p34.2  2.455  2.324  2.212  2.122  2.529  1.852  3.558  3.091  2.261  2.934  3.345  2.643  2.94  3.202  2.935  2.719  2.863  2.412  3.248  3.074  3.09  3.226  2.015  2.455  3.063  2.243  3.544  3.295  3.151  2.878  3.384  3.281  2.742  1.406  2.076  3.856  3.257  2.602  2.953  3.506  2.775  1.319  3.785  2.909  2.207  2.499  3.343  2.49  3.192  2.998  2.957  1.313  3.07  2.351  2.579  2.622  2.843  2.731  3.701  2.741  
 
 RIMKLA  284716  1  42846467  42889900  1p34.2  0.208  0.031  0.099  0.126  0.114  0.224  0.047  0.037  0.387  0.067  0.67  0.396  0.173  0.383  0.459  0.464  1.215  0.266  0.495  0.033  0.032  0.614  0.42  0.031  0.013  0.019  0.166  0.015  0.065  0.329  0.05  0.04  0  0.153  0.254  0.717  0.013  0.034  0.09  0.252  0.347  0.411  0.172  0.761  0.033  0.135  0.563  0.084  0.105  0.124  1.014  0.691  0.036  0.096  0.221  0.088  0.109  0.167  0.013  0.05  
 
 ZMYND12  84217  1  42896000  42921938  1p34.2  0  0.483  0.091  0  0  0.114  0.058  0  0.09  0.041  0  0.07  0  0.156  0  0.082  0.156  0.201  0.05  0.303  0  0.117  0.222  0  0  0.101  0.067  0.124  0.263  0.064  0.428  0.108  0  0.118  0  0.166  0.109  0.192  0.115  0.275  0  0.558  0.05  0  0.175  0  0.024  0  0  0.069  0.063  0.475  0  0.04  0.412  0  0  0  0  0.402  
 
 PPCS  79717  1  42922011  42939049  1p34.2  2.695  3.687  2.136  3.188  3.176  1.481  1.694  2.345  2.643  2.179  3.537  2.455  2.411  3.78  2.104  2.962  2.598  3.847  3.462  3.152  0.898  2.099  2.751  1.533  2.365  2.235  2.608  1.766  2.636  2.154  3.419  2.893  1.853  2.202  1.886  4.119  1.958  2.431  4.442  2.289  3.449  2.61  3.889  2.062  1.749  2.72  2.835  1.336  2.245  3.576  2.68  1.978  1.811  1.667  2.786  2.351  2.014  2.605  3.014  2.547  
 
 CCDC30  728621  1  43000559  43120335  1p34.2  0  0.025  0.109  0  0.131  0.072  0.054  0.029  0.107  0.126  0.114  0  0.079  0.145  0  0  0.035  0.207  0  0.034  0  0.032  0.092  0  0.045  0.085  0.098  0.027  0  0.042  0  0  0  0.104  0.042  0.227  0  0.047  0.089  0  0.051  0.179  0.323  0  0  0  0.025  0  0.183  0  0.04  0  0  0.021  0.04  0.115  0.047  0  0  0  
 
 PPIH  10465  1  43124047  43142429  1p34.1  2.661  3.395  1.658  2.563  3.197  2.761  4.218  3.273  1.892  3.026  4.18  2.75  3.006  4.741  2.643  3.511  3.573  2.425  5.692  5.604  4.763  4.58  3.116  3.107  3.208  2.32  2.552  2.288  2.278  0.887  3.573  3.465  2.959  1.749  2.897  4.139  2.911  2.352  4.282  2.932  2.617  2.557  2.759  3.79  3.071  2.961  2.308  2.722  2.705  5.106  3.175  1.717  3.028  2.515  3.436  3.13  2.508  3.593  3.987  3.125  
 
 YBX1  4904  1  43148065  43168020  1p34  6.764  7.303  7.113  7.623  6.161  6.948  8.016  7.814  7.596  7.973  8.465  7.292  7.501  8.394  7.435  7.347  6.924  7.693  9.414  7.927  7.884  7.962  7.479  7.407  7.633  6.516  7.345  6.125  7.371  6.345  8.657  8.749  7.235  5.472  6.365  8.376  8.131  7.644  7.269  8.201  6.748  6.983  8.21  7.656  6.998  7.411  5.633  7.201  7.554  8.73  8.121  6.094  7.527  5.653  7.368  7.798  7.751  7.601  8.895  7.932  
 
 CLDN19  149461  1  43198763  43205925  1p34.2  0  0  0.206  0  0.029  0  0  0.039  0.18  0.95  0  0.044  0  0.02  0.14  0.042  0  0.038  0  0.043  0  0.119  0.673  0  0  0  0.06  0.036  0  0.056  0  0  0  0.183  0  0.101  0  0  0.064  0  0  0.449  0.032  0  0.06  0.104  0.02  0.061  0  0  0.031  0.428  0  0.055  0.04  0  0  0  0  0  
 
 P3H1  64175  1  43212005  43232755  1p34.1  0.923  1.779  4.067  4.549  1.238  2.589  5.027  5.995  2.825  5.846  4.46  0.902  1.72  2.689  1.839  1.071  1.989  0.325  4.366  2.833  2.662  2.908  1.731  1.707  2.747  2.951  4.218  3.646  3.85  1.812  3.816  4.994  3.481  1.701  1.674  4.624  4.149  3.704  2.735  4.18  1.391  2.871  4.07  3.457  1.167  1.995  2.454  2.212  2.975  3.457  3.442  1.923  4.15  2.905  2.982  3.585  4.656  3.07  3.669  3.112  
 
 C1orf50  79078  1  43232915  43241413  1p34.2  2.38  2.529  1.486  2.221  2.466  1.695  2.987  2.223  1.997  2.251  2.369  1.791  1.7  2.417  1.726  2.1  2.202  2.248  3.756  3.263  1.958  3.322  2.387  1.797  1.724  1.416  2.121  1.171  1.763  1.03  2.361  2.033  1.337  0.995  0.98  2.925  1.942  1.655  3.362  1.96  1.795  1.216  3.285  2.013  1.259  1.991  2.661  1.349  1.608  2.871  2.182  0.898  1.682  1.458  2.171  1.978  1.629  1.923  2.76  1.838  
 
 LOC100129924  100129924  1  43253660  43263901  1p34.2  0.58  0  0.174  0  0.106  0  0  0  0.245  0.06  0  0  0  0.513  0  0.129  0.695  0  0.12  0  0  0.087  0  0  0  0  0.057  0.084  0.174  0.066  0  0.146  0  0  0.294  0  0.318  0  0.156  0.223  0  0  0.782  0  0  0.125  0  0  0  0  0.137  0.413  0.102  0.83  0  0.252  0.364  0  0.26  0  
 
 SVBP  374969  1  43272722  43283059  1p34.2  1.842  5.037  1.764  2.464  1.985  3.313  2.491  2.744  3.167  2.752  3.315  2.706  1.428  3.123  1.647  2.945  2.404  5.562  4.784  3.298  3.48  3.209  1.813  1.75  2.19  1.351  2.465  1.025  1.496  1.308  2.328  2.122  1.292  1.275  2.121  3.012  1.781  2.351  2.64  2.336  2.168  2.601  3.462  2.617  1.439  2.142  3.46  2.117  2.889  3.932  2.355  1.696  2.241  1.809  2.553  2.038  2.584  3.106  3.274  1.887  
 
 ERMAP  114625  1  43282775  43310660  1p34.2  0.953  0.669  0.396  0.928  1.145  0.516  1.285  1.169  1.233  1.294  0.864  0.642  0.826  0.705  0.592  1.179  0.717  1.35  1.295  0.764  2.385  0.586  0.687  0.245  0.299  0.666  0.534  0.479  0.453  0.213  0.657  0.655  0.451  0.387  0.499  1.873  1.047  0.95  0.98  1.033  1.02  0.53  0.579  0.636  0.365  0.514  0.356  0.368  0.981  0.621  1.229  0.716  0.905  0.69  1.045  0.597  1.222  0.747  1.67  0.829  
 
 ZNF691  51058  1  43312243  43318146  1p34.2  1.91  0.961  1.22  2.266  1.756  0.831  2.088  1.842  2.221  2.195  2  1.176  1.558  2.415  1.257  1.655  1.966  0.316  3.434  2.124  2.047  2.518  2.38  1.115  1.363  1.575  2.164  1.357  1.663  1.812  2.298  2.125  1.563  1.31  0.741  2.632  2.017  1.744  2.028  2.089  1.28  1.762  2.87  1.551  1.151  1.265  1.034  1.309  1.225  2.743  1.358  0.912  1.779  0.957  1.938  1.464  1.455  0.799  2.251  1.428  
 
 LOC339539  339539  1  43323292  43354463  1p34.2  0.447  0.068  0.578  0.302  0.324  0.164  0  0.035  0.135  0.059  1.027  0.046  0  0.412  0  0.746  0.104  0.417  0.086  0.174  0  0.518  0.805  0.303  0.314  0.161  0.254  0.032  0.563  0.411  0  0.102  0  0.225  0.097  0.309  0.071  0.206  1.163  0  0  0.48  0.483  0.108  0.119  0.261  0.346  0.135  0.146  0.055  0.073  0.786  0.065  0.192  1.441  0.513  0.375  0  0.065  0.051  
 
 SLC2A1  6513  1  43391045  43424847  1p34.2  4.157  3.816  4.639  2.076  2.287  3.606  4.99  4.736  5.261  4.364  6.694  3.335  5.672  3.868  3.967  5.522  5.295  4.451  3.812  2.049  3.834  2.083  2.477  2.86  4.166  2.656  3.826  4.054  4.47  2.923  2.936  4.479  3.252  1.734  3.039  4.298  4.685  6.012  3.348  2.876  5.193  5.073  2.254  4.76  3.422  4.042  3.848  2.695  4.489  4.704  3.598  3.732  5.551  4.875  4.147  4.22  6.249  4.092  5.001  4.837  
 
 SLC2A1-AS1  440584  1  43424719  43449029  1p34.2  0.576  0  0.296  0  0.766  0.488  0.288  0.236  0.371  0.316  0.618  0  0.763  0.283  0.179  0.817  0.444  0.656  0.085  0  0.196  0.171  0.498  0  0  0.461  0.196  0.134  0.381  0.345  0.204  0.266  0.246  0.219  0.543  0.544  0.121  1.132  0.091  0.206  0.607  0.354  0  0.226  0  0.214  0.139  0  0.213  0.289  0.424  0.317  0.627  0.692  0.432  0  0.751  0  0.455  0.581  
 
 FAM183A  440585  1  43613593  43622067  1p34.2  0.455  0.252  0  0  1.094  0  0  0  0  0  0  0.223  0  0.211  0  0.613  0  0.781  0  0  0  0  0  0  0  0  0  0  0.334  0.068  0  0  0  0  0  0.87  0.397  0  0.427  0.617  0  0  0.154  0  0  0  2.347  0  0  0  0  0  0.169  0.432  0.725  0  0  0  0  0  
 
 EBNA1BP2  10969  1  43629844  43638241  1p35-p33  4.028  7.259  4.181  5.455  4.506  4.711  5.248  4.785  3.833  4.262  5.423  4.119  4.427  5.35  4.313  4.996  4.692  7.29  6.309  5.602  5.094  4.668  4.081  4.635  5.056  3.523  3.857  5.468  4.53  3.216  5.352  6.291  5.153  3.847  4.372  6.106  5.296  4.853  6.087  5.617  4.667  4.229  5.344  4.998  4.71  5.052  5.024  4.785  4.456  6.657  4.884  4.224  4.446  3.715  4.917  4.314  4.541  6.218  5.795  5.399  
 
 CFAP57  149465  1  43638000  43720029  1p34.2  0  0.093  0.117  0.192  0.281  0.082  0.291  0.051  0.185  0.194  0.074  0.164  0.235  0.187  0.098  0.253  0.189  0.064  0.14  0.102  0  0.083  0.319  0.043  0.163  0.084  0.191  0.239  0.118  0.125  0.053  0.109  0.052  0.239  0.113  0.142  0.307  0.137  0.368  0.202  0.068  0.076  0.538  0.069  0.13  0.061  0.202  0.104  0.063  0.462  0.132  0.421  0.07  0.221  0.1  0.142  0.051  0.085  0.359  0.129  
 
 TMEM125  128218  1  43735664  43739673  1p34.2  1.653  0  0.235  0.179  3.356  0.305  0.067  0.097  0.143  0.164  0.089  3.117  3.07  1.623  2.969  3.05  2.279  0.266  0.088  0.114  0  0.255  0.678  0.069  0.086  0.054  0.438  0.045  0  0.255  0.264  0.107  0.258  0.178  0.092  0.138  0.333  0  0.147  0  2.405  0.459  0.18  0.273  1.749  2.378  0.098  0.14  0  0  0.074  0.953  0.079  0.202  0.12  0.097  0  0.499  0  0  
 
 C1orf210  149466  1  43747556  43751288  1p34.2  2.175  0  0.31  0  2.732  0.369  0  0.081  0.27  0.095  0.08  3.271  1.802  1.396  1.957  2.716  2.301  1.582  0  0  0  0.133  0.439  0  0  0  0.367  0.075  0  0.272  0.237  0.138  0.153  0.236  0.084  0.121  0  0  0.108  0  1.368  0.48  0.121  0.245  1.564  2.468  0.106  0  0  0  0.125  0.389  0  0.158  0.094  0  0  0.305  0  0  
 
 TIE1  7075  1  43766565  43788781  1p34-p33  0.198  0.047  0.179  0.131  0.149  0.527  0.412  0.219  0.145  0.504  0.314  0.241  0.071  0.205  0.134  0.241  0.18  0.132  0.72  0.233  0.121  0.399  0.371  0.083  0.112  0.277  0.542  0.215  0.235  0.372  0.502  0.213  0.198  0.111  0.174  0.141  1.336  0.196  0.462  0.195  0.099  0.397  0.399  0.094  0.082  0.237  0.177  0.33  0.057  0.53  0.443  0.784  0.408  0.252  0.33  0.312  0.185  2.062  0.286  0.566  
 
 MPL  4352  1  43803474  43820135  1p34  0.316  0.106  1.271  0  0.188  0.454  0.192  0.093  1.649  0.259  0.263  0.151  0.227  0.459  0.288  0.299  0.277  0.576  0.108  0.106  0.063  0.329  0.539  0.136  0.081  0.143  0.47  0.229  0.244  0.25  0.348  0.175  0.155  0.569  0.174  0.312  0.253  0.212  0.27  0.282  0.127  0.26  0.604  0.149  0.11  0.478  0.499  0.248  0.236  0.295  0.219  0.703  0.283  0.202  0.413  0.154  0.273  0.237  0.68  0.218  
 
 CDC20  991  1  43824625  43828873  1p34.1  5.383  3.598  4.647  5.246  4.526  5.348  7.107  5.828  2.57  5.928  6.923  4.612  5.709  6.724  5.889  5.399  5.297  2.113  7.429  5.137  5.243  4.994  3.167  4.192  5.831  3.92  6.025  5.437  5.462  4.471  5.999  6.616  5.101  2.71  5.011  6.404  6.98  6.05  4.352  6.621  5.475  5.135  6.812  5.741  5.053  5.682  5.762  5.768  6.09  7.517  5.803  4.979  6.655  4.03  6.206  5.527  5.959  6.01  7.448  5.682  
 
 ELOVL1  64834  1  43829067  43833745  1p34.2  4.738  2.722  4.883  5.21  5.446  4.524  7.124  5.331  4.066  5.52  5.588  4.871  5.637  5.085  4.716  5.557  5.088  0.74  5.207  4.106  3.867  3.42  3.977  4.363  5.322  4.768  6.617  5.09  5.235  5.047  5.726  6.126  4.963  3.15  4.322  6.518  5.89  6.11  5.601  5.031  5.39  4.499  6.442  4.927  3.519  4.929  5.293  4.469  4.85  6.458  5.688  3.707  5.487  4.128  4.915  5.015  4.805  5.317  6.405  5.451  
 
 MED8  112950  1  43849578  43855483  1p34.2  2.417  3.509  3.36  3.933  3.273  2.357  4.414  4.033  3.018  3.95  3.886  2.578  2.599  3.39  2.22  3.084  2.939  3.008  4.19  2.977  2.908  2.635  3.851  2.628  2.999  2.84  3.179  2.988  3.132  2.024  4.155  3.83  3.026  2.238  2.134  4.076  2.889  3.168  3.935  2.944  2.783  2.712  3.713  2.593  2.386  2.755  3.414  2.468  2.326  4.416  3.199  2.022  2.891  2.967  3.126  3.313  3.28  3.382  3.707  2.936  
 
 SZT2  23334  1  43855555  43919918  1p34.2  1.235  0.278  1.222  1.435  1.299  1.178  1.625  2.308  1.124  2.505  2.364  0.84  1.641  1.276  1.947  1.308  0.87  0.154  1.91  1.043  1.118  1.644  0.864  1.21  0.987  1.542  1.679  2.095  2.061  1.052  2.105  1.807  1.512  0.537  0.806  2.805  2.19  1.952  1.941  1.969  1.531  1.059  1.391  1.55  1.083  1.331  0.96  1.502  1.717  1.727  1.91  1.307  1.903  1.15  1.419  1.349  2.199  1.844  2.3  1.426  
 
 HYI  81888  1  43916673  43919660  1p34.2  0.868  2.629  1.579  1.836  2.274  3.509  3.223  2.979  1.27  2.386  2.426  1.471  0.54  1.918  1.899  2.178  0.286  0.554  5.097  2.359  0  3.367  0  0  2.544  2.199  3.081  2.13  2.161  0  3.697  0.894  2.037  0.771  0.551  4.258  3.823  3.453  5.729  1.955  0.357  2.209  1.68  2.121  0.393  1.953  2.949  2.677  1.351  5.179  3.022  2.222  2.916  2.551  0  3.313  3.341  5.913  3.968  4.244  
 
 PTPRF  5792  1  43996546  44089343  1p34  4.458  3.282  3.593  3.508  4.359  3.781  3.632  3.93  3.581  3.001  4.033  4.345  4.607  4.395  4.147  4.329  3.96  1.999  3.881  0.568  0.753  0.613  0.797  0.573  4.133  1.62  3.528  5.101  2.467  2.739  1.742  3.981  4.368  1.795  3.632  5.756  6.345  5.089  5.109  5.44  4.723  2.367  4.206  4.668  2.945  3.66  2.973  4.657  4.601  5.087  4.489  4.439  5.179  3.522  5.174  5.551  4.558  6.031  6.282  5.778  
 
 KDM4A  9682  1  44115796  44171189  1p34.1  2.223  0.916  1.583  1.777  1.389  1.183  2.062  2.259  1.945  2.224  2.74  1.385  2.582  2.441  2.266  1.746  1.757  0.901  2.254  2.12  1.762  1.768  1.276  1.131  1.852  1.761  2.081  2.189  2.071  1.432  2.09  2.314  1.884  0.624  1.126  2.994  2.081  2.246  2.157  2.867  1.465  1.16  2.661  2.528  1.826  2.099  0.875  1.576  1.674  1.998  2.373  1.047  1.8  1.526  1.533  1.966  2.569  1.714  2.396  2.522  
 
 KDM4A-AS1  100132774  1  44165408  44173012  1p34.1  0.681  0.123  0.758  0.445  0.486  0.589  1.03  0.676  0.256  0.989  1.033  0.272  0.217  0.418  0.594  0.508  0.37  0.166  1.284  0.562  0.485  0.734  0.852  0.454  0.511  0.486  1.199  0.63  0.808  0.1  1.335  0.885  0.67  0.467  0.398  1.58  1.035  0.851  0.889  0.955  0.492  0.576  1.471  0.74  0.187  0.267  0.957  0.318  0.442  0.46  0.72  0.604  1.211  0.437  0.673  0.475  0.877  0.485  1.022  0.592  
 
 ST3GAL3  6487  1  44173203  44396837  1p34.1  1.145  1.075  2.573  3.2  1.042  1.9  2.822  2.45  3.087  2.752  2.151  0.472  0.43  1.238  1.098  0.66  0.484  0.28  3.754  3.425  1.591  2.102  1.765  1.17  1.327  1.795  3.049  1.784  1.985  1.14  2.392  1.294  1.631  1.155  0.833  3.06  2.422  3.232  3.077  2.263  1.154  1.748  2.315  1.123  0.634  1.182  2.075  1.431  1.66  2.546  2.64  1.2  1.648  2.917  2.284  1.383  1.911  1.726  2.528  1.847  
 
 LOC101929592  101929592  1  44175075  44193014  1p34.1  0  0  0  0  0  0  0  0  0  0  0  0  0  0  0  0  0  0  0  0  0  0  0  0  0  0  0.125  0.243  0  0  0  0  0  0  0.322  0  0  0  0  0  0  0  0  0  0  0  0  0  0  0  0  0  0  0  0  0  0  0  0  0  
 
 ARTN  9048  1  44398991  44402912  1p33-p32  1.227  1.153  1.481  1.674  2.871  0.476  0.584  0.702  0.427  1.15  0.761  0.577  1.071  2.175  0.729  0.148  0.18  0.684  0.465  0.159  0.133  0.376  0.81  0.161  0.125  0  0.817  0.189  0.128  0.143  0.104  0.108  0.252  0.131  0.153  0.987  0.427  3.141  0.696  1.947  4.071  2.256  0.164  0.273  0.385  1  0.337  1.413  1.672  2.089  0.394  1.044  2.705  1.829  0.243  0.131  0.546  3.705  0.154  0  
 
 IPO13  9670  1  44412477  44433694  1p34.1  1.788  0.54  2.03  2.056  2.354  1.527  3.544  2.893  2.254  3.275  3.431  1.374  2.505  1.656  2.466  1.436  1.219  0.097  3.242  1.944  2.375  2.22  1.549  2.012  1.932  2.394  2.963  2.605  2.791  2.231  3.557  3.406  2.626  1.125  1.36  3.942  2.878  3.129  2.078  3.204  2.466  1.904  2.581  1.277  1.577  2.258  1.162  1.821  2.324  2.374  3.099  1.624  3.008  2.5  1.777  2.016  3.981  1.433  2.837  1.893  
 
 DPH2  1802  1  44435652  44439043  1p34  2.585  2.217  2.317  3.427  2.606  1.827  4.247  3.175  2.49  2.912  3.822  2.796  3.554  3.554  3.502  3.297  2.545  2.004  4.893  4.221  3.91  3.437  3.367  3.672  3.182  2.407  4.102  4.162  3.557  2.74  3.728  4.069  4.346  2.536  2.179  4.431  3.939  2.906  2.811  4.354  2.687  2.789  4.516  2.52  2.928  2.926  3.124  2.743  2.783  4.014  3.197  2.01  3.522  2.374  2.725  2.966  2.957  3.04  4.367  2.931  
 
 ATP6V0B  533  1  44440319  44443972  1p32.3  4.83  2.972  4.703  6.374  5.996  4.852  7.25  5.71  5.776  5.745  6.479  5.439  4.805  5.816  4.745  5.409  5.604  0.586  7.123  7.413  5.173  5.534  6.778  6.043  5.174  4.936  6.136  5.427  5.585  5.596  7.179  6.608  5.02  3.587  3.771  6.914  5.358  5.259  5.885  5.896  5.026  5.372  5.787  4.177  3.919  5.435  5.898  4.72  4.494  6.635  5.608  3.611  5.04  4.688  4.2  4.912  5.261  5.136  6.007  4.612  
 
 B4GALT2  8704  1  44444873  44456843  1p34-p33  3.48  0.905  4.613  4.614  3.412  3.604  4.752  4.604  4.047  4.843  4.669  2.756  3.379  3.603  3.808  4.01  2.933  0.172  2.794  2.58  3.607  1.96  3.169  3.369  3.938  2.853  4.194  3.305  3.51  3.341  4.435  4.744  3.983  2.5  3.185  5.398  4.087  4.123  3.544  4.104  3.524  4.188  4.55  3.358  3.031  3.219  2.018  3.543  3.881  4.685  4.564  3.237  4.669  3.254  3.856  3.994  4.041  4.015  4.986  3.958  
 
 CCDC24  149473  1  44457279  44462198  1p34.1  1.609  0.24  1.019  1.545  2.501  0.641  1.934  1.285  1.074  1.528  1.408  0.693  1.164  2.358  1.844  1.54  1.254  0.226  1.055  0.142  0.456  0.338  0.701  0.546  1.069  0.942  1.037  1.65  1.275  0.484  1.814  1.829  0.933  0.376  1.328  2.948  1.718  1.321  1.568  1.909  2.108  1.312  2.799  0.552  1.02  1.008  0.366  1.35  1.233  3.009  1.473  1.565  1.43  0.987  2.077  1.241  1.269  2.089  2.24  1.155  
 
 SLC6A9  6536  1  44462154  44497164  1p33  1.443  0.471  3.278  3.923  1.386  0.278  2.883  1.978  3.996  1.918  2.148  0.954  0.466  1.258  2.159  0.319  1.429  0.377  0.746  0.33  1.765  0.708  3.54  1.202  0.264  0.344  2.269  0.103  0.09  0.123  0.07  0.07  1.448  0.691  0.689  3.356  1.325  1.051  1.75  1.756  1.502  0.787  1.207  0.437  1.843  1.547  0.35  0.646  0.676  0.973  2.433  1.256  0.089  0.419  0.41  0.393  2.534  0.345  1.663  0.142  
 
 KLF17  128209  1  44584521  44600809  1p34.1  0  0.141  0.369  0.319  0  0  0.241  0.092  0.171  0.277  0.091  0.035  0  0.017  0  0  0  0.057  0.03  0  0  0.053  0.458  0  0.103  0  0.074  0.038  0  0.055  0.084  0.066  0  0.228  0  0.706  0  0  0.215  0  0  0.264  0.036  0  0  0.03  0.03  0  0  0  0.402  0.215  0  0.143  0  0.053  0.08  0  0  0  
 
 DMAP1  55929  1  44679124  44686351  1p34  2.888  1.607  2.146  3.217  2.992  2.48  3.884  3.323  3.176  3.27  3.727  2.286  2.54  3.206  2.932  2.853  2.541  0.889  5.008  2.088  2.255  3.578  2.512  2.514  2.069  2.27  4.116  2.695  2.764  2.198  3.616  3.228  2.827  1.488  1.769  4.035  3.369  3.014  2.64  3.343  3.067  2.721  4.118  2.351  2.654  2.877  2.083  3.224  2.214  4.94  3.242  2.004  3.358  1.956  3.009  2.627  2.85  2.867  3.921  2.789  
 
 ERI3  79033  1  44686741  44820951  1p32  4.715  1.202  4.304  4.951  4.792  4.084  6.456  5.735  4.724  5.683  5.907  4.086  4.548  5.499  4.34  4.775  4.798  0.406  6.496  5.057  4.049  5  4.55  4.218  4.795  3.948  5.888  4.45  5.149  4.325  5.721  5.889  4.875  3.313  4.018  5.927  5.172  5.414  4.801  5.617  4.921  4.18  6.24  3.744  4.452  5.022  5.481  4.446  4.391  5.995  5.208  2.945  5.002  3.669  4.681  4.803  4.734  5.364  5.709  4.849  
 
 ERI3-IT1  100874278  1  44688266  44709955  -  0  0  0.694  0  0.341  0  0.576  0.748  0.417  1.078  0.986  0.306  0.744  0.576  0.887  0.579  0.409  0  0.684  0.39  0  0.361  0.911  0.557  0  0.565  1.118  0.595  0.847  0.224  0.58  0.496  0.387  0  0.32  0.835  0.928  1.042  0.633  0.577  0.689  0.809  0.949  0.407  0  0.904  0  0.491  0.619  0.695  0.815  0.715  0.954  0.494  0.49  0  1.063  0.775  1.085  0.356  
 
 RNF220  55182  1  44870959  45117396  1p34.1  2.537  1.137  2.869  3.341  2.532  2.438  3.822  3.559  3.113  3.659  3.897  3.084  3.235  3.316  3.604  3.282  3.032  0.39  4.569  3.412  2.635  3.779  3.221  2.929  3.092  2.724  3.897  3.265  3.518  3.157  3.579  3.372  3.34  1.795  2.117  3.75  3.503  3.163  3.008  3.751  3.06  2.544  3.548  2.716  2.367  2.491  1.767  2.873  2.614  4.072  2.793  2.228  3.447  2.463  3.199  3.138  3.246  3.067  4.537  2.957  
 
 TMEM53  79639  1  45118918  45140280  1p34.1  0.414  0.333  0.745  1.986  0.756  0.752  1.92  1.122  2.221  0.758  1.33  1.7  1.006  1.219  0.79  1.697  1.313  0.482  1.283  0.962  0.37  0.856  1.904  1.292  0.56  1.229  1.464  1.781  1.409  1.913  1.851  1.26  1.234  1.257  0.875  1.55  1.352  1.353  0.97  1.102  0.711  1.253  2.008  0.815  0.619  0.876  0.97  0.806  0.794  1.783  0.95  1.346  1.066  0.942  1.593  0.882  0.611  1.425  1.043  0.675  
 
 C1orf228  339541  1  45140393  45191263  1p34.1  0  0.098  0.152  0.351  0.167  0  0.347  0.181  0.312  0.293  0.139  0.195  0.124  0.218  0.266  0.102  0.141  0  5.638  4.011  0  5.2  1.08  0.24  0  0  0.607  0.065  0  0.504  0  0  0  0.31  0.201  0.228  0.154  0.225  0.194  0.173  0.119  0.459  0.65  0  0  0.246  0.592  0  0  0.174  0.128  0.664  0.045  0.116  0.191  0  0  0  0  0  
 
 KIF2C  11004  1  45205489  45233438  1p34.1  3.232  3.448  2.841  3.742  2.698  3.552  4.237  3.462  1.497  4.191  5.049  2.681  3.03  4.617  3.223  3.492  3.954  2.332  5.544  3.083  4.121  3.485  2.833  2.337  2.669  2.446  3.924  2.895  3.117  2.728  4.184  3.924  3.436  1.796  2.588  4.314  4.116  3.976  3.134  4.504  3.524  2.703  4.381  3.508  2.383  3.282  2.873  3.495  3.553  5.184  3.696  2.099  3.455  2.181  3.52  2.914  4.013  3.595  4.535  3.487  
 
 RPS8  6202  1  45241245  45244412  1p34.1-p32  9.275  9.909  8.31  8.187  9.375  9.022  9.635  9.106  8.936  9.167  9.491  9.103  8.617  10.244  8.463  9.121  10.033  10.023  11.312  11.184  10.159  10.086  10.102  9.496  9.338  7.821  9.417  7.879  8.194  8.281  9.558  9.548  8.257  7.122  8.67  9.662  8.604  8.86  10.212  8.472  8.574  8.89  10.049  9.95  8.028  8.401  9.772  8.562  8.547  11.214  8.882  7.266  8.988  7.777  9.426  9.015  8.482  9.603  9.827  9.011  
 
 BEST4  266675  1  45249256  45253426  1p33-p32.3  0  0.054  0.115  0  0.039  0.144  0.143  0.182  0.287  0.063  0.105  0.108  0  0.226  0.093  0.18  0.123  0.07  0.152  0.044  0  0.24  0.376  0  0  0  0.149  0.097  0.058  0.097  0  0.066  0  0.148  0.061  0.08  0  0.092  0.121  0  0  0.392  0.073  0.079  0  0.069  0.068  0.103  0  0.743  0.081  0.684  0.09  0.222  0.338  0  0.136  0.084  0.138  0.163  
 
 PLK3  1263  1  45266035  45271667  1p34.1  0.477  0.331  1.156  0.989  0.344  0.829  2.068  1.999  1.498  1.383  1.922  0.868  1.65  3.236  1.375  1.744  1.235  0.096  2.539  0.482  1.385  1.692  1.988  1.912  1.557  2.961  1.113  1.796  2.381  0.716  3.362  2.809  1.109  0.339  0.647  2.176  2.479  1.451  2.583  1.484  1.259  1.284  1.588  1.358  0.466  1.861  0.623  1.302  1.071  2.219  1.537  1.229  0.688  1.035  1.573  1.375  1.192  1.228  2.155  2.403  
 
 TCTEX1D4  343521  1  45271581  45272957  1p34.1  0  0.067  0.396  0  0.153  0.103  0.321  0.639  0.321  0.321  0.587  0.078  0.418  0.432  0.402  0.611  0.219  0.235  0.252  0.271  0  0.514  0.749  0.848  0  0.87  0.198  0.547  0.334  0.149  0.637  0.258  0.117  0.219  0.109  0.422  0.544  0.338  1.186  0.298  0.208  0.704  0.086  0.229  0  0.466  0.056  0.394  0  0.097  0.197  1.094  0.182  0.227  0.278  0.23  0.217  0.144  0.473  0.3  
 
 BTBD19  149478  1  45274153  45279801  1p34.1  0  0  0.938  0  0  0.107  0.488  1.393  0.588  0.786  1.216  0.132  0.133  0.266  0.176  0.738  0.103  0  0.069  0.127  0  0.327  0.86  0.975  1.217  0.89  0.341  1.418  2.137  0.199  2.143  1.762  0  0  0.403  0.24  0.882  0.443  1.384  0  0  0.32  0.098  0.385  0  0.451  0.168  0.216  0.262  0.298  0.112  0.84  0.208  0.953  0.51  0.42  0.428  0.227  0.483  0.341  
 
 PTCH2  8643  1  45285515  45308616  1p34.1  0.079  0.055  0.237  0.097  0.127  0.084  0.083  0.205  0.336  0.308  0.375  0.045  0.074  0.27  0.342  0.091  0.075  0.103  0.129  0.156  0.123  0.213  0.685  0.242  0.059  0.065  0.317  0.105  0.222  0.104  0.261  0.43  0.04  0.121  0.152  0.207  0.178  0.204  0.352  0.199  0.1  0.24  0.66  0.268  0.157  0.135  0.055  0.191  0.104  0.402  0.113  0.537  0.185  0.284  0.188  0.123  0.104  0  0.079  0.06  
 
 EIF2B3  8891  1  45316193  45452394  1p34.1  2.095  3.194  2.054  2.372  2.439  2.06  4.171  2.77  1.935  2.988  3.375  1.933  2.712  2.47  2.003  2.153  1.571  2.062  4.476  3.669  3.998  3.133  2.546  2.572  2.168  1.997  2.827  2.042  2.538  1.587  3.637  4.167  2.149  1.168  1.675  3.18  3.116  2.645  2.823  3.031  2.247  1.733  3.669  1.753  1.647  2.679  3.524  2.294  1.967  4.08  3.355  1.362  2.326  1.27  2.197  2.439  2.056  2.21  3.121  2.447  
 
 HECTD3  79654  1  45468219  45477027  1p34.1  1.984  0.905  2.414  2.322  2.484  1.431  3.571  2.616  1.928  3.458  3.237  2.321  3.242  2.346  2.919  1.779  2.096  0.176  2.481  2.056  2.915  2.224  1.622  1.784  2.492  2.428  3.349  2.944  3.004  2.922  3.312  3.314  3.043  1.238  1.784  3.506  3.262  2.859  2.577  2.468  1.828  1.702  2.776  2.103  1.67  2.479  1.883  2.457  2.681  2.807  3.551  1.695  3.328  2.392  2.683  2.592  3.364  2.405  4.14  2.857  
 
 UROD  7389  1  45477804  45481341  1p34  3.267  4.585  3.444  4.059  3.764  3.471  5.495  4.394  3.542  4.768  5.074  3.132  3.761  4.32  3.911  3.233  4.003  4.032  5.298  4.18  6.207  3.788  2.941  3.19  4.21  3.763  5.371  4.148  4.999  3.63  4.966  5.399  4.605  2.883  2.804  4.236  4.795  4.896  4.653  3.835  3.246  3.849  4.583  3.402  3.069  3.616  4.684  4.068  3.03  6.299  4.82  2.843  4.737  4.101  4.336  3.602  4.143  4.182  5.326  4.575  
 
 ZSWIM5  57643  1  45482075  45672250  1p34.1  1.011  0.07  0.153  0.064  0.467  0.391  0.165  0.155  0.57  0.142  0.577  0.695  0.545  0.249  1.011  0.101  0.213  0.884  0.064  0.06  0.071  0.505  0.474  0  0.098  0.018  0.129  0.021  0.139  0.685  0.03  0.101  0.037  0.228  0.263  1.575  0.093  0  0.247  0.795  0.439  0.637  1.357  0.187  0.065  0.066  1.424  0.088  0.103  0.19  0.395  0.773  0.043  0.116  0.075  0.085  0.036  0.039  0  0  
 
 LINC01144  400752  1  45769581  45771291  1p34.1  0.256  0.114  0.262  0  0.46  0.227  0.162  0.098  0.354  0.233  0.12  0.092  0.305  0.241  0.296  0.281  0.366  0  0.364  0.129  0  0.509  0.519  0.096  0.104  0.106  0.454  0.328  0.234  0.109  0.478  0.228  0.257  0.382  0.264  0.262  0.117  0  0.185  0.333  0.218  0.632  0.313  0.136  0  0.186  0.057  0.164  0.274  0.274  0.098  0.367  0.067  0.173  0.331  0  0.156  0.157  0.21  0.083  
 
 HPDL  84842  1  45792544  45794346  1p34.1  2.33  0.118  0.283  2.056  0.219  0.283  0.059  0.34  0.251  0.252  0.227  1.994  3.914  0.166  0.185  2.504  0.56  3.486  2.832  3.884  0  1.754  2.218  2.19  0.142  0.798  1.104  1.297  0.255  0.435  0.902  0.368  0.132  0.416  0.749  2.05  0.215  0.256  0.295  2.322  0.267  3.224  0.074  1.073  0.263  0.445  1.565  1.649  0.238  3.79  0.094  1.384  0.206  0.44  0.473  0.25  0.151  0.142  0.287  0.11  
 
 MUTYH  4595  1  45794913  45806142  1p34.1  1.678  1.181  1.156  1.424  1.606  1.249  1.842  1.553  1.563  2.562  1.84  1.82  2.11  2.861  2.087  2.052  3.28  1.262  4.571  1.708  1.983  3.375  2.04  2.142  1.59  1.607  2.735  2.075  2.257  1.318  3.141  2.559  2.223  0.678  1.01  2.583  3.04  1.909  1.361  3.05  2.157  2.071  2.336  2.266  1.32  1.513  2.724  1.136  1.611  2.802  1.462  1.345  1.644  1.173  2.496  1.635  1.834  2.093  2.893  1.758  
 
 TOE1  114034  1  45805341  45809650  1p33  1.958  1.356  1.537  2.442  1.943  1.655  3.065  2.49  1.452  2.35  3.084  1.986  2.713  3.049  2.499  2.305  2.492  1.265  4.388  3.245  2.727  3.32  2.108  1.967  2.34  1.603  2.939  2.513  2.651  1.757  3.113  2.953  2.941  1.564  1.59  2.901  2.159  1.421  2.712  3.124  1.866  1.786  2.775  2.101  1.292  1.866  2.39  2.198  1.609  3.443  1.877  1.493  2.302  1.292  2.128  1.839  2.331  1.955  2.981  1.867  
 
 TESK2  10420  1  45809554  45956840  1p32  1.353  0.608  0.277  0.475  1.536  0.209  0.476  0.437  0.35  0.43  0.73  0.57  0.734  1.352  0.907  0.682  0.872  0.632  1.477  0.424  0.407  1.255  1.2  0.357  0.117  1.789  1.854  0.956  3.188  2.885  3.375  2.09  1.116  0.944  0.132  0.661  0.472  0.151  0.6  0.715  0.489  0.232  1.085  0.23  0.742  0.519  0.699  0.456  0.113  1.265  0.762  0.576  0.58  0.15  0.186  0.207  0.253  0.54  1.165  0.101  
 
 CCDC163P  126661  1  45959597  45965751  1p34.1  0.56  0.348  0.579  0.581  0.788  0.852  0.722  0.565  0.71  0.46  1.407  0.754  1.149  0.911  1.357  0.427  0.895  0.972  2.32  0.696  0.991  1.593  1.518  0.667  0.242  0.797  0.413  0.564  0.236  0.159  0.522  0.579  0.266  0.525  0.437  1.487  1.267  0.632  0.473  0.869  0.684  0.593  1.528  0.562  0.497  0.595  0.401  0.595  0.25  1.492  1.232  0.842  0.664  0.519  0.367  0.653  0.302  0.857  1.695  0.321  
 
 MMACHC  25974  1  45965855  45976739  1p34.1  1.48  0.524  0.843  1.357  1.401  1.59  1.672  1.53  0.505  1.354  1.352  1.453  2.035  1.672  1.99  1.088  1.69  0.473  1.836  1.742  1.915  1.448  1.197  2.117  1.001  1.848  1.033  2.129  0.962  1.005  2.03  2.478  1.212  1.156  0.792  2.084  1.408  1.488  0.486  1.765  1.333  0.49  2.743  1.813  1.208  1.552  1.284  1.475  1.089  2.001  1.558  1.171  1.604  0.525  1.093  1.179  0.868  1.867  1.923  1.292  
 
 PRDX1  5052  1  45976706  45988562  1p34.1  7.294  7.191  6.12  6.858  7.792  6.517  8.827  6.963  7.232  7.069  9.208  7.44  7.673  9.265  6.756  7.847  7.866  6.941  9.239  7.92  7.168  7.891  6.946  6.91  7.135  6.314  6.931  6.744  7.328  5.963  5.273  6.723  0.25  1.652  8.14  8.144  7.747  7.278  8.704  8.005  7.803  8.483  8.747  7.683  7.136  7.144  7.254  6.887  7.719  9.255  8.387  6.199  7.832  6.899  8.187  8.148  7.377  7.642  9.155  7.846  
 
 AKR1A1  10327  1  46016454  46035723  1p33-p32  3.38  1.827  3.498  4.458  4.59  2.813  5.681  4.699  4.267  4.485  5.166  4.41  4.574  5.244  4.807  4.465  5.11  2.408  6.36  4.293  3.657  4.791  4.774  4.505  4.003  3.732  4.74  4.119  6.031  3.135  4.918  4.267  3.614  2.528  3.034  4.401  4.071  3.684  3.838  3.238  4.281  4.026  5.145  3.577  3.967  3.371  4.54  3.15  3.76  5.936  4.617  3.336  4.163  2.999  4.391  4.619  3.965  2.869  5.336  4.115  
 
 NASP  4678  1  46049659  46084578  1p34.1  3.38  4.368  2.279  3.445  3.285  3.392  4.427  4.053  2.901  4.112  4.875  3.574  3.513  4.762  3.829  4.467  4.529  4.381  6.68  4.906  4.643  5.482  3.915  3.714  3.534  2.886  3.484  3.248  3.005  2.796  4.368  4.262  3.692  2.359  3.315  3.872  3.976  3.346  3.586  4.275  2.487  3.359  5.287  4.507  3.866  3.548  4.527  3.253  3.483  5.633  3.362  2.549  3.71  2.609  3.952  3.265  3.622  3.561  4.499  3.756  
 
 CCDC17  149483  1  46085715  46089731  1p34.1  0  0.377  0.219  0  0.373  0.058  0.296  0.122  0.14  0.33  0.361  0  0.284  0.316  0.46  0.352  0.167  0.307  0.703  0.295  0.184  0.385  0.597  0.063  0.163  0.159  0.455  0.154  0.191  0.462  0.357  0.347  0.195  0.155  0.085  0.336  0.323  0.429  0.425  0.305  0.226  0.231  0.611  0.241  0  0.171  0.686  0.232  0.321  0.457  0.227  0.623  0.223  0.182  0.229  0.183  0.242  0  1.053  0  
 
 GPBP1L1  60313  1  46092975  46152302  1p34.1  3.202  3.001  2.466  3.353  2.509  1.45  3.249  3.061  3.027  2.907  3.57  2.781  3.481  4.051  3.327  3.278  3.378  2.636  4.376  2.83  3.115  2.966  2.648  2.043  3.106  2.475  2.92  3.103  3.007  2.596  3.422  3.493  2.654  1.647  1.751  3.416  3.314  3.069  3.039  3.525  2.733  1.749  4.166  2.817  2.521  2.802  2.366  2.666  2.411  4.205  3.393  1.926  3.031  1.675  2.829  2.684  3.224  2.361  4.115  2.955  
 
 RPS15AP10  728963  1  46111451  46112357  1p34.1  2.053  2.389  1.05  0.573  1.292  1.188  1.287  0.783  1.067  1.227  1.064  1.475  1.178  2.132  1.176  2.19  2.237  3.474  2.059  4.004  0.681  3.534  1.705  2.123  0.917  0.784  1.211  0.679  0.96  0.532  1.373  1.309  0.601  1.351  2.004  0.735  0.825  1.005  3.206  0.4  1.109  1.588  2.001  0.852  0.572  1.061  2.767  1.278  0.649  1.515  0.684  0.367  1.625  1.222  2.795  1.331  0.82  1.251  1.596  0.411  
 
 TMEM69  51249  1  46153846  46160108  1p34.1  2.72  4.276  2.427  3.738  3.722  2.548  3.086  2.809  2.591  3.07  3.405  2.405  3.438  4.278  2.925  3.683  3.689  4.236  4.91  4.295  4.157  3.297  3.993  3.085  3.295  2.959  3.309  2.967  3.266  2.562  4.213  4.121  3.135  2.765  2.039  3.108  2.753  3.358  3.796  3.327  2.945  2.58  4.825  3.224  2.95  2.75  2.17  2.81  2.651  5.082  3.164  2.508  2.692  1.871  3.212  2.736  3.266  3.136  4.431  3.282  
 
 IPP  3652  1  46159997  46216485  1p34-p32  0.757  0.721  0.717  0.875  0.932  0.644  0.977  1.196  0.725  1.51  1.568  0.773  1.35  1.474  1.274  0.658  0.751  0.684  1.075  0.785  0.874  0.912  0.788  0.417  0.728  1.389  1.031  0.706  0.981  0.378  1.465  1.142  0.532  0.406  0.694  1.777  1.12  1.132  1.077  1.472  1.056  0.555  1.309  1.216  0.671  0.879  0.553  0.33  1.058  0.599  1.402  0.849  0.668  0.619  0.707  0.925  0.894  0.499  1.097  1.074  
 
 MAST2  23139  1  46269284  46501796  1p34.1  2.409  0.986  2.571  3.231  2.393  2.067  3.508  3.412  2.318  3.801  3.587  1.978  2.948  3.136  3.341  2.451  2.284  0.318  2.364  0.905  2.293  2.149  1.175  1.601  1.795  2.474  3.656  3.012  2.769  2.17  3.035  2.999  2.887  1.079  1.614  3.28  3.641  3.424  2.3  3.591  2.362  1.814  3.84  2.364  2.041  2.707  1.248  2.278  3.47  2.718  3.65  1.822  2.992  1.714  2.374  2.964  3.311  2.393  4.041  2.77  
 
 PIK3R3  8503  1  46505811  46642167  1p34.1  2.74  0.409  0.871  2.662  1.851  0.559  1.726  1.503  1.18  1.527  2.444  0.605  1.232  0.287  1.263  1.204  0.846  0.876  3.43  0.765  0.687  2.723  1.598  1.894  0.506  3.338  1.419  2.926  3.858  0.662  3.085  1.64  0.652  0.437  0.16  1.829  0.717  0.728  0.843  2.06  2.14  0.808  2.142  1.994  1.911  2.022  0.507  0.547  0.719  0.73  2.066  0.586  0.816  0.54  0.491  0.794  0.746  0.805  0.958  1.104  
 
 LOC101929626  101929626  1  46599042  46604752  1p34.1  0  0.357  0  0  1.113  0  0  0.284  0  0  0.934  0  0  0  0  0.478  0  0.755  0.669  0  0  0.534  1.138  0.703  0  0  0  0  0  0  0.454  0  0  0  0  0.556  0  0  0  0  0  0.33  0  0.724  0  0  0  0  0  0  0  0  0.303  0  0.475  0  0  0  0  0  
 
 TSPAN1  10103  1  46640748  46651634  1p34.1  0.499  0.078  0.239  0.605  2.245  0.362  0.43  0.12  0.383  0.251  0.885  2.019  2.632  1.713  2.338  5.867  2.072  0.287  0.382  1.158  0  0.694  0.473  0.49  0.226  0.544  0.653  0.962  0.965  0.237  0.998  0.436  0.23  0.275  0.193  0.854  0.671  0.486  0.879  0.496  5.619  1.388  0.249  0.495  1.721  2.958  2.582  0.429  4.642  2.182  3.543  1.008  1.019  0.564  4.03  2.542  0.307  0.848  4.692  2.549  
 
 POMGNT1  55624  1  46654352  46685977  1p34.1  1.907  1.485  2.769  3.297  2.845  2.724  3.816  3.963  3.587  3.86  4.302  2.461  3.108  3.288  3.175  2.97  2.731  0.85  3.993  1.645  2.866  2.64  1.502  2.478  3.117  3.793  3.438  4.139  4.605  2.67  5.139  4.749  4.388  2.538  2.103  4.426  4.05  3.756  3.411  4.041  3.484  2.905  3.806  2.404  2.403  3.166  3.443  2.988  3.168  4.245  4.258  2.818  3.293  2.88  3.367  3.988  3.406  3.526  4.273  3.406  
 
 LURAP1  541468  1  46669005  46686928  1p34  0  0  0.456  0.646  0.585  0.634  0.1  0.333  0.545  0.328  0.254  0  0  0.213  0  0  0  0  0.132  0  0.111  0.207  0.444  0  0  1.951  0.81  0.644  1.453  0.741  2.408  1.462  1.306  0.826  0  0  0.326  0.313  0.062  0  0  0.298  0  0  0  0.234  0.052  0.541  0.8  1.359  0.137  0.128  0.062  0.084  0.881  0.66  0.108  0.698  0.828  0.643  
 
 RAD54L  8438  1  46713366  46744145  1p32  2.194  0.625  1.273  2.259  1.444  2.187  2.648  2.196  0.816  2.707  3.013  1.442  2.486  2.383  2.653  2.717  2.616  0.553  4.878  2.246  2.766  3.532  1.626  2.032  2.031  1.781  2.813  2.479  2.331  1.678  2.957  2.512  2.698  1.204  1.402  2.618  2.884  2.087  1.898  3.044  1.809  1.771  2.954  2.105  1.487  1.917  1.897  2.614  2.269  3.896  2.548  1.476  2.238  1.356  2.456  1.679  2.375  2.102  3.112  1.947  
 
 LRRC41  10489  1  46744071  46769038  1p34.1  2.336  0.636  3.263  4.108  3.24  2.9  4.255  4.305  3.685  4.222  4.554  2.128  4.395  3.051  3.921  2.404  2.341  0.183  4.419  2.29  3.499  3.347  2.913  2.591  2.683  3.404  3.402  3.39  3.926  2.159  4.593  4.577  3.661  2.192  2.323  4.064  3.55  3.724  3.551  3.57  3.348  3.737  3.506  3.607  2.152  2.935  1.935  3.14  1.907  4.104  3.956  2.179  3.339  2.781  3.119  3.854  4.702  2.996  4.057  0.493  
 
 UQCRH  7388  1  46769284  46782449  1p34.1  6.657  8.31  5.216  6.074  7.209  6.585  7.533  6.482  5.662  6.558  7.263  7.016  6.688  8.356  5.823  6.924  7.91  9.876  8.973  10.12  8.199  7.934  7.365  7.678  7.324  5.601  6.465  5.822  6.795  5.807  8.072  7.795  6.995  5.995  6.536  7.387  6.147  6.36  8.212  6.66  6.458  6.296  8.063  7.399  6.314  1.337  8.001  6.274  0.541  8.848  6.647  5.061  6.309  5.841  6.678  7.167  5.075  7.248  7.52  0  
 
 NSUN4  387338  1  46805848  46830824  1p34  1.913  0.853  1.527  2.368  1.839  1.164  2.398  1.591  1.591  1.785  2.029  1.909  1.97  2.475  1.557  1.552  1.875  2.068  2.908  2.645  2.189  2.22  2.292  1.892  1.633  1.692  2.624  1.82  1.763  1.454  2.749  2.156  1.942  1.262  1.162  2.262  1.843  1.315  2.178  1.943  1.412  1.161  2.94  1.959  1.228  1.569  1.689  1.872  1.812  2.679  1.678  1.07  1.626  1.31  2.105  1.98  2.069  1.595  2.956  1.732  
 
 FAAH  2166  1  46859938  46879520  1p35-p34  2.46  0  0.123  0.487  2.386  0.166  1.526  0.339  0.369  0.425  1.076  2.681  2.613  2.517  2.67  2.458  1.79  0.163  0.789  0.562  0.205  0.766  0.667  0.478  0  0.089  0.248  0.172  0.128  0.242  0.271  0.361  0  0.099  0.218  1.959  0.589  0.145  0.278  1.183  0.13  0.669  0.435  1.372  0.792  1.566  1.735  0.433  1.442  1.069  0.38  1.468  0.165  1.149  0.096  0.13  0.961  0.296  2.28  0.487  
 
 FAAHP1  729041  1  46899498  46911374  1p33  0  0  0  0  0.241  0  0.167  0.07  0.293  0.113  0.114  0  0.157  0.197  0  0  0  0  0  0  0.17  0.122  0.864  0  0  0.16  0.084  0.123  0  0.175  0.409  0.179  0  0.293  0  0.614  0.113  0  0.242  0.252  0  0.353  0.317  0  0  0.116  0  0  0  0.352  0.092  0.392  0.304  0.659  0.375  0  0.167  0  0.414  0.157  
 
 LINC01398  101929651  1  46912344  46915376  1p33  0  0  0.296  0  0  0  0  0  0.171  0  0.074  0  0  0.074  0  0  0  0.166  0  0  0  0  0.355  0  0  0.096  0.086  0.077  0  0.265  0  0  0  0  0  0.126  0  0  0  0  0  0.238  0  0  0  0  0  0  0  0.116  0  0  0  0.161  0.074  0  0  0  0.224  0  
 
 DMBX1  127343  1  46972667  46979886  1p33  0.469  0.043  0.156  0  0.865  0.108  0.102  0.441  0.225  0.095  1.111  0.539  0  0.805  0.647  1.442  0.151  0.069  0.036  0.058  0.907  0.116  0.513  0  0  0.032  0.083  0.027  0.26  0.455  0.387  0.071  0.053  0.205  0.624  0.607  0.048  0.291  0.094  0.651  0.92  0.475  0.864  0.49  0.293  1.077  0.03  0.48  0.805  0.789  0.036  0.464  0.319  0.636  0.09  0.097  1.436  0.347  0.078  0  
 
 MKNK1-AS1  100507423  1  47004367  47035927  1p33  0  0  0.206  0  0.05  0.105  0  0.076  0.123  0.11  0.131  0.052  0.17  0.088  0.144  0.108  0.053  0  0.031  0.079  0  0.076  0.456  0  0  0.071  0.073  0.065  0.075  0.033  0  0.051  0.131  0.247  0  0.214  0.076  0.109  0.066  0  0.106  0.192  0.019  0.122  0  0.1  0  0.061  0.093  0  0.137  0.154  0.112  0.073  0.05  0  0.159  0.144  0.173  0.064  
 
 KNCN  148930  1  47011315  47016887  1p33  0  0  0.053  0  0  0  0  0  0.179  0  0.032  0  0  0  0  0.037  0  0  0  0  0  0.074  0.517  0  0  0  0.055  0.052  0  0.062  0  0  0  0.239  0  0  0  0  0.04  0  0  0  0.024  0  0  0.035  0.022  0  0  0  0.031  0.295  0.042  0  0  0  0  0  0  0  
 
 MKNK1  8569  1  47023078  47069966  1p33  2.369  2.604  1.068  1.551  2.723  0.988  2.295  2.424  2.035  2.483  3.227  1.654  2.084  2.931  2.167  2.404  2.108  2.564  3.393  3.162  2.175  2.074  2.078  1.441  1.151  2.151  3.106  2.214  2.161  2.14  2.546  2.099  2.071  1.081  1.356  2.929  2.221  1.946  2.489  2.157  2.621  1.786  3.675  2.059  0.868  2.168  2.779  2.185  2.088  3.36  2.739  1.379  2.307  1.378  2.103  1.406  2.294  1.193  3.402  2.017  
 
 MOB3C  148932  1  47073386  47082563  1p33  0.55  0.336  0.777  1.15  1.105  0.642  1.952  2.306  1.316  1.912  2.017  0.898  1.476  1.299  1.207  2.297  0.853  0.126  0.698  0.573  0.992  0.584  1.444  0.835  0.783  1.337  1.765  1.304  0.979  0.963  1.251  1.191  1.501  0.655  0.352  1.497  1.047  1.203  1.696  1.104  2.23  0.682  1.467  0.274  0.507  0.779  0.557  1.228  1.291  1.964  2.573  0.994  1.222  1.02  1.204  1.54  1.678  1.529  1.325  1.034  
 
 ATPAF1  64756  1  47100710  47134099  1p33-p32.3  3.726  4.082  2.561  2.583  4.045  2.254  4.181  2.985  3.687  3.642  3.952  2.944  2.721  4.489  3.695  3.08  3.188  4.831  4.777  3.703  1.83  3.751  3.359  2.562  1.91  2.777  3.432  2.926  3.131  3.279  3.897  3.774  3.151  2.317  2.119  3.576  3.148  3.178  4.02  3.429  2.844  3.02  4.096  3.185  2.47  2.528  3.943  1.989  2.985  3.184  3.748  1.902  3.02  1.808  3.195  3.152  2.479  2.778  3.516  3.54  
 
 TEX38  374973  1  47137504  47139256  1p33  0  0  0.369  0  0.249  0.25  0.465  0.248  0  0.452  0.351  0  0  0.432  0.435  0  0  0  0  0  0  0.203  0  0  0  0.385  0.28  0.265  0.167  0.055  0.283  0.327  0.436  0.363  0.283  0.33  0.254  0.235  0.174  0  0  0.554  0.8  0  0  0.496  0.119  0.263  0.375  0  0.236  0.44  0.513  0  0.212  0.32  0.398  0  0  0  
 
 EFCAB14-AS1  100130197  1  47139707  47157769  1p33  0.639  0.203  0.784  0.869  0.227  0  0.273  0.611  0.811  0.656  0.631  0.65  1.102  0.697  1.546  0.699  0.292  0  0  0  0  0.411  0  0  0.45  0.525  0.586  0.951  1.536  0.956  0.646  0.566  1.075  0.608  0.778  0.465  0.834  0.973  0.581  0.523  0.407  1.421  0.212  0  0  0.896  0  0.774  0  0.243  0.724  0.419  1.371  0.732  0.501  0.663  0.836  0.409  1.656  0.377  
 
 EFCAB14  9813  1  47140830  47184736  1p33  2.481  3.06  2.268  2.587  3.444  1.965  3.242  3.236  2.972  3.507  3.207  2.56  2.871  3.688  3.024  3.232  2.97  2.656  3.434  2.644  2.155  2.963  2.204  2.012  2.645  3.027  2.771  2.644  3.146  1.751  3.299  2.852  2.364  1.747  1.865  3.704  2.781  3.275  3.492  3.188  3.088  1.73  2.667  2.871  2.064  2.99  2.179  2.407  2.978  3.151  3.412  1.79  3.258  2.91  3.374  3.045  3.592  2.703  4.5  3.636  
 
 CYP4B1  1580  1  47264669  47285021  1p34-p12  0.344  0  0.096  0  0.072  0  0  0.036  0.138  0  0  0  0  0.022  0  0.055  0  0  0  0  0  0.168  0.261  0  0  0  0.072  0  0  0.054  0  0  0  0.112  0  0  0  0  0.033  0  0.74  0.358  0.019  0.131  0.745  0.179  0.023  0  0  0  0  0.214  0.031  0.037  0  0  0  0  0  0  
 
 CYP4Z2P  163720  1  47308766  47366147  1p33  0  0  0  0  1.303  0  0  0  0  0  0  0  0  0.035  0  0  0  0.072  0.068  0  0  0.049  0.196  0  0  0  0.049  0.042  0  0.06  0  0  0  0  0  0  0  0  0  0  0  0  0.063  0  0  0  0.028  0  0  0  0  0.102  0  0  0  0.105  0  0  0  0  
 
 CYP4A11  1579  1  47394845  47407156  1p33  0  0  0.12  0  0  0  0  0  0.112  0  0  0  0  0  0  0  0  0  0  0  0  0.036  0.472  0  0  0  0.065  0  0  0.054  0  0  0  0.179  0  0  0  0  0.03  0  0  0.234  0.025  0  0  0  0.03  0  0  0  0  0.353  0  0.04  0.035  0  0  0  0  0  
 
 CYP4X1  260293  1  47489239  47516423  1p33|1  0  0  0.077  0  1.304  0  0  0  0  0.03  0  0  0  0.028  0  0  0  0.049  0  0.051  0  0.101  0.289  0  0  0  0.038  0.039  0  0.066  0  0  0  0.15  0  0.084  0  0  0.044  0  0  0.479  0.021  0.079  0.364  0.551  0.03  0  0  0.036  0  0.307  0  0.061  0.041  0  0  0  0  0.111  
 
 CYP4Z1  199974  1  47533159  47583992  1p33  0  0  0  0  0.331  0  0  0  0  0  0  0  0  0  0  0.056  0  0  0  0.048  0  0.073  0.224  0  0  0  0.048  0  0  0.041  0  0  0  0  0  0  0  0  0  0  0  0  0.029  0  0  0  0.042  0  0  0  0  0.29  0  0  0  0  0  0  0  0  
 
 CYP4A22  284541  1  47603096  47614526  1p33  0  0  0  0  0.046  0  0  0  0.084  0  0.035  0  0.083  0.03  0  0.046  0  0.102  0  0.048  0  0.092  0.394  0  0  0  0.087  0  0  0.043  0  0  0  0.138  0  0  0  0  0.056  0  0.144  0  0.045  0  0  0  0.044  0  0  0  0  0.46  0.069  0.076  0.092  0  0.082  0  0.094  0  
 
 LINC00853  100874253  1  47644921  47646011  -  0  0  0.339  0  1.316  0.229  0  0  0.473  0.232  0  0.259  0.375  0.863  0.279  0.359  0.402  0.47  0.226  0  0  0.389  0.662  0  0.282  0.25  0.583  0  0  0  0.665  0  0.251  0.894  0.445  2.454  0  0  0.176  0  0.772  0.49  1.449  0.381  0  0.177  0.74  0  0.364  0.848  0.163  0.893  0.122  0.525  0.844  0.345  0.289  0  0.444  0.218  
 
 PDZK1IP1  10158  1  47649260  47655771  1p33  0  0  0  0  0.219  0  0.346  0  0  0  0  0.579  2.959  0  1.181  3.421  0  0  0.545  0  0.258  0.071  0  0  0  0  0.409  0  0.395  0.063  0  0  0  0  1.7  4.182  0  0.853  2.834  0  0  0.655  0  4.464  0  2.119  2.877  0.272  3.366  0.126  0.121  0.532  4.377  5.629  6.941  7.067  3.95  0  7.001  5.352  
 
 TAL1  6886  1  47681961  47698007  1p32  0.108  0.036  0.209  0.093  0.057  0.13  0.151  0.08  0.184  0.087  0.079  0.121  0.147  0.195  0.065  0.106  0.094  0.106  3.534  0.1  2.86  2.612  0.466  0.097  0.09  0.029  0.111  0.04  0.072  0.132  0.05  0.093  0  0.213  0.107  0.047  0.059  0.049  0.167  0.091  0.041  0.212  0.387  0.109  0.127  0.098  0.614  0.092  0.071  0.067  0.116  0.582  0.054  0.121  0.241  0.278  0.06  0.124  0.12  0  
 
 STIL  6491  1  47715810  47779819  1p32  2.04  3.001  1.351  1.907  1.745  1.675  2.494  2.151  0.733  2.779  3.213  1.48  2.523  3.244  2.703  2.379  2.503  2.032  2.305  2.085  2.312  2.442  1.152  1.334  1.944  1.514  2.265  1.783  2.21  1.107  2.56  2.238  2.003  0.901  1.531  2.794  2.59  2.337  2.172  3.33  2.149  1.191  2.572  2.287  1.673  2.084  1.12  2.613  2.314  3.078  2.522  1.466  2.29  1.648  2.115  1.997  2.933  1.988  3.48  2.525  
 
 CMPK1  51727  1  47799468  47844511  1p32  2.513  4.138  3.223  3.112  2.542  2.9  3.679  3.704  3.783  3.936  4.42  3.469  4.277  4.352  2.545  3.433  3.658  3.652  4.931  3.793  3.457  3.345  3.413  2.454  3.103  2.741  2.802  2.582  2.826  2.049  4.104  3.901  2.788  2.349  2.177  3.722  3.063  3.343  4.394  3.08  2.723  2.837  4.146  2.671  2.133  2.959  2.707  2.611  3.088  4.463  4.37  1.922  3.08  2.717  2.479  3.529  3.767  2.492  3.951  3.463  
 
 LINC01389  102724077  1  47846467  47902989  1p33  1.334  0.399  0.289  0.33  1.088  0.644  0.408  0.465  0  0.918  0.482  0  0.416  0.388  0.316  0.292  0  0  1.643  0  0  0.409  0  0.214  0  0  0.378  0  0  0.135  0.508  0.139  0  0  0  1.45  0  0.61  0.567  0  0.403  0.1  0  0  0  0.104  0.602  0.183  0  0.299  0.215  0.26  0.4  0.085  0.158  0.179  0.34  0  0.744  0.334  
 
 FOXE3  2301  1  47881743  47883724  1p32  0  0.129  0.188  0  0.1  0.181  0.152  0.242  0.246  0.161  0.235  0.107  0.092  0.236  0.158  0.14  0.097  0.282  0.134  0.251  0  0.514  0.655  0.054  0  0.068  0.364  0.079  0.06  0.235  0.088  0.188  0.111  0.233  0.158  0.183  0.058  0  0.294  0.316  0.103  1.173  0.264  0  0.109  0.238  0.239  0.087  0.13  0.099  0.084  1.423  0.101  0.285  0.252  0.159  0.096  0  0.141  0.092  
 
 FOXD2-AS1  84793  1  47897806  47900313  1p33  0.935  0.348  0.898  1.128  0.665  0.468  1.032  1.337  1.574  1.71  1.474  0.378  1.082  1.089  0.866  0.761  0.339  0.126  2.286  0.957  1.891  0.912  0.938  0.409  0.733  1.09  1.805  2.497  1.369  0.746  1.381  2.552  1.541  1.132  0.773  1.677  1.385  1.186  0.828  1.894  0.6  1.026  2.1  0.93  0.235  0.328  0.397  1.024  1.428  1.366  1.433  1.068  1.146  0.664  0.911  0.889  0.944  0.698  1.568  0.569  
 
 FOXD2  2306  1  47901688  47906363  1p34-p32  0.322  0.115  0.323  0.212  0.896  0.384  0.41  0.588  0.435  0.968  0.381  0.226  0.22  0.537  0.372  0.383  0.255  0.305  0.583  0.217  0.535  0.529  0.586  0.133  0.391  0.379  1.046  0.993  0.464  0.465  0.791  1.06  0.845  0.427  0.308  0.924  0.327  0.342  0.365  0.868  0.174  0.973  0.477  0.303  0.136  0.21  0.476  0.695  0.684  0.551  0.388  1.128  0.48  0.38  0.433  0.29  0.389  0.491  0.753  0.188  
 
 TRABD2B  388630  1  48226199  48462562  1p33  0.073  0.03  0.139  0.068  0.036  0.052  0.03  0.037  0.168  2.503  0.039  0.046  0.02  0.172  0.135  0.029  0.021  0.028  0.016  0.044  0  0.145  0.605  0.046  0  0.108  0.315  0.529  0.069  0.259  0.339  0.529  0  0.181  0.205  1.07  0.014  0  0.521  0.054  0.026  1.111  0.092  0.616  0.036  0.048  0.081  0  0.026  0.014  0.044  0.438  0.679  0.379  1.035  0.192  1.401  0.026  1.318  0.183  
 
 SKINTL  391037  1  48567386  48648100  1p33  0  0  0  0  0  0  0  0  0.111  0.092  0  0  0  0.074  0  0  0  0  0  0  0  0.073  0.161  0  0  0  0.027  0  0  0.074  0  0  0  0  0  0  0  0  0  0  0  0.059  0  0  0  0  0  0  0  0  0  0  0  0  0  0  0  0  0  0  
 
 SLC5A9  200010  1  48688356  48714316  1p33  0  0  0.088  0  0  0  0  0  0.147  0.3  0.045  0  0  0.073  0  0.045  0  0  0  0.101  0  0.102  0.254  0  0  0.075  0.081  0.881  0.1  0.092  0.35  0.216  0.125  0.165  0.168  0.261  0  0  0.017  0  0  0.216  0.025  0.045  0  0  0.034  0  0  0  0  0.185  0  0.071  0.022  0  0  0  0  0  
 
 SPATA6  54558  1  48761043  48937880  1p33  0.179  0  0.144  0.39  0.371  0.048  0.556  0.187  1.582  1.348  0.716  0.084  0.214  0.786  0.274  0.057  0.169  0.711  0  0.092  0  0.135  0.222  0.114  0.124  0.739  0.475  0.76  0.533  0.671  1.004  1.225  1.073  0.637  0.084  0.662  0.238  0.165  0.051  0.593  0.198  0.394  1.14  0.402  0.276  0  0.85  0.243  0.783  0.12  0.114  0.353  0.382  0.224  0.559  0.122  0.139  0.384  0.848  0.523  
 
 AGBL4  84871  1  48998526  50489626  1p33  0  0  0.107  0  0.044  0  0.033  0.025  0.103  0.158  0.035  0.117  0  0.054  0  0.025  0  0.161  0.041  0.062  0  0.052  0.251  0  0  0  0.122  0.069  0.047  0.139  0  0  0  0.204  0.042  0.047  0  0  0.054  0  0  0.226  0.085  0  0  0.027  0.076  0  0  0  0.075  0.159  0.037  0.104  0.042  0.053  0  0.041  0  0  
 
 BEND5  79656  1  49193194  49242641  1p33  0  0  0.564  0  0.081  0  0.058  0.526  1.326  1.593  0.758  0.06  0  0.054  0  0.077  0  0.171  2.592  0.161  0  1.84  0.227  0.089  0  0  0.236  0.095  0.857  0.13  0.641  0.254  0  0  0  0  0.059  0  0.05  0  0  0.539  0.037  0  0.149  0.063  0.047  0  0  0  0.118  0.736  0.08  0.218  0.218  0  0.757  0  0  0.082  
 
 LOC101929721  101929721  1  49723082  49734966  1p33  0  0  0  0  0  0  0  0  0  0  0  0  0  0  0  0  0  0  0  0  0  0  0  0  0  0  0  0  0  0  0  0  0  0  0  0  0  0  0  0  0  0  0  0  0  0  0  0  0  0  0  0.353  0  0  0  0  0  0  0  0  
 
 AGBL4-IT1  100874313  1  49839872  49937757  -  0  0  0  0  0  0  0  0  0  0  0  0  0  0  0  0.202  0  0  0  0  0  0  0  0  0  0  0.218  0  0  0.15  0  0  0  0  0  0  0  0  0  0  0  0  0.112  0  0  0  0  0  0  0  0  0  0  0  0  0  0  0  0  0  
 
 ELAVL4  1996  1  50513685  50669457  1p34  0  0  0.079  0  0.038  0  0.032  0  0.068  0  0  0.035  0  0.032  0  0  0  0  0  0  0  0.146  0.32  0  0  0  0.121  0  0  0.058  0  0  0  0.114  0  0  0  0  0.03  0.15  0  0.218  0.09  0  0  0  0.089  0  0  0  0.037  0.267  0  0.058  0  0  0  0  0  0  
 
 DMRTA2  63950  1  50883222  50889119  1p32.3  0  0.091  0.223  0.599  0.081  1.884  0.54  2.544  0.173  0.185  0.034  0.471  0.908  0.115  1.09  0.158  0.091  0.295  0.05  0.252  0  0.566  0.498  0.091  0.083  0.102  0.208  0.073  0.044  0.179  0.214  0.054  0.407  0.325  0.162  0.074  0.064  0  0.166  0.243  0.055  2.092  0.091  0.066  0.079  0.164  0.107  0.358  0  0.635  0.088  1.366  0.069  0.191  0.168  0.071  0.052  0  0  0.09  
 
 FAF1  11124  1  50906934  51425936  1p33  1.949  2.845  2.486  3.233  3.029  2.521  3.545  3.627  0.059  3.312  0.049  2.446  3.05  3.43  2.984  3.126  3.113  1.858  5.447  4.002  3.778  3.69  0.091  0  3.181  2.486  2.378  3.013  2.841  1.942  4.109  3.324  2.882  2.07  1.728  4.061  3.22  3.003  3.828  3.523  2.846  2.073  3.29  3.019  2.227  3.161  2.922  2.817  3.094  4.726  3.047  2.1  2.812  2.147  3.257  2.643  3.387  2.817  3.522  3.122  
 
 CDKN2C  1031  1  51434366  51440309  1p32  1.96  2.961  3.205  4.43  3.18  3.371  4.933  3.613  0  5.964  0.073  1.113  0.769  1.678  2.07  1.652  2.609  1.874  6.433  3.028  2.613  3.626  0  0.164  2.396  2.619  3.018  2.088  2.619  1.302  2.914  2.847  2.8  2.303  1.647  3.067  2.217  3.284  2.883  1.912  2.169  2.757  3.63  1.934  1.124  1.632  1.708  2.463  2.531  3.822  3.765  2.284  3.424  2.365  2.849  2.053  3.576  1.769  2.288  1.361  
 
 C1orf185  284546  1  51567905  51613754  1p32.3  0  0  0  0  0  0  0  0  0  0  0  0  0  0  0  0  0  0  0  0  0  0  0  0  0  0  0.124  0  0  0.085  0  0  0  0  0  0  0  0  0  0  0  0  0  0  0  0  0  0  0  0  0  0  0  0  0  0  0  0  0  0  
 
 RNF11  26994  1  51701944  51739119  1p32  2.228  2.433  3.363  2.852  2.727  3.257  2.989  3.411  3.664  3.272  3.682  1.386  2.258  3.463  2.073  1.867  2.279  2.15  3.158  1.616  1.231  2.099  2.39  1.192  3.058  2.296  1.821  2.565  2.645  1.156  3.131  3.197  1.965  1.895  1.282  3.791  3.308  3.092  4.149  2.869  2.706  1.738  2.729  2.584  2.001  2.806  2.263  2.603  3.054  4.125  3.825  1.967  2.696  1.85  2.384  2.619  3.019  3.432  3.708  3.18  
 
 TTC39A  22996  1  51752929  51810788  1p32.3  3.11  0.066  0.331  0.241  4.779  0  0.305  0.209  0.757  0.168  0.491  1.648  1.753  2.114  1.403  1.724  0.509  0.58  0.091  0.132  0  0.123  1.043  0.396  0  0.432  0.108  0.673  0.892  0.24  5.264  2.136  0  0.263  0.346  1.993  0.322  0.669  0.246  0.492  0.992  0.367  0.087  0.554  0.433  0.251  0.15  0.106  0.328  0.625  0.552  0.9  0.163  0.254  1.408  0.522  1.189  0.163  2.384  0.364  
 
 TTC39A-AS1  102724097  1  51795325  51796953  1p32.3  0.685  0  0  0  0.82  0  0  0  0  0  0  0  0  0  0  0  0  0  0  0  0  0.103  0.399  0  0  0  0.076  0  0  0.059  0  0  0  0  0  0  0  0  0  0  0  0  0  0  0  0  0  0  0  0  0  0  0  0  0  0  0  0  0  0  
 
 EPS15  2060  1  51819934  51984995  1p32  2.101  3.251  1.559  1.84  1.798  1.631  2.275  2.203  2.343  2.367  3.006  2.102  1.936  2.57  1.662  1.876  1.988  3.114  3.17  2.268  2.251  1.773  1.626  0.998  1.918  2.162  2.685  2.206  2.245  2.362  3.341  2.513  2.172  1.707  1.03  3.087  2.639  2.322  2.291  1.972  2.347  1.439  2.513  1.823  1.623  2.214  3.153  2.046  2.355  3.003  2.921  1.107  1.995  1.106  1.897  1.946  2.373  1.863  2.636  2.463  
 
 OSBPL9  114883  1  52082545  52254891  1p32.3  3.016  3.593  1.262  1.75  2.908  1.992  3.038  3.237  2.18  2.115  3.476  1.819  3.274  3.368  2.541  2.483  3.028  3.117  3.708  3.269  3.265  1.608  3.081  2.614  2.483  2.745  2.985  2.772  3.438  2.643  4.387  3.337  2.273  1.369  1.786  3.681  3.19  3.292  3.136  2.864  3.206  1.593  3.724  2.288  1.81  2.752  3.369  2.338  2.529  4.009  2.327  1.394  3.057  2.21  3.24  2.948  3.414  2.332  4.038  3.434  
 
 NRDC  4898  1  52254865  52344609  1p32.2-p32.1  3.396  3.131  2.772  3.417  3.631  2.977  4.466  4.233  2.943  4.16  4.779  2.643  3.361  4.525  2.585  3.183  3.194  2.362  5.403  4.447  3.995  4.068  2.899  3.013  3.234  2.971  3.24  3.221  3.141  2.656  3.947  4.155  2.678  1.333  2.848  5.518  4.058  4.161  4.758  4.914  3.957  2.929  4.315  3.362  2.393  3.777  3.908  3.579  3.634  5.911  4.266  3.116  3.355  2.705  3.825  3.656  3.993  4.273  4.155  4.046  
 
 RAB3B  5865  1  52373627  52456436  1p32-p31  0.75  0.015  1.151  1.295  0.054  1.827  1.218  2.113  0.252  0.542  1.115  0.015  0.032  2.596  0.174  0.015  0.069  0.026  0.084  0.049  0.124  0.106  0.359  0.083  0.047  0.06  0.138  0.541  0.016  0.205  0.012  0.026  0.018  0.246  0.966  0.88  3.457  2.364  2.788  0.139  0.219  3.1  1.754  3.113  0.083  0.138  1.112  3.448  1.88  3.244  0.69  2.499  1.164  0.326  3.577  3.755  1.563  3.498  3.852  4.028  
 
 TXNDC12  51060  1  52485803  52521843  1p32.3  2.993  4.762  3.211  4.555  3.623  2.375  4.419  4  3.98  3.997  4.406  3.107  3.095  4.762  2.8  3.582  4.589  5.434  4.825  4.894  2.858  3.799  4.255  2.79  3.157  2.945  3.047  2.765  2.973  2.228  3.892  3.681  3.234  3.082  2.633  4.292  3.121  3.304  4.991  3.492  2.568  3.616  4.063  4.177  2.299  3.091  4.391  2.527  3.649  4.322  3.487  2.603  3.199  2.673  4.349  3.71  3.394  3.231  3.765  3.82  
 
 KTI12  112970  1  52497776  52499472  1p32.3  2.068  2.627  1.931  3.331  1.31  1.711  3.306  2.709  2.395  2.462  2.96  1.598  2.102  3.083  1.488  2.042  1.425  3.332  3.903  3.221  2.292  2.92  2.432  1.976  2.208  1.882  2.947  2.215  2.457  1.942  3.026  3.183  2.746  1.952  1.177  2.642  2.255  2.046  2.675  2.332  1.825  2.2  3.597  2.076  1.406  1.738  1.492  2.04  2.162  3.174  2.261  1.249  2.056  1.411  2.431  2.286  1.982  1.96  2.956  2.34  
 
 TXNDC12-AS1  104355143  1  52516589  52518355  1p32.3  0  0  0  0  0  0  0.276  0.335  0  0  0  0  0  0.3  0  0  0  0  0  0  0  0.143  0  0  0  0  0  0  0  0  0  0  0  0  0  0  0  0  0  0  0  0  0  0  0  0  0  0  0  0  0  0  0.197  0  0.209  0  0.348  0  0  0  
 
 BTF3L4  91408  1  52521856  52556388  1p32.3  1.935  3.177  1.597  2.528  2.823  1.931  2.517  2.534  2.693  2.254  2.989  1.414  1.536  3.184  1.879  2.331  2.181  3.184  3.607  2.967  1.795  2.295  2.427  1.647  2.183  1.616  1.382  1.62  1.342  0.638  2.286  2.188  1.397  1.29  1.031  3.107  1.981  1.929  3.63  2.16  2.082  1.316  3.314  2.695  1.544  2.095  2.042  1.676  2.173  3.62  2.394  1.129  1.715  1.451  1.828  1.955  2.056  2.09  3.077  2.47  
 
 ZFYVE9  9372  1  52607765  52812358  1p32.3  0.693  0.87  0.94  0.516  0.671  0.838  0.792  1.249  0.76  1.151  1.426  0.294  0.961  1.337  1.216  0.685  0.707  0.4  0.677  0.328  0.908  0.614  0.319  0.315  0.794  0.966  0.614  1.072  1.102  0.537  1.061  1.073  0.892  0.444  0.609  1.938  1.131  0.923  1.255  1.033  0.871  0.561  1.164  1.516  0.93  0.817  0.442  0.948  1.656  0.976  1.329  0.678  0.818  0.581  0.772  0.888  1.276  0.953  1.037  0.983  
 
 CC2D1B  200014  1  52816264  52831877  1p32.3  1.168  0.578  0.978  1.346  1.132  1.235  2.176  1.781  1.374  2.102  2.563  0.783  2.013  1.433  1.515  1.6  1.083  0.155  2.395  0.727  1.568  1.891  1.273  1.066  1.421  2.226  2.231  1.854  2.03  1.761  3.166  2.564  1.931  0.683  0.964  2.777  2.413  2.139  1.866  1.991  1.536  1.22  2.144  1.823  0.84  1.412  1.322  1.677  1.501  2.426  1.624  1.108  1.841  1.053  1.839  1.397  1.722  1.53  2.392  1.785  
 
 ORC1  4998  1  52838500  52870143  1p32  2.115  1.248  0.807  1.62  1.475  1.474  2.576  1.619  0.592  2.017  3.352  2.073  2.709  2.636  2.504  2.435  2.429  1.217  4.34  3.019  3.204  2.745  1.852  2.234  1.916  1.738  2.383  2.287  2.152  1.698  2.604  2.777  2.444  0.994  1.824  3.002  2.389  1.592  1.446  3.295  1.874  1.587  3.151  2.828  1.449  1.636  0.864  2.241  1.927  3.047  1.889  1.418  2.298  1.34  1.868  1.265  2.392  1.764  3.263  1.821  
 
 PRPF38A  84950  1  52870218  52883992  1p32.3  2.792  3.206  1.568  1.903  2.782  1.791  2.946  3.088  2.014  2.859  3.492  2.596  2.749  3.878  2.854  2.827  2.87  4.674  5.205  3.51  3.463  3.247  2.473  2.537  2.485  1.683  2.485  2.225  2.167  1.312  3.259  3.228  3.022  1.617  1.54  3.311  2.869  2.229  2.24  2.713  2.393  1.312  3.37  2.817  2.377  2.313  2.191  2.392  2.566  3.769  2.593  1.002  2.711  1.386  2.229  2.58  2.768  2.431  3.403  2.603  
 
 ZCCHC11  23318  1  52888947  53018762  1p32.3  0.054  1.947  1.02  1.677  1.686  1.113  1.968  1.984  1.526  2.063  2.314  0.699  1.516  2.494  2.132  1.378  1.575  2.148  3.306  1.518  1.592  2.426  1.568  1.246  1.621  1.369  1.855  1.242  1.6  1.427  2.251  1.754  1.667  0.658  1.092  3.151  2.083  1.589  1.741  2.226  1.851  1.039  3.247  1.764  1.32  1.234  0.778  1.946  1.686  3.009  1.929  1.203  1.674  0.805  1.89  1.661  1.966  1.558  2.464  1.917  
 
 GPX7  2882  1  53068042  53074723  1p32  0  1.172  2.794  1.704  0  0.58  0.578  2.811  0  2.358  1.766  0.123  0  0.102  0  0  0  2.312  5.853  0.166  0  1.699  4.568  3.794  0  0.26  0.091  0  0  0.077  0  0  0  0.2  0  2.952  1.348  0.586  0  1.397  0  0.479  0.72  3.069  0  0.897  0.046  0.782  0  1.816  0  0.69  0  0.073  0  0  3.029  0.131  1.292  1.858  
 
 FAM159A  348378  1  53099065  53122737  1p32.3  0  0  0  0  0  0  0  0.172  0.29  0  0  0  0  0  0  0  0  0.287  0.659  0.484  0  0.287  2.939  2.266  0  0  0.114  0  0  0.173  0  0  0  0  0  0  0  0  0.118  0  0  0.641  0.551  0  0  0.119  0.089  0  0  1.206  0  0.817  0  0  0  0  0  0  0  0  
 
 COA7  65260  1  53152013  53164038  1p32.3  1.723  0.966  1.67  2.586  1.767  1.692  1.969  1.535  1.545  1.563  1.94  1.731  2.673  2.345  2.109  1.791  1.84  0.69  2.373  1.863  2.986  1.582  2.398  2.392  2.231  1.45  1.44  2.124  1.951  1.902  2.568  2.899  2.077  1.043  1.432  2.811  2.189  1.708  1.41  2.636  1.638  1.417  3.196  2.176  1.534  1.685  1.381  2.459  1.672  3.063  2.179  1.227  1.847  1.338  1.808  1.945  1.708  2.23  3.24  1.812  
 
 ZYG11B  79699  1  53192130  53293013  1p32.3  0.956  1.367  1.275  1.149  1.326  1.236  1.388  1.573  1.728  1.764  2.086  0.678  1.431  1.933  1.68  0.65  1.026  1.023  1.424  0.803  1.295  0.623  0.809  0.642  0.818  1.295  1.219  1.404  1.181  0.962  1.885  1.765  1.437  0.981  0.862  2.542  1.569  1.508  1.53  1.729  1.067  0.847  1.753  1.584  0.7  1.189  0.887  1.263  1.885  1.634  2.242  0.682  1.247  0.663  1.007  1.266  1.497  1.213  1.59  1.641  
 
 ZYG11A  440590  1  53308182  53360247  1p32.3  0.979  0.631  0.183  0.578  0.85  0  0.048  0.119  0.122  0.095  0.027  0.047  0  0.034  0  0.025  0.044  0.228  0.022  0.089  1.343  0.136  0.529  0.671  0  0  0.638  0.019  0.261  0.074  0.038  0.03  0.307  0.293  0.489  0.585  1.054  0.756  0.04  1.06  0.09  0.408  1.238  0.411  0.861  1.185  0.186  1.505  0.985  1.946  0.1  0.844  0.044  0.527  0.064  0.474  0.876  0.92  0.902  0.325  
 
 ECHDC2  55268  1  53361581  53387446  1p32.3  2.045  2.376  1.554  2.226  2.323  0.279  0.066  1.738  0.525  1.544  2.214  1.968  2.136  2.134  0  1.388  0.201  1.03  3.197  1.656  0.386  0.707  0.403  0.412  0.193  0.205  0.693  0  0.15  0.156  0.098  0  0.431  0.49  1.016  3.017  1.573  1.618  2.523  1.647  1.618  1.149  1.95  0.258  0.913  1.209  1.004  1.433  1.86  1.249  1.389  1.258  0.946  1.17  2.676  1.668  1.602  1.925  0.98  1.379  
 
 SCP2  6342  1  53392900  53517289  1p32  2.927  4.835  3.713  3.781  4.25  2.462  4.238  3.912  3.91  4.284  4.407  3.976  3.982  4.576  2.86  3.767  4.421  5.754  6.528  4.219  3.092  3.944  3.64  2.404  3.077  3.054  3.048  2.219  3.209  2.214  4.191  3.607  2.781  2.958  2.038  4.944  3.298  3.336  4.925  2.509  3.362  2.602  4.308  3.117  2.823  3.771  2.357  2.399  3.251  4.748  4.418  1.793  3.014  2.569  3.238  3.459  3.699  3.831  4.085  3.914  
 
 PODN  127435  1  53527723  53551174  1p32.3  0  0.045  1.399  0.399  0.023  0  0.042  0.046  0.265  0.364  0.041  0.065  0  0.06  0  0.064  0  0.178  1.591  0.124  0  0.356  0.623  0  0  0  0.127  0  0  0.185  0.413  0.073  0  0.111  0  0.123  0.909  0  0.076  0  0  0.246  0.045  0  0.184  0.085  0.037  0  0.074  0  0.039  1.014  0.027  0.15  0.056  0.085  0  0  0  0  
 
 SLC1A7  6512  1  53552850  53608304  1p32.3  0  0.048  0.688  0  0.053  0.136  0.278  0.093  0.192  0.283  0  0.185  0.358  0.222  0  0.103  0.176  0.264  1.484  0.183  0  0.443  0.904  0  0  0  0.253  0.103  0  0.136  0.153  0  0  0.276  0  0.17  3.126  0.86  0.09  0  0  0.499  0.1  0  0  0.069  0.061  0  0  0  0.079  0.689  0  0.253  0.127  0.093  0  0  0  0  
 
 CPT2  1376  1  53662100  53679869  1p32  2.606  1.07  1.474  2.644  3.274  0.964  2.529  2.32  2.57  2.571  3.193  2.594  3.384  2.411  2.894  2.82  3.174  2.083  3.223  2.009  1.799  1.706  2.401  2.127  1.126  2.588  2.398  2.143  2.29  1.684  3.285  2.983  2.265  1.715  1.051  3.275  2.85  1.832  1.815  2.862  1.546  1.907  3.139  2.102  2.02  2.089  1.662  1.322  1.994  2.082  2.562  1.714  1.872  0.93  2.001  1.569  1.895  1.408  2.486  2.515  
 
 C1orf123  54987  1  53679771  53686311  1p32.3  3.28  4.946  3.186  3.021  3.603  3.249  4.267  3.534  3.29  4.037  3.853  2.559  2.953  4.111  2.677  3.573  2.91  5.566  5.527  4.461  3.313  4.881  3.007  2.564  2.751  2.783  3.485  2.781  2.832  1.631  3.67  3.533  2.687  2.152  2.682  3.965  3.588  3.04  4.291  3.498  2.886  2.882  4.917  2.655  2.333  2.942  4.111  3.304  2.992  5.309  3.802  2.197  3.7  2.288  4.334  3.274  3.259  3.985  3.955  3.482  
 
 MAGOH  4116  1  53692563  53704282  1p32.3  2.512  3.891  2.033  3.184  2.307  4.035  3.855  3.576  2.864  3.452  4.002  3.081  3.685  4.203  2.866  3.652  4.094  3.547  5.893  3.619  3.796  3.839  3.33  3.268  3.93  2.492  2.826  2.595  2.65  1.428  3.801  3.516  2.858  1.514  2.864  3.297  3.546  2.721  3.588  3.536  3.019  3.194  4.481  3.367  3.73  3.431  3.373  3.406  3.087  5.361  3.102  1.979  3.322  2.958  3.262  3.226  2.966  4.339  4.823  2.938  
 
 LOC100507564  100507564  1  53704281  53708455  -  0.487  0  0.273  0  0.66  0.658  0.32  0.108  0.547  0.75  0.285  0.63  0.185  0.496  0.327  0.352  0.43  2.345  0.514  1.178  0  2.023  0.743  0.235  0  0.189  0.542  0.267  0.379  0.108  0.492  0.456  0.168  0.439  0.638  1.046  0.125  0.264  1.575  0.667  0.504  0.547  0.063  0.277  0.239  0.447  0.771  0  0.319  0.393  0.345  0.321  0.297  0.272  0.45  0.566  0.316  0.217  0.368  0.402  
 
 LRP8  7804  1  53708040  53793821  1p34  1.95  1.638  1.362  2.08  1.322  1.191  3.34  2.282  1.76  2.647  3.365  1.733  3.027  2.681  2.253  2.797  2.049  1.824  3.194  2.25  1.58  2.475  1.545  1.89  1.593  2.73  3.856  2.647  2.932  1.7  3.165  2.785  2.688  1.12  1.546  3.127  3.078  2.585  2.155  1.957  2.34  2.212  2.301  1.977  0.815  1.867  1.73  1.976  1.692  2.247  2.782  1.821  2.218  1.276  2.167  1.522  2.055  1.745  2.984  1.383  
 
 LOC105378732  105378732  1  53793904  53802181  -  0  0  0  0  0  0.446  0.36  0  0.327  0  0  0  0  0.147  0  0.52  0  0  0  0  0  0.309  0  0.227  0  0.318  0.297  0.163  0.282  0.528  0.339  0  0  0  0.375  0.374  0  0  0  0.502  0.232  0.836  0.447  0  0  0  0  0  0  0  0  0  0.123  0.383  0.156  0  0  0  0  0  
 
 SLC25A3P1  163742  1  53904042  53905693  1p32.3  0  0  0.372  0  0.227  0  0.122  0  0.337  0.172  0.101  0  0.156  0.196  0  0.254  0  0.193  0  0.153  0  0.552  1.095  0  0  0  0.308  0.139  0  0.45  0  0.17  0  0.435  0  0.169  0  0  0.286  0  0  0.475  0.155  0  0  0  0.079  0  0  0  0  1.534  0.136  0.35  0.241  0  0  0  0  0  
 
 DMRTB1  63948  1  53925071  53933160  1p32.3  0  0.077  0.089  0.143  0.043  0  0.051  0.08  0.122  0.071  0  0  0  0.047  0  0.085  0  0.063  0  0.058  0  0.221  0.606  0  0  0  0.218  0.038  0  0.112  0  0  0  0.194  0  0.078  0  0  0.045  0  0  0.435  0.066  0  0.214  0.181  0.033  0  0  0  0  0.525  0  0.157  0.06  0  0  0  0  0  
 
 GLIS1  148979  1  53971905  54199877  1p32.3  0.088  0.053  1.765  0.815  0.071  0.628  1.292  2.537  0.274  2.809  0.653  0.286  0.067  0.308  0.061  0.139  0.176  0.163  0.045  0.144  0.207  0.255  0.906  0  0  0.05  0.265  0.087  0.096  0.212  0.126  0.353  0.085  0.247  0.069  0.102  0.203  0  0.215  0.33  0.048  0.42  0.1  0.074  0  0.095  0.193  0.087  0.107  0.106  1.667  0.638  1.963  1.51  2.215  1.796  2.191  0.074  0.06  1.774  
 
 NDC1  55706  1  54231133  54304225  1p32.3  2.49  3.753  1.723  2.197  3.179  1.989  3.045  2.308  1.345  2.396  3.331  2.669  3.012  3.661  3.066  2.9  3.258  3.636  4.276  3.314  3.018  2.578  2.147  2.332  2.779  1.786  2.319  2.134  2.238  1.844  3.171  2.87  2.375  1.317  1.842  3.769  3.099  2.567  2.587  3.488  2.77  1.765  3.367  3.022  2.11  2.217  2.734  2.4  2.939  3.591  3.132  1.523  2.317  1.352  2.453  2.12  2.931  2.576  3.568  2.831  
 
 YIPF1  54432  1  54317391  54355487  1p33-p32.1  1.819  1.333  1.753  1.718  3.45  1.537  3.105  1.898  2.442  2.335  2.422  1.563  1.651  1.837  1.431  1.628  1.551  1.151  3.076  1.813  1.198  1.902  2.571  1.451  1.895  1.839  2.275  1.8  2.091  1.196  2.592  2.36  1.636  1.38  0.845  2.792  1.968  1.982  2.372  2.042  1.625  1.335  2.339  1.636  1.241  1.294  1.952  1.705  1.34  2.886  2.067  1.124  1.472  1.245  1.456  1.318  2.094  1.761  2.424  1.65  
 
 DIO1  1733  1  54359860  54376759  1p33-p32  0.184  0  0.141  0  0.365  0  0.098  0.102  0.168  0.265  0.222  0.076  0  0.123  0.197  0.066  0  0  0.126  0  0  0.166  0.504  0  0  0.073  0.25  0  0  0.308  0  0  0  0.179  0  0  0.116  0.238  0.101  0  0.148  0  0.605  0  0  0.237  0.047  0  0  0  0.086  0  0.056  0.082  0.069  0  0.217  0.252  0  0  
 
 HSPB11  51668  1  54387233  54411288  1p32  1.924  6.895  2.139  2.257  5.705  3.954  3.966  3.751  2.174  4.268  4.8  2.013  2.113  4.542  2.377  4.323  3.791  7.14  6.448  3.399  4.405  4.071  2.74  2.357  3.38  2.87  2.352  2.237  2.737  0.736  4.623  3.746  2.763  2.046  2.929  3.966  3.091  3.086  4.475  3.361  4.324  3.859  3.561  3.583  2.59  3.684  3.668  2.85  4.26  5.21  3.434  1.844  3.644  3.159  3.598  3.617  4.096  4.182  4.227  3.667  
 
 LRRC42  115353  1  54411998  54433841  1p33-p32.1  1.655  3.899  2.662  2.85  3.024  2.41  3.015  2.837  2.323  3.054  3.223  1.617  3.361  3.899  2.941  3.536  3.062  3.966  4.258  1.79  2.713  2.426  2.13  1.123  2.657  2.249  2.24  2.205  2.348  1.531  2.95  3.106  2.279  1.495  1.658  3.631  2.921  2.598  3.51  3.319  3.091  1.887  3.981  2.715  2.238  2.875  2.2  3.041  2.981  5  2.862  1.891  2.814  1.991  2.903  2.672  3.594  3.168  4.143  3.184  
 
 LDLRAD1  388633  1  54472970  54483859  1p32.3  0  0  0.106  0  0.18  0  0.068  0  0  0.044  0  0.076  0.109  0.125  0  0  0  0  0  0  0  0.083  0.486  0  0  0  0.102  0.046  0  0.237  0  0  0  0.191  0  0  0  0  0  0  0.137  0.137  0.048  0.094  0  0  0.196  0  0  0  0  0.138  0  0.102  0.046  0  0  0  0  0  
 
 TMEM59  9528  1  54492353  54519246  1p32.3  3.236  3.56  3.577  4.411  3.379  2.028  4.44  3.837  4.588  5.09  3.733  3.326  3.636  3.704  2.926  3.991  4.623  3.896  4.531  2.425  2.626  2.648  4.704  3.272  2.714  3.05  4.112  3.08  3.862  2.065  4.452  3.993  2.926  2.513  1.901  4.61  3.048  3.227  3.49  3.009  2.903  3.123  2.672  2.147  2.507  3.242  1.326  2.93  1.995  4.761  3.405  2.454  2.735  2.849  2.906  2.235  3.115  2.232  2.893  2.837  
 
 TCEANC2  127428  1  54519244  54578192  1p32.3  0.766  0.552  0.784  0.93  0.737  0.469  1.071  0.845  0.704  1.3  1.01  0.942  0.788  0.877  0.773  0.646  0.847  0.401  1.916  0.853  0.872  1.137  1.314  0.564  0.73  0.697  0.734  0.797  0.761  0.482  1.222  0.981  0.932  0.796  0.422  1.099  0.742  0.802  0.627  1.061  0.469  0.608  1.669  0.408  0.45  0.519  0.341  0.664  0.471  1.271  1.084  0.532  0.712  0.355  0.716  0.448  0.748  0.435  0.939  0.665  
 
 CDCP2  200008  1  54604667  54618679  1p32.3  0  0.048  0.138  0  0  0  0  0.055  0.213  0.041  0.071  0.078  0  0.027  0  0.061  0  0.153  0  0.062  0  0.182  0.504  0  0  0  0.093  0  0  0.052  0.079  0  0  0.197  0  0  0  0  0.051  0  0  0.1  0.047  0  0.11  0.102  0  0  0  0  0  0.558  0  0.056  0.032  0  0  0  0  0  
 
 CYB5RL  606495  1  54638026  54665746  1p32.3  1.103  0.173  0.481  0.86  0.922  0.556  1.36  0.849  1.079  0.997  1.087  0.841  0.913  1.266  1.276  0.636  0.623  0.373  1.676  0.957  1.211  0.904  1.189  0.837  0.555  0.711  1.453  0.947  1.001  0.774  1.503  0.684  0.847  0.437  0.56  1.007  1.319  1.042  0.526  1.286  0.879  0.724  1.709  0.615  0.565  0.704  1.318  0.927  0.85  1.33  0.748  0.674  0.765  0.672  0.863  0.531  0.751  1.057  1.534  0.744  
 
 MRPL37  51253  1  54665839  54684056  1p32.1  4.945  3.155  4.32  5.363  5.082  4.593  6.209  6.032  4.691  5.659  6.335  5.207  5.461  5.406  5.126  5.347  5.587  2.82  7.034  5.344  5.467  5.211  5.007  4.82  5.186  4.442  6.034  4.892  5.784  4.856  6.442  6.322  5.162  3.536  4.021  5.772  5.724  5.17  4.94  5.707  5.255  5.135  6.355  4.681  4.583  5.03  4.601  5.429  4.854  6.971  5.551  3.645  5.447  4.382  4.812  4.981  5.191  5.409  6.502  5.049  
 
 SSBP3  23648  1  54691103  54872068  1p32.3  2.05  0.783  1.013  1.819  2.387  1.125  2.692  3.95  1.894  3.768  2.899  3.521  1.993  2.601  1.771  1.561  2.255  1.602  4.639  2.568  3.627  4.185  2.484  2.084  3.484  1.589  2.719  2.051  1.489  1.096  2.505  2.91  2.3  1.286  1.065  3.584  2.912  1.861  1.384  2.824  1.826  1.855  3.848  2.299  2.187  3.021  1.405  2.484  1.972  3.577  2.968  0.993  2.374  1.93  0.825  1.596  3.341  1.615  3.682  1.869  
 
 C1orf191  619518  1  54702115  54704736  1p32.3  0.322  0.079  0.188  0.186  0.212  0  0.192  1.191  0.273  0.748  0.494  0.086  0.314  0.217  0.38  0.192  0.153  0.434  0.934  0.441  0.252  0.96  0.649  0.102  0.163  0.157  0.51  0.296  0.094  0.142  0.33  0.286  0.233  0.259  0.108  0.427  0.544  0.213  0.116  0.205  0.102  0.197  0.808  0.154  0  0.387  0.097  0.359  0.205  0.365  0.38  0.222  0.383  0.119  0.114  0.104  0.616  0  0.382  0.052  
 
 ACOT11  26027  1  55013806  55100417  1p32.3  0.164  0  0.152  0  0.11  0.094  0.364  0.666  0.132  0.402  0.805  1.973  2.21  1.7  1.476  1.967  0.871  0.129  0.138  0.872  0  0.22  0.373  0  0.679  1.301  0.106  0.588  0.601  0.668  0.329  0.072  0.53  0.151  0.531  1.503  0.495  0.081  0.138  1.372  0.885  0.42  1.262  0.063  0.565  0.833  0.087  0.44  0.184  0.438  0  0.664  0.299  0.285  0.296  0.115  0.718  0.097  0.338  0.258  
 
 FAM151A  338094  1  55074849  55089200  -  0  0  0  0  0  0  0.08  0.06  0.091  0.045  0.081  0.17  0.173  0.215  0.092  0.225  0  0  0  0.126  0  0.051  0.431  0  0  0.08  0.087  0.042  0  0.126  0  0  0  0  0  0  0  0  0  0  0  0.056  0.031  0  0  0  0  0  0  0  0  0.204  0.06  0  0.076  0  0  0  0  0  
 
 MROH7  374977  1  55107412  55175940  1p32.3  0  0  0.12  0  0  0  0.06  0.109  0.103  0.28  0.025  0  0.26  0.092  0.075  0.061  0.082  0.079  0  0.176  0  0.057  0.265  0  0  0  0.055  0.056  0  0.028  0  0  0  0.205  0.06  0  0.067  0  0.049  0  0.057  0.066  0  0  0  0.042  0  0  0  0.057  0  0.208  0.029  0.058  0.05  0.061  0  0  0.103  0  
 
 MROH7-TTC4  100527960  1  55107412  55208328  1p  0  0  0.039  0  0.116  0  0  0  0  0.062  0.033  0.045  0.124  0.053  0.037  0.062  0  0  0  0  0  0.057  0  0  0.029  0  0.049  0.014  0  0.048  0.047  0  0  0  0  0  0.022  0  0  0.051  0  0.02  0.051  0  0  0.021  0.014  0  0  0  0  0.123  0  0.034  0  0.045  0  0  0  0  
 
 TTC4  7268  1  55181494  55208328  1p32.3  1.906  2.653  2.021  2.709  2.295  1.863  3.86  2.327  1.914  2.534  3.409  1.697  2.635  3.314  1.951  2.072  2.123  2.734  4.24  2.603  2.905  2.721  2.638  1.778  2.698  1.94  2.497  2.081  2.279  1.453  3.135  3.23  2.179  1.461  1.832  3.174  2.872  2.347  3.209  3.11  2.147  1.906  3.478  2.351  1.391  1.955  2.29  2.071  2.241  3.655  3.049  1.386  2.819  2.1  2.82  2.122  2.583  2.277  3.422  2.673  
 
 PARS2  25973  1  55222570  55230226  1p32.2  1.37  0.947  0.855  1.623  1.271  0.329  1.509  0.811  1.611  1.368  1.484  1.288  1.827  2.058  1.689  1.516  1.703  0.581  1.895  1.78  1.635  1.352  1.694  0.916  0.674  1.16  1.131  1.079  1.365  1.12  1.886  1.491  1.355  0.967  0.795  1.732  1.751  1.444  1.026  1.854  0.929  0.692  1.557  0.978  1.136  1.086  0.136  1.245  1.002  1.418  1.538  0.917  1.225  0.698  1.318  1.129  1.148  0.527  1.749  1.415  
 
 TTC22  55001  1  55246751  55266941  1p32.3  0  0.073  0.195  0  0.152  0.122  0.097  0.122  0.212  0.083  0.082  1.107  1.626  0.209  0  1.233  0.169  0.284  0.05  0.211  0  0.672  0.616  0.519  0  0.061  0.177  0.036  0  0.146  0.06  0  0  0.285  0.124  0.746  0  0  0.289  0.321  1.133  0.848  0.094  0  0.315  0.463  0.384  0.151  0.086  0.131  0.728  2.036  0  0.227  0.291  0.234  0.133  0.081  0.359  0.09  
 
 LEXM  163747  1  55271735  55307937  1p32.3  0  0  0.112  0  0.089  0  0  0.079  0  0.064  0.072  0  0.094  0.097  0  0.072  0  0.102  0.092  0.12  0  0.118  0.351  0.156  0  0.072  0.043  0  0  0.053  0  0  0  0  0  0.106  0  0  0.039  0  0  0.065  0.038  0  0  0.271  0.066  0  0  0.056  0.079  0.323  0.056  0.23  0.08  0  0.064  0  0.156  0  
 
 DHCR24  1718  1  55315299  55352921  1p32.3  4.106  2.625  5.046  4.995  5.815  3.903  4.742  6.147  3.715  6.458  6.775  4.716  5.441  4.33  5.44  4.86  4.567  0.892  4.874  4.307  4.393  3.895  3.438  4.709  4.086  4.301  5.547  4.769  5.052  3.414  5.291  4.459  4.774  2.931  2.082  6.26  5.693  4.365  4.695  4.585  4.457  3.879  3.6  5.325  6.297  6.349  3.472  5.972  6.055  6.298  5.306  4.017  6.095  6.284  6.487  6.16  5.485  4.528  6.821  7.692  
 
 TMEM61  199964  1  55446336  55457966  1p32.3  0.19  0  0.244  0  1.512  0  0  0  0.282  0.058  0.249  0  0  1.482  0.167  0  0.133  0.278  0  0  0  0.205  1.571  2.415  0  0  0.156  0  0  0.106  0  0  0  0.173  0.105  0.413  0  0  0.129  0  0.106  0.072  0.048  0  0.182  0.069  1.128  0.149  0.171  0.614  0.165  0.864  0.124  0.128  0.233  0  0  0.184  0  0  
 
 BSND  7809  1  55464616  55474465  1p32.1  0  0  0.117  0  0.042  0  0  0  0.189  0.089  0  0  0  0.066  0  0  0  0  0  0  0  0.116  0.648  1.112  0  0  0.083  0  0  0.076  0  0  0  0.154  0  0  0  0  0.084  0  0  0.185  0  0  0  0  0.038  0  0  0  0  0.51  0  0  0  0  0  0  0  0  
 
 PCSK9  255738  1  55505148  55530526  1p32.3  0.284  0.308  0.159  3.171  0.067  0.061  0.276  0.188  0.169  0.247  0.254  3.581  3.542  2.682  1.237  0.19  5.296  0.593  0.062  0.154  2.015  0.364  0.651  0  0  0.734  0.15  0.045  0.702  0.123  2.105  0.392  0  0.237  0.9  3.336  1.654  0  0.257  0.078  0.051  0.96  0.065  0.198  0.078  1.886  2.529  1.375  0.465  2.094  0.242  0.811  0.415  0.333  0.549  0.245  0.103  0  0.098  1.3  
 
 USP24  23358  1  55532031  55681039  1p32.3  1.222  1.929  1.121  1.564  1.381  1.078  1.878  1.745  1.139  2.005  2.451  1.106  2.009  2.277  1.478  1.908  1.405  2.052  2.621  2.031  1.798  1.842  0.827  0.794  1.541  1.397  1.782  1.855  1.878  1.122  2.052  2.171  1.911  0.775  0.892  3.095  2.321  2.014  1.652  2.434  2.057  0.917  1.98  1.627  1.321  1.571  1.916  1.631  2.214  2.131  2.281  1.225  1.862  0.935  1.793  1.689  1.916  1.471  2.768  1.957  
 
 LOC100507634  100507634  1  55681080  55683128  1p32.3  0.238  0.553  0.222  0.133  0.629  0.178  0.326  0.218  0.252  0.395  0.245  0.057  0.298  0.362  0.209  0.194  0.246  0.558  0.42  0.279  0.385  0.54  0.301  0.112  0.137  0.25  0.188  0.586  0.376  0.179  0.523  0.642  0.41  0.241  0.085  0.676  0.305  0.32  0.44  0.448  0.611  0.413  1.497  0.156  0.149  0.277  0.377  0.234  0.682  0.62  0.328  1.027  0.222  0.225  0.667  0.353  0.417  0.179  0.515  0.246  
 
 PLPP3  8613  1  56960418  57045257  1p32.2  1.038  1.867  1.663  2.861  0.914  0.707  0.286  1.208  0.354  4.169  0.979  0.715  1.05  1.063  0.524  0.934  0.962  3.31  0  0.133  0.079  0.143  0.494  0.309  0  2.019  2.16  3.144  2.943  0.856  1.789  3.354  2.005  1.579  1.214  3.567  0.445  1.338  1.651  1.66  1.328  0.885  0.063  2.238  1.047  0.77  1.398  0.883  1.587  1.55  3.329  1.776  0.856  0.924  0.125  0.959  2.053  1.105  0.656  0.746  
 
 PRKAA2  5563  1  57110989  57181008  1p31  0.055  0.585  0.269  0.314  0.658  0.659  0.187  0.306  0.497  0.469  1.376  0.02  0  0.672  0  0.008  0  1.537  0.015  0.011  0  0.248  0.14  0  0  0.011  0.047  0.847  0.078  0.39  0  0.65  0  0.171  0.509  1.251  0.243  0.244  0.512  0.672  0.817  0.937  0.017  0.882  0.369  0.943  0.873  0.484  1.237  0.953  1.185  0.469  0.358  0.358  1.023  0.524  1.19  0.491  1.68  1.128  
 
 C1orf168  199920  1  57184476  57285369  1p32.2  0.124  0  0.088  0  0.738  0.036  0  0.021  0.047  0.058  0.039  0  0.111  0.02  0.075  0  0  0.032  0.084  0.032  0  0.034  0.233  0.236  0.231  0.03  0.042  0.051  0  0.035  0  0  0  0.082  0  0  0.041  0  0.036  0  0  0.026  0.012  0  0  0  0  0  0  0.078  0.067  0.067  0.269  0.028  0.029  0  0.025  0  0.252  0.052  
 
 C8A  731  1  57320442  57383894  1p32  0  0  0  0  0  0  0  0  0  0.13  0  0  0  0  0  0  0  0.184  0  0.047  0  0.035  0  0  0  0  0.061  0  0  0.031  0  0  0  0.114  0  0  0  0  0.039  0  0  0.123  0.02  0  0  0  0.025  0  0  0  0  0.333  0.033  0  0  0  0  0  0  0  
 
 C8B  732  1  57394882  57431813  1p32.2  0  0  0  0  0  0  0  0  0.078  0.071  0  0  0  0  0  0  0  0  0  0  0  0.109  0.244  0  0  0  0.064  0  0  0.051  0  0  0  0.159  0  0  0  0  0  0  0  0.195  0.031  0  0  0.043  0  0  0  0  0  0.165  0  0.044  0  0  0  0  0  0  
 
 DAB1  1600  1  57463578  58716211  1p32-p31  0  0  0.155  0  0.05  0  0  0  0.083  1.183  0.733  0.083  0  0.042  0  0.04  0  0.086  0.976  0.055  0  0.323  0.183  0  0  0  0.168  0.035  0  0.129  0  0.305  0  0.18  0  0.078  0  0  0.077  0  0  0.342  0.079  0.045  0.136  0  0.053  0  0  0  0.04  0.242  0.034  0.079  0.06  0.051  0  0  0  0  
 
 DAB1-AS1  101926890  1  58326214  58328786  -  0  0  0  0  0  0  0  0  0  0  0  0  0  0  0  0  0  0  0  0  0  0  0  0  0  0  0  0  0  0.06  0  0  0  0  0  0  0  0  0  0  0  0  0  0  0  0  0  0  0  0  0  0.413  0  0  0  0  0  0  0  0  
 
 OMA1  115209  1  58946390  59012471  1p32.2-p32.1  1.512  2.273  0.612  1.065  1.973  0.389  0.988  1.136  0.88  1.639  1.571  1.309  1.769  2.687  1.411  1.66  1.849  2.769  3.109  1.767  1.671  1.668  1.242  1.429  1.258  1.886  1.438  1.113  1.657  1.035  2.804  2.136  1.327  1.121  0.707  1.742  1.497  1.339  1.526  1.823  1.478  0.735  1.52  1.326  0.771  1.152  0.514  0.519  1.239  1.621  1.442  0.813  1.066  0.786  1.372  1.606  1.04  0.573  1.64  1.86  
 
 TACSTD2  4070  1  59041094  59043166  1p32  4.774  4.169  0.298  0.132  5.837  0.067  0.064  0.117  0.141  0.16  0.1  3.575  1.81  0.311  3.213  5.288  0.622  0.344  0.098  0.255  0  0.494  0.555  0  0  0.07  0.255  0.105  0.095  0.212  0.093  0.124  0.13  1.017  0.206  0.687  1.492  4.243  0.24  0.246  6.122  1.174  0.214  0  3.262  5.222  0.101  0.402  2.904  1.086  0.394  3.591  0.071  0.196  0.271  2.268  1.534  0.112  0.832  0  
 
 MYSM1  114803  1  59120410  59165747  1p32.1  1.11  1.279  0.674  0.934  1.111  0.751  0.93  1.474  0.825  1.456  1.738  0.517  1.298  2.031  1.418  1.321  1.488  1.788  2.392  1.589  1.394  1.542  0.509  0.562  1.096  1.49  1.734  1.163  1.471  0.948  1.916  1.671  1.625  0.798  0.582  1.337  1.076  1.017  1.623  1.363  0.046  0.922  1.234  1.526  0.713  1.201  0.264  1.135  1.046  1.858  1.763  0.551  1.241  0.723  1.289  0.989  1.635  0.811  2.013  1.145  
 
 JUN  3725  1  59246462  59249785  1p32-p31  0.76  3.817  3.376  4.391  1.469  3.971  3.191  4.231  4.583  3.921  4.07  1.974  2.487  0.732  1.81  1.936  3.08  3.087  1.302  0.592  2.004  0.807  1.09  1.654  3.644  0.52  1.629  2.49  1.004  0.911  0.633  1.206  1.512  1.467  1.589  2.069  2.963  3.022  4.744  1.872  1.938  2.205  2.428  1.845  2.718  2.772  2.239  3.745  3.345  4.265  3.135  2.03  3.645  3.067  3.545  2.537  4.081  3.047  3.173  3.971  
 
 LINC01135  100131060  1  59250822  59365384  1p32.1  0  0  0  0  0  0.337  0  0  0  0.073  0  0  0  0  0  0  0  0  0  0  0  0.115  0.523  0  0  0  0.079  0  0  0.137  0  0  0  0  0  0  0  0  0.184  0  0  0  0  0  0  0.341  0  0  0  0  0  0  0  0  0  0  0  0.238  0  0  
 
 LINC01358  101926925  1  59486147  59553919  -  0  0.793  0  0  0  0.393  0  0  0  0.202  0.219  0  0  0  0  0  0  0  0  0  0  0  0  0  0.534  0  0.114  0  0  0  0  0  0  0  0  0  0  0  1.712  0  0  0  0  0  0  0  0  0  0  0  0  0  0  0  0.284  0  0  0  0  0  
 
 HSD52  729467  1  59597607  59612479  1p32.1  0  0  0  0  0  0.181  0  0  0  0.083  0  0  0  0  0  0  0  0  0  0  0  0  0.324  0  0  0  0.08  0  0  0.08  0  0  0  0  0  0  0.233  0  0.471  0  0  0  0  0  0  0  0.055  0  0  0  0  0.224  0  0  0.128  0  0  0  0  0  
 
 FGGY  55277  1  59762624  60228402  1p32.1  0.619  0.703  0.426  0.423  1.143  0.761  1.648  1.333  0.611  1.591  1.118  1.237  0.554  1.106  0.627  0.835  0.989  4.359  3.262  0.893  1.069  1.11  0.423  0.24  1.027  1.029  1.057  1.512  0.985  0.838  1.489  1.449  0.502  0.513  0.259  0.787  1.51  1.147  1.023  1.557  1.268  0.557  0.571  0.403  0.245  0.831  1.153  0.591  1.058  2.425  1.347  0.555  0.872  0.998  1.674  1.15  1.244  0.394  1.564  1.888  
 
 LOC101926944  101926944  1  60238466  60254501  -  0  0  0  0  0  0  0  0  0  0  0  0  0  0  0  0  0  0.356  0  0  0  0  0  0  0  0  0  0  0  0  0  0  0  0  0  0  0  0  0  0  0  0  0  0  0  0  0  0  0  0.334  0  0.373  0  0  0  0  0  0  0  0  
 
 HOOK1  51361  1  60280532  60342050  1p32.1  1.448  0.022  0.066  0  1.73  0.049  0  0.019  0.039  0.015  0.014  1.545  2.068  2.29  2.359  1.654  2.012  2.927  0.013  0.026  0.049  0.068  1.311  1.072  0  0.014  0.088  0.6  0.168  0.044  0.018  0  0  0.096  0.276  0.075  0.469  0  0.096  0.394  1.788  0.069  0.032  2.325  0.864  1.678  1.023  0.593  1.487  1.55  0.802  0.642  0.528  0.202  1.255  1.497  0.076  0  1.76  1.48  
 
 CYP2J2  1573  1  60358979  60392423  1p31.3-p31.2  2.426  0.129  0.109  0  1.131  0  0  0.08  0.732  0.972  0.47  1.163  1.268  0.347  0.38  0.284  1.09  0.719  0  0.063  0  0.227  0.203  0.159  0  0  0.173  1.328  0.327  0.137  0.572  1.076  1.327  0.641  0  0  0  0  0.346  0.364  1.056  0  0  0.318  0.838  0.985  0.351  0.522  0.532  1.878  0.141  0.603  0  0.269  0.697  0.152  0.108  0  0.997  0.528  
 
 C1orf87  127795  1  60456065  60539442  1p32.1  0  0  0.133  0  0  0  0  0  0.076  0  0  0  0  0.03  0  0  0  0  0  0  0  0.044  0.32  0  0  0  0.02  0  0  0.03  0  0  0  0  0  0  0  0  0  0  0  0  0  0  0  0  0.044  0  0  0  0  0.089  0  0  0  0  0  0  0  0  
 
 LOC101926964  101926964  1  61125302  61291256  -  0  0  0.168  0  0  0  0  0  0.078  0  0  0  0  0.022  0  0  0  0  0  0  0  0.032  0.216  0  0  0  0.049  0.04  0  0.039  0.065  0.12  0  0.18  0  0  0  0  0.036  0  0  0  0  0  0  0  0.079  0  0  0  0  0.165  0  0.044  0  0  0  0  0  0  
 
 LOC100996570  100996570  1  61405915  61436448  -  0  0  0  0  0  0  0  0  0.216  0.176  0.174  0  0  0  0  0  0  0  0  0  0  0.115  0.286  0  0  0  0.168  0.069  0  0.104  0  0.322  0  0  0  0  0  0  0  0  0  0  0.05  0  0  0  0.089  0  0  0  0  0.367  0  0.072  0  0  0  0  0  0  
 
 NFIA  4774  1  61542945  61928460  1p31.3-p31.2  1.451  1.186  0.189  0.053  3.597  0.583  0.154  0.38  0.404  0.053  0.657  0.41  1.765  1.659  2.556  1.78  2.184  0.22  0.084  0.973  1.537  0.251  1.772  2.046  1.562  0.526  0.352  1.279  0.437  1.385  2.348  2.545  1.279  0.816  1.39  3.014  0.738  1.203  1.46  1.995  1.174  1.594  0.359  0.035  1.158  1.745  1.089  0.081  2.098  0.195  2.086  1.203  0.405  1.412  0.586  1.385  0.898  0.075  0.055  0.425  
 
 LOC645030  645030  1  61714616  61719190  1p31.3  0  0  0  0  0  0  0  0  0  0  0  0  0  0  0  0.251  0  0  0  0  0  0  0  0  0  0  0  0  0  0  0  0  0  0  0  0  0  0  0  0  0  0  0  0  0  0  0  0  0  0  0  0  0  0  0  0  0  0  0  0  
 
 MGC34796  414927  1  62119913  62121800  1p32  0  0  0.213  0  0  0.164  0.068  0  0.1  0.038  0  0  0  0.073  0  0  0.059  0.1  0  0.065  0  0.133  0.522  0  0  0  0.158  0  0  0.149  0  0.08  0  0.283  0.056  0  0  0  0.064  0  0  0.337  0.144  0  0  0.048  0.226  0  0  0  0  0.615  0  0.091  0.062  0  0  0  0  0  
 
 TM2D1  83941  1  62146718  62191095  1p31.3  0.963  3.31  1.171  1.715  3.139  1.621  1.795  2.34  2.206  2.525  2.433  1.906  1.34  2.132  0.681  2.044  2.784  4.263  3.068  2.521  1.843  1.566  1.468  1.343  1.768  1.851  1.277  1.067  1.905  0.751  2.866  2.524  1.427  1.781  1.075  2.534  1.138  2.205  3.798  1.453  1.939  2.432  1.678  1.7  1.245  2.069  1.034  1.058  1.462  3.154  1.971  0.971  1.244  1.862  2.437  2.099  2.262  1.933  2.864  2.051  
 
 INADL  10207  1  62208148  62629591  1p31.3  1.424  0.393  0.228  0.112  2.487  0.206  0.355  0.627  0.446  0.303  1.188  1.094  1.639  1.502  1.774  1.42  1.301  0.973  0.023  0.03  0.07  0.052  0.414  0.511  0  0.301  0.349  0.082  0.367  0.328  0.429  0.264  0.194  0.158  0.593  1.346  0.48  0.096  0.588  0.96  1.753  0.522  0.904  1.909  0.963  2.166  0.087  0.368  1.751  0.787  0.398  0.824  0.884  1.171  0.747  0.585  1.461  0.199  1.585  0.857  
 
 L1TD1  54596  1  62660473  62678001  1p31.3  0  0  0.104  0  0.022  0  0.024  0  0.123  0  0  0  0  0.033  0  0  0  0  0  0  0  0.033  0.283  0  0  0  0.061  0  0  0.051  0  0  0  0.167  0  0.238  0  0  0.022  0  0  0.067  0.029  0  0  0  0.034  0  0  0  0  0.098  0  0.029  0.021  0  0  0  0  0  
 
 KANK4  163782  1  62701836  62785083  1p31.3  0  0.03  0.151  0.087  0.036  0.042  0.391  0.04  0.422  0.36  0.104  0.036  0  0.031  0.041  0.039  0  0.035  0  0.059  0  0.112  0.454  0.034  0.028  0  0.085  0.014  0.021  0.071  0  0.026  0  0.139  0.024  0.206  0  0  0.169  0.045  0  0.197  0.048  0.162  1.334  0.371  0.054  0  0  0  0.032  0.48  0  0.062  0.04  0.028  0.02  0  0  0  
 
 USP1  7398  1  62901974  62917475  1p31.3  2.515  4.646  1.966  3.208  2.314  2.539  3.05  3.306  2.412  3.572  3.95  2.275  2.771  3.623  2.935  3.174  3.147  4.032  5.332  3.629  2.701  3.447  2.955  2.343  2.915  2.351  2.32  2.228  2.333  1.64  3.454  2.786  2.54  1.691  1.569  2.856  3.003  2.246  3.227  3.313  2.774  2.747  4.647  3.887  2.025  2.941  2.644  2.805  2.998  4.499  3.137  1.774  2.825  1.498  2.097  2.294  3.233  2.835  4  3.01  
 
 DOCK7  85440  1  62920396  63154039  1p31.3  1.047  2.519  1.399  1.114  1.121  1.38  1.531  2.158  2.366  2.509  2.413  0.602  1.227  2.383  1.586  1.028  1.376  1.917  1.901  0.742  0.636  0.782  0.248  0.394  1.522  2.069  2.13  1.824  2.328  1.243  2.83  2.133  2.102  1.079  0.676  1.96  1.507  1.793  1.833  1.695  1.444  1.168  2.071  1.683  0.918  1.667  0.829  1.235  1.914  1.939  2.011  0.905  1.385  1.015  1.49  1.298  2.208  1.008  2.074  2.037  
 
 ANGPTL3  27329  1  63063157  63071976  1p31.3  0  0.064  0  0  0.041  0  0.057  0.118  0  0.096  0.063  0  0.074  0.046  0.056  0.042  0.055  0.127  0  0  0  0.039  0.101  0  0  0.078  0.056  0.031  0  0.052  0.149  0.08  0  0.142  0.074  0.044  0.065  0.05  0.108  0  0  0.159  0.186  0.063  0  0.066  0.05  0.06  0.117  0.034  0.046  0  0.024  0.022  0.029  0.11  0.097  0.161  0.1  0  
 
 ATG4C  84938  1  63249776  63330941  1p31.3  0.861  1.883  0.512  0.997  0.61  0.462  0.84  0.871  1.028  1.424  1.248  0.646  1.004  1.659  0.776  0.474  1.127  1.976  2.753  1.343  0.807  1.029  1.269  0.509  0.932  1.018  0.38  0.652  0.593  0.284  1.483  1.212  0.485  0.554  0.436  1.01  0.788  0.725  0.724  1.131  0.802  0.707  1.251  0.666  0.425  0.861  0.198  0.609  0.682  1.915  1.28  0.372  0.711  0.402  0.764  0.841  0.773  0.819  1.325  1.08  
 
 LINC00466  199899  1  63624753  63782901  1p31.3  0  0  0  0  0  0  0  0  0  0.031  0  0  0  0.111  0  0  0  0  0  0  0  0.057  0  0  0  0  0.043  0.04  0  0.049  0  0  0  0.153  0  0  0  0  0.024  0  0.084  0  0  0  0  0  0.04  0  0  0  0  0.153  0  0  0  0  0  0  0  0  
 
 LOC100996301  100996301  1  63786554  63790112  -  0.587  1.18  0  1.631  1.301  0  0.34  0.697  1.447  1.258  0.863  0  0  0.494  0  0  0  0.371  0  0  0.671  0  0.958  0  0.352  1.319  1.277  1.65  1.585  0.064  2.878  1.576  1.562  1.748  0.776  1.334  0.399  0  0  0.513  0.908  0.609  0  0  0.826  1.041  0.902  1.729  0.649  2.748  0  0.826  0  0  0.301  0.586  0  0  0  0.378  
 
 FOXD3  27022  1  63788729  63790797  1p31.3  0.404  0.078  0.223  0.961  0.143  0.092  0.153  0.236  0.276  0.205  0.144  0.149  0  0.156  0  0.057  0.133  0.369  0.038  0.127  0.229  0.622  0.288  0  0.128  0.897  1.382  1.068  0.871  0.153  1.161  0.649  1.434  1.157  0.155  0.297  0.091  0  0.16  0.221  0.162  0.681  0.068  0  0.217  0.214  0.064  0.765  0  0.821  0.061  1.623  0.044  0.15  0.187  0.136  0  0.066  0  0.056  
 
 ALG6  29929  1  63833260  63904233  1p31.3  0.8  1.597  0.357  0.875  1.25  0.13  0.894  1.006  0.698  1.895  1.358  0.909  1.24  1.495  0.68  0.921  0.849  1.46  2.9  0.939  0.79  1.39  1.177  0.845  0.923  1.093  0.617  0.575  0.985  0.264  2.018  1.899  0.78  0.551  0.482  1.234  1.064  0.863  1.32  1.32  1.03  0.493  1.389  1.318  0.235  1.104  0.396  0.55  1.099  1.51  0.941  0.51  0.718  0.617  0.703  0.837  0.649  0.666  1.368  1.156  
 
 ITGB3BP  23421  1  63906440  63988944  1p31.3  2.418  4.375  1.276  1.664  2.152  2.23  3.231  2.536  1.315  3.069  3.145  2.096  1.972  4.124  1.779  2.69  3.728  6.361  4.713  3.844  2.845  3.285  2.756  2.605  2.124  1.872  1.691  1.592  1.916  0.668  3.08  2.72  1.537  1.437  2.122  2.208  2.712  2.065  3.107  1.932  1.742  2.251  2.758  2.154  1.439  2.135  2.209  2.072  2.072  4.031  2.61  1.254  2.007  1.794  2.348  2.511  2.129  2.016  3.133  2.127  
 
 EFCAB7  84455  1  63988971  64038364  1p31.3  0.46  1.153  0.284  0.614  0.721  0.427  1.046  0.761  0.584  1.406  0.982  0.502  0.439  1.303  0.444  0.462  0.843  2.313  2.218  0.41  0.28  0.867  0.421  0.357  0.536  0.654  0.675  0.569  0.851  0.336  1.394  1.04  0.5  0.42  0.278  0.664  0.478  0.403  0.486  0.63  0.514  0.376  0.646  0.373  0.367  0.573  0.298  0.211  0.641  1.499  0.547  0.205  0.437  0.458  0.518  0.511  0.46  0.627  0.823  0.609  
 
 DLEU2L  79469  1  64014650  64016307  1p31-p22  0  0.252  0.277  0.161  0.236  0.374  0.096  0.19  0.207  0.584  0.405  0.103  0.286  0.67  0.127  0.372  0.333  0.619  0.959  0.552  0  1.023  0.29  0.238  0.198  0.285  0.433  0.238  0.287  0.235  0.98  0.469  0.129  0.279  0.299  0  0.099  0.25  0.356  0.26  0.231  0.334  0.376  0.072  0.416  0.08  0.212  0.154  0.17  0.348  0.176  0.169  0.327  0.106  0.184  0  0.348  0  0.32  0.1  
 
 PGM1  5236  1  64058946  64125916  1p31  1.247  4.253  3.9  3.199  2.581  3.097  3.962  4.573  3.902  5.132  4.127  2.215  2.88  3.781  3.611  3.15  4.054  3.396  4.409  4.285  0  3.121  1.902  1.853  2.05  3.805  5.192  3.199  4.669  3.914  5.298  5.816  4.073  2.784  2.243  3.78  3.937  3.816  3.484  4.08  3.096  2.614  4.128  2.604  2.793  4.107  3.799  2.798  3.996  3.972  4.577  2.506  4.132  2.142  3.858  4.232  4.956  2.932  4.904  3.631  
 
 ROR1  4919  1  64239689  64647179  1p32-p31  0  3.922  2.787  0.993  0.053  1.601  1.273  2.295  0.842  2.211  1.142  0.078  0.238  0.801  0.479  3  0.747  1.414  0.044  0.055  0  0.156  1.39  1.079  0  1.259  1.366  1.389  1.479  0.639  1.499  2.611  1.225  0.587  1.262  1.663  2.869  2.826  2.74  0.81  1.199  0.262  0.236  0.928  1.644  1.335  1.874  0.294  1.589  0.121  2.199  1.726  3.285  1.245  2.648  1.8  3.215  0.968  4.133  2.881  
 
 ROR1-AS1  101927034  1  64560124  64579474  -  0  0.211  0.289  0  0  0.088  0.138  0.115  0.117  0.201  0.057  0  0  0.097  0  0.406  0  0  0  0  0  0  0.483  0  0  0.174  0.248  0.097  0.1  0.05  0.101  0.127  0  0.347  0  0  0.774  0.465  0.316  0  0  0  0.028  0  0.269  0.182  0.051  0  0.112  0  0.135  0.23  0.354  0  0.17  0  0.375  0  0.627  0  
 
 UBE2U  148581  1  64669489  64710027  1p31.3  0  0  0  0  0  0  0  0  0  0  0  0  0  0  0  0  0  0  0  0  0  0.039  0  0  0  0  0  0  0  0  0  0  0  0  0  0  0  0  0.062  0  0  0  0  0  0  0  0  0  0  0  0  0  0  0  0  0  0  0  0  0  
 
 CACHD1  57685  1  64936475  65158741  1p31.3  0.386  0.11  0.171  0.633  0.25  0.621  1.914  0.693  0.454  0.985  2.97  0.572  0.397  1.48  0.736  0.017  0.946  1.148  0.022  0.112  0  0.522  0.202  0.04  1.595  1.54  1.893  1.453  1.691  0.577  1.668  1.678  0.555  0.23  0.256  1.964  0.313  0  0.531  2.567  0.914  0.309  1.847  1.402  1.435  0.994  0.539  0.512  0.129  0.672  0.974  0.772  0.719  0.336  1.215  0.785  1.915  0.441  0.541  0.676  
 
 RAVER2  55225  1  65210777  65298914  1p31.3  1.141  1.363  0.272  1.116  1.661  0.864  1.41  1.439  1.295  0.608  1.922  0.574  1.539  3.269  2.147  0.429  1.852  1.466  2.443  2.249  0.539  1.863  0.638  1.011  1.308  1.489  1.587  1.215  1.707  0.9  2.005  1.659  1.705  0.872  0.847  1.888  1.466  0.326  1.214  1.623  1.549  0.866  3.013  1.655  1.176  1.44  1.888  1.349  1.468  2.122  1.794  0.882  1.264  0.708  1.344  1.396  1.324  0.942  2.284  0.952  
 
 JAK1  3716  1  65298905  65432187  1p32.3-p31.3  2.276  4.048  2.866  3.595  4.942  2.595  3.767  3.856  2.808  3.869  3.437  2.955  2.631  3.702  2.493  2.141  3.063  4.117  3.688  1.968  2.031  2.088  2.647  2.058  2.881  2.903  3.086  2.851  3.214  1.891  3.279  3.176  2.828  1.726  2.985  4.147  3.601  3.112  3.831  3.734  2.74  1.83  2.888  0.57  2.623  3.476  2.212  2.964  4.08  3.999  3.736  1.84  4.609  3.759  4.766  4.085  4.77  3.37  5.741  4.638  
 
 LINC01359  101927084  1  65445259  65468159  -  0  0  0  0  0.117  0  0  0  0  0  0  0  0  0  0  0  0  0  0  0.099  0  0  0  0  0  0  0.181  0  0  0.08  0  0  0  0.167  0  0  0  0  0.105  0  0  0  0  0  0  0.08  0.12  0  0  0  0  0  0  0  0  0  0  0  0.122  0  
 
 AK4  205  1  65613231  65697828  1p31.3  1.862  2.47  1.962  2.002  2.22  1.709  0.112  2.153  2.243  3.337  3.056  0.174  0.479  2.576  1.888  1.771  2.146  2.044  0.063  1.685  1.589  0.443  0.348  0.277  0.933  0.428  1.763  0.715  2.849  1.123  1.935  2.804  2.155  1.163  1.087  0.234  1.736  2.741  1.891  2.042  2.019  1.362  1.607  2.627  1.689  3.052  0.935  1.5  2.462  2.215  3.068  1.409  3.439  1.146  2.287  2.265  4.116  0.806  4.03  2.162  
 
 DNAJC6  9829  1  65730376  65881552  1p31.3  0.369  1.239  1.19  0.371  0.706  1.937  0.093  0.155  0.543  1.211  1.274  0.045  0.126  1.023  0.849  0.059  0.658  1.125  0.174  0.04  2.23  0.432  0.424  0.173  0.24  0.881  0.906  1.347  0.208  0.035  0.204  0.223  0.743  0.524  0.348  1.117  0.707  0.698  1.101  0.275  1.161  0.542  0.491  0.189  0.326  1.089  0.145  1.693  0.612  2.367  0.321  0.425  0.295  0.712  0.577  0.897  0.26  0.63  1.345  1.806  
 
 LEPROT  54741  1  65886130  65901690  1p31.3  1.935  4.514  3.141  2.296  3.098  2.577  2.989  3.243  2.621  3.139  2.879  1.817  1.692  2.294  1.725  2.456  1.95  2.883  2.576  1.947  2.3  1.217  1.952  1.441  2.422  2.737  2.485  2.603  2.521  1.672  3.27  3.304  2.198  1.718  2.282  3.434  2.44  3.371  3.542  2.38  3.043  2.374  2.95  2.314  1.675  3.526  2.811  2.358  3.037  4.111  2.977  1.735  2.4  2.376  4.036  3.218  3.353  3.467  4.351  4.678  
 
 LEPR  3953  1  65886334  66103176  1p31  0.34  0.584  0.446  0.449  0.735  0.239  0.237  1.008  0.808  0.421  1.555  0.442  0.21  1.148  0.132  0.146  0.541  0.839  0.921  0.12  2.533  0.181  0.242  0.052  0.438  0.135  0.375  0.151  0.051  0.05  0.122  0.336  0.147  0.193  0.236  1.745  1.53  0.606  0.785  0.208  0.353  0.514  0.238  0.242  0.074  0.389  0.114  0.233  0.87  0.36  0.164  0.224  0.372  0.552  0.58  0.818  0.553  0.835  1.011  0.632  
 
 PDE4B  5142  1  66258192  66840262  1p31  0.252  0.404  0.276  2.337  0.053  0  0.306  0.533  2.044  1.438  1.719  0  0  4.597  0.178  0  0.78  0.071  0.058  1.054  0  0.085  0.471  0.229  1.27  3.035  2.548  2.145  3.594  1.891  2.601  1.061  2.9  1.453  1.189  2.427  0.67  1.73  0.073  3.626  0  1.463  0.184  0.224  0.263  0.269  0.022  0.06  0.085  0.08  0.236  0.352  0.036  0.289  0.105  0.134  0.616  0.133  0  0.049  
 
 LOC101927139  101927139  1  66508182  66516401  1p31.3  0  0  0  0  0  0  0  0  0  0.319  0.241  0  0  0  0  0  0  0.315  0  0  0  0  0  0  0  0  0  0  0  0  0  0.322  0  0  0  0  0  0  0  0  0  0  0  0  0  0  0  0  0  0  0  0  0  0  0  0  0  0  0  0  
 
 SGIP1  84251  1  66999251  67216822  1p31.3  0  0.015  0.238  0.031  0.009  0.078  0.029  0  0.039  0.012  0.023  0.013  0  0.026  0  0  0.012  0.051  0.037  0.015  0  0.053  0.1  0.015  0  0.159  0.058  0.014  0  0.041  0  0.015  0  0.122  0  0.043  0.071  0  0.027  0.021  0.011  0.11  0.02  0.016  0.124  0.802  0.015  0  0  0.009  0  0.125  0  0.013  0.023  0.038  0.013  0.03  0  0.382  
 
 TCTEX1D1  200132  1  67218139  67244730  1p31.3  0  0  0  0  0.025  0  0  0  0  0  0  0  0  0  0  0  0  0  0  0  0  0.099  0  0.048  0  0.306  0.033  0  0  0.024  0  0  0  0.077  0  0  0  0  0.038  0  0  0.034  0.021  0  0  0  0  0  0  0  0  0  0  0  0  0  0  0  0  0  
 
 INSL5  10022  1  67263423  67266942  1p31.3  0  0  0  0  0  0  0  0  0  0  0  0  0  0  0  0  0  0  0  0  0  0  0  0  0  0  0.097  0  0  0.071  0  0  0  0.376  0  0  0  0  0  0  0  0  0  0  0  0  0.075  0  0  0  0  0  0  0  0  0  0  0  0  0  
 
 WDR78  79819  1  67278571  67390570  1p31.3  0.125  0.105  0.208  0.619  0.112  0.11  0.168  0.416  0.242  0.282  0.178  0.059  0.486  0.385  0.272  0.321  0.19  1.216  0.628  0.038  0.143  0.249  0.165  0.149  0.134  0  0.053  0.06  0.095  0.051  0  0.144  0  0.081  0  0.531  0.182  0.33  0.429  0.489  0.218  0.348  0.161  0  0.144  0.481  0.511  0.151  0.412  0.25  0.341  0.426  0.482  0.197  0.753  0.193  0.166  0  0.439  0.47  
 
 MIER1  57708  1  67390577  67454302  1p31.3  0.825  2.635  1.545  1.563  1.555  1.075  1.585  1.997  1.543  1.857  2.575  1.282  1.689  2.177  1.667  2.123  2.17  2.865  2.81  2.384  1.335  2.752  1.398  1.291  1.719  1.316  1.064  1.136  1.306  0.577  1.59  1.356  0.85  0.945  0.842  1.895  1.592  1.673  3.078  1.523  1.506  1.191  1.733  2.164  1.03  1.899  0.696  1.681  1.902  3.003  1.476  1.371  1.56  1.207  1.74  1.673  1.98  1.204  2.873  2.173  
 
 SLC35D1  23169  1  67465014  67520080  1p32-p31  0.264  1.071  0.951  0.379  0.804  0.66  1.665  1.701  0.961  1.566  1.312  0.575  1.258  1.034  0.751  0.381  1.204  1.204  1.74  0.564  0.804  1.152  0.577  0.427  0.798  1.028  0.922  0.684  0.872  0.427  0.861  1.106  0.737  0.313  0.472  1.244  0.627  0.905  1.236  1.038  0.576  0.403  1.398  0.62  0.401  0.994  0.705  0.607  0.803  1.065  1.14  0.519  0.576  0.638  0.439  0.856  0.904  0.394  0.883  0.624  
 
 C1orf141  400757  1  67557858  67600654  1p31.3  0  0  0  0  0  0  0  0  0  0  0  0  0  0  0  0  0  0  0  0  0  0  0  0  0  0  0.034  0  0  0.023  0  0  0  0.13  0  0  0  0  0  0  0  0  0.027  0  0  0  0.027  0  0  0  0  0  0  0  0  0  0  0  0  0  
 
 IL23R  149233  1  67632168  67725650  1p31.3  0  0  0.054  0  0  0  0  0  0.068  0  0  0  0  0  0  0  0.086  0  0  1.47  1.274  0  0.133  0  0  0  0.038  1.136  0  0.326  0  0  0  0.083  0  0  0  0  0  0  0  0.03  0  0  0  0  0.023  0  0.118  0  0  0.074  0  0  0  0  0  0  0  0  
 
 IL12RB2  3595  1  67773046  67862583  1p31.3-p31.2  0  0  0.129  0  0.037  0  0.068  0.118  0.13  0.092  0.126  0.052  0.178  0.078  0.152  0.047  0  0.078  0.042  0.297  0.17  0.111  0.119  0.09  0  1.19  2.355  0.656  1.642  0.672  0.063  0.104  0.922  0.156  0  0.111  0  0.51  0.043  0.066  0.074  0.077  0.041  0.142  0.139  0.511  0.028  0.546  0.145  1.485  0.046  0.367  0.045  0.037  0.047  0.093  0.066  0  0.138  0  
 
 SERBP1  26135  1  67873492  67896123  1p31  4.019  5.32  3.675  4.372  3.464  3.808  4.445  4.504  3.815  4.332  4.926  3.945  4.224  5.387  4.188  4.353  4.546  6.074  5.965  5.492  4.741  4.544  3.28  3.524  4.419  3.046  3.802  3.689  3.581  2.941  4.814  4.955  3.921  3.21  3.316  4.428  4.452  3.36  4.7  4.441  3.972  4.368  4.627  4.624  3.966  4.336  3.622  4.018  4.333  5.621  4.129  3.004  4.432  2.909  3.949  4.17  4.277  3.564  4.835  4.695  
 
 GADD45A  1647  1  68150859  68154021  1p31.2  0.497  5.19  4.92  4.307  0.841  4.458  4.209  4.919  3.889  2.412  3.59  1.861  1.943  3.795  1.535  3.015  4.552  6.657  1.82  1.14  3.807  1.78  3.203  3.078  3.572  2.157  2.01  1.761  3.551  1.395  3.691  3.709  1.088  1.367  1.487  2.247  3.995  2.405  7.243  2.608  2.974  2.478  0.653  3.471  1.813  4.443  3.431  3.523  3.492  6.085  4.766  2.225  3.383  2.672  4.015  2.606  3.695  4.145  5.59  3.858  
 
 GNG12  55970  1  68167148  68299155  1p31.3  1.66  4.571  5.102  4.006  2.632  3.818  4.52  4.606  3.533  4.589  4.516  2.821  3.253  4.068  2.147  3.374  3.445  1.957  0.119  0.172  0.709  0.399  0.423  0.154  4.462  4.289  5.09  4.436  4.5  3.555  5.37  5.655  5.172  3.639  2.903  4.887  3.726  3.851  5.189  3.741  2.808  2.936  0.251  2.526  2.682  4.424  3.676  3.337  5.339  4.625  4.406  2.371  4.358  3.804  4.628  4.435  4.679  3.841  5.403  5.664  
 
 GNG12-AS1  100289178  1  68297970  68668670  1p31.3  0  0.036  0.17  0.211  0.079  0.073  0.302  0.152  0.149  0.219  0.21  0.104  0.238  0.163  0.134  0.199  0.171  0.194  0.077  0.059  0.144  0.117  0.206  0.122  0.146  0.171  0.399  0.288  0.171  0.106  0.274  0.285  0.155  0.299  0.07  0.176  0.232  0.102  0.268  0.124  0.057  0.207  0.063  0.174  0  0.226  1.187  0.112  0.135  0.174  0.192  0.433  0.18  0.152  0.163  0.211  0.072  0.072  0.231  0.142  
 
 DIRAS3  9077  1  68511644  68516460  1p31  0  0.071  0.152  0  0.081  0.099  2.861  0.172  0.274  1.497  4.237  0.059  0  0.538  0  0.066  0.426  0.289  0  0.069  1.922  0.3  0.204  0  0  0.898  0.23  0.177  0  0.086  0.406  0.09  0.124  0.355  0.082  6.308  0  0.221  0.251  0  0  0.584  0.033  0  0  0.475  0  0.116  0.126  0.386  0.087  0.756  0.071  0.068  0.618  0.114  0  0  0  0.379  
 
 WLS  79971  1  68564141  68698284  1p31.3  0.133  1.615  1.008  2.64  0.1  1.48  4.722  3.09  1.889  3.348  4.047  2.347  2.942  3.015  2.434  3.16  3.089  2.052  0.113  0.066  0  0.167  0.211  0  3.264  1.914  3.266  3.377  2.558  2.045  3.623  4.224  3.071  1.746  0.715  1.743  2.814  3.365  2.112  1.725  2.077  2.153  0.528  2.031  2.838  4.602  2.057  2.799  0.558  4.405  2.539  1.64  1.82  0.768  2.239  1.761  0.456  1.516  2.399  2.327  
 
 RPE65  6121  1  68894506  68915642  1p31  0  0.035  0  0  0  0  0  0  0.045  0.042  0.11  0  0  0.835  0  0  0.095  0  0  0  0  0  0  0  0  0  0.047  0  0  0.043  0  0  0.067  0  0  0  0  0  0  0  0  0.306  0.019  0  0  0.033  0.036  0  0.262  0  0.567  0.178  0  0.029  0.105  0  0  0  0  0  
 
 DEPDC1  55635  1  68939834  68962904  1p31.2  1.459  3.534  1.572  1.735  1.522  1.718  2.954  2.684  0.545  2.88  3.767  1.145  1.605  3.671  1.794  1.655  2.125  2.487  3.238  1.432  1.243  0.187  0.804  0.586  2.305  1.755  1.341  1.518  1.788  0.992  2.673  2.89  1.579  1.173  1.25  2.515  2.247  2.727  2.369  2.526  2.303  2.455  2.352  2.588  1.317  2.192  1.847  2.024  1.527  3.278  2.569  1.623  2.263  1.142  2.154  2.248  2.524  1.316  2.918  2.597  
 
 DEPDC1-AS1  101927220  1  68962358  69004310  -  0  0.273  0.109  0  0.096  0.282  0.353  0.136  0  0.131  0.378  0.093  0  0.408  0  0  0.266  0.248  0.269  0.141  0  0.046  0  0  0.2  0  0.128  0.066  0  0.03  0  0.232  0.183  0.197  0.111  0.112  0  0  0.126  0.245  0.176  0.115  0  0  0  0.119  0.039  0  0  0.516  0.392  0  0.146  0.066  0.11  0  0.139  0  0.172  0.197  
 
 LRRC7  57554  1  70225857  70589171  1p31.1  0  0.014  0.105  0  0.011  0.054  0.033  0.031  0.098  0.302  0.148  0.021  0  0.046  0  0  0  0.015  0  0.023  0  0.065  0.053  0  0.193  0.013  0.048  0.06  0  0.041  0  0  0  0.113  0  0  0.427  0.028  0.05  0  0  0.137  0.017  0.021  0.037  0.807  0.036  0  0  0.021  0.013  0.209  0.026  0.037  0.024  0  0  0.082  0  0  
 
 PIN1P1  5301  1  70385004  70386000  1p31  0.237  0  0.337  0  0.073  0  0.264  0  0.28  0.219  0.122  0.2  0  0.067  0  0.154  0.231  0  0.382  0  0  0.224  0.296  0  0  0  0.67  0.157  0.109  0.366  0  0  0  0.261  0  0  0.144  0  0.136  0  0  0.644  0.091  0  0  0.281  0.543  0  0  0.281  0.192  0.859  0  0.079  0.23  0  0  0  0  0  
 
 LRRC40  55631  1  70610484  70671361  1p31.1  0.951  3.057  0.959  1.513  1.225  1.212  1.407  1.578  1.229  1.771  2.237  0.793  1.501  2.257  1.254  1.562  1.744  3.066  3.609  2.158  1.469  1.882  0.673  0.635  1.809  1.265  0.747  0.692  1.142  0.468  1.855  1.027  0.7  0.564  1.056  1.908  1.002  1.468  2.242  1.501  1.135  1.037  2.138  1.767  0.718  1.579  0.797  1.23  0.9  2.661  2.284  0.773  1.37  0.856  1.551  1.297  1.298  1.331  2.421  2.107  
 
 SRSF11  9295  1  70671364  70717701  1p31  3.057  5.84  2.75  3.068  3.897  3.033  3.903  4.235  3.265  4.709  4.845  2.922  4.086  4.978  4.207  4.062  4.28  5.582  5.896  4.85  4.137  5.102  2.934  2.71  3.604  3.61  3.631  3.129  3.459  2.361  4.639  3.875  3.44  2.517  2.771  4.105  3.46  3.591  4.869  3.855  3.766  3.313  4.506  4.647  3.28  3.919  3.324  3.751  3.302  5.211  3.995  2.787  3.804  2.464  4.175  3.595  3.556  2.977  4.788  4.166  
 
 ANKRD13C  81573  1  70724684  70820417  1p32.3-p31.3  0.799  2.957  0.942  1.145  1.116  0.891  1.413  1.739  1.069  1.77  1.768  0.561  1.13  1.637  1.06  0.89  0.934  1.357  1.892  0.645  0.827  0.86  0.317  0.451  1.123  1.176  1.18  0.95  1.002  0.528  1.279  0.962  0.763  0.488  0.566  1.653  0.847  1.524  1.447  1.335  1.254  0.863  1.033  1.235  0.62  1.235  0.999  1.324  0.942  2.124  1.91  0.621  1.484  0.816  1.035  1.041  1.433  0.666  1.695  1.306  
 
 HHLA3  11147  1  70820492  70833705  1p31.1  1.581  0.383  3.049  3.061  1.821  2.282  3.559  2.821  2.076  4.048  2.812  1.369  3.105  2.73  2.288  1.31  2.807  0.899  3.071  1.135  1.487  2.814  1.142  1.288  0.82  1.558  2.228  1.691  1.487  0.43  2.187  1.337  1.031  1.117  1.052  1.476  2.002  2.5  1.675  2.489  2.889  1.084  3.279  2.11  1.761  3.242  2.626  1.98  1.866  3.519  3.01  1.446  2.756  2.055  1.799  3.305  1.553  1.852  1.858  2.294  
 
 CTH  1491  1  70876900  70905534  1p31.1  0.323  3.692  0.955  0.454  0.755  0.875  0.607  0.688  0.886  0.702  1.548  0.113  1.057  1.908  0.769  0.555  1.504  1.302  0.503  0.929  1.909  0.643  2.166  0.33  0.339  1.021  0.953  0.952  1.129  0.672  1.366  0.739  0.441  0.39  0.377  0.244  0.609  0.786  0.8  2.197  0.478  0.539  0.765  1.888  0.434  0.945  0.524  0.831  1.722  2.567  1.845  0.695  0.62  0.593  0.521  0.858  0.737  0.218  1.213  0.452  
 
 LOC101927244  101927244  1  71172135  71252151  1p31.1  0  0  0.082  0  0  0  0.066  0.105  0.09  0  0  0  0  0  0  0  0  0  0  0  0  0.036  0  0  0  0  0.034  0  0  0.037  0  0  0  0.178  0  0  0  0  0  0  0  0  0  0  0  0  0  0  0  0  0  0.108  0  0.037  0  0  0  0  0  0  
 
 PTGER3  5733  1  71318035  71513491  1p31.2  0.228  0.286  0.571  0  4.177  0  0.185  0  0.09  0.061  0  0  0  0.034  0  0  0  0.158  0  0  1.49  0.092  0  0  0  0  0.117  0  0  0.106  0  0  0  0.189  0  0  0  0  0  0  0  0.174  0.053  0  0  0.082  0.072  0.133  0  0.113  0  0.621  0  0.055  0  0  0  0  0  0  
 
 ZRANB2-AS1  100132618  1  71512188  71532865  1p31.1  0.201  0.166  0.506  0.409  0.611  0.195  0.123  0.267  0.503  0.359  0.341  0  0.136  0.235  0.106  0.463  0.283  0.401  0.497  0.435  0.516  0.806  0.308  0.108  0.192  0.082  0.231  0.187  0.234  0.049  0.256  0.079  0  0.397  0.186  0.187  0.139  0.196  0.644  0.286  0.142  1.194  0  0.126  0  0.33  0  0  0  0.324  0.266  1.173  0.201  0.403  0.397  0  0.295  0.39  0.418  0.167  
 
 ZRANB2  9406  1  71528973  71546972  1p31  1.381  3.758  1.924  2.806  2.854  1.898  2.396  2.678  2.655  2.954  3.371  1.327  2.359  2.826  1.953  2.89  2.503  4.079  4.965  4.124  2.904  3.725  2.285  1.818  2.502  1.866  1.828  2.085  2.426  0.932  3.021  2.379  2.392  1.766  1.315  2.529  1.893  1.896  3.828  2.245  2.027  2.308  2.804  2.582  1.892  2.504  1.464  2.043  1.59  4.233  2.968  1.538  2.262  1.906  2.482  2.158  2.892  2.072  3.017  2.696  
 
 ZRANB2-AS2  100852410  1  71547006  71703406  1p31.1  0  0.285  0  0  0.119  0  0  0  0.165  0.14  0  0  0  0.198  0  0  0  0.585  0.276  0  0  0.097  0.382  0.211  0.208  0.17  0.158  0  0.17  0.05  0.219  0  0  0.28  0  0.163  0  0.209  0  0  0  0.132  0  0.179  0  0  0  0  0  0  0  0  0.163  0  0.437  0  0.306  0  0.366  0  
 
 NEGR1  257194  1  71868624  72748277  1p31.1  0  1.246  0.91  0.442  0.031  1.723  0.054  0.262  0.121  0.426  1.1  0.068  0.039  0.031  0.044  0.015  0.035  0.043  0.365  0.051  0.051  0.137  0.09  0.066  0.364  0.059  0.067  0.793  0.038  0.067  0.027  0.253  0.033  0.122  0.033  0.118  0.877  0.419  0.458  0.083  0.26  0.068  0.064  0.036  0.361  0.084  0.05  0.527  0.096  0.982  1.964  0.108  0.167  0.156  0.698  0.112  0.04  0.237  0.196  1.567  
 
 LINC01360  101927295  1  73771852  73804560  -  0  0  0  0  0  0  0  0  0  0  0  0  0  0  0  0  0  0.097  0  0  0  0  0  0  0  0  0  0.139  0  0  0  0  0  0  0  0  0  0  0  0  0  0.192  0  0  0  0  0  0  0  0  0  0.406  0  0  0  0  0  0  0  0  
 
 LRRIQ3  127255  1  74491701  74663871  1p31.1  0  0.356  0.066  0.122  0.116  0  0.06  0.15  0.065  0.03  0.213  0.157  0.07  0.117  0  0.034  0  0.647  0.496  0.087  0  0.197  0.237  0.059  0.06  0.053  0.073  0.134  0.212  0.154  0.36  0.098  0  0.129  0.053  0.098  0.096  0  0  0.17  0.272  0.218  0.19  0.052  0  0  0.043  0  0  0.24  0.072  0.081  0.231  0.141  0.204  0.153  0.06  0.115  0.124  0.24  
 
 FPGT  8790  1  74663895  74674386  1p31.1  0.282  1.655  0.494  0.691  1.156  0.202  0.808  0.605  0.811  1.092  1.132  0.429  0.811  0.962  0.581  0.627  0.74  1.19  1.169  0.952  0.542  0.462  0.285  0.175  0.693  0.737  0.491  0.468  0.836  0.388  1.133  0.808  0.519  0.403  0.414  1.426  0.19  0.626  1.186  0.875  0.746  0.54  0.973  0.75  0.35  0.521  0.778  0.296  0.253  1.002  0.649  0.284  0.535  0.526  0.561  0.594  0.712  0.41  0.627  0.938  
 
 FPGT-TNNI3K  100526835  1  74663895  75010116  1p31.1  0  0.039  0  0.107  0  0  0.038  0  0  0.108  0.146  0.103  0  0.117  0  0  0.078  0  0.096  0  0  0  0  0  0  0  0.02  0.083  0  0  0  0.123  0  0  0  0  0.055  0  0  0  0  0.084  0  0.123  0  0.051  0  0  0  0  0.064  0.165  0  0.022  0  0  0  0  0  0  
 
 TNNI3K  51086  1  74701070  75010116  1p31.1  0  0  0  0  0  0  0  0  0.083  0  0  0.124  0  0  0  0  0  0.08  0  0  0  0.062  0.125  0.061  0  0  0.05  0.08  0  0.036  0  0  0  0.057  0  0  0  0  0.019  0  0  0  0.022  0  0  0  0.023  0  0  0  0  0  0  0  0  0  0  0  0  0.043  
 
 ERICH3  127254  1  75033794  75139422  1p31.1  0  0.012  0.107  0  0.038  0.021  0.016  0.016  0.098  0.047  0.039  0.018  0  0.018  0  0.016  0  0.045  0.009  0.029  0  0.069  0.122  0.107  0  0  0.064  0.008  0  0.045  0  0.017  0  0.134  0.015  0  0  0  0.034  0  0  0.156  0.017  0  0.019  0.034  0.075  0  0  0  0  0.224  0.011  0.068  0.014  0  0.013  0  0  0  
 
 ERICH3-AS1  101927320  1  75043113  75091782  1p31.1  0  0  0.085  0  0  0  0  0  0.066  0  0  0  0  0  0  0  0  0  0  0  0  0.019  0.15  0  0  0  0.042  0.02  0  0.022  0  0  0  0.109  0  0  0  0  0.012  0  0  0.019  0.026  0  0  0  0.031  0  0  0  0  0.115  0  0.014  0  0  0  0  0  0  
 
 CRYZ  1429  1  75171171  75199092  1p31.1  1.871  3.819  1.387  1.638  2.783  1.809  1.602  1.915  1.948  2.901  3.478  1.593  2.762  3.078  2.102  3.346  2.805  3.504  4.025  1.607  2.224  1.747  1.243  0.745  2.249  1.402  1.324  1.57  1.665  0.853  2.4  1.113  1.471  0.985  2.843  4.458  1.379  2.269  1.406  3.201  1.758  2.201  3.112  2.833  1.298  2.431  0.174  1.925  1.949  3.784  3.027  1.453  4.04  2.925  4.153  3.684  3.81  0.843  3.364  4.339  
 
 TYW3  127253  1  75198835  75232360  1p31.1  1.802  5.113  1.589  1.898  1.818  1.531  2.836  2.353  1.126  2.546  2.568  0.95  2.171  3.901  2.404  2.02  1.739  4.262  3.614  3.444  2.558  2.366  1.432  1.261  1.945  1.765  2.911  2.204  2.151  1.763  1.923  2.159  1.94  1.244  1.214  2.535  1.873  2.2  1.96  2.776  1.82  1.432  3.227  2.234  1.346  1.374  2.418  2.215  1.402  3.263  2.182  1.105  2.503  1.223  2.163  2.124  2.007  1.425  2.643  2.168  
 
 LHX8  431707  1  75594118  75627218  1p31.1  0  0  0.099  0  0.038  0.059  0.508  0.496  0  2.539  0.046  0  0  0.034  0  0  0  0  0  0  0  0.118  0.12  0.062  0  0  0.203  0.029  2.017  0.049  0  0.07  0  0.136  0.925  0.407  0  0  0.03  0  0  0.447  0.024  0  0  0.037  0.024  0  0  0.24  0  0.366  0  0.331  0  0.073  0  0  0  0  
 
 SLC44A5  204962  1  75667815  76076799  1p31.1  0  0.023  0.068  0  0.115  0.11  0.051  0.22  0.365  0.085  0.247  0.247  0  0.513  0.065  1.058  0.172  0.826  0.577  0.032  0.233  0.379  0.169  0.087  0.056  0  0.079  0.027  0.047  0.082  0  0.077  0  0.076  0.037  0.408  0.167  0.536  0.053  0.71  1.118  0.395  0.051  0.436  0  0.028  0.058  0.074  0.059  0.255  0.072  0.158  0.024  0.038  0.032  0.133  0.054  0  0  0.096  
 
 ACADM  34  1  76190031  76229363  1p31  1.157  4.34  1.237  2.211  1.848  1.362  1.941  2.177  2.085  2.202  2.934  1.474  2.047  3.132  1.865  1.635  3.16  4.134  5.033  3.094  2.543  3.124  1.289  0.986  2.119  2.546  1.534  1.635  2.275  1.092  3.285  1.783  1.916  1.818  1.015  2.557  1.293  1.806  2.71  1.927  1.226  2.161  2.453  1.651  0.714  1.752  1.31  1.656  1.024  3.789  2.373  1.287  1.217  1.278  2.224  1.431  2.267  1.265  2.239  2.398  
 
 DLSTP1  1744  1  76207686  76210698  1p31  1.758  0.495  0.663  1.186  1.197  0.76  0.54  0.749  0.738  1.033  0.969  1.015  1.201  1.101  1.246  0.923  1.327  0.396  1.11  1.322  0.545  1.468  0.422  0.458  0.776  0.943  1.355  1.111  1.092  0.884  0.775  0.521  0.989  0.827  1.074  0.79  0.9  0.911  1.469  1.12  0.584  1.066  1.362  0.626  0.51  0.757  2.503  0.93  0.681  0.981  1.002  0.745  1.249  0.62  1.728  0.503  1.051  0.569  0.894  0.567  
 
 RABGGTB  5876  1  76251878  76260775  1p31  2.736  7.187  2.734  2.842  2.843  2.646  4.665  3.59  2.939  3.419  4.416  2.223  3.669  5.653  3.214  3.346  4.412  6.589  5.364  3.664  3.741  3.604  1.555  1.793  3.709  3.057  3.267  3.327  3.348  2.819  3.996  3.355  3.169  2.49  2.754  3.825  2.857  3.42  5.377  3.094  2.719  3.333  4.702  2.695  1.95  2.819  3.406  3.523  1.684  5.813  3.573  1.69  2.811  2.707  3.166  2.474  3.11  3.49  4.041  3.071  
 
 MSH4  4438  1  76262555  76378923  1p31  0  0.106  0  0  0  0  0  0  0  0.043  0.03  0.045  0  0.298  0.131  0.043  0.177  0.044  0.048  0  0  0.078  0  0  0  0  0.047  0.022  0.037  0.154  0  0  0  0.063  0  0.048  0  0  0.024  0  0  0.1  0.966  0.215  0  0.074  0.38  0  0  0.091  0  0.122  0  0.029  0.029  0  0.033  0  0  0  
 
 ASB17  127247  1  76384557  76398116  1p31.1  0  0  0  0  0  0  0  0  0  0  0  0  0  0  0  0  0  0  0  0  0  0  0  0  0  0  0.066  0  0  0  0  0  0  0  0  0  0  0  0  0  0  0  0  0  0  0  0  0  0  0  0  0  0  0  0  0  0  0  0  0  
 
 LOC101927342  101927342  1  76477008  76485041  1p31.1  0  0  0  0  0  0  0  0  0  0  0  0  0  0  0  0  0  0  0  0  0  0.42  0  0  0  0  0  0  0  0  0  0  0  0  0  0  0  0  0  0  0  0.915  0  0  0  0  0  0  0  0  0  0  0  0  0  0  0  0  0  0  
 
 ST6GALNAC3  256435  1  76540388  77096669  1p31.1  0  0  0.267  0.455  0  0  0.391  0.302  0.241  0.975  0.701  0  0  0.47  0  0.104  0  0  0.882  0.077  0  0.074  0  0  0.166  1.466  2.905  0.629  1.155  1.609  3.254  1.855  2.518  1.311  0.061  0.167  0  0  0.044  0  0  0.149  0.081  0.246  0  0.363  0.353  0.416  0  0.535  0  0.35  0  0.074  0.301  0  0  0.358  0.132  0.109  
 
 ST6GALNAC5  81849  1  77333185  77529737  1p31.1  0.806  0.3  3.406  0.809  0.061  1.418  0.371  0.259  0.12  0.3  0.435  0.125  0  0.904  0  0.059  0  0.473  0  0.071  0  0.199  0.163  0.13  0  0  0.124  0.494  0  0.173  0  0.118  0  0.175  0  0.288  0  0.981  0.273  0  0.531  0.157  0.069  0  0.555  0.04  0.331  0  0  0.129  0.036  0.563  0.23  0.118  0.118  0.303  0.089  0.979  0  1.271  
 
 PIGK  10026  1  77554666  77685132  1p31.1  0.731  3.543  1.401  1.257  1.462  0.873  2.21  1.982  1.462  2.317  2.276  0.596  1.011  1.769  0.753  0.871  1.664  1.747  2.568  1.804  1.089  0.756  0.688  0.569  1.596  1.593  1.029  1.209  1.624  0.489  1.988  1.31  1.062  0.852  0.668  1.978  0.945  1.559  1.656  1.424  1.231  1.087  1.227  1.266  0.779  1.726  0.721  0.925  0.614  2.374  2.519  0.673  1.437  0.997  1.608  0.943  1.82  1.189  1.885  2.11  
 
 AK5  26289  1  77747661  78025654  1p31  0.068  0.373  1.148  0.246  0.042  0.492  0.845  0.44  0.436  2.389  0.9  0.054  0.089  0.088  0.091  0.049  0.05  0.222  0.047  0.201  0.094  0.164  0.234  0.052  3.216  0.048  3.202  0.706  1.214  0.096  0.519  0.313  0.643  0.395  0.242  0.171  0.538  0.064  0.804  0  0.047  0.384  0.603  0.09  0.078  0.374  0.47  0.099  0.154  0.314  2.195  0.417  0.839  0.721  2.629  1.415  1.485  0.079  2.207  1.287  
 
 ZZZ3  26009  1  78028100  78149112  1p31.1  2.017  4.291  1.404  1.725  1.189  1.258  2.4  2.315  1.764  2.247  2.799  0.968  2.048  2.86  1.964  1.867  1.815  2.284  2.93  1.946  1.38  1.687  0.865  0.685  1.718  1.766  1.98  1.981  2.119  1.192  2.759  1.443  1.935  0.949  0.752  1.982  1.748  2.141  2.195  2.053  1.679  1.037  2.137  1.617  1.378  1.672  1.639  1.598  1.189  2.703  2.437  0.94  1.762  1.072  1.945  1.465  2.112  0.866  2.221  2.179  
 
 USP33  23032  1  78161673  78225564  1p31.1  1.208  3.807  1.535  1.861  1.684  1.537  2.423  2.409  2.276  2.563  3.075  1.006  2.244  2.691  1.963  1.837  2.567  2.02  3.509  2.039  1.684  1.849  0.945  0.661  2.018  2.132  1.69  1.98  1.78  1.23  2.469  1.845  1.766  1.173  1.204  2.524  1.713  2.009  2.167  2.451  1.561  1.171  2.211  1.971  1.496  2.657  0.785  1.372  1.301  2.978  2.461  1.135  2.007  1.36  1.905  1.4  2.397  1.356  2.246  2.513  
 
 FAM73A  374986  1  78245308  78345225  1p31.1  0.425  1.924  0.749  0.943  0.711  0.671  1.481  1.064  0.963  1.131  1.078  0.256  1.248  0.95  0.805  0.558  0.939  0.591  0.959  0.664  0.284  0.601  0.287  0.135  0.945  1.082  0.676  1.132  1.126  0.521  1.325  1.129  0.925  0.581  0.469  1.368  0.727  1.049  1.739  1.323  1.07  0.508  1.369  0.916  0.656  1.487  0.591  0.687  0.59  1.237  1.532  0.452  0.955  0.678  0.876  0.687  1.183  0.496  1.059  1.236  
 
 NEXN-AS1  374987  1  78347032  78355224  1p31.1  0  0.582  0.933  0.117  0.041  1.157  0.709  0.615  0.22  0.411  0.39  0.061  0  0.099  0.067  0  0  0.171  0.03  0.17  0  0.173  0.311  0  0  0.035  0.088  0.046  0  0.121  0.045  0  0  0.226  0  0.397  0.403  0.439  0.55  0  0  0.536  1.126  0.254  0  0.482  0.309  0.921  0  1.028  0.311  0.86  0.517  0.374  1.103  0.125  0.498  0  0.67  0.454  
 
 NEXN  91624  1  78354199  78409578  1p31.1  0.113  4.212  3.264  0.149  0.191  2.367  2.475  2.7  0.348  1.897  1.887  0.031  0.21  0.415  0.329  0.188  0.147  0.497  1.734  0.466  0.199  2.387  0.114  0  0.105  0.352  0.561  0.144  0.226  0.166  0.227  0.092  0.42  0.431  0.296  1.54  1.306  1.661  1.995  0.278  0.181  0.593  2.092  0.295  0.318  2.518  0.484  1.511  0.613  1.773  1.719  0.85  1.908  1.638  2.697  0.874  2.559  0.392  2.045  3.042  
 
 FUBP1  8880  1  78412166  78444889  1p31.1  3.286  3.787  2.184  2.913  3.645  3.058  3.876  4.101  2.289  3.677  4.887  3.366  4.408  3.85  3.625  3.913  3.078  3.945  5.094  2.783  3.999  3.837  1.716  2.863  4.033  3.022  3.037  3.647  3.142  2.537  3.912  3.763  3.082  1.265  3.226  4.135  3.916  3.305  3.141  4.246  3.81  2.758  4.682  3.337  3.663  3.456  2.004  3.419  3.43  4.136  3.549  2.69  3.95  2.39  3.582  3.081  4.139  4.048  4.303  4.518  
 
 DNAJB4  11080  1  78470635  78482995  1p31.1  0.082  2.598  2.128  2.444  0.435  1.939  2.066  1.913  1.484  2.648  2.684  0.43  2.116  1.518  0.665  1.056  1.198  0.797  1.269  0.379  0.902  0.558  0.594  0.542  2.009  0.922  0.548  0.626  0.763  0.349  1.55  0.747  0.524  0.517  1.157  1.704  1.039  2.023  3.825  1.343  1.374  1.423  0.476  0.655  0.421  1.285  0.022  1.999  0.394  3.109  1.639  1.147  1.19  2.088  1.459  1.041  2.107  3.299  1.12  2.556  
 
 GIPC2  54810  1  78510645  78604133  1p31.1  0  0.023  0.038  0  0  0  0.043  0.046  0.048  0.038  0.038  0.928  1.047  0.025  0  0.968  1.653  0.141  0  0  0  0.056  0  0  0  0  0.058  0  0  0.123  0  0  0  0.174  0  0.035  0  0  0.057  0  0  0.048  0.029  0  0  0.025  0.106  0  0  0  0  0.23  0.017  0.028  0.025  0  0.027  0.416  0  0.173  
 
 MGC27382  149047  1  78695282  78835147  1p31.1  0  0  0  0.276  0  0  0  0  0  0.478  0  0  0  0  0  0  0  0  0  0  0  0  0  0  0  0  0.072  0  0  0.037  0  0  0  0  0  0  0  0  0.043  0  0  0  0  0  0  0  0  0  0  0  0.156  0.315  0  0  0  0  0  0  0  0  
 
 PTGFR  5737  1  78956727  79006386  1p31.1  0  0.111  0.583  1.428  0  0.307  2.033  0.592  0.104  2.763  0.776  0  0  0  0  0.226  0  0.031  0  0.031  0  0.081  0.07  0  0.184  0  0.038  0.05  0  0.036  0  0  0  0.152  0  0  0.018  0  1.025  0  0  0.208  0.015  0  0  0.013  0.018  0  0  0.084  1.92  0.228  0.452  0.172  0.024  0.619  1.144  0.147  0.044  0.047  
 
 IFI44L  10964  1  79086066  79111830  1p31.1  0.123  0.081  0.078  4.25  0.015  0  1.234  1.099  0.212  0.967  2.087  0.107  0.985  0.013  0  4.095  0  0  0.033  0.023  0  0.194  0.277  2.043  1.169  0  0.277  0.016  0  0.103  0.018  0.078  0  0.139  0.03  0  0.042  0.267  1.669  0.075  0.284  1.024  0.075  0  0.097  0.044  0.39  0  0  0.091  0.258  0.114  0.243  2.476  0.232  0.465  3.47  0  0.044  0.093  
 
 IFI44  10561  1  79115476  79129763  1p31.1  0  3.605  0.18  4.63  0.109  0.552  3.625  3.165  0.68  2.945  3.883  0  1.321  0.139  0  6.127  0.195  0  3.412  0.159  0  2.386  0.316  2.414  4.494  0.332  1.687  1.238  1.306  0.052  0.128  1.596  1.346  0.827  0  0  0.064  3.647  4.254  0  1.072  1.632  0  0  0.555  0.196  1.194  0.962  0.13  2.774  1.115  0.268  1.206  3.314  0.941  1.259  4.503  0.486  2.952  0.835  
 
 ADGRL4  64123  1  79355448  79472495  1p33-p32  0  0  0.077  0  0  0  0.513  0  0.054  0.018  0  0.068  0  0  0  0  0  0.035  0.023  0.029  0  0.053  0.116  0.034  0.457  0  0.074  0.384  0  0.041  0  0  0  0.057  0  0  0.028  0  0.024  0.074  0  0.328  0.028  0  0  0  0.025  0  0  0  0.029  0.113  0.019  0.038  0  0.046  0  0.105  0.057  0  
 
 LOC101927412  101927412  1  81001439  81112473  1p31.1  0  0  0  0  0  0  0  0  0  0  0  0  0  0  0  0  0  0  0  0  0  0  0  0.554  0  0  0.082  0  0  0  0  0  0  0  0  0  0  0  0  0  0  0  0  2.49  0  0  0  0  0  0  0  0  0.304  0  1.237  1.059  0  0  0.565  0  
 
 LOC101927434  101927434  1  81979564  82023387  1p31.1  0  0  0  0  0  0  0  0  0  0  0  0  0  0  0  0  0  0.461  0  0  0  0  0  0  0  0  0  0  0  0  0  0  0  0  0  0  0  0  0  0  0  0  0  0  0  0  0  0  0  0  0  0  0  0  0  0  0  0  0  0  
 
 ADGRL2  23266  1  82165454  82458422  1p31.1  0.55  1.585  1.746  2.53  0.075  0.816  0.035  1.916  1.486  0.684  1.631  0.027  0.07  0.025  0  0  0  0.375  0.087  0.063  0.116  0.054  0.124  0.129  2.591  0  1.239  0.018  0.098  0.087  0.2  1.992  0  0.114  1.197  0.066  1.228  0.111  2.64  0.875  2.581  0.43  0.325  4.16  2.644  2.999  1.931  1.311  2.37  3.738  2.159  0.155  2.543  0.177  2.746  1.071  0  0.214  2.071  0  
 
 LINC01361  101927498  1  83439565  83451891  -  0  0  0.096  0  0.051  0  0  0  0  0.069  0  0  0  0  0  0  0  0  0  0  0  0  0.262  0.209  0  0  0.06  0  0  0.03  0  0  0  0.192  0  0  0  0  0.044  0  0  0  0  0  0  0  0  0  0  0  0.056  0.179  0  0  0  0  0  0  0  0  
 
 LOC101927587  101927587  1  84041470  84326679  -  0  0  0.124  0  0.063  0.09  0.061  0.025  0.048  0.059  0.029  0.119  0  0.052  0  0  0  0.085  0.054  0.084  0  0.05  0.181  0  0  0.132  0.298  0.029  0.05  0.215  0.13  0.129  0  0.193  0.048  0  0  0  0.064  0  0.038  0.322  0.054  0  0.085  0.085  0.242  0  0  0  0  0.092  0  0.092  0.024  0  1.909  0.039  0  0  
 
 LOC101927560  101927560  1  84267198  84326229  -  0  0.04  0.09  0  0.064  0  0  0  0.128  0.204  0  0  0  0.033  0  0  0  0  0  0  0  0.027  0.254  0  0  0.329  0.098  0.049  0.073  0.043  0.194  0.2  0  0.203  0  0  0  0  0  0  0  0.265  0.025  0  0  0  0.03  0  0.111  0.104  0  0  0  0.125  0  0  0  0  0  0  
 
 TTLL7  79739  1  84335056  84464833  1p31.1  0.059  2.323  0.662  0.761  1.033  0.86  0.885  1.474  0.604  1.576  2.832  0  0.075  1.511  0.381  0  0.144  1.331  0.032  0.085  0  0.143  0.078  0.041  0.151  0.062  0.669  0.452  0.475  0.763  1.08  0.348  0.464  0.293  0.249  1.098  0.408  1.305  0.64  0.899  0.446  0.629  0.474  0.316  0.219  0  0.047  0.678  0.122  0.95  0.765  0.538  0.045  0.628  0.457  0.163  0.892  0.344  0.213  0.313  
 
 PRKACB  5567  1  84543657  84704181  1p31.1  0.896  3.773  2.009  1.042  1.261  2.022  2.478  2.3  2.345  2.526  3.195  0  0.693  4.353  1.06  0.205  0.075  2.614  4.012  2.087  0.266  2.828  1.767  1.418  2.493  1.462  1.86  2.221  2.005  1.369  2.716  2.691  2.363  2.178  0.893  2.627  1.477  0.576  3.091  1.833  1.754  1.484  1.905  0.916  0.644  0.13  0.999  0.843  1.266  1.493  2.251  0.824  1.755  1.425  1.498  0.902  2.325  0.286  1.417  1.934  
 
 SAMD13  148418  1  84764048  84816481  1p31.1  0.163  0.702  0  0  0.218  0  0  0.112  0.349  0.083  0.488  0.311  0.403  1.823  0  0.321  0.347  0.453  1.164  0.428  0  0.416  0  0  0.292  0.344  0.935  0.233  0.255  0.147  0.563  0.288  0.367  0.516  0.133  0.72  0.204  0.19  0.085  0.184  0.383  0.374  0.039  0  0  0  0.051  0  0  0.253  0.242  0.259  0.049  0  0.317  0  0.082  0  0  0.141  
 
 UOX  391051  1  84830640  84863576  1p22  0  0.127  0.127  0  0  0.173  0.097  0  0  0.093  0.055  0  0  0.412  0  0  0  0.13  0  0  0  0.06  0  0  0  0  0.248  0.047  0  0.033  0.073  0  0  0  0  0  0  0.098  0  0  0  0.761  0.038  0  0  0  0.026  0  0  0.062  0  0  0  0  0  0  0  0  0  0  
 
 DNASE2B  58511  1  84864214  84880691  1p22.3  0  0  0.166  0  0  0  0  0  0  0  0  0  0  0.252  0  0  0  0  0  0  0  0.084  0  0  0  1.458  0.11  0.123  0.139  0  1.864  0.235  0  0  0  0  0  0  0  0  0  0  0  0  0  0  0  0  0  0  0  0  0  0  0  0  0  0  0  0  
 
 RPF1  80135  1  84944919  84964033  1p22.3  1.774  6.129  1.793  2.699  2.792  2.258  3.233  3.027  2.315  2.548  3.845  1.819  1.513  4.833  1.74  2.754  2.764  4.945  4.685  3.189  2.924  2.941  1.904  1.259  2.917  2.184  2.398  2.111  2.521  1.258  3.338  2.655  2.077  1.903  1.796  2.762  1.963  2.41  3.29  2.863  2.545  2.42  3.179  2.666  2.045  2.046  1.595  2.492  1.587  4.303  3.273  1.513  2.134  1.566  2.624  1.794  2.756  2.239  2.616  2.627  
 
 GNG5  2787  1  84964005  84972262  1p22  3.601  9.521  4.322  5.804  5.529  5.985  5.825  5.769  4.865  5.517  5.872  4.975  4.797  6.297  3.562  5.769  5.826  8.394  7.239  6.735  6.248  5.557  3.967  4.377  6.029  4.642  4.318  4.162  4.852  2.955  6.383  5.193  4.562  4.446  4.994  5.339  4.133  5.657  6.244  4.884  5.381  5.121  6.298  5.565  4.71  6.009  4.606  5.488  4.682  6.743  5.225  3.534  5.153  4.829  5.499  4.97  5.627  5.885  6.221  5.171  
 
 SPATA1  100505741  1  84971983  85022178  1p22.3  0  0.529  0  0  0.037  0  0.039  0.201  0  0.19  0.162  0  0  0.195  0.071  0.09  0.099  0.134  0  0.074  0  0.043  0  0.091  0  0.109  0.1  0.092  0.157  0.076  0.055  0  0  0.127  0.077  0.085  0.098  0.195  0.046  0  0.117  0.087  0.032  0  0  0.127  0.025  0.069  0  0  0.091  0.17  0.061  0.052  0  0  0.126  0  0  0  
 
 CTBS  1486  1  85018803  85040163  1p22  0.606  4.651  1.221  1.518  1.868  0.838  2.178  1.891  1.706  1.87  2.154  0.456  1.161  2.43  0.884  1.552  1.667  1.654  1.859  1.68  1.006  0.899  1.15  0.903  1.075  1.558  1.216  1.214  1.762  0.719  2.207  1.827  0.805  1.095  0.839  2.001  1.403  2.081  1.524  1.619  2.124  1.129  0.804  0.749  0.283  1.195  1.64  0.607  0.888  1.534  2.017  0.624  0.909  1.36  1.753  0.708  1.545  0.896  1.178  1.677  
 
 LINC01461  103695366  1  85063646  85086714  1p22.3  0  0.215  0  0  0  0  0.217  0.228  0.289  0.162  0.182  0  0  0.19  0  0  0  0  0  0  0  0.369  0  0  0  0  0  0  0.358  0  0.236  0  0  0  0  0  0  0.38  0  0  0  0.347  0  0  0  0  0  0  0  0  0.196  0.728  0.142  0  0.251  0  0.208  0  0  0  
 
 LINC01555  439927  1  85093912  85100703  1p22.3  0  0  0.114  0  0.024  0  0  0  0.097  0.018  0.038  0  0  0.031  0  0  0  0.042  0  0.077  0  0.051  0.114  0  0  0  0.12  0  0  0.067  0  0  0  0.195  0  0  0  0  0.026  0  0  0.33  0.029  0  0  0.055  0.109  0  0  0  0  0.113  0  0.034  0.033  0  0  0  0  0  
 
 SSX2IP  117178  1  85109389  85156240  1p22.3  0.946  3.698  0.557  1.493  1.008  1.008  1.973  1.088  1.572  1.549  3.054  0.413  1.115  2.952  1.332  1.096  1.853  2.198  1.993  1.282  1.995  1.214  0.539  0.509  0.764  1.159  1.352  1.1  1.117  0.619  1.34  0.896  0.847  0.526  0.775  1.618  0.753  1.285  1.303  1.764  1.121  1.277  1.922  1.102  0.951  1.227  1.419  1.191  1  2.131  2.228  0.748  1.141  0.867  1.427  1.001  1.212  0.935  1.263  1.2  
 
 LPAR3  23566  1  85279085  85358896  1p22.3  0  0  0.172  0.61  0.047  0.093  0.403  0.191  0.168  0.037  0  0.085  0  1.869  0  0.043  0.076  0.129  0  0.111  0  0.291  0.299  0  0.499  0  1.678  0.167  2.225  0.743  0.211  0  0  0.197  0.104  0  0.659  0  0.066  1.236  0  0.841  0.036  0.316  1.073  0.566  0.081  1.511  0.687  1.525  2.76  0.715  0  0.117  0.082  0  0  1.277  0.124  0  
 
 MCOLN2  255231  1  85391265  85462805  1p22  0.083  1.256  0.195  0.319  0.678  0.199  1.411  0.095  0.867  0.242  1.926  0  0.172  1.007  1.21  0.027  1.351  0  0.023  0.111  0  0.531  0.13  0  0.641  0.844  0.968  1.327  1.778  0.927  1.387  1.507  1.222  0.736  0.414  0.086  0.036  1.068  0.558  0.613  0.073  1.14  0.243  0.176  0  0  0.969  0.441  0.099  1.157  0.315  0.295  0.047  0.2  0.637  0.182  0.075  0.297  1.63  0.226  
 
 MCOLN3  55283  1  85483764  85514223  1p22.3  0.133  2.144  0.181  0.344  0.998  0.443  0.552  1.145  0  0.314  0.053  0.04  0.15  2.511  0.897  0  0.779  0.071  0  0  0  0.045  0  0.048  0  0.525  4.4  1.305  2.763  0.221  1.051  1.186  1.923  1.124  0.322  0.055  0.207  0.346  0.582  0.474  0.929  0.044  0.371  0.11  0.61  0.644  1.18  0.427  0.539  1.402  0.169  0.656  0  0.24  0.581  0  0  0.223  0  0.482  
 
 WDR63  126820  1  85527980  85598821  1p22.3  0  0  0.052  0  0  0.133  0.327  0.073  0.048  0.044  0.171  0.308  0  0.37  0  0.028  0.073  0  0  0  0  0.074  0  0  0.163  0.28  1.308  0.097  0.257  0.181  0.13  1.007  0.201  0.122  0.047  0  0  0.105  0.118  0  0  0.388  0.034  0.057  0.13  0.109  0.221  0  0  0.043  0  0.127  0  0.395  0.323  0.052  0  0.09  0.049  0.254  
 
 SYDE2  84144  1  85623355  85666728  1p22.3  1.442  1.857  0.476  0.443  0.785  0.846  0.774  1.337  0.361  0.324  0.58  0.831  0.355  0.566  0.922  0.392  0.811  0.071  0.111  0.244  0.455  0.436  0.099  0.081  0.512  0.481  0.774  0.969  0.221  0.43  0.375  0.476  0.333  0.276  0.341  1.083  0.542  0.531  0.906  0.665  0.924  0.789  1.512  0.408  0.545  1.132  0.621  1.374  0.685  0.797  0.574  0.562  0.515  0.292  0.558  0.401  0.522  0.455  0.757  0.653  
 
 C1orf52  148423  1  85715636  85725355  1p22.3  1.205  3.892  1.351  1.763  1.139  1.112  1.83  2.078  1.799  1.631  2.108  0.921  1.072  2.302  0.945  1.559  1.747  2.634  2.62  2.258  1.436  1.957  0.636  0.554  1.289  0.962  1.197  1.296  1.149  0.684  1.584  1.279  1.05  0.936  0.843  1.393  0.969  1.308  3.069  1.259  1.233  1.252  2.789  1.447  1.168  1.66  1.385  1.31  0.674  2.364  1.743  0.604  1.832  1.117  1.921  1.097  1.58  1.253  1.733  1.284  
 
 BCL10  8915  1  85731459  85742587  1p22  1.2  4.564  1.564  2.78  1.386  1.5  2.214  2.54  1.847  1.868  2.738  1.247  1.766  2.464  1.061  1.969  2.924  2.396  2.297  1.535  1.436  1.591  1.019  0.529  2.219  1.204  1.446  1.003  1.121  0.713  1.31  1.197  0.983  1.082  0.804  1.461  1.263  2.148  2.974  1.539  1.795  1.041  3.326  1.912  1.094  1.731  1.394  1.678  1.058  2.589  3.353  1.044  2.012  1.568  2.36  1.604  2.589  1.549  2.223  2.717  
 
 LOC646626  646626  1  85742040  85743771  1p22.3  0  1.06  0.194  0  0.158  0  0.28  0.115  0.231  0.374  0.161  0  0  0.337  0  0.228  0.64  0.396  0.17  0.176  0.444  0.252  0  0  0.431  0  0.179  0.086  0  0.044  0.178  0.214  0  0  0  0  0  0.417  0.63  0  0.343  0  0  0  0  0  0.254  0.211  0  0.588  0.152  0.259  0.195  0  0.667  0  0.256  0  0  0.388  
 
 DDAH1  23576  1  85784167  86044046  1p22  1.796  6.913  4.174  3.58  2.78  4.514  1.803  4.199  2.296  3  3.491  3.686  4.28  2.673  3.614  3.714  4.017  1.643  0.115  1.506  0.162  0.871  0.453  1.301  2.625  0.049  0.305  2.067  0.127  0.822  0.843  0.9  1.373  0.824  0.737  3.248  2.545  3.394  4.978  2.079  2.241  1.564  4.772  3.587  2.634  3.773  2.336  3.786  1.653  4.018  3.551  2.118  2.927  1.901  3.119  1.882  3.682  2.399  2.568  3.819  
 
 CYR61  3491  1  86046443  86049648  1p22.3  1.27  8.931  7.704  6.731  2.87  6.586  4.629  7.872  4.517  3.937  4.975  0.146  0.527  2.102  1.784  0.577  1.139  0.777  1.246  1.01  0.621  0.819  0.249  0.372  4.994  0.764  0.666  5.783  0.742  0.339  0.684  1.251  1.73  0.951  2.782  4.663  5.543  5.251  7.549  4.363  3.612  3.302  6.857  5.121  4.586  7.496  5.026  6.857  4.798  8.583  4.369  4.591  5.596  4.456  6.784  4.436  6.942  5.863  6.659  6.6  
 
 ZNHIT6  54680  1  86115105  86174116  1p22.3  0.794  4.157  1.059  1.682  1.245  1.423  1.493  1.612  1.064  1.314  1.942  0.575  1.682  1.774  1.228  0.967  0.975  2.586  2.719  2.171  1.483  1.758  0.822  0.808  1.671  1.047  1.484  1.414  1.384  0.552  1.682  1.43  1.105  0.923  0.993  1.425  1.004  1.758  1.979  1.639  1.426  0.847  2.76  1.578  1.291  1.743  1.631  1.922  0.82  2.844  2.063  0.829  2.016  1.049  1.883  1.533  2.214  1.438  2.008  2.122  
 
 COL24A1  255631  1  86194915  86622121  1p22.3  0  0.083  0.043  0.071  0.017  0.03  0.017  0.522  0.066  0.027  0.238  0.03  0  0.024  0  0.012  0  0.036  0  0.055  0  0.028  0.157  0.025  0  0  0.078  0.319  0  0.076  0.031  0.113  0  0.1  0  0.059  0  0.284  0.023  0.348  0.021  0.154  0.021  0.034  0.045  0.073  0.091  0  0  0.013  0.021  0.11  0.022  0.037  0.023  0.036  0.028  0  0  0  
 
 ODF2L  57489  1  86812506  86862025  1p22.3  0.425  3.832  0.708  1.443  0.622  0.474  0.596  1.574  0.455  1.27  1.618  0.349  0.771  1.495  0.616  1.182  0.573  2.475  1.513  0.667  0.089  1.375  0.292  0.105  1.263  0.642  1.115  0.441  1.108  0.675  1.054  0.747  0.604  0.443  0.444  1.28  0.94  1.468  0.462  1.133  1.258  0.353  1.755  0.678  0.81  0.997  0.408  1.507  0.686  2.499  0.997  0.533  0.782  0.643  1.193  0.462  1.026  0.578  1.027  0.929  
 
 CLCA2  9635  1  86889768  86922240  1p22.3  0  0.025  0.102  0.162  0.039  0  0  0.022  0.034  2.256  0  0  0  0  0  0  0.034  0.04  0  0  0  0.015  0.115  0  0  0  0.05  0  0  0.076  0  0.042  0  0.128  0  0  0  0  0.297  0.039  0.051  0.116  0.019  0  0  0.024  0  0  0  0  0.025  0.157  0  0.038  0  0  0  0  0  0  
 
 CLCA1  1179  1  86934525  86965974  1p22.3  0  0.025  0.073  0  0  0  0  0  0.059  0  0  0  0  0.017  0  0.032  0  0  0.052  0  0  0.057  0  0  0  0  0.033  0  0  0.027  0  0  0  0.113  0  0  0  0  0.028  0  0  0.027  0.026  0  0  0.022  0.022  0  0  0  0  0.192  0  0.024  0  0  0  0  0  0  
 
 CLCA4  22802  1  87012758  87046432  1p31-p22  0  0.033  0.087  0  0  0  0  0  0.052  0  0  0  0  0  0  0.029  0  0.126  0  0  0  0.057  0.106  0  0  0  0.05  0  0  0.035  0  0  0  0.098  0  0  0  0  0.033  0  0  0.027  0.026  0  0  0  0.029  0  0  0  0  0.069  0  0.027  0  0  0  0  0  0  
 
 CLCA3P  9629  1  87099958  87121059  1p31-p22  0  0.236  0.082  0  0  0  0  0  0  0  0  0  0  0.045  0  0  0  0.048  0  0  0  0.021  0  0  0.151  0  0.081  0.032  0  0.044  0  0  0  0.123  0  0  0  0.043  0.022  0  0  0.017  0.016  0  0  0  0.048  0  0  0.068  0  0.051  0  0  0  0  0  0  0  0  
 
 SH3GLB1  51100  1  87170252  87213867  1p22  1.231  5.08  2.085  2.222  1.65  1.703  2.919  3.109  2.493  2.942  2.998  0.962  1.83  2.557  1.335  2.098  1.775  2.43  3.237  1.906  1.316  1.823  1.124  0.736  2.086  1.708  2.457  1.675  1.95  0.732  2.161  1.785  1.686  1.183  1.092  2.691  1.574  2.621  3.268  2.392  1.747  1.296  2.666  1.672  1.064  1.99  1.912  2.144  1.378  3.279  2.646  1.023  2.165  1.849  2.065  1.698  2.972  1.481  2.28  2.33  
 
 SEP15  9403  1  87328127  87380107  1p31  3.562  7.848  3.763  3.751  3.94  3.492  4.997  4.683  4.462  4.914  5.215  3.821  4.473  5.576  3.28  4.874  5.227  6.219  6.384  4.998  3.663  4.482  4.244  3.286  4.501  3.935  3.475  3.401  4.14  2.241  4.655  4.206  3.293  3.008  3.121  4.708  3.687  4.613  5.392  4.413  4.01  3.698  4.317  4.064  2.919  4.239  4.428  3.974  2.598  6.056  4.937  2.809  3.847  3.667  4.534  3.848  4.487  3.748  4.445  4.777  
 
 HS2ST1  9653  1  87380334  87575681  1p22.3  1.27  2.921  1.068  1.867  1.61  1.423  2.225  2.006  2.23  2.514  2.329  0.918  1.956  2.035  1.695  2.463  2.016  2.293  3.167  1.642  1.86  1.83  0.788  0.554  1.829  1.546  1.574  1.454  1.941  1.241  2.251  1.785  1.55  1.357  1.095  2.833  1.453  1.966  1.765  2.706  1.688  1.242  2.48  2.021  0.965  1.452  1.616  1.558  0.986  2.379  2.155  0.654  1.747  1.168  1.508  1.224  1.698  1.008  1.727  1.954  
 
 LINC01140  339524  1  87595447  87634886  1p22.3  0  1.415  0.194  0.23  0.085  0  0.052  0.095  0.565  0.217  0.542  0  0  0.254  0  0.065  0  0.291  0.332  0.132  0  0.14  0.551  0  0.098  0.186  0.154  0.36  0  0.093  0.097  0.284  0  0.284  0.129  0.788  0.08  0.114  0.237  0.192  0  0.54  0.066  0.608  0  0.166  0.077  0.132  0  0.206  0.089  0.728  0.043  0.204  0.141  0.09  0.087  0  0  0  
 
 LOC101927844  101927844  1  87678351  87717014  -  0  0.073  0  0  0  0  0  0  0.148  0  0.077  0  0  0  0  0  0  0  0  0  0  0.057  0.413  0  0  0  0.058  0  0  0.045  0  0  0  0.223  0  0  0  0  0.052  0  0  0.054  0.046  0  0  0  0.05  0  0  0  0  0  0  0  0  0  0  0  0  0  
 
 LMO4  8543  1  87794150  87814607  1p22.3  0.694  3.335  2.662  4.212  3.79  1.616  2.797  2.497  5.173  2.924  3.479  0.562  1.081  1.719  1.403  3.22  2.412  2.273  2.172  2.616  1.256  0.973  1.826  0.977  1.759  1.325  2.245  1.882  1.691  0.682  1.339  2.007  1.408  1.344  1.662  3.585  1.371  2.313  3.279  0.91  1.327  1.829  1.732  3.296  1.911  2.765  1.378  1.778  2.38  3.245  1.913  0.962  2.573  1.044  1.867  3.283  2.058  1.381  1.626  1.234  
 
 LINC01364  100505768  1  87819209  87837338  -  0  0.1  0  0  0.072  0  0  0.152  0  0  0.227  0  0  0.061  0  0  0  0  0  0  0  0  0  0  0  0  0  0  0  0.043  0  0  0  0  0  0.14  0  0  0  0  0  0  0  0  0  0  0  0  0  0  0  0  0.16  0  0  0  0  0  0  0  
 
 PKN2-AS1  101927891  1  89003195  89150887  -  0.074  0.1  0.124  0  0  0.041  0.028  0.021  0.071  0.02  0.049  0.043  0  0.102  0  0.042  0  0.067  0.02  0.041  0  0.045  0.156  0  0.039  0  0.067  0  0  0.054  0  0  0  0.064  0.041  0.13  0  0.065  0.076  0  0.101  0.133  0.039  0  0  0.091  0.051  0  0  0  0.044  0.084  0.02  0.06  0.025  0  0.044  0  0  0  
 
 PKN2  5586  1  89149921  89301938  1p22.2  0.858  3.223  1.163  1.525  1.582  1.4  1.436  1.837  1.712  1.851  2.325  0.726  1.668  2.111  1.818  2.159  2.212  1.527  1.862  1.064  1.088  1.415  0.404  0.496  1.878  1.514  1.228  1.392  1.483  0.712  2.215  1.457  1.238  0.957  1.132  2.321  1.293  1.596  2.302  2.169  2.212  0.998  1.854  2.458  0.89  2.074  1.214  2.12  1.509  2.912  2.369  1.062  1.77  1.065  1.761  1.215  2.38  1.617  2.168  2.256  
 
 GTF2B  2959  1  89318320  89357301  1p22-p21  1.761  5.502  1.398  2.243  2.583  1.778  3.138  2.676  2.084  2.439  3.414  1.919  2.642  4.156  1.749  2.663  2.861  5.107  4.857  3.697  2.629  3.256  2.213  1.387  2.69  2.078  2.257  2.095  2.471  1.003  3.329  2.499  1.873  1.351  1.43  2.534  1.624  2.273  3.497  2.242  2.253  1.577  3.471  2.213  1.519  2.213  2.065  2.555  1.288  4.098  2.473  0.814  1.917  1.857  2.497  1.583  2.558  1.905  2.667  2.641  
 
 CCBL2  56267  1  89401455  89458643  1p22.2  1.491  5.364  0.698  1.319  2.29  1.813  2.343  1.882  2.252  2.305  2.311  0.695  2.546  2.509  2.32  2.034  2.492  3.198  3.943  2.66  1.93  2.151  1.107  0.508  1.35  2.017  1.582  0.652  1.591  0.462  2.202  0.95  1.231  0.894  0.628  1.866  1.321  2.738  2.001  1.987  1.841  1.172  1.828  1.989  1.356  1.948  1.161  1.527  0.922  3.077  1.358  0.802  0.86  0.741  1.477  1.641  1.293  0.73  1.199  1.875  
 
 RBMXL1  494115  1  89445138  89458643  1p22.2  1.931  3.628  1.18  2.055  1.621  1.035  1.578  1.172  1.618  1.627  1.908  1.037  1.692  1.924  1.662  1.758  1.529  1.662  2.169  2.186  1.538  1.98  1.086  0.781  1.535  1.232  1.534  2.005  1.53  1.357  1.529  1.663  1.642  1.53  0.986  1.048  1.337  1.138  1.709  1.32  1.297  1.016  2.234  1.557  1.626  1.413  0.962  1.556  0.693  2.359  1.414  0.605  1.58  0.947  1.494  1.267  1.37  1.476  1.118  1.367  
 
 GBP3  2635  1  89472359  89488549  1p22.2  0  5.106  1.48  1.172  0.098  1.065  2.617  2.707  0.598  2.712  1.703  0.98  1.668  0.387  1.114  2.124  1.648  0.117  1.544  0.504  0  1.358  0.31  0.401  1.285  1.559  1.839  0.168  0.847  0.036  0.65  0.142  0.992  0.546  0.204  1.261  1.351  2.36  4.51  0.087  1.625  0.286  0.019  0.245  0.596  1.696  1.444  0.881  1.034  2.013  2.666  0.072  2.144  1.278  2.417  1.279  1.351  0.077  1.747  1.99  
 
 GBP1  2633  1  89517986  89531043  1p22.2  0  4.678  1.949  4.274  0.219  0.89  3.607  4.758  0.423  3.842  1.81  0.229  0.61  0.038  0  1.218  0.102  0.051  1.597  0.045  0  2.271  0.101  0.406  0.859  0.408  1.665  0.632  0.656  0.181  0.116  1.964  1.489  0.934  0.05  0.192  0.909  0.685  2.974  0.152  0.054  0.494  0.026  0.113  2.412  3.681  0.149  0.704  0.366  1.726  0.412  0.183  2.031  2.703  1.751  0.892  4.464  1.09  0.573  1.778  
 
 GBP2  2634  1  89571815  89591842  1p22.2  0.069  2.432  0.369  2.021  0.108  0.044  2.281  2.032  0.719  2.618  1.724  0.972  0.228  0.069  0  1.038  0.068  0.146  0.103  0.154  0  0.141  0.145  0.052  0.174  0.189  1.402  0.29  0.291  0.038  0.31  0.887  1.104  0.891  0.044  2.151  0.373  1.254  0.423  0  0.278  0.129  0.015  0.061  1.59  1.93  0.857  0.105  0.097  0.573  0.265  0.159  0.291  0.833  0.777  0.433  1.158  0.386  0.106  0.899  
 
 GBP7  388646  1  89597433  89641723  1p22.2  0  0.063  0.114  0.518  0  0  0.101  0.242  0.081  0.078  0  0.056  0  0  0  0.09  0  0.056  0  0  0  0.133  0  0  0  0  0.046  0  0  0.044  0  0.058  0  0.196  0  0.097  0  0  0.026  0  0  0.23  0.026  0  0  0.029  0.055  0  0  0  0.067  0.12  0  0.127  0  0  0.073  0.164  0  0  
 
 GBP4  115361  1  89646830  89664633  1p22.2  0  0.409  0.083  2.136  0.033  0.834  1.481  0.139  0.088  0.091  0.056  0.051  0.103  0.007  0  1.437  0  0  0.609  0  0  1.129  0.251  0.129  0.115  0.023  0.239  0.104  0.156  0.032  0  0.078  0.083  0.125  0  0  0.035  0.144  0.322  0  0  0.082  0.121  0  0.369  0.091  0.025  0  0  0  0.024  0.054  0.016  0.833  0.04  0.069  0.325  0.189  0.027  0.03  
 
 GBP5  115362  1  89724633  89738544  1p22.2  0  0.184  0.128  2.165  0.029  0.063  0.394  1.318  1.011  0.436  2.756  0.03  0  0.06  0.055  0.115  0  0.095  1.198  0.037  0  0.208  0.313  0  0.431  0  0.126  0.134  0.076  0.044  0  0  0  0.268  0  0.091  0  0.096  0.284  0.076  0.117  0.33  0.082  0  1.563  0.8  0.022  0  0.431  0.027  0.1  0.108  0.04  0.204  0.052  0  0.319  0  0  0  
 
 LOC729930  729930  1  89754940  89756045  1p22.2  0  0.077  0  0  0  0  0  0  0.196  0.083  0  0.091  0  0.068  0  0  0  0  0  0  0  0.065  0.224  0  0  0  0.057  0  0  0  0  0  0  0  0.098  0  0  0  0  0  0  0  0  0  0  0  0.212  0  0  0  0  0.139  0.169  0  0.091  0  0  0.124  0  0  
 
 GBP6  163351  1  89829435  89853719  1p22.2  0  0  0.115  0  0  0  0.046  0.054  0.065  0.036  0  0  0.048  0.013  0  0  0  0.028  0.033  0.025  0  0.052  0.137  0  0  0  0.03  0.057  0  0.028  0  0.022  0  0.264  0  0  0  0  0.014  0  0.078  0.237  0.023  0.03  0.04  0.042  0.018  0  0  0  0  0.099  0  0.037  0  0  0  0  0  0  
 
 GBP1P1  400759  1  89873237  89890493  1p22.2  0  0.094  0.38  1.405  0  0  0.498  0.488  0  0.597  0  0  0  0  0  0.145  0  0.188  0.413  0  0  0.448  0  0  0  0  0.123  0.065  0  0.046  0  0  0  0.177  0  0  0  0  0.265  0  0  0  0  0  0.146  0.174  0  0  0  0  0  0  0.259  0.545  0.175  0  0.849  0  0  0  
 
 LRRC8B  23507  1  89990396  90063420  1p22.2  1.5  0.819  0.306  1.02  1.511  0.267  0.516  0.404  0.29  1.354  1.519  0.657  2.228  1.8  1.42  1.677  1.706  1.768  1.473  1.487  1.523  1.191  0.561  0.422  0.852  1.49  0.445  0.419  0.566  0.615  1.35  0.631  0.063  0.141  0.681  1.236  0.429  0.379  0.637  1.479  1.553  0.495  2.724  1.487  1.162  1.52  1.17  0.837  0.39  1.225  1.456  0.792  0.807  0.408  1.306  0.301  0.8  0.768  1.918  1.012  
 
 FLJ27354  400761  1  90090407  90098453  1p22.2  0  0  0  0  0  0.655  0.4  0  0.209  0.322  0.161  0  0  0.132  0  0.269  0  0  0.592  0.306  0  0.309  0  0  0.504  0.614  0.103  0.084  0.422  0.341  1.188  0.382  0  0  0  0.333  0  0  0.133  0.463  0  0.106  1.113  0  0  0  0.067  0  0  0.257  0  0.46  0  0.098  0.323  0  0  0.478  0  0  
 
 LRRC8C  84230  1  90098643  90185094  1p22.2  0.08  2.319  0.523  0.776  0.064  0.826  1.626  0.99  0.184  0.627  1.257  0.052  0.155  1.513  0.898  0.335  0.098  0.639  1.843  1.715  0.812  0.765  0.403  0.382  1.492  1.498  0.708  0.378  0.903  0.178  1.086  0.696  0  0.161  0.526  0.509  0.868  0.42  1.234  1.073  0.412  0.312  1.297  2.081  0.312  0.319  0.677  0.546  1.008  0.665  1.641  0.647  0.607  0.47  0.644  0.093  0.896  0.565  0.839  1.024  
 
 LRRC8D  55144  1  90286572  90401989  1p22.2  1.244  3.58  1.436  2.795  1.26  1.628  2.536  1.794  3.952  1.938  3.391  1.602  2.272  1.228  1.458  1.362  1.664  1.392  2.71  2.446  1.445  1.848  1.813  1.246  1.603  2.086  1.881  1.61  1.901  1.507  2.325  1.806  2.229  1.589  1.772  2.205  1.409  0.935  3.538  1.533  2.204  1.813  3.488  2.809  1.785  2.281  0.698  1.897  2.267  2.883  2.379  0.981  1.852  2.06  1.793  1.641  2.215  1.157  2.669  1.99  
 
 GEMIN8P4  492303  1  90458823  90460525  1p22  0.431  0.399  0.578  0.488  0.463  0.103  0.622  0.37  0.672  0.509  1.095  0.373  0.524  0.719  0.311  0.563  0.658  0.336  1.457  0.598  0.798  0.721  0.552  0.284  0.275  0.314  0.818  0.48  0.894  0.653  1.453  0.704  0.826  0.56  0.763  0.528  0.225  0.566  0.407  0.651  0.753  0.596  2.816  0.83  0.223  0.878  0.362  0.579  0.203  1.59  0.382  0.373  0.33  0.491  0.567  0.301  0.496  0.216  0.814  0.193  
 
 ZNF326  284695  1  90460677  90494094  1p22.2  1.334  2.96  1.261  1.018  1.593  2.196  1.748  2.044  1.149  2.173  2.529  1.627  2.307  2.143  1.986  2.009  2.072  2.523  3.036  2.084  1.745  2.385  0.521  0.873  2.136  1.554  1.507  1.771  1.783  0.94  2.524  1.874  1.88  0.859  1.642  2.001  1.331  1.78  2.357  2.462  2.015  1.915  2.522  2.93  1.597  1.752  1.064  2.233  2.183  2.693  1.905  1.041  2.293  1.597  1.538  1.357  2.141  1.405  2.202  2.452  
 
 BARHL2  343472  1  91177578  91182794  1p22.2  0  0.133  0.269  0  0.044  0  0  0.062  0.249  0.107  0.104  0.113  0  0.066  0  0.068  0  0.086  0  0.11  0  0.296  0.309  0  0  0  0.314  0.072  0.183  0.582  0.131  0  0  0.309  0  0  0  0  0.126  0  0  0.449  0.042  0  0.163  0.115  0.036  1.698  0  1.31  0.063  0.689  0.059  0.143  0.071  0  0.167  0  0  0  
 
 ZNF644  84146  1  91380856  91487812  1p22.2  1.588  4.165  1.352  1.909  1.861  1.389  2.19  2.972  2.196  2.532  3.031  1.1  1.933  2.949  2.219  2.261  2.498  3.301  3.392  2.657  1.601  2.458  0.96  1.08  2.117  1.828  1.844  2.062  2.055  1.482  2.607  1.792  1.911  1.213  1.091  2.343  1.486  1.881  2.663  2.45  2.159  1.296  3.02  2.029  1.655  2.038  1.844  2.28  1.259  3.022  2.296  1.089  1.823  1.212  1.873  1.388  4.06  1.344  2.516  2.306  
 
 HFM1  164045  1  91726322  91870426  1p22.2  0  0.083  0.031  0  0.014  0.059  0  0.047  0  0.035  0.034  0.087  0  0.057  0  0.019  0.034  0.346  0.014  0.028  0.182  0.035  0  0  0.022  0.037  0.068  0.061  0.089  0.049  0.058  0.062  0.091  0.072  0.048  0  0.041  0.035  0.039  0  0  0.227  0.073  0.129  0.031  0.028  0.017  0  0.043  0.132  0.025  0.132  0.027  0.124  0  0.051  0  0  0  0.037  
 
 CDC7  8317  1  91966403  91991321  1p22  1.619  3.484  0.357  1.224  1.059  1.091  1.376  1.41  0.34  1.66  1.514  0.897  1.787  2.525  1.784  1.598  1.541  3.197  3.113  1.691  2.072  1.653  0.527  0.438  1.233  1.318  0.938  1.053  1.124  0.78  1.926  1.46  1.622  0.884  0.954  1.607  1.07  1.61  1.213  2.023  1.739  1.198  2.339  2.314  0.864  1.832  1.261  1.883  0.906  3.369  1.535  0.903  1.579  0.828  1.032  0.82  1.388  1.069  1.501  1.315  
 
 TGFBR3  7049  1  92145899  92371559  1p33-p32  0.205  0.619  0.285  1.031  0.199  0.203  0.388  0.204  0.239  1.159  0.245  1.921  0.331  0.728  1.78  1.228  1.434  0.715  0.056  0.072  1.647  0.081  0.508  0.5  0.49  1.79  1.805  1.384  2.141  0.845  1.986  1.123  1.831  0.877  0.525  0.946  0.331  0.977  0.593  2.058  0.852  0.368  0.416  2.623  0.588  1.034  2.518  1.218  0.475  1.111  0.697  0.37  0.134  0.092  0.085  0.13  0.499  0.646  0.697  0.208  
 
 BRDT  676  1  92414927  92479985  1p22.1  0  0  0  0  0  0  0  0  0  0.033  0  0  0  0.029  0  0  0  0.094  0  0  0  0.078  0  0  0  0  0.057  0  0  0.051  0  0  0  0.113  0  0  0  0  0.023  0  0  0.046  0.032  0  0  0  0.053  0  0  0  0  0.908  0  0.025  0  0  0  0  0  0  
 
 EPHX4  253152  1  92495532  92529093  1p22.1  0.161  3.887  0.093  0  0  0.285  1.406  0.429  1.937  0.038  1.529  0.993  0.636  1.414  0.801  0.305  1.122  3.908  0.617  0.127  0  0.579  0  0  0.1  0  0.054  0  0  0.124  0.212  0  0  0  0.102  0  0.569  1.061  0.037  0.38  1.344  0.442  0.04  0.576  0.876  1.059  0.317  0.614  0.303  1.441  0.303  0.959  0  0.088  0.167  0.3  0  0.229  0  1.355  
 
 SETP18  646817  1  92539992  92541029  1p22.1  1.631  0.663  1.256  2.284  0.94  0.894  0.969  0.857  1.73  1.172  1.171  1.359  2.12  1.966  2.449  1.517  1.897  0.379  1.215  1.411  0.745  1.672  1.38  1.54  1.42  0.682  0.699  1.331  0.841  0.419  0.923  0.991  0.87  1.803  1.159  0.482  1.481  0.633  1.367  0.851  0.866  1.483  1.209  1.124  0.84  0.578  0  0.734  0.785  0.983  1.321  0.901  1.698  1.385  1.333  1.379  0.767  1.649  1.301  0.586  
 
 BTBD8  284697  1  92545861  92613401  1p22.1  0  0.318  0  0  0.078  0  0.059  0.131  0  0.15  0.204  0.132  0.25  0.183  0.329  0.185  0.205  0  0  0.092  0  0.18  0.169  0  0.121  0.061  0.224  0.131  0  0.044  0.254  0  0  0  0.09  0  0.09  0.165  0.035  0  0.135  0.349  0.033  0  0  0  0.04  0  0  0.067  0.079  0.399  0.11  0.111  0.146  0  0  0.11  0.122  0.229  
 
 KIAA1107  23285  1  92632608  92650280  1p22.1  0.247  1.018  0.154  0  0.339  0  0.178  0.231  0.311  0.146  0.241  0.185  0.493  0.45  0.486  0.271  0.591  0.692  0.112  0.06  0.067  0.165  0.222  0.062  0.243  0.074  0.039  0.192  0.089  0.067  0.231  0.075  0  0.137  0.138  0.121  0.218  0.258  0.102  0.224  0.312  0.383  0.138  0.13  0.168  0.045  0.026  0.184  0.062  0.192  0.209  0.31  0.075  0.139  0.376  0.147  0.029  0.033  0.121  0.489  
 
 C1orf146  388649  1  92683572  92711367  1p22.1  0  0  0  0  0  0  0  0  0  0.157  0.151  0  0  0  0.26  0.112  0  0  0  0.132  0  0.13  0  0  0  0  0.134  0  0  0  0.231  0  0  0  0  0  0  0  0.335  0  0  0.761  0  0  0  0  0  0  0  0  0  0  0.203  0  0  0  0  0.3  0.187  0  
 
 GLMN  11146  1  92711954  92764566  1p22.1  0.594  3.273  0.376  0.703  0.669  0.595  0.804  1.191  0.441  1.284  1.699  0.347  1.094  2.211  0.654  0.988  1.37  2.052  2.958  1.424  1.205  0.913  0.349  0.242  1.068  0.885  0.561  0.605  0.743  0.236  1.502  0.941  0.746  0.262  0.301  1.414  0.518  1.062  1.181  1.672  1.155  0.644  1.768  1.392  0.409  1.534  0.021  1.052  0.659  2.865  1.235  0.29  0.548  0.422  0.502  0.414  1.257  0.561  1.219  1.285  
 
 RPAP2  79871  1  92764521  92853732  1p22.1  0.717  2.731  0.892  0.946  0.933  0.767  1.514  1.16  1.104  1.425  1.83  0.534  1.19  2.052  1.365  0.906  1.379  1.624  2.368  1.408  1.266  1.292  0.857  0.48  1.064  1.197  1.213  0.738  1.178  0.312  1.89  0.95  0.671  0.618  0.627  1.388  0.797  1.329  1.358  1.556  1.117  0.86  1.858  1.523  0.952  1.314  0.907  0.998  0.923  2.073  1.589  0.519  1.047  0.604  0.897  0.883  1.586  0.742  1.35  1.452  
 
 GFI1  2672  1  92940317  92952433  1p22  0.228  0.062  0.101  0.12  0  0  0.463  0.989  0.201  0.059  0.149  0.098  0.115  0.936  1.083  0.072  0.113  0.235  3.866  4.075  0.085  2.846  0.881  0.699  0.214  0  0.089  0.051  0.102  0.103  0.054  0.05  0  0.198  0.05  0.07  0.125  0.069  0.173  0.272  0.071  0.178  0.32  0  0  0.123  0.061  0.658  0  1.482  0.062  0.605  0.035  0.067  0.051  0.11  0  0  1.101  0  
 
 EVI5  7813  1  92974252  93257961  1p22.1  0.574  2.611  0.969  0.455  0.678  1.018  1.165  1.571  1.256  1.673  1.686  0.395  0.962  1.268  1.047  0.53  0.905  1.21  0.267  0.912  0.858  0.538  0.368  0.374  1.236  1.672  1.372  1.629  1.162  0.824  1.371  1.197  1.031  0.73  0.667  1.474  1.074  1.876  1.787  1.245  0.696  0.56  1.699  1.153  0.388  0.908  0.486  1.158  0.977  1.403  1.917  0.5  0.904  0.533  0.757  0.921  1.135  0.635  1.011  1.547  
 
 RPL5  6125  1  93297593  93307481  1p22.1  8.429  11.051  6.797  6.751  8.347  7.362  9.299  8.384  7.291  8.215  9.108  7.482  7.758  10.144  7.856  8.023  9.097  10.739  10.581  10.208  8.493  8.786  7.688  7.473  8.676  6.885  8.429  7.668  7.499  7.746  7.803  8.142  7.84  6.025  7.044  7.95  7.873  8.512  8.875  8.331  7.72  7.153  9.696  8.814  7.611  8.213  8.249  8.695  6.807  10.54  8.268  6.47  8.198  6.248  8.408  7.57  8.295  8.291  8.531  8.635  
 
 FAM69A  388650  1  93298285  93427079  1p22.1  0.979  4.497  2.156  0.475  0.693  0.575  1.94  2.819  3.028  3.237  2.388  0.992  3.205  2.044  0.695  0.615  1.103  2.565  2.513  0.284  0.351  1.208  0.89  0.833  1.1  1.718  0.809  1.345  1.384  1.104  1.945  1.141  1.35  1.238  1.025  1.125  0.884  1.456  1.187  1.052  0.712  1.762  2.515  0.957  0.321  1.907  0.167  1.342  0.897  2.012  1.365  0.494  1.16  1.199  0.807  0.277  1.634  0.889  1.03  1.403  
 
 SNORA66  26782  1  93306275  93306408  1p22.1  0  3.317  0  0  2.128  0  0  0  0  2.576  2.715  0  0  2.747  0  0  0  0  3.625  0  0  2.485  0  0  0  0  1.423  2.013  0  1.357  0  0  0  0  0  0  0  0  0  0  0  0  0  0  0  3.263  0  0  0  3.015  0  0  0  1.928  0  0  0  0  3.44  0  
 
 MTF2  22823  1  93544791  93604638  1p22.1  1.309  4.422  1.099  1.471  1.664  1.007  1.91  2.404  1.368  1.675  2.65  1.075  2.175  3.253  1.634  2.008  2.188  3.313  4.725  2.822  2.434  3.49  0.979  0.859  1.881  1.42  1.531  1.793  1.227  0.969  2.339  1.643  1.21  0.678  0.86  1.796  1.573  1.767  2.327  2.442  1.53  0.966  3.112  2.696  2.121  2.126  1.959  2.01  1.234  3.269  1.823  0.873  1.826  0.921  1.91  1.277  2.333  1.215  2.314  2.062  
 
 TMED5  50999  1  93615298  93646246  1pter-q31.3  1.344  4.488  1.539  2.664  1.914  1.679  2.47  2.333  2.167  3.167  3.003  1.685  2.64  2.771  1.702  2.409  2.545  2.602  3.244  2.417  2.153  1.874  1.798  1.458  2.729  2.014  1.706  1.725  1.801  0.83  2.587  1.916  1.595  1.192  1.201  2.543  1.707  2.696  2.897  2.227  1.889  1.463  2.977  2.526  2.285  2.18  1.599  2.364  0.922  3.46  2.577  1.151  2.1  2.011  2.548  1.633  2.907  1.282  2.77  2.789  
 
 CCDC18  343099  1  93646272  93744287  1p22.1  0.449  3.036  0.296  0.771  0.467  0.342  0.795  0.976  0.245  1.332  1.501  0.3  0.688  1.634  0.766  0.827  0.962  2.724  2.454  0.683  0.851  0.788  0.446  0.324  0.745  0.693  0.431  0.43  0.688  0.071  1.09  0.623  0.466  0.221  0.53  0.941  0.568  1.076  0.539  1.175  0.625  0.557  0.586  0.573  0.767  1.05  0.527  1.086  0.577  1.462  1.093  0.432  1.148  0.395  0.86  0.542  1.801  0.234  1.218  1.128  
 
 CCDC18-AS1  100131564  1  93775665  93811368  1p22.1  0.97  1.642  0.326  0.21  1.185  0.123  0.379  0.544  0.539  0.815  0.604  0.077  0.665  1.275  0.561  0.481  0.409  1.129  0.87  0.436  0.815  0.652  1.136  0.8  0.178  0.661  0.54  0.395  0.43  0.276  1.118  0.497  0.367  0.283  0.458  0.854  0.61  1.505  0.574  0.629  0.751  0.343  1.033  0.46  0.505  0.611  0.816  0.193  0.42  0.332  0.51  0.496  0.483  0.179  1.014  0.246  1.003  0.2  0.623  0.402  
 
 DR1  1810  1  93811477  93828148  1p22.1  1.53  4.775  2.623  2.991  2.421  2.341  2.81  2.735  2.69  2.833  3.339  1.241  2.377  2.972  2.175  2.382  2.673  2.913  4.149  2.476  2.2  2.708  1.975  1.928  2.709  1.992  1.631  2.256  1.782  0.807  2.609  2.056  2.126  1.725  1.308  2.676  2.168  2.41  3.312  2.574  1.945  2.248  2.628  2.341  3.078  2.546  1.568  2.979  1.906  3.763  3.435  1.676  2.312  1.873  2.193  1.711  2.944  2.021  2.576  2.565  
 
 FNBP1L  54874  1  93913687  94020218  1p22.1  1.76  3.725  1.695  1.364  2.198  1.492  0.924  2.356  2.049  1.034  2.946  1.65  2.504  2.653  2.397  2.092  3.006  4.634  1.537  0.105  0.383  1.188  0.451  0.308  1.58  1.359  1.24  1.851  2.105  1.861  2.751  1.599  2.024  1.057  1.218  2.838  1.058  1.75  1.598  2.051  2.015  0.902  3.432  2.7  3.057  2.433  1.591  1.609  1.42  2.495  2.49  1.052  2.241  1.341  2.151  1.727  2.405  1.29  2.648  2.744  
 
 BCAR3  8412  1  94027297  94312706  1p22.1  1.042  5.001  2.991  3.622  2.734  2.508  3.016  3.917  3.028  2.696  2.954  1.057  1.093  2.266  1.035  2.398  1.212  1.884  0.316  0.134  0.492  0.323  0.256  0.067  3.105  2.016  2.266  2.469  2.506  0.907  2.682  1.585  2.147  1.369  1.489  2.686  3.015  3.045  3.981  1.852  2.295  2.356  1.175  1.648  1.74  2.38  2.06  3.447  2.297  4.711  4.232  2.479  2.942  2.271  3.944  2.824  3.495  3.174  3.899  3.91  
 
 LOC100129046  100129046  1  94057524  94065587  1p22.1  0.191  0.348  0.399  0.525  0.392  0.294  0.354  0.824  0.278  0.365  0.424  1.134  1.471  0.235  0.831  0.863  0.677  0.959  0  0  0  0.082  0.97  0.506  0.221  0.206  0.438  0.204  0.368  0.233  0  0.133  0.324  0.292  0.135  0.195  0.473  0.653  0.428  0.129  0.206  0.165  0.062  0.319  0.863  0.36  0.053  0.7  0.311  0.286  0.812  0.486  0.382  0.137  0.898  0.426  1.048  0.516  0.686  0.383  
 
 DNTTIP2  30836  1  94335013  94344762  1p22.1  2.554  6.921  2.035  3.291  2.933  1.945  3.286  3.323  2.709  2.964  3.831  1.791  3.648  4.092  2.895  3.171  1.995  5.09  4.483  2.902  3.198  3.099  2.089  1.949  3.203  2.772  2.381  2.904  2.465  1.657  3.257  2.68  2.911  2.312  2.283  3.283  2.634  3.03  3.399  3.652  3.098  2.532  3.175  2.371  2.801  3.256  2.804  2.932  1.954  3.994  3.576  2.031  3.232  2.528  2.992  2.23  3.721  2.162  3.901  3.333  
 
 GCLM  2730  1  94350755  94375154  1p22.1  1.021  4.236  1.624  2.029  0.885  1.182  3.27  2.488  2.182  3.205  3.518  0.932  3.117  2.341  0.988  1.644  1.353  2.014  2.999  1.576  1.958  1.972  0.94  0.527  1.186  1.023  1.472  1.281  1.07  1.051  2.161  1.256  1.33  0.741  2.696  2.608  1.81  1.918  4.305  2.332  2.62  3.265  1.78  2.556  0.964  1.875  0.372  1.766  1.539  2.423  2.263  1.729  2.219  2.867  2.008  1.105  3.15  1.742  2.264  2.319  
 
 ABCA4  24  1  94458393  94586705  1p22  0.05  0.036  0.18  0.057  0.1  0.1  0.047  0.046  0.065  0.068  0.043  0.038  0  0.041  0  0.032  0.019  0.04  0.118  0.041  0  0.254  0.139  0.02  0.015  0  0.064  0.042  0.043  0.054  0.106  0.017  0.017  0.107  0.032  0  0.11  0.023  0.188  0  0.115  1.122  0.038  0.486  0.368  0.336  0.028  0.047  0.038  0.122  0.071  0.296  0.025  0.068  0.018  0.077  0.054  0  0.023  0  
 
 ARHGAP29  9411  1  94634462  94703307  1p22.1  0.338  5.473  2.044  1.381  1.348  1.6  1.726  0.669  1.186  1.54  3.117  0.016  0.053  3.77  0.793  2.486  0.927  0.462  1.591  0.072  0.065  0.599  0.117  0  0.027  0.036  0.113  1.059  0.587  0.186  1.362  0.37  0.258  0.2  1.084  1.398  2.69  2.223  3.728  0.438  1.417  1.113  0.045  2.524  3.511  3.652  1.866  1.92  3.831  3.464  4.544  1.984  2.614  1.823  3.42  3.546  3.403  1.474  3.385  3.603  
 
 ABCD3  5825  1  94883932  94984219  1p21.3  1.737  3.886  1.77  1.436  1.961  2.233  3.154  2.385  2.817  2.948  3.566  2.496  2.27  3.238  2.204  2.667  2.427  3.04  4.618  2.539  1.829  2.716  0.94  0.665  1.824  1.976  2.17  1.974  2.019  1.138  2.78  1.737  1.943  1.177  1.952  3.313  1.863  2.32  2.402  2.368  2.059  2.006  2.963  2.732  1.645  2.673  1.705  2.092  2.717  3.313  2.869  1.246  2.336  1.471  1.732  1.506  2.726  1.333  2.396  2.619  
 
 F3  2152  1  94994731  95007413  1p22-p21  0  7.629  3.475  0.191  0.401  3.8  4.95  1.835  1.208  2.139  3.533  1.545  1.619  2.359  1.498  0.455  5.269  0.969  0.265  0.459  0.212  0.428  0.175  0  0.562  0.088  0.251  0.463  0.414  0.114  0.317  0.277  0.196  0.256  0.346  2.215  3.975  3.938  5.719  0.413  4.918  1.742  0.229  4.057  0.699  2.676  1.978  5.545  5.737  5.09  1.076  1.441  1.233  1.225  0.408  0.636  5.172  1.218  2.108  0.828  
 
 LINC01057  101928079  1  95123088  95285837  1p21.3  0  0.11  0.274  0  0.238  0.116  0.125  0.459  0.285  0.171  0.243  0.363  0.533  0.531  0.365  0.199  1.608  0  0  0  0  0.139  0.235  0  0  0.325  0.222  0.537  0.788  0.287  0.65  0.087  0  0.302  0.101  1.169  0.348  0.292  0.409  0.18  0.598  0.079  0.551  0.927  0  0.802  0.287  0.412  0.832  0.812  0.387  0.369  0  0.149  0.326  0.103  0.417  0.213  0.484  0.223  
 
 SLC44A3  126969  1  95285897  95360803  1p21.3  0.226  0.206  0.157  0  1.03  0.085  0.186  0.703  0.159  0.438  0.314  1.678  1.836  1.36  1.293  1.125  2.797  3.041  0  0.06  0  0.08  0.363  0.077  0  0.815  0.256  0.408  0.872  0.556  0.631  0.128  0  0.196  0.267  2.069  0.205  0.23  0.397  0.531  1.58  0.114  0.36  2.215  0.168  1.737  0.975  0  1.405  0.273  0.11  0.344  0.256  0.338  0.746  0.171  1.224  0.448  0.833  0.69  
 
 CNN3  1266  1  95362504  95392779  1p22-p21  0.121  8.874  5.324  5.254  5.299  6.144  6.096  6.58  5.972  6.293  7.207  1.15  0.972  5.742  5.246  0.336  4.249  6.362  3.86  0.389  0.292  1.621  1.078  1.379  4.579  4.706  5.581  4.839  4.564  1.315  5.09  4.606  4.504  2.99  2.969  5.266  6.022  5.836  6.388  2.699  4.127  4.647  5.724  5.093  5.213  5.731  3.107  5.461  4.885  6.656  3.374  3.42  5.71  4.844  5.307  4.219  6.514  4.808  6.159  6.487  
 
 LOC729970  729970  1  95393583  95428826  1p21.3  0  1.937  0.565  1.29  0.354  0.916  2.032  1.8  1.801  0.813  2.119  2.446  0  0.101  0.694  0.107  0  2.839  0.285  0.634  0  0.229  0.263  0.361  0.294  0.373  1.064  0.398  0.29  2.248  0.796  0.843  0.159  0.227  0.402  1.062  1.153  0.803  1.396  0.222  0.697  1.508  0.757  0.407  0.827  0.876  3.643  0.734  0.346  1.487  0  0.606  0.708  0.561  0.879  0.498  1.089  1.443  1.239  1.814  
 
 ALG14  199857  1  95448278  95538507  1p21.3  1.803  5.066  1.6  1.329  2.504  1.279  3.248  2.576  1.905  2.434  3.065  2.312  1.996  2.655  1.502  1.727  1.53  5.034  3.752  2.2  1.893  2.257  1.22  1.404  1.381  2.206  2.319  1.648  1.847  1.972  2.604  2.037  1.559  1.051  1.458  2.056  1.289  1.926  3.209  1.002  1.838  1.977  2.59  1.314  0.959  1.723  1.785  1.969  1.794  3.392  2.236  0.904  2.267  1.61  2.253  1.158  2.451  1.768  1.533  1.705  
 
 TMEM56  148534  1  95558072  95663161  1p21.3  0.375  2.108  0.253  1.383  1.154  0.337  0.358  1.403  0.539  0.611  1.493  0.066  0.315  1.357  0.967  0.343  1.102  1.445  0.018  0.032  1.196  0.192  0.349  0.1  0.024  0.667  0.205  0.721  0.385  0.607  0.694  0.279  0.852  0.685  0.905  1.625  0.315  0.531  0.866  0.25  0.621  0.416  0.644  0.43  0.159  0.454  0.516  0.58  2.828  1.143  1.339  0.822  1.097  1.101  2.225  1.444  2.22  0.42  2.096  1.156  
 
 TMEM56-RWDD3  100527978  1  95583478  95710509  1p  0.23  0.952  0  0.265  0.3  0  0.24  0.603  0.234  0.232  0.702  0  0.344  0.461  0.407  0.101  0.307  0.471  0.172  0  0.604  0.199  0  0  0.12  0.304  0.25  0.242  0.21  0.057  0.309  0  0  0.249  0.37  0.714  0.157  0.418  0.219  0.332  0.322  0.056  0  0  0  0.111  0  0.299  0.98  0.249  0.451  0.226  0.391  0.373  0.413  0.186  0.94  0  0.781  0  
 
 LOC101928118  101928118  1  95628774  95699538  1p21.3  0  0.129  0.125  0.153  0.187  0.049  0.041  0.247  0.104  0.274  0.198  0  0  0.13  0.144  0.044  0.064  0.045  0  0  0  0.053  0.198  0  0  0.121  0.091  0.104  0.05  0.186  0.254  0.045  0.288  0.279  0.081  0.183  0  0  0.028  0  0.139  0.079  0.019  0.052  0  0.127  0.03  0.13  0.492  0.038  0.136  0.188  0.17  0.043  0.339  0  0.554  0  0.251  0  
 
 RWDD3  25950  1  95699710  95712781  1p21.3  1.049  4.285  0.981  1.668  1.599  1.103  1.352  1.572  1.403  1.785  2.469  0.584  0.955  2.245  0.875  1.392  1.604  3.917  3.274  0.9  1.424  2.033  0.579  0.439  1.444  1.125  1.189  0.746  1.078  0.621  2.018  1.284  1.321  1.201  0.945  1.78  0.635  1.693  2.528  1.056  1.096  1.795  1.87  1.336  0.781  1.318  0.073  1.087  1.539  2.905  1.792  0.624  1.295  1.103  2.073  1.424  2.033  0.678  2.143  1.323  
 
 FLJ31662  440594  1  95940292  95944912  1p21.3  0  0  0.061  0  0  0  0  0  0.067  0.02  0  0  0  0  0  0  0  0  0  0  0  0.028  0.258  0  0  0  0.038  0.024  0  0.036  0  0  0  0.197  0  0  0  0  0  0  0  0.13  0  0  0  0  0  0  0  0  0  0.071  0  0  0  0  0  0  0  0  
 
 LOC100996635  100996635  1  95975671  95981020  -  0  0  0  0  0  0  0  0  0  0  0  0  0  0.029  0  0  0  0  0  0  0  0  0  0  0  0  0.042  0  0  0.021  0  0  0  0.197  0  0  0  0  0  0  0  0  0  0  0  0  0  0  0  0  0  0  0  0  0  0  0  0  0  0  
 
 LOC102723661  102723661  1  96457623  96488436  1p21.3  0  0  0  0  0  0  0  0  0  0  0  0  0  0  0  0  0  0.145  0  0  0  0.067  0  0  0  0  0  0  0  0  0  0  0  0  0  0  0  0  0  0  0  0  0  0  0  0  0  0  0  0  0  0  0  0  0  0  0  0  0  0  
 
 LOC101928241  101928241  1  96719624  96839681  -  0  0.063  0  0  0  0  0  0  0  0.043  0  0  0  0.04  0  0  0  0  0  0.067  0  0.046  0.271  0  0  0  0.052  0  0  0.052  0  0  0  0.217  0  0  0  0  0.038  0  0  0.047  0.073  0  0  0  0.024  0  0  0  0  0.101  0  0  0  0  0  0  0  0  
 
 PTBP2  58155  1  97187160  97280605  1p21.3  0.871  2.483  0.733  0.71  0.583  0.713  0.424  1.244  0.693  1.558  1.687  0.363  0.764  1.962  1.184  1.174  0.991  2.758  2.821  0.86  0.9  1.517  0.253  0.205  1.012  0.917  0.578  0.842  0.807  0.332  1.381  0.841  0.733  0.332  0.716  0.92  0.802  1.31  1.196  1.077  0.77  0.774  1.693  1.254  0.657  1.266  0.599  1.576  0.877  2.012  1.567  0.699  1.249  0.337  1.017  0.787  2.02  1.023  1.337  1.232  
 
 DPYD  1806  1  97543299  98386615  1p22  0  4.881  1.175  0.771  0.031  2.044  2.03  1.821  2.219  3.124  2.408  0  0  0.33  0.086  0.557  0.23  0.159  3.115  0.495  0  1.758  0.907  0.656  1.963  0.049  1.004  1.114  0.078  0.093  0  1.113  1.724  0.954  0.163  1.689  1.029  1.597  1.712  0.133  1.42  0.649  3.172  2.72  0.143  0.139  0.703  0.645  3.407  2.305  1.667  0.49  0.695  0.627  1.246  0.94  2.026  0.677  0.738  2.011  
 
 DPYD-AS1  100873932  1  97561478  97788511  -  0  0  0.31  0  0  0  0  0.184  0.4  0.438  0  0  0  0  0  0  0  0  0.178  0  0  0.144  0  0  0  0  0  0  0  0  0  0  0  0  0  0  0  0  0.254  0  0  0  0  0  0  0  0  0  0  0  0  0  0  0  0  0  0  0  0  0  
 
 DPYD-AS2  100873933  1  98262476  98263607  -  0  1.039  0.173  0  0  0  0  0  0  0.475  0.13  0  0  0.067  0  0  0  0  0  0  0  0.32  0  0  0  0  0.507  0  0  0  0  0  0.228  0  0  0  0  0  0.171  0  0  0  0  0  0  0  0  0  0.315  0  0  0  0  0  0.097  0.207  0  0  0  0  
 
 MIR137HG  400765  1  98453555  98515249  1p21.3  0  0.583  0.139  0  0  0.535  0  0.154  0.16  0.079  0  0  0  0.017  0  0  0  0.155  0  0  0  0.045  0  0  0.131  0  0.045  0  0  0.045  0  0  0  0.124  0  0.109  0.055  0  0.12  0  0.196  0.063  0.92  0.053  0  0.033  0  0  0.199  0.223  0.464  0.187  0  0.033  0.037  0.105  0  0.114  0  0.098  
 
 MIR2682  100616452  1  98510798  98510907  -  0  0  0  0  0  0  0  0  0  0  0  0  0  0  0  0  0  0  0  0  0  0  0  0  0  0  0  0  0  0  0  0  0  0  0  0  0  0  0  0  0  0  5.443  0  0  0  0  0  0  0  0  0  0  0  0  0  0  0  0  0  
 
 LOC729987  729987  1  98676266  98738214  1p21.3  0  0.354  0.103  0  0  0.202  0.046  0.148  0.296  0.06  0.156  0  0  0  0  0  0  0  0  0  0  0.026  0.106  0  0.169  0.073  0.026  0.28  0  0.024  0  0.068  0  0.128  0.046  0.199  0.132  0.664  0.171  0  0  0  0.735  0.072  0  0.093  0  0.218  0  0.176  0.102  0  0.194  0.096  0.561  0.092  0.205  0.205  0.086  0.681  
 
 SNX7  51375  1  99127235  99226056  1p21.3  0.418  6.477  2.586  3.003  0  1.786  3.405  3.388  3.192  3.074  4.577  1.41  2.126  3.432  1.696  2.445  2.426  4.342  0.645  0  0  0.417  0  0.074  2.436  1.759  2.723  1.511  1.711  1.171  2.191  1.692  2.288  1.697  1.307  3.094  1.697  3.276  2.693  1.83  2.112  2.611  3.023  3.05  0.959  2.214  1.631  2.503  3.05  4.13  3.808  1.521  2.698  2.24  2.932  2.959  3.649  2.616  3.078  3.378  
 
 PLPPR5  163404  1  99355800  99470449  1p21.3  0  0  0.092  0  0  0  0  0.031  0.265  0.042  0.105  0.092  0  0.015  0  0.025  0.029  0.05  0  0.03  0  0.086  0.099  0  0  0.034  0.05  0.019  0  0.048  0  0.059  0  0.144  0  0  0  0  0.022  0  0  0.103  0.062  0  0.052  0.051  0.021  0.046  0  0.078  2.49  0.352  0.053  0.053  0.209  0.034  0.041  0  0  0  
 
 LOC100129620  100129620  1  99469831  99614408  1p21.3  0  0  0.081  0  0  0  0  0  0  0  0  0  0  0  0  0  0  0.075  0  0  0  0.064  0.102  0  0  0  0.054  0  0  0.053  0  0  0  0.202  0  0  0  0  0.053  0  0  0.313  0.191  0  0  0  0  0  0  0  0.045  0.164  0.024  0.082  0.027  0  0  0  0  0  
 
 PLPPR4  9890  1  99729847  99775138  1p21.2  0  0  0.603  0  0.02  0.528  0.374  0.076  1.609  0.068  1.899  0  0  0.207  0  0  0  0.028  0.022  0  0  0.04  0.194  0  0  0  0.661  0  0  0.057  0  0  0  0.141  0.042  0  0  0  0.029  0.302  0  0.254  0.028  0  0.05  0.07  0.036  0.03  0  0.028  0.053  0.158  0  0.013  0.03  0  0.022  0  0  0  
 
 PALMD  54873  1  100111430  100160097  1p22-p21  0  0  0.147  0.126  0.407  0  0.197  0.033  0.285  3.206  0.285  0  0  0.051  0  0  0  0  0  0.059  0.207  0.067  0  0  0  0.039  0.045  0  0  0.039  0  0  0  0.204  0.201  1.896  0  0  0.035  2.634  0.351  0.319  0.025  0  0.566  1.816  0.025  0  0  0  2.649  0.132  0.037  0.193  0  0.987  0.288  0  0  0.387  
 
 FRRS1  391059  1  100174258  100231349  1p21.2  0.215  0.268  0.489  0  0.461  0.157  0.186  1.015  0.069  0.123  0.877  0.552  1.418  0.496  0.51  0.454  0.46  0.291  0.166  0.147  1.182  0.129  0  0.237  0.39  0.39  0.601  0.137  0.59  0.136  0.43  0.502  0.87  0.172  0.075  0.489  0.395  0.265  0.037  0.686  0.612  0.052  0.027  0.477  0.519  0.532  0.048  0.514  0.209  0.327  1.014  0.162  0.223  0.333  0.227  0.257  0.471  0  0.354  0.269  
 
 AGL  178  1  100315639  100389579  1p21  0.91  1.306  0.462  0.228  1.458  0.549  0.804  1.245  0.643  0.968  1.642  0.816  1.568  2.254  1  1.168  1.655  1.961  1.843  1.106  1.535  1.576  0.114  0.19  0.685  0.922  0.846  0.89  0.75  0.339  1.13  0.606  0.859  0.486  0.47  1.658  0.565  0.879  1.041  1.3  1.103  0.516  1.097  1.139  0.599  0.872  0.679  1.318  0.971  2.072  1.19  0.648  0.992  0.411  0.878  0.681  1.103  0.131  0.922  1.296  
 
 SLC35A3  23443  1  100435344  100492534  1p21  0.719  1.896  0.936  0.945  1.194  0.524  0.821  0.813  0.882  1.08  1.25  0.562  1.938  1.426  1.192  1.418  2.37  1.096  1.967  0.666  0.797  1.013  0.5  0.336  0.316  0.747  0.297  0.708  0.625  0.427  1.086  0.532  0.538  0.394  0.38  1.179  0.387  1.177  1.856  1.111  1.52  0.675  1.021  0.71  0.695  0.733  0.234  2.297  1.572  3.048  2.121  0.626  0.74  0.469  0.866  0.774  1.488  0.556  1.164  1.196  
 
 MFSD14A  64645  1  100503788  100548929  1p21.2  2.073  3.67  2.013  2.852  2.322  1.928  2.402  3.028  2.563  2.854  3.43  1.605  2.856  2.711  2.57  3.246  2.726  2.533  4.369  2.885  2.147  2.838  1.58  1.703  2.627  2.42  2.336  2.234  2.012  1.275  2.787  2.131  2.1  1.449  1.566  3.373  2.087  2.725  3.388  2.667  2.567  1.656  2.474  2.162  1.334  1.849  1.685  2.965  2.654  4.423  2.685  1.582  2.313  1.931  2.709  2.703  3.224  1.973  3.249  3.141  
 
 SASS6  163786  1  100549100  100598651  1p21.2  0.652  2.963  0.395  0.631  1.168  0.66  0.684  0.763  0.334  0.971  1.576  0.481  0.98  1.963  0.874  1.356  1.288  2.527  2.524  1.368  1.513  1.49  0.377  0.493  0.89  0.692  0.494  0.705  0.556  0.388  1.073  0.655  0.849  0.577  0.494  1.353  0.393  0.895  1.225  1.023  1.038  0.697  1.226  1.264  0.708  0.737  0.333  1.325  1.314  2.096  1.226  0.551  0.92  0.444  0.973  0.714  1.471  0.727  1.26  1.026  
 
 TRMT13  54482  1  100598705  100616054  1p21.2  0.536  2.331  0.618  0.816  1.042  0.402  1.063  0.887  0.721  1.262  0.955  0.432  0.768  1.417  0.733  1.051  1.111  2.767  2.474  1.067  1.152  1.384  0.632  0.508  1.289  0.855  0.712  0.841  0.438  0.095  1.065  0.717  0.83  0.611  0.442  1.007  0.703  1.283  1.082  0.878  0.653  0.716  1.086  0.787  0.625  0.465  0.304  0.944  0.71  2.131  1.292  0.427  0.853  0.506  0.746  0.751  1.362  0.325  0.626  1.167  
 
 LRRC39  127495  1  100614003  100643829  1p21.2  0  0.255  0  0  0.155  0  0.073  0.17  0.109  0.251  0.426  0  0.105  0.465  0.136  0.131  0.161  0.687  1.422  0.206  0  0.353  0  0  0.133  0.183  0.049  0.064  0  0.125  0.42  0.1  0  0.131  0  0.231  0  0.258  0.213  0.159  0  0.722  0  0  0  0  0  0.136  0  0.65  0.177  0  0  0.094  0.085  0  0.149  0  0  0  
 
 DBT  1629  1  100652477  100715409  1p31  0.847  1.266  0.626  0.858  1.306  0.422  1.16  0.982  1.072  1.333  1.226  0.651  1.006  1.481  0.934  0.737  0.989  1.108  1.274  1.107  0.75  0.876  0.736  0.322  0.754  0.912  0.787  0.652  0.435  0.253  1.016  0.686  0.634  0.655  0.415  0.833  0.507  1.038  1.259  1.041  0.716  0.453  1.362  0.841  0.674  0.638  0.445  1.205  0.497  1.493  1.592  0.324  0.685  0.481  0.764  0.778  1.1  0.356  0.743  0.894  
 
 LOC100506007  100506007  1  100730297  100731730  -  0  0  0.344  0  0.249  0.642  0.15  0  0  0.359  0.134  0.182  0.29  0.346  0.213  0  0  0.362  0.316  0.159  0  0.194  0.362  0.42  0.378  0.148  0.125  0.105  0.178  0.085  0.346  0.221  0.272  0  0  0.424  0  0  0.203  0  0  0.638  0.903  0  0  0  0.093  0  0  0.186  0  0.469  0  0.171  0.241  0.383  0.145  0.479  0  0  
 
 RTCA  8634  1  100731713  100758325  1p21.2  1.252  5.048  1.822  2.698  1.458  1.631  3.406  2.805  1.799  2.887  3.528  1.391  2.385  3.18  2.006  2.216  2.568  4.897  4.086  2.592  2.34  2.431  1.539  1.386  2.766  2.654  2.318  2.028  2.343  1.252  3.293  2.47  2.205  1.545  1.478  2.856  1.885  3.17  3.766  2.584  1.983  1.587  2.935  1.673  1.846  1.998  1.177  2.871  1.767  4.281  3.189  1.46  2.27  2.06  2.556  2.164  3.383  1.308  2.839  2.903  
 
 CDC14A  8556  1  100818022  100985833  1p21  0.218  1.506  0.348  0.554  0.608  0.726  0.509  0.614  0.342  0.617  0.541  0.282  0.619  0.88  0.588  0.375  1.363  1.446  1.46  0.846  1.883  0.754  0.393  0.159  0.689  0.463  0.523  0.664  0.336  0.331  0.635  0.692  0.597  0.407  0.337  1.159  0.54  0.977  0.453  1.104  0.504  0.321  1.532  0.835  0.424  0.315  0.263  1.229  0.44  1.329  0.616  0.628  0.625  0.348  0.527  0.308  0.77  0.253  0.543  0.519  
 
 GPR88  54112  1  101003727  101007583  1p21.3  0  0.058  0.169  0.123  0.054  0.125  0  0.045  0.112  0.302  0.033  0.078  0  0.056  0  0.037  0.089  0.283  0.072  0.058  0  0.376  0.229  0.052  0.053  0.034  0.091  0.044  0  0.118  0  0  0  0.175  0.049  0.078  0.038  0  0.101  0  0.029  0.691  0.04  0  0  0.07  0.033  0.078  0  0.072  0.026  1.289  0.026  0.15  0.105  0.073  0  0  0  0.043  
 
 VCAM1  7412  1  101185195  101204601  1p32-p31  0  0  0.072  0.141  0  0  0.409  0.956  0.513  4.346  0.156  0  0  0.017  0  0  0  0.051  0  0.048  0  0.077  0  0  0  0  0.022  0  0  0.033  0  0  0  0.227  0  0.052  2.953  4.176  0.042  0  0  0.057  0.038  0  0  0  0  0  0  0  0  0.087  2.055  1.563  0.648  4.628  3.363  0  0.222  3.345  
 
 EXTL2  2135  1  101337927  101360735  1p21  0.538  3.009  1.354  1.619  1.091  0.554  1.893  2.458  1.889  2.39  2.331  0.645  1.492  2.29  1.358  1.364  1.428  2.172  2.876  1.21  1.29  1.403  1.062  0.821  2.147  1.891  1.802  1.701  1.528  0.485  1.898  1.369  1.692  1.502  0.716  2.173  0.848  1.699  1.093  1.654  0.956  1.058  2.695  0.771  0.774  0.508  0.986  1.832  1.046  3.1  1.031  0.833  1.061  0.228  1.072  1.289  1.681  1.117  1.449  1.521  
 
 SLC30A7  148867  1  101361631  101447311  1p21.2  0.787  2.763  1.053  1.325  1.429  1.087  2.039  2.151  1.529  1.999  2.195  0.927  1.56  1.868  1.487  1.793  1.537  1.604  2.327  1.379  0.722  1.405  0.877  0.661  1.797  1.703  1.287  1.881  1.431  0.675  1.824  1.379  1.582  1.126  0.679  1.901  1.349  1.631  2.307  1.692  1.053  0.873  1.772  1.405  1.185  1.043  0.621  1.695  1.311  2.508  1.903  0.7  1.384  0.917  1.132  1.653  2.043  0.718  1.572  2.136  
 
 DPH5  51611  1  101455179  101491362  1p21.2  2.106  3.777  1.538  2.233  2.221  1.461  3.118  1.577  1.867  2.178  2.74  1.116  2.414  3.376  1.36  1.903  2.765  2.796  4.22  3.04  2.372  3.383  1.793  1.382  2.588  1.889  2.009  1.87  1.9  2.015  2.943  2.237  1.444  1.08  1.838  2.268  1.884  2.384  3.155  2.068  1.666  1.699  3.656  1.668  1.141  1.371  1.99  2.555  1.478  4.614  2.499  0.904  2.388  1.433  2.917  2.073  2.176  1.403  2.254  2.473  
 
 LOC102606465  102606465  1  101491401  101552935  1p21.2  1.158  2.091  1.064  1.705  1.107  1.194  1.628  1.194  0.942  1.75  1.569  0.412  1.525  1.756  0.376  0.935  0.659  3.159  3.707  0.818  0.649  1.942  0.78  0.798  0.536  0.951  0.565  0.365  0.278  0.251  1.387  0.745  0.242  0.185  0.499  1.852  0.903  1.513  0.941  1.136  0.854  0.921  0.979  0.709  0.566  0.861  1.46  1.508  0.566  1.502  1.115  0.601  1.498  0.527  1.524  1.054  1.178  0.583  1.253  1.148  
 
 LOC101928370  101928370  1  101700428  101702084  -  0  0.316  0  0  0  0.824  0  0  0.121  0.074  0.589  0  0  0.144  0  0  0  0.346  0.418  0  0  0.336  0  0  0.22  0  0.118  0  0  0.128  0  0  0  0  0  0.162  0  0  0.398  0  0  0.291  2.096  0  0  0  0  0.287  0  0.879  0.305  0.474  0.26  0.114  0.651  0.201  0.247  0  0.424  0.43  
 
 S1PR1  1901  1  101702304  101707076  1p21  0  1.378  0.124  0.799  0.027  1.866  0.324  1.185  0.239  1.755  2.835  0.059  0  0.079  0  0.04  0  0.074  2.855  0.116  0  2.559  0.138  0.357  0.873  0  0.114  0  0  0.091  0  0  0  0.149  0.049  0.587  0.114  0  2.021  0  0.208  0.515  3.251  0  0.704  0.239  0.029  2.705  1.1  3.502  1.773  0.644  2.203  0.261  2.01  2.1  1.933  0.571  1.951  1.388  
 
 LINC01307  104355286  1  101789392  101842869  1p21.2  0  0  0  0  0  0  0  0  0  0  0  0  0  0  0  0  0  0  0  0  0  0.363  0  0  0  0  0  0  0  0  0  0  0  0  0  0  0  0  0  0  0  0  0  0  0  0  0  0  0  0  0  0  0  0  0  0  0  0  0  0  
 
 OLFM3  118427  1  102268122  102462790  1p22  0  0  0.12  0  0  0  0  0  0.06  0.02  0  0  0  0.023  0  0  0  0  0.239  0.055  0  0.059  0.091  0  0  0  0.087  0.087  0  0.082  0.079  0  0  0.185  0  0  0  0  0.022  0  0  0.201  0.026  0  0  0  0.083  0  0  0  0  0.164  0  0.04  0  0  0  0  0  0  
 
 DNAJA1P5  94236  1  102337567  102360299  1p22  0  0  0.099  0  0  0  0  0  0  0.05  0  0  0  0  0  0  0  0  0  0  0  0.032  0  0  0  0  0.126  0  0  0.066  0  0  0  0  0  0  0  0  0.047  0  0  0.034  0.087  0  0  0  0.051  0  0  0  0  0.119  0  0  0  0  0  0  0  0  
 
 COL11A1  1301  1  103342022  103574052  1p21  0  0.017  0.06  0  0.01  0.043  0.212  0.048  1.856  5.066  4.817  0  0  0.011  0  0  0  0.093  0  0.022  0  0.193  0  0.1  0  3.286  0.131  0.373  1.512  0.105  1.877  0.324  0  0.077  0  0  0.022  0  0.022  0  0  0.726  4.858  0  0.558  0.023  0.022  0  0  0  0.018  0.071  0.014  0.03  0.017  0.189  1.123  0.082  0  0  
 
 LOC101928436  101928436  1  103960700  104068105  -  0  0  0  0  0  0  0  0.16  0  0  0  0  0  0  0  0  0  0  0  0  0  0  0  0.441  0  0  0.084  0  0  0.199  0  0  0  0  0  0  0  0  0  0  0  0  0.142  0  0  0  0.155  0  0  0  0.185  0  0  0  0  0  0  0  0  0  
 
 RNPC3  55599  1  104068577  104097859  1p21  0.788  2.559  0.326  0.662  1.469  0.782  1.223  0.873  0.813  1.536  1.625  0.764  0.844  1.534  0.927  1.469  1.356  2.838  2.086  2.142  1.004  2.219  0.73  0.559  0.912  0.792  0.991  1.01  0.992  0.341  1.288  0.967  0.86  0.542  0.65  1.296  0.614  1.213  1.976  1.008  1.032  0.841  2.183  1.07  0.754  0.983  0.747  1.324  0.871  1.599  1.023  0.66  0.686  0.886  1.317  0.793  1.281  0.829  1.325  1.112  
 
 AMY2B  280  1  104097265  104122156  1p21  0.113  0.501  0.129  0.324  0.302  0  0.112  0.104  0.593  0.265  0.419  0.218  0.129  0.424  0.144  0.326  0.616  0.537  2.161  0.866  0  0.617  0.318  0.195  0.214  0.226  0.047  0.156  0.084  0.034  0.345  0.238  0  0.135  0.048  0.232  0.063  0.347  0.773  0  0.335  0.321  0.689  0.454  0  0.08  0.04  0.228  0.319  1.16  0.242  0  0.049  0.094  0.372  0.184  0.328  0  0.495  0.119  
 
 ACTG1P4  648740  1  104112025  104114008  1p21.1  2.023  0.374  2.519  1.68  1.565  0.768  3.227  1.728  1.789  1.978  2.264  1.231  2.01  1.338  1.261  1.394  2.078  0.128  2.234  1.626  0.388  2.527  0.876  0.597  1.428  1.497  3.458  1.535  2.293  3.606  0.869  0.883  1.032  1.134  1.402  1.341  2.221  1.036  2.009  1.523  0.855  1.265  3.97  0.8  0.651  0.681  2.307  1.176  0.488  1.969  1.712  0.883  2.955  1.871  2.844  2.161  1.554  0.882  2.059  1.366  
 
 AMY2A  279  1  104159953  104168402  1p21  0  0  0.144  0  0  0  0  0  0.106  0  0  0  0  0.038  0  0  0.078  0  0.334  0  0  0.069  0  0  0  0  0  0.036  0  0  0  0  0  0.172  0  0  0  0  0.08  0  0  0.374  0.243  0.085  0  0  0  0  0  0.121  0  0  0  0  0.059  0  0  0  0.089  0  
 
 AMY1C  278  1  104198302  104207172  1p21  0  0  0  0  0  0  0  0  0  0  0  0  0  0  0  0  0  0  0.174  0.086  0  0  0  0  0  0  0  0  0  0  0  0  0  0  0  0  0  0  0  0  0  0  0  0  0  0  0  0  0  0  0  0  0  0  0  0  0  0  0  0  
 
 AMY1B  277  1  104230040  104238889  1p21  0  0.143  0  0  0  0  0.049  0  0  0  0  0  0  0.051  0  0  0  0  0  0.11  0  0  0  0  0  0  0  0  0  0  0  0  0  0  0  0  0  0  0.148  0  0  0.04  0  0  0  0  0  0  0  0  0  0  0.035  0  0  0.078  0  0  0  0  
 
 AMY1A  276  1  104292278  104301311  1p21  0  0.138  0.207  0  0.071  0  0.619  0.131  0.218  0.093  0.154  0.14  0.277  0.054  0  0.059  0.199  0  0.396  0.124  0  0.358  0  0.109  0.094  0.183  0.339  0.125  0.211  0.675  0  0  0  0.201  0.189  0  0.355  0.093  0.196  0  0.079  0.071  0.78  0  0  0.083  0.194  0  0  0.203  0.115  0  0.227  0  0.267  0.186  0  0  0.217  0  
 
 LOC100129138  100129138  1  104615644  104619693  1p21.1  0  0.069  0.453  0.23  0.141  0  0.267  0  0.402  0.282  0.192  0  0  0.056  0  0  0.184  0  0.18  0.115  0  0.105  0.464  0  0  0  0.284  1.808  0.089  0.083  0  0  0  0.167  0  0  0.079  0  0  0  0  0.179  0.293  0  0  0  0.051  0  0  0  0.155  0.463  0  0  0.086  0  0  0  0  0  
 
 PRMT6  55170  1  107599266  107601916  1p13.3  4.47  1.268  0.932  2.098  2.135  1.023  2.087  1.878  2.192  2.501  2.832  1.289  2.586  1.716  2.016  1.978  1.345  2.278  3.435  2.466  2.411  1.98  0.837  1.121  2.106  0.955  1.851  2.863  1.933  0.81  2.176  2.627  1.871  1.038  0.049  2.529  1.457  0  1.747  2.065  2.224  2.003  2.197  2.375  2.105  1.121  0.942  1.827  2.298  2.33  2.142  1.262  2.388  1.521  1.779  1.801  2.058  1.428  1.699  2.147  
 
 NTNG1  22854  1  107682628  108024475  1p13.3  0  2.305  0.096  0  0.036  0.055  0.114  0.237  0.105  3.348  1.575  0.047  0.058  0.031  0  0.029  0  0.143  0  0.046  0  0.136  0.286  0  1.946  0  0.084  0.371  0  0.09  0  0  0  0.196  0  0.076  0.427  0  2.922  0  0.592  0.465  0.042  0  0.076  0.075  0.035  0.912  2.229  2.239  3.248  0.343  2.633  1.07  0.984  0.578  2.511  0  1.436  2.041  
 
 VAV3  10451  1  108113781  108507545  1p13.3  0.79  0.025  0.149  0.137  3.954  0  0.792  0.04  0.276  1.369  0.411  0.041  0  0.022  0  0  0.056  0  4.522  1.347  0.136  2.254  0.232  0.051  0  0.214  0.313  0.035  2.819  0.062  2.484  1.186  1.988  1.506  1.24  0.207  0  0.057  0.04  0.382  0.308  0.608  0.113  3.517  1.34  0.098  0.048  0  1.678  0.151  0.339  0.117  0.173  0.712  2.358  1.42  0.324  0  0.047  0  
 
 VAV3-AS1  100873946  1  108507064  108537229  -  0  0  0  0  0  0  0  0  0  0  0  0  0  0  0  0  0  0  0.46  0  0  0.172  0  0  0  0  0  0  0  0.092  0  0  0  0  0  0  0  0  0  0  0  0  0  0  0  0  0.12  0  0  0  0  0  0  0  0  0  0  0  0  0  
 
 SLC25A24  29957  1  108677343  108742980  1p13.3  2.317  5.631  1.706  2.569  2.566  1.792  1.832  2.515  1.522  2.246  2.639  1.099  2.556  2.627  2.499  2.856  3.301  2.943  2.133  1.234  1.517  1.289  1.138  0.844  2.929  1.891  2.186  2.174  1.842  0.86  2.264  2.226  1.655  1.068  1.574  2.953  2.305  3.1  3.402  1.971  2.037  1.621  2.859  2.572  1.209  1.928  0.758  2.812  2.324  3.806  3.145  1.437  2.12  2.176  2.473  2.511  2.718  2.449  3.015  3.178  
 
 NBPF4  148545  1  108765962  108786703  1p13.3  0.211  0  0.074  0  0.051  0  0  0  0.116  0  0  0  0  0  0  0  0.1  0.107  0  0.077  0.586  0.03  0.171  0.066  0.124  0.047  0.091  0.19  0  0.044  0.201  0.198  0  0  0.048  0.07  0  0  0  0  0  0.042  0.032  0  0  0  0.025  0.258  0  0.643  0  0.113  0  0  0  0  0  0  0  0  
 
 NBPF6  653149  1  108992903  109013260  1p13.3  0.349  0  0.084  0  0.084  0  0  0  0.081  0  0  0  0  0  0  0  0.182  0.083  0  0  0.827  0.026  0.287  0.066  0.103  0.089  0.028  0.292  0  0.029  0.142  0.34  0  0.118  0  0.073  0  0  0.025  0  0  0.04  0  0  0  0  0  0.368  0  0.565  0.041  0  0  0  0  0  0  0  0  0  
 
 FAM102B  284611  1  109102970  109181949  1p13.3  3.053  3.893  0.678  1.003  0.964  0.675  1.805  0.98  0.874  0.851  1.607  1.172  1.173  1.751  0.753  0.643  2.263  1.168  1.653  0.889  1.278  0.891  0.385  0.165  0.773  1.874  0.848  2.666  3.256  0.749  1.529  1.099  0.592  0.425  0.782  2.55  0.926  1.219  1.109  1.501  0.855  0.801  1.826  0.477  0.218  0.106  0.626  1.929  1.546  2.409  2.685  0.57  0.781  0.819  0.802  0.947  1.057  2.041  0.617  0.759  
 
 HENMT1  113802  1  109190909  109204148  1p13.3  2.876  1.902  0.737  2.13  0  0  0.121  0.05  0.138  0.09  0  1.906  2.002  0.028  2.231  2.164  0  1.169  5.133  2.092  0  4.079  0.789  0.103  1.334  0  0.165  0.61  1.921  0.085  0  0  0  0.172  0.411  2.316  0.985  0  0.1  3.115  2.063  0.303  3.275  2.555  1.105  1.287  1.452  1.666  0.997  3.659  0.656  1.087  0  0.045  0.198  0.576  0  1.638  0  0  
 
 PRPF38B  55119  1  109234931  109244422  1p13.3  3.024  4.83  2.003  2.578  1.95  1.823  2.464  2.642  2.163  2.695  2.717  1.892  2.373  3.43  2.693  2.557  2.635  4.437  4.384  2.974  2.79  3.361  1.951  1.652  2.301  2.217  2.265  3.057  2.816  1.334  2.875  2.74  2.448  1.947  1.814  2.31  1.888  2.052  2.482  2.663  2.291  1.926  2.697  2.888  1.581  1.815  2.143  2.887  2.364  3.322  2.699  1.482  2.458  1.758  2.494  2.147  2.782  2.179  2.808  2.541  
 
 FNDC7  163479  1  109255555  109285367  1p13.3  0.071  0.088  0.136  0  0.046  0.1  0.059  0.098  0.157  0.064  0.071  0.044  0.091  0.049  0  0.06  0  0  0.033  0  0  0.116  0.315  0  0  0  0.077  0.091  0.074  0.046  0  0.035  0  0.271  0  0  0  0  0.058  0  0.047  0  0.026  0  0.051  0.05  0.169  0.09  0  0.031  0.051  0.058  0.025  0.051  0.045  0  0.054  0  0.054  0  
 
 STXBP3  6814  1  109289284  109352148  1p13.3  2.036  4.402  0.972  1.234  1.764  0.926  1.538  1.614  1.694  1.984  2.423  1.04  1.952  2.476  1.253  1.728  2.175  3.762  3.4  1.705  1.583  1.365  0.797  0.647  1.417  1.873  0.963  1.918  1.913  0.57  2.219  2.121  1.333  0.85  0.784  1.958  0.917  1.882  2.347  1.908  1.228  1.121  1.964  1.589  0.748  1.103  0.799  1.282  1.806  3.123  2.077  0.759  1.254  1.087  1.586  1.496  1.917  1.106  1.884  2.048  
 
 AKNAD1  254268  1  109358519  109400864  1p13.3  0  0.158  0.062  0  0.034  0  0  0.035  0.119  0.06  0.162  0  0  0.067  0  0.028  0  0.243  0  0  0  0.063  0.11  0  0  0  0.128  0.056  0  0.055  0.038  0.057  0.047  0.147  0  0.044  0  0  0.026  0  0.065  0.23  0.036  0  0  0  0.028  0  0  0  0.073  0.16  0  0.052  0.023  0.087  0  0.098  0  0  
 
 SPATA42  642864  1  109399838  109401146  1p13.3  0  0  0  0  0  0  0  0  0  0  0  0  0  0  0  0  0  0  0  0  0  0  0  0  0  0  0  0  0  0.093  0  0  0  0  0  0  0  0  0  0  0  0  0  0  0  0  0  0  0  0  0  0  0  0  0  0  0  0  0  0  
 
 GPSM2  29899  1  109419602  109473044  1p13.3  2.864  4.206  1.557  1.839  2.069  1.398  1.885  2.12  1.504  2.874  2.836  1.189  2.236  2.927  2.621  2.329  2.408  2.683  2.65  2.064  1.195  1.524  0.632  0.48  1.333  1.521  1.909  2.203  1.825  0.595  1.966  1.615  1.93  0.982  1.25  1.973  1.328  2.43  1.55  2.224  2.238  1.21  1.89  1.715  1.182  1.288  0.833  1.313  1.893  1.965  2.681  1.623  1.838  1.114  2.165  1.93  2.518  1.73  2.329  1.74  
 
 CLCC1  23155  1  109472129  109506121  1p13.3  2.502  3.936  1.752  2.174  1.79  1.505  2.668  2.072  2.349  2.831  3.054  0.672  1.948  1.959  2.346  1.695  1.736  2.172  1.264  2.44  2.395  1.382  1.714  1.328  1.49  1.618  2.504  2.403  2.041  1.466  2.245  2.507  1.882  1.292  1.533  2.683  1.54  2.267  2.589  2.475  1.808  1.081  2.616  2.062  1.16  1.311  1.957  1.828  2.355  2.121  2.631  1.285  1.741  1.547  1.835  2.082  2.333  2.048  1.992  2.333  
 
 WDR47  22911  1  109512837  109584850  1p13.3  1.098  2.546  0.903  1.301  0.597  0.79  1.484  1.294  1.374  1.612  1.899  0.19  0.939  1.33  0.878  0.397  0.673  0.831  1.712  0.669  0.286  0.871  0.52  0.122  0.78  1.246  1.579  1.125  1.554  0.308  1.262  1.405  1.054  0.541  0.392  1.007  0.717  1.27  0.993  1.17  0.566  0.37  1.933  1.407  0.496  0.56  0.137  1.408  1.111  1.825  1.645  0.355  0.984  0.594  0.947  0.924  1.549  0.456  1.076  1.43  
 
 TAF13  6884  1  109606997  109618624  1p13.3  0.67  3.049  0.583  0  0.753  0.978  0.807  1.497  0  0.883  1.376  0.397  0.588  0.629  0  0.33  0  0.803  0.242  0  1.912  0.218  0  0.302  1.367  0.401  0.295  1.645  0.86  0.184  0.527  0.68  0.538  0  0  1.342  0.349  0.679  1.178  0.878  0.966  0.141  0  2.444  0.703  1.347  0  1.353  1.758  1.01  0.821  0.403  0.58  1.709  0.476  0.828  0.921  0.896  0.742  0.795  
 
 TMEM167B  56900  1  109633402  109639554  1p13.3  2.176  4.089  2.399  2.226  2.334  1.735  3.278  2.51  2.963  2.393  2.627  1.346  1.932  2.316  0.917  1.851  1.98  2.295  3.207  2.266  1.951  2.12  1.832  0.952  1.944  1.487  1.925  2.097  2.178  0.631  2.048  2.128  1.364  1.298  0.925  1.613  1.357  2.032  2.533  1.597  1.403  1.261  2.675  1.704  0.952  1.555  2.076  1.435  1.47  3.184  2.909  0.71  1.532  1.645  1.816  1.844  2.036  1.806  1.697  1.83  
 
 SCARNA2  677766  1  109642814  109643234  1q13.1  4.071  0.564  3.21  2.562  2.036  6.596  1.109  3.398  1.989  2.414  2.878  3.14  2.872  2.216  3.397  3.361  3.218  0  0.952  2.182  5.268  2.809  1.631  3.959  3.128  2.41  2.138  4.919  4.374  0.21  2.832  2.972  2.31  1.343  2.941  2.692  2.851  3.484  2.046  3.967  3.285  2.393  2.091  4.606  4.248  3.194  1.953  4.274  2.642  2.209  2.512  4.196  3.952  3.589  2.076  3.019  3.292  3.974  3.386  2.764  
 
 C1orf194  127003  1  109648572  109656479  1p13.3  0  0  0  0  0.704  0  0  0  0  0  0  0  0  0.08  0  0  0  0  0  0.215  0  0.093  0  0  0  0  0.108  0  0  0.085  0  0  0  0  0  0  0  0  0  0  0  0  0  0  0  0.135  0.077  0  0  0  0  0  0  0  0  0  0  0  0  0  
 
 KIAA1324  57535  1  109656584  109749403  1p13.3  2.314  0.366  0.212  0  3.113  0.292  0.455  0.402  0.19  0.324  0.637  0.264  0.052  0.114  0.046  0.035  0.935  0.073  0.099  0.047  0.067  0.109  0.312  0  0.074  0.059  0.152  0.348  0.057  0.094  0.036  0.061  0.023  0.225  0.107  0.384  0.102  0.055  1.93  0.097  0.07  0.205  0.059  0  0.042  0.072  0.325  0.171  0.117  0.168  0.088  0.312  0.174  0.876  0.547  0.148  0.118  0.764  0.074  1.029  
 
 SARS  6301  1  109756514  109780804  1p13.3  5.109  6.755  5.56  6.306  4.024  4.041  5.492  5.3  6.237  5.285  5.591  3.877  4.668  5.588  4.53  4.653  5.643  4.735  6.087  4.684  5.16  4.869  4.546  2.816  4.359  3.834  5.326  5.09  4.992  4.305  4.888  5.483  4.056  3.2  3.426  4.441  4.522  5.015  6.102  4.678  3.809  3.784  5.932  4.129  3.925  4.164  5.398  5.424  4.702  6.619  5.067  3.223  4.443  4.187  5.038  4.352  5.008  4.565  4.899  4.789  
 
 CELSR2  1952  1  109792640  109818378  1p21  3.295  0.5  0.643  0.655  1.974  0.737  0.738  0.369  2.886  3.16  2.986  0.3  1.535  1.438  2.321  0.737  0.702  0.273  0.74  0.062  1.937  0.786  0.438  0.377  0.025  2.409  2.505  2.697  1.554  1.068  2.483  1.105  1.05  0.483  0.798  2.687  0.636  0.47  0.533  1.263  1.64  1.132  1.526  1.31  2.682  2.178  0.397  2.195  3.874  1.427  0.726  1.231  1.668  2.256  1.113  2.674  2.091  0.981  2.205  1.637  
 
 PSRC1  84722  1  109822175  109825790  1p13.3  4.398  4.386  2.626  3.052  2.207  3.338  3.292  2.776  2.64  3.982  4.885  1.681  2.62  3.935  3.283  3.16  3.422  1.522  4.787  2.156  2.753  3.386  0.808  0.92  2.757  2.263  3.098  3.808  3.306  2.134  3.714  3.885  3.366  2.215  2.292  2.462  3.046  4.034  2.679  3.9  2.196  3.516  3.369  3.054  2.16  2.598  2.356  4.172  3.407  4.892  3.149  2.254  3.458  2.129  3.602  2.561  3.481  3.822  3.431  2.658  
 
 MYBPHL  343263  1  109834986  109849663  1p13.3  0  0  0  0  0.078  0.116  0  0  0.165  0.089  0.081  0.25  0  0.069  0  0.077  0  0.144  0  0  0  0.372  0  0  0  0  0.11  0.307  0  0.077  0  0  0  0  0  0  0  0  0.157  0  0  0  0.064  0  0  0  0.049  0  0  0  0  0.477  0  0.21  0.06  0  0  0  0  0  
 
 SORT1  6272  1  109852187  109940563  1p13.3|1p21.3-p13.1  3.038  3.263  2.222  0.454  3.277  2.801  1.63  2.279  3.637  2.919  3.92  1.592  2.614  2.306  2.864  2.496  2.441  1.068  3.529  0.1  2.982  2.367  0.844  1.359  0.022  3.999  3.367  4.649  4.989  2.755  4.264  2.84  2.8  1.476  0.887  3.327  2.348  2.525  1.633  1.376  3.173  0.962  2.51  1.608  2.534  2.517  1.906  2.672  3.178  2.692  1.286  1.235  1.892  0.117  1.494  2.006  1.204  2.712  0.068  3.963  
 
 PSMA5  5686  1  109941652  109969108  1p13  3.959  6.105  1.871  2.505  3.422  2.616  4.917  3.891  2.066  3.322  4.703  2.827  3.63  4.846  2.776  4.001  3.908  4.331  5.414  4.026  4.134  3.736  1.808  2.643  3.561  2.526  3.102  3.88  3.418  1.936  3.795  4.219  3.108  1.382  2.679  3.597  3.087  3.706  4.364  3.925  3.344  2.404  4.202  3.114  2.971  2.925  1.932  3.697  3.631  5.199  3.697  2.24  3.424  3.005  3.318  3.567  3.474  4.227  3.809  3.696  
 
 SYPL2  284612  1  110009099  110024764  1p13.3  0.09  0.027  0.113  0.069  0.137  0.251  0.06  0.051  0.435  0.083  0.086  0.027  0  0.318  0.153  0.036  0.053  0.068  0.028  0.062  0  0.088  0.152  0.074  0  0.148  0.147  0.039  0.037  0.095  0.052  0.203  0  0.192  0.132  0.084  0.102  0.08  0.061  0.33  0  0.146  2.351  0.038  0  0.066  0.054  0.084  0.048  0.025  0.029  0.321  0  0.104  0.057  0.085  0.043  0.038  0  0.05  
 
 ATXN7L2  127002  1  110026560  110035420  1p13.3  1.464  0.3  0.455  1.299  0.639  0.61  1.29  1.486  1.087  1.356  1.477  0.507  1.253  1.148  1.385  1.172  1.034  0.086  2.741  0.722  1.617  2.174  0.583  0.731  1.003  0.776  1.118  1.259  1.058  0.786  1.135  1.489  1.372  0.487  0.374  0.93  0.605  1.225  0.739  1.797  0.88  1.321  2.451  0.952  0.771  0.656  0.796  1.589  1.156  2.382  0.788  1.021  0.702  0.556  1.027  0.902  1.202  0.911  0.972  0.804  
 
 CYB561D1  284613  1  110036657  110043063  1p13.3  1.619  0.756  0.98  0.747  1.086  0.485  1.592  1.901  1.558  1.907  1.466  0.821  1.192  0.918  1.121  1.291  0.621  0.28  2.002  0.982  0.67  0.78  0.86  0.419  0.885  1.365  1.758  1.961  1.431  0.861  1.245  1.265  1.106  0.708  0.549  1.053  0.757  1.524  0.983  1.085  0.889  0.777  1.158  0.673  0.912  0.706  1.546  1.02  1.324  1.521  1.118  0.653  1.024  0.806  0.683  0.9  1.049  0.396  1.1  1.078  
 
 AMIGO1  57463  1  110049445  110052336  1p13.3  0.825  0.788  0.447  0.289  0.821  0.044  0.109  0.095  0.97  0.335  0.723  0.149  0.691  0.074  1.177  0.17  0.134  0.154  0.126  0.26  0.203  0.321  0.481  0.135  0.137  0.151  0.654  0.814  0.635  0.432  0.062  0.222  0.682  0.41  0.267  0.867  0.214  0.983  0.117  1.241  0.211  0.289  0.557  0.259  0.347  0.215  0.295  0.688  1.072  0.353  0.342  0.797  0  0.312  0.289  0.376  0.103  0.308  0.08  0.539  
 
 GPR61  83873  1  110082493  110088455  1p13.3  0.271  0.057  0.301  0  0.24  0.066  0.395  0.161  0.178  0.37  0.334  0.207  0.258  0.174  0.237  0.326  0.266  0.107  0.235  0.331  0  0.635  0.367  0.052  0.143  0.117  0.453  0.44  0.318  0.215  0.124  0.136  0.125  0.131  0.074  0.142  0.232  0.124  0.526  0.299  0.086  0.308  0.465  0.094  0  0.044  0.475  0.052  0.087  0.062  0.167  0.27  0.574  0.395  0.455  0.156  0.489  0.051  0.267  0.068  
 
 GNAI3  2773  1  110091185  110138454  1p13  3.369  5.849  2.877  3.024  2.766  2.359  3.796  3.609  2.928  3.377  4.066  1.989  3.056  4.22  2.409  2.906  3.018  4.193  4.256  2.884  2.785  2.51  1.708  1.34  3.166  2.292  2.776  3.714  3.185  2.165  3.286  2.939  2.572  1.962  1.987  2.848  2.678  3.101  3.272  3.418  2.244  2.153  4.009  2.641  2.495  2.296  2.791  3.077  3.378  4.249  3.305  1.497  3.523  2.21  2.783  3.023  3.809  2.525  3.836  3.366  
 
 GNAT2  2780  1  110145888  110155705  1p13.1  0.212  0.148  0.265  0  0.231  0  0.229  0.358  0.173  0.144  0.338  0  0.271  0.18  0.296  0.145  0  0  0.086  0.137  0  0.176  0  0  0  0.151  0.167  0.454  0.369  0.061  0.283  0.256  0.158  0  0.268  0.328  0.109  0.313  0.213  0.244  0.189  0.51  0.063  0.477  0  0  0.33  0.48  0.123  0.128  0.271  0.384  0.223  0.182  0.168  0.1  0.347  0  0.265  0.337  
 
 AMPD2  271  1  110162434  110174677  1p13.3  2.71  1.682  3.085  3.298  2.207  2.008  4.646  4.476  2.942  3.809  4.149  1.886  3.106  2.762  3.096  2.323  2.292  0.177  4.741  2.37  2.735  3.233  1.651  1.908  3.483  2.601  3.707  4.787  3.994  2.742  3.354  4.411  3.627  2.052  1.921  3.211  3.317  3.581  3.011  4.43  2.282  3.07  3.811  2.89  2.054  2.191  1.087  2.693  1.453  3.691  3.274  1.732  4.597  3.263  3.379  3.321  4.463  2.61  3.663  3.535  
 
 GSTM4  2948  1  110198697  110208123  1p13.3  4.008  3.45  2.689  0.977  3.921  1.623  3.701  0.941  2.492  2.162  5.308  3.506  3.268  2.867  3.57  0.473  0.189  0.508  3.384  4.56  2.716  2.909  1.715  1.918  1.45  4.283  4.604  4.471  4.298  4.992  4.237  4.26  4.391  2.837  1.761  4.181  2.938  3.927  0.709  2.922  0.763  3.752  3.761  3.052  1.303  3.925  1.131  1.166  1.989  1.45  2.799  1.96  2.333  3.916  1.959  2.092  3.615  2.702  0  2.772  
 
 GSTM2  2946  1  110210643  110226619  1p13.3  0.453  1.717  2.131  0.607  3.992  1.889  2.368  0.278  1.61  0.947  3.169  0.249  0.415  0.694  0.99  0  0  0  2.272  1.033  0.726  3.837  0.389  0.64  0  3.241  1.344  2.045  2.95  1.124  2.144  2.857  0.502  0.41  0.637  3.05  1.472  3.383  0.128  3.129  0  3.222  0.804  1.242  1.852  1.745  0  1.581  0.214  1.488  2.454  1.596  0.086  1.996  1.477  0.837  2.23  3.468  0  0.874  
 
 GSTM1  2944  1  110230417  110236367  1p13.3  0.553  1.941  2.618  0.571  3.179  2.097  2.223  0.282  1.34  0.682  2.773  0.41  0.395  0.471  0.811  0  0  0  1.601  1.022  0.294  4.052  0.32  1.218  0  3.791  1.457  1.533  2.413  1.165  1.493  2.053  0.587  0.421  0.46  3.371  1.116  2.448  0.119  3.596  0  3.683  0.834  0.684  1.22  0.951  0  1.016  0.178  0.907  1.503  2.272  0.091  2.703  1.16  0.624  3.137  2.175  0  0.473  
 
 GSTM5  2949  1  110254863  110260890  1p13.3  0.188  0.058  0.166  0  0.171  0  0.169  0.08  0.147  0.103  0.346  0.114  0  0.063  0.129  0  0  0  0.165  0  0.17  0.168  0.226  0  0.091  0.249  0.17  0.166  0.254  0.257  0.18  0.169  0.174  0.206  0.077  0.215  0.186  0.224  0.082  0  0  0.15  0.143  0.185  0  0.131  0.067  0  0.101  0.089  0.135  0.214  0.081  0.234  0  0  0.139  0.175  0  0.114  
 
 GSTM3  2947  1  110276553  110283660  1p13.3  2.821  4.506  1.087  1.043  5.725  2.134  3.391  0.427  1.743  2.468  3.017  0.098  0.094  0.03  2.764  0.057  0  0.057  0.205  0.656  0.311  1.41  0.17  0  0  0.908  0.839  1.231  0.747  0.21  1.75  1.431  1.151  0.83  0.469  1.39  0.69  0.046  2.927  1.212  0.435  2.275  1.721  2.384  1.775  2.625  0.972  1.679  0.766  3.189  2.006  0.979  2.037  2.355  2.078  0.73  1.41  0.069  2.009  1.566  
 
 EPS8L3  79574  1  110292701  110306644  1p13.3  0  0  0.107  0  0.082  0  0  0  0.08  0  0  3.925  4.099  0.322  1.034  3.498  1.286  2.452  0  0  0  0.042  0.159  0  0  0  0.056  0  0  0.057  0  0  0  0  0.677  0  0.087  0  0.041  0  0  0.661  0.046  0  0  0.037  0.082  0  0  0  0.038  0.258  0  0.57  0  0  0  0  0  0  
 
 CSF1  1435  1  110453232  110473616  1p13.3  0.231  3.797  3.062  3.993  0.433  1.239  4.444  5.553  2.787  5.274  2.39  0.088  1.125  0.792  1.498  0.669  0  0  1.988  0.146  0.756  0.353  0.52  0.325  0.894  1.126  0.701  1.564  0.085  1.586  1.03  1.494  0.63  0.78  1.471  1.857  3.676  4.159  4.144  0.777  0.378  2.179  0.595  2.208  0.908  0.71  1.016  0.553  2.122  0.463  0.911  3.219  5.232  4.619  1.399  4.008  4.204  1.538  4.292  2.668  
 
 AHCYL1  10768  1  110527386  110566364  1p13.2  3.942  5.243  3.706  3.355  4.193  2.727  5.397  4.504  4.15  4.554  5.199  3.114  4.445  4.966  4.317  3.721  4.298  3.761  5  4.006  3.449  3.763  2.78  2.156  3.356  3.436  4.18  4.8  4.511  4.654  5.071  4.437  3.403  2.607  3.156  4.056  4.036  4.045  5.089  4.224  3.897  3.138  4.926  3.938  2.743  2.886  4.506  3.293  4.579  3.837  4.334  2.714  4.147  3.464  4.096  4.233  4.384  3.137  4.319  4.37  
 
 STRIP1  85369  1  110574198  110597263  1p13.3  1.79  1.294  1.57  2.14  1.628  1.576  2.283  2.167  2.141  2.373  2.448  1.055  2.26  1.67  2.353  1.944  1.507  0.272  3.251  1.452  2.147  1.835  0.786  1.02  1.697  1.853  2.185  3.325  2.488  1.742  2.443  2.42  2.001  0.851  1.119  2.316  1.818  2.032  1.743  2.654  1.606  1.492  2.437  1.957  1.652  1.398  0.675  2.387  2.013  3.005  2.386  1.325  2.153  1.142  1.781  1.696  2.357  1.326  2.099  2.131  
 
 ALX3  257  1  110602996  110613322  1p13.3  0.263  0  0.32  0.486  0.142  0.294  0.31  0.253  0.455  0.435  0.58  0.22  0.573  0.246  0.911  0.19  0.267  0.126  0.419  0  0.274  0.227  0  0.14  0.178  0.398  0.371  1.102  0.46  0.166  0.504  0.29  0.467  0.198  0.108  0.415  0.331  0.522  0.163  0.798  0.323  0.403  0.66  0.552  0.267  0.209  0  0.732  0.526  0.49  0.653  0.743  0.504  0.43  0.285  0.19  0.725  0  0.616  0.3  
 
 LINC01397  104355139  1  110625309  110652341  1p13.3  0  0  0  0  0  0  0  0  0  0  0  0  0  0  0  0  0  0  0  0  0  0  0  0  0  0  0.112  0  0  0.081  0.276  0  0  0  0  0  0  0  0.087  0  0  0  0  0  0  0  0  0  0  0  0  0  0  0  0  0  0  0  0  0  
 
 UBL4B  164153  1  110655061  110656569  1p13.3  0  0  0.089  0  0.132  0  0  0  0.126  0  0  0  0  0.108  0  0  0  0  0  0.11  0  0.115  0.387  0  0  0  0.057  0  0  0.311  0.133  0  0  0.225  0  0  0  0  0.111  0  0  0.082  0.035  0  0  0  0  0  0  0.084  0.07  0.452  0  0.078  0  0  0  0  0  0  
 
 SLC6A17  388662  1  110693131  110744823  1p13.3  0  0.027  0.169  0  0.607  0.073  0.055  0.069  0.163  0.051  0.046  0.062  0  1.49  0  0.05  0.052  0.083  0.026  0.087  0  0.156  0.246  0.029  1.481  0.497  1.087  1.035  1.327  0.926  3.152  0.845  0  0.221  0.378  0.911  0  0  0.255  2.388  0  0.13  1.435  0.091  0.037  0.15  0.125  1.807  1.08  0.793  0.042  0.721  0.014  0.107  0.522  0.04  0.125  0  0  0  
 
 KCNC4-AS1  100873974  1  110751072  110752609  -  0  0  0.178  0  0.108  0  0  0  0  0  0  0  0.382  0.099  0  0  0  0.153  0.253  0.127  0  0.276  0.259  0  0  0  0.207  0.131  0  0.119  0.275  0  0  0  0  0  0  0  0.422  0  0  0.181  0.789  0.379  0  0  0.059  0  0  0.088  0  0.171  0.064  0.146  0.076  0  0.088  0  0  0  
 
 KCNC4  3749  1  110753335  110776674  1p21  0.621  0.482  0.864  0.294  0.766  0.622  0.838  0.529  0.453  1.261  0.434  0.661  1.88  1.19  1.483  0.423  1.024  0.336  2.065  1.163  0.382  1.296  0.566  0.502  1.065  1.236  2.556  2.227  1.696  2.035  1.513  2.021  1.103  0.603  0.643  1.791  0.798  0.71  2.423  1.658  0.347  1.173  2.096  2.205  0.273  0.678  1.131  1.465  1.143  0.877  2.201  1.252  1.467  0.704  0.999  1.184  1.203  0.169  1.785  0.797  
 
 LOC440600  440600  1  110828998  110881793  1p13.3  0  0.126  0.159  0  0.041  0.11  0.187  0.116  0.15  0.103  0.056  0  0.11  0.149  0.107  0.092  0  0.146  0.184  0  0.106  0.238  0.324  0.065  0  0.039  0.168  0.063  0.091  0.057  0.083  0.083  0.09  0.34  0  0.083  0.048  0.074  0.164  0.108  0  0.053  0.018  0.071  0  0  0  0.073  0  0.103  0.086  0.235  0.1  0.118  0.055  0  0.078  0  0  0.045  
 
 RBM15  64783  1  110881944  110889303  1p13  2.769  2.431  1.606  2.178  1.862  1.95  2.088  2.918  1.193  2.57  2.888  1.81  2.457  2.917  2.829  2.343  2.259  3.34  2.214  2.303  3.15  2.317  1.274  1.66  1.791  1.828  2.058  2.921  2.732  1.695  2.255  2.139  2.217  0.847  1.523  2.359  2.316  2.309  1.575  3.055  2.316  1.543  2.219  2.6  1.869  1.965  1.493  2.854  2.697  2.018  2.372  1.874  2.772  1.783  1.901  2.037  2.652  2.014  2.93  2.107  
 
 SLC16A4  9122  1  110905472  110933704  1p13.3  0.53  0.817  0.553  0  0.483  0.249  2.714  0.355  0.941  1.122  2.475  0.774  0.59  0.436  0.637  0.652  0.421  0.457  0.517  0.294  0.411  0.542  0.221  0.158  0.16  2.387  1.08  2.298  1.528  1.092  1.156  2.48  1.313  1.048  1.414  0.295  0.581  1.449  0.203  0.455  0.261  1.859  0.217  0.815  0.122  0.225  0.186  0.767  0.439  0.207  0.284  0.24  0.308  2.843  2.252  3.131  0.753  0.45  0.643  1.489  
 
 LAMTOR5  10542  1  110943876  110950546  1p13.3  4.976  8.25  4.035  3.868  5.296  4.898  6.329  4.554  4.006  4.971  5.553  4.29  4.722  5.702  3.926  4.783  5.414  7.576  6.582  6.496  4.636  6.108  3.385  4.014  4.723  4.952  5  5.515  5.442  4.319  6.046  5.629  4.272  3.473  4.68  4.896  3.968  5.44  6.759  4.427  4.411  4.207  5.916  4.452  3.951  4.183  5.438  4.308  4.508  5  5.091  3.076  4.976  4.605  5.896  5.794  4.815  5.485  5.324  4.914  
 
 LAMTOR5-AS1  101410535  1  110950430  110958896  1p13.3  0.376  0.942  0  0  0.714  0.382  0.678  0  0  0.265  0.163  0.719  0.513  1.008  0  0.241  0.573  0.93  0.724  0.679  0  0.516  0  0  0  0.274  0.249  0.211  0.553  0.152  0  0.562  0  0  0.527  0  0.204  0  0.639  0  0.426  0.209  0  0  0  0.235  0  0  0  0.457  0.453  0.358  0.397  0.422  0.447  0  0  0  0.34  0  
 
 PROK1  84432  1  110993787  110999976  1p21  0  0  0.098  0  0  0  0  0  0.148  0  0  0  0  0  0  0  0  0  0  0  0  0.072  0  0  0  0  0.062  0  0  0.047  0  0  0  0  0  0  0  0  0  0  0  0  0.042  0  0  0  0  0  0  0  0  0  0  0  0  0  0  0  0  0  
 
 CYMP  643160  1  111023387  111033891  1p13.3  0  0  0  0  0  0  0  0  0  0  0  0.134  0  0.069  0  0  0.222  0  0.492  0  0  0.528  0  0  0  0  0.06  0  0  0.043  0  0  0  0.151  0  0  0  0  0.061  0  0  0  0  0  0  0  0  0  0  0  0  0  0.055  0.058  0  0  0  0  0  0  
 
 LOC440602  440602  1  111030301  111032880  1p13.3  0  0  0.156  0  0  0  0  0  0.099  0.039  0  0  0  0  0  0  0.09  0  0.048  0  0  0.076  0.329  0  0  0  0.102  0.026  0  0.058  0  0  0  0.133  0  0  0  0  0  0  0  0.039  0.049  0  0  0  0  0  0  0  0.044  0.349  0  0  0.033  0  0  0  0  0  
 
 KCNA10  3744  1  111059838  111061797  1p13.1  0  0.048  0.223  0  0  0  0  0  0.107  0.036  0  0.237  0  0  0  0  0  0  0  0  0  0.08  0.169  0  0  0  0.073  0  0  0.061  0  0  0  0.221  0  0  0  0  0.123  0  0  0.543  0.035  0  0  0  0.027  0  0  0  0.038  0.441  0  0.05  0.039  0  0  0  0  0  
 
 KCNA2  3737  1  111136201  111174096  1p13  0.116  0.094  0.266  0.21  0.172  0.241  0.192  0.169  0.255  0.744  0.079  0.109  0.408  0.049  0.388  0.102  0.121  0.088  0.239  0.126  0.201  0.178  0.473  0.438  0.26  0.351  0.173  0.268  0.067  0.176  0.558  1.185  0.393  0.186  0.101  0.225  0.233  0.162  0.09  0.191  0.241  0.218  0.083  0.182  0.171  0.259  0.123  0.188  0.245  0.268  0.249  0.387  0.213  0.148  0.085  0.172  0.084  0.192  0.421  0.289  
 
 KCNA3  3738  1  111196185  111217655  1p13.3  0  0.107  0.242  0  0.076  0.2  0.124  0.084  0.231  0.104  0.054  0.102  0  0.091  0  0.069  0.071  0.434  2.178  0.157  0  2.217  1.955  1.827  0.054  0.046  0.286  0.036  0  0.315  0  0.17  0.042  0.248  0.091  0.046  0.293  0  0.149  0  0.049  0.982  0.097  0  0.187  0.126  0.335  0.06  0  0.038  0.051  1.081  0.043  0.197  0.194  0.068  0.028  0  0  0  
 
 CD53  963  1  111413820  111442558  1p13  0  0  0.118  0  0  0  0.148  0  0  0.202  0  0  0  0.029  0  0  0  0  6.057  3.86  2.804  4.305  3.794  2.746  0  0  0.057  0.223  0  0.026  0  0  0  0  0  0.129  0  0.152  0.049  0  0  0.289  0  0  0  0.085  0  0  0  0.226  0.074  0.205  0  0  0  0  0  0  0  0  
 
 LRIF1  55791  1  111489811  111506566  1p13.3  1.717  3.539  1.274  2.071  2.256  1.768  1.79  2.265  1.606  2.226  2.001  1.566  2.004  2.687  0.823  1.841  2.33  3.208  3.544  2.875  3.385  0.213  2.076  0.91  2.346  1.93  1.042  1.735  1.108  0.654  2.346  2.371  0.841  0.89  1.888  2.192  0.523  2.388  3.01  2.245  2.185  1.271  2.104  1.666  1.504  1.403  0.777  2.089  2.494  2.948  2.783  1.637  1.161  1.26  2.209  1.557  1.274  1.69  2.009  1.957  
 
 DRAM2  128338  1  111659953  111682838  1p13.3  1.666  4.115  1.469  2.312  2.385  0.644  2.417  2.221  2.037  2.452  2.414  1.632  2.476  2.811  1.937  2.302  2.967  3.483  3.555  2.343  2.009  1.498  1.406  0.577  1.395  2.147  2.105  1.584  2.243  1.079  2.431  2.051  1.223  1.302  1.038  2.142  1.169  2.097  2.576  1.586  1.756  1.365  2.849  1.79  0.829  0.985  1.154  1.409  1.826  2.756  2.002  1.111  1.718  1.194  2.207  2.192  2.083  1.32  2.845  2.166  
 
 CEPT1  10390  1  111682248  111727724  1p13.3  1.881  2.626  1.22  2.11  2.402  1.088  1.93  1.788  1.815  2.202  2.116  1.433  2.645  3.188  1.988  1.941  1.909  1.727  4.622  1.347  1.967  2.448  1.517  1.06  1.495  1.54  1.574  2.064  1.855  0.687  2.311  2.151  1.335  1.299  0.75  1.955  1.6  1.65  1.768  1.99  1.482  1.137  1.214  1.881  0.824  1.162  0.58  1.831  1.489  2.762  2.386  0.979  1.656  1.071  1.828  1.637  1.746  1.231  2.115  2.148  
 
 DENND2D  79961  1  111728590  111747160  1p13.3  2.279  1.064  0.214  0.131  2.298  0  0.317  0.186  0.138  0.189  0.292  1.338  1.641  2.11  1.268  2.987  1.523  1.315  4.356  0.448  0  4.892  1.78  1.837  0  0.17  0.116  0.472  0.121  0.251  1.295  0.222  0.06  0.177  0.17  0.265  0.374  0.55  1.317  0.688  0.238  0.47  0.026  0.058  0.939  1.198  1.457  0.723  0.559  0.984  0.794  0.92  0.141  0.419  1.943  0.88  0.577  0.127  1.647  1.824  
 
 CHI3L2  1117  1  111770280  111786062  1p13.3  0  0  0  2.001  0  0  0.655  0  0.212  1.738  0.962  0.104  0  0.043  0  0  0  0  7.008  0.079  0  5.548  0.256  0.151  0  0  0.17  0.384  0  0.077  0  0.291  0  0  0  0  0  0.193  1.777  0  0  0  0  0  0  0  0.327  0  0  0.28  0.163  0.214  0  0.231  0  0  0  0  0  0  
 
 CHIAP2  149620  1  111823145  111828730  1p13.2  0  0.036  0.096  0  0  0  0  0  0.074  0  0  0.624  0  0.021  0  0  0  0.052  0  0.061  0  0.043  0.207  0  0  0  0.038  0.034  0  0.045  0  0  0  0.168  0  0  0  0  0.02  0  0  0.025  0.024  0  0  0  0.027  0  0  0  0  0.107  0  0.028  0  0  0  0  0  0  
 
 CHIA  27159  1  111833473  111863188  1p13.2  0  0  0.157  0  0  0  0  0  0  0  0  0.798  0  0  0  0  0  0  0  0  0  0.177  0  0  0  0  0.058  0.121  0  0.054  0  0  0  0.225  0  0  0  0  0.049  0  0  0.463  0.062  0  0  0  0  0  0  0  0  0.209  0  0  0  0  0  0  0  0  
 
 PIFO  128344  1  111889181  111895639  1p13.2  0  0.08  0.123  0.253  1.238  0.065  0.066  0  0.161  0.055  0.065  0.533  0  0.543  0  0.064  0  0.184  0.16  0.06  0  0.253  0.197  0  0  0.06  0.109  0.07  0  0.053  0.086  0  0  0.309  0  0  0  0.126  0.126  0.129  0  0.231  1.143  0  0  0.084  0.024  0  0  0.201  0  0.25  0  0.109  0.276  0  0  0  0.069  0.506  
 
 PGCP1  441897  1  111927140  111932473  1p13.2  0  0  0  0  0  0  0  0  0  0  0  0.299  0  0  0  0  0  0  0  0  0  0  0  0  0  0  0  0  0  0  0  0  0  0  0  0  0  0  0  0  0  0  0  0  0  0  0  0  0  0  0  0.415  0  0  0  0  0  0  0  0  
 
 OVGP1  5016  1  111956936  111970399  1p13  0.218  0  0.229  0.278  0.301  0.116  0.138  0.306  0.612  0.16  0.657  0.696  0.278  0.837  0.154  0.352  0.496  0.348  1.451  0.158  0  0.259  0.454  0.096  0.063  0  0.162  0.152  0.072  0.178  0.069  0.27  0.078  0.158  0.056  0.429  0.072  0.298  0.598  0.326  0.083  0.115  0  0.46  0  0.093  0.734  0.194  0.234  0.39  0.62  0.158  0.075  0.072  0.083  0  0.076  0  0.29  0  
 
 WDR77  79084  1  111982511  111991830  1p13.2  3.837  4.012  2.656  3.209  3.34  2.874  4.618  3.48  2.451  3.218  4.432  2.786  3.73  4.302  3.651  3.752  3.221  1.976  5.727  3.551  3.985  4.177  2.126  2.969  3.894  2.94  3.737  4.439  4.071  3.469  4.335  4.626  3.439  1.796  2.872  3.901  4.021  4.279  3.759  4.744  3.207  2.827  4.895  3.302  2.843  2.865  3.066  4.374  3.224  5.393  4.088  2.297  4.08  2.285  3.443  3.694  3.577  3.569  4.36  3.964  
 
 ATP5F1  515  1  111991742  112004525  1p13.2  6.165  8.208  3.794  4.237  5.476  3.982  6.232  5.005  4.141  5.029  5.925  4.685  5.136  6.76  4.612  4.917  5.548  8.287  6.913  6.515  5.652  5.507  3.719  3.835  5.139  4.231  5.253  5.063  5.521  4.536  5.88  6.073  4.741  3.588  3.69  5.264  4.125  5.195  5.79  5.127  4.503  3.989  6.055  4.689  4.06  4.191  5.04  5.118  4.097  6.505  5.312  3.095  5.136  3.595  4.988  4.714  4.833  4.866  5.257  5.079  
 
 C1orf162  128346  1  112016490  112021134  1p13.2  0.277  0.267  0  0  0.154  0  0.379  0  0.404  0.266  0.317  0  0  0.288  0  0.241  0.221  0  0.709  4.475  0  0.376  0  0.164  0  0  0.136  0  0  0.094  0.249  0  0  0  0  0  0.189  0.218  0.663  0  0  0.108  0  0  0  0.178  0  0  0  0.521  0.108  0  0.14  0  0.207  0  0.128  0  0  0  
 
 TMIGD3  57413  1  112025969  112106625  1p13.2  0  0.113  0  0  0  0  0  0  0  0  0  0  0  0  0  0  0  0  0  0.789  0  0  0  0  0  0.109  0.06  0.089  0  0.064  0.251  0  0  0.37  0  0  0  0  0.073  0  0  0  0.628  0  0  0  0.05  0  0  0  0  1.068  0  0  0  0  0  0  0  0  
 
 ADORA3  140  1  112042050  112046743  1p13.2  0  0  0.164  0  0  0  0  0  0.145  0  0.047  0  0  0  0  0  0  0  0  0.387  0  0.037  0.201  0  0  0  0.045  0  0  0.051  0  0  0  0.264  0  0  0  0  0.038  0  0  0.075  0  0  0  0  0  0  0  0  0  0.271  0  0.032  0  0  0  0  0  0  
 
 LINC01160  100129269  1  112141628  112150940  1p13.2  0  0.13  0.158  0  0  0  0.066  0.142  0.078  0.104  0.06  0  0  0  0  0  0  0  0  0  0  0.055  0.318  0.072  0  0.09  0.241  0.085  0  0.031  0  0  0  0.191  0  0  0  0.098  0  0  0  0  0  0  0  0  0.058  0  0  0.094  0.061  0  0.073  0  0.068  0  0.217  0  0.082  0  
 
 RAP1A  5906  1  112162404  112259317  1p13.3  2.162  4.816  1.45  1.34  1.699  1.306  2.21  2.292  1.757  2.49  2.693  1.011  1.597  2.602  1.193  1.691  1.932  3.507  3.121  2.27  1.524  1.287  1.26  0.807  1.496  1.677  1.092  2.093  1.753  0.344  2.133  2.426  1.351  1.07  0.638  1.933  1.091  2.084  2.385  1.759  1.514  0.846  2.181  1.371  1.109  1.327  0.457  1.577  1.698  3.291  2.449  0.653  1.425  1.015  1.536  1.598  2.083  1.475  2.053  2.126  
 
 FAM212B  55924  1  112264685  112298419  1p13.2  0.739  0.212  0.897  0.082  0.648  0.233  0.601  1.263  0.727  1.912  0.992  0.318  0.328  0.478  0.439  0.223  0.352  0.333  0.137  0.137  0.036  0.386  0.398  0.257  0.071  0.485  0.785  1.087  0.443  0.186  0.465  2.348  1.282  0.738  0.222  0.13  0.063  0.376  0.708  0.57  0.208  0.342  0.311  0.297  0.074  0.579  0.315  0.393  0.239  0.267  0.344  0.483  0.233  0.136  0.223  0.034  0.16  0.113  0.484  0.505  
 
 FAM212B-AS1  100506343  1  112282462  112290420  -  0  0.276  0.236  0  0.133  0.314  0.06  0  0.145  0.108  0.504  0  0  0.109  0  0  0.117  0.237  0  0  0  0.075  0  0  0  0  0.102  0  0  0.058  0  0.354  0.096  0.296  0.086  0.088  0.056  0  0.158  0.253  0  0.043  0.559  0  0  0.165  0.321  0.121  0  0.417  0.177  0.203  0  0  0.111  0  0.178  0  0.103  0.281  
 
 LOC101928718  101928718  1  112287938  112298131  1p13.2  0.248  0.053  0.088  0  0.092  0  0.066  0.05  0.14  0.149  0.113  0  0  0  0  0  0  0  0  0.046  0  0.223  0.25  0  0  0.068  0.116  0.157  0.072  0.026  0.058  0  0.077  0.119  0  0  0  0.066  0.057  0  0  0  0.055  0  0  0  0.026  0.107  0  0  0  0  0.034  0.046  0.03  0  0.05  0  0  0  
 
 DDX20  11218  1  112298189  112310199  1p21.1-p13.2  2.577  4.261  1.172  1.704  1.855  1.231  2.006  1.76  1.437  1.65  2.281  1.021  1.914  2.755  1.724  1.621  1.552  3.063  3.326  2.123  1.964  1.907  0.928  0.76  1.741  1.663  1.553  2.555  1.954  0.784  2.174  2.386  1.557  0.991  0.845  2.001  1.244  1.416  2.233  2.103  1.319  1.156  2.683  1.815  1.29  1.071  1.477  1.735  1.161  2.6  1.746  1.012  1.68  0.801  1.501  1.302  1.713  1.337  2.236  1.712  
 
 KCND3  3752  1  112318453  112531777  1p13.3  0.191  0.067  0.274  0  0.079  0.046  0.097  0.063  0.102  0.873  0.041  0.121  0  0.144  0  0.051  0.062  0.142  0  0.056  0  0.331  0.256  0  0  0  0.142  0.759  0  0.435  0.077  0.13  0  0.182  0  0  0  0  0.664  0.07  0  0.475  0.146  0.068  0  0.045  0.165  0.087  0.082  0  0.039  0.881  0.051  0.119  0.196  0.085  0.038  0  0  0  
 
 KCND3-IT1  100874295  1  112396383  112399527  -  0  0  0  0  0  0  0  0  0  0  0  0  0  0  0  0  0  0  0  0  0  0  0  0  0  0  0.112  0  0  0  0  0  0  0  0  0  0  0  0  0  0  0  0  0  0  0  0  0  0  0  0  0  0  0  0  0  0  0  0  0  
 
 KCND3-AS1  100873995  1  112452278  112453552  -  0  0  0  0  0  0  0  0  0  0  0  0  0  0  0  0  0  0  0  0  0  0.15  0  0  0  0  0.105  0  0  0  0  0  0  0  0  0  0  0  0  0  0  0  0  0  0  0  0  0  0  0  0  0  0  0  0  0  0  0  0  0  
 
 LOC643355  643355  1  112533189  112541463  1p13.2  0.304  0  0.117  0  0  0  0  0.026  0.091  0.098  0.026  0.039  0  0.019  0  0  0  0  0  0.055  0  0.091  0.291  0  0  0  0.039  0.046  0  0.043  0  0  0  0.143  0  0  0  0  0.456  0  0  0.115  0.035  0  0  0.03  0.035  0  0  0  0  0.243  0  0.039  0  0  0  0  0  0  
 
 CTTNBP2NL  55917  1  112938799  113003786  1p13.2  1.27  2.08  1.601  1.892  0.693  1.143  1.87  2.648  1.617  1.87  2.595  1.059  1.853  1.599  1.347  2.017  1.901  0.88  0.594  0.259  0.169  0.645  0.134  0  1.795  1.545  1.609  2.614  1.694  0.652  1.324  1.687  1.417  0.772  0.786  1.672  1.269  1.838  2.2  1.673  1.628  0.675  2.488  2.247  1.126  1.45  0.873  2.088  1.976  1.81  2.176  0.73  1.879  1.57  2.365  2.188  2.238  1.66  2.244  3.031  
 
 WNT2B  7482  1  113009162  113063910  1p13  1.324  0.283  0.315  1.55  0.125  0.111  0.278  0.516  0.342  0.294  0.322  0.109  0.197  0.625  0.246  0.236  0.172  0.249  0.065  0.092  0  0.329  0.256  0.06  0.079  0.233  0.568  0.237  0.198  0.223  0.141  0.361  0.149  0.249  0.13  0.433  0.112  0.488  0.318  0.621  0.269  0.821  0.992  0.497  0.394  0.349  0.509  0.153  0.982  0.304  0.173  0.845  0.528  0.787  1.74  1.33  0.222  0.23  1.939  0.943  
 
 ST7L  54879  1  113066140  113162040  1p13.2  0.994  2.152  0.576  1.224  0.974  0.77  1.216  1.036  0.856  1.043  1.06  0.359  0.844  1.185  0.886  0.93  1.136  1.756  1.233  0.586  0.661  0.991  0.397  0.302  0.711  0.523  0.91  0.96  0.828  0.787  1.139  1.549  0.805  0.672  0.591  0.916  0.795  1.135  1.262  1.203  0.765  0.801  1.574  0.598  0.629  0.778  1.405  1.059  0.882  1.476  1.021  0.854  0.825  0.653  1.621  1.106  0.793  0.712  1.282  0.685  
 
 CAPZA1  829  1  113162074  113214241  1p13.2  4.06  7.475  3.686  4.524  3.836  3.841  4.995  4.845  3.607  4.806  5.629  3.779  4.684  5.581  3.984  4.508  4.636  6.236  6.02  5.447  5.063  4.083  3.635  2.992  4.893  4.16  3.875  4.773  4.615  2.551  4.723  4.915  3.635  2.628  3.144  4.445  3.902  4.88  4.994  4.82  4.243  3.52  4.974  4.615  3.222  3.23  3.801  4.423  4.246  6.015  5.149  3.236  4.642  3.614  3.881  4.63  4.978  4.665  4.933  4.879  
 
 MOV10  4343  1  113216933  113243368  1p13.2  3.117  0.859  2.009  3.417  2.53  1.981  3.267  3.988  1.833  3.578  3.504  1.271  3.054  3.863  3.746  4.06  2.745  0.146  4.133  0.373  3.324  3.359  0.498  1.196  2.339  2.542  2.49  3.858  3.743  1.941  2.717  3.325  3.021  1.374  1.734  2.989  2.588  3.02  3.592  3.424  3.098  2.279  3.69  2.821  2.088  2.182  1.267  2.816  3.056  3.869  3.015  1.633  3.334  2.741  3.442  3.05  3.862  2.504  3.712  3.107  
 
 RHOC  389  1  113243748  113250025  1p13.1  4.455  2.991  6.895  7.33  6.697  5.336  7.619  6.542  6.085  7.301  7.015  4.428  5.612  5.483  4.226  5.898  5.614  0  5.335  0.778  5.506  3.304  1.604  1.433  6.505  5.167  6.754  7.081  6.489  4.512  5.491  7.363  5.574  4.587  5.137  6.258  5.263  7.051  6.747  5.762  6.284  5.634  6.412  5.498  4.72  5.82  3.927  6.174  6.211  7.352  7.194  4.535  6.55  6.134  6.48  6.695  6.211  6.637  7.254  6.492  
 
 PPM1J  333926  1  113252615  113257950  1p13.2  0.91  0.114  0.687  1.383  2.157  0.306  0.429  0.214  0.325  0.441  0.225  0  0.305  1.525  0.489  0.623  0.686  0.143  0.098  0.067  0.748  0.24  0  0  0.542  0.399  0.322  1.432  0.837  0.313  0.247  1.166  0.379  0.189  0.158  0.539  0.139  0.428  0.473  0.918  0.712  0.463  1.475  0.494  0.289  0.486  0.755  0.7  0.486  1.326  1.044  0.721  0.898  0.354  1.055  0.626  0.489  0.691  1.673  0.627  
 
 FAM19A3  284467  1  113263188  113269856  1p13.2  0  0.185  0.2  0.772  0  0.164  0.828  0.132  0  0.319  0  0  0  0.66  0  0  0  0  0.295  0  0  0.228  0.353  0  0  1.308  0.095  2.157  1.214  0.062  0.471  1.176  0  0  0.141  0.166  0.142  0  0.335  0  0.179  0.404  0.051  0  0  0.107  0  0  0  0.52  0.351  0.464  0.113  0.271  0.654  1.004  0.149  0  0  0  
 
 LOC100996702  100996702  1  113362790  113393265  1p13.2  0  1.397  0.46  0.628  0.332  0.27  0.293  0.083  0.499  0.419  0.243  0.083  1.164  0.14  0.429  0.4  0.85  0.21  0.172  0.088  0  0.341  0.511  0.296  0.198  0.317  0.349  0.589  1.021  0.227  0.826  0.195  0.237  0.267  0.057  0.129  0.465  0.478  0.547  0.376  1.013  0.623  1.076  0  0.088  0.629  0.096  1.523  0  2.051  0.66  0.711  0.539  0.252  0.876  0.327  0.209  1.067  0.336  0.383  
 
 SLC16A1  6566  1  113454469  113498975  1p12  1.322  0.454  3.272  3.633  0.177  2.991  2.838  2.953  5.306  3.885  4.691  0.614  3.752  4.211  3.458  2.634  4.157  3.482  4.387  3.732  3.744  3.152  2.022  2.5  3.143  2.139  1.918  3.468  3.826  1.328  3.243  4.084  2.62  1.924  1.889  3.017  2.704  3.184  4.073  3.334  1.967  2.673  3.446  3.658  2.922  2.533  1.403  3.813  2.863  4.624  4  1.699  3.41  2.319  2.763  3.326  3.532  2.479  3.465  3.639  
 
 AKR7A2P1  246182  1  113465971  113467295  1p12  0.317  0.157  0.524  0.285  0.384  0.468  0.608  0.39  0.367  0.746  0.848  0.376  0.471  0.414  0.53  0.584  0.473  0.136  0.308  0.206  0.221  0.459  0.668  0.405  0.18  0.31  1.254  0.845  1.234  0.635  0.441  0.646  0.19  0.54  0.348  0.265  0.451  0.521  0.227  0.393  0.101  1.046  1.326  0.346  0.355  0.285  0.233  0.979  0.188  0.3  0.504  0.866  0.746  0.387  0.433  0.418  0.932  0.297  0.66  0.316  
 
 LOC100506392  100506392  1  113499036  113506690  1p13-p12  0  0.39  1.142  1.25  0.124  1.475  0.939  1.159  0.539  1.113  1.01  0  1.101  1.131  0.876  0.722  0.961  0.979  1.751  0.241  1.58  1.233  0.497  0.544  0.812  0.72  0.881  1.612  1.431  0.432  1.201  0.921  1.037  0.468  0.432  0.737  0.571  0.975  0.878  0.557  0.223  0.642  1.31  0.354  0.257  0.97  0.543  1.704  0.362  1.962  1.072  0.914  1.193  0.757  1.317  1.096  1.329  1.642  1.571  1.042  
 
 LOC100996251  100996251  1  113554308  113615724  -  0  0.042  0.146  0.135  0.041  0.073  0.107  0.307  0.119  0.579  0.249  0.106  0.253  0.282  0.154  0.06  0.073  0.152  0.348  0.149  0.218  0.31  0.165  0  0.065  0.188  0.446  0.293  0.239  0.097  0.322  0.106  0.144  0.31  0.136  0.082  0.168  0.545  0.278  0.08  0.054  0.136  0.031  0  0  0.216  0.231  0.394  0  0.241  0.236  0.219  0.256  0.157  0.136  0.082  0.324  0.35  0.182  0.077  
 
 LRIG2  9860  1  113615791  113667824  1p13.1  1.213  1.397  0.692  0.922  0.81  0.731  1.113  1.354  0.96  1.195  1.329  0.331  1.009  0.962  1.235  0.985  0.936  0.368  1.306  0.633  1.207  0.94  0.444  0.369  0.59  0.783  0.819  1.591  1.232  0.67  1.203  1.163  0.652  0.351  0.444  1.09  0.742  1.382  0.755  1.46  0.85  0.566  1.391  1.064  0.714  0.646  0.425  1.423  1.103  1.501  1.507  0.687  1.069  0.492  0.803  0.747  1.263  0.695  1.108  0.883  
 
 LOC643441  643441  1  113739403  113748875  1p13.2  0  0  0.072  0  0.045  0  0  0  0.086  0  0  0  0  0  0  0  0  0  0  0  0  0.089  0.233  0  0  0  0.07  0  0  0.06  0  0  0  0.228  0  0  0  0  0  0  0  0.043  0.021  0  0  0  0.057  0  0  0  0  0.212  0  0  0  0  0  0  0  0  
 
 MAGI3  260425  1  113933474  114228545  1p12-p11.2  1.027  2.084  0.591  0.871  1.347  0.524  1.244  1.091  0.679  1.354  2.298  0.701  1.643  1.295  1.449  1.374  1.234  1.451  1.075  0.443  0.399  0.571  0.284  0.15  0.503  0.363  0.502  1.603  0.856  0.605  0.459  0.453  0.442  0.279  0.714  1.771  0.776  1.286  0.565  0.688  1.222  0.985  0.992  0.916  0.468  0.591  1.006  1.541  1.37  1.325  1.539  0.848  1.084  0.556  0.767  0.739  1.068  0.556  1.159  1.049  
 
 PHTF1  10745  1  114239823  114301777  1p13  1.642  3.992  0.806  1.237  0.803  0.771  1.814  1.871  1.152  1.931  2.881  0.658  0.886  1.693  0.853  1.181  0.67  2.903  1.161  0.949  0.892  0.147  0.778  0.462  0.683  1.526  1.593  1.78  1.805  1.302  1.797  1.863  1.056  0.681  0.526  1.834  1.325  1.393  1.853  1.155  0.575  0.91  2.234  0.759  0.656  0.743  1.311  1.22  1.12  2.06  1.866  0.717  1.173  0.688  1.092  1.118  2.015  0.603  0.877  1.841  
 
 RSBN1  54665  1  114304453  114355098  1p13.2  1.769  2.018  0.977  1.361  0.671  0.599  0.969  0.858  1.305  1.084  1.244  0.542  0.868  1.472  0.861  0.836  1.285  1.081  2.145  1.489  1.277  1.72  0.534  0.473  0.799  0.536  0.797  0.926  0.872  0.385  0.875  0.833  0.503  0.613  0.373  0.903  0.447  1.052  1.595  1.142  0.713  0.445  1.308  0.992  0.588  0.579  0.388  1.075  0.82  1.314  1.25  0.582  0.733  0.456  0.715  0.537  1.091  0.44  0.518  0.925  
 
 AP4B1-AS1  100287722  1  114355233  114443859  1p13.2  1.543  0.117  0.34  0.53  0.368  0.252  0.269  0.327  0.32  0.341  0.416  0  0.467  0.223  0.243  0.312  0.125  0  0.306  0.154  0.374  0.418  0.147  0.212  0.262  0.168  0.423  0.468  0.357  0.224  0.202  0.388  0.214  0.372  0.291  0.278  0.249  0.392  0.142  0.383  0.131  0.086  0.235  0.256  0  0.122  0.15  0.274  0.114  0.248  0.408  0.242  0.364  0.156  0.173  0.107  0.517  0.258  0.377  0  
 
 PTPN22  26191  1  114356432  114414381  1p13.2  0.3  0.907  0.107  0  0.036  0  0.204  0.299  0  0.072  0.026  0.22  0  0.031  0  0.058  0.052  1.414  2.894  1.807  0.128  1.644  0.371  0  1.965  0  0.1  0.143  0.122  0.081  0  0  0  0.088  0  0  0.042  0.93  0.044  0  0  0  0.016  0  0  0  0.061  0  0  0.218  0.043  0.19  0.15  0.039  0.028  0  0.323  0  0.089  0.229  
 
 BCL2L15  440603  1  114419435  114430169  1p13.2  0.362  0.086  0.122  0  0.053  0  0.085  0.05  0.099  0.053  0.072  1.636  1.386  0.141  0.254  0.741  0.518  1.307  0.149  0.113  0.056  0.152  0.19  0  0  0.017  0.156  0.025  0  0.092  0  0.043  0  0.149  0.179  0.536  0  0  0.052  0  0  0.02  0.022  0.512  0  0.128  0.173  0  0  0.015  0  0.087  0.524  0.094  0.041  0.069  0.174  0  0.169  0.044  
 
 AP4B1  10717  1  114436816  114447746  1p13.2  3.307  2.87  1.283  1.729  1.046  1.032  2.399  1.524  0.994  1.881  2.164  0.71  1.61  2.02  1.336  1.426  1.125  2.255  3.61  2.308  1.479  2.084  1.52  1.059  1.27  1.026  2.548  2.001  1.725  0.866  1.649  2.045  1.609  0.915  0.686  1.549  0.992  1.738  1.332  1.704  0.829  1.046  2.614  1.513  0.681  1.056  0.7  1.458  0.701  2.532  1.734  0.737  1.639  0.7  1.349  1.182  1.882  0.932  1.36  1.22  
 
 DCLRE1B  64858  1  114447914  114456708  1p13.2  3.758  3.083  1.415  2.004  1.554  1.894  2.243  2.032  1.18  2.25  2.443  1.115  1.941  1.997  1.58  1.565  1.854  1.497  2.94  1.78  2.29  2.422  0.866  1.195  1.901  1.426  1.784  2.147  1.906  1.253  1.826  1.934  1.861  1.053  1.07  1.963  1.509  2.181  1.872  2.34  1.272  1.354  2.593  1.885  1.011  1.362  0.708  2.534  1.883  2.601  2.234  1.214  2.286  1.363  1.629  1.489  2.326  1.876  2.415  1.469  
 
 HIPK1-AS1  101928846  1  114466622  114472147  -  0.352  0.252  0.184  0  0.222  0  0  0  0.168  0.119  0.109  0  0  0.119  0  0.113  0  1.225  0.1  0.117  0  0.126  0.713  0  0  0  0.165  0  0  0.14  0  0  0  0.186  0  0  0  0  0.072  0  0  0.188  0  0  0  0  0.076  0  0  0.432  0  0.217  0  0.1  0  0.239  0  0  0.208  0  
 
 HIPK1  204851  1  114471995  114520491  1p13.2  5.203  2.501  2.229  2.025  2.879  2.073  3.567  4.023  2.12  3.477  3.913  2.042  3.229  2.536  3.537  2.218  3.393  1.294  3.328  3.049  2.849  3.308  1.071  1.399  2.667  2.462  3.149  4.43  3.842  2.177  2.957  3.162  2.591  0.824  1.898  3.68  3.332  3.356  2.427  3.981  2.522  1.398  3.277  2.584  2.502  2.404  2.074  3.681  3.3  2.547  3.693  1.713  3.19  1.855  2.142  2.766  3.316  2.301  3.261  3.12  
 
 OLFML3  56944  1  114522012  114524876  1p13.2  0.256  0  4.147  0  5.501  1.081  0  5.788  0.244  0.67  0.643  1.652  0.121  0.12  0  0  0  0  0.085  0  0  0.144  0.321  0  0.323  1.801  0.252  2.948  0.103  0.066  0.516  3.943  0  0.15  2.872  0.109  0.063  0.36  1.687  2.13  0.125  0.499  0.827  0.176  0  0.614  0.826  1.462  0.353  2.434  0.084  0.505  0.154  1.743  0.085  0.162  1.144  0  0  0.348  
 
 SYT6  148281  1  114631913  114696472  1p13.2  0  0.045  0.174  0  0.026  0  0.044  0.023  0.112  0.034  0.037  0.032  0  0.05  0  0.063  0  0.066  0.024  0.043  0.044  0.127  0.221  0  0.036  0.042  0.204  0.21  0.027  0.077  0.133  0.281  0  0.222  0.66  0  0  0  0.073  0.28  0  0.208  0.064  0  0  0.033  0.055  0  0.069  0  0.044  0.392  0  0.089  0.054  0.052  0  0  0  0  
 
 TRIM33  51592  1  114935398  115053781  1p13.1  4.014  3.926  1.124  1.511  1.823  1.196  1.831  2.041  1.544  1.987  2.607  1.262  2.23  2.792  2.345  2.449  2.24  2.924  3.33  2.441  2.096  2.78  0.651  0.767  1.544  2.09  1.816  3.19  2.609  1.357  2.475  2.457  1.906  0.983  1.132  2.054  1.506  2.235  2.484  2.498  1.817  1.167  2.453  1.956  1.452  1.389  1.557  2.238  2.201  2.327  2.188  1.053  1.936  1.142  1.877  1.517  2.201  1.283  2.248  2.192  
 
 BCAS2  10286  1  115110180  115124265  1p13.2  5.398  6.511  2.425  2.648  3.422  3.182  3.653  3.321  4.037  3.477  4.289  2.247  2.637  4.272  1.9  3.156  3.432  6.316  4.896  3.903  3.391  3.672  2.102  1.874  3.186  2.266  2.949  2.999  3.43  1.346  3.56  3.611  2.302  1.819  2.172  3.121  2.305  2.8  4.395  2.968  2.68  3.195  4.316  3.096  2.495  2.642  3.375  3.306  2.297  4.941  3.54  1.682  3.06  2.476  3.237  2.918  3.059  2.867  3.335  3.294  
 
 DENND2C  163259  1  115127195  115212732  1p13.2  0.642  0.222  0.17  0  0.19  0.15  0.188  0.501  0.226  0.38  0.211  0.061  0.09  0.473  0.255  0.132  0.174  0  0.094  0.098  0.058  0.305  0.159  0.035  0.088  0.092  0.157  0.303  0.371  0.087  0.115  0.384  0  0.082  0.111  0.067  0.078  0.233  0.276  0.376  0.263  0.2  0.485  0.129  0.091  0.118  0.123  0.259  0.349  0.114  0.291  0.213  0.19  0.158  0.116  0.113  0.265  0.15  0.12  0.086  
 
 AMPD1  270  1  115215719  115238239  1p13  0.217  0  0.092  0  0.027  0  0  0.03  1.704  0  0  0  0  0.051  0  0  0  0  0  0.042  0  0.178  0.185  0  0  0  0.521  0  0  0.079  0  0  0  0.128  0.117  0  0  0  0.025  0  0.069  0.778  0.032  0.088  0  0.047  0.104  0  0  0.106  0.042  0.269  0.167  0.073  0  0  0  0  0  0  
 
 NRAS  4893  1  115247084  115259515  1p13.2  3.736  4.253  2.458  3.3  1.932  1.996  2.256  3.421  3.517  2.749  3.869  1.507  2.694  3.176  2.54  2.404  2.311  2.512  3.877  2.104  2.821  2.384  1.743  1.393  3.01  2.369  1.833  3.327  2.872  1.478  3.317  3.111  2.009  1.195  1.455  3.137  2.217  2.979  2.72  2.822  2.113  2.113  3.452  2.736  2.036  2.238  0.878  2.678  2.832  3.585  2.555  1.517  3.165  2.027  2.268  2.55  3.637  2.668  3.654  3.292  
 
 CSDE1  7812  1  115259533  115300671  1p22  7.446  6.414  4.947  3.431  4.846  4.57  5.712  5.617  6.078  5.813  6.13  3.499  4.912  6.264  4.971  4.752  5.156  4.38  6.633  5.394  4.867  5.566  3.717  3.388  4.785  4.395  4.647  5.939  5.559  3.817  5.475  5.764  4.201  3.125  3.578  5.587  4.822  5.254  5.686  6.018  4.964  4.305  6.296  4.944  4.569  4.283  4.05  5.287  5.112  6.618  5.774  3.703  5.012  3.631  4.76  4.983  5.354  4.76  5.363  5.562  
 
 SIKE1  80143  1  115312104  115323308  1p13.2  3.695  3.827  1.351  2.006  1.889  1.631  1.96  2.434  2.549  2.342  2.805  0.994  2.217  2.786  1.909  1.923  2.33  2.449  3.462  2.705  2.31  2.65  1.061  1.05  1.943  1.385  1.533  2.13  1.709  0.634  2.229  1.992  1.661  1.022  0.784  2.155  1.133  1.815  2.614  2.091  1.709  1.451  2.589  1.92  1.504  1.413  1.123  2.123  1.763  2.724  2.38  1.041  2.111  1.468  2.025  1.732  2.476  1.891  2.185  2  
 
 SYCP1  6847  1  115397423  115537990  1p13-p12  0.091  0.095  0.087  0  0.104  0.068  0.078  0.084  0.087  0.049  0.067  0.078  0.187  0.028  0.166  0.065  0.044  0.16  0.144  0.044  0.158  0.068  0  0.079  0.152  0.135  0.065  0.112  0.044  0.077  0.089  0.057  0.083  0.081  0.09  0.115  0.108  0  0.035  0.088  0.058  0.115  0.053  0.117  0.065  0.131  0.059  0.395  0.128  1.129  0.137  0.145  0.106  0.09  0.064  0.076  0.058  0.053  0.228  0.182  
 
 TSPAN2  10100  1  115590632  115632121  1p13.2  0  1.343  0.85  0  0.03  0.485  0.324  0.374  0.759  0.199  1.656  0  0.095  0.174  0  0.037  0  0.162  1.483  0.38  0  0.766  0  0  0  0  0.029  0.064  0  0.074  0  0  0  0.118  0.052  0  0.282  0.206  0.261  0.078  0.611  0.518  0.027  0.234  0.195  0.773  0.027  0.408  1.155  0.611  0.035  0.311  2.122  1.052  1.997  0.592  2.47  0  2.058  0.386  
 
 NGF  4803  1  115828536  115880857  1p13.1  0  1.394  4.339  4.011  0  4.777  0.838  0.423  0.853  1.092  0.403  0  0  0.047  0  0.086  0  0.559  0.206  0.185  0  0.467  0.237  0  0  0  0.104  0.607  0  0.099  0  0  0  0  0.131  0  0.092  0.206  4.243  0.371  0.448  0.529  0.06  0  0  0.273  0.081  1.397  0.349  1.298  1.979  1.996  0.766  0.175  1.829  0  2.36  0  1.224  0.826  
 
 VANGL1  81839  1  116184573  116240845  1p13.1  2.578  2.15  1.479  1.677  1.418  0.592  1.806  2.444  1.767  2.2  3.013  1.802  2.031  2.508  2.114  1.972  1.145  0.727  2.883  1.155  1.105  2.597  0.693  0.941  1.514  1.655  2.328  2.736  2.404  1.05  1.989  1.811  2.019  1.026  1.35  2.161  1.466  2.168  1.992  2.176  2.487  1.763  1.813  1.956  1.446  1.611  1.794  2.113  2.392  1.73  2.267  1.352  2.553  1.496  2.006  1.937  2.671  1.46  2.727  1.944  
 
 CASQ2  845  1  116242625  116311426  1p13.1  0  0.046  0.055  0  0.112  0  0  0  0.19  0.025  0.236  0  0  0.021  0  0  0  0  0  0  0  0.064  0.172  0  0  0.175  0.043  0.062  0.165  0.05  0.255  0.084  0  0.257  0  0  0  0  0  0  0.301  0  0.027  0  0  0.069  0.015  0  0  0  0  0.143  0.036  0.054  0  0  0  0  0  0  
 
 NHLH2  4808  1  116378998  116383747  1p12-p11  0  0.055  0.08  0  0.065  0.08  0.037  0.042  0.149  0.024  0.053  0.066  0.054  0.071  0  0.068  0.057  0.161  0  0  0  0.356  0.132  0  0  0  0.119  0.029  0  0.097  0  0  0  0.198  0  0  0  0  0.186  0  0  0.191  0.031  0  0.112  0.073  0.061  0  0  0  0  1.064  0.055  0.147  0.119  0.124  0  0.06  0  0  
 
 LOC101928995  101928995  1  116461996  116468529  1p13.1  0  0  0  0  0  0  0  0  0  0  0  0  0  0  0  0  0  0  0  0  0  0  0.256  0  0  0  0.027  0  0  0  0  0  0  0  0  0  0  0  0  0  0  0  0  0  0  0  0  0  0  0  0  0  0  0  0  0  0  0  0  0  
 
 LOC101928977  101928977  1  116465193  116519897  1p13.1  0  0  0  0  0  0  0  0  0  0  0  0  0  0.119  0  0  0  0  0  0  0  0  0  0  0  0  0.272  0.118  0  0  0  0  0  0  0  0  0  0  0.259  0  0  0  0  0  0  0  0  0  0  0  0  0  0  0  0.116  0  0  0  0  0  
 
 SLC22A15  55356  1  116519118  116612675  1p13.1  0.061  0.349  0.128  0  0.111  0.445  0.02  0.357  0.457  0.329  0.328  0.052  0.338  0.442  0.321  0.019  0  0.348  0.031  0.636  0  0.064  0.154  0  0  0.565  0.562  0.227  0.874  0.163  0.556  0.296  0.296  0.38  0.068  0.112  0.056  0  0.465  0.046  0.206  0.375  0.019  0.121  0.207  0.128  0.023  0.56  0.289  0.748  0.074  0.302  0.189  0.268  0.162  0.094  0.833  0  0.509  0.04  
 
 MAB21L3  126868  1  116654375  116677861  1p13.1  0  0  0.136  0  0.022  0  0  0.029  0.156  0.043  0.054  0.25  0  0.062  0  0  0  0.106  0  0  0  0.045  0.157  0  0  0  0.077  0.068  0.075  0.034  0.04  0.08  0  0.192  0  0  0  0  0.033  0  0.053  0.169  0.022  0.352  0.694  0.486  0.019  0.065  0  0.043  0.092  0.182  0.697  0.079  0.079  0.051  0.186  0  0.175  0.073  
 
 ATP1A1  476  1  116915794  116947396  1p21  4.989  4.515  3.785  4.409  4.275  3.683  5.536  5.131  8.431  6.301  6.58  5.186  5.555  4.874  5.256  4.488  6.29  2.628  4.91  4.492  5.25  4.021  3.549  4.054  4.77  6.234  7.243  6.162  7.236  7.172  7.27  6.738  6.677  5.05  3.18  4.695  5.745  5.577  4.404  4.796  5.047  4.517  4.254  4.513  5.367  4.765  4.972  4.837  4.68  5.595  5.589  3.542  4.49  4.101  4.165  4.84  5.1  3.281  4.509  5.353  
 
 ATP1A1-AS1  84852  1  116935486  116961244  1p13.1  4.041  2.274  3.041  4.353  2.358  1.9  3.695  2.401  8.094  4.239  4.18  3.195  3.191  2.981  3.478  3.097  4.401  1.647  2.453  3.392  2.109  2.763  3.338  2.646  2.073  3.771  6.469  4.059  5.923  5.968  4.462  3.799  3.981  4.832  1.989  2.11  3.53  3.101  3.183  2.694  2.443  3.333  3.256  1.574  2.559  1.993  2.699  2.796  1.606  2.38  3.117  1.657  2.929  2.375  3.026  2.734  2.74  0.709  2.161  1.949  
 
 LOC101929023  101929023  1  116966345  117021448  1p13.1  1.02  0.328  0  0  1.004  0  0.475  0  1.197  0.688  0.783  0.304  0.438  0.486  0  0.825  0.436  0  0  0  0  0.212  0  0  0  0.891  0.566  1.552  1.876  0.475  0.38  0  0  0  0.676  0.476  0  0  0.956  0  0  0  0.474  0  0  0  0  0  0  0  0  0  0  0.634  0.307  0.468  0.812  0  0.456  0  
 
 CD58  965  1  117057155  117113715  1p13  1.841  5.267  1.594  1.619  1.835  2.062  3.133  3.265  2.987  3.653  3.831  2.073  2.156  3.193  1.547  2.443  2.616  5.04  3.731  1.525  2.238  2.31  0.924  0.825  2.001  2.89  2.867  2.651  2.746  1.702  3.224  3.43  2.198  1.567  1.229  3.078  1.569  2.854  3.005  1.849  2.318  2.294  2.858  1.209  2.328  1.315  2.474  1.698  1.741  2.653  2.069  0.965  2.744  1.806  2.259  2.427  3.061  1.404  3.472  2.472  
 
 IGSF3  3321  1  117117019  117210377  1p13  3.059  0.301  1.037  0.215  2.913  0.211  1.597  2.122  3.386  2.406  3.142  1.592  1.445  1.627  1.365  1.387  1.696  0.33  0.09  0.059  1.366  0.23  0.243  0  0.02  3.664  2.726  3.951  3.434  1.52  3.276  3.369  2.519  1.157  1.253  2.515  0.814  0.165  0.784  2.02  1.718  0.861  0.912  1.677  1.563  0.717  0.485  1.283  1.971  1.336  0.113  1.209  2.677  1.435  1.61  2.184  2.453  0  2.983  2.218  
 
 C1orf137  388667  1  117236733  117249225  1p13.1  0  0  0  0  0  0  0  0  0  0  0  0  0  0  0  0  0  0  0  0  0  0.199  0  0  0  0  0  0  0  0  0  0  0  0  0  0  0  0  0  0  0  0  0  0  0  0  0  0  0  0  0  0  0  0  0  0  0  0  0  0  
 
 CD2  914  1  117297085  117311851  1p13.1  0  0  0.129  0  0  0  0.291  0  0.109  0.151  0  0  0  0.039  0  0  0  0  2.588  0.081  0  5.424  0  0  0  0  0.065  0  0  0.036  0  0  0  0.253  0  0  0  0  0  0  0  0  0  0  0  0  0.057  0  0  0  0  0.306  0  0.046  0  0  0  0  0  0  
 
 PTGFRN  5738  1  117452544  117532980  1p13.1  2.517  0.045  3.992  0.197  3.381  1.842  3.02  4.242  2.961  4.739  4.033  1.811  2.808  3.239  3.118  1.853  2.231  1.671  0.019  1.008  1.785  0.359  0.23  0  0  3.177  3.605  0.216  3.741  1.916  2.836  4.42  3.135  1.591  1.34  3.52  0.929  0  1.635  1.222  2.313  2.246  1.206  2.64  2.032  1.962  1.11  0.723  1.792  1.412  0.167  1.659  3.512  2.99  2.08  3.424  3.855  0  2.255  3.914  
 
 CD101  9398  1  117544371  117579173  1p13  0.333  0.045  0.296  0  0.345  0.156  0.293  0.311  0.313  0.461  0.581  0.119  0.132  0.417  0.246  0.045  0.143  0.207  0.086  0.404  0.099  0.187  0.259  0  0  0.085  0.361  0.06  0.092  0.088  0.105  0.595  0.296  0.27  0  0.586  0  0  0.133  0.078  0.221  0.289  0.145  0.096  0.111  0.171  0.014  0.056  0.057  0.044  0.043  0.338  0.152  0.261  0.058  0  0.591  0  0.146  0.081  
 
 LOC101929099  101929099  1  117568103  117602112  -  0.985  0.061  0.202  0  0.359  0  0.15  0.095  0.195  0.144  0.287  0.29  0.329  0.475  0.173  0.157  0.283  0.178  0.804  0  0  0.261  0.348  0.096  0  0.063  0.167  0.052  0.108  0.288  0.097  0.139  0.189  0.224  0.128  0.265  0  0  0.09  0.247  0.381  0.086  0.024  0  0  0.149  0  0  0.306  0.043  0  0.313  0.086  0.078  0.138  0.082  0.192  0  0.16  0.087  
 
 TTF2  8458  1  117602948  117645491  1p22  3.161  2.89  1.25  1.471  1.684  1.713  2.354  1.743  1.113  2.189  2.92  1.116  2.26  2.699  2.59  2.202  2.004  2.784  4.087  2.997  2.191  2.766  1.163  1.286  1.38  1.507  2.52  3.072  2.641  2.741  2.552  2.591  2.513  1.126  1.117  2.283  1.733  1.862  1.975  1.148  1.607  1.728  2.459  1.935  1.251  1.83  1.301  1.789  2.196  2.258  2.411  1.529  1.986  1.201  2.346  1.291  2.339  1.377  2.543  2.503  
 
 TRIM45  80263  1  117653676  117664411  1p13.1  1.927  0.955  0.511  1.054  1.206  0.752  0.812  0.825  1.449  1.455  1.823  0.189  0.876  0.89  0.741  0.472  0.291  0.393  0.309  0.494  0.687  0.491  0.409  0.102  0.45  0.808  0.941  1.057  0.992  1.544  1.012  0.874  0.633  0.359  0.418  1.352  0.477  0.633  0.676  1.038  0.835  0.784  1.835  1.222  0.756  0.705  0.731  0.9  0.581  1.307  1.004  0.832  0.543  1.003  0.762  0.662  0.692  0.367  0.936  0.473  
 
 VTCN1  79679  1  117686208  117753582  1p13.1  0.213  0  0.176  0  2.644  0  0  0  0.199  0.038  0.185  0.405  0  0.066  0  0  0  0  0  0  0  0.041  0.179  0  0  0  0.134  0.034  0  0.211  0  0  0  0.257  0  0.532  0  0  0.035  0  2.109  0.228  0  0.509  1.352  4.012  0  0  0  0  0.082  0.206  0  0.348  0  0.111  0  0  0.174  0  
 
 MAN1A2  10905  1  117910084  118068320  1p13  2.499  3.074  1.557  1.504  2.054  1.809  2.374  2.843  2.835  2.588  2.868  1.329  2.115  2.762  2.091  1.679  1.889  3.179  1.656  1.139  1.943  1.54  1.175  1.249  1.675  1.948  1.389  2.844  2.225  1.269  2.284  2.549  1.811  1.067  1.193  2.682  1.517  1.844  1.742  2.339  2.411  1.344  2.322  2.258  1.898  1.701  0.561  2.149  2.097  1.672  2.439  1.214  1.679  1.131  1.133  1.643  2.105  1.552  1.735  2.136  
 
 FAM46C  54855  1  118148603  118171011  1p12  0.529  1.599  0.323  0.285  0.335  0  0.763  0.091  0.328  1.342  0.737  0.289  0.048  0.315  0  0.031  0.248  0.12  2.794  1.969  0.976  0.251  3.35  3.253  0  0.036  0.233  1.079  0.373  0.306  0.423  1.063  0.172  0.448  0.027  0.07  0.141  0.19  0.121  0.062  0.079  0.377  0.033  0.042  0.208  0.125  0.113  0.072  0  0.195  1.29  0.251  0.018  0.104  0.025  0.043  0.057  0  0  0  
 
 GDAP2  54834  1  118406106  118472302  1p12  1.154  2.245  0.857  0.712  1.124  1.045  1.404  1.731  1.264  1.512  2.159  0.429  1.429  1.601  1.121  1.21  1.054  1.359  1.628  0.91  1.052  1.08  0.561  0.408  1.089  1.219  1.177  2.543  1.956  0.815  1.899  1.367  1.549  0.877  0.73  1.72  1.008  1.086  1.363  1.52  1.164  0.877  1.571  0.886  0.955  0.967  0.813  1.251  0.947  1.322  1.797  0.846  1.156  0.718  0.972  1.028  1.567  1.047  1.656  1.262  
 
 WDR3  10885  1  118472371  118503049  1p12  2.318  3.55  1.514  1.941  1.487  1.605  2.245  1.691  2.212  1.831  3.007  1.127  1.873  3.297  2.016  1.855  1.797  2.704  3.73  2.811  2.912  1.987  1.514  1.59  2.343  1.926  1.828  4.004  3.022  1.499  2.676  3.394  2.796  1.972  1.57  2.458  2.042  1.627  2.37  3.096  1.638  1.439  2.362  2.73  2.289  1.416  0.809  1.949  1.758  2.99  2.589  1.178  2.038  1.277  1.604  1.964  1.996  2.119  2.293  2.165  
 
 SPAG17  200162  1  118496287  118727848  1p12  0.044  0.019  0.071  0  0.044  0  0.071  0.022  0.093  0.469  1.336  0.024  0  0.027  0  0.017  0.031  0.048  0.025  0.065  0.049  0.082  0.076  0.016  0.024  0.015  0.062  0.055  0.025  0.126  0.024  0  0.032  0.095  0.024  0.013  0.013  0.043  0.021  0.022  0.015  0.189  0.025  0.034  0.085  0.032  0.034  0.035  0.019  0.092  0.025  0.07  0.027  0.091  0.064  0.022  0.032  0  0.181  0.08  
 
 TBX15  6913  1  119425665  119532179  1p11.1  0.062  0.061  0.911  1.452  0  0.38  1.864  2.363  0.752  2.429  1.05  0.162  0  0.055  0  0  0.086  0.079  0.038  0.085  0.529  0.081  0.204  0.03  0  0  0.089  0.13  0  0.066  0.259  0.088  0  0.165  0  0.038  0  0.606  0.05  0  0  0.273  0.032  0  0.216  0.047  0.073  0.324  2.029  0.205  0.295  0.151  1.729  0.235  1.087  0.261  2.407  0  1.949  0.615  
 
 WARS2  10352  1  119573838  119683295  1p12  1.681  2.648  1.116  1.319  1.197  1.006  1.164  1.453  3.163  1.434  2.086  0.944  1.613  1.424  1.366  1.581  1.456  3.323  2.098  1.532  1.573  1.183  0.777  0.692  1.15  1.108  1.599  1.826  1.849  1.189  1.988  1.757  1.335  0.868  0.841  1.172  0.785  0.945  1.428  1.373  1.109  1.774  1.929  1.232  1.258  1.425  1.192  1.978  1.91  2.476  1.583  1.001  1.705  0.6  2.018  1.354  1.827  0.933  2.153  1.556  
 
 WARS2-IT1  104472716  1  119590027  119605910  1p12  0  0  0  0  0  0  0  0  0.708  0.168  0  0  0  0  0  0  0  0  0  0  0  0  0  0  0  0  0  0  0  0.508  0  0  0  0.336  0  0  0  0  0  0  0  0  0  0  0  0  0  0  0  0  0  0  0.171  0  0  0  0  0  0  0  
 
 LOC101929147  101929147  1  119683018  119818596  1p12  0.619  1.878  0.567  0.979  0.787  0.44  1.025  1.236  3.488  1.449  2.177  0.425  1.604  1.173  1.347  1.692  0.707  2.606  1.656  1.016  1.156  1.168  0.682  0.493  1.102  0.861  1.108  1.891  1.436  0.765  1.176  1.604  0.22  0.502  0.627  0.633  1.107  1.057  1.1  1.148  1.48  1.686  2.111  0.525  1.419  0.625  0.651  1.992  1.049  2.709  1.228  1.296  1.32  0.508  1.731  0.635  1.248  0.824  2.081  0.869  
 
 HAO2  51179  1  119911398  119936753  1p13.3-p13.1  0  0  0.105  0  0  0  0  0  0.463  0.041  0  0  0  0  0  0  0  0.086  0  0  0  0.082  0  0  0  0  0.025  0  0  0.079  0  0  0  0.197  0  0  0  0  0.029  0  0  0.256  0.039  0  0  0  0  0  0  0  0  0.142  0  0.057  0  0  0  0  0  0  
 
 HSD3B2  3284  1  119957553  119965662  1p13.1  0  0  0.201  0  0  0  0  0  0.554  0  0  0  0  0  0  0  0  0.085  0  0  0  0.068  0.233  0  0  0  0.066  0.051  0.124  0.044  0.281  0.073  0  0.203  0  0  0  0  0.054  0  0  0.515  0.029  0  0  0  0  0  0  0  0  0.302  0  0.037  0  0  0  0  0  0  
 
 HSD3B1  3283  1  120049825  120057681  1p13.1  0  0.076  0.147  0  0  0  0  0  0.577  0  0  0.151  0  0.041  0.18  0.108  0  0  0  0  0  0.101  0.3  0  0  0  0.147  0.067  0.12  0.028  0  0  0  0.215  0  0.376  0.098  0  0.419  0  0  0.803  0.028  0  0  0  0  0  0  0  0  0.277  0  0  0  0  0  0  0  0  
 
 HSD3BP4  128102  1  120106502  120115199  1p13.1  0  0.011  0.152  0  0.009  0.012  0.03  0.033  0.535  0.032  0.018  0.019  0  0.018  0  0  0  0.061  0.011  0  0  0.044  0.189  0.014  0  0.097  0.441  0.407  0.499  0.135  0.182  0.193  0.223  0.257  0.018  0.023  0.018  0.428  0.04  0  0.057  0.315  0.026  0.015  0  0.036  0.021  0  0  0  0.016  0.112  0.012  0.034  0.017  0.017  0.21  0  0  0  
 
 LINC00622  644242  1  120140324  120141914  1p12  0  0.382  0.31  0  0  0  1.362  0.461  0.549  0.346  0.348  0.176  0  0.207  0  0  0  1.063  0  0  0  0  0.441  0  0.076  0.829  2.379  1.713  2.377  1.151  1.98  1.86  1.667  0.781  0  0.167  0.059  0.873  0.146  0  0.109  0.676  0  0  0  0  0  0.095  0  0.099  0.126  0  0  0  0.046  0  0.764  0  0  0  
 
 ZNF697  90874  1  120161999  120190390  1p12  0.316  1.704  0.717  0.361  0.202  0.409  1.301  1.977  2.012  0.701  1.631  0.461  0.37  0.663  0.243  0.248  0.597  1.924  0.146  0.373  0.831  0.266  0.457  0.078  1.323  2.356  2.023  3.479  3.502  1.918  2.658  2.942  2.064  1.146  0.207  0.369  1.17  1.148  0.83  0.713  0.442  0.756  1.498  0.431  0.423  0.275  0.186  0.58  0.087  0.6  1.063  0.783  1.146  0.377  0.571  0.415  1.325  0.376  1.245  0.525  
 
 PHGDH  26227  1  120254418  120286849  1p12  4.429  0.476  5.633  5.525  3.916  4.032  4.914  5.606  8.741  4.977  5.81  3.608  3.195  5.291  4.594  2.858  6.053  3.59  7.199  4.921  3.247  5.493  4.301  1.439  0  3.249  5.689  4.56  5.799  4.365  5.927  5.285  5.178  4.038  3.409  0.077  3.943  3.746  5.356  0.596  3.757  5.48  5.07  3.361  5.256  5.535  4.213  4.639  0.954  5.857  4.519  3.221  0  3.022  4.121  4.277  4.423  0.339  4.752  3.356  
 
 HMGCS2  3158  1  120290618  120311555  1p13-p12  0  0  0.121  0  0.79  0  0  0  0.472  0  0  5.528  0  0.029  0  0.23  3.023  0  0  0.043  0  0.028  0.351  0  0  0  0.059  0.033  0  0.051  0  0  0  0.128  0  0  0  0  0.044  0  0  0.193  0.026  0  0  0  0  0  0  0  0  0.174  0  0  0  0  0  0  0  0  
 
 REG4  83998  1  120336640  120354203  1p13.1-p12  0  0  0.18  0  0.109  0  0.126  0.163  0.715  0.245  0  0.197  1.051  0.048  0  0.203  7.816  0  1.662  0  0  0.656  0.649  0  0  0.19  0.104  0.398  0  0.128  0  0  0  0.306  0  0  0  0  0.053  0  0  0.074  0.079  0  0  0  0  0  0  0  6.542  0.433  0  0.108  0  0  0  0  0  0  
 
 NBPF7  343505  1  120377387  120387503  1p12  0  0  0.129  0  0  0  0  0  0.426  0.061  0  0  0  0.029  0  0  0  0  0  0  0  0.054  0.214  0  0  0  0.031  0.052  0  0.043  0  0  0  0.156  0  0  0  0  0.032  0  0  0.223  0.037  0  0  0  0  0  0  0  0.14  0.119  0  0.058  0  0  0  0  0  0  
 
 ADAM30  11085  1  120436155  120439147  1p12  0.068  0.025  0.157  0  0.019  0  0.067  0.049  0.451  0.207  0.07  0.06  0.055  0.031  0  0.041  0.23  0.082  0.128  0  0.064  0.078  0.261  0  0  0  0.126  0.085  0  0.1  0.051  0  0  0.264  0.058  0  0.047  0  0.042  0  0  0.345  0.048  0.059  0  0.037  0  0.058  0  0  0.056  0.23  0.058  0.203  0.041  0.139  0  0  0  0  
 
 NOTCH2  4853  1  120454175  120612317  1p13-p11  3.755  2.601  3.614  2.6  3.038  3.014  3.814  4.343  4.007  4.243  3.646  1.43  2.532  2.987  3.699  1.943  2.442  0.115  1.341  2.173  0.248  2.308  1.926  3.259  3.725  3.935  3.816  5.473  5.193  3.004  3.112  3.65  3.278  1.55  1.838  3.133  4.067  4.031  3.599  4.375  2.701  2.558  3.714  3.069  3.741  3.719  1.703  4.463  3.072  3.183  3.614  1.463  4.138  3.122  3.167  3.98  4.374  2.708  4.524  4.648  
 
 FAM72B  653820  1  120839004  120855681  1p11.2  1.566  2.636  0.892  1.431  1.238  1.151  1.911  1.638  0.843  1.716  2.246  0.699  1.138  2.079  0.491  0.847  1.616  1.822  3.652  1.903  2.203  2.025  1.891  1.427  1.599  1.078  1.057  1.588  1.11  0.921  1.616  1.918  0.83  0.937  1.134  1.601  2.371  0.937  1.511  1.87  1.707  2.217  1.79  1.098  0.986  1.174  1.383  1.009  1.338  1.919  2.42  1.737  1.365  0.66  1.72  1.601  0.779  1.541  1.411  1.315  
 
 HIST2H2BA  337875  1  120906033  120914842  1p12-p11.2  5.572  3.417  2.61  1.706  3.094  4.793  3.296  2.706  1.767  3.096  2.74  3.971  1.444  4.047  2.829  3.717  4.415  1.398  1.817  3.399  2.503  5.389  1.214  3.269  2.664  1.45  3.73  2.507  2.845  1.078  2.979  1.803  2.363  1.174  4.924  3.188  2.405  2.064  2.628  2.841  3.137  5.09  2.794  2.719  3.089  2.794  1.515  2.602  2.439  0.705  2.047  4.122  3.081  3.469  3.533  2.383  2.19  3.04  1.824  1.919  
 
 FCGR1B  2210  1  120926127  120935944  1p11.2  0.407  0  0.184  0  0.316  0  0  0  0.704  0.062  0.1  0  0  0  0  0  0  0  0  1.772  0  0  0.898  0  0  0  0.041  0  0  0.028  0  0  0  0.344  0  0.124  0  0  0.086  0  0  0.488  0.227  0  0  0  0.042  0  0  0  0.066  0  0  0  0  0  0  0  0  0  
 
 SRGAP2-AS1  100873165  1  121102016  121139765  1q32.1  0.32  0.161  0.236  0.109  0.206  0.146  0.271  0.295  0.367  0.429  0.239  0  0.141  0.176  0.132  0.1  0.164  0  0  0.204  0.159  0.162  0.566  0.095  0  0.383  0.541  0.525  0.554  0.289  0.418  0.338  0.227  0.361  0.157  0.108  0.474  0.165  0.251  0.094  0.136  0.643  0.111  0.083  0  0.136  0.233  0.219  0.246  0.095  0.136  0.109  0.201  0.05  0.195  0.245  0.316  0.084  0.184  0.098  
 
 SRGAP2B  647135  1  121107151  121134685  1q21.1  1.982  2.929  1.585  1.663  1.454  1.749  2.018  3.067  3.225  3.036  2.467  0.887  0.742  1.782  0.513  0.736  1.332  0.943  1.217  1.353  0.791  0.831  1.244  0.844  1.936  2.953  2.278  2.864  1.984  1.368  2.451  2.204  2.142  1.63  1.188  2.086  3.025  1.674  2.972  2.271  1.057  1.832  2.137  1.683  0.933  1.881  1.035  1.884  1.821  1.911  1.874  1.407  2.018  0.797  2.057  2.092  2.519  1.615  1.978  1.812  
 
 EMBP1  647121  1  121260909  121313686  1p11.2  0.336  0.071  0.245  0.154  0.027  0  0.172  0.117  0.184  0  0  0  0  0.319  0  0  0.074  0.363  1.8  0.881  0  1.116  0.903  0.623  0.193  0.09  0.034  0.29  0.464  0.086  0.113  0.126  0  0.106  0.069  0.423  0.257  0  0.294  0.223  0.392  0.321  0.702  0  0.087  0.177  0.081  0.296  0.19  0.481  0.259  0.388  0.021  0.039  0.297  0.158  0  0  0.214  0.231  
 
 ANKRD20A12P  100874392  1  142697420  142713605  -  0.218  0  0.224  0  0.38  0  0.105  0.055  0.153  0.136  0.239  0  0  0.049  0.081  0  0  0.313  0  0  0.349  0.078  0.216  0.083  0.844  0.484  0.19  0.137  0.239  0.065  0.389  0.668  0.176  0.325  0.15  0  0.119  0.554  0.333  0  0.093  0.083  0.027  0  0  0.175  0.072  0  0  0  0.063  0.197  0.063  0.395  0.067  0.168  0  0  0  0.46  
 
 LOC102723769  102723769  1  143134602  143202239  22p11  0.537  0.059  0.632  0  0.894  0  0.18  0.084  0.414  0.318  0.578  0  0.239  0.127  0.288  0.217  0  0  0  0  0.98  0.254  1.077  0.502  1.058  1.06  0.236  0.753  0.559  0.839  0.484  1.078  1.001  0.685  0.2  0.282  0.607  0.804  0.835  0  0.539  0.443  0  0  0  0.404  0  0.11  0  0  0.264  0.342  0.094  0.173  0.383  0.123  0.08  0  0  0.483  
 
 LOC100132057  100132057  1  143687126  143717173  1q21.2  0  0  0.128  0  0.055  0  0.074  0.206  0.092  0.134  0.08  0.197  0  0.117  0  0.129  0  0.29  0.331  0.145  0  0.245  0  0  0  0.066  0.11  0.068  0  0.094  0  0.116  0.2  0.118  0  0.144  0.131  0.14  0.046  0.11  0.097  0  0  0  0  0.06  0.123  0  0  0  0.076  0  0.061  0.073  0.077  0  0.082  0  0  0  
 
 LINC01138  388685  1  143717587  143744519  1q21.1  0  1.178  0.795  0  1.298  0.398  1.951  1.638  0.89  1.121  1.151  0.926  0.625  1.333  0.675  1.88  0.608  1.798  1.681  2.084  0.318  0.722  1.254  0.811  1.279  0.546  1.266  0.699  1.248  1.097  0.823  2.08  1.79  1.275  0.226  1.567  1.175  1.166  0.921  0.696  1.139  0.687  0.832  0.821  0.851  1.22  1.646  0.441  0.951  1.124  0.932  0.698  0.388  0.764  0.458  0.11  1.289  0.112  0.305  0.509  
 
 PPIAL4G  644591  1  143767143  143767881  1q21.1  3.071  1.608  2.551  2.892  2.945  2.026  3.614  2.241  2.575  2.287  2.411  1.86  2.343  2.863  1.694  2.83  2.49  0.659  3.219  3.412  0.553  3.613  3.55  2.284  1.997  1.138  2.542  1.748  1.87  2.543  2.022  2.445  1.602  1.581  1.929  1.37  1.681  2.039  3.254  1.902  1.551  2.697  3.131  0.92  1.066  1.141  4.278  1.692  0.439  2.463  2.05  1.201  2.509  1.935  2.902  1.472  1.505  2.818  1.937  1.619  
 
 FAM72D  728833  1  143896451  143913143  1q21.1  0  2.496  0.588  1.316  0.77  0.535  0.313  0.749  0  1.207  1.093  0  0  1.105  0.198  0.436  1.069  1.91  2.755  1.765  1.736  1.622  1.325  0.728  1.232  0.831  0.305  0  0  0  1.406  0.901  0.388  0.93  0.174  0.179  0.715  0  1.524  0.9  0  1.837  0.286  0.734  0  1.036  0.2  0.452  1.34  0.628  1.22  0.97  0.186  0.408  1.612  0.448  0.242  0  0.668  0  
 
 NBPF20  100288142  1  144146810  146467744  1q21.2  0.112  0.02  0.056  0  0.056  0.025  0.038  0.108  0.015  0.059  0.049  0.03  0.037  0.055  0.04  0.019  0.018  0  0.016  0.009  0  0.027  0.024  0.031  0.055  0.047  0.085  0.09  0.073  0.033  0.053  0.035  0.055  0.027  0.028  0.054  0.128  0.079  0.049  0.085  0.03  0.026  0.162  0.031  0.017  0.021  0.019  0.086  0.043  0.026  0.124  0.032  0.074  0.023  0.05  0.048  0.064  0.067  0.067  0.023  
 
 LINC00869  57234  1  144300467  144341077  1q21.1  0.975  0.14  0.488  0.432  0.314  0.261  0.347  0.224  0.559  0.217  0.319  0.274  0.312  0.376  0.445  0.633  0.472  0.123  0.305  0.219  0.089  0.315  0.566  0.364  0.399  0.245  0.117  0.359  0.405  0.361  0.255  0.241  0.235  0.518  0.141  0.337  0.498  0.189  0.275  0.34  0.248  0.477  0.351  0.168  0.142  0.164  0.089  0.283  0.141  0.226  0.282  0.206  0.427  0.278  0.156  0.311  0.257  0.096  0.181  0.112  
 
 PFN1P2  767846  1  144610814  144612719  1q21  0.794  0.569  0.577  0.623  0.608  0.676  0.77  1.103  0.608  1.26  0.846  0.403  0.721  0.643  0.527  0.551  0.512  0.245  0.745  1.188  0.223  0.601  0.424  0.256  0.695  1.019  1.204  1.956  1.13  0.75  0.7  0.601  0.649  0.357  1.033  0.963  1.469  1.001  1.065  0.528  0.297  0.773  1.344  0.542  0.278  0.56  1.381  1.132  0.656  0.51  1.215  0.453  1.35  0.598  1.352  0.681  0.835  0.758  1.058  0.482  
 
 NBPF9  400818  1  144614958  145370304  1q21.1  1.98  1.703  0.97  1.208  2.26  1.017  1.896  1.787  1.019  1.856  1.983  0.505  1.269  1.198  1.081  0.964  0.84  1.032  2.253  0.324  0.481  0.712  1.175  1.236  1.626  1.796  1.562  1.41  1.311  1.607  1.779  1.77  1.739  0.642  0.624  2.049  2.084  2.393  1.21  1.81  1.252  0.936  3.235  1.445  0.824  1.095  0.268  1.173  0.903  2.736  2.295  1.295  1.153  0.728  1.451  1.406  1.812  1.143  1.768  1.101  
 
 LOC653513  653513  1  144676436  144680025  1q21.1  0  0.381  0.719  2.242  1.414  0.812  1.105  1.285  1.116  3.2  1.972  0.171  0  0  0  0.298  0  0  0  0  3.739  0.197  0.439  0  0.322  2.151  0.59  1.055  0.527  0.215  0.74  0.955  0.18  0.551  0  0.358  1.21  0  0.328  0  0.217  1.185  0  0  0  0.59  0.204  0.993  0  0.712  0.425  0  0.086  1.008  0.103  1.325  0.626  0.597  0  0  
 
 PDE4DIP  9659  1  144851423  145076186  1q12  2.503  2.521  1.969  2.764  2.641  2.481  2.341  2.626  2.549  4.22  3.337  1.106  1.629  1.195  1.696  1.061  1.13  0.586  1.428  0.224  5.394  1.289  1.862  0.454  3.082  3.345  1.378  2.172  2.802  1.301  3.567  1.905  1.268  1.515  0.495  2.123  4.131  2.568  2.903  1.544  1.807  2.852  0.985  1.36  1.144  2.089  0.522  2.401  1.16  2.507  2.389  1.912  1.116  1.925  0.917  2.754  2.286  2.366  1.079  1.112  
 
 SEC22B  9554  1  145096406  145116997  1q21.1  2.843  4.388  3.116  2.723  2.577  3.743  3.426  3.818  5.623  3.924  4.674  2.555  2.919  3.835  2.795  2.447  3.052  3.84  4.262  2.174  2.39  2.657  2.785  2.597  3.479  3.485  1.776  4.379  4.335  1.992  3.893  3.697  2.615  2.51  1.913  3.425  4.309  2.913  4.31  3.024  2.785  4.255  2.577  2.845  3.055  3.615  1.144  2.937  2.747  3.896  4.317  1.726  3.017  3.237  2.527  3.402  3.707  3.217  3.437  3.928  
 
 NOTCH2NL  388677  1  145209112  145286270  1q21.2  3.23  3.316  1.871  0.906  3.289  2.188  3.236  2.884  1.298  2.79  3.165  1.001  1.434  2.472  2.207  1.686  1.547  0.473  1.678  1.738  1.061  1.661  2.017  2.231  2.626  2.663  2.401  3.087  2.688  2.699  2.262  2.707  2.295  1.319  1.559  2.584  3.059  2.649  2.991  2.457  2.058  1.542  2.713  1.489  1.539  2.415  2.832  2.6  1.104  2.976  2.998  1.667  2.708  1.509  2.215  2.857  2.562  2.269  2.87  1.956  
 
 NBPF10  100132406  1  145293370  146466121  1q21.1  0.439  0.211  0.117  0.039  0.322  0.259  0.173  0.52  0.065  0.345  0.387  0.083  0.146  0.297  0.289  0.115  0.063  0  0.075  0.06  0.109  0.091  0.075  0.221  0.352  0.39  0.088  0.394  0.249  0.081  0.179  0.232  0.247  0.029  0.097  0.41  0.621  0.577  0.248  0.475  0.329  0.087  0.139  0.191  0.202  0.331  0.128  0.427  0.2  0.117  0.658  0.271  0.215  0.154  0.173  0.245  0.526  0.22  0.287  0.224  
 
 LOC101928979  101928979  1  145376205  145382404  1q21.1  0.825  0.29  0.22  0  0.876  0  0.234  0.203  0.288  0.196  0.476  0.505  0.353  0.446  0  0.298  0.627  0.182  0  0  0  0.422  0  0  0.344  0.288  0.051  0.506  0  0.078  0.264  0.301  0  0.354  0.123  0.57  0.906  0.657  0.09  0.612  0.458  0.101  0.848  0  0.324  0.521  0.239  0  0  0.246  0.307  0.496  0.191  0.234  0.436  0.414  0.337  0.978  0  0.303  
 
 HFE2  148738  1  145413190  145417545  1q21.1  0  0  0.169  0  0.078  0  0  0.087  0.093  0.232  0.182  0.122  0  0.091  0  0.241  0.16  0  0  0  0.594  0.242  0.382  0.109  0  0  0.263  0  0.117  0.163  0  0  0  0.266  0.328  0  0  0  0.202  0  0  0.077  0.06  0.087  0  0.067  0.252  0  0  0  0.085  0.492  0  0.398  0.305  0.171  0  0  0  0  
 
 TXNIP  10628  1  145438437  145442628  1q21.1  5.435  5.927  1.954  3.47  3.269  3.273  4.082  1.049  0.861  5.69  4.537  3.311  3.043  0.056  3.974  2.823  5.446  5.203  3.577  4.551  4.494  4.231  4.96  4.707  2.904  3.649  3.82  1.965  2.905  2.489  3.055  2.872  2.99  2.787  0.284  1.371  1.913  5.546  5.869  0.865  2.535  2.831  0.04  3.641  1.933  3.75  4.463  0.666  4.895  3.093  5.324  0.315  2.047  2.946  4.244  4.321  3.993  2.198  2.75  2.847  
 
 POLR3GL  84265  1  145456235  145470387  1q21.1  3.843  4.803  1.917  1.578  3.6  2.366  4.138  3.093  2.125  3.18  3.089  2.49  2.714  4.158  2.045  1.853  2.866  4.595  4.642  3.226  3.202  2.679  3.314  3.277  2.983  3.175  2.619  1.845  2.337  1.991  3.012  2.849  2.72  2.139  2.106  2.822  2.589  3.504  2.767  2.215  2.388  1.784  3.779  2.047  1.136  2.084  3.253  0.996  2.308  2.959  3.032  1.903  2.615  1.788  3.607  2.712  2.037  2.902  2.543  3.146  
 
 ANKRD34A  284615  1  145470507  145475647  1q21.1  2.267  0.303  0.762  0.638  2.42  0.629  0.783  0.885  0.406  1.2  0.924  0.189  0.436  0.46  0.434  0.365  0.26  0.114  0.254  0.255  0.491  0.396  1.603  2.049  1.713  1.124  1.157  0.604  0.738  0.372  0.892  0.781  1.41  1.422  0.185  1.045  0.416  0.918  0.589  0.121  0.622  0.661  1.722  0.148  0.213  0.334  0.387  0.259  0.146  0.289  0.59  1.006  0.229  0.469  0.726  0.426  0.325  0.234  0.163  0.327  
 
 LIX1L  128077  1  145477066  145501669  1q21.1  0.406  3.491  2.35  2.824  1.285  2.165  3.309  3.137  2.761  3.825  3.539  0.084  0.163  2.759  0.55  0.879  0.319  2.776  4.214  2.885  1.912  3.39  2.636  2.467  3.164  3.151  2.643  2.101  2.13  2.52  2.894  2.833  3.427  2.699  1.834  3.617  2.608  3.619  3.008  3.085  0.288  2.534  2.855  2.183  0.529  1.203  2.327  1.963  2.926  2.953  3.283  2.873  2.36  2.027  3.079  3.357  2.869  2.741  2.757  3.114  
 
 RBM8A  9939  1  145507556  145513535  1q21.1  4.684  5.225  2.185  2.671  4.102  2.999  4.171  3.478  3.098  3.234  4.23  3.369  2.974  4.183  2.796  3.005  3.831  5.515  5.311  4.055  3.056  4.416  2.859  3.079  3.332  2.966  2.502  2.529  2.385  2.701  3.343  3.488  3.24  2.052  2.981  3.905  3.236  3.133  4.069  3.949  3.375  3.056  4.235  3.141  2.445  3.513  4.108  2.881  3.324  4.651  3.695  3.102  3.319  2.368  3.682  3.291  3.062  3.728  3.436  3.209  
 
 GNRHR2  114814  1  145509751  145516076  1q12  0.777  0.246  0.262  0  1.093  0.122  0.479  0.161  0.573  0.324  0.419  0.426  0.494  0.256  0.438  0.296  0.171  0.507  0.824  0.224  0.289  0.622  0.807  0.707  0.409  0.275  0.371  0.377  0.316  0.414  0.279  0.254  0.746  0.552  0.312  0.893  0.34  0  0.526  0.574  0.575  0.296  0.202  0.281  0  0.491  0.648  0.225  0.411  0.238  0.55  0.198  0.33  0.176  0.389  0.148  0.194  0  0.157  0.238  
 
 PEX11B  8799  1  145516164  145523732  1q21.1  3.917  3.168  1.314  2.559  3.966  1.316  3.508  1.856  2.751  2.762  2.809  2.176  2.155  3.106  2.487  2.414  2.322  3.33  4.326  3.693  1.809  2.956  3.401  2.225  2.066  2.598  2.28  1.434  1.846  2.33  2.449  2.503  2.358  2.281  1.651  3.104  2.227  2.825  2.632  1.998  2.297  2.126  2.666  1.302  1.202  2.209  3.345  1.211  2.049  3.072  2.553  1.342  2.157  1.526  2.929  2.414  2.181  1.57  2.479  2.722  
 
 ITGA10  8515  1  145524890  145543868  1q21  0.056  0.459  0.253  1.41  0.615  0.027  0.125  0.777  0.115  1.916  0.099  0.099  0  0.121  0.102  0.035  0.074  0.113  0.341  0.044  0  0.18  0.262  0.661  0.054  0.54  0.406  0.195  0.56  0.333  0.256  1.719  0.277  0.398  0.061  0.182  0.088  0.553  0.098  0.058  0.103  0.118  0.031  0  0.416  0.067  2.629  0  0.126  0.145  0.192  0.216  0.066  0.037  0.067  0.068  0.4  0.031  0.075  0.069  
 
 ANKRD35  148741  1  145549208  145568526  1q21.1  0.116  0  0.239  0  0.111  0.182  0  0.054  0.146  0.155  0.045  0.091  0  0.035  0.056  0.086  0.044  0.059  0.165  0.137  0  0.111  0.909  0  0  0.067  0.066  0.038  0  0.096  0  0.069  0  0.368  0  0  0  0  0.106  0  0  0.233  0.054  0  0.15  0.306  0.452  0  0  0  0.039  0.477  0.07  0.06  0.104  0  0  0  0  0.048  
 
 PIAS3  10401  1  145575987  145586546  1q21  5.045  2.453  2.846  3.044  4.54  2.372  3.549  4.049  2.554  3.941  3.229  2.142  1.472  3.543  2.929  3.425  2.838  0.848  3.113  1.605  2.004  2.185  1.603  1.451  2.765  2.561  2.611  2.708  2.452  3.28  2.829  3.537  3.225  2.139  2.377  3.856  3.187  3.822  2.868  4.387  3.37  2.519  3.927  2.944  1.714  3.911  3.52  2.407  3.032  3.936  3.438  2.567  2.919  2.164  3.05  3.452  2.968  2.65  3.123  3.256  
 
 NUDT17  200035  1  145586492  145589435  1q21.1  1.266  0.279  0.523  0.533  1.414  0  0.396  0.604  0.465  0.418  0.463  0.137  0.159  0.453  0.849  0.36  0.484  0.147  1.296  0.139  0.865  0.389  0.495  0.336  0.391  0.87  0.253  0.589  0.304  0.928  0.893  1.216  0.609  0.345  0.489  2.258  0.958  0.956  0.362  0.567  0.775  1.06  0.998  0.293  0.24  0.502  0.213  0.836  0.473  2.152  0.425  1.228  0.11  0.331  1.334  0.519  0.173  0.43  1.203  0.87  
 
 POLR3C  10623  1  145590666  145611044  1q21.1  3.419  3.433  1.245  2.061  2.209  1.578  2.735  2.684  1.327  2.231  2.693  1.67  2.269  2.735  1.786  1.605  1.825  2.261  3.154  2.863  2.029  2.252  1.48  1.958  1.851  2.162  1.951  2.424  1.912  2.374  2.807  2.711  2.714  1.381  1.424  2.81  3.021  2.434  2.145  2.482  2.102  1.227  2.964  1.817  1.563  2.532  2.391  1.842  2.031  3.076  2.119  2.087  2.427  1.876  2.589  2.49  2.409  1.603  2.766  2.414  
 
 RNF115  27246  1  145610989  145689005  1q21.1  3.864  4.073  2.248  2.036  3.266  2.348  3.62  3.182  1.711  2.583  3.478  2.175  2.056  2.962  2.017  2.052  2.056  2.748  3.575  2.286  2.118  2.48  1.475  1.936  1.837  2.324  2.056  2.02  1.783  2.545  3.204  2.925  2.6  1.44  2.142  3.196  2.859  2.679  2.351  2.767  2.896  1.651  3.332  2.255  1.753  2.872  3.107  2.094  2.675  3.47  2.822  2.97  2.956  2.068  3.088  3.21  2.737  1.986  3.365  3.072  
 
 CD160  11126  1  145695797  145715639  1q21.1  0.271  0.657  0.225  0  0.162  0  0.266  0.301  0.177  0.276  0.387  0  0  0.348  0.284  0.177  0  0.744  0.731  0.37  0.222  0.758  0.5  0.225  0  0.184  0.04  0.122  0.137  0.292  0.183  0.158  0.155  0.237  0.372  0.369  0.248  0.171  0.278  0  0.258  0.111  0.568  0.216  0  0.22  1.002  0.188  0  0.279  0.209  0.554  0.243  0.167  0.24  0.129  0.13  0.226  0.35  0  
 
 PDZK1  5174  1  145727665  145764206  1q21  0.539  0.305  0.082  0  2.202  0  0.171  0.202  0.156  0.193  0.292  1.544  0  0.334  0.179  0.285  0.08  1.294  0.212  0.43  0  0.525  0.218  0.158  0  0.254  0.106  0.145  0.087  0.065  0.252  0  0  0.236  0.442  0.31  0.2  0.305  0.252  0  0.151  0.271  0.305  0.101  0  0.205  1.19  0  0.801  0.352  0.398  0.157  1.709  2.293  2.664  2.427  0.814  0  2.58  0.109  
 
 GPR89A  653519  1  145764594  145827103  1q21.1  3.501  4.241  0.995  1.36  3.147  1.625  3.049  1.847  1.241  2.082  1.92  2.088  1.706  2.317  1.34  1.658  1.44  3.237  3.171  2.914  1.955  2.636  1.994  2.11  1.213  1.71  1.673  1.322  1.435  1.488  2.282  1.985  1.868  0.844  1.21  2.485  2.355  2.18  1.783  1.919  1.976  1.104  2.717  1.671  1.055  2.116  3.15  1.372  1.751  2.644  2.132  1.216  2.092  1.359  2.126  2.165  1.606  1.668  2.101  1.773  
 
 NBPF11  200030  1  146032541  146082633  1q21.1  0.853  0.259  0.179  0.275  0.595  0.373  0.509  0.416  0.269  0.522  0.539  0.137  0.399  0.362  0.462  0.271  0.234  0.15  0.797  0.1  0.294  0.448  0.276  0.24  0.258  0.511  0.433  0.554  0.416  0.594  0.539  0.508  0.613  0.25  0.311  0.692  0.594  0.604  0.336  0.628  0.374  0.271  0.637  0.485  0.161  0.378  0.167  0.395  0.279  0.495  0.809  0.398  0.277  0.145  0.443  0.317  0.521  0.349  0.348  0.317  
 
 NBPF12  149013  1  146373856  146467639  1q21  0.826  0.172  0.247  0.405  0.841  0.284  0.687  0.599  0.438  0.658  0.736  0.15  0.538  0.514  0.58  0.333  0.275  0.052  1.366  0.065  0.265  0.495  0.355  0.234  0.236  0.432  0.698  0.46  0.493  0.752  0.513  0.616  0.665  0.239  0.261  0.73  0.686  0.642  0.206  0.768  0.54  0.358  1.389  0.6  0.191  0.47  0.166  0.46  0.306  0.91  0.859  0.458  0.379  0.195  0.533  0.369  0.752  0.413  0.572  0.309  
 
 LOC728989  728989  1  146490894  146514599  1q21.1  0  0.196  0  0  0.158  0.237  0.288  0.241  0.567  0.863  0.345  0  0  0.129  0.175  0  0  0  0.13  0  0.526  0.116  0.66  0  0  0.364  0.109  0.475  2.457  0.052  2.246  0.457  0.146  0  0  0  1.182  0.443  0.357  0  0  0.25  0.6  0  0  0.102  0  0.287  0  0.405  0.294  0.199  0  0.155  0  0  0.289  0  0  0  
 
 RNVU1-8  101447996  1  146556162  146556303  1q21.1  0  0  0  0  0  0  0  0  0  0  0  0  0  0  0  0  0  0  0  0  0  1.364  0  0  0  0  0  0  0  0  0  0  0  0  0  0  0  0  0  0  0  0  0  0  0  0  0  0  0  0  0  0  0  0  0  0  0  0  0  0  
 
 NBPF13P  644861  1  146571065  146585928  1q21.1  0  0  0  0  0.721  0  0.211  0  0  0.092  0  0  0  0  0  0  0  0  0  0  0  0  0  0  0  0  0.084  0.085  0  0.05  0  0  0  0.175  0  0  0  0  0  0  0  0.067  0.137  0  0  0  0.043  0  0  0  0  0  0  0  0  0  0  0  0  0  
 
 PRKAB2  5565  1  146626684  146644168  1q21.1  2.153  1.009  1.28  1.046  1.701  0.77  1.498  1.792  1.804  1.869  2.284  0.831  1.251  1.736  1.321  0.73  1.294  1.536  1.741  0.936  0.853  1.163  0.91  0.504  1.242  1.947  1.076  1.322  0.836  1.001  1.291  1.725  1.193  0.892  0.762  1.795  1.311  1.34  1.532  1.466  1.619  1.247  1.861  1.494  0.62  1.492  1.109  0.85  1.247  2.079  2.264  1.179  1.269  1.145  1.643  1.454  1.874  0.895  1.882  1.417  
 
 PDIA3P1  171423  1  146649429  146651528  1q21.1  1.656  0.968  2.859  3.157  2.551  2.159  1.682  3.015  4.135  2.789  3.797  3.135  2.811  1.997  2.818  3.093  2.737  0.396  1.74  2.188  1.384  1.589  2.088  1.677  2.254  2.949  2.204  2.504  3.065  1.668  2.129  2.379  1.715  1.494  1.538  2.367  2.546  1.95  2.086  2.318  1.997  2.745  2.623  2.153  1.69  1.578  1.14  1.58  1.529  2.083  2.191  1.552  2.946  2.53  2.382  2.402  2.707  1.633  1.827  1.715  
 
 FMO5  2330  1  146655883  146697230  1q21.1  0.446  0.054  0.154  0  0.816  0.113  0.231  0.175  0.147  0.226  0.17  1.304  0.359  0.082  0.199  0.088  0.481  0.146  0  0.099  0  0.089  0.241  0.058  0.078  0.186  0.128  0.064  0.062  0.114  0.108  0  0  0.242  0.104  0  0.078  0.313  0.036  0  0.09  0.101  0.13  0  0  0.057  0.024  0  0.252  0.154  0.218  0.475  0.039  0.151  0.079  0  0.086  0.079  0.12  0.104  
 
 CHD1L  9557  1  146714290  146767447  1q12  2.683  2.803  1.792  1.718  2.47  1.812  2.392  2.405  1.825  2.631  2.996  2.005  2.051  2.898  2.416  2.226  2.8  2.28  4.539  3.77  2.465  3.357  1.497  1.83  2.207  3.261  2.243  2.058  2.452  2.804  3.227  3.122  2.489  1.244  2.278  2.897  2.69  2.687  2.933  3.099  2.827  2.292  3.992  2.403  1.317  1.91  2.589  1.912  1.66  3.447  3.009  2.284  2.036  1.194  2.934  2.532  2.367  1.995  1.811  2.535  
 
 LINC00624  100289211  1  146853913  146989699  1q21.1  0.054  0  0.142  0  0.031  0  0  0  0.067  0.09  0.082  0.108  0  0.111  0  0.056  0  0.159  0.033  0  0.21  0.108  0.214  0  0  0  0.047  0.031  0.062  0.032  0.038  0.064  0  0.298  0  0.054  0.034  0  0.039  0.079  0.15  0.027  0.027  0.049  0  0.058  0.029  0  2.55  0  0.058  0.148  0.125  0.086  0.06  0  0.032  0  0.036  0  
 
 BCL9  607  1  147013270  147098020  1q21  2.42  0.715  0.717  1.054  2.014  1.247  2.235  1.736  1.871  2.456  2.959  1.86  1.881  2.442  2.351  1.918  1.717  0.749  2.135  0.117  0.841  2.09  0.89  1.702  1.928  2.686  2.374  2.441  2.25  1.854  1.848  2.796  3.087  1.364  1.309  2.605  2.045  2.272  0.839  3.448  2.129  1.699  2.422  2.982  1.74  2.246  1.517  1.585  1.911  1.286  2.54  1.981  2.371  1.715  2.336  3.106  2.151  1.296  2.618  2.295  
 
 ACP6  51205  1  147119167  147142634  1q21  2.138  2.41  0.23  0.208  2.088  0.96  2.232  0.914  1.716  3.36  3.297  2.773  2.099  2.345  1.427  2.968  2.924  3.018  2.292  0.9  0.114  1.979  1.899  2.254  1.456  1.759  1.937  1.496  1.431  1.785  2.205  2.645  2.224  1.469  1.216  1.862  2.469  1.864  0.706  1.349  3.106  2.283  1.363  1.148  0.855  1.965  1.441  1.081  1.107  3.377  2.79  2.127  1.752  0.526  2.072  1.136  1.211  1.429  1.539  1.557  
 
 GJA5  2702  1  147228331  147245466  1q21.1  0  0  0.195  0  0.123  0.268  0  0.029  0.129  0.38  0  0.046  0  0.023  0  0.056  0  0  0  0  0  0.042  0.46  0  0  0  0.067  1.449  0.194  0.053  0  0  0  0.547  0  0  0  0  0.638  0  0.069  5.134  0.043  0  0  0.045  0.153  0.191  0  0.751  0.232  0.23  0  0.021  0.023  0  0  0  0  0  
 
 GJA8  2703  1  147374945  147381395  1q21.1  0  0  0.136  0  0  0  0.071  0  0.165  0.048  0.07  0.143  0  0.076  0  0.064  0  0.191  0  0.085  0  0.205  0.482  0  0  0  0.142  0  0  0.107  0  0  0  0.283  0  0  0  0  0  0  0  0.568  0.072  0  0.175  0  0.039  0  0  0  0  0.515  0.054  0.134  0.103  0  0  0  0  0  
 
 GPR89B  51463  1  147400505  147465753  1q21.1  3.009  3.159  0.779  1.019  2.547  1.174  2.388  1.422  0.949  1.687  1.486  1.641  1.282  1.726  1.129  1.344  1.076  2.441  2.735  2.139  1.396  2.076  1.851  1.64  0.853  1.374  1.459  1.051  1.111  1.279  1.869  1.552  1.642  0.926  0.904  2.027  1.938  1.7  1.227  1.49  1.51  1.118  2.168  1.183  0.799  1.705  2.708  0.918  1.233  2.027  1.794  0.993  1.555  0.94  1.647  1.573  1.26  0.924  1.475  1.242  
 
 PDZK1P2  100034743  1  147466093  147487188  1q21.2  0.269  0.18  0.1  0  0.929  0  0.096  0.071  0.068  0.079  0.14  0.622  0.078  0.24  0.121  0.153  0.081  0.397  0.088  0.244  0  0.319  0.138  0  0  0.101  0.116  0.096  0  0.093  0.162  0.064  0  0.141  0.243  0.074  0.101  0.158  0.128  0  0.093  0.302  0.363  0  0  0.085  0.357  0  0.225  0.058  0.138  0.111  0.957  1.246  1.625  1.138  0.283  0  1.21  0  
 
 LOC101927468  101927468  1  147680369  147719342  1q21.2  0  0  0.116  0  0  0.171  0  0  0.143  0.059  0  0  0  0.044  0  0  0  0  0  0  0  0.118  0.386  0  0  0  0.061  0  0  0.041  0  0.083  0  0.171  0  0  0  0  0.047  0  0.216  0  0  0  0  0  0  0  0  0.147  0  0  0  0  0  0  0  0  0  0.1  
 
 RNU1-13P  26860  1  147860641  147860803  1q21.1  0  0  0  0  0  0  0  0  0  0  0  0  0  0  0  0  0  0  0  0  0  1.029  0  0  0  0  0  0  0  0  0  0  0  0  0  0  0  0  0  0  0  0  0  0  0  0  0  0  0  0  0  0  0  0  0  0  0  0  0  0  
 
 NBPF14  25832  1  148250248  148346756  1q21.1  0.059  0.041  0  0  0.017  0  0.014  0.033  0  0.021  0.027  0  0  0.027  0.029  0  0  0  0.013  0  0  0  0  0  0.02  0.018  0.012  0.055  0.014  0.01  0  0.021  0.021  0  0.014  0.037  0.057  0.047  0.009  0.051  0  0  0.008  0.016  0  0.014  0.066  0.022  0  0.03  0.063  0.022  0.012  0  0.015  0  0.034  0  0.03  0.021  
 
 NBPF15  284565  1  148558187  148596267  1q21.2  3.089  2.612  1.956  2.591  2.578  1.798  2.797  2.383  2.381  2.738  2.211  0.841  1.916  1.782  1.792  1.594  1.539  1.303  2.724  1.016  0.623  1.494  1.91  1.327  1.941  2.361  2.948  2.368  2.616  2.944  2.502  2.279  2.726  2.009  1.421  2.358  2.919  2.08  2.913  2.539  1.428  2.162  3.868  1.533  0.684  1.579  1.904  2.213  1.46  3.47  3.124  1.613  2.053  1.228  2.565  2.235  2.199  1.692  2.516  1.575  
 
 DRD5P2  1818  1  148901844  148904054  1q21.1  0  0.19  0.642  0.174  0.082  0.163  0.054  0.059  0.565  0.039  0.051  0  0  0.041  0  0.056  0.08  0.281  0.058  0.123  0  0.345  1.67  0.093  0  0  0.193  0.026  0  0.132  0  0.059  0  1.053  0  0  0  0  0.139  0.082  0  0.719  0.061  0  0.084  0.064  0.103  0.241  0  0.599  0  1.162  0.04  0.124  0.141  0.088  0  0  0  0  
 
 LOC645166  645166  1  148928312  148953054  1q21.1  3.246  3.163  3.317  0  1.476  3.313  3.549  3.164  0.764  3.197  3.667  2.226  1.546  2.804  2.33  1.885  0  3.618  1.961  0.22  1.768  2.654  1.937  2.073  6.142  2.008  2.955  1.016  3.831  3.566  3.728  5.089  3.561  2.311  3.613  2.802  5.224  3.658  4.476  4.511  2.891  2.378  4.304  0.927  1.426  1.302  4.51  5.128  1.66  6.995  5.093  3.068  3.823  3.361  3.519  5.568  3.884  5.843  3.38  4.934  
 
 LOC388692  388692  1  149287129  149291743  1q21.2  0  0  0.738  0.129  0.265  0.18  0.492  1.478  0.751  0.525  0.254  0.322  0.46  0.384  0.433  0.828  0.29  0  0  0.205  0  0.056  1.493  0.239  0.554  0.087  0.1  0.633  0.639  0.197  0.241  1.101  0.862  0.819  0  0.962  0.541  0  0.244  0.942  0.886  0.15  0.749  0.69  0.482  0.718  0.043  0.284  1.086  0.342  0.889  1.037  0.387  0.392  0  0  0.724  0  0.201  0.151  
 
 LOC644634  644634  1  149287450  149291743  1q21.2  0.324  0.927  0.582  0  1.515  0  1.187  1.373  0.315  0.8  0.601  0.833  0.362  1.177  0.434  1.612  0.542  1.877  0.068  1.791  0.217  0.387  0.544  0.832  0.842  0  1.032  0.181  0.427  1.236  0.696  1.409  1.093  1.319  0.089  0.346  0.48  0  1.553  0.436  0.775  0.848  1.265  0.409  0.165  0.141  0.955  0.283  0.524  1.102  0  0.595  0.414  0.109  0.527  0  0.771  0.091  0  0.397  
 
 FCGR1C  100132417  1  149369293  149378303  1q21.2  0  0  0.098  0  0.058  0  0  0  0.174  0  0  0  0  0.045  0  0  0  0  0  1.039  0  0  0.367  0  0  0  0.035  0  0  0  0  0  0  0  0  0.204  0  0  0.039  0  0  0.28  0  0  0  0  0  0  0  0  0  0  0  0  0  0  0  0  0  0  
 
 PPIAL4A  653505  1  149553002  149553787  1q21.1  1.149  0.616  1.297  1.364  1.333  0.792  1.698  1.031  1.281  1.057  1.137  1.02  1.444  1.717  0.715  1.467  1.374  0  1.461  1.482  0.423  1.75  2.081  1.053  1.015  0.407  0.978  0.715  0.953  0.904  0.762  1.612  0.747  0.94  1.186  0.5  0.833  0.846  1.373  0.496  0.676  1.399  1.768  0.48  0.324  0.442  2.551  0.636  0  1.157  1.058  0.703  1.213  1.068  1.617  0.803  0.558  1.386  1.069  0.691  
 
 LOC103091866  103091866  1  149576261  149672983  1q21.1  2.305  0.263  0.54  0  0.361  0.308  0.587  1.133  0.681  0.853  0.29  0.412  0.209  0.419  0.175  0.691  0.275  0  0.087  0.366  0.289  0.313  0.946  0.35  0.563  0  0.388  0.57  0.471  0.208  0.268  0.764  0.666  0.676  0  0.822  0.369  0  0.443  0.463  0.675  0.343  0.901  0.522  0.348  0.45  0.062  0.522  0.872  0.418  0.497  1.082  0.422  0.351  0.248  0  0.411  0  0.18  0.145  
 
 RNVU1-20  101954268  1  149605916  149606079  1q21.2  0  0  0  0  0  0  0  0  0  0  0  0  0  0  0  0  0  0  0  0  0  1.003  0  0  0  0  0  0  0  0  0  0  0  0  0  0  0  0  0  0  0  2.45  0  0  0  0  0  0  0  0  0  0  0  0  0  0  0  0  0  0  
 
 HIST2H2BF  440689  1  149754244  149783928  1q21.2  8.984  6.438  6.008  4.9  5.909  7.565  6.487  5.906  3.168  6.338  5.453  7.423  4.996  7.492  6.369  6.881  7.671  4.31  4.992  6.579  6.27  8.692  2.789  6.985  6.043  4.162  6.785  4.975  5.272  4.401  6.289  4.772  5.272  3.906  8.14  5.93  5.198  5.237  5.508  6.111  6.376  7.041  6.129  6.284  6.258  5.94  6.165  5.783  5.767  3.189  4.874  7.192  6.6  6.483  6.635  5.641  4.754  6.304  5.297  5.027  
 
 FCGR1A  2209  1  149754249  149764074  1q21.2-q21.3  0.429  0.036  0.124  0  0.299  0  0.086  0  0.169  0.049  0.035  0.107  0  0.036  0  0.035  0  0.169  0.054  2.079  0  0.021  0.46  0  0  0  0.041  0  0  0.037  0  0.046  0  0.193  0  0.094  0  0  0.074  0  0.098  0.227  0.187  0  0.136  0  0  0  0  0  0.08  0.105  0  0.04  0.032  0  0  0  0  0  
 
 HIST2H3D  653604  1  149784779  149785236  1q21.2  4.612  1.271  2.52  1.436  2.13  4.483  2.312  2.174  0.811  2.141  2.056  3.279  1.239  3.037  2.338  3.352  3.79  1.444  1.821  2.694  3.627  4.393  1.149  3.311  2.565  1.186  2.505  1.705  1.999  1.494  2.915  1.663  1.884  0.932  3.983  2.999  1.866  1.788  1.854  2.371  2.974  4.066  1.586  3.065  3.468  2.538  0.626  2.082  2.346  0.975  1.478  3.998  2.844  2.949  2.826  2.093  1.449  2.181  2.091  1.91  
 
 HIST2H4A  8370  1  149804220  149804616  1q21.2  7.976  5.722  5.332  3.386  4.309  7.379  5.177  4.319  1.99  4.527  4.832  7.228  4.046  6.324  5.31  6.055  6.116  1.946  3.758  4.782  6.392  6.236  2.521  6.588  5.361  3.288  4.569  4.297  4.435  3.476  4.73  3.753  3.877  2.673  6.581  5.289  4.538  4.27  3.638  5.557  5.02  6.131  4.017  6.399  5.526  5.036  2.811  4.434  5.168  2.584  4.43  6.151  5.681  6.111  4.968  4.786  4.026  5.664  5.126  4.228  
 
 HIST2H3A  333932  1  149812258  149812765  1q21.2  6.336  2.494  3.969  2.738  3.519  6.209  4.148  3.762  1.073  3.538  2.639  4.912  2.466  4.745  3.754  4.698  5.141  0.927  3.067  3.811  5.42  5.589  2.13  5.046  4.044  1.493  3.045  2.856  2.544  2.637  3.998  2.516  2.798  1.902  5.523  4.425  3.105  2.734  2.942  3.766  4.318  5.09  3.018  4.106  4.317  3.991  2.369  3.457  3.483  1.704  2.316  5.544  4.193  4.461  4.322  3.484  2.243  4.467  3.626  2.759  
 
 HIST2H2AA4  723790  1  149813784  149814318  1q21.2  8.6  4.875  5.183  6.589  6.328  7.652  6.907  6.529  3.819  7.502  6.154  7.4  5.88  7.751  6.801  7.972  8.137  2.026  5.8  5.95  7.053  7.084  5.123  7.7  7.09  4.702  6.503  5.536  5.848  6.122  7.212  6.073  6.097  5.55  7.358  7.106  6.918  6.112  6.01  6.622  7.4  7.597  5.799  6.213  7.098  6.584  5.633  6.388  5.364  4.511  5.965  7.952  6.945  6.326  6.043  5.917  5.14  7.161  6.431  5.889  
 
 HIST2H2BC  337873  1  149821758  149822340  1q21.2  4.215  0.875  1.392  1.466  2.704  2.141  2.232  1.151  0.713  2.422  1.508  2.825  1.723  2.646  1.75  2.828  2.942  0  2.47  2.365  2.313  4.102  1.623  2.408  1.651  1.281  1.718  1.586  1.499  1.699  2.195  2.023  1.694  1.004  2.978  2.333  1.588  1.199  2.054  1.973  2.23  2.555  2.252  1.696  1.761  2.123  2.696  0.847  1.236  1.168  1.23  3.275  1.994  2.432  2.737  1.694  0.89  1.821  1.467  1.294  
 
 HIST2H2AA3  8337  1  149822627  149823161  1q21.2  8.759  4.8  5.401  6.63  6.465  7.692  6.967  6.584  3.89  7.621  6.251  7.468  5.905  7.793  6.948  8.157  8.35  1.985  5.938  5.948  7.07  7.233  4.758  7.812  7.169  4.792  6.641  5.618  5.984  6.274  7.335  6.223  6.159  5.447  7.455  7.233  6.981  6.181  5.897  6.719  7.455  7.666  5.934  6.269  7.178  6.667  5.56  6.455  5.448  4.621  6.077  8.105  7.076  6.408  6.211  6.017  5.245  7.241  6.507  6.017  
 
 HIST2H3C  126961  1  149824180  149824687  1q21.2  6.752  2.548  4.299  3.193  3.905  6.593  4.376  3.977  1.51  3.733  2.972  5.397  2.693  4.904  4.158  5.164  5.606  0.935  3.42  3.946  5.678  5.825  2.672  5.517  4.432  1.698  3.864  3.068  2.785  3.333  4.245  2.835  3.158  2.664  5.865  4.736  3.405  2.904  3.065  4.053  4.676  5.633  3.285  4.486  4.501  4.363  2.521  3.705  3.909  1.85  2.627  5.928  4.569  4.856  4.647  3.679  2.572  4.798  3.992  3.068  
 
 HIST2H4B  554313  1  149832329  149832725  1q21  7.904  5.688  5.31  3.282  4.186  7.223  5.071  4.334  1.946  4.385  4.748  6.952  4.038  6.272  5.2  5.723  5.873  1.907  3.687  4.624  6.227  6.066  2.478  6.272  4.938  3.23  4.586  4.289  4.333  3.593  4.622  3.488  3.773  2.623  6.487  5.208  4.493  4.291  3.474  5.487  4.853  5.928  4.109  6.294  5.366  4.739  2.11  4.285  4.993  2.569  4.34  5.957  5.71  5.874  4.878  4.706  3.921  5.589  4.95  4.122  
 
 HIST2H2BE  8349  1  149856009  149858232  1q21.2  5.901  3.724  3.135  2.898  3.952  5.103  3.844  2.97  1.693  4.077  2.794  4.688  2.693  3.991  3.578  4.326  4.915  2.807  2.244  3.894  4.246  5.677  2.184  4.549  3.556  2.137  3.817  2.558  2.707  2.616  3.485  2.593  2.717  2.494  5.092  3.424  2.984  2.514  3.606  3.56  3.846  4.372  3.185  3.69  3.865  3.275  3.496  3.116  3.008  1.185  3.155  4.862  3.828  4.165  3.928  3.164  2.427  3.618  2.823  2.591  
 
 HIST2H2AC  8338  1  149858524  149858961  1q21.2  7.283  3.785  4.33  5.048  5.073  6.346  5.034  4.879  3.466  5.707  4.336  6.013  3.844  5.792  4.93  6.463  6.542  2.843  4.652  4.286  5.692  6.386  3.236  5.969  5.096  3.05  4.532  3.748  3.852  4.018  5.585  4.44  4.196  3.694  5.979  5.3  5.049  4.284  4.7  4.89  5.484  6.369  4.335  5.307  5.776  5.068  3.313  4.843  4.18  3.117  4.269  6.508  5.325  5.093  5.292  4.335  3.457  5.402  4.534  4.114  
 
 HIST2H2AB  317772  1  149859018  149859466  1q21  7.69  6.46  3.513  2.777  4.548  4.861  4.483  3.38  1.53  4.412  3.661  5.594  3.476  6.848  3.527  5.018  5.493  5.077  3.632  4.847  4.387  7.468  1.824  4.855  4.224  1.657  4.085  2.397  2.568  3.186  4.087  3.471  3.093  2.419  6.355  4.499  3.002  3.375  3.712  3.023  4.482  5.041  4.452  4.499  3.909  3.571  0.784  3.269  3.502  1.424  2.381  4.678  4.233  4.731  5.326  4.361  2.369  4.55  4.229  3.166  
 
 BOLA1  51027  1  149871154  149872348  1q21  4.966  1.5  1.001  3.337  3.923  1.81  1.773  1.874  0.951  2.735  1.186  2.527  1.923  3.853  1.181  2.246  3.067  2.197  4.959  3.117  0  3.81  2.311  1.032  1.706  2.024  2.931  1.54  2.195  2.453  1.843  2.956  2.743  2.532  2.751  3.313  1.942  1.895  0.91  2.645  2.632  3.304  0.813  2.702  0.538  2.136  3.365  2.357  1.778  4.086  1.693  2.929  0.133  1.754  3.574  3.084  1.07  2.199  1.934  1.848  
 
 SV2A  9900  1  149874871  149889434  1q21.2  0.173  0  2.97  0.556  0.127  1.816  5.648  4.587  4.35  1.341  4.165  0.073  0.072  0.155  0.085  0.431  0.068  0.308  4.05  0.349  0  3.68  0.559  0.158  0  3.279  3.069  2.022  0.088  3.808  1.804  0.585  3.059  1.642  0.09  0.622  4.252  3.873  0.13  2.203  0.062  1.713  5.081  0.103  1.408  2.388  0.71  2.095  0  3.035  0.088  0.837  0.173  0.145  2.393  3.471  0.488  2.424  1.449  2.441  
 
 SF3B4  10262  1  149895208  149900144  1q21.2  6.059  3.3  3.809  4.924  6.325  4.592  5.87  5.653  4.17  5.277  5.854  4.151  4.724  5.455  4.786  4.913  4.66  1.826  6.221  4.306  4.699  5.529  3.796  5.004  5.614  5.021  4.789  5.331  4.855  5.176  5.612  6.205  5.931  4.078  5.15  5.845  5.807  5.361  4.895  6.154  5.759  5.218  5.758  4.802  4.719  5.777  4.165  4.974  4.899  5.887  4.841  4.864  5.252  4.596  5.063  5.235  4.751  5.599  6.356  5.468  
 
 MTMR11  10903  1  149900542  149908791  1q12-q21  1.63  1.454  0.757  1.978  1.359  0.526  0.859  1.026  0.926  1.044  1.283  2.527  2.175  1.307  0.666  1.726  1.355  1.349  0.191  0.203  0.083  0.178  0.549  0.134  0.779  0.991  0.426  0.323  0.287  0.097  0.458  0.45  0.53  0.361  1.02  1.674  0.831  1.758  0.572  1.506  1.131  0.712  2.011  0.493  0.505  0.9  0.731  0.728  0.947  1.855  1.004  1.068  0.534  0.648  1.353  1.633  1.307  0.963  1.141  1.138  
 
 OTUD7B  56957  1  149912228  149982686  1q21.2  2.001  1.282  0.903  1.237  1.342  1.112  1.63  1.727  0.866  2.197  2.133  0.901  1.43  1.611  1.144  1.231  1.174  0.672  1.177  0.458  1.202  0.176  0.628  0.618  1.161  2.776  1.708  2.243  2.116  1.98  2.327  1.646  2.195  0.954  0.814  1.554  1.467  1.517  1.235  2.053  1.795  0.93  1.945  1.739  0.823  1.363  1.518  1.036  1.225  1.367  1.846  1.281  1.287  0.992  1.185  1.513  1.439  1.227  1.417  1.256  
 
 VPS45  11311  1  150039349  150117505  1q21.2  3.815  3.578  1.599  2.388  3.932  1.261  3.487  2.86  2.46  3.004  3.404  2.044  1.846  3.594  2.159  1.781  2.395  3.041  4.444  2.012  2.003  2.764  2.178  1.334  1.73  2.581  2.077  1.938  2.188  1.722  2.645  2.942  2.744  1.682  1.641  2.988  2.453  2.441  3.053  2.935  2.344  1.795  3.796  1.944  1.685  2.77  2.724  1.339  1.835  3.39  2.732  1.956  2.128  1.423  2.972  2.109  2.415  2.107  2.351  2.403  
 
 PLEKHO1  51177  1  150121623  150132260  1q21.2  2.67  1.066  3.53  3.422  1.021  1.927  2.658  3.985  2.551  2.798  3.208  0.114  0.25  1.679  0.997  0.169  0.955  0.415  6.04  1.788  1.149  4.928  3.438  2.132  1.462  2.956  2.446  0.995  2.228  1.521  2.54  2.579  2.614  1.888  1.044  1.463  2.725  2.422  2.672  1.345  0.206  1.957  3.958  1.602  1.421  2.754  1.077  0.988  1.732  1.498  1.442  1.578  2.399  0.726  2.232  1.847  3.235  1.675  2.219  2.069  
 
 ANP32E  81611  1  150190716  150208504  1q21.2  2.753  3.525  2.325  2.684  2.332  2.75  2.373  2.836  1.537  2.552  3.988  2.128  2.971  3.047  2.917  2.679  3.211  2.984  5.047  3.239  2.69  3.146  2.702  2.667  2.652  2.482  1.599  1.729  1.965  1.592  2.81  2.677  2.455  1.812  2.112  3.232  2.759  2.28  2.078  3.897  3.118  2.825  2.548  2.77  2.357  3.138  1.991  1.799  2.533  3.673  2.968  2.763  2.76  1.682  2.51  3.173  2.79  3.125  3.203  2.98  
 
 CA14  23632  1  150230137  150237480  1q21  0.284  0  0.101  0.213  0.17  0.258  0.211  0.123  0.237  0.169  0.221  0.061  0.227  0.228  0.166  0.127  0.099  0.159  0.223  0  0  0.138  0.44  0.119  0  1  0.546  1.845  0.167  2.674  4.457  1.462  3.132  1.955  0  0.074  0.119  0.122  0.113  0.213  0.123  0.168  0.426  0  0.15  0.087  0.044  0.082  0  0  0.77  0.357  0.12  0.05  0.079  0.122  0.068  0.153  0.121  0.067  
 
 APH1A  51107  1  150237798  150241609  1p36.13-q31.3  5.096  0.842  3.17  4.868  4.036  3.974  4.103  4.302  4.498  4.2  4.548  3.867  4.434  3.832  4.032  3.973  3.764  0.162  5.259  1.924  3.376  3.054  4.225  3.891  3.866  4.195  3.447  3.676  3.72  4.321  4.288  4.104  4.808  3.692  3.256  3.937  3.975  4.642  3.068  4.704  4.124  4.5  3.689  3.616  3.494  4.278  1.593  3.045  3.769  4.708  4.744  3.762  3.787  3.247  3.824  4.134  3.875  4.307  4.517  4.109  
 
 C1orf54  79630  1  150244686  150253335  1q21.2  0.648  0  0  0  0.283  0  1.405  2.464  0  1.063  0.383  0  0.471  0.812  0  0.32  0.391  0  0.983  0.861  0  0.353  0  0  0  0.46  0.851  0  0.421  0.931  1.336  0  0.483  0  0  0  0  0  0.507  0  0  0  0  0  0  0.7  0.162  0  0  0.433  0.931  0  0  0  0.299  0  0  0  0.721  0  
 
 CIART  148523  1  150254942  150259504  1q21.2  3.529  0.386  1.621  2.6  2.406  1.82  1.939  1.696  2.581  2.74  1.134  0  0.568  0.204  0  0.131  0.183  0  0.593  0.124  0  0.513  0.404  0  0  0.638  1.551  0.16  1.677  0.191  0.316  0.255  2.31  1.299  0  0.221  0.495  1.359  2.081  1.259  0  1.099  0  0  1.166  1.951  0.08  0.499  1.746  0.79  3.426  0.307  1.976  0.604  3.286  1.961  2.41  0.498  2.365  0.209  
 
 MRPS21  54460  1  150266261  150281414  1q21  5.78  5.176  3.934  5.375  5.337  4.783  4.512  4.237  4.353  5.06  5.033  4.338  2.97  5.74  2.169  4.906  4.088  5.904  6.15  6.686  4.603  5.331  5.239  5.396  4.512  4.299  4.372  3.617  4.12  3.659  4.66  4.747  4.891  4.766  4.856  4.858  2.386  3.953  5.996  4.397  3.399  5.647  5.607  4.76  3.418  4.448  5.438  3.476  3.422  5.81  4.106  4.232  3.674  2.915  5.803  5.399  3.612  4.425  4.475  3.914  
 
 PRPF3  9129  1  150293927  150325704  1q21.1  3.77  2.998  1.687  2.797  3.202  2.071  3.141  3.051  1.935  3.111  3.616  2.382  2.369  3.197  2.557  2.445  1.963  1.557  4.139  3.182  2.567  3.805  2.077  2.397  2.547  2.652  2.518  2.665  2.181  2.054  2.722  3.034  2.928  1.509  2.718  3.132  3.457  2.379  2.819  3.703  2.565  2.361  3.677  2.413  2.559  2.093  3.595  2.359  2.194  3.347  2.885  2.428  2.559  1.894  2.959  2.982  2.468  2.358  2.941  2.392  
 
 RPRD2  23248  1  150336586  150449041  1q21.3  3.678  2.023  1.722  2.321  2.638  1.451  2.548  2.587  2.007  2.852  3.094  2.176  2.659  2.957  2.566  2.508  2.429  1.846  2.934  2.264  2.126  2.706  2.03  2.639  2.264  2.768  2.053  2.011  2.282  2.159  2.605  2.603  2.573  1.351  1.874  3.149  2.339  2.603  2.259  3.569  2.888  1.874  2.659  3.051  2.028  2.599  1.513  2.178  2.305  2.546  2.798  2.474  2.202  1.846  3.257  2.878  2.474  2.215  3.213  2.556  
 
 TARS2  80222  1  150459839  150480085  1q21.3  3.405  1.723  1.463  2.76  2.869  1.45  2.305  1.942  1.895  2.206  2.492  2.482  2.293  1.607  2.582  2.25  3.078  1.148  3.071  2.019  2.81  1.962  2.723  2.408  1.81  2.991  1.889  2.093  2.206  2.264  2.58  3.07  2.877  1.878  1.804  2.508  2.52  2.411  1.801  3.454  1.99  2.098  2.601  2.098  1.584  2.377  1.873  1.994  1.697  3.692  2.934  2.44  1.929  1.539  2.579  2.903  1.934  2.645  2.64  2.528  
 
 ECM1  1893  1  150480486  150486265  1q21  0.512  2.761  4.698  0.471  0.229  2.48  4.588  5.853  1.78  4.026  3.61  4.54  1.06  1.006  0.487  0.686  3.162  0.181  1.22  0.33  2.945  0.502  0.474  0.083  4.865  6.535  6.202  4.039  5.349  6.03  7.047  6.761  6.556  4.957  0.241  1.134  1.94  3.45  2.447  0.661  0.325  2.196  0.852  0.324  0  0.277  3.118  3.958  0.532  5.84  2.412  1.023  0.72  2.508  4.254  1.78  2.421  6.263  2.434  1.613  
 
 FALEC  100874054  1  150488232  150490508  -  0  0.307  0  0  0.353  0.381  0.456  0.283  0  0.192  0.267  0  0  0.264  0  0  0  0  0  0  0  0  0  0.368  0  0.319  0.295  0  0  0.091  0.626  0  0  0  0.323  0.532  0  0  0.129  0.334  0  0.16  0.848  0.27  0  0.243  0.163  0.397  0  0  0.38  0.792  0.236  0  0.246  0  0  0  0  0  
 
 ADAMTSL4  54507  1  150521844  150533412  1q21.3  0.349  0.667  1.898  1.434  1.45  0.081  0.289  0.722  0.208  0.439  0.36  0.038  0.145  0.493  0.313  0.106  0.452  0  0.18  0.261  4.125  0.105  0.623  0  0.347  0.537  0.472  0.495  0.204  0.629  0.157  0.534  0.243  0.22  0.334  1.654  1.122  0.8  1.367  1.759  0.622  1.479  1.976  1.779  0.14  0.684  1.236  4.086  0.439  5.12  0.651  1.357  0.175  0.137  3.48  1.969  0.684  5.613  1.96  0.815  
 
 C1orf138  574406  1  150533370  150547028  1q21.2  0  0.254  0.311  0  0.26  0  0.18  0.435  0  0.173  0.118  0  0  0.178  0.169  0.115  0.201  0  0  0.135  0.421  0.093  0.436  0.143  0  0.164  0.456  0.303  0.154  0.056  0  0.188  0.176  0.273  0  0.228  0.304  0.263  0.169  0.291  0.215  0.829  0.408  0.37  0  0.454  0.107  0.206  0.361  0.174  0.217  0.35  0.102  0.188  0.116  0.134  0.323  0.589  0.339  0  
 
 MCL1  4170  1  150547026  150552214  1q21  3.669  4.375  3.448  4.756  3.68  2.969  3.768  5.39  3.675  3.912  4.179  2.869  3.283  3.42  3.451  4.011  4.997  3.272  4.376  3.052  3.81  3.12  3.501  2.944  4.629  3.844  3.217  2.978  2.839  1.9  3.093  3.674  3.323  2.745  2.78  4.317  4.284  3.95  4.609  5.073  3.704  3.348  4.233  3.94  3.172  4.122  3.11  3.115  3.609  5.122  3.919  3.453  3.483  3.05  4.21  4.192  3.821  4.449  3.875  3.933  
 
 ENSA  2029  1  150594598  150602098  1q21.3  5.667  5.544  3.844  5.303  6.636  4.654  4.766  4.632  4.802  4.784  5.326  4.443  4.298  4.519  4.37  5.174  5.59  5.015  6.335  4.865  4.726  5.632  5.117  4.622  4.537  4.32  3.773  3.271  3.734  3.618  4.63  4.649  4.486  3.917  4.19  4.847  4.411  4.986  5.116  5.001  5.418  5.12  4.98  4.78  4.502  5.64  4.879  4.099  4.354  6.388  4.431  4.506  4.132  3.636  5.093  4.838  3.941  5.4  5.129  4.408  
 
 GOLPH3L  55204  1  150618700  150669672  1q21.3  3.371  1.385  0.529  2.192  1.899  1.038  2.107  2.216  1.957  2.061  2.48  2.349  2.508  2.92  1.977  2.107  3.057  1.527  3.05  2.715  1.501  1.749  2.704  1.707  1.702  1.958  1.39  1.153  1.587  0.81  1.633  1.678  1.468  1.322  1.325  1.939  2.174  2.231  2.803  2.04  2.293  1.166  2.32  1.485  1.685  2.265  1.764  1.168  1.255  2.524  2.67  1.236  1.713  1.762  2.087  2.759  1.704  1.731  2.029  2.32  
 
 HORMAD1  84072  1  150670534  150693364  1q21.3  0  0  0  0.278  0  0  0  0  0  0  0.06  0  0  0  0  0  0  0  0  0  0  0  0  0  0  0  0.028  0  0  0.206  0  0  0.081  0.137  0.496  1.969  0  0  0  0  0.602  0.206  0  0  0.249  0  0.031  0  0  0  0  0  0  0  0  0  0  0.7  0  0  
 
 CTSS  1520  1  150702671  150738433  1q21  0  1.767  0.13  0.12  0.089  0  2.87  0.377  0.213  0.064  0.24  1.532  0.626  0.026  0  0.637  0.144  0.057  0.483  2.65  0.393  0.105  3.115  2.127  0.087  0.192  0.277  0.064  0.261  0.022  0.383  0.224  0.091  0.295  0.321  0.199  0.471  1.684  0.102  0.523  0.138  0.132  0.02  0  0.152  0.484  0.038  0  0.073  0.072  0.284  0.1  0.237  1.276  0.22  1.518  0.756  0  0.146  0.083  
 
 CTSK  1513  1  150768683  150780917  1q21  1.565  1.011  0.316  1.212  1.085  0.251  2.055  0.747  0.739  1.132  1.099  0.222  0.378  1.251  0.853  0.546  0.542  0.722  1.654  0.635  0  0.652  0.503  0  0.346  4.222  3.994  0.461  4.615  0.314  5.501  3.853  0.422  0.668  0.387  1.497  0.667  1.011  1.313  0.919  0.71  0.977  0.688  1.463  0.156  0.812  1.018  0.3  0.441  1.106  0.536  1.04  0.335  0.163  1.063  0.658  0.991  0.432  0.912  0.205  
 
 ARNT  405  1  150782180  150849244  1q21  2.696  0.978  1.24  1.739  2.195  1.453  1.904  2.398  1.348  2.456  2.127  1.04  1.637  1.917  2.109  1.178  2.057  0.24  2.013  1.435  1.967  1.265  1.16  1.351  1.619  2.318  1.29  1.671  1.397  1.456  1.813  1.828  1.808  0.892  1.35  3.158  2.174  2.076  1.175  3.345  1.873  1.049  2.372  2.154  1.18  2.428  1.178  1.297  2.139  2.071  2.194  1.798  1.564  0.902  2.006  2.48  2.178  1.544  2.074  2.049  
 
 SETDB1  9869  1  150898814  150937220  1q21  3.743  1.392  1.174  1.578  2.432  1.309  2.169  2.197  1.516  2.164  2.246  1.641  2.204  2.54  2.539  1.963  1.759  1.229  3.131  2.394  2.233  2.516  1.891  1.724  1.452  2.219  1.836  1.921  1.738  1.688  2.084  1.964  2.146  1.156  1.646  2.235  2.259  2.193  1.609  2.988  2.195  1.267  2.604  2.112  1.84  2.61  1.256  1.503  2.264  2.347  1.983  1.978  2.184  1.653  2.402  2.433  2.105  1.934  2.397  1.905  
 
 CERS2  29956  1  150937648  150947479  1q21.3  5.385  4.173  3.848  4.86  6.08  3.951  5.392  4.781  4.985  4.744  5.116  3.87  4.709  5.107  4.128  4.761  4.175  1.756  5.397  3.671  4.502  4.037  3.757  4.096  4.277  4.829  4.239  4.367  3.973  4.18  4.968  5.366  5.019  3.574  4.095  6.013  5.353  5.099  4.017  5.29  4.58  4.544  5.258  4.277  3.56  4.982  3.888  3.845  4.843  5.148  5.656  4.708  4.351  4.653  4.591  5.047  4.266  4.8  4.989  4.566  
 
 ANXA9  8416  1  150954498  150968114  1q21  3.07  0  0.087  0  4.331  0.072  0.134  0.1  0.214  0.11  0.444  1.45  1.903  0.824  1.474  2.706  0.209  0.159  0.122  0.162  0  0.096  0.366  0.395  0.101  0.616  0.196  0.132  0.057  0.235  0.284  0.424  0.092  0.142  0.474  0.399  0.162  0.133  0.607  0.434  3.033  0.249  0.402  0.318  1.438  2.76  0.088  0.19  0.354  0.537  0.162  0.358  0.086  0.648  0.252  0.17  0.067  0.158  0  0  
 
 FAM63A  55793  1  150969300  150980854  1q21.3  2.442  0.155  0.949  1.102  2.831  0.438  1.67  1.108  1.412  1.188  1.685  2.242  1.745  0.344  1.395  1.485  0.614  0.159  0.88  0.658  1.035  0.186  2.555  2.746  1.085  1.488  1.486  0.955  1.428  2.355  1.98  1.306  1.537  1.053  0.483  1.922  1.159  1.295  0.164  0.745  1.931  1.295  1.718  0.775  0.943  1.653  2.279  1.001  1.06  1.806  0.612  1.228  0.574  1.577  1.223  1.179  0.587  1.397  1.315  1.432  
 
 PRUNE  58497  1  150980866  151008189  1q21  3.949  1.879  1.418  2  3.083  1.064  2.289  2.204  2.041  2.098  2.311  2.634  2.134  2.689  2.195  1.835  2.272  1.693  3.812  2.253  2.242  2.802  1.983  1.514  1.411  2.218  1.397  1.613  1.527  1.483  1.918  2.197  2.272  1.524  1.357  2.328  2.294  1.76  1.462  2.95  2.087  1.676  2.743  2.163  1.801  2.309  2.361  1.442  2.289  2.943  1.999  2.009  1.561  2.28  1.969  2.368  1.851  1.703  2.084  1.862  
 
 BNIPL  149428  1  151009028  151020076  1q21.3  0.631  0.091  0  0  4.096  0.111  0  0.039  0.193  0.15  0.067  0.177  0.083  0.126  0.169  0.064  0.096  0.1  0.289  0.138  0.116  0.17  0.638  0  0  0.055  0.121  0  0.058  0.043  0  0.106  0.128  0.312  0.08  0.171  0.078  0.126  0.182  0.127  0.36  0.13  0.033  0.169  0.133  0.162  0  0.1  0.112  0.232  0.165  0.29  0.094  0.104  0.064  0.086  0.105  0.072  0.09  0  
 
 C1orf56  54964  1  151020258  151023871  1q21.3  2.535  1.914  1.071  1.62  2.416  1.341  2.121  1.674  1.637  1.851  1.744  1.088  1.742  0.498  0.996  1.205  0.948  1.364  1.525  1.643  0.712  0.892  1.687  1.033  0.834  1.619  1.143  1.331  1.198  2.651  2.229  0.753  1.693  1.818  1.957  2.142  1.35  1.612  1.742  2.527  1.883  2.7  2.833  0.881  1.031  1.559  3.017  1.055  0.668  2.052  1.246  1.9  1.062  0.528  1.744  1.721  0.754  1.666  1.4  0.804  
 
 CDC42SE1  56882  1  151023446  151032125  1q21.3  4.276  1.757  2.204  2.84  4.589  2.705  3.796  4.529  2.491  3.63  4.136  3.101  3.551  3.257  3.097  2.838  2.879  0.905  3.999  2.771  3.908  2.822  2.162  2.755  3.743  4.382  3.127  3.126  2.772  2.676  3.324  3.905  3.741  2.237  2.461  4.233  3.145  3.306  3.116  3.583  3.996  2.219  2.899  2.765  3.202  3.569  2.549  2.903  3.036  3.621  3.02  2.372  3.025  3.613  3.582  3.578  3.236  3.752  3.422  3.533  
 
 MLLT11  10962  1  151032150  151040973  1q21  0.821  0.675  2.384  1.797  1.86  2.403  4.622  3.85  3.358  1.889  5.715  0.393  2.659  0.32  0.596  0.766  0.068  2.843  5.525  0.16  2.794  4.211  0.535  0.307  0.135  1.975  3.266  0.288  1.028  0.744  1.978  0.803  1.054  1.141  2.096  2.722  2.37  0.341  0.882  1.353  0.555  4.678  5.557  0.292  1.364  1.632  0.17  2.215  0.561  4.069  2.474  0.39  1.034  3.929  2.141  3.238  3.798  1.414  1.113  2.599  
 
 GABPB2  126626  1  151043079  151091007  1q21.3  2.138  1.136  0.789  0.736  1.899  0.474  1.097  1.311  0.399  1.422  1.199  0.903  1.396  1.177  1.533  0.818  0.547  0.451  1.478  1.22  0.548  1.446  0.829  0.646  0.816  0.848  0.746  0.798  0.603  0.638  0.546  0.903  0.788  0.35  0.426  1.403  1.019  0.652  0.741  1.706  1.329  0.933  0.941  0.871  1.075  0.791  0.943  0.684  1.212  0.802  1.027  1.008  1.059  0.811  0.777  1.064  0.619  1.015  1.074  0.938  
 
 SEMA6C  10500  1  151104162  151119140  1q21.2  0.467  0.112  0.312  0.516  0.577  0.48  0.404  0.607  0.674  0.959  0.655  0.248  0.216  0.6  0.652  0.142  0.168  0.159  0.549  0.098  0.416  0.578  0.42  0.128  0.373  0.435  0.464  0.49  0.391  0.393  0.184  0.601  0.541  0.625  0.476  0.517  0.375  0.758  0.334  1.016  0.139  0.528  1.083  1.29  0.275  0.314  0.311  0.444  0.297  0.975  0.634  1.053  0.617  0.414  0.453  0.456  0.369  0.515  0.722  0.35  
 
 TNFAIP8L2  79626  1  151129104  151132225  1q21.3  0.481  0  0.284  0.237  0.381  0  0.197  0.282  0.293  0.685  0.503  0  0.147  0.145  0.731  0.092  0.091  0.212  1.418  3.132  0.24  0.3  1.156  0.61  0  0.722  0.148  0.2  0.109  0.393  0.393  0.256  0.273  0.341  0.275  0.176  0.446  0.601  0.122  0.429  0  0.398  0.38  0  0.315  0.347  0.041  0.155  0.28  0.097  0.28  0.815  0.144  0.14  0.161  0  0.24  0  0.197  0.15  
 
 TNFAIP8L2-SCNM1  100534012  1  151129104  151142773  1q21.3  0  0  0.15  0  0  0  0.645  0  0  0  0  1.072  0  0.307  0  0  0.867  1.135  0.375  3.26  0  2.511  1.959  1.089  0.176  0.277  0.209  0.572  0.873  0.757  0.628  0  1.548  0.702  0.522  0  0.983  0.853  2.815  0.556  0  0.354  3.056  0  0  0  2.362  0.459  0  0  0  0  0.137  0  0.089  0  0.158  0  1.393  0  
 
 LYSMD1  388695  1  151132223  151138424  1q21.3  1.57  0.35  0.981  1.47  1.175  0.795  0.733  1.249  0.553  1.242  1.216  0.494  1.016  0.989  1.157  0.425  1.121  0.392  2.077  0.743  0.733  1.005  0.818  0.507  0.773  1.248  0.614  0.906  0.875  0.86  1.394  1.53  1.181  0.84  0.619  1.185  0.922  1.226  0.871  0.985  0.265  0.897  1.497  0.78  0.757  1.296  1.092  0.603  0.786  0.965  1.044  0.595  1.044  0.753  1.127  1.306  0.719  0.905  0.737  0.679  
 
 SCNM1  79005  1  151138497  151142773  1q21.3  2.551  2.005  1.584  2.394  2.903  2.097  2.949  2.532  1.764  2.562  2.832  1.189  1.846  2.016  1.313  2.064  2.046  0  3.629  0.788  1.977  1.898  1.735  1.766  1.82  1.786  1.772  1.204  0.973  1.374  2.474  2.881  1.792  1.037  1.479  2.31  1.545  1.702  0.808  2.074  2.419  1.729  1.69  1.838  1.95  2.542  0.674  1.565  1.788  3.309  2.168  1.983  1.923  1.903  2.861  2.198  1.968  2.281  2.345  1.832  
 
 TMOD4  29765  1  151142462  151148547  1q12  0.647  0.235  0.327  0  0.494  0.285  0.661  0.081  0.128  0.324  0.248  0.21  0.286  0.672  0.567  0.119  0.114  0.305  0.649  0.433  0  0.759  0.27  0.185  0.093  0.809  0.455  0.475  0.123  0.36  0.325  0.181  0.416  0.173  0.128  0.681  0.307  0.417  0.171  0.454  0.242  0.195  0.259  0.367  0.171  0.062  0.24  0.436  0.233  0.172  0.157  0.282  0.77  0.2  0.126  0.245  0.36  0.164  0.232  0.219  
 
 VPS72  6944  1  151148775  151162689  1q21  3.867  1.944  2.82  4.359  3.571  2.976  4.083  3.005  3.673  3.375  3.737  2.768  3.114  2.974  3.115  2.976  2.848  1.131  4.349  2.595  3.431  3.137  3.729  3.391  3.188  2.914  3.123  3.017  2.539  4.119  3.522  3.834  3.371  2.581  2.639  3.546  3.556  3.494  3.03  3.897  3.317  3.724  5.198  3.089  3.201  3.271  2.5  2.68  3.311  3.676  3.576  3.152  2.813  2.417  3.439  3.538  2.903  3.456  3.74  3.288  
 
 PIP5K1A  8394  1  151171020  151222007  1q21.3  3.843  2.281  1.783  2.027  3.263  2.762  3.348  4.048  1.579  3.27  4.278  1.686  2.867  3.693  2.881  2.493  2.935  0.599  3.956  1.627  1.662  2.5  1.042  2.125  3.298  3.411  2.059  2.67  2.503  2.278  2.578  3.484  2.944  1.071  2.045  3.824  4.013  3.197  3.135  4.145  3.366  1.944  4.601  3.65  2.623  3.082  1.306  3.234  3.388  4.494  3.448  3.213  2.99  2.794  3.676  3.314  3.001  3.47  3.348  3.518  
 
 PSMD4  5710  1  151227196  151239954  1q21.3  6.572  6.241  4.049  4.838  5.532  4.268  6.379  5.218  4.083  5.176  6.129  4.832  5.082  6.266  4.568  4.746  4.786  5.554  6.724  5.405  5.04  5.26  4.486  4.955  4.767  5.045  5.255  5.029  4.999  5.27  5.719  6.058  5.625  3.46  4.701  5.654  5.915  5.516  5.411  6.545  5.433  4.888  6.772  4.993  5.044  5.154  6.094  4.415  4.694  6.658  5.386  4.875  5.073  4.488  5.719  5.73  4.731  5.151  5.215  5.182  
 
 ZNF687  57592  1  151254030  151264381  1q21.3  3.612  0.163  1.264  2.447  3.007  1.384  2.349  2.898  2.233  2.87  3.015  2.298  2.876  2.146  3.237  2.443  2.423  0.128  2.369  0.873  2.159  1.839  1.312  2.168  1.72  2.692  2.187  3.085  2.461  3.303  2.541  2.734  3.184  1.417  1.793  3.014  3.159  2.772  1.137  3.866  2.974  2.357  2.582  2.346  1.899  2.504  0.222  1.976  2.341  2.363  2.537  2.788  2.407  1.768  2.388  2.91  2.208  2.087  2.636  2.488  
 
 PI4KB  5298  1  151264272  151300191  1q21  4.32  1.495  2.284  3.453  3.266  2.124  4.042  3.816  3.534  3.886  3.892  2.508  3.008  2.963  3.075  2.763  2.52  0.618  4.036  2.301  2.888  2.901  2.774  2.867  3.092  3.44  3.39  3.228  2.937  4.004  3.389  3.765  3.591  2.324  1.941  3.422  3.727  3.497  3.26  3.905  2.882  3.124  3.901  2.666  2.417  3.149  2.728  2.309  2.813  3.704  3.755  2.745  2.853  2.743  2.811  3.268  3.045  2.517  3.001  3.037  
 
 RFX5  5993  1  151313115  151319769  1q21  3.691  2.851  1.62  3.051  2.853  1.069  2.415  2.597  1.909  2.06  2.492  1.889  2.59  3.211  2.554  2.29  2.239  2.694  4.592  3.077  1.611  3.346  2.969  2.707  2.201  2.459  3.275  2.881  2.446  3.307  2.529  2.588  3.43  2.538  1.676  2.995  2.358  2.745  2.653  3.495  1.214  1.927  3.086  2.797  1.523  2.279  2.412  2.194  1.993  3.403  1.934  2.453  2.021  1.81  2.793  2.688  2.302  2.674  2.81  2.512  
 
 GBAT2  101927886  1  151319485  151320367  -  0.822  2.342  0.597  1.164  0.962  0.664  1.19  0.389  0  0.365  0.686  0  0  1.146  0  0  0.785  2.452  4.591  2.748  0.685  3.789  0.863  1.158  0.584  0.695  0.416  0.747  0.451  0.42  1.341  0.803  0.679  0  1.04  0.809  0.564  0.497  1.31  0.614  0  0.254  3.284  1.056  0  0.935  0  0  0  1.792  0.331  1.12  0.436  0.366  0.551  1.77  0  0.986  0.502  0.574  
 
 SELENBP1  8991  1  151336777  151345210  1q21.3  6.061  0.933  0.23  0.732  4.451  0  0.288  0.94  0.549  3.097  3.279  2.944  2.833  0.075  2.882  2.064  1.3  0.487  0.141  0  2.768  0.167  0.569  0.078  0  4.236  0.209  0.112  0  0.101  0.635  1.501  0.109  0.473  1.564  2.915  2.193  1.698  0.145  0.232  0.15  4.096  0.206  0.123  0.852  1.291  3.222  0  0  0  2.949  0.374  0  2.209  0.056  2.152  0.265  0  1.143  0.833  
 
 PSMB4  5692  1  151372040  151374412  1q21  7.879  8.193  5.256  6.058  7.59  5.894  8.311  7.222  5.575  6.966  7.864  6.352  6.36  8.078  6.143  6.532  6.056  7.152  8.501  7.245  6.528  6.737  6.305  6.663  6.462  7.301  7.504  7.382  7.213  6.494  7.899  8.006  7.674  5.425  5.857  7.195  6.994  6.913  7.027  8.129  6.873  6.578  7.769  6.72  6.574  7.078  6.931  6.222  6.808  8.298  6.82  6.492  6.656  5.937  7.089  7.366  6.268  7.836  7.122  6.922  
 
 POGZ  23126  1  151375199  151431941  1q21.3  4.457  2.821  1.775  2.395  3.097  1.989  2.765  3.225  2.338  3.119  3.265  1.803  2.421  3.328  3.382  2.669  2.548  1.935  3.754  2.846  2.16  3.566  2.19  2.078  2.162  3.298  2.676  3.048  2.346  3.046  2.897  2.622  2.939  1.617  2.189  3.435  3.418  3.018  2.393  3.815  3.025  2.272  3.557  2.608  2.139  2.76  2.63  2.428  2.641  3.026  2.773  2.762  2.625  1.86  2.988  2.952  2.937  2.496  3.124  2.631  
 
 CGN  57530  1  151483861  151511167  1q21  2.662  0.102  0.247  0.214  2.837  0.119  0.221  0.083  0.346  0.383  0.925  2.193  2.996  1.454  2.248  2.136  1.278  0.203  0.6  0.113  0.042  0.791  0.396  0.256  0.074  1.117  0.178  0.757  0.339  1.056  0.112  0.124  0.217  0.301  0.565  1.225  0.587  0.079  0.307  2.191  3.21  0.294  1.563  0.513  1.793  2.954  0.318  0.559  1.547  1.403  0.338  1.273  0.265  0.154  0.529  0.226  0.113  0.647  0.801  0.968  
 
 TUFT1  7286  1  151512780  151556059  1q21  2.042  2.206  1.873  3.008  2.887  2.104  1.325  3.585  1.421  1.231  3.314  1.883  1.445  1.528  1.126  1.811  1.494  1.384  0.111  0.407  0.242  0.524  1.269  0.858  0  1.214  0.573  0.97  0.88  0.529  1.256  0.808  1.399  0.791  1.195  3.199  1.8  1.915  2.973  1.76  2.351  0.683  3.072  1.858  2.072  3.545  0.958  2.437  3.224  4.169  1.967  2.71  1.913  2.242  3.883  2.738  2.802  3.278  3.204  2.917  
 
 SNX27  81609  1  151584661  151671559  1q21.3  3.166  0.97  1.584  1.709  2.481  1.383  2.793  2.635  2.201  2.345  2.554  1.878  2.508  2.426  2.56  1.978  2.171  0.718  2.694  1.722  1.43  1.832  1.139  1.515  1.993  2.592  2.026  2.458  2.434  2.77  2.682  2.628  2.729  1.546  1.795  2.821  2.431  2.783  1.636  3.393  2.316  1.754  2.805  2.253  1.693  2.005  1.95  1.843  2.186  2.245  2.655  2.26  2.113  1.763  2.41  2.673  1.852  1.976  2.415  2.803  
 
 CELF3  11189  1  151672533  151689296  1q21  0.426  0.028  0.125  0  0.069  0  0.059  0.074  0.086  0.044  0.079  0.059  0.041  0.069  0.108  0.067  0.035  0.038  0.083  0.07  0  0.107  0.465  0  0  0.058  0.072  0.034  0.032  0.179  0.058  0.031  0.058  0.193  0.036  0.039  0.08  0.089  0.062  0.091  0.04  0.114  0.09  0.046  0.04  0.06  0.024  0.038  0.043  0  0.07  0.326  0.065  0.088  0.068  0.051  0.025  0.047  0.068  0  
 
 RIIAD1  284485  1  151694012  151702082  1q21.3  0  0  0  0  0  0  0  0  0  0  0  0  0  0  0  0  0  0  0  0  0  0  0  0  0  0  0.117  0  0  0  0  0  0  0  0  0  0  0  0  0  0  0  0  0  0  0  0  0  0  0  0  0  0  0  0  0  0  0  0  0  
 
 MRPL9  65005  1  151732118  151736392  1q21  4.998  5.933  2.634  3.885  4.799  3.112  4.105  4.149  3.133  3.798  4.421  3.742  3.678  4.76  3.214  3.816  4.025  6.724  5.82  5.371  3.635  4.779  3.424  3.339  3.939  3.251  3.098  2.902  3.058  2.829  3.822  4.06  3.9  2.629  3.135  4.056  4.055  3.745  4.498  4.205  4.034  3.784  4.783  3.701  3.349  3.588  3.79  3.604  2.854  5.471  3.867  3.686  3.783  3.062  4.775  4.115  3.368  4.15  4.483  3.73  
 
 OAZ3  51686  1  151735444  151743806  1q21.3  1.422  0  0.43  0.942  2.179  0.735  0.797  0.808  0.854  0.967  0.868  0.354  0.393  0.385  0.368  0.633  0.724  0  0.37  0.137  0  0.39  0.778  0.29  0.311  0.771  1.005  0.439  1.165  0.366  0.814  0.798  0.365  0.332  0.315  0.516  0.465  0.279  0.586  0.595  0.614  1.311  1.632  0  0.484  0.404  0.195  0.452  0  0.853  0.408  1.198  0.318  0.428  0.795  0  0.263  1.033  0.48  0.458  
 
 TDRKH  11022  1  151744040  151763010  1q21  1.842  0.653  0.165  1.813  1.993  0.913  1.003  0.643  0.646  0.989  0.943  0.927  1.014  0.532  1.215  1.148  0.752  0.376  2.737  0.501  0.695  1.376  1.309  1.035  0.597  1.424  0.442  0.543  0.952  0.303  0.734  0.303  1.221  0.811  0.843  1.47  1.489  0.92  0.187  2.53  0.885  0.565  1.296  0.664  1.097  1.866  1.158  0.849  0.559  1.833  1.69  1.661  1.033  0.523  1.28  1.336  0.794  1.943  1.412  1.263  
 
 LINGO4  339398  1  151772739  151777918  1q21.3  0.082  0.05  0.191  0  0.204  0.063  0  0  0.144  0.05  0.035  0  0  0.041  0  0.036  0  0.069  0.23  0.048  0  0.112  0.597  0  0  0  0.058  0.034  0  0.056  0  0  0  0.287  0  0  0  0  0.039  0  0  0.248  0.042  0  0  0.041  0.046  0  0  0  0  0.292  0.022  0.031  0.03  0  0  0  0  0  
 
 RORC  6097  1  151778546  151804348  1q21  0.443  0  0.123  0  1.461  0  0  0.11  0.251  0.02  0  0.351  0.166  0.172  0  0.089  0  0  2.512  0  0  0.599  0.565  0.074  0  0.247  0.122  0.05  0  0.066  0  0.055  0  0.148  0  0.075  0  0.462  0.192  0.141  0  0.454  0.098  0  0.198  0.263  0.045  0  0  0.04  0.068  0.311  0.027  0.096  0.032  0  0  0  0  0  
 
 C2CD4D  100191040  1  151810338  151813033  1q21.3  0.668  0.075  0.219  0  0.31  0.094  0.069  0.113  0.314  0.156  0.204  0.593  0.165  0.112  0.152  0.256  0.338  0.22  0.051  0.136  0  0.356  0.569  0  0.084  0.383  0.421  0.345  0.235  0.749  0.645  0.445  0.776  0.806  0.085  0  0.161  0.393  0.164  0.142  0  0.901  0.088  0.091  0.223  0.2  0.113  0.103  0  0.126  0.073  1.054  0  0.231  0.198  0.121  0.073  0  0  0  
 
 LOC100132111  100132111  1  151810944  151816641  1q21.3  0.262  0.251  0  0  0.362  0  0  0  0  0  0  0.51  0  0  0  0  0  0  0  0  0  0  0  0  0  0.194  0  0.134  0  0  0  0  0  0  0  0.223  0.186  1.304  0  0.558  0  0  0  0  0  0.153  0  0  0  0  0  0.809  0  0  0  0  0  0  0  0  
 
 THEM5  284486  1  151819576  151826173  1q21.3  0  0  0  0  0.17  0  0  0  0.159  0  0  0.271  0  0  0  0  0  0.241  0  0  0  0  0.38  0  0  0  0.068  0  0  0.077  0  0  0  0.307  0.107  0  0  0.322  0  0  0  0.12  0.054  0  0  0.105  0  0  0  0  0  0.349  0  0  0  0  0  0  0  0  
 
 THEM4  117145  1  151843342  151882361  1q21  1.631  1.365  1.001  0.693  1.673  0.192  1.057  0.796  1.373  1.056  1.163  2.026  0.599  1.82  0.756  1.439  1.452  1.109  0.031  1.843  1.512  0.06  1.986  0.59  1.181  3.148  1.974  1.599  2.203  1.973  1.581  2.556  2.79  2.292  1.319  1.424  1.297  0.906  0.598  2.211  0.804  1.266  1.043  0.333  0.62  0.696  1.94  0.414  0.624  1.377  0.884  1.316  0.747  0.719  1.47  1.094  0.693  0.812  1.132  1.038  
 
 S100A10  6281  1  151955385  151966714  1q21  6.377  8.541  5.957  5.796  8.289  6.893  7.187  5.831  5.289  7.474  7.555  8.093  6.251  6.782  6.478  7.826  8.3  7.986  4.313  2.148  0.742  3.964  3.183  1.095  4.958  6.77  6.188  4.783  6.015  4.49  5.353  5.978  6.094  5.715  7.761  7.034  6.046  6.517  9.14  7.028  7.95  6.888  6.003  6.099  6.741  7.316  8.121  6.103  7.03  7.947  8.031  6.483  6.556  6.999  8.485  7.007  6.951  7.294  7.726  6.789  
 
 NBPF18P  441908  1  151990753  151994985  1q21.3  0  0  0  0  0.104  0  0  0  0  0.086  0  0  0  0  0  0  0  0.111  0  0  0  0  0  0  0  0  0  0.062  0  0.062  0  0  0  0  0  0  0  0  0  0  0  0  0  0  0  0.092  0  0  0  0  0  0  0  0.086  0.1  0  0  0  0  0  
 
 S100A11  6282  1  152004981  152009511  1q21  8.609  9.111  8.729  9.667  10.331  9.11  9.332  8.952  7.612  8.82  8.48  10.711  7.919  7.611  6.496  9.868  10.324  5.694  6.693  7.661  6.637  4.853  8.563  9.375  9.491  8.655  8.71  6.977  8.185  7.71  8.44  8.859  8.484  7.605  9.118  9.613  8.086  9.326  10.228  9.952  9.778  8.64  0.459  8.34  7.551  9.196  9.401  7.881  8.579  9.362  8.91  8.117  9.379  9.563  10.08  10.09  9.182  9.591  10.229  8.862  
 
 LOC100131107  100131107  1  152051888  152053400  1q21.3  0  0.071  0.181  0  0  0  0  0  0.221  0.036  0  0  0  0  0  0  0  0.123  0  0  0  0.047  0.583  0  0  0  0.124  0  0  0.069  0  0  0  0.293  0  0  0  0  0  0  0  0.141  0.043  0  0  0  0  0  0  0  0  0.209  0  0.045  0  0  0  0  0  0  
 
 TCHHL1  126637  1  152056619  152061540  1q21.3  0  0  0.12  0  0.038  0  0  0  0.113  0  0  0.048  0  0.015  0  0  0  0  0  0  0  0.036  0.367  0  0  0  0.068  0  0  0.03  0  0  0  0.368  0  0  0  0  0.012  0  0.118  0.229  0.027  0  0  0  0.016  0  0  0  0  0.162  0  0.026  0  0  0  0  0  0  
 
 TCHH  7062  1  152078792  152087930  1q21.3  0.227  0.063  0.159  0.169  0.121  0.078  0.053  0.054  0.177  0.046  0.072  0.127  0.238  0.06  0.204  0.068  0.08  0.164  0.028  0.101  0.039  0.416  0.687  0.047  0.037  0.027  0.134  0.036  0.031  0.133  0.028  0.035  0  0.364  0.087  0.451  0.029  0  0.148  0.593  0.172  0.411  0.067  0.082  0.041  0.059  0.139  0.315  0.043  0.395  0.059  0.782  0.042  0.26  0.163  0.051  0.138  0.046  0.026  0.028  
 
 RPTN  126638  1  152126070  152131704  1q21.3  0  0  0.159  0.076  0  0  0  0.022  0.099  0  0  0  0  0.014  0  0  0  0  0  0  0  0.043  0.487  0  0  0  0.106  0  0  0.029  0  0  0  0.291  0  0  0  0  0.018  0  0  0.111  0.025  0  0  0  0.011  0  0  0  0  0.109  0  0.015  0  0  0  0  0  0  
 
 HRNR  388697  1  152184551  152196672  1q21.3  0  0.218  0.161  0.062  0.02  0  0.036  0.012  0.133  0.036  0.263  0.023  0.072  0.037  0.041  0.012  0  0.036  0  0.042  0  0.086  0.715  0.028  0.786  1.331  0.066  1.342  0.457  0.167  0.779  1.144  0  0.292  0.053  0.428  0.016  0.681  0.795  1.289  0.294  0.241  0.33  0.396  0.161  0.016  0.258  1.145  0  0.366  0.193  1.144  0.155  0.109  1.044  0.02  0.287  0.257  0.23  0.269  
 
 FLG  2312  1  152274650  152297679  1q21.3  0  3.203  0.111  0.108  0.01  0.02  0.032  0.692  0.202  0.034  1.817  0.023  0.027  0.019  0.018  0  0  0.058  0.019  0.027  0.05  0.104  0.429  0  1.59  1.034  0.052  2.449  0.159  0.103  0.903  0.748  0  0.277  0.038  0.477  0.018  2.394  0.27  1.802  0.229  0.137  0.273  2.68  0.993  0.013  0.017  0.628  0  0.391  0.538  0.798  2.708  0.102  0.645  1.153  2.782  0.44  1.346  0.132  
 
 FLG-AS1  339400  1  152285934  152339168  1q21.3  0  0.396  0.116  0.184  0  0  0.07  0  0.305  0.02  0.583  0.041  0  0.025  0  0  0  0.038  0  0.037  0  0.094  0.178  0  1.989  1.132  0.051  1.066  0.066  0.324  1.739  0.748  0  0.269  0.038  0.422  0  0.154  0.552  0.975  0.094  0.231  1.082  0.155  0.066  0  0.396  0.386  0  1.505  0.41  0.408  0.465  0.07  0.986  0.042  0.322  0.545  0.588  0.12  
 
 FLG2  388698  1  152321212  152332482  1q21.3  0  0.078  0.184  0  0.009  0  0  0.009  0.096  0.01  0.069  0.016  0  0.018  0  0.008  0.027  0.091  0  0.025  0  0.066  0.54  0  0.086  0.156  0.039  0.287  0.019  0.039  0.159  0.057  0  0.271  0  0.045  0  0.022  0.11  0.053  0.024  0.214  0.021  0.033  0  0.02  0.069  0.118  0  0.034  0.04  0.211  0.068  0.029  0.169  0  0.053  0.089  0.059  0  
 
 CRNN  49860  1  152381718  152386750  1q21  0  0  0.148  0  0  0  0  0  0.145  0  0  0  0  0  0  0  0  0  0  0  0  0.068  0.664  0  0  0  0.061  0  0  0.046  0  0  0  0.282  0  0  0  0  0.1  0  0  0.111  0.106  0  0  0  0.026  0  0  0  0  0.146  0  0.037  0  0  0  0  0  0  
 
 LCE5A  254910  1  152483319  152484653  1q21.3  0  0  0  0  0  0  0  0  0  0  0  0  0  0.128  0  0  0  0  0  0  0  0  0.516  0  0  0  0.066  0  0  0.061  0  0.152  0  0.307  0  0  0  0  0  0  0  0.651  0  0  0  0  0  0  0  0.246  0  0  0  0  0  0  0  0  0  0  
 
 CRCT1  54544  1  152486977  152488481  1q21  0  0  0  0  0  0.266  0  0  0.189  0  0  0  0  0.089  0  0  0  0.257  0  0  0  0.293  0.371  0  0  0  0.133  0  0  0.118  0  0  0  0  0.2  0  0  0  0.145  0  0.179  0  0.113  0  0  0.148  0  0  0  0  0  0.682  0  0  0  0  0  0  0  0  
 
 LCE3E  353145  1  152538174  152539229  1q21.3  0  0  0  0  0  0  0  0  0  0  0  0  0  0  0  0  0  0  0  0  0  0  0  0  0  0  0.194  0  0  0.18  0  0  0  0  0  0  0  0  0.119  0  0  0.183  0  0  0  0  0  0  0  0  0.208  0.634  0  0  0  0  0  0  0  0  
 
 LCE3D  84648  1  152551859  152552980  1q21  0  0  0  0  0  0  0  0  0  0  0  0  0  0  0  0  0  0  0  0  0  0  0  0  0  0  0  0  0  0.07  0  0  0  0  0.239  0  0  0  0.135  0  0  0.181  0  0  0  0  0.214  0  0  0  0  0.683  0  0  0  0  0  0  0  0  
 
 LCE3C  353144  1  152573137  152573562  1q21.3  0  0  0  0  0  0  0  0  0  0  0  0  0  0  0  0  0  0  0  0  0  0  0  0  0  0  0.098  0  0  0  0  0  0  0  0  0  0  0  0  0  0  0  0  0  0  0  0  0  0  0  0  0  0  0  0  0  0  0  0  0  
 
 LCE3B  353143  1  152586286  152586574  1q21.3  0  0  0  0  0  0  0  0  0  0  0  0  0  0  0  0  0  0  0  0  0  0  0  0  0  0  0  0  0  0  0  0  0  0  0  0  0  0  0  0  0  0  0  0  0  0  0  0  0  0  0  0.99  0  0  0  0  0  0  0  0  
 
 LCE3A  353142  1  152595309  152595579  1q21.3  0  0  0.704  0  0  0  0  0  0  0  0  0  0  0  0  0  0  0  0  0  0  0.307  0  0  0  0  0.28  0  0  0.381  0  0  0  0  0  0  0  0  0  0  0  0  0  0  0  0  0.216  0  0  0  0  1.346  0  0  0  0  0  0  0  0  
 
 LCE2D  353141  1  152635886  152637135  1q21.3  0  0  0  0  0  0  0  0  0  0  0  0  0  0  0  0  0  0  0  0  0  0  0  0  0  0  0.108  0  0  0.071  0  0  0  0.436  0  0  0  0  0  0  0  0.169  0  0  0  0  0  0  0  0  0  0  0  0  0  0  0  0  0  0  
 
 LCE2C  353140  1  152647790  152649049  1q21.3  0  0  0  0  0  0  0  0  0  0  0  0  0  0  0  0  0  0  0  0  0  0  0  0  0  0  0.129  0  0  0.087  0  0  0  0  0  0  0  0  0  0  0  0.241  0  0  0  0  0.078  0  0  0  0  0  0  0  0  0  0  0  0  0  
 
 LCE2B  26239  1  152658598  152659876  1q21  0  0  0  0  0  0  0  0  0  0  0  0  0  0  0  0  0  0  0  0  0  0.162  0  0  0  0  0.158  0  0  0.099  0  0  0  0  0  0  0  0  0  0  0  0.149  0  0  0  0  0  0  0  0  0  0  0  0  0  0  0  0  0  0  
 
 LCE2A  353139  1  152670839  152671918  1q21.3  0  0  0  0  0  2.01  0.239  0  0  0  0  0  0  0  0  0  0  0  0  0  0  0.183  0.589  0  0  0.654  0.413  0.27  1.349  0.558  2.306  1.066  1.015  1.08  0  0  0  0  0  0  0  0  0  0  0  0  0.093  0  0  0.491  0  0  0  0  0  0  0  0  0  0  
 
 LCE4A  199834  1  152681522  152681910  1q21.3  0  0  0  0  0  0  0  0  0  0  0  0  0  0  0  0  0  0  0  0  0  0  0  0  0  0  0  0  0  0  0  0  0  0.541  0  0  0  0  0  0  0  0.721  0  0  0  0  0  0  0  0  0  0  0  0  0  0  0  0  0  0  
 
 C1orf68  100129271  1  152691997  152692905  1q21.3  0  0  0  0  0  0  0  0  0  0  0  0  0  0  0  0  0  0  0  0  0  0  0.551  0  0  0  0.158  0  0  0.12  0  0  0  0  0  0  0  0  0  0  0  0.752  0  0  0  0  0.048  0  0  0  0  0  0  0  0  0  0  0  0  0  
 
 KPRP  448834  1  152730505  152734529  1q21.3  0  0  0.164  0  0.066  0.101  0  0  0.119  0.035  0.033  0.067  0  0.019  0  0  0  0  0  0.057  0  0.12  0.479  0  0  0  0.187  0.09  0  0.097  0  0  0  0.294  0  0  0  0  0.021  0  0  0.265  0.043  0  0  0  0.087  0  0  0.074  0  0.355  0  0.067  0.031  0  0  0.757  0  0  
 
 LCE1F  353137  1  152748847  152749445  1q21.3  0  0  0.315  0  0  0  0  0  0.277  0  0  0  0  0  0  0  0  0  0  0  0  0  0.616  0  0  0  0.131  0  0  0.098  0  0  0  0.397  0  0  0  0  0.871  0  0  1.241  0  0  0  0  0.14  0  0  0  0  0  0  0  0  0  0  0.523  0  0  
 
 LCE1E  353135  1  152758752  152760901  1q21.3  0  0  0  0  0  0  0  0  0  0  0  0  0  0  0  0  0  0  0  0.111  0  0.083  0.491  0  0  0  0.084  0.067  0  0.075  0  0  0  0.191  0  0  0  0  0.469  0  0  0  0  0  0  0  0  0  0  0  0  0  0  0  0  0  0  0  0  0  
 
 LCE1D  353134  1  152769226  152770657  1q21.3  0  0  0  0  0  0  0  0  0  0  0  0  0  0  0  0  0  0  0  0  0  0  0  0  0  0  0.279  0  0  0.153  0  0  0  0  0  0  0  0  0  0  0  0  0  0  0.453  0  0.27  0  0  0  0  0  0  0.288  0  0  0  0  0  0  
 
 LCE1C  353133  1  152777310  152779107  1q21.3  0  0  0  0  0  0  0  0  0.224  0  0  0  0  0  0  0  0  0  0  0  0  0  0  0  0  0  0.081  0  0  0.057  0  0  0  0  0  0  0  0  0.765  0  0  0  0  0  0  0  0.102  0  0  0  0  0.272  0  0  0  0  0  0  0  0  
 
 LCE1B  353132  1  152784446  152785585  1q21.3  0  0  0  0  0.074  0  0  0  0.129  0  0  0.247  0  0  0  0  0  0  0  0  0  0.163  0.406  0  0  0  0.056  0  0  0.055  0  0  0  0.324  0  0  0  0  0.064  0  0.119  0.054  0.049  0  0  0  0.111  0  0  0  0  0.357  0  0.137  0.067  0  0  0  0  0  
 
 LCE1A  353131  1  152799948  152800573  1q21.3  0  0  0  0  0  0  0  0  0.214  0  0  0  0  0  0  0  0  0  0  0  0  0.11  0.555  0  0  0  0.06  0  0  0  0.409  0  0  0.489  0  0  0  0  0  0  0  0.131  0  0  0  0  0  0  0  0  0  0.412  0  0  0  0  0  0  0  0  
 
 LCE6A  448835  1  152815329  152816459  1q21.3  0  0  0  0  0  0  0  0  0.207  0  0  0  0  0  0  0  0  0  0  0.217  0  0.136  0  0  0  0  0  0  0  0  0  0  0  0.4  0  0  0  0  0  0  0  0.649  0  0  0  0  0  0  0  0  0  0  0  0  0  0  0  0  0  0  
 
 SMCP  4184  1  152850797  152857523  1q21.3  0  0  0.22  0  0  0  0  0  0  0  0  0  0  0  0  0  0  0  0  0  0  0  0.498  0  0  0  0  0  0  0.467  0  0  0  0.41  0  0  0  0  0.108  0  0  0.108  0  0  0  0  0  0  0  0  0  0  0  0  0  0  0  0  0  0  
 
 IVL  3713  1  152881038  152884362  1q21  0  0.057  0.232  0  2.542  0  0  0  0.14  0.033  0  0  0  0  0  0.394  0.053  0.249  0.037  0  0  0.033  0.441  0  0.503  0  0.036  0  0  0.029  0  0  0  0.232  0  0  0  0  1.091  0  2.059  0.562  0  0  0  0.06  0  0  0  0  0  0.41  0  0.045  0  0  0.143  0  0  0  
 
 SPRR4  163778  1  152943128  152945049  1q21.3  0  0  0.192  0  0  0  0  0  0  0  0  0  0  0  0  0  0  0  0  0  0  0  0.645  0  0  0  0  0  0  0  0  0  0  0.296  0  0  0  0  0.065  0  0  0  0  0  0  0  0  0  0  0  0  0.263  0  0  0  0  0  0  0  0  
 
 SPRR1A  6698  1  152956563  152958290  1q21-q22  0  0  0  0  0.126  0  0  0  0  0  0  5.403  0  0  0  0.362  0  0  0  0  0  0  0  0.334  0  0  0  0  0  0  0  0  0  0.35  0  0.247  0  0  0  0  1.274  0  0  0  0  0  0  0  0  0  0  0  0  0  0  0  0  0  0  0  
 
 SPRR3  6707  1  152974222  152976332  1q21-q22  0  0  0  0  0.084  0  0  0  0.167  0  0  2.914  0  0  0  0.125  0  0  0  0  0  0  0.423  0  0  0  0.155  0  0.168  0  0.283  0  0  0.414  0  0.163  0  0  0  0  5.029  0  0  0  0  0  0  0  0  0  0  0  0  0  0  0  0  0  0  0  
 
 SPRR1B  6699  1  153003678  153005376  1q21-q22  0  0  0.238  0  0  0  0  0  0.269  0  0  2.002  0  0  0  0.415  0  0  0  0  0  0  0.527  0  0  0  0.086  0  0  0  0  0  0  0  0  0  0  0  0  0  3.482  0  0  0  0  0  0  0  0  0  0  0  0  0  0  0  0  0  0  0  
 
 SPRR2D  6703  1  153012200  153013594  1q21-q22  0  0  0  0  0  0  0  0  0  0  0  0.848  0  0  0  0  0  0  0  0  1.051  0  0.933  0  0  0.701  3.676  0  3.746  1.553  2.655  0.779  0.395  0.883  0  0  0  0.579  1.285  0  0.305  0  0  0  0  0  0  0  0  0  0  0  0  0  0  0  0  0  0  0  
 
 SPRR2A  6700  1  153028595  153029988  1q21-q22  0  0  0  0  0  0  0  0  0  0  0  0.16  0  0  0  0  0  0  0  0  0  0  0.505  0  0  0  0.613  0  0.542  0.224  0.243  0  0  0.467  0  0  0  0  0  0  0.565  0  0  0  0  0  0  0  0  0  0  0  0  0  0  0  0  0  0  0  
 
 SPRR2B  6701  1  153042703  153044084  1q21-q22  0  0  0  0  0  0  0  0  0  0  0  0  0  0  0  0  0  0  0  0  0  0  0  0  0  0  0.968  0  0.602  0.284  0.262  0  0  0  0  0  0  0  0  0  0  0  0  0  0  0  0  0  0  0  0  0  0  0  0  0  0  0  0  0  
 
 SPRR2E  6704  1  153065610  153067004  1q21-q22  0  0  0  0  0  0  0  0  0  0  0  0  0  0  0  0  0  0  0  0  0  0  0  0  0  0  0.926  0  0.654  0.314  0.293  0  0  0  0  0  0  0  0.089  0  0  0  0  0  0  0  0  0  0  0  0  0  0  0  0  0  0  0  0  0  
 
 SPRR2F  6705  1  153084599  153085991  1q21-q22  0  0  0  0  0  0  0  0  0  0  0  0.627  0  0  0  0  0  0  0  0  0  0  0.515  0  0  0  0.881  0  0.668  0.176  0.305  0  0  0.413  0  0  0  0  0  0  0.273  0.255  0  0  0  0  0  0  0  0  0  0  0  0  0  0  0  0  0  0  
 
 SPRR2C  6702  1  153112593  153113969  1q21-q22  0  0  0.263  0  0  0.444  0  0  0  0  0  0  0  0  0  0  0  0  0  0  0  0.092  0  0  0  0  0.106  0  0.225  0  0  0  0  0.314  0  0  0  0  0  0  0  0  0  0  0  0  0  0  0  0  0  0  0  0  0  0  0  0  0  0  
 
 SPRR2G  6706  1  153122057  153123427  1q21-q22  0  0  0  0  0  0.61  0  0  0  0  0  0  0  0  0  0  0  0  0  0  0  0  0  0.328  0.914  0  0.165  0  0.275  0.464  0.3  0  0  0  0  0  0  0  0  0  0  0  0  0  0  0  0  0  0  0  0  0  0  0  0  0  0  0  0  0  
 
 LOC101928009  101928009  1  153146993  153164152  -  0  0  0  0  0  0  0  0  0.093  0  0  0  0  0  0  0  0  0.097  0  0  0  0  0.307  0  0  0  0.057  0  0  0.216  0  0  0  0.233  0  0  0  0  0  0  0  0.251  0.022  0  0  0  0.024  0  0  0  0  0  0  0  0  0  0  0  0  0  
 
 LELP1  149018  1  153175905  153177601  1q21.3  0  0  0.28  0  0  0  0  0  0  0  0  0  0  0  0  0  0  0.404  0  0.194  0  0  0.858  0  0  0  0  0  0  0  0  0  0  0.362  0  0  0  0  0  0  0  0  0  0  0  0  0  0  0  0  0  0  0  0  0  0  0  0  0  0  
 
 PRR9  574414  1  153190059  153191793  1q21.3  0  0  0.153  0  0  0.311  0  0  0.1  0  0  3.418  0  0  0  0  0  0  0  0  0  0.037  0.472  0  0  0  0.054  0  0  0.036  0.146  0  0  0.422  0  0  0  0  0.635  0  0  0  0  0  0  0  0  0  0  0  0  0.152  0  0  0  0  0  0  0  0  
 
 LOR  4014  1  153232178  153234600  1q21  0  0.095  0  0  0.057  0.155  0  0.102  0  0.069  0  0.082  0  0.059  0  0.144  0  0.341  0.104  0.157  0  0.472  0.237  0  0  0  0.128  0.095  0  0.119  0  0  0  0.173  0  0  0  0  0.13  0  0  0.3  0.086  0  0  0.118  0.054  0  0  0  0  0.825  0  0.182  0.197  0  0  0  0  0  
 
 PGLYRP3  114771  1  153270337  153283194  1q21  0  0  0  0  0.06  0.101  0  0  0  0  0  0  0  0  0  0  0  0  0  0  0  0  0.445  0  0  0  0.065  0  0  0.054  0  0  0  0.224  0  0  0  0  0  0  0  0  0  0  0  0  0  0  0  0  0  0.343  0  0  0  0  0  0  0  0  
 
 PGLYRP4  57115  1  153302596  153321317  1q21  0  0  0.15  0  0.047  0  0  0  0  0  0  0.058  0  0  0  0  0  0.061  0.055  0  0  0.053  0.468  0.129  0  0  0.049  0  0  0.045  0  0  0  0.189  0  0  0  0  0.027  0  0  0.373  0.019  0  0  0  0  0  0  0  0  0.375  0  0.081  0.053  0  0  0  0  0  
 
 S100A9  6280  1  153330329  153333503  1q21  0.516  0.79  1.025  1.171  3.085  0  0  0  1.891  0.135  0  0  0  0  0  0.588  0.464  0.592  0  3.993  0  0.154  2.451  0.414  0  0  0.518  0  0  0.107  0  0  0  1.182  0  5.104  0  0  0.237  3.415  8.019  2.302  0  0.581  0.717  2.676  0  0  0  0  0.338  0.343  0  0  0.2  0  0  0  0.672  0  
 
 S100A12  6283  1  153346183  153348075  1q21  0  0  0  0  0  0  0  0  0  0  0  0  0  0  0  0  0  0  0  1.589  0  0  0  0  0  0  0  0  0  0  0  0  0  0  0  0.321  0  0  0  0  0  0  0  0  0  0  0  0  0  0  0  0  0  0  0  0  0  0  0  0  
 
 S100A8  6279  1  153362507  153363664  1q21  0  0.374  0.343  0  1.808  0  0  0  0.329  0.718  0  0.219  0  0.151  0  0.556  0.777  0  0  5.005  0  0  0  0  0  0  0.194  0  0  0  0  0  0  0  0  0.75  0  0  0.329  1.191  0.736  0  0  0  0  0  0.246  0  0  0.302  0  0  0.157  0  0.525  0.52  0  0  0  0  
 
 S100A7A  338324  1  153388999  153395701  1q21.3  0  0  0.102  0  0.034  0  0  0.022  0.165  0  0.03  0.029  0  0.022  0  0.081  0.063  0  0  0.027  0  0.05  0.532  0  0  0  0.05  0  0  0.039  0  0  0  0.294  0  0  0  0  0.039  0  0  0.172  0.028  0  0  0  0.028  0  0  0  0  0.108  0.024  0.017  0.026  0  0  0  0  0  
 
 S100A7  6278  1  153430219  153433137  1q21  0  0  0  0  0  0  0  0  0  0  0  0  0  0.342  0  0  0  0  0  0  0  0  0  0  0  0  0  0  0  0  0  0  0  0  0  0  0  0  0.214  0.614  0  0.332  0  0  0  0  0  0  0  0  0  0  0  0  0  0  0  0  0  0  
 
 S100A6  6277  1  153507075  153508717  1q21  6.183  12.07  8.531  8.57  9.49  9.038  10.292  8.153  9.614  9.74  9.595  10.619  8.249  9.991  6.308  11  12.236  9.536  5.574  6.757  3.329  5.378  6.143  7.53  7.47  8.632  8.12  6.884  4.861  1.86  7.803  8.815  7.855  8.185  9.637  9.809  6.959  9.22  10.6  8.963  0.25  9.858  0.747  7.642  8.429  7.301  9.688  7.202  10.45  9.567  8.713  8.156  8.307  9.235  10.92  9.646  8.26  7.945  9.298  7.368  
 
 S100A5  6276  1  153509622  153514241  1q21  0  1.17  0.259  0  0  0  0.196  0  0  0.218  0.136  2.992  0.809  1.892  0.358  2.283  0.231  0  0.566  0  0  0.082  0.702  0  0  0.191  0.309  0.75  0  0  0  0  0  0.336  0  0  0.395  0.99  0.114  0.792  0  0.722  1.02  0.313  0.245  0.457  0  0  1.696  0.615  0.956  0.301  0.489  0  0.678  0  0.322  0  0.91  0  
 
 S100A4  6275  1  153516094  153518282  1q21  2.531  6.883  5.015  2.702  5.658  0.781  2.152  5.886  2.675  1.913  2.639  8.204  4.532  7.52  0.977  9.115  4.059  4.841  7.445  6.768  2.364  0.313  6.266  7.88  0.508  7.127  2.251  1.487  0  0.12  1.172  2.937  1.23  2.37  7.217  5.796  1.579  8.45  0.917  4.343  1.882  7.771  0.118  8.155  4.341  4.485  7.085  0  8.79  1.6  5.238  1.79  0.742  1.204  3.021  2.106  4.192  1.209  1.241  0  
 
 LOC101928034  101928034  1  153518217  153524245  1q21.3  0  0.101  0.503  0  0.165  0.108  0.343  0.123  0.129  0.104  0.186  0.851  0.221  0.466  0  1.853  0  0.234  0.508  0.177  0.162  0.097  1.02  1.239  0.176  0.42  0.684  0.047  0  0.13  0  0.211  0  0.548  0.568  0.316  0.183  1.389  0.106  0.271  0  0.984  0.061  0.628  0.121  0.087  0.033  0  1.443  0.098  0.324  0.464  0.211  0.314  0.287  0.173  0.265  0  0.61  0  
 
 S100A3  6274  1  153519808  153521734  1q21  0  0.726  2.416  0.581  0.123  1.022  3.792  1.425  0.637  1.564  1.765  1.25  0  1.945  0  1.699  0.605  0.163  0  0.14  0  0.177  0.7  0  1.823  1.727  2.689  0.362  0  0.153  0.565  1.954  2.616  2.841  3.429  3.254  1.202  2.228  1.417  0.526  0.167  1.944  0.086  0  1.138  0.749  2.813  0.934  2.891  2.314  3.387  1.428  1.712  2.079  2.903  1.677  1.763  1.574  2.731  0.827  
 
 S100A2  6273  1  153533584  153538306  1q21  0.303  3.512  1.27  3.028  1.078  3.895  2.851  4.296  3.232  2.087  6.904  1.709  1.189  2.342  1.213  5.837  3.809  0.751  0.608  0.12  0  1.443  0.635  0.167  1.992  0.487  0.82  1.3  0.178  0.21  0.614  0.844  1.276  1.104  1.383  2.717  4.464  3.472  3.47  2.449  2.16  3.302  0.057  2.227  4.196  3.652  3.803  5.106  6.037  6.752  4.844  2.103  5.959  2.742  5.228  3.536  3.401  2.864  8.051  4.827  
 
 S100A16  140576  1  153579366  153585514  1q21  7.122  5.23  6.451  0  8.195  5.276  6.898  4.253  6.353  5.755  7.653  6.285  6.94  6.599  5.444  7.286  8.299  4.437  0.774  0.65  0.931  2.068  0.673  0  7.108  5.351  1.024  4.971  1.124  2.968  4.194  4.297  3.772  3.053  5.539  5.449  6.911  6.365  8.083  0.614  7.889  5.331  0.503  5.768  3.749  7.425  6.802  5.536  7.29  7.536  8.419  6.316  6.042  4.141  6.497  7.186  6.37  6.589  6.495  7.187  
 
 S100A14  57402  1  153586731  153588808  1q21.3  6.092  0  0  0  0.698  0  0.134  0.083  0.375  0.137  0.291  3.257  7.92  5.569  5.22  5.184  7.011  0.638  0  0.09  0  0.09  0.436  0  0  0  0.206  0.074  0  0.065  0  0.152  0  0.279  0  0.178  0.091  0  0.273  0  5.366  0.763  0  0.439  2.416  5.774  0.326  0  1.003  0.816  1.108  0.428  0.066  0.116  0.107  0.21  0  0  0.625  0  
 
 S100A13  6284  1  153591275  153606568  1q21  6.82  4.794  5.039  5.234  7.371  5.66  7.233  5.956  5.487  6.842  6.387  4.604  4.769  5.618  4.408  6.053  3.231  2.963  2.119  1.44  3.672  2.131  1.67  0.866  5.164  6.869  6.616  5.151  5.589  5.674  6.723  6.755  6.023  5.877  5.125  4.981  4.664  5.412  7.591  4.16  6.083  5.606  3.445  6.152  5.476  7.571  5.665  5.543  6.791  7.752  6.14  5.216  5.705  5.278  7.538  6.202  5.429  5.535  6.678  6.238  
 
 S100A1  6271  1  153600872  153604513  1q21  1.656  0.288  0.25  0.725  5.424  0.288  0.615  0.223  1.657  3.422  0.909  0.979  0.957  0.71  1.139  1.096  0.519  0  0.756  0.602  2.34  0.283  1.366  0.675  0.539  8.59  7.382  6.397  7.582  6.343  7.844  7.531  7.119  7.334  0.379  0.437  0.42  0.531  0.393  0.46  0.478  0.791  0.923  3.185  3.73  3.377  0.502  1.044  1.434  2.074  1.265  1.318  0.555  1.335  3.367  1.994  1.645  0.351  0.784  0.505  
 
 CHTOP  26097  1  153606457  153618782  1q21.3  4.274  5.024  2.911  3.712  4.133  3.067  4.718  3.611  3.691  3.754  4.192  3.386  3.709  5.115  4.047  3.923  3.993  5.204  5.535  4.944  3.533  4.847  3.553  3.33  3.203  3.252  4.089  2.793  3.454  3.8  3.855  4  3.746  2.691  3.29  3.491  3.944  3.238  4.361  4.514  3.955  3.789  4.45  3.538  3.229  4.074  4.625  3.33  3.006  5.158  3.829  3.117  3.536  2.55  4.317  3.613  3.006  4.137  3.832  3.547  
 
 SNAPIN  23557  1  153631129  153634328  1q21.3  4.651  5.189  2.876  3.331  5.197  3.622  4.408  3.57  4.251  3.87  4.564  3.345  3.026  4.907  2.899  3.712  3.757  5.194  5.172  3.881  2.312  3.777  3.876  3.567  3.325  3.817  3.276  2.831  3.053  3.452  4.04  3.744  3.654  3.082  3.406  3.962  3.069  3.493  4.311  4.394  3.777  3.626  5.131  3.756  2.991  4.055  4.381  2.736  4.201  5.079  3.654  3.447  3.007  2.915  4.329  4.017  3.076  4.428  3.615  3.477  
 
 ILF2  3608  1  153634263  153643504  1q21.3  6.403  6.555  3.997  3.637  6.098  4.256  6.534  5.722  4.018  5.292  6.281  4.855  5.32  7.38  5.379  4.859  5.474  6.119  8.058  6.42  5.538  6.49  4.402  4.869  5.175  5.32  4.432  4.585  4.503  5.125  5.732  5.951  5.068  3.329  4.667  5.617  5.908  5.325  5.735  6.793  5.603  4.511  6.89  5.459  4.907  5.882  5.745  4.872  5.068  6.881  5.772  4.899  5.319  4.451  5.549  5.841  5.088  5.452  5.73  5.408  
 
 NPR1  4881  1  153651163  153666468  1q21-q22  0.147  0.119  0.195  0.164  0.161  0.09  0.177  0.088  0.156  0.107  0.091  0.106  0.164  0.143  0.817  0.154  0.214  0.263  0.094  0.126  0.191  0.28  0.381  0.113  0.075  3.56  0.458  0.071  0.086  0.265  0.183  0.061  0.06  0.269  0.18  0.875  0.535  0.057  0.181  1.27  0.174  0.977  0.14  0.258  1.914  1.434  0.284  0.62  0.28  1.425  0.156  0.9  0.088  0.19  0.411  0.796  0.365  0.828  0.09  0.101  
 
 INTS3  65123  1  153700566  153746555  1q21.3  3.615  1.392  1.655  2.459  2.903  1.937  2.706  3.05  2.412  3.133  3.245  2.409  2.968  2.87  3.518  2.548  2.574  0.565  3.661  2.049  2.106  2.53  1.792  2.24  2.4  3.466  2.827  3.184  3.989  3.118  3.67  3.338  3.663  1.956  2.003  4.088  3.442  2.639  2.43  4.475  3.115  2.704  3.778  3.227  2.196  3.357  2.256  2.757  2.162  3.519  3.297  2.917  2.377  2.334  2.583  3.404  3.224  2.261  2.878  2.504  
 
 SLC27A3  11000  1  153747767  153752633  1q21.3  3.195  0.043  0.511  0.557  2.045  0.173  0.67  1.117  1.523  1.167  0.937  1.244  1.871  1.091  2.292  1.419  1.022  0.334  0.788  0.712  0.161  0.417  0.499  0.172  0.542  6.087  3.075  2.75  4.643  1.247  5.404  2.576  1.347  0.362  0.356  0.861  0.878  0.564  0.487  1.131  0.556  0.636  1.611  1.015  1.653  1.906  1.571  0.385  0.09  1.303  1.001  1.459  0.917  0.974  0.636  0.848  1.014  0.414  1.364  0.125  
 
 LOC343052  343052  1  153766422  153768778  1q21.3  0  0  0.221  0  0.157  0.232  0  0.12  0  0.133  0  0  0  0.14  0  0  0  0  0  0  0  0  0.739  0  0  0  0.627  0  0  0.264  0  0  0  0  0.175  0  0  0  0.102  0  0  0.126  0.13  0  0  0.317  0.09  0  0  0  0  0.472  0  0.179  0.177  0  0  0  0  0  
 
 GATAD2B  57459  1  153777202  153895451  1q21.3  3.139  1.699  1.832  2.447  3.38  1.746  2.675  3.11  1.974  2.772  2.92  1.866  2.012  3.072  2.187  2.655  2.537  0.913  3.13  2.729  2.247  3.321  1.973  2.474  2.609  2.784  2.811  2.454  2.493  2.642  2.689  3.156  3.117  1.733  1.766  2.96  2.465  2.485  2.37  3.574  2.351  1.799  2.46  1.818  2.251  3.167  2.1  2.28  2.5  2.444  2.821  2.276  2.295  1.92  2.731  2.57  2.798  2.55  2.955  2.47  
 
 DENND4B  9909  1  153901976  153919154  1q21  3.099  0.513  1.552  2.066  2.524  1.419  2.281  2.74  1.9  2.702  2.86  1.636  2.817  2.338  3.1  2.436  2.219  0.192  3.76  1.837  3.018  2.947  1.718  2.082  2.138  2.763  2.491  3.222  2.352  3.076  3.153  3.082  3.552  1.409  1.818  3.483  3.607  2.626  1.746  3.489  2.705  2.47  2.715  1.898  2.17  3.064  1.783  2.227  2.033  2.799  2.639  2.8  2.584  1.483  2.797  2.464  2.595  2.17  3.01  2.081  
 
 CRTC2  200186  1  153920147  153931132  1q21.3  3.255  1.102  1.937  3.093  3.544  2.418  3.638  3.677  2.453  3.559  3.655  2.309  3.155  2.994  2.954  3.09  2.805  0.254  3.98  2.196  2.511  2.761  2.231  2.777  3.165  3.236  3.678  3.552  3.12  3.456  3.334  3.691  4.454  2.243  2.323  3.555  3.832  4.128  2.348  4.73  3.147  2.815  3.9  2.759  2.691  3.997  2.41  3.136  3.145  3.585  3.156  3.137  3.131  2.951  3.263  3.694  3.345  3.203  3.766  3.073  
 
 SLC39A1  27173  1  153931574  153940660  1q21  5.077  2.916  3.784  4.775  5.714  3.824  5.005  5.096  4.396  5.002  5.054  4.35  5.639  4.246  4.758  4.449  4.519  1.339  4.528  2.656  3.359  2.818  2.96  3.089  4.711  4.564  4.978  4.378  4.366  5.577  5.208  5.053  5.584  3.917  4.1  6.044  5.435  4.995  3.583  5.88  4.925  5.348  5.691  4.528  3.684  4.732  4.266  4.416  3.533  5.723  4.063  4.725  5.033  4.588  5.088  5.469  4.926  4.925  5.235  5.1  
 
 CREB3L4  148327  1  153940314  153946840  1q21.3  5.065  0.951  0.417  1.087  5.891  0.453  1.443  1.281  1.213  1.556  1.654  2.357  1.976  2.299  1.988  2.222  1.749  1.172  3.155  2.381  1.145  2.273  2.436  1.746  1.677  1.772  2.046  1.632  1.339  1.958  1.922  1.969  2.387  1.547  1.018  1.844  1.89  0.911  0.82  2.368  2.212  1.643  3.126  0.939  1.505  2.213  3.641  0.843  0.463  3.01  1.788  1.275  0.87  0.99  1.669  1.789  1  1.83  1.529  1.465  
 
 JTB  10899  1  153946744  153950451  1q21  5.802  5.684  4.147  5.599  6.005  4.179  5.752  5.307  5.47  5.106  5.328  5.063  4.659  5.497  3.992  4.897  5.229  4.305  6.739  6.797  4.849  5.575  6.116  5.633  4.018  4.254  3.816  3.356  3.754  4.273  4.626  4.86  4.033  3.606  4.381  5.77  4.615  4.752  5.884  4.742  5.044  5.872  5.105  4.218  3.752  5.793  5.021  3.679  3.626  5.924  4.428  5.253  4.418  4.327  4.983  5.421  4.046  5.003  4.665  4.397  
 
 RAB13  5872  1  153954092  153958853  1q21.2  5.121  4.03  4.001  3.98  5.823  3.727  5.66  5.374  3.553  5.623  5.023  3.924  3.487  4.698  4.067  3.526  4.407  3.225  1.639  0.097  2.897  0.696  0.362  0  5.124  5.436  4.233  4.394  4.443  3.846  5.405  5.89  4.966  4.136  4.149  5.283  5.113  5.211  4.129  5.072  1.956  4.399  5.284  3.682  3.648  5.823  4.934  3.427  2.931  5.738  4.497  4.158  4.703  3.559  4.978  4.892  5.116  5.03  4.93  5.177  
 
 RPS27  6232  1  153963238  153964631  1q21  9.403  11.9  6.548  5.944  10.033  9.597  9.621  8.837  6.39  8.848  9.045  9.461  7.998  10.15  7.359  8.903  9.894  12.008  11.402  11.8  9.726  9.97  7.281  9.846  9.745  8.641  7.699  7.297  7.614  8.357  9.257  9.179  8.444  5.416  9.397  9.687  8.226  8.76  10.33  9.009  9.183  8.444  9.451  9.692  8.842  9.752  9.464  7.576  8.415  11.116  8.017  8.405  8.213  8.389  9.608  9.481  7.537  9.84  9.512  8.759  
 
 NUP210L  91181  1  153965167  154127592  1q21.3  1.383  0.025  0.058  0  0.125  0.076  0.137  0.044  0.071  0.136  0.109  0.112  0.067  0.141  0.249  0.124  0.133  0.083  0.064  0.135  0.067  0.264  0.228  0.081  0.039  0.067  0.286  0.068  0.099  0.305  0.056  0.074  0.065  0.155  0.115  0.061  0.054  0.037  0.204  0.109  0.049  0.092  0.13  0.039  0.06  0.418  0.279  0.059  0.031  0.037  0.062  0.167  0.115  0.092  0.183  0.124  0.075  0.042  0.099  0.027  
 
 TPM3  7170  1  154127779  154164611  1q21.2  5.314  6.405  4.493  5.403  5.993  4.702  6.107  5.693  4.351  5.451  6.424  5.433  5.407  5.873  4.892  5.794  4.7  5.27  6.927  5.872  5.01  5.682  4.942  4.65  5.862  5.503  5.226  5.075  4.896  5.324  5.489  5.717  5.837  4.723  4.779  6.244  5.845  5.556  6.026  6.516  5.301  5.355  6.56  5.227  4.514  5.35  5.612  5.164  5.311  6.841  6.116  5.444  5.537  5.008  5.989  6.143  5.599  5.248  6.197  5.856  
 
 C1orf189  388701  1  154171561  154178841  1q21.3  0  0  0  0  0.407  0  0.132  0  0  0  0.218  0  0  0  0  0  0  0  0  0  0  0.135  0.365  0  0  0  0.15  0  0  0.057  0  0  0  0  0  0  0  0  0.179  0  0  0.12  0  0  0  0  0.09  0  0  0  0  0  0  0  0  0  0  0  0  0  
 
 C1orf43  25912  1  154179176  154193273  1q21.2  5.882  5.323  4.242  5.333  6.061  3.651  6.062  5.062  5.757  5.114  5.532  5.025  5.306  5.932  4.142  4.877  5.508  5.453  6.521  5.33  4.183  4.771  5.64  4.665  4.676  5.184  4.793  4.53  4.684  5.769  5.992  5.821  5.129  4.504  4.06  6.389  5.268  5.368  5.262  5.671  5.168  5.035  6.126  4.305  3.725  4.856  5.164  3.674  3.837  5.717  5.095  4.199  4.143  3.955  5.203  5.177  3.975  4.323  4.966  4.502  
 
 UBAP2L  9898  1  154192647  154243986  1q21.3  5.059  4.45  3.288  4.38  5.304  3.781  5.375  5.331  3.671  5.035  5.437  3.628  5.001  5.094  5.201  4.576  4.158  3.091  5.001  4.92  4.561  4.856  3.231  4.416  4.877  5.456  4.756  5.077  4.822  5.42  5.343  5.486  5.618  3.233  3.779  5.561  5.09  5.244  4.694  6.135  4.753  3.855  5.712  5.175  4.07  4.869  4.619  4.517  4.23  4.802  4.925  4.238  4.718  4.163  4.944  5.501  4.958  4.262  5.178  5.433  
 
 HAX1  10456  1  154245038  154248355  1q21.3  5.236  3.003  4.206  4.954  5.701  4.333  5.767  5.098  4.367  4.778  5.49  4.687  4.48  5.394  4.183  4.469  5.241  2.262  6.75  5.587  5.096  5.032  5.741  5.283  4.83  5.168  4.044  4.029  4.093  4.54  5.178  6.013  5.072  3.838  4.164  5.732  4.929  4.811  5.375  5.718  5.317  4.935  5.739  4.129  3.989  5.673  4.602  4.217  4.041  6.158  5.045  3.888  4.322  4.479  4.85  5.246  4.171  5.256  5.445  4.593  
 
 AQP10  89872  1  154293591  154297801  1q21.3  0  0  0.106  0  0  0  0.077  0  0.067  0.053  0.063  0  0  0.05  0  0  0.101  0  0  0.199  0.66  0.086  0.488  0  0  0  0.227  0  0.059  0.132  0  0  0  0.237  0  0  0  0  0.059  0  0  0.083  0.053  0  0  0  0.023  0  0  0  0  0.136  0.038  0.052  0.065  0.109  0  0  0  0  
 
 ATP8B2  57198  1  154298035  154323780  1q21.3  1.622  0.089  2  2.204  1.424  1.156  2.072  3.325  1.354  2.944  1.432  0.059  0  1.666  0.128  0.091  0.065  0  2.208  0.167  1.917  2.063  1.943  2.545  2.475  3.093  2.736  3.005  1.911  2.663  2.277  3.502  3.731  1.824  1.026  2.7  2.425  2.258  0.387  3.119  0.071  1.116  2.057  1.957  0.539  0.823  0.312  1.75  1.014  2.044  2.697  1.895  1.406  0.471  1.492  2.057  1.452  1.799  1.582  1.369  
 
 IL6R  3570  1  154377668  154441926  1q21  0.138  0.037  0.204  0.394  0.391  0.173  0.848  0.879  0.402  0.554  1.139  0.7  0.29  0.879  0.074  0.164  0.436  0.194  0.08  2.843  0.062  0.126  2.579  1.59  1.102  3.074  2.926  1.298  0.66  4.454  3.332  2.06  3.102  2.082  0.532  1.267  0.44  0.234  0.645  1.998  0.771  1.139  0.122  0.402  0.493  0.428  2.146  0.502  0.905  1.184  1.719  1.352  0.587  0.73  0.819  1.039  0.564  1.354  0.774  0.336  
 
 SHE  126669  1  154451953  154474526  1q21.3  0  0.024  0.139  0.043  0.02  0  0.027  0.051  0.142  0.035  0.04  0.024  0  0.021  0  0.024  0.025  0.049  0.016  0.069  0  0.072  0.36  0  0  0.018  0.061  0.056  0  0.058  0.017  0.037  0  0.24  0.039  0  0.1  0  0.057  0.329  0  0.073  0.038  0.022  0.123  0.061  0.037  0.114  0  0.048  0.047  0.329  0.022  0.05  0.057  0.026  0  0.056  0  0  
 
 TDRD10  126668  1  154474694  154520623  1q21.3  0  0.117  0.092  0  0.043  0.144  0.109  0.177  0.194  0.173  0.048  0.084  0.111  0.067  0  0.053  0  0.076  0.054  0.077  0  0.276  0.724  0.544  0  0.051  0.159  0.107  0.243  0.102  0  0  0.059  0.241  0.077  0  0.043  0  0.097  0.125  0  0.119  0.104  0  0.145  0.045  0.839  0  0  0.086  0.067  0.477  0.052  0.145  0.176  0.064  0.256  0  0  0  
 
 UBE2Q1  55585  1  154521050  154531120  1q21.3  3.668  3.842  1.918  2.628  3.628  2.234  3.932  3.397  3.458  3.201  3.903  2.755  3.346  3.388  2.8  3.097  3.002  4.272  4.365  3.114  2.273  3.103  3.039  2.792  2.997  3.442  2.888  3.167  2.548  3.757  3.492  3.395  3.588  2.268  2.299  4.054  3.598  3.203  3.567  4.122  3.317  2.647  4.394  2.737  2.073  3.222  3.316  2.564  1.991  4.307  3.424  2.414  2.157  2.397  3.239  3.371  2.663  2.75  3.085  2.916  
 
 UBE2Q1-AS1  100874097  1  154526084  154527504  -  2.04  0  1.232  1.713  0.404  0.62  0.479  1.058  2.732  0.882  1.132  1.129  1.068  1.321  1.203  0.793  0.798  0  0.595  0.812  0  0.695  1.387  0.901  0.523  0.963  1.44  1.304  1.002  2.363  0.79  1.003  1.221  1.445  0.383  0.951  1.147  0.96  0.723  0.98  0.854  0.824  1.805  0.67  0  0.622  0  0.892  0  0.891  1.142  0.482  0.857  0.809  1.186  1.098  0.735  1.257  0.718  0  
 
 CHRNB2  1141  1  154540256  154552353  1q21.3  0.266  0.049  0.161  0.123  0.253  0.095  0.207  0.152  0.29  0.26  0.294  0.093  0.179  0.272  0.29  0.227  0.125  0.11  0.099  0.194  0.136  0.408  0.635  0.171  0.061  0.133  1.036  0.198  0.187  0.308  0.275  0.244  0.138  0.356  0.221  0.237  0.237  0.169  0.327  0.417  0.322  0.306  0.874  0.117  0.116  0.139  0.14  0.13  0.271  0.036  0.152  0.63  0.152  0.364  0.266  0.127  0.267  0.029  0.274  0.068  
 
 ADAR  103  1  154554533  154600456  1q21.3  5.014  3.86  3.045  5.259  4.633  2.652  4.739  4.46  4.74  4.369  4.82  3.6  3.928  4.793  4.553  5.576  3.815  4.028  5.232  4.417  3.47  4.687  3.804  4.893  4.244  4.502  3.773  4.038  3.614  4.82  4.053  4.362  4.3  2.846  3.482  4.876  4.36  4.218  4.723  5.767  4.82  3.868  4.754  4.393  3.407  4.353  4.038  3.27  4.008  4.327  4.456  3.909  4.433  4.358  4.614  4.613  4.93  3.504  4.664  4.307  
 
 KCNN3  3782  1  154669941  154842754  1q21.3  0.029  0.044  0.139  0.047  0.059  0.028  0.699  0.083  0.238  0.124  0.118  0.088  0.053  0.176  0.036  0.049  0.062  0.107  0.038  0.068  0  0.113  1.176  1.15  0.357  0.059  0.369  0.171  0.04  0.133  0.051  0.19  0.262  0.61  0.058  0.022  0.125  0.045  0.125  0.188  0.038  0.45  0.126  0.018  0.058  0.149  0.232  0.034  0.016  0.044  0.118  0.43  0.026  0.134  0.066  0.067  0.048  0.03  0.021  0.054  
 
 PMVK  10654  1  154897207  154909484  1q22  3.864  0.457  2.551  4.243  5.416  3.021  4.439  3.67  4.171  4.173  3.684  4.43  3.584  3.115  2.226  3.388  3.701  0.145  5.072  3.682  1.869  3.989  4.583  3.93  3.007  3.112  3.789  2.657  2.673  3.408  3.594  3.29  3.55  2.613  3.394  4.087  3.464  3.912  2.072  4.246  4.088  4.527  4.967  3.522  2.559  4.018  5.041  2.339  2.335  4.27  3.488  2.64  2.89  3.293  3.734  3.635  2.65  3.537  3.343  3.066  
 
 PBXIP1  57326  1  154916558  154928567  1q21.3  1.66  0.45  1.828  2.628  2.813  0.78  1.694  2.863  2.882  2.587  1.807  1.532  1.437  1.527  1.485  1.52  1.047  0.159  3.09  0.718  0.36  1.456  2.378  2.858  1.462  2.455  1.952  1.977  2.087  1.654  1.728  1.495  1.395  0.891  1.111  1.67  1.62  2.265  1.588  2.459  1.403  1.549  1.164  1.91  1.661  1.877  0.951  0.995  0.624  2.504  1.911  1.235  1.011  1.693  2.009  2.355  1.288  2.198  1.578  2.325  
 
 PYGO2  90780  1  154929501  154934258  1q21.3  2.967  0.904  1.861  2.931  3.482  2.09  2.835  2.623  3.67  3.051  3.303  2.907  2.844  2.688  3.171  3.043  2.688  0.115  3.8  1.1  2.773  2.264  2.903  2.749  2.26  2.662  2.323  2.606  2.038  2.253  2.69  2.318  3.3  2.244  2.051  3.31  2.776  3.073  2.001  3.968  3.035  3.165  3.439  2.292  2.798  2.463  0.63  2.225  2.501  3.511  2.809  2.436  2.089  1.913  2.503  2.594  1.934  2.488  2.139  2.367  
 
 SHC1  6464  1  154934773  154946959  1q21  3.28  3.637  4.254  6.404  4.754  3.788  5.463  5.366  4.626  5.696  6.061  4.379  5.102  4.725  4.019  4.579  3.698  1.397  4.412  3.182  3.245  2.993  3.375  3.456  5.281  5.311  4.438  3.879  4.165  3.723  4.538  5.736  5.17  3.835  4.139  6.208  5.335  5.105  6.041  6.497  4.116  5.985  6.464  4.134  2.975  4.43  5.031  4.353  3.664  5.955  4.683  5.237  4.006  4.89  5.059  6.227  4.913  6.489  4.509  4.342  
 
 CKS1B  1163  1  154947117  154951725  1q21.2  5.96  9.048  3.964  4.841  5.992  4.844  6.109  4.97  3.322  5.176  6.212  5.31  4.721  6.559  4.591  5.522  6.146  8.576  7.233  6.055  5.345  5.345  5.212  4.871  5.38  4.381  4.547  4.019  3.94  4.446  5.063  4.795  4.853  3.603  5.486  5.534  5.813  5.46  5.81  6.455  5.757  5.094  6.222  5.433  4.76  5.419  6.235  4.397  4.956  6.91  5.527  4.831  5.197  4.617  6.335  5.776  5.168  6.502  5.996  5.105  
 
 FLAD1  80308  1  154955769  154965587  1q21.3  3.726  1.382  2.348  4.003  3.971  2.556  4.757  3.454  3.803  3.021  4.151  4.177  4.462  3.961  3.667  3.778  3.183  1.1  4.415  4.104  3.714  3.317  3.56  4.419  3.456  3.336  3.778  3.001  3.085  4.142  3.71  4.052  3.636  2.306  3.165  3.853  4.06  3.514  3.113  4.507  3.713  3.883  4.971  3.325  3.463  4.025  4.792  2.752  2.574  4.299  3.386  2.927  3.115  3.326  3.668  3.661  2.972  3.282  4.079  3.157  
 
 LENEP  55891  1  154966061  154966791  1q22  0.596  0  0  0  0.26  0  0.252  0.183  0.513  0.274  0.269  0  0  0.104  0.29  0  0  0  0  0  0  0.128  0  0.227  0  0  0.382  0  0  0.348  0.202  0  0.217  0.293  0  0  0  0  0.156  0  0.24  0  0.334  0  0  0.164  0  0  0  0  0.168  0.288  0.159  0.198  0  0  0.17  0  0  0  
 
 ZBTB7B  51043  1  154975105  154991001  1q21.3  2.853  0.224  1.601  3.229  3.23  0.662  2.406  3.124  3.029  2.754  3.585  2.7  3.487  1.893  3.095  2.568  2.035  0.26  0.328  1.864  2.462  0.197  2.446  3.043  1.367  1.85  1.457  2.245  1.714  2.413  1.795  2.033  2.525  1.474  1.8  2.874  2.679  2.46  1.059  3.652  2.945  3.347  1.682  1.611  2.479  2.942  0.843  1.194  1.943  1.732  2.431  2.465  2.213  2.88  2.238  2.689  2.468  1.946  2.366  1.884  
 
 DCST2  127579  1  154991002  155006257  1q22  0.477  0  0.067  0.431  0.757  0.078  0.087  0.102  0.316  0.371  0.178  0.084  0.166  0.378  0.248  0.229  0.084  0  0.062  0.074  0  0.114  0.562  0.362  0  0.058  0.219  0.088  0  0.129  0.063  0  0.245  0.271  0.071  0.143  0.076  0.073  0.119  0.195  0.231  0.573  0.22  0  0.077  0.222  0.31  0.105  0  0.274  0.191  0.531  0.072  0.286  0.26  0  0.116  0  0.092  0.09  
 
 DCST1  149095  1  155006281  155023406  1q22  0.165  0  0  0  0.167  0  0  0.05  0.062  0.096  0.055  0  0  0.065  0.088  0.089  0  0.11  0  0.051  0  0.11  0.282  0  0  0.076  0.095  0.032  0  0.187  0  0  0.128  0.153  0  0  0  0  0.048  0  0.072  0.183  0.335  0.068  0  0.067  0.023  0  0  0  0.087  0.4  0.033  0.172  0.069  0  0  0  0  0  
 
 LOC100505666  100505666  1  155017667  155036467  -  1.922  0.898  0.339  1.677  2.503  0.589  1.193  0.29  1.146  1.092  1.082  0.652  1.248  1.426  1.149  1.236  0.456  0.667  0.294  1.013  0.736  0.449  2.39  2.124  0.514  0.748  1.81  0.7  0.634  1.551  0.886  0.728  2.166  1.637  1.187  1.654  0.891  0.657  0.923  1.424  0.927  1.61  2.779  0.407  0.402  0.616  3.684  0.875  1.068  2.025  1.37  1.973  1.213  1.297  1.642  0.835  0.438  0.199  0.824  0.478  
 
 ADAM15  8751  1  155023747  155035252  1q21.3  3.899  0.187  2.497  4.36  5.111  1.999  4.136  3.106  4.023  4.919  4.098  3.681  5.281  3.204  4.351  3.837  3.326  0  2.711  2.166  2.623  0.347  3.075  5.487  2.498  3.62  3.404  4.129  3.961  3.529  4.275  5.096  4.564  2.401  2.534  4.414  4.951  4.689  2.364  5.041  4.41  5.428  2.581  3.272  3.922  4.089  1.64  3.713  2.627  4.386  5.747  3.85  3.984  3.077  3.477  3.664  4.044  3.641  3.91  4.773  
 
 EFNA4  1945  1  155036212  155042029  1q21-q22  2.667  0.092  1.457  2.756  3.508  1.571  2.268  1.821  3.04  2.53  2.739  2.321  2.48  1.983  2.07  2.464  2.808  0  3.231  0.293  1  1.517  2.277  1.956  1.555  0.984  1.571  0.878  0.908  1.776  1.97  1.552  2.133  1.734  1.309  2.673  1.492  1.76  1.58  2.147  1.778  2.568  3.681  2.174  2.591  1.958  1.374  1.481  1.724  3.316  2.16  1.86  2.136  1.816  2.461  2.533  1.508  1.806  2.586  2.161  
 
 EFNA3  1944  1  155051347  155060014  1q21-q22  2.902  0.159  1.081  1.75  4.297  0.792  1.224  1.35  2.485  1.128  1.596  1.893  3.024  2.894  1.812  0.819  2.375  0.057  1.633  0.705  1.39  0.68  2.36  2.843  0.9  0.609  0.75  1.04  0.947  2.553  0.716  1.749  2.56  1.53  0.743  2.468  0.54  0.514  0.558  2.322  2.071  2.253  2.22  0.889  1.075  1.022  1.629  0.947  1.058  2.483  1.581  2.182  0.794  1.097  1.175  1.627  1.003  1.421  1.594  0.672  
 
 EFNA1  1942  1  155100348  155107386  1q21-q22  5.047  0.491  0.492  2.8  4.707  0.493  3.045  2.844  3.547  2.478  2.963  2.524  3.295  3.416  3.881  2.483  4.091  2.358  1.118  0.19  0.353  0.482  2.328  1.612  0.218  1.847  2.607  1.22  0.796  4.602  0.915  2.564  3.387  2.84  1.484  5.25  2.068  1.973  3.483  4.949  3.251  3.258  3.487  1.146  2.014  2.808  3.217  1.297  2.548  2.93  2.098  2.091  2.023  1.081  2.432  2.971  0.841  1.647  2.999  2.618  
 
 SLC50A1  55974  1  155107819  155111334  1q22  3.394  1.584  2.432  4.084  4.42  2.105  2.326  3.356  2.82  2.909  3.065  3.714  3.534  2.488  3.332  3.014  3.716  0.794  3.131  1.2  3.259  1.165  4.416  2.709  2.609  2.817  2.216  2.803  2.231  3.483  2.747  3.474  3.121  1.919  2.01  3.758  2.928  3.079  1.703  3.985  3.741  3.22  4.079  2.61  2.593  4.037  1.663  1.894  2.377  4.219  3.741  3.34  2.094  2.656  2.444  3.312  2.423  2.878  3.021  2.41  
 
 DPM3  54344  1  155112366  155112996  1q22  5.719  4.332  4.733  6.548  6.042  5.514  6.221  4.923  6.732  5.593  4.979  4.447  3.918  6.311  4.03  5.322  6.024  1.894  7.037  6.42  4.267  5.995  6.533  6.321  3.526  4.399  5.119  3.606  4.541  4.431  6.16  5.369  5.276  5.604  5.117  5.806  3.367  4.431  5.322  4.748  4.803  6.706  5.739  5.131  5.348  5.311  5.098  3.873  3.759  5.81  3.27  5.004  3.549  5.069  5.522  5.571  3.504  4.173  3.958  3.592  
 
 KRTCAP2  200185  1  155141883  155145804  1q22  6.021  7.379  4.695  6.015  6.836  5.278  6.9  5.807  5.75  6.136  5.242  6.11  5.183  5.872  4.413  5.819  6.022  6.282  7.033  7.516  6.421  6.322  6.54  7.08  5.689  5.145  5.1  3.886  4.558  6.419  6.227  5.77  4.612  3.911  5.764  6.488  5.166  5.479  6.611  5.978  6.245  6.029  6.388  5.551  5.782  6.603  7.238  4.645  5.725  6.661  5.446  5.252  5.566  5.394  6.366  6.371  4.846  5.567  5.882  4.876  
 
 TRIM46  80128  1  155146262  155157447  1q22  1.463  0.3  1.088  2.134  1.879  0.881  1.005  0.894  0.707  1.7  1.233  0.491  0.568  0.92  0.641  0.786  0.656  0.239  2.396  0.912  0.302  1.768  1.065  0.539  0.935  0.893  1.345  0.634  1.12  1.508  1.043  0.899  1.593  0.601  0.797  0.881  1.149  1.278  1.06  2.054  0.761  0.948  1.06  0.277  1.298  0.703  1.329  1.862  0.334  2.256  0.934  1.485  0.687  1.273  1.939  0.8  1.501  0.977  0.652  0.849  
 
 MUC1  4582  1  155158299  155162706  1q21  5.611  0  2.493  4.784  8.554  0.908  0.89  2.355  1.43  3.731  1.579  4.736  6.335  0.967  1.105  1.813  0.846  0  1.483  0  2.96  0.577  4.092  3.823  0.782  0.605  0.923  0.483  0  2.047  0.493  0.988  1.175  1.265  0.486  5.125  0.299  0.855  2.482  2.961  4.264  2.467  3.591  1.218  0.906  3.008  0.348  2.696  2.972  4.665  1.252  4.168  1.488  2.919  2.865  1.872  3.804  0.985  3.046  0.924  
 
 THBS3  7059  1  155165378  155177772  1q21  1.587  0.163  2.024  3.086  2.293  0.393  1.05  1.226  1.822  2.417  1.204  0.562  0.845  0.715  0.999  0.634  0.521  0.156  1.459  0.146  0.252  0.79  0.991  0.643  0.719  2.24  1.334  1.338  0.721  1.548  0.494  1.457  1.277  0.66  0.9  1.723  0.798  1.762  1.277  2.389  1.353  1.412  3.457  0.533  1.061  0.931  0.368  1.896  0.862  3.951  1.376  1.096  0.712  0.953  3.358  1.649  3.025  0.942  2.721  1.254  
 
 MTX1  4580  1  155178489  155183630  1q21  3.853  1.882  2.108  3.013  4.379  2.442  4.301  3.617  2.768  3.322  3.562  3.747  3.091  4.304  3.183  3.112  3.641  1.595  5.383  3.453  3.579  3.57  2.957  3.131  3.168  2.972  3.307  3.129  2.973  4.076  3.804  4.138  3.639  1.94  2.935  4.249  3.641  3.437  3.15  4.647  4.141  4.158  4.797  3.723  3.575  3.7  3.581  3.23  2.707  5.54  3.179  3.748  3.269  2.771  4.39  3.993  2.668  4.086  4.099  3.481  
 
 GBAP1  2630  1  155183615  155197325  1q21  2.202  0.869  1.131  2.729  2.854  1.225  2.497  1.939  1.365  2.479  1.53  1.216  1.9  1.081  1.57  1.169  1.074  0.203  2.402  0.646  0.672  1.409  1.853  1.588  1.466  2.589  2.268  2.177  2.575  4.511  3.522  2.505  2.57  1.286  0.903  2.23  1.903  1.859  1.247  2.672  1.734  1.779  2.387  0.926  1.333  2.082  1.76  0.805  1.056  1.879  1.345  1.3  1.266  1.75  2.179  1.668  1.522  1.334  1.333  1.592  
 
 GBA  2629  1  155204238  155214653  1q21  3.424  2.162  2.133  4.174  4.327  2.583  3.998  4.265  2.494  4.031  3.47  2.817  3.514  2.224  3.104  2.44  2.157  0.313  3.174  1.599  2.01  1.938  2.381  3.487  3.394  4.774  3.83  4.446  4.717  6.435  5.746  4.695  4.695  2.229  2.063  4.379  3.465  4.119  2.401  4.975  3.767  2.787  3.694  2.748  2.865  3.917  2.702  2.005  2.877  3.57  3.161  3.088  2.663  3.809  3.541  3.557  3.428  3.594  3.139  3.628  
 
 FAM189B  10712  1  155216995  155225274  1q21  4.706  1.997  2.637  4.205  4.252  2.221  3.599  3.202  2.76  3.391  3.482  2.57  4.047  4.237  3.825  2.929  3.209  1.605  4.084  2.352  2.479  2.999  2.084  2.48  3.605  3.347  3.433  3.086  2.87  4.036  3.836  4.491  4.124  3.098  2.515  4.185  3.984  3.945  3.759  4.767  2.905  4.369  5.231  2.591  3.21  2.815  3.403  3.483  2.04  4.555  3.972  4.074  2.928  2.492  3.734  3.899  3.213  4.022  3.761  2.933  
 
 SCAMP3  10067  1  155225769  155232176  1q21  5.232  3.501  4.263  5.286  5.839  3.957  6.294  5.355  5.219  5.097  5.331  4.253  4.313  4.557  3.972  4.483  3.827  0.623  6.09  5.003  4.511  4.916  4.961  4.843  4.399  5.018  4.628  4.815  4.427  5.908  5.057  5.523  5.687  4.675  4.156  5.467  4.79  4.522  5.263  5.684  4.816  5.417  5.297  4.061  4.522  4.662  5.559  3.938  3.324  5.625  4.975  4.319  4.283  4.261  4.621  4.985  3.904  4.619  4.675  4.17  
 
 CLK2  1196  1  155232658  155243320  1q21  3.859  2.132  1.733  2.279  3.784  1.731  2.928  2.29  2.982  3.39  3.082  2.296  2.813  3.908  3.104  2.84  2.85  1.1  5.41  4.265  2.872  3.834  2.721  3.061  2.275  3.032  2.945  2.455  2.354  2.548  2.912  3.297  2.942  1.334  2.363  3.299  3.146  2.556  3.384  3.536  3.149  3.185  4.222  3.192  2.448  2.764  4.527  2.353  2.461  3.944  2.828  2.812  2.637  1.877  3.796  3.166  2.868  2.296  2.857  2.401  
 
 HCN3  57657  1  155247217  155259639  1q22  2.586  0.193  0.528  1.408  1.741  0.312  1.109  0.761  1.035  1.25  1.038  0.704  1.305  1.779  2.222  1.335  1.284  0.175  2.045  0.526  0.94  1.011  0.996  0.668  0.581  0.602  1.039  1.047  0.637  0.994  1.36  0.615  1.242  0.38  0.89  2.412  1.375  0.669  0.487  2.136  0.965  1.523  2.735  0.37  0.529  0.545  1.702  1.152  0.992  1.429  0.826  1.274  1.362  0.717  1.816  0.951  1.254  1.068  1.649  0.54  
 
 PKLR  5313  1  155259083  155271225  1q21  0.201  0.16  0.153  0.215  0.335  0  0.14  0.115  0.306  0.118  0.155  0.521  0.062  0.146  0.134  0.153  0.242  0.048  0.196  0.175  3.377  0.342  0.59  0.136  0  0.294  0.236  0.067  0.086  0.215  2.08  0.336  0.132  0.285  0.154  0.149  0.045  0.121  0.267  0.112  0.089  0.615  0.329  0.047  0  0.114  1.161  0.067  0  0.173  0.06  0.466  0.032  0.14  0.293  0.142  0.362  0.071  0.124  0.058  
 
 FDPS  2224  1  155278538  155290457  1q22  4.747  6.331  4.252  6.071  5.331  4.579  5.79  6.785  4.091  5.372  5.445  5.232  5.241  5.534  4.848  4.857  5.645  4.093  7.163  5.947  5.093  5.403  5.15  5.883  5.276  4.792  4.314  4.017  4.132  4.55  5.674  5.283  4.947  3.54  4.246  6.384  5.901  4.692  5.25  5.398  5.686  4.952  5.162  4.771  4.647  5.96  4.771  4.484  4.675  6.798  4.921  5.117  4.459  4.106  5.784  5.107  4.611  5.603  5.784  5.985  
 
 RUSC1-AS1  284618  1  155290250  155293938  1q22  1.61  2.451  2.394  4.164  2.131  1.324  1.215  1.422  2.099  1.559  1.133  1.831  1.126  1.266  1.147  2.173  2.45  2.737  1.958  3.897  1.137  3.522  3.196  1.932  1.286  0.835  1.581  0.757  0.965  0.818  1.565  1.041  1.279  1.992  1.464  1.368  1.11  1.156  4.056  1.081  1.135  3.211  0.81  1.333  0.727  1.554  2.199  0.765  1.038  1.596  0.65  1.54  1.271  1.705  3.295  1.795  1.203  1.169  1.334  0.578  
 
 RUSC1  23623  1  155290639  155300909  1q21-q22  5.69  4.059  2.505  3.548  5.377  2.377  4.246  3.735  2.722  3.459  4.208  3.209  4.028  4.524  3.259  4.033  3.57  3.42  3.972  3.034  2.941  2.933  2.042  2.115  2.393  2.747  2.923  2.55  2.392  2.983  3.297  3.102  3.383  1.752  2.927  4.186  3.86  3.58  3.54  4.333  3.088  4.115  5.334  2.522  2.955  3.488  4.275  3.161  2.35  4.88  3.712  3.15  3.873  2.942  3.933  3.575  3.727  3.187  4.603  2.631  
 
 ASH1L  55870  1  155305051  155532324  1q22  3.141  2.722  1.312  1.659  2.849  1.473  2.119  2.617  2.213  2.687  2.628  1.704  2.247  2.659  2.41  2.054  1.649  1.752  1.595  1.726  1.623  2.067  1.147  1.429  1.788  2.542  1.517  1.982  1.691  1.507  2.001  2.108  2.029  1.097  1.626  3.124  2.211  2.657  2.278  3.213  2.644  1.903  2.2  2.337  1.556  2.452  1.673  1.609  2.384  1.449  2.594  1.884  2.094  1.609  2.332  2.313  2.367  1.602  2.512  2.027  
 
 POU5F1P4  645682  1  155402970  155404053  1q22  0.313  0  0.233  0  0.328  0  0  0.449  0.256  0.415  0.293  0.133  0.358  0.131  0.307  0.322  0.127  0  0  0.144  0.428  0.281  0.813  0.282  0  0.279  0.271  0.111  0  0.225  0.211  0  0.335  0  0.168  0.198  0.255  0.376  0.121  0  0.39  0.643  0.052  0.447  0.185  0.629  0.171  0.322  0.578  0  0.324  0.642  0.429  0.124  0.271  0.187  0.519  0  0.648  0  
 
 ASH1L-AS1  645676  1  155531771  155533735  1q22  1.496  0.856  0.366  0.632  1.332  1.111  0.79  0.504  1.028  0.752  0.625  0.461  0.801  0.714  0.527  1.147  0.638  2.037  1.731  0.907  1.083  1.092  1.03  0.728  0.495  0.416  0.592  0.671  0.463  0.366  0.628  0.725  0.674  0.61  0.61  0.78  0.503  0.709  0.692  0.894  0.888  1.745  0.809  0.694  0.477  0.585  0.419  0.487  0.243  1.307  0.404  1.178  0.672  0.788  1.351  0.926  0.491  0.804  1.003  0.226  
 
 MSTO1  55154  1  155579960  155584758  1q22  2.953  2.046  1.971  2.492  3.15  2.534  3.398  2.581  2.818  2.744  3.082  2.075  2.577  2.796  2.616  2.129  1.869  0.872  3.87  2.689  2.447  3.383  2.334  2.318  2.239  2.842  2.363  2.253  2.772  2.063  2.983  3.196  2.678  1.463  2.412  2.972  3.301  2.848  2.406  3.796  2.781  2.909  3.662  2.811  1.803  2.849  3.162  1.958  2.007  3.407  2.521  2.052  2.789  2.215  2.951  2.691  2.423  2.39  2.79  1.903  
 
 YY1AP1  55249  1  155629232  155658823  1q22  4.768  3.008  2.875  4.079  4.826  2.971  3.965  3.966  4.029  4.2  4.354  3.757  3.716  4.068  3.657  3.871  3.846  1.5  5.129  4.274  3.576  4.371  3.536  3.547  3.383  3.643  3.184  3.264  3.191  2.999  3.511  3.755  3.665  2.539  2.987  4.375  4.068  3.99  4.212  4.92  3.978  3.351  4.444  3.849  2.789  3.885  3.602  3.146  3.511  4.515  4.065  3.399  3.533  3.103  3.972  3.806  3.706  3.705  4.529  3.465  
 
 DAP3  7818  1  155658881  155708800  1q22  5.397  5.636  3.082  4.163  4.866  3.293  5.022  3.769  3.998  3.722  4.826  4.18  4.231  5.183  3.256  4.145  4.264  4.976  5.696  5.841  4.34  4.945  4.089  3.504  3.613  3.876  4.644  3.502  3.665  4.192  4.353  4.566  4.356  3.014  3.195  4.25  4.173  4.586  5.066  5.345  3.861  3.919  5.341  3.636  3.709  4.154  6.198  3.281  2.961  5.519  4.176  3.226  3.709  2.819  4.708  4.283  3.422  3.829  4.104  3.827  
 
 MSTO2P  100129405  1  155715558  155720479  1q22  2.036  0.176  1.258  1.25  2.136  1.667  2.412  1.894  1.692  1.767  2.042  1.329  1.716  1.358  1.68  1.358  0.988  0.102  2.982  1.154  1.525  2.284  1.618  1.479  1.425  1.829  2.212  1.304  1.924  1.632  2.05  2.338  1.715  0.912  1.646  1.896  2.409  2.103  0.848  2.535  1.778  2.263  2.462  1.734  1.107  1.948  2.573  1.267  1.041  2.391  1.482  1.275  1.956  1.418  1.836  1.638  1.531  1.659  1.845  0.846  
 
 GON4L  54856  1  155719448  155829191  1q22  3.235  2.26  1.55  2.307  2.909  1.431  2.281  2.356  2.397  2.534  2.849  2.126  2.075  2.929  2.135  2.131  1.901  1.939  2.62  2.329  2.197  2.728  1.938  1.964  1.72  2.438  1.797  2.235  2.008  1.428  2.187  2.167  2.412  1.403  1.68  2.616  2.387  2.542  2.347  3.382  2.349  1.898  2.982  2.209  2.419  2.503  2.263  1.713  1.755  2.121  2.567  1.821  2.122  1.683  2.737  2.527  2.497  1.872  2.586  2.148  
 
 SYT11  23208  1  155829259  155854990  1q21.2  0.071  0.051  0.55  2.021  0.064  1.141  2.432  1.07  4.658  2.391  3.31  0.196  0.059  0.098  0.072  0.044  0.036  0.781  1.006  0.139  0.055  0.58  0.559  0.151  0.297  1.656  1.083  1.325  1.057  0.254  1.145  0.789  0.305  0.33  0.187  0.257  0.343  1.848  0.098  0.85  0.178  1.398  3.413  0.029  1.478  0.464  0.435  0.071  0.113  0.104  0.127  0.349  0.095  0.124  1.708  0.117  2.095  2.206  0.587  2.188  
 
 RIT1  6016  1  155867598  155881193  1q22  1.629  2.304  1.145  1.485  1.831  1.152  2.042  1.727  2.429  1.995  2.394  0.962  1.73  1.153  1.013  1.181  0.576  1.978  2.324  0.738  1.073  1.471  1.156  0.657  1.139  1.357  0.793  0.833  0.958  0.511  1.693  1.406  0.712  0.684  0.928  1.65  1.448  1.286  1.891  1.564  2.204  1.578  2.582  1.526  1.305  1.486  1.159  0.73  0.974  1.829  1.645  1.042  1.309  2.014  1.338  1.905  2.669  1.566  1.177  1.361  
 
 KIAA0907  22889  1  155882833  155904233  1q22  2.74  3.641  1.478  2.163  3.19  1.781  2.485  2.579  2.441  2.767  3.101  1.769  2.51  3.098  2.406  2.674  2.478  3.864  4.411  2.56  2.882  2.776  2.333  1.713  1.99  2.216  2.003  2.175  2.108  1.307  2.775  2.472  2.828  1.536  1.885  2.912  2.81  2.574  2.906  2.57  2.713  2.655  3.153  2.242  2.501  2.765  2.421  2.218  2.394  3.481  2.603  2.061  2.068  2.164  2.807  2.193  2.639  2.227  2.783  1.913  
 
 SNORA80E  677823  1  155889699  155889833  1q22  0  0  0  0  0  4.898  0  2.8  0  0  2.269  0  0  0  0  1.817  0  0  0  0  0  1.56  0  0  0  0  1.812  0  0  0  0  0  2.924  0  0  0  0  0  0  0  0  0  0  0  0  0  0  0  0  0  2.271  0  0  1.813  0  0  0  0  0  0  
 
 SCARNA4  677771  1  155895748  155895877  1q22  0  0  0  0  0  0  0  0  0  0  0  0  0  0  0  0  0  0  0  0  0  0  0  0  0  0  0  1.994  0  0  0  0  0  0  0  0  0  0  0  0  0  0  0  0  0  0  0  0  0  0  0  0  0  0  2.288  0  0  0  0  0  
 
 RXFP4  339403  1  155911479  155912625  1q22  0  0.094  0.282  0  0.194  0  0.141  0.103  0.566  0.21  0.203  0.113  0.491  0.113  0.168  0.174  0.165  0  0.207  0.179  0  0.418  0.853  0.441  0  0  0.654  0.086  0.114  0.109  0  0  0.115  0.451  0  0  0.096  0.212  0.214  0  0.183  0.473  0.077  0  0  0.136  0.375  0  0  0.142  0.119  0.869  0.072  0.146  0.117  0  0  0  0  0  
 
 ARHGEF2  9181  1  155916629  155948336  1q21-q22  1.521  1.869  3.217  4.713  2.305  2.439  2.609  3.125  4.746  3.594  3.298  2.52  3.209  1.932  1.822  2.378  2.298  1.099  4.057  2.83  3.245  2.727  2.531  3.13  3.628  3.028  2.629  3.369  3.506  1.858  3.464  3.75  3.934  2.413  1.457  2.084  3.725  4.035  3.649  2.361  2.534  3.101  2.236  1.812  1.869  2.305  2.05  2.02  1.856  2.535  1.871  2.606  1.419  2.846  2.43  3.129  2.82  2.451  2.37  1.991  
 
 SSR2  6746  1  155978838  155990758  1q21-q23  6.113  4.411  5.571  7.094  6.483  5.733  7.454  6.974  7.122  6.873  7.126  6.438  5.493  6.406  4.605  5.744  6.153  2.716  7.18  6.057  5.132  4.773  7.026  6.532  5.954  5.621  5.603  5.514  5.225  4.942  5.979  6.556  6.721  5.738  5.156  6.661  5.994  6.341  5.86  6.465  5.271  6.699  7.175  6.456  6.457  5.833  5.469  4.743  4.656  6.784  6.027  4.402  5.694  5.491  6.072  6.625  5.418  6.399  6.18  5.919  
 
 UBQLN4  56893  1  156005084  156023616  1q21  4.344  2.128  2.854  3.369  4.282  2.293  4.078  3.305  3.821  3.416  3.725  3.18  4.046  3.909  3.627  3.392  3.293  1.495  4.265  3.162  3.57  3.538  2.84  3.735  3.293  3.792  3.446  4.034  3.047  3.153  3.265  3.896  4.251  2.467  2.977  4.285  3.971  4.385  2.965  5.425  3.731  3.255  4.586  3.669  4.053  3.738  3.015  3.08  2.868  3.73  3.635  3.716  3.378  2.943  3.955  4.224  3.399  3.705  4.165  3.76  
 
 LAMTOR2  28956  1  156024516  156028301  1q22  5.503  4.566  3.184  5.084  6.046  3.869  5.62  4.702  5.195  5.341  4.681  4.822  4.791  5.377  3.317  4.933  5.047  3.163  6.637  5.961  3.981  4.827  4.904  4.594  4.613  4.343  4.414  3.696  3.818  4.135  4.93  5.095  4.134  3.32  4.642  5.457  3.18  4.222  5.173  5.355  4.295  4.865  5.833  4.24  4.392  4.924  5.239  3.705  3.714  6.22  4.754  4.259  4.116  4.38  5.587  5.006  3.393  4.992  4.758  4.057  
 
 RAB25  57111  1  156030965  156040295  1q22  6.095  0  0  0  6.449  0  0.099  0  0.179  0.103  0.076  5.495  5.952  5.887  5.043  5.966  6.248  0.125  0.152  0.107  0  0.227  0.474  0  0  0  0.126  0  0  0.063  0  0  0  0  0  0  0  0  0.089  0.242  6.046  0.147  0.476  0  4.433  3.842  0  0  0  0  0  0.955  0  0.103  0.107  0.127  0  0  0  0  
 
 MEX3A  92312  1  156041803  156051789  1q22  2.614  0.102  1.129  2.649  1.612  1.271  1.163  1.442  2.414  1.251  1.686  0.34  1.462  1.086  1.219  1.941  0.627  0.99  1.774  0.67  1.601  1.625  0.582  0.213  0.97  0.923  1.349  1.09  0.46  0.717  1.327  1.227  1.485  1.122  0.578  2.089  1.502  1.108  0.515  1.929  0.569  1.377  3.493  2.122  3.394  2.213  0.798  1.267  0.313  1.748  1.932  1.122  0.623  0.544  1.055  1.599  0.887  1.181  0.777  1.836  
 
 LMNA  4000  1  156052336  156109880  1q22  5.777  3.351  5.849  7.127  6.471  5.245  7.673  6.747  5.312  7.754  6.892  6.586  6.346  6.21  5.383  7.112  6.899  1.28  0.869  4.156  4.387  1.674  4.138  4.114  5.478  6.904  8.157  6.786  6.65  6.72  6.393  7.496  6.862  5.41  5.328  6.673  6.919  7.811  6.253  6.159  6.979  7.326  5.39  6.118  6.383  5.739  6.606  6.02  5.099  7.349  6.654  6.288  6.069  5.243  6.476  6.29  5.939  6.36  6.687  6.587  
 
 SEMA4A  64218  1  156119734  156147542  1q22  3.174  0.066  0.143  0.226  3.375  0.049  0.279  0.374  0.151  0.263  0.166  1.344  0.098  0.398  0.342  1.404  0.38  0.044  4.047  2.57  0.24  0.302  1.733  0.393  0.122  0.288  0.154  0.617  0  0.179  0.444  0.16  0  0.275  0  0.22  0.131  0.545  0.5  0  1.992  0.221  0.357  2.499  1.554  1.658  0.235  0.245  0.087  1.068  0.214  0.381  0.148  0.315  0.09  0.077  0.366  0.496  0.128  0.145  
 
 SLC25A44  9673  1  156163722  156182587  1q22  3.388  2.225  1.969  2.851  3.691  1.757  3.984  3.435  2.814  3.144  3.496  2.44  2.707  2.259  2.634  2.334  2.367  1.072  4.038  2.463  2.122  2.542  2.262  2.224  2.574  3.379  2.755  3.566  3.204  3.24  3.143  3.573  4.123  2.544  2.49  3.706  2.944  3.479  3.112  4.076  2.802  2.429  3.513  2.647  2.642  3.037  1.828  2.211  2.052  3.062  3.219  2.321  2.821  2.385  2.614  2.965  2.537  2.57  2.926  2.643  
 
 PMF1  11243  1  156182778  156209868  1q12  5.067  1.898  3.13  4.088  5.408  3.701  4.711  4.71  3.946  4.507  4.585  4.453  3.368  4.405  3.173  4.408  3.997  1.393  6.402  4.329  3.697  4.34  4.108  3.693  3.622  3.791  4.441  4.056  3.872  3.802  5  4.158  4.509  2.918  3.515  3.977  4.137  4.282  4.155  4.733  4.369  4.764  5.03  4.178  3.397  3.819  3.99  3.802  3.178  5.341  3.619  3.925  4.037  3.198  4.783  4.236  3.544  5.107  4.411  3.733  
 
 PMF1-BGLAP  100527963  1  156182778  156213123  1q  0.569  0.996  0.837  0.939  1.945  1.199  2.249  1.264  2.152  1.769  1.358  0.889  0.708  0.965  0.872  0.416  1.061  0.256  2.199  0  0  0.615  0.656  1.142  0.459  0  0.724  0.301  0.735  0.374  2.471  1.506  1.315  0.91  0.894  0.972  0.96  0.932  1.915  1.22  2.023  2.098  0.844  0.466  1.272  1.509  0.898  1.081  0.832  1.867  0.855  0.815  0.989  0.58  1.207  1.337  0.437  1.979  0.788  0.701  
 
 BGLAP  632  1  156211752  156213123  1q22  0.438  0  0.35  0  0.797  0.482  0.397  0.945  0  1.346  1.065  0  0  0.548  0.389  0.317  0  0.374  1.452  0.955  0.734  1.167  1.272  0.26  0  0.458  0.448  0.36  0  0.166  0.296  0  0.515  0  0  0  0.593  1.908  1.11  0.796  0.344  0.591  1.815  0.608  0  0  1.274  0  0  0.578  0.506  0.77  0.362  0.77  0.284  0  0.202  1.119  0  0  
 
 PAQR6  79957  1  156213111  156217908  1q22  1.671  0.113  0.423  0.327  3.045  0.23  1.306  0.964  1.165  2.778  2.052  0.89  0.369  1.905  2.009  0.847  0.816  0.167  2.337  0.302  0.437  1.106  0.96  0.777  0.23  0.941  1.641  0.639  0.55  1.076  0.921  1.712  1.577  0.577  0.818  1.735  0.232  0.627  1.277  1.776  1.923  1.21  2.586  1.718  0.499  0.634  0.655  1.228  0.449  2.829  0.662  1.588  0.742  0.785  1.34  0.25  0.371  0.693  1.489  0.258  
 
 SMG5  23381  1  156219014  156252620  1q21.2  2.993  1.051  2.519  3.474  3.407  2.435  3.434  4.211  2.932  3.802  4.121  3.334  3.215  2.551  3.726  3.703  2.955  0.136  4.731  2.543  3.644  3.191  2.289  2.905  3.386  3.169  3.373  4.031  3.901  2.884  3.988  3.95  4.217  1.755  2.306  3.854  4.822  4.701  3.002  5.179  3.617  2.933  3.512  3.372  3.733  3.542  3.425  3.507  3.674  4.04  3.632  3.228  3.649  2.719  3.148  4.172  4.05  2.948  3.899  3.58  
 
 TMEM79  84283  1  156252703  156262234  1q22  2.04  0.543  1.062  1.567  3.262  0.711  2.548  1.015  1.135  1.957  1.355  1.408  1.436  0.963  1.129  1.044  0.973  0.373  2.863  1.026  1.63  1.784  1.438  0.876  0.6  1.281  1.771  1.18  0.74  0.654  1.304  0.972  1.931  0.7  1.043  1.917  2.114  2.181  1.392  2.955  2.307  1.609  1.209  0.543  0.883  1.625  2.181  0.982  0.777  1.645  1.614  1.86  1.482  1.392  1.461  0.974  1.074  1.321  1.607  0.956  
 
 GLMP  112770  1  156262477  156265480  1q22  3.621  1.23  2.51  4.336  3.845  0.59  3.211  4.374  2.151  3.875  3.02  3.732  3.209  2.839  1.852  2.954  3.472  0.364  2.786  2.115  2.338  1.775  3.288  3.751  2.356  5.357  3.959  4.186  4.732  5.325  6.588  5.625  4.986  3.902  2.498  4.213  3.464  5.168  3.777  2.221  2.245  3.615  0.167  0.129  3.009  3.882  2.266  2.874  1.187  4.926  2.827  3.214  0.076  3.792  4.324  4.559  2.329  3.156  3.886  3.626  
 
 VHLL  391104  1  156268414  156269428  1q22  0  0  0.261  0  0.275  0  0  0.07  0.137  0.226  0.15  0  0  0.057  0.146  0.119  0  0  0.077  0  0  0.272  0.604  0  0  0  0.383  0.138  0  0.129  0.137  0  0.228  0.43  0  0  0  0.185  0.063  0.168  0  0.472  0.061  0  0  0.161  0  0  0  0.194  0  0.454  0.111  0.135  0.206  0  0  0  0  0  
 
 CCT3  7203  1  156278751  156308206  1q23  6.364  5.146  5.216  6.075  6.472  4.86  7.108  6.111  5.194  5.782  6.859  5.854  6.424  7.7  5.903  5.78  6.23  4.085  7.746  6.975  6.125  6.386  5.845  6.142  6.247  5.888  5.911  5.506  5.776  5.718  6.522  7.086  6.107  4.441  5.709  6.545  6.786  6.542  6.602  7.779  6.081  6.383  7.253  6.332  5.542  5.872  6.467  5.847  5.03  8.043  6.336  5.483  6.139  5.495  6.626  6.618  5.908  5.633  6.615  6.272  
 
 TSACC  128229  1  156307104  156316785  1q22  0.944  1.885  0.409  0  0.864  0.582  0.695  0.563  0  0.598  0.461  0.441  0.576  1.199  0.784  0.646  0.877  0.996  0.782  0.729  0  1.329  1.08  0.494  0.478  0.595  0.3  0.283  0  0.173  0  0.425  0  0.62  0.833  0.528  0.491  0.479  1.565  1.094  1.472  1.751  2.512  0.768  0.714  0.71  0.336  0.451  0.693  1.114  0.421  1.366  0.659  0.868  0.719  0.704  0.379  0  0.383  0  
 
 RHBG  57127  1  156338979  156355013  1q21.3  1.165  0  0.202  0  0.341  0  0.242  0.1  0.189  0.164  0.114  0.087  0.27  1.096  0.258  0.124  0.187  0.077  0.099  0.09  0  0.186  0.364  0.08  0  0.11  0.18  0.09  0.163  0.125  0.132  0.32  0.092  0.23  0  0.124  0.117  0  0.174  0.163  0.152  0.32  0.292  0  0  0.142  0.373  0.121  0  0.145  0.089  0.487  0.07  0.146  0.152  0.18  0.098  0  0.085  0  
 
 C1orf61  10485  1  156374054  156399184  1q22  0  0  0  0  0  0  0.253  0.277  0  1.157  0  0  3.188  2.036  0  0  0  2.462  0.177  0  0  0.274  0  0  0  0  0.079  0.447  0  0.081  0  0.407  0  0  0.771  0  0.113  0  0.107  0.879  4.241  0.398  0.059  0  0  0.117  0  0.65  0  1.492  0  0  0  0.095  0  0  0  0  0  0  
 
 MEF2D  4209  1  156433512  156470634  1q12-q23  1.992  0.87  2.197  2.572  2.544  1.589  2.815  4.606  2.067  2.822  2.635  1.518  2.063  2.616  2.218  2.127  2.422  0.307  2.616  2.554  1.356  2.02  2.308  3.284  3.573  3.979  2.769  3.556  3.127  3.405  3.446  3.365  3.239  1.747  2.741  4.32  3.425  4.145  2.322  4.915  3.026  2.186  3.598  2.438  1.889  2.803  1.977  2.337  1.92  2.398  2.65  3.15  2.082  2.559  2.565  3.211  2.792  3.416  3.124  2.613  
 
 IQGAP3  128239  1  156495196  156542396  1q21.3  2.502  2.963  2.033  1.803  2.519  2.405  2.131  3.159  0.383  3.36  3.329  1.766  2.794  2.382  2.626  2.143  2.273  1.169  2.046  1.282  1.388  1.117  0.657  0.861  1.573  3.093  3.16  3.656  2.423  1.107  1.677  1.958  3.986  1.568  2.019  2.413  3.624  3.983  2.658  3.693  2.208  2.214  1.878  2.168  1.688  3.06  2.326  2.921  2.854  2.693  3.209  2.903  2.44  2.491  3.274  3.064  3.301  2.493  3.707  2.734  
 
 TTC24  164118  1  156549518  156556562  1q23.1  0  0  0.155  0  0.06  0  0.55  0.162  0.122  0.691  0.095  0.11  0.555  0.134  0  0.086  0.572  0.061  1.988  0.148  0.159  0.77  0.402  0  0  0.154  0.344  0.283  0.092  0.338  0.258  0.054  0.071  0.296  0.12  0  0.214  0.074  0.171  0.383  0  0.367  0.19  0  0  0.068  0.04  0.075  0.08  0  0.058  0.482  0.061  0.108  0.159  0.133  0.073  0  0.406  0  
 
 APOA1BP  128240  1  156561557  156564091  1q21  5.796  3.73  3.776  4.544  6.265  4.553  5.503  4.811  4.522  4.747  5.602  4.473  4.963  5.661  4.285  4.832  4.705  2.798  6.294  4.979  4.191  5.209  4.896  5.198  4.484  5.226  4.154  4.403  4.699  5.445  6.343  5.711  5.087  3.842  5.01  5.863  4.719  5.493  5.375  6.204  5.615  5.48  6.908  4.502  4.055  5.805  5.255  3.533  4.395  6.109  4.918  4.265  4.371  4.027  5.48  5.083  3.652  5.12  5.287  5.54  
 
 GPATCH4  54865  1  156564099  156571279  1q22  4.422  4.584  3.081  3.747  3.981  3.162  5.164  3.779  2.618  3.005  4.43  3.902  4.026  4.643  4.12  3.709  3.235  3.67  5.123  4.198  4.354  4.311  3.693  4.313  4.14  3.922  3.932  4.236  3.466  3.982  4.097  5.042  4.624  3.333  3.836  4.661  4.415  4.037  4.035  4.898  4.419  3.377  4.783  4.321  3.347  3.814  4.589  3.255  3.184  4.322  3.847  3.716  4.073  2.993  4.149  4.008  3.339  3.602  4.188  3.638  
 
 HAPLN2  60484  1  156589085  156595517  1q23.1  0  0.061  0.089  0.211  0.178  0.196  0.054  0.135  0.186  0.426  0.108  0.126  0.143  0.261  0  0.122  0.155  0.363  0  0.112  0  0.346  0.391  0.097  0  0.112  0.221  0.063  0.133  0.192  0.09  0  0  0.186  0.105  0  0.113  0  0.283  0.115  0.111  1.042  0.111  0  0.208  0.153  0.084  0  0  0.199  0.096  1.299  0.085  0.365  0.189  0.138  0  0  0.082  0  
 
 BCAN  63827  1  156611739  156629324  1q31  0.383  0.054  0.295  0  0.263  0.332  0.359  0.121  0.986  0.28  0.902  0.665  1.506  0.96  1.037  1.528  1.143  0.102  0.187  0.385  0.931  0.506  0.628  0.088  0.662  4.389  1.305  1.842  0.457  3.322  4.011  1.205  1.758  1.079  0.85  0.334  1.53  0.514  0.486  0.463  1.244  2.799  0.501  0.785  0.11  0.205  0.151  0.247  0.315  0.108  0.27  1.818  0.533  1.037  1.15  0.541  0.289  0.426  0.453  0.633  
 
 NES  10763  1  156638555  156647189  1q23.1  0.871  0.103  0.255  0.077  0.23  0.08  3.493  0.126  4.873  4.978  5.987  0.222  3.38  1.343  0.248  0.198  0.089  0.785  0.068  0.048  0.676  0.525  0.542  0.153  0.025  4.35  4.997  3.954  0.058  1.627  4.439  3.437  4.942  2.598  0.362  0.532  2.112  2.586  0.087  2.297  0.048  2.705  0.363  2.061  0.402  1.62  2.869  3  0.577  3.479  4.268  0.896  0.247  0.156  0.425  0.573  3.296  0.214  0.682  0.245  
 
 CRABP2  1382  1  156669399  156675608  1q21.3  6.326  0.699  0.508  0.963  8.386  0.68  3.425  3.436  1.516  1.608  3.14  0  5.836  6.444  2.545  6.839  1.146  1.656  1.163  0.475  0  0.606  0.622  0.37  0.793  0.418  0.355  0.308  0.163  0.239  0.464  0.476  0  0.19  0.708  2.377  4.07  4.329  1.23  4.552  3.063  1.878  0.38  1.039  3.752  4.897  2.386  2.115  0.648  5.622  4.698  2.469  2.76  4.058  2.827  3.738  2.855  0.868  2.386  1.678  
 
 ISG20L2  81875  1  156691682  156698231  1q23.1  3.803  2.441  2.098  3.303  4.093  1.977  3.635  3.425  2.796  3.318  3.735  3.112  3.912  3.081  3.364  3.247  3.166  1.718  4.068  3.874  3.899  3.475  2.924  3.032  3.334  2.944  2.551  3.52  2.244  2.114  3.157  3.914  4.203  2.892  2.547  3.916  3.684  3.116  3.249  4.479  3.544  3.102  3.989  3.563  2.95  3.459  3.142  2.222  2.965  3.186  3.646  2.455  3.018  2.846  3.103  3.541  2.777  2.221  3.542  3.12  
 
 RRNAD1  51093  1  156698262  156706752  1q23.1  2.56  1.043  1.079  2.315  3.332  1.596  2.506  1.614  2.555  2.453  2.357  2.481  2.574  2.252  2.18  2.608  2.409  1.635  2.602  2.124  2.356  1.843  2.856  2.208  0.979  1.885  1.655  1.746  1.466  1.649  2.233  1.731  2.207  1.506  1.856  2.294  2.463  2.565  2.28  2.877  2.561  2.437  1.955  1.085  1.072  2.073  3.393  1.19  1.756  2.72  1.706  1.981  1.491  1.923  2.435  2.369  1.407  1.189  1.777  1.511  
 
 MRPL24  79590  1  156707093  156710923  1q23.1  4.82  2.469  3.975  4.044  5.375  3.907  5.199  4.189  3.903  4.259  4.483  4.769  3.825  4.837  3.308  4.5  4.286  0.468  5.332  3.437  3.951  4.395  3.916  4.212  4.378  4.691  3.548  3.847  4.365  3.861  5.32  5.145  3.838  2.898  5.111  5.217  4.519  4.991  4.644  5.493  4.832  4.77  4.491  4.497  3.73  4.623  4.742  3.311  3.613  5.095  4.354  3.87  4.653  3.865  5.585  5.136  3.439  4.733  4.498  4.06  
 
 HDGF  3068  1  156711898  156722240  1q21-q23  6.802  5.005  5.825  6.344  6.493  5.244  6.192  6.026  4.932  6.245  6.531  6.01  6.81  6.222  5.535  6.792  6.579  3.757  7.036  6.805  5.907  5.775  5.721  5.794  6.517  5.269  4.983  4.862  5.303  4.55  5.781  6.248  6.256  4.993  5.802  6.778  6.403  6.427  6.271  7.201  6.614  6.488  6.295  6.388  5.107  6.181  6.111  5.623  6.269  6.636  6.041  5.563  6.184  5.709  6.696  6.716  6.411  6.47  7.406  6.45  
 
 PRCC  5546  1  156737273  156770609  1q21.1  3.983  2.001  2.671  3.945  4.62  2.882  3.7  3.898  3.641  4.013  4.405  3.107  3.844  3.502  3.627  3.666  3.533  1.12  4.875  2.744  3.803  4.045  3.47  3.652  3.585  3.346  2.806  2.961  2.771  2.406  3.665  4.29  3.791  2.51  3.133  4.17  3.665  3.806  3.744  4.603  4.011  4.025  3.686  3.74  3.415  3.969  2.286  3.288  3.295  4.6  3.569  3.372  3.374  3.64  3.961  3.862  3.367  3.5  4.139  3.55  
 
 SH2D2A  9047  1  156776034  156786640  1q21  0.167  0  0  0  0.29  0  0.471  0  0.112  0.129  0  1.607  1.778  0.78  0.577  0.989  1.469  0.474  3.416  0.34  2.472  1.633  3.126  2.782  1.057  0.852  0.19  0.131  0  0.252  0  0  0  0.278  0.885  0.848  1.174  1.535  0.498  1.764  0  2.536  0.121  0.421  0.242  0.983  0.131  1.132  0  2.217  1.534  0.653  0.086  1.182  0.067  0  0.223  0.515  0.477  0.568  
 
 NTRK1  4914  1  156785541  156851642  1q21-q22  0.167  0  0.134  0  0.153  0.174  0.12  0.078  0.152  0.929  0.181  0.066  0  0.215  0.137  0.166  3.137  0.108  0.471  0.18  1.038  0.24  0.454  0  0.051  0.228  0.217  0.204  0.366  0.332  0.066  0.231  0  0.182  0.064  0.132  0.249  0.089  0.212  0.699  0.052  0.597  0.208  0  0  0.111  0.159  0.184  0  0.158  0.145  0.893  0.067  0.139  0.144  0  0.091  0  0  0  
 
 INSRR  3645  1  156810664  156828712  1q21-q23  0.07  0.029  0.122  0.095  0.062  0  0  0.053  0.137  0.925  0.13  0.06  0.07  0.112  0.052  0.065  0.073  0.149  0  0.129  0.043  0.264  0.653  0.042  0.04  0.05  0.303  0.136  0.038  0.189  0.06  0.062  0  0.27  0.091  0.044  0.073  0  0.185  0.044  0.052  0.634  0.161  0.048  0.062  0.096  0.335  0.044  0.154  0.039  0.082  0.668  0.059  0.223  0.364  0.117  0.073  0.053  0.058  0.037  
 
 PEAR1  375033  1  156863522  156886226  1q23.1  0  0.037  0.195  0.079  0.654  0.177  0.149  0.062  0.109  0.077  0.958  0.27  0.678  1.091  0.335  0.139  0.026  0.032  0.032  0.083  0.777  0.076  0.445  0.073  2.116  0.062  0.092  0.244  0.05  0.091  0.031  0.049  0.029  0.183  0.418  0.687  2.333  0.398  1.045  0.194  0.043  0.289  0.089  0.131  0.07  0.191  1.812  0.901  0.667  0.956  3.073  1.244  0.848  0.823  2.356  1.838  0.526  2.875  1.901  2.161  
 
 LRRC71  149499  1  156890423  156902880  1q23.1  0.305  0  0.115  0  0.154  0  0.367  0.061  0.09  0.639  0.539  0.478  0  0.432  0  0.994  0.801  0.194  0  0.501  0.152  0.741  0.224  0.061  0  0.06  0.718  0.14  0.372  0.322  0  0  0  0.147  0.895  0.088  0.061  0  0.708  0.081  0.071  0.465  0.467  0.196  0  0.086  0.064  0  0.257  0.047  0.61  0.884  0.061  0.555  1.06  0.866  0.428  0.135  0  0  
 
 ARHGEF11  9826  1  156904631  157015162  1q21  2.263  0.735  1.274  1.45  2.536  1.162  2.472  2.437  1.853  2.85  2.571  1.594  2.221  2.126  2.477  1.935  1.692  0.186  0.95  0.551  1.432  0.912  0.924  0.958  1.944  4.189  2.961  4.416  3.511  2.304  3.041  3.689  3.587  1.388  1.7  3.214  2.682  2.932  1.56  3.948  2.58  1.718  3.04  2.337  1.472  2.239  2.747  1.841  2.177  1.947  2.21  1.983  1.975  1.859  2.376  2.249  2.39  1.649  2.125  2.275  
 
 ETV3L  440695  1  157061834  157069600  1q23.1  0  0  0.105  0  0.07  0  0  0.053  0.165  0.059  0.063  0  0  0.047  0  0.059  0.093  0.123  0.036  0.061  0  0.12  0.473  0.055  0.077  0.085  0.079  0.093  0.073  0.042  0.094  0.071  0.093  0.171  0  0.072  0  0  0.045  0.082  0  0.184  0.231  0.082  0.124  0.1  0  0  0  0  0.043  0.285  0.084  0.11  0.108  0  0.053  0  0  0  
 
 ETV3  2117  1  157094458  157108383  1q21-q23  1.856  0.781  0.818  0.523  1.739  0.941  1.357  1.874  0.527  1.408  1.653  1.16  1.292  1.643  1.145  1.146  0.816  0.263  0.492  0.739  1.368  0.781  0.884  1.084  1.173  2.422  1.425  1.932  1.944  0.87  1.59  1.404  1.754  0.709  0.905  1.442  1.361  1.449  0.959  2.314  1.713  0.648  1.588  1.634  1.179  1.195  0.711  1.199  1.42  0.602  1.176  1.119  1.288  1.505  1.2  1.473  1.28  1.418  1.379  1.185  
 
 CYCSP52  360155  1  157098153  157098463  1q22  1.039  1.226  0  0  1.151  0  1.029  0.985  0  0.696  0.875  0  0  0.917  0.714  0.825  0  0  0  0  0  0.196  0  0  0  1.294  0.613  0.747  0.901  0.607  0  0.828  1.525  0.725  0  0  0.651  0  0.314  0.877  1.118  0.393  2.536  0  0  0  0  0.762  0  0  0.692  0  0.322  0  0.506  0  0  1.581  0.627  0  
 
 FCRL5  83416  1  157483166  157522310  1q21  0  0  0.106  0  0.017  0  0  0  0.152  0.016  0  0  0  0.015  0  0  0  0.04  0.017  0.037  0  0.037  0.368  0  0  0  0.044  0.015  0.018  0.061  0  0  0  0.272  0  0  0  0  0.032  0  0  0.128  0.066  0  0  0.034  0.065  0  0  0.017  0.024  0.243  0  0.037  0.018  0  0  0  0  0  
 
 FCRL4  83417  1  157543538  157567870  1q21  0  0  0.08  0  0  0  0  0  0.044  0  0  0  0  0.013  0  0  0  0  0.034  0.026  0  0.03  0.215  0  0  0  0.038  0  0  0.034  0  0  0  0.215  0  0  0  0  0  0  0  0.026  0.037  0  0  0  0.015  0  0  0  0  0.153  0  0.018  0  0  0  0  0  0  
 
 FCRL3  115352  1  157647977  157670647  1q21-q22  0  0  0.072  0  0  0  0  0  0.091  0.017  0  0  0  0.015  0  0  0  0.032  0  0.035  0  0.046  0.299  0  0  0  0.042  0  0  0.031  0  0  0  0.245  0  0  0  0  0.037  0  0.05  0.152  0.026  0  0  0  0  0  0  0  0  0.057  0  0.039  0.025  0  0  0  0  0  
 
 FCRL2  79368  1  157715522  157746922  1q21  0  0  0  0  0  0  0  0  0.087  0  0  0  0  0  0  0  0  0.085  0  0  0  0.025  0.338  0  0  0  0.045  0.071  0  0.044  0  0  0  0.206  0  0  0  0  0  0  0  0.087  0.027  0  0  0  0.052  0  0  0  0  0.17  0  0  0  0  0  0  0  0  
 
 FCRL1  115350  1  157764193  157789940  1q21-q22  0  0  0  0  0  0  0.037  0  0.092  0.022  0  0.042  0  0  0  0  0  0  0  0.053  0  0.033  0.249  0  0  0  0.107  0.158  0  0.036  0  0  0  0.161  0  0  0  0  0  0  0  0.039  0.037  0  0  0.027  0.046  0  0  0  0  0.071  0.023  0  0  0  0  0  0  0  
 
 CD5L  922  1  157800703  157811634  1q21-q23  0  0.057  0.121  0  0  0.08  0.108  0  0.094  0  0  0  0  0.026  0  0  0  0.044  0  0  0  0.039  0.388  0  0  0  0.06  0.801  0  0.489  0  0  0.078  0.242  0  0  0  0  0  0  0  0.041  0.032  0  0  0  0.022  0  0  0  0  0.108  0  0  0  0  0  0  0  0  
 
 KIRREL  55243  1  157963062  158070052  1q21-q25  0.055  1.593  2.858  3.599  0.817  2.505  3.443  4.703  2.869  4.677  4.008  0.059  0.766  1.635  1.332  0.58  0.035  0.332  1.409  0.066  0.275  1.148  0.502  0.078  3.376  3.99  3.741  3.885  4.267  1.794  3.003  4.322  3.984  1.688  1.858  3.037  3.252  3.675  2.631  3.34  1.431  2.065  0.831  4.75  3.193  3.472  2.355  2.589  4.15  3.192  2.716  2.765  4.458  4.146  4.402  5.945  4.505  1.216  4.172  4.47  
 
 LOC646268  646268  1  158101833  158110430  1q23.1  0  0  0.169  0  0  0  0  0  0  0.086  0  0  0  0  0  0  0  0  0  0  0  0  0  0  0  0  0.133  0  0  0.062  0  0  0  0  0  0  0  0  0.133  0  0  0  0  0.447  0  0  0  0  0  0  0  0.416  0  0.108  0  0  0  0  0  0  
 
 CD1D  912  1  158149736  158156216  1q22-q23  0  0.025  0.092  0.085  0.025  0  0.062  0  0.13  0.068  0.149  0.042  0  0.028  0  0.023  0.034  0  3.83  0.178  0  1.96  0.371  0  0  0  0.145  0  0  0.121  0  0.054  0  0.245  0  0  0.054  0  0.103  0  0  0.322  0.047  0.209  0  0.084  0.063  0  0  0.044  0  0.333  0  0.111  0.035  0.531  0  0  0  0.055  
 
 CD1A  909  1  158223926  158228058  1q22-q23  0  0  0.109  0  0  0  0.187  0  0.089  0.063  0  0  0  0  0  0.036  0  0  4.505  0  0  4.43  0.364  0  0  0.152  0.06  0  0  0.022  0  0  0  0.323  0  0  0  0  0.04  0  0  0.285  0.027  0  0  0.1  0  0  0  0.084  0  0.141  0  0.033  0  0  0  0  0  0  
 
 CD1C  911  1  158259562  158264564  1q22-q23  0  0  0.06  0  0  0  0  0  0.076  0  0  0  0  0.017  0  0  0  0  4.588  0  0  2.547  0.153  0  0  0  0.029  0  0  0.023  0  0  0  0.258  0  0  0  0  0.031  0  0  0.347  0  0  0  0  0  0  0  0.094  0  0.143  0  0.047  0  0  0  0  0  0  
 
 CD1B  910  1  158297739  158301321  1q22-q23  0.183  0  0  0  0.06  0  0.713  0  0  0.251  0.081  0.625  0  0  0  0  0  0  4.216  0.16  0  6.301  0  0.099  0  0  0.861  0  0  0.677  0  0  0  0  0.075  0  0  0  0.114  0  0  0.16  0.057  0  0  0.067  1.519  0  0  0  0  0.224  0  0.056  0  0.121  0.068  0  0  0  
 
 CD1E  913  1  158323485  158327343  1q22-q23  0  0  0.231  0  0  0  0.52  0  0.155  0.195  0  0  0  0  0  0  0  0  6.619  0  0  6.303  0.314  0  0  0  0.232  0  0  0.191  0  0  0  0.261  0  0  0  0  0  0  0  0.104  0  0  0  0  0.348  0  0  0.286  0  0  0  0  0  0  0  0  0  0  
 
 OR10T2  128360  1  158368311  158369256  1q23.1  0.648  0  0.229  0  0  0  0  0  0  0  0  0  0  0  0  0  0  0  0  0  0  0.409  0.489  0  0  0  0  0  0  0  0  0  0  0.363  0  0  0  0  0  0  0  0.245  0  0  0  0  0  0  0  0  0  0.233  0  0  0  0  0  0  0  0  
 
 OR10K2  391107  1  158389717  158390656  1q23.1  0.858  0  0  0  0  0  0  0  0.134  0  0  0  0  0  0  0  0  0  0  0  0  0.057  0.512  0  0  0  0  0  0  0  0  0  0  0.413  0  0  0  0  0  0  0  0.148  0  0  0  0  0  0  0  0  0  0  0  0  0  0  0  0  0  0  
 
 OR10K1  391109  1  158435351  158436293  1q23.1  0  0  0.171  0  0  0  0  0  0  0  0  0  0  0  0  0  0  0  0  0  0  0.125  0.426  0  0  0  0.24  0  0  0.052  0  0  0  0.38  0  0  0  0  0.071  0  0  0  0  0  0  0  0  0  0  0  0  0.349  0  0.152  0  0  0  0  0  0  
 
 OR10R2  343406  1  158449667  158450675  1q23.1  0  0  0  0  0  0  0  0  0  0  0  0  0  0  0  0  0  0  1.025  0  0  1.179  0  0  0  0  0  0  0  0.039  0  0  0  0.278  0  0  0  0  0  0  0  0  0  0  0  0  0  0  0  0  0  0  0  0  0.068  0  0  0  0  0  
 
 OR6Y1  391112  1  158516917  158517895  1q23.1  0  0  0.246  0  0  0  0  0  0  0  0  0  0  0  0  0  0  0  0  0  0  0  0.512  0  0  0  0  0  0  0  0  0  0  0.381  0  0  0  0  0  0  0  0.113  0  0  0  0  0  0  0  0  0  0  0  0  0  0  0  0  0  0  
 
 OR6P1  128366  1  158532440  158533394  1q23.1  0  0  0.2  0  0  0  0  0  0.132  0  0  0  0  0  0  0  0  0  0  0  0  0.129  0.262  0  0  0  0  0  0  0  0  0  0  0.238  0  0  0  0  0  0  0  0  0  0  0  0  0  0  0  0  0  0.259  0  0  0.088  0  0  0  0  0  
 
 OR10X1  128367  1  158548708  158549689  1q23.1  0  0  0.168  0  0  0  0  0  0  0  0  0  0  0  0  0  0  0  0  0  0  0  0  0  0  0  0  0  0  0  0  0  0  0.38  0  0  0  0  0.047  0  0  0.291  0  0  0  0  0  0  0  0  0  0.361  0  0  0  0  0  0  0  0  
 
 OR10Z1  128368  1  158576228  158577170  1q23.1  0  0  0.148  0  0  0  0  0  0.196  0  0  0  0  0  0  0  0  0  0  0  0  0  0.571  0  0  0  0.054  0  0  0.063  0  0  0  0.264  0  0  0  0  0  0  0  0  0  0  0  0  0  0  0  0  0  0  0  0  0  0  0  0  0  0  
 
 SPTA1  6708  1  158580495  158656506  1q21  0  0.026  0.076  0  0.035  0  0.06  0.029  0.057  0.032  0.011  0.041  0.02  0.069  0  0.019  0.036  0.031  0.146  0.031  2.04  0.064  0.189  0  0.017  0.019  0.05  0.044  0.028  0.044  0.05  0  0  0.147  0.043  0  0.043  0  0.045  0.045  0  0.122  0.126  0  0.023  0.009  0.107  0  0  0.021  0.019  0.089  0.043  0.041  0.019  0.033  0.012  0  0.015  0  
 
 OR6K2  81448  1  158669467  158670442  1q23.1  0  0  0  0  0  0  0  0  0  0  0  0  0  0  0  0  0  0  0  0  0  0.081  0.433  0  0  0  0.07  0  0  0.053  0  0  0  0.472  0  0  0  0  0  0  0  0.325  0  0  0  0  0.062  0  0  0  0  0  0  0  0  0  0  0  0  0  
 
 OR6K3  391114  1  158686957  158687905  1q23.1  0  0  0  0  0  0  0  0  0.133  0  0  0  0  0  0  0  0  0  0  0  0  0  0.357  0  0  0  0.042  0  0  0  0  0  0  0.192  0  0  0  0  0  0  0  0  0  0  0  0  0  0  0  0  0  0.257  0  0  0  0  0  0  0  0  
 
 OR6K6  128371  1  158724605  158725637  1q23.1  0  0  0.134  0  0  0  0  0  0  0  0  0  0  0  0  0  0  0.109  0  0  0  0  0.285  0  0  0  0.058  0  0  0.062  0  0  0  0.361  0  0  0  0  0  0  0  0  0  0  0  0  0  0  0  0  0  0  0  0  0  0  0  0  0  0  
 
 OR6N1  128372  1  158735533  158736472  1q23.1  0  0  0.176  0  0  0  0  0  0  0.073  0  0  0  0  0  0  0  0  0  0  0  0.081  0.649  0  0  0  0.061  0  0  0.052  0  0  0  0.475  0  0  0  0  0  0  0  0  0  0  0  0  0  0  0  0  0  0.214  0  0  0  0  0  0  0  0  
 
 OR6N2  81442  1  158746471  158747425  1q23.1  0  0.135  0.227  0  0.108  0.226  0.341  0.109  0  0.103  0.097  0.108  0.234  0  0  0.159  0.185  0.147  0.122  0.205  0.437  0.229  0.355  0.142  0.134  0.116  0.173  0.091  0.14  0.143  0.151  0.292  0.197  0.452  0.2  0  0.201  0.213  0  0.18  0  0.172  0.11  0.339  0.176  0.18  0.224  0.195  0  0.18  0.273  0.231  0.118  0  0.109  0  0  0.174  0.215  0  
 
 MNDA  4332  1  158801167  158819270  1q22  0  0  0  0  0  0  0  0  0  0  0  0  0  0  0  0  0  0  0  3.903  0  0.054  0  0  0  0  0.031  0  0  0.025  0  0  0  0.213  0  0  0  0  0.063  0  0  0.237  0  0  0  0  0  0  0  0  0  0.121  0  0  0  0  0  0  0  0  
 
 PYHIN1  149628  1  158901336  158946849  1q23.1  0  0  0.209  0  0  0.071  0  0.107  0.091  0.442  0.097  0  0  0  0  0.131  0  0  2.503  0  0  0.417  0.489  0.2  0.317  0.353  0.615  0.113  0.646  0.212  0.221  0.5  0.664  0.573  0  0  0.093  0  0.544  0  0  0.072  0  0  0  0  0  0  0  0.094  0.076  0.134  0.103  0.2  0.285  0.125  0.136  0  0  0.11  
 
 IFI16  3428  1  158979681  159024945  1q22  0  4.286  2.679  1.843  0  1.681  3.348  3.193  3.37  4.878  3.665  0  0.055  0  0  3.063  0  0.041  6.95  1.331  0.072  4.599  4.542  4.482  4.867  4.719  4.404  3.201  4.527  3.274  5.191  6.104  4.626  3.867  0.056  0.534  3.077  1.236  4.963  0.658  0.714  2.85  0.135  0  0  1.35  2.208  1.204  1.2  4.025  1.982  0.284  1.868  3.616  3.564  3.161  4.544  1.96  1.749  3.657  
 
 AIM2  9447  1  159032274  159046647  1q22  0  1.755  0  0  0  0  0.448  0.327  0  0.049  0  0  0  0  0  0.063  0  0.154  0  0  0  0.059  0.956  0.555  1.913  0.371  0.071  0.33  0.579  0.049  0  0  0  0.218  0  0  1.417  2.206  0.041  0  0  0.287  0  0  0  0  0  0  0  0  0  0.104  0  0.092  0  0  1.722  0  0  0  
 
 CADM3  57863  1  159141376  159172932  1q21.2-q22  0  0  0.171  0  0.024  0.127  0.113  0.081  0  2.619  0  0.08  0  0.028  0  0  0  0.054  0  0.125  0  0.084  0.455  0  0  0.046  1.671  0.264  0  0.14  0.106  0  0  0.127  0  0.417  0.051  0  3.366  0  0  0.2  0.037  0  0  0.059  0.024  0  0  0  0  0.256  0  0.074  0  0.046  0  0  0  0  
 
 CADM3-AS1  100131825  1  159165774  159172212  1q23.2  0  0  0.126  0  0.02  0  0  0  0.139  0.274  0.022  0  0  0.022  0  0.032  0  0  0  0  0  0.034  0.344  0  0  0  0.858  0.027  0  0.048  0  0  0  0.237  0  0  0  0  0.716  0  0.031  0.232  0.025  0  0  0  0.031  0  0  0  0  0.105  0  0.036  0.03  0  0  0  0  0  
 
 ACKR1  2532  1  159173802  159176290  1q21-q22  0  0  0.11  0  0  0  0.059  0  0.246  0.874  0  0  0  0  0  0  0  0  0  0.099  0  0.098  0.699  0  0  0  1.252  0  0  0.052  0  0  0  0.451  0  0.088  0  0  2.17  0  0  0.044  0.043  0  0  0  0  0  0  0  0  0.363  0  0.038  0.046  0  0  0  0  0  
 
 FCER1A  2205  1  159259503  159278014  1q23  0  0  0  0  0  0  0  0  0  0  0  0  0  0  0  0  0  0  0  0  0  0.057  0  0  0  0  0.037  0  0  0.037  0  0  0  0  0  0  0  0  0  0  0  0.093  0  0  0  0  0  0  0  0  0  0  0  0  0  0  0  0  0  0  
 
 OR10J3  441911  1  159283459  159284449  1q23.2  0  0  0.14  0  0  0  0  0  0.197  0  0  0  0  0  0  0  0  0  0  0  0  0  0.466  0  0  0  0.081  0  0  0.056  0.188  0  0  0.251  0  0  0  0  0  0  0  0.215  0  0  0  0  0  0  0  0  0  0.211  0  0  0  0  0  0  0  0  
 
 OR10J1  26476  1  159409511  159410600  1q23.2  0  0  0.126  0  0  0  0  0  0.24  0  0  0  0  0.051  0  0  0  0  0  0  0  0.092  0.564  0  0  0  0.056  0  0  0.04  0  0  0  0.351  0  0  0  0  0.048  0  0  0.628  0  0  0  0  0  0  0  0  0  0.188  0  0  0  0  0  0  0  0  
 
 OR10J5  127385  1  159504867  159505797  1q23.2  0  0  0.15  0  0  0  0  0  0  0  0  0  0  0  0  0  0  0  0  0  0  0.134  0.365  0  0  0  0  0.089  0  0.053  0  0  0  0.311  0  0  0  0  0  0  0  0.069  0  0  0  0  0  0  0  0  0  0.173  0  0  0  0  0  0  0  0  
 
 APCS  325  1  159557615  159558661  1q21-q23  0  0  0.15  0  0  0  0  0  0  0  0  0  0  0  0  0  0  0  0  0  0  0.078  0.269  0  0  0  0  0  0  0.049  0  0  0  0.232  0  0  0  0  0  0  0  0  0  0  0  0  0  0  0  0  0  0.385  0  0  0  0  0  0  0  0  
 
 CRP  1401  1  159682078  159684379  1q23.2  0  0  0.119  0  0  0  0  0  0.17  0.055  0  0  0  0.024  0  0  0  0  0  0  0  0.069  0.391  0  0  0  0.092  0  0  0.087  0  0  0  0.477  0  0  0  0  0.067  0  0  0.2  0.198  0  0  0  0.108  0  0  0  0  0.112  0  0.072  0  0  0  0  0  0  
 
 DUSP23  54935  1  159750758  159752333  1q23.2  4.362  3.585  2.973  4.05  4.866  0.253  3.679  3.951  0.414  0.183  0.194  4.506  3.767  4.432  2.335  4.114  0  0.198  0.331  5.103  2.721  2.132  4.721  4.343  0.447  3.871  4.26  3.937  3.473  4.512  5.212  4.32  4.371  3.76  4.355  4.653  2.128  0.766  4.175  5.06  2.948  3.325  0.201  0.246  2.711  3.823  0  3.268  2.629  4.867  2.458  4.376  3.779  4.397  5.358  5.024  3.361  0.884  5.049  2.939  
 
 FCRL6  343413  1  159770300  159786047  1q23.2  0  0  0.107  0  0  0  0.068  0.071  0  0.144  0  0  0  0.028  0  0  0  0  0  0  0  0.053  0.494  0  0  0.054  0.069  0.048  0  0.034  0  0  0  0.27  0  0.071  0  0  0  0  0  0.21  0  0  0  0  0  0  0  0  0  0.234  0  0.053  0.037  0  0  0  0.098  0  
 
 SLAMF8  56833  1  159796478  159807282  1q23.2  0.118  0  0.386  0.254  0.259  0.19  0.305  0.395  0.276  0.381  0.21  0.157  0.123  0.055  0.063  0.197  0.108  0  0.167  0.35  0.074  0.146  0.386  0.107  0  0.188  0.222  0.127  0.106  0.12  0.188  0.108  0.326  0.236  0.078  0.11  0.254  0.297  0.353  0.429  0.29  0.412  0.029  0  0.151  0.207  0.016  0.272  0.063  0.264  0.244  0.401  0.604  0.13  0.224  0.181  0.798  0  0.232  0.128  
 
 C1orf204  284677  1  159804263  159825137  1q23.2  0  0.128  0.09  0  0.107  0.081  0.045  0.196  0.188  0.342  0.232  0.209  0  0.158  0.058  0.036  0.063  0.085  0.062  0  0  0.072  0.214  0  0.111  0.067  0.066  0  0.074  0.05  0.11  0  0.145  0.256  0.102  0.367  0.208  0.103  0.041  0.291  0.204  0.063  0  0  0  0.046  0  0  0.142  0.106  0.283  0.299  0.188  0.161  0.108  0.08  0.254  0.117  0  0.112  
 
 VSIG8  391123  1  159824105  159832447  1q23.2  0  0.102  0.26  0  0.148  0.325  0.077  0.109  0.14  0.246  0.202  0.306  0  0.093  0.212  0.1  0.066  0.257  0  0.102  0  0.283  0.344  0  0  0.063  0.209  0.102  0.076  0.123  0.101  0.106  0.22  0.124  0.075  0.136  0.145  0.082  0.083  0.27  0.11  0.501  0.081  0  0.1  0.212  0.056  0.086  0.101  0.105  0.124  0.669  0.07  0.212  0.213  0  0.149  0.116  0.069  0  
 
 CFAP45  25790  1  159842153  159869906  1q22  0.353  0.487  0.271  0.196  1.322  0.545  0.349  0.253  0.079  0.336  0.641  0.224  0.133  0.242  0.132  0.484  0.245  0  0.267  0.118  0  0.264  0.338  0  0.475  0.067  0.239  0.199  0.07  0.064  0.257  0.288  0.241  0.191  0  0.278  0.978  0.197  0.723  0.469  0.239  0.399  0.042  0.107  0.454  0.215  0  0.108  0.417  0.357  0.392  0.529  0.252  0.301  0.318  0.353  0.209  0  0.196  0.233  
 
 TAGLN2  8407  1  159887896  159895332  1q21-q25  6.751  3.141  7.844  8.152  7.853  7.589  8.563  8.105  6.903  8.655  8.919  8.383  6.952  5.04  5.311  7.724  6.974  1.084  7.637  7.054  7.02  5.413  7.209  7.12  5.726  6.266  7.311  6.998  4.87  5.617  6.563  6.64  7.657  5.839  7.135  7.653  7.777  8.033  7.88  9.114  6.749  7.249  6.659  6.302  6.694  7.81  5.929  7.144  6.978  8.598  8.413  7.145  7.954  7.77  7.677  8.087  7.266  5.754  8.221  8.17  
 
 IGSF9  57549  1  159896828  159915386  1q22-q23  2.111  0  0.082  1.468  2.474  0.041  0.064  0.063  0.172  0.121  0.091  0.628  0.941  0.958  1.022  1.215  0.252  0.163  0  0.051  0.068  0.288  0.422  0.249  0  0.041  0.187  0.133  0.04  0.081  0.174  0  0  0.179  0.101  0.502  0.079  0.064  0.139  1.958  1.454  0.316  1.384  0.224  2.342  1.07  0.452  0.342  0  0.806  0.986  0.7  0.141  0.175  0.171  0.045  0.185  0  0.073  0.411  
 
 SLAMF9  89886  1  159921281  159924044  1q23.2  0  0  0  0  0.667  0  0.136  0  0  0.098  0.123  0.416  0  0.055  0  0  0  0  0  0.138  0  0.101  0.669  0  0  1.419  0.22  2.528  1.937  1  3.829  1.42  0.234  0.309  0  0  0  0  0.082  0  0.194  0  0  0  0  0  0  0  0  0  2.54  0.287  0  0  0  0  0  0  0  0  
 
 LINC01133  100505633  1  159931013  159948876  1q21-q23  0  0  3.819  3.635  0  0.384  1.697  0.261  0.129  0.579  0.077  0.155  1.43  0  0  0.257  0.332  0  0  0  0  0  0.37  0  0  0  0.057  0  0  0  0  0  0  0.379  0  0.164  0  0  3.21  0  0  0.082  0.038  0  1.207  1.085  0  0  0  0  5.167  0  0  0.321  0  0  0  0  0  0  
 
 PIGM  93183  1  159997461  160001783  1q23.2  2.544  1.016  0.955  0.706  3.301  0.687  1.74  1.479  1.603  1.855  1.237  1.19  1.765  1.543  1.504  1.362  0.819  1.652  2.764  1.82  1.26  1.579  1.86  1.955  1.043  1.695  0.96  1.627  0.738  0.816  1.636  1.453  1.669  1.132  1.332  2.198  0.986  1.381  1.229  1.91  1.667  1.13  1.723  1.248  1.165  1.173  1.908  0.939  1.16  1.509  1.671  1.36  0.86  0.53  1.271  0.788  1.031  0.993  1.133  1.49  
 
 KCNJ10  3766  1  160007256  160040051  1q23.2  0.061  0  0.185  0.083  0.153  0  0.041  0.036  0.43  0.52  0.092  0.076  0.079  0.107  0.116  0.059  0.037  0.05  0.086  0.061  0.043  0.124  0.505  0.03  0  0.989  0.205  0.431  0.257  0.064  0.423  0.083  0.055  0.345  0.036  0.02  0.055  0  0.072  0.117  0.045  0.205  0.039  0.042  0  0.062  0.424  0.035  0  0.039  0.06  0.205  0.069  0.076  0.047  0.038  0.051  0  0.049  0  
 
 KCNJ9  3765  1  160051359  160059212  1q23.2  0.215  0.069  0.398  0.269  0.477  0.128  0.264  0.111  0.328  0.336  0.243  0.281  0.169  0.255  0.258  0.191  0.225  0.218  0.294  0.215  0.365  0.714  0.601  0.237  0.238  0.507  0.671  0.311  0.241  0.311  0.326  0.123  0.168  0.406  0.346  0.191  0.166  0.226  0.314  0.239  0.049  0.732  0.355  0.343  0.146  0.246  0.228  0.074  0.357  0.215  0.298  1.363  0.255  0.328  0.269  0.31  0.219  0.057  0.348  0.27  
 
 IGSF8  93185  1  160061128  160068618  1q23.1  2.028  0.366  2.44  3.479  3.332  1.601  3.552  3.089  4.157  4.903  3.346  1.378  3.556  2.379  3.026  1.852  2.358  0.12  4.137  0.551  1.174  2.549  2.241  2.607  1.196  5.936  4.665  5.317  5.401  4.139  5.901  5.002  4.915  3.574  1.453  2.188  2.925  4.217  1.377  4.06  2.094  3.033  2.546  2.5  2.608  3.201  1.201  2.224  1.613  3.337  2.429  2.272  2.756  2.056  2.896  2.325  2.46  1.132  3.29  2.373  
 
 ATP1A2  477  1  160085519  160113374  1q23.2  0.101  0.059  0.12  0  0.03  0.053  0.025  0.057  0.119  0.046  0.028  0  0  0.035  0  0.02  0.08  0.075  0.015  0.055  0  0.105  0.363  0  0  0.022  0.096  0.029  0.019  0.095  0  0.048  0  0.175  0.032  0  0.058  0.271  0.069  0.053  0.112  0.17  0.068  0  0  0.096  0.027  0.032  0  0.017  0.027  0.364  0.025  0.119  0.13  0.082  0.029  0  0.042  0.032  
 
 ATP1A4  480  1  160121351  160156767  1q23.2  0.157  0  0.203  0  0  0  0  0  0.173  0.042  0  0  0  0.057  0  0  0.122  0.075  0  0  0  0.091  0.513  0  0  0  0.128  0  0  0.113  0  0  0  0.241  0  0  0.063  0  0.058  0  0  0.302  0.117  0  0  0.099  0.049  0  0  0  0  0.287  0  0.06  0.046  0.1  0  0  0  0  
 
 CASQ1  844  1  160160284  160171676  1q21  0  0  0.151  0  0  0  0.056  0.077  0.191  0.078  0.486  0  0  0.09  0  0  0  0  0  0  0  0.054  0.564  0  0  0.092  0.102  0  0  0.062  0  0  0  0.132  0.053  0  0  0.244  0.066  0  0  0.18  0.056  0  0  0.226  0.024  0  0  0  0.049  0.209  0.051  0.077  0.19  0  0  0  0  0  
 
 LOC729867  729867  1  160171988  160178659  1q23.2  0.469  0.181  1.08  0.935  1.056  2.152  0.594  1.25  0.874  1.628  1.02  0.589  0.239  0.428  0.432  0.78  0.495  0.089  0.196  0.185  0.361  0.559  0.642  0.575  0.812  0.677  0.867  0.763  0.965  0.392  0.64  0.768  0.889  0.272  0.493  0.515  0.731  1.054  2.049  0.557  0.515  1.083  0.371  0.475  0.829  1.272  0.729  1.256  0.979  0.325  0.364  0.958  1.078  1.456  1.776  1.181  0.776  1.221  0.914  0.704  
 
 PEA15  8682  1  160175108  160185166  1q21.1  3.647  4.67  5.709  4.384  4.937  6.147  5.668  6.431  5.274  6.774  6.072  3.447  3.74  3.844  2.792  3.824  2.917  0.716  2.968  2.011  3.25  2.567  3.535  3.088  5.663  5.636  5.405  5.393  5.698  3.832  4.976  5.406  5.527  3.696  3.708  4.172  5.564  6.624  6.336  4.887  4.461  4.231  4.885  4.222  4.97  5.952  5.212  5.146  4.832  5.571  4.919  3.287  5.218  5.637  6  5.879  5.25  6.324  5.516  6.335  
 
 DCAF8  50717  1  160185504  160232350  1q22-q23  3.255  2.682  1.663  2.111  3.349  1.977  3.22  2.688  2.688  2.97  3.26  2.544  3.22  3.066  3.327  2.63  2.226  1.748  3.748  2.482  2.698  2.83  3.128  3.038  2.505  3.527  2.936  3.516  2.888  2.767  3.019  3.183  3.078  1.877  2.383  2.978  3.077  3.221  2.516  4.173  2.764  2.449  3.8  2.824  1.747  2.903  3.031  2.385  2.533  3.227  3.014  2.693  2.781  1.973  3.202  3.197  2.542  2.7  3.136  2.932  
 
 PEX19  5824  1  160246598  160254941  1q23.2  2.818  3.426  2.209  2.438  3.643  1.505  3.25  2.834  3.061  3.023  3.398  2.813  2.806  3.208  2.605  3.13  2.996  2.453  3.886  2.548  2.684  2.696  2.407  2.255  2.579  3.45  2.331  2.776  2.961  2.332  3.591  3.477  3.113  2.106  1.857  2.781  3.484  3.666  3.164  3.823  3.198  2.124  3.474  2.484  1.828  2.937  2.325  1.884  2.079  3.546  3.889  2.221  2.648  2.216  3.176  2.983  2.471  2.577  2.798  3.038  
 
 COPA  1314  1  160258376  160313354  1q23.2  2.892  3.63  3.465  3.975  4.57  2.801  4.222  4.977  3.204  4.865  4.479  3.346  3.506  3.795  3.722  3.331  3.237  3.187  4.521  3.733  3.231  3.063  3.337  2.94  3.171  4.6  3.569  4.648  4.358  3.109  4.457  5.177  4.452  3.333  2.551  4.238  4.292  4.225  4.104  5.065  3.42  2.536  3.72  3.64  2.545  4.233  2.934  2.875  3.356  4.544  4.278  2.721  3.574  3.405  3.584  4.744  4.539  2.804  4.026  4.302  
 
 SUMO1P3  474338  1  160287054  160288260  1q22  2.606  3.407  1.989  2.404  1.736  1.567  2.289  1.502  2.898  1.897  1.806  1.496  2.075  3.24  1.517  2.711  2.345  3.942  2.786  2.144  0.878  2.114  2.564  0.931  2.177  1.554  1.636  1.519  2.44  1.74  1.507  1.663  2.005  2.429  1.853  1.141  1.636  1.274  2.2  1.516  1.508  1.842  3.891  1.239  1.66  1.474  2.108  1.248  1.296  2.59  1.561  0.772  1.836  1.618  2.792  1.569  1.871  2.201  2.065  1.099  
 
 NCSTN  23385  1  160313062  160328742  1q22-q23  3.029  2.263  3.233  4.701  5.104  2.541  4.993  4.717  4.326  5.153  4.384  3.236  3.98  3.476  3.028  3.764  3.611  0.894  4.062  4.018  3.119  3.091  3.77  3.443  3.777  4.841  4.053  4.164  4.234  3.69  3.965  4.933  4.302  3.142  2.577  4.531  4.189  4.749  4.677  5.296  4.224  3.681  4.401  4.156  2.492  3.857  4.964  2.778  3.196  4.361  3.549  3.59  4.06  3.73  4.344  4.389  4.328  3.159  4.311  4.654  
 
 NHLH1  4807  1  160336860  160342638  1q22  0.185  0  0.15  0.17  0.138  0.104  0.276  0.231  0.317  0.168  0.569  0.107  0  0.347  0  0.041  0.09  0.104  0.169  0.121  0.319  0.262  0.541  0  0.055  0.087  0.378  0.086  0.072  0.226  0.214  0.319  0.137  0.488  0.141  0.074  0.352  0.177  0.414  0.342  0.261  0.418  0.431  0.058  0.099  0.215  0.824  0.149  0  0.1  0.175  0.707  0.243  0.113  0.111  0  0.097  0.122  0.158  0.08  
 
 VANGL2  57216  1  160370363  160398468  1q22-q23  0.042  0.021  0.147  0  0.028  0.34  0.051  0.027  1.446  0.2  1.18  0.026  0.367  0.184  0.053  1.044  0  0.092  0.046  0.067  0.764  1.337  0.517  0  0  2.254  0.522  0.479  0.668  1.214  1.982  0.68  0  0.268  0.289  1.164  0.592  0.027  0.107  0.843  0.047  0.231  1.527  0.775  1.243  1.215  0.165  1.494  0  1.853  0.043  0.748  0.037  1.118  2.063  1.033  0.031  0  1.256  2.02  
 
 SLAMF6  114836  1  160454819  160493052  1q23.2  0  0  0.103  0  0  0  0  0.03  0.059  0.099  0.057  0.203  0  0  0  0  0  0.059  2.243  0.273  0.207  2.262  0.329  0  0  0  0.093  0.032  0  0.083  0  0  0  0.25  0  0  0  0  0  0  0  0.034  0.042  0  0.088  0.038  0.025  0  0  0.115  0  0.247  0  0  0.085  0  0  0  0  0  
 
 CD84  8832  1  160510883  160549306  1q24  0  0.032  0.114  0  0  0  0.034  0.011  0.081  0.014  0.012  0.021  0  0.014  0  0  0.022  0  1.934  1.23  0.104  2.305  0.363  0  0  0  0.045  0  0  0.036  0  0  0  0.288  0  0  0  0  0.017  0  0  0.037  0.021  0  0  0.017  0.027  0  0  0.013  0  0.104  0.015  0.016  0  0.02  0  0  0  0  
 
 SLAMF1  6504  1  160579608  160617101  1q23.3  0  0  0  0  0  0  0  0  0.093  0  0  0  0  0  0  0  0  0.127  0  0  0  0.063  0.227  0  0  0  0.051  0  0  0.044  0  0  0  0.203  0  0  0  0  0  0  0  0  0.027  0  0  0  0  0  0  0  0  0.114  0  0  0  0  0  0  0  0  
 
 CD48  962  1  160648535  160681641  1q21.3-q22  0  0  0.13  0  0  0  0.144  0  0.155  0.058  0  0  0  0  0  0  0  0  1.642  3.789  0  3.652  1.062  1.126  0  0  0.128  0.063  0  0.082  0  0  0  0.285  0  0  0  0  0  0  0  0.085  0.058  0  0  0  0.053  0  0  0  0  0.229  0  0.088  0  0  0  0  0  0  
 
 SLAMF7  57823  1  160708846  160724608  1q23.1-q24.1  0  0  0.158  0  0  0.168  0.607  0  0.085  0  0  0.106  0  0.085  0  0  0  0  0  0  0  0.031  1.176  0.243  0  1.281  1.145  2.925  3.497  0.62  0.684  0.341  0.45  0.465  0  0.084  0.268  0.747  0.233  0  0.134  0.044  0.049  0  0  0.415  0.069  0  0  0  0  0.379  0.58  0.148  0  0  0  0  0  0.959  
 
 LY9  4063  1  160765863  160798045  1q23.3  0.188  0.052  0.41  1.034  0.277  0  0.387  0.201  0.398  0.203  0.318  0.21  0.242  0.197  0.199  0.243  0.083  0.071  1.888  0.338  0  0.435  1.231  0.527  0.102  0  0.802  0.047  0  0.816  0.332  0.182  0.177  0.453  0.208  0  0.148  0  0.057  0.121  0.152  0.845  0.157  0  0  0.159  0.1  0.192  0  0.271  0.157  0.579  0.079  0.132  0.491  0.203  0  0.214  0.182  0.114  
 
 CD244  51744  1  160799949  160832692  1q23.3  0  0  0.082  0  0.028  0  0  0  0.11  0  0  2.031  0  0  0  0  0  0  1.108  1.366  0  0.352  0.328  0  0  0.075  0.051  0.03  0  0.064  0  0  0  0.255  0  0  0  0  0  0  0  0.039  0.055  0  0  0  0  0  0  0  0  0.135  0  0.032  0  0  0  0  0  0  
 
 ITLN1  55600  1  160846329  160854960  1q21.3  0  0  0  0  0  0  0  0  0  0  0  0  0  0  0  0  0  0  0  0  0  0.077  0.413  0  0  0  0.157  0.085  0  0.155  0  0  0  0.236  0  0  0  0  0.058  0  0  0  0.084  0  0  0  0  0  0  0  0  0.352  0  0  0  0  0  0  0  0  
 
 LOC101928372  101928372  1  160902254  160919712  -  0  0  0.078  0  0  0  0.281  0  0.102  0.097  0.169  0  0  0.068  0  0.1  0.057  0.061  0.04  0  0  0.149  0.475  0  0  0  0.065  0.046  0  0.061  0.048  0  0  0.363  0  0  0  0  0.07  0  0  0.099  0.222  0  0  0  0.149  0  0  0  0  0  0  0.06  0.127  0  0.049  0  0  0  
 
 ITLN2  142683  1  160914815  160924589  1q22-q23  0  0  0  0  0  0  0  0.058  0.118  0  0  0  0  0.07  0  0  0  0  0  0  0  0.067  0.45  0  0  0  0.065  0.093  0.112  0.03  0.304  0.129  0  0.2  0  0  0  0  0  0  0  0  0  0  0  0  0.048  0  0  0  0  0.177  0  0  0  0  0  0  0  0  
 
 F11R  50848  1  160965000  160991133  1q21.2-q21.3  2.724  0.349  0.125  0.779  4.068  0.197  0.403  0.248  0.524  0.643  1.894  3.666  3.11  2.625  3.105  3.29  3.103  2.522  1.999  1.875  2.277  1.429  1.882  1.998  0.103  1.819  1.229  1.746  1.687  0.259  1.819  0.49  1.218  0.525  1.261  2.91  1.616  0.996  1.787  1.79  4.028  0.861  2.387  3.1  2.413  3.284  2.07  1.036  2.367  2.47  2.878  2.295  2.274  2.393  2.273  3.06  0.771  1.679  3.02  2.666  
 
 TSTD1  100131187  1  161007421  161008774  1q23.3  5.83  4.883  0  3.691  7.787  0  0.304  0  1.183  0.242  4.283  5.198  4.12  6.49  4.468  5.941  5.721  5.393  7.041  5.761  0  5.672  5.193  6.037  0  4.685  0.171  0  4.533  0.838  1.448  0  0.353  0.414  0.575  3.865  1.489  2.116  6.217  2.672  3.339  1.89  4.173  4.674  3.775  5.256  5.515  0  2.335  0.506  0  3.77  0.257  4.403  5.467  3.368  1.709  0  3.981  2.207  
 
 USF1  7391  1  161009040  161015769  1q22-q23  2.201  0.514  1.676  2.778  3.463  1.602  3.494  3.249  2.503  3.506  3.784  2.445  3.073  3.647  3.161  3.164  2.922  0.336  5.286  1.55  2.405  4.288  2.321  2.681  2.324  2.748  2.75  2.701  2.452  2.014  3.099  3.379  3.776  1.945  1.858  2.811  3.24  3.034  3.093  3.911  2.764  2.672  3.5  3.414  2.224  2.868  0.944  2.126  2.763  3.523  2.954  2.27  2.559  2.351  3.329  3.412  2.67  2.769  2.829  2.868  
 
 ARHGAP30  257106  1  161016731  161039760  1q23.3  0.359  0  0.155  0.168  0.727  0  0.211  0.144  0.162  0.209  0.249  0.185  0.247  0.33  0.398  0.298  0.204  0  4.338  1.77  0.28  3.042  0.805  0.858  0.133  0.411  0.144  1.227  0.344  0.171  0.205  0.305  0.17  0.344  0.183  0.455  0.472  0.442  0.129  0.613  1.132  0.577  1.018  0.401  0.297  0.309  0.068  0.128  0.28  0.128  0.194  0.493  0.277  0.227  0.36  0.173  0.284  0.443  0.192  0.072  
 
 PVRL4  81607  1  161040780  161059385  1q22-q23.2  1.909  0  0.122  0.117  3.636  0.053  0.096  0.084  0.184  0.231  0.334  1.474  1.066  0.386  0.731  1.353  0.371  0.182  0.058  0.141  0  0.393  0.44  0.092  0  0.106  0.185  0.045  0.146  0.185  0.092  0.041  0  0.269  0.036  0  0.031  0.083  0.412  0.116  3.051  0.26  0.094  0.038  0.449  1.017  0.118  0  0.063  0.027  0.051  0.462  0.077  0.312  0.28  0.219  0.057  0  0.051  0.046  
 
 KLHDC9  126823  1  161068150  161070138  1q23.3  2.209  0.904  0  0.582  3.23  0  0.096  0.088  0.368  0.1  0.346  0.556  0.976  0.392  0.805  1.068  0.23  1.275  0.837  0.205  0  0.321  2.339  0.879  0  0.537  0.204  0  0  0.111  0  0.297  0  0.243  0.282  1.768  0.299  0.845  0.122  0  0  1.04  2.255  1.007  0  0.649  1.231  0.356  1.465  1.447  0.938  0.666  0.34  0.204  1.887  1.084  0  0.401  0.831  1.034  
 
 PFDN2  5202  1  161070345  161087866  1q23.3  4.938  6.72  4.857  5.604  5.794  4.836  6.726  5.443  5.054  4.963  5.545  4.717  4.718  6.443  3.725  5.512  6.042  5.963  7.047  6.579  5.663  5.994  4.981  4.571  5.813  5.05  4.971  4.184  4.774  4.053  6.009  5.757  5.152  4.538  5.134  4.889  5.212  4.978  7.246  5.481  5.374  5.377  6.237  5.002  4.306  4.736  6.341  4.274  4.007  6.626  5.65  4.59  4.915  4.554  6.26  5.897  3.991  6.363  4.723  4.698  
 
 NIT1  4817  1  161087861  161095235  1q21-q22  2.351  1.925  2.584  2.961  3.846  1.637  3.375  2.485  2.972  2.832  3.271  2.686  3.127  3.1  2.697  2.52  2.869  1.976  3.974  1.392  2.147  2.283  3.286  2.304  2.67  3.098  3.053  2.581  2.611  2.337  3.531  3.279  3.142  2.202  2.343  2.807  3.013  3.003  2.793  3.973  3.243  3.297  4.511  2.141  1.928  2.766  3.24  1.826  2.273  3.824  2.622  2.255  2.398  2.231  3.387  2.816  2.205  2.686  2.391  2.827  
 
 DEDD  9191  1  161090768  161102478  1q23.3  3.714  2.105  2.395  3.248  3.773  2.164  3.889  3.27  3.096  3.336  3.705  3.155  2.892  3.742  2.735  3.133  2.838  1.824  4.069  2.645  2.123  3.225  2.624  2.299  2.834  2.933  3.185  2.585  2.224  2.805  2.943  2.993  3.147  2.139  2.107  2.898  3.225  3.069  2.774  3.667  2.966  2.666  4.133  2.944  2.226  2.678  3.673  2.447  2.359  3.761  2.95  2.535  2.746  2.129  3.177  3.521  2.65  2.903  3.258  2.626  
 
 UFC1  51506  1  161123533  161128646  1q23.3  4.525  3.697  3.228  3.637  5.23  3.182  5.705  4.798  4.097  4.591  5.009  4.887  4.717  4.724  3.947  4.285  4.303  2.329  5.954  5.929  4.285  5.049  4.781  4.651  4.02  4.444  4.547  3.473  4.13  3.298  4.609  4.62  4.647  3.456  3.558  4.405  4.215  4.444  4.243  4.263  4.386  4.009  6.072  3.89  3.217  4.896  5.33  3.497  3.125  5.408  4.204  3.632  3.707  4.39  5.166  5.212  3.693  4.667  4.587  3.793  
 
 USP21  27005  1  161129253  161135516  1q22  2.655  1.115  1.326  1.901  3.247  1.342  2.408  1.967  1.944  2.273  2.3  1.742  1.498  2.153  1.992  2.154  2.522  0.507  3.786  1.985  2.442  2.737  1.866  1.689  1.815  2.051  1.908  1.612  1.637  1.578  2.552  2.207  2.428  1.182  1.48  2.293  2.034  2.724  2.147  3.274  2.619  2.219  2.927  1.691  1.31  2.197  1.906  1.989  1.861  3.335  2.201  1.998  1.799  1.244  2.59  2.466  1.742  2.464  2.629  2.196  
 
 PPOX  5498  1  161136180  161141010  1q22  1.778  1.383  1.141  1.544  3.309  1.181  2.463  1.472  1.38  2.225  2.431  1.235  1.092  2.239  1.769  1.496  1.824  0.468  3.938  2.057  2.422  2.459  2.121  1.765  1.375  1.885  2.004  1.6  1.728  1.206  2.405  2.182  2.279  1.093  1.458  2.343  2.057  2.054  1.92  1.919  1.854  2.481  2.855  1.463  1.01  1.807  2.816  1.64  1.283  3.629  2.341  1.778  1.438  1.284  2.87  2.69  1.636  1.592  2.933  1.908  
 
 B4GALT3  8703  1  161141099  161147758  1q21-q23  3.372  1.43  2.262  2.872  3.738  2.026  3.455  3.46  3.193  2.804  3.68  2.765  3.164  3.519  2.503  3.165  2.738  0.356  3.936  2.031  3.573  2.353  4.099  3.366  2.856  3.229  2.463  2.726  2.593  2.931  3.48  3.307  3.368  2.179  1.886  2.869  3.264  3.441  2.666  4.3  3.258  3.404  3.769  2.806  2.255  3.146  2.337  2.305  1.899  3.919  3.184  2.402  2.721  2.33  3.164  3.562  2.423  2.499  3.566  2.659  
 
 ADAMTS4  9507  1  161159537  161168845  1q21-q23  0.408  0.043  1.478  2.65  0.559  0.109  0.171  0.109  0.55  2.122  2.458  0.364  0.138  0.269  0.258  0.501  0.416  0.045  0.392  0.385  0.209  0.596  0.615  0.084  0.126  1.488  0.269  0.127  0.22  0.16  0.294  0.187  0.101  0.292  0.122  0.092  0.14  0.073  0.572  0.225  0.164  0.417  0.361  0.054  0.054  0.204  0.841  0.147  0.058  0.159  0.144  0.376  0.211  0.213  0.4  0.319  0.068  0.066  0.099  0.081  
 
 NDUFS2  4720  1  161169104  161184184  1q23  4.755  3.75  3.007  3.964  5.525  2.826  4.664  4.161  3.497  4.069  4.842  4.51  4.128  4.621  3.794  4.204  4.723  3.622  5.576  5.243  3.885  4.017  3.306  3.597  3.776  4.937  4.347  3.804  4.133  4.295  5.076  5.284  5.14  3.674  3.612  4.466  4.131  4.663  4.561  5.578  4.17  3.51  4.929  3.847  3.113  4.131  4.421  3.324  2.972  5.192  3.916  3.792  3.503  3.254  4.863  4.461  3.558  3.594  4.533  4.082  
 
 FCER1G  2207  1  161185086  161189038  1q23  0  0  0  0  0.207  0  0.363  0.225  0  0.269  0.344  0  0  0.931  0.673  0  0.259  0  0.482  4.655  0  0.362  0  0  0  6.796  1.242  0.934  1.879  0.067  0.849  0.514  0  0  0  0  0  0  1.162  0  0  0.676  0  0.309  0  0  0.625  0  0  0  0.282  0.286  0  0  0  0  0  0  0.526  0  
 
 APOA2  336  1  161192082  161193418  1q23.3  0  0  0  0  0.257  0  0  0  0  0  0  0  0  0.242  0  0  0  0.634  0  0  0  0.133  0  0  0  0  0  0  0  0  0  0  0  0  0  0  0  0  0  0  0  0  0  0  0  0  0  0  0  0  0  0  0  0  0  0  0  0  0  0  
 
 TOMM40L  84134  1  161195728  161200536  1q23.3  1.583  1.94  0.976  1.863  2.447  0.884  1.947  1.737  1.474  1.637  2.026  1.153  2.854  1.826  1.754  0.898  1.575  1.614  3.093  2.776  2.204  2.299  1.83  1.88  2.082  2.187  1.591  1.527  2.249  1.82  2.504  2.487  2.716  1.471  0.77  1.477  1.682  1.857  1.504  2.294  1.047  1.894  2.817  1.233  1.239  1.632  2.3  1.498  0.318  3.337  2.092  1.527  1.23  0.87  1.555  1.299  1.096  1.208  1.308  1.492  
 
 NR1I3  9970  1  161199455  161208000  1q23.3  0  0  0  0  0.2  0  0  0  0.237  0.226  0  0  0  0.174  0  0.172  0  0  0.223  0.196  0  0.223  0  0  0  0  0.5  0  0  0.09  0  0  0  0  0  0  0  0  0.313  0  0  0.239  0  0  0  0.226  0  0  0  0.501  0  0.573  0  0.343  0.115  0  0  0  0  0  
 
 PCP4L1  654790  1  161228516  161255240  1q23.3  0  0  0  0  2.815  0  0  0  0.137  0.114  0  0  0  0.152  0  0  0  0  0  0  0  0.081  0.396  0  0  0.184  0.061  0  0  0.085  0  0  0  0.293  0  0  0  0  0.069  0  0  0.267  0.077  0  0  0.621  0  0  0  0  0  0.568  0  0.082  0  0  0  0  0  0  
 
 MPZ  4359  1  161274524  161279762  1q23.3  0.232  0.055  0.212  0  0.076  0  0.296  0.202  0.142  6.348  0.141  0.229  0.15  0.685  0.091  0.076  0.059  0.119  0.744  0.579  1.053  0.496  0.431  0.191  0.095  4.032  0.141  1.948  1.067  0.217  0.895  1.423  0.575  0.619  0  0  0.319  0.145  0.121  0.099  0  2.981  0.135  0.09  0.16  0.075  0.565  0  0  0.149  0.091  0.448  0.066  0.497  0.253  0.086  0.108  0.081  0  0.361  
 
 SDHC  6391  1  161284165  161334535  1q23.3  2.554  2.22  1.88  1.926  3.298  2.346  2.381  2.681  1.871  2.6  2.842  2.939  2.844  3.413  2.014  2.373  1.802  1.677  3.724  1.658  2.795  1.565  1.916  2.249  2.409  3.365  1.457  2.484  2.251  1.377  2.936  2.958  2.7  1.969  2.338  2.878  2.689  2.335  2.063  3.169  2.48  2.827  2.212  2.699  1.932  2.704  1.003  1.644  2.537  3.515  2.706  2.094  2.155  2.3  2.752  3.021  2.245  2.696  2.896  2.693  
 
 CFAP126  257177  1  161334520  161337673  1q23.3  0  0  0.199  0  1.417  0.301  0  0.252  0.27  0.45  0.313  0.378  0.308  0.394  0.657  0.377  0  0  0  0  0  0.199  0  0  0  0.808  0.258  0.5  0.222  0.136  0.248  0.333  0.403  0.363  0.153  0.163  0.397  0.408  0.058  0.952  0.198  0.179  0.053  0.342  0.259  0.256  0.063  0.296  0.344  0  0.364  0.376  0.194  0.474  0.381  0.625  0.417  0.496  0.39  0.176  
 
 FCGR2A  2212  1  161475204  161489360  1q23  0  0.079  0.175  0  0  0.087  0.733  0.108  1.257  1.058  0.131  0.065  0  0.077  0  0  0  0  0  1.899  3.663  0.07  0.767  0.308  0.059  1.545  1.307  2.973  2.002  0.494  3.103  2.023  1.781  1.757  0  0  0  0.297  0.053  0  0  0.342  0.035  0  0  0  0.163  0  0  0.268  0.511  0.124  0  0.055  0.13  0.122  0  0  0  0  
 
 HSPA6  3310  1  161494329  161496687  1q23  0.077  0.152  0.254  0  0.157  0.091  0.42  1.808  0.781  0.423  0.31  0.148  0  0.051  0.093  0.061  1.166  0.179  0.039  0.093  0.718  0.317  0.926  0.132  0.055  1.032  0.283  1.337  0.945  0.385  0.46  0.27  0.166  0.497  0.124  0.227  0.075  0.517  0.166  0.55  0.165  0.738  0.118  0.076  0.129  0.138  0.641  0.279  0  0.155  0.67  0.971  0.248  0.588  0.162  0.158  0.103  0  0.071  0.067  
 
 FCGR3A  2214  1  161511550  161520413  1q23  0  0  0.098  0  0  0  0  0  0.073  0  0.443  0  0  0  0  0  0  0  0  0.074  0  0  0.41  0  0  0  0.076  0  0  0.078  0  0  0  0.393  0  0  0  0  0  0  0  0.078  0.063  0  0  0  0.204  0  0  0  0  0.098  0  0  0.97  0  0  0  0  0  
 
 FCGR2C  9103  1  161551128  161571010  1q23.3  0  0.037  0.078  0  0.026  0.191  0.279  0.037  0.531  0.361  0.038  0  0  0.027  0  0  0  0  0.039  0.724  1.338  0.052  0.576  0.083  0  0.67  0.621  1.581  1.068  0.642  1.362  0.582  0.803  1.076  0  0  0  0.082  0.037  0  0  0.257  0.023  0  0  0.037  0.062  0  0  0.037  0.112  0.112  0.043  0.064  0.058  0  0  0  0  0  
 
 HSPA7  3311  1  161575848  161578341  1q23.3  0  0.077  0.24  0  0.073  0.139  0.105  0.717  0.657  0.245  0.165  0.107  0  0.039  0  0.059  0.627  0.172  0  0.103  0.286  0.268  0.893  0.068  0  0.752  0.262  0.792  0.473  0.376  0.211  0.198  0.082  0.466  0.058  0.075  0  0.254  0.127  0.215  0  0.369  0.112  0  0.118  0.11  0.482  0.128  0.097  0.086  0.297  0.819  0.116  0.469  0.135  0.098  0.053  0  0  0.057  
 
 FCGR3B  2215  1  161592987  161601753  1q23  0  0  0.108  0  0  0  0  0  0.155  0  0  0  0  0.026  0  0  0  0  0  0.11  0  0  0.274  0  0  0  0.053  0  0  0.062  0  0  0  0.49  0  0  0  0  0  0  0  0.038  0.047  0  0  0  0.157  0  0  0  0  0.15  0  0.05  0.037  0  0  0  0  0  
 
 FCGR2B  2213  1  161632904  161648444  1q23  0  0  0  0  0  0  0  0  0.132  0.041  0  0  0  0  0  0  0  0  0  0.061  0.385  0  0.512  0.072  0  0.371  0.049  0.495  0.388  0.332  1.123  0.365  0  0.372  0  0  0  0  0  0  0  0.099  0  0  0  0  0  0  0  0  0  0.117  0  0.036  0  0  0  0  0  0  
 
 RPL31P11  641311  1  161653494  161655042  1q23.3  1.231  0.944  0.438  0  0.708  0.339  0.47  0.129  0.314  0.545  0.43  0.909  0.85  1.12  0.415  1.231  1.674  1.604  1.004  3.472  0.283  2.19  0.855  0.999  1.008  0.487  1.116  0.514  0.79  0.738  0.411  0.293  0.238  0.606  1.406  0.188  0.508  0.216  2.597  0.198  0.175  0.641  1.51  0.408  0.239  0.142  2.506  0.286  0.315  1.811  0.239  2.717  0.99  0.772  1.912  0.76  0.473  0.478  0.935  0.342  
 
 FCRLA  84824  1  161676761  161684142  1q23.3  0  0  0.633  0  0  0.535  1.067  0.125  0.163  1.429  0.08  0  0  0  0  0  0  0  0  0  0  0.049  3.917  1.364  0.496  3.287  2.792  4.794  2.683  3.471  3.073  3.389  1.885  2.45  0  0  0  0.228  0.609  0  0  0.202  0.04  0  0  0  0  0  0  0  0.066  0.294  0  0.117  0  0  0  0  0  0  
 
 FCRLB  127943  1  161691333  161697933  1q23.3  0.284  0.385  0.179  0  0.46  0.414  0.529  0.315  0.283  0.481  0.341  0  0.31  0.41  0  0.391  0.155  0.491  0  0.233  0.287  0.305  0.98  0.36  1.195  1.098  0.311  0.758  0.821  0.27  1.104  1.313  0.899  0.744  0.337  0.305  0.31  0.739  1.213  0.221  0.48  1.014  1.049  0.141  0  0.128  0.961  0.207  0  0.474  0.811  1.279  0.223  0.317  0.929  0.308  0.404  0  0  0.249  
 
 DUSP12  11266  1  161719557  161726954  1q21-q22  3.074  4.852  1.938  2.485  3.663  2.512  3.781  2.995  2.894  2.938  3.674  2.074  2.634  3.905  2.494  3.229  3.583  3.439  4.596  3.307  2.966  3.156  2.264  2.078  2.856  2.409  2.397  2.6  2.546  1.775  3.31  3.462  2.886  1.849  2.518  3.123  3.47  2.401  3.5  3.459  3.29  3.188  3.697  2.846  2.022  2.413  4.651  2.359  1.653  4.762  2.373  2.591  2.85  2.052  3.726  2.763  2.295  3.345  3.741  2.567  
 
 ATF6  22926  1  161736033  161933860  1q22-q23  1.517  2.685  1.591  1.872  2.315  1.215  2.601  1.979  2.2  2.193  2.093  1.505  1.842  2.227  1.528  1.588  1.654  1.459  2.649  2.36  1.906  1.506  1.595  1.66  1.748  2.422  1.213  1.895  1.524  1.25  1.81  1.654  1.926  1.225  0.905  2.079  2.021  1.995  2.355  2.439  1.958  1.474  1.444  1.465  1.096  1.548  1.388  1.122  1.409  2.178  2.103  0.822  1.521  1.256  2.23  2.266  1.813  1.638  1.98  2.386  
 
 OLFML2B  25903  1  161952981  161994255  1q23.3  0  0.384  2.89  1.668  0.058  0.183  2.197  3.521  1.401  5.371  0.565  0.133  0.059  0.457  0.044  0.053  0.108  0.18  0.051  0.092  0  0.471  1.126  1.288  0.06  0.579  0.217  0.069  0.065  0.164  0.233  0.097  0.054  0.263  0.163  0.171  0.162  0.42  0.137  1.194  0.054  0.531  0.664  0.079  0.053  0.051  0.128  0.125  0.472  0.548  0.165  0.676  1.04  0.216  1.208  3.042  0.95  0.694  0.166  0.945  
 
 NOS1AP  9722  1  162039580  162339813  1q23.3  0.955  0.296  0.313  0.169  0.34  0.279  0.285  0.119  0.432  0.421  0.392  0.584  0.528  0.717  0.925  0.217  0.46  0.174  0.031  0.05  0.071  0.326  0.574  0.049  0  0.956  0.234  0.548  0.042  0.66  0.323  0.252  0.789  0.469  0.189  0.756  0.757  0.918  0.31  0.17  0.31  0.535  0.216  0.805  0.168  0.189  1.162  0.28  0.792  0.158  0.426  0.747  0.352  0.315  0.502  0.278  0.066  0.397  0.263  0.302  
 
 C1orf111  284680  1  162343514  162346644  1q23.3  0  0  0.116  0  0  0  0  0  0  0.082  0.134  0  0  0.077  0.239  0  0  0  0  0  0  0.085  0.323  0  0  0.274  0.056  0.098  0  0.058  0  0  0.133  0.405  0  0.165  0.203  0.365  0  0  0  0  0.046  0.325  0  0  0  0.181  0.175  0  0.167  0.5  0  0  0.164  0  0  0  0  0  
 
 C1orf226  400793  1  162348695  162356608  1q23.3  1.824  0.515  0.364  0.474  0.819  0.255  1.201  0.15  1.378  0.833  1.273  1.771  1.786  1.954  1.979  0.579  1.42  0.107  0.054  0.104  0.305  0.443  0.657  0  0  2.139  0.257  1.212  0.088  1.133  0.568  0.656  1.746  0.945  0.312  1.731  1.281  2.139  0.839  0.447  0.87  0.844  0.188  2.254  0.235  0.672  2.604  0.528  1.399  1.149  1.511  0.685  0.459  0.757  0.858  0.746  0.166  0.479  0.45  0.89  
 
 SH2D1B  117157  1  162365055  162381928  1q23.3  0  0.057  0.101  0  0  0  0.629  0  0.126  0.063  0.11  0  0  0.015  0  0  0  0.108  0  0  0.504  0.083  0.298  0  0.4  0.297  0.048  0.214  0  0.025  0  0.34  0  0.313  0  0.044  0.071  0.058  0.062  0  0  0.173  0.024  0  0  0  0.582  0  0  0.041  0.207  0  0.058  0.03  0  0  0  0  0  0  
 
 UHMK1  127933  1  162466963  162499419  1q23.3  2.004  2.785  1.764  2.102  2.841  2.161  2.944  2.582  1.977  2.586  3.454  1.646  2.17  3.237  2.532  2.098  2.144  1.353  2.703  2.217  1.941  2.383  1.409  1.687  2.687  3.675  1.809  2.663  2.163  1.637  2.465  2.758  2.714  2.212  1.461  3.013  3.019  3.047  3.159  2.843  2.325  1.731  2.509  2.068  1.722  2.272  1.518  1.451  2.459  2.043  3.426  2.065  2.594  2.374  2.211  3.05  2.321  2.674  3.112  3.009  
 
 UAP1  6675  1  162531295  162569633  1q23.3  2.424  6.172  4.601  6.379  2.47  3.043  3.013  4.533  2.434  3.615  4.885  1.969  2.457  3.655  1.885  2.249  2.619  3.249  4.216  2.259  4.072  2.105  3.318  3.281  4.156  2.812  2.133  2.284  2.078  1.435  2.495  3.21  2.889  2.047  1.224  2.18  4.345  3.123  5.302  2.903  2.429  2.109  5.087  2.143  1.64  2.987  1.885  4.201  2.186  6.406  4.354  3.371  2.75  2.19  3.341  3.838  3.2  6.302  3.756  4.52  
 
 DDR2  4921  1  162602227  162750247  1q23.3  0  0.051  3.651  3.129  0.079  2.11  2.866  3.925  2.622  4.422  2.606  0.044  0.077  0.146  0.17  0.044  0.061  0.041  0.064  0.111  0.101  0.236  0.261  0.241  0.154  2.53  2.903  2.523  1.988  2.087  1.915  3.569  3.487  2.213  0.061  0.4  0.212  0.143  4.289  0.089  0.062  0.223  0.294  0.18  0.474  0.153  0.253  0.118  0.16  0.105  0.174  0.389  0.199  0.078  0.069  0  0.093  0.14  0.105  0.052  
 
 HSD17B7  51478  1  162760491  162782608  1q23  2.02  1.956  1.58  1.678  1.955  0.299  2.474  2.419  1.509  1.624  2.16  1.884  1.661  2.751  1.829  1.722  2.386  1.264  3.44  2.563  1.507  2.647  2.016  1.296  1.401  2.246  1.37  1.894  1.766  1.292  2.038  1.138  1.435  1.031  1.168  1.593  3.075  1.748  2.036  1.757  2.216  1.672  2.323  1.676  0.898  1.784  1.375  0.952  1.16  2.306  1.734  1.468  1.776  1.005  2.287  1.482  0.845  2.209  1.281  2.768  
 
 CCDC190  339512  1  162824086  162838605  1q23.3  0  3.69  2.929  0  0  0.467  0  0.131  0.093  0.4  0  0  0  0  0  0  0  0  0  0  0  0.029  0.338  0  0  0  0.036  0  0  0.039  0  0  0  0.114  0  0  0.197  0.356  0.124  0  0  0.798  0  0  0  0.047  0  0  0  0  0.2  0.164  0  0  0  0  0.085  0  0  0  
 
 RGS4  5999  1  163038395  163046592  1q23.3  0  1.423  1.09  2.215  0  1.249  0.923  0.388  1.967  0.2  3.399  0  0  0.073  0  0  0  0.076  0  0.059  0  0.169  0.219  0.097  3.573  0  0.087  0.692  0  0.085  0  0.304  0  0.246  0  0  3.305  3.881  3.801  0.152  0  1.044  5.044  0  0  0  0.808  0  0  0  2.849  0.242  0.326  0.172  0.152  0.899  0.722  0.174  0.113  0.698  
 
 RGS5  8490  1  163112088  163291581  1q23.1  0.185  1.233  3.036  0.342  0.467  0.228  0.477  0.435  0.218  0.524  0.674  0.377  0.442  0.571  0.334  0.25  0.32  0.504  0.483  0.262  0.427  0.33  0.633  0.324  0.383  0.421  0.28  0.438  0.558  0.149  0.518  1.378  0.365  0.332  0.176  0.522  0.563  1.094  0.392  0.699  0.496  0.661  2.987  0.462  0.192  0.287  0.092  0.24  0.377  0.225  0.583  0.387  0.414  0.244  0.864  1.477  0.403  0.28  0.44  0.546  
 
 LOC101928404  101928404  1  163131464  163182813  -  0  0.052  0.129  0  0  0  0  0  0.142  0.034  0.06  0  0  0.087  0.074  0  0  0.05  0  0  0  0.021  0.108  0  0.043  0.07  0.032  0.04  0  0.033  0.058  0.047  0  0.282  0  0  0.071  0.099  0.098  0  0  0  0.027  0  0  0  0  0  0  0  0  0  0.037  0.028  0.084  0  0.082  0  0.082  0  
 
 NUF2  83540  1  163291722  163325553  1q23.3  1.845  5.551  1.795  2.253  2.337  2.259  1.767  2.151  0.555  2.867  3.24  1.67  1.469  3.176  1.623  1.672  2.947  5.368  4.95  1.832  2.006  2.188  1.672  1.115  1.956  2.148  0.993  1.255  1.854  0.407  2.499  2.181  1.707  1.373  1.478  2.014  2.197  2.673  2.55  1.729  2.319  2.842  1.794  2.061  1.416  1.861  1.91  1.343  1.972  3.392  2.51  1.477  1.725  1.202  2.989  2.373  2.448  2.056  2.299  2.235  
 
 LOC100422212  100422212  1  163390859  163392981  -  0  0  0.133  0  0  0  0  0  0.056  0  0  0  0  0  0  0  0  0  0  0  0  0  0  0  0  0  0.029  0  0  0.033  0  0  0  0.175  0  0  0  0  0  0  0  0  0.024  0  0  0  0.025  0  0  0  0  0.191  0  0  0  0  0  0  0  0  
 
 PBX1  5087  1  164528596  164821060  1q23  2.894  1.077  1.191  1.887  4.623  0  1.016  0.929  2.635  2.135  2.879  0.213  0.365  1.151  0.994  0.698  0.748  0.168  0.017  0  0.278  0.2  1.15  2.038  0.073  0.021  0.067  0.017  0  0.188  0  0.242  0.025  0.229  2.921  3.848  1.081  2.029  1.084  1.399  2.342  2.952  0.543  0.124  2.011  2.191  0.202  0.039  0.036  0.19  0.984  0.54  0.031  2.659  1.502  2.951  0.189  0.438  0.273  1.64  
 
 LOC100505795  100505795  1  164738352  164743878  -  0.215  0  0.128  0  0.665  0  0.073  0  0.119  0.18  0.207  0  0  0.116  0.089  0  0  0.063  0  0  0  0.063  0.439  0.14  0  0  0.063  0  0  0.045  0  0  0  0.189  0.256  0.323  0  0.192  0.183  0  0.076  0.415  0.021  0  0  0.092  0  0  0  0  0  0.247  0  0.083  0.08  0.31  0  0  0  0  
 
 LMX1A  4009  1  165171103  165325952  1q24.1  0  0.026  0.131  0  0.045  0  0.063  0.033  0.134  0.049  0  0.049  0  0.03  0  0  0  0  0  0.058  0  0.118  0.3  0  0  0  0.149  0.038  0  0.135  0.045  0  0  0.221  0  0  0  0  0.042  0  0  0.128  0.043  0  0.155  0.037  0.038  0  0  0  0  0.378  0.022  0.075  0.031  0  0  0  0  0  
 
 RXRG  6258  1  165370158  165414592  1q22-q23  0  0  0.174  0  0  0  0  0  0  0.094  0  0  0  0.073  0  0  0  0  0  0  0  0.14  0.606  0  0  3.832  4.201  0.364  3.254  2.59  4.763  4.452  4.644  3.433  0  0  0  0  0.095  0  0  0.587  0.127  0  0  0  0.056  0  0  0  0  0.649  0  0.106  0  0  0  0  0  0  
 
 LOC400794  400794  1  165446078  165551341  1q23.3-q24.1  0  0  0.105  0  0.033  0  0.079  0  0.13  0.077  0  0  0  0.087  0  0.057  0.122  0.083  0.08  0.064  0  0.154  0.317  0  0  0.087  0.266  0.062  0  0.173  0  0  0  0.34  0  0  0  0  0  0  0.069  0.316  0.16  0  0  0  0.034  0  0  0.089  0  0.413  0  0.088  0.064  0  0  0  0  0  
 
 LRRC52  440699  1  165513477  165533185  1q24.1  0  0  0  0  0  0  0  0  0.131  0  0  0  0  0.056  0  0  0  0  0  0  0  0.253  0.673  0  0  0.099  0.038  0  0  0.094  0  0  0  0.16  0  0  0  0  0.094  0  0  0  0.062  0  0  0.107  0  0  0  0  0  0.323  0  0.062  0  0  0  0  0  0  
 
 MGST3  4259  1  165600109  165625372  1q23  2.978  4.193  3.568  3.542  5.142  3.714  4.66  3.978  3.848  4.331  3.735  5.45  3.862  4.595  2.74  2.789  4.769  3.815  2.357  5.083  2.77  3.381  1.544  1.109  3.502  4.278  4.744  2.605  3.747  3.4  4.654  3.687  3.251  2.444  3.962  4.554  2.884  4.222  4.313  3.318  3.884  4.246  4.744  3.813  2.7  3.611  4.49  2.508  4.131  4.812  3.615  2.383  2.084  3.117  5.446  4.573  3.703  4.556  4.298  3.673  
 
 ALDH9A1  223  1  165631448  165667900  1q23.1  3.487  5.776  3.008  2.766  4.775  2.452  3.718  3.515  3.627  3.741  3.786  3.247  3.637  4.382  3.579  3.285  4.31  5.299  4.807  3.623  2.689  3.165  2.701  2.457  3.069  4.031  3.337  3.183  2.789  3.543  5.024  4.193  3.525  2.574  2.6  3.603  3.437  3.244  4.188  3.369  3.243  3.162  3.898  3.218  1.814  3.03  3.722  2.699  2.355  4.618  3.573  2.089  2.836  2.12  3.791  2.626  2.947  3.337  3.25  3.426  
 
 LOC440700  440700  1  165667986  165679199  1q24.1  0  0  0.175  0  0  0  0.164  0  0.159  0.047  0  0  0  0.33  0  0  0  0  0.082  0  0  0.155  0.399  0  0  0.168  0.061  0.223  0  0.51  0.56  0.116  0.149  0.32  0  0  0  0  0.091  0  0  0  0.412  0  0  0.094  0.044  0  0  0  0  0  0  0  0.097  0  0  0  0  0  
 
 TMCO1  54499  1  165693527  165738159  1q22-q25  2.367  4.26  2.018  2.334  4.074  1.878  3.482  3.047  2.906  3.008  3.346  3.138  2.463  3.047  1.941  2.465  1.919  4.166  4.068  2.59  2.718  2.518  2.762  2.348  2.527  2.75  1.874  1.983  1.992  2.356  2.769  3.145  2.401  2.038  1.957  2.922  2.51  2.529  3.142  2.219  2.658  2.647  2.592  2.065  2.143  2.592  2.987  1.701  1.886  3.616  2.75  1.625  2.142  2.061  2.737  3.206  2.213  2.263  2.652  2.646  
 
 LOC100147773  100147773  1  165738165  165744685  -  0.181  0.074  0.188  0  0.238  0.129  0  0.13  0.139  0.14  0.171  0.044  0  0.156  0  0.108  0  0.07  0.197  0.05  0.216  0.181  0.43  0.112  0.123  0.201  0.072  0.219  0.058  0.361  0.196  0.228  0.108  0.313  0.159  0.151  0.169  0.161  0.038  0.075  0.295  0.279  0.051  0  0.088  0  0  0  0  0.148  0.138  0.327  0.181  0.103  0.135  0.203  0.094  0  0.091  0.095  
 
 UCK2  7371  1  165796731  165880855  1q23  2.962  3.312  2.676  2.964  3.018  2.274  3.245  3.331  2.408  2.468  2.625  3.326  3.391  3.295  2.835  3.09  3.027  2.392  4.603  3.093  2.994  3.071  2.715  2.974  2.956  2.624  2.862  2.818  2.542  2.909  2.975  3.761  3.073  1.613  2.853  2.678  3.372  2.931  3.161  3.392  2.959  3.333  4.081  3.123  2.295  2.692  3.431  2.783  2.388  4.065  2.582  2.437  2.89  1.922  3.285  3.373  2.503  3.771  3.557  2.857  
 
 FAM78B  149297  1  166039255  166135958  1q24.1  0.485  0.288  0.226  0  0.265  0  0.702  0.086  0.329  0.671  0.349  0  0.362  0.299  0.153  0.074  0.275  0  1.709  0.235  0.16  0.568  0.752  0.248  1.934  0.494  3.625  2.656  2.196  0.695  2.173  1.691  2.16  0.911  0.237  0.657  0  1.73  0.289  0.214  0.089  0.508  0.098  0  0.106  0.109  0.119  0  0.134  0  0.168  0.928  0.227  0.743  0.213  0.235  0.484  0  1.182  0  
 
 FMO9P  116123  1  166573152  166594473  1q24.1  0  0  0  0  0  0  0  0  0  0.04  0  0  0  0  0  0  0  0  0  0  0  0  0  0  0  0  0.041  0  0  0.085  0  0  0  0  0.079  0  0  0  0  0  0.121  0  0  0  0  0.061  0.049  0  0  0  0  0.245  0  0  0  0  0  0  0  0  
 
 POGK  57645  1  166808723  166823709  1q24.1  3.178  3.056  1.723  3.333  3.347  1.971  2.67  3.069  3.059  2.865  3.327  1.773  3.145  2.921  3.023  2.796  2.362  2.318  3.869  3.27  2.458  3.306  2.244  2.364  2.643  3.223  2.334  2.686  2.482  2.459  3.246  2.709  2.719  1.725  2.345  3.094  3.097  2.943  2.739  3.592  3.017  2.234  3.953  3.023  2.269  2.822  2.768  2.395  2.665  3.095  2.941  2.084  2.738  2.538  3.215  3.623  3.005  2.531  3.497  2.919  
 
 TADA1  117143  1  166825748  166845654  1q24.1  1.782  1.948  0.524  1.383  2.237  0.719  1.798  1.478  1.474  1.449  2.097  1.491  1.849  2.346  1.606  1.49  1.65  2.138  3.526  2.054  1.619  1.957  1.253  1.46  0.943  1.386  0.782  0.945  0.905  1.301  1.643  1.553  1.187  0.81  1.123  1.608  1.469  1.523  1.123  1.781  1.416  1.486  2.709  1.088  0.569  1.433  0.561  0.859  0.911  2.091  1.617  1.171  1.344  1.121  1.859  1.952  1.495  1.096  2.12  1.545  
 
 ILDR2  387597  1  166882440  166944561  1q24.1  0  0.935  0.143  0  0.021  0.532  0.312  2.408  0.436  0.816  1.2  0.115  0.078  0.203  0.266  0.028  0.129  0.312  0.81  0.473  0  0.901  0.332  0.297  0.014  0.014  0.168  0.02  0.032  0.105  0.015  0  0  0.147  0.012  0.016  0.838  1.735  0.064  0.037  0.055  0.403  0.035  0.05  0.018  0.045  0.243  0.182  0  0.143  0.021  0.283  1.174  0.139  1.051  0.053  1.16  0.043  0  0.788  
 
 MAEL  84944  1  166944818  166991449  1q24.1  0  1.35  0  0  0.047  0  0.116  0.387  0.085  0.134  0.134  0.154  0  0.069  0  0.081  0  0.149  0.111  0.106  0  0.34  0.258  0  0  0  0.115  0.104  0  0.073  0  0  0  0  0  0  0  0.384  0.062  0  0  0.225  0.148  0  0  0  0.067  0  0  0  0  0.272  0.136  0.042  0.401  0  0.122  0  0  0  
 
 GPA33  10223  1  167022072  167059868  1q24.1  0  0.037  0.089  0  0.02  0  0.051  0.635  0.086  0.037  0  6.193  4.752  0.117  0  0.07  3.068  0  3.27  0.095  0  1.151  0.536  0  0  0  0.084  0.033  0  0.072  0  0  0  0.197  0.193  0  0.088  0  0  0  0  0.047  0.033  0  0.084  0  0.059  0  0.375  0.041  0.1  0.221  0.092  0.036  0.054  0  0  0  0  0  
 
 DUSP27  92235  1  167064086  167098402  1q22-q24  0.1  0  0.164  0  0.045  0.097  0.098  0.467  0.186  0.059  0.066  5.84  0.08  0.053  0.099  0.066  0.045  0.09  0.022  0.064  0  0.214  0.568  0.038  0  0  0.13  0.032  0.034  0.092  0.04  0  0  0.291  0.094  0  0.194  0  0.05  0.039  0  0.278  0.053  0.797  0.047  0.083  0.19  0  0  0  0.033  0.297  0.039  0.095  0.083  0.04  0.027  0  0  0  
 
 LINC01363  101928484  1  167144598  167165042  -  0  0  0.153  0  0  0.109  0  0  0.089  0  0  0  0  0  0  0  0  0.089  0  0  0  0  0.328  0  0  0  0.084  0.041  0  0  0  0  0  0.204  0  0  0  0  0.046  0.104  0  0.42  0  0  0  0  0.022  0  0  0  0  0  0  0  0  0  0  0  0  0  
 
 POU2F1  5451  1  167190065  167396582  1q24.2  1.607  1.297  0.676  1.086  1.392  0.631  1.006  1.416  0.832  1.299  1.261  1.278  1.503  1.631  1.996  1.349  1.402  1.093  2.294  1.509  1.464  1.745  0.93  0.869  1.311  1.269  1.398  1.483  1.033  1.267  1.456  1.43  1.33  0.544  1.16  1.446  1.704  1.824  0.958  2.062  1.653  0.991  1.819  1.947  1.306  1.371  1.45  1.105  1.781  1.427  1.319  0.888  1.476  0.767  1.573  1.214  1.545  0.961  1.361  1.577  
 
 CD247  919  1  167399876  167487847  1q24.2  0  0  0.169  0  0.034  0  0.392  0.056  0.126  0.132  0.072  0  0.112  0.202  0  0.077  0  0.079  6.078  0.718  0  5.306  0.525  0.296  0  0.071  0.082  0.057  0.105  0.229  0  0.13  0  0.174  0.085  0.079  0.077  0  0.115  0.49  0  0.484  0.228  0  0  0.137  0.042  0  0.13  0.226  0.055  0.268  0.085  0.185  0.117  0  0  0  0  0  
 
 CREG1  8804  1  167510250  167523056  1q24  3.442  4.678  0.821  0.708  5.479  1.699  3.168  2.807  2.159  3.866  1.93  3.83  3.802  4.63  3.261  2.941  3.74  5.363  3.853  3.854  3.906  1.783  6.974  5.909  0  3.504  2.266  2.726  2.685  2.155  3.737  3.338  2.506  2.537  2.736  3.429  2.69  3.402  3.622  2.315  4.132  5.172  3.039  3.326  3.499  4.72  3.179  2.003  3.199  4.047  3.114  2.239  2.808  4.453  3.63  3.122  2.583  2.176  2.993  2.777  
 
 RCSD1  92241  1  167599473  167675486  1q24.2  0  0.994  0.139  0  0.019  0.076  0  0.025  0.15  0.048  0  0.032  0  0.025  0  0  0  0.2  4.028  1.966  2.729  2.549  3.014  3.42  0  0  0.062  0  0  0.05  0  0  0  0.249  0  0  0.57  1.684  0.061  0  0  0.191  0.033  0  0  0  0.038  0  0  0.031  0  0.234  0  0.062  0.049  0  0.134  0  0  0  
 
 MPZL1  9019  1  167691186  167761156  1q24.2  2.49  3.666  2.895  3.482  3.118  2.326  4.346  4.13  3.434  3.805  3.398  2.953  3.169  3.14  3.44  2.493  3.013  2.456  3.43  1.871  2.926  3.123  3.417  3.217  2.993  3.104  2.398  2.306  2.632  2.869  3.396  3.746  2.474  1.72  2.903  3.755  3.547  3.81  3.913  2.949  3.452  3.132  3.799  3.877  2.202  2.857  3.76  2.538  2.934  3.647  3.3  2.405  3.448  3.527  4.68  4.668  4.162  2.558  4.375  3.711  
 
 ADCY10  55811  1  167778624  167883464  1q24  0  0.034  0.075  0  0.042  0  0.156  0.028  0.142  0.09  0.022  0.045  0  0.035  0.065  0  0.093  0  0.04  0.033  0.047  0.179  0.286  0.075  0  0.03  0.072  0.033  0.025  0.047  0  0  0  0.119  0.035  0.073  0.046  0.068  0.095  0  0  0.293  0.1  0.089  0.063  0.04  0.288  0  0  0  0.041  0.169  0.059  0.053  0.065  0  0.043  0.058  0.075  0  
 
 MPC2  25874  1  167885912  167906307  1q24  2.382  4.818  1.916  2.791  3.663  2.323  2.713  2.671  2.427  2.91  2.79  3.169  1.987  2.901  1.75  2.006  3.326  5  4.32  2.754  3.035  2.876  2.631  2.053  1.587  2.182  1.284  1.258  1.341  0.994  2.183  2.152  1.254  1.206  2.587  2.397  1.609  2.598  3.803  1.805  2.244  2.847  2.768  4.194  1.031  2.761  2.122  1.324  2.012  3.103  2.315  1.81  1.975  2.587  5.678  3.453  2.344  2.111  4.019  2.321  
 
 DCAF6  55827  1  167905796  168045083  1q24.2  2.168  3.003  1.666  2.026  2.989  1.538  2.546  2.909  1.957  2.775  2.465  1.67  1.955  2.792  2.527  1.761  2.201  2.031  2.743  1.416  2.104  1.635  1.584  1.335  1.794  2.448  1.371  2.287  1.884  1.394  2.003  2.665  1.572  1.054  1.623  2.086  2.319  2.82  2.484  2.914  2.531  1.423  2.447  2.751  1.459  1.755  1.469  2.129  2.254  3.75  2.521  1.813  2.236  2.026  3.759  3.191  2.677  2.165  3.346  2.896  
 
 GPR161  23432  1  168048779  168106905  1q24.2  0.163  1.193  1.115  1.541  0.627  0.891  1.75  1.565  1.369  1.608  1.986  0.069  0.177  1.429  0.936  0.078  0.233  0.266  0.104  0.047  0.13  0.455  0.467  0.027  0.626  1.94  2.496  0.826  2.051  2.846  2.553  1.324  1.602  0.704  0.679  1.418  1.333  0.895  0.431  0.744  1.136  0.673  2.308  0.637  1.272  1.489  0.536  1.483  1.464  1.53  1.654  0.685  1.031  0.466  1.311  1.751  1.715  0.745  0.867  1.369  
 
 TIPRL  261726  1  168148082  168171351  1q23.2  2.586  5.581  2.016  2.784  3.535  2.6  3.671  3.256  2.315  3.178  3.811  2.616  2.446  3.628  2.021  2.239  2.382  4.369  4.714  2.871  2.981  2.633  2.518  2.676  2.67  2.89  2.628  2.465  2.589  2.721  3.6  3.735  2.986  1.791  2.214  2.775  2.535  2.609  3.081  2.796  3.608  2.694  3.035  2.717  2.098  3.217  1.78  2.208  2.153  3.855  2.891  2.299  2.549  2.395  2.673  3.291  2.284  3.352  2.574  2.65  
 
 SFT2D2  375035  1  168195254  168212088  1q24.2  2.098  2.674  1.368  1.53  2.732  2.126  3.213  3.725  1.118  3.222  3.925  3.169  2.634  2.525  2.528  1.974  1.878  2.928  2.629  1.877  2.57  1.944  0.669  2.062  2.225  4.432  2.614  3.225  3.375  2.237  3.113  3.13  3.02  0.825  2.294  3.183  2.855  2.843  1.881  3.15  3.21  0.798  2.365  2.935  2.826  4.21  3.637  1.607  2.863  2.285  3.414  2.085  2.685  2.77  2.558  3.237  3.091  2.529  3.774  3.645  
 
 ANKRD36BP1  84832  1  168214818  168216668  1q24.2  1.946  3.784  1.102  1.087  3.143  1.703  2.66  2.712  1.476  2.867  3.403  2.365  2.138  2.983  1.725  1.804  1.558  2.964  0.981  1.713  2.132  2.066  0.721  1.336  1.799  4.191  2.234  2.377  2.513  2.386  2.486  2.1  2.266  1.099  2  2.517  1.989  2.334  1.987  2.093  2.494  1.721  2.508  2.418  1.927  3.556  1.1  0.937  2.601  0.415  3.061  1.32  2.333  2.565  2.545  2.69  2.505  2.178  3.084  3.111  
 
 TBX19  9095  1  168250277  168283664  1q24.2  0.167  0.387  0.482  1.075  0.236  0.311  0.621  0.406  0.645  0.939  0.875  0.327  0.344  0.771  0.711  0.21  0.308  0.413  1.095  0.477  0.294  0.563  0.514  0.104  0.184  0.987  1.201  0.861  0.807  0.774  1.123  0.663  0.449  0.352  0.252  0.634  0.566  0.827  0.699  0.424  0.713  0.396  0.625  0.268  0.43  1.214  1.206  0.493  0.566  1.087  0.99  0.303  0.289  0.335  0.611  0.638  0.659  0.135  0.915  0.167  
 
 LOC100505918  100505918  1  168369426  168391894  1q23-q24  0  0  0.083  0  0  0  0.041  0  0.104  0  0.041  0.085  0  0.108  0  0  0  0  0  0  0  0  0.334  0  0  0  0.232  0  0  0.096  0  0  0  0.19  0  0  0  0  0.042  0  0  0.181  0.032  0  0  0.077  0.317  0  0  0  0  0.133  0  0  0  0  0  0  0  0  
 
 LOC101928565  101928565  1  168433351  168464882  1q24.2  0  0  0  0  0  0  0  0  0  0  0  0  0  0  0  0  0  0.385  0  0  0  0  0  0  0  0  0.077  0  0  0  0  0  0  0  0  0  0  0  0  0  0  0  0  0  0  0  0  0  0  0  0  0  0  0  0  0  0  0  0  0  
 
 XCL2  6846  1  168510002  168513235  1q24.2  0  0  0  0  0  0  0  0  0  0  0  0  0  0.112  0  0  0  0  0.579  0  0  0  0  0  0  0  0  0  0  0  0  0  0  0  0  0  0  0  0  0  0  0  0.129  0  0  0  0  0  0  0  0  0  0  0  0  0  0  0  0  0  
 
 XCL1  6375  1  168545710  168551315  1q23  0  0  0  0  0  0  0  0.162  0  0.047  0.135  0  0  0.062  0  0  0  1.088  2.195  0  0  0.065  0.3  0  0  0  0.037  0  0  0  0  0  0  0.167  0  0  0  0  0.1  0  0  0  0  0  0  0.067  0  0  0  0  0  0.15  0  0  0  0  0  0  0  0  
 
 DPT  1805  1  168664694  168698442  1q12-q23  0  0  0.26  0  0  0  0.566  1.292  0.142  0.088  0  0  0  0  0  0  0  0  0  0  0  0.026  0.312  0  0  0  0.093  0  0  0.07  0  0  0  0.215  0  0  0  0  0  0  0  0  0.04  0  0  0  0  0  0  0  0  0  0  0.056  0  0  0  0  0  0  
 
 LINC00626  79100  1  168756178  168762126  1q24.2  0  0  0  0  0  0  0.197  1.279  0  0.15  0.212  0  0  0  0  0  0  0  0  0  0  0  0  0  0  0  0.06  0  0  0  0  0  0  0  0  0  0  0  0  0  0  0  0  0  0  0  0  0  0  0  0  0  0  0  0  0  0  0  0  0  
 
 LINC00970  101978719  1  168873142  169056243  1q24.2  0  0.089  0.121  0  0.103  0  0.165  0.308  0.226  0.32  0.238  0  0.325  0.106  0.233  0.218  0.986  0  0.107  0.129  0  0.168  0.285  0  0.111  0.101  0.099  0.141  0  0.058  0  0  0  0.261  0.18  0  0.563  0  0.371  0.178  0.214  0.353  0  0  0  0.149  0.101  0  0  0.144  0.089  0  0.34  0.185  0.183  0  0.208  0.189  0.324  0  
 
 ATP1B1  481  1  169075946  169101960  1q24  2.513  5.616  1.71  1.726  3.264  2.227  4.428  6.201  4.188  4.518  5.484  4.233  4.72  3.957  3.017  4.164  7.16  4.164  0.765  0.479  0.316  1.191  0.356  0.277  3.519  2.863  1.653  3.061  1.526  1.886  1.054  3.325  1.593  1.263  3.963  4.962  5.589  3.658  4.548  2.293  4.594  3.619  2.529  4.208  1.419  3.506  5.798  2.573  3.52  4.133  3.244  2.71  3.682  3.784  3.602  4.016  4.903  3.918  4.152  3.691  
 
 NME7  29922  1  169101767  169337201  1q24  1.551  5.611  1.215  1.612  2.692  1.157  3.283  3.132  1.81  2.219  3.425  2.061  1.945  3.342  1.111  2.036  3.314  5.135  3.87  2.028  1.789  2.254  0.969  0.896  3.101  1.695  0.975  1.844  0.863  0.521  1.408  1.823  1.097  0.949  2.306  2.178  3.326  2.899  3.392  1.484  1.828  1.246  2.875  1.237  1.496  1.252  4.343  1.455  1.419  3.524  2.152  1.171  2.407  2.579  3.008  2.848  2.452  2.978  2.72  2.878  
 
 BLZF1  8548  1  169337193  169365780  1q24  1.239  2.223  0.929  1.776  1.956  0.797  1.399  2.221  0.994  1.869  1.9  0.887  1.35  1.512  0.915  1.725  0.802  1.493  1.799  0.818  1.418  0.641  0.578  0.597  1.787  1.596  0.978  1.662  1.075  0.836  1.308  1.692  1.329  0.794  0.559  1.695  1.534  1.865  1.531  1.383  1.495  0.808  0.871  1.274  1.308  1.398  0.416  0.903  1.04  1.775  1.811  0.69  1.328  1.704  1.293  1.918  2.083  1.413  1.623  1.692  
 
 CCDC181  57821  1  169364107  169429907  1q24  0  0.054  0.126  0  0.045  0.374  0.849  0.107  0.62  0.099  0.865  0  0  0.118  0  0.109  0  0  0.757  0.063  0  0.642  0.234  0  0  0.054  0.062  0.213  0  0.051  0  0.395  0  0.183  0.212  0  0.502  0.713  0.121  0.339  0  0.686  0.026  0  0.178  0  0  0.537  0  1.357  0.049  0.249  0  0.091  1.206  0.455  0.141  0.362  0.079  0.468  
 
 SLC19A2  10560  1  169433148  169455208  1q23.3  2.703  2.034  0.576  0.748  1.35  0.622  0.746  1.767  0.823  1.049  1.145  0.789  1.486  1.762  1.59  1.231  1.39  1.497  1.616  1.671  1.576  0.887  0.688  0.54  1.682  1.864  1.444  1.855  1.997  2.849  3.068  1.92  1.859  1.273  0.557  1.405  1.475  1.371  1.093  0.787  1.038  0.621  0.445  1.093  0.683  1.089  0.371  0.452  0.918  1.031  1.033  0.796  0.994  0.462  1.302  1.126  0.837  0.525  0.924  1.199  
 
 F5  2153  1  169481191  169555769  1q23  0  0  0.085  0.029  0  0  0  0.013  0.087  0.013  0  0.997  0.271  0.05  0  1.736  0.36  0.267  0  0.077  0  0.026  0.238  0.012  0  0.009  0.03  0.006  0.012  0.027  0  0.227  0  0.171  0.388  0.037  0.01  0.029  0.021  0.053  0  0.026  0.021  0  0  0.015  0.013  0  0  0  0.029  0.075  0  0.013  0.011  0  0  0  0  0  
 
 SELP  6403  1  169558087  169599377  1q22-q25  0.104  0  0.107  0  0.058  0  0.069  0  0.094  0.041  0.049  0.033  0  0.032  0.084  0.064  0.045  0.113  0.085  0.07  0.107  0.097  0.238  0.058  0.098  0.075  0.102  0.059  0.045  0.161  0  0  0  0.165  0  0.076  0.137  0  0.038  0  0  0.538  0.062  0.061  0  0  0.057  0  0.084  0  0.107  0.145  0.057  0.064  0.057  0.081  0  0  0.102  0.053  
 
 SELL  6402  1  169659805  169680843  1q23-q25  0.32  0.776  0.316  0.373  0.225  1.284  0.662  1.305  0.434  0.165  0.435  0.273  0.295  0.574  0.335  0.183  0.196  1.005  2.405  0.245  0.673  2.379  0.45  0.459  0.399  0.334  0.365  0.388  0.222  0.311  1.451  1.733  1.179  0.478  0.214  0.324  0.323  1.123  0.111  0.248  1.372  1.107  0.256  0.492  1.565  1.487  0.489  1.073  0.491  0.426  0.368  0.273  0.297  0.211  0.224  0.306  0.218  1.508  0.366  0.287  
 
 SELE  6401  1  169691780  169703220  1q22-q25  0  0  0.072  0  0.029  0  0  0  0.073  0.016  0.02  0  0  0.028  0  0  0  0  0  0.039  0  0.037  0.182  0  0  0  0.047  0  0  0.035  0  0  0  0.161  0  0  0  0  0.015  0  0  0.033  0.019  0  0  0  0.016  0  0  0  0  0.067  0  0.027  0  0  0  0  0  0  
 
 METTL18  92342  1  169761669  169764061  1q24.2  1.533  1.4  0.712  1.237  1.651  0.611  0.954  1.054  1.222  1.466  1.549  1.02  0.968  1.885  0.677  1.393  1.208  0.968  2.969  1.058  0.938  1.117  1.521  0.853  0.687  1.244  0.698  0.386  0.755  0.613  1.257  1.179  0.597  0.927  0.793  0.717  0.79  0.824  0.742  0.745  1.829  0.972  0.933  0.866  0.636  1.004  1.278  0.18  0.596  1.591  1.744  0.555  0.66  0.543  1.162  0.815  1.063  0.827  0.541  0.945  
 
 C1orf112  55732  1  169764549  169822229  1q24.2  1.369  2.694  0.712  1.039  1.576  0.726  1.793  1.667  0.38  1.739  1.941  1.206  0.867  1.67  1.466  1.344  1.327  1.826  3.425  2.223  1.775  1.841  0.577  0.938  1.132  1.568  1.225  1.431  1.404  1.383  2.114  1.573  1.76  0.621  0.845  1.378  1.679  1.676  1.339  1.592  1.593  0.942  2.19  0.865  1.218  1.523  1.602  0.847  1.07  2.379  0.953  0.646  1.227  0.818  1.237  1.077  1.767  0.399  1.54  1.253  
 
 SCYL3  57147  1  169822214  169863100  1q24.2  1.219  1.222  0.463  0.801  1.534  0.505  0.832  0.851  0.807  1.19  1.024  0.818  1.245  1.239  0.929  1.411  1.312  1.104  1.792  1.056  0.742  1.21  0.687  0.572  0.612  1.141  0.468  0.979  0.796  0.625  0.805  1.131  0.82  0.387  0.592  0.997  1.206  1.009  0.708  0.991  1.36  0.536  0.567  0.762  0.439  0.754  1.069  0.495  0.736  1.181  0.446  0.588  0.628  0.583  1.07  0.621  0.959  0.638  0.748  0.704  
 
 KIFAP3  22920  1  169890469  170043879  1q24.2  1.739  1.987  1.346  1.442  2.157  1.164  2.551  2.29  1.686  2.612  2.65  1.642  1.397  1.764  0.852  1.342  2.761  2.151  3.244  1.009  1.02  2.404  1.072  0.632  1.203  2.354  2.177  1.904  1.433  0.609  1.781  2.641  1.498  1.079  0.945  1.546  1.167  1.619  1.09  2.716  1.553  1.069  2.45  1.541  0.742  1.184  1.187  0.806  1.104  2.296  1.194  0.911  0.946  1.887  1.578  1.548  1.728  1.469  1.55  1.562  
 
 METTL11B  149281  1  170115187  170136923  1q24.2  0  0  0  0  0  0  0  0  0.16  0  0  0  0  0  0  0  0  0  0  0  0  0.079  0.45  0  0  0  0.064  0.068  0  0.048  0  0.191  0  0.195  0  0  0  0  0  0  0  0  0  0  0  0  0  0  0  0  0  0.201  0  0.076  0  0  0  0  0  0  
 
 LINC01142  284688  1  170240545  170253349  1q24.2  0  0  0.092  0  0  0  0  0  0.193  0.061  0  0  0  0  0  0  0  0  0  0  0  0.052  0.244  0  0  0  0.022  0.109  0  0.043  0  0  0  0.131  0  0  0  0  0  0  0  0  0  0  0  0  0  0  0  0  0  0  0  0  0  0  0  0  0  0  
 
 LOC101928650  101928650  1  170430457  170501750  1q24.2  0  0  0  0  0  0  0  0  0  0  0  0  0  0  0  0  0  0  0.137  0  0  0.116  0  0  0  0  0.084  1.298  0.395  0.091  0.389  0.725  0.379  0.301  0  0  0  0  0  0  0  0  0  0  0  0  0  0  0  0  0  0  0  0  0  0  0  0  0  0  
 
 GORAB  92344  1  170501262  170522974  1q24.2  0.993  2.111  0.92  1.458  1.079  0.796  0.892  1.216  1.219  1.584  1.399  0.522  0.652  1.337  0.769  0.853  1.048  1.483  2.068  0.758  0.473  1.05  0.89  0.978  0.863  1.127  0.62  0.861  0.813  0.176  0.946  1.101  0.634  0.832  0.359  0.961  0.767  0.808  1.264  1.075  1.249  0.727  0.513  0.738  0.34  0.656  0.487  0.315  0.662  1.507  1.279  0.523  0.637  0.93  1.227  1.128  1.199  0.613  1.364  0.742  
 
 PRRX1  5396  1  170633312  170708541  1q24  0  0.027  2.762  2.755  0.016  1.987  2.992  0.623  1.747  4.038  2.629  0.026  0  0.042  0  0  0  0.403  0  0  0  0.158  0.27  0.032  1.316  0.383  4.168  0.046  0  0.088  0.029  4.188  2.737  2.316  0.035  0.032  0.086  0  0.03  3.365  0.233  0.749  0.025  0  0  0  0.04  0.087  0  0.541  0.021  0.297  0  0.034  0.031  0  0  0  0  0  
 
 MROH9  80133  1  170904611  171033906  1q24.3  0  0  0.055  0  0  0  0  0  0.041  0.044  0  0  0  0.044  0  0  0  0  0  0  0  0.02  0.117  0  0  0.046  0.076  0  0  0.071  0  0  0  0.081  0  0  0  0  0.026  0  0  0.033  0.023  0  0  0  0  0  0  0  0  0.138  0  0.035  0  0  0  0  0  0  
 
 FMO3  2328  1  171060017  171086959  1q24.3  0  0  0.165  0  0.111  0  0.087  0  0  0.035  0  0  0  0.03  0  0  0  0.564  0  0  0  0  0.265  0  0  0  0.027  0.235  0  0.022  0  0  0  0.1  0  0  0  0  0.038  0  0  0.04  0.035  0  0  0.085  0  0  0  0  0  0.09  0  0.04  0  0  0  0  0  0  
 
 FMO6P  388714  1  171106878  171130702  1q24.3  0.111  0  0.1  0  0  0.053  0  0.045  0.056  0  0  0  0  0.043  0  0  0  0  0  0.053  0  0.066  0.238  0  0  0  0.036  0  0  0.028  0  0.063  0  0.211  0  0  0  0  0  0  0  0.04  0  0  0  0  0  0  0  0  0  0.19  0  0  0  0  0  0  0  0  
 
 FMO2  2327  1  171154346  171181822  1q24.3  0  0.02  0.061  0  0  0  0  0.027  0.049  0  0  0  0  0.034  0  0  0  0.033  0  0  0  0.042  0.119  0  0  0  0.015  0  0  0.024  0  0  0  0.119  0  0  0  0  0.012  0  0  0.175  0.014  0  0  0.029  0.01  0  0  0  0  0.084  0  0.016  0  0  0  0  0  0  
 
 FMO1  2326  1  171217609  171255117  1q24.3  0  0  0.096  0  0  0  0  0.085  0.085  1.473  0  0  0  0.048  0  0  0  0.08  0  0  0  0.032  0.324  0  0  0  0.025  0  0  0.032  0  0  0  0.257  0  0  0  0  0.033  0  0  0.048  0.034  0  0  0  0.032  0  0  0  0  0.171  0  0  0  0  0  0  0  0  
 
 FMO4  2329  1  171283321  171311223  1q24.3  0.613  0  0.23  0.451  0.854  0.061  0.397  0.636  0.281  0.796  0.651  0.064  0.199  0.617  0.084  0.192  0.213  0.244  0.495  0.216  0  0.394  0.238  0.081  0.218  0.725  0.275  0.398  0.536  0.538  0.903  0.642  0.151  0.224  0.156  0.133  0.072  0.419  0.192  0.518  0.335  0.08  0.02  0.051  0.125  0.195  0.104  0  0.176  0.284  0.351  0.189  0.221  0.371  0.506  0.125  0.384  0  0.295  0.637  
 
 TOP1P1  7151  1  171308034  171310463  1q23-q24  1.098  0.709  0.497  0.795  0.623  0.565  0.699  0.329  1.048  0.619  0.5  0.699  0.661  1.106  0.681  1.183  1.179  1.113  1.495  1.229  0.416  2.052  0.812  0.471  0.603  0.431  0.526  0.413  0.51  0.642  0.707  0.733  0.628  0.826  0.479  0.4  0.579  0.43  1.779  0.712  0.645  0.918  1.423  0.536  0.368  0.525  1.447  0.366  0.15  0.851  0.59  0.311  0.649  0.341  1.005  0.725  0.324  0.345  0.345  0.533  
 
 PRRC2C  23215  1  171454665  171562650  1q23.3  4.32  4.815  2.87  4.136  4.566  3.207  4.39  4.699  3.104  4.315  5.035  3.249  4.448  4.664  4.618  4.183  3.9  3.686  3.901  4.338  4.493  4.783  2.82  3.568  4.015  4.62  3.787  4.327  4.116  3.94  4.511  4.82  4.185  2.123  3.627  4.522  4.974  4.401  4.142  4.922  4.583  3.605  5.1  4.137  3.565  4.122  3.778  3.822  3.859  3.473  5.738  2.549  4.438  3.692  4.959  4.56  4.517  3.806  4.738  4.492  
 
 MYOC  4653  1  171604556  171621773  1q23-q24  0  0.047  0.071  0  0.071  0  0.053  0  0.175  0.051  0.041  0.086  0  0.043  0.082  0.069  0.056  0  0.07  0.102  0  0.111  0.385  0.097  0.151  0.142  0.08  0.073  0  0.041  0  0  0  0.234  0  0.102  0  0  0.073  0.117  0  0.292  0.047  0.073  0  0.05  0.031  0  0.075  0.101  0.115  0.297  0.085  0.07  0.063  0  0.046  0  0.172  0.078  
 
 VAMP4  8674  1  171669295  171711379  1q24-q25  0.761  1.617  0.82  0.848  1.162  0.445  2.051  0.893  1.035  1.163  1.106  0.682  0.799  1.1  0.402  0.505  0.965  0.857  2.486  0.549  0.902  1.089  1.035  0.511  0.747  0.995  0.765  0.489  0.537  0.315  1.005  0.771  0.538  0.453  0.453  0.93  0.864  2.106  0.831  0.867  0.695  0.865  1.216  0.46  0.278  0.63  0.733  0.365  0.388  1.232  1.92  0.754  0.414  0.559  0.816  1.102  1.109  0.589  1.025  0.623  
 
 METTL13  51603  1  171750760  171766856  1q24-q25.3  3.018  2.682  1.797  3.197  3.133  1.258  3.498  2.371  2.058  2.666  2.894  2.273  3.23  2.65  2.743  2.616  2.28  2.529  4.142  3.132  2.779  2.959  2.561  2.505  2.494  3.026  2.786  3.268  2.779  2.218  3.07  3.265  3.165  2.064  2.021  2.729  3.308  2.65  2.483  3.167  2.37  2.45  3.585  2.35  2.25  2.755  2.754  1.806  1.615  3.248  3.856  2.032  2.807  2.113  3.051  2.879  2.489  1.839  3.159  2.651  
 
 DNM3  26052  1  171810617  172381857  1q24.3  0.094  0.325  0.103  0.079  0.037  0.237  0.348  0.925  0.431  1.362  1.781  0.027  0.616  0.596  0.083  0.317  0.045  0.506  0.404  0.095  0  0.231  0.291  0.071  0.087  0.03  0.406  0.302  0.18  0.093  0.299  0.252  0.77  0.281  0.139  1.178  0.056  0.425  0.116  0.088  0.343  0.468  1.97  1.311  0.646  0.718  0.126  0.047  0.698  0.123  0.112  0.508  0.088  0.205  0.094  0.506  0.525  0.236  0.228  0.072  
 
 DNM3-IT1  100874284  1  171833331  171833827  -  0  0.364  0  0  0  0  0  0  0  0.359  0  0  0  0  0  0  0  0  0  0  0  0  0  0  0  0  0  0  0  0.801  0  0  0  0  0  0  0  0  0  0  0  0  0  0  0  0  0  0  0  0  0  0  0  0  0  0  0  0  0  0  
 
 DNM3OS  100628315  1  172106018  172113975  1q24.3  0.045  0.119  0.314  0  0.024  0.035  0.1  1.149  0.081  0.441  0.163  0.026  0.167  0.17  0.023  0.02  0  0.311  0.118  0.046  0.042  0.091  0.184  0  0  0.016  0.257  0.051  0.02  0.041  0.043  0.076  0.17  0.121  0.042  0.098  0  0.116  0.047  0  0.061  0.309  0.879  0.481  0.109  0.134  0.055  0  0.097  0.043  0.046  0.167  0.011  0.049  0.012  0.058  0.173  0.085  0.055  0.021  
 
 MIR214  406996  1  172107937  172108047  1q24.3  0  0  0  0  0  0  0  4.177  0  0  0  0  0  0  0  0  0  0  0  0  0  0  0  0  0  0  0  0  0  0  0  0  0  0  0  0  0  0  0  0  0  0  0  0  0  0  0  0  0  0  0  0  0  0  0  0  0  0  0  0  
 
 C1orf105  92346  1  172389827  172437969  1q24.3  0  0  0  0  0  0  0  0  0  0  0  0  0  0  0  0  0  0.813  0  0  0  0  0.372  0  0  0  0.062  0  0  0  0  0  0  0  0  0  0  0  0  0  0  0  0  0  0  0  0.049  0  0  0  0.159  0  0  0.086  0  0  0  0  0  0  
 
 PIGC  5279  1  172410596  172413230  1q23-q25  2.77  3.749  1.905  2.557  3.125  1.635  3.132  2.875  2.955  3.128  3.099  2.318  2.49  2.405  2.259  3.316  2.517  3.096  4.237  2.257  2.671  2.151  3.16  2.145  2.486  3.184  2.997  2.605  2.388  2.44  3.15  3.357  2.381  2.079  2.061  2.882  2.876  2.797  2.208  2.627  3.116  2.586  3.814  2.964  1.995  2.038  2.848  1.664  2.054  4.253  3.667  1.933  2.213  1.527  3.111  3.129  2.215  2.055  3.163  2.649  
 
 SUCO  51430  1  172501488  172580975  1q24  1.741  2.909  0.856  1.124  1.955  1.327  1.609  2.055  2.168  1.753  2.62  1.082  1.481  2.052  1.341  2.015  1.517  1.425  2.27  0.831  0.933  0.983  0.712  0.567  1.406  2.17  0.912  1.652  1.844  0.582  1.595  1.712  1.078  0.721  0.724  1.881  1.734  1.627  1.641  1.616  2.118  1.171  1.138  1.743  0.918  1.399  1.061  0.812  0.822  2.015  3.181  1.102  1.326  1.072  1.716  2.114  1.556  0.798  1.869  1.719  
 
 FASLG  356  1  172628147  172636012  1q23  0  0.116  0.113  0  0  0  0  0.039  0.102  0  0  0.096  0  0.029  0  0  0  1.936  0.062  0.08  0  0.08  0.295  0  0  0  0.059  0.048  0  0.074  0  0  0  0.242  0  0  0  0  0.029  0  0  0.041  0.044  0  0  0.093  0.478  0  0  0  0.148  0.297  0  0.056  0  0  0  0  0  0  
 
 TNFSF18  8995  1  173010359  173020103  1q23  0  0.147  0  0  0  0  0  0  0  0  0  0  0  0.658  0  0  0  0  0  0  0  0  0  0  0  0  0  0  0  0  0  0  0  0  0  0  0  0  0.587  0.324  0  0  0  0  0  0  0  0  0  0  0  0.413  0  0  0  0  0  0  0  0  
 
 TNFSF4  7292  1  173152869  173176452  1q25  0  0.109  0.179  0  0.018  0.491  1.301  0.126  0.15  0.145  1.097  0.476  0  0.552  0  0  0  0.031  0.134  0.047  0  0.576  0.332  0.22  0.091  0.184  0.044  0.151  0.093  0.023  0.224  0.047  0  0.188  0.057  0.265  0.082  0.337  0.4  0.089  0.174  0.101  0.026  0  0  0.029  0.044  0  0  0.026  0.603  0.129  0.028  0.079  0.027  0  0.118  0  0  0  
 
 LOC100506023  100506023  1  173204198  173446294  1q23-q25  0.152  0.071  0.175  0.182  0.095  0.105  0.184  0.133  0.138  0.138  0.14  0.109  0.168  0.186  0.055  0.077  0.103  0  0.172  0.174  0.099  0.111  0.314  0.071  0.104  0.163  0.218  0.113  0.096  0.089  0.146  0.123  0.099  0.286  0.086  0.205  0.163  0.134  0.197  0.1  0.17  0.286  0.229  0.087  0.033  0.113  0.096  0.045  0.042  0.119  0.424  0.167  0.137  0.14  0.148  0.1  0.113  0.048  0.12  0.054  
 
 LOC101928673  101928673  1  173386927  173430501  1q25.1  0.4  0.214  0.338  0.416  0.428  0.497  0.432  0.404  0.369  0.557  0.398  0.135  0.265  0.376  0.426  0.192  0.075  0.192  0.571  0.145  0.566  0.172  0.659  0.095  0.168  0.614  0.127  0.257  0.139  0.324  0.794  0.706  0.492  0.312  0.091  0.309  0.466  0.677  0.183  0.173  0.65  1.438  0.968  0.066  0.413  0.843  0.588  0.332  0.346  0.334  1.024  0.2  0.212  0.116  0.337  0.162  0.496  0.666  0.28  0.093  
 
 PRDX6  9588  1  173446485  173457946  1q25.1  5.604  6.993  5.054  5.376  6.224  4.722  6.679  5.665  5.342  6.071  6.635  4.841  5.408  6.839  5.017  5.643  6.012  6.617  6.752  6.139  6.133  5  5.56  5.112  5.49  5.266  4.593  4.805  4.797  3.771  5.711  6.745  4.876  4.407  4.93  5.584  5.501  5.751  7.364  5.943  6.241  6.363  6.008  6.041  4.957  6.196  5.484  4.787  5.236  7.412  7.526  4.043  5.159  5.99  5.856  6.179  5.588  5.926  5.968  5.905  
 
 SLC9C2  284525  1  173469603  173572233  1q25.1  0  0  0  0  0  0  0  0  0  0  0  0  0  0.013  0  0  0  0  0  0  0  0.016  0  0  0  0  0.045  0.034  0  0.035  0  0.03  0  0.065  0  0  0  0  0.015  0  0  0.069  0.015  0  0  0.032  0.048  0  0  0  0.019  0  0  0  0  0  0  0  0  0  
 
 ANKRD45  339416  1  173577474  173639001  1q25.1  0  0  0.091  0.14  0  0.042  0.053  0.023  0.109  0.029  0.409  0  0  0.249  0  0  0  0  0  0  0  0.05  0.301  0  0  0.088  0.056  0.19  0  0.039  0.068  0.895  0  0.237  0  0  0  0.189  0.039  0.165  0.048  0.219  0  0  0.218  0.178  0.085  0.066  0  0.041  0.085  0.097  0  0.09  0.066  0  0  0  0  0.053  
 
 LOC730159  730159  1  173604660  173606272  1q25.1  0  0  0  0  0  0  0  0  0  0  0  0  0  0  0  0  0  0  0  0  0  0  0  0  0  0  0  0  0  0  0  0  0  0  0  0  0  0  0  0  0  0  0  0  0  0  0.083  0  0  0  0  0  0  0  0  0  0  0  0  0  
 
 KLHL20  27252  1  173684079  173755840  1q25.1  1.471  2.44  0.95  1.198  1.68  1.22  2.297  1.954  1.551  2.275  2.281  0.871  1.431  1.942  1.338  1.087  0.995  2.163  2.338  1.335  1.328  1.252  1.111  0.892  1.514  2.215  1.53  1.561  1.128  1.237  1.987  2.66  1.761  1.067  0.929  1.781  1.701  1.772  2.114  1.384  1.565  1.218  2.135  1.05  1.179  1.398  1.46  1.074  0.839  2.332  3.486  0.767  1.243  1.066  1.039  1.444  1.344  1.147  1.32  1.523  
 
 CENPL  91687  1  173768687  173793777  1q25.1  1.414  2.035  1.422  1.967  1.91  1.466  2.424  2.282  1.03  2.617  2.845  1.654  1.511  1.269  1.482  1.508  1.908  0.746  3.267  1.619  1.896  1.928  1.566  1.018  1.783  2.06  1.391  1.097  1.254  1.116  1.938  2.279  1.552  1.061  1.008  1.742  2.118  1.958  2.117  2.236  1.745  1.062  2.556  1.673  1.181  2.081  1.644  1.14  1.9  2.42  3.781  1.678  1.895  1.579  1.696  1.745  2.213  1.429  2.359  1.389  
 
 DARS2  55157  1  173793796  173827682  1q25.1  3.891  3.045  1.293  2.229  3.363  1.375  2.227  2.619  1.101  2.022  3.187  2.564  2.458  3.123  1.733  2.179  2.531  3.709  3.34  3.095  2.709  1.872  2.049  2.108  2.727  3.413  2.146  2.94  2.562  1.929  2.864  3.521  2.346  1.671  2.492  2.835  2.535  2.068  2.099  2.998  3.264  2.032  3.033  2.272  1.67  2.453  2.333  2.383  1.696  4.087  2.858  1.854  2.37  1.871  3.068  2.768  2.521  1.847  2.756  2.747  
 
 GAS5-AS1  100506046  1  173832385  173833079  -  0.926  0.886  0  0  1.055  0.896  0.413  0.723  0.189  0.728  1.032  0.41  0.65  0.772  0.85  0.481  0.514  0.334  0.764  0.471  0.697  0.581  0  0.449  0  1.001  0.303  0.641  0.418  0.454  0.73  0.288  0.41  0.5  0.423  0.387  0.561  0.626  0.274  0.542  0.537  0.302  0.327  0.729  0.384  0.335  0.096  0.576  0.774  0.254  0.247  0.31  0.421  0.198  0.751  0.918  0.927  1.061  1.01  0.385  
 
 GAS5  60674  1  173833038  173837125  1q25.1  6.323  8.135  4.097  4.643  6.519  5.151  6.373  4.91  4.563  4.84  6.503  5.677  5.566  7.348  4.364  6.006  7.68  9.755  7.827  8.804  6.537  6.756  5.328  4.866  5.805  5.108  5.379  4.172  4.447  4.621  5.781  5.738  4.862  3.061  5.659  4.248  6.337  5.817  8.314  4.255  4.955  5.355  6.209  6.123  4.842  4.805  6.875  5.079  4.317  8.573  3.54  5.119  4.582  4.307  7.712  6.352  4.652  6.647  7.602  4.832  
 
 ZBTB37  84614  1  173837492  173855774  1q25.1  1.321  0.583  0.342  0.435  1.109  0.784  0.552  1.297  0.49  0.915  1.079  0.429  0.803  0.925  1.186  0.738  0.435  0.542  0.533  0.533  1.24  0.611  0.486  0.408  0.721  1.341  0.461  1.222  0.892  0.691  1.215  1.163  0.866  0.369  0.509  1.074  1.256  0.799  0.439  1.648  1.364  0.679  1.368  0.849  0.52  0.585  0.107  0.644  0.874  0.219  0.4  0.871  1.053  0.644  0.916  1.16  1.059  0.695  0.808  0.842  
 
 SERPINC1  462  1  173872941  173886516  1q25.1  0  0  0.083  0  0.065  0  0.094  0  0.137  0.035  0.056  0  0  0.12  0.089  0  0  0  0.27  0  0  0.16  0.366  0  0  0.069  0.044  0.049  0  0.052  0  0  0  0  0  0.081  0  0  0.089  0  0  0.093  0.521  0  0  0.068  0.047  0  0.161  0.104  0  0.193  0.084  0.044  0  0  0  0  0  0  
 
 RC3H1  149041  1  173900221  173962210  1q25.1  1.7  1.182  0.815  0.799  1.493  0.641  1.317  1.643  0.888  1.407  1.641  1.245  1.467  1.657  1.685  1.293  1.132  0.739  1.216  1.247  1.221  1.256  0.83  1.098  1.297  1.69  0.93  1.274  1.171  0.881  1.309  1.491  1.119  0.508  0.995  1.823  1.742  1.562  1.312  1.671  1.364  0.748  1.442  1.691  1.33  1.435  0.693  1.101  1.323  1.145  0.902  0.908  1.603  1.189  1.186  1.325  1.564  0.869  1.954  1.913  
 
 LOC102724601  102724601  1  174090775  174128425  1q25.1  0  0  0  0  0.169  0  0  0  0  0  0  0  0  0.156  0  0  0  0  0  0  0  0  0  0  0  0  0.178  0  0  0.115  0  0  0  0  0  0  0  0  0.166  0  0  0  0  0  0  0  0  0  0  0  0  0  0  0  0  0  0  0  0  0  
 
 RABGAP1L  9910  1  174128551  174964445  1q24  1.441  2.695  0.932  1.046  1.844  0.848  1.124  2.148  1.077  1.549  1.309  2.019  2.387  2.202  1.318  3.549  2.535  4.088  2.759  1.684  2.145  1.433  2.177  3.056  1.298  2.101  1.128  1.659  0.984  1.197  1.524  1.849  1.289  1.186  0.805  1.913  1.094  1.896  2.052  1.662  3.756  0.784  1.911  2.142  2.622  2.367  1.172  0.813  2.053  1.353  1.16  1.366  1.232  0.734  1.261  1.063  1.558  1.222  1.699  1.382  
 
 GPR52  9293  1  174417211  174418683  1q24  0.162  0  0.16  0  0.243  0  0  0.185  0.098  0.131  0.145  0.135  0.202  0.292  0.294  0.186  0  0  0  0.085  0.273  0.162  0.314  0  0  0.326  0.449  0.178  0.109  0.052  0.111  0  0.159  0.195  0  0.244  0.183  0.273  0.119  0  0.122  0.279  0.498  0.178  0.251  0.357  0.256  0.199  0.62  0  0  0.352  0.183  0.048  0.108  0.163  0.14  0  0.103  0  
 
 LOC101928696  101928696  1  174904083  174923398  1q25.1  0  0  0  0  0  0  0  0  0  0  0  0  0  0  0  0.176  0  0  0  0  0  0  0  0  0  0  0.069  0  0  0  0  0  0  0  0  0  0  0  0  0  0  0  0  0  0  0  0  0  0  0  0  0  0  0  0  0  0  0  0  0  
 
 CACYBP  27101  1  174968570  174981163  1q24-q25  3.801  6.611  2.753  3.324  4.213  3.056  4.007  3.858  3.135  3.537  4.281  3.621  3.739  5.1  2.892  3.61  4.165  6.703  5.673  4.352  4.421  3.642  3.517  3.896  3.757  3.591  2.634  2.877  2.645  2.075  4.163  3.978  3.467  2.656  3.429  3.484  3.98  3.804  4.351  3.461  3.762  4.021  4.467  4.059  2.763  3.903  3.848  3.214  3.012  5.22  2.42  2.516  3.678  3.231  4.323  4.75  3.604  3.914  4.384  3.849  
 
 MRPS14  63931  1  174982093  174992591  1q25.1  3.021  4.355  1.721  1.946  3.854  1.92  2.794  2.393  2.256  2.752  2.624  2.428  2.321  3.235  2.049  2.436  2.512  5.021  4.039  3.997  2.57  3.005  2.845  2.397  2.269  2.284  1.781  1.261  1.988  1.291  2.832  2.785  1.633  1.198  1.957  1.95  2.036  1.99  3.352  1.859  2.515  2.608  2.298  2.279  1.462  2.307  3.519  1.776  1.733  3.713  1.144  1.454  1.76  1.735  3.092  2.585  1.462  2.581  2.054  2.174  
 
 TNN  63923  1  175036993  175117202  1q23-q24  0  0.088  0.119  0  0.017  0.022  0  0  0.148  0.024  0.045  0.03  0  0.032  0  0.026  0.024  0.072  0.023  0.041  0  0.082  0.341  0.08  0  0.025  0.087  0.073  0.05  0.062  0  0  0  0.134  0  0  0  0  0.044  0  0  0.043  0.035  0  0.284  0.031  0.434  0  0  0.022  0.022  0.329  0.021  0.051  0.032  0.036  0.038  0  0  0  
 
 KIAA0040  9674  1  175126122  175162229  1q24-q25  1.827  0.037  0.154  0  2.958  0.226  0.12  0.355  1.006  0.227  0.272  0.838  1.249  2.599  1.516  1.342  2.445  0.765  0.161  0.388  1.048  1.189  2.41  2.559  0.08  0.424  1.317  0.733  0.646  1.398  0.539  0.907  0.861  0.615  0.274  0.547  0.069  0.056  0.561  0.73  2.434  0.847  0.139  0.427  2.11  1.384  2.014  0.095  0.458  0.047  1.507  0.911  0.566  0.503  0.324  0.114  0.28  0.111  1.398  1.005  
 
 TNR  7143  1  175291934  175712752  1q24  0.039  0  0.079  0  0.038  0.057  0.025  0.022  0.091  0.014  0.029  0.032  0  0.026  0  0.035  0.027  0.041  0  0.057  0  0.087  0.346  0  0.029  0  0.121  0.03  0  0.087  0  0  0  0.19  0  0  0  0  0.039  0  0  0.087  0.05  0  0  0.026  0.035  0  0  0.026  0.041  0.367  0.016  0.093  0.054  0.064  0.02  0  0.035  0  
 
 LOC101928751  101928751  1  175846478  175849600  1q25.1  0  0  0  0  0  0  0  0  0.143  0  0  0  0  0  0  0  0  0  0  0  0  0.062  0.369  0  0  0  0.035  0  0  0.021  0  0  0  0.116  0  0  0  0  0  0  0  0.078  0.027  0  0  0.056  0  0  0  0  0  0.099  0  0.043  0  0  0  0  0  0  
 
 RFWD2  64326  1  175913961  176176380  1q25.1-q25.2  2.982  2.524  1.51  1.623  2.885  1.249  2.914  2.505  1.998  2.64  2.819  2.28  2.607  3.133  1.869  2.449  2.661  2.19  4.09  2.736  2.363  2.433  1.726  1.431  2.295  2.195  1.977  2.214  1.691  1.467  2.768  2.441  2.089  0.907  1.429  2.268  2.492  2.131  2.4  2.462  2.393  1.801  3.48  1.932  1.61  2.75  2.167  1.438  1.965  3.093  0.469  1.747  1.918  1.519  2.534  2.662  1.861  1.538  2.726  2.526  
 
 SCARNA3  677679  1  175937532  175937676  1q25.1  0  0  0  0  0  0  0  0  0  0  0  0  0  0  0  0  0  0  0  0  0  1.145  0  0  0  0  0.802  0  0  0  0  0  0  0  0  0  0  0  0  0  0  0  0  0  0  0  0  0  0  0  0  0  1.483  0  0  0  0  0  0  0  
 
 PAPPA2  60676  1  176432306  176811970  1q23-q25  0  0.031  0.556  0.278  0.029  0  0.176  0.81  0.358  0.826  0.097  0  0  0.026  0  0.021  0.036  0.029  0  0.03  0  0.071  0.369  0  0.614  0.023  0.047  0.056  0  0.147  0.018  0  0  0.204  0  0  0.036  0.201  0.2  0  0.024  0.409  0.038  0  0  0.013  0.035  0  0  0  0.12  0.161  0.037  0.13  0.04  0  0.162  0.067  0  0  
 
 ASTN1  460  1  176826440  177134040  1q25.2  0  0.045  0.144  0  0.032  0.03  0.067  0.045  0.107  0.829  0.026  0.064  0  0.032  0  0.053  0.036  0.075  0.03  0.028  0.093  0.165  0.363  0  0  0  0.117  0.022  0  0.096  0  0  0  0.216  0.048  0.03  0.027  0  0.071  0.041  0  0.504  0.067  0  0  0.029  0.045  0  0  0  0.041  0.521  0.022  0.09  0.056  0.038  0  0.445  0  0  
 
 BRINP2  57795  1  177140523  177251558  1q24  1.091  0  0.201  0  0.03  0.049  0.039  0.055  0.109  0.28  0.047  0.098  0  0.039  0  0.061  0.046  0.113  0.021  0.038  0.081  0.139  0.422  0.038  0  0.035  0.163  0.024  0  0.149  0  0.034  0  0.174  0  0.082  0.024  0  0.073  0  0.062  0.887  0.069  0.058  0.07  0.054  0.056  0  0  0  0.408  0.385  0.026  0.12  0.064  0.084  0  0  0  0  
 
 LOC101928778  101928778  1  177669658  177679465  -  0  0  0.126  0  0  0  0  0  0.1  0  0  0  0  0  0  0  0  0  0  0  0  0.07  0  0  0  0  0.057  0  0  0.033  0  0  0  0.146  0  0  0  0  0  0  0  0.078  0  0  0  0  0  0  0  0  0  0.103  0  0.056  0  0  0  0  0  0  
 
 SEC16B  89866  1  177898241  177939050  1q25.2  0  0.086  0.197  0  0.048  0  0.204  0.328  0.042  0.93  0.04  0.905  0.71  0.152  0.041  0.283  0.081  0  0.064  0.071  0  0.095  0.408  0.37  0.106  0.415  0.112  0.323  0.055  0.141  0.147  0.168  0.062  0.157  0.097  0.154  0.091  0.529  0.038  0.115  0.06  0.265  0.221  0.431  0.049  0.129  0.118  0  0.094  0.087  0.085  0.247  0.111  0.832  1.095  0.472  1.015  0  0.174  0.152  
 
 LOC730102  730102  1  177975274  178007142  1q25.2  0.301  1.668  0.54  0.21  0.801  0.09  0.201  0.449  0.072  0.053  0.129  2.206  2.298  1.027  0  0.901  0.049  0.425  0  0.975  0  0.079  0.209  0  0.15  2.474  1.65  1.756  0.712  1.597  0.805  1.464  1.989  1.251  0.896  1.27  0.421  1.807  0.33  0.737  0.431  0.57  0.852  1.579  0.356  1.147  0.986  0.102  0.435  0.207  0.231  0.932  0.808  1.459  3.925  1.85  2.005  0.495  0.476  1.229  
 
 RASAL2-AS1  100302401  1  178060642  178063128  -  0.082  0.479  0.332  0.122  0.245  0.174  0.42  0.348  0.235  0.473  0.276  0.06  0.245  0.133  0.085  0.173  0.08  0.272  0  0.211  0  0.069  0.373  0  0  0.847  0.288  0.429  0.433  0.024  0.536  0.316  0.431  0.416  0.283  0.199  0.139  0.37  1.039  0.249  0.287  0.241  0.601  0.206  0.161  0.33  0.053  0.268  0.402  0.893  0.167  0.339  0.296  0.174  0.869  0.449  0.357  0.368  0.725  0.383  
 
 RASAL2  9462  1  178062863  178448648  1q24  0.856  1.937  0.966  1.485  1.386  0.937  1.67  1.437  0.85  2.052  1.771  0.8  0.949  1.437  0.878  1.068  0.578  0.664  0.035  0.774  0.086  0.115  0.259  0.026  0.717  2.597  1.117  1.981  1.241  1.029  1.176  0.948  1.018  0.594  0.753  1.364  1.366  2.03  2.471  1.486  2.159  0.748  1.233  1.579  0.939  1.751  1.345  1.946  1.842  2.153  0.678  1.435  1.853  1.184  3.037  2.217  1.757  1.595  2.705  1.921  
 
 TEX35  84066  1  178482211  178492635  1q25.2  0  0  0  0  0  0  0  0  0  0  0  0  0  0  0  0  0  0  0  0  0  0  0  0  0  0  0  0  0  0  0  0  0  0  0  0  0  0  0  0  0  0  0  0  0  0  0  0  0  0  0  0.292  0  0  0  0  0  0  0  0  
 
 C1orf220  400798  1  178511930  178518024  1q25.2  0.624  0.161  0.241  0.145  0.193  0.237  0.136  0.302  0.093  0.634  0.145  0.058  0.251  0.096  0.292  0.133  0.136  0.144  0  0.088  0  0.068  0.537  0  0  0.668  0.683  0.363  0.194  0.085  0.22  0.273  0.476  0.313  0.191  0.265  0.211  0.372  0.158  0.163  0.265  0.276  0.465  0.08  0.099  0.22  0.675  0.156  0.072  0.17  0.121  0.246  0.339  0.114  0.484  0.299  0.27  0.207  0.813  0.064  
 
 RALGPS2  55103  1  178694281  178890977  1q25.2  1.398  1.845  1.237  2.437  1.292  1.008  1.054  0.909  0.502  2.004  1.063  1.551  0.843  1.338  1.104  1.46  1.367  0.689  1.042  0.786  0.245  0.776  0.218  0.292  0.534  1.864  2.579  1.042  0.694  1.827  0.936  0.858  1.511  0.765  0.797  0.736  1.119  1.405  1.097  0.557  1.288  0.765  1.403  1.43  1.21  1.677  2.289  0.831  1.402  1.957  1.414  1.278  0.976  0.809  1.578  2.393  1.502  0.682  2.211  1.571  
 
 ANGPTL1  9068  1  178818669  178840215  1q25.2  0.129  0.135  0.12  0.106  0.16  0  0.036  0.125  0.18  0.179  0.074  0.049  0.087  0.181  0.14  0.169  0.08  0.111  0.019  0.068  0  0.103  0.08  0.03  0  0.154  0.152  0.085  0.069  0.047  0.112  0.456  0.437  0.18  0.075  0.031  0.115  0.143  0.069  0  0.136  0.09  0.026  0.14  0.066  0.189  0.116  0.136  0  0.077  0.047  0.233  0.082  0  0.218  0.191  0.178  0  0.285  0  
 
 FAM20B  9917  1  178995073  179045702  1q25  2.97  2.927  1.859  2.132  2.657  2.181  2.971  2.559  1.597  2.787  2.822  2.079  2.188  2.693  2.153  1.876  1.818  2.121  2.79  2.209  2.445  1.78  1.33  1.466  1.904  2.626  2.168  2.45  2.38  1.52  2.353  2.352  2.485  1.327  1.786  2.224  2.802  2.396  2.486  2.492  2.916  2.122  2.602  2.231  1.719  2.298  2.321  1.551  2.023  2.298  1.595  1.391  2.145  1.609  2.132  2.655  2.073  1.938  2.322  2.07  
 
 TOR3A  64222  1  179051111  179065129  1q25.2  2.99  1.413  1.908  2.584  3.012  1.882  3.751  2.992  2.575  2.89  3.699  2.538  3.26  2.93  2.57  2.273  2.223  0.477  4.6  1.947  2.811  2.512  3.35  2.508  1.682  2.851  2.624  1.929  2.242  1.467  2.504  3.386  2.515  1.645  2.123  2.602  2.694  1.978  2.101  3.235  3.09  4.571  3.401  2.857  1.508  2.628  2.738  1.626  1.862  2.976  1.8  1.841  2.314  1.095  2.198  2.409  2.077  2.352  2.629  2.021  
 
 ABL2  27  1  179068461  179198819  1q25.2  1.553  2.778  1.772  3.499  1.657  2.003  2.268  3.14  2.012  2.525  2.775  0.519  1.148  1.824  1.269  0.807  0.854  0.995  1.572  1.123  1.526  1.148  1.111  1.123  2.544  3.479  2.64  3.514  2.511  2.49  2.518  2.204  2.715  1.425  0.996  2.247  3.085  2.924  3.435  2.138  2.085  1.247  2.666  1.791  0.895  1.833  1.425  2.656  2.403  2.655  1.312  2.241  2.133  1.894  2.558  2.527  2.559  3.108  3.097  3.066  
 
 SOAT1  6646  1  179262848  179327814  1q25  0.293  2.898  0.969  1.675  1.154  1.687  1.617  1.669  1.176  1.187  1.881  0  0.901  1.476  0.739  0.04  0.618  1.365  2.101  0.748  0.978  1.382  1.215  0.921  1.722  1.892  1.216  1.721  2.238  0.95  2.489  1.803  1.472  0.962  0.546  1.506  1.73  1.728  2.528  1.591  1.564  0.988  1.702  1.606  0.556  1.22  0.713  1.166  1.123  2.624  0.864  0.846  1.331  1.497  2.127  1.974  2.046  1.74  2.67  1.824  
 
 AXDND1  126859  1  179334854  179523870  1q25.2  0  0.054  0.05  0  0.04  0.069  0.031  0  0.045  0  0.074  0  0  0.224  0  0  0  0.151  0.042  0  0  0.048  0.116  0  0  0.025  0.045  0.179  0  0.049  0  0.056  0  0.068  0  0.05  0.035  0  0.07  0  0.045  0.027  0.034  0.037  0  0.04  0.036  0  0  0  0  0.152  0  0.043  0  0  0  0  0  0  
 
 NPHS2  7827  1  179519673  179545087  1q25.2  0  0  0.11  0  0  0.067  0  0.074  0.123  0  0  0  0  0.044  0  0.051  0  0  0  0  0  0.07  0.247  0  0  0  0.181  0.081  0  0.043  0  0  0  0.145  0  0  0  0  0  0  0  0.391  0.031  0  0  0.042  0  0  0  0  0.055  0.584  0  0.144  0.124  0  0  0  0  0  
 
 TDRD5  163589  1  179560747  179660407  1q25.2  0.864  0  0.105  0  0.833  0  0  0.028  0.068  0.053  0.034  0.086  0  0.156  0  0.082  0.059  0.186  0  0  0  0.139  0.313  0.052  0.056  0.051  0.069  0.049  0.081  0.062  0  0  0  0.104  0  0.097  0.044  0  0.188  0.297  0.291  0.053  0.032  0  0.195  0.079  0.094  0.204  0  0.598  0  0.402  0  0.056  0.054  0.104  0  0  0  0  
 
 FAM163A  148753  1  179712297  179785333  1q25.2  0.134  0  0.134  0.093  0.04  0  0  0.03  0.104  0.022  0.054  0.047  0  0.023  0  0.048  0.047  0.117  0.024  0  0  0.109  0.396  0  0  0.034  0.084  0.04  0  0.072  0  0  0  0.18  0  0  0  0  0.218  0.067  0  0.948  0.037  0  0.054  0  0.549  0  0  0  0  0.315  0  0.092  0  0.062  0  0  0  0  
 
 TOR1AIP2  163590  1  179809101  179846941  1q25.2  2.75  3.241  2.044  2.317  3.189  2.167  2.795  3.273  2.229  2.979  2.968  2.012  2.818  3.352  3.012  2.221  2.473  2.164  2.874  2.761  2.86  2.103  1.89  2.159  2.82  3.119  2.287  3.393  2.558  1.811  2.676  3.625  3.142  2.099  1.897  2.993  3.184  2.934  3.443  3.091  2.789  1.779  2.954  2.819  2.61  2.921  2.775  2.721  2.202  3.895  1.482  2.226  2.388  2.343  2.417  3.227  2.654  2.632  2.876  2.753  
 
 TOR1AIP1  26092  1  179851176  179889212  1q24.2  2.413  3.537  1.721  1.942  2.869  1.372  2.568  3.001  2.435  2.727  2.624  1.889  1.92  2.495  1.727  1.811  2.403  2.387  3.573  2.698  2.014  1.94  1.36  1.25  2.141  3.267  2.143  1.916  2.309  1.262  2.705  3.242  2.614  1.915  1.55  2.716  1.663  1.97  2.799  2.233  2.546  1.931  2.207  2.221  1.279  2.352  1.821  1.39  1.782  2.924  1.285  1.539  1.96  1.928  2.285  2.592  1.896  1.553  2.512  2.541  
 
 CEP350  9857  1  179923907  180084015  1q25.2  2.133  2.664  0.997  1.179  1.969  0.89  1.565  1.92  1.294  1.759  1.908  1.116  1.505  1.996  1.47  1.231  1.365  1.924  2.201  1.952  1.532  1.732  1.196  0.93  0.997  2.421  1.292  1.686  1.5  1.135  1.761  1.988  1.354  0.7  0.986  1.647  1.662  1.426  1.673  1.888  1.758  1.062  1.771  1.561  0.877  1.762  1.243  1.028  1.332  1.247  0.865  0.98  1.307  0.923  1.646  1.496  1.713  1.112  1.589  1.344  
 
 QSOX1  5768  1  180123967  180167169  1q24  2.613  2.346  5.506  5.785  2.308  4.925  5.445  5.143  4.199  5.687  5.427  3.793  3.295  2.815  3.522  3.935  4.35  0.42  3.817  4.792  2.067  2.027  1.775  1.337  4.301  4.151  3.304  4.567  3.591  2.617  3.151  4.504  3.677  2.538  2.585  3.837  6.085  5.468  5.97  4.176  4.188  4.161  3.335  4.106  4.201  5.644  5.597  3.928  4.519  5.6  5.73  4.08  4.338  3.737  4.458  5.387  5.228  4.833  4.215  5.318  
 
 FLJ23867  200058  1  180167143  180169859  1q25.2  1.415  1.101  2.078  1.003  2.566  1.059  1.339  1.092  0.815  1.29  1.505  3.153  1.676  1.931  0.791  3.11  2.316  1.275  0.46  0.645  0.149  0.242  0.681  0.135  0.766  0.828  0.676  0.558  0.169  0.238  0.477  0.633  0.566  0.53  0.333  0.709  1.639  1.434  2.837  0.768  3.752  0.69  0.291  0.621  3.476  5.501  2.059  0.525  1.51  1.017  2.367  1.582  0.888  0.535  1.378  1.113  1.973  1.795  0.918  1.43  
 
 LHX4  89884  1  180199432  180244188  1q25.2  1.256  0.247  0.31  0  0.786  0.563  0.132  0.283  0.161  0.521  0.337  0.189  0.389  0.482  1.309  0.465  0.214  0.099  0.105  0.425  0.383  0.39  0.341  0.301  0.079  0.46  0.433  0.941  0.303  0.485  0.183  0.254  0.453  0.226  0.38  0.568  0.787  0.38  0.401  0.167  0.524  0.484  1.042  0.53  0.107  0.459  0.031  0.322  0.585  0.265  0.075  0.587  0.433  0.184  0.434  0.414  0.414  0.149  0.469  0.181  
 
 LHX4-AS1  100527964  1  180238797  180243816  -  1.863  0.413  0.482  0.512  1.442  0.327  0.798  0.529  0.368  1.466  0.96  0.292  0.976  1.354  1.752  1.338  0.537  0.532  2.212  0.919  1.197  1.674  1.099  0.879  0.296  0.993  1.315  1.045  0.882  0.858  1.089  0.829  0.962  0.321  0.764  1.008  1.016  1.543  1.007  0.987  1.008  1.208  2.081  0.964  0.427  1.296  1.381  0.854  0.876  1.535  0.182  0.758  0.994  0.608  1.344  0.709  0.985  0.574  1.343  0.403  
 
 ACBD6  84320  1  180257351  180472022  1q25.1  4.234  5.14  2.759  3.648  4.16  2.8  4.298  3.995  4.15  3.563  4.294  2.943  2.993  4.125  3.4  3.15  3.165  3.843  5.469  4.458  3.799  3.731  3.299  3.259  3.252  3.154  2.971  3.272  2.505  3.01  3.799  3.728  2.999  2.075  3.283  3.867  3.689  3.278  4.048  3.263  4.086  3.85  3.844  3.74  2.695  3.985  3.371  3.052  2.724  4.332  1.9  2.728  3.469  2.819  4.346  3.705  3.163  3.497  3.303  3.362  
 
 OVAAL  148756  1  180528109  180535654  1q25.3  0  0.117  0.112  0  0  0  0.463  0  0  0  0  0  0  0  0  0  0  0  0  0  0  0.053  0.554  0.148  5.286  2.069  1.024  4.266  0.094  3.258  0  0.303  0.506  1.62  0  0  0  0  0.068  0  0  0  0.062  0.213  0  0.353  0.127  0  0  0  0  0  0.054  0.069  0  0  0  0  0  0  
 
 XPR1  9213  1  180601145  180859415  1q25.1  1.554  1.99  0.755  0.96  1.577  0.961  1.481  1.687  1.295  1.699  1.716  1.338  1.041  2.043  0.979  0.723  1.45  1.611  1.403  0.989  1.937  0.862  0.794  0.887  1.537  1.17  0.754  1.306  0.892  0.895  0.996  1.5  0.834  0.595  1.21  2.539  1.473  1.187  1.409  2.064  1.689  0.813  1.804  1.521  1.033  1.773  1.115  0.683  1.177  1.206  0.589  1.126  1.079  1.301  1.699  1.727  1.248  1.431  1.823  1.772  
 
 KIAA1614  57710  1  180882312  180915239  1q25.3  0.071  0.044  0.361  0.077  0.17  0.191  0.053  0.788  0.326  1.048  0.259  0.125  0.085  0.152  0.098  0.038  0.061  0.123  0.148  0.088  0.078  0.298  0.627  0.081  0.048  0.165  0.153  0.29  0.042  0.092  0.291  0.135  0.229  0.249  0.092  0.48  0.128  0.204  0.193  0.183  0.113  0.441  0.061  0.272  0.079  0.298  0.046  0.073  0.117  0.044  0.043  0.706  0.13  0.14  0.194  0.108  0.103  0.206  0.085  0.028  
 
 STX6  10228  1  180941849  180992257  1q25.3  2.12  1.786  1.595  1.757  1.885  1.209  1.891  1.709  2.273  1.693  2.151  1.052  1.673  1.592  1.472  1.367  1.524  1.1  2.861  0.951  1  1.451  1.608  1.185  1.398  1.975  1.336  1.76  1.435  1.147  2.036  1.872  1.606  0.888  0.999  1.765  1.791  1.363  1.775  1.745  1.609  1.174  2.145  1.651  0.994  1.858  0.939  0.77  1.052  1.982  1.029  1.071  1.218  1.012  1.621  1.708  0.98  1.302  1.45  1.687  
 
 MR1  3140  1  181002560  181031074  1q25.3  0.568  0.268  0.585  0.719  1.51  0.195  1.253  1.617  0.712  1.059  0.739  0.443  0.344  0.696  0.275  0.128  0.303  0.282  1.294  0.768  0.323  0.872  1.31  0.777  0.181  0.756  0.415  0.519  0.553  0.366  0.6  0.722  0.249  0.462  0.196  0.759  0.715  0.836  0.828  0.163  0.514  0.406  0.183  0.332  0.044  0.866  0.351  0.115  0.069  0.29  0.118  0.28  0.362  0.929  1.11  0.571  0.316  0.131  0.603  1.232  
 
 IER5  51278  1  181057637  181059979  1q25.3  1.515  3.849  2.447  2.375  3.05  2.609  1.853  2.439  2.779  1.815  3.068  1.858  0.797  3.354  1.297  1.999  1.346  3.019  2.954  1.742  2.516  1.422  2.694  2.771  2.357  2.469  1.246  2.271  1.799  1.897  2.786  2.732  1.714  1.279  2.215  2.595  3.643  2.945  4.083  2.541  3.321  2.625  1.929  2.204  1.399  2.473  1.714  2.745  1.936  4.349  1.418  2.013  2.388  2.326  1.907  2.528  1.533  3.321  1.909  2.548  
 
 LOC101928973  101928973  1  181143619  181151342  1q25.3  0  0  0  0  0.132  0  0  0  0  0.123  0  0  0  0  0  0  0  0  0  0  0  0  0  0  0  0  0  0.747  0  0  0  1.408  0  0  0  0  0  0  0  0  0  0  0  0  0  0  0  0  0  0  0  0.38  0  0  0.196  0  0  0  0  0  
 
 CACNA1E  777  1  181452685  181775921  1q25.3  0.024  0.014  0.108  0.047  0.025  0.046  0.018  0.021  0.108  0.037  0.02  0.214  0.03  0.025  0.014  0.018  0.027  0.065  0.015  0.081  0  0.073  0.339  0.064  0.012  0.19  0.139  0.077  0.008  0.109  0.027  0.018  0.02  0.156  0.033  0.02  0.012  0.269  0.053  0.02  0.015  0.141  0.054  0.268  0.013  0.047  0.075  0  0  0.013  0.022  0.231  0.012  0.061  0.028  0.032  0.016  0.019  0  0  
 
 ZNF648  127665  1  182023704  182030847  1q25.3  0  0.035  0.122  0  0.063  0  0.101  0.038  0.129  0.029  0.022  0.047  0  0.234  0  0.055  0  0.116  0  0.039  0.1  0.192  0.463  0  0.039  0.349  0.29  0.644  0.093  0.178  0.147  0.041  0.077  0.24  0  0.041  0  0  0.108  0  0.029  0.448  0.054  0.216  0.063  0.038  0.041  0  0  0  0.031  0.82  0.019  0.087  0.036  0  0  0  0  0  
 
 FLJ46134  400799  1  182173079  182283196  1q25.3  0  0  0  0  0  0  0  0  0  0  0  0  0  0  0  0  0  0  0  0  0  0  0  0  0  0  0  0  0  0.088  0  0  0  0  0  0  0  0  0  0  0  0  0  0  0  0  0.132  0  0  0  0  0  0  0  0  0  0  0  0  0  
 
 GLUL  2752  1  182347227  182361341  1q31  2.001  2.507  0.182  1.197  3.369  1.545  1.144  2.508  3.958  2.329  2.627  0.108  1.903  2.821  2.173  1.574  1.581  3.992  3.878  3.97  4.862  3.913  0.903  1.573  0  1.982  0.924  2.16  0.193  2.316  2.902  0.601  1.988  1.649  0.3  2.455  2.546  0  1.772  1.519  2.233  1.551  0.257  2.85  2.174  2.723  2.219  0  0.413  0.044  2.277  1.58  0.043  0.039  1.886  0.034  1.321  1.908  1.296  2.074  
 
 TEDDM1  127670  1  182367251  182369751  1q25.3  0.163  0  0.175  0  0.051  0  0  0.034  0.158  0  0.038  0.054  0  0.083  0.206  0  0.066  0  0  0.042  0  0.114  0.22  0  0  0.051  0.183  0.064  0  0.054  0  0  0  0.163  0.058  0  0.036  0  0.08  0  0.152  0.098  0.037  0  0  0.045  0.088  0  0  0  0  0.415  0  0.028  0.031  0  0  0  0.077  0  
 
 LINC00272  388719  1  182376755  182383948  1q25.3  0  0  0  0  0  0  0  0  0  0  0  0  0  0  0  0  0  0  0  0  0  0  0  0  0  0  0  0  0  0.045  0  0  0  0  0  0  0  0  0  0  0  0  0  0  0  0  0.088  0  0  0  0  0  0  0  0  0  0  0  0  0  
 
 RGSL1  353299  1  182419255  182529732  1q25  0  0  0  0  0  0.074  0  0  0.173  0  0  0  0  0.02  0  0.029  0  0.047  0.022  0  0  0.033  0.338  0  0  0  0.036  0.027  0  0.05  0  0  0  0.107  0  0  0  0  0.014  0  0  0.046  0.027  0  0  0  0.021  0  0  0  0  0.192  0  0.027  0  0  0  0  0  0  
 
 RNASEL  6041  1  182542768  182558394  1q25  0.792  0.026  0.13  0.154  0.784  0  0.945  0.983  0.928  0.828  1.147  0.48  0.864  0.522  0.365  0.455  0.367  0.035  1.342  0.705  0.537  0.525  0.434  0.168  0.245  0.776  0.761  0.489  0.433  0.188  0.705  0.761  0.436  0.363  0.218  0.448  0.16  0.151  0.488  0.414  0.41  0.437  0.032  0.333  0.375  0.202  0.101  0.095  0.068  0.195  0.177  0.326  0.29  0.792  0.4  0.524  0.387  0.158  0.186  0.439  
 
 RGS16  6004  1  182567757  182573548  1q25-q31  1.748  0  0.22  0.664  0.836  0  0.1  0.17  6.66  0.983  1.946  0.263  0.899  0.929  0.858  0.244  0.557  0.658  1.816  1.188  0.139  0.961  2.948  2.072  0.471  0  0.182  0.199  0  0.216  0.098  1.362  0  0.478  0  0.072  0  0.426  0.265  1.41  0  2.045  0.041  1.155  0.197  0.188  0.356  0.143  0  0.698  0.353  0.29  0.031  0.091  0  0  0.053  0  0  0  
 
 LOC284648  284648  1  182584274  182585764  1q25.3  0  0  0  0  0.139  0  0.132  0.093  1.013  0.06  0.237  0.11  0  0  0  0  0  0.195  0  0.075  0  0.083  0.365  0  0  0  0.099  0.089  0  0.062  0  0  0  0.33  0  0  0  0  0.049  0.11  0  0.592  0  0  0  0  0.028  0  0  0.106  0  0.556  0  0.063  0.045  0.091  0  0  0  0  
 
 RGS8  85397  1  182615791  182642067  1q25  0  0  0  0  0  0  0  0  0.191  0  0  0  0  0  0  0  0  0  0  0  0  0.145  0  0  0  0  0.108  0  0  0.075  0  0  0  0  0  0  0  0  0  0  0  0.504  0  0  0  0  0  0  0  0  0  0.219  0  0.114  0  0  0  0  0  0  
 
 NPL  80896  1  182758583  182799519  1q25  0.218  0  0.089  0.213  0.828  0  0.217  0.163  0.453  0.407  0.308  0.272  0.269  0.068  0.15  0.166  0.516  0.272  0.413  0.678  0.258  0.489  1.555  0.665  0.253  0.703  0.433  0.612  0.666  0.051  1.375  0.864  0.375  0.241  0.084  0.072  0  0.228  0.141  0.386  0.409  0.323  0.29  0.276  0.242  0.117  0.243  0  0.404  0.534  0.07  0.509  0  0.476  0.057  0  0  0.348  0  0  
 
 DHX9  1660  1  182808438  182857117  1q25  4.281  3.719  2.588  2.996  4.188  3.08  4.816  3.932  3.358  3.988  5.082  3.403  3.861  4.783  4.147  4.01  3.998  3.254  6.114  3.414  4.441  3.947  2.667  3.121  3.945  4.237  3.84  3.937  4.038  3.807  4.55  4.949  4.249  2.416  3.403  4.388  4.587  3.784  3.267  4.929  3.928  3.428  5.075  4.588  3.297  3.848  2.476  3.582  4.084  5.443  3.508  3.114  3.998  3.18  4.023  4.087  3.961  3.807  4.588  4.585  
 
 SHCBP1L  81626  1  182868999  182922553  1q25.3  0  0  0.338  0.187  0.088  0.06  0.252  0.118  0.259  0.517  0.1  0.079  0.159  0.098  0.078  0.076  0.093  0.063  0  0.088  0  0.194  0.276  0  0.056  0.148  0.509  0.126  0.237  0.143  0.146  0.128  0.108  0.27  0.175  0.071  0.157  0  0.569  0.068  0.063  0.335  0.261  0.206  0  0.122  0.219  0.166  0  0.045  0.09  0.413  0.263  0.122  0.365  0.142  0.173  0.127  0.199  0  
 
 LAMC1  3915  1  182992594  183114727  1q31  2.093  2.297  4.576  3.588  2.042  2.102  4.163  4.564  3.432  5.596  4.081  0.265  2.92  2.402  2.502  1.496  2.529  0.844  0.06  0.24  0.549  0.316  0.576  0.625  2.685  4.197  3.228  2.958  3.832  2.994  3.24  4.916  3.85  2.122  2.392  3.104  3.736  3.186  4.627  4.124  3.493  3.216  3.585  6.044  3.218  3.681  3.182  3.27  4.264  3.625  2.224  2.465  4.009  2.806  3.998  4.367  4.376  3.15  4.211  3.98  
 
 LAMC2  3918  1  183155173  183214262  1q25-q31  0  0.876  0.786  0.2  0.395  0.027  0.147  2.223  0.12  0.159  0.349  1.229  2.592  1.316  1.126  1.701  2.362  0.436  0.078  0.063  0.1  0.082  0.343  0  0.17  0.053  0.077  0.058  0.076  0.046  0  0.176  0.026  0.252  0.726  2.776  0.754  1.766  3.504  1.887  4.436  0.221  0.032  1.612  2.154  2.962  2.925  2.385  4.332  3.983  4.318  1.718  3.364  0.281  3.507  2.748  3.686  1.11  5.213  2.461  
 
 NMNAT2  23057  1  183217371  183387634  1q25  0.254  0.36  0.734  0.48  0.534  0.16  0.649  0.583  1.402  0.466  1.026  0.06  0.072  0.066  0.51  0.05  0.042  0.049  0.045  0.045  0.128  0.116  0.424  0  0.07  0.026  0.176  0.04  0  0.051  0  0.022  0  0.274  0.181  0.101  0.747  0.068  2.803  0.844  0.343  0.901  0.583  3.279  1.26  0.473  2.966  2.428  1.065  2.607  1.43  0.314  0.028  0.033  0.092  0.305  0.122  2.533  0.484  0.138  
 
 SMG7-AS1  284649  1  183430010  183441117  1q25.3  0.261  0  0.174  0  0.29  0.255  0.043  0.164  0.174  0.123  0.16  0.193  0.194  0.147  0.112  0.131  0.24  0.115  0.14  0.09  0.1  0.203  0.316  0.256  0.071  0.189  0.179  0.17  0.136  0.14  0.099  0.103  0.189  0.183  0.131  0.197  0.115  0.173  0.09  0.094  0.276  0.168  0.168  0.267  0.266  0.242  0.038  0.084  0.133  0  0.065  0.273  0.133  0.243  0.101  0.206  0.138  0.161  0.212  0.098  
 
 SMG7  9887  1  183441505  183523328  1q25  3.994  2.786  2.116  2.625  4.066  1.973  3.343  3.907  2.718  3.457  4.006  3.232  3.476  3.619  3.024  3.235  3.26  1.779  3.749  2.899  3.356  3.804  2.986  3.097  3.184  3.374  2.72  3.2  3.2  2.529  3.038  3.497  3.123  1.711  2.317  3.513  3.709  3.236  3.788  3.786  3.901  2.297  3.756  3.56  3.168  3.372  2.602  2.175  2.172  2.521  2.586  2.255  2.829  2.453  2.562  3.159  2.977  2.703  3.257  2.636  
 
 NCF2  4688  1  183524696  183560056  1q25  0.21  0.477  0.175  0.189  1.329  0.426  0.175  1.423  0.128  0.103  0.476  0.183  0.494  0.17  0.232  0.148  1.023  0.115  0.086  0.433  0  0.274  2.418  0.866  0.075  0.135  0.115  0.154  0.114  0.041  0.148  0  0.138  0.111  0.245  0.13  0.562  0.134  1.49  0  1.416  0.317  0.03  0.846  0  0.649  0.747  0.746  0.546  1.42  0.876  0.18  0.376  1.876  0.077  0  0.538  0.083  0.293  0.326  
 
 ARPC5  10092  1  183595327  183605076  1q25.3  4.15  6.447  4.724  4.924  4.319  3.465  4.514  5.6  4.579  5.098  5.177  4.004  3.926  4.625  2.902  4.099  5.193  6.2  6.414  4.32  3.753  4.102  4.662  3.677  3.931  4.734  3.434  3.356  3.348  2.642  4.715  4.22  3.717  3.452  3.233  4.629  3.859  3.467  5.557  3.906  3.826  4.102  4.472  4.655  2.922  4.868  2.557  3.496  3.982  5.589  3.621  2.922  3.388  4.031  4.033  4.392  4.081  3.984  4.023  4.615  
 
 RGL1  23179  1  183605181  183897685  1q25.3  0.142  0.767  0.808  0.461  0.173  0.403  1.269  1.173  1.393  2.288  1.462  0.212  0.204  0.474  0.192  0.039  0.32  2.003  0.676  0.092  0.061  0.483  2.175  1.842  0.215  0.852  0.289  0.345  0.369  0.406  3.457  1.788  1.222  0.723  0.186  0.892  0.667  0.773  0.713  0.315  0.314  0.6  0.835  0.69  0.55  0.689  0.719  0.52  0.162  0.457  0.202  0.345  0.183  0.389  0.702  0.351  0.483  0.109  0.047  1.548  
 
 APOBEC4  403314  1  183615410  183622448  1q25.3  0  0  0.065  0  0.041  0  0  0.043  0.087  0.06  0  0.034  0  0  0  0  0  0  0  0.033  0  0.081  0.206  0  0  0.089  0.031  0  0  0.016  0.071  0  0  0.098  0  0  0.032  0  0.033  0  0  0.046  0  0  0  0  0.021  0  0  0  0  0.101  0  0  0.033  0  0.032  0  0  0  
 
 COLGALT2  23127  1  183898795  184006904  1q25  0.122  0.63  0.533  0.634  0.071  0.433  0.114  0.93  2.467  0.691  1.658  0.907  0  0.082  0.063  0.077  0.127  2.441  0.148  0.622  2.382  0.452  0.462  0.107  0.324  0.849  0.246  0.436  0.35  1.17  0.812  2.252  0.561  0.873  0.098  0  0.055  0.501  0.291  0.117  0.637  0.356  3.895  0.279  0.073  0.291  0.971  0.336  0  0.81  0.056  0.92  0.06  0.181  0.257  0.09  0.264  0.093  0  0.071  
 
 TSEN15  116461  1  184020784  184043344  1q25  2.605  4.919  2.758  2.579  3.296  2.645  2.856  3.296  3.453  2.933  3.655  2.726  1.805  3.254  1.792  2.454  3.029  4.353  4.556  2.839  3.423  3.186  3.858  2.552  2.272  2.786  2.02  2.588  1.774  1.785  2.748  2.816  2.357  1.866  2.1  1.971  2.618  2.247  3.134  2.696  2.824  2.574  4.085  2.013  1.8  3.029  3.381  1.776  2.177  3.697  1.731  1.708  2.657  1.916  3.126  2.798  2.312  2.754  2.794  2.955  
 
 C1orf21  81563  1  184356149  184598155  1q25  1.395  2.029  0.742  0.772  0.179  0.764  0.802  0.699  0.791  1.897  0.94  0.717  0.384  0.818  0.432  1.011  1.943  0.078  1.311  0.054  0  0.844  0.407  0.13  1.005  1.389  1.799  0.725  1.182  0.418  1.726  1.669  1.069  0.924  0.176  0  0.095  0.022  1.748  0.087  0.522  0.466  1.352  0.647  0.168  0.772  0.63  0.312  0.331  0.782  0.311  0.37  0.008  0.35  0.041  0.097  0.242  0.633  0.075  0.026  
 
 EDEM3  80267  1  184659624  184724041  1q25  1.278  2.079  0.936  1.449  1.746  0.534  1.522  2.061  1.39  2.071  1.635  1.419  1.925  1.206  1.031  1.115  1.512  1.15  2.538  2.024  1.597  1.559  1.699  1.489  1.353  2.159  0.861  1.233  1.285  0.394  1.47  1.861  0.978  0.682  0.483  1.568  1.415  1.226  1.498  1.343  1.304  1.055  1.019  1.07  0.72  1.574  1.697  0.395  0.762  0.677  1.149  0.902  1.067  1.021  0.823  1.491  1.254  0.709  1.208  1.368  
 
 FAM129A  116496  1  184760158  184943718  1q25  0.294  3.793  1.876  3.072  0.952  1.895  2.721  1.554  3.104  2.595  4.226  0.016  0.064  0.91  0.16  0.565  1.502  0.134  1.059  1.827  1.573  0.233  1.76  0.328  2.504  1.648  1.84  0.269  0.606  1.07  0.02  0.531  1.27  1.404  0.178  0.036  1.191  1.667  3.903  0.677  0.398  1.376  0.33  0.946  1.048  2.51  0.766  1.741  1.998  2.841  0.875  1.408  2.369  2.424  3.357  2.953  2.398  2.72  3.11  2.075  
 
 RNF2  6045  1  185014550  185071740  1q25.3  1.74  2.177  1.065  1.371  1.413  1.245  1.692  1.927  1.262  1.33  1.895  0.838  1.504  2.867  1.51  1.568  1.474  1.041  2.887  1.287  1.698  1.387  0.729  0.745  2.008  1.766  1.105  1.502  1.358  1.43  2.058  1.905  1.315  0.687  0.966  1.119  1.963  1.338  1.34  1.945  1.572  1.118  2.256  2.117  1.021  1.532  0.841  1.349  1.111  2.608  0.991  1.051  1.643  0.822  1.671  2.091  1.564  1.649  1.545  1.821  
 
 TRMT1L  81627  1  185087217  185126230  1q25.2  1.242  1.942  0.532  0.926  1.914  0.742  1.441  1.147  0.796  1.387  1.867  0.615  1.814  1.78  1.203  0.812  1.307  1.669  2.322  0.789  1.439  1.032  1.203  0.797  1.246  1.58  0.707  1.306  0.896  0.537  1.641  1.633  0.977  0.719  0.935  1.718  1.687  1.044  1.861  1.575  1.353  1.148  1.94  1.036  0.747  1.442  1.089  0.498  0.895  1.605  0.702  1.029  0.912  0.994  1.291  1.339  1.16  0.944  1.046  1.768  
 
 SWT1  54823  1  185126191  185260913  1q25  0.614  0.675  0.22  0.216  0.333  0.151  0.387  0.466  0.305  0.511  0.4  0.159  0.476  1.015  0.269  0.35  0.441  0.759  0.938  0.187  0.298  0.39  0.81  0.278  0.282  0.753  0.454  0.428  0.418  0.425  0.573  0.402  0.379  0.345  0.15  0.24  0.229  0.319  0.404  0.24  0.148  0.311  0.518  0.5  0.249  0.48  0.407  0.272  0.191  0.814  0.12  0.263  0.234  0.149  0.482  0.439  0.467  0.293  0.367  0.218  
 
 IVNS1ABP  10625  1  185265521  185286461  1q25.1-q31.1  2.561  3.007  1.709  3.002  2.07  2.132  2.581  3.019  2.053  3.018  2.891  1.639  2.732  2.828  2.792  2.621  2.81  3.572  4.224  2.171  2.459  3.015  1.599  1.422  2.624  4.765  3.158  2.657  3.762  2.82  4.481  2.787  2.446  1.574  1.654  2.909  2.751  1.38  1.966  2.768  2.155  1.908  2.68  3.319  3.606  3.807  2.183  1.71  3.219  3.238  1.155  1.41  1.903  1.84  2.743  3.593  2.323  1.488  2.621  2.161  
 
 GS1-279B7.1  100288079  1  185292978  185304171  1q25.3  0  0  0  0  0  0  0  0  0  0  0  0  0  0.121  0  0  0  0  0  0  0  0  0  0  0  0  0  0  0  0.074  0  0  0  0  0  0  0  0  0  0  0  0  0  0  0  0  0  0  0  0  0  0  0  0  0  0  0  0  0  0  
 
 LINC01350  101929093  1  185527511  185597620  -  0  0  0  0  0  0  0  0  0  0  0  0  0  0  0  0  0  0  0  0  0  0  0  0  0  0  0.137  0  0  0  0  0  0  0  0  0  0  0  0  0  0  0  0  0  0  0  0  0  0  0  0  0.521  0  0  0  0  0  0  0  0  
 
 HMCN1  83872  1  185703682  186160085  1q25.3-q31.1  0.493  0.023  1.086  0.124  0.117  0.068  0.156  2.395  0.051  3.102  0.557  0.007  0.022  0.095  0.013  0.009  0.011  0.019  0.005  0.011  0.024  0.041  0.15  0.01  0.065  2.819  2.644  1.679  1.636  2.508  1.943  2.755  2.847  1.171  0.008  0.015  0.087  0.026  0.024  0.094  0.043  0.104  0.318  0.027  0.183  0.057  0.022  0.011  0.025  0.03  0.053  0.106  0.011  0.022  0.039  0.092  0.096  0.012  0  1.236  
 
 PRG4  10216  1  186265404  186283694  1q25-q31  0.405  0.16  0.238  0.354  0.454  0.085  0.269  0.127  0.212  0.254  0.204  0.09  0.157  0.456  0.184  0.244  0.258  0.16  0.478  0.906  0  0.689  0.638  0.198  0.134  0.145  0.307  0.122  0.168  0.209  0.189  0.192  0.077  0.288  0.24  0.161  0.163  0.069  0.687  0.221  0.104  0.222  0.321  0.108  0  0.093  0.65  0.129  0  0.232  0.084  0.089  0.21  0.092  0.689  0.208  0.085  0.048  0.198  0.051  
 
 TPR  7175  1  186280785  186344457  1q25  3.144  4.118  1.526  1.995  3.171  1.744  2.692  2.888  1.72  3.105  3.309  1.574  2.022  3.95  2.619  2.474  2.451  3.746  3.912  3.227  3.064  2.88  1.542  2.12  2.241  2.874  1.981  2.494  2.156  2.207  2.825  2.918  2.433  1.114  2.082  3.51  2.974  2.412  2.72  3.636  2.952  1.802  3.191  3.386  1.748  2.875  2.397  2.135  2.501  3.559  1.59  2.014  2.507  1.686  3.272  2.974  2.898  2.14  2.97  3.211  
 
 C1orf27  54953  1  186344889  186390503  1q25  1.935  2.592  0.668  0.947  2.662  0.933  1.507  1.461  1.292  1.638  1.986  1.586  1.515  2.051  1.164  1.801  1.791  3.414  2.181  1.466  1.838  1.505  1.602  1.325  1.46  2.076  0.713  1.095  0.975  0.72  1.754  1.814  1.064  0.912  1.031  2.153  1.477  1.413  2.099  1.381  1.789  1.107  1.686  1.625  0.994  1.785  1.159  0.886  1.328  1.876  0.763  0.881  1.097  1.185  2.252  2.047  1.67  1.109  2.01  1.477  
 
 OCLM  10896  1  186369703  186370587  1q25  0.718  0.299  0  0  0.787  0.402  0.214  0.484  0  0.53  0.666  0  1.26  0.975  0.572  0.354  0.318  0.265  0  0.152  0.399  0.422  0  0.255  0.227  0.977  0.091  0.392  0.304  0.569  0.265  0.388  0.31  0  0.242  0.556  0.746  0.431  0.307  0.303  0.75  0.219  0.49  0.466  0  0.586  0.059  0  0.524  0.243  0.262  0.407  0.458  0.202  0.684  0.622  0.576  0.385  0.624  0.263  
 
 LOC102724919  102724919  1  186404292  186439423  1q31.1  0  0  0  0  0  0  0  0.245  0  0.202  0  0  0  0.092  0  0  0  0  0.232  0  0  0  0  0  0  0  0  0  0  0  0  0  0  0  0  0  0  0  0  0  0  0  0  0  0  0  0.414  0  0  0  0  0  0  0  0  0  0  0  0  0  
 
 PDC  5132  1  186412697  186430240  1q25.2  0  0  0  0  0  0  0  0.065  0  0.21  0.13  0  0  0.165  0  0  0  0  0  0  0  0.052  0.245  0  0  0  0.118  0  0  0  0  0  0  0.207  0  0.21  0  0.203  0.078  0  0  0  0  0  0  0.195  0.174  0.137  0  0  0  0  0  0.06  0  0  0  0  0  0  
 
 PTGS2  5743  1  186640943  186649559  1q25.2-q25.3  0  0  0.637  0.106  0.027  0  0  0.579  0.035  0.149  0.134  0  0.078  0.102  0  0.265  0.233  0.045  0  0.024  0  0.051  0.157  0  0.465  0  0.275  0  0.212  0.038  0  0.307  0  0.09  1.068  3.642  0.623  0  0.043  0.245  1.294  0.484  0.017  0  0.04  0.021  0.18  0  0  1.209  1.003  0.264  0  0.097  0.046  0.044  0.435  0  0.09  0.105  
 
 PACERR  103752588  1  186649785  186650578  1q31.1  0  0  0  0  0  0  0  0  0.193  0.188  0  0  0  0  0  0.206  0  0.263  0  0.217  0  0.186  0  0  0  0  0.264  0  0  0.084  0  0  0  0.358  0.479  0.99  0  0  0.06  0  0.237  0.147  0  0  0  0  0.086  0  0  0  0  0  0  0  0  0  0.184  0  0  0  
 
 PLA2G4A  5321  1  186798031  186958113  1q25  0  0.042  0  0.455  0.06  0  0.178  1.075  0.084  1.64  1.528  0  0.914  1.647  0.064  1.438  2.547  0.088  0  1.538  0  0.047  1.815  1.893  0.647  0.619  1.458  0.064  1.23  1.239  1.356  1.989  0.275  0.195  1.01  3.15  0.617  0.129  0.05  0.389  1.793  1.349  0.019  0  0.203  0.046  0.06  0.204  0  0.925  0.59  0.096  0  0.162  0.062  0.207  0.05  0.144  0.693  0.638  
 
 LINC01036  104169671  1  187061973  187329376  1q31.1  0  0  0  0  0  0  0  0  0  0  0  0  0  0  0  0  0  0  0  0  0  0  0  0  0  0  0.045  0  0  0  0  0  0  0  0  0  0  0  0  0  0  0  0  0  0  0  0.066  0  0  0  0  0  0.119  0  0.103  0  0  0  0  0  
 
 BRINP3  339479  1  190066796  190446759  1q31.1  0  0  0.14  0  0.076  0  2.129  0  0.049  0.029  0.364  0  0  0.024  0  0  0  0  0  0  0.143  0.087  0.198  0  0.04  0.029  0.091  0.065  0  0.079  0  0  0  0.083  0  0  0  0  0.054  1.977  0  0.146  0.235  0  0  0.033  0.047  0  0  0  0  0.316  0.023  0.019  0.021  0  0  0  0  0  
 
 LINC01351  101929120  1  190447389  190450524  -  0  0  0  0  0.404  0  0.266  0  0  0  0.153  0  0  0.286  0  0  0  0  0  0  0  0  0  0  0  0  0.163  0  0  0.08  0  0  0  0  0  0  0  0  0  0.844  0  0.09  0  0  0  0  0.064  0  0  0.076  3.013  0.17  0  0  0  0  0  0  0  0  
 
 LOC440704  440704  1  190594019  190770788  1q31.1-q31.2  0  0  0  0  0  0  0  0  0.064  0  0  0  0  0  0  0  0  0  0  0  0  0  0.266  0  0  0  0.035  0  0  0.019  0  0  0  0.143  0  0  0  0  0  0  0  0  0.026  0  0  0  0.046  0  0  0  0.042  0  0  0  0  0  0  0  0  0  
 
 RGS18  64407  1  192127591  192154945  1q31.2  0  0  0  0  0  0  0  0  0  0  0  0  0  0  0  0  0  0  1.514  1.148  0  0  0  0.099  0  0  0.035  0  0  0.021  0  0  0  0.097  0  0  0  0  0.041  0  0  0  0  0  0  0  0.023  0  0  0  0  0  0  0  0  0  0  0  0  0  
 
 RGS21  431704  1  192286121  192336414  1q31.1  0  0  0  0  0  0  0  0  0  0  0  0  0  0  0  0  0  0  0  0  0  0.029  0  0  0  0  0.03  0  0  0.019  0  0  0  0  0  0  0  0  0  0  0  0  0  0  0  0  0  0  0  0  0  0  0  0  0  0  0  0  0  0  
 
 RGS1  5996  1  192544856  192549159  1q31  0  0  0  0  0  0  0  0  0  0  0.07  0  0  0  0  0  0  0  0.914  0  0  0.338  0.254  0.268  0  2.653  0.083  2.04  1.829  0  0.994  0.145  0  0.174  0  0  0  0  0.072  0  0  0  0  0  0  0  0  0  0  0  0  0.206  0  0  0  0  0  0  0  0  
 
 RGS13  6003  1  192605267  192629440  1q31.2  0  0  0  0  0.074  0  0.067  0  0  0  0  0  0  0  0.141  0  0  0  0.125  0  0  0.047  0  0  0.128  0.091  0.09  0.074  0  0.098  0  0  0  0.127  0  0  0.071  0  0.037  0  0  0  0.031  0  0  0  0.063  0  0  0.092  0.088  0  0.083  0.054  0  0  0  0  0.116  0  
 
 RGS2  5997  1  192778168  192781407  1q31  0  1.255  0.549  1.017  0.234  0.615  2.17  1.413  0.786  0.747  1.885  0  1.753  4.005  0.249  3.222  4.155  1.846  1.481  0.599  0.354  1.556  0.358  0.525  2.985  0.121  0.41  2.354  0.822  0.549  0.871  2.604  0.238  0.582  1.63  4.107  1.324  1.307  0.85  1.383  1.687  4.745  0.878  0.668  0.166  0.568  6.144  0.605  0.305  2.59  5.565  0.924  2.189  0.791  2.55  2.542  1.409  2.054  1.096  0.724  
 
 LINC01032  102724954  1  192904873  192917387  1q31.2  0  0  0  0  0  0  0  0  0  0  0.408  0  0  0.651  0  0  0  0.338  0  0  0  0  0  0  0  0  0  0  0  0  0  0  0  0  0  0  0  0  0.345  0  0  0  0  0  0  0  0.203  0  0  0  0  0  0  0  0  0  0  0  0  0  
 
 UCHL5  51377  1  192981495  193029237  1q32  2.496  4.249  1.461  2.053  2.263  1.852  2.249  1.895  1.465  2.311  3.191  2.192  3.057  3.385  2.249  2.494  2.701  4.376  4.527  2.349  3.576  2.134  2.215  2.621  2.385  2.509  1.874  2.079  2.255  1.804  2.964  3.232  2.408  1.477  1.77  2.709  2.681  2.05  1.905  2.466  2.656  2.428  2.905  2.467  1.829  2.574  1.704  1.796  2.276  3.555  1.692  1.915  2.29  2.131  2.414  2.618  2.662  1.917  2.872  2.702  
 
 TROVE2  6738  1  193028551  193060906  1q31  3.063  4.453  1.607  2.266  3.413  1.677  2.854  2.294  2.757  3.169  3.455  1.453  2.449  4.043  2.174  2.262  2.371  4.234  3.35  3.617  3.161  2.971  2.214  2.318  2.796  2.978  2.27  2.423  2.058  1.431  3.328  3.715  2.451  1.542  1.799  3.127  2.549  2.264  3.324  2.603  2.547  2.235  3.321  2.086  1.63  2.398  3.221  1.948  2.108  3.234  1.791  1.665  2.231  1.518  2.735  2.978  2.628  2.137  2.824  2.862  
 
 GLRX2  51022  1  193065594  193075244  1q31.2  2.523  5.159  2.18  3.085  3.627  3.207  2.973  1.875  1.943  2.802  3.247  3.002  2.199  3.359  1.349  2.788  2.997  5.175  3.863  3.322  3.511  2.488  2.973  2.631  2.676  3.165  1.885  1.724  2.027  0.839  3.63  3.275  2.062  2.143  2.621  2.849  2.329  1.571  3.72  1.896  2.25  2.884  3.708  1.604  1.537  2.856  2.856  1.814  2.166  4.097  1.661  1.718  2.23  2.832  2.887  2.886  2.407  3.286  2.716  2.22  
 
 CDC73  79577  1  193091087  193223942  1q25  1.471  2.153  0.992  1.123  2.17  1.109  1.437  1.532  1.026  1.683  2.191  1.137  1.748  2.111  1.35  1.504  1.89  1.884  2.538  1.223  1.82  1.573  0.958  1.109  1.55  1.773  0.739  1.265  1.149  0.797  1.565  1.747  0.936  0.539  0.891  2.173  1.839  0.888  1.793  1.868  2.037  1.358  1.735  1.761  0.908  1.549  1.016  1.282  1.612  2.753  0.859  1.182  1.239  1.262  1.739  1.761  1.829  1.476  1.945  2.083  
 
 B3GALT2  8707  1  193147859  193155743  1q31  0.223  0.107  0.141  0  0.259  0.091  0.083  0.163  0.062  1.139  0.205  0.041  0.218  0.29  0.159  0.15  0.142  0.093  0.058  0.102  0.079  0.201  0.291  0.065  0.077  0.323  0.201  0.946  0.079  0.131  0.118  0.148  0.135  0.16  0  0.175  0.232  0.139  0.067  0.044  0.231  0.206  0.21  0.07  0.111  0.19  0.051  0.144  0.093  0.145  0.044  0.223  0.194  0.069  0.126  0.085  0.283  0.111  0.143  0.059  
 
 LINC01031  101929184  1  193273874  193335083  1q31.2  0  0  0  0  0  0  0  0.12  0  0  0  0  0  0.384  0  0.119  0  0  0  0  0  0.097  0  0  0  0  0.095  0.114  0  0.103  0  0  0  0  0  0  0  0.248  0  0  0  0  0.08  0  0  0  0  0  0  0  0  0  0.211  0  0.091  0  0  0  0  0  
 
 KCNT2  343450  1  196194909  196577561  1q31.3  0  0  0.076  0.087  0.016  0  0.062  0.291  0.04  0  0.153  0  0  0.009  0  0  0  0  0  0  0  0.091  0.112  0  0.035  0  0.04  0  0  0.048  0  0  0  0.075  0.029  0.05  0.109  0  0.014  0.112  0  0.188  0.013  0  0.085  0  0.048  0.031  0  0.144  0.072  0.119  0  0.041  0.151  0.422  0.025  0.094  0  0  
 
 CFH  3075  1  196621007  196716634  1q32  0  0.135  2.797  1.594  0.071  0.226  3.345  2.043  0.152  1.114  0.369  0  0.063  0.05  0  3.337  0  0  0.307  0  2.189  0.197  0.168  0.37  0  0.121  0.041  0.676  0  0.081  0  0.175  0  0.133  0.522  1.864  0.733  0.071  0.368  0.961  1.012  1.286  0.029  0  0.092  0.11  1.933  0  0  0.037  0.157  0.182  0.389  1.905  0.025  2.286  0.532  0  0  0.349  
 
 CFHR3  10878  1  196743929  196763203  1q32  0.151  0  0.433  0.207  0.582  0  0.169  0  0  0  0  0  0  0  0  0.276  0  0.261  0.109  0  0  0  0  0  0  0  0.026  0  0  0.077  0  0  0  0.156  0.174  0  0  0  0  0  0.162  0.099  0  0  0  0  0.225  0  0  0  0  0.106  0  0.166  0  0.151  0  0  0  0  
 
 CFHR1  3078  1  196788860  196801319  1q32  0  0  1.286  0.699  0.085  0  1.591  0.402  0  0.245  0.239  0  0  0.201  0  0.828  0  0  0.184  0  0.448  0.147  0  0  0  0  0  0.253  0  0  0  0.184  0  0  0.369  0.43  0.199  0  0.272  0.177  0.163  0.571  0  0  0  0  0.993  0  0  0  0  0  0.091  1.065  0  0.968  0  0  0  0  
 
 CFHR4  10877  1  196857143  196887843  1q32  0  0  0.147  0  0.227  0  0  0  0  0.072  0.082  0  0  0  0  0  0  0  0  0  0  0.038  0  0.092  0.226  0  0  0.345  0.169  0.024  0  0  0  0  0.185  0  0  0  0  0  0  0.059  0  0  0  0  0  0  0  0  0  0  0  0  0  0  0  0  0  0  
 
 CFHR5  81494  1  196946666  196978803  1q31.3  0  0  0  0  0  0  0  0  0.058  0  0  0  0  0  0  0  0  0  0  0  0  0.027  0.179  0  0  0  0.033  0  0  0.027  0  0  0  0.142  0  0  0  0  0  0  0  0.222  0  0  0  0  0.017  0  0  0  0  0.096  0  0  0  0  0  0  0  0  
 
 F13B  2165  1  197008320  197036397  1q31-q32.1  0  0  0  0  0  0  0  0  0  0  0  0  0  0  0  0  0  0  0.063  0  0  0.052  0  0  0  0  0.026  0  0  0.026  0  0  0  0  0  0  0  0  0  0  0  0  0  0  0  0  0  0  0  0  0  0  0  0.049  0  0  0  0  0  0  
 
 ASPM  259266  1  197053256  197115824  1q31  2.861  4.323  1.407  1.557  2.659  2.394  2.021  2.334  0.561  3.026  3.315  1.234  2.165  2.841  2.537  2.192  2.072  4.163  3.871  1.983  2.37  2.337  0.969  1.559  2.079  2.02  1.829  1.868  2.049  1.345  2.331  2.31  2.044  1.234  2.006  2.755  2.647  3.191  2.477  2.588  2.628  2.313  2.194  2.817  1.516  2.219  1.283  2.187  2.371  2.902  1.425  1.761  2.506  2.616  3.151  2.597  3.036  2.113  2.986  2.358  
 
 ZBTB41  360023  1  197122813  197169672  1q31.3  1.142  1.051  0.523  0.706  1.369  0.36  0.712  0.903  0.489  1.067  1.231  0.233  1.138  1.231  0.691  0.653  1.033  0.72  0.863  0.463  0.361  0.412  0.361  0.238  0.746  1.189  0.543  0.644  0.76  0.425  1.026  0.846  0.484  0.409  0.345  1.589  0.643  0.984  1.603  0.869  1.072  1.423  0.963  0.79  0.376  0.805  0.207  0.227  0.444  0.582  0.563  0.556  0.537  1.172  0.924  1.151  0.747  0.414  0.717  1.11  
 
 CRB1  23418  1  197170591  197447585  1q31-q32.1  0  0  0.11  0.11  0  0  0  0.04  0.095  0  0.036  0  0  0.02  0  0  0  0  0.039  0.034  0  0.038  0.285  0  0  0  0.043  0.06  0.079  0.036  0.045  0  0  0.137  0  0.863  0  0  0.058  0  0.038  0.485  0.018  0  0  0  0.039  0  0  0  0  0.187  0  0.027  0.036  0  0  0  0  0  
 
 DENND1B  163486  1  197473878  197744623  1q31.3  1.18  1.086  0.563  0.62  0.943  0.403  0.805  1.128  0.659  1.097  1.269  0.493  0.813  1.129  0.804  1.253  1.065  1.143  2.041  0.894  0.627  1.585  1.321  0.872  0.546  0.276  0.562  0.186  0.382  0.458  0.414  0.588  0.467  0.392  0.415  1.901  0.574  0.805  1.059  0.51  1.343  0.931  0.264  0.666  0.723  0.947  0.228  0.171  1.183  0.767  0.602  0.725  0.666  0.415  0.881  0.798  0.915  0.321  0.523  1.027  
 
 C1orf53  388722  1  197871681  197876497  1q31.3  1.786  2.677  1.192  1.065  1.614  0.702  1.275  1.358  1.494  1.873  0.692  0.235  0.564  1.167  0.466  1.052  1.245  2.739  1.954  1.231  0  1.278  0.901  0.721  1.067  0.6  0.443  0.478  0.563  0.211  1.416  1.45  0.384  0.551  1.381  2.11  0.388  1.221  1.649  1.199  2.119  2.211  0.093  1.767  1.09  2.071  1.898  0.515  0  1.896  0.361  1.62  0.45  0.718  2.068  1.217  0.437  0.699  0.797  0.497  
 
 LHX9  56956  1  197881634  197899273  1q31.1  0  0  0.404  0.263  0.039  0.318  0.062  0.118  0.16  1.1  0.155  0  0  0.053  0.19  0.064  0.194  0.32  0  0.053  0  0.144  0.363  0  0  0  0.086  0  0  0.101  0  0.078  0  0.172  0.113  2.56  0  0  0.046  0  0  0.519  0.036  0  0.166  0.1  0.03  0.109  0  0.288  0.034  0.337  0  0.064  0.043  0.096  0  0.116  0  0  
 
 NEK7  140609  1  198126107  198291548  1q31.3  1.907  4.188  3.254  2.62  2.726  2.676  1.928  2.679  1.713  2.786  2.423  1.213  2.29  2.308  1.678  2.342  3.12  2.311  3.695  2.205  1.155  2.128  1.395  0.972  2.641  2.164  1.163  1.494  1.592  0.875  2.362  2.518  1.636  1.236  0.99  3.2  2.358  2.208  4.386  1.597  2.642  2.137  1.802  1.987  1.451  2.362  1.906  1.282  1.885  3.026  1.526  1.079  1.39  1.406  1.688  2.762  2.41  2.828  2.009  2.868  
 
 ATP6V1G3  127124  1  198492351  198510075  1q31.3  0  0  0  0  0  0  0  0  0  0  0  0  0  0  0  0  0  0  0  0  0  0  0  0  0  0  0  0  0  0.051  0  0  0  0  0  0  0  0  0  0  0  0  0  0  0  0  0  0  0  0  0  0  0  0  0  0  0  0  0  0  
 
 PTPRC  5788  1  198608097  198726605  1q31-q32  0  0  0.25  0  0.047  0  0.15  0  0.178  0.076  0  0  0  0.045  0  0  0  0  5.722  2.495  2.069  4.063  0.33  0  0  0  0.114  0.041  0  0.14  0  0.087  0  0.175  0  0  0  0  0.044  0  0  0.232  0.042  0  0  0.042  0.069  0  0  0.126  0  0.313  0  0.141  0  0  0  0  0  0  
 
 MIR181A1HG  100131234  1  198777131  198906558  1q32.1  0.505  1.499  0.395  0  0.425  0.679  0.23  0.377  0.316  0.662  0.674  0  0  0.349  0  0.951  0.257  1.43  1.485  1.309  0  2.44  0  0  0  0.256  0.124  0.401  0  0.133  0.605  0.998  0.381  0  0.314  0.979  0.472  0.713  0  0  1.083  0.176  0  0  0.67  0.751  0.438  0.369  0.717  0.215  0  0  0.613  0.281  0.891  0.524  0.335  0.396  0.375  0.376  
 
 LINC01222  102800316  1  198961717  198988093  1q32.1  0  0  0  0  0  0  0  0  0  0  0  0  0  0  0  0  0  0  0.274  0.154  0  0.808  0  0  0  0  0  0  0  0.061  0  0  0  0  0  0  0  0  0  0  0  0  0  0  0  0  0  0  0  0  0  0  0  0  0  0  0  0  0  0  
 
 LINC01221  104266961  1  198985261  199045864  1q32.1  0  0  0  0  0  0  0  0  0  0  0  0  0  0  0  0  0  0  1.225  0  0  2.848  0  0  0  0  0  0  0  0  0  0  0  0  0  0  0  0  0  0  0  0  0  0  0  0  0  0  0  0  0  0  0  0  0  0  0  0  0  0  
 
 NR5A2  2494  1  199996729  200146550  1q32.1  0.358  0  0.173  0.696  0.414  0  0.04  0.079  0.129  0.106  0.053  0.022  0.62  0.359  0.082  0.109  0.059  0.05  0  0.033  0  0.054  0.293  0  0  0.162  0.069  0  0.107  0.414  0.207  0.135  0  0.186  0.844  0.265  0.105  0.065  0.057  0.881  0.525  0.081  0.556  0.11  0  0.031  0.136  0  0  0.046  0.061  0.289  0.101  0.24  0.121  0.814  0.034  0.287  0  0  
 
 LINC00862  554279  1  200311671  200342920  1q32.1  0  0.091  0.131  0  0.074  0  0  1.238  0.086  0  0  0  0  0  0  0  0  0  0  0  0  0.047  0.341  0  0.1  0.122  0.053  0  0.075  0.035  0.143  0  0  0.125  0.157  0.298  0.128  0  0.101  0.142  0  0  0.044  0  0  0  0.042  0  0  0.096  0  0  0.242  0.68  0  0  1.258  0  0  0  
 
 ZNF281  23528  1  200374074  200379186  1q32.1  2.451  3.49  2.554  3.774  3.165  1.574  2.508  4.079  2.318  2.643  3.115  1.199  1.951  3.202  1.949  2.066  1.753  3.039  3.102  2.838  2.094  2.338  1.813  1.516  2.784  2.69  1.658  1.431  1.829  1.593  2.511  2.714  1.657  1.384  2.435  3.071  2.809  2.524  2.915  3.249  3.116  1.73  3.027  2.546  1.424  2.079  2.485  1.979  2.158  2.939  3.009  1.562  2.439  2.707  2.647  3.03  3.374  2.402  3.086  2.768  
 
 KIF14  9928  1  200520624  200589862  1q32.1  2.042  2.41  0.849  1.131  1.074  1.168  1.233  1.678  0.299  1.634  2.234  0.589  0.934  2.089  1.003  1.077  1.333  1.87  2.694  1.299  1.657  1.314  0.788  0.868  1.43  1.527  0.979  0.95  0.995  0.776  1.231  1.358  1.138  0.649  1.106  1.681  1.82  2.049  1.494  1.538  1.731  1.364  1.158  1.602  0.844  1.285  0.823  1.188  1.107  1.726  2.079  1.11  1.328  1.054  2.035  1.655  1.764  1.198  1.881  1.558  
 
 DDX59  83479  1  200613164  200639126  1q32.1  2.507  2.629  1.205  2.035  2.441  1.032  1.877  1.681  1.695  1.784  1.921  0.98  1.349  2.089  1.204  1.325  1.509  1.944  2.589  1.064  0.76  1.122  1.799  1.333  1.132  1.94  1.737  1.144  1.322  1.512  2.022  1.867  1.649  1.186  1.214  1.765  1.767  1.812  1.811  2.279  2.085  1.484  2.412  1.404  0.532  1.556  1.068  0.703  1.272  1.826  2.352  0.859  0.932  1.117  1.609  1.462  1.219  1.396  1.474  1.306  
 
 LOC101929224  101929224  1  200638634  200663378  -  0  0  0.243  0  0  0  0.183  0  0.548  0.25  0.137  0  0  0.524  0  0  0  0  0.218  0.256  0  0.246  1.142  0  0  0  0.093  0  0  0.057  0  0.206  0  0  0  0  0  0.506  0.257  0  0  0.145  0  0  0  0.355  0  0  0  0  0.165  0  0  0.236  0  0  0  0  0  0  
 
 CAMSAP2  23271  1  200708685  200829835  1q32.1  2.098  2.497  1.321  1.641  2.168  1.459  2.041  2.382  1.727  2.381  2.627  0.696  1.298  1.995  1.653  1.269  1.229  2.014  0.064  0.144  0.322  0.159  0.665  0.704  2.028  2.374  1.266  1.711  1.394  1.099  1.829  2.037  1.705  1.019  1.205  2.456  2.123  1.987  2.296  2.117  1.955  1.479  2.623  2.017  1.138  1.87  1.33  1.391  1.796  2.02  3.034  1.072  1.538  1.748  1.888  2.291  1.981  1.485  1.728  2.385  
 
 GPR25  2848  1  200842082  200843306  1q32.1  0  0.067  0.199  0  0.146  0.279  0.219  0.139  0.212  0.142  0.096  0.196  0  0.109  0  0.124  0.193  0.447  0.072  0.277  0  0.763  0.649  0  0  0  0.234  0.049  0  0.209  0  0  0  0  0.12  0.235  0.123  0  0.263  0  0  1.225  0.102  0  0  0.102  0.089  0.161  0  0  0  2.111  0  0.306  0.206  0.129  0  0  0  0  
 
 C1orf106  55765  1  200860626  200884864  1q32.1  0.246  0.064  0.545  0.404  2.024  0.384  0.316  0.518  0.201  0.419  0.925  2.92  2.965  0.832  2.668  2.634  2.442  0.596  0.025  1.401  0.088  0.069  1.542  1.758  0.083  0.153  0.109  0.068  0  0.104  0.581  0.073  0.099  0.274  1.965  2.342  1.406  0.116  0.083  0.544  2.833  0.153  1.837  3.155  1.527  2.608  0.942  0.419  1.752  1.14  1.889  0.468  0.703  1.703  1.561  2.16  0.69  0.068  1.55  1.887  
 
 KIF21B  23046  1  200938513  200992828  1q32.1  0.038  0.088  0.182  0.054  0.078  0.097  0.462  0.904  0.238  0.517  0.259  1.21  0.71  0.727  0.118  0.51  0.238  0.328  3.233  1.856  1.507  2.275  2.597  2.026  0.743  0.092  0.155  0.07  0.071  0.69  0.48  0.173  0  0.134  0.489  0.268  1.783  2.014  0.162  0.274  0.122  0.35  0.61  0.183  0.259  0.423  0.66  0.511  0.305  0.496  0.05  0.67  0.022  0.476  0.277  0.27  0.107  0.084  0.385  0.468  
 
 CACNA1S  779  1  201008639  201081694  1q32  0  0.022  0.095  0.095  0.028  0.072  0.024  0.03  0.124  0.047  0.017  0.043  0  0.053  0.045  0.032  0.027  0.105  0  0.043  0  0.098  0.231  0  0.032  0.019  0.111  0.051  0.022  0.078  0  0  0.033  0.086  0.058  0  0.039  0  0.063  0.032  0.028  0.203  0.051  0  0.03  0.025  0.027  0.028  0  0.047  0.024  0.472  0.293  0.245  0.827  0.052  0.112  0.039  0.255  0.038  
 
 ASCL5  647219  1  201083080  201084500  1q32.1  0.192  0  0.294  0  0.114  0.178  0.101  0.185  0.174  0.25  0.107  0.414  0.367  0.199  0.168  0.179  0.098  0.381  0.078  0.188  0  0.332  0.296  0  0  0.108  0.19  0.131  0  0.217  0  0  0  0.225  0.247  0.341  0.083  0  0.156  0  0.143  1.23  0.123  0.188  0.262  0.234  0.073  0  0  0.09  0.108  1.089  0.099  0.432  0.568  0.23  0.329  0.116  0.288  0  
 
 TMEM9  252839  1  201103898  201140710  -  5.479  3.124  4.398  5.499  5.416  3.152  5.658  5.14  5.212  5.447  5.026  4.405  3.964  4.647  4.537  4.547  5.131  3.205  5.048  3.36  3.638  3.541  3.333  3.059  3.858  4.22  4.691  4.072  4.321  4.443  4.23  4.789  4.045  2.997  3.748  5.813  5.007  5.328  4.212  4.924  3.946  5.306  5.11  4.17  3.911  4.498  4.951  3.589  3.75  5.571  5.996  3.491  5.237  4.618  5.798  5.203  5.848  4.568  5.29  4.713  
 
 IGFN1  91156  1  201159952  201198080  1q32.1  0.03  0.018  0.387  1.7  0.022  0.049  0.677  1.249  0.127  0.39  0.08  0.052  0.02  0.037  0.022  0.027  0.019  0.046  0.02  0.029  0.02  0.083  0.405  0.013  0.509  0.143  0.168  2.276  0.13  0.081  0.057  0.049  0.025  0.154  0.056  0.215  2.444  2.992  0.104  0.053  0.045  0.343  0.053  0.032  0.041  0.115  0.018  1.673  0.555  0.841  0.07  0.259  4.378  3.68  5.773  2.029  4.04  3.401  5.233  2.89  
 
 PKP1  5317  1  201252579  201302121  1q32  1.178  0  0.091  0.112  0.034  0  0.017  0.037  0.121  0.05  0.033  2.939  0  0.048  0.088  0.039  0.034  0.125  0.123  0.045  0  0.192  0.428  0  0  0.241  0.081  0.012  0.035  0.081  0  0  0.025  0.178  0.03  0.143  0  0.449  0.072  0  0.044  0.205  0.058  0  0.082  0.224  0.051  0.284  0  0.301  5.72  0.576  0.016  0.227  0.07  0.045  3.131  0  0  0.064  
 
 TNNT2  7139  1  201328135  201346828  1q32  0  0  0  0  0.103  0  0  0  0  0  0  0.14  0  0.063  0.645  0  0  0.247  0  0  0  0.065  0.337  0  0  0  0.071  0  0  0.07  0  0  0  0  0  0  0  0  0.09  0  0  0  0  0.98  0  0.549  0.481  0.941  0  2.395  0  0.477  0  0  0  0  0  0  0  0  
 
 LAD1  3898  1  201349965  201368669  1q25.1-q32.3  3.79  0.027  0.135  0.815  2.925  0.082  0.076  0.056  0.22  0.165  0.085  5.755  5.211  2.826  3.719  4.528  4.579  0.507  0.086  0.232  0.12  0.133  0.465  0.12  0.062  0.519  0.143  0.602  0.189  0.108  0.045  0.185  0  0.213  0.671  0.645  0.352  0  0.148  0.181  4.261  0.113  0.065  3.402  3.8  4.264  3.222  0  0.197  0.459  1.711  1.327  0.081  0.486  0.414  0.196  0.127  0.344  1.939  5.238  
 
 TNNI1  7135  1  201372894  201390874  1q31.3  0.234  0  0.124  0  0.286  0.018  0  0.021  0.136  0.046  0.029  0.235  0.597  0.327  0.214  0.399  0.178  0.383  0.038  0.032  0  0.102  0.503  0  0.043  0.076  0.088  0.055  0.051  0.068  0  0  0  0.198  0  0  0.03  0.032  0.048  0  0.201  0.353  0.051  0.119  0.151  0.269  0.244  0  0.032  0.056  0.087  0.194  0.056  0.059  0.064  0.04  0.15  0  0.078  0.691  
 
 PHLDA3  23612  1  201433411  201438299  1q31  2.494  0.157  3.108  4.374  3.829  1.967  3.309  4.537  3.873  4.708  3.325  1.982  0.455  3.015  1.818  3.414  0.162  0.079  1.494  0.371  0.278  1.952  0.697  0.098  4.41  5.226  0.256  4.753  4.867  2.209  4.813  5.766  0  0.421  3.595  3.034  2.759  4.79  5.456  2.366  3.855  3.901  1.864  4.676  2.209  4.49  0.568  2.429  2.275  3.791  2.211  2.82  3.1  4.263  4.927  4.203  3.207  1.36  4.341  4.6  
 
 CSRP1  1465  1  201452657  201476387  1q32  5.792  3.241  6.815  5.103  5.827  4.569  5.478  7.119  6.049  6.066  6.104  5.342  4.943  4.845  4.539  4.918  3.25  0.901  5.514  4.438  2.739  4.075  4.006  5.325  5.458  6.16  5.618  5.733  5.727  3.78  5.216  5.62  6.619  4.752  5.675  6.336  6.001  6.646  6.549  5.193  3.214  5.725  3.794  5.4  4.18  7.72  5.143  5.466  4.819  5.95  4.212  3.993  5.072  3.611  3.892  2.633  5.653  5.347  4.924  6.146  
 
 RPS10P7  376693  1  201489031  201489720  1q32.1  4.322  2.464  3.335  3.233  4.189  2.404  2.739  1.924  3.619  2.746  2.395  2.581  2.371  3.279  2.296  4.569  3.005  0.786  3.784  3.998  1.973  4.404  4.293  3.176  2.983  1.792  3.286  1.984  2.609  3.673  2.774  2.241  2.34  2.889  2.708  1.849  2.541  2.28  3.359  1.922  1.499  2.617  3.75  2.127  2.08  2.232  3.848  2.728  1.371  4.45  1.729  2.014  3.229  1.724  3.813  2.383  1.69  2.55  2.486  1.452  
 
 NAV1  89796  1  201617449  201796102  1q32.3  0.752  1.099  2.314  1.957  0.168  2.686  3.173  4.203  1.501  4.441  2.902  1.767  1.267  2.212  0.472  0.114  0.095  0.959  0.133  0.119  1.227  0.263  0.746  1.235  3.212  3.386  2.715  2.476  3.127  1.174  2.961  2.604  2.235  0.784  0.776  2.108  2.5  2.909  4.039  1.35  0.326  0.766  2.363  0.695  0.636  0.672  1.538  1.315  1.374  1.055  1.52  1.367  2.927  1.726  2.989  2.777  3.449  2.491  2.623  3.035  
 
 IPO9-AS1  100873949  1  201657383  201798687  -  1.071  0.117  0.649  0.752  0.802  1.465  0.813  0.775  0.675  0.861  0.66  0.496  0.853  0.528  0.912  1.023  0.614  0.216  0.96  0.425  0.92  0.951  0.596  0.87  0.378  0.479  0.753  0.605  0.786  0.665  0.496  0.333  0.664  0.434  1.261  0.656  0.457  0.478  0.471  0.85  0.67  0.938  0.62  0.733  0.425  0.792  0.252  0.737  0.684  0.538  0.195  0.712  1.304  1.152  1.093  0.866  0.717  0.399  0.526  0.362  
 
 MIR5191  100847050  1  201688635  201688755  -  0  0  0  0  0  0  0  0  0  2.548  0  0  0  0  0  0  0  0  0  0  0  0  0  0  0  0  0  0  0  0  0  0  0  0  0  0  0  0  0  0  0  0  0  0  0  0  0  0  0  0  0  0  0  0  0  0  0  0  0  0  
 
 MIR1231  100302158  1  201777738  201777830  -  0  0  0  0  0  0  0  0  0  0  0  0  0  16.674  0  0  0  0  0  0  0  0  0  0  0  0  0  0  0  0  0  0  0  0  0  0  0  0  0  0  0  0  0  0  0  0  0  0  0  0  0  0  0  0  0  0  0  0  0  0  
 
 IPO9  55705  1  201798287  201853422  1q32.1  2.418  2.538  1.813  2.525  2.833  1.764  2.952  3.534  2.213  3.047  3.268  1.873  2.689  3.063  2.715  2.6  2.245  1.424  3.271  2.289  2.344  2.557  1.409  1.775  2.349  2.725  2.368  2.322  2.32  2.685  2.71  2.484  1.954  0.748  2.025  3.26  3.108  3.072  2.659  3.547  2.759  2.148  3.315  2.649  2.388  2.699  2.637  2.163  2.539  2.961  1.462  2.048  2.569  1.893  3.07  2.771  2.73  2.43  2.915  2.762  
 
 SHISA4  149345  1  201857796  201861715  1q32.1  0.704  0.296  3.944  2.639  3.281  1.924  4.291  4.12  4.118  3.77  4.02  0.324  0.663  2.568  1.103  0.811  0.228  0.097  0.153  0.22  0  0.288  1.115  0.811  1.656  4.423  2.996  2.681  3.13  2.977  3.161  4.342  3.058  2.06  2.029  3.193  2.439  3.769  2.931  2.834  1.631  2.821  3.01  1.612  1.715  3.356  0.469  2.048  1.374  3.594  1.987  2.207  2.191  2.843  3.301  2.925  2.213  4.291  2.512  2.741  
 
 LMOD1  25802  1  201865583  201915716  1q32  0.063  0.071  3.358  0.12  0.219  0.149  0.369  0.212  0.278  0.476  0.34  0.055  0.05  0.18  0.095  0  0  0.045  0  0.056  0.057  0.136  0.682  0.137  0.041  0.22  0.301  0.137  0.047  0.074  0.037  0.259  0.074  0.289  0.125  0.05  0.096  0.108  0.421  0.055  0.092  0.293  0.08  0.213  0.081  2.245  0.485  0.149  0  0.157  0.065  0.511  0.045  0.118  0.288  0.053  0.054  0.15  0.057  0  
 
 TIMM17A  10440  1  201924618  201939789  1q32.1  3.264  4.969  3.122  3.618  4.326  2.847  4.216  3.899  3.415  3.922  4.761  3.444  3.776  5.338  2.989  3.458  4.048  4.249  5.309  4.738  4.424  3.784  3.561  3.065  3.705  3.671  2.746  2.74  3.096  2.957  4.338  4.448  3.419  2.706  3.02  4.511  4.005  3.36  4.867  3.614  3.493  3.855  4.126  3.819  3.084  4.161  2.989  2.677  2.928  5.056  2.348  2.738  3.367  3.14  4.043  3.839  3.41  3.755  4.233  3.78  
 
 RNPEP  6051  1  201951765  201975275  1q32  3.941  2.353  3.304  3.639  4.963  2.793  4.3  4.133  3.828  4.128  4.922  3.138  4.097  4.655  4.001  4.573  4.446  1.29  4.237  4.639  3.669  2.346  3.804  3.001  2.759  4.292  4.136  3.445  3.668  3.763  4.617  4.484  3.535  2.33  3.254  4.883  3.926  3.711  3.998  4.044  4.313  4.782  4.424  3.959  3.353  3.973  3.684  2.808  3.828  4.761  2.47  3.479  3.593  3.515  4.504  4.255  3.664  3.859  4.027  3.597  
 
 MIR6740  102465443  1  201972251  201972364  -  0  0  0  0  0  0  0  0  0  0  0  0  0  0  0  0  0  0  0  0  0  0  0  0  0  0  0  0  0  0  0  0  0  0  0  0  0  0  0  0  0  0  0  0  0  3.674  0  0  0  0  0  0  0  0  0  0  0  0  0  0  
 
 ELF3  1999  1  201979689  201986315  1q32.2  3.304  0.034  0.457  1.2  2.895  0.071  0.108  0.023  2.099  0.096  0.909  4.696  4.899  3.662  3.138  4.694  4.602  1.593  0.024  0.04  0.119  0.091  0.648  0  0.097  0  0.105  0.021  0.045  0.136  0.04  0  0  0.287  3.737  4.138  0.679  0.085  0.351  0.297  5.389  0.629  0.038  2.928  2.755  4.09  3.172  0  2.637  0.122  1.884  1.734  3.106  3.471  3.116  4.15  2.84  0.107  3.232  2.668  
 
 GPR37L1  9283  1  202092028  202098634  1q32.1  0.924  0.076  0.173  0.132  1.381  0.108  0  0.036  0.233  0.41  0.145  0  0.3  0.065  0.122  0.072  0.096  0.118  0.046  0.096  0  0.24  0.728  0  0  0.068  0.148  0.036  0  0.091  0  0  0  0.245  0.114  0.194  0.044  0  0.221  0.065  0.308  0.156  0.062  0.054  0.181  0.083  0.078  0  0  0.053  0.053  0.582  0.199  0.348  0.064  0.195  0.351  0  0.259  0  
 
 ARL8A  127829  1  202102531  202113871  1q32.1  2.727  1.746  2.481  3.957  3.138  2.576  3.056  3.165  4.116  3.446  3.581  1.128  2.542  2.308  2.714  1.929  1.745  1.226  3.339  1.456  1.485  1.96  3.072  1.981  1.578  3.442  2.641  2.236  2.28  2.352  2.848  2.638  2.567  2.014  2.108  3.228  2.851  3.353  2.913  2.33  2.543  3.467  3.371  2.394  2.075  2.694  0.383  2.185  2.573  2.894  1.569  2.225  2.203  2.869  3.072  3.141  2.375  2.042  3.012  2.957  
 
 PTPN7  5778  1  202116140  202130716  1q32.1  0  0  0.146  0  0.075  0  0.05  0.044  0.154  0.101  0.057  0  0  0.067  0  0.95  0  0.078  6.567  4.463  3.873  2.925  3.102  3.883  0  0.302  0.231  0.277  0  0.094  0.073  0.09  0  0.214  0.11  0.139  1.12  1.445  0.087  0  0  0.33  0.156  0.059  0  0.067  0.04  0.235  0  3.797  0  0.35  0.029  0.063  0.052  0.063  0.079  0  0  0  
 
 PTPRVP  148713  1  202137178  202158577  1q32.1  0.074  0.025  0.079  0  0.064  0.063  0  0.024  0.15  0.059  0.042  0  0  0.071  0  0.056  0  0.057  0.327  0.037  0.849  0.102  0.472  0.207  0  0.071  0.091  0.032  0.036  0.095  0  0  0  0.13  0.042  0.064  0  0  0.022  0.065  0.047  0.399  0.183  0  0  0.075  0.031  0  0  0.069  0.038  0.41  0.031  0.099  0.089  0  0  0  0  0  
 
 LGR6  59352  1  202163117  202288889  1q32.1  0  0.052  0.134  0  0.152  0.128  0.153  0.145  0.297  0.241  0.179  0.142  0.458  0.087  0.636  0.092  0.226  0.526  0.031  0.148  0  0.139  0.354  0.046  0  0.036  0.168  0.04  0  0.199  0  0  0  0.245  0.113  1.833  0.17  0  0.147  0  0.082  0.228  0.236  0.093  0.09  0.265  0.21  0  0.104  0  0.027  0.775  0.043  0.181  0.15  0.199  0.033  0  0  0  
 
 UBE2T  29089  1  202300784  202311094  1q32.1  5.689  7.685  2.838  3.854  5.306  4.142  5.509  4.1  1.423  4.556  5.131  4.312  4.231  5.78  3.438  4.689  5.315  8.376  6.92  4.719  4.433  5.389  3.267  3.472  3.809  4.376  3.779  3.642  3.55  3.562  4.836  4.43  4.018  2.396  4.215  4.739  4.379  4.728  4.53  4.324  4.932  4.473  5.484  4.246  3.271  4.314  4.577  3.56  3.728  5.776  2.984  3.222  3.637  3.376  5.275  4.38  3.584  4.464  4.381  3.77  
 
 PPP1R12B  4660  1  202317829  202557697  1q32.1  1.851  0.689  0.554  0.626  1.028  0.403  0.678  0.774  0.679  1.319  0.85  1.32  0.704  1.063  0.802  1.399  0.798  0.475  1.035  0.886  0.86  0.808  0.925  0.837  0.325  0.773  0.596  0.789  1.118  0.737  0.909  0.557  0.903  0.756  0.868  1.809  0.608  1.484  0.457  0.578  1.096  0.613  0.854  0.775  0.481  1.605  0.448  0.871  0.8  0.708  0.465  0.797  0.531  0.496  0.824  0.575  0.829  0.397  0.678  0.434  
 
 SYT2  127833  1  202559724  202679551  1q32.1  0.352  0.108  0.166  0.073  0.176  0.043  0.089  0.1  0.114  0.357  0.132  0.133  0.059  0.238  0.166  0.229  0.038  0.143  0.084  0.173  0.069  0.157  0.506  0.229  0.022  0.127  0.234  0.098  0.122  0.202  0.083  0.09  0.069  0.198  0.173  0.365  0.105  0.58  0.077  0.08  0.127  0.252  0.17  0.162  0.116  0.299  0.125  0.087  0.085  0.053  0.041  0.264  0.088  0.113  0.102  0.064  0.161  0.039  0.119  0  
 
 KDM5B  10765  1  202696531  202777549  1q32.1  2.849  2.191  2.081  2.613  3.071  0.935  1.821  2.926  2.413  2.691  2.628  0.95  1.542  1.874  0.759  1.5  1.426  0.786  2.585  2.056  2.561  1.688  0.365  0.062  1.626  1.668  1.097  2.011  1.972  2.439  2.025  2.728  1.324  0.923  1.29  3.134  1.974  2.411  1.746  2.361  2.517  0.861  3.169  2.78  2.196  2.672  0.785  1.399  1.498  2.225  2.398  1.082  1.276  1.772  1.967  2.664  2.044  1.101  1.67  2.528  
 
 PCAT6  100506696  1  202780073  202781041  -  0.896  0.656  2.314  1.672  3.095  1.328  0.477  1.315  1.375  1.835  1.232  0  0.694  0.651  0  0.381  0.653  0.297  0.865  0.294  0.592  0.896  0.766  0.203  0.524  0.98  0.336  1.174  1.218  1.959  2.347  1.978  0.83  1.344  0.807  2.846  0.9  0.978  0.563  0.506  1.412  1.551  2.512  0.789  1.317  2.409  0  0.459  0  2.086  1.557  1.469  0.334  1.15  0.925  2.035  0.993  0  0.323  0.959  
 
 MGAT4EP  641515  1  202789384  202796341  1q31  0  0.056  0.178  0  0.115  0  0  0.028  0.09  0.036  0  0  0  0.036  0  0  0  0.061  0  0.053  0.072  0.072  0.256  0  0  0.034  0.078  0.067  0  0.081  0  0  0  0.256  0  0  0  0  0.039  0  0.051  0.302  0.034  0  0  0.103  0.042  0  0  0  0.034  0.407  0  0.064  0.028  0  0  0  0  0  
 
 LOC148709  148709  1  202830881  202844369  1q32.1  1.642  0.161  0.339  0.192  1.11  0.339  0.333  0.25  0.225  0.432  0.402  0.281  0.373  0.627  0.73  0.256  0.486  0.122  0.209  0.194  0  0.251  0.601  0.047  0  0.359  0.78  0.243  0.323  0.802  0.636  0.242  0.156  0.232  0.181  0.198  0.26  0.193  0.141  0.234  0.433  0.411  1.498  0.066  0.401  0.604  0.059  0.233  0.068  0.477  0.6  0.392  0.407  0.196  0.216  0.252  0.09  0.746  0.19  0.111  
 
 RABIF  5877  1  202847409  202858385  1q32.1  3.17  3.187  1.295  2.157  2.619  1.691  2.293  1.743  1.581  1.96  2.011  1.402  1.479  1.751  1.257  1.674  1.535  1.785  2.948  2.229  1.528  2.375  1.524  1.387  1.443  2.133  1.71  1.426  1.387  1.36  2.163  1.719  1.515  0.981  1.715  2.514  1.376  1.96  2.542  1.669  1.609  1.664  2.836  1.375  0.925  1.823  1.761  1.529  1.151  2.86  1.869  1.078  1.182  1.15  2.273  1.804  1.391  1.685  1.913  1.488  
 
 KLHL12  59349  1  202860223  202897772  1q32.1  3.335  2.763  1.217  1.544  2.702  1.321  2.53  2.315  1.935  2.42  2.178  1.815  2.96  3.208  2.009  1.93  2.774  2.953  3.602  2.558  2.182  2.344  2.111  1.828  1.295  2.506  1.854  1.781  1.779  2.473  2.576  2.483  1.687  1.079  1.72  2.874  2.997  2.963  2.474  2.897  2.285  1.216  3.524  2.161  1.263  1.803  2.707  1.15  1.604  2.54  2.368  1.438  2.099  1.487  2.891  2.63  1.831  1.708  2.156  2.408  
 
 ADIPOR1  51094  1  202909952  202927700  1q32.1  5.8  4.328  3.742  3.881  5.633  3.713  5.475  4.98  4.919  5.138  5.146  4.208  4.685  5.517  4.369  4.468  4.898  4.041  5.368  3.605  4.38  4.208  4.251  4.25  4.433  5.198  4.237  4.72  4.95  5.39  5.698  5.467  4.628  3.644  3.612  5.792  5.308  5.504  5.01  4.946  5.402  4.374  4.798  4.254  3.553  5.026  3.126  3.86  4.001  5.066  4.975  3.614  4.324  4.082  4.571  5.129  4.068  4.584  4.821  4.465  
 
 CYB5R1  51706  1  202931000  202936404  1q32.1  4.011  4.023  2.885  2.363  4.172  2.126  3.693  3.253  3.599  2.414  3.818  2.305  2.109  3.099  1.979  2.426  2.238  2.914  3.302  2.748  2.449  2.198  2.83  2.526  2.132  3.915  3.373  2.423  3.34  3.208  4.143  3.804  2.909  1.935  1.668  3.433  2.883  3.484  4.391  2.496  2.827  2.399  3.13  2.133  1.799  3.474  3.131  2.243  1.295  4.395  2.775  1.852  2.136  2.273  2.954  2.833  2.451  3.388  2.045  2.87  
 
 LOC100506747  100506747  1  202955579  202976393  -  0.23  0  0.111  0  0.087  0  0  0  0.076  0  0  0  0.174  0.043  0  0  0.061  0.093  0.05  0.159  0  0.07  0.292  0  0  0  0.279  0.041  0  0.051  0  0  0.088  0.192  0.071  0  0  0  0.043  0  0.114  0.377  0.026  0  0  0  0.058  0  0  0  0.057  0.199  0  0.139  0  0  0.092  0  0.118  0  
 
 TMEM183A  92703  1  202976533  202993197  1q32.1  1.471  1.003  1.281  2.012  1.052  0.934  0.949  0.623  2.137  1.209  1.252  0.894  1.057  1.564  0.813  1.041  1.495  0.978  2.215  1.736  1.205  1.454  1.389  0.673  0.713  0.972  1.201  0.54  0.393  0.323  1.134  0.68  0.498  0.526  1.049  1.064  1.016  0.602  1.535  0.969  0.904  1.237  0.791  1.328  0.526  0.807  0.062  0.249  0.647  1.635  1.018  0.312  0.657  1.037  1.266  1.315  0.668  0.596  1.083  1.114  
 
 TMEM183B  653659  1  202976535  202992668  3q25.1  5.563  2.893  2.941  3.338  4.281  2.508  4.444  3.894  3.598  3.981  4.102  3.339  3.828  4.325  3.446  3.387  3.851  1.223  5.234  3.682  3.844  3.759  3.396  3.298  3.122  2.881  3.669  2.666  3.064  4.159  3.941  4.118  2.981  1.458  2.241  4.505  3.691  3.316  2.924  3.916  3.485  3.184  4.374  3.312  2.848  4.122  2.473  2.872  3.051  4.251  3.429  2.467  3.572  2.69  3.41  4.023  3.273  3.173  4.358  3.425  
 
 PPFIA4  8497  1  202995648  203047864  1q32.1  0.633  0.135  0.61  0.249  0.15  0.468  0.112  1.019  0.367  2.409  1.122  0.065  0.111  0.572  0.15  0.066  0.505  0.077  0.103  0.194  0.06  0.27  0.447  0.089  0.032  0.509  1.082  0.461  1.068  1.315  0.53  1.029  0.622  0.22  0.089  0.284  0.065  0.751  0.161  0.381  0.118  0.382  0.629  0.845  0.178  0.802  0.409  0.339  0.083  0.834  0.193  1.018  0.458  0.385  0.488  0.594  2.065  0.032  0.396  0.066  
 
 MYOG  4656  1  203052256  203055166  1q31-q41  0  0.051  0  0  0.057  0.151  0.088  0.084  0.154  0.141  0  0.306  0  0.027  0  0.051  0  0  0  0.068  0  0.072  0.412  0  0  0  0.314  0.046  0  0.238  0  0  0  0.148  0  0  0  0  0.036  0  0  0.229  0.062  0  0.117  0.117  0.514  0  0  0  0  0.39  0  0.18  0.114  0  0.069  0  0  0  
 
 ADORA1  134  1  203096835  203136533  1q32.1  0.321  0.052  0.226  0  0.433  0.596  0.585  0.615  1.835  2.234  1.554  0  0.056  0.599  0.744  0.061  0.057  0.155  0  0.041  0  0.368  0.718  0.254  0.098  0  0.878  0.081  0.228  0.173  0.076  0.298  0.921  0.588  0.172  0.674  0.926  2.535  0.337  1.29  0.068  0.785  0.163  2.708  2.896  2.589  0.131  1.08  0.07  1.752  0  1.329  2.765  3.407  3.488  3.133  1.517  0.828  1.958  1.834  
 
 MYBPH  4608  1  203136938  203144942  1q32.1  0  0.053  0.147  0  0  0  0  0.145  0.131  4.456  0.045  0  0  0.228  0  0  0  0.158  0  0.408  0  0.149  0.369  0.139  0  0  0.074  0  0  0.066  0  0  0  0  0  0  0  2.958  0.03  0  0  0.208  0  0  0.125  0.193  0.022  0  0  0.051  0  0.392  0.124  0.256  0.224  0.082  0.188  0  0  0  
 
 CHI3L1  1116  1  203148058  203155922  1q32.1  0  0  0  0.79  0.065  0.093  0.21  1.22  1.638  10.658  0.211  0.079  0  0.047  0  0  0  0.302  0.064  4.647  0  1.413  0.748  0.739  0  0.35  0.137  0.183  0  0.144  0  0  0  0.158  0  0.079  0  0.792  0.774  0.136  0  0.036  0.059  0.098  0.086  2.832  0.105  0  0  0  0  0.412  0.073  0.179  0.121  0  0.24  0  0  0  
 
 CHIT1  1118  1  203185206  203198860  1q32.1  0  0  0.077  0  0  0  0  0  0.117  0  0  0.081  0  0.12  0  0  0  0.067  0  0  0  0.047  0.399  0  0  0  0.065  0  0  0.067  0  0  0  0.163  0  0  0  0  0.033  0  0  0.345  0.049  0  0  0  0  0  0  0  0  0.316  0.042  0.18  0  0  0  0  0  0  
 
 LOC100506775  100506775  1  203256279  203257924  -  0  0  0  0  0  0  0  0  0  0.097  0  0  0  0  0  0  0  0  0.345  0  0  0.314  1.184  0.199  0  0  0.122  0  0  0  0  0  0  0  0  0  0  0  0  0  0  0  0.067  0  0  0  0  0  0  0  0  0  0  0  0  0  0  0  0  0  
 
 LINC01136  730227  1  203267885  203274453  1q32.1  0  0  0.158  0  0.225  0  0  0  0.139  0.047  0  0.051  0  0.038  0  0.044  0  0.117  0.11  0.039  0  0.145  0.535  0.093  0  0  0.067  0  0  0.116  0  0  0  0.21  0.059  0.087  0  0  0.043  0.089  0  0.045  0.02  0  0  0.095  0.045  0  0  0  0  0.153  0  0.052  0.053  0  0.06  0.169  0  0  
 
 BTG2  7832  1  203274663  203278729  1q32  1.325  0.459  0.371  0.29  0.863  0.36  0.391  1.07  4.35  0.669  1.868  0.678  1.469  2.305  1.367  0.766  1.096  1.693  2.351  0.271  1.374  1.396  4.611  3.792  1.089  1.789  0.165  0.259  0.472  0.432  0.697  2.105  0.313  0.366  0.38  1.244  0.22  0.455  2.895  1.461  0.358  1.561  1.756  2.803  1.003  3.154  0.156  0.229  0.285  0.787  0.217  0.95  0.492  1.713  2.338  1.34  0.245  0.2  1.175  2.94  
 
 FMOD  2331  1  203309748  203320557  1q32  0  0.113  0.122  0  0.655  0.056  0  0.031  0.142  0.338  0.083  0.061  0  0.048  0  0  0  0.093  0  0.051  0  0.128  0.603  0  0  0.843  0.076  0.641  0  0.058  0  5.536  0  0.228  0  0.305  0.677  0.315  0.033  0  0  0.589  0.047  0  0  0.058  0.034  0  0  0  0.028  0.211  0.025  0.066  0.068  0.065  0  0  0  0.53  
 
 PRELP  5549  1  203444882  203460479  1q32  0.05  0.135  0.181  0  0.074  0.028  0.035  0.05  0.23  2.844  0.038  0.031  0  0.031  0  0.025  0.026  0.105  0  0.052  0  0.102  0.523  0.032  0.031  1.6  0.095  0.471  0.146  0.387  0.546  0.406  0.024  0.286  0.03  0.036  0.093  0.07  0.113  0.032  0.021  0.102  0.043  0.075  0.072  0.045  0.043  0.062  0.05  0.146  0.088  0.412  0.048  0.084  0.064  0.065  0  0.039  0.041  0.074  
 
 OPTC  26254  1  203463270  203478077  1q32.1  0  0  0.197  0  0  0  0  0  0  0  0.054  0  0  0.029  0  0  0  0  0  0  0  0.098  0.417  0  0  0  0.073  0  0  0.044  0.079  0  0  0.123  0  0  0  0.106  0.079  0  0  0.049  0.046  0  0  0.127  0.058  0  0  0  0  0.329  0  0  0  0  0  0  0  0.122  
 
 ATP2B4  493  1  203595914  203713209  1q32.1  2.219  2.806  3.074  3.734  1.541  1.882  3.33  3.008  1.944  4.256  3.699  0.987  0.759  2.694  0.914  0.614  0.256  0.365  3.502  2.504  1.763  2.991  1.202  1.136  0.452  3.85  2.361  2.63  3.395  2.958  2.815  2.764  2.331  1.6  0.816  3.707  3.137  4.549  2.52  2.691  1.851  1.259  1.294  2.574  1.83  3.175  1.516  1.871  2.054  1.775  3.657  2.267  3.74  2.766  1.776  2.445  3.948  1.336  3.603  4.345  
 
 SNORA77  677843  1  203698708  203698833  1q32.1  0  0  0  0  0  0  0  0  0  0  0  0  0  2.226  0  0  0  0  0  0  0  0  0  0  0  0  0  0  0  0  0  0  0  0  0  0  0  0  0  0  0  5.359  4.928  0  0  0  1.44  0  0  0  0  0  1.794  0  0  0  0  0  0  0  
 
 LINC00260  84719  1  203699704  203700979  1q32.1  0.695  0.399  0.819  0.78  0.248  0.446  0.606  0.833  0.22  1.449  1.23  0.096  0  0.628  0.157  0.189  0  0.102  2.211  0.475  0.439  1.056  0.753  0.108  0  1.367  1.038  0.931  1.024  0.407  1.234  0.805  0.53  0.28  0  0.871  1.145  2.023  0.436  0.503  0.532  0.84  0.087  0.482  0.345  1.402  0.575  0.661  0  0.389  1.1  0.828  1.243  0.228  0.515  0.21  1.525  0.384  1.156  0.754  
 
 LAX1  54900  1  203734283  203745480  1q32.1  0  0  0.176  0  0  0.093  0.043  0  0.149  0.035  0.041  0  0  0.023  0  0.038  0  0.055  1.035  0.141  0  1.066  0.549  0.142  0  0  0.08  0  0  0.051  0.068  0  0  0.218  0  0  0  0  0.024  0  0  0.313  0.034  0  0  0  0.281  0  0  0  0  0.205  0  0.038  0  0  0  0  0  0  
 
 ZC3H11A  9877  1  203764750  203823256  1q32.1  3.851  4.383  2.074  2.779  3.75  2.175  3.002  3.888  2.999  3.58  3.474  2.052  2.796  3.581  3.155  3.104  3.089  3.817  4.56  2.951  2.783  3.106  2.54  1.953  3.195  3.409  2.134  2.501  2.837  2.389  3.156  3.091  2.422  1.553  1.989  4.196  3.108  3.459  3.422  3.405  3.423  2.433  3.063  3.06  2.181  2.837  3.243  2.297  2.135  3.877  3.199  2.447  2.419  2.22  3.463  3.233  2.988  2.767  3.499  3.061  
 
 ZBED6  100381270  1  203766650  203769590  1q32.1  3.532  2.733  1.063  1.032  2.543  0.944  1.491  2.826  1.08  2.129  1.911  1.01  1.875  2.31  2.235  1.65  0.757  1.341  0.655  0.957  1.483  0.822  0.615  0.847  2.001  2.395  1.338  1.547  1.053  1.134  1.291  1.107  1.086  0.492  0.886  3.324  1.906  2.672  1.904  1.583  2.068  0.991  1.046  2.379  0.907  1.537  0.242  1.426  1.325  0.5  1.734  1.62  1.267  1.271  2.462  2.337  1.953  1.366  1.919  1.819  
 
 SNRPE  6635  1  203830712  203840280  1q32  5.026  7.501  2.419  2.825  4.998  4.684  5.021  4.043  2.288  3.78  4.792  4.362  3.674  5.393  2.801  4.207  4.249  8.828  6.401  6.383  4.955  5.486  2.944  4.667  4.626  3.838  3.058  2.835  3.022  3.262  4.461  4.365  3.198  2.449  4.474  4.378  3.962  4.283  5.495  3.82  4.093  3.675  4.615  4.739  3.677  4.29  4.545  3.174  3.557  5.551  3.546  2.784  4.078  3.905  5.007  5.06  3.544  5.341  4.546  4.121  
 
 LINC00303  284573  1  204001574  204010392  1q32.1  0  0  0.114  0  0  0  0  0  0.137  0  0.093  0  0  0.041  0  0  0  0  0  0  0  0.043  0.342  0.123  0  0  0.082  0  0  0.069  0  0  0  0.225  0  0  0  0  0.035  0  0  0.039  0.05  0  0  0  0.05  0  0  0  0  0  0  0.077  0.063  0  0  0  0  0  
 
 SOX13  9580  1  204042245  204096871  1q32  2.859  0.861  0.75  1.88  3.932  1.427  1.54  1.024  3.001  3.124  3.577  1.848  1.987  1.789  2.118  2.513  3.177  0.94  0.044  0.057  0.143  0.268  0.52  1.995  0.425  4.11  3.833  2.943  4.256  2.044  3.278  2.401  2.852  1.586  0.937  2.116  3.102  2.268  0.985  2.435  3.864  2.355  1.636  3.595  1.471  2.663  1.819  1.438  2.224  1.907  2.454  1.519  1.241  0.872  0.8  1.741  1.435  1.9  1.186  1.091  
 
 ETNK2  55224  1  204100188  204121310  1q32.1  3.645  0.727  1.282  1.255  3.606  1.519  2.589  2.374  3.345  1.661  2.641  0.135  0.462  2.097  0.212  0.833  0.131  0.151  0.082  0.055  0  1.274  0.421  0.171  0.11  2.753  2.655  1.533  1.895  2.436  2.967  1.698  1.028  0.598  1.015  2.143  2.837  2.683  1.245  2.695  1.965  1.045  1.865  1.749  1.366  2.01  0.757  1.8  2.073  3.377  1.475  1.304  0.908  0.387  1.945  1.994  0.627  1.913  0.328  2.514  
 
 LOC101929441  101929441  1  204110535  204112137  1q32.1  0.213  0  0  0  0.266  0.308  0  0.105  0.144  0.111  0.108  0  0.123  0.167  0  0  0  0  0.091  0  0  0.051  0.337  0  0  0.122  0.691  0.065  0  0.101  0.154  0  0  0.196  0  0.096  0  0  0.083  0  0.135  0  0.05  0.632  0  0.11  0.038  0  0  0  0.101  0.262  0  0.064  0.114  0  0  0  0  0  
 
 REN  5972  1  204123943  204135465  1q32  0.174  0.117  0.161  0  0.294  0  0.058  0  0  0  0.067  0  0  0.253  0  3.471  0  0.135  0  0  0.345  0.071  0.282  0.123  0.119  0  0.065  0.09  0  0.049  0  0  0  0  0  0.811  0.157  0  0.108  0  0.718  0.177  0.033  0  0  0.114  1.245  0  0  0  0.183  0.396  0  0  0  0  0  0.202  0  0  
 
 KISS1  3814  1  204159468  204165619  1q32  0.36  2.09  0.638  0  0.446  3.497  0  0  0.376  0.184  0.169  0.557  0.553  0.289  0  0.654  0.584  0  0.566  0.244  0  0.354  0  0  4.532  0  0.222  0.97  0  0.194  0  0  0  0  0.265  0.227  2.544  0  1.487  0  0.301  0.598  0.088  0  0.277  0.241  1.312  3.306  0  6.577  0.179  1.225  0  0.506  0.413  0  0  0.353  0  1.076  
 
 GOLT1A  127845  1  204167287  204183220  1q32.1  2.514  0.131  0.227  0  2.31  0.294  0.538  0.159  1.019  0.112  0.681  2.692  2.819  1.842  0.433  3.626  2.927  0.55  0  0  0  0.097  0.765  0  0  0.147  0.169  0.124  0  0.145  0  0.236  0  0  0.55  1.792  0.469  0.291  1.322  0.894  2.284  0.508  0.05  0.498  0.875  1.136  0.5  0.52  0.349  1.522  0.403  1.639  0.195  0.428  1.605  0.827  0  0  0.457  0.751  
 
 PLEKHA6  22874  1  204187978  204329057  1q32.1  2.575  0.816  0.345  0.33  2.701  0.064  2.138  0.06  0.216  0.141  0.89  2.46  2.955  0.53  1.628  2.54  1.823  0.599  0.04  0.218  0.044  0.094  0.446  0.024  0.049  1.511  0.162  0.042  0.113  0.113  0.047  0.032  0  0.213  1.495  2.59  2.946  2.309  0.358  0.656  1.228  0.546  0.075  0.288  0.399  0.584  1.377  1.829  2.001  1.605  2.311  0.487  1.332  1.413  2.277  2.214  0.403  0.03  2.865  2.368  
 
 LINC00628  127841  1  204337557  204338847  1q32.1  0.56  0.165  0.222  0  0.535  0  0.295  0  0.149  0  0.253  0.238  0.274  0.126  0.236  0.405  0.408  0  0  0  0  0.11  0.48  0  0  0.161  0.059  0  0  0.059  0  0  0  0.244  0.134  0  0.274  0.153  0.099  0  0  0.235  0  0  0  0.114  0  0.19  0.447  0.188  0.461  0.141  0.112  0.284  0.548  0.246  0  0  0.447  0.189  
 
 PPP1R15B  84919  1  204372491  204380945  1q32.1  3.139  2.845  1.427  2.338  2.799  1.634  2.19  3.327  2.433  2.322  2.952  2.242  2.528  3.218  2.557  2.609  2.917  2.24  3.503  1.988  2.554  2.376  2.215  2.007  2.313  2.502  1.263  2.232  1.676  1.652  2.185  2.378  1.924  1.215  1.85  3.597  2.855  2.48  2.304  2.723  2.52  2.172  1.809  3.007  1.955  2.633  0.837  1.508  2.259  2.21  2.867  1.631  2.507  2.268  2.351  2.742  2.398  1.701  2.799  2.329  
 
 PIK3C2B  5287  1  204391757  204459474  1q32  2.214  0.101  0.395  0.581  1.848  0.167  0.749  0.566  0.542  0.744  0.758  2.039  2.031  1.143  2.051  2.02  1.541  0.225  1.662  0.244  0.118  0.996  0.393  0.396  0.017  2.087  0.629  0.841  0.789  1.161  0.834  0.828  0.869  0.384  0.739  2.478  0.793  0.457  0.691  1.753  1.427  0.8  1.565  1.11  1.098  1.31  0.335  0.409  0.858  0.437  1.743  0.759  0.985  0.702  0.637  0.655  0.759  0.383  1.106  0.678  
 
 MDM4  4194  1  204485506  204527248  1q32  2.599  1.99  0.66  1.439  1.879  1.022  1.081  1.603  1.177  1.375  1.631  0.551  1.25  2.49  1.816  1.742  1.183  1.527  2.49  1.612  1.788  1.744  1.132  1.323  1.24  1.898  0.994  1.485  0.822  1.486  1.469  1.383  1.295  0.714  1.214  2.248  1.494  1.497  1.356  1.2  1.352  1.244  1.403  1.969  0.977  1.15  1.142  1.004  1.205  1.524  1.848  1.059  1.067  0.7  1.458  1.34  1.313  1.562  1.804  0.752  
 
 LRRN2  10446  1  204586302  204654597  1q32.1  0.241  0  0.21  2.062  1.197  0  0.235  0.06  0.585  0.377  0.065  0.1  0  0.85  0.084  0.064  0.046  0.126  0  0.098  0  0.257  0.742  2.249  0  0.041  0.349  0.03  0  0.3  0  0.103  0  0.237  1.303  3.063  0.033  0  1.093  1.556  1.77  1.727  0.916  0.142  1.144  1.088  0.399  0.082  0.182  0.073  0.117  1.112  0.031  0.184  0.09  0.077  0.03  0.105  0  0  
 
 NFASC  23114  1  204797781  204991950  1q32.1  0  0.051  0.193  0  0.078  2.109  0.353  0.62  0.92  4.182  0.298  0.083  0.093  0.131  0.073  0.093  0.036  0.132  0.034  0.135  0  0.181  0.615  0.034  0  0.162  0.367  0.139  0.369  1.169  1.218  0.704  0.073  0.261  0.161  1.459  0.065  0  3.468  1.625  0  0.586  1.891  0.087  0.803  0.154  0.036  0  0.299  0.025  0.093  0.84  0  0.553  0.152  0.075  0.258  0  0.079  0  
 
 CNTN2  6900  1  205012339  205047171  1q32.1  0.061  0  0.119  0.073  0.048  0.029  0.057  0.04  0.127  0.047  0.104  0.048  0  0.149  0.085  0.035  0.028  0.068  0.027  0.08  0  0.11  0.394  0.028  0  0.022  0.068  0.043  0.051  0.148  0.032  0.024  0.021  0.213  0.052  0.094  0.019  0.054  0.098  0.028  0.041  0.243  0.096  0.03  0  0.069  0.027  0.035  0.057  0.036  0.065  0.379  0.022  0.171  0.089  0.036  0.041  0  0.034  0  
 
 TMEM81  388730  1  205052256  205053588  1q32.1  0.776  0.156  0.636  1.511  0.607  0.105  1.441  0.695  1.462  0.836  1.28  0.62  0.424  0.716  0.584  0.519  0.758  0  1.564  0.718  0.556  0.952  1.288  0.579  0.317  0.731  0.835  0.768  0.199  1.306  0.684  0.701  0.671  0.513  0.614  1.082  0.946  0.662  0.931  0.978  0.456  0.623  1.779  0.545  0.438  0.27  0.339  0.532  0.127  1.356  1.131  0.229  0.397  0.482  0.874  0.796  0.67  0.213  1.139  0.463  
 
 RBBP5  5929  1  205055269  205091150  1q32  2.46  2.05  1.274  2.126  2.15  1.192  2.409  1.99  2.16  2.237  2.422  2.01  1.977  2.065  1.585  1.677  1.769  1.161  2.75  2.072  2.23  1.973  2.104  1.816  1.669  2.34  1.375  1.745  1.34  1.749  2.142  2.261  1.894  1.365  1.494  2.837  2.027  1.977  1.938  2.384  1.761  1.235  2.458  1.773  1.521  1.771  1.344  1.351  1.543  2.221  2.876  1.122  1.725  1.41  2.092  2.268  1.887  1.362  2.187  1.942  
 
 DSTYK  25778  1  205111630  205180727  1q32.1  1.676  1.253  1.154  2.019  1.725  0.919  2.264  1.818  1.651  1.915  1.932  0.674  0.935  1.426  1.246  0.796  0.812  0.546  1.198  1.087  0.823  0.878  1.196  0.999  0.837  3.718  3.617  2.2  3.173  3.484  2.885  3.509  2.806  2.324  1.002  2.144  1.212  1.869  2.226  1.692  1.311  1.111  2.045  1.236  0.924  0.719  1.272  1.011  1.127  1.648  1.918  1.032  1.075  1.573  2.244  2.377  1.846  1.339  1.697  1.408  
 
 TMCC2  9911  1  205197037  205242471  1q32.1  0.237  0.19  0.629  1.03  0.354  0.709  1.483  0.725  1.37  1.12  2.635  0.105  0.104  0.478  0.433  0.106  0.191  0.123  0.124  0.149  0.21  0.415  2.038  1.942  0.119  2.297  2.255  1.789  2.359  4.424  2.788  2.021  2.284  1.867  0.439  0.853  0.808  1.02  1.47  0.968  0.725  0.736  1.998  0.272  0.633  0.604  0.588  0.68  0.356  1.373  1.37  1.235  0.349  1.484  1.284  1.15  0.677  1.067  0.485  0.52  
 
 NUAK2  81788  1  205271190  205290919  1q32.1  0.968  1.144  1.118  2.363  0.496  0.706  1.244  2.265  1.366  1.328  2.869  0.413  1.414  0.791  2.192  0.724  0.201  0.859  0.036  0.13  0.2  0.283  1.452  2.149  0  0.251  0.476  0.595  0.141  0.427  0.15  0.237  0.258  0.353  3.053  3.349  1.002  1.001  3.125  2.947  0.737  1.592  2.115  2.761  1.842  2.702  1.537  1.254  1.572  1.619  0.248  1.837  2.064  3.079  3.456  2.209  2.721  0.955  3.874  2.65  
 
 KLHDC8A  55220  1  205305192  205326218  1q32.1  0  0.057  0.135  0  0.06  0  0.096  0.037  0.426  0.181  0.352  0.127  0  0.072  0  0.045  0.062  0.137  0  0.074  0  0.158  0.437  0  0  0.426  0.19  0.046  0  0.222  0  0  0  0.271  0.2  0.089  0  0  0.07  0.126  0.045  0.579  0.067  0  0.961  0.181  0.124  0  0  0  0.066  0.633  0  0.134  0.093  0  0  0  0  0.096  
 
 LEMD1-AS1  284576  1  205342379  205356568  1q32.1  0  0  0.143  0  0  0  0  0  0.116  0.031  0.181  0  0.056  0.045  0  0  0  0.07  0  0  0  0.045  0.412  0  0  0  0.087  0  0  0.068  0  0  0  0.183  0  0  0  0  0.021  0  0.065  0.089  0.052  0  0  0.077  0.121  0  0  0  0  0.422  0  0.038  0  0  0  0  0  0  
 
 LEMD1  93273  1  205350505  205391214  1q32.1  0.536  0  0  2.951  0  0  0.473  0  1.814  0  3.782  1.589  0.898  2.406  0  0.314  0.388  0.628  0  0  0  0  0  0  0  0  1.764  0.125  0  0  0  0  0  0  0  0  0  0  0.608  0  1.907  0.429  0  0.409  0.473  1.94  0.471  0  0  0  0  0  0  0.85  0.161  0  0  0  0  0  
 
 BLACAT1  101669762  1  205404013  205425214  1q32.1  0.407  0  0.098  1.895  0.048  0  0.362  0.071  0.572  0.145  2.66  0.999  1.825  1.097  0.066  1.077  0.236  1.461  0  0.048  0.162  0.076  0.492  0  0.082  0.598  0.77  0.786  0.134  0.973  0.381  0.509  0.142  0.141  0  0.059  0.171  0.233  0.584  0  1.74  0.056  0.031  1.112  0.715  1.006  0.784  0  0.083  0.299  0.196  0.435  0.205  0.497  0.622  0.436  0  0  0.168  0.237  
 
 CDK18  5129  1  205473683  205501921  1q31-q32  3.516  0.054  1.205  0.508  2.108  0.283  0.737  1.998  0.651  3.751  1.048  1.775  2.969  2.669  2.254  3.012  2.462  0.202  0.935  0.154  0.109  1.127  1.501  1.44  0.058  2.771  2.35  2.668  1.511  2.028  2.292  0.948  2.153  1.451  0.58  0.618  1.744  1.14  0.933  1.809  1.147  1.08  1.265  0.968  1.63  1.769  2.106  1.285  0.089  1.631  1.031  1.27  2.561  2.159  3.305  1.804  3.17  0.284  2.108  2.756  
 
 LOC284578  284578  1  205523400  205525763  1q32.1  0  0  0.098  0  0  0  0  0  0.063  0.026  0  0  0  0.06  0  0.162  0.064  0  0  0.063  0  0.029  0.41  0  0  0  0.064  0  0  0.031  0  0  0  0.123  0  0  0  0  0.033  0  0  0.251  0.034  0  0  0  0  0  0  0  0.056  0.077  0  0.04  0.028  0  0  0  0  0.05  
 
 MFSD4  148808  1  205538111  205572046  1q32.1  0.14  0.024  0.124  0.1  0.24  0  0.058  0.094  0.177  0.177  0.186  0.172  0.153  0.298  0.154  0.586  0.514  0.195  0.461  0.044  0.062  0.31  0.288  0.112  0.047  0.311  0.098  0.081  0.063  0.184  0.101  0.1  0.047  0.211  0.154  0.2  0.06  0.126  0.158  0.114  0.219  0.084  0.594  0.063  0.122  0.137  0.033  0.059  0.139  0.063  0.169  0.502  0.132  0.261  0.458  0.441  0.088  0.036  0.09  0.339  
 
 ELK4  2005  1  205577070  205602000  1q32  2.923  2.862  1.603  1.612  2.584  1.882  2.419  2.664  1.614  2.796  2.599  1.328  2.026  2.649  2.057  2.279  2.419  2.24  2.628  1.843  1.438  3.298  1.891  2.049  2.248  2.521  1.915  2.421  1.93  2.235  2.014  2.134  2.255  1.4  1.723  2.918  2.413  2.157  2.695  2.224  2.284  1.653  2.348  1.937  2.808  1.603  1.744  1.77  2.633  1.611  2.484  1.769  2.247  1.627  2.787  2.85  2.372  2.801  2.768  2.296  
 
 SLC45A3  85414  1  205626980  205649630  1q32.1  0.494  0.299  0.611  0.692  0.57  0.356  1.22  1.185  0.595  0.688  1.54  3.395  1.365  1.314  1.582  2.792  1.565  0.183  0.094  0.37  0.326  0.273  0.76  0.34  1.397  1.495  1.108  0.98  0.83  1.265  1.408  1.445  1.535  0.613  0.492  1.686  2.351  2.274  0.665  1.345  1.27  0.543  1.536  0.753  0.89  0.72  1.264  0.906  0.683  1.284  1.34  0.944  0.649  0.737  1.413  0.801  0.74  0.602  0.554  1.549  
 
 NUCKS1  64710  1  205681946  205719372  1q32.1  6.233  6.198  3.7  3.971  5.198  4.135  4.509  5.14  3.898  5.307  5.437  3.147  3.926  4.956  4.055  4.079  4.204  5.466  5.772  4.571  4.241  4.398  2.806  3.092  4.153  4.22  3.345  3.407  2.692  2.756  4.193  4.197  3.226  2.145  3.967  5.051  4.645  4.158  5.166  4.377  4.976  4.546  4.367  4.597  4.62  3.342  4.45  2.744  4.644  4.221  4.519  3.555  4.475  3.368  4.611  4.752  4.293  4.346  4.643  4.482  
 
 RAB29  8934  1  205737113  205744610  1q32  1.86  2.091  1.919  3.408  2.144  1.284  2.041  2.408  2.475  2.729  1.906  2.084  2.138  1.896  1.677  1.695  2.536  1.315  2.063  1.338  1.313  1.548  1.841  1.665  2.365  2.804  1.641  1.559  1.946  2.872  2.934  2.858  1.821  1.823  0.91  1.234  2.083  2.669  2.768  1.95  1.5  0.829  1.948  1.786  1.724  1.688  0.63  1.802  2.754  3.331  0.895  1.522  1.529  1.575  3.574  2.546  1.99  1.58  2.776  2.594  
 
 SLC41A1  254428  1  205758220  205782324  1q32.1  2.378  2.841  1.988  2.187  1.849  1.683  2.575  3.109  2.22  2.884  2.584  1.128  1.889  2.697  2.052  1.478  2.045  0.573  2.086  0.434  1.711  2.278  1.03  1.179  2.015  2.284  2.274  2.477  1.9  2.099  2.046  2.47  2.441  1.223  1.331  3.356  3.155  2.84  2.536  2.601  2.004  1.82  2.635  2.183  2.503  1.882  1.426  1.924  2.164  2.62  1.904  2.355  2.591  2.543  2.815  3.148  2.4  2.269  2.853  2.47  
 
 PM20D1  148811  1  205797149  205819276  1q32.1  0.316  0.079  0.07  0  0.091  0.08  0.096  0.159  0.099  0.155  0.155  0.087  0  0.112  0.175  0  0.08  0.128  0.063  0.116  0.14  0.198  0.362  0.066  0.066  0.038  0.066  0  0.086  0.136  0  0.091  0  0.125  0.068  0.137  0.155  0  0.101  0  0.162  0.054  0.155  0.068  0.265  0.179  0.029  0.262  0.167  0.048  0.083  0.376  0.076  0.118  0.268  0.092  0.124  0.144  0.168  0.112  
 
 LOC284581  284581  1  205831206  205865215  1q32.1  1.753  0  0.679  0.53  0.885  0.984  1.137  1.377  1.163  1.016  1.375  0  0  0.846  0.691  0.456  0  0  2.408  0.045  0  1.225  0.481  0  0.853  0  0.36  0.098  0.586  0.994  0  1.156  0  0.168  0.504  0.404  0.769  0.079  1.565  0  0.503  0.646  1.641  0  0.094  1.144  0.231  0.75  0.651  2.075  0.541  0.238  0.166  0.669  2.372  0.08  1.745  1.801  1.22  0.578  
 
 SLC26A9  115019  1  205882176  205912588  1q32.1  0  0.034  0.106  0  0.022  0  0  0.036  0.113  0.094  0.33  1.167  0.162  0.024  0  0.356  0.157  0.111  0  0.093  0  0.105  0.481  0  0  0  0.039  0  0  0.048  0  0  0  0.173  0.027  0.252  0.116  0.048  0.046  0  0.505  0.292  0.042  0.18  0.125  0.057  0.248  0  0  0.033  0.019  0.242  0.078  0.171  0.052  0.094  0.123  0  0  0  
 
 FAM72A  729533  1  206138910  206155074  1q32.1  0.687  2.842  0  0.78  1.121  0  1.822  0.756  0  1.022  2.074  0.291  0.654  2.041  0.429  0.736  1.208  2.312  3.741  1.802  0  1.844  1.643  0  0.969  1.001  0.681  0.437  0.577  0.376  1.207  1.446  0.439  0.297  0  0.768  1.825  0.873  1.504  1.318  1.263  0.881  1.709  0.378  0  0  1.467  0  0  1.598  2.166  0  0.588  0.12  1.21  0.958  0.356  0.686  1.358  1.075  
 
 AVPR1B  553  1  206224282  206231482  1q32  0  0  0.279  0  0  0  0  0  0  0.035  0  0  0  0.059  0  0  0.097  0.292  0  0.156  0  0.123  0.691  0.148  0  0  0.066  0  0  0.03  0  0  0  0.235  0  0  0  0  0.046  0  0  0.23  0.038  0  0  0.726  0  0  0  0  0.163  0.644  0  0.032  0  0  0  0  0  0  
 
 C1orf186  440712  1  206238871  206288647  1q32.1  0  0  0  0  0  0  0  0  0.138  0  0  0  0  0  0  0  0  0  2.219  1.016  0  0.511  1.802  0.601  0  0  0.057  0  0  0.03  0  0  0  0.178  0  0  0  0  0.073  0  0  0  0.027  2.57  3.785  4.555  0.045  0  3.863  0  0  0.16  0.678  1.885  2.173  2.829  2.117  0  4.185  1.763  
 
 CTSE  1510  1  206317458  206332104  1q31  0  0.132  0  0  0  0  0  0  0.062  0.107  0.438  0.241  0.572  0.021  0  1.18  1.241  0.057  0.042  0.094  0  0.06  0.293  0  0  0.156  0.078  0.967  0.126  0.074  0.059  0.369  0.197  0.254  0  0  0.134  0.118  0  0  0.206  0.073  0.024  0.09  0.508  0.332  0.027  0  0  0  0  0.25  0  0.028  0.083  0.085  0  0  0.131  0  
 
 SRGAP2C  653464  1  206516199  206581301  1p11.2  0.977  1.121  0.475  0  0  0.935  0  1.198  0  0  1.335  0  0.526  0.107  0  0  0  0  0  0  0  0  0  0.361  0  0  0  2.692  1.843  0  0  0.07  1.229  0  0  1.062  2.098  0  0  0.133  0.417  0  0  0.649  0  0.791  0.233  1.447  1.471  0  1.284  0  0.445  0  0  0  1.764  0.083  0  1.086  
 
 SRGAP2  23380  1  206516199  206637783  1q32.1  3.567  2.512  1.992  2.605  2.852  2.091  3.398  3.452  2.008  3.687  3.14  1.387  1.695  2.655  2.293  1.911  1.582  1.487  2.246  2.014  1.664  1.633  2.126  2.132  2.575  4.015  3.311  3.864  3.211  2.691  3.273  3.937  2.996  1.713  1.902  2.848  3.74  3.714  3.76  3.261  2.562  1.982  3.348  2.61  1.769  1.799  2.353  1.698  2.118  2.075  2.87  2.131  2.962  2.31  3.04  3.154  3.248  2.498  3.108  2.792  
 
 IKBKE  9641  1  206643585  206670223  1q32.1  1.027  0.251  0.774  0.499  1.36  0.247  2.025  1.764  0.48  1.14  1.486  1.51  1.206  0.815  1.05  1.527  0.788  0.12  3.185  1.911  0.352  1.703  1.275  1.852  1.923  1.94  1.278  1.029  0.648  1.264  1.168  1.463  1.665  0.66  0.799  2.556  2.427  2.742  1.906  0.118  1.707  0.937  1.946  2.122  1.221  1.281  1.42  0.45  0.392  1.019  1.872  1.26  1.867  1.598  1.053  2.014  2.254  0.682  1.572  1.979  
 
 RASSF5  83593  1  206680862  206762616  1q32.1  0.674  0.938  0.438  0.145  1.283  0.193  1.089  2.249  0.568  0.238  0.811  1.6  0.049  1.283  0.471  1.423  0.126  1.251  4.989  2.873  2.168  3.583  4.467  3.677  0.317  0.65  0.281  0.305  0.394  0.337  0.119  0.609  0.124  0.327  0.479  1.832  1.69  1.338  0.628  0.507  1.501  0.585  0.233  0.415  0.148  0.48  1.232  0.269  0.178  0.924  0.843  1.046  0.858  0.561  0.219  0.351  0.737  0.317  0.222  0.098  
 
 EIF2D  1939  1  206764973  206785904  1q32.1  3.647  2.501  2.197  0  3.853  1.879  3.921  2.23  2.983  3.151  3.239  2.938  2.457  4.083  3.266  2.85  3.674  1.203  4.242  3.922  3.679  3.462  3.192  3.361  2.981  3.206  2.998  1.828  2.881  2.786  3.353  3.736  3.267  1.49  2.524  4.356  3.771  4.092  3.421  3.17  3.27  2.643  3.872  2.741  2.533  2.1  2.937  1.853  2.747  3.365  3.75  2.72  3.223  2.432  3.63  3.867  3.095  3.184  3.578  3.351  
 
 DYRK3  8444  1  206808880  206822542  1q32.1  0.603  1.745  1.291  2.502  0.592  0.718  1.738  1.951  0.874  1.651  1.444  0.064  0.409  1.468  0.378  0.102  0.193  1.079  2.696  0.434  1.894  1.545  0.365  0.223  2.418  2.453  1.452  1.22  1.891  2.002  2.169  2.526  2.981  1.462  0.526  2.672  1.504  2.032  1.871  1.588  0.206  0.793  1.588  0.148  0.492  0.335  1.279  0.822  0.74  1.266  2.088  0.661  1.247  1.944  1.639  1.926  1.577  1.408  1.205  1.522  
 
 MAPKAPK2  9261  1  206858364  206907630  1q32  3.681  2.668  3.974  4.408  4.094  3.264  4.129  4.875  4.177  4.92  3.958  2.346  3.525  3.273  3.19  2.844  3.328  0.484  4.818  3.026  3.325  3.206  2.404  2.253  3.416  4.198  3.9  3.575  3.258  3.705  3.525  3.871  4.223  2.814  2.719  5.323  4.943  5.157  3.163  3.861  3.441  3.505  3.645  3.397  3.004  3.331  2.421  2.148  3.664  3.332  4.516  3.187  4.088  3.492  4.687  4.595  4.542  3.091  4.444  4.346  
 
 IL10  3586  1  206940947  206945839  1q31-q32  0.205  0  0.126  0  0.101  0  0  0  0.134  0  0  0  0  0.037  0  0  0  0.802  0.176  0.098  0  0.116  0.454  0.305  0.112  0.125  0.274  0.131  0  0.053  0  0.134  0  0.165  0  0  0  0  0.077  0  0  0  0.049  0  0  0  0.062  0  0  0.238  0  0.166  0.082  0  0  0  0  0  0  0  
 
 IL19  29949  1  206972214  207016326  1q32.2  0  0  0.172  0  0  0  0  0  0.175  0.078  0  0  0  0.079  0  0  0  0  0  0  0  0.06  0.368  0.254  0  0  0.151  0  0  0.057  0.093  0  0  0.175  0  0  0  0  0.056  0  0  0.078  0.036  0  0  0  0  0  0  0  0  0.217  0  0  0.068  0  0  0  0  0  
 
 IL20  50604  1  207039153  207042568  1q32  1.649  0  0  0  0  0  0  0  0  0  0  0  0  0  0  0  0  0  0  0  0  0.043  0.254  0  0  0  0.059  0  0  0.04  0  0  0  0  0  0  0  0  0.074  0  0  0.267  0  0  0  0  0  0  0  0  0  0  0  0  0  0  0  0  0  0  
 
 IL24  11009  1  207070787  207077484  1q32  0.462  2.641  0  0.328  0  0  0.202  0.249  0  0.19  0  0  0  0  0  0  0  0  0  0  0  0  0.486  0  1.305  0.067  1.307  2.764  0.339  0.188  0  1.028  0.686  0.82  0  0.229  1.182  1.489  0.308  0  0  0  0.031  0  0  0  0  0  0  0  0  0.525  0  0  0  0  0.123  0.25  0  0  
 
 FCMR  9214  1  207076630  207095378  1q32.1  1.605  2.558  0.133  0  0.368  0.161  0.46  0.258  0.161  1.23  0.168  0  0.675  0.186  0.196  0.188  0.212  0  0.726  0.168  0  0.937  3.697  3.46  0  1.855  3.903  2.989  2.957  1.696  2.074  1.935  3.803  2.784  0.271  2.294  1.446  2.116  0.585  0.072  0.848  0.368  0.058  0  0.129  0.14  0.681  0.237  0  0.792  0.074  1.456  0.293  0.87  0.338  0.06  0.656  0.436  0.062  0  
 
 PIGR  5284  1  207101866  207119811  1q31-q41  0  0.027  0.134  0  0.028  0  0.022  0.019  0.135  0.015  0.015  0  0.176  0.018  0  0  0.123  0  0  0.071  0  0.112  0.456  0  0  0  0.073  0.043  0  0.051  0  0  0  0.22  0  0  0  0  0.042  0  0  0.177  0.033  0.139  0.058  0.201  0.029  0  0  0.018  0.02  0.262  0.016  0.048  0.041  0.049  0  0  0  0  
 
 FCAMR  83953  1  207131311  207143970  1q32.1  0  0  0.079  0  0  0  0  0  0.15  0  0  0  0.13  0.021  0  0  0  0  0  0.089  0  0.06  0.49  0  0  0  0.062  0  0  0.077  0  0  0  0.116  0  0  0  0  0.029  0  0  0.107  0.032  0  0  0  0.024  0  0  0  0  0.17  0.387  0.043  0.111  0  0.056  0  0  0  
 
 C1orf116  79098  1  207191865  207206101  1q32.1  0  0.098  0.145  0.06  0.056  0.039  0.044  0.069  0.246  0.042  0.091  1.983  2.43  0.488  2.462  2.709  2.163  1.231  0.029  0.053  0.457  0.07  0.384  0  0.03  0.118  0.053  0.222  0.061  0.132  0  0.078  0.035  0.272  0.638  1.962  0.466  0.353  0.112  0  3.141  0.124  0.031  1.389  1.198  1.285  0.158  0.032  2.429  0.128  2.169  1.37  1.876  2.06  2.067  1.711  0.145  0.224  1.008  0.304  
 
 YOD1  55432  1  207217193  207226325  1q32.2  0  0.819  0.338  0.54  0.682  0.645  0.439  0.679  0.597  0.815  1.144  0.589  1.3  1.35  1.356  1.017  1.136  0.594  1.407  0.95  2.585  1.034  0.504  0.29  1.143  1.311  0.381  1.155  0.802  0.72  0.896  1.054  0.496  0.398  0.643  1.878  1.247  1.148  1.063  0.829  1.292  0.909  0.601  0.982  0.711  0.587  0.174  0.365  1.079  0.858  1.875  0.877  1.239  0.737  1.209  1.425  1.21  1.352  1.532  0.938  
 
 PFKFB2  5208  1  207226619  207254368  1q31  0.592  0.685  0.255  0.454  1.492  0.587  0.465  0.408  0.546  0.678  0.634  1.755  1.588  1.106  1.358  0.901  1.248  1.32  0.294  0.223  0.871  0.584  0.533  0.135  0.448  1.713  0.816  1.121  1.063  1.365  1.018  0.976  0.936  0.748  0.499  2.268  0.888  0.923  0.487  0.659  1.764  0.142  1.036  0.652  0.803  0.913  1.024  0.247  1.079  0.911  1.712  1.102  0.856  0.649  1.694  1.135  0.488  0.743  1.136  0.864  
 
 C4BPB  725  1  207262186  207273337  1q32  0  6.146  0  0  0.531  2.921  0  0  0  0  0  1.005  0.891  0  3.554  0.944  1.553  0  0  0  0  0.106  1.433  0.796  0  0  0  0  0  0.048  0  0  0  0  0.197  0  4.566  0  2.001  0  0  0  0  0  0  0  0.604  0  0  0  0.222  0.249  1.231  0  0  0  0  3.038  0  0  
 
 C4BPA  722  1  207277606  207318317  1q32  0  0  0  0  0  0  0  0  0  0  0  0.622  0  0.018  0  0  0  0  0  0  0  0.033  0.226  0  0  0  0.035  0  0  0.047  0  0  0  0  0.068  0.771  0  0  0  0  0.048  0.622  0.034  0  0  0  0.033  0  0  0  0  0.127  0  0.033  0  0  0  0  0  0  
 
 CD55  1604  1  207494816  207534311  1q32  3.002  5.703  2.955  3.289  3.833  2.737  4.054  1.861  1.629  2.541  1.796  0  2.731  3.468  2.549  2.707  5.407  0.719  0.472  2.514  3.514  1.096  2.456  2.456  2.398  3.379  1.877  2.498  2.762  2.856  2.412  4.028  2.681  2.239  2.04  4.573  3.768  3.462  3.766  2.697  2.935  3.157  0.363  2.136  1.683  1.838  2.537  1.374  2.647  2.375  5.783  2.086  2.027  1.645  2.746  4.004  2.046  3.835  2.738  2.384  
 
 CR2  1380  1  207627644  207663240  1q32  0.108  0  0.074  0  0.024  0  0.277  0  0.066  0.031  0  0.128  0  0.526  0.758  0.021  0  0  0.361  0.082  0.158  1.493  0.912  1.672  0  0  0.471  0  0  0.276  0  0  0  0.113  0  0  0  0  0.023  0  0.072  0.277  0.04  0  0.038  0.032  0.662  0  0  0.06  0  0.241  0  0.026  0.018  0  0  0  0  0  
 
 CR1  1378  1  207669472  207815110  1q32  0  0  0.065  0  0.008  0  0.032  0  0.054  0.008  0.009  0.027  0  0.011  0  0.012  0.017  0.021  0  0.122  0.038  0.03  0.191  0  0.016  0  0.103  0.01  0  0.061  0  0  0  0.091  0  0  0  0  0.013  0  0  0.106  0.02  0  0  0.008  0.078  0  0  0  0.009  0.065  0  0.014  0  0.016  0  0  0  0  
 
 CR1L  1379  1  207818457  207897036  1q32.1  0  0.077  0.085  0  0.153  0.157  0  0  0.119  0.07  0.049  0.087  0  0  0  0.113  0  0  0.069  0  1.748  0.08  0.26  0.123  0  0  0.039  0.039  0  0.06  0  0  0  0  0  0.091  0  0  0.107  0  0  0.074  0.033  0  0  0  0  0  0  0  0  0.293  0  0.123  0  0  0.064  0  0  0  
 
 CD46  4179  1  207925382  207968861  1q32  2.454  4.868  2.291  3.278  5.185  2.374  3.488  3.083  3.371  3.371  3.622  4.07  4.102  3.969  3.456  4.44  3.964  6.18  4.671  2.658  3.118  2.402  3.015  2.581  3.022  3.467  2.299  2.709  2.435  2.444  3.01  3.402  2.35  2.004  2.427  5.514  3.624  4.063  4.01  3.119  4.265  2.994  3.42  3.614  2.312  2.867  2.8  1.427  3.236  3.921  3.463  2.185  2.875  3.179  3.991  4.002  3.58  2.996  3.884  4.016  
 
 LOC148696  148696  1  207991723  207995941  1q32.2  0.078  0.085  0.193  0  0.363  0.092  0.045  0.115  0.133  0.158  0.165  0.111  0.175  0.092  0.305  0.214  0.069  0.112  0.145  0.116  0.082  0.486  0.546  0.439  0.031  0.186  0.091  0.046  0.046  0.131  0.04  0.051  0  0.173  0.128  0.371  0.244  0.625  0.127  0.052  0.13  0.464  0.257  0.126  0.103  0.069  0.022  0  0.307  0  0.183  0.471  0.122  0.243  0.082  0.119  0.256  0.132  0.126  0.103  
 
 CD34  947  1  208059882  208084683  1q32  0  0.12  0.234  0  0.07  0.239  0.175  0.042  0.235  0.173  2.103  0.038  0  0.019  0  0  0  0  0.517  0.047  0  2.509  0.482  0.108  0.062  0.339  0.055  0.261  0  0.051  0.049  0.067  0  0.132  0  0.164  0.184  0.138  0.566  0  0  0.33  0.054  0  0.393  0.18  0.033  0  0.936  0.114  0  0.253  0.071  0.185  0.042  0.845  0.159  0.288  0  0.126  
 
 PLXNA2  5362  1  208195587  208417665  1q32.2  0.216  0.627  0.66  0.421  0.088  0.819  0.474  0.879  1.352  0.249  0.75  1.041  0.523  0.148  0.305  1.863  0.082  0.087  0.026  0.084  0.125  0.213  0.605  0.741  0.578  1.22  0.836  0.136  0.867  0.399  0.667  0.317  0.35  0.422  0.125  2.175  0.409  1.991  1.216  0.606  1.017  1.29  0.09  0.054  0.351  0.808  0.929  0.029  0.464  0.021  1.792  0.546  0.079  0.206  0.091  0.056  0.867  0.078  0.353  0.446  
 
 MIR205HG  642587  1  209602167  209605892  1q32.2  0.485  0  0  0  4.068  0  0  0  2.037  0  0  0.184  0  0  0  6.049  0  0.251  0  0  0  0.124  0  0  0  0  0.092  0  0  0.088  0  0  0  0  0  0  0  0  0.058  0  4.714  0.383  0  0  0  0.452  0.104  0  0  0  0  0  0  0  0  0  0  0  0.904  0  
 
 CAMK1G  57172  1  209757044  209787284  1q32.2  0  0  0.124  0  0  0  0.075  0  0.224  0  0.079  0  0  0.036  0  0  0  0.094  0  0  0  0.042  0.385  0  0.059  0.504  0.087  0.058  0  0.058  1.579  0.203  0  0.207  0  0  0.301  1.16  0.055  0  0.077  0.143  0.04  0  0  0  0.019  0  0.137  0  0.09  0.19  0.09  0.112  0.056  0  0.114  0  0  0  
 
 LAMB3  3914  1  209788217  209825820  1q32  0.087  1.64  0.569  0.245  0.363  0.992  1.45  0.876  2.06  2.106  2.114  1.615  3.67  1.615  1.771  2.919  3.98  0.471  0.078  0.13  0.202  0.15  1.839  0.798  3.645  1.05  0.949  1.894  0.54  0.274  1.214  1.596  0.548  0.414  0.688  2.544  5.895  6.143  0.782  0.13  5.588  1.209  0.042  0.971  2.763  2.214  0.779  1.933  4.168  3.096  4.727  4.058  3.986  3.746  0.545  0.727  1.972  0.327  2.463  4.486  
 
 G0S2  50486  1  209848669  209849735  1q32.2  0  8.029  1.654  0  0.097  1.591  3.896  0  0.155  1.891  1.124  0.312  0.623  0  0.253  1.148  0  5.194  0  0.215  1.423  0.301  3.964  1.712  0  0.174  0.304  0.231  0  0.149  0  0  0  0.224  0.566  0  6.58  8.539  0.164  0.367  0.175  2.564  0.076  0  0  0.286  2.86  1.131  3.313  2.572  5.989  2.594  5.015  4.155  0  1.691  3.576  1.2  0.166  3.836  
 
 HSD11B1  3290  1  209859524  209908295  1q32-q41  0  0  0  0  0  0.787  0.713  0  0  0.271  0  0  0  0  0  0  0  0  0  0  0  0  0.478  0  0  0  0.171  0  0  0.035  0  0  0  0.269  0  0  3.916  0.528  0  0  0  0.099  0  0  0  0.108  0  0  0  0  0  0  0  0  0  0  0  0  0  0  
 
 TRAF3IP3  80342  1  209929376  209955668  1q32  0  0  0.109  0  0  0  0  0.061  0  0  0  0  0  0  0  0  0  0  5.088  2.121  0  3.145  0.578  0.162  0  0  0.081  0.116  0  0.059  0  0.098  0  0.163  0  0  0  0  0  0  0  0.139  0.049  0  0  0.07  0  0  0  0.097  0.128  0.232  0  0.088  0  0  0  0.095  0  0  
 
 C1orf74  148304  1  209952552  209958075  1q32.2  0.652  0.471  0.338  0.524  0.806  0.355  1.024  0.441  0.562  0.772  0.838  0.682  0.985  1.227  0.667  0.703  0.663  0.68  1.524  1.07  0.672  1.06  1.004  0.449  0.349  0.853  0.372  0.634  0.297  0.479  0.907  0.902  0.527  0.483  0.447  0.465  0.817  0.969  0.332  0.964  0.862  0.734  1.274  0.325  0.479  0.336  0.966  0.128  0.319  0.445  0.868  0.473  0.665  0.395  0.794  0.818  0.463  0.637  1.061  0.703  
 
 IRF6  3664  1  209958967  209979520  1q32.3-q41  1.256  0.044  0.112  0  2.303  0  0  0  0.104  0.028  0.044  1.576  2.419  1.777  1.724  2.642  2.499  0.817  0  0.048  0  0.063  0.352  0  0  0.443  0.251  0  0  0.045  0.032  0  0  0.219  0  0.165  0.029  0  0.023  0.221  2.02  0.107  0.021  0.191  1.081  1.559  0.556  0  0.472  0  0.406  0.404  0  0.064  0.079  0.073  0.045  0  0.272  0.177  
 
 DIEXF  27042  1  210001311  210030910  1q32.2  1.06  1.749  0.654  1.399  1.272  0.602  1.501  1.414  0.896  1.097  1.407  1.014  1.287  1.995  1.181  1.243  1.087  1.285  1.754  0.946  1.279  1.191  1.062  0.931  1.556  1.335  1.478  1.154  0.75  0.95  1.29  1.113  1.259  0.745  0.885  1.265  1.876  1.491  1.228  1.649  1.22  1.047  1.379  1.127  0.774  0.807  0.418  0.515  0.629  1.044  1.142  0.871  1.014  1.264  1.424  1.747  1.089  1.087  1.824  1.527  
 
 SYT14  255928  1  210111518  210337633  1q32.2  0  0  0.078  0.515  0.024  0.293  0.587  1.103  0.191  0.044  0.108  0  0  0.013  0  0  0  0.066  0  0.033  0  0.03  0.186  0  0.33  1.137  0.836  0.115  0.517  0.467  0.822  0.87  0.758  0.462  0  0  0.359  0  0  0.236  0  0.119  0.074  0.05  0  0.297  0.036  0  0.119  0  0  0.094  0  0.071  1.086  0.995  0  0.358  1.664  1.306  
 
 SERTAD4-AS1  574036  1  210404803  210407466  1q32.2  0  5.131  1.707  0  1.536  3.123  1.422  1.33  1.304  1.343  1.103  0  0  0  0  0.349  0.505  0  0  0  0  0.362  0  0  0  0  1.24  1.657  0.139  0.542  0  1.872  1.94  0.703  0.485  1.897  1.475  1.955  1.601  0.274  0.808  1.427  0  0  0  1.336  0.849  0.846  0.449  1.737  0  1.072  1.005  1.621  2.9  2.225  0.905  1.377  1.556  1.265  
 
 SERTAD4  56256  1  210406194  210416440  1q32.1-q41  1.672  3.241  1.324  0.136  3.303  1.045  0.781  1.826  1.682  1.008  1.202  0.377  0  0.095  0.242  0.709  0.658  0.067  0  0  0  0.183  0.273  0  0  0  1.698  2.288  0  0.381  0  2.554  3.188  1.513  0.269  0.916  1.741  1.137  0.363  0.441  1.431  0.405  0.318  0  0  1.212  2.331  1.108  0.551  0.71  0  0.505  0.902  1.109  2.466  2.052  0.849  0.872  2.283  1.326  
 
 HHAT  55733  1  210501595  210849638  1q32  0.324  0.615  0.253  0.228  0.952  0.458  0.419  1.618  0.812  0.376  0.999  0.235  0.246  0.504  0.064  0.139  0.153  0.104  0.127  0.045  0  0.439  0.374  0  0.323  1.596  0.481  2.254  1.011  1.492  1.559  0.619  0.733  0.435  0.176  0.837  1.071  0.642  0.2  1.104  0.878  0.435  1.44  0.159  0  0.222  0.844  0.354  0.583  0.627  0.058  0.711  0.955  0.783  2.074  1.53  0.267  0.233  1.176  2.274  
 
 KCNH1  3756  1  210851656  211307457  1q32.2  0.104  0.103  0.152  0.696  0.911  0.355  0.111  1.487  0.241  0.948  0.479  0.031  0  0.05  0  0.014  0.017  0.07  0.04  0.044  0  0.092  0.426  0  0.018  1.937  0.182  0.862  0.563  1.36  0.333  0.055  0.203  0.163  0.178  1.038  0.104  0.191  0.046  0.067  0.382  0.184  0.126  0.027  0  0.017  0.296  0.2  0.035  0.106  0.013  0.606  0.487  0.646  2.521  0.846  0.681  0.067  1.592  1.289  
 
 RCOR3  55758  1  211432707  211489725  1q32.2  1.957  2.858  1.233  1.29  2.947  1.467  2.151  2.16  1.912  2.508  2.736  0.865  1.841  2.641  2.003  1.942  1.605  2.369  2.914  2.136  1.524  1.957  1.81  1.855  1.325  2.638  1.879  1.533  1.695  1.414  2.041  1.93  1.625  1.112  1.254  2.344  1.796  2.671  1.488  2.097  2.489  1.852  3.045  2.018  1.521  1.617  1.295  1.119  1.56  1.679  1.684  1.473  1.932  1.634  2.461  2.199  2.199  1.655  2.477  2.179  
 
 TRAF5  7188  1  211499956  211548286  1q32  1.219  2.065  2.013  0.536  0.899  0.814  0.619  1.165  0.455  0.624  1.152  2.247  1.846  1.231  1.748  0.801  1.23  4.542  3.253  0.197  0.763  1.365  1.223  1.034  0.205  1.903  1.087  0.864  1.032  1.5  1.738  1.17  1.415  0.658  1.05  1.046  1.313  1.709  0.234  0.57  0.088  0.832  1.17  0.712  0.135  0.187  0.73  0.374  0.958  0.746  0.636  0.693  0.075  0.584  1.774  1.75  1.474  0.706  1.481  0.941  
 
 LINC00467  84791  1  211556096  211605877  1q32.3  1.411  4.346  0.705  0.814  4.047  1.797  0.222  1.3  0.919  2.147  1.934  1.682  1.502  2.309  0.678  1.497  2.267  4.214  1.908  0.797  1.861  0.835  2.854  1.682  1.539  2.33  0.797  0.709  0.927  0.599  3.029  2.498  0.46  0.496  1.65  1.336  2.124  2.159  0.809  1.451  2.001  2.059  1.982  1.891  1.433  1.171  1.116  0  0.951  0.872  1.349  2.121  1.811  0.655  2.954  1.628  0.579  1.117  0.578  1.659  
 
 RD3  343035  1  211649863  211666259  1q32.3  0  0  0.164  0  0.021  0.072  0.031  0.031  0.134  0.034  0.024  0.067  0  0.033  0  0.03  0.038  0.151  0.033  0.051  0  0.152  0.541  0.031  0  0.023  0.085  0.031  0.033  0.084  0  0  0  0.153  0.024  0  0.034  0  0.067  0  0.037  0.246  0.052  0.062  0.1  0.251  0.057  0  0  0.074  0.053  0.353  0.017  0.056  0.036  0  0.03  0.038  0  0  
 
 SLC30A1  7779  1  211748380  211752099  1q32.3  1.093  2.638  1.004  0.981  1.874  0.795  1.026  3.783  0.874  1.162  1.032  1.334  2.177  1.917  1.433  1.696  1.556  1.935  1.18  1.173  1.508  0.71  1.446  1.634  1.414  2.2  1.286  1.667  1.801  0.592  1.328  1.937  1.738  0.968  1.182  3.057  1.889  2.965  1.67  1.663  1.623  1.43  2.539  1.229  1.57  1.422  1.435  0.922  1.642  1.139  0.981  1.781  1.648  1.788  1.727  1.955  1.962  0.788  2.469  1.945  
 
 LOC105748977  105748977  1  211813149  211827963  -  0  0  0  0  0  0  0  0  0  0  0  0  0  0  0  0  0  0  0  0  0  0  0.552  0  0  0  0  0  0  0.065  0  0  0  0  0  0  0.149  0  0.083  0  0  0.275  0  0  0  0  0  0  0  0  0  0.393  0.116  0.292  0.492  0.861  0  0  0.327  0  
 
 NEK2  4751  1  211831598  211848972  1q32.2-q41  2.564  4.401  2.07  2.509  3.547  2.292  3.051  2.204  1  2.866  3.481  2.484  2.463  3.896  2.889  2.663  3.33  3.6  4.434  2.07  2.664  2.241  2.081  1.981  2.041  2.487  2.29  1.781  1.955  2.131  2.368  2.329  2.113  1.607  2.271  3.013  3.082  3.655  2.275  3.238  3.442  2.436  3.032  2.453  1.567  1.455  1.922  1.39  1.788  2.757  2.374  2.226  2.567  1.674  3.25  2.961  2.354  2.804  3.139  2.396  
 
 LPGAT1  9926  1  211916798  212004114  1q32  1.186  2.267  1.522  0.9  1.666  1.759  2.074  2.143  1.756  2.427  2.234  0.926  1.755  2.5  1.571  1.137  1.933  1.491  2.525  1.135  1.431  0.799  0.315  0.837  1.507  2.279  1.671  1.49  1.601  1.977  2.177  2.055  2.033  1.144  1.622  4.312  1.639  2.293  1.444  2.276  1.968  1.743  2.13  1.5  0.993  0.761  0.746  0.585  1.675  1.137  1.37  1.221  1.628  1.813  1.998  2.477  1.984  1.464  2.113  1.853  
 
 INTS7  25896  1  212113740  212209002  1q32.3  1.588  2.917  1.208  1.568  2.501  1.021  2.664  1.845  1.455  2.431  2.567  1.744  2.186  2.521  1.842  2.102  1.838  2.323  3.647  2.388  2.15  2.605  1.503  1.804  1.911  2.108  2.029  2  1.829  1.713  1.943  2.172  2.151  1.029  1.419  2.825  2.279  2.346  1.281  2.597  2.037  1.266  2.86  1.643  1.278  1.422  1.933  1.039  1.608  2.113  2.546  1.536  1.822  1.476  2.035  2.219  1.783  0.98  2.13  1.998  
 
 DTL  51514  1  212208894  212278348  1q32  1.578  3.54  1.141  1.846  2.941  1.784  2.831  1.663  1.027  2.626  2.668  1.808  2.073  3.09  1.932  2.36  2.753  3.403  4.316  2.809  2.679  3.132  1.932  2.136  2.069  2.879  1.942  2.163  2.042  1.835  2.561  2.323  2.358  1.554  2.057  2.654  2.575  2.373  1.616  2.662  2.386  1.97  2.375  2.693  1.078  1.265  1.654  1.001  1.992  2.202  2.837  1.55  2.125  1.776  2.958  2.421  2.223  2.004  3.024  2.59  
 
 LOC101929541  101929541  1  212386343  212458363  1q32.3  0  0  0  0  0  0  0  0  0  0  0.339  0  0  0.452  0  0  0  0.562  0  0  0  0  0.64  0  0  0  0.243  0.589  0  0.205  0.445  0.809  0  0  0  0  0  0  0  0  0.432  0  0  0  0  0  0  0  0  0  0  0  0  0  0  0  0  0  0  0  
 
 PPP2R5A  5525  1  212458878  212535205  1q32.2-q32.3  2.244  2.169  1.611  1.205  3.226  1.575  2.832  1.682  1.748  2.141  2.294  2.198  2.658  3.768  2.255  2.527  3.135  2.096  4.126  2.902  2.856  2.024  2.244  1.659  2.128  3.095  1.734  1.915  2.179  3.064  2.989  2.869  1.994  1.429  1.629  2.736  2.142  2.791  1.743  2.794  2.612  1.763  3.32  2.627  1.29  2.519  3.55  0.81  1.723  2.393  2.99  1.688  1.984  1.393  3.036  2.352  2.061  2.321  2.978  2.404  
 
 TMEM206  55248  1  212537815  212588267  1q32.3  1.409  3.465  1.289  1.887  2.418  1.183  2.51  0.943  2.67  1.809  2.718  0.834  1.958  2.856  1.423  1.296  1.733  3.566  4.09  2.599  1.6  2.934  1.863  1.126  1.96  1.747  1.935  2.042  1.558  2.116  2.514  2.39  1.895  1.085  1.397  1.609  2.522  2.198  2.075  1.77  2.035  1.017  2.803  1.536  0.609  1.198  1.621  1.057  1.529  2.146  2.801  1.134  1.361  1.446  2.546  1.968  0.903  1.736  2.116  1.695  
 
 NENF  29937  1  212606228  212619721  1q32.3  4.078  5.913  4.394  5.093  5.944  5.043  5.709  4.677  4.921  5.819  5.398  3.753  3.717  5.933  3.437  4.254  4.858  3.299  6.458  5.774  4.632  5.641  4.296  4.312  4.76  4.551  4.096  3.971  4.542  4.65  5.06  5.29  3.849  3.527  5.073  4.912  4.244  6.029  5.548  4.979  4.882  5.492  5.832  4.47  3.13  3.556  4.775  2.196  4.631  3.992  5.38  4.264  4.454  4.85  5.684  5.511  4.475  5.399  5.66  3.976  
 
 ATF3  467  1  212738675  212794119  1q32.3  0  1.098  1.471  3.427  1.847  1.002  1.714  0.995  2.796  1.57  1.459  0  0.799  0.889  0.659  0.969  2.508  1.212  2.106  0.134  1.776  1.126  2.986  0.891  1.481  0.305  1.242  1.303  0.285  1.793  0.3  1.42  1.69  1.384  0.623  0.395  2.143  1.429  2.911  1.455  2.281  1.052  1.503  0.722  1.109  2.271  0.145  0.502  0.281  1.982  2.024  0.858  0.473  2.221  2.675  1.573  1.953  1.629  2.52  1.954  
 
 FAM71A  149647  1  212797788  212800120  1q32.3  0  0  0.232  0.139  0  0.061  0  0  0.206  0.052  0.076  0  0  0.026  0  0  0  0.09  0.041  0.101  0  0.151  0.573  0  0  0  0.054  0  0  0.053  0  0  0.065  0.289  0  0  0  0  0.085  0  0.052  0.243  0.042  0  0  0.063  0.024  0  0  0  0  0.754  0  0.07  0  0  0  0  0  0  
 
 BATF3  55509  1  212859758  212873327  1q32.3  0  1.469  0.783  1.932  0.321  2.104  1.781  0.769  1.438  0.845  0.845  0.185  0.264  1.725  0.182  0.179  0.728  0.212  4.705  0.981  0.745  3.334  1.063  0.493  1.884  0.454  1.544  0.882  0.167  1.39  0.415  2.48  2.142  1.403  2.169  1.276  0.78  0.455  1.692  2.312  0.854  1.753  1.221  0  0.332  0.631  0.531  1.051  0.407  2.667  0.244  1.451  0.183  2.001  3.144  1.188  1.174  3.064  1.913  1.358  
 
 NSL1  25936  1  212899494  212965139  1q41  0.745  1.056  0.788  0.843  1.835  1.442  1.442  1.054  1.823  1.883  1.756  1.155  0.924  1.319  0.913  1.451  1.745  1.333  2.795  0.985  1.915  1.403  1.074  0.783  1.04  1.139  0.883  0.892  1.003  1.306  1.558  1.098  1.002  0.648  1.316  1.606  1.709  1.704  0.438  1.298  1.509  1.828  1.711  1.105  1.123  0.638  0.378  0.48  0.849  1.276  1.288  1.298  1.003  0.741  1.805  1.448  1.071  1.184  1.4  1.555  
 
 TATDN3  128387  1  212965169  212990167  1q32.3  0.422  1.21  0.461  1.161  1.998  0.872  1.335  0.58  0.91  0.924  1.26  1.012  0.869  1.636  0.491  0.813  1.508  2.504  2.923  1.297  1.64  1.228  1.875  1.005  0.913  1.685  0.818  0.429  0.661  0.81  1.716  1.704  0.92  0.858  0.76  1.371  0.898  1.252  0.712  1.36  1.022  1.127  1.864  1.236  0.354  0.417  0.485  0.276  0.539  1.258  1.212  0.794  0.689  0.623  1.433  1.003  0.565  0.865  1.293  1.048  
 
 SPATA45  149643  1  213003484  213020991  1q32.3  0  0.523  0  0  0  0  0.404  0  0  0.21  0  0  0  0  0  0  0  0  0.401  0  0  0  0  0  0  0  0  0  0  0  0  0  0  0  0  0  0  0  0  0  0  0  0  0  0  0  0  0  0  0  0  0  0  0.315  0  0  0  0  0  0  
 
 FLVCR1-AS1  642946  1  213029945  213031480  1q32.3  0.598  1.654  0.29  1.6  2.757  0.817  0.991  0.439  1.24  1.509  2.482  0.909  1.125  2.944  0.934  1.151  2.918  2.506  3.456  0.804  0.846  0.869  2.419  2.304  2.187  2.207  1.756  0.894  1.535  1.17  2.224  1.77  2.024  1.714  1.343  0.901  1.884  2.766  1.236  1.972  2.439  2.1  3.885  1.361  0.4  1.084  3.838  0.213  1.146  0.447  2.287  1.684  2.121  0.843  3.038  2.183  0.692  2.299  1.595  1.177  
 
 FLVCR1  28982  1  213031596  213072705  1q32.3  1.269  2.099  0.428  0.796  2.336  0.762  1.924  1.508  0.701  1.218  1.611  1.381  2.279  2.548  1.879  1.377  1.96  2.597  2.611  1.791  1.199  1.616  0.968  1.219  1.21  1.928  1.346  1.002  0.844  2.145  1.646  1.27  1.366  0.553  1.381  1.964  1.805  2.189  1.045  1.97  1.99  1.285  3.007  1.737  0.816  0.919  2.053  0.602  0.75  1.077  1.298  1.357  1.43  1.167  1.663  2.08  1.401  1.296  1.774  1.154  
 
 VASH2  79805  1  213123861  213164927  1q32.3  0.378  0.125  0.211  0.339  0.533  0.226  0.19  0.494  0.101  0.633  0.19  0.051  0.227  1.042  0.421  0.154  0.269  0.909  1.725  0.092  0  1.408  0.53  0.331  0  0.095  0.081  0  0  0.484  0.14  0.18  0.174  0.235  0.176  0.131  0.056  0.12  0.075  0.264  0.136  0.364  1.213  0.838  0.144  0.18  0.215  0.193  0  0.211  0.588  0.305  0.036  0.114  0.093  0.087  0.062  0.287  0.489  0.17  
 
 ANGEL2  90806  1  213165523  213189217  1q32.3  0.933  1.695  0.704  1.3  1.558  0.81  1.461  1.088  1.664  1.523  1.554  0.557  1.212  1.652  0.741  0.97  1.002  1.808  2.241  1.29  0.994  1.308  1.253  0.741  1.029  1.727  0.775  0.95  0.903  1.006  1.152  0.982  1.175  0.801  0.95  1.226  1.181  2.076  0.809  1.406  1.055  1.26  1.785  1.005  0.733  0.669  0.348  0.372  0.96  1.048  1.417  0.716  0.987  1.013  1.421  1.398  1.199  1.017  1.181  1.347  
 
 RPS6KC1  26750  1  213224574  213446808  1q41  0.817  2.063  1.147  1.556  1.633  0.614  1.828  1.73  1.504  1.928  2.086  0.624  0.903  1.581  0.769  1.122  0.573  1.803  1.449  0.623  1.465  0.751  0.667  0.536  1.328  1.787  0.878  1.538  1.574  1.718  1.539  1.637  1.381  0.765  0.721  1.528  1.477  1.88  0.992  2.002  1.674  1.082  1.92  1.069  1.269  0.742  0.648  0.379  0.973  1.164  2.129  1.032  1.258  1.124  1.375  1.722  1.608  1.468  1.473  1.362  
 
 PROX1-AS1  100505832  1  213992983  214159496  1q32.3  0  0  0.111  0  0  0  0  0  0.191  0.042  0.069  0.097  0  0.02  0  0.039  0.132  1.765  0  0  0  0.081  0.43  0  0  0  0.052  0  0  0.046  0  0  0  0.208  0  0  0  0  0.017  0  0.031  0.175  0.033  0.053  0  0  0.078  0  0  0  0.049  0.172  0  0.029  0  0  0  0  0  0  
 
 LINC00538  100861504  1  214098091  214099996  1q32.3  0  0  0.096  0  0  0  0  0  0  0.029  0  0  0  0  0  0  0  0.076  0  0  0  0.044  0.243  0  0  0  0.024  0  0  0.029  0  0  0  0.127  0.055  0  0  0  0  0  0  0  0  0  0  0  0  0  0  0  0  0  0  0  0  0  0  0  0  0  
 
 PROX1  5629  1  214161277  214214847  1q41  0  0.023  0.103  0.154  0.036  0.056  0.025  0.01  1.295  0.762  0.601  2.114  0.754  0.027  0.044  0.048  0.747  3.111  0.013  0.031  0  0.184  0.667  0.19  0.072  0.107  0.332  0.044  0.028  0.169  0.266  0.169  0.517  0.625  0.033  0.048  0.022  0.026  0.041  0.04  0.073  0.28  0.045  0.019  0.023  0.039  0.109  0  0  0.012  0.18  0.281  0  0.062  0.05  0.041  0  0  0  0  
 
 SMYD2  56950  1  214454564  214510477  1q32.3  2.841  4.998  2.244  1.513  4.437  2.354  3.528  3.167  2.68  2.776  3.625  2.84  2.69  3.634  2.488  2.701  3.018  4.868  4.495  2.792  2.614  2.846  1.076  1.877  2.977  2.929  3.539  2.434  3.036  3.676  3.697  2.898  3.519  1.744  1.95  3.152  3.29  3.829  2.296  3.316  3.688  2.399  4.681  3.144  1.847  2.237  2.324  1.65  2.729  3.752  3.051  1.824  2.805  2.094  3.677  3.791  3.365  2.411  3.936  2.737  
 
 PTPN14  5784  1  214522038  214725024  1q32.2  1.019  3.048  2.518  1.819  2.613  2.881  2.662  3.498  3.127  3.325  3.146  0.833  1.175  2.277  2.469  1.905  0.993  1.483  0.214  0.215  0.124  0.443  0.288  0.066  2.316  2.996  2.323  2.637  2.48  1.242  1.785  2.298  2.607  1.358  1.242  3.025  3.522  3.254  2.943  2.635  2.494  1.87  2.863  2.351  2.344  1.826  2.655  1.624  2.765  1.609  3.087  2.616  3.997  2.335  3.884  3.474  3.467  1.611  3.843  3.958  
 
 CENPF  1063  1  214776531  214837914  1q41  2.847  5.119  2.336  2.246  3.577  2.853  3.421  2.984  1.453  3.939  4.376  1.944  0.606  4.11  3.378  2.906  2.708  4.312  4.473  3.233  2.598  3.12  1.276  1.75  2.346  3.478  3.421  2.972  3.418  2.857  3.712  3.177  3.73  2.256  2.699  3.171  3.481  3.981  1.792  3.647  3.416  3.104  3.671  3.529  2.555  2.348  2.737  1.833  2.941  2.319  3.324  2.36  3.18  2.28  4.063  3.399  3.741  2.667  3.776  3.281  
 
 KCNK2  3776  1  215178884  215410436  1q41  0.073  0.183  1.09  1.124  4.379  1.022  0.061  0.064  0.148  1.509  0.177  0.127  0  0.025  0  0.027  0.051  0.069  0  0.039  0  0.107  0.231  0  0.243  0  0.344  0.413  0.087  0.499  0.273  0.095  0.221  0.148  0.038  0  0.552  0  0.187  0.266  1.716  1.178  0.041  0  0.772  0.029  0.153  0  0  0  0.164  0.198  0  0.098  0.139  0.359  0.759  0.331  0.058  0  
 
 KCTD3  51133  1  215740734  215795149  1q41  3.916  3.744  1.609  1.214  5.024  1.466  2.223  1.731  2.089  2.658  2.675  2.77  2.738  3.545  2.883  2.579  2.52  3.971  2.428  1.601  2.021  0.703  1.646  1.55  2.216  3.195  2.19  2.485  1.983  2.034  2.5  2.426  2.398  1.336  1.669  2.486  2.596  2.413  0.911  2.549  2.792  2.351  2.583  1.533  1.188  1.326  0.318  0.961  1.959  2.333  3.165  1.687  2.079  1.962  2.337  2.858  2.363  1.467  2.443  2.388  
 
 USH2A  7399  1  215796235  216596738  1q41  0.022  0.016  0.107  0.032  0.079  0.011  0.034  0.019  0.087  0.025  0.025  0.027  0.015  0.032  0.032  0.027  0.03  0.044  0.023  0.022  0.033  0.051  0.247  0.016  0.016  0.038  0.098  0.087  0.03  0.081  0.047  0.036  0.023  0.137  0.016  0.019  0.014  0.026  0.019  0.023  0.03  0.186  0.033  0.027  0.02  0.017  0.03  0  0.021  0.018  0.037  0.206  0.027  0.03  0.029  0.024  0.02  0  0.02  0.019  
 
 LOC102723833  102723833  1  216245806  216260259  1q41  0  0  0  0  0  0  0  0  0  0  0  0  0  0  0  0  0  0.903  0  0  0  0  0  0  0  0  0  0  0  0  0  0  0  0  0  0  0  0  0  0  0  0.308  0  0  0  0  0  0  0  0  0  0  0  0  0  0  0  0  0  0  
 
 ESRRG  2104  1  216676587  217311097  1q41  0  0.089  0.123  0  0.031  0.138  0.052  0.147  0.309  0.061  0.254  0.059  0  0.028  0  0.034  0  0.26  0.029  0  0  0.066  0.381  0.248  0  0.289  0.094  0.028  0  0.219  0.194  0.21  0.12  0.138  0  0  0.085  0.081  0.032  0.062  0.166  0.239  0.049  0.066  0.598  0.373  0.236  0.127  0  0.107  0.035  0.14  0.036  0.136  0.263  0.142  0  0.137  0  0.197  
 
 GPATCH2  55105  1  217600334  217804444  1q41  2.494  1.766  0.887  1.127  1.742  0.855  1.604  1.464  1.131  1.641  1.779  0.805  1.512  2.18  1.2  1.205  1.396  2.528  1.711  1.614  0.13  1.496  0.908  0.884  0.952  1.491  0.867  1.282  0.893  1.009  1.366  1.587  1.057  0.828  1.215  1.359  1.151  1.427  1.189  1.629  1.7  1.162  1.511  1.468  1.041  0.65  1.713  1.045  1.215  1.364  1.621  0.809  1.432  0.957  1.571  1.669  1.452  1.262  1.485  1.185  
 
 SPATA17  128153  1  217804665  218041495  1q41  1.628  0.041  0  0  0.369  0.144  0.063  0.111  0.193  0.076  0.135  0.205  0.204  0.386  0  0.074  0.223  0.709  0  0  0  0.021  0.165  0.059  0  0.119  0.058  0.052  0  0.2  0  0.196  0  0.134  0.27  0.124  0.139  0.127  0.045  0.226  0.321  0.395  0.025  0.062  0.179  0.048  0.044  0  0.174  0.103  0.291  0.076  0.124  0.078  0.286  0.133  0  0.055  0.096  0.136  
 
 LINC00210  100885798  1  218066241  218094146  1q41  0  0  0  0  0  0  0  0  0  0  0  0  0  0  0  0  0  0  0  0  0  0.052  0.292  0  0  0  0.045  0  0  0.052  0  0  0  0  0  0  0  0  0.037  0  0  0  0  0  0  0  0  0  0  0  0  0.215  0  0  0  0  0  0  0  0  
 
 LOC101929631  101929631  1  218216846  218232482  -  0  0  0  0  0  0  0  0  0.069  0  0  0  0  0  0  0  0  0  0  0  0  0.042  0.228  0  0  0  0.026  0  0  0.035  0  0  0  0.15  0  0  0  0  0  0  0  0  0  0  0  0  0  0  0  0  0  0  0  0  0  0  0  0  0  0  
 
 RRP15  51018  1  218458628  218511325  1q41  2.036  3.174  0.869  0.958  1.796  1.212  1.719  1.024  1.049  1.2  1.38  0.886  1.098  2.061  1.017  1.224  1.175  3.006  2.486  2.01  1.658  1.702  0.987  1.037  1.407  1.557  0.905  1.02  0.692  0.924  1.79  1.539  0.86  0.634  1.272  1.798  1.552  2.047  1.539  1.437  1.8  1.271  1.794  1.475  0.985  0.646  0.863  0.942  0.868  2.266  2.175  0.668  1.238  0.877  1.418  1.399  1.017  1.044  1.372  1.471  
 
 TGFB2-AS1  728463  1  218517537  218519020  1q41  0  2.276  0.348  0  0.15  0.942  0  0.258  0  0.184  0.563  0  0  0  0  0  0  0  0  0  0  0  0  0  0  0  0.173  0  0  0  0  0  0  0.39  0.685  0.938  0.438  0.37  0.312  0  0.331  0.289  0  0  0.332  0  0  0  0  0  0.395  0.594  0.806  0  0.692  0  0.432  0  0.324  0.913  
 
 TGFB2  7042  1  218518675  218617961  1q41  0.037  3.656  3.774  1.016  0.479  3.246  2.095  3.206  1.798  1.428  3.029  0.064  0.039  0.159  0.258  0.115  0.089  0.098  0.046  0.111  0.076  0.271  0.237  0.033  0.117  0.141  0.568  0.168  0.05  0.24  0.489  0.231  1.215  0.692  1.775  3.785  4.792  2.427  1.86  1.033  2.387  1.884  0.13  0.163  1.521  1.117  2.162  1.248  0.451  0.97  1.128  1.336  3.447  2.801  3.383  3.403  2.533  0.132  3.262  4.868  
 
 TGFB2-OT1  103611157  1  218615967  218617337  1q41  0  0  0  0  0  0  0  0  0  0  0  0  0  0  0  0  0  0  0  0  0  0  0  0  0  0  0  0  0  0.416  0  0  0  0  0  0  0  0  0  0  0  0  0  0  0  0  0  0  0  0.579  0  0  0  0  0  0  0  0  0  0  
 
 LYPLAL1-AS1  643723  1  219254316  219347130  1q41  0  0.215  0.44  0  0  0.219  0.09  0.058  0.088  0.103  0.078  0  0  0.077  0  0  0  0  0.165  0  0  0.129  0.258  0  0.241  0.064  0.097  0  0  0.041  0  0  0  0.189  0  0  1.64  0  0.855  0  0.157  0.334  0  0  0  0.057  0.038  0  0  0.063  0.208  0.221  0  0  0  0  0.073  0  0  0  
 
 LYPLAL1  127018  1  219347172  219386207  1q41  1.948  3.293  0.857  1.091  2.271  1.255  1.544  1.246  1.255  1.765  1.793  1.148  1.35  3.099  1.354  1.466  1.212  3.343  2.348  2.031  2.19  1.524  2.117  1.272  1.392  2.126  0.761  0.972  1.283  0.73  2.158  2.571  0.935  1.06  1.587  1.873  1.846  2.384  2.243  1.742  2.162  1.616  2.19  1.097  0  0  1.473  0.529  0.482  2.021  2.223  1.253  1.223  1.23  2.511  2.234  1.208  1.694  1.522  2.393  
 
 SLC30A10  55532  1  220087605  220101993  1q41  0.118  0.063  0.167  0  0.092  0  0.222  0.067  0.115  0.144  0.119  0.096  0.115  0.073  0  0.126  0.318  0.127  0.058  0.156  0.848  0.286  0.471  0.081  0  0.045  0.132  0.048  0.086  0.171  0.083  0  0  0.173  0.158  0  0.119  0  0.142  0.069  0.058  0.785  0.06  0  0  0.049  0.145  0  0  0  0.103  0.76  0  0.176  0.049  0.067  0.038  0.092  0.09  0  
 
 EPRS  2058  1  220141941  220220000  1q41  2.943  4.647  2.997  3.363  3.881  1.905  3.69  3.234  3.246  3.67  4.122  2.934  3.796  5.013  3.579  3.419  4.348  4.948  4.693  4.516  4.284  2.99  3.929  3.427  3.076  3.787  2.421  2.579  2.655  2.601  3.367  4.12  2.614  1.788  2.508  3.796  3.376  3.755  3.12  3.94  3.398  2.95  3.677  4.073  2.633  2.814  1.988  2.523  3.004  4.342  4.533  2.671  2.992  2.987  3.929  4.399  4.036  2.59  3.873  4.09  
 
 BPNT1  10380  1  220230823  220263195  1q41  0.958  1.45  0.65  1.695  2.419  1.113  1.367  1.728  0.9  1.842  2.053  1.735  1.853  1.498  1.923  2.149  3.321  1.744  3.825  1.319  2.21  1.549  0.715  1.828  1.861  2.148  0.842  2.06  1.542  0.744  2.04  3.053  2.049  1.563  1.018  2.076  1.982  2.256  0.799  2.014  2.055  2.145  0.823  1.627  1.585  1.848  0.422  1.461  1.101  3.624  2.213  1.179  1.357  1.826  1.957  1.942  1.709  1.361  2.19  2.108  
 
 IARS2  55699  1  220267454  220321383  1q41  3.378  5.16  2.079  2.138  4.679  2.082  3.635  2.636  2.938  3.368  4.159  2.996  3.253  4.576  3.243  3.526  4.302  5.335  4.855  3.502  3.515  2.83  2.832  2.884  3.181  4.308  2.81  3.378  3.079  3.372  4.213  4.588  3.319  2.418  3.018  4.115  3.784  4.378  2.505  4.225  3.7  3.505  4.166  3.659  2.326  2.459  3.056  2.103  2.902  4.32  4.09  2.898  3.311  2.844  4.098  3.94  3.429  3.2  3.949  3.729  
 
 RAB3GAP2  25782  1  220321609  220445843  1q41  1.257  2.709  0.93  1.601  1.974  1.001  1.946  1.422  1.89  1.775  1.954  0.983  1.811  2.052  1.671  1.508  1.155  2.367  2.816  2.348  1.968  1.649  1.509  1.288  1.5  2.163  1.376  1.429  1.643  1.29  2.026  2.142  1.621  1.041  1.032  2.113  1.591  2.024  1.792  1.985  1.989  1.234  2.18  0.889  1.156  0.959  1.071  1.119  1.431  2.574  1.872  0.989  1.249  1.5  1.991  2.031  1.818  1.114  2.118  1.992  
 
 AURKAPS1  6791  1  220439520  220441057  1q41  1.485  0.747  0.676  0.929  1.062  0.338  0.886  0.577  0.471  0.982  1.318  0.626  0.669  1.13  0.872  1.157  0.857  0.14  1.609  0.596  0.372  0.856  0.873  0.33  0.677  0.651  0.928  0.911  1.342  0.696  0.999  1.112  0.734  0.69  0.959  0.522  1.143  0.786  0.496  1.102  0.732  1.378  1.138  0.722  0.679  0.313  0.622  0.755  0.394  1.177  0.925  0.547  1.153  0.574  1.211  0.84  0.855  0.405  0.925  0.414  
 
 MARK1  4139  1  220701524  220837799  1q41  0.118  0.505  0.095  1.349  0.135  0.559  0.107  0.595  0.999  0.657  1.227  0.039  0.092  1.122  0.832  0.041  0.058  1.552  0.032  0.067  0  0.155  0.31  0.208  0.386  0  0.124  0.037  0  0.967  0.299  0.872  0  0.12  0.086  0.042  0.811  0  0.542  0.627  1.387  0.863  1.13  0  0.409  0.044  0.036  0.518  0.106  1.898  0.382  0.848  0.019  0.168  0.083  0.054  0  0.609  0  0  
 
 C1orf115  79762  1  220863627  220872499  1q41  1.037  0.034  0.16  0.933  1.821  0.318  0  0.499  0.951  0.144  0.58  0.07  0.281  2.387  2.038  0.04  1.213  0.188  0.061  0.064  0  0.161  0.599  0  0  1.411  0.524  0  0.594  2.083  1.388  1.667  0  0.209  1.239  0.875  0.789  0  0.532  1.014  1.498  0.639  1.236  1.399  0.575  0.134  0.976  0.981  1.391  2.408  1.192  1.539  0.043  0.702  0.48  0.262  0.031  1.186  0  0.527  
 
 MARC2  54996  1  220921675  220957596  1q41  0.382  1.225  0.101  0  2.311  0.344  0.5  0.723  1.279  0.425  1.12  0  0.137  2.096  1.309  0.435  1.333  2.51  0.066  0.79  0  0.138  0  0  0  0.161  0.074  0.039  0  0.115  0.811  0.844  0  0  0.744  1.501  1.016  0  0.179  0.192  1.215  0.965  0.883  0.221  0.227  0.69  0.575  0.653  0.928  2.708  1.564  0.981  0.303  0.359  1.038  0.493  0.265  0.673  0  0.851  
 
 MARC1  64757  1  220960038  220987741  1q41  2.501  0.24  0.087  0  2.15  0.14  0.128  0.041  0.118  0.108  0.414  0  2.518  2.811  2.472  1.051  1.909  2.619  0.041  0.539  0  0.189  0.235  0.4  0  0.236  0.088  0.055  0  0.279  0  0  0  0.144  1.376  2.274  0.822  0  0.342  0.534  1.23  1.582  0.196  1.143  2.031  0.482  1.657  0.979  0.637  2.12  2.738  1.378  0  0.103  2.558  0.766  0.17  0.715  0.232  1.233  
 
 LINC01352  101929730  1  221002596  221005770  -  0  0  0  0  0  0  0  0  0  0.199  0  0  0  0.112  0  0  0  0.19  0  0  0  0.063  0  0  0  0  0.056  0  0  0.048  0  0  0  0  0  0  0  0  0  0  0  0  0  0  0  0  0  0  0  0  0  0.188  0  0  0  0  0  0  0  0  
 
 HLA-AS1  100873924  1  221006104  221053482  -  0  0  0  0  0  0  0  0  0  0.167  0  0  0  0  0  0  0  0  0  0  0  0  0  0  0  0  0  0  0  0  0  0  0  0  0  0  0  0  0  0  0  0  0  0  0  0  0.123  0  0  0  0  0  0  0  0  0  0  0  0  0  
 
 HLX  3142  1  221052742  221058400  1q41  0.12  0.206  1.537  1.763  0.088  1.195  1.495  0.804  0.525  2.156  0.361  0.16  0  0.466  0  0.09  0.163  0.146  0.037  1.75  0.229  0.347  0.449  0  1.811  0.435  1.066  0.235  0.302  1.839  1.613  1.189  0.78  0.762  0.938  0.886  1.353  0  0.624  0.341  0  0.592  0.112  0.129  0.246  0.117  0.729  0.762  0.362  1.285  1.646  1.112  1.059  1.283  0.503  1.347  2.286  1.908  0.657  0.55  
 
 C1orf140  400804  1  221503269  221509638  1q41  0.059  0.192  0.129  0  0.078  0  0.143  0.018  0.086  0.106  0.062  0.079  0.047  0.028  0.098  0.045  0.053  0  0.07  0.076  0.187  0.123  0.302  0.084  0.1  0.113  0.117  0.082  0.06  0.102  0.038  0  0  0.177  0.055  0.116  0.131  0.039  0.042  0.104  0  0.033  0.084  0.133  0  0.029  0.107  0  0.126  0.098  0.112  0.168  0.107  0.068  0.053  0.071  0.063  0  0.129  0.072  
 
 DUSP10  11221  1  221874761  221915518  1q41  0  4.151  1.551  0.913  0.776  0.664  2.384  1.312  1.475  2.747  1.346  0.838  0.101  0.661  0.256  0.21  2.21  0.239  1.505  0.671  0.993  0.472  0.766  0.473  1.872  4.015  2.445  3.251  3.519  2.095  4.289  3.197  2.189  1.667  0.19  1.013  1.647  2.648  0.503  1.146  2.522  0.615  0.045  0.207  1.086  0.747  0.06  0.468  1.911  0.798  0.408  0.73  2.257  1.325  1.427  1.388  3.285  1.126  3.111  1.779  
 
 HHIPL2  79802  1  222695601  222721444  1q41  0  0.094  0.161  0.165  0.083  0.379  0.314  2.288  0.122  0.701  0.235  0.043  0  0.13  0  0  0  0.266  0.05  0.044  0  0.183  0.205  0  0.135  0.051  0.048  0.044  0.193  0.212  0.062  0.082  0  0.24  0.785  0.123  0.194  3.02  0.155  0.923  0.064  1.555  0.079  0  0.11  0.057  0.05  0  0.09  0.201  0.242  0.559  0.703  0.277  0.064  0  1.609  0.2  0.054  0  
 
 TAF1A  9015  1  222731243  222763275  1q42  0.517  2.143  0.667  0.497  0.828  0.475  1.125  0.757  0.74  1.254  1.456  0.672  1.129  2.115  0.911  1.033  1.256  1.386  2.495  1.014  1.43  1.524  0.585  0.475  1.321  1.096  0.525  0.637  0.507  0.144  1.179  1.193  0.775  0.623  0.695  0.769  1.534  1.353  0.914  1.198  1.222  0.986  2.028  1.166  0.776  0.519  0.456  0.957  1.185  2.209  2.055  0.499  0.917  0.777  1.752  1.435  1.149  1.472  1.147  1.26  
 
 LOC100506161  100506161  1  222763261  222765975  -  0.347  1.745  0.264  0  0.593  0.638  0.503  0.14  0.618  0.477  0.861  0.281  0.279  1.239  0.234  0.384  0.888  1.927  1.071  0.192  0.424  0.759  1.241  0.633  0.516  0.387  0.528  0.236  0.672  0.062  0.529  0.793  0.465  0.307  0.15  0  0.242  0.586  0.346  0.948  0.948  0.731  0.666  0  0  0  0.283  0  0  0.928  1.134  0.642  0.511  0.797  1.007  0.772  0.354  0  0.48  0  
 
 MIA3  375056  1  222791443  222841354  1q41  1.184  2.082  1.346  1.555  2.685  1.301  2.723  2.524  1.915  2.769  2.611  1.181  1.634  2.481  2.233  1.493  1.927  1.89  3.223  2.309  2.003  2.056  1.825  1.699  1.533  2.371  1.62  1.629  1.78  1.216  2.069  2.242  1.52  0.902  1.312  2.164  2.236  2.4  1.735  2.048  1.96  1.284  2.131  2.133  1.052  1.08  1.436  1.398  1.273  2.849  2.855  1.271  1.861  1.617  2.503  2.451  2.102  1.734  2.254  2.121  
 
 AIDA  64853  1  222841354  222885864  1q41  1.389  4.864  2.791  2.606  2.129  2.087  3.413  2.823  2.539  3.515  3.255  1.781  2.521  3.526  2.63  2.129  2.671  4.306  3.408  2.66  2.382  1.888  2.077  1.8  3.415  3.299  2.623  2.61  2.558  2.142  2.9  3.302  2.656  1.763  1.868  3.03  3.378  4.17  2.597  3.463  2.47  2.358  4.022  3.222  1.632  2.038  2.401  1.981  2.869  3.832  4.509  1.946  3.214  2.553  3.518  3.764  3.7  2.888  4.153  3.481  
 
 BROX  148362  1  222885894  222908538  1q41  2.077  3.695  1.732  1.798  3.419  1.844  2.615  2.154  2.196  2.771  2.864  2.588  2.449  3.596  2.438  2.486  2.908  3.395  2.887  2.838  2.223  1.92  2.677  2.041  2.611  2.493  1.733  2.064  2.042  0.997  3.183  3.201  2.154  1.885  1.761  2.744  2.349  2.905  2.712  2.385  2.767  1.913  2.178  2.011  1.242  1.85  2.089  1.666  2.23  3.296  3.274  2.273  2.278  2.456  2.969  2.931  2.547  2.311  3.164  2.707  
 
 FAM177B  400823  1  222910557  222924002  1q41  0  0  0  0  0  0.105  0.064  0  0.091  0  0  2.19  0  0.048  0  0  0.254  0  0  0  0  0.077  0  0  0  0  0.081  0  0  0.045  0  0  0  0  0  0  0  0  0  0  0  0.113  0.039  0.103  0  0.054  0.039  0  0  0  0.149  0  0  0.121  0  0  0  0  0  0  
 
 DISP1  84976  1  222988430  223179337  1q41  0.579  0.509  0.537  0.827  0.757  0.3  1.329  0.761  1.237  1.318  1.562  0.653  0.388  0.72  0.728  0.339  0.428  0.207  0.697  0.312  0.253  0.665  0.44  0.125  0.389  1.021  0.911  0.803  0.327  0.893  0.318  0.389  0.742  0.439  0.352  1.197  0.746  1.001  0.304  0.648  0.503  1.386  0.48  0.76  0.266  0.195  0.219  0.207  0.752  0.33  0.822  0.767  0.675  0.728  0.624  0.744  0.803  0.344  0.438  0.364  
 
 TLR5  7100  1  223282747  223316624  1q41-q42  0.23  0.281  0.148  0.404  0.659  0.123  0.603  0.169  0.165  0.326  0.059  0.297  0.049  0.195  0.094  0.036  0.184  0.037  0.035  0.032  0.106  0.355  0.298  0  0  0.024  0.134  0.064  0.037  0.095  0.025  0  0  0.184  0  0.533  0.064  0.865  0.107  0.078  0.933  0.249  0.021  0.192  0.1  0.127  0.048  0.041  0.293  0.192  0.083  0.199  0.142  0.202  0  0  0.112  0.135  0.061  0  
 
 SUSD4  55061  1  223394160  223537544  1q41  1.72  0.549  0.248  0.93  0.254  0.14  0.648  0.66  0.452  1.089  0.808  0  0  0.137  0.973  0.124  0.331  0.132  0.095  0  0  1.863  0.285  0  0  0  0.114  0.057  0  0.065  0  0  0  0.14  0  1.797  0.36  0.461  0.386  0.142  2.179  0.47  0.035  0.19  1.508  1.162  0  0.783  1.365  2.013  0.244  0.468  1.14  0.329  0.157  0  1.109  0.113  0.702  0.118  
 
 CCDC185  164127  1  223566714  223568812  1q41  0  0  0.105  0  0.081  0  0.072  0.044  0.124  0.073  0.036  0  0  0.064  0  0.066  0  0.065  0.067  0.096  0  0.235  0.597  0.082  0  0  0.129  0  0  0.117  0  0  0  0.199  0  0.077  0  0  0.092  0  0  0.323  0.04  0  0  0.066  0.039  0  0.088  0.043  0  0.767  0  0.191  0.09  0  0.135  0  0.186  0  
 
 CAPN8  388743  1  223714971  223853436  1q41  0  0.064  0.22  0  0.167  0.12  0.104  0.054  0.111  0.084  0.095  1.888  0.111  0.113  0  3.303  0.867  0.106  0  0.105  0  0.066  0.163  0  0.08  0.047  0.196  0.052  0.067  0.111  0  0  0  0.123  0  0.489  0.054  0.14  0.209  0.105  1.909  0.083  0.074  0  0  0.108  0.112  0  0  0  0.198  0.331  0.114  0.12  0.282  0.111  0.053  0  0.08  0.091  
 
 CAPN2  824  1  223889294  223963720  1q41-q42  0.342  7.22  4.242  4.904  3.979  3.908  5.551  4.025  4.396  4.601  5.288  3.561  4.005  5.737  3.337  4.042  4.481  5.885  0.452  1.289  3.117  0.815  0.593  0.805  4.886  4.651  3.996  2.945  3.939  2.629  4.151  4.119  3.979  3.11  3.765  5.543  5.365  5.892  5.576  3.625  5.401  4.003  3.967  4.305  2.505  4.236  5.316  4.161  4.556  6.177  5.756  4.516  4.791  4.372  6.13  4.952  4.565  5.338  5.104  5.614  
 
 TP53BP2  7159  1  223967594  224033674  1q41  1.715  2.865  1.808  2.338  2.168  1.933  2.939  2.15  2.079  2.583  2.954  1.534  2.273  2.937  2.215  2.05  2.43  2.25  1.968  3.056  2.681  2.784  0.578  0.594  2.649  2.843  2.446  2.299  2.184  1.982  2.528  2.657  2.413  1.098  1.446  2.762  2.888  3.147  1.968  2.799  2.818  1.59  3.656  2.722  1.963  2.378  2.087  2.756  2.459  4.085  2.363  1.895  2.903  3.277  3.697  2.503  3.922  2.787  4.072  3.355  
 
 LOC441124  441124  1  224138967  224180346  1q42.11  0.914  0.373  0.483  0.827  1.863  0.658  0.934  0.337  0.921  1.293  0.918  0.484  0.928  1.06  1.256  1.533  1.015  0.096  1.605  0.809  1.302  1.586  1.658  0.738  0.457  1.025  0.956  1.112  0.741  0.982  1.288  1.214  0.715  0.43  0.795  1.264  1.588  2.142  0.666  1.244  1.191  1.5  1.518  0.956  0.621  0.463  0.663  1.081  0.793  1.735  0.817  0.979  0.966  0.758  1.658  0.653  1.058  1.216  0.911  0.613  
 
 FBXO28  23219  1  224301788  224349749  1q42.11  1.521  3.012  1.055  1.775  1.799  1.053  1.707  1.629  1.891  1.714  2.084  1.284  2.082  2.856  1.69  1.665  1.893  2.028  2.48  1.529  1.354  1.346  1.268  1.104  2.185  2.21  1.284  1.664  1.429  0.98  2.021  2.097  1.504  1.213  0.951  1.92  1.84  2.236  1.39  2.487  2.122  1.237  2.07  1.74  1.258  1.204  1.543  1.413  1.307  2.933  2.747  1.145  1.729  1.617  1.653  1.782  1.897  1.584  1.953  1.763  
 
 DEGS1  8560  1  224370909  224381142  1q42.11  1.389  4.077  4.078  3.953  3.668  3.285  4.641  4.451  4.328  4.75  4.505  1.781  1.974  3.552  2.781  2.837  2.647  2.128  3.899  3.546  2.797  2.953  2.872  2.289  3.802  4.297  3.061  2.751  3.641  2.488  4.13  4.718  3.362  2.73  2.899  4.077  3.964  4.781  3.671  4.515  2.855  3.512  3.808  3.685  2.393  4.988  2.764  3.466  3.633  6.036  4.217  2.576  3.75  4.419  4.297  4.509  4.738  3.328  4.167  4.268  
 
 LOC101927143  101927143  1  224396449  224400981  -  0  0  0.214  0  0  0.334  0  0  0.2  0  0  0  0  0.076  0  0  0  0  0  0  0  0.195  0.524  0  0  0  0.199  0  0  0.054  0  0  0  0.399  0  0  0  0  0.076  0  0  0.749  0.119  0  0  0.717  0  0  0  0  0.107  0  0  0.18  0  0  0  0  0  0  
 
 LOC101927164  101927164  1  224407314  224415745  -  0.24  0.186  0.238  0.581  0.575  0.292  0.363  0.186  0.353  0.426  0.307  0  0.198  0.307  0.133  0.459  0.23  0.147  0.421  1.218  0  1.243  0.904  0.229  0  0.313  0.225  0.096  0  0.081  0.405  0.233  0.157  0.352  0  0.14  0.192  0.267  0.677  0.288  0.128  0.631  0.904  0.148  0.226  0.251  0.473  0.197  0  0.458  0.143  0.211  0.132  0.108  0.637  0.2  0.152  0  0.145  0  
 
 NVL  4931  1  224415035  224517891  1q41-q42.2  1.146  2.15  0.909  1.808  2.023  0.977  1.751  1.393  1.351  1.992  1.966  0.932  1.298  1.627  1.7  1.486  1.387  1.849  3.4  1.643  2.261  1.979  1.156  1.281  1.308  1.745  0.985  1.492  1.337  1.074  1.9  1.76  1.407  0.51  0.935  1.75  1.852  2.252  1.179  2.778  1.711  1.493  1.838  2.059  1.153  1.298  0.206  1.77  1.462  3.412  1.958  0.964  1.48  1.636  1.76  1.533  2.056  1.444  1.976  1.69  
 
 CNIH4  29097  1  224544512  224567153  1q42.11  3.509  6.882  3.123  3.241  4.641  3.754  4.762  3.712  3.161  4.09  4.265  3.273  3.41  4.652  3.035  3.707  4.197  6.526  4.745  4.797  2.569  3.663  2.416  2.835  3.851  3.712  3.8  3.121  3.085  2.749  4.219  4.109  3.327  2.031  3.912  3.888  4.161  4.913  3.989  3.542  3.916  3.405  5.54  3.897  2.926  3.102  5.215  3.62  3.281  5.3  3.938  2.712  4.348  3.433  4.652  5.114  3.686  4.643  4.593  3.714  
 
 WDR26  80232  1  224572844  224622001  1q42.13  1.891  3.501  1.915  2.237  3.698  1.47  2.731  2.582  3.293  2.814  2.819  1.387  2.279  3.065  2.185  2.017  2.216  3.144  3.372  1.586  2.063  1.829  1.776  1.227  2.675  2.931  1.78  2.474  2.211  1.902  2.995  2.96  2.172  1.445  1.345  2.637  2.939  2.724  2.233  2.978  2.856  1.509  3.104  2.637  1.815  1.656  1.721  2.19  1.814  3.729  2.851  1.858  2.197  1.795  2.276  2.79  2.258  2.17  2.359  2.633  
 
 CNIH3  149111  1  224804178  224928249  1q42.12  0  2.258  0.197  0.216  0.128  0.122  1.206  0.169  2.583  0.337  0.33  0.356  0.073  0.128  0  0.061  0  1.668  0  0.141  0  0.251  0.541  0  0.067  4.296  0.517  0.884  1.711  0.141  0.708  1.711  0  0.342  0.131  0.08  2.49  3.824  0.646  0.287  0.21  0.267  0.069  0.062  0  0.083  0.038  0.241  0.414  0.68  1.111  0.356  0.419  0.132  0.417  0.123  0.46  0  0  1.346  
 
 DNAH14  127602  1  225117355  225586996  1q42.12  0.598  2.378  0.721  0.5  1.852  0  0  0  0  0.038  0  0.717  1.644  1.777  0.748  0.459  0  3.401  0.133  0.967  2.369  0.14  0.742  0.895  1.21  1.358  0.746  0.779  0.851  0.809  1.755  1.173  0.966  0.854  1.135  1.501  1.142  1.57  0.997  1.155  1.637  0.606  2.7  0.853  0.894  0.766  0.657  0.898  0.857  3.13  1.639  0.714  0.909  0.766  1.032  1.362  1.097  1.01  0.971  1.522  
 
 LBR  3930  1  225589203  225616557  1q42.1  1.689  5.736  1.487  2.252  2.143  2.737  2.316  2.444  2.331  2.272  3.157  3.212  3.521  4.147  2.95  3.377  4.418  6.101  5.588  4.106  3.981  4.213  3.258  3.332  2.683  2.357  0.993  1.58  1.271  1.41  3.238  2.445  1.747  1.191  2.814  2.965  3.577  3.536  2.024  2.75  2.742  3.08  3.326  3.209  1.849  2.08  3.62  2.436  2.388  4.226  2.022  1.902  2.458  2.003  3.192  3.024  2.533  2.86  2.714  3.551  
 
 ENAH  55740  1  225674533  225840845  1q42.12  1.67  2.174  2.889  1.703  2.639  2.101  2.751  3.401  2.282  3.596  4.476  0.125  2.107  2.656  1.859  1.517  2.021  2.766  0.24  0.242  0.061  0.761  0.873  0.196  0.669  2.422  2.152  2.503  1.748  1.783  1.892  3.161  1.937  0.961  1.667  2.687  2.429  2.737  1.735  2.657  2.104  2.179  4.56  2.089  3.327  2.832  1.397  2.064  1.981  2.98  2.697  1.707  2.809  2.304  3.173  2.809  3.034  1.376  3.275  2.842  
 
 SRP9  6726  1  225965514  225978168  1q42.12  4.416  7.056  3.572  4.487  5.732  3.941  5.405  4.97  4.75  5.516  6.3  5.012  5.028  6.768  4.878  5.155  5.716  6.987  7.576  6.654  5.503  6.288  4.631  4.17  4.792  4.696  3.772  3.883  4.171  3.311  5.185  5.111  4.338  3.483  3.961  5.053  4.876  5.439  4.951  5.123  5.062  4.794  6.258  4.929  4.187  4.33  4.895  4.4  4.7  7.151  5.578  3.638  4.173  4.372  4.913  5.343  5.123  4.878  5.231  5.3  
 
 EPHX1  2052  1  225997775  226033262  1q42.1  2.155  1.483  3.001  3.692  4.081  1.205  5.291  4.177  6.276  5.388  5.39  3.257  5.654  3.659  3.661  4.503  3.492  0.206  1.857  0.923  1.913  2.054  2.705  3.331  0.55  4.028  2.526  2.131  2.781  3.486  5.06  4.01  2.524  2.006  4.249  4.694  3.582  3.945  2.866  3.853  4.954  6.928  5.57  3.637  3.35  4.316  2.633  2.375  4.629  4.444  3.892  3.115  3.393  5.743  2.937  3.074  3.376  2.707  3.75  3.366  
 
 TMEM63A  9725  1  226033232  226070420  1q42.12  1.378  0.819  1.323  1.346  2.613  0.518  2.012  1.576  1.213  2.458  1.676  3.127  2.781  1.094  2.86  3.252  2.376  0.12  1.671  0.734  2.218  1.509  2.046  3.651  1.057  2.717  2.558  2.946  2.344  2.44  2.213  2.15  3.333  0.923  0.729  2.354  2.828  2.655  0.742  2.12  3.192  1.488  2.01  1.653  1.167  1.601  1.535  1.725  1.493  2.987  2.078  1.568  1.495  0.746  1.624  2.522  1.992  1.348  1.771  1.802  
 
 LEFTY1  10637  1  226073981  226076846  1q42.1  0  0.066  0.138  0  0.06  0.079  0.189  0.076  0.545  0.196  0.255  0.163  0  0.143  0  0.073  0  0.212  0.053  0.172  0  0.268  0.745  0.684  0  0.055  0.329  0  0  0.199  0  0.113  0  0  0  0  0  0  0.117  0  0  0.669  0.29  1.267  0.204  0.132  0.905  0.109  0  0  0.085  0.784  0.043  0.218  0.157  0.195  0  0  0  0  
 
 PYCR2  29920  1  226107576  226112040  1q42.12  2.635  2.744  3.196  3.723  3.589  3.152  4.52  3.586  4.243  4.27  4.304  4.049  4.568  4.158  4.248  3.974  4.932  2.289  5.987  3.724  5.197  4.289  4.707  5.146  3.425  4.797  3.868  4.65  4.302  3.164  4.666  4.789  4.411  3.099  3.441  3.985  4.857  4.53  2.592  4.4  4.19  4.809  4.77  3.405  3.062  3.237  2.972  3.885  2.891  5.421  4.493  3.476  3.817  3.38  4.545  4.479  3.384  4.733  4.104  3.751  
 
 LEFTY2  7044  1  226124297  226129083  1q42.1  0  0  0.236  0  0.051  0  0  0.063  0.482  0.055  0.059  0.059  0  0.027  0  0.055  0  0.117  0  0.069  0  0.163  1.436  2.92  0  0  0.126  0  0  0.106  0  0  0  0.215  0  0  0  0  0.052  0  0  0.397  0.25  0.102  0.091  0.082  0.046  0.087  0  0  0  0.536  0  0.119  0.051  0  0  0  0  0  
 
 SDE2  163859  1  226170402  226187066  1q42.12  0.645  1.182  0.721  1.227  1.523  1.025  1.608  1.381  1.241  1.43  1.83  1.025  2.006  1.47  1.519  1.202  1.459  1.659  2.627  1.35  2.386  1.578  1.355  1.286  1.313  1.831  0.992  1.216  0.99  0.916  1.513  1.894  0.818  0.691  0.649  1.509  1.85  1.576  0.959  1.814  1.378  1.098  1.535  1.763  0.94  1.076  0.768  1.184  1.169  2.323  1.922  1.001  1.046  1.13  1.13  1.505  1.332  1.386  1.567  1.547  
 
 H3F3A  3020  1  226250407  226259703  1q42.12  0  0  0.535  0  0.467  0  0  0  0  0  0  0  0  0.336  0  0  0  0  0  0  0  0  0  0  0  0  0  0  0  0  0  0  0  0  0  0  0  0  0  0  0  0  0  0  0  0  0.13  0  0  0  0.57  0  0  0  0  0  0  0  0  0  
 
 H3F3AP4  440926  1  226250427  226259703  2q31.1  6.17  7.981  5.102  6.076  8.181  6.159  7.039  6.456  6.777  7.17  6.94  7.234  6.147  7.334  6.161  7.433  7.669  8.955  9.924  8.59  7.426  8.605  6.843  6.995  6.416  6.138  5.918  5.481  5.209  4.376  6.574  6.532  5.544  4.99  6.314  6.864  6.192  6.476  5.69  6.669  6.842  6.689  7.926  7.27  6.62  6.246  6.836  6.013  6.407  8.666  6.375  5.616  5.715  5.193  6.88  7.072  5.659  7.09  6.67  6.561  
 
 ACBD3  64746  1  226332379  226374423  1q42.12  1.705  2.731  2.043  2.583  2.817  1.586  2.47  2.93  2.426  2.831  2.873  2.251  2.558  2.547  2.315  2.084  2.411  2.16  2.847  1.927  2.267  1.583  1.762  1.638  2.078  3.192  1.806  2.334  2.078  1.801  2.478  2.659  2.329  1.729  1.753  2.243  2.409  2.783  2.123  2.512  2.429  1.828  2.379  2.435  1.721  1.745  1.243  2.013  2.077  3.31  3.808  1.473  2.201  2.669  1.989  3.504  2.54  1.87  2.401  2.602  
 
 MIXL1  83881  1  226411318  226414756  1q42.12  0  0.056  0.122  0.175  0.04  0.102  0  0.226  0.073  0.034  0.039  0.125  0  0.044  0  0.072  0.07  0.161  0.091  0.05  0  0.166  0.502  0  0  0.048  0.224  0.045  0  0.082  0  0  0  0.254  0  0  0.247  0.089  0.099  0.284  0  0.145  0.854  0  0  0.061  0.057  0  0.088  0.053  0.042  0.617  0.043  0.125  0.065  0.105  0  0  0  0  
 
 LIN9  286826  1  226418849  226497449  1q42.12  0.618  1.561  0.547  0.977  1.072  0.662  0.979  0.653  0.596  1.141  1.422  0.864  0.907  0.989  0.974  1.317  1.365  2.018  2.466  1.127  1.279  0.97  0.795  0.652  1.018  0.87  0.482  0.534  0.721  0.348  1.13  0.933  0.814  0.534  0.753  1.322  1.056  1.063  0.733  1.627  1.228  0.927  1.889  1.135  0.58  0.849  0.132  0.889  0.784  1.924  1.806  0.664  0.886  0.816  0.963  0.84  1.009  1.126  1.355  1.003  
 
 PARP1  142  1  226548391  226595801  1q41-q42  3.843  3.351  2.622  3.795  4.453  3.547  4.78  4.042  3.585  4.838  4.874  3.321  4.26  5.33  4.524  3.574  4.188  2.992  5.545  4.172  4.784  3.683  4.465  5.149  3.502  4.6  3.972  4.741  4.243  4.244  4.916  4.6  4.503  2.352  3.261  4.433  4.719  4.731  2.608  5.446  4.127  3.599  5.202  5.013  3.739  3.517  2.465  4.264  4.143  5.541  5.006  3.712  3.814  3.648  4.038  4.329  3.964  3.937  4.211  4.282  
 
 C1orf95  375057  1  226736500  226796915  1q42.12  0.028  0  0.189  0.08  0.079  0.025  0.057  0.043  0.186  0.133  0.036  0.084  0  0.033  0  0.111  0.144  0.089  0.023  0.058  0.097  0.105  0.502  0.097  0.027  0.066  0.096  0.043  0.046  0.075  0.14  0.088  0.036  0.225  0.085  0.05  0.018  0  0.074  0.056  0.037  0.248  0.091  0.114  0.069  0.096  0.036  0.048  0.068  0.037  0.03  0.565  0.024  0.075  0.143  0.057  0.026  0.032  0.019  0.034  
 
 ITPKB  3707  1  226819390  226926876  1q42.13  0.767  0.064  0.185  0.131  1.191  0.435  0.918  1.083  1.936  2.363  1.542  0.889  0.637  0.52  0.999  0.649  0.753  0.157  3.295  1.044  0.048  1.307  0.835  0.864  0.578  3.808  2.314  1.509  3.273  2.687  4.694  2.619  2.588  1.346  0.116  0.995  0.498  0.26  0.143  1.106  1.528  0.761  0.063  1.203  0.753  0.495  0.239  0.327  0.27  0.705  1.162  0.851  0.313  0.716  0.583  0.631  0.294  0.025  0.75  0.57  
 
 ITPKB-IT1  100506443  1  226843780  226862769  1q42.13  0  0.082  0  0  0.043  0  0  0  0.264  0.156  0.067  0  0  0.042  0  0  0  0.156  0  0.067  0  0.096  0.488  0.083  0  0.411  0.052  0.085  0.301  0.152  0.413  0.103  0.154  0.206  0  0.106  0  0  0  0  0  0.132  0  0  0  0.13  0.035  0  0  0.072  0  0.355  0  0  0  0  0  0  0  0  
 
 PSEN2  5664  1  227058272  227083804  1q31-q42  0.978  1.671  1.019  1.61  2.444  0.801  2.162  1.222  2.285  1.88  1.973  1.414  1.887  1.78  1.574  1.347  0.994  0.757  0.063  0.374  1.735  0.232  1.123  1.611  0.167  3.231  3.156  2.606  3.236  3.153  5.043  3.279  2.176  1.043  1.838  2.805  1.854  2.902  0.92  2.303  2.033  1.753  2.927  1.906  0.587  1.106  0.793  1.149  1.975  2.734  2.852  1.489  1.286  1.611  2.014  1.199  1.26  1.098  1.595  1.575  
 
 ADCK3  56997  1  227127937  227175246  1q42.13  1.842  0.91  0.826  2.061  2.085  0.834  1.587  0.809  1.597  2.78  1.21  3.116  2.449  2.672  2.479  1.976  3.124  0.4  3.109  3.614  2.266  2.832  3.659  2.907  1.395  1.797  3.085  1.427  1.955  1.791  2.075  2.631  2.406  1.724  1.594  1.031  1.707  2.354  1.03  2.669  1.141  2.08  2.214  1.828  1.314  1.011  1.321  1.75  0.536  3.257  2.079  1.183  0.849  0.902  1.468  1.733  0.875  1.274  1.951  1.341  
 
 CDC42BPA  8476  1  227177565  227505826  1q42.11  0.999  2.555  1.613  1.861  2.64  1.219  2.351  2.375  2.212  2.392  2.332  2.564  1.631  2.777  1.945  2.397  1.22  1.358  0.87  0.491  2.441  0.847  0.171  0.012  1.721  2.628  1.232  1.706  1.456  1.345  1.815  1.698  1.389  0.67  1.318  2.776  2.375  2.706  1.238  2.271  2.136  1.686  2.14  1.843  1.273  1.16  0.822  1.901  1.472  2.332  2.843  1.497  1.365  1.761  2.309  2.287  1.988  1.818  1.993  1.839  
 
 ZNF678  339500  1  227751219  227865144  1q42.13  0.55  0.683  0.199  0.271  0.937  0.536  0.131  0.343  0.459  0.49  0.746  0.365  0.498  0.766  0.586  0.462  0.518  0.604  1.337  0.395  0.444  0.757  0.411  0.161  0.655  0.787  0.225  0.349  0.207  0.357  0.677  0.597  0.259  0.193  0.369  0.948  0.388  1.052  0.247  0.681  0.773  0.225  1  1.139  0.238  0.331  0.272  0.377  0.348  0.666  1.205  0.48  0.416  0.655  0.653  0.382  0.753  0.388  0.839  0.619  
 
 LOC100130093  100130093  1  227916239  227922055  1q42.13  1.361  0.088  0.614  1.97  1.745  1.351  0.992  1.276  2.053  1.04  1.238  0.743  1.201  0.571  1.808  0.897  0.674  0  1.577  0.402  0.888  0.856  1.285  1.317  0.569  1.358  1.078  1.507  1.533  1.651  1.857  1.915  1.545  1.026  0.764  0.988  1.464  1.266  0.428  1.885  1.54  2.493  2.053  1.094  0.706  0.97  0.656  1.447  0.581  2.025  1.959  1.132  1.211  0.928  1.614  1.152  0.964  1.241  1.327  0.874  
 
 JMJD4  65094  1  227918889  227923112  1q42.13  1.995  0.563  1.635  2.648  3.355  1.791  3.154  2.017  2.315  2.634  2.421  2.347  2.689  2.112  2.567  2.525  2.203  0.131  3.991  2.678  2.75  3.024  2.207  2.529  2.423  3.028  2.41  2.944  2.603  2.702  3.315  4.016  2.93  1.581  2.103  2.668  2.983  3.134  1.705  3.377  3.56  3.459  3.724  2.068  1.714  2.093  1.584  2.103  1.85  4.119  3.437  2.086  2.666  2.184  2.582  3.332  1.928  2.352  2.646  1.92  
 
 SNAP47  116841  1  227922696  227968932  1q42.13  2.403  1.824  2.047  3.128  3.154  1.978  3.6  2.761  2.959  3.03  3.331  1.82  2.474  2.838  2.606  2.32  2.481  1.627  3.803  1.159  2.349  2.315  2.12  2.022  2.066  2.527  2.437  2.612  2.229  2.581  2.674  2.991  2.486  1.409  1.92  2.779  3.5  3.475  1.252  3.58  3.01  2.841  3.953  2.144  2.211  1.989  1.311  3.113  1.802  4.636  3.694  2.455  2.73  2.181  2.833  2.924  1.962  2.301  2.787  2.26  
 
 PRSS38  339501  1  228003417  228034171  1q42.13  0  0  0.171  0  0.062  0  0  0  0  0.047  0.067  0  0  0  0  0  0  0.133  0  0.094  0  0.136  0.355  0  0  0  0.171  1.118  0  0.133  0  0  0  0.171  0  0  0  0  0.109  0  0  0.637  0.04  0  0  0  0  0  0  0  0  0.237  0.136  0.086  0  0.163  0  0  0  0  
 
 WNT9A  7483  1  228109164  228135676  1q42  0.202  0.106  0.178  0  0.29  0  0.148  0.185  0.312  0.454  0.366  0.16  0  0.271  0  0.175  0  0.435  0  0.18  0  0.337  0.628  0.254  0  0  0.405  0.665  0  0.361  0  0.127  0  0.185  0.137  0.329  0  0  0.184  0.849  1.243  0.37  0.151  0.296  0  0.276  0.126  0.259  0.48  0.091  0.83  1.595  0.093  0.555  0.218  0.319  0  0.225  0  0.099  
 
 WNT3A  89780  1  228194722  228248972  1q42  0.293  0.067  0.244  0.226  0.263  0.118  0.366  0.212  0.386  0.279  0.267  0.194  0.175  0.559  0.143  0.324  0.212  0.193  0.311  0.173  0  0.677  0.685  0.209  0.135  0.097  0.367  0.151  0.227  0.395  0.131  0.19  0.087  0.219  0.239  0.151  0.166  0.205  0.287  0.22  0.15  0.483  0.217  0.138  0.053  0.254  0.412  0.311  0.089  0.426  0.208  0.927  0.234  0.373  0.373  0.244  0.144  0.082  0.166  0.053  
 
 ARF1  375  1  228270360  228286913  1q42  6.515  6.735  6.26  7.184  7.189  5.794  7.871  6.679  6.961  7.043  6.987  6.753  6.849  7.232  6.516  6.63  6.938  6.177  7.624  6.2  6.529  6.318  7.034  6.568  6.393  6.83  7.035  6.314  6.436  6.46  6.666  7.166  6.691  5.36  5.846  6.599  7.283  7.512  5.714  6.883  6.76  7.315  6.87  6.227  5.517  5.607  5.374  6.888  5.575  8.097  7.694  5.642  6.695  6.341  6.831  7.514  5.998  6.796  6.736  6.328  
 
 C1orf35  79169  1  228288427  228291163  1q42.13  2.88  1.333  1.821  3.076  3.842  2.404  3.549  2.042  2.84  2.976  3.076  2.431  2.788  3.063  2.383  3.452  3.089  0.528  4.976  3.27  2.913  4.026  3.334  2.791  2.268  2.38  2.808  2.399  1.798  1.908  2.815  2.682  1.991  1.355  2.798  2.536  2.721  3.233  2.378  3.048  3.105  4.145  4.363  2.78  2.129  2.108  2.962  3.101  2.507  4.627  3.218  2.175  3.111  2.4  3.989  2.993  2.366  3.053  3.481  2.212  
 
 MRPL55  128308  1  228294379  228297013  1q42.13  4.821  3.512  4.286  5.074  5.9  4.633  6.923  4.788  4.646  5.293  4.775  4.331  5.023  5.031  4.569  4.909  5.07  2.27  6.652  6.805  4.84  6.253  4.962  5.262  4.594  4.523  5.538  4.552  4.863  5.424  5.082  5.602  4.094  2.821  5.068  4.607  5.413  5.253  4.847  5.122  5.423  5.587  6.536  4.457  4.348  4.632  6.979  5.487  3.485  6.991  5.398  4.123  5.374  4.58  6.031  5.631  3.748  5.683  5.187  4.012  
 
 GUK1  2987  1  228327784  228336655  1q32-q41  5.628  2.371  5.239  6.018  6.232  5.076  7.878  5.696  6.235  6.77  6.261  5.198  6.262  6.002  5.469  6.252  6.131  0.439  8  5.232  5.565  6.579  5.776  5.999  5.539  5.525  6.651  5.387  5.592  6.23  6.192  6.802  5.853  4.259  4.476  5.992  5.987  7.318  5.165  6.015  5.959  6.285  6.583  5.157  4.74  5.433  5.235  6.277  4.27  7.969  6.848  5.088  6.359  5.585  6.246  6.653  5.883  6.542  6.327  5.692  
 
 GJC2  57165  1  228337414  228347527  1q42.13  0.075  0.08  0.347  0.627  0.195  0.293  0.406  0.31  0.675  0.579  0.402  0.123  1.433  0.193  0.189  1.133  0.188  0.299  1.606  0.17  0  0.582  0.551  0.075  0.203  0.122  0.532  0.152  0.151  0.185  0.208  0.141  0.263  0.094  0.125  0.183  0.467  0.396  0.205  0.274  0.236  0.896  0.144  0.511  0.253  0.26  0.09  0.392  0.091  0.248  0.524  1.006  0.204  0.391  0.201  0.206  0.248  0.285  0.151  0.164  
 
 C1orf148  574432  1  228351786  228353213  1q42.13  0.533  0.458  0  0  0.718  0  0.393  0.283  0  0.478  0.561  0.37  0.473  0.368  0.679  0.544  0  0  1.274  0  0  0.576  0.713  0.779  0  0.676  0.871  0.354  0.464  0  0.873  0  0.556  0  0.321  0.595  0.794  0.618  0.209  0.727  0.893  0.44  0.733  0.346  0  0.251  0.117  0.826  0  1.124  0.706  0.694  0.593  0.165  0.458  0.583  0.606  0.534  0  0  
 
 IBA57  200205  1  228353508  228369958  1q42.13  0.602  0.171  0.405  0.385  1.191  0.403  0.58  0.613  0.431  0.602  0.574  1.025  0.829  0.788  0.888  0.838  0.767  0.128  1.304  0.509  0.782  0.984  0.921  0.466  0.589  0.478  0.852  0.615  0.585  1.037  0.827  0.96  0.956  0.435  0.399  0.572  0.699  0.713  0.215  0.834  0.627  0.931  0.696  0.914  0.407  0.404  0.892  1.114  0.49  1.567  0.754  0.733  0.579  0.372  0.544  0.784  0.559  0.564  0.666  0.446  
 
 C1orf145  574407  1  228391206  228401365  1q42.13  0.66  0  0.281  0  1.116  0  0.278  0.685  0.409  0.265  0.167  0.09  0.7  0.406  0.476  0.423  0.375  0.146  0.687  0.073  0  0.45  0.612  0.126  0  0.17  0.437  0.056  0.44  0.415  0.511  0.403  0  0.226  0.275  0.616  0.211  0  0.642  0.65  0.623  0.681  1.005  0.295  0.287  0.616  0.945  0.481  0.279  1.779  0.196  1.113  0.123  0.767  0.508  0.351  0.407  0  0.406  0  
 
 OBSCN  84033  1  228395830  228566575  1q42.13  1.377  0.059  0.188  0.494  0.875  0.168  0.105  0.625  0.245  0.243  0.082  0.139  1.224  0.48  1.109  0.228  0.336  0.204  1.271  0.166  0.444  0.682  0.634  0.179  0.289  0.37  0.774  0.128  0.255  0.331  0.71  0.312  0.026  0.203  0.544  1.063  0.597  0.039  0.299  1.409  0.36  1.372  0.412  1.155  0.421  0.866  0.677  1.921  0.308  1.531  0.33  1.184  0.262  0.75  1.187  0.613  0.828  0.131  1.32  0.09  
 
 TRIM11  81559  1  228581376  228594517  1q42.13  1.69  0.682  1.802  3.224  2.809  1.415  2.754  2.268  2.323  2.476  2.664  1.904  2.485  2.248  2.334  2.5  2.528  0.536  3.915  1.684  2.695  2.109  2.744  1.837  2.233  2.379  2.043  2.493  1.888  1.853  2.577  2.929  2.497  1.853  1.641  2.339  2.945  2.722  1.382  2.783  2.575  3.085  2.729  2.023  1.919  2.109  1.421  2.629  1.689  4.298  2.354  2.161  2.21  2.034  2.55  2.33  1.991  2.125  2.773  2.289  
 
 TRIM17  51127  1  228595635  228604583  1q42  0.343  0  0.392  0.43  1.867  0.496  0.317  0.458  0.283  0.939  0.516  0.173  0.43  0.411  0.631  0.385  0.13  0.171  0.775  0.197  0.308  0.411  0.666  0.316  0.075  0.94  0.442  0.425  0.32  0.298  0.41  0.26  0.668  0.273  0.252  0.779  2.171  1.255  0.104  0.694  0.429  0.739  1.323  0.513  0.445  1.004  0.031  1.281  0.501  1.85  0.45  1.024  0.626  0.644  1.006  0.505  0.639  0.1  0.472  0.437  
 
 HIST3H3  8290  1  228612545  228613026  1q42  1.249  0  0.856  0  0.742  1.717  0.271  0.39  0  0.553  0.395  1.546  0.423  0.885  0.585  0.817  1.258  0.347  0.271  0.784  0  1.899  0.739  0.597  0  0.288  0.613  0.203  0.482  0.349  0  0  0  0  1.207  0.823  0.258  0  0.609  0  0.477  0.754  0.331  0.626  1.045  0  0  0.658  0  0.392  0.505  2.143  0.727  0.599  1.007  0  0.401  0  0.551  0  
 
 HIST3H2A  92815  1  228645064  228645560  1q42.13  2.807  0.291  1.144  4.096  3.273  2.896  0.951  0.634  4.033  1.328  1.674  2.09  2.359  3.633  1.63  3.311  3.919  0.789  1.149  1.694  2.723  4.628  4.693  3.355  1.392  1.552  3.132  1.834  1.496  2.92  2.643  2.395  1.678  3.003  2.77  2.258  2.446  0  1.4  2.13  2.567  4.638  4.421  2.461  1.572  2.196  1.099  2.758  2.092  3.065  1.364  2.812  1.035  2.168  2.587  1.634  0  1.371  0.663  0.846  
 
 HIST3H2BB  128312  1  228645807  228646259  1q42.13  2.775  0.656  0.71  2.304  2.504  1.358  0.926  0.732  1.486  0.98  1.97  3.829  1.101  2.724  2.249  3.927  4.232  0.748  0.455  3.201  2.556  5.286  1.346  4.551  1.517  1.391  1.76  1.222  1.718  0.519  2.222  2.126  1.505  0.675  3.265  3.03  2.268  0  2.026  3.15  3.287  3.331  3.173  3.895  2.528  3.122  1.641  3.392  2.387  1.458  1.146  3.211  0.839  3.338  2.143  1.525  0  2.382  1.541  0  
 
 RNF187  149603  1  228675067  228683889  1q42.13  3.152  2.458  3.94  4.79  4.942  2.955  4.348  3.701  6.29  4.469  3.931  3.429  3.994  3.599  3.976  3.122  4.374  2.792  5.647  3.265  3.421  4.349  5.42  3.871  3.619  4.754  4.136  4.052  3.8  4.189  4.276  5.074  3.528  2.814  3.359  4.772  3.981  4.663  3.4  4.398  3.461  4.785  6.365  3.33  2.754  3.68  2.241  3.995  3.384  5.957  4.23  3.607  3.85  4.141  4.341  4.21  3.42  4.304  4.138  3.692  
 
 BTNL10  100129094  1  228698059  228699989  1q42.13  0  0  0  0  0  0  0  0  0  0  0  0  0  0.167  0.231  0  0  0  0  0  0  0.124  0.592  0  0  0  0.081  0  0  0.078  0  0  0  0.344  0  0  0  0  0.105  0  0  0  0.654  0  0  0  0  0  0  0  0  0.284  0  0  0  0  0  0  0  0  
 
 RHOU  58480  1  228780393  228882416  1q42.11-q42.3  0.397  0.631  0.279  0.461  0.064  0.111  0.131  0.35  0.484  1.06  0.337  1.337  2.314  0.694  0.713  1.361  2.212  0.294  2.316  2.157  0  3.689  0.369  0.049  0.094  0.056  0.283  0.021  0.041  0.218  0.082  0.172  0  0.221  0.436  1.695  0.185  1.157  0.216  2.483  0.496  0.572  1.227  0.511  0.164  0.236  1.156  0.058  0.351  0.036  0.335  0.73  0.037  0.377  0.62  0.088  0.401  0  0.288  0.06  
 
 DUSP5P1  574029  1  228780656  228788159  1q42.13  0  0.5  0.504  0.441  0.138  0.158  0.056  0.588  0.276  0.58  0.258  0.061  0.514  0.379  1.391  0.095  0.058  0  0.679  0.424  0  0.414  0.664  0  0.388  0.143  0.221  0.312  0.18  0.204  0.219  0  0.297  0.354  0.236  0.445  0.164  2.129  0.055  0.185  0.166  0.423  1.21  0.075  0  0.034  0.109  0.598  0  0.517  0.378  0.561  0.101  0.114  0.959  0.121  0.88  0  0.553  0  
 
 RAB4A  5867  1  229406808  229441640  1q42-q43  1.689  2.599  1.39  1.498  2.473  1.171  2.465  1.456  2.506  2.226  2.414  2.257  2.118  3.173  1.846  1.745  2.014  3.053  3.503  3.211  1.654  2.078  1.133  0.721  1.39  1.935  1.409  1.134  1.178  1.297  2.643  2.404  0  0.224  1.486  2.602  1.822  1.952  1.243  2.197  2.036  1.921  2.988  1.381  1.402  1.802  2.033  1.687  1.797  3.638  1.978  1.115  1.465  1.167  2.009  2.224  1.25  1.353  1.585  1.648  
 
 CCSAP  126731  1  229456751  229478688  1q42.13  0.748  1.946  1.253  0.954  1.105  0.862  1.334  1.195  1.165  1.604  1.732  0.55  1.125  1.727  1.51  0.993  1.362  1.672  3.019  1.184  0.923  1.95  0.787  0.497  0.675  1.683  1.288  1.045  1.417  0.876  1.899  1.835  1.122  0.703  0.729  1.783  0.922  1.453  0.734  1.541  1.265  0.957  2.279  1.268  0.703  0.806  0.647  0.649  1.151  1.225  1.221  0.57  1.058  0.865  0.873  1.202  1.207  0.618  0.898  0.736  
 
 ACTA1  58  1  229566992  229569843  1q42.13  1.295  0.391  2.741  1.796  1.244  1.685  2.199  2.336  2.11  1.917  2.011  1.389  1.42  1.155  1.117  1.329  1.865  0.561  1.682  1.143  0.824  1.869  0.677  0.906  1.453  1.394  2.77  1.245  2.142  3.144  1.531  1.262  1.731  1.268  1.546  1.455  1.989  1.515  1.616  1.587  0.955  1.773  2.036  1.026  1.025  1.272  0.429  1.414  0.976  1.512  1.637  1.467  2.549  1.679  2.19  2.337  1.252  1.444  1.788  1.046  
 
 NUP133  55746  1  229577043  229644088  1q42.13  1.508  2.702  1.065  1.568  2.23  1.145  1.749  1.42  1.754  2.213  2.296  1.234  2.281  2.556  1.999  1.77  2.228  2.757  3.581  2.094  2.366  2.071  1.687  1.554  1.331  2.16  1.403  1.486  1.433  1.58  2.15  1.857  1.4  0.849  1.328  2.584  2.163  2.145  1.086  2.553  1.903  1.523  2.512  2.099  1.474  1.191  1.179  1.525  1.616  3.216  2.364  1.237  1.568  1.12  1.958  1.696  1.869  1.447  2.149  2.216  
 
 LOC101927478  101927478  1  229644176  229649834  -  0  0  0  0  0.199  0  0  0  0  0  0  0.335  0  0  0  0  0  0  0  0  0  0.132  0  0  0  0  0.103  0  0  0  0  0  0  0  0.394  0  0  0  0  0  0  0.461  0  0  0  0  0  0  0  0  0  0.518  0  0  0  0  0  0  0  0  
 
 ABCB10  23456  1  229652328  229694442  1q42.13  0.698  2.577  0.66  0.745  1.272  0.924  0.831  0.745  1.115  1.385  1.369  0.83  1.628  1.955  1.486  1.168  1.252  2.139  3.572  3.383  2.021  1.486  1.298  1.093  0.699  1.049  0.425  0.875  0.88  0.656  1.375  1.485  0.869  0.782  0.726  2.152  1.408  1.873  1.05  1.176  1.293  1.935  1.568  1.723  0.413  0.779  0.527  0.993  1.57  1.944  1.738  0.937  0.638  0.778  1.291  1.254  1.308  1.226  1.377  1.408  
 
 TAF5L  27097  1  229728865  229761794  1q42.13  1.912  2.875  1.366  1.449  2.144  1.817  2.074  2.634  1.193  2.088  2.292  2.01  2.343  2.303  2.043  2.368  1.673  2.625  2.636  2.613  2.238  2.649  1.455  2.145  2.076  1.937  1.765  2.11  2.054  1.839  1.966  2.15  2.052  0.929  1.707  2.173  2.537  2.669  1.607  2.511  1.986  1.367  2.413  2.551  1.847  1.211  1.463  2.201  1.753  2.141  1.964  1.599  2.242  1.638  2.13  2.576  1.841  2.023  2.315  2.101  
 
 URB2  9816  1  229761980  229795946  1q42.13  1.193  1.195  0.914  1.283  1.527  0.864  1.756  1.29  1.176  1.191  1.688  1.124  2.107  1.687  2.153  1.475  0.928  0.777  2.666  1.149  2.155  1.628  1.179  1.689  1.289  1.802  1.154  2.004  1.321  1.23  1.682  1.841  1.728  0.763  1.16  2.315  2.664  1.83  0.601  2.508  1.409  1.192  1.694  1.99  1.376  0.878  0.644  1.743  1.245  2.094  1.863  1.264  1.653  0.966  1.155  1.488  1.353  1.087  1.814  1.496  
 
 GALNT2  2590  1  230193535  230417876  1q41-q42  1.388  2.267  3.501  3.972  2.986  3.307  4.288  5.046  3.515  5.319  3.84  2.266  2.989  2.525  2.577  2.538  2.468  1.195  5.183  2.005  3.4  3.611  2.429  2.69  2.781  4.357  4.544  4.279  4.784  4.822  5.104  5.122  4.128  2.386  2.97  5.03  4.304  5.134  2.92  4.222  4.081  3.595  2.303  4.048  2.907  2.329  2.391  3.317  3.096  4.409  4.586  3.457  3.814  3.715  3.567  4.067  4.017  3.561  3.408  4.275  
 
 PGBD5  79605  1  230457391  230561674  1q42.13  0.472  0  0.215  0  0.138  0.091  0.076  1.606  1.155  0.57  0.191  0.048  0.138  0.043  0  0.107  0.049  0.161  2.217  0.467  0.067  0.245  0.368  0.214  0.055  0.775  0.227  0.349  0.364  0.423  0.432  0.298  0  0.11  0.082  0.309  0  0.094  0.29  0.354  0.151  0.698  0.172  2.903  0.117  0.153  1.033  0.112  0.271  0.039  0.063  0.829  0.891  1.162  1.214  0.14  2.898  0  1.671  0.099  
 
 COG2  22796  1  230778201  230829731  1q42.2  1.983  1.955  0.826  1.398  2.707  1.119  1.925  1.478  1.966  1.627  1.796  1.85  1.875  2.664  1.422  2.023  2.447  2.386  3.824  2.508  1.998  2.725  1.849  1.807  1.669  2.686  1.285  1.795  1.782  1.714  1.999  1.839  1.293  0.92  1.28  2.591  2.04  2.258  1.429  2.36  2.05  1.822  2.138  1.904  1.254  0.857  1.77  1.282  1.173  3.209  2.601  1.258  1.433  1.202  2.331  1.853  1.878  1.071  1.93  1.817  
 
 AGT  183  1  230838271  230850336  1q42.2  0  0  0.354  0.348  0.054  0.078  0.268  1.832  0.72  0.672  0.116  2.334  0  0.045  0  0.065  0  0.215  0  0.06  0  0.145  0.401  0.041  0  0.294  0.107  1.255  0  0.147  0.154  0.113  0  0.266  0.078  3.215  0  2.911  0.043  0  0  0.332  0.025  0.08  0  0.149  0.881  0  0  0.068  0.062  0.309  0.124  0.575  0.084  0.105  1.387  0  0.31  0  
 
 CAPN9  10753  1  230883129  230937749  1q42.11-q42.3  0.17  0  0.068  0  0.293  0  0  0  0.057  0.083  0  0.142  0  0.027  0  0.205  0.048  0.194  0.033  0  0  0.117  0.277  0.045  0  0.055  0.088  0.032  0  0.099  0  0.067  0  0.076  0.047  0.078  0  0  0.064  0  0.068  0.276  0.052  0.058  0  0.161  0.03  0  0  0.039  0.074  0.351  0  0.123  0  0.09  0  0  0  0  
 
 C1orf198  84886  1  230972864  231005335  1q42.2  1.97  1.422  4.547  3.409  3.224  3.436  2.272  4.038  2.396  4.14  3.02  2.07  2.901  2.75  2.976  2.959  2.946  1.272  3.098  1.225  1.703  1.683  0.892  0.618  1.656  3.957  2.125  4.392  1.852  2.812  2.327  3.313  1.899  1.23  2.113  3.568  2.786  2.353  1.674  1.36  3.236  2.453  2.927  3.015  1.966  3.242  1.333  2.27  1.85  3.779  2.589  1.974  3.113  2.784  2.7  2.769  3.144  1.699  3.056  3.259  
 
 LOC101927604  101927604  1  231010591  231014761  -  0  0  0  0  0.242  0  0  0.181  0  0.096  0  0  0  0.131  0  0  0.213  0.177  0  0  0  0.104  0  0  0  0  0  0.115  0  0.061  0  0  0  0  0  0  0.22  0  0.112  0  0  0.545  0.61  0  0  0.198  0  0  0  0.275  0.15  0  0  0  0.128  0  0  0  0  0  
 
 TTC13  79573  1  231041986  231114618  1q42.2  1.015  2.553  0.534  1.298  1.536  0.726  1.392  1.388  1.185  1.799  1.46  1.036  1.514  1.212  1.495  1.379  1.311  1.775  2.875  1.671  0.507  1.408  1.344  1.382  0.346  1.634  0.948  1.42  1.004  0.774  1.142  0.663  0.621  0.414  0.963  2.463  1.585  1.291  0.742  1.542  1.545  1.134  1.544  1.592  0.62  1.218  1.307  0.862  0.623  1.542  1.312  0.799  0.879  0.729  1.192  1.283  1.324  0.568  1.401  1.206  
 
 ARV1  64801  1  231114822  231136479  1q42.2  2.571  4.15  1.301  1.81  3.555  2.01  3.467  2.275  2.343  3.035  2.594  2.327  1.779  1.531  1.618  2.283  1.444  5.025  3.819  4.671  3.428  2.716  1.808  2.073  2.082  2.807  2.502  1.875  2.944  2.646  3.552  3.314  2.668  2.246  2.332  3.165  2.108  3.034  2.948  2.013  2.767  1.961  3.755  1.248  1.5  1.97  3.852  1.372  1.081  3.259  2.066  1.534  1.643  2.017  2.805  1.778  1.557  1.895  1.944  2.038  
 
 FAM89A  375061  1  231154703  231175995  1q42.2  0.954  2.991  1.387  2.283  0.228  0.952  1.771  2.511  2.174  2.207  1.35  3.281  2.435  3.36  1.592  2.61  2.441  2.432  3.589  1.395  3.177  1.323  1.731  1.558  1.626  3.628  3.86  3.43  3.86  1.729  4.368  4.236  3.379  2.991  2.167  2.94  1.28  1.021  1.086  1.229  1.856  2.16  0.871  2.352  1.186  0.803  2.216  0.656  0.533  2.018  0.75  1.452  1.559  1.373  1.85  0.839  1.311  0.769  1.35  0.288  
 
 TRIM67  440730  1  231298673  231357314  1q42.2  0  0.046  0.195  0.059  0.057  0.022  0.028  0.087  0.14  0.084  0.037  0.051  0.054  0.07  0.042  0.051  0.077  0.105  0.039  0.073  0.031  0.166  0.438  0.049  0.025  0.686  0.104  0.084  0.056  0.157  0.056  0.048  0.017  0.203  0.363  0.091  0.02  0.04  0.071  0.034  0.045  0.188  0.113  0.1  0.342  0.066  0.034  0  0.022  0.014  0.045  0.533  0.037  0.106  0.131  0.036  0.113  0.022  0.061  0.017  
 
 LOC149373  149373  1  231319844  231323373  1q42.2  0  0  0.256  0  0  0  0.278  0  0.2  0  0  0  0  0  0  0  0  0  0  0  0  0.041  0.432  0  0  0.126  0.311  0  0  0.24  0  0  0  0.157  0  0  0  0  0.068  0  0  0.582  0  0  0  0  0.396  0  0  0  0  0.32  0  0.061  0.049  0  0  0  0  0  
 
 C1orf131  128061  1  231359508  231376933  1q42.2  2.38  2.246  1.566  1.819  2.976  0.494  2.582  2.061  1.749  2.361  2.559  2.598  2.227  3.084  1.922  2.393  2.677  3.449  4.339  3.505  3.177  3.296  2.445  2.005  1.628  1.785  1.462  1.068  0.927  0.651  1.941  2.628  1.288  1.002  2.071  2.736  1.581  1.288  1.714  2.126  2.055  2.272  2.775  2.89  0.984  1.699  1.28  1.35  1.565  2.848  1.662  1.304  1.82  1.553  2.317  2.112  1.658  1.711  1.813  2.046  
 
 GNPAT  8443  1  231376918  231413719  1q42  1.82  4.524  1.995  2.175  2.981  2.141  3.127  2.614  2.833  3.266  3.204  2.357  2.696  3.537  2.137  3.141  2.957  4.794  4.641  4.057  2.582  3.248  2.206  2.154  2.106  2.61  2.055  1.634  1.86  1.664  2.773  2.405  1.716  1.005  2.23  3.719  3.091  3.371  2.017  2.931  2.642  2.408  3.582  2.726  1.805  2.482  2.669  2.247  2.073  4.169  3.056  1.909  2.564  1.857  3.115  2.801  2.51  1.794  3.068  2.828  
 
 EXOC8  149371  1  231468476  231473618  1q42.2  0.674  0.816  0.678  0.921  1.235  0.484  0.744  0.889  0.895  1.067  1.033  0.76  1.17  1.28  0.995  0.979  1.018  0.371  1.274  0.822  1.142  0.845  0.826  0.77  0.856  1.527  0.655  0.996  0.794  0.781  1.01  0.885  0.859  0.6  0.774  1.53  1.101  1.513  0.797  1.411  1.065  0.835  0.805  1.002  0.675  1.278  0.484  1.174  0.852  1.48  1.235  1.078  1.043  1.021  0.946  1.027  0.998  0.95  1.205  1.002  
 
 SPRTN  83932  1  231473681  231490773  1q42.12-q43  1.006  2.222  0.776  1.307  1.84  0.823  1.383  1.143  1.062  1.398  1.513  0.967  1.387  1.72  1.012  1.218  0.808  1.868  2.397  1.378  1.27  1.223  0.73  0.885  0.915  1.356  0.724  0.769  0.887  0.635  1.308  1.469  0.758  0.437  0.998  1.777  1.277  1.494  0.966  1.378  1.276  1.358  1.522  1.316  0.736  1.336  1.287  1.667  0.648  3.049  1.148  0.991  1.112  0.835  1.354  1.094  1.084  1.267  1.449  1.253  
 
 EGLN1  54583  1  231499496  231560790  1q42.1  0.833  2.323  1.829  2.525  1.87  1.43  1.606  2.037  2.546  2.247  2.47  0.825  1.895  2.196  1.57  1.344  2.221  1.276  1.987  1.718  1.184  1.119  0.97  0.766  2.06  2.633  1.171  1.791  2.341  1.932  2.162  2.052  1.567  1.017  1.924  2.526  1.66  2.06  2.04  1.767  1.676  1.773  2.031  2.014  1.218  1.752  1.532  1.865  1.513  3.366  1.652  1.805  1.502  1.835  2.284  2.219  2.211  1.689  2.052  2  
 
 SNRPD2P2  645339  1  231611509  231612271  1q42.2  0  0.155  0  0  0  0  0  0  0  0  0  0  0  0.164  0  0  0  0  0.238  0.149  0  0.18  0.556  0  0  0  0.301  0  0  0.167  0  0  0  0.245  0  0  0  0  0.105  0  0  0  0.203  0  0  0  1.41  0  0  0  0  0  0  0.111  0.128  0  0  0  0  0  
 
 TSNAX  7257  1  231664398  231702269  1q42.1  1.969  2.683  1.542  2.269  3.279  1.266  2.617  1.908  2.519  2.459  3.049  1.77  2.32  2.812  1.814  2.346  2.787  2.39  3.981  2.859  2.67  2.55  2.271  1.615  2.273  4.26  1.519  2.436  2.625  1.932  3.339  3.127  2.545  2.109  1.863  3.313  2.417  2.644  2.224  2.735  2.588  1.972  2.783  2.455  1.787  2.604  1.624  1.716  1.521  3.753  2.577  1.695  1.903  1.777  2.626  2.528  2.022  2.217  2.275  2.644  
 
 TSNAX-DISC1  100303453  1  231664398  232177019  1q42.1  0  0  0  0  0  0.116  0  0  0  0  0.151  0  0  0.11  0.076  0  0  0  0  0  0  0.032  0  0  0  0  0.028  0.059  0  0.044  0.109  0.104  0.077  0.102  0  0.085  0  0.072  0.061  0  0  0.039  0.026  0  0  0.201  0.056  0  0  0.111  0  0.09  0.179  0.078  0  0  0.094  0  0  0  
 
 LINC00582  100287814  1  231727037  231747836  1q42.2  0  0  0.316  0  0  0  0  0.16  0  0  0  0  0  0  0  0  0  0  0  0  0  0.084  0  0  0  0  0.063  0  0  0  0  0  0  0  0  0  0  0  0.114  0  0  0.276  0.079  0  0  0  0  0  0  0  0  0  0  0  0  0  0  0  0  0  
 
 DISC1  27185  1  231762560  232177019  1q42.1  0.708  0.353  0.86  0.454  0.089  0.639  0.746  0.574  0.486  0.32  0.192  0.299  0.197  0.408  0.823  0  0.241  0.58  1.137  1.408  0  0.671  0.981  0.411  0.803  2.213  1.339  0.875  0.443  0.601  1.415  0.644  1.16  0.861  0.196  0  1.45  0.347  0.413  0.221  0  0.752  0.263  0.711  1.009  0.754  1.001  1.227  0  0.892  0.683  0.749  0.235  0.422  1.286  1.292  0.826  0.407  0.799  0.834  
 
 DISC2  27184  1  231950371  231954263  1q42.1  0  0.04  0.088  0  0  0.03  0.034  0.025  0.084  0  0  0.04  0  0.014  0  0  0  0.031  0.018  0.044  0  0.023  0.295  0.025  0  0.111  0.087  0  0  0.053  0.096  0  0  0.192  0  0  0.05  0  0.015  0  0  0.073  0.012  0  0  0.057  0.164  0  0  0.018  0.018  0.075  0.016  0  0.04  0.034  0  0  0  0  
 
 DISC1-IT1  104472714  1  232061579  232080951  1q42.2  0  0  0.232  0  0  0  0  0  0  0  0  0  0  0  0  0  0  0  0  0  0  0.103  0  0  0  0.447  0  0  0  0.067  0.385  0  0  0  0  0  0  0  0  0  0  0  0  0  0  0  0  0  0  0.123  0  0  0  0  0  0  0  0  0  0  
 
 SIPA1L2  57568  1  232533711  232651243  1q42.2  0.483  0.035  1.667  1.199  1.058  0.759  2.407  0.57  3.408  3.362  1.592  2.129  0.531  0.826  0.318  0.481  1.501  0.077  0.596  0.161  0.038  1.475  0.584  1.087  0.025  2.273  2.072  1.625  1.794  1.082  1.868  2.614  1.79  0.967  0.833  2.337  0.179  0.041  0.399  1.377  1.46  0.757  0.876  0.198  2.11  1.423  1.43  0.513  0.853  0.649  0.911  0.624  1.629  2.286  0.11  0.599  3.042  0.201  2.12  0.192  
 
 LOC101927683  101927683  1  232862999  232878461  -  0  0  0.156  0  0  0  0  0  0.058  0  0  0  0  0  0  0  0  0  0  0  0  0  0.423  0  0  0  0.028  0  0  0.035  0  0  0  0.11  0  0  0  0  0.021  0  0  0  0.023  0  0  0  0  0  0  0  0  0.089  0  0.028  0  0  0  0  0  0  
 
 MAP10  54627  1  232940637  232946092  1q42.2  0.307  1.153  0.144  0.097  0.127  0.045  0.134  0.315  0.067  0.772  0.071  0.021  0.03  0.026  0  0.022  0  0.151  1.565  0.396  0.073  0.531  0.383  0.221  0  0  0.056  0.09  0  0.047  0  0  0  0.161  0.102  0.797  0.175  0.311  0.029  0.344  0.046  0.152  0.168  0  0.032  0.061  0.366  0.079  0.096  0.092  0  0.34  0  0.053  0.08  0.582  0.587  0.154  0  0  
 
 NTPCR  84284  1  233086369  233114219  1q42.2  3.349  4.231  3.2  3.619  4.394  2.88  4.552  3.227  2.732  3.439  2.634  4.159  3.02  3.599  2.243  3.321  3.911  3.23  3.436  4.604  2.992  3.57  3.41  3.243  3.647  3.242  2.896  2.782  2.92  3.116  3.423  3.634  3.243  2.477  3.141  2.694  2.459  3.026  3.604  3.384  3.577  3.42  4.425  3.183  3.472  3.776  4.728  3.301  2.021  5.474  3.279  2.389  3.42  2.978  4.624  3.866  3.397  3.86  3.607  3.006  
 
 PCNXL2  80003  1  233119881  233431459  1q42.2  0.563  0.327  0.586  0.158  0.89  0.74  1.445  1.061  0.85  1.515  1.365  0.749  0.904  1.086  1.405  0.85  0.944  0.692  1.216  0.14  0.032  1.493  0.568  0.465  0.658  0.504  0.699  0.917  0.675  0.195  0.643  0.902  1.092  0.381  0.82  1.304  0.909  1.547  0.96  0.485  1.498  0.915  0.055  0.773  0.832  1.231  0.762  1.22  1.038  1.686  1.252  1.027  0.93  1.156  1.327  0.838  1.367  0.418  1.516  0.804  
 
 KIAA1804  84451  1  233463513  233520894  1q42  0.606  1.453  0.099  0  0.725  0.023  0.875  0.316  0.648  0.155  1.187  1.125  1.941  2.057  2.366  1.07  1.933  1.828  0.543  1.932  0.058  0.714  1.232  1.359  0.185  0.136  0.267  0.025  0.194  0.462  0.057  0.828  0.537  0.416  1.517  1.719  0.51  0.427  0.442  0.391  1.563  1.058  0.044  1.502  0.645  1.769  1.196  0.996  1.355  1.607  1.279  1.232  1.97  1.492  2.663  1.984  1.674  0.575  1.782  1.481  
 
 KCNK1  3775  1  233749749  233808258  1q42-q43  2.11  0  0.186  0  1.152  0.578  2.598  1.99  0.606  1.549  2.868  2.408  2.686  3.421  2.518  1.844  4.778  0.42  0.797  0.086  1.773  1.4  2.754  0.915  0  0  0.1  0.034  0  0.101  0  0  0  0.169  1.246  0  1.218  0  1.861  0.108  3.543  1.793  0.034  3.336  0.467  1.169  0.966  1.933  3.657  3.33  3.708  1.42  0.301  1.893  0.623  2.789  1.603  0  0.111  0.612  
 
 SLC35F3  148641  1  234040457  234460264  1q42.2  0  1.16  0.141  0.254  0.135  0.606  0.415  0.105  0.299  0.246  0.142  0.228  0.246  1.421  0  0.232  0.819  0.141  0.497  0.124  0.18  0.541  0.465  0.065  0.084  0.058  0.43  0.123  0.044  0.26  0.093  0.044  0.059  0.246  1.287  1.611  1.303  0  0.913  0.136  0.531  0.875  0.207  0.197  0.097  0.13  2.686  1.14  1.32  2.279  1.857  1.601  0.822  1.136  2.098  1.294  1.026  1.674  1.781  1.106  
 
 LOC101927765  101927765  1  234508552  234509339  -  0.949  1.324  0.887  0  2.442  1.509  1.242  0.472  1.623  1.327  1.099  0.575  0.864  1.405  0.49  1.028  1.131  1.214  2.482  1.487  1.401  2.146  1.804  1.757  0.438  1.074  0.411  1.037  1.168  0.49  2.2  1.066  0.555  0.767  2.152  1.208  0.793  0.808  1.515  0.747  1.212  2.405  0  0.927  0.657  1.371  0.085  0.708  0.555  1.768  0.214  0.983  0.711  1.34  1.81  1.188  0.465  0.736  1.016  0.364  
 
 COA6  388753  1  234509182  234519795  1q42.2  2.819  6.053  2.38  4.17  4.142  3.961  3.677  3.095  2.909  3.262  4.104  3.995  3.81  3.923  2.305  3.441  4.013  6.307  4.927  5.356  4.964  3.858  3.716  3.811  3.944  4.058  2.698  2.133  2.728  2.661  4.706  4.453  2.92  2.689  3.902  3.951  3.215  3.455  4.753  3.069  3.578  4.828  4.273  3.684  2.385  3.918  3.464  3.081  2.677  5.041  1.826  2.771  3.022  3.429  4.306  4.407  2.852  4.192  3.815  2.727  
 
 TARBP1  6894  1  234527058  234614849  1q42.3  0.813  2.105  0.735  1.54  1.324  0.679  1.535  0.965  0.802  1.516  1.486  0.759  1.64  0.988  1.461  1.913  1.2  2.866  3.021  2.371  0.944  2.394  0.987  0.691  0.927  2.006  1.319  0.865  0.911  1.332  1.605  1.356  1.299  0.754  0.776  2.342  1.163  1.49  1.053  1.515  1.505  1.595  2.487  1.191  0.549  1.808  1.437  0.855  1.124  2.691  0.773  1.182  0.971  0.91  1.687  1.237  1.301  0.661  0.729  1.03  
 
 LINC01354  100506795  1  234663636  234667525  1q42-q43  0  0.036  0.098  0  0.02  0.11  0  0  0.15  0.036  0.039  0  0  0.034  0  0.039  0  0  0.067  0  0  0.087  0.326  0  0  0  0.072  0.039  0  0.071  0.099  0  0  0.241  0  0  0  0  0  0.082  0.037  0.28  0.066  0  0  0.103  0.035  0  0  0  0  0.105  0.044  0.041  0.032  0  0  0  0  0  
 
 IRF2BP2  359948  1  234740014  234745271  1q42.3  2.054  3.781  3.236  5.414  3.992  2.551  4.199  4.695  4.024  3.845  3.923  3.43  4.076  2.54  3.339  3.573  3.716  4.615  3.863  4.643  2.535  3.136  4.26  3.378  3.168  3.315  3.135  2.766  2.501  2.938  3.519  3.708  2.777  2.326  2.673  4.194  3.64  4.203  2.669  4.191  3.329  3.108  5.228  3.396  2.172  3.454  3.053  2.57  1.969  4.176  2.412  2.455  2.86  2.843  2.632  3.417  3.041  2.689  3.242  3.517  
 
 LINC00184  100302691  1  234765056  234770526  1q42.3  0  0.075  0.093  0  0  0  0.036  0.042  0.072  0.15  0.073  0  0  0  0  0.045  0  0.063  0  0.061  0  0.044  0.449  0  0  0  0.034  0  0  0.06  0  0  0  0.177  0  0.213  0.147  0  0  0  0  0.331  0.036  0  0  0.04  0  0  0  0  0.062  0.158  0.034  0  0.049  0  0.063  0  0  0  
 
 LOC101927787  101927787  1  234782034  234796670  1q42.3  0  0.097  0.16  0  0  0  0  0  0.106  0.067  0  0  0  0  0  0.056  0  0  0  0  0  0.064  0.396  0  0  0  0.063  0  0  0.103  0  0  0  0.202  0  0  0.08  0.33  0  0  0  0  0.055  0  0  0  1.088  0  0  0  0.073  0  0  0.137  0  0  0  0  0  0  
 
 LINC01132  100506810  1  234859788  234867390  1q42-q43  0  0  0.183  0  0.116  0.105  0.143  0.027  0.193  0.046  0.066  0.154  0.098  0.039  0.176  0.243  0.045  0.261  0.066  0.087  0.193  0.168  0.563  0.277  0.067  0.065  0.139  0.078  0.032  0.094  0  0  0  0.181  0.043  0.197  0.098  0.081  0.045  0.317  0.07  0.074  0.191  0.167  0  0.04  0.082  0  0  0.069  0.074  0.248  0.047  0.071  0.036  0.071  0  0  0.053  0.058  
 
 LOC101927851  101927851  1  235093089  235099746  1q42.3  0  0  0  0  0.179  0  0.195  0.186  0.205  0.074  0  0.779  0  0  0  0.252  0  0  0  0  0  0  0.52  0  0  0  0.111  0  0  0.08  0  0  0  0.207  0  0.324  0.215  0.181  0.063  0  0.184  0.349  0.058  0.264  0  0  0.064  0  0  0  0  0.558  0.101  0  0  0  0  0  0  0  
 
 TOMM20  9804  1  235272657  235292256  1q42  3.643  5.99  3.105  3.644  4.866  3.05  4.686  3.511  3.977  4.058  4.644  3.823  3.848  5.211  3.595  4.172  4.507  5.791  5.242  5.134  4.473  3.917  4.52  3.796  4.061  4.426  3.613  3.777  3.326  3.301  4.555  4.634  3.63  3.094  3.401  4.259  4.254  4.502  3.197  3.862  3.97  4.154  5.507  4.656  3.394  4.09  3.952  3.435  3.241  5.436  2.71  2.931  3.866  3.173  4.477  4.753  3.263  4.081  4.365  4.32  
 
 SNORA14B  677802  1  235291117  235291252  1q42.3  0  0  0  0  0  0  0  0  0  0  0  0  0  0  0  0  0  0  0  0  0  0  0  2.356  0  0  0  0  0  0  0  0  0  0  0  0  0  0  0  0  0  0  0  0  0  0  0  0  0  0  0  0  0  0  0  0  0  0  0  0  
 
 RBM34  23029  1  235294497  235324571  1q42.3  1.463  4.322  1.651  2.781  3.206  1.406  2.359  1.795  2.15  2.536  2.868  1.976  1.963  3.204  1.38  2.403  2.499  5.49  3.827  2.518  2.502  2.507  2.386  1.746  2.103  2.108  1.146  1.445  1.578  0.92  2.257  2.259  1.846  1.82  1.865  2.909  1.872  2.099  2.47  2.214  2.132  2.884  2.615  2.467  1.678  2.208  1.569  1.455  1.688  4.129  1.25  1.74  1.472  1.953  3.064  2.469  1.895  1.772  2.621  2.256  
 
 ARID4B  51742  1  235330209  235491532  1q42.1-q43  1.052  2.704  0.866  1.202  1.731  1.007  1.369  1.509  1.49  1.838  1.969  0.837  1.059  2.164  1.517  1.347  1.362  2.511  2.464  1.079  1.5  1.608  0.96  0.892  1.108  1.599  0.727  0.923  0.802  0.384  1.206  1.331  0.775  0.658  0.915  2.072  1.423  1.68  1.187  1.719  1.792  1.23  1.582  1.737  1.206  1.727  1.105  1.247  1.483  2.09  0.895  0.741  1.156  1.024  1.365  1.717  1.607  0.843  1.398  2.025  
 
 GGPS1  9453  1  235491752  235507844  1q43  1.711  2.615  1.703  2.983  3.221  1.034  2.491  1.831  2.095  2.387  2.019  1.755  2.202  2.409  1.35  2.169  2.131  3.29  3.112  1.191  2.188  1.925  1.791  0.89  1.407  1.977  1.427  1.104  0.988  0.813  1.679  1.668  1.145  1.275  1.071  2.344  1.865  2.408  1.737  1.655  2.166  1.702  3.182  0.98  0.727  2.227  1.751  1.269  1.551  3.472  1.254  1.015  1.403  1.505  1.842  2.225  1.867  1.205  2.017  1.929  
 
 TBCE  6905  1  235530674  235612283  1q42.3  0.877  2.608  0.821  1.428  2.526  0.971  1.973  1.432  1.106  2.232  2.308  1.265  1.558  1.939  0.995  1.299  1.526  2.262  2.934  1.118  2.4  0.844  1.227  1.327  1.363  1.875  0.664  1.408  1.228  0.308  2.142  1.911  1.601  0.701  1.344  2.257  1.968  1.857  0.813  2.194  2.647  1.69  2.227  1.517  1.088  2.212  0.833  1.096  1.66  3.46  0.862  1.179  1.446  1.202  1.977  1.879  1.997  1.227  2.133  2.382  
 
 B3GALNT2  148789  1  235610504  235667781  1q42.3  1.239  3.376  1.474  1.851  2.174  1.328  2.053  1.546  1.934  1.969  2.3  1.354  1.998  2.895  2.253  1.845  2.003  3.601  2.29  2.349  1.389  1.786  1.968  1.649  1.562  2.178  1.213  1.502  1.176  1.353  1.934  2.328  1.8  1.153  1.291  2.291  2.331  2.478  1.377  2.428  2.143  1.78  2.653  2.295  1.463  2.022  1.872  1.931  2.044  3.362  1.146  1.706  2.511  1.5  2.3  2.525  2.078  1.993  2.321  2.337  
 
 GNG4  2786  1  235710984  235814054  1q42.3  0.265  0.032  0.752  1.641  0.045  0.461  0.117  0.754  1.119  0.342  0.19  0.022  0.566  1.048  0  0.056  0.95  1.87  3.002  0.15  0  0.308  1.144  0.046  0.093  0.169  0.166  0.457  0.458  0.542  1.966  0.392  0  0.277  0.956  0.137  0.543  2.471  0.664  1.43  1.809  0.444  1.853  0.072  0.064  0.048  0.443  0.579  0  2.403  0.022  1.437  2.68  3.317  0.643  4.486  3.06  1.091  0.853  0.072  
 
 LYST  1130  1  235824330  236047008  1q42.1-q42.2  0.264  1.245  0.691  1.56  0.834  0.603  1.697  0.902  0.891  1.53  1.443  1.122  0.356  0.606  0.364  0.565  0.655  0.614  0.898  3.432  0.344  1.418  0.589  0.206  0.291  4.019  3.359  1.696  2.814  2.437  3.041  2.57  2.343  1.588  0.638  0.977  0.79  1.079  0.795  0.8  1.22  0.764  1.521  0.299  0.39  0.578  1.729  0.622  0.883  1.262  0.482  0.818  0.925  1.074  1.888  1.15  1.039  0.978  1.144  0.94  
 
 NID1  4811  1  236139131  236228481  1q43  0  0.06  0.142  1.427  0.028  1.893  3.679  3.453  2.655  3.436  3.427  0.042  0  0.046  0.059  0.039  0.032  1.619  0  0.319  0  0.306  0.362  0.03  0  4.07  2.033  2.11  0.197  0.16  0.125  3.501  0.095  0.205  0.287  0  3.548  0.058  0.277  1.398  0.127  1.036  0.451  0.043  0.326  0.312  0.058  1.141  0.068  0.788  1.238  1.372  0.024  2.828  3.15  4.115  0.773  3.528  0  3.038  
 
 GPR137B  7107  1  236305831  236372209  1q42-q43  0.795  0.645  0.108  0.8  2.656  1.11  1.29  1.02  2.427  2.609  1.425  0.141  0.327  1.848  1.8  1.119  1.018  0.897  1.26  0.423  0  0.819  0.432  0.11  1.006  5  3.64  3.278  4.856  3.474  4.806  3.258  2.569  2.227  1.019  0.482  1.561  0  0.933  0.707  0.829  0.764  2.433  0.657  0.95  1.403  0.64  1.297  0.283  2.86  0.272  1.173  1.012  1.162  1.665  2.156  0.299  1.419  0.743  1.763  
 
 ERO1B  56605  1  236378421  236445339  1q42.2-q43  0.085  0.426  0.077  0.169  0.85  0.395  0.164  0.413  1.306  0.393  0.638  0.182  0.061  0.707  0.769  0.057  0.694  0.774  0.317  0.526  0.274  0.411  0.427  0.093  0.255  0.982  0.608  0.232  0.357  0.293  0.973  0.539  0.478  0.496  0.379  0.45  0.238  0.047  0.334  0.555  0.409  0.37  0.477  0.163  0.055  0.31  0.212  0.257  0.237  0.904  0.085  0.255  0.194  0.314  0.359  0.443  0.111  0.732  0.314  0.588  
 
 EDARADD  128178  1  236557679  236648008  1q42.3  2.306  0.196  4.407  5.013  2.551  1.767  3.403  1.974  5.004  3.25  3.657  2.376  2.574  2.778  2.589  2.832  3.221  0.238  2.78  1.251  2.103  1.915  3.686  2.304  2.008  1.424  3.742  2.668  4.085  3.018  2.331  2.546  2.135  3.076  2.573  2.215  3.657  1.662  1.143  2.969  1.438  4.555  3.101  1.818  1.87  1.665  1.996  1.959  1.908  3.564  3.304  2.093  3.597  1.723  3.156  2.338  2.905  1.729  3.128  2.018  
 
 LGALS8  3964  1  236681513  236716279  1q43  1.293  3.308  1.014  1.149  2.878  1.226  2.158  1.485  1.629  1.527  3.018  1.558  1.765  2.587  2.647  2.863  1.942  3.835  3.146  3.062  1.278  2.214  1.111  0.657  1.04  2.832  1.295  1.152  1.229  0.696  1.869  0.657  0.864  1.065  1.34  2.097  1.87  2.278  1.969  2.126  2.524  1.862  1.446  0.977  1.044  2.06  2.829  1.655  1.031  3.217  1.033  1.056  1.738  1.093  2.862  1.938  2.269  1.707  1.53  1.974  
 
 LGALS8-AS1  100287902  1  236686368  236687808  1q43  0.218  0.121  0.21  0  0.555  0.146  0.289  0.071  0.322  0.109  0.316  0.097  0.259  0.218  0.659  0.579  0.452  0.096  0.12  0.313  0  0.308  0.556  0  0.124  0.232  0.144  0.091  0.132  0.081  0.101  0.131  0  0.182  0.105  0.339  0.178  0.151  0.085  0.422  0.741  0.847  0.396  0.217  0.147  0.218  0.037  0  0  0.315  0.094  0.575  0.188  0.249  0.4  0.315  0.098  0.185  0.114  0.169  
 
 HEATR1  55127  1  236712304  236767841  1q43  1.292  1.237  0.993  1.588  1.787  1.046  1.648  1.736  1.154  1.757  2.197  1.18  1.647  2.493  2.162  1.641  1.438  1.125  2.329  1.896  2.785  1.492  1.152  1.723  2.041  2.087  1.171  2.187  1.641  1.323  1.931  2.637  2.118  0.965  1.408  2.765  2.337  2.401  1.014  2.947  2.002  1.429  2.168  2.32  1.611  2.017  1.069  1.797  1.477  2.742  1.2  1.255  1.871  1.207  2.228  2.154  2.143  1.979  2.38  2.505  
 
 ACTN2  88  1  236849753  236927927  1q42-q43  0.096  0.032  0.169  0.222  0.446  0.103  0.118  0.049  0.116  0.172  0.154  0.07  0.104  0.087  0.088  0.167  0.101  0.12  0.03  0.079  0.099  0.171  0.222  0.04  0.05  0.074  1.147  0.323  0.798  0.226  0.368  1.006  0.583  0.272  0.086  0.1  0.132  0  0.245  0.207  0  0.325  0.178  0.035  0  0.069  0.111  0.143  0.06  0.501  0.076  0.42  0.144  0.135  0.194  0.063  0.222  0.046  0.088  0.075  
 
 MTR  4548  1  236958580  237067281  1q43  1.286  2.137  0.91  0.941  1.562  0.935  1.646  1.554  1.001  1.821  1.375  0.689  1.734  1.64  1.681  1.192  1.236  1.798  2.362  1.682  1.827  1.416  1.102  1.399  1.157  1.937  1.576  1.434  1.437  1.311  1.592  1.966  1.72  0.878  1.088  1.773  1.543  1.803  0.685  1.758  1.303  1.024  1.997  1.672  0.865  1.29  0.907  1.377  1.574  2.004  0.961  1.321  1.627  1.219  1.805  1.648  1.943  1.16  1.877  1.663  
 
 MT1HL1  645745  1  237167402  237167718  1q43  0  0.903  0  0  0  0  0  1.26  0  0  0  0  0  0.732  0  0  0  0  0  0  0  0.374  0  0  0  0  1.437  0  0  0  0  0  0  0  0.444  0  0  0  0.884  0  0  0.382  0  0  0  0  0.97  0  0  0.934  0  1.25  0.49  0  2.387  0  0  0  0  0  
 
 RYR2  6262  1  237205701  237997288  1q43  0.025  0.018  0.08  0.02  0.046  0.068  0.057  0.03  0.074  0.04  0.194  0.096  0.051  0.051  0.035  0.054  0.079  0.044  0.021  0.063  0.057  0.088  0.137  0.05  0.046  0.039  0.092  0.094  0.051  0.111  0.018  0.553  0.036  0.112  0.085  0.025  0.064  0.01  0.036  0.049  0.086  0.245  0.049  0.055  0.02  0.078  0.05  0.035  0.04  0.02  0.089  0.26  0.078  0.44  0.048  0.095  0.053  0.032  0.033  0.027  
 
 LOC100130331  100130331  1  238025474  238091619  1q43  1.943  0.19  2.8  1.851  1.281  3.181  2.746  2.773  2.372  2.348  2.454  2.063  2.284  1.974  1.448  1.562  2.508  0.042  1.979  1.269  0.703  3.208  0.498  1.276  2.314  2.388  3.372  2.078  3.574  4.424  2.268  1.342  2.382  1.699  2.905  1.929  3.283  2.039  1.614  2.191  1.016  2.117  3.513  1.447  1.393  2.175  2.008  1.995  1.583  2.038  2.324  1.75  3.851  2.33  3.053  3.562  1.364  1.94  2.832  1.545  
 
 ZP4  57829  1  238041163  238054222  1q43  0  0  0  0  0  0  0.512  0  0.091  0  0  0  0  0  0  0  0  0.049  0  0  0  0.023  0.152  0  0.713  0  0.049  0.042  0  0.061  0  0  0  0.084  0  0  0  0  0.032  0  0  0.182  0.036  0  0  0  0.04  0  0  0  0  0.088  0  0.026  0  0  0  0  0  0  
 
 LINC01139  339535  1  238643683  238649317  1q43  0  0  0  0  2.508  0  0.831  0  0.109  0  0  0  0  0  0.342  0  0  0.448  0.076  0  0  0.035  0.159  0  0.547  0  0.035  0.667  0  0.067  0  1.763  0  0.137  0.223  0  0  1.699  1.434  0.791  1.206  1.148  0.707  0  0.399  0.672  0.038  0.609  0.891  2.5  0.436  0  0.836  0.937  2.585  1.777  1.203  0  2.058  0  
 
 CHRM3  1131  1  239792372  240072717  1q43  0.781  0.063  0.206  0.191  0.552  0.225  0.124  0.04  1.383  1.941  1.265  0.119  0.479  0.028  0.651  0.918  1.175  0.639  1.901  0.08  0  0.289  1.963  1.813  0.287  0.054  1.202  0.258  0.31  0.189  0.096  0.284  0.833  0.605  0.044  0.321  0.245  0.52  0.072  0.119  1.866  0.267  0.065  0  0.191  0.078  0.145  0.131  0  0.178  1.541  0.358  0.029  0.116  0.071  0  0.13  0.129  0.453  0  
 
 CHRM3-AS2  100506915  1  239870425  239882419  1q43  0  0  0  0  0  0  0  0  0  0.117  0.084  0  0  0  0  0  0  0.129  0.872  0  0  0  0  0  0  0  0.113  0  0  0.081  0  0  0  0  0  0  0  0  0  0  0.195  0  0  0  0  0  0  0  0  0  0  0  0  0  0  0  0  0  0  0  
 
 CHRM3-AS1  100873984  1  240061318  240063172  -  0  0  0  0  0  0  0  0  0  0  0  0  0  0  0  0  0  0  0  0  0  0  0  0.328  0  0  0  0  0  0  0  0  0  0  0  0  0  0  0  0  0  0  0  0  0  0  0  0  0  0  0  1.072  0  0  0  0  0  0  0  0  
 
 RPS7P5  645884  1  240170823  240176560  1q43  0  0.4  0.205  0  0.064  0  0  0  0.268  0  0  0  0  0.287  0  0.177  0.303  0.73  0.225  0.315  0  0.442  0.508  0  0.134  0  0.175  0  0.115  0.132  0  0.168  0  0.371  0  0  0  0  0.331  0  0  0.141  0.309  0  0  0  0.244  0  0  0.234  0.088  0.193  0.075  0  0.243  0  0  0.229  0  0  
 
 FMN2  56776  1  240255184  240638489  1q43  0  0.03  0.283  0  0.189  2.604  0.242  1.382  0.706  0.665  1.264  0.064  0  0.03  0  0.056  0.04  0.065  0.057  0.079  0  0.167  0.165  0.03  0.837  0.079  0.334  0.399  1.332  1.833  1.2  1.671  0.356  0.432  0.949  0.043  1.275  0  1.086  0.344  0.037  0.621  0.131  0.026  0.052  0.068  0.054  2.188  0.027  2.808  0.485  0.703  0.018  0.253  1.039  1.748  0.019  2.823  0  1.461  
 
 GREM2  64388  1  240652872  240775462  1q43  0  0  0.15  0  0.934  0.266  0.406  0.12  0.113  0.354  0.076  0.028  0  0.014  0  0  0  0.119  0  0.078  0  0.098  0.282  0  0  0.023  0.093  0.049  0.048  0.161  0  0.054  0  0.312  0.132  0.844  0.057  0  0.074  0.051  0.089  1.78  0.044  0  0.048  0.035  0.035  0.12  0  0.139  0  0.313  0.019  0.056  0.087  0.052  0  0.119  0  0.051  
 
 RGS7  6000  1  240938813  241520530  1q43|1q23.1  0  0.581  0.114  0  0  0.966  0.403  0.086  0  0.62  0.698  0.091  0  0.025  0  0.187  0.408  0.056  0  0.397  0  0.174  0  0  0.608  0  0.101  0.267  0  0.797  0  0  0  0  0.076  0  1.013  0  0.42  0.127  0.076  0.092  0.038  0  0.148  0.056  0.269  0  0  0.73  0.213  0.208  0  0.083  0.113  0.73  0  0.57  0  0.152  
 
 FH  2271  1  241660856  241683085  1q42.1  3.552  5.643  2.52  3.617  4.234  3.118  4.785  3.76  3.465  4.297  4.811  3.783  4.16  4.452  3.825  3.881  4.593  6.103  6.136  5.09  4.67  4.256  3.998  4.453  3.418  4.104  3.638  3.537  3.888  3.366  4.821  4.774  3.74  2.441  3.049  4.544  4.282  3.768  2.773  4.154  4.019  4.027  3.655  3.451  3.348  4.176  3.128  3.613  3.695  5.927  5.242  2.795  3.65  3.182  4.08  4.034  3.387  3.458  3.508  4.197  
 
 KMO  8564  1  241695433  241758949  1q42-q44  0.057  0  0.101  1.069  0.59  0.063  0.089  0.033  0.237  0.046  0.144  0.02  0.044  0.013  0  0.042  0.064  0.098  0.071  0.093  0.054  0.044  0.531  0.37  0.026  0.078  0.023  0.031  0  0.027  0.035  0.071  0  0.124  0.069  0.348  0.033  0.172  0.063  0.189  0.144  0.507  0.061  0  0  0.039  0  0  0.129  0.027  0.198  0  0.249  0.421  0.196  0.746  1.402  0  0.059  0.06  
 
 OPN3  23596  1  241756451  241803701  1q43  1.92  0.953  1.698  3.233  1.023  1.47  1.453  1.339  2.375  1.739  2.44  0.99  1.266  0.974  1.132  1.351  1.772  2.109  2.477  1.688  1.993  1.038  2.133  2.164  1.493  1.985  0.768  1.375  0.634  0.634  1.872  2.317  1.052  1.19  2.135  4.528  2.291  2.822  1.226  1.817  2.527  3.499  1.882  0.623  0.616  1.437  2.518  1.277  0.907  1.574  3.678  1.254  2.135  2.299  2.629  2.367  1.975  0.777  1.651  2.301  
 
 CHML  1122  1  241792166  241799232  1q42-qter  0.807  1.875  0.721  0.85  1.205  0.748  0.697  0.631  0.4  0.756  1.51  0.576  1.142  0.969  1.216  1.165  0.731  1.729  1.478  1.965  2.584  1.538  0.469  1.113  1.255  1.13  0.461  2.173  0.808  0.479  0.836  1.776  1.578  0.832  1.111  1.528  1.789  0.67  0.386  1.426  1.329  1.219  1.16  0.924  0.688  1.082  0.235  1.173  1.171  2.163  2.572  0.662  1.414  0.855  1.107  2.003  1.256  1.132  1.48  1.333  
 
 WDR64  128025  1  241815579  241965434  1q43  0  0  0.058  0  0.02  0  0  0  0.037  0.019  0.024  0.029  0  0.05  0  0.033  0  0.025  0  0  0  0.079  0.106  0  0  0  0.051  0  0  0.039  0  0  0  0.094  0  0  0  0  0  0  0  0.139  0.032  0  0  0.021  0.036  0  0  0  0  0.084  0.052  0.041  0.027  0  0  0  0  0  
 
 EXO1  9156  1  242011492  242053241  1q42-q43  1.833  3.115  1.034  1.685  1.843  1.451  2.422  1.72  0.48  2.318  2.142  1.483  1.663  2.798  2.063  1.878  2.13  2.349  3.521  1.922  2.877  2.27  1.121  1.734  1.776  2.5  1.699  2.118  1.68  2.218  2.568  2.617  2.614  1.213  1.753  2.631  2.186  2.012  1.132  2.871  2.01  2.789  2.845  2.286  1.446  1.96  1.205  1.82  1.675  3.084  2.142  1.609  2.007  1.612  2.036  2.081  1.819  1.603  2.342  1.946  
 
 BECN1P1  441925  1  242121068  242122364  1q43  0  0  0  0  0.055  0  0  0  0.219  0  0  0  0  0  0  0  0  0.247  0  0.103  0  0.234  0.642  0  0  0  0.15  0.059  0  0.097  0  0  0  0.341  0  0.256  0  0  0.084  0  0  0.1  0.06  0  0  0.148  0.051  0  0  0  0  0.782  0  0.181  0.136  0.105  0  0  0  0  
 
 MAP1LC3C  440738  1  242158791  242162385  1q43  0  0  0.136  0  0  0  0.152  0.116  0.665  0  0.668  0.084  0  0  0  0  0  0  0  0  0  0  0.438  0  0  0.088  0.102  0.175  0  0.136  0  0.118  0  0.179  0  0.125  0  0.226  0  0  0  0.141  0  0.187  0  0.319  0.104  0  0  0.199  0.083  0  0.065  0.227  0.246  0.475  0  0  0  0  
 
 PLD5  200150  1  242251688  242687998  1q43  0  0  0.094  0  0  0.138  0  0  0.139  0.027  0.388  0.076  0  0.026  0  0  0  0.076  0  0.058  0  0.148  0.25  0  0  0  0.107  0.024  0  0.102  0  0.039  0  0.177  0.493  0.077  0  0  0.062  0.123  0.071  0.406  0.048  0  0  0.288  0.073  0.199  0  0.661  0.03  0.51  0.229  0.054  0.047  0.051  0  0  0.379  0  
 
 LINC01347  731275  1  243219615  243265046  1q43  1.208  0.188  0.228  0.45  0.919  0.352  0.712  0.494  0.367  0.802  0.696  0.206  0.844  0.928  1.091  0.931  0.495  0.902  0.3  0.645  1.027  1.106  0.396  0.417  0.223  0.901  1.134  1.251  0.869  0.944  0.843  0.659  0.916  0.271  0.567  0.987  1.135  1.365  0.43  0.996  0.812  0.371  1.114  0.672  0.372  0.569  0.427  0.791  0.594  0.293  0.66  0.821  0.938  0.615  1.01  0.533  1.094  0.627  0.71  0.549  
 
 CEP170  9859  1  243287729  243418708  1q44  0.665  3.699  1.725  2.26  0.883  1.72  1.943  2.574  1.453  2.801  2.375  0.42  1.171  2.513  1.525  1.433  2  2.451  2.28  1.966  2.091  1.753  0.453  0.494  2.388  3.653  1.591  2.582  2.406  1.391  2.822  2.768  2.499  1.596  1.308  2.439  2.151  2.54  3.189  2.54  1.202  1.266  2.816  2.279  0.996  1.619  0.991  2.896  1.849  2.898  4.253  1.673  1.902  1.702  2.803  3.124  1.425  2.16  2.31  3.097  
 
 SDCCAG8  10806  1  243419306  243663393  1q43  0.705  1.795  0.837  0.833  1.335  0.692  1.521  1.173  0.945  1.929  1.127  1.062  1.782  2.621  1.267  0.946  1.783  1.768  2.464  1.111  1.228  1.113  0.544  0.544  3.798  1.751  1.434  0.957  1.247  0.752  1.839  1.951  0.997  0.38  1.557  2.113  1.068  2.207  1.569  0.727  1.322  0.709  1.087  0.969  0.695  2.497  0.353  1.137  1.159  2.961  2.743  0.706  0.609  0.909  1.751  1.335  0.926  0.484  1.504  1.783  
 
 AKT3  10000  1  243651534  244006886  1q44  0  2.744  2.755  1.961  0.033  1.625  1.881  3.341  2.156  2.717  1.93  0.059  0.101  0.225  0.307  0.046  0.217  0.615  2.408  0.359  0  1.403  0.192  0.084  2.149  2.63  2.086  2.098  1.856  1.315  2.407  2.54  1.814  1.108  1.709  0.129  2.795  2.806  2.822  1.774  2.39  0.876  0.457  0  0  0.114  1.63  2.233  0.858  3.058  1.083  1.192  1.757  1.73  2.504  3.09  2.79  3.302  2.944  2.655  
 
 LOC339529  339529  1  244080703  244210619  1q44  0  0  0.109  0  0  0.064  0  0  0.066  0.062  0.047  0.068  0  0  0  0  0  0  0  0  0  0.05  0  0  0  0  0.088  0  0  0.101  0  0  0  0.097  0.096  0  0  0  0.059  0  0  0.144  0.043  0  0  0  0.067  0  0  0  0  0.189  0  0.045  0.061  0.127  0  0  0  0  
 
 ZBTB18  10472  1  244212240  244220780  1q44-qter  0.097  3.529  2.026  2.516  0.137  1.815  1.79  1.713  2.129  1.694  2.13  2.305  1.123  0.378  1.325  1.307  1.626  2.993  2.655  1.344  0.336  2.423  0.613  0.357  0.937  1.327  1.004  1.385  1.033  1.07  1.1  0.999  0.837  0.444  1.252  0.612  2.397  2.043  2.585  1.205  1.604  0.861  0.258  0  0.44  0.354  0.601  1.585  0.786  2.211  0.747  1.356  2.408  1.771  2.993  2.604  1.588  1.954  2.191  2.773  
 
 C1orf100  200159  1  244515936  244552388  1q44  0  0  0  0  0  0  0  0  0  0  0  0  0  0  0  0  0  0  0  0  0  0  0  0  0  0  0.141  0  0  0  0  0  0  0  0  0  0  0  0.163  0  0  0  0  0  0  0  0.196  0  0  0  0  0  0  0  0  0  0  0  0  0  
 
 ADSS  159  1  244571793  244615436  1q44  1.203  3.111  1.42  1.02  1.967  1.843  1.748  1.738  1.433  2.338  2.035  1.866  1.953  2.416  1.2  2.416  2.045  2.709  3.788  2.388  2.077  2.267  1.188  1.442  1.783  2.661  1.549  1.988  1.983  0.714  2.305  2.427  1.988  1.217  1.742  2.321  1.914  1.848  2.285  2.051  2.343  2.131  1.227  2.841  1.215  2.114  0.936  1.374  1.701  3.204  2.055  1.575  1.515  1.705  2.04  2.202  1.805  1.499  1.969  2.206  
 
 C1orf101  257044  1  244624672  244803662  1q44  0  0.149  0.057  0.157  0.031  0.043  0  0.027  0.092  0.103  0.028  0.044  0  0.096  0  0  0.062  0.116  0.132  0.075  0  0.11  0.17  0  0  0.087  0.14  0.053  0.047  0.046  0.072  0.054  0  0.079  0.049  0.043  0  0.099  0.123  0  0  0.032  0.362  0.084  0  0.049  0.024  0  0  0.302  0.037  0.18  0  0.036  0.048  0.137  0.068  0  0  0  
 
 DESI2  51029  1  244816349  244872336  1q44  1.937  4.16  2.354  2.518  2.127  1.924  3.074  2.858  2.744  3.103  3.33  2.268  2.713  3.72  2.606  2.032  3.305  3.866  4.234  2.692  2.381  2.97  1.629  1.797  2.369  2.718  2.38  2.136  1.795  1.432  2.711  2.448  2.532  1.742  1.824  2.728  3.008  3.202  2.789  3.319  2.525  2.166  3.532  2.543  1.76  2.661  2.612  2.59  2.59  4.147  3.664  2.088  2.403  1.588  2.517  3.277  2.56  2.263  2.649  2.506  
 
 COX20  116228  1  244998638  245008359  1q44  1.685  4.103  2.753  1.838  3.185  2.256  2.427  2.122  2.937  2.145  3.301  2.539  2.029  3.104  2.074  2.464  2.56  4.465  3.084  2.005  2.517  2.212  2.022  2.387  2.636  2.806  1.317  1.877  1.923  1.251  2.301  2.209  1.529  1.499  2.216  3.288  1.623  2.446  3.605  2.793  2.742  2.549  2.305  2.501  2.041  2.314  2.201  1.71  1.503  3.914  2.779  1.764  1.876  1.45  2.463  1.274  2.37  3.038  2.127  2.673  
 
 HNRNPU  3192  1  245013601  245027827  1q44  3.65  5.075  3.103  3.436  3.871  3.796  4.083  4.12  3.542  4.096  4.923  3.59  4.17  4.495  4.162  3.961  3.965  4.647  5.615  4.108  4.47  4.398  3.078  3.514  4.05  3.769  3.226  3.685  3.515  2.942  4.232  4.407  3.923  2.688  3.916  4.482  4.144  3.895  3.737  4.477  3.938  4.019  4.473  4.306  3.61  3.916  2.829  3.948  3.815  5.185  4.173  3.327  4.12  3.611  4.203  4.067  4.013  4.357  4.458  4.619  
 
 LOC101928068  101928068  1  245128505  245134171  1q44  0.045  0.336  0.176  0  0.343  0  0.106  0.064  0.072  0.087  0.107  0.033  0.174  0.193  0.143  0.219  0.182  0.67  0.208  0.149  0.048  0.261  0.453  0.194  0.032  0.075  0.116  0.08  0.061  0.084  0.161  0.11  0.16  0.279  0.163  0.646  0.053  0.104  0.149  0.044  0.267  0.15  0.425  0.097  0.053  0.206  0.036  0.062  0.184  0.357  0.198  0.265  0.127  0.108  0.235  0.158  0.098  0  0.108  0.052  
 
 EFCAB2  84288  1  245133170  245288530  1q44  1.251  2.042  0.53  0.342  0.69  0.74  1.114  1.405  0.978  1.172  1.09  0.399  0.482  1.522  0.617  0.651  1.223  2.944  2.687  0.818  1.625  0.628  0.569  0.393  0.485  1.049  0.51  0.114  0.169  0.633  0.582  0.658  0.511  0.342  0.797  1.236  1.303  1.793  1.501  1.003  1.609  1.164  1.355  0.945  0.399  1.226  1.143  0.57  0.86  2.122  0.737  0.858  0.516  0.686  1.008  1.311  0.843  1.468  1.621  0.99  
 
 KIF26B  55083  1  245318286  245866428  1q44  0.052  0.041  1.878  0.143  0.052  0.177  0.859  2.711  1.003  1.654  1.817  0.115  0.029  0.133  0.064  0.066  0.461  0.135  0.031  0.144  0.053  0.305  0.645  0.05  0.238  0.057  0.659  0.088  0.047  0.147  0.286  1.099  0.087  0.37  0.034  1.402  0.064  0.374  0.288  0.113  0.145  0.723  0.234  0.07  0.186  1.112  0.081  0.238  0.046  0.271  0.061  1.118  1.82  0.17  1.077  0.123  1.23  0.041  2.308  0.231  
 
 SMYD3  64754  1  245912641  246670644  1q44  2.122  4.037  2.811  1.668  2.139  1.639  3.904  3.339  2.338  3.119  3.063  3.225  1.696  3.09  1.987  2.102  2.606  4.528  4.364  3.427  3.831  3.116  3.602  3.833  1.773  2.164  2.72  3.027  1.814  1.494  2.483  2.57  1.794  1.191  1.582  2.307  2.744  4.393  2.387  2.212  2.719  3.806  2.519  2.496  1.099  2.756  2.469  2.67  1.859  5.148  2.795  2.207  2.308  1.673  3.258  2.571  2.339  1.956  2.973  1.495  
 
 LOC255654  255654  1  246679340  246687589  1q44  0  0  0.237  0  0  0  0  0  0.155  0  0  0  0  0  0  0  0  0  0  0  0  0.083  0.259  0  0  0  0.118  0  0  0.137  0  0  0  0  0  0  0  0  0  0  0  0.399  0  0  0  0  0  0  0  0  0  0.439  0  0  0  0  0  0  0  0  
 
 TFB2M  64216  1  246703862  246729565  1q44  1.903  3.991  1.421  2.318  2.354  1.232  2.205  2.261  1.935  2.092  2.306  1.875  2.006  3.575  1.64  2.57  2.217  5.727  3.715  2.961  3.301  2.011  2.279  2.009  2.408  2.747  1.405  2.255  1.936  1.451  2.561  3.1  2.776  2.332  1.722  2.717  2.234  2.076  2.448  2.49  1.789  2.109  2.441  2.283  1.564  2.285  1.814  1.582  1.124  4.143  2.301  1.331  1.933  1.776  2.629  2.433  1.796  1.825  2.525  2.523  
 
 CNST  163882  1  246729638  246831884  1q44  0.996  1.541  1.433  1.128  1.552  1.121  1.922  1.706  1.318  1.521  1.472  0.813  1.335  1.428  1.052  0.985  1.303  1.576  2.081  1.099  2.085  1.603  1.139  1.165  1.43  2.199  2.046  2.022  1.44  1.086  1.504  2.226  2.15  1.407  0.645  1.681  1.501  1.362  1.504  1.804  1.613  1.251  1.651  1.02  0.974  1.282  0.644  1.171  0.7  2.29  1.8  1.1  0.988  0.907  1.214  1.347  0.911  1.057  1.091  1.532  
 
 SCCPDH  51097  1  246887377  246931440  1q44  1.082  4.79  1.734  2.033  2.641  2.131  3.237  2.378  2.819  3.258  3.022  2.149  1.106  0.029  2.107  2.464  1.868  4.1  5.199  4.079  2.336  4.076  0.18  0.367  1.586  3.815  2.29  3.112  2.237  1.838  1.859  3.537  0  0.219  1.849  3.337  2.957  3.101  2.43  2.063  3.215  3.769  4.244  1.853  1.404  2.406  2.657  2.701  1.704  5.414  2.934  1.989  2.301  0.942  3.886  3.502  2.6  2.138  3.012  2.965  
 
 LINC01341  149134  1  246952918  246954788  1q44  0  0  0  0  0  0  0.158  0.087  0.212  0.292  0.142  0  0  0  0.36  0.088  0  0  0.949  0  0  0.21  0.455  0  0  0.36  0.138  0.211  0.177  0.066  0  0.278  0  0.16  0.178  0  0  0.241  0.125  0.206  0.286  1.631  1.521  0  0  0.165  0.044  0.318  0  0.674  0.125  0.392  0  0.117  0.339  0.24  0  0  0  0  
 
 AHCTF1  25909  1  247002401  247094726  1q44  1.478  3.52  1.061  1.747  1.787  1.175  1.832  2.039  1.251  2.004  2.399  1.057  1.592  2.66  2.113  1.878  1.823  2.864  2.861  1.688  2.269  1.656  0.862  1.284  1.825  2.334  0.904  1.842  1.6  1.22  2.077  2.375  1.706  0.905  1.247  2.681  2.051  2.179  1.852  3.006  2.217  1.511  1.955  2.517  1.299  2.009  0.85  1.916  1.692  3.119  2.284  1.229  1.695  1.249  2.117  2.242  1.884  1.526  2.22  2.283  
 
 ZNF695  57116  1  247108848  247171395  1q44  1.285  1.316  0.138  0.864  1.364  0.537  0  0  0.116  0  0  1.129  0.546  2.234  1.195  1.476  1.642  2.4  2.537  0.57  0.822  2.028  0.227  0  0.351  0.36  0.525  0  0.629  0  0.17  0.809  0.895  0.435  0.79  1.631  0.95  0.351  1.263  0.973  1.301  0.646  1.825  1.176  0.854  1.469  0.436  1.027  1.18  2.199  1.118  1.136  1.034  0.759  0.863  1.506  1.448  1.126  2.213  0.896  
 
 ZNF670-ZNF695  100533111  1  247108848  247242115  1q  0.25  0.37  0  0  0.174  0.191  0  0.061  0  0  0  0  0  0.536  0.255  0.24  0.348  0.257  0.358  0  0  0.421  0  0  0  0  0.089  0  0  0.097  0.224  0.185  0  0  0  0.235  0  0  0  0  0.275  0  0.806  0.213  0  0.241  0.05  0.326  0  0.235  0.111  0  0.086  0.146  0.128  0.191  0.167  0.203  0.588  0  
 
 ZNF670  93474  1  247197939  247242115  1q44  0.265  1.339  0.388  0.402  0.495  0.3  0.296  0.302  0.523  0.348  0.606  0.512  0.49  1.047  0.59  0.438  0.847  1.017  1.475  0.957  0.624  0.787  0.501  0.414  0.39  0.545  0.222  0.663  0.386  0.29  0.487  0.842  0.427  0.418  0.199  0.873  0.944  0.33  0.72  0.53  0.421  0.319  1.263  0.744  0.302  0.323  0.021  0.565  0.388  1.339  0.922  0.214  0.477  0.272  0.431  0.563  0.622  0.632  1.087  0.621  
 
 ZNF669  79862  1  247263263  247267674  1q44  0.921  1.049  0.552  0.73  1.014  0.816  0.669  0.836  0.754  0.674  1.271  0.745  0.483  1.175  0.73  0.595  0.872  0.931  0.962  0.708  0.953  1.228  0.542  0.722  0.549  0.853  0.418  0.885  0.316  0.597  0.923  0.938  0.079  0.253  0.503  1.228  0.753  0.916  0.726  0.943  0.767  0.87  1.241  1.241  0.667  0.751  0.033  0.667  0.825  0.924  1.034  1.118  0.885  0.477  0.758  1.07  0.884  0.639  1.123  0.818  
 
 C1orf229  388759  1  247273461  247275719  1q44  0  0.051  0.198  0  0.172  0.435  0  0.109  0.319  0.143  0.084  0.089  0  0.117  0.156  0.058  0.117  0.158  0  0.102  0  0.363  0.429  0.071  0  0.121  0.227  0.055  0  0.118  0.083  0.063  0  0.146  0.076  0.195  0  0.088  0.133  0  0.079  0.275  0.368  0.228  0  0.126  0.068  0.09  0  0.093  0.081  0.781  0.027  0.127  0.115  0.226  0.133  0.145  0.137  0  
 
 ZNF124  7678  1  247285276  247335319  1q44  1.898  2.22  0.807  0.761  1.067  0.927  1.393  1.235  1.098  1.386  2.037  1.326  1.793  2.233  1.153  1.16  1.236  3.01  3.066  2.012  1.563  2.404  1.13  1.083  1.249  1.451  0.876  1.288  0.873  0.457  1.719  1.6  1.068  0.785  1.106  1.679  1.43  1.698  1.112  1.219  1.437  1.185  2.158  1.877  1.117  1.187  0.937  1.126  0.952  1.719  1.533  0.94  1.574  0.636  1.716  1.974  1.808  1.083  2.076  1.886  
 
 VN1R5  317705  1  247419373  247420447  1q44  0  0  0  0  0  0  0  0  0.159  0  0  0  0  0.072  0  0.076  0  0  0  0.126  0  0.062  0.231  0  0  0  0.231  0  0  0.173  0  0  0  0.23  0  0  0  0  0.076  0  0  0.059  0.11  0  0  0  0.062  0  0  0  0  0  0  0.145  0  0  0  0  0  0  
 
 ZNF496  84838  1  247463621  247495045  1q44  1.357  0.178  1.154  1.838  1.184  1.339  1.511  2.55  1.308  1.853  1.787  0.529  1.457  1.457  2.013  1.431  1.558  0  1.908  0.837  2.365  1.868  0.539  1.073  1.589  1.134  1.134  1.82  1.433  1.109  1.626  1.727  1.743  0.611  0.958  2.546  2.608  1.914  0.821  1.66  2.074  1.314  1.703  1.764  1.487  1.768  1.146  2.502  1.784  2.503  2.415  2.09  1.845  0.795  1.968  2.352  1.725  1.758  2.572  1.67  
 
 NLRP3  114548  1  247579457  247612406  1q44  0  0.451  0.18  0  0.023  0.144  0.278  0.391  0.091  0.058  0.023  0.049  0  0.04  0  0.119  0.062  0.116  0.092  1.353  0  0.194  0.468  0  1.892  0.506  0.091  0.208  0.257  0.069  0.049  0  0  0.21  0  0.044  0.857  0  0.603  0  0  0.272  0.044  0  0.07  0.06  0.053  0.415  0  0.696  0.083  0.377  0.148  0.079  0.657  0.208  0.17  1.02  0  0.825  
 
 OR2B11  127623  1  247614330  247615284  1q44  0  0  0.214  0  0  0  0  0  0.229  0  0  0  0  0  0  0.195  0  0.142  0  0  0  0.229  0.355  0  0  0  0.098  0  0  0.117  0  0  0  0.261  0  0  0  0  0.143  0  0  0.472  0.077  0  0  0.129  0  0  0  0  0  0.784  0  0  0.14  0  0  0  0  0  
 
 OR2W5  441932  1  247654369  247655711  1q44  0  0  0.12  0  0.061  0  0  0  0.126  0.063  0  0  0  0  0  0  0  0.204  0  0  0  0.063  0.49  0  0  0  0.123  0  0  0.073  0  0  0  0.254  0  0  0  0  0.052  0  0  0.255  0.049  0  0  0  0  0  0  0  0  0.303  0  0.05  0  0  0  0  0  0  
 
 GCSAML  148823  1  247670359  247740992  1q44  0  0  0.066  0  0  0.082  0  0  0.086  0  0  0  0  0.022  0  0  0  0  0.101  0.076  0  0.076  0.182  0  0  0  0.071  0  0  1.467  0  0  0.118  0.344  0  0  0  0  0.024  0  0  0.127  0.033  0  0  0  0.03  0  0  0  0  0.213  0  0.053  0  0  0  0  0  0  
 
 GCSAML-AS1  148824  1  247687980  247694106  1q44  0  0  0.135  0  0  0  0  0  0.156  0  0  0  0  0  0  0  0  0  0  0  0  0  0.329  0  0  0  0.021  0  0  0.273  0.177  0  0  0  0  0  0  0  0  0  0  0  0  0  0  0  0  0  0  0  0  0.111  0  0.051  0  0  0  0  0  0  
 
 OR2C3  81472  1  247693433  247697141  1q44  0  0  0.138  0  0  0  0  0  0.111  0.021  0  0  0  0.022  0  0  0  0  0  0.066  0  0.175  0.335  0.064  0  0.061  0.08  0.072  0.094  1.031  1.884  0.313  0  0.147  0  0  0  0  0.026  0  0  0.036  0.03  0  0  0.058  0.038  0  0  0  0  0.44  0.048  0.089  0.049  0  0  0  0  0  
 
 OR2G2  81470  1  247751661  247752615  1q44  0  0  0  0  0  0  0  0  0  0  0  0  0  0  0  0  0  0.23  0  0  0  0  0.564  0  0  0  0.067  0  0  0.057  0  0  0  0.261  0  0  0  0  0  0  0  0  0  0  0  0  0  0  0  0  0  0.216  0  0  0  0  0  0  0  0  
 
 OR2G3  81469  1  247768887  247769817  1q44  0  0  0.178  0  0  0  0  0  0.161  0  0  0  0  0  0  0  0  0  0  0  0  0  0.269  0  0  0  0.053  0  0  0  0  0  0  0.196  0  0  0  0  0  0  0  0  0  0  0  0  0  0  0  0  0  0  0  0  0  0  0  0  0  0  
 
 OR13G1  441933  1  247835419  247836343  1q44  0  0  0  0  0  0  0  0  0.188  0  0  0  0  0  0  0  0  0  0  0  0  0.102  0  0  0  0  0.057  0.262  0  0  0  0  0  0.198  0  0  0  0  0  0  0  0.417  0  0  0  0  0  0.406  0  0.129  0.2  0  0  0  0  0  0  0  0  0  
 
 OR6F1  343169  1  247875130  247876057  1q44  0  0  0.151  0  0  0  0  0  0  0  0  0.153  0  0  0  0  0  0  0  0  0  0.08  0.498  0  0  0  0.044  0.255  0  0.049  0  0  0  0.269  0  0  0  0  0  0  0  0.069  0  0  0  0  0  0.404  0  0.111  0.09  0  0  0  0  0  0  0  0  0  
 
 OR1C1  26188  1  247920763  247921708  1q44  0  0  0.281  0  0  0.152  0  0  0.133  0  0  0  0  0  0  0  0  0  0  0  0  0.068  0.569  0  0  0  0.063  0.395  0  0.064  0  0  0  0.264  0  0  0  0  0  0  0  0  0  0  0  0  0  0  0  0.16  0.122  0  0  0  0  0  0  0  0  0  
 
 OR14A16  284532  1  247978101  247979031  1q44  0  0  0  0  0  0  0  0  0  0  0  0  0  0  0  0  0  0  0  0  0  0  0.318  0  0  0  0.055  0  0  0.051  0  0  0  0  0  0  0  0  0  0  0  0.554  0.709  0  0  0  0  0  0  0  0  0  0  0  0  0  0  0  0  0  
 
 OR11L1  391189  1  248004229  248005198  1q44  0  0  0.197  0  0  0  0  0  0.271  0  0  0  0  0  0  0  0  0  0  0  0  0.097  0.477  0  0  0  0.06  0.104  0  0.074  0  0  0  0.188  0  0  0  0  0.053  0  0  0  0.065  0  0  0  0  0  0  0  0  0.184  0  0  0  0  0  0.171  0  0  
 
 TRIM58  25893  1  248020500  248043438  1q44  0  0.063  0.536  0  0.18  0.057  0  0.022  0.148  0.022  0  0  0  0.019  1.423  0.02  0.028  0.033  0.179  0.128  2.183  0.294  0.726  1.217  0.091  0.572  1.755  0.809  1.684  0.659  1.356  2.865  0.05  0.143  0  1.179  2.633  0  4.295  1.805  0  0.33  0.195  0.03  0.064  0.179  0.022  5.247  2.463  5.029  3.301  0.439  0.023  0.098  0.048  0.091  0.026  5.093  0  0  
 
 OR2W3  343171  1  248058888  248059833  1q44  0  0  0.147  0  0  0  0  0  0.255  0  0  0  0  0  0.835  0  0  0.355  0  0  0.892  0.253  0.529  0.603  0  0.121  0.557  0.738  0.867  0.134  0.436  1.51  0  0.264  0  0  1.65  0  0.768  0.544  0  0.085  0  0  0  0  0  3.049  1.454  3.793  1.988  0.277  0  0.129  0  0  0  3.202  0  0  
 
 OR2T8  343172  1  248084319  248085258  1q44  0  0  0.443  0  0  0.153  0  0  0  0  0  0  0  0  0  0  0  0  0  0  0  0.062  0.387  0  0  0  0.098  0  0  0.063  0  0.15  0  0.218  0  0  0  0  0.295  0  0  0.376  0  0  0  0  0  0.258  0  0.773  0.209  0.233  0.082  0  0  0  0  0  0  0  
 
 OR2L13  284521  1  248100330  248264224  1q44  0  0  0.197  0  0  0  0  0  0.121  0  0  0.086  0  0.027  0  0  0  0.147  0  0.056  0  0.078  0.285  0  0.216  0  0.089  0.084  0  0.053  0  0  0  0.138  0  0  0  0  0.054  0  0  0.194  0.047  0  0  0  0.044  0  0  0  0.104  0.281  0  0.056  0.078  0  0  0.145  0  0  
 
 OR2L8  391190  1  248112159  248113098  1q44  0  0  0  0  0  0  0  0  0  0  0  0  0  0  0  0  0  0  0  0  0  0.06  0.295  0  0  0  0  0.099  0  0  0  0  0  0.274  0  0  0  0  0  0  0  0.398  0  0  0  0  0  0  0  0  0  0  0  0  0  0  0  0  0  0  
 
 OR2AK2  391191  1  248128633  248129641  1q44  0  0  0  0  0  0  0  0  0.124  0  0  0  0  0  0  0  0  0  0  0  0  0.054  0  0  0  0  0.04  0.17  0  0  0  0  0  0.213  0  0  0  0  0.077  0  0  0  0.061  0  0  0  0  0  0  0  0.098  0.218  0  0  0  0  0  0.486  0  0  
 
 OR2L1P  26247  1  248153568  248154493  1q44  0  0  0.151  0  0  0  0  0  0.136  0  0  0  0  0  0  0  0  0  0  0  0  0.109  0  0  0  0  0  0.322  0  0  0  0  0  0.27  0  0  0  0  0  0  0  0  0  0  0  0  0  0  0.198  0  0.125  0  0  0  0  0  0  0  0  0  
 
 OR2L5  81466  1  248185249  248186188  1q44  0  0  0  0  0  0  0  0  0.16  0  0  0  0  0  0  0  0  0  0  0  0  0  0  0  0  0  0  0  0  0  0  0.158  0  0  0  0  0  0  0  0  0  0  0  0  0  0  0  0  0  0  0  0  0  0  0  0  0  0.15  0  0  
 
 OR2L2  26246  1  248201473  248202607  1q44  0  0  0.151  0  0  0  0  0  0  0  0  0  0  0  0  0  0  0  0  0  0  0  0.218  0  0  0  0  0.162  0  0  0  0  0  0  0  0  0  0  0.076  0  0  0  0  0  0  0  0  0  0  0  0  0  0  0  0  0  0  0  0  0  
 
 OR2L3  391192  1  248223983  248224922  1q44  0  0  0  0  0  0  0  0  0  0  0  0  0  0  0  0  0  0  0  0  0  0.108  0.353  0  0  0  0  0  0  0  0  0  0  0  0  0  0  0  0  0  0  0.387  0  0  0  0  0  0  0  0  0  0  0  0  0  0  0  0  0  0  
 
 OR2M5  127059  1  248308449  248309388  1q44  0  0  0.148  0  0  0  0  0  0  0  0  0  0  0  0  0  0  0  0  0  0  0  0  0  0  0  0  0.256  0  0  0  0  0  0.248  0  0  0  0  0  0  0  0  0  0  0  0  0  0  0  0  0  0.653  0  0  0  0  0  0  0  0  
 
 OR2M2  391194  1  248343287  248344331  1q44  0  0  0.23  0  0  0  0  0  0.119  0  0  0  0  0  0  0  0  0  0  0  0  0.075  0.302  0  0  0  0  0.118  0  0  0  0  0  0  0  0  0  0  0  0  0  0  0  0  0  0  0  0  0  0  0  0  0  0  0  0  0  0  0  0  
 
 OR2M3  127062  1  248366369  248367308  1q44  0  0  0  0  0  0  0  0  0.172  0  0  0  0  0  0  0  0  0  0  0  0  0  0  0  0  0  0  0.165  0  0  0  0  0  0  0  0  0  0  0  0  0  0  0  0  0  0  0  0  0  0  0  0  0  0  0  0  0  0  0  0  
 
 OR2M4  26245  1  248402230  248403166  1q44  0  0  0  0  0  0.379  0  0  0  0  0  0.11  0  0  0  0  0  0.168  0.109  0  0  0.114  0  0  0  0  0.075  0.237  0  0  0  0  0  0  0  0  0  0  0  0  0  0  0.047  0  0  0  0  0  0  0  0.107  0.478  0  0  0  0  0  0  0  0  
 
 OR2T33  391195  1  248436153  248437116  1q44  0  0  0.569  0  0  0.349  0  0  0.217  0  0  0  0  0  0.149  0  0  0  0  0  0  0.096  0.466  0  0.132  0  0.103  0.114  0  0.076  0  0  0  0.288  0  0  0.186  0  0.325  0  0  0.38  0  0  0  0  0  0.415  0.306  1.013  0.251  0.184  0.088  0.062  0  0  0  0.321  0  0  
 
 OR2T12  127064  1  248457917  248458880  1q44  0  0  0.292  0  0  0  0  0  0  0  0  0  0  0  0  0  0  0  0  0  0  0.112  0  0  0  0  0.062  0  0  0  0  0  0  0  0  0  0  0  0  0  0  0.115  0  0  0  0  0  0  0  0.156  0  0.166  0  0.062  0  0  0  0  0  0  
 
 OR2M7  391196  1  248486931  248487870  1q44  0  0  0  0  0  0  0  0  0  0  0  0  0  0  0  0  0  0  0  0  0  0  0  0  0  0  0  0  0  0.062  0  0  0  0.311  0  0  0  0  0  0  0  0  0  0  0  0  0  0  0  0  0  0  0  0  0  0  0  0  0  0  
 
 OR14C36  127066  1  248512076  248513015  1q44  0  0  0.176  0  0  0  0  0  0.134  0  0  0  0  0  0  0  0  0  0  0  0  0.08  0.315  0  0  0  0  0  0  0  0  0  0  0.333  0  0  0  0  0  0  0  0  0  0  0  0  0  0  0  0  0  0.272  0  0  0  0  0  0  0  0  
 
 OR2T4  127074  1  248524882  248525929  1q44  0  0  0.221  0  0  0  0  0  0.142  0  0  0  0  0  0  0  0  0  0  0  0  0  0.639  0  0  0  0.077  0  0  0  0  0  0  0  0  0  0  0  0  0  0  0.079  0  0  0  0  0  0  0  0  0  0  0  0  0  0  0  0  0  0  
 
 OR2T6  254879  1  248550909  248551836  1q44  0  0  0.261  0  0  0  0  0  0.136  0  0  0  0  0.059  0  0  0  0  0  0  0  0.18  0  0  0  0  0.149  0  0  0.089  0  0  0  0.234  0  0  0  0  0.069  0  0  0.132  0.071  0  0  0  0  0  0  0  0  0  0  0  0  0  0  0  0  0  
 
 OR2T1  26696  1  248569295  248570405  1q44  0  0  0  0  0  0  0  0  0  0  0  0  0  0  0  0  0  0  0  0  0  0  0.355  0  0  0  0.084  0  0  0.042  0  0  0  0  0  0  0  0  0  0  0  0  0.059  0  0  0  0  0  0  0  0  0.157  0  0  0  0  0  0  0  0  
 
 OR2T2  401992  1  248616098  248617073  1q44  0  0  0.247  0  0.107  0  0  0  0.177  0  0  0  0  0  0  0  0  0  0  0  0  0.059  0.412  0  0  0  0.104  0  0  0.05  0  0  0  0.443  0  0  0  0  0  0  0  0.943  0  0  0  0  0  0  0  0  0  0.284  0  0  0  0  0  0  0  0  
 
 OR2T3  343173  1  248636651  248637608  1q44  0  0  0.226  0  0  0  0  0  0  0  0  0  0  0  0  0  0  0  0.082  0  0  0  0.601  0  0  0  0.073  0  0  0.109  0  0  0  0.243  0  0  0  0  0.1  0  0  0.092  0  0  0  0  0  0  0  0  0  0.207  0  0  0  0  0  0  0  0  
 
 OR2T5  401993  1  248651889  248652837  1q44  0  0  0.233  0  0  0  0  0  0.3  0  0  0  0  0  0  0  0  0  0  0  0  0  0.459  0  0  0  0.054  0  0  0  0  0  0  0.222  0  0  0  0  0  0  0  0.594  0  0  0  0  0  0  0  0  0  0.265  0  0  0  0  0  0  0  0  
 
 OR2G6  391211  1  248684947  248685898  1q44  0  0  0  0  0  0  0  0  0  0  0  0  0  0  0  0  0  0.143  0  0  0  0  0.566  0  0  0  0.058  0  0  0.048  0  0  0  0.262  0  0  0  0  0  0  0  0  0.064  0  0  0  0  0  0  0  0  0.287  0  0  0  0  0  0  0  0  
 
 OR2T29  343563  1  248721844  248722792  1q44  0  0  0  0  0  0  0  0  0.207  0  0  0  0  0  0  0  0  0  0  0  0  0  0.315  0  0  0  0  0  0  0  0  0  0  0.222  0  0  0  0  0  0  0  0.272  0  0  0  0  0  0  0  0  0  0.217  0  0  0  0  0  0  0  0  
 
 OR2T34  127068  1  248737101  248738058  1q44  0  0  0.173  0  0  0  0  0  0  0  0  0  0  0  0  0  0  0  0  0  0  0.076  0.36  0  0  0  0.085  0  0  0  0  0  0  0.243  0  0  0  0  0  0  0  0.118  0  0  0  0  0  0  0  0  0  0.413  0  0  0  0  0  0  0  0  
 
 OR2T10  127069  1  248756130  248757069  1q44  0  0  0  0  0  0  0  0  0  0  0  0  0  0  0  0  0  0  0  0  0  0  0  0  0  0  0.036  0  0  0  0  0  0  0  0  0  0  0  0  0  0  0  0  0  0  0.116  0  0  0  0  0  0.291  0  0  0  0  0  0  0  0  
 
 OR2T11  127077  1  248789478  248790429  1q44  0  0  0  0  0  0  0  0  0  0  0  0  0  0  0  0  0  0  0  0  0  0.129  0  0  0  0  0.051  0  0  0.04  0  0  0  0  0  0  0  0  0  0  0  0  0  0  0  0  0  0  0  0  0  0  0  0  0  0  0  0  0  0  
 
 OR2T35  403244  1  248801587  248802559  1q44  0  0  0.297  0  0  0  0  0  0.178  0  0  0  0  0  0  0  0  0  0  0  0  0.067  0.413  0  0  0  0.073  0  0  0  0  0  0  0.289  0  0  0  0  0  0  0  0.709  0  0  0  0  0  0  0  0  0  0  0  0  0  0  0  0  0  0  
 
 OR2T27  403239  1  248813231  248814185  1q44  0  0  0.2  0  0  0  0  0  0.241  0  0  0  0  0  0  0  0  0  0  0  0  0.073  0.525  0  0  0  0.114  0  0  0.043  0  0  0  0.278  0  0  0  0  0.154  0  0  0  0.162  0  0  0  0  0  0  0  0  0.412  0  0  0  0  0  0  0  0  
 
 OR14I1  401994  1  248844669  248845605  1q44  0  0  0  0  0  0  0  0  0  0  0  0  0  0  0  0  0  0  0  0  0  0  0.316  0  0  0  0  0  0  0  0  0  0  0.231  0  0  0  0  0  0  0  0  0  0  0  0  0  0  0  0  0  0  0  0  0.074  0  0  0.178  0  0  
 
 LOC101928226  101928226  1  248860362  248862411  1q44  0  0  0  0  0  0  0  0  0  0  0  0  0  0  0  0  0  0  0  0  0  0  0  0  0  0  0  0  0  0  0  0  0  0  0  0  0  0  0  0  0  0  0  0  0  0  0  0  0  0  0  0  0  0.322  0  0  0  0  0  0  
 
 LYPD8  646627  1  248902716  248903151  1q44  0  0  0  0  0.209  0  0  0  0  0  0  0  0  0  0  0  0  0  0  0  0  0.571  0.635  0  0  0  0  0  0  0.098  0  0  0  0  0  0  0  0  0.813  0  0  0  0  0  0  0.456  0  0  0  0.463  0  0  0  0  0  0  0  0.49  0  0  
 
 SH3BP5L  80851  1  249104650  249120154  1q44  1.593  0.085  2.296  2.184  1.788  1.566  2.393  2.717  2.366  2.684  2.377  2.019  2.087  1.46  2.04  1.687  1.754  0.112  3.045  1.483  1.727  1.795  1.533  1.583  2.461  2.282  2.07  2.205  1.167  1.62  2.001  2.115  2.158  0.824  1.314  2.33  2.357  2.024  2.746  2.048  1.691  2.45  2.371  1.992  2.278  2.013  1.272  2.849  1.756  3.639  2.932  1.536  2.046  2.464  2.731  2.348  2.279  2.086  2.571  2.339  
 
 ZNF672  79894  1  249132376  249143716  1q44  2.001  0.682  1.786  2.933  1.837  1.609  2.961  2.871  2.797  2.694  2.384  2.274  2.815  2.229  2.757  1.97  2.339  0.47  3.719  2.028  2.207  2.788  1.856  2.015  1.65  2.845  2.663  3.038  1.836  2.848  3.052  2.969  2.608  1.619  1.572  2.894  2.708  2.816  2.428  2.917  2.398  3.155  3.247  1.855  1.599  2.686  1.974  2.42  2.218  2.975  2.961  2.247  2.395  1.678  2.426  2.385  1.839  2.049  2.056  2.134  
 
 ZNF692  55657  1  249144202  249153315  1q44  2.485  0.424  1.805  2.074  3.261  0.857  2.324  1.835  1.908  2.894  2.31  1.699  2.617  2.81  2.903  2.434  2.325  0  4.55  3.098  2.798  3.422  2.199  2.567  1.371  2.379  2.673  2.258  1.881  2.033  3.195  2.988  2.573  1.068  1.887  2.76  2.406  2.281  2.671  2.569  2.445  3.126  3.172  2.716  1.751  2.296  2.697  2.878  1.786  4.502  2.776  2.099  1.881  1.55  3.8  2.499  2.425  2.607  2.199  1.867  
 
 PGBD2  267002  1  249200441  249213345  1q44  0.986  0.686  0.676  0.629  0.753  0.37  1.267  0.752  0.788  1.324  0.736  0.42  0.835  1.173  0.872  1.111  0.48  0.887  1.625  0.867  1.04  0.66  0.549  0.348  0.571  0.633  1.175  1.146  0.999  1.067  1.288  0.906  0.985  0.464  0.422  0.957  0.602  0.482  0.499  1.223  0.673  0.574  0.925  0.786  0.841  0.861  1.182  0.952  0.543  1.876  0.732  0.638  0.538  0.563  0.733  1.002  0.731  0.4  0.56  0.667  
 
 LOC100289097  100289097  2  14738  36767  -  0  0  0  0  0  0  0  0  0  0  0.124  0  0  0  0  0  0  1.157  0.134  0  0  0  0  0  0  0  0.075  0  0  0  0  0.217  0  0  0  0  0  0  0  0  0  0  0.199  0  0  0  0.076  0  0  0  0  0  0.116  0  0.119  0  0.232  0  0  0  
 
 FAM110C  642273  2  38813  46588  2p25.3  0.589  0  0.122  0  1.006  0.059  0.039  0.029  0.36  0.023  0.221  1.023  0.677  1.532  0.326  1.627  0.193  1.673  0.024  0.08  0  0.988  0.162  0  0  0  0.093  0.017  0  0.053  0  0  0  0.215  0.273  0.218  0.037  0  1.14  0.049  1.377  0.318  0.026  0.536  0.068  0.351  0.645  0  0.724  0  0.423  0.802  0  0.339  0.84  0.048  0  0  0.386  0.592  
 
 SH3YL1  26751  2  218135  264866  2p25.3  2.569  1.37  0.643  0.715  3.041  0.185  0.99  1.042  1.544  1.987  0.798  2.803  2.854  2.174  2.039  2.568  3.595  0.93  3.152  0.876  0.212  1.893  0.564  0.149  0.27  1.562  0.927  0.724  0.759  0.714  1.025  0.622  0.856  0.66  0.271  1.381  0.682  1.11  1.34  0.891  2.331  0.688  1.55  0.417  0.63  1.758  1.462  0.352  0.322  1.137  0.766  1.085  0.645  0.856  1.336  1.22  0.861  0.219  0.23  0.441  
 
 ACP1  52  2  264868  278282  2p25  4.859  5.615  3.805  3.551  4.374  2.984  5.294  4.439  4.74  4.417  5.323  4.757  4.028  5.686  4.108  4.772  5.156  5.693  6.111  4.153  4.324  5.005  4.574  3.418  4.348  4.294  4.33  3.9  4.471  4.231  4.825  4.827  4.211  3.506  4.588  4.167  4.299  4.412  4.681  4.921  4.611  4.607  5.668  4.285  3.862  3.602  5.163  3.473  3.794  5.669  4.508  3.236  4.368  3.949  5.009  4.351  3.928  4.651  4.172  4.13  
 
 FAM150B  285016  2  279560  288308  2p25.3  0  0  0.133  1.519  0.068  0.15  0  0.074  0  0.111  0.082  0.123  0  0.168  0  0.189  0.205  0  0.119  0.084  0  0.437  0.223  0.111  0  0.115  0.222  0.066  0  0.249  0  0  0  0  0  0  0  0  0.182  0.116  0.134  0.687  0.074  0.178  0.202  0.162  0.128  0  0  0  0  0.858  0  0.225  0.171  0.135  0.299  0  0.091  1.165  
 
 TMEM18  129787  2  667972  677439  2p25.3  3.023  2.957  1.962  2.097  3.01  1.262  3.707  2.857  2.016  2.694  2.216  2.341  2.109  2.39  2.459  3.034  3.173  2.045  3.852  3.152  1.819  3.494  2.375  1.593  2.195  2.358  2.948  1.826  2.536  2.98  3.157  2.436  2.739  1.888  2.61  2.519  2.458  2.376  2.503  3.358  2.853  2.481  3.666  2.613  1.381  1.974  3.577  2.526  2.043  4.54  2.619  1.843  2.593  2.16  3.379  2.839  2.188  2.43  2.048  2.43  
 
 LINC01115  339822  2  779836  864112  2p25.3  0  0  0.166  0  0  0  0.09  0  0  0.117  0  0  0  0  0  0  0  0  0.618  0  0  0.362  0.36  0  0  0  0.127  0  0  0.114  0  0  0  0.294  0  0  0  0  0.048  0  0  0  0  0  0  0  0.063  0  0  0  0  0.53  0  0.134  0  0  0  0  0  0  
 
 LOC101060385  101060385  2  895901  901137  -  0  0  0  0  0.151  0  0  0  0  0.301  0  0  0  0  0  0  0  0  0  0  0  0  0  0  0  0  0  0  0  0.074  0  0  0  0  0  0  0  0  0  0  0  0  0  0  0  0  0  0  0  0  0  0.559  0  0  0  0  0  0  0  0  
 
 SNTG2  54221  2  946553  1371384  2p25.3  0  0.042  0  0  0.066  0  0.06  0.044  0  0.885  0  0  0.087  0.047  0  0  0.061  0  0.061  0.09  0  0.399  0.146  0  0  0.075  0.148  0.031  0.087  0.101  0  0  0  0.11  0.151  0  0.048  0  0.038  0  0  0.131  0.072  0.098  0  0.043  0.085  0  0  0.069  0.054  0.511  0.081  0.094  0.077  0  0  0  0  0.115  
 
 TPO  7173  2  1417232  1546499  2p25  0.148  0  0.124  0  0.213  0.114  0.066  0.05  0.061  0.071  0.052  0.096  0  0.047  0.113  0.097  0  0.208  0.174  0.199  0  0.223  0.363  0.32  0  0  0.134  0.067  0  0.156  0  0  0  0.202  0.072  0  0  0  0.118  0  0.135  0.514  0.083  0  0  0.062  0.043  0  0  0  0.04  0.729  0  0.171  0.113  0.066  0  0  0  0  
 
 PXDN  7837  2  1635658  1748319  2p25  2.311  2.933  6.206  4.086  2.986  3.424  6.046  6.569  4.312  5.708  4.72  0.151  0  1.779  0.313  0.095  0.142  0.495  3.432  0.2  0  2.359  2.201  0.767  0.036  0.054  3.537  0.04  0.164  0.268  0.961  1.096  0  0.185  0.291  0.096  4.795  0  4.749  4.423  2.518  1.893  4.401  0.154  4.031  2.395  0.129  0.195  2.323  0.04  4.087  1.12  0.057  5.302  3.591  3.531  5.14  2.323  3.091  5.395  
 
 MYT1L  23040  2  1792884  2335085  2p25.3  0  0.028  0.115  0  0.046  0  0.022  0.046  0.059  0.031  0.031  0.035  0  0.028  0.096  0.03  0.04  0.133  1.384  0.043  0  0.196  0.111  0  0  0.017  0.125  0.027  0.019  0.106  0  0  0  0.219  0.034  0.025  0  0  0.054  0  0.02  0.269  0.051  0.02  0.034  0.029  0.108  0  0  0.016  0.024  0.441  0.02  0.083  0.046  0.039  0.018  0  0  0  
 
 MYT1L-AS1  730811  2  2323003  2330880  2p25.3  0  0  0.13  0  0  0  0  0  0.081  0  0.02  0  0  0.015  0  0  0  0  0  0.036  0  0.044  0.273  0  0  0  0.064  0  0  0.054  0  0  0  0.157  0  0  0  0  0.024  0  0  0.219  0.03  0  0  0  0.021  0  0  0  0.031  0.108  0  0.025  0  0  0  0  0  0  
 
 LINC01250  101927554  2  2898819  3129798  2p25.3  0  0  0.098  0  0.055  0  0.122  0.033  0  0.04  0.052  0  0  0.058  0  0.087  0.071  0  0  0  0  0.043  0.287  0.069  0.074  0.04  0.095  0.06  0.06  0.054  0.068  0  0  0.257  0  0  0  0  0.147  0  0  0  0.132  0  0.066  0.031  0.042  0.1  0  0  0.032  0.168  0  0.085  0.063  0  0.066  0  0  0  
 
 TSSC1  7260  2  3192740  3381653  2p25.3  3.57  2.806  2.249  2.375  2.717  1.663  4.051  2.942  2.542  2.816  3.501  3.151  3.639  3.917  2.96  3.739  3.573  2.253  5.164  3.57  3.052  3.154  2.54  2.667  3.365  2.766  3.262  2.822  3.377  3.733  3.682  3.73  3.536  2.239  2.906  3.195  2.493  3.173  3.425  4.053  4.009  3.193  4.218  2.405  2.302  2.369  3.103  2.784  1.298  4.206  3.112  2.676  2.974  2.062  4.043  2.98  2.734  2.923  2.892  3.151  
 
 TRAPPC12  51112  2  3383445  3483342  2p25.3  2.789  1.86  1.807  2.065  2.288  0.963  3.084  2.662  2.61  2.898  2.526  1.903  2.283  2.501  2.126  2.603  2.634  1.087  3.752  2.523  1.847  2.903  1.562  1.503  2.451  2.21  2.709  2.073  2.475  2.252  2.807  2.635  2.901  1.863  2.358  2.839  2.313  2.671  2.568  2.989  3.326  2.65  2.987  2.029  1.901  1.629  4.03  2.499  1.675  3.322  2.645  2.265  2.507  1.714  2.987  2.027  2.195  2.07  1.416  2.196  
 
 ADI1  55256  2  3501689  3523350  2p25.3  6.101  5.127  3.585  2.919  4.992  2.561  4.55  3.725  4.507  4.107  4.6  3.465  4.667  5.107  3.876  4.203  4.135  3.742  5.133  4.793  2.941  3.683  4.352  3.74  2.963  3.99  4.318  3.139  4.045  3.067  4.71  4.881  5.029  4.415  3.273  3.159  3.667  3.941  4.496  5.128  4.754  4.271  4.435  4.151  2.762  3.544  3.298  4.16  3.242  5.639  4.019  3.535  3.262  3.673  3.892  3.858  3.434  4.583  2.895  3.363  
 
 RNASEH1  246243  2  3592481  3605961  2p25  3.05  2.873  2.274  2  2.729  1.832  4.155  3.173  3.029  3.147  3.589  2.189  2.731  3.705  2.423  2.891  2.447  2.76  4.059  3.47  3.194  3.651  2.888  2.497  3.465  2.851  2.894  2.607  2.977  2.707  3.79  3.968  3.289  2.231  3.097  3.509  3.233  2.575  3.61  3.369  3.759  2.631  3.899  2.234  2.304  2.364  4.031  2.902  2.207  3.894  3.11  2.788  3.277  2.164  3.659  2.557  2.786  2.814  2.795  3.078  
 
 RNASEH1-AS1  100506054  2  3605975  3609340  2p25  1.887  1.896  1.706  2.071  1.266  1.911  2.765  0.969  1.486  0.996  1.115  1.507  1.836  1.654  1.008  1.734  1.754  1.249  2.462  0.981  1.331  1.711  2.293  2.153  1.833  1.109  2.325  1.996  0.956  1.399  2.833  2.294  2.996  2.133  2.401  1.834  1.976  1.837  1.191  2.881  2.193  2.031  2.61  1.521  1.251  0.802  2.402  1.576  1.092  2.345  1.95  2.158  2.136  1.476  2.545  1.082  1.032  2.175  2.086  1.538  
 
 RPS7  6201  2  3622852  3628509  2p25  7.353  10.829  6.15  6.097  8.323  6.324  7.81  7.218  7.239  7.473  7.789  7.201  6.992  8.601  6.278  7.754  8.529  12.15  9.961  10.194  8.161  8.691  8.142  7.19  8.077  6.594  6.456  6.014  6.651  5.819  8.103  7.987  7.264  6.25  7.664  7.487  6.86  6.957  8.985  7.338  8.175  8.476  7.958  8.205  6.823  6.711  7.943  6.964  7.039  9.941  7.164  6.332  6.749  6.727  8.579  6.986  6.899  7.878  7.07  7.688  
 
 COLEC11  78989  2  3642421  3692234  2p25.3  0  0  0  0  0.206  0  0.115  0  0.165  0.084  0.196  0  0  0.155  0  0  0  0.422  0.111  0.12  0.322  0.335  0.39  0  0  0.105  0.225  0.204  0  0.176  0  0  0  0.334  0.327  0.437  0  0  0.1  0.342  0.156  1.158  0.479  0  0  0.142  0.259  0.27  0  0.278  0  1.068  0  0.176  0.096  0  0  0  0  0  
 
 ALLC  55821  2  3705785  3750260  2q35  0  0  0  0  0  0  0  0  0  0  0  0  0  0  0  0  0  0  0  0  0  0.118  0  0  0  0.165  0.158  0  0  0.151  0  0  0  0  0  0  0  0  0.073  0  0  0  0.072  0  0  0  0.067  0  0  0  0  0.179  0  0.083  0  0.104  0  0  0  0  
 
 DCDC2C  728597  2  3751181  3895000  2p25.3  0  0  0  0  0.063  0.098  0.079  0.053  0  0.067  0.059  0.164  0  0.054  0  0.083  0.079  0  0  0.069  0  0.294  0.298  0  0  0  0.466  0  0  0.18  0  0  0  0  0  0  0  0  0.127  0  0  0.635  0.093  0  0  0.056  0.071  0  0  0  0.075  1.161  0  0.169  0.149  0  0  0  0  0  
 
 LINC01304  100505964  2  4005244  4021622  2p25  0  0  0.127  0  0  0  0  0  0.049  0.035  0  0.04  0  0.02  0  0  0  0  0  0.038  0  0.037  0.262  0  0  0  0.058  0  0  0.045  0  0  0  0.179  0  0  0  0  0  0  0  0  0.035  0  0  0  0.031  0  0  0  0  0.121  0  0.028  0  0  0  0  0  0  
 
 LINC01249  727982  2  4675807  4703812  2p25.2  0  0  0.105  0  0.044  0  0  0  0.055  0  0  0  0  0.023  0  0  0  0  0  0  0  0.056  0.124  0.154  0  0  0.056  0  0  0.049  0  0  0  0.15  0  0  0  0  0.016  0  0.047  0.022  0.028  0  0  0  0.022  0  0  0.029  0  0.167  0  0  0  0  0  0.069  0  0  
 
 LINC01248  102723818  2  5774272  5831250  2p25.2  0  0  0  0  0  0  0  0  0  0  0.114  0  0  0  0  0  0  0  0  0  0  0  0  0  0  0  0.076  0  0  0.068  0  0  0  0  0  0  0  0  0  0  0  0  0  0  0  0  0.573  0  0  0  0  0  0  0.158  0  0  0  0  0  0  
 
 SOX11  6664  2  5832798  5841517  2p25  0.026  0.043  0.133  1.427  0.065  1.174  0.509  0.036  0.105  0.209  1.281  0.056  0  0.04  0.015  0.058  0.047  0.182  0.022  0.059  0  0.256  0.268  0.052  0.03  0.024  0.127  0.027  0.032  0.123  0.034  0.04  0.018  0.153  0.045  0.032  0.302  0  0.076  0.085  0.028  0.303  0.062  0.038  1.539  0.111  0.092  0.019  0  0.015  0.044  0.639  0.024  0.873  0.196  0.066  0.027  0.018  0  0.062  
 
 LINC01105  150622  2  6072818  6120350  2p25.2  0  0.016  0.142  0  0.014  0.015  0  0.019  0.096  0.01  0.01  0.015  0  0.012  0  0.019  0.014  0.029  0.01  0.018  0  0.056  0.297  0.017  0  0.008  0.064  0  0  0.057  0.01  0.021  0  0.265  0.021  0  0.013  0  0.019  0  0  0.099  0.03  0  0  0.035  0.035  0  0  0  0.007  0.166  0.008  0.028  0.01  0  0.012  0  0  0  
 
 LOC400940  400940  2  6122109  6128364  2p25.2  0  0.034  0.116  0  0  0  0  0  0.106  0  0.024  0  0  0.021  0  0  0  0.082  0  0.05  0  0.053  0.257  0.037  0  0  0.059  0.017  0  0.069  0  0  0  0.27  0  0  0  0  0.036  0  0  0.07  0.041  0  0.05  0  0.033  0  0  0  0  0.346  0  0.051  0.023  0  0  0  0  0  
 
 LINC01247  101929390  2  6506141  6515554  2p25.2  0  0  0.047  0  0  0  0.024  0  0.086  0  0  0  0  0.011  0  0.027  0  0.057  0  0  0  0.013  0.144  0  0  0  0.039  0  0  0.041  0  0  0  0.214  0  0  0  0  0.037  0  0  0.125  0.02  0  0  0.02  0.064  0  0  0  0  0.109  0  0.025  0  0  0  0  0  0  
 
 LINC01246  102800315  2  6766240  6774838  -  0  0  0  0  0  0  0  0  0  0  0  0  0  0  0  0  0  0  0  0  0  0.286  0  0  0  0  0  0  0  0  0  0  0  0  0  0  0  0  0  0  0  0  0  0  0  0  0  0  0  0  0  0  0  0  0  0  0  0  0  0  
 
 MIR7515HG  102800314  2  6789238  6790651  2p25.2  0  0  0  0  0  0  0  0  0  0  0  0  0  0  0  0  0  0  0  0  0  0.117  0  0  0  0  0.073  0  0  0.074  0  0  0  0  0  0  0  0  0  0  0  0  0  0  0  0  0  0  0  0  0  0  0  0.148  0  0  0  0  0  0  
 
 LINC00487  400941  2  6869299  6910442  2p25.2  0  0  0.086  0  0  0  0  0  0.145  0  0  0.051  0  0  0  0  0  0  0.043  0  0  0.071  0.267  0  0  0  0.05  0  0  0.057  0  0  0  0.322  0  0  0  0  0  0  0  0.144  0.024  0  0  0  0  0  0  0  0  0.176  0  0.044  0  0  0  0  0  0  
 
 NRIR  104326052  2  6968684  6980595  2p25.2  0  0  0  0  0  0  0  0  0  0  0  0  0  0  0  0.14  0  0  1.404  0  0  0.444  0  0  0  0  0.094  0.711  0  0.102  0  1.027  0  0  0  0  0  0  0.096  0  0  0  0  0  0  0  0.099  0  0  0  0  0  0  0.24  0  0  0  0  0  0  
 
 CMPK2  129607  2  6980683  7006766  2p25.2  0.135  0.102  0.181  2.894  0.123  0  0.201  0.361  0.228  0.361  0.444  0.192  1.431  0.341  0.313  4.968  0.334  0.222  3.49  0.266  0.674  2.045  0.485  0.232  0.582  0.127  0.679  0.845  0.713  0.216  0.142  0.535  0.54  0.659  0.151  0.166  0.061  0  1.973  0  0.075  0.531  0.221  0.141  0.202  0.181  0.167  0  0.109  0.118  0.554  1.262  0  1.763  0.227  0  2.113  0  0  0  
 
 RSAD2  91543  2  7017795  7038363  2p25.2  0  0.09  0.089  4.492  0.044  0  0.042  0.308  0.081  0.147  0.021  0  0.628  0.018  0  4.478  0  0  0.994  0.033  0  0.808  0.265  0.449  0.276  0  0.099  0.063  0.033  0.063  0  0  0  0.256  0  0  0.025  0.136  2.701  0  0.065  0.142  0.034  0  0  0.132  0.253  0  0  0  0.201  0.116  0  1.62  0  0  2.221  0  0.88  0  
 
 RNF144A-AS1  386597  2  7052406  7058813  2p25.2  0  0.038  0.115  0  0  0  0  0  0.125  0.036  0  0  0  0.02  0  0.037  0  0.044  0  0.201  0  0.087  0.278  0  1.112  1.158  0.644  1.616  1.879  0.511  1.491  1.43  0.054  0.269  0  0.129  0.339  0.259  0  0.265  0  0.431  0.85  0  0  0  0.938  0.177  0  1.017  0.444  0.209  0  0.111  0  0  0.074  0  0.22  0  
 
 RNF144A  9781  2  7057522  7184309  2p25.2  0.545  0.109  0.272  0.753  0.13  0.133  0.144  0.83  0.541  2.289  1.362  0.054  0.516  1.311  1.348  0.244  0.571  0.055  2.081  1.268  1.062  1.563  0.247  0.352  1.949  3.45  2.633  2.821  3.831  2.805  3.66  3.669  2.951  1.539  0.797  1.748  1.093  1.774  0.705  1.48  0.036  0.723  2.325  0.861  0.72  0.859  0.622  0.802  1.516  1.105  2.156  0.762  0.028  0.657  0.236  0.038  1.32  0.106  1.387  0.154  
 
 LOC101929452  101929452  2  7202857  7218011  2p25.1  0  0  0  0  0  0  0  0  0  0.154  0  0  0  0  0  0  0  0  0  0  0  0  0  0  0  0.429  0.165  0.22  0.472  0.322  0.676  0.263  0.325  0  0  0  0  0  0  0  0  0  0  0  0  0  0  0  0  0  0  0.331  0  0  0  0  0  0  0  0  
 
 LOC100506274  100506274  2  7561391  7590381  2p25  0  0  0  0  0  0  0  0  0  0  0  0  0  0  0  0  0  0  0  0  0  0  0  0  0  0  0.047  0.101  0  0  0  0  0  0  0  0  0  0  0  0  0  0  0  0  0  0  0  0  0  0  0  0  0  0  0  0  0  0  0  0  
 
 LOC101929551  101929551  2  8026897  8039786  2p25.1  0  0  0  0  0  0  0  0  0  0  0  0  0  0  0  0  0  0  0  0  0  0  0  0  0  0  0  0  0  0  0  0  0  0  0  0  0  0  0  0  0  0  0  0  0  0  0  0  0  0  0  0.254  0  0  0  0  0  0  0  0  
 
 LINC00298  339788  2  8062555  8116945  2p25.1  0  0  0.108  0  0  0  0  0  0  0  0  0  0  0  0  0  0  0  0  0.041  0  0.027  0.291  0  0  0  0.024  0  0  0.028  0  0  0  0.149  0  0  0  0  0  0  0  0  0.025  0  0  0  0  0  0  0  0  0.12  0  0  0  0  0  0  0  0  
 
 LINC00299  339789  2  8147900  8468549  2p25.1  0  0  0.083  0  0  0.147  0  0  0.114  0.033  0  0  0  0.059  0  0  0  0  0  0  0  0.079  0.289  0  0  0  0.1  0  0  0.124  0  0  0  0.201  0  0  0  0  0.042  0  0  0.369  0.039  0  0  0  0.136  0  0  0  0  0.105  0.039  0.059  0.039  0  0  0  0  0  
 
 LOC101929567  101929567  2  8699962  8723922  2p25.1  0.342  0  0.116  0.03  0.202  0  0  0.025  0.122  0.021  0.022  0.148  0.161  0.18  0.234  0.097  0.045  0.066  0  0.031  0  0.053  0.275  0.017  0  0  0.078  0.019  0.011  0.071  0.03  0  0  0.207  0.036  0.139  0.058  0.023  0.028  0.029  0  0.133  0.045  0.035  0.049  0.063  0.038  0  0  0  0.019  0.12  0.011  0.044  0.131  0.049  0.02  0  0  0.015  
 
 LOC100506299  100506299  2  8811113  8821993  -  1.208  0.291  0.589  0  0.447  0.211  0.709  0.335  0.395  0.713  0.257  0.351  0.613  0.971  0.546  0.422  0.369  0.205  0  0.331  0  0.146  0.944  0  0.25  0.535  1.144  0.871  0.583  1.657  1.144  0.518  0.624  0.799  1.058  1.283  0.267  0.375  0.718  0.727  0.932  0.838  1.21  0.295  0.337  0.323  0.862  0  0.51  0.286  0.479  1.088  0.369  0.563  0.814  0.626  0.435  0.426  0.627  0.485  
 
 ID2  3398  2  8822112  8824583  2p25  4.031  0.106  2.653  4.195  2.761  3.242  3.385  3.524  2.286  4.339  2.632  2.414  3.953  5.489  4.703  2.332  2.955  4.344  0.897  3.35  0.753  1.412  3.836  3.993  1.049  2.385  2.123  1.281  2.377  1.708  1.63  1.833  1.586  0.931  5.114  4.578  2.375  1.879  3.094  3.717  2.809  4.017  6.08  1.965  2.514  2.288  4.103  0  1.652  0.276  3.191  2.215  3.545  2.031  3.203  1.174  3.913  1.351  2.713  1.72  
 
 KIDINS220  57498  2  8868986  8977755  2p24  1.738  1.926  1.42  1.199  1.583  1.066  1.825  2.524  2.359  2.419  2.477  1.337  1.692  2  2.146  1.154  1.502  1.734  2.464  1.737  2.135  1.812  0.872  0.894  1.756  2.222  1.774  1.965  1.879  2.079  2.202  2.509  2.36  1.276  1.368  2.468  1.989  2.135  1.824  2.604  1.85  1.314  2.286  1.823  1.494  1.762  1.451  1.422  1.451  1.862  2.347  1.267  1.729  1.495  1.819  1.497  2.234  1.608  1.742  2.422  
 
 MBOAT2  129642  2  8996700  9143876  2p25.1  0.932  2.658  1.618  1.339  1.49  1.442  1.793  2.196  1.572  1.987  1.323  1.953  0.434  1.602  1.269  0.797  1.522  2.502  0.031  0.035  1.766  1.201  0.155  0.08  1.169  1.37  0.74  1.093  1  0.532  1.205  1.266  1.247  0.63  0.945  1.187  1.534  1.885  3.093  1.593  3.164  0.801  2.184  0.856  0.721  0.951  1.875  2.257  2.295  2.985  3.76  1.551  1.768  0.954  1.413  1.8  1.93  2.09  1.315  1.561  
 
 ASAP2  8853  2  9346893  9545812  2p25|2p24  2.247  4.106  1.903  1.598  1.41  1.086  2.232  2.435  1.961  1.95  2.52  1.651  2.176  1.942  2.505  3.207  2.631  3.492  0.091  0.106  0.534  0.98  0.795  0.752  1.61  2.657  2.355  2.433  2.629  0.916  2.195  2.231  2.231  1.323  1.631  2.909  2.897  2.434  3.917  1.595  2.895  1.452  2.674  2.212  1.303  2.302  3.599  1.408  2.559  2.617  3.102  1.786  2.616  2.149  2.81  1.87  2.683  3.065  2.42  3.018  
 
 ITGB1BP1  9270  2  9545814  9563643  2p25.2  3.059  6.303  2.744  1.931  3.054  2.11  4.384  3.703  2.723  3.529  3.979  2.185  2.92  3.901  2.396  2.778  3.015  5.473  4.9  4.223  2.489  3.781  2.254  2.519  3.727  3.993  2.975  2.48  3.401  2.876  4.368  3.971  3.416  2.403  2.937  3.421  3.114  3.552  4.799  3.256  3.112  2.893  4.623  2.681  2.215  2.152  4.248  2.995  2.61  4.908  3.339  2.593  3.552  2.619  4.024  3.222  3.525  4.198  3.43  3.026  
 
 CPSF3  51692  2  9563665  9613239  2p25.1  3.845  4.718  1.795  1.992  2.692  1.783  3.663  2.965  2.36  3.05  3.473  2.622  3.352  4.564  2.93  3.127  3.663  4.935  5.046  4.559  3.315  3.754  2.449  2.466  3.129  3.092  2.772  3.08  3.257  2.62  3.797  3.592  3.536  2.289  2.832  3.433  3.451  3.018  2.937  4.385  3.031  1.992  4.246  3.142  2.406  2.336  3.885  1.923  2.358  3.908  3.15  2.14  3.056  2.334  3.778  2.9  3.162  2.76  2.633  3.389  
 
 IAH1  285148  2  9614669  9628591  2p25.1  4.337  5.901  3.395  2.909  4.456  2.393  4.981  4.418  3.731  4.272  4.124  3.058  3.391  4.174  3.247  4.238  3.684  4.834  5.493  3.806  0.254  5.246  2.383  2.039  3.278  4.308  4.038  2.504  3.537  2.76  4.32  3.792  2.49  2.209  4.176  4.335  3.614  4.114  4.324  4.305  3.55  3.556  6.06  2.938  1.608  2.048  5.306  2.698  3.562  4.417  4.126  2.954  3.999  3.532  5.859  3.502  3.848  3.686  3.563  3.995  
 
 ADAM17  6868  2  9629410  9695917  2p25  1.766  3.008  1.621  2.324  1.326  0.974  2.516  2.705  3.004  2.113  2.712  1.37  2.611  2.587  1.753  2.139  1.928  2.152  2.726  2.231  1.747  1.982  1.348  1.095  2.71  1.778  1.179  2.344  1.846  1.326  2.024  2.396  2.15  1.122  2.102  2.359  2.864  1.832  3.574  3.007  2.762  1.293  1.912  2.208  1.619  1.414  3.006  1.313  1.509  2.81  1.832  1.644  1.992  2.002  2.825  2.289  2.922  2.471  1.79  2.662  
 
 YWHAQ  10971  2  9724095  9771184  2p25.1  5.868  8.126  5.225  5.251  5.36  4.401  7.467  6.038  6.129  6.232  6.988  5.437  5.52  7.096  4.92  5.508  5.698  7.746  7.812  5.885  4.812  5.884  4.889  4.944  6.015  5.279  5.123  5.436  5.397  4.605  5.666  5.741  5.691  4.614  5.612  5.983  6.179  6.308  6.239  6.447  5.521  5.488  7.2  5.529  5.608  4.789  5.712  4.863  5.037  7.141  6.444  4.81  5.751  6.032  6.081  5.842  6.348  5.781  5.635  6.041  
 
 TAF1B  9014  2  9983570  10074545  2p25  1.502  3.98  0.618  0.767  1.025  0.891  1.814  1.809  1.001  1.526  1.814  0.904  1.46  2.474  1.405  1.437  0.933  2.715  2.636  1.548  1.372  1.337  0.345  0.425  2.058  1.72  1.091  0.975  1.441  0.815  2.018  2.254  1.909  1.1  1.576  1.913  1.367  1.147  1.881  2.342  1.727  1.079  1.88  1.481  1.061  1.036  1.195  1.213  1.103  2.748  1.921  0.903  1.57  0.744  1.748  1.155  1.515  2.208  0.87  1.819  
 
 GRHL1  29841  2  10091791  10142412  2p25.1  1.81  0.148  0.2  0.104  1.786  0.071  0.136  0.541  0.329  0.416  0.383  0.889  0.556  0.865  0.752  1.481  1.164  1.078  0.476  0.058  0.259  0.286  0.492  0.265  0.085  0.696  0.396  0.343  0.241  0.232  0.108  0.104  0.524  0.314  0.205  0.258  0.259  0.168  0.238  0.409  1.732  0.081  0.426  0.499  0.973  0.678  0.511  0.189  0.202  0.362  0.549  0.467  0.348  0.235  0.173  0.149  0.333  0.334  0.473  0.213  
 
 KLF11  8462  2  10183681  10194963  2p25  1.25  1.84  1.064  1.895  1.536  0.284  0.397  1.652  1.177  1.503  1.084  0.617  1.811  1.491  1.324  1.432  2.01  0.734  0.054  0.364  1.24  0.155  1.254  0.985  0.946  1.672  1.214  1.684  1.091  1.977  0.941  0.792  2.207  1.43  0.697  1.281  0.947  1.019  1.182  1.877  1.112  0.801  1.842  1.2  1.222  1.541  0.16  1.025  2.113  1.876  1.754  1.595  1.593  1.947  2.525  2.038  2.229  1.257  2.682  1.584  
 
 CYS1  192668  2  10196925  10220538  2p25.1  0.086  0.082  0.599  0.347  0.679  0.128  0.154  1.018  0.783  0.462  0.335  0.047  0.072  0.74  0.153  0.079  0.089  0.081  0  0.058  0  0.153  0.454  0.051  0  0.091  0.184  0.041  0  0.164  0  0.111  0  0.239  0.176  0.455  0.131  0  0.088  0.379  0.359  0.432  1.274  2.019  0.115  0.164  0.429  0.153  0.368  0.764  0.094  0.964  0.235  0.237  0.18  0.314  0.858  0.325  0  1.455  
 
 RRM2  6241  2  10262694  10271546  2p25-p24  4.452  6.308  4.034  3.421  4.465  4.002  6.056  4.602  2.004  5.071  5.371  4.299  4.093  6.319  5.132  4.85  5.158  4.951  7.145  5.359  4.16  5.408  3.159  3.504  4.919  4.818  3.918  3.981  5.234  2.931  5.846  5.112  5.034  3.268  4.394  4.513  4.673  4.274  4.286  6.061  4.908  4.361  5.392  5.022  4.231  3.374  5.987  3.789  4.197  5.344  4.98  3.484  4.35  3.485  4.189  3.738  4.379  4.734  3.614  4.151  
 
 C2orf48  348738  2  10281508  10351856  2p25.1  0.713  0.369  0.359  0.353  0.454  0  0.325  0.869  0.241  0.615  1.228  0.384  0.933  1.042  0.714  0.717  0.427  0.195  3.048  0.829  0.683  1.406  0.482  0.33  0.162  0.289  1.046  0.319  0.269  0.411  0.479  0.186  0.997  0.393  0.561  0.24  0.691  0.296  0.181  0.657  1.178  0.795  0.557  0.144  0.245  0.796  0.458  0.846  0.258  0.866  0.221  0.495  1.029  0.287  1.441  0.21  0.706  1.455  0.797  0.115  
 
 HPCAL1  3241  2  10443029  10567743  2p25.1  3.606  6.018  4.324  4.851  2.912  2.133  7.072  5.302  4.531  4.943  4.871  4.146  3.098  4.834  2.627  4.178  2.505  3.693  3.198  4.698  3.468  3.131  4.003  3.581  3.375  5.61  4.795  5.335  3.896  3.288  3.931  4.657  4.321  4.191  3.672  4.036  6.605  5.449  4.374  4.04  3.857  5.639  3.892  2.217  1.971  2.528  5.907  3.314  2.814  5.062  6.106  5.38  4.355  5.083  4.75  4.792  4.787  5.764  3.506  3.8  
 
 ODC1  4953  2  10580496  10588680  2p25  5.309  6.273  4.271  3.885  3.221  3.816  7.039  4.376  4.839  3.957  6.441  4.487  5.072  7.066  5.53  4.851  6.774  6.668  7.179  6.619  6.147  6.317  5.478  5.415  5.076  3.568  3.369  4.825  3.247  3.788  4.994  5.949  2.617  2.383  4.4  5.841  6.157  5.011  5.689  6.591  3.956  5.582  7.205  5.842  4.808  3.893  5.742  4.52  3.725  6.669  7.007  4.263  5.326  2.925  5.105  4.168  4.334  5.059  5.2  5.885  
 
 SNORA80B  100302743  2  10586839  10586975  2p25.1  0  0  0  0  2.372  0  2.82  0  0  0  0  0  0  0  0  2.253  2.458  0  2.973  2.75  0  3.106  0  3.78  0  0  2.849  0  0  0  0  0  0  0  0  0  0  0  2.001  0  0  0  3.404  0  3.447  0  0  0  0  2.927  0  0  0  0  0  0  0  0  0  0  
 
 LOC101929715  101929715  2  10589853  10595678  2p25.1  0  0  0  0  0  0  0.178  0  0  0.081  0.116  0  0.459  0.539  0  0.353  0.427  0  0.217  0.111  0.443  0.155  0.728  0  0  0  0.072  0.134  0  0.441  0  0  0  0  0.272  0.431  0.368  0.307  0.137  0.5  0  0.297  0.916  0.183  0  0.09  0  0  0  0.278  0.389  0.732  0  0.198  0.375  0  0  0  0.154  0.181  
 
 NOL10  79954  2  10710891  10830113  2p25.1  1.745  2.638  1.397  1.253  1.804  1.076  2.901  2.082  2.143  1.966  2.387  1.563  1.959  2.548  1.737  1.925  1.716  1.055  3.284  2.813  2.429  2.062  1.834  1.374  2.474  1.989  1.599  1.912  1.846  1.304  2.321  2.806  2.521  1.528  1.977  2.517  2.101  1.979  2.137  3.098  2.18  2.013  2.62  2.313  1.519  1.345  2.914  1.403  1.541  3.05  2.12  1.391  2.06  1.561  2.089  1.722  1.978  2.005  1.653  2.207  
 
 ATP6V1C2  245973  2  10861774  10925236  -  2.134  2.115  2.858  3.216  1.161  0.483  2.294  1.366  3.985  1.985  1.353  1.525  1.014  1.859  1.783  2.111  2.184  1.165  1.781  2.036  0.305  1.817  3.958  1.682  1.418  1.826  2.358  1.261  2.379  1.661  1.733  1.677  1.364  2.037  1.47  1.019  1.64  1.221  1.711  1.728  1.857  2.457  2.851  0.881  0.811  0.89  4.45  1.003  0.345  1.461  1.152  0.721  1.952  1.054  2.836  1.55  1.387  0.878  1.008  0.839  
 
 PDIA6  10130  2  10923516  10978103  2p25.1  4.129  6.706  4.945  5.273  4.266  4.077  5.324  6.014  6.352  5.976  5.787  5.4  4.648  4.922  4.979  5.427  5.522  5.868  5.916  6.09  4.618  3.876  5.339  5.395  5.43  5.865  4.13  4.967  5.433  4.449  6.147  6.381  5.223  3.94  4.519  6.048  5.599  5.405  5.899  6.378  5.23  5.088  4.908  5.667  4.704  4.788  4.244  4.135  4.561  6.554  5.631  4.353  5.097  4.623  5.498  5.467  5.95  5.066  4.92  6.048  
 
 KCNF1  3754  2  11052062  11054351  2p25  1.057  0.185  0.265  0  2.505  0.214  0.524  0.403  0.554  0.659  0.359  0.379  0.092  0.432  0.127  0.408  0.993  0.36  0.144  0.478  0.104  0.715  0.541  0.242  0.181  0.1  0.434  0.135  0.321  0.331  0.078  0.104  0  0.445  1.147  1.681  0.551  0  0.471  2.411  0.948  1.475  1.795  0  0.231  0.249  0.953  0.277  0.096  0.506  0.339  1.961  0.247  0.351  0.521  0.234  0.185  0.224  0.059  0.093  
 
 FLJ33534  285150  2  11239976  11272302  2p25.1  0  0  0  0  0.401  0  0  0  0.09  0.114  0  0.059  0  0.08  0  0  0  0  0  0  0  0.099  0.289  0  0  0  0.059  0  0  0.047  0  0  0  0  0  0  0  0  0.04  0  0  0.567  0.032  0  0  0  0.034  0  0  0  0  0.253  0  0  0.059  0  0  0.096  0  0  
 
 C2orf50  130813  2  11273178  11286916  2p25.1  0.282  0  0  0  0.32  0  0  0  0.206  0  0.069  0  0  0.178  0.211  0.124  0  0  0  0  0  0.163  0.399  0  0  0  0.062  0  0  0.07  0  0  0  0.265  0  0  0  0  0  0.161  0  0  0  0  0  0  0.046  0  0  0.126  0  0.434  0  0.283  0  0  0  0.216  0  0  
 
 PQLC3  130814  2  11295497  11318998  2p25.1  3.45  4.064  1.677  1.403  3.615  0.566  2.219  1.461  1.958  2.082  1.693  1.111  2.174  3.338  1.705  1.529  1.266  2.307  2.116  1.333  0.834  1.46  1.316  0.854  1.638  3.091  2.449  1.928  2.197  2.244  2.259  2.249  1.788  1.807  2.453  2.962  1.724  2.362  2.405  2.099  1.565  1.434  3.484  1.785  0.453  0.679  3.509  0.962  1.866  2.007  2.123  1.514  1.914  2.109  2.188  1.451  2.054  1.69  1.729  2.113  
 
 ROCK2  9475  2  11321777  11484711  2p24  1.848  3.716  1.552  0.997  1.355  1.26  2.043  2.591  1.517  2.357  2.38  2.004  2.683  2.752  2.363  2.41  1.86  2.99  1.639  0.943  1.533  1.23  0.573  0.649  2.19  2.762  1.647  2.91  2.116  1.068  1.811  2.409  2.619  1.26  2.236  2.894  2.37  2.499  2.123  2.826  1.779  1.205  2.41  2.052  1.471  1.349  1.458  1.627  2.136  2.194  2.653  1.019  1.761  1.184  2.423  1.731  2.687  1.353  1.473  2.38  
 
 LINC00570  100874055  2  11534106  11543203  -  0  0  0.128  0  0  0.155  0  0  0.144  0  0  0  0  0  0  0  0  0.128  0  0  0  0.127  0.348  0  0  0  0.054  0  0  0.065  0  0  0  0.463  0  0  0  0  0  0  0  0.052  0  0  0  0  0.051  0  0  0  0  0.363  0  0  0  0  0  0  0  0  
 
 E2F6  1876  2  11584500  11606303  2p25.1  2.182  2.57  0.944  1.196  1.82  0.682  2.6  1.642  1.717  1.549  2.082  1.356  2.017  2.744  1.207  1.84  1.541  1.615  2.843  2.026  1.755  1.836  1.387  0.792  1.856  1.849  1.956  1.56  1.879  1.774  2.547  2.148  1.875  1.204  1.811  1.721  1.716  1.518  2.098  2.551  1.843  1.276  2.936  1.416  1.121  0.849  2.596  1.182  1.019  3.143  1.437  1.188  1.221  1.153  2.13  1.305  1.449  1.639  1.417  1.357  
 
 GREB1  9687  2  11674241  11782912  2p25.1  4.421  0.058  0.293  0.129  4.576  0.042  0.191  0.14  0.236  0.153  0.129  0.103  0.084  0.669  0  0.062  0.062  0.149  0.366  0.147  0  0.34  0.669  0.176  0.341  2.77  0.563  2.018  3.228  2.804  3.755  3.073  1.987  0.913  0.197  0.095  0.288  0.109  0.534  0.322  0.149  0.4  1.185  1.15  0.28  0.218  0.122  0.252  0.104  0.374  0.087  0.804  0.057  0.604  0.205  0.145  0.057  0  0  0.155  
 
 NTSR2  23620  2  11798303  11810329  2p25.1  0  0.073  0.115  0  0.174  0.148  0.052  0.049  0.15  0.077  0.056  0.184  0  0.078  0  0.08  0  0.419  0  0.145  0  0.571  0.501  0.119  0  0  0.274  0  0  0.191  0.071  0.156  0  0.24  0.126  0  0  0  0.178  0  0  0.621  0.107  0  0  0.064  0.107  0  0  0  0.065  1.571  0  0.211  0.222  0.083  0  0  0  0  
 
 LPIN1  23175  2  11817704  11967533  2p25.1  1.931  3.401  1.703  2.685  0.992  1.168  2.647  2.523  2.41  3.159  3.517  1.28  1.582  1.809  1.672  0.96  3.048  1.288  2.649  1.612  2.213  2.636  3.031  2.537  3.13  2.548  2.775  2.134  2.932  2.032  3.036  2.61  2.519  2.179  1.465  2.177  3.111  2.493  2.708  2.376  2.416  1.975  1.843  1.948  2.114  1.849  1.441  1.044  1.527  2.681  2.808  1.989  2.892  1.858  2.82  1.96  2.217  3.53  2.008  2.972  
 
 LOC100506457  100506457  2  12147241  12718474  -  0  1.503  0.25  0  0  0.667  0  0  0.751  0.584  0.629  0.17  0  0  0  0  0  0  0  0  0.468  0.396  0.486  0  0  0.192  1.121  0.741  0.477  0.271  0.279  3.53  0.955  1.45  0  0  0.298  1.105  0.221  0  0  1.321  0  0  0  0  0.814  0  0.405  0  0  0.446  0  0.282  0  0  0  0  0  0  
 
 TRIB2  28951  2  12856997  12882858  2p24.3  0.288  2.037  2.278  1.481  0.063  0.266  1.335  0.77  5.621  3.504  2.894  1.418  0  1.944  0.318  0.104  0.847  0.231  2.048  0.103  1.839  1.678  0.884  0.078  0.725  3.765  4.329  2.9  4.229  1.388  4.583  4.764  4.026  3.138  0.072  1.815  1.456  0  0.202  1.109  2.375  1.326  0.765  1.3  1.972  0.58  1.552  0.217  2.057  0.94  1.388  0.619  0.157  0.265  0.142  0.075  0.859  0.113  0.046  0.154  
 
 LOC100506474  100506474  2  13106907  13147138  2p24  0  0.049  0.081  0  0  0  0  0  0.214  0.038  0  0  0  0.033  0  0  0  0  0  0  0  0.039  0  0  0  0.386  0.76  0  0.16  0.368  0.895  2.453  0.526  0.616  0  0  0  0.121  0  0  0  0  0  0  0  0  0.029  0  0  0  0  0  0  0.029  0  0  0  0  0  0  
 
 LINC00276  100499171  2  14368997  14541082  2p24.3  0  0  0.077  0  0  0.077  0  0  0.084  0  0  0  0  0  0  0  0  0  0  0  0  0  0.133  0  0  0  0.024  0  0  0.023  0  0  0  0.169  0  0  0  0  0.015  0  0  0.07  0.015  0  0  0  0.022  0.083  0  0.022  0  0.05  0  0  0  0  0  0  0  0  
 
 FAM84A  151354  2  14772809  14780168  2p24.3  0.034  0.068  0.214  1.323  0.031  0.245  0.757  0.027  0.517  0.084  1.336  0.049  2.059  0.026  0.088  0.042  0.165  0.071  0.028  0.08  0.1  0.204  0.203  0.034  0.294  0.034  0.119  0.015  0.064  0.085  0  0  0  0.161  0.034  0.033  0.034  0  0.061  0  1.67  0.44  0.051  1.05  0.053  0.051  0.367  0.162  0.1  0.231  1.032  0.605  0.03  0.337  0.106  0.066  0.031  0  0  0  
 
 LOC653602  653602  2  14775214  14790943  2p24.3  0  0  0.099  0.829  0  0  0.501  0  0.667  0.174  0.951  0  1.51  0  0.127  0  0.226  0  0  0  0.144  0  0.204  0  0.247  0  0.024  0  0.061  0.028  0  0  0  0.156  0  0  0  0  0.04  0.101  1.231  0  0.251  0  0  0  0.091  0.12  0  0.694  0.644  0  0  0.375  0  0  0  0  0  0  
 
 NBAS  51594  2  15307031  15701472  2p24  0.996  0.916  1.393  0.893  1.279  0.974  1.556  1.781  1.582  2.02  1.567  0.819  1.468  1.484  1.727  1.122  1.5  0.528  2.415  1.363  2.363  1.337  0.922  0.896  1.516  1.73  1.256  1.578  1.903  1.595  1.443  1.81  1.587  0.81  1.07  1.885  1.544  1.412  1.315  2.17  1.541  0.952  1.373  1.454  0.803  1.106  0.924  1.158  1.159  1.815  1.925  1.13  1.251  0.884  1.983  1.584  2.026  1.116  1.254  1.901  
 
 DDX1  1653  2  15731744  15771235  2p24  3.392  5.348  2.348  2.226  3.412  2.631  3.957  3.638  2.579  3.44  3.691  2.734  3.271  4.96  3.149  3.495  3.627  5.724  5.572  4.139  4.114  3.56  2.409  2.67  3.824  3.638  2.973  3.587  3.632  2.968  4.526  4.859  3.613  1.974  3.089  4.015  3.695  3.459  3.82  4.673  3.517  2.954  4.075  3.818  2.776  3.287  3.581  3.126  3.171  5.952  3.41  2.411  3.355  2.726  3.946  3.11  4.208  3.335  3.08  3.978  
 
 LOC101926966  101926966  2  15830905  15859085  2p24.3  0  0  0  0  0  0  0  0  0.142  0  0  0  0  0  0  0  0  0.066  0  0.049  0  0.047  0.259  0  0  0  0.058  0.328  0  0.062  0  0  0  0.195  0  0  0  0  0  0  0  0.063  0.036  0.324  0  0  0.031  0  0  0  0  0.206  0  0  0  0  0  0  0  0  
 
 MYCNUT  103752554  2  16060520  16076140  2p24.3  0  0  0.133  0  0  0  0  0  0  0  0  0  0  0  0  0  0  0  0  0  0  0.092  0.325  0  0  0  0.053  0  0  0.064  0  0  0  0.206  0  0  0  0  0  0  0  0.256  0.04  0  0  0  0.049  0  0  0  0  0.361  0  0  0  0  0  0  0  0  
 
 MYCNOS  10408  2  16076386  16081845  2p24.1  0  0  0.263  0  0.102  0  0  0  0.134  0.076  0.09  0.396  0  0.057  0  0  0  0.171  0.705  0  0  0.264  0.492  0.16  0  0  0.197  0  0  0.15  0  0  0  0.363  0  0.428  0  0  0.176  0.362  0  0.107  0.058  0.283  0  0.188  0.07  0  0  0  0  0.691  0  0.158  0.117  0  0  0  0  0  
 
 MYCN  4613  2  16080559  16087129  2p24.3  0.17  0.177  0.198  0  0.221  0.844  0.118  0.358  0.819  1.068  0.083  2.868  0  0.206  0.13  0.174  0.201  0.227  2.388  0.256  0.204  0.879  0.557  1.275  0.115  0.083  1.098  0.129  0.077  0.618  0.296  0.343  0.175  0.238  0.109  1.094  0.07  0.112  0.252  2.905  0.221  1.541  0.189  0.717  0.733  0.683  0.852  0.229  0.193  0.074  0.119  1.091  0.14  0.415  0.175  0.338  0.061  0.413  0  0.154  
 
 GACAT3  104797537  2  16190548  16225811  2p24.3  0  0  0  0  0  0  0  0  0  0  0  0  0  0  0  0  0  0  0  0  0  0  0.387  0  0  0  0  0  0  0.038  0  0  0  0  0  0  0  0  0.056  0  0  0  0  0  0  0  0  0  0  0  0  0.266  0  0.08  0  0  0  0  0  0  
 
 FAM49A  81553  2  16730729  16847134  2p24.2  0  0  0.143  0  0.118  0.314  0.999  0.075  0.411  0.098  0.136  0.031  0  0.152  0  0  0  0  0  0.154  0  0.073  0.141  0  0.024  0  0.095  0.021  0.067  0.074  0  0.063  0  0.127  0.027  0  0  0.098  0.812  0.059  0.053  0.138  0.023  0.054  0.634  0.606  0.33  0  0.461  0  0.02  0.138  0.882  1.71  4.151  0.89  1.822  0  2.125  2.921  
 
 RAD51AP2  729475  2  17691985  17699706  2p24.2  0  0  0.068  0  0  0  0  0  0  0  0  0  0  0.039  0  0  0.039  0.145  0  0  0  0  0.142  0  0  0  0.024  0  0  0.024  0  0  0  0.22  0  0  0  0  0  0  0  0.019  0.018  0  0  0  0.016  0  0  0  0  0.069  0  0  0.017  0  0  0  0  0  
 
 VSNL1  7447  2  17721806  17837706  2p24.3  0  0.055  0.611  0  0.036  0.361  2.826  0.482  0.568  0.472  3.609  2.946  1.567  2.578  3.857  0.077  3.398  5.199  0  0.064  0  0.413  0.173  0  0.384  0.063  0.065  0  0  0.137  0.109  0.236  0.08  0.212  0  0.104  0.354  0.244  0.049  0.351  0.269  2.531  0.388  0  0.165  0.094  0.062  0.075  0.322  0  0  0.442  0.052  0.035  0.097  0.092  0.12  0.142  0  0  
 
 SMC6  79677  2  17845078  17935096  2p24.2  1.45  3.666  0.892  0.845  1.108  1.266  1.865  1.375  0.833  1.635  2.141  1.02  1.603  2.456  1.456  1.752  1.874  3.327  1.763  1.462  2.082  1.4  1.137  0.804  1.92  1.94  1.214  1.458  1.29  0.707  1.677  1.481  2.118  1.064  1.729  2.495  1.445  1.8  1.741  2.069  1.07  1.371  2.689  2.005  0.932  1.579  1.257  1.319  1.568  2.552  1.93  1.444  1.368  1.146  2.168  1.57  2.038  1.627  1.569  1.977  
 
 GEN1  348654  2  17935176  17966632  2p24.2  1.52  1.918  0.529  0.443  0.892  0.794  1.011  1.318  0.328  1.211  1.372  0.791  0.963  1.591  1.35  1.369  1.004  2.073  1.643  1.036  0.951  1.195  0.69  0.712  0.989  1.375  0.949  1.107  1.05  0.717  1.368  1.075  1.245  0.796  1.141  1.434  1.19  1.321  1.012  1.718  1.072  0.729  1.107  1.134  0.742  1.121  0.665  0.866  1.132  1.523  1.096  0.909  1.209  0.93  1.189  0.85  1.709  0.656  1.342  1.131  
 
 MSGN1  343930  2  17997785  17998367  2p24.2  0  0  0  0  0  0  0  0  0  0  0  0  0  0  0  0  0  0  0  0  0  0.179  0.452  0  0  0  0  0  0  0  0  0  0  0.446  0  0  0  0  0  0  0  0  0  0  0  0  0  0  0  0  0  0  0  0  0  0  0  0  0  0  
 
 KCNS3  3790  2  18059113  18114225  2p24  1.437  0.188  1.173  1.23  1.618  0.936  1.8  1.101  1.514  2.225  0.056  2.248  1.001  1.22  0.701  0  0.068  0.55  0  0.077  0.142  0.185  1.259  0.957  0  1.726  3.51  2.016  0.688  1.553  0.916  0.197  3.509  1.966  0.535  1.711  0.209  0  0.281  0.61  0.976  0.392  0.065  0.06  0  0.077  0.227  0  0.553  0  0.04  1.243  1.9  1.214  2.212  0.108  1.982  0  1.581  0.239  
 
 RDH14  57665  2  18735988  18741959  2p24.2  1.921  4.012  1.68  2.139  2.439  1.163  2.16  1.925  2.434  2.317  2.359  1.237  2.281  2.557  1.599  2.224  1.875  3.155  2.551  1.665  1.607  1.842  1.706  0.803  1.729  1.889  1.179  1.408  1.627  1.086  2.294  2.492  1.758  1.712  1.644  2.609  1.913  1.559  2.914  2.158  2.327  2.61  2.41  1.851  1.092  1.224  1.881  1.014  1.546  2.898  2.342  1.697  1.805  1.471  2.504  1.907  1.386  1.461  1.592  1.877  
 
 NT5C1B-RDH14  100526794  2  18735988  18770846  2p  0  0  0  0  0.059  0.122  0  0.065  0  0.122  0.037  0.072  0.087  0.03  0  0.051  0  0.31  0  0.079  0  0.197  0  0  0  0.072  0  0  0  0.048  0  0  0.081  0.163  0  0.051  0  0  0.26  0  0.083  0.134  0.695  0  0  0.078  0  0  0  0.039  0.109  0.239  0.1  0.16  0.111  0  0  0  0  0.054  
 
 NT5C1B  93034  2  18744136  18770846  2p24.2  0  0  0.086  0  0.077  0  0  0  0  0  0  0.098  0  0.034  0  0  0  0  0.04  0  0  0.107  0.218  0  0  0  0.115  0.043  0.059  0.05  0  0  0  0.114  0  0  0  0  0.098  0  0  0.116  0  0  0  0.041  0.065  0  0  0  0.06  0.215  0  0.086  0.038  0  0.057  0  0  0  
 
 OSR1  130497  2  19551245  19558372  2p24.1  0  0.237  1.244  0.172  0.061  0.5  0.199  0.474  1.515  3.88  0.201  0.795  0.241  0.283  0.118  0.194  0.785  0.208  0  0.118  0  0.271  0.588  0  0  0.11  0.199  0  0  0.156  0  0  0  0.226  0.755  0.896  0.303  0  1.555  1.052  0.815  1.587  0.257  0  0.101  0  0.43  0  0  0  0.601  0.842  0  0.59  0.516  0.092  0.091  0.098  0.206  0.089  
 
 LINC00954  400946  2  20068614  20084808  2p24.1  0  0  0.089  0  0.075  0  0.033  0.031  0.083  0.054  0  0  0  0.025  0  0.039  0  0.033  0.121  0.071  0  0.152  0.306  0  0  0  0.219  0  0  0.058  0  0  0  0.255  0.059  0  0.045  0.173  0.025  0.083  0.049  0.142  0.037  0  0  0.029  0.141  0  0  0  0.024  0.256  0.035  0.061  0.035  0  0.165  0  0  0  
 
 TTC32  130502  2  20096513  20101744  2p24.1  1.614  3.379  0.699  1.257  2.496  0.927  1.061  1.266  1.014  1.518  1.481  1.386  1.13  1.943  0.727  2.084  1.151  3.215  2.994  2.137  2.522  1.633  2.146  1.252  1.114  0.966  0.964  0.624  1.028  1.336  1.754  1.541  1.302  1.55  1.588  1.7  0.256  0.771  2.46  1.611  1.82  1.694  2.241  1.318  0.804  1.042  1.524  0.488  0.839  2.546  1.846  0.916  0.811  1.299  2.02  0.71  1.45  0.696  0.982  1.584  
 
 WDR35  57539  2  20110028  20189884  2p24.1  0.422  1.668  0.413  0.605  0.676  0.417  0.745  0.752  1.078  0.744  1.003  0.272  0.487  1.09  0.48  0.603  0.686  1.586  0.013  1.115  0.691  0.078  0.258  0.309  1.062  1.131  0.783  1.342  1.175  0.852  1.035  1.394  1.464  0.859  0.87  1.279  0.79  1.267  0.528  2.177  0.835  0.618  1.7  0.848  0.567  0.477  0.079  0.692  0.734  1.7  1.213  0.769  0.766  0.604  1.649  0.721  0.857  0.79  0.607  1.227  
 
 LOC101928222  101928222  2  20189964  20204567  2p24.1  0.421  0  0.168  0.243  0.57  0.131  0.236  0.097  0.178  0.071  0.09  0  0  0.17  0  0.158  0.22  0.27  0  0.104  0  0.149  0.436  0  0  0.104  0.188  0  0.129  0.08  0  0  0  0.242  0.214  0.162  0.229  0.135  0.056  0.228  0.61  0.087  0.045  0  0.37  0.17  0.693  0  0  0.211  0.247  0.501  0.071  0.175  0.563  0  0.119  0  0  0.154  
 
 MATN3  4148  2  20191812  20212455  2p24-p23  0  1.646  1.064  0.364  2.432  0.284  0.591  0.801  0.079  0.374  0.43  0.109  0.069  0.046  0.096  0.129  0.096  0.13  0.026  0.074  0  0.076  0.154  0  0.051  0.262  0.09  0.124  0.226  0.073  0.26  0.452  0.069  0.18  0.424  1.939  0.917  1.388  0.293  0.677  0.168  0.465  0.794  0.764  0.081  0.097  0.211  0.638  0.878  1.666  1.009  0.348  0.13  0.622  2.87  1.169  1.532  0.127  0  1.527  
 
 LAPTM4A  9741  2  20232410  20251789  2p24.1  3.276  5.838  4.474  4.33  4.032  3.704  4.634  4.656  4.272  5.165  4.298  3.121  3.617  4.263  2.852  3.496  3.851  4.584  3.968  2.586  2.378  2.333  3.021  2.108  3.555  3.775  3.425  2.89  3.935  2.257  3.887  3.928  3.369  2.592  3.53  4.605  2.988  4.03  5.43  4.691  3.774  3.608  3.489  3.915  2.981  3.642  2.999  3.279  4.942  5.628  4.206  2.946  3.381  4.685  5.566  4.894  4.683  4.237  3.493  4.686  
 
 SDC1  6382  2  20400557  20425194  2p24.1  2.78  1.779  3.221  3.255  3.086  2.632  4.963  4.647  4.032  2.794  4.693  6.487  4.473  2.853  3.439  4.315  4.555  0.516  0.208  0.274  0.351  0.529  3.894  4.115  3.756  0.688  2.234  0.785  2.404  1.287  1.229  1.561  3.733  2.375  3.884  4.351  3.045  4.089  4.052  5.295  5.509  4.469  4.05  2.139  3.755  3.436  4.079  3.631  3.752  4.715  5.558  4.125  4.248  4.592  4.73  4.101  4.048  3.341  3.101  4.264  
 
 PUM2  23369  2  20448452  20550611  2p22-p21  2.942  3.774  1.645  1.7  2.222  1.595  2.756  2.711  2.599  2.635  3.031  2.161  2.733  3.467  3.095  2.831  2.811  3.522  4.263  3.111  2.145  3.263  2.094  2.063  2.607  2.785  2.313  2.707  2.753  2.292  3.018  2.686  3.001  1.784  2.043  2.933  2.983  2.578  2.594  3.45  2.88  1.644  2.919  2.662  2.255  2.252  2.588  2.096  2.081  3.139  2.622  1.913  2.05  1.878  2.82  2.317  2.639  2.22  2.197  2.848  
 
 RHOB  388  2  20646831  20649204  2p24  5.231  1.635  2.304  2.597  4.633  2.585  3.071  4.274  3.519  3.828  4.197  2.17  3.438  4.123  3.017  2.327  1.458  2.243  0.521  0.608  1.072  0.945  2.247  2.394  1.479  3.477  3.272  3.64  2.503  3.568  2.4  2.51  3.372  2.666  2.953  2.689  1.809  2.91  2.627  2.401  2.645  5.974  2.512  3.535  3.728  3.646  2.399  3.719  2.534  5.563  2.018  2.088  2.01  3.388  5.639  2.832  5.215  2.266  1.775  2.493  
 
 HS1BP3-IT1  100874343  2  20790534  20792308  -  0  0  0  0  0  0  0  0.134  0  0  0.159  0.477  0  0  0  0.253  0  1.407  0  0  0  0.215  0  0  0  0  0.168  0  0  0  0  0  0  0.534  0  0.228  0  0.386  0  0  0  0  0  0.256  0  0  0.933  0  0  0  0  0  0.141  0  0.632  0  0.884  0  0  0  
 
 HS1BP3  64342  2  20817563  20850864  2p24.1  1.436  0.184  1.325  2.459  1.732  1.792  2.437  1.881  2.553  2.42  2.143  1.987  2.476  1.527  1.773  1.703  2.074  0.051  1.456  0.986  0.896  0.609  1.544  1.314  1.919  2.831  2.984  2.556  3.138  3.328  3.711  3.168  2.804  1.943  2.202  2.044  1.823  2.963  1.41  2.793  2.293  2.516  3.459  2.339  0.9  1.638  2.596  1.275  1.388  2.615  1.925  1.534  1.097  2.919  3.486  2.298  2.108  2.248  1.595  2.064  
 
 GDF7  151449  2  20866423  20871250  2p24.1  0  0.039  0.205  0  0.147  0.167  0.048  0.108  0.209  0.126  0.09  0.144  0  0.113  0  0.131  0.167  0.279  0.046  0.164  0.134  0.74  0.442  0.125  0.084  0.085  0.209  0.082  0.059  0.452  0.13  0.068  0  0.267  0.128  0.14  0.067  0  0.206  0  0.068  1.148  0.146  0.082  0.218  0.204  0.081  0  0.098  0.066  0.127  1.565  0.072  0.226  0.322  0.183  0.076  0.113  0  0.127  
 
 LDAH  60526  2  20883773  21022890  2p24.1  1.572  1.746  0.972  0  1.43  0.663  2.455  1.837  1.789  1.459  1.611  1.341  1.829  2.833  1.766  1.644  1.537  2.506  3.58  2.721  1.917  2.823  1.458  1.328  1.998  1.873  1.234  1.747  1.336  1.264  2.16  1.664  1.276  0.881  1.469  1.703  2.122  1.577  1.196  2.043  1.961  1.45  2.167  1.377  0.736  0.72  2.946  0.583  1.118  1.183  0  0.927  1.594  0.809  2.169  1.396  1.602  1.229  1.29  1.676  
 
 APOB  338  2  21224300  21266945  2p24-p23  0  0  0.099  0  0.014  0  0.012  0.016  0.059  0.016  0.02  0.018  0  0.018  0  0.025  0  0.053  0.013  0.029  0  0.074  0.186  0  0  0.009  0.045  0.009  0  0.044  0  0.009  0  0.197  0.008  0  0  0  0.028  0  0.012  0.19  0.027  0  0.017  0.011  0.027  0  0  0  0.01  0.387  0.009  0.039  0.023  0.017  0  0  0  0  
 
 TDRD15  100129278  2  21346857  21367238  2p24.1  0  0.015  0.057  0  0.009  0  0  0  0.046  0.011  0  0.023  0  0.015  0  0  0.022  0.074  0  0.024  0.034  0.028  0.074  0  0  0  0.034  0.02  0  0.026  0  0  0  0.215  0  0  0  0  0.02  0  0  0.019  0.018  0  0  0  0.022  0  0  0  0  0.147  0  0.011  0.015  0.024  0  0  0  0  
 
 LOC645949  645949  2  21910305  21933524  2p24.1  0  0  0.283  0  0  0  0.08  0.03  0.179  0.025  0  0  0  0  0  0  0  0  0  0  0  0.045  0.465  0  0  0.183  0.04  0  0.06  0.29  0  0.407  0.411  0.859  0  0  0  0  0  0  0  0.038  0  0  0  0  0.058  0  0  0  0  0.085  0  0.046  0  0  0  0  0  0  
 
 KLHL29  114818  2  23608297  23931483  2p24.1  0.19  0.938  1.292  0.414  0.068  0.589  1.231  0.118  0.636  1.785  0.527  0.672  0.165  1.478  0.848  0.14  0.084  0.312  0.191  0.135  0.092  0.508  0.294  0.131  1.49  1.27  0.731  2.32  0.606  0.7  1.836  2.03  0  0.243  0.587  0.48  1.395  1.686  1.883  0.758  0.227  0.641  0.124  0.634  1.78  1.284  1.873  1.057  0.712  1.287  0.397  1.203  0.844  0.689  1.559  1.085  1.139  1.196  1.171  1.198  
 
 ATAD2B  54454  2  23971533  24149984  2p24.1-p23.3  1.111  0.683  0.339  0.415  0.74  0.319  0.436  0.822  0.536  0.571  0.824  0.318  0.649  0.773  0.687  0.562  0.412  0.769  1.631  0.628  0.905  0.929  0.301  0.111  0.381  0.713  0.324  0.412  0.366  0.3  0.593  0.521  0.425  0.314  0.577  1.226  0.52  0.36  0.579  1.096  1.176  0.464  1.287  0.94  0.378  0.742  0.513  0.257  0.535  0.431  0.62  0.414  0.372  0.468  0.496  0.332  0.716  0.322  0.329  0.433  
 
 UBXN2A  165324  2  24163375  24223693  2p23.3  1.69  2.764  1.501  1.168  1.501  1.39  1.634  1.944  2.093  2.065  1.976  0.966  1.502  2.041  1.278  1.33  1.625  1.952  2.706  0.789  1.86  1.402  1.2  0.665  1.642  2.435  1.254  1.234  1.682  0.845  2.316  2.341  1.433  1.341  1.531  1.843  1.516  1.257  1.847  2.206  2.087  1.488  2.475  1.804  1.176  1.817  1.69  1.113  1.985  2.451  1.663  1.217  1.31  1.343  1.977  1.532  1.838  1.559  1.329  1.738  
 
 MFSD2B  388931  2  24232952  24247145  2p23.3  0.214  0  0.114  0  0  0  0.077  0.049  0  0.069  0.113  0  0  0.086  0.167  0  0  0  0.103  0  0.739  0.102  0  0  0  0  0.233  0.077  0  0.104  0  0  0.083  0.17  0.111  0.163  0  0  0.082  0  0  0.243  0.082  0  0  0.068  0.097  0  0  0  0.068  0.465  0  0.066  0  0.167  0  0.088  0  0  
 
 C2orf44  80304  2  24252205  24270296  2p23.3  1.229  1.732  0.921  0.953  0.706  0.781  1.82  1.397  1.319  1.282  1.403  0.721  1.115  1.825  1.362  1.03  0.737  1.357  2.433  1.612  1.843  2.06  0.885  0.818  1.603  1.483  0.991  1.333  1.52  1.423  1.697  1.519  1.555  1.016  1.35  1.237  1.678  1.536  1.153  2.447  0.973  0.928  2.314  1.333  0.963  0.794  1.2  1.162  0.577  1.705  1.733  0.941  0.919  0.993  1.308  1.171  0.991  1.055  0.753  1.376  
 
 FKBP1B  2281  2  24272583  24286550  2p23.3  1.346  0.247  0.961  0.385  0.465  0.472  3.086  0.644  1.156  0.327  2.135  0.522  0.324  2.952  0.243  0  0.845  3.762  0.957  0  0.528  0.668  0  0  0.345  3.193  1.041  0.331  2.556  1.272  0.669  0.533  0  0.249  1.765  1.443  2.181  1.483  1.267  2.901  0  0.446  5.211  1.259  0.537  1.121  3.65  1.139  0.613  2.881  0.859  0.961  0.865  0.236  1.911  1.574  0.875  1.477  0.455  2.263  
 
 SF3B6  51639  2  24290453  24299314  2pter-p25.1  4.692  8.088  3.988  4.455  5.223  4.592  5.323  5.206  4.623  5.018  5.099  4.245  4.337  5.793  3.553  5.625  6.179  9.178  6.844  6.537  5.151  5.272  4.856  4.043  6.156  4.38  3.589  4.045  4.437  3.561  5.541  5.045  4.847  4.888  4.749  4.474  4.05  4.371  6.483  5.415  5.226  4.995  5.324  5.302  4.991  5.141  5.521  4.221  3.913  6.963  4.911  3.873  4.262  4.65  5.642  4.802  4.839  5.574  4.508  4.734  
 
 FAM228B  375190  2  24299743  24392507  2p23.3  0  2.244  0.452  0  1.444  0.331  0.733  1.045  0.876  0.882  0.182  0  0  0.689  0  0  0.128  0.302  1.263  1.231  0  0.953  0.399  0  0  0.728  0.443  0.19  0.345  0.056  0.667  0.305  0.319  0.743  0.63  0.51  0.299  0.761  1.434  0.629  2.234  0.716  0.306  0.212  0  0.279  1.758  0  0.192  0.778  1.226  0  0.357  1.004  1.086  0.274  0.958  0.333  0  0.587  
 
 TP53I3  9540  2  24300302  24308085  2p23.3  1.881  0.782  1.947  1.154  7.02  1.714  3.141  3.077  2.718  4.116  3.094  1.298  2.806  2.218  2.322  2.594  2.428  0.896  1.486  0.53  0.892  0.931  0.397  0.476  2.808  2.842  2.941  1.07  2.938  2.335  2.774  3.687  2.373  1.452  4.354  3.336  1.795  2.704  5.727  3.927  6.016  3.52  6.66  1.199  1.476  2.7  2.892  3.263  1.122  5.553  2.507  2.459  1.265  2.797  3.734  1.558  2.248  2.636  1.808  2.574  
 
 PFN4  375189  2  24337678  24346347  2p23.3  0.173  0.273  0.198  0  0.4  0.276  0.427  0.383  0.16  0.349  0.146  0  0.185  0.257  0  0.123  0.097  0.118  0.583  0.187  0  0.324  0.283  0  0.163  0.231  0.139  0.116  0.313  0.306  0.471  0.074  0.116  0.282  0.185  0.266  0.222  0.36  0.342  0.54  1.238  0.417  1.352  0  0.15  0.278  0.431  0  0  0.283  0.21  0.525  0.373  0.274  0.391  0.207  0.339  0.441  0.118  0.243  
 
 FAM228A  653140  2  24397911  24414567  2p23.3  0  0  0.093  0  0  0  0.066  0.12  0.115  0.071  0  0.11  0  0.054  0  0  0  0  0.122  0.056  0  0.083  0.229  0  0  0  0.057  0.041  0  0.052  0  0  0  0.194  0  0  0  0  0  0  0  0.159  0.029  0  0  0.055  0.028  0  0  0.056  0.077  0.116  0  0.084  0.1  0  0  0  0  0  
 
 ITSN2  50618  2  24425734  24583397  2pter-p25.1  1.297  2.11  0.63  0.427  1.096  0.616  1.241  1.643  0.949  1.308  1.575  0.828  1.235  1.712  0.907  1.014  1.157  1.883  2.021  1.136  0.905  1.316  0.812  0.565  1.443  1.579  0.991  1.362  1.373  1.123  1.646  1.405  1.497  0.728  1.092  1.621  1.19  1.242  1.765  1.86  1.885  0.858  1.508  1.182  0.69  0.961  1.216  0.861  1.356  1.298  1.488  1.008  0.94  0.958  1.497  0.958  1.642  1.126  1.084  1.447  
 
 NCOA1  8648  2  24807345  24993570  2p23  2.212  2.267  1.177  0.849  2.109  1.031  2.231  2.166  2.188  1.844  2.679  0.195  1.686  1.904  1.778  0.926  1.72  1.865  2.671  1.079  1.682  2.1  0.849  0.568  1.381  1.894  1.357  1.578  1.731  1.453  1.866  1.78  1.854  0.965  1.449  2.122  1.386  1.717  1.433  2.311  2.468  1.309  2.413  0.632  1.252  1.866  0.844  0.816  1.951  1.515  1.768  1.223  1.387  1.085  1.846  0.923  1.969  1.275  1.386  1.633  
 
 PTRHD1  391356  2  25013135  25016251  2p23.3  4.557  6.299  3.209  2.69  4.773  4.304  4.738  3.543  3.605  3.344  3.604  4.427  3.231  4.444  2.878  4.128  4.083  7.033  5.485  6.101  3.477  5.264  3.745  3.66  4.194  3.383  4.183  3.029  3.882  3.817  4.948  4.704  4.061  3.539  4.616  4.058  2.764  3.155  5.453  3.91  4.72  4.574  5.636  3.63  3.925  3.53  5.802  3.943  3.51  6.047  3.144  3.598  3.136  2.999  4.784  4.012  2.727  4.213  2.889  2.852  
 
 CENPO  79172  2  25016174  25045245  2p23.3  2.99  2.072  2.159  1.955  1.999  2.143  3.629  2.959  1.746  2.854  2.969  1.7  2.438  2.853  2.684  2.418  2.243  1.051  4.025  1.907  2.615  2.951  1.527  1.737  2.639  2.558  2.873  2.523  2.508  2.75  2.958  2.988  3.407  1.624  2.635  2.595  3.351  2.819  1.751  3.8  3.196  2.653  3.32  2.423  2.468  2.424  2.58  2.539  2.788  3.454  2.823  2.401  2.827  2.527  3.003  2.2  3.108  2.78  2.626  2.567  
 
 ADCY3  109  2  25042038  25142055  2p23.3  2.393  2.251  1.502  1.42  1.149  1.486  2.463  1.756  1.878  3.066  2.55  0.213  3.275  2.64  3.247  2.605  2.01  1.689  2.539  1.216  2.34  1.647  1.404  2.419  2.595  2.78  2.911  3.072  3.423  1.836  2.952  3.397  3.681  1.805  2.343  2.543  2.131  2.764  2.377  3.639  2.651  2.508  1.969  2.104  1.48  1.743  1.617  2.027  2.739  2.461  2.43  2.541  2.24  2.269  2.799  2.439  2.988  2.356  2.251  2.639  
 
 DNAJC27  51277  2  25166504  25194963  2p23.3  0.5  0.839  0.372  0.297  0.589  0.253  0.448  0.492  0.574  0.491  0.634  0.211  0.545  0.691  0.667  0.303  0.48  0.815  1.184  0.653  0.263  0.821  0.45  0.117  0.184  0.567  0.646  0.283  0.357  0.484  0.641  0.556  0.43  0.456  0.431  0.423  0.351  0.436  0.797  0.707  0.48  0.56  0.881  0.505  0.322  0.365  0.848  0.226  0.683  0.419  0.377  0.334  0.383  0.236  0.619  0.39  0.477  0.33  0.255  0.356  
 
 DNAJC27-AS1  729723  2  25194980  25262563  2p23.3  0.186  0.019  0.163  0.067  0.157  0  0.038  0.024  0.17  0.051  0.045  0.074  0.052  0.128  0.083  0.03  0.039  0.108  0.035  0  0  0.098  0.463  0.027  0  0.061  0.104  0.025  0  0.072  0.087  0.038  0  0.323  0.076  0.123  0.039  0  0.05  0.134  0.236  0.117  0.074  0  0  0.114  0.109  0  0  0.031  0.091  0.208  0.023  0.055  0.024  0.054  0  0.054  0  0.025  
 
 EFR3B  22979  2  25264972  25382004  2p23.3  0.774  0.031  0.137  0.747  0.13  0.44  0.313  0.448  0.851  1.362  1.508  0.052  0.097  0.126  0.564  0.038  0.072  0.118  0.072  0.047  0  0.159  0.33  0.074  0.326  2.366  1.625  1.027  1.493  1.167  1.492  1.074  2.538  0.908  0.817  1.695  0.061  0.129  0.371  1.295  0.519  1.029  2.366  0.325  0.078  0.366  0.358  0.182  0.292  0.194  2.36  1.086  0.036  0.221  0.099  0.047  0.238  0.815  0  0.036  
 
 POMC  5443  2  25383721  25391559  2p23.3  0  0.093  0.17  0  0.107  0  0  0.112  0.277  0.09  0  0  0  0.096  0  0.081  0.106  0.301  0.096  0.166  0  0.306  2.039  0.483  0  0  0.174  0.097  0  0.17  0  0  0  0.299  0.107  0.154  0  0  0.181  0.147  0  0.569  0.138  0  0.144  0.093  0.092  0  0  0.095  0  1.254  0  0.166  0.179  0  0  0.286  0  0  
 
 DNMT3A  1788  2  25455829  25565459  2p23  2.342  0.805  0.797  1.567  0.571  0.511  0.93  2.107  1.576  1.622  1.535  1.221  1.521  1.422  1.072  1.63  0.884  0.832  4.088  0.882  1.093  3.371  1.097  0.953  1.658  1.808  1.697  1.151  1.29  1.614  2.027  1.65  1.852  0.902  1.115  2.282  1.658  1.019  1.087  1.954  1.522  1.054  1.81  0.981  1.766  1.281  1.714  1.48  0.948  2.557  1.158  1.487  0.443  0.745  2.555  1.448  0.778  0.867  1.578  1.332  
 
 DTNB  1838  2  25600066  25896516  2p24  2.506  0.719  0.362  1.064  1.74  1.221  1.746  1.415  1.531  1.595  1.519  0.66  0.895  1.922  1.88  0.849  2.076  0.692  2.262  1.225  0  1.547  0.906  1.482  1.225  1.869  1.973  1.918  1.411  2.478  2.154  1.314  1.905  0.922  1.39  2.027  1.692  1.109  1.209  2.752  2.172  1.16  2.955  1.298  1.281  1.618  2.048  1.41  2.273  2.282  1.569  1.428  0.808  0.661  1.673  1.057  0.638  1.448  0.958  1.043  
 
 ASXL2  55252  2  25962252  26101312  2p24.1  2.651  2.532  1.449  1.349  1.871  1.552  2.631  2.631  1.739  2.257  2.628  2.066  2.363  2.829  2.683  2.352  2.299  1.718  1.906  2.779  2.022  2.368  1.139  1.618  2.453  2.897  2.395  3.055  2.929  2.494  3.008  2.622  3.319  1.661  2.156  2.622  2.631  2.582  2.137  3.82  3.179  1.391  2.985  2.161  2.203  2.212  2.316  2.204  2.675  1.606  2.721  2.094  2.49  1.924  3.066  2.31  2.814  2.223  2.296  2.808  
 
 KIF3C  3797  2  26149454  26205443  2p23  1.049  1.059  1.929  1.306  0.372  0.737  2.168  1.957  1.534  1.883  2.687  1.882  1.072  0.912  1  0.652  0.792  0.938  1.745  0.342  0.503  1.249  0.599  0.481  1.639  3.446  3.066  2.212  3.186  2.875  2.861  2.703  3.621  2.491  2.296  1.356  1.903  2.288  1.394  1.597  0.4  1.368  3.021  0.791  1.323  0.953  0.879  1.836  1.74  2.401  2.345  1.599  1.563  3.078  2.865  2.609  2.401  1.677  1.418  1.877  
 
 RAB10  10890  2  26256728  26360323  2p23.3  3.848  5.323  2.971  3.065  3.423  2.461  4.221  3.643  4.226  3.699  4.473  3.631  4.8  4.79  3.249  3.756  4.245  4.599  4.649  3.589  3.421  3.394  3.333  2.74  4.172  3.876  2.699  3.035  3.292  2.849  4.034  3.787  3.427  2.783  4.36  4.242  3.474  3.558  4.594  4.063  4.643  3.822  3.855  3.231  2.938  3.527  2.741  3.05  3.567  4.587  4.235  2.911  3.2  3.891  3.896  3.426  3.866  3.354  3.427  3.946  
 
 GAREM2  150946  2  26395959  26412532  2p23.3  0.495  0.058  0.326  1.297  0.129  0.276  0.128  0.849  0.375  0.428  0.646  0.617  0.556  1.59  0.367  0.344  0.157  0.198  0.355  0.763  1.201  0.42  0.408  0.101  0.064  1.535  0.406  0.574  0.399  1.38  1.581  0.277  0.071  0.354  1.453  1.301  1.104  0.516  0.408  2.303  0.891  1.085  2.485  1.097  0.975  0.731  0.628  1.559  0.35  2.326  0.138  1.89  0.64  1.141  1.19  0.994  0.642  0.765  0.468  0.557  
 
 HADHA  3030  2  26413503  26467594  2p23  4.698  3.518  3.373  4.14  4.348  2.765  4.891  4.26  4.235  4.192  4.37  3.994  4.325  4.734  4.144  3.588  4.907  2.181  5.904  5.946  4.45  4.292  4.41  3.408  4.562  4.485  4.733  4.23  4.906  5.287  5.066  4.842  4.419  3.664  3.63  4.369  4.212  4.435  4.066  4.952  4.181  4.069  5.208  3.812  3.503  3.941  4.813  3.643  3.772  5.71  4.369  3.496  3.401  3.005  4.678  3.701  4.438  3.811  3.777  4.617  
 
 HADHB  3032  2  26467615  26513333  2p23  3.825  4.532  2.575  2.935  4.049  1.994  4.049  3.056  3.618  3.618  3.757  2.956  3.497  4.286  2.835  3.058  3.985  4.635  5.24  4.642  3.39  2.372  2.924  2.137  3.484  4.847  3.635  2.96  4.644  3.855  5.063  4.399  3.723  3.149  2.829  4.03  2.975  2.801  3.755  4.481  3.504  3.092  4.376  3.14  2.567  3.163  3.576  2.84  2.737  5.361  3.626  2.523  2.712  2.306  4.099  3.081  3.256  2.827  2.925  3.753  
 
 ADGRF3  165082  2  26531040  26569685  2p23.3  0.092  0.085  0.22  0  0.076  0.064  0.067  0.09  0.152  0.147  0.104  0.166  0.196  0.093  0.177  0.264  0.147  0.095  0.621  0.131  0.113  0.688  0.397  0.16  0.051  0.118  0.238  0.105  0.121  0.138  0.073  0.053  0.125  0.332  0.163  0.084  0.11  0.086  0.093  0.054  0.369  0.413  0.125  0.239  0.227  0.378  0.054  0.078  0.053  0.14  0.069  0.473  0.119  0.217  0.172  0.146  0.174  0.038  0.054  0.067  
 
 EPT1  85465  2  26568953  26618759  2p23.3  1.824  1.699  0.759  0.908  1.358  0.623  1.791  1.337  0.834  1.159  1.402  1.547  2.664  2.252  1.929  1.718  2.054  1.086  2.923  1.064  1.579  1.454  1.309  1.183  1.708  1.682  0.96  1.697  1.606  1.246  2.004  1.832  1.554  0.944  1.219  1.709  1.546  1.538  1.191  2.245  2.578  1.528  1.91  1.905  1.562  1.706  0.907  1.157  1.63  2.627  1.795  1.004  1.337  1.252  1.371  1.225  1.522  1.109  1.212  1.926  
 
 DRC1  92749  2  26624783  26679579  2p23.3  0  0  0.063  0  0  0  0.092  0.044  0.047  0.034  0  0  0  0.065  0  0  0  0  0.079  0.078  0  0.148  0.186  0  0  0  0.068  0.023  0  0.055  0  0  0  0.155  0  0.055  0  0  0.06  0  0  0.171  0.046  0.046  0.078  0  0.044  0  0  0.119  0  0.16  0  0.061  0.05  0  0  0  0  0  
 
 OTOF  9381  2  26680070  26781566  2p23.1  0  0.023  0.141  0.443  0.027  0.044  0.059  0.027  0.123  0.033  0.038  0.059  0  0.06  0  0.057  0.055  0.104  0.139  0.094  0  0.252  0.309  0.079  0.033  0.033  0.111  0.028  0  0.144  0.067  0.03  0  0.234  0.051  0.066  0.024  0  0.117  0.147  0.035  0.635  0.054  0.297  0.715  0.061  0.081  0.076  0  0.25  0.039  0.739  0.058  0.122  0.076  0.037  0.035  0.132  0  0  
 
 C2orf70  339778  2  26785480  26802395  2p23.3  0  0  0  0  0  0  0.285  0  0.34  0  0.297  1.614  0.398  2.009  0.227  2.046  1.129  0.832  0  0  0  0.174  0.496  0  0  1.464  0.102  0.582  0  0.077  0  0  0  0  0.289  0.291  0.295  0  0  0  0  0.525  0  0  0.58  0.521  0.862  1.282  0  2.423  0  1.562  0.121  0.648  2.778  0  0  0  0.363  0  
 
 CIB4  130106  2  26804072  26864211  2p23.3  0.417  0  0  0  0.192  0  0.563  0  0  0.168  0.378  0  0.299  0.136  0.292  0.227  0  0  0.205  0  0  0.228  0  0  0  0  0.599  0.226  0.364  0.269  0  0  0  0  0.392  0.422  0.265  0  0.14  0.367  0.226  0.201  0.07  0  0  0  0.745  0  0.333  0.233  0.167  0.288  0.677  0.152  0.628  0.247  0.216  0  0  0  
 
 KCNK3  3777  2  26915580  26954066  2p23  0.067  0.08  0.192  1.077  0.141  0.19  0.176  0.321  0.212  0.297  0.239  0.14  0  0.121  0  0.104  0.105  0.234  0.075  0.146  0.065  0.464  0.572  0.103  0.064  0.103  0.492  0.058  0.065  0.248  0.217  0.117  0.051  0.347  0.54  2.055  0.139  0.12  0.195  0.391  0.054  1.185  0.104  0.076  0.169  0.305  0.186  0.07  0  0.285  0.067  1.392  1.823  2.053  3.408  1.848  2.817  0.106  1.858  0.993  
 
 SLC35F6  54978  2  26987141  27004099  2p23.3  3.566  1.638  2.648  3.196  3.068  2.892  4.196  3.711  3.101  3.579  3.83  2.8  3.075  3.415  3.874  2.631  2.447  0.886  3.041  1.536  3.506  2.203  1.778  2.163  3.185  3.511  3.681  3.405  3.882  4.067  4.098  4.079  4.215  2.757  3.732  4.759  3.516  3.665  2.89  4.416  4.297  2.895  3.34  3.502  2.369  3.601  2.798  2.989  5.095  3.568  2.908  3.434  4.08  4.581  4.235  4.06  3.933  3.508  3.399  3.108  
 
 CENPA  1058  2  27008881  27017455  2p23.3  3.566  5.101  2.483  2.538  2.465  2.756  3.342  2.563  1.433  3.162  3.626  2.183  2.255  3.557  2.737  2.703  3.018  5.331  5.256  3.474  2.476  3.313  2.231  2.093  2.972  2.28  3.278  2.201  2.47  2.663  3.425  3.275  3.282  2.743  2.979  3.052  3.123  3.408  3.425  3.523  3.683  3.075  3.646  2.48  2.14  2.796  4.084  2.77  2.864  4.499  3.183  2.285  3.205  2.272  3.733  2.641  3.105  3.791  3.108  2.663  
 
 DPYSL5  56896  2  27070968  27173219  2p23.3  1.381  0.032  0.143  0  0.059  0.062  0.139  0.052  1.064  1.525  1.375  0.069  0.213  1.461  0.031  0.067  0.072  0.152  0.038  0.061  0.046  0.21  0.461  0.037  0.027  0.041  0.204  0.038  0.898  0.465  0.095  0.902  0.041  0.307  1.473  0.029  0.059  0  0.111  1.74  0.045  0.607  2.899  1.784  0.069  0.052  0.298  0.039  1.302  0  0.033  0.732  0.064  0.103  2.044  0.103  0.038  0  0.058  0  
 
 MAPRE3  22924  2  27193238  27250087  2p23.3-p23.1  1.087  0.299  1.827  1.125  1.634  0.933  3.768  1.793  2.795  2.816  2.611  1.644  1.367  1.499  1.248  1.874  1.895  0.17  0.252  0.225  1.959  0.234  0.792  0.374  1.14  2.568  1.413  1.425  1.813  0.854  1.794  3.115  1.914  1.468  1.484  2.376  1.689  2.6  1.589  2.27  2.12  2.508  2.23  0.801  1.247  0.953  1.296  1.296  1.357  2.025  2.793  1.662  1.539  2.983  1.647  1.325  1.462  1.608  0.355  1.057  
 
 TMEM214  54867  2  27255773  27264565  2p23.3  3.77  1.725  3.823  3.105  3.922  2.947  5.29  5.465  3.485  4.514  4.3  4.447  3.847  3.896  4.118  3.475  3.88  0.63  4.094  3.606  3.825  3.095  3.225  3.82  3.672  4.216  4.477  3.978  4.609  4.726  4.471  4.801  4.674  2.784  3.489  4.621  3.892  3.976  4.061  4.404  4.479  3.517  4.229  3.787  2.61  3.796  4.492  3.458  3.376  4.703  4.275  3.141  3.788  3.953  4.072  4.277  4.187  3.558  3.296  3.965  
 
 AGBL5-AS1  100874031  2  27272550  27273132  -  0  0  0  0  0.331  0  0  0.275  0  0  0  0  0  0.203  0  0  0  0  0  0  0  0.131  0  0  0  0  0.18  0  0  0.148  0  0  0  0.509  0  0  0  0  0  0  0  0.319  0  0  0  0.293  0  0  0  0  0  0  0  0.263  0.367  0  0  0  0  0  
 
 AGBL5  60509  2  27274490  27293490  2p23.3  2.645  0.534  2.169  1.673  2.852  1.902  3.156  3.036  3.214  2.542  2.734  2.386  2.074  2.513  3.031  3.175  3.006  0.234  4.746  1.992  2.561  2.997  2.187  2.311  3.353  2.17  2.627  2.597  3.016  2.346  3.027  3.084  3.248  1.582  2.115  3.551  3.443  3.091  2.77  3.989  3.553  2.743  3.967  2.282  2.484  2.748  1.633  2.841  2.765  4.068  2.994  2.989  2.704  1.954  3.367  3.03  2.45  2.676  2.669  3.042  
 
 OST4  100128731  2  27293341  27294567  2p23.3  6.497  8.759  6.237  4.33  6.872  7.716  7.468  7.365  5.83  6.517  6.378  7.121  6.39  7.409  5.06  6.176  6.96  7.415  8.03  8.649  6.717  7.547  5.545  7.491  7.982  6.379  5.361  5.394  6.247  5.419  7.257  7.596  6.679  6.007  7.696  6.909  5.565  6.843  8.595  6.744  7.917  6.549  7.115  6.52  6.02  7.339  7.654  5.872  6.407  7.892  6.652  5.399  6.3  7.291  7.443  7.54  6.277  7.914  6.407  6.33  
 
 EMILIN1  11117  2  27301434  27309265  2p23.3-p23.2  0.084  0.047  3.724  0.092  0.096  0.12  0.162  5.173  0.307  1.68  0.115  0.114  0.13  0.159  0.173  0.09  0.101  0.21  0.251  0.234  0.074  0.364  0.611  0.107  0.083  1.62  1.739  0.259  0.097  0.261  0.166  4.431  0.278  0.366  0.115  0.351  0.088  0.219  0.117  0.263  0.249  0.567  0.235  0.092  0.07  0.13  0.048  0.123  0.092  0.348  0.096  0.884  0.148  0.317  0.136  0.148  0.121  0.032  0.068  0.064  
 
 KHK  3795  2  27309610  27323619  2p23.3  2.137  0.09  0.504  0.278  0.939  0.81  0.751  0.147  1.879  0.743  1.031  0.983  2.404  1.378  2.359  0.828  1.308  0.247  2.232  0.642  0.943  1.018  1.319  1.118  0.133  0.83  0.927  0.903  1.224  1.185  1.415  0.996  2.103  0.914  1.969  1.182  0.723  1.098  0.327  1.669  1.08  1.649  1.763  0.986  0.469  0.773  1.045  1.075  0.276  1.847  0.874  0.793  0.052  0.57  1.7  0.251  0.775  0.991  1.156  0.189  
 
 CGREF1  10669  2  27322220  27341995  2p23.3  2.942  0  2.214  2.58  2.285  0.842  3.214  0.481  1.707  2.929  0.436  0.48  2.866  0.933  2.834  2.448  0.305  1.118  0.434  2.653  0  0.8  1.085  0.282  0.129  1.159  2.775  0.237  1.397  2.082  1.857  2.879  2.014  1.624  1.562  1.089  0.542  2.468  0.804  2.377  0.343  3.083  1.487  0.882  1.09  0.919  1.203  2.089  0  3.377  2.28  1.873  0.138  1.232  0.719  0.308  0.502  1.835  1.675  0.291  
 
 ABHD1  84696  2  27346656  27353680  2p23.3  0.769  0.051  0.197  0  1.476  0.16  0.242  0.126  0.377  0.284  0.145  0.068  0.139  0.156  0.404  0.132  0.112  0.079  0.061  0.202  0.146  0.235  0.803  0.345  0  0.125  0.19  0.116  0.095  0.805  0.197  0.095  0.104  0  0.448  0.177  0.181  0.219  0.091  0.286  0.144  0.455  0.785  0  0.236  0.181  0.207  0  0  0.155  0.166  0.601  0.113  0.238  0.724  0.17  0.146  0  0  0  
 
 PREB  10113  2  27353624  27357542  2p23.3  3.611  2.424  3.166  2.801  3.319  2.481  5.076  3.341  3.702  3.571  4.168  3.454  4.015  3.863  3.775  3.24  3.661  1.442  5.171  3.771  3.551  3.917  5.303  4.869  3.208  3.593  3.889  3.45  3.974  4.117  4.391  5.061  4.373  2.685  3.169  4.189  4.056  3.756  3.098  4.591  3.847  3.854  4.893  3.216  2.445  3.648  3.952  3.315  3.405  5.143  3.866  3.072  3.04  2.944  3.764  3.197  3.103  3.33  2.818  3.238  
 
 PRR30  339779  2  27359714  27362332  2p23.3  0  0  0.129  0  0.05  0  0  0.041  0.18  0.041  0  0  0  0.065  0  0.043  0  0.13  0  0.06  0  0.193  0.524  0  0  0  0.13  0  0  0.096  0  0  0  0.29  0  0  0  0  0.158  0  0  0.09  0.083  0  0  0.058  0.048  0  0  0  0  0.43  0  0.096  0.109  0  0  0  0  0  
 
 TCF23  150921  2  27371944  27375819  2p23.3  0  0.116  0  0  0  0  0  0  0.164  0  0  0  0  0  0  0  0  0  0  0.198  0  0.277  0.462  0.196  0  0  0.142  0  0  0.101  0  0  0  0.236  0  0  0  0  0.08  0  0  0.466  0.113  0  0  0  0.074  0  0  0  0  1  0  0.21  0  0  0  0  0  0  
 
 SLC5A6  8884  2  27422454  27435175  2p23  3.529  2.031  1.624  1.653  3.801  1.95  3.787  2.453  2.34  2.302  2.93  2.321  3.461  3.003  3.161  2.753  2.343  1.033  4.248  3.358  3.861  3.297  2.206  2.931  3.662  3.167  4.038  3.394  4.84  3.845  3.953  4.788  4.543  2.337  2.938  3.584  3.507  2.799  2.611  4.639  3.908  2.844  5.028  3.286  1.57  2.481  3.708  2.434  2.037  3.925  2.314  2.22  2.676  2.032  2.941  2.114  2.291  2.354  2.887  2.523  
 
 ATRAID  51374  2  27434898  27440046  2p23.3  5.61  6.1  4.075  4.577  5.519  3.852  6.253  5.797  5.04  5.626  4.874  4.783  4.751  6.194  4.274  4.811  5.761  5.987  6.686  5.639  4.131  5.036  5.009  4.935  5.14  4.969  4.42  4.617  4.89  4.281  5.688  6.078  4.857  3.811  4.945  5.965  4.688  5.299  5.748  6.319  6.324  4.776  5.6  4.651  3.279  4.54  6.147  3.423  4.569  5.804  5.047  4.233  4.696  3.816  6.119  4.702  4.818  5.318  4.139  5.268  
 
 CAD  790  2  27440257  27466660  2p22-p21  3.362  0.628  2.369  1.82  2.029  2.395  3.513  3.579  1.61  3.059  3.255  1.979  3.781  3.458  4.545  2.869  2.721  0.174  4.577  3.136  3.984  3.25  1.992  3.207  3.029  3.058  3.297  3.444  3.795  3.649  3.887  4.359  4.255  1.56  3.026  3.65  4.194  3.66  2.509  5.136  4.142  2.816  4.233  3.817  2.301  2.861  2.751  3.712  3.425  4.548  3.531  2.916  3.521  2.167  3.86  3.174  3.577  3.254  3.597  3.616  
 
 SLC30A3  7781  2  27477439  27485960  2p23.3  1.026  0.061  1.163  0  0.163  0.325  1.675  0.324  1.637  0.753  0.586  0.091  0.711  2.369  0.458  0  0  0.116  0  0.087  0.452  0.373  1.072  0.882  0  0  1.175  0.072  0.313  0.995  0.267  1.511  0  0.258  1.036  1.934  0.1  0.085  1.258  1.073  1.849  1.307  1.794  0.157  0.217  0.125  0.49  0.775  0  2.213  0.057  1.144  0.24  0.501  0.195  0.07  0  1.703  0.093  0  
 
 DNAJC5G  285126  2  27498288  27504298  2p23.3  0  0  0.118  0  0.068  0  0  0  0.155  0  0  0  0  0.499  0.114  0.066  0  0  0.057  0  0  0.097  0.56  0.082  0  0  0.119  0  0  0.075  0  0  0  0.35  0  0  0  0  0.045  0  0  0.082  0.059  0  0  0  0.04  0.411  0  1.095  0  0.469  0  0.087  0.051  0  0  0  0  0  
 
 TRIM54  57159  2  27505296  27530307  2p23.3  0.682  0.053  0.384  0.17  0.223  0  0.598  0.138  0.475  0.326  0.248  0.209  0.567  0.455  0.386  0.343  0.242  0.198  0.31  0.374  0.108  0.466  0.884  0.474  0.258  0.176  0.563  0.205  0.568  0.577  0.158  0.337  0.329  0.468  0.297  0.155  0.386  0.209  0.253  0.421  0.357  1.103  0.856  0.131  0  0.109  0.702  0.376  0  2.053  0.305  0.744  0.221  0.327  0.409  0.131  0.358  0  0.165  0.085  
 
 UCN  7349  2  27530264  27531130  2p23-p21  0.431  0.408  0.521  1.118  0.844  0.622  1.433  0.368  1.848  1.308  1.294  0.203  0  1.18  0.28  0.615  1.134  0  2.595  0.613  0.643  1.561  1.493  0  0.308  0.184  1.197  0.804  0.475  0.457  0.216  0.444  1.394  0.974  0.619  0.876  0.701  0.977  1.614  0.803  0.957  2.043  0.908  0  0  0.422  0.096  0.65  0  1.146  0.258  1.632  0.56  0.677  1.814  0  0  0.337  0.52  0.305  
 
 MPV17  4358  2  27532359  27545969  2p23.3  4.745  3.642  4.07  3.104  4.681  3.71  5.739  4.88  3.536  4.655  4.536  4.188  3.674  4.57  3.154  4.084  4.043  4.305  5.408  5.342  3.905  4.628  3.499  4.258  4.865  4.921  4.959  4.101  4.691  4.776  5.424  4.782  5.093  3.959  4.052  5.211  4.606  5.064  5.322  5.007  5.632  3.679  6.021  3.267  3.168  3.883  6.045  3.254  2.99  4.85  4.438  3.52  4.183  3.747  5.759  4.867  4.218  5.397  3.441  4.638  
 
 GTF3C2  2976  2  27548715  27579901  2p23.3  3.377  2.569  1.96  1.992  2.859  2.157  3.646  3.126  2.447  2.692  3.41  2.718  3.29  3.289  3.484  3.226  2.853  3.865  4.219  3.643  3.085  2.848  2.234  2.818  3.347  3.115  3.037  2.877  3.367  3.479  3.838  3.832  3.801  1.993  2.902  3.883  3.506  3.368  2.533  4.729  4.234  2.54  4.844  3.53  2.74  3.095  2.844  2.483  2.996  3.693  3.342  2.414  3.058  2.466  3.469  3.016  3.459  3.192  2.969  3.241  
 
 GTF3C2-AS1  100505624  2  27558408  27560670  2p23.3  0.565  0  0.327  0.361  0.239  0.779  0.388  0.509  0.621  0.272  0.453  0.358  0.78  0.529  0.958  0.764  0.428  0  0.316  0.489  0  0.495  0  0.654  0.441  0.398  0.449  0.57  1.009  0.204  0.65  0.64  0.811  0.57  0.758  0.589  0.772  0.451  0.399  1.158  0.733  0.614  0.074  0.508  0.275  0.339  0  0.4  0.286  0.236  0.469  0.312  0.619  0.298  0.707  0.429  0.621  0.478  0.445  0.487  
 
 EIF2B4  8890  2  27587218  27593324  2p23.3  3.333  3.981  2.654  2.22  3.712  2.403  4.345  3.232  2.765  3.265  3.764  2.48  3.113  3.96  2.928  2.939  2.856  2.318  4.799  4.225  3.293  3.646  2.44  2.824  3.328  2.766  3.424  2.799  3.439  2.835  4.298  4.469  3.588  1.857  3.194  3.744  3.747  3.015  3.927  4.07  3.641  2.434  5.04  3.181  2.271  2.749  4.173  2.476  2.989  4.679  3.166  2.418  2.725  2.08  3.642  2.776  3.147  3.504  2.525  3.08  
 
 SNX17  9784  2  27593362  27600400  2p23-p22  3.988  2.473  3.814  3.598  4.292  3.369  5.238  4.346  4.591  4.071  4.341  3.293  3.905  4.606  3.572  3.428  3.723  0.467  6.047  4.595  3.03  4.284  3.961  3.201  4.367  4.168  4.253  3.807  4.412  4.418  5.03  5.299  4.271  3.394  3.913  4.655  4.172  4.745  4.669  5.118  4.08  4.256  4.716  3.584  1.962  3.433  4.322  3.164  3.859  5.579  4.166  3.605  3.61  3.21  4.835  3.74  3.968  4.15  3.32  4.27  
 
 ZNF513  130557  2  27600097  27603611  2p23.3  0.674  0  0.405  1.803  1.179  1.429  1.891  1.963  2.305  1.961  1.793  0.853  1.76  1.508  1.903  1.677  0.441  0.189  2.038  0.268  1.128  0.839  1.054  0.798  1.737  1.967  2.05  1.75  2.028  1.586  2.453  2.418  2.052  0.873  1.013  1.529  1.868  1.546  0.599  1.233  0.781  1.604  2.453  1.33  0.986  0.786  0.121  0.672  1.155  1.123  1.441  1.765  0.85  1.058  2.495  1.372  1.455  0.865  0.966  1.025  
 
 PPM1G  5496  2  27604065  27632550  2p23.3  5.194  4.466  3.788  4.415  4.126  3.875  5.6  5.018  4.354  4.164  5.106  4.075  5.368  5.259  5.231  5.189  5.053  2.637  6.227  4.28  4.755  4.563  4.447  4.274  5.203  4.051  4.381  5.341  4.525  4.938  4.754  5.361  5.88  4.37  4.712  4.963  5.659  5.044  3.834  5.863  5.031  4.865  5.943  4.608  4.484  4.693  3.83  4.902  4.594  6.294  4.753  4.514  4.735  4.034  5.115  4.697  4.542  5.07  4.619  5.036  
 
 FTH1P3  2498  2  27615489  27616443  2p23.3  4.46  2.939  4.753  6.789  3.857  2.543  4.061  2.066  6.815  4.488  3.242  3.731  4.71  4.397  2.983  4.963  4.749  2.685  4.186  3.122  2.042  3.775  5.027  2.871  3.249  2.999  3.427  2.045  3.357  4.455  4.549  2.857  2.247  4.006  4.59  2.886  3.846  2.221  7.036  2.693  3.842  5.503  4.769  2.881  2.53  2.936  4.42  2.28  4.382  3.993  5.041  2.744  3.602  3.939  4.502  3.96  2.752  2.552  4.395  3.214  
 
 NRBP1  29959  2  27651472  27665124  2p23  3.893  1.561  3.893  2.869  3.208  3.398  5.153  5.18  3.97  4.201  4.751  2.46  3.696  3.614  3.912  3.243  2.634  0.178  3.973  1.5  2.301  2.398  2.144  2.144  3.987  3.654  3.831  4.031  3.706  3.081  4.038  4.81  4.175  2.245  3.691  4.633  4.381  4.359  2.834  5.775  4.175  3.573  4.516  3.919  3.367  4.033  1.578  3.144  4.266  4.508  4.083  2.936  3.98  3.58  4.181  4.365  4.417  3.76  3.591  4.144  
 
 KRTCAP3  200634  2  27665232  27669348  2p23.3  3.687  0.168  0.308  0.328  0.704  0  0.573  0.153  0.628  0.515  0.386  4.669  3.824  4.466  2.557  4.092  4.545  0.389  0.853  0.176  0  0.337  0.705  0.747  0.225  0.6  0.342  0.245  0  0.234  0  0  0.423  0.26  0.137  0.503  0.198  0.204  0.429  0  0.776  0.291  0.086  0.251  0.317  3.278  0.977  0.29  0.337  0.242  0.231  1.22  0.229  0.254  1.041  0  0.489  0.248  0.321  0  
 
 IFT172  26160  2  27667239  27712678  2p23.3  0.906  0.427  0.436  0.362  0.885  0.645  1.204  1.013  0.584  1.208  1.214  0.792  0.642  0.657  0.947  0.63  0.682  0.16  1.458  0.889  0.822  0.851  0.285  0.411  0.146  1.107  1.488  1.11  1.487  1.323  1.289  0.718  1.335  0.362  0.605  1.012  1.172  0.973  0.523  1.205  1.011  0.69  0.939  0.745  1.074  1.088  0.68  0.776  1.237  1.633  0.855  0.486  0.692  0.547  1.083  0.903  1.274  0.6  0.288  1.079  
 
 FNDC4  64838  2  27714749  27718126  2p23.3  0  0.377  2.177  0.637  0.175  1.035  2.017  0.462  2.896  1.061  2.002  0  0  1.105  0  0.126  0.067  0.145  0  0  0.449  0.316  0.409  0  0  0.279  0.421  0  0.402  0.397  0.161  0.127  0  0.241  0.719  0.956  1.153  2.487  0.683  1.27  0.176  0.888  1.579  0.158  0.127  0.919  1.673  0.499  1.356  0.104  0.25  0.805  0.764  0.261  1.89  0.814  1.266  1.333  0  1.863  
 
 GCKR  2646  2  27719705  27746550  2p23  0  0  0.584  0  0  0  0.127  0  0.075  0  0.066  0  0  0.025  0  0.034  0  0  0  0.072  0  0.046  0.141  0  0  0  0.082  0  0  0.07  0  0  0  0.158  0.22  0  0.133  0  0.415  0  0  0.039  0.047  0  0  0.072  0.025  0  0.102  0.043  0.07  0.176  0.262  0  0.137  0  0  0  0  0.071  
 
 C2orf16  84226  2  27799388  27805589  2p23.3  0.172  0.1  0.162  0  0.134  0.14  0.058  0.066  0.147  0.13  0.177  0.045  0.162  0.137  0  0.173  0  0.212  0.178  0.032  0.116  0.088  0.257  0.049  0.083  0.11  0.152  0.084  0.029  0.12  0.128  0.06  0.101  0.356  0.071  0.443  0.085  0.048  0.076  0.222  0.492  0.18  0.372  0.175  0.095  0.062  0.067  0.101  0.153  0.093  0.019  0.257  0.071  0.117  0.082  0.076  0.15  0.17  0.046  0.14  
 
 ZNF512  84450  2  27805835  27846082  2p23  1.462  2.962  1.121  1.241  1.498  1.26  2.26  1.979  0.109  1.798  2.46  1.371  1.182  2.969  0  1.622  0.071  3.07  3.937  3.115  2.394  1.285  1.31  1.206  2.072  1.364  1.361  1.411  1.563  0.332  1.453  1.513  2.586  2.037  1.688  2.093  1.724  1.951  2.196  1.953  1.734  1.117  2.849  1.959  1.701  1.687  1.943  1.452  1.595  2.265  0  1.032  1.223  1.63  2.58  1.903  2.294  1.308  1.531  1.797  
 
 CCDC121  79635  2  27848505  27851898  2p23.3  0.628  0.229  0.179  0.17  0.683  0  0.107  0.213  0.522  0.321  0.839  0.254  0.341  0.536  0.395  0.25  0.28  0.192  0.593  0.154  0.313  0.214  0.312  0.062  0.25  0.366  0.306  0.292  0.283  0.297  0.49  0.39  0.43  0.412  0.344  0.929  0.117  0.075  0.328  1.13  0.718  0.34  0.917  0.732  0.092  0.558  0.048  0.095  0.485  0.145  0.284  0.299  0.157  0.153  0.466  0.076  0.278  0.313  0.103  0.277  
 
 GPN1  11321  2  27851514  27873713  2p23.3  3.8  5.375  2.327  2.103  3.032  2.085  4.19  3.526  2.85  2.743  4.904  2.734  3.172  4.571  2.951  3.385  3.275  4.864  4.779  4.409  2.914  3.715  2.405  2.195  3.75  2.874  2.762  2.797  2.959  2.531  3.546  3.44  3.556  2.36  2.774  3.539  3.526  3.314  4.144  4.041  4.094  2.235  4.588  2.86  2.74  3.249  3.684  2.6  2.667  4.383  3.44  2.166  3.287  2.961  4.572  3.217  3.508  3.234  3.364  3.741  
 
 SUPT7L  9913  2  27873675  27886707  2p23.3  2.45  3.1  1.732  2.026  2.167  1.465  2.795  2.547  2.407  2.341  2.558  1.301  2.237  2.897  2.202  2.126  2.122  3.595  3.445  2.242  1.962  2.437  2.251  1.696  2.025  2.573  2.28  2.089  2.447  2.438  2.869  2.754  2.62  1.715  1.773  2.613  2.235  2.213  2.693  2.784  2.599  1.988  3.16  1.8  1.409  1.87  2.186  1.556  1.524  3.229  2.033  1.536  1.744  2.011  2.959  1.937  2.438  2.239  1.791  1.883  
 
 SLC4A1AP  22950  2  27886337  27917847  2p23.3  1.79  3.292  1.38  1.486  2.228  1.52  1.998  2.175  2.026  1.893  2.555  1.46  1.854  2.413  2.006  1.761  2.092  3.023  2.972  1.853  2.22  2.133  1.517  1.419  2.304  2.215  1.215  1.768  1.86  1.328  2.55  2.472  2.038  1.529  1.949  3.048  1.849  1.827  2.713  2.61  2.949  1.54  2.124  2.147  1.75  1.933  1.06  1.77  1.96  2.978  2.24  1.715  1.653  2.259  2.597  1.754  2.259  2.157  1.696  2.358  
 
 MRPL33  9553  2  27994583  28002608  2p21  3.932  8.663  4.597  4.448  4.877  6.202  4.853  4.997  5.473  5.226  6.05  4.788  3.33  5.276  2.768  4.77  5.255  8.63  6.321  6.384  4.863  5.381  4.222  4.14  5.252  4.345  2.452  3.72  3.798  2.114  4.909  4.638  4.016  4.733  6.758  5.467  3.718  4.97  7.216  4.261  5.227  6.119  5.106  6.194  4.527  5.837  4.923  4.096  5.746  7.27  4.407  3.936  4.764  6.04  6.931  6.134  4.544  6.232  4.311  5.157  
 
 RBKS  64080  2  28004230  28113263  2p23.3  1.223  1.348  0.442  0.543  1.485  0.237  1.43  1.079  0.581  0.905  0.841  0.54  0.747  1.182  0.44  0.27  1.045  1.127  1.626  0.551  0  0.854  0.838  0.168  0.535  0.351  0.268  0.193  0.277  0.099  0.67  0.503  0.179  0.242  1.532  1.952  0.513  0.757  0.735  1.488  1.009  0.447  0.03  2.157  0.968  0.903  0.313  0.242  0.921  0.355  0.821  1.039  1.583  1.283  3.294  1.631  1.515  0.265  1.416  1.611  
 
 BRE-AS1  100302650  2  28112322  28113981  2p23  0.123  0.08  0.336  0.315  0.25  0.187  0.146  0.437  0.284  0.41  0.164  0.235  0  0.185  0  0.193  0.276  0  0.407  0.252  0.243  0.423  0.469  0  0.07  0.088  0.1  0.434  0.095  0.406  0.685  0.226  0.158  0.312  0.632  0.505  0.172  0.304  0.254  0.211  0.244  0.642  0.045  0.421  0.246  0.407  0.198  0.252  0.557  0.275  0.144  0.58  0.502  0.6  1.527  0.35  0.582  0.208  0.358  0.261  
 
 BRE  9577  2  28113481  28561767  2p23.2  2.307  2.933  1.965  2.071  3.041  1.994  3.131  3.244  2.443  4.168  2.808  1.942  2.225  2.801  1.604  2.11  3.379  2.139  4.687  2.541  2.24  2.899  2.301  1.774  2.613  2.886  2.037  2.619  2.633  1.504  3.464  2.667  2.548  1.836  2.824  3.956  2.765  3.381  2.603  3.42  3.689  2.189  1.894  3.7  0.822  2.622  2.684  1.458  3.156  3.348  2.263  2.441  2.369  2.495  3.972  2.683  2.937  2.523  2.562  3.881  
 
 LOC100505736  100505736  2  28371423  28429828  2p23.2  0.286  0.095  0.21  0  0.084  0  0.249  0.128  0.078  0.326  0.083  0  0.18  0.236  0.318  0.08  0.146  0.142  0.062  0.193  0  0.208  0.234  0.289  0  0.213  0.144  0.219  0.244  0.048  0.195  0  0.147  0.362  0.406  0.294  0.236  0.476  0.094  0  0.35  0.337  0.093  0.17  0  0.153  0.025  0.092  0.237  0  0.142  0.124  0.205  0.071  0.3  0.138  0.478  0.16  0.097  0.068  
 
 LOC100505716  100505716  2  28530557  28533326  2p23  0.39  0.275  0.166  0  0.155  0.156  0.183  0.405  0.179  0.533  0.197  0  0.321  0.61  0.32  0.161  0.617  0.328  0.099  0.215  0.127  0.44  0.445  0.145  0.131  0.254  0.243  0.289  0.369  0.179  0.385  0.147  0.327  0.22  0.299  0.394  0.294  0.542  0.139  0.224  0.602  0.647  0.258  0.682  0  0.3  0.153  0.184  0.619  0.071  0.139  0.253  0.259  0.111  0.644  0.236  0.585  0.191  0.245  0.154  
 
 FLJ31356  403150  2  28607275  28617539  2p23.3  0.064  0.046  0.219  0  0.024  0  0.301  0.114  0.069  0.202  0.069  0  0  0  0  0.063  0.075  0.116  0  0  0  0.081  0.323  0  0  0  0.045  0  0  0.047  0  0  0  0.254  0  0.091  0.124  0.072  0.049  0  0.055  0.056  0.021  0  0.079  0.258  0.024  0  0  0  0.078  0.135  0.12  0.107  0.065  0.211  0.139  0  0.074  0.128  
 
 FOSL2  2355  2  28615778  28637516  2p23.3  1.715  0.79  3.409  3.435  1.164  3.059  3.269  5.601  4.115  4.646  3.992  1.963  2.682  1.307  2.048  1.72  3.332  0.103  1.175  1.069  0.264  0.768  0.689  0.637  3.508  2.977  1.34  2.116  1.887  1.429  1.858  1.714  1.84  0.965  2.865  4.449  3.766  4.549  2.874  3.171  2.897  2.418  2.74  4.21  2.699  3.209  1.871  3.207  3.707  3.226  2.603  2.984  4.198  3.911  4.386  3.381  5.013  2.976  4.45  3.855  
 
 PLB1  151056  2  28718937  28866653  2p23.2  0  0.029  0.054  0  0.021  0  0.071  0.066  0.167  0.047  0.157  0.089  0.046  0.037  0  0.022  0  0.051  0.019  0.928  0  0.039  0.133  0  0.038  0.987  0.065  0.325  0.044  0.061  0.976  0.094  0.026  0.113  0.03  0  0.073  0.061  0.05  0  0.099  0.168  0.042  0.071  0.083  0.041  0.021  0  0.046  0.055  0.078  0.143  0.022  0.067  0.065  0.069  0.062  0  0.053  0.082  
 
 PPP1CB  5500  2  28974613  29025806  2p23  3.265  4.241  2.748  2.991  2.923  2.514  3.066  3.537  5.159  3.226  5.027  3.284  3.496  4.783  3.637  3.231  3.948  5.059  4.091  3.658  2.646  3.361  1.92  2.01  3.366  3.522  2.062  2.869  2.894  1.543  3.566  3.062  2.836  2.129  2.832  3.557  3.285  3.134  3.813  3.922  3.942  3.513  3.151  3.524  3.743  3.714  2.842  2.158  3.891  3.832  3.803  2.386  3.332  2.865  3.564  3.19  4.172  3.522  3.365  3.85  
 
 SPDYA  245711  2  29033699  29073475  2p23.2  0.501  0.439  0.455  0.351  0.332  0.098  0.327  0.176  0.61  0.24  0.636  0.259  0.313  0.413  0.374  0.401  0.186  0.578  0.634  0.844  0  0.916  0.704  0  0.306  0.216  0.253  0.173  0.245  0.542  0.404  0.338  0.181  0.566  0.371  0.252  0.206  0.257  1.3  0.242  0.357  0.359  0.873  0.224  0  0.346  0.913  0.169  0  0.534  0.285  0.16  0.262  0.283  0.596  0  0.332  0.389  0.259  0  
 
 TRMT61B  55006  2  29072687  29093175  2p23.2  1.375  3.252  1.109  1.259  1.422  1.336  1.848  1.546  1.85  1.415  1.544  1.43  1.925  1.985  1.165  1.618  1.555  4.59  3.007  1.044  1.587  1.609  1.554  0.975  1.664  1.769  0.952  1.33  1.427  0.998  2.032  1.803  1.764  1.369  1.363  1.604  1.491  1.363  1.724  2.301  1.824  1.442  1.914  1.035  1.086  1.201  0.373  1.148  1.296  3.171  2.045  1.093  1.344  1.371  2.109  1.413  1.182  1.141  1.283  2.055  
 
 WDR43  23160  2  29117508  29171086  2p23.2  3.096  4.456  1.815  1.879  2.287  2.666  3.226  2.989  1.807  1.833  3.126  2.632  3.663  4.404  3.047  2.811  3.054  4.758  4.041  3.316  3.564  3.298  1.961  2.878  3.792  2.85  2.602  2.747  2.919  3.143  3.582  3.962  3.166  1.839  3.381  3.057  3.409  2.825  3.184  4.138  3.593  1.978  3.307  3.762  2.79  2.736  2.592  2.484  2.679  3.686  3.143  2.539  3.022  2.392  3.599  3.093  2.964  3.069  2.954  3.587  
 
 FAM179A  165186  2  29204163  29275096  2p23.2  0  0.386  0.035  0  0.026  0.048  0.109  0.036  0.136  0.032  0.047  0.076  0  0.101  0  0.03  0.038  0.098  0.672  0.05  0  0.423  0.268  0  0  0.059  0.094  0.036  0.167  0.128  0  0  0  0.227  0  0.194  0.16  0  0.067  0  0.071  0.291  0.048  0.912  0  0.089  0.057  0.104  0  0.258  0.034  0.437  0.026  0.066  0.07  0  0.062  0  0  0  
 
 C2orf71  388939  2  29284555  29297127  2p23.2  0  0  0.135  0  0.021  0  0.025  0.038  0.186  0.039  0.056  0.022  0.03  0.03  0  0.019  0.038  0.085  0  0.031  0  0.075  0.451  0  0  0.074  0.069  0.026  0.019  0.135  0.019  0  0  0.265  0.045  0  0  0  0.038  0  0  0.274  0.034  0  0.029  0.022  0.042  0  0  0  0.023  0.349  0.016  0.072  0.033  0.02  0.016  0  0  0  
 
 CLIP4  79745  2  29320541  29406679  2p23.2  0  3.81  1.237  0.725  0  0.683  1.928  1.276  1.894  1.702  2.239  0.099  0.517  1.764  0  0.319  0.069  0.279  1.134  0  0  1.096  0.65  0.189  1.054  2.425  0.782  2.279  2.284  1.127  1.548  1.458  0.353  0.535  1.006  1.991  2.117  1.884  3.175  0.783  2.68  1.686  0.799  1.723  0.447  1.502  1.87  0.877  2.33  2.404  1.228  1.457  1.032  1.183  2.24  1.41  1.574  1.835  0.21  2.365  
 
 ALK  238  2  29415639  30144477  2p23  0  0.035  0.084  0  0.048  0.051  0.05  0.091  0.2  0.731  0.082  0.072  0.028  0.07  0.026  0.038  0.043  0.044  0.016  0.084  0.049  0.176  0.337  0.025  0.031  0.216  0.123  0.247  0.283  0.323  0.585  0.199  0.033  0.249  0.068  0.069  0.072  0.069  0.175  0.083  0.257  0.243  0.06  0.482  0.791  0.165  0.075  0.031  0.087  0.031  0.208  0.349  0.042  0.081  0.083  0.046  0.572  0.034  0  0  
 
 YPEL5  51646  2  30369749  30383399  2p23.1  2.061  4.471  3.042  2.796  2.639  1.468  2.498  3.261  3.773  2.602  2.214  1.308  1.273  2.533  1.094  1.481  1.715  3.191  2.615  1.613  0.847  1.796  2.514  0.677  2.999  2.063  2.028  1.696  1.859  1.825  1.784  2.401  1.826  2.418  1.626  2.717  1.874  2.394  4.293  2.51  2.866  1.394  3.544  1.835  1.421  2.394  2.117  1.059  2.012  3.422  2.749  1.746  1.549  2.411  2.504  2.215  2.674  2.706  1.721  2.995  
 
 LBH  81606  2  30454396  30482899  2p23.1  0  0.04  2.997  0.204  0.034  2.303  2.192  3.18  1.448  3.106  1.808  0.222  0  0.045  0  0.109  0  0.152  3.045  0.162  0.905  2.45  0.965  3.537  0  1.956  0.446  0.213  0.738  1.401  1.012  2.504  0  0.682  3.57  3.075  0.128  0.201  0.61  0.435  3.194  1.768  1.59  3.654  3.989  4.206  1.064  0.448  1.95  1.234  0.076  0.605  0.449  4.344  0.113  0.147  1.745  1.815  0  3.116  
 
 LOC285043  285043  2  30569511  30573024  2p23.3  0  0  0  0  0  0  0  0  0  0  0  0  0  0  0  0  0  0  0  0  0  0  0  0  0  0  0.073  0  0  0  0  0  0  0.437  0  0  0  0  0  0  0  0  0  0  0  0  0  0  0  0  0  0.339  0  0  0  0  0  0  0  0  
 
 LCLAT1  253558  2  30670091  30867091  2p23.1  2.358  3.414  1.485  1.233  1.8  1.576  2.679  1.815  1.827  1.768  2.494  2.505  2.37  3.095  2.145  2.164  2.227  3.126  2.517  2.02  1.876  1.705  1.445  2.14  2.382  2.362  1.522  2  2.447  1.59  2.128  2.674  2.192  1.282  2.45  3.133  1.694  1.934  1.891  2.676  3.202  1.459  2.574  2.498  2.055  1.911  2.603  1.88  2.323  2.446  2.554  1.691  2.005  2.025  1.899  1.621  2.016  2.277  1.693  1.985  
 
 CAPN13  92291  2  30945637  31030311  2p22-p21  0  0  0.047  0  0.589  0  0.037  0.026  0  0.044  0  0.058  0  0.055  0  0.099  0.06  0.164  0  0  0  0.066  0.213  0.066  0  0  0.161  0.039  0.038  0.116  0  0  0  0.126  0  0  0  0  0.047  0  0.19  0.108  0.093  0.199  0.381  2.169  0.238  0  0.098  0.079  0  0.257  0.026  0.135  0.037  0.055  0  0  0.114  0  
 
 GALNT14  79623  2  31133330  31361592  2p23.1  2.314  0  0.126  0.149  0.136  0  0  0.077  0.105  0.102  0.062  0.071  0  1.729  0  2.845  0  0.265  0.186  2.411  0  0.232  1.665  2.488  0  0.041  0.172  0.047  0.119  1.693  2.271  0.438  0  0.241  2.56  0.436  0.187  0  1.198  2.934  3.682  2.365  2.491  0.085  2.143  1.033  2.784  2.499  0.486  3.851  0.037  1.923  2.855  2.144  5.186  4.304  3.709  0.828  3.436  2.912  
 
 CAPN14  440854  2  31395921  31440411  2p23.1-p21  0  0  0.083  0  0  0.071  0  0  0.12  0.083  0.041  0  0  0.018  0  0  0  0  0.082  0  0  0.124  0.223  0.11  0  0.102  0.066  0.366  0  0.07  0.27  0.037  0  0.227  0.063  0.028  0  0  0.025  0  0.42  0.032  0.033  0  0.059  0.109  0.193  0  0  0  0.019  0.217  0  0.044  0.026  0  0  0  0  0  
 
 EHD3  30845  2  31456879  31491260  2p21  1.28  0.069  1.242  0.793  0.471  1.517  2.444  1.946  1.377  1.748  1.897  0.09  0  0.053  0.105  0.132  0.044  0.147  0.249  0.157  0.048  0.611  0.586  0.683  0.071  2.069  0.139  2.5  0.133  0.415  1.016  0.257  0.142  0.274  1.209  1.689  2.121  0.767  0.454  2.527  1.22  0.661  2.556  1.287  2.003  0.917  1.338  0.464  0.417  0.869  0.912  1.346  0.35  1.097  0.808  1.258  1.534  1.737  0.043  0.871  
 
 XDH  7498  2  31557187  31637611  2p23.1  0.041  0.176  0.121  0.101  0.031  0.109  0.088  0.142  0.406  0.049  0.146  0.045  0  0.045  0.191  0.55  0.034  0.077  0.023  0.034  0  0.067  0.352  0.475  0.453  0.021  0.19  0.119  0.017  0.153  0.037  0.052  0  0.155  0.125  0.79  0.138  0.078  1.006  0.153  1.299  0.046  0.096  0.757  0.78  0.314  0.053  0.464  0.379  2.16  0.122  0.283  0.023  0.076  0.056  0.166  0.105  0.225  0.173  0.042  
 
 SRD5A2  6716  2  31749655  31806040  2p23  0  0  0.081  0  0.049  0  0  0  0.048  0.04  0  0  0  0.021  0  0  0  0.055  0  0.069  0  0.106  0.325  0  0  0.041  0.068  0.199  0  0.074  0  0  0  0.196  0  0  0  0  0.031  0  0  0.164  0.025  0  0  0.061  0.089  0  0  0  0  0.189  0  0.062  0  0  0  0  0  0  
 
 MEMO1  51072  2  32092878  32236121  2p22-p21  2.795  3.949  2.269  1.812  2.722  2.456  3.132  3.104  2.556  2.75  3.295  2.595  3.042  3.936  2.549  2.94  3.015  4.3  5.085  3.615  2.974  3.462  2.656  2.539  3.538  3.105  2.792  2.589  2.765  2.718  3.766  4.116  3.083  1.836  2.85  3.168  3.044  2.882  3.767  3.897  3.179  2.33  4.167  2.235  2.175  2.942  3.597  2.898  3.126  4.804  3.307  1.949  2.559  1.702  3.157  2.595  2.963  3.079  3.247  3.347  
 
 DPY30  84661  2  32248971  32264844  2p22.3  4.133  6.077  2.451  2.623  4.627  3.261  4.132  3.416  2.812  3.312  3.776  3.599  3.731  4.703  2.913  4.319  4.554  7.404  5.396  4.529  3.584  4.466  3.08  3.288  3.846  3.192  2.267  2.576  3.274  1.202  4.061  3.771  2.982  2.697  3.936  3.505  3.191  3.43  5.29  3.92  4.719  3.241  5.716  3.456  3.742  3.915  3.832  3.622  3.261  6.311  3.957  2.525  3.199  2.502  4.651  4.132  2.874  4.54  2.923  3.544  
 
 SPAST  6683  2  32288679  32382706  2p24-p21  0.857  1.172  0.525  0.55  0.855  0.712  1.051  0.999  1.108  0.862  1.327  0.699  1.099  1.263  0.935  0.924  0.974  1.527  2.319  0.987  1.153  1.397  0.718  0.51  1.141  0.893  0.552  0.707  0.747  0.385  1.023  0.897  0.935  0.701  0.936  1.551  0.758  0.601  1.239  1.665  1.248  0.697  1.671  0.911  0.752  0.708  0.393  0.756  1.424  1.577  1.017  0.792  0.591  0.749  1.018  0.888  0.998  0.839  0.62  1.091  
 
 SLC30A6  55676  2  32390909  32449181  2p22.3  2.015  2.841  1.15  1.015  1.484  0.965  2.399  1.787  1.761  1.48  1.824  1.14  2.073  2.455  1.472  1.56  1.705  1.901  2.603  1.481  1.418  1.38  1.198  1.003  1.76  1.774  1.585  1.694  1.611  1.175  1.619  1.44  2.372  1.416  1.405  1.793  2.217  1.734  1.926  2.614  2.233  1.118  2.655  1.31  1.478  1.565  1.49  1.342  1.654  2.539  1.978  1.285  1.372  1.596  1.967  1.497  2.076  1.555  1.521  1.941  
 
 NLRC4  58484  2  32449517  32490812  2p22-p21  0  0.096  0.181  0  0  0  0.132  0.142  0.105  0.062  0.155  0.167  0  0.127  0.144  0.145  0.122  0.112  0.145  0.321  0  0.115  0.333  0  0  0.089  0.108  0.043  0.089  0.048  0.193  0  0.216  0.328  0  0.116  0.113  0  0.14  0.15  0  0.31  0.024  0  0.08  0.117  0.058  0  0.121  0.219  0.096  0.219  0.056  0.076  0.095  0  0.082  0  0.101  0  
 
 YIPF4  84272  2  32502957  32531658  2p22.3  1.557  3.934  1.755  1.785  1.817  1.418  2.126  2.179  2.763  2.058  2.175  1.279  2.016  2.507  1.63  1.963  2.451  3.255  2.935  1.666  1.538  1.505  1.83  0.816  1.785  2.386  1.071  1.377  2.011  0.917  2.096  2.309  1.69  1.631  1.549  2.046  1.304  1.442  3.04  2.099  2.585  2.027  2.37  1.186  1.565  1.51  0.799  1.436  1.21  3.605  2.186  1.146  0.984  1.463  1.821  1.541  1.691  1.942  1.348  2.02  
 
 BIRC6  57448  2  32582095  32843965  2p22.3  2.161  2.3  1.216  0.969  1.533  1.145  2.15  2.422  1.426  1.781  2.23  1.322  2.553  2.387  2.536  1.965  1.677  1.971  2.162  1.703  2.088  1.735  0.766  1.156  1.989  2.499  1.761  2.573  2.179  2.024  2.21  2.308  2.69  1.119  1.724  2.373  2.023  2.1  1.511  2.989  2.681  0.95  2.358  2.091  1.696  1.937  1.12  2.013  2.243  1.914  2.238  1.46  1.859  1.369  2.083  1.736  2.245  1.634  1.832  2.297  
 
 BIRC6-AS2  103752586  2  32782587  32799885  2p22.3  0  0  0  0  0  0  0  0  0  0  0  0  0  0  0  0.232  0  0  0  0  0  0  0  0  0  0  0.138  0  0  0.445  0.27  0  0  0  0.352  0  0.401  0  0  0  0.311  0.633  0  0  0  0.168  0  0.559  0  0.212  0.189  0  0.18  0  0  0  0  0.603  0  0  
 
 TTC27  55622  2  32853086  33046118  2p22.3  1.561  3.272  1.139  1.123  1.662  1.322  1.796  1.261  1.159  1.069  2.082  0.938  1.834  1.886  1.648  1.884  1.55  3.626  3.698  2.609  2.722  1.973  1.096  1.158  2.147  1.423  0.96  1.327  1.291  1.131  1.776  2.183  1.479  0.658  1.752  1.967  1.932  1.284  2.347  2.622  2.448  1.399  1.79  1.529  1.337  1.544  1.971  1.45  1.435  3.762  1.997  1.289  1.724  0.862  2.097  1.58  1.692  1.871  1.557  2.243  
 
 LINC00486  285045  2  33050509  33162270  2p22.3  0  0  0.174  0  0  0  0  0  0  0  0.081  0  0  0  0  0  0  0  0  0  0  0.071  0.214  0  0  0  0.062  0  0  0.076  0  0  0  0.523  0  0  0  0  0.056  0  0  0.298  0.041  0  0  0.148  0.042  0  0  0  0  0  0  0  0  0  0  0  0  0  
 
 LOC100271832  100271832  2  33152193  33171202  2p22.3  0.369  0.066  0.222  0  0.185  0  0.076  0  0.145  0.052  0.076  0.088  0  0.095  0.103  0  0  0.183  0  0  0  0.072  0.302  0  0  0  0.29  0  0  0.275  0  0  0  0.176  0  0.179  0  0  0  0  0.328  0  0.346  0  0  0.065  0.176  0  0  0  0.057  0  0  0  0  0  0  0  0  0  
 
 LTBP1  4052  2  33172368  33624575  2p22-p21  2.167  0.62  3.686  2.037  2.604  0.564  3.271  1.735  2.264  3.046  3.166  0.069  0.953  0.597  1.2  0.105  1.233  0.053  1.033  0.03  1.464  0.608  0.189  0.237  0.805  0.036  0.243  1.455  0.141  0.377  0.944  1.841  0.429  0.254  0.764  2.475  1.365  1.57  0.763  1.947  1.678  0.397  2.497  1.216  3.68  1.126  0.772  0.275  1.558  0.498  3.985  0.589  0.121  0.274  0.105  0.075  1.166  0  0  0.06  
 
 RASGRP3  25780  2  33661415  33789798  2p25.1-p24.1  0  0.757  0.164  0.104  0.063  0.278  1.836  0.038  0.231  0.179  0.199  0.173  0.046  0.073  0.054  0.225  0  0.157  0.124  0.154  0  0.224  0.253  0.111  0.053  0.039  0.257  0.7  0.089  1.514  0.484  0.272  0.102  0.324  0.03  0.615  0.235  0  0.187  0.05  0.094  0.594  0.062  0.052  0  0.088  0.016  0.055  0  0.274  0.115  0.214  0.02  0.388  0.059  0.081  0.059  0  0.035  0  
 
 FAM98A  25940  2  33808727  33824429  2p22.3  1.785  3.275  2.714  2.154  2.24  2.456  3.448  2.756  1.911  3.121  2.838  1.703  2.467  2.436  1.948  1.896  1.974  2.526  3.357  2.516  2.584  1.909  2.136  1.895  2.964  2.781  1.87  2.548  2.54  1.777  3.12  3.459  2.614  2.167  2.467  2.267  1.839  1.831  3.205  2.671  2.091  1.816  2.478  2.775  2.268  2.246  1.24  1.988  1.874  3.524  2.849  1.936  2.482  2.971  2.593  2.9  2.63  2.492  2.062  3.005  
 
 LINC01317  104355287  2  33931952  34522813  2p22.3  0  0  0  0  0  0  0  0  0  0  0  0  0  0  0  0  0  0  0  0  0  0.176  0.523  0.382  0  2.542  2.099  2.084  2.231  2.018  3.508  0.66  2.023  0.951  0  0.396  0  0  0.076  0  0  0.803  0.115  0  0  0  0.081  0  0  0  0  0  0  0  0  0  0  0  0  0  
 
 MYADML  151325  2  33951127  33953284  2p22.3  0  0  0.272  0  0.052  0  0.046  0.092  0.241  0.044  0  0  0  0.054  0  0.071  0.096  0.099  0  0.18  0  0.184  0.476  0  0.074  1.407  1.26  1.321  1.101  1.916  1.409  0.272  1.014  0.325  0.108  0.147  0  0  0.18  0  0  0.302  0.083  0  0  0.092  0.071  0  0  0  0.074  0.548  0.089  0.242  0.077  0.099  0  0  0.099  0  
 
 LINC01320  104355288  2  34902623  34947630  2p22.3  0  0  0  0  0  0.125  0  0  0.109  0  0  0  0  0  0  0  0  0.162  0  0  0  0.065  0.426  0.214  0  1.325  0.252  0.329  0  0.667  0.896  0  0.285  0.619  0  0  0  0  0  0  0  0.287  0.043  0.783  0.198  0.849  0  0  0.284  0  0  0.19  0.602  0.913  0  0.345  2.328  0  0  0.324  
 
 LOC100288911  100288911  2  36581891  36582713  2p22.3  0.721  3.722  1.977  1.11  2.619  3.272  0.768  0.739  1.297  1.146  2.384  0.246  0  1.183  0  0.131  0.331  1.041  0  0.142  0  0.329  0.537  0.363  0.565  0  0.263  0.659  0  0.185  0  0  0.396  0.309  2.344  1.829  1.428  0.93  4.123  1.261  1.155  1.561  4.552  0.992  1.653  2.137  3.776  1.437  1.105  3.226  0.914  2.268  1.289  3.177  4.188  2.282  1.133  2.099  2.321  2.485  
 
 CRIM1  51232  2  36583369  36778278  2p21  1.238  4.864  4.352  2.721  2.942  3.996  2.976  2.603  2.265  2.9  3.483  1.088  0.387  2.312  2.351  0.798  1.798  0.444  0.204  0.618  0.232  0.495  0.993  1.104  3.602  0.133  0.983  3.36  0.595  0.32  0.103  2.023  2.488  1.162  3.304  3.252  4.591  3.194  5.208  3.935  2.564  2.378  3.493  2.785  3.051  3.676  3.77  3.75  3.687  3.812  3.101  3.331  2.913  4.367  4.751  4.198  3.277  3.831  3.298  5.3  
 
 FEZ2  9637  2  36779403  36825332  2p21  2.185  5.043  2.63  1.855  1.945  3.219  3.885  2.593  3.616  3.12  3.99  0.926  2.138  2.888  1.731  2.516  2.507  3.451  3.267  3.068  1.998  2.292  0.933  0.859  4.127  2.723  1.968  2.309  2.076  1.37  2.817  3.361  2.374  2.093  2.857  2.944  3.042  2.515  4.17  2.947  3.2  2.428  3.694  2.281  1.296  2.44  3.106  2.518  3.14  4.476  3.917  1.909  2.335  2.029  3.252  2.777  2.498  3.983  2.543  3.19  
 
 VIT  5212  2  36923832  37041937  2p22.2  0  0.048  1.015  0  0.048  1.778  2.016  0.061  1.232  1.112  0.636  0  0.139  0.06  0  0  0  0.186  0.076  1.969  0  0.297  0.25  0  0  0  0.206  0.127  0.117  0.098  0  0.158  0.109  0.333  0  0  0.193  0  0.066  1.652  0.102  1.186  0.045  0  1.291  0  0.038  0.081  0  0.096  0  0.527  0.058  0.093  0  0.3  0  0  0  0  
 
 STRN  6801  2  37064840  37193615  2p22.2  1.084  1.466  0.697  0.544  1.209  0.749  1.252  1.248  0.851  1.123  1.422  0.941  1.131  1.417  1.031  0.915  1.149  1.249  1.709  1.035  1.062  1.159  0.722  0.459  1.354  1.184  0.817  1.027  0.958  0.938  1.312  1.221  1.339  0.703  0.873  1.452  1.264  1.002  1.152  1.81  1.61  0.599  1.091  1.033  0.667  1.186  0.704  0.723  1.4  1.13  1.021  0.805  0.85  0.895  1.171  1.046  1.148  1.052  0.789  1.091  
 
 HEATR5B  54497  2  37208143  37311488  2p22.2  1.046  0.907  0.562  0.435  1.266  0.432  0.802  0.587  0.703  0.906  1.133  0.549  1.33  0.969  0.8  0.708  0.781  0.622  1.674  0.954  1.103  0.843  0.986  0.783  0.875  1.501  1.112  1.099  1.365  1.044  1.5  1.14  1.474  0.687  0.542  1.395  1.051  1.065  1.02  1.615  1.071  0.553  1.349  0.876  0.59  0.919  0.324  0.667  0.801  1.219  1.021  0.653  0.592  0.565  1.001  0.696  0.922  0.833  0.58  1.257  
 
 GPATCH11  253635  2  37311593  37326387  2p22.2  1.348  2.496  0.957  0.559  1.038  1.066  2.376  1.099  1.559  1.369  1.563  0.821  1.129  1.275  0.926  0.7  1.176  1.666  2.483  1.442  1.431  1.392  1.997  1.182  1.495  1.894  1.236  1.098  1.705  0.617  1.726  1.464  1.643  1.202  0.831  1.179  1.218  1.704  1.3  1.265  0.989  1.001  2.008  0.932  0.48  0.834  1.363  0.881  1.063  1.658  1.389  0.633  0.937  0.876  1.4  0.863  1.06  1.22  0.744  1.348  
 
 EIF2AK2  5610  2  37332283  37384190  2p22-p21  2.202  2.997  1.58  2.632  2.043  1.87  2.413  2.339  1.828  2.233  3.038  1.555  2.567  2.929  2.484  4.15  2.187  1.752  2.644  1.663  1.547  2.712  1.472  2.57  3.085  2.127  1.8  1.807  2.114  1.441  2.608  2.336  2.194  1.482  2.003  2.236  2.178  2.172  3.745  2.803  2.977  1.887  2.447  2.389  1.523  2.279  1.456  1.742  2.391  2.003  2.526  1.426  2.035  2.736  2.567  2.489  3.67  1.981  1.936  2.65  
 
 SULT6B1  391365  2  37394962  37415690  2p22.2  0  0  0  0  0  0  0  0  0  0.063  0  0  0  0  0  0  0  0  0  0  0  0.057  0  0  0  0  0  0  0  0  0  0  0  0  0  0  0  0  0  0  0  0  0  0  0  0  0  0  0  0  0  0.224  0  0  0  0  0  0  0  0  
 
 CEBPZOS  100505876  2  37423634  37431886  2p22  2.377  3.212  1.614  1.104  2.318  2.378  2.615  1.693  2.171  1.971  3.385  2.466  1.976  2.987  2.196  2.291  2.543  3.315  1.728  3.871  2.272  2.13  1.774  2.112  2.294  2.603  1.821  1.935  3.031  1.336  3.353  3.098  2.606  2.095  2.607  2.504  1.838  2.855  3.368  2.489  2.757  2.238  3.019  2.299  1.633  2.436  2.957  1.548  2.382  2.666  3.059  1.639  1.993  2.323  2.868  2.269  2.254  2.404  1.587  2.294  
 
 CEBPZ  10153  2  37428774  37458740  2p22.2  2.542  4.562  1.551  1.971  2.681  1.885  2.411  2.127  2.372  2.242  2.798  2.179  3.015  3.046  2.5  2.931  2.995  4.971  3.849  3.191  2.929  2.761  1.897  2.388  3.06  2.906  1.663  2.602  2.494  2.346  3.456  3.25  3.215  2.417  2.737  2.955  2.484  2.039  3.13  3.268  2.664  2.289  2.693  3.059  2.221  2.444  1.874  1.782  2.141  3.302  2.493  1.824  2.162  2.035  2.758  2.423  2.346  2.2  2.307  2.84  
 
 NDUFAF7  55471  2  37458773  37476303  2p22.2  1.509  1.992  0.897  0.992  1.451  1.187  1.887  0.891  1.43  1.389  1.637  1.268  1.422  1.367  1.084  1.841  1.872  2.77  2.897  1.759  1.181  1.551  1.471  0.873  1.292  1.326  1.418  1.251  1.372  1.064  2.028  2.025  2.06  1.705  1.477  1.566  1.258  1.085  1.793  1.591  1.175  1.347  2.74  1.385  0.975  1.225  1.031  0.852  1.146  2.475  1.298  1.128  1.058  1.008  2.049  1.211  1.256  1.503  1.005  1.34  
 
 PRKD3  23683  2  37477645  37544222  2p21  1.562  3.259  1.667  1.353  1.382  1.98  2.068  2.002  1.617  2.15  1.959  1.31  1.251  1.392  1.687  2.133  2.15  2.62  2.747  1.487  1.196  1.322  1.977  1.706  2.555  3.236  2.551  2.297  3.187  3.701  5.064  3.046  3.161  1.811  1.128  2.292  2.089  1.742  1.41  2.214  1.504  1.54  2.456  1.926  1.629  1.643  1.926  0.976  2.214  2.032  2.144  0.958  1.594  1.311  2.168  1.778  2.438  1.528  0.172  2.18  
 
 QPCT  25797  2  37571752  37600465  2p22.2  0  0.11  0  0.848  0.56  0  1.003  1.795  0.131  0.058  0  2.259  0  0  1.876  3.966  0.08  1.736  0.109  0.326  0  0.037  0.402  0.707  2.319  4.992  3.47  2.453  5.932  5.593  7.348  4.773  4.511  4.329  1.636  2.597  1.827  0.626  1.776  0.924  0  3.755  1.496  0  1.729  0.065  1.616  0.382  0.228  1.175  0.05  0.273  0  0.12  2.183  0.16  2.932  0.289  0  2.224  
 
 CDC42EP3  10602  2  37869024  37899678  2p21  0.202  5.999  4.318  2.54  1.785  3.854  4.102  2.152  4.092  2.322  4.175  1.02  0.193  3.431  0.861  2.028  1.417  2.922  0.861  1.437  0.162  1.37  1.369  0.77  2.844  2.894  3.084  3.484  2.615  2.844  2.056  4.648  3.812  2.921  1.801  2.949  3.213  3.852  4.02  0.729  3.136  3.223  2.583  0.866  2.684  3.385  3.911  2.603  3.222  3.888  2.453  1.729  2.805  2.265  4.114  3.141  2.952  4.649  2.577  4.118  
 
 LINC00211  100873959  2  38053389  38103006  -  0  0.331  0.092  0  0  0  0.108  0  0.162  0.07  0  0  0  0  0  0  0  0.104  0  0  0  0.081  0.35  0  0  0  0.064  0.053  0  0.039  0.065  0.087  0  0.209  0  0  0.109  0  0.149  0  0  0  0.032  0  0  0  0.085  0  0  0.061  0  0.285  0  0.051  0  0  0  0  0  0.071  
 
 RMDN2  151393  2  38152461  38294285  2p22.2  0.212  0.924  0.331  0  0.32  0.316  0.609  0.615  0.499  0.591  0.624  0.476  0.39  0.604  0.407  0.478  0.31  0.949  0  0.224  0.259  0.219  0.197  0  0.669  1.197  0.275  0.351  0.618  0.365  1.014  0.799  0.373  0.48  0.269  0.559  0.318  0.588  0.328  0.489  0.885  0.509  1.125  0.252  0.356  0.727  0.234  0.129  0.468  0.544  0.271  0.302  0.354  0.329  0.591  0.249  0.728  0.517  0.298  0.626  
 
 RMDN2-AS1  101410544  2  38177476  38263484  2p22.2  0  0  0  0  0  0  0  0  0  0  0  0  0  0  0  0  0  0  0  0  0  0  0  0  0  0  0.074  0  0  0  0  0  0  0  0  0  0  0  0  0  0  0  0  0  0  0  0  0  0  0  0  0  0  0  0  0  0  0  0  0  
 
 CYP1B1  1545  2  38294745  38303323  2p22.2  2.023  4.782  1.222  3.601  3.833  0.468  2.259  2.052  0.297  4.356  0.422  0.139  0.25  0.469  0.851  1.586  1.175  0.303  0.038  1.627  0.06  0.445  0.249  0.039  0.095  2.397  0.583  0.031  0  0.426  0  0.034  0  0.34  2.126  4.418  2.937  2.785  4.544  4.413  6.457  3.623  2.291  0.07  0.286  0.843  1.885  0.681  5.075  1.387  1.182  2.098  2.343  2.607  2.326  1.482  4.686  0.511  1.803  0.448  
 
 CYP1B1-AS1  285154  2  38358246  38408993  2p22.2  0.304  0  0.972  0  0.285  0  0.151  0.324  0.146  1.288  0.055  0  0  0.075  0  0.365  0  0.06  0  0.459  0  0.06  0.177  0  0  0.066  0.053  0.034  0  0.039  0  0  0  0.284  0  0.254  0.143  0.102  0.133  0.384  0.482  0.159  0.035  0  0.091  0.138  0  0  0.483  0  0  0.176  0.048  0.113  0.046  0.076  0.336  0  0  0  
 
 ATL2  64225  2  38521098  38604432  2p22.3  2.272  3.693  1.484  1.409  1.507  1.216  2.659  1.869  1.91  2.098  3.004  1.882  1.881  3.396  2.661  2.758  2.712  3.01  3.913  1.913  2.101  2.28  1.979  1.472  2.116  2.788  2.099  2.239  2.411  2.041  2.876  2.745  2.519  1.819  1.994  2.63  2.71  2.446  2.053  3.092  2.907  2.035  2.643  2.56  1.675  1.965  1.76  1.394  1.788  3.276  2.462  1.384  1.757  1.498  2.148  1.865  2.123  2.022  2.003  1.896  
 
 LOC101929596  101929596  2  38685779  38742882  2p22.1  0  0.118  0.121  0  0.106  0  0.168  0  0.13  0.061  0.099  0.115  0  0.058  0  0  0  0.219  0  0  0  0.101  0.363  0  0  0.263  0.241  0.182  0.241  0.547  0.426  0.262  0.139  0.274  0.109  0  0.105  0  0.239  0  0  0.278  0.095  0  0  0.066  0.098  0  0  0.185  0  0  0  0.071  0  0  0  0  0  0  
 
 HNRNPLL  92906  2  38790327  38830178  2p22.1  2.293  4.295  1.508  1.262  2.112  1.67  3.258  1.906  1.866  2.208  2.961  1.937  2.443  3.534  2.092  2.326  2.899  4.601  4.461  2.371  2.599  2.439  1.626  1.05  2.142  2.356  1.591  1.791  2.113  1.61  2.752  2.707  2.008  1.59  1.448  2.419  2.08  2.211  2.45  2.786  2.22  1.499  3.181  2.458  1.274  1.972  1.871  1.179  1.799  2.93  2.58  1.913  0.569  0.929  0.884  0.953  1.581  2.026  0.088  0.552  
 
 GALM  130589  2  38893051  38961909  2p22.1  1.961  1.13  0.499  0.307  1.617  0.153  0.231  1.313  0.395  0.096  1.525  2.722  2.667  2.066  1.466  2.67  1.784  0.906  2.919  1.211  2.1  1.756  3.144  1.455  0.82  2.146  1.287  1.603  1.719  2.506  2.82  0.966  1.098  0.966  2.185  1.705  1.541  2.308  0.052  1.711  1.148  0.69  0.041  1.787  1.075  1.31  1.742  1.049  0.363  1.739  1.597  1.2  0.054  0.363  0.425  0.299  0.519  1.609  0.141  0  
 
 SRSF7  6432  2  38970740  38978636  2p22.1  4.526  5.816  2.819  3.268  4.542  3.202  5.034  4.081  3.217  3.953  4.613  3.868  3.657  5.095  3.783  4.431  4.61  6.291  6.841  5.128  4.314  5.395  3.131  3.33  4.145  3.898  3.85  3.455  3.847  3.607  4.086  4.446  4.13  3.114  3.805  4.079  3.942  3.49  4.56  4.257  3.974  3.935  4.887  4.297  3.618  3.784  4.02  3.347  3.712  5.635  4.185  2.94  3.92  3.143  4.798  3.883  3.413  4.155  3.936  4.32  
 
 GEMIN6  79833  2  39005326  39009106  2p22.1  4.215  5.267  2.479  2.386  3.388  3.076  5.047  2.908  2.198  2.891  3.759  2.959  3.826  4.741  2.396  3.23  3.045  3.858  5.307  3.55  3.218  3.965  2.79  3.152  4.322  3.397  2.654  3.731  3.802  2.37  4.329  4.85  4.398  3.069  4.478  3.369  3.703  3.525  3.107  4.309  4.134  2.71  4.913  3.854  2.768  3.189  4.376  2.697  2.376  4.53  3.125  2.438  3.516  3.341  4.129  3.374  2.447  5.229  2.916  3.132  
 
 DHX57  90957  2  39024875  39103021  2p22.1  1.529  1.556  0.714  0.987  1.528  1.044  1.983  1.063  1.221  1.425  1.907  0.583  1.145  1.895  1.583  0.965  0.965  1.082  2.08  0.974  1.352  1.379  0.898  0.801  1.478  1.497  1.152  1.462  1.406  0.552  1.517  1.952  1.51  0.84  1.405  1.652  1.207  1.366  1.286  2.414  1.676  0.894  2.541  1.575  1.097  1.318  0.617  0.963  1.351  1.787  1.492  0.981  1.17  0.951  1.667  1.077  1.125  1.74  1.217  1.624  
 
 MORN2  729967  2  39103102  39109850  2p22.1  1.96  4.054  1.512  1.818  3.3  2.558  3.384  2.122  2.053  2.846  3.347  2.113  1.598  2.485  1.109  2.017  1.829  4.581  2.847  2.243  1.608  1.31  0.94  1.245  2.896  2.424  2.033  1.927  2.058  1.174  2.895  3.842  2.281  1.66  2.689  2.944  1.741  1.939  3.221  2.535  2.896  1.975  3.048  1.733  2.196  2.214  2.638  1.771  2.119  4.18  2.539  1.117  2.563  1.729  2.766  2.376  1.268  3.914  2.447  2.532  
 
 ARHGEF33  100271715  2  39146503  39202590  2p22.1  0  0.028  0.087  0  0.028  0  0  0.052  0.033  0.05  0.032  0.026  0  0.047  0.044  0.05  0.039  0.063  0.022  0.081  0  0.147  0.159  0  0  0.032  0.062  0.026  0  0.065  0  0  0  0.177  0  0.089  0  0.081  0.054  0.412  0  0.177  0.038  0.036  0  0.065  0.059  0.121  0.047  0.497  0  0.678  0  0.072  0.064  0  0  0.026  0  0  
 
 LOC375196  375196  2  39186428  39187485  2p22.3  0  0  0  0  0.122  0.305  0.431  0.109  0.162  0.162  0.126  0  0  0.072  0  0.238  0.232  0.209  0  0.152  0  0.666  0.305  0  0  0  0.161  0.161  0.156  0.122  0  0  0  0.454  0  0.238  0.158  0  0.38  0.807  0  1.205  0.449  0  0  0.303  0.174  0.404  0  1.127  0  1.135  0.245  0.185  0.389  0  0  0  0  0.133  
 
 SOS1  6654  2  39208689  39347604  2p21  2.091  2.505  1.226  1.325  1.302  1.222  2.098  1.443  1.42  1.758  2.106  0.875  1.585  2.055  1.921  1.223  2.189  2.273  1.752  2.011  1.742  1.992  0.692  0.857  1.851  2.015  1.366  1.656  1.474  1.278  1.603  1.746  1.834  0.866  1.435  2.171  1.672  1.61  2.131  2.12  1.877  0.927  2.656  1.822  1.252  1.698  1.503  1.207  1.946  1.683  2.125  1.078  1.388  1.184  1.772  1.685  1.787  1.562  1.002  1.865  
 
 CDKL4  344387  2  39405687  39456673  2p22.1  0  0  0  0  0  0  0  0  0.106  0.068  0  0  0  0.026  0  0  0  0.094  0  0  0  0  0  0  0  0  0.026  0  0  0.03  0  0  0  0.197  0  0  0  0  0.08  0  0  0.39  0  0.263  0  0.054  0.027  0  0  0  0.073  0.167  0  0.036  0  0  0  0  0  0  
 
 MAP4K3  8491  2  39476406  39664453  2p22.1  0.94  2.12  0.807  0.366  1.471  0.78  1.79  1.178  1.06  1.216  1.869  0.734  1.469  1.716  1.216  1.252  1.692  2.167  1.778  0.695  0.875  0.984  0.273  0.317  1.517  2.209  0.988  1.163  1.669  0.754  2.465  1.629  1.264  0.636  1.114  1.922  1.57  1.213  2.331  1.7  2.077  0.716  2.04  2.366  1.25  1.951  1.705  0.938  1.992  2.629  2.219  1.115  0.886  1.317  2.139  1.262  1.376  1.265  1.381  2.01  
 
 LOC728730  728730  2  39664556  39828484  2p22.1  0.093  0.961  0.266  0  0.281  0.159  0.296  0.218  0.367  0.14  0.255  0.318  0.518  0.69  0.066  0.162  0.758  1.291  0.473  0.045  0.13  0.152  0.167  0.063  0.163  0.393  0.282  0.341  0.109  0.175  0.728  0.286  0  0.317  0.323  0.322  0.481  0.612  0.775  0.118  0.495  0.263  0.335  0.606  0.075  0.468  0.424  0  0.29  0.281  1.39  0.163  0.108  0.164  0.671  0.192  0.137  0.503  0.123  0.438  
 
 TMEM178A  130733  2  39892637  39945104  2p22.1  0  0.505  0.203  0  0.253  0.175  0.082  0.102  1.075  0.152  0.515  0.14  0.193  0.287  0  0.082  0.294  0.379  0.314  0.1  0.161  0.292  0.439  0  0  0.199  0.294  0.17  0  0.153  0.236  0.103  0  0.363  0  0.106  0.43  0.683  0.621  0  0.235  0.753  1.77  2.301  1.838  0.598  0.294  0  0.332  0.063  0.501  1.087  0  0.246  0.339  0  0.118  0.186  0  0.184  
 
 THUMPD2  80745  2  39963199  40006416  2p22.1|2p22-p21  1.296  2.803  1.315  0.665  1.241  0.838  1.434  0.937  0.972  0.808  1.483  1.064  1.421  2.42  1.003  1.418  1.432  2.991  2.919  1.69  1.78  2.133  0.938  0.806  1.773  0.908  0.736  0.742  0.798  0.723  1.66  1.264  1.097  0.803  1.129  1.167  1.155  1.021  2.062  1.213  1.371  1.067  2.229  1.788  0.677  1.063  1.686  0.755  0.552  2.444  1.046  0.899  0.797  0.782  1.783  1.102  0.789  1.635  1.148  1.008  
 
 SLC8A1-AS1  100128590  2  40144773  40482349  2p22.1  0  0  0.176  0  0  0  1.035  0  0  0  0.189  0.543  0  0.057  0  0  0  0  1.217  0.129  0  0.399  0  0.141  0.649  0  1.2  0  0  1.294  0  0  0  0.226  0.149  0  0.117  0.548  0.185  0  0  0  0.151  0.881  0  0.495  1.403  0  0.509  0.106  0.098  0.203  0.121  0.23  0.155  0  0.713  0.257  0  0  
 
 SLC8A1  6546  2  40339285  40739575  2p22.1  0.557  0  1.513  1.133  0.017  0.733  0.217  0.712  0.311  0.401  0.856  0.091  0  0.017  0.029  0.078  0.044  0.089  0.032  0.11  0.117  0.227  0.243  0.071  2.002  0  0.314  0.173  0.146  2.343  0.227  0.198  0.033  0.261  0.085  0.054  0.368  0.156  0.225  1.584  0.433  0.277  0.046  2.707  0.423  1.376  0.219  0.147  1.492  0.232  0.409  0.229  0.456  1.582  0.799  0.202  2.046  1.358  0  0.8  
 
 LOC388942  388942  2  42104694  42121186  2p21  0  0  0.177  0  0  0  0.096  0  0.153  0.043  0.038  0  0  0.031  0.121  0.063  0.107  0  0  0  0.147  0.076  0.256  0.091  0.097  0.063  0.129  0.196  0  0.055  0  0  0  0.213  0.134  0  0.073  0.191  0.096  0.124  0  0.066  0.071  0  0  0.052  0.424  0  0  0.087  0.082  0.479  0.053  0.134  0.142  0.115  0.075  0  0.121  0  
 
 LOC101929723  101929723  2  42158738  42160863  2p21  0  0  0  0  0  0  0.53  0.103  0  0.517  0  0  0  0  0  0  0  0  0  0  0  0  0  0  0  0.136  0.053  0.281  0.411  0  0.394  0  0  0  0.992  0  0.222  0  1.128  0  0  0  0  0  0  0  0  0  0  0  0  0.199  0.446  0.389  0  0  0.496  0  0  0  
 
 C2orf91  400950  2  42162507  42180943  2p21  0  0  0.152  0  0  0  0  0  0.115  0.019  0  0  0  0.012  0  0.023  0.029  0  0  0.034  0  0.04  0.32  0  0  0  0.072  0  0  0.03  0  0  0  0.225  0.045  0  0  0  0.045  0  0  0.047  0.02  0  0  0.043  0.023  0  0  0  0.026  0.111  0  0.03  0  0  0  0  0  0  
 
 PKDCC  91461  2  42275160  42285668  2p21  0.204  0.125  0.878  0.802  0.126  0.516  1.097  1.113  0.712  1.666  0.547  0.153  2.296  0.083  0.387  2.085  3.011  0.207  0.036  0.056  0.35  0.323  0.41  0.055  0.066  0.212  0.374  0.924  0.506  0.783  0.764  1.319  0.155  0.231  3.539  0.613  0.948  0.539  0.61  0.701  0.088  1.485  0.068  2.153  0  0.111  1.221  0.437  0.25  0.905  0.726  0.905  0.983  1.468  0.926  1.918  1.197  0.302  0.145  0.145  
 
 LOC102723824  102723824  2  42369574  42397371  2p21  0.141  0  0.173  0  0.084  0  0.095  0  0.159  0.145  0.101  0.239  0  0.104  0.098  0.35  0.642  0  0.097  0.105  0  0.257  0.167  0  0  0  0.116  0.096  0.155  0.068  0  0  0  0  0.162  0  0.086  0  0.283  0  0.094  0  0.255  0.173  0  0.154  0.043  0.104  0.221  0  0.068  0.188  0.191  0.093  0.247  0.243  0.136  0  0.137  0  
 
 EML4  27436  2  42396477  42559688  2p21  2.861  3.397  1.429  1.41  1.767  2.062  3.176  2.003  1.643  2.156  2.837  2.921  3.075  3.611  3.178  3.773  3.047  2.276  3.457  1.542  2.235  2.057  1.365  1.334  2.556  2.53  1.951  2.433  2.575  2.205  3.008  2.773  2.565  1.226  2.903  3.211  2.803  2.843  1.827  4.117  2.952  1.963  3.115  2.782  2.02  2.347  3.94  2.164  2.042  3.313  2.507  1.71  2.366  2.434  3.064  2.296  2.549  2.801  2.338  2.807  
 
 COX7A2L  9167  2  42577644  42588356  2p21  4.972  5.28  4.2  4.472  4.609  3.905  5.395  4.152  4.941  4.188  4.952  3.922  4.319  5.465  3.685  4.756  5.231  4.645  6.478  4.904  4.282  4.788  5.118  3.152  5.245  4.413  4.244  3.77  4.551  4.589  5.191  5.028  4.324  4.144  4.587  4.421  4.056  4.346  5.251  4.912  4.295  4.682  5.327  4.24  4.045  4.681  4.501  3.655  4.393  6.258  4.695  2.994  3.738  3.886  4.764  4.479  3.879  5.278  3.933  4.474  
 
 KCNG3  170850  2  42669156  42721237  2p21  0.151  0.098  0.13  0.152  0.184  0.161  0.185  0.086  0.055  0.1  0.079  0.135  0.098  0.278  0.069  0.22  0.265  0.168  0.225  0.184  0  0.662  0.247  0.042  0.092  0.209  0.227  0.26  0.387  0.327  0.476  0.168  0  0.173  0.234  0.079  0.138  0.085  0.35  0.399  0.089  0.765  0.425  0.259  0.217  0.249  0.301  0.136  0.491  0.158  0.16  0.998  0.091  0.245  0.579  0.205  0.06  0.135  0.181  0.046  
 
 MTA3  57504  2  42721708  42984087  2p21  2.624  3.265  1.3  1.288  3.058  1.787  2.292  1.78  1.818  1.949  2.433  1.884  2.141  3.038  2.144  2.486  2.547  3.236  4.456  0.931  1.931  3.023  0.502  0.231  2.258  2.31  1.646  1.747  1.914  1.898  2.759  2.582  1.994  1.52  2.043  2.758  2.419  1.856  2.951  3.565  3.408  1.462  3.588  3.276  1.844  2.317  1.824  1.673  2.038  3.548  1.93  1.842  1.82  1.684  3.067  2.537  2.184  3.107  1.758  2.747  
 
 OXER1  165140  2  42989638  42991401  2p21  0.311  0  0.188  0  0.074  0  0.057  0  0.12  0.081  0.11  0.064  0  0.037  0.179  0  0  0.114  0.073  0  0  0.208  0.584  0  0  0.064  0.076  0.104  0  0.084  0  0  0.085  0.28  0  0  0.245  0  0.035  0.893  0.115  0.487  0.102  0.253  0.202  0.135  0.063  0.115  0  0.062  0  0.786  0  0.071  0.238  0  0  0  0  0  
 
 HAAO  23498  2  42994228  43019751  2p21  0.39  0  0  0  0.075  0  0.133  0.114  0  0.099  0  0  0.253  0.095  0.113  0  0.092  0  0.122  0.303  0  0.179  0.376  0  0  0  0.207  0.06  0  0.049  0.15  0  0  0.404  0  0  0.506  0  0.041  1.372  0.15  0.055  0.059  0  0.223  0.201  0  0  0  0  0  0.627  0  0.067  0.591  0.176  0.105  0.161  0.117  0  
 
 LOC102723854  102723854  2  43254991  43266682  2p21  0  0  0.146  0  0.03  0  0  0  0.149  0.031  0.042  0.314  0  0  0  0.197  0  0.228  0  0.063  0  0.104  0.411  0  0  0.04  0.062  0.08  0  0.038  0  0  0  0.269  0  0  0.067  0  0.031  0  0  0.041  0.037  1.236  0.157  0  0.809  0.198  0  1.073  0  0.384  0  0.03  0  0  0  0.072  0  0  
 
 ZFP36L2  678  2  43449540  43453745  2p22.3-p21  2.424  2.914  3.39  4.215  3.767  2.673  2.605  1.81  2.853  2.665  3.513  3.71  3.537  2.686  2.005  3.394  4.674  3.453  5.146  5.276  3.005  5.056  4.588  4.313  2.179  3.375  2.838  2.367  2.752  2.739  3.883  4.064  3.292  2.729  2.851  2.45  2.209  3.133  2.955  2.899  2.062  2.988  2.619  3.879  3.294  3.06  2.585  2.499  2.292  3.784  2.697  2.48  1.459  1.747  1.871  2.354  1.91  3.677  2.38  1.583  
 
 LINC01126  100129726  2  43454349  43455994  2p21  0.586  0.07  0.158  0  0.397  0.136  0.158  0.1  0.156  0.269  0.151  0.095  0.214  0.171  0.446  0.263  0.112  0.065  0.245  0.302  0.242  0.388  0.538  0.334  0.122  0.229  0.394  0.424  0.438  0.239  0.225  0.241  1  0.37  0.246  0.395  0.189  0.244  0.132  0.362  0.463  0.436  0.388  0.401  0.118  0.266  0  0.214  0  0  0.186  0.806  0.176  0.311  0.29  0.198  0.321  0.183  0.297  0.145  
 
 THADA  63892  2  43457974  43823185  2p21  1.247  0.786  1.181  0.821  1.484  0.866  1.988  1.229  1.339  1.329  1.676  0.903  1.678  1.364  1.444  1.305  0.631  0.828  2.506  1.354  1.683  1.572  0.784  0.784  1.393  1.619  1.198  1.693  1.495  1.752  1.762  2.09  1.665  0.858  1.408  1.931  1.8  1.308  1.181  2.551  1.708  1.066  1.654  1.365  0.995  1.283  1.321  0.773  1.457  1.933  1.608  0.882  1.024  0.777  1.416  1.135  1.284  1.546  1.003  1.824  
 
 PLEKHH2  130271  2  43864438  43995126  2p21  0.187  0.771  0.111  0  0.244  0.302  0.285  0.169  0.74  0.571  0.753  0  0.063  0.94  0.06  1.07  0  0.684  0.017  0.033  0.296  0.198  0.159  0  0.074  1.088  0.456  0.83  0.63  0.72  0.963  1.073  0.52  0.221  0.298  0.555  0.264  0.046  0.098  0.183  0.154  0.076  0.836  0.901  0.053  0.603  0.495  0.131  0.273  0.279  1.307  0.27  0.153  0.18  1.849  1.062  0.123  0.691  0.631  1.053  
 
 C1GALT1C1L  728819  2  43902291  43903461  2p21  0.973  1.395  0.305  0  0.746  0.492  0.596  0.174  0.628  0.256  0.13  0  0  0.367  0  0.469  0  2.465  0  0.649  0.352  0.118  0  0  0  0.319  0.082  0  0  0.211  0.163  0.476  0  0.292  0.376  1.027  0.714  0.243  0.066  0  0  0.615  0  0  0.293  0.277  0.741  0  0.357  0  0  0.33  0.227  0  1.269  1.106  0.413  0.799  0.772  0.96  
 
 DYNC2LI1  51626  2  44001177  44037149  2p25.1-p24.1  1.288  2.553  0.564  0.444  2.175  0.199  1.39  0.684  1.241  1.093  1.563  1.079  1.119  2.585  0.849  1.341  2.235  4.343  2.372  1.063  0.96  0.681  0.898  0.165  1.082  1.554  0.733  0.919  1.063  0.531  1.794  1.917  0.476  0.436  1.005  1.567  0.788  0.849  1.445  1.365  1.682  1.008  0.721  1.01  0.539  1.148  0.296  0.304  1.283  2.088  1.346  0.691  0.767  0.888  2.537  1.539  0.925  1.342  0.875  2.082  
 
 ABCG5  64240  2  44039610  44065958  2p21  0  0  0.055  0  0.036  0.047  0  0.042  0.08  0.025  0.032  0  0  0.018  0  0.059  0  0  0  0  0  0.027  0.243  0  0  0.242  0.341  0.207  0.144  0.073  0.207  0.332  0  0.185  0  0  0  0  0.051  0  0.08  0.177  0.029  0  0  0.05  0.064  0  0  0  0  0.28  0.025  0.029  0.114  0.053  0  0  0  0  
 
 ABCG8  64241  2  44066102  44105605  2p21  0  0.033  0.11  0  0.038  0.062  0  0  0.087  0.054  0  0  0  0.049  0  0.055  0  0.143  0  0.095  0  0.174  0.355  0  0  0  0.108  0.027  0  0.08  0  0.046  0  0.271  0  0.062  0  0  0.048  0  0  0.393  0.039  0  0.09  0.062  0.076  0  0  0.042  0  0.669  0  0.055  0.042  0  0  0  0  0  
 
 LRPPRC  10128  2  44113362  44223144  2p21  3.034  4.237  1.324  1.814  2.872  1.595  2.273  1.755  2.235  1.819  2.869  2.283  2.344  4.215  3.453  3.008  3.345  4.779  4.445  3.655  3.332  2.698  2.328  2.244  2.721  3.12  2.44  2.574  2.639  3.157  3.501  3.339  3.331  2.332  2.621  3.012  3.025  2.603  2.38  4.516  2.814  2.565  3.776  3.707  2.005  2.617  2.173  2.12  2.452  4.314  2.805  2.333  2.111  1.915  3.198  2.871  2.559  3.007  2.365  2.823  
 
 PPM1B  5495  2  44395941  44461742  2p21  2.405  3.806  1.591  1.443  1.985  1.629  2.056  1.23  2.412  1.739  2.41  1.635  2.549  2.894  2.234  2.008  2.431  3.552  3.964  2.338  1.5  2.269  1.854  0.928  1.628  2.044  1.567  1.652  1.778  1.451  2.153  1.825  1.804  1.665  2.074  2.561  2.225  1.614  2.483  2.462  2.002  1.441  2.981  2.978  1.356  2.045  2.813  1.496  2.301  3.153  1.926  1.542  1.698  1.75  2.842  2.079  2.008  2.231  1.633  2.273  
 
 SLC3A1  6519  2  44502596  44547962  2p16.3  0.095  0.049  0.131  0  0  0  0  0.048  0.153  0.032  0.05  0.055  0.366  0.165  0  0.06  0  0.099  0.14  0  0  0.081  0.142  0  0  0  0.124  0.058  0  0.048  0  0  0  0.167  0.694  0.15  0.144  0  0  0  0  0.03  0.027  1.581  0  0.204  0.047  0  0.121  0.04  0.077  0.299  0.077  0.469  1.328  0.198  0.195  0  0.167  0.122  
 
 PREPL  9581  2  44544747  44589001  2p21  2.03  1.655  1.686  1.546  2.617  1.168  2.669  1.863  2.343  2.114  2.161  1.633  1.872  2.281  1.813  1.659  2.096  2.843  2.957  2.105  1.769  1.82  1.477  1.125  1.927  2.15  1.518  1.55  1.969  1.804  2.318  2.595  2.168  1.52  1.673  2.556  1.749  1.793  2.469  2.611  2.246  1.394  2.721  1.604  1.191  2.051  2.177  1.187  1.765  2.687  2.386  0.995  1.164  1.513  2.347  1.869  1.575  2.254  1.111  2.279  
 
 CAMKMT  79823  2  44589042  44999731  2p21  1.126  0.588  0.409  0.611  1.62  0.51  1.127  0.538  0.852  0.62  1.099  1.158  0.84  1.528  0.911  1.17  1.579  1.083  2.7  1.842  0.542  1.553  1.212  0.89  0.719  0.667  0.888  0.483  0.655  0.604  0.991  0.899  0.766  0.607  0.891  1.496  0.523  0.654  0.306  0.798  1.31  0.847  0.717  1.513  0.494  1.298  1.035  0.434  0.817  0.949  1.217  0.795  0.459  0.536  1.074  0.533  0.733  0.881  0.739  0.576  
 
 SIX3-AS1  100506108  2  45167292  45169017  2p21  0  0  0.253  0  0.788  0  0.236  0  0  0.282  0.128  0.264  0.258  0.456  0.212  0.14  0.142  0.257  0.264  0  0.485  0.576  0.327  0.161  0  0.347  0.362  0.171  0  0.137  0  0  0  0.262  0.239  0.38  0.404  0  0.066  0  0  0.433  1.726  0.575  0  0.213  0.176  0  0.431  0.317  0.254  0.733  0.245  0.099  0.104  0.253  0.168  0  0.305  0.214  
 
 SIX3  6496  2  45169036  45173216  2p21  0  0.083  0.242  1.622  1.403  0.11  0.135  0.066  0.283  0.379  0.338  0.128  0  0.573  0  0.141  0.109  0.339  0.085  0.21  0  1.077  0.528  0.098  0.062  0.107  0.421  0.049  0.085  0.264  0.131  0.074  0  0.318  0.223  0.544  0.789  0  0.166  0.353  0  0.77  3.609  1.575  0.24  0.176  0.487  0.236  0.074  0.441  0.105  1.272  0.039  0.189  0.201  0.166  0.043  0.095  0  0  
 
 SIX2  10736  2  45232323  45236542  2p21  0.311  0.1  0.209  2.596  0.237  0.204  0.06  0.186  0.237  0.16  0.133  0.098  0  0.149  0  0.111  0.08  0.363  0.043  0.106  0  0.339  0.274  0  0.074  0.101  0.226  0.072  0  0.166  0  0.065  0  0.312  0.091  0.66  0.5  0  0.14  0.264  0  1.252  0.15  0.121  1.552  0.804  0.129  1.368  0  1.718  0.777  1.11  0.075  1.032  0.237  0.127  1.019  0.193  0  0  
 
 LINC01121  400952  2  45401479  45482080  2p21  0  0  0.125  0  0  0  0  0.061  0  0  0  0  0  0.049  0  0  0  0  0  0  0  0  0  0  0  0  0.065  0  0  0.136  0  0  0  0.283  0  0  0  0  0  0  0  0.335  0  0  0  0  0.039  0  0  0  0  0  0  0.066  0.13  0  0  0  0  0  
 
 SRBD1  55133  2  45615818  45838433  2p21  1.541  2.644  0.722  0.473  1.152  0.776  1.264  0.951  0.75  1.14  1.644  0.675  1.568  2.184  1.638  1.143  1.841  1.941  2.776  1.578  1.249  1.999  1.011  0.863  1.435  1.743  1.095  1.112  1.586  1.063  1.921  1.537  1.7  0.842  0.856  1.549  1.647  1.223  0.856  2.018  1.372  0.834  1.607  1.411  1.136  0.913  1.02  0.774  1.281  1.857  1.52  1.01  1.111  0.702  1.224  0.812  1.545  1.189  0.906  1.815  
 
 PRKCE  5581  2  45879042  46415129  2p21  0.621  1.6  0.381  1.008  0.939  0.442  0.819  0.567  0.611  0.683  0.67  0.629  0.794  0.707  0.611  0.845  0.327  0.196  1.466  0.227  0.07  0.758  0.632  0.532  2.13  1.361  1  1.204  1.498  1.029  1.78  1.202  1.335  0.915  0.622  1.477  1.814  1.114  0.864  0.79  0.67  0.424  0.653  0.492  0.842  0.753  0.51  0.56  0.577  0.805  0.553  0.877  0.634  0.883  2.449  1.163  0.857  1.999  0.768  1.757  
 
 EPAS1  2034  2  46524540  46613842  2p21-p16  0.075  2.5  3.368  3.843  3.374  3.58  1.837  2.737  2.988  5.186  1.364  2.027  1.471  2.031  1.271  1.389  1.639  0.026  0.261  0.195  0.762  0.393  1.138  0.848  1.596  2.463  0.838  1.002  1.927  1.165  2.99  1.704  1.318  0.803  3.325  4.176  3.242  3.472  4.177  1.744  3.102  2.042  0.064  2.877  2.06  3.319  3.858  0.814  2.288  0.944  3.079  1.633  2.716  3.002  3.996  2.773  4.472  1.622  3.955  4.143  
 
 LOC101805491  101805491  2  46656328  46668972  -  0  0  0  0  0  0  0  0  0  0  0  0  0  0  0  0  0  0  0  0  0  0  0  0  0  0  0.087  0  0  0  0  0  0  0  0  0  0  0  0  0  0  0  0  0  0  0  0  0  0  0  0  0  0  0  0  0  0  0  0  0  
 
 TMEM247  388946  2  46706703  46711564  2p21  0  0  0  0  0  0  0  0  0  0  0  0  0  0  0  0  0  0  0  0  0.992  0.212  0.38  0  0  0  0.404  0  0  0.139  0  0  0  0.376  0  0  0  0  0  0  0  0  0.077  0  0  0  0  0  0  0  0  0.832  0  0  0  0  0  0  0  0  
 
 ATP6V1E2  90423  2  46738985  46747096  2p21  0.734  0.913  0.571  0.538  0.439  0  0.483  0.225  0.833  0.658  0.446  0.142  0.891  1.125  0.348  0.038  0.728  0.578  0.622  0.382  0.515  0.407  1.789  0.315  0.845  0.516  0.912  0.7  1.051  0.859  1.088  0.603  0.434  1.04  1.244  0.547  0.25  0.526  0.4  1.85  0.548  0.404  1.873  0.367  0  0.229  0.595  0  0.698  0.744  0.971  0.681  0.095  0.675  0.701  0.677  0.275  0.517  0.111  0.296  
 
 RHOQ  23433  2  46769866  46811827  2p21  1.717  3.203  2.021  1.95  1.64  3.073  2.511  2.498  2.366  2.354  2.586  1.322  1.55  3.037  1.099  1.112  0.835  1.722  2.144  2.014  1.376  1.011  1.719  0.985  2.982  4.125  4.379  3.049  4.567  3.352  4.545  3.639  2.949  2.75  2.32  2.929  1.916  2.437  3.004  2.086  1.518  2.742  2.968  2.291  1.596  2.16  2.502  1.38  2.439  2.66  2.605  1.388  2.06  1.89  2.719  2.422  2.481  3.145  1.61  2.258  
 
 LOC100506142  100506142  2  46795395  46807377  2p21  0.174  0.331  0.195  0  0.255  0.419  0.299  0.196  0.198  0.4  0.4  0  0.413  0.676  0.502  0.124  0  0.097  0.105  0.15  0.158  0.172  0.398  0.123  0.258  1.029  0.969  0.633  1.079  0.916  1.367  0.919  0.581  0.471  0.297  0.463  0.512  0.545  0.292  0.169  0.182  0.436  0.059  0.304  0  0.176  0.232  0.331  0.443  0.27  0.473  0.39  0.484  0.093  0.671  0.274  0.573  0.485  0.23  0.106  
 
 PIGF  5281  2  46808412  46844251  2p21-p16  2.458  3.946  1.025  0.725  2.148  1.219  3.88  2.152  1.909  2.443  2.985  1.688  2.284  4.144  2.276  1.618  2.551  3.494  3.763  2.734  2.146  2.398  1.993  2.167  2.794  2.921  2.235  2.038  2.491  1.809  3.089  2.776  2.811  1.775  1.988  2.805  3.046  2.22  2.298  3.224  2.45  1.298  3.285  2.338  0.617  2.024  2.91  0.968  2.414  3.192  3.021  1.364  2.151  1.136  2.66  2.808  1.719  2.85  1.561  2.879  
 
 CRIPT  9419  2  46844310  46857315  2p21  0.623  1.735  0.662  0.503  1.029  0.74  0.787  0.784  1.079  1.193  1.037  0.706  0.685  1.261  0.639  1.091  1.183  2.345  1.581  1.006  0.616  1.047  0.978  0.635  1.235  1.225  0.594  0.931  0.967  0.235  1.423  1.207  1.436  1.267  1.044  1.005  0.578  0.797  1.668  0.971  0.792  0.88  1.166  0.648  0.723  1.094  0.923  0.386  0.525  1.499  0.991  0.602  0.519  0.6  1.14  1.011  0.805  1.193  0.677  1.215  
 
 SOCS5  9655  2  46926098  46989927  2p21  1.499  2.991  1.513  0.968  0.829  1.158  1.952  1.005  2.005  1.966  1.644  0.521  1.367  2.315  1.362  1.207  1.096  1.424  1.765  1.53  0.733  0.818  0.751  0.705  1.436  1.366  0.925  1.416  1.071  0.796  1.097  1.661  0.998  0.918  1.136  2.352  1.938  1.635  1.578  2.23  1.145  0.821  1.878  1.243  0.945  1.108  1.511  0.933  1.29  2.128  1.48  0.722  1.347  0.798  1.122  1.241  1.546  1.898  0.893  1.445  
 
 LINC01118  388948  2  47043806  47049799  2p21  0  0  0.132  0  0.082  0  0.084  0  0.142  0.185  0  0  0  0.076  0  0.098  0  0  0  0  0  0  0.238  0  0  0  0.075  0  0  0  0  0  0  0.237  0  0.223  0  0  0.205  0  0  0  0  0  0  0  0.042  0.149  0  0.142  0  0.226  0.104  0  0.095  0  0  0  0  0  
 
 LINC01119  100134259  2  47055002  47086145  2p21  0.173  0.576  0.615  0.272  0.126  0.729  1.042  1.21  0.649  2.013  1.136  0  0  0.547  0.12  0.208  0.12  0.09  0.345  0.15  0  0.277  0.399  0  0.305  0  0.065  0.25  0  0.039  0.283  0.33  0  0.451  0.173  1.316  2.107  0.861  0.794  0.24  0.734  0.713  0.583  0  0  0.175  1.084  0.206  0  0.705  0.437  0.242  0.768  0.085  0.548  0.617  0.217  0.571  0.319  0.381  
 
 MCFD2  90411  2  47129008  47168994  2p21  2.588  6.133  2.915  2.204  2.215  2.785  4.387  3.003  4.062  3.02  3.886  2.218  2.831  3.707  3.142  2.897  2.449  3.295  3.281  3.232  3.259  2.242  2.932  2.26  4.322  3.115  2.015  3.264  2.894  2.592  3.638  3.697  3.066  2.379  2.659  3.819  4.323  4.66  4.394  4.003  3.386  2.723  3.446  3.124  1.927  2.515  2.767  2.527  3.132  4.267  3.845  2.055  2.8  2.878  3.602  3.447  3.302  3.82  2.626  3.739  
 
 TTC7A  57217  2  47143267  47303275  2p21  1.315  0.502  0.917  0.706  1.309  0.958  1.852  1.843  1.458  1.596  1.568  1.643  2.321  1.633  2.93  1.771  1.427  0.192  2.994  2.004  1.423  3.343  2.145  2.58  0.955  0.994  1.375  1.505  0.798  0.689  0.828  1.341  2.05  0.638  2.261  2.573  2.151  2.478  1.821  2.079  3.032  1.814  2.017  1.776  1.59  2.158  1.362  1.633  2.344  2.571  1.317  1.586  1.697  2.206  2.442  1.575  1.886  1.933  2.205  1.686  
 
 C2orf61  285051  2  47314129  47382517  2p21  0  0.43  0  0  0  0.276  0.291  0  0.168  0.135  0.086  0  0  0.264  0.366  0.123  0  0.247  0.067  0  0  0.09  0.363  0  0  0  0.159  0  0  0.106  0  0  0  0.301  0  0.199  0  0  0.244  0  0.16  0.075  0  0.165  0  0.115  0.096  0  0  0  0.095  0.219  0  0.088  0  0  0.136  0  0  0  
 
 CALM2  805  2  47387220  47404075  2p21  6.534  10.311  6.85  5.717  6.39  6.752  8.166  6.294  6.354  6.998  7.665  6.333  6.453  7.667  6.443  6.584  7.093  9.038  8.895  6.769  5.557  7.194  5.902  5.356  6.344  6.851  6.136  6.533  6.204  5.089  6.629  7.048  6.564  5.68  6.997  6.922  7.034  7.159  8.311  6.729  7.133  6.971  7.055  6.425  5.444  6.097  7.774  5.938  6.071  8.528  6.655  5.323  6.007  5.575  7.775  6.849  6.407  7.873  6.404  6.972  
 
 LOC101927043  101927043  2  47419543  47572213  2p21  0.15  0.411  0.301  0  0.141  0.193  0.483  0.108  0.351  0.274  0.326  0.196  0.452  0.64  0.255  0.561  0.562  0.263  0.24  0.334  0  0.749  0.269  0.142  0.203  0.081  0.273  0.145  0.38  0.082  0.18  0  0  0.288  0.305  0.381  0.257  0  0.967  0.209  0.381  0.338  0.375  0  0  0.158  1.187  0.145  0  0.247  0.217  0.468  0.233  0.474  1.048  0.593  0.177  0  0  0.168  
 
 EPCAM  4072  2  47596286  47614167  2p21  5.392  2.367  0  0  5.049  0.093  0.078  0  0  0.052  0.051  6.661  6.524  6.498  5.963  6.352  6.688  9.551  1.119  0.209  3.103  0.151  3.336  2.796  0  0.114  0.062  0.071  0.081  0.064  0  0  0  0.194  0.421  2.345  1.12  1.264  0.115  0.926  5.854  0.418  0.81  3.411  4.049  5.103  3.875  1.035  2.98  4.05  3.168  2.825  1.077  0.223  3.808  0.643  0.755  0.157  3.472  2.466  
 
 MSH2  4436  2  47630205  47710367  2p21  2.906  4.885  1.123  2.097  2.73  2.093  2.784  1.949  1.513  2.333  3.013  2.349  3.042  3.79  2.939  3.101  2.651  5.377  5.371  2.988  3.169  2.835  1.803  1.978  2.73  2.602  2.168  2.009  2.342  1.947  3.217  2.689  2.822  1.78  1.928  2.816  3.041  2.179  2.182  3.884  2.634  1.965  4.361  2.731  2.296  2.776  2.28  2.209  2.505  4.595  2.935  1.897  2.448  1.905  2.845  2.445  2.781  3.364  2.185  2.933  
 
 KCNK12  56660  2  47747914  47797470  2p16.3  0.641  0.114  0.213  0  0.201  0.333  0.296  0.143  0.497  0.772  0.131  0.297  0.113  0.119  0  0.196  0.234  0.483  2.142  0.437  0  1.019  2.595  0.866  0  0  0.296  0.103  0.097  0.215  0.117  0  0  0.261  0.143  0.118  0.113  0.162  0.362  0.501  0.092  1.788  0.136  0.156  0  0.186  0.148  0  0  0.178  0.44  2.539  0.058  0.915  0.462  0.342  0.163  0  0  0  
 
 LOC644093  644093  2  47754675  47756303  2p16.3  0  0  0  0  0  0  0  0  0  0  0  0  0  0  0  0  0  0  0  0  0  0  0  0  0  0  0  0  0  0  0  0  0  0  0  0  0  0  0  0  0  0  0  0  0  0  0  0  0  0  0  0  0  0.189  0  0  0  0  0  0  
 
 MSH6  2956  2  48010220  48034092  2p16  3.108  4.94  1.803  2.374  3.048  2.181  3.094  2.531  2.655  2.528  3.334  1.9  3.438  3.106  1.034  2.686  1.757  4.699  4.865  2.93  3.191  3.111  2.114  2.396  2.766  2.919  2.166  3.103  2.872  2.375  2.884  3.148  3.357  2.028  2.815  2.839  3.116  2.449  2.36  4.115  2.874  2.314  3.706  0.903  2.039  2.454  2.172  2.148  2.746  4.177  2.396  1.834  2.617  2.562  3.312  3.043  2.61  3.088  2.578  2.805  
 
 FBXO11  80204  2  48034058  48132932  2p16.3  2.129  3.313  1.386  1.184  1.514  1.581  2.794  2.127  2.066  2.164  2.705  1.255  2.35  3.324  2.069  1.988  2.532  2.427  3.54  2.38  1.698  1.796  1.462  1.426  2.45  2.608  1.947  2.134  2.573  1.978  2.69  2.71  2.416  1.215  1.876  2.9  2.326  2.428  2.135  3.487  2.608  1.196  3.955  2.493  1.436  2.131  2.486  1.487  2.49  2.976  2.647  1.476  1.882  1.522  2.261  2.214  2.385  2.468  1.894  2.545  
 
 FOXN2  3344  2  48541794  48606434  2p22-p16  0.753  1.139  0.564  0.554  0.64  0.489  1.132  0.57  0.658  1.025  0.726  0.651  1.115  1.276  1.306  0.888  1.703  1.182  1.823  1.553  1.339  1.6  0.675  0.819  1.808  1.701  1.007  1.413  1.477  0.478  1.511  1.651  1.701  1.337  0.756  1.256  1.343  0.964  1.165  1.636  0.976  0.717  1.06  2.135  0.597  0.938  0.28  0.853  1.294  1.25  1.052  0.821  0.776  0.861  1.083  0.937  0.944  1.542  0.738  1.057  
 
 PPP1R21  129285  2  48667907  48742531  2p16.3  1.397  1.312  0.656  0.574  1.197  0.466  1.718  0.927  1.081  1.116  1.82  0.519  0.838  1.844  1.225  0.865  1.167  1.397  2.544  1.07  0.563  1.074  0.911  0.54  1.193  1.507  0.964  1.11  1.281  0.642  1.519  0.983  1.213  0.643  0.976  1.77  1.129  1.324  1.571  1.998  1.67  0.717  2.141  1.141  0.642  1.178  0.515  0.457  1.207  1.758  1.766  0.828  0.924  0.69  1.853  0.89  1.387  0.738  1.079  1.66  
 
 STON1-GTF2A1L  286749  2  48757063  49003656  2p16.3  0  0  0  0  0  0.133  0  0.138  0.18  0  0.239  0  0  0  0  0  0  0  0  0  0  0.103  0.105  0  0  0  0.089  0.033  0  0.038  0  0  0  0.106  0  0  0.069  0  0.018  0  0  0.45  0  0  0.153  0.037  0  0  0  0  0  0.114  0  0.031  0  0  0.038  0.226  0  0  
 
 STON1  11037  2  48757307  48825654  2p16.3  0.303  0.077  0.392  0.234  0.099  0.057  0.352  0.824  1.552  1.218  0.575  0  0  0.259  0  0  0  0.028  0.147  0.022  0.045  0.07  0.255  0  0.112  0.678  0.27  0.015  0.104  0.099  0.074  0.145  1.015  0.655  0.067  0.038  0.364  0.047  0.409  0.523  0.25  0.296  0.298  0.036  0.364  0.161  0.022  0.171  0  0.504  0.097  0.128  0.017  0.136  0.052  0.138  0.077  0.54  0.122  0.106  
 
 GTF2A1L  11036  2  48844918  48906751  2p16.3  0  0  0.101  0  0  0  0  0  0.238  0.037  0  0  0  0.064  0  0  0  0  0  0  0  0  0  0  0  0  0  0  0  0.046  0  0  0  0  0  0  0  0  0  0  0  0.046  0  0  0  0  0  0  0  0  0  0  0  0  0  0  0  0  0  0  
 
 LHCGR  3973  2  48913912  48982880  2p21  0  0  0.05  0  0  0  0  0  0.06  0  0  0  0  0.015  0  0.031  0  0  0  0.034  0  0.123  0.107  0  0  0  0.095  0  0  0.048  0  0  0  0.206  0  0  0  0  0.056  0  0  0.052  0.013  0  0.074  0.023  0.023  0  0  0  0  0.309  0  0.024  0.03  0  0  0  0  0  
 
 FSHR  2492  2  49189295  49381666  2p21-p16  0.611  0  0.158  0  0.046  0  0  0  0.079  0.029  0  0  0  0.046  0  0  0  0  0  0  0  0.094  0.22  0  0  0  0.148  0  0  0.071  0  0  0  0.263  0  0  0  0  0.022  0  0  0.421  0.033  0  0  0.038  0.045  0  0  0  0  0.161  0  0.023  0.045  0  0  0  0  0  
 
 NRXN1  9378  2  50145642  51259674  2p16.3  0  0.034  0.133  0  0.051  0.023  0.039  0.04  0.072  0.046  0.031  0.079  0  0.042  0  0.031  0.032  0.165  0.011  0.066  0  0.181  0.273  0.032  0.038  0.016  0.115  0.542  0.015  0.102  0  0  0  0.261  0.043  0  0  0  0.072  0.031  0.024  0.34  0.159  0.022  0.063  0.045  0.057  0  0.025  0.027  0.035  0.718  0.013  0.08  0.053  0.057  0.019  0.056  0  0.026  
 
 ASB3  51130  2  53897116  54014146  2p16.2  1.28  2.205  0.977  0.629  1.113  1.325  2.356  1.381  1.217  1.865  1.974  1.117  1.247  2.096  1.194  1.587  1.593  3.086  3.882  2.21  1.365  2.365  1.355  0.647  1.512  1.756  0.95  1.251  1.295  0.71  1.597  1.955  1.737  1.486  1.13  1.53  1.924  1.605  2.097  1.65  1.611  1.25  2.32  1.336  0.877  0.878  2.313  0.98  1.227  2.736  1.677  1.258  1.089  1.116  1.918  1.383  1.446  2.114  1.007  1.845  
 
 GPR75-ASB3  100302652  2  53897449  54087170  2p16  0.379  0  0.374  0.545  0  0  0.52  0.216  0.978  0.056  0  0  0.269  0.591  0.973  0  0.153  0.207  1.713  0  0  0.411  0.316  0.259  0  0.102  0.654  0.571  0.22  0.562  0.727  0.438  0.718  0.221  0  0.786  0.252  0.141  0  1.018  0.4  0.535  0  0.112  0.178  0  0  0.502  0  1.188  0.181  0  0.416  0  0.059  0  0.366  0  0  0  
 
 CHAC2  494143  2  53994928  54002287  2p16  1.509  3.85  0.619  0.935  1.388  1.542  1.611  0.698  0.547  1  2.531  0.855  2.187  2.756  1.258  1.498  1.797  3.195  3.813  2.919  2.01  2.002  1.673  1.644  1.582  1.438  0.373  0.998  1.086  0.863  1.806  1.935  1.407  1.132  1.646  2.083  1.412  1.197  2.239  1.602  1.672  1.735  2.915  1.801  0.917  1.318  1.308  0.854  1.487  2.91  1.89  0.776  0.725  0.504  1.758  1.079  0.939  1.812  1.25  1.667  
 
 ERLEC1  27248  2  54014067  54045956  2p16.2  2.357  4.514  2.248  1.87  3  2.181  3.709  3.008  2.973  2.916  3.319  2.559  2.547  3.357  1.948  2.22  2.62  3.803  3.691  2.961  1.652  1.933  2.622  2.463  2.911  3.271  1.681  2.684  2.787  2.223  3.087  3.704  2.881  2.396  2.374  3.679  2.458  2.997  3.711  3.032  3.341  2.444  2.932  1.946  1.951  1.495  2.731  1.34  2.024  3.198  2.781  1.518  1.922  2.295  2.844  2.843  2.567  2.825  1.643  3.039  
 
 GPR75  10936  2  54080049  54087170  2p16  0.637  0.969  0.686  0  0.771  0.645  0.94  1.038  0.139  0.856  0.913  0.355  0.644  0.723  1.016  0.743  0.203  0.372  0.379  0.549  0.606  0.945  0.339  0.287  0.494  0.717  0.563  0.661  0.626  0.214  0.611  0.653  0.876  0.52  0.794  0.577  1.188  1.29  0.952  1.096  0.563  0.426  1.116  0.659  0.521  0.454  0.213  0.783  0.655  0.102  0.808  1.063  0.655  0.456  0.865  0.264  0.759  0.569  0.57  0.194  
 
 PSME4  23198  2  54091203  54197977  2p16.2  2.132  3.367  1.983  1.049  2.326  1.711  2.932  2.077  1.612  1.923  3.113  1.6  2.708  3.007  2.113  2.058  1.931  2.36  3.012  2.019  2.552  1.85  1.596  1.748  2.173  2.153  1.876  2.565  2.092  1.619  2.639  2.783  2.487  1.178  1.752  2.529  2.878  2.308  2.174  2.938  2.657  1.498  3.183  2.388  2.2  1.95  2.392  1.686  2.179  2.485  2.673  1.49  1.941  1.471  1.806  1.716  2.243  2.29  1.598  2.249  
 
 ACYP2  98  2  54342409  54532435  2p16.2  0.633  3.521  0.895  0.262  1.243  1.225  1.439  0.865  1.755  1.322  1.579  0.67  1.013  2.234  0.814  0.993  1.741  3.658  2.738  1.753  0.748  2.231  1.213  0.96  0.696  0.938  0.782  0.667  0.821  0.274  1.279  1.187  1.081  1.017  1.022  1.668  0.895  0.959  2.28  1.17  1.427  1.874  0.857  0.441  0.458  0.985  1.4  0.495  0.814  1.889  1.472  0.868  0.394  0.941  0.99  0.912  0.746  2.075  0.252  0.712  
 
 TSPYL6  388951  2  54480314  54483409  2p16.2  0  0  0.153  0  0.037  0  0  0.06  0.096  0.073  0.051  0.036  0.117  0.146  0.111  0.032  0  0.051  0.042  0  1.199  0.153  0.292  0  0  0  0.152  0.057  0.077  0.13  0.051  0  0.074  0.273  0  0  0.086  0  0.061  0  0  0.111  0.049  0  0.069  0.086  0.445  0  0.055  0  0.077  0.3  0  0.038  0.047  0.065  0.086  0.137  0.048  0  
 
 C2orf73  129852  2  54558070  54588714  2p16.2  0  0  0.123  0  0  0.067  0.057  0  0.121  0  0.044  0  0  0.088  0  0  0  0.115  0  0  0  0.022  0.169  0  0  0  0.096  0  0  0.079  0  0  0  0.209  0  0  0  0  0  0  0  0.052  0  0  0  0.059  0.198  0  0  0  0  0.138  0  0  0.047  0  0  0  0  0  
 
 SPTBN1  6711  2  54683453  54898583  2p21  2.943  4.732  4.23  2.474  2.471  3.867  4.082  3.256  3.355  3.887  3.978  4.396  4.167  3.688  4.593  3.726  4.74  3.324  5.108  3.009  2.82  4.899  1.931  2.574  2.283  4.762  4.873  5.014  5.685  4.747  4.765  5.055  4.723  2.885  4.011  5.101  4.658  4.827  3.874  4.907  4.85  3.227  4.766  4.31  3.453  3.614  5.144  3.889  4.739  3.613  4.813  3.857  4.682  4.494  4.957  4.6  5.285  4.048  4.667  4.956  
 
 RPL23AP32  56969  2  54756358  54756978  2p16.2  0  2.505  0.807  0  0.276  0.32  1.435  0.7  0.647  0.886  1.039  0  0  1.624  0.644  0.687  0.473  1.922  0.667  0.939  0  1.471  0.756  0  0.446  1.647  2.4  0.895  1.369  1.174  2.254  1.968  1.022  0.745  1.162  0.526  0.772  1.128  1.385  0  0  1.011  2.128  0.534  0.287  0  3.832  0.86  0.398  1.044  0.511  0.411  0.6  0.23  1  1.048  1.234  0.538  0.869  0.284  
 
 EML6  400954  2  54952148  55199156  2p16.1  0.403  0.359  0.16  0.094  0.12  0.187  0.202  0.126  0.142  0.237  0.252  0.076  0.167  0.678  0.246  0.118  0.309  0.215  0.664  0.113  0.055  0.667  0.361  0.243  0.188  0.087  0.133  0.236  0.067  0.132  0.119  0.141  0.363  0.223  0.117  0.195  0.204  0.281  0.149  0.171  0.188  0.172  0.244  0.073  0.049  0.11  0.143  0.128  0.086  0.117  0.491  0.35  0.143  0.089  0.161  0.083  0.245  0.534  0.205  0.032  
 
 RTN4  57142  2  55199326  55277734  2p16.3  4.514  8.345  6.349  5.486  5.112  5.602  7.233  5.374  6.446  6.632  6.852  4.688  5.792  6.404  4.35  5.645  5.333  6.631  5.874  5.428  4.878  5.559  4.617  4.127  7.355  5.393  5.541  5.31  5.668  4.68  5.729  7.132  5.162  4.793  5.29  5.471  6.545  6.178  7.795  5.689  5.606  5.651  6.646  4.46  4.436  4.193  6.81  4.938  5.925  7.293  6.45  4.573  6.452  5.496  7.1  6.485  6.29  6.668  6.078  6.938  
 
 CLHC1  130162  2  55399686  55459699  2p16.1  0.225  0.857  0.189  0.092  0.281  0.068  0.35  0.221  0.263  0.249  0.282  0.061  0.309  0.483  0.164  0.166  0.185  0.672  1.17  0.099  0.218  0.421  0.284  0  0  0.514  0.135  0.081  0.089  0.057  0.247  0.165  0.059  0.188  0.111  0.107  0.274  0.244  0.419  0.077  0.339  0.194  0.401  0.09  0.183  0.065  0.475  0.127  0.061  0.606  0.122  0.131  0.245  0.128  0.458  0.099  0.212  0.046  0.222  0.165  
 
 RPS27A  6233  2  55459038  55462989  2p16  7.765  11.004  6.718  5.943  8.298  8.176  8.495  7.166  7.002  7.293  8  7.716  7.38  9.054  6.965  8.265  9.111  12.336  10.155  10.516  8.184  9.223  7.864  7.939  8.86  6.843  6.554  6.824  6.943  6.634  8.335  8.488  7.435  6.949  8.614  7.863  7.502  7.741  9.555  7.485  8.234  7.872  8.646  8.317  7.637  7.34  8.415  7.167  7.415  9.95  7.454  7.079  7.615  7.347  9.172  8.231  7.16  9.369  7.967  7.879  
 
 MTIF2  4528  2  55463755  55496384  2p16.1  2.638  4.427  1.213  1.375  1.936  1.215  2.303  1.419  1.68  1.616  2.601  2.199  2.76  3.452  2.528  2.068  2.714  4.273  3.541  2.508  3.173  1.841  1.915  1.821  2.516  2.735  1.832  2.571  2.438  2.275  3.407  3.305  2.537  1.648  2.073  2.805  2.459  2.314  2.222  3.125  2.344  1.915  2.996  2.244  1.587  1.868  1.892  1.27  2.285  3.145  2.709  1.43  2.137  1.804  2.841  2.005  2.184  2.385  1.803  2.553  
 
 PRORSD1P  344405  2  55509454  55511607  2p16.1  0.747  0.552  0.199  0  0.043  0.17  0.262  0.224  0.189  0.228  0.271  0.207  0.471  0.294  0.333  0.39  0.192  1.291  0.863  0.812  0.563  0.383  0.731  0.181  0.142  0.405  0.298  0.385  0.628  0.431  0.459  0.375  0.366  0.299  0.437  0.431  0.158  0.477  0.347  0.373  0.163  0.314  0.198  0.305  0  0.187  0.482  0.259  0.355  0.42  0.292  0.657  0.203  0.225  0.737  0.212  0.352  0.089  0.457  0.175  
 
 CCDC88A  55704  2  55514977  55647057  2p16.1  0.17  3.307  1.741  1.085  0.138  1.486  2.205  1.982  1.248  2.044  2.281  0.371  0.173  1.634  0.24  0.059  2.237  2.821  2.99  1.877  1.911  1.883  0.237  0.248  1.909  2.621  0.989  1.516  2.191  2.063  2.506  2.161  1.871  1.164  1.429  2.52  1.703  1.849  1.407  2.79  0.89  1.441  2.655  1.965  0.617  0.727  0.965  1.585  1.951  2.271  2.791  1.608  1.68  1.021  2.108  1.69  2.131  2.085  1.628  2.024  
 
 CFAP36  112942  2  55746730  55772216  2p16.1  2.654  4.983  1.777  1.642  3.424  2.065  3.79  1.967  2.751  2.368  3.141  2.155  1.521  4.006  2.027  2.437  3.289  5.853  3.771  2.294  1.998  2.386  0.969  0.94  2.363  2.258  1.985  1.979  2.504  1.132  2.886  3.283  2.326  1.604  2.712  2.564  2.302  1.882  3.54  3.069  2.683  1.74  3.24  2.401  2.017  1.75  1.975  2.097  2.311  4.185  2.896  1.505  2.001  2.094  3.21  2.644  1.739  3.168  1.679  2.26  
 
 PPP4R3B  57223  2  55774427  55844860  2p16.1  1.919  2.777  1.428  1.206  1.592  1.252  1.69  1.579  2.343  1.78  2.23  1.743  2.141  2.176  1.9  1.764  1.837  2.498  2.876  1.942  2.055  1.744  1.728  1.179  1.956  1.915  0.895  1.367  1.649  1.034  2.008  1.787  1.721  1.533  1.498  2.226  1.863  1.678  2.389  2.344  2.35  1.648  1.92  1.633  1.352  1.244  1.157  1.09  1.932  1.941  1.919  1.308  1.445  1.651  1.985  1.744  1.926  2.068  1.396  1.784  
 
 PNPT1  87178  2  55861197  55921045  2p15  1.687  3.493  0.845  2.164  1.659  1.545  2.014  1.232  1.314  1.395  2.187  1.461  1.295  2.177  1.892  3.71  1.853  4.039  3.492  3.115  2.45  2.152  1.424  2.037  2.757  2.406  1.114  1.571  1.533  1.078  2.502  2.425  2.009  1.038  1.601  2.364  2.021  2.017  2.864  3.011  1.764  1.601  2.134  1.913  1.382  1.111  1.63  1.452  1.578  3.152  2.209  1.527  1.443  1.514  2.487  1.453  1.764  1.972  1.581  2.272  
 
 EFEMP1  2202  2  56093096  56151298  2p16  4.596  4.301  3.709  2.15  4.518  2.062  0.419  2.396  3.831  7.441  6.392  0  0  0.026  0  0  0  0.143  0.061  0.331  0  1.261  0.209  0  0  0  0.055  0.028  0.134  0.057  0  0.057  0  0.271  0.31  4.549  3.869  4.241  6.515  3.248  3.952  4.506  0.035  0.066  0.819  0.768  0.036  1.655  0.325  3.272  0.584  2.916  1.931  2.411  0.094  2.097  2.729  6.22  0  6.842  
 
 MIR217HG  104355290  2  56190540  56274461  2p16.1  0  0  0  0  0  0  0  0  0  0.109  0.229  0  0  0  0  0  0  0  0  0  0  0.072  0  0  0  0  0.088  0  0  0.061  0  0  0  0.276  0  0  0  0  0.106  0  0  0  0  0  0  0  0  0  0  0  0  0  0  0  0  0  0  0  0  0  
 
 LOC100129434  100129434  2  56400668  56412905  2p16.1  0  0.027  0.228  0  0.594  0  0  0  0.083  0.024  0.158  0  0.146  0  0  0  0  0  0  0  0.111  0.035  0.233  0  0  0  0.022  0.025  0  0.016  0  0  0  0.273  0  0.438  0  0  0.071  0  0  0.13  0.106  0  0  0  0  0  0  0  0.102  0.158  0  0  0  0  0  0  0  0  
 
 CCDC85A  114800  2  56411257  56613309  2p16.1  0  0.027  0.547  0  0.459  0.545  0.044  0.026  0.105  0.071  0.272  0.079  0.156  0.118  0  0.038  0.074  0.145  0  0.067  0  0.088  0.219  0  0.151  0  0.109  0.017  0  0.083  0  0  0  0.182  0.06  0.859  0.048  0  0.494  0  0.04  0.302  0.182  0  0.071  0.048  0.083  0.22  0  0.352  0.21  0.386  0  0.094  0.044  0.117  0  0.355  0  0  
 
 VRK2  7444  2  58134785  58387055  2p16.1  1.448  3.43  1.358  0.914  1.718  0.594  0.193  0  0.227  0.123  0.178  1.133  1.752  3.272  1.829  2.279  2.665  3.485  2.498  1.342  2.147  0.335  1.321  1.194  2.304  1.988  1.959  1.45  1.689  1.088  2.221  2.634  1.901  1.607  1.973  2.543  1.75  1.957  1.962  2.9  2.307  1.096  2.524  2.006  1.118  1.089  1.334  1.04  2.013  2.265  1.266  1.658  1.568  1.643  2.66  1.696  2.145  2.122  1.747  2.764  
 
 FANCL  55120  2  58386377  58468515  2p16.1  1.808  3.386  0.511  0.779  1.562  1.729  2.311  1.455  1.278  1.968  2.329  1.816  1.809  3.144  1.687  3.01  2.726  4.981  4.036  2.205  2.062  1.549  0.853  0.99  2.055  2.064  1.506  1.222  2.139  1.405  2.38  2.602  2.368  1.506  1.62  2.078  1.79  1.419  2.167  2.768  2.322  1.603  2.869  1.216  1.32  1.076  1.831  1.223  1.916  3.68  0.979  1.234  1.134  1.353  2.5  1.865  1.252  1.775  1.588  1.689  
 
 LINC01122  400955  2  58747887  59290901  2p16.1  0  0.022  0.113  0  0  0  0.031  0  0.078  0  0  0  0  0  0  0  0  0.046  0.02  0  0  0.029  0.247  0  0  0  0.05  0  0  0.057  0  0  0  0.179  0  0  0  0  0.022  0.059  0  0.13  0.011  0  0.077  0  0.075  0  0  0.025  0  0.074  0  0.032  0  0  0  0  0  0  
 
 LOC101927285  101927285  2  59444842  59506535  2p16.1  0  0  0  0  0  0  0  0  0  0  0  0  0  0  0  0  0  0  0  0  0  0.047  0.185  0  0  0  0.02  0  0  0.032  0  0  0  0.166  0  0  0  0  0.022  0  0  0  0.026  0  0  0  0.02  0  0  0  0  0  0  0.027  0  0  0  0  0  0  
 
 BCL11A  53335  2  60678301  60780633  2p16.1  0  0.029  0.216  0  0.053  2.103  0.123  0.045  0.455  0.139  0.087  1.162  1.589  0.127  0.932  0.068  1.542  1.794  4.373  3.132  0  3.705  1.6  1.705  2  0.112  0.226  0.067  0.064  0.798  0.286  0.46  0  0.311  0  0.274  0  0.092  0.105  2.725  0.925  0.782  0.254  0  0.433  0.079  0.278  1.473  0.786  2.919  0.111  0.793  0.034  0.178  0.08  0.076  0.042  0.127  0  0  
 
 PAPOLG  64895  2  60983364  61029221  2p16.1  0.6  0.642  0.43  0.348  0.443  0.24  0.704  0.727  0.673  0.588  0.928  0.622  0.693  1.051  0.776  0.709  0.946  1.007  1.574  0.928  0.784  1.024  0.532  0.386  1.068  0.8  0.407  0.49  0.652  0.339  0.704  0.616  0.589  0.503  0.557  0.941  0.717  0.578  0.81  1.457  0.729  0.335  1.175  0.709  0.553  0.355  0.265  0.504  0.703  1.149  0.85  0.516  0.453  0.428  0.797  0.559  0.599  0.471  0.512  0.985  
 
 LINC01185  400957  2  61074894  61108449  2p16.1  0  0  0.123  0  0  0  0.065  0  0.097  0  0  0  0  0.03  0  0.05  0  0  0  0  0  0.079  0.296  0  0  0  0.03  0  0  0.046  0  0  0  0.158  0  0  0.068  0  0.026  0  0  0.192  0.035  0  0  0  0.084  0  0  0.089  0.069  0.285  0  0.038  0  0  0  0  0  0  
 
 REL  5966  2  61108629  61155291  2p13-p12  2.566  1.417  0.627  0.695  1.296  0.56  1.31  1.212  0.825  0.746  0.845  1.157  1.733  1.405  1.193  1.369  1.493  1.204  1.294  1.812  1.054  2.055  0.487  0.598  1.065  1.033  0.892  0.825  1.024  0.874  1.182  0.884  0.814  0.636  1.191  1.455  0.953  0.961  1.106  3.027  1.679  0.616  1.572  1.554  1.031  0.944  0.869  0.611  1.462  0.759  0.593  0.976  1.029  0.879  1.233  0.742  1.229  1.103  1.206  1.076  
 
 PUS10  150962  2  61167547  61245365  2p16.1  0.365  0.262  0.304  0.151  0.236  0.104  0.322  0.181  0.186  0.207  0.367  0.136  0.336  0.38  0.242  0.216  0.233  0.318  0.966  0.574  0.481  0.685  0.139  0.105  0.284  0.296  0.179  0.165  0.278  0.196  0.275  0.332  0.207  0.193  0.181  0.525  0.257  0.188  0.546  0.564  0.351  0.079  0.195  0.262  0.146  0.19  0.376  0.159  0.112  0.41  0.304  0.31  0.177  0.29  0.384  0.255  0.305  0.224  0.213  0.429  
 
 PEX13  5194  2  61244811  61279125  2p16.1  1.537  1.713  0.85  0.743  1.51  0.697  1.221  1.196  0.876  0.963  1.384  1.342  1.505  1.676  1.174  1.269  1.303  1.601  1.517  0.906  0.761  0.903  0.793  0.5  1.147  0.962  0.759  0.649  1.001  0.467  0.866  1.17  0.728  0.693  0.865  1.67  0.975  0.95  1.599  1.933  1.913  0.669  1.806  1.385  0.778  0.907  0.648  0.716  1.302  1.577  1.402  0.759  0.831  1.049  1.209  1.082  1.044  1.195  0.713  1.43  
 
 KIAA1841  84542  2  61293005  61365169  2q14  0.431  1.324  0.398  0.43  0.396  0.397  0.515  0.609  0.314  0.78  0.935  0.24  0.651  0.617  0.532  0.491  0.236  0.902  0.769  0.191  0.255  0.327  0.213  0.192  0.375  0.631  0.512  0.27  0.578  0.572  0.624  0.437  0.459  0.409  0.554  0.894  0.361  0.52  0.581  0.841  0.537  0.345  1.283  0.159  0.32  0.182  0.114  0.244  1.231  0.616  0.714  0.401  0.512  0.491  0.831  0.36  0.931  0.615  0.737  0.735  
 
 LOC339803  339803  2  61368726  61372110  2p15  0.284  0.535  0.829  0  2.315  1.233  0.489  0.935  0.915  1.239  1.533  0.084  0  0.029  0  0.033  0  0.05  1.239  0.996  0  0.105  0.699  0.602  1.377  0.9  0.684  0.748  1.042  0.596  1.499  1.335  1.006  1.284  2.022  1.526  0.972  0.585  1.456  2.205  1.081  1.083  0.876  0  0.503  0.394  2.398  0.967  0.679  2.117  1.487  1.231  1.185  0.988  2.386  1.343  1.014  1.689  1.051  1.495  
 
 C2orf74  339804  2  61372242  61391964  2p15  2.082  3.936  1.559  0  2.102  1.219  3.07  2.111  2.181  2.275  3.193  0  0  0.111  0  0  0  0.199  3.542  0.841  0  0.202  2.345  1.35  2.601  2.26  1.266  1.451  1.814  1.552  2.635  2.029  1.098  1.073  1.776  2.794  2.321  1.077  2.168  2.793  2.597  2.372  2.584  0  1.704  0.774  1.902  1.49  2.203  3.517  1.94  1.366  2.643  2.633  2.834  1.417  2.382  3.014  2.108  2.665  
 
 AHSA2  130872  2  61404554  61414686  2p15  1.876  1.722  0.643  0.784  1.939  0.61  1.213  0.963  1.092  1.728  1.366  0.503  1.752  1.889  2.145  2.107  1.222  4.112  4.097  1.463  1.159  2.185  1.412  1.071  0.967  1.568  2.015  1.296  1.691  1.31  2.361  1.476  1.793  1.011  1.737  2.376  1.5  1.647  2.628  1.512  1.814  1.587  2.951  1.103  0.55  1.772  1.811  1.462  1.298  2.72  1.395  1.379  1.271  1.082  2.939  1.233  1.471  1.442  1.616  1.008  
 
 USP34  9736  2  61414589  61697849  2p15  2.303  2.635  1.274  0.823  1.736  1.208  2.071  1.952  1.746  1.937  2.488  1.306  2.032  2.871  2.152  1.972  1.891  2.058  2.742  1.809  1.965  2.055  1.08  1.122  2.234  2.083  1.521  2.138  2.131  1.375  2.161  2.484  2.233  1.03  1.783  2.904  2.301  2.001  2.02  3.271  2.445  1.193  2.813  2.2  1.577  2.008  1.69  1.564  2.688  2.425  2.302  1.763  1.774  1.175  2.409  2.094  2.609  2.236  1.86  2.336  
 
 SNORA70B  100124537  2  61644378  61644512  2p15  0  0  0  0  0  0  0  0  0  0  0  0  0  0  0  0  0  0  0  0  0  1.331  0  0  0  0  0  0  0  0  0  0  0  0  2.304  0  0  0  0  0  0  0  0  0  0  0  0  0  0  0  0  0  0  0  0  0  0  0  0  0  
 
 XPO1  7514  2  61705068  61765418  2p15  3.352  5.129  2.585  2.82  3.075  3.166  3.488  2.924  2.675  3.348  4.381  2.618  3.748  4.244  3.986  4.026  3.833  5.567  5.632  3.486  4.098  3.733  2.441  2.618  3.861  3.776  2.653  3.123  3.667  2.725  4.182  3.85  3.478  2.312  3.259  3.939  3.946  3.446  4.002  4.458  3.501  3.236  3.996  3.94  3.271  3.164  2.815  2.757  3.544  4.965  4.004  2.703  3.257  2.952  3.598  3.233  3.49  3.832  3.349  4.011  
 
 FAM161A  84140  2  62051982  62081278  2p15  0.717  2.078  0.425  1.001  0.404  0.46  1.037  0.494  0.803  0.574  0.912  0.221  0.729  1.486  0.826  0.373  0.924  1.709  1.359  0.729  0.161  0.918  0.805  0.484  0.73  1.203  0.506  0.801  1.18  1.392  1.081  0.774  0.667  0.731  1.159  0.548  0.547  0.654  0.468  1.614  0.706  0.83  1.407  0.898  0.272  0.439  1.08  0.447  0.603  1.104  0.8  0.747  0.612  0.42  1.191  0.645  0.808  1.438  0.661  0.895  
 
 CCT4  10575  2  62095261  62115806  2p15  4.628  7.127  3.83  4.175  4.931  3.705  5.597  4.433  4.469  4.553  5.395  3.919  4.981  6.654  4.621  4.971  5.769  6.971  7.08  5.967  5.198  5.56  4.439  3.963  5.711  4.575  3.988  4.294  4.783  4.492  5.51  5.465  4.701  3.781  4.75  5.072  5.242  4.517  5.895  6.285  4.922  5.013  5.774  5.149  4.303  4.552  4.798  4.178  4.316  6.922  5.132  3.933  4.49  4.416  5.521  4.986  4.68  4.975  4.477  5.432  
 
 COMMD1  150684  2  62132802  62363205  2p15  3.505  5.84  3.336  2.37  4.399  3.776  4.515  3.07  3.298  3.587  3.455  2.603  2.327  3.731  2.339  1.931  2.375  5.722  5.554  4.921  2.691  4.122  3.582  3.273  4.355  3.554  2.459  2.23  3.42  2.551  4.153  3.938  2.868  2.667  3.598  3.927  2.604  3.195  5.243  3.717  4.029  3.364  3.873  3.348  2.932  3.822  4.205  2.275  3.519  4.591  3.765  2.122  2.99  2.637  4.469  2.791  2.965  4.364  2.945  3.887  
 
 B3GNT2  10678  2  62423261  62451866  2p15  1.453  1.799  1.051  1.241  1.046  0.563  1.084  1.235  2.319  1.196  1.783  1.468  2.123  2.203  1.768  1.895  2.706  1.637  3.576  1.336  1.361  2.465  0.837  0.92  2.197  2.064  1.112  1.498  1.734  0.781  1.323  1.796  1.376  1.235  1.126  1.859  1.205  1.248  2.084  1.256  1.853  0.917  0.871  1.281  0.902  1.449  0.8  0.98  1.275  2.066  1.242  1.143  1.133  1.198  1.613  0.961  1.76  0.968  1.353  1.696  
 
 TMEM17  200728  2  62727355  62733604  2p15  0.236  0.778  0.57  0.259  0.691  0.348  0.838  0.769  0.713  0.879  0.794  0.531  0.698  0.87  0.334  0.425  0.721  0.829  0.658  0.502  0.27  0.187  0.178  0.345  0.837  1.083  1.373  0.859  0.927  0.378  1.536  1.437  1.37  1.274  0.721  1.346  0.884  0.863  0.694  0.765  1.073  0.626  1.225  0.509  0.33  0.391  2.124  0.201  0.49  0.743  0.704  0.904  0.489  0.493  0.884  0.963  0.672  0.891  0.174  0.553  
 
 EHBP1  23301  2  62900985  63273621  2p15  1.582  3.802  1.619  1.481  1.289  1.328  2.077  2.149  1.487  2.806  2.626  0.948  2.296  3.173  1.549  1.962  2.282  3.344  2.189  1.551  0.957  1.38  0.624  0.86  1.731  2.967  1.75  2.091  2.122  0.797  2.079  2.984  2.438  1.96  2.304  3.41  1.756  2.096  1.792  2.001  2.343  1.226  1.809  1.547  0.9  1.275  3.299  1.106  1.778  2.494  2.653  1.316  1.511  1.949  2.021  1.764  2.276  2.242  1.553  1.77  
 
 LOC100132215  100132215  2  63271099  63275656  2p15  0.288  0.433  0.376  1.131  0.187  0.111  0.503  0.267  0.317  0.582  0.269  0.207  0.172  0.526  0.226  0.521  0.191  0.119  0.139  0.18  0  0.356  0.484  0  0  0.468  0.482  0.226  0.287  0.125  0.218  0.543  0.275  0.458  0.557  0.548  0.152  0.242  0.304  0.115  0.275  0.393  0.163  0.138  0.097  0.116  0.402  0.115  0.138  0.198  0.334  0.438  0.187  0.379  0.455  0.219  0.36  0.487  0.109  0  
 
 OTX1  5013  2  63277191  63284966  2p13  0.82  0.466  0.51  3.05  1.106  0.396  0.321  0.868  0.661  0.709  0.822  0.89  1.185  2.007  1.552  1.958  1.475  0.736  1.077  0.879  1.102  1.811  0.671  0.659  0.994  0.824  1.367  0.528  1.626  1.692  1.906  0.912  1.437  0.668  1.182  1.965  0.639  0.652  0.634  1.511  1.787  1.183  1.309  1.412  0.707  1.077  1.005  1.146  0.956  1.243  0.701  1.45  1.327  0.954  1.705  1.108  0.593  1.444  1.207  0.914  
 
 DBIL5P2  100169989  2  63344985  63346677  2p15  0  0  0.182  0  0  0  0  0  0.125  0.077  0  0  0  0.124  0.12  0.052  0  0  0.18  0.133  0  0.068  0.438  0  0  0.088  0.343  0.059  0  0.35  0.08  0  0  0.285  0.087  0.067  0  0  0  0  0  0  0  0  0  0.213  0  0.252  0.187  0.065  0  0  0.048  0  0.135  0  0.162  0  0  0  
 
 WDPCP  51057  2  63348534  63815867  2p15  0.965  0.705  0.478  0.287  0.755  0.336  0.514  0.588  0.584  0.738  0.521  0.346  0.444  0.805  0.632  0.575  0.58  0.927  1.651  0.737  0.319  0.912  0.277  0.208  0.62  1.139  0.772  0.846  1.013  0.569  1.049  0.94  1.017  0.61  0.578  0.79  0.468  0.337  0.721  0.928  0.697  0.777  0.628  0.354  0.628  0.53  0.504  0.461  0.744  0.844  0.661  0.61  0.588  0.409  1.255  0.306  0.803  0.839  0.528  0.797  
 
 MDH1  4190  2  63815742  63834330  2p13.3  4.575  6.157  3.521  3.405  5.134  3.657  5.511  4.527  3.736  4.576  5.452  4.582  4.769  6.215  4.355  4.362  5.753  7.125  7.098  5.812  4.54  4.823  3.951  3.861  4.926  5.557  4.489  4.684  5.441  5.443  6.513  6.04  5.288  4.775  4.23  4.942  4.251  4.275  5.493  5.469  5.074  4.238  5.507  4.649  3.986  4.844  3.738  3.979  4.342  6.664  5.091  3.989  4.072  4.028  5.87  4.537  4.389  5.186  4.076  5.159  
 
 UGP2  7360  2  64068097  64118696  2p14-p13  2.808  5.649  3.468  2.282  3.183  2.107  3.438  2.947  2.945  3.603  3.722  3.278  3.603  4.055  2.736  3.282  3.326  5.367  5.464  3.65  2.498  2.861  2.254  2.023  3.486  3.546  2.868  3.164  3.74  1.958  3.628  4.58  3.21  2.979  2.841  3.781  2.87  3.428  3.583  3.57  3.471  2.738  4.171  2.382  2.357  3.547  2.734  2.26  3.141  4.818  3.645  2.059  2.832  3.115  3.502  3.24  3.278  3.227  3.068  3.64  
 
 VPS54  51542  2  64119666  64246214  2p13-p14  0.499  2.224  0.734  0.542  1.4  0.876  1.148  1.286  1.478  1.27  1.828  1.023  1.672  1.592  1.468  1.234  1.45  1.816  2.264  0.78  0.908  1.064  0.597  0.515  1.479  1.714  0.731  1.06  1.18  0.749  1.852  1.489  1.131  0.663  0.988  1.75  1.325  1.592  1.472  1.753  1.613  0.926  1.251  1.452  0.79  1.06  0.66  0.603  1.392  2.121  1.483  0.718  1.041  0.944  1.778  1.349  1.372  1.515  1.09  1.606  
 
 PELI1  57162  2  64319785  64371605  2p13.3  0.245  1.336  0.674  0.809  0.651  0.456  0.76  0.867  2.286  1.167  1.688  1.296  2.532  1.064  2.296  2.856  3.341  2.568  1.095  0.855  0.713  1.245  2.215  1.418  0.986  1.281  1.298  1.312  0.909  0.217  1.277  1.774  1.365  1.085  0.171  0.731  0.397  0.604  0.526  0.409  0.902  0.517  0.788  0.999  2.94  2.094  0.462  0.796  0.811  2.139  0.801  0.764  0.683  0.967  2.227  1.587  1.233  0.821  1.038  1.338  
 
 LINC00309  150992  2  64412212  64432619  2p15  0  0  0.055  0  0  0  0.074  0  0.07  0.03  0  0.039  0  0.015  0  0.043  0  0  0  0  0  0.033  0.231  0  0  0  0.043  0  0  0.042  0  0  0  0.256  0  0  0  0  0.02  0  0  0.048  0.029  0  0  0  0.029  0  0  0  0.022  0.153  0  0.023  0  0  0  0  0  0  
 
 LOC100507006  100507006  2  64455534  64479665  -  0  0.266  0  0  0  0  0  0  0.409  0.188  0.255  0  0  0.117  0  0.592  0  0.543  0  0  0  0.397  0.753  0.293  0  0.303  0.071  0.294  0  0.293  0.333  0.439  0  0  0  0  0  0  0  0  0  0  0.076  0.309  0  0  0  0  0  0.447  0  0.435  0  0  0.859  0  0  0  0  0  
 
 LGALSL  29094  2  64681326  64688517  2p14  0.704  1.352  0.281  0.287  1.13  0.663  0.593  0.696  1.429  0.451  1.059  1.178  1.709  1.824  1.367  0.599  1.01  1.456  0.057  0.154  0  0.398  0.61  0.452  0.043  0.924  0.813  0.454  0.605  0.838  1.24  1.401  1.208  0.864  0.763  1.433  0.293  0.519  1.325  0.796  1.25  1.138  1.767  1.135  0.918  1.354  1.181  0.656  1.296  2.18  1.086  0.832  0.263  0.579  1.399  0.945  0.403  1.368  0.494  0.605  
 
 AFTPH  54812  2  64751438  64820138  2p14  1.948  3.226  1.221  0.967  2.224  1.366  1.962  1.464  1.963  1.533  1.898  1.951  2.552  2.15  1.951  2.504  2.309  3.21  2.878  2.781  1.241  1.98  1.778  1.368  1.482  1.801  1.301  1.46  1.595  1.01  1.971  1.512  1.935  1.708  1.644  2.162  1.408  1.472  2.633  2.088  2.352  1.337  1.898  1.756  1.37  1.445  1.8  1.122  1.968  2.435  1.865  1.264  1.106  1.32  3  2.074  1.72  1.345  1.457  1.877  
 
 LOC339807  339807  2  64834445  64843616  2p14  0  0  0  0  0  0  0.046  0  0.119  0  0  0  0  0.055  0  0  0  0  0  0  0  0  0.387  0  0  0  0.072  0  0  0.039  0  0  0  0.461  0  0.089  0  0  0.037  0  0.097  0.275  0.026  0  0  0  0.058  0  0  0  0  0.159  0  0  0.057  0  0  0  0  0  
 
 SERTAD2  9792  2  64858754  64881046  2p14  2.117  4.99  2.972  1.582  1.834  2.805  2.748  3.252  1.791  2.052  2.111  1.637  2.423  4.035  2.5  2.354  3.21  2.079  2.524  2.916  1.783  2.728  1.531  1.52  2.481  2.954  2.377  2.611  2.205  2.385  2.16  2.494  2.738  1.89  2.145  2.055  2.482  3.162  2.701  2.633  2.992  1.749  5.366  1.911  2.194  2.436  3.79  2.575  3.203  3.105  3.92  2.342  2.934  2.338  4.171  2.651  3.221  3.087  2.887  2.252  
 
 LOC101927438  101927438  2  65073263  65090765  2p14  0  0  0.122  0  0  0  0  0  0.1  0  0  0  0  0  0  0  0  0.076  0  0.174  0  0.039  0.358  0  0  0  0.04  0  0  0.055  0  0  0  0.302  0  0  0  0  0.037  0  0  0  0.05  0  0  0  0.579  0  0  0  0  0.102  0  0  0  0  0  0  0  0  
 
 LOC400958  400958  2  65128973  65159581  2p14  0  0  0.099  0  0  0  0  0  0.123  0  0.024  0  0  0.019  0  0  0  0  0.03  0.04  0  0.069  0.456  0.103  0  0.027  0.06  0.028  0  0.069  0  0  0  0.271  0  0  0  0  0.025  0  0  0.028  0.039  0  0  0  0.038  0  0  0.029  0.094  0.157  0.029  0.036  0  0  0.039  0  0  0  
 
 SLC1A4  6509  2  65215578  65251000  2p15-p13  0.65  1.05  3.951  1.5  1.991  2.356  2.542  1.867  3.276  1.874  2.208  3.257  0.316  1.116  1.918  0.718  1.932  1.314  4.593  2.554  1.696  2.795  4.319  3.533  0.063  5.281  4.719  5.311  5.431  6.67  4.355  5.577  5.187  4.342  1.354  2.231  1.71  1.744  1.785  1.405  0.502  1.619  2.466  0.468  0.814  1.442  0.911  1.039  1.391  1.447  1.583  1.189  2.056  1.151  1.8  1.623  2.576  1.269  1.455  1  
 
 CEP68  23177  2  65283494  65314142  2p14  1.143  0.721  1.015  0.557  0.731  0.785  0.831  0.9  1.555  1.277  0.868  0.399  1.162  1.782  1.584  0.625  0.997  0.887  1.767  0.968  1.019  1.396  1.298  0.892  0.924  1.806  0.861  1.553  1.501  1.434  1.639  1.917  1.604  1.148  1.024  1.214  1.358  1.448  0.933  2.049  1.147  0.865  2.188  1.283  0.8  1.088  1.136  0.662  1.696  1.354  1.216  1.033  1.181  0.673  1.416  0.943  1.186  1.29  1.183  1.272  
 
 RAB1A  5861  2  65313987  65357435  2p14  4.058  5.737  3.56  2.917  4.173  2.909  5.045  4.026  4.023  3.945  4.578  3.641  3.992  5.08  3.442  4.06  4.621  5.004  4.602  3.247  3.104  2.945  3.221  2.341  4.325  4.343  3.115  3.106  4.172  3.326  4.965  4.806  3.519  3.064  3.466  4.454  3.76  3.695  4.545  4.397  4.084  3.607  4.63  4.082  2.758  3.179  3.011  2.965  3.245  5.324  4.173  2.62  3.49  3.948  4.449  4.172  3.989  3.605  3.322  4.465  
 
 ACTR2  10097  2  65454828  65498390  2p14  3.105  4.749  4.403  2.816  3.929  3.552  3.671  3.883  3.437  3.775  4.538  3.502  3.744  3.889  2.968  3.837  3.859  4.074  5.699  5.31  3.716  4.052  3.141  2.513  4.775  4.124  4.382  2.72  3.38  2.172  3.816  3.928  3.517  3.278  3.378  3.766  3.263  3.47  5.014  4.147  3.958  3.484  4.005  3.752  2.759  3.042  3.261  2.862  3.909  4.899  4.308  2.629  3.57  3.52  4.303  3.87  3.869  4.06  3.533  4.441  
 
 SPRED2  200734  2  65537984  65659656  2p14  1.888  2.779  1.988  1.772  2.825  1.286  2.998  2.783  3.208  2.181  2.892  2.536  2.479  3.876  2.862  3.535  3.14  3.13  3.215  2.469  2.68  2.85  1.961  1.852  4.032  3.578  3.492  3.203  4.11  3.024  3.289  3.55  4.043  2.666  1.982  3.158  2.973  3.324  2.549  4.261  2.953  1.876  4.963  3.007  1.956  1.884  3.229  2.416  3.034  3.039  2.815  2.213  2.095  1.826  2.943  2.293  2.255  2.694  2.561  2.515  
 
 LOC729348  729348  2  66610437  66619578  2p14  0.203  0  0.161  0  0.094  0  0  0  0.142  0.047  0  0.126  0.109  0.072  0  0.258  0.143  0  0.043  0  0  0.072  0.581  0  0  0  0.133  0.056  0  0.042  0  0  0  0.532  0  0.134  0  0  0.034  0  0.091  0.483  0  0  0  0.049  0.201  0  0  0  0  0.168  0  0  0  0  0  0.143  0  0  
 
 MEIS1-AS3  730198  2  66650474  66660602  2p14  0  0.046  0.098  0  0.044  0.041  0.054  0.057  0.078  0.051  0.029  0.08  0  0.027  0  0.018  0.044  0.099  0  0.058  0  0.134  0.188  0  0.039  0.032  0.16  0.023  0  0.062  0.081  0.044  0  0.23  0.053  0  0  0  0.03  0  0.109  0.215  0.052  0  0.108  0.131  0.098  0.125  0  0.03  0.03  0.575  0.027  0.034  0.031  0  0  0.117  0  0  
 
 MEIS1  4211  2  66662531  66799891  2p14  0.39  0.035  0.735  0.3  0.35  0.937  1.238  0.669  0.776  0.861  1.126  0.125  0.301  0.018  0  0.436  0.404  0.616  0.068  0.063  0.61  0.51  0.392  0.375  0.614  0.516  0.885  0.666  0.864  0.533  0.863  1.108  1.6  0.539  0.45  0.59  0.323  0  0.23  0.542  0.715  0.351  0.337  0  1.976  2.018  3.025  0.383  1.57  0.59  0.345  0.339  0.417  0.424  0.264  0.091  0.825  0.316  0  0.546  
 
 MEIS1-AS2  100873998  2  66666226  66667462  -  0  0  0.244  0  0  0  0.133  0.347  0  0.255  0.337  0.21  0  0  0  0.452  0  0  0  0.255  0  0.148  0  0  0  0.22  0.286  0  0.192  0.193  0  0  0.437  0  0  0.186  0  0  0.171  0  0.335  0.424  0.082  0  0.5  0.851  0.114  0  0  0  0  0.397  0  0.19  0  0  0.269  0  0  0.203  
 
 LOC100507073  100507073  2  66801161  66922720  -  0  0.12  0  0  0  0  0  0  0  0  0  0  0  0  0  0  0  0  0  0  0  0  0  0  0  0  0.108  0  0  0  0  0  0  0  0  0  0  0  0  0  0  0  0  0  0  0  0.14  0  0  0  0  0  0  0  0  0  0  0  0  0  
 
 LOC101927577  101927577  2  66924161  66930359  2p14  0  0  0  0  0  0  0  0  0  0  0  0  0  0  0  0  0  0  0  0.214  0  0  0  0  0  0  0.064  0  0  0.058  0  0  0  0  0  0  0  0  0  0  0  0  0  0  0  0  0  0  0  0  0  0  0  0  0  0  0  0  0  0  
 
 LOC101060019  101060019  2  67131567  67198598  -  0  0  0  0  0  0  0  0  0  0  0  0  0  0  0  0  0  0  0  0  0  0  0  0  0  0  0  0  0  0.061  0  0  0  0  0  0  0  0  0  0  0  0  0  0  0  0  0  0  0  0  0  0  0  0  0  0  0  0  0  0  
 
 LOC102800447  102800447  2  67313577  67516376  2p14  0  0  0  0  0  0  0  0  0  0  0  0  0  0  0  0  0  0  0  0  0  0  0  0  0  0  0  0.192  0  0  0  0  0  0.349  0  0  0  0  0.126  0  0  0  0.075  0  0  0  0  0  0  0  0  0  0  0  0  0  0  0  0  0  
 
 LOC644838  644838  2  67350488  67442451  2p14  0  0  0.075  0  0  0  0  0  0  0  0  0  0  0  0  0  0  0.068  0  0  0  0  0  0  0  0  0.068  0.08  0  0.057  0  0  0  0.161  0  0  0  0  0  0  0  0  0  0  0  0  0.139  0  0  0  0  0  0  0  0  0  0  0  0  0  
 
 ETAA1  54465  2  67624441  67637533  2p14  0.573  1.846  0.666  0.599  1.043  0.878  1.102  0.712  1.075  1.229  1.375  0.767  0.811  1.533  1.018  1.256  1.664  2.155  2.301  0.756  0.645  1.212  0.902  0.462  1.132  1  0.72  0.937  1.172  0.617  1.23  1.08  1.25  1.035  0.983  1.614  0.887  0.701  0.95  1.098  1.203  1.297  1.048  0.779  0  0.752  0.358  0.587  1.124  1.374  1.243  0.995  0.833  1.198  1.608  1.034  1.05  1.451  0.97  1.261  
 
 LOC101927701  101927701  2  68023185  68052694  2p14  0  0.128  0  0  0  0  0.203  0  0  0  0.128  0  0  0  0  0  0  0  0  0  0  0.077  0  0  0.468  0.136  0  0.976  0.4  0.073  0.362  1.678  0.567  1.043  0  0  0  0  0  0  0  0  0  0  0  0  0  0  0  0  0  0  0  0.131  0  0  0  0  0  0  
 
 C1D  10438  2  68269331  68290159  2p13-p12  1.84  4.841  1.536  1.465  3.03  2.086  2.632  1.885  2.053  2.15  3.162  2.039  2.043  3.633  1.782  2.535  2.552  4.621  4.658  3.491  2.623  2.898  2.098  1.537  2.633  2.689  0.93  1.552  1.606  0.681  2.825  2.995  1.788  1.999  2.336  2.617  1.83  1.989  3.775  2.247  2.719  2.195  2.511  2.268  1.655  1.958  1.621  1.301  1.787  3.556  2.573  1.362  1.459  1.902  3.114  2.516  1.806  2.915  1.799  2.775  
 
 WDR92  116143  2  68357280  68384692  2p14  1.522  2.086  1.144  1.266  1.501  1.082  1.835  1.128  1.12  1.604  1.624  1.332  1.598  1.556  1.24  1.712  1.136  1.527  2.604  1.623  1.088  1.399  1.669  1.185  1.382  1.117  1.192  0.646  1.21  0.557  1.6  1.469  0.816  0.893  1.235  1.486  1.397  1.36  1.685  1.938  1.336  1.057  2.037  1.093  1.189  1.333  0.662  1.063  1.211  2.336  2.11  1.007  1.26  0.902  1.872  0.988  1.48  0.98  1.313  1.922  
 
 PNO1  56902  2  68385004  68403094  2p14  2.692  4.19  2.197  2.193  2.619  2.351  4.113  2.396  2.594  2.209  3.389  2.032  2.914  3.697  2.188  2.743  2.266  3.621  4.336  3.381  3.402  2.95  2.456  2.367  3.455  2.641  2.855  2.655  3.073  2.621  3.694  3.815  3.007  2.124  3.018  2.996  3.495  2.783  3.452  4.056  3.234  2.367  4.237  2.79  2.345  2.632  3.445  2.086  2.082  3.943  2.972  1.707  2.607  2.332  3.306  2.473  2.587  2.923  2.275  2.955  
 
 PPP3R1  5534  2  68405988  68479651  2p15  1.945  1.544  1.695  1.474  1.58  2.36  1.998  2.109  2.103  1.893  2.524  2.015  2.808  2.388  2.048  2.123  2.4  1.36  3.224  1.382  2.201  1.953  1.782  2.006  2.632  2.395  1.196  2.265  2.308  1.375  2.376  2.453  2.438  1.561  2.005  2.071  2.273  1.872  1.817  3.017  2.514  1.952  1.614  2.347  1.991  2.137  0.335  1.599  2.206  1.873  2.601  1.859  1.787  2.095  1.995  1.856  1.924  2.396  1.707  2.269  
 
 CNRIP1  25927  2  68511302  68547183  2p14  0.218  0.118  1.923  1.818  0.138  2.426  5.126  3.164  1.734  2.521  4.532  0  0.155  0.072  0.106  0.147  0  0.068  0.061  0.376  0  0.667  0.382  0.092  3.038  0.066  3.305  0.308  3.342  2.923  3.673  2.89  2.708  2.08  0.359  0  2.259  0  2.555  0.572  0  2.076  0.163  0  0.147  0.082  0.106  1.025  0.123  2.104  2.044  1.203  1.338  0.321  2.678  0.618  1.928  0.791  0.118  1.332  
 
 PLEK  5341  2  68592321  68624585  2p13.3  0  0  0.124  0  0  0  0  0  0.061  0  0  0.058  0  0.016  0  0  0  0.129  0.104  4.246  0  0.186  3.359  0.186  0  0  0.065  0  0  0.04  0  0  0  0.254  0.046  0  0  0  0.04  0  0  0.027  0.025  0  0  0  0.12  0  0  0  0  0.131  0.102  0.071  2.083  0  0.199  0  0  0  
 
 FBXO48  554251  2  68689504  68694390  2p13.3  0.222  1.34  0.249  0.194  0.176  0.355  0.368  0.221  0.223  0.339  0.395  0.282  0.265  0.301  0.158  0.151  0.385  0.742  0.481  0.121  0.146  0.34  0.549  0.296  0.218  0.364  0.317  0.198  0.302  0.068  0.458  0.331  0.249  0.314  0.167  0.242  0.325  0.449  0.393  0.536  0.356  0.71  0.106  0.2  0.071  0.262  0.34  0.163  0.186  0.282  0.317  0.17  0.233  0.222  0.284  0.135  0.388  0.283  0.139  0.277  
 
 APLF  200558  2  68694690  68807294  2p13.3  0.145  1.937  0.339  0.303  0.026  0.102  0.388  0.182  0.279  0.495  0.204  0  0.338  0.314  0.1  0.037  0.23  0.75  0.813  0.078  0.081  0.268  0.274  0.133  0.459  0.54  0.122  0.275  0.249  0.106  0.536  0.281  0.175  0.216  0.133  0.673  0.211  0.447  0.282  0.49  0.351  0.131  0.4  0.273  0.056  0.271  0.256  0.115  0.132  0.287  0.343  0.353  0.27  0.245  0.658  0.223  0.767  0.139  0.285  0.458  
 
 PROKR1  10887  2  68872953  68882708  2p13.1  0  0  0.186  0  0.109  0  0  0  0.134  0.055  0  0  0  0.04  0  0  0  0.197  0  0.089  0  0.364  0.266  0  0  0.996  0.075  0.097  0.241  0.378  0  0  0  0.365  0  0  0  0  0.122  0  0  0.221  0.071  0  0  0  0.053  0  0  0  0.086  1.271  0  0.159  0  0  0  0  0  0  
 
 ARHGAP25  9938  2  68961912  69053957  2p13.3  0  0.065  0.722  0  0.025  0.099  0.87  0.071  0.27  0.228  0.529  0  0.135  0.024  0  0.034  0.107  0.136  1.268  2.331  0.714  2.361  0.665  0.149  0  0.077  0.132  0.101  1.13  0.073  0.048  1.393  0.102  0.344  0  0.161  0.05  0  0.062  0.137  0.109  0.18  0.043  0  0.1  0.18  0.495  0  0  0.092  0  0.256  0  0.082  0.046  0  0.09  0  0  0  
 
 BMP10  27302  2  69092612  69098649  2p13.3  0  0  0.105  0  0  0  0  0  0.076  0  0  0  0  0.025  0  0  0  0.091  0.181  0  0  0.208  0.238  0  0  0  0.046  0  0  0.056  0  0  0  0.292  0  0  0  0  0  0  0  0.063  0.034  0  0  0  0  0  0  0  0  0.244  0  0  0  0  0  0  0  0  
 
 GKN2  200504  2  69172363  69180102  2p13.3  0  0  0  0  0  0  0  0  0  0  0  0  0  0  0  0  0  0  0  0  0  0  0  0  0  0  0  0  0  0  0  0  0  0  0  0  0  0  0  0  0  0  0  0  0  0  0  0  0  0  1.971  0.722  0  0  0  0  0  0  0  0  
 
 GKN1  56287  2  69201704  69208112  2p13.3  0  0  0  0  0  0  0  0  0  0  0  0.16  0  0  0  0  0  0  0  0.165  0  0.073  0  0  0  0  0.092  0  0  0.056  0  0  0  0  0  0  0  0  0.541  0  0  1.041  0  0  0  0  0  0.229  0  0.49  0  0  0  0  0  0  0  0  0  0  
 
 ANTXR1  84168  2  69240275  69476459  2p13.1  1.941  3.301  2.513  2.626  1.017  2.601  2.965  4.96  3.264  3.663  3.036  0.101  0.196  2.902  0  0.047  0.064  1.015  1.636  0.134  0  2.461  0.26  0  3.413  3.745  2.366  2.714  2.975  2.848  3.112  3.841  3.771  2.13  3.513  5.188  3.479  2.091  4.064  1.757  0.615  4.129  1.009  1.005  1.975  1.191  0.427  2.711  1.646  3.183  3.136  3.394  1.986  1.751  3.338  4.044  3.226  3.939  1.987  3.111  
 
 GFPT1  2673  2  69546900  69614386  2p13  1.844  2.751  1.792  1.067  1.643  1.592  1.944  1.891  1.797  1.766  2.395  2.617  2.611  2.235  2.187  2.189  2.423  1.963  2.444  1.049  1.542  1.544  1.244  0.815  1.472  2.407  1.285  1.703  2.239  0.927  2.644  2.271  2.417  1.231  1.331  2.276  2.305  1.704  2.702  2.011  2.181  1.248  1.654  1.694  0.97  2.172  1.209  1.332  1.74  2.587  2.523  1.174  1.62  1.969  2.053  2.373  2.352  1.624  1.564  2.434  
 
 NFU1  27247  2  69623244  69664760  2p15-p13  1.706  4.87  1.764  1.053  2.914  2.523  3.445  2.392  1.92  2.592  3.076  2.321  2.576  3.541  1.864  2.491  3.002  4.592  3.976  2.393  2.568  2.102  0.523  0.603  2.684  2.472  1.128  1.324  2.253  1.877  3.263  3.385  2.259  1.311  2.643  2.917  2.393  2.778  3.241  3.096  3.012  2.475  3.667  2.41  1.634  2.16  2.523  1.387  3.103  3.824  2.482  2.16  1.676  1.824  3  2.351  2.257  2.921  1.866  2.862  
 
 AAK1  22848  2  69685126  69870977  2p14  0.779  1.722  1.358  0.959  0.957  1.288  2.551  2.043  1.192  1.917  1.988  0.551  0.791  1.523  0.878  1.09  0.981  0.476  1.888  0.518  0.545  1.426  0.449  0.253  1.214  1.888  1.395  1.702  2.041  1.454  1.707  1.901  2.035  0.978  1.24  2.095  2.182  2.096  1.778  1.965  2.014  0.844  1.546  1.041  1.115  1.643  1.219  1.438  1.657  1.268  1.884  1.215  1.637  1.6  1.786  1.157  2.073  1.38  1.451  1.389  
 
 ANXA4  307  2  69969126  70053596  2p13  2.839  3.96  1.697  2.126  2.611  0.935  3.074  2.284  2.078  2.183  2.707  4.868  4.384  3.829  2.069  4.725  3.472  5.31  2.59  2.655  0.974  1.871  1.318  1.006  3.018  3.202  3.448  1.392  2.706  1.249  2.884  2.88  3.256  2.482  4.691  6.387  1.874  3.091  4.209  3.039  2.877  1.317  2.638  5.232  1.583  3.847  2.374  1.35  4.988  2.817  3.045  2.342  2.73  4.689  4.177  4.593  4.49  1.401  3.316  4.692  
 
 GMCL1  64395  2  70056817  70106727  2p13.3  1.706  2.553  0.713  0.555  1.372  0.654  0.868  1.102  1.306  1.348  1.416  1.344  1.655  2.078  1.726  1.396  1.73  2.216  3.168  1.43  1.556  1.693  0.832  0.819  0.757  1.424  0.846  1.459  1.05  0.661  1.283  0.976  1.918  1.091  1.154  2.367  1.046  1.256  0.593  2.028  1.519  0.927  1.505  1.546  1.271  1.332  0.095  1.266  1.702  1.652  1.291  0.93  0.718  0.847  0.856  0.954  1.058  1.308  0.728  1.176  
 
 SNRNP27  11017  2  70121074  70132368  2p13.3  2.41  4.677  2.066  1.949  2.956  2.733  2.719  2.231  2.803  2.834  3.156  2.255  2.196  3.487  1.82  2.89  2.922  4.295  4.585  2.988  3.069  3.156  2.086  1.825  3.033  2.454  1.175  1.604  2.389  1.476  3.077  2.654  2.224  2.223  2.542  2.989  2.139  2.08  4.29  2.437  2.666  2.716  3.227  2.836  2.058  2.483  2.565  2.289  2.826  3.649  2.829  2.113  2.129  2.345  3.42  2.503  2.607  3.442  2.289  2.888  
 
 MXD1  4084  2  70142172  70170076  2p13-p12  1.2  1.34  0.802  0.78  0.767  0.361  1.342  0.778  0.689  0.797  0.362  2.434  2.062  0.957  0.813  1.45  2.58  1.968  1.24  1.842  1.163  0.989  1.13  0.728  0.834  0.988  0.762  0.406  0.795  0.333  0.496  0.811  0.624  0.618  0.349  0.666  0.498  1.057  1.3  0.724  0.773  0.232  1.002  0.642  0.762  0.825  0.806  0.337  0.238  0.414  1.149  0.602  0.316  0.96  0.897  0.597  1.159  0.571  0.483  0.535  
 
 ASPRV1  151516  2  70187223  70189397  2p13.3  0.254  0.043  0.219  0.218  0.13  0  0.256  0.07  0.323  0.189  0.53  0.909  0.241  0.302  0.135  0.062  0.185  0.253  1.08  0.211  0  0.382  0.458  0.136  0.096  0.092  0.666  0.251  0.186  0.069  0  0.198  0.488  0.364  0.157  0.104  0.073  0  0.187  0.307  0  0.187  0.044  0.111  0  0.046  0.021  0.16  0.221  0.37  0.157  0.375  0.025  0.251  0.064  0  0.157  0.065  0  0  
 
 PCBP1-AS1  400960  2  70189394  70314147  2p14  0.312  0.045  0.227  0  0.15  0.057  0.173  0.175  0.286  0.209  0.332  0.337  0.22  0.302  0.309  0.142  0.169  0.073  0.638  0.26  0.199  0.646  0.429  0.163  0.117  0.199  0.564  0.377  0.31  0.072  0.079  0.172  0.768  0.265  0.139  0.219  0.068  0.11  0.193  0.202  0.14  0.265  0.077  0.212  0.105  0.167  0.073  0.435  0.151  0.257  0.101  0.377  0.169  0.256  0.231  0  0.17  0.106  0.136  0  
 
 PCBP1  5093  2  70314584  70316334  2p13-p12  6.636  7.157  5.969  6.353  6.434  5.267  7.141  5.778  6.006  6.329  6.29  6.042  6.291  6.902  6.128  6.402  6.618  7.323  8.431  7.069  6.25  6.837  6.478  6.4  6.443  6.472  6.7  6.347  7.043  6.66  7.167  7.3  6.752  5.821  6.2  6.836  6.353  6.056  6.487  7.137  6.807  6.828  6.99  5.998  5.736  5.669  6.705  6.389  5.356  7.36  5.877  5.599  5.692  5.31  6.618  5.936  6.19  6.801  6.043  6.138  
 
 LOC100133985  100133985  2  70351167  70352448  2p13.3  0.686  0  0.357  0  0.345  0.871  0.172  0.248  0  0.372  0.216  0  0.283  0.103  0.445  0.408  0.53  0.214  1.111  0  0  1.294  1.169  0.558  0.414  0.369  0.391  0.264  0.385  0.617  1.65  0.412  0.73  1.37  0.754  1.761  0.298  0.641  0.19  1.272  0.723  0.928  2.143  0.265  0  1.033  0.397  0.417  0  1.131  0.137  1.272  0  0.333  1.22  0.223  0  0.282  0.219  0.218  
 
 C2orf42  54980  2  70377016  70418151  2p13.3  1.233  2.071  0.917  1.071  0.997  0.67  1.53  1.224  1.664  1.409  1.228  0.726  0.998  1.695  1.402  0.885  1.165  1.832  1.829  1.297  0.847  1.568  1.283  1.049  0.893  1.407  0.948  1.266  1.42  0.5  1.218  1.404  1.209  0.753  1.045  1.203  1.111  1.139  1.894  1.753  1.024  0.923  1.227  0.683  0.783  1.051  1.202  0.857  0.952  1.468  1.494  0.596  0.972  0.921  1.322  1.358  1.213  0.644  0.706  1.161  
 
 TIA1  7072  2  70436575  70475779  2p13  1.667  2.354  1.456  1.546  1.771  1.301  2.138  1.53  2.55  2.117  2.595  0.946  1.788  2.683  1.995  2.233  2.099  2.994  4.379  2.105  1.75  2.915  1.469  1.186  1.615  1.918  1.396  1.113  1.557  0.946  2.229  1.912  1.528  1.013  1.324  2.474  1.582  1.725  2.216  2.44  1.432  1.741  2.823  2.02  1.243  1.949  1.618  1.403  1.802  3.303  2.392  1.171  1.117  1.277  2.298  1.144  1.994  1.381  1.402  2.171  
 
 PCYOX1  51449  2  70485230  70508317  2p13.3  2.569  3.187  2.164  1.379  2.918  1.247  3.331  2.385  3.536  3.447  2.606  1.387  2.627  2.964  2.525  1.928  2.145  1.739  1.611  1.137  1.361  1.299  1.989  1.586  2.163  2.729  1.987  1.996  2.388  2.044  2.787  2.741  2.44  2.083  2.197  3.543  2.218  2.84  1.959  3.444  2.657  1.994  3.732  2.538  2.267  2.511  2.517  1.374  2.921  2.393  2.484  1.362  2.088  2.106  2.416  2.486  2.904  2.31  2.25  2.365  
 
 SNRPG  6637  2  70508505  70520869  2p13.3  5.302  9.178  3.614  2.822  5.754  6.25  6.238  4.801  3.397  4.984  5.688  5.644  5.169  6.503  4.573  5.911  5.881  9.892  7.732  7.53  6.162  6.822  3.991  5.308  6.245  4.673  4.434  3.968  4.769  3.577  5.699  5.673  5.408  3.624  6.709  5.353  4.599  5.069  7.119  5.187  5.482  5.627  6.635  5.346  4.988  4.847  6.896  5.028  4.976  7.179  4.785  4.235  5.243  5.451  6.665  6.007  4.285  7.368  5.076  5.065  
 
 FAM136A  84908  2  70523107  70529220  2p13.3  4.62  5.47  2.794  3.523  3.648  3.224  4.233  2.883  3.781  3.263  4.725  3.535  4.189  5.292  4.152  4.443  4.393  5.145  5.785  5.616  4.146  4.726  4.067  3.535  4.05  3.096  3.64  3.162  3.278  3.61  4.373  4.515  4.362  3.327  4.08  4.311  3.319  3.195  5.083  4.69  4.192  3.752  5.6  4.457  3.951  4.338  4.36  3.279  3.704  5.147  3.763  3.026  3.632  3.124  4.635  3.495  3.06  4.172  3.314  4.174  
 
 TGFA  7039  2  70674411  70781147  2p13  0.24  2.618  0.108  0  1.007  0.057  0.199  0.026  4.497  0.741  2.494  1.612  1.261  2.177  1.193  2.331  1.042  1.401  0  0.067  0  0.171  1.834  1.07  0.047  0.104  0.134  1.868  0.779  0.368  0.077  2.381  0.2  0.345  0.79  0.935  0.778  2.556  0.981  0.287  3.265  1.221  1.296  0.073  0.994  1.227  3.227  0.062  1.953  0.931  2.145  1.306  1.943  2.674  3.449  2.201  2.713  0.042  2.479  2.765  
 
 TGFA-IT1  100874273  2  70694516  70708772  -  0  0  0  0  0  0  0  0  0  0  0  0  0  0  0  0  0  0  0  0  0  0  0  0  0  0  0  0  0  0  0  0  0  0.473  0  0  0  0  0  0  0  0  0  0  0  0  0  0  0  0  0  0  0.279  0  0.242  0  0  0  0  0  
 
 ADD2  119  2  70889215  70995375  2p13.3  0  0.05  0.276  0  0.056  1.603  0.501  0.038  2.047  1.314  3.977  0.048  0  0.055  0  0.082  0.052  0.112  0.04  0.438  3.45  0.554  1.43  2.583  0  0  0.131  0.041  0.053  1.663  1.316  0.73  0  0.34  2.714  0.042  0.293  0  0.787  1.924  1.275  2.758  2.986  0.057  1.798  0.664  0.259  3.107  0.088  2.664  0.042  2.985  0.035  2.544  3.199  0.172  0.041  0  0  0  
 
 FIGLA  344018  2  71004441  71017775  2p13.3  0  0  0  0  0  0  0  0  0  0  0  0  0  0  0  0  0  0  0  0  0  0.188  0  0  0  0  0.107  0  0  0.116  0  0  0  0  0  0  0  0  0.124  0  0  0.294  0  0  0  0  0  0  0  0  0  0.551  0  0  0  0  0  0  0  0  
 
 CLEC4F  165530  2  71035775  71047732  2p13.3  0  0  0.136  0  0.083  0.069  0  0  0.094  0.039  0  0  0  0.024  0  0  0  0.059  0  0.082  0  0.072  0.328  0  0  0  0.042  0  0  0.032  0  0.2  0  0.2  0  0  0  0  0.049  0  0.093  0  0.03  0  0  0  0.022  0  0  0.068  0.038  0.197  0  0.038  0  0  0  0  0  0  
 
 CD207  50489  2  71057342  71062953  2p13  0  0  0.13  0  0.074  0.159  0  0  0.211  0.045  0.128  0  0  0.042  0  0  0.12  0  0  0.119  0  0.051  0.303  0  0  0.058  0.048  0  0  0.136  0.172  0.244  0  0.288  0.145  0  0.239  0  0.055  0.23  0.114  0.267  0.043  0  0  0.051  0.031  0.144  0  0.115  0  0.283  0  0.148  0.103  0  0  0  0.193  0  
 
 LINC01143  104355141  2  71115000  71117114  2p13.3  0  0  0  0  0  0.53  0  0  0  0  0  0  0  0  0  0  0  0.341  0  0  0  0.124  0  0  0  0  0.161  0  0  0.102  0  0.599  0  0  0  0.418  0  0  0  0  0  0.447  0  0  0  0  0  0  0  0  0  0.613  0  0.33  0  0  0  0  0  0  
 
 VAX2  25806  2  71127719  71160575  2p13  0.994  0.407  0.601  0.33  1.099  1.945  1.532  0.794  1.372  1.637  0.926  0.107  0  0.208  0  0.165  0.133  0.428  0  0.523  0  0.495  0.255  0  0.74  1.126  1.587  0  1.185  0.969  2.236  1.611  1.137  0.814  1.181  1.272  0.309  0.204  1.009  0.855  1.309  1.94  2.764  1.427  0  0.566  1.704  0.536  0.861  1.356  0.424  1.28  0.075  0.312  0.166  0  0  0.511  0  1.215  
 
 ATP6V1B1  525  2  71162997  71192561  2p13.1  0.621  0.114  0.193  0  0.582  0.343  0.323  0.299  0.656  1.014  0.401  0.081  0  0.155  0  0.78  0  0.314  0  0.124  0  0.33  0.183  0.072  0  0.372  0.305  0.192  0.26  0.176  0.674  0.308  0.182  0.191  0.474  1.086  0.059  0  0.509  0.206  0.725  1.074  0.169  1.045  0.135  0.453  0.034  0  0.319  0.171  0  1.002  0  0.21  0.173  0  0  0.08  0  0.18  
 
 ATP6V1B1-AS1  101927750  2  71169142  71175741  2p13.3  0  0.776  0.461  0  0.238  0.461  0.679  0.217  0.37  1.298  1.372  0  0  0.379  0  0.125  0  0.304  1.005  0.138  0  0.22  0.437  0  2.002  1.086  0.333  2.809  1.645  1.022  2.68  2.608  1.457  1.355  0.406  0.401  1.008  1.519  0.838  0.667  0.365  1.305  0  0.171  0  0  0.394  0.791  0.279  2.172  0.24  1.035  0.514  1.033  1.698  0.227  0  0.605  0.304  0.817  
 
 ANKRD53  79998  2  71205574  71212629  2p13.3  0.215  0.105  0.142  0  0.12  0.093  0  0.107  0.352  0.192  0.145  0.152  0.121  0.105  0  0.117  0.081  0.158  0.078  0.134  0  0.324  0.285  0  0.058  0.425  0.157  0.071  0.152  0.103  0.153  0.085  0  0.209  0.093  0.115  0.124  0.191  0.137  0.511  0.12  0.286  0.045  0  0.207  0.178  0.05  0.116  0.088  0.171  0.069  0.852  0.102  0.136  0.399  0  0.064  0.215  0  0.115  
 
 TEX261  113419  2  71213067  71222001  2p13.3  3.471  3.416  3.266  2.082  3.74  3.453  4.814  3.842  4.219  4.398  4.267  3.857  2.832  3.492  3.87  3.551  3.349  1.46  4.938  3.512  3.75  4.272  3.064  2.815  3.959  3.749  3.743  3.477  4.54  3.347  4.066  4.391  4.458  2.818  3.744  4.29  4.513  4.521  3.954  5.157  3.744  3.326  4.209  3.322  2.952  3.384  3.553  3.484  4.145  4.694  4.117  3.239  3.737  3.617  3.562  3.821  4.042  3.866  3.742  4.205  
 
 OR7E91P  79315  2  71251204  71257060  2p13.3  0  0  0.111  0  0.885  0  0  0  0.119  0.072  0  0.862  0.177  0.035  0  0.437  0.441  0  0  0  0  0.097  0.376  0  0  0  0.291  0  0  0.154  0  0  0  0.363  0  0  0  0  0.105  0  0.507  0.254  0.092  0  0.169  0  0.918  0  0  0  0  0.316  0  0.22  0.084  0  0  0  0  0  
 
 NAGK  55577  2  71295407  71305998  2p13.3  2.259  1.073  3.151  2.378  3.494  2.93  4.176  3.34  2.51  3.832  2.838  2.423  2.511  2.053  2.041  2.006  2.091  0.476  4.15  1.811  0.681  2.779  2.712  1.828  2.479  2.706  3.401  2.311  3.375  2.92  3.78  3.523  3.288  1.879  2.383  3.02  2.498  2.972  3.615  3.044  3.282  2.552  3.571  2.017  2.344  3.257  3.146  2.703  2.449  4.535  3.084  2.096  2.25  2.657  3.218  2.828  3.188  3.164  2.584  3.172  
 
 MCEE  84693  2  71336805  71357394  2p13.3  1.765  2.805  0.971  1.115  3.178  1.15  1.748  1.719  1.202  1.83  1.332  1.655  1.091  2.075  0.869  1.552  2.114  4.367  2.903  2.269  1.471  1.624  2.674  1.805  1.194  1.549  0.69  0.582  1.267  0.576  1.859  1.818  1.163  1.178  1.387  1.664  0.721  0.886  1.389  1.44  1.459  1.756  0.95  1.234  0.589  1.12  0.154  0.606  0.626  1.994  1.293  0.694  0.746  1.032  1.436  0.895  1.204  1.078  1.13  0.806  
 
 MPHOSPH10  10199  2  71357443  71377232  2p13.3  2.171  3.981  1.259  1.55  2.704  1.323  2.215  2.023  1.733  1.993  2.761  1.653  2.272  3.163  1.854  2.327  2.389  4.803  3.958  2.619  2.892  2.733  1.85  1.843  2.44  2.314  1.389  1.428  1.579  0.944  2.69  2.929  2.24  1.882  2.333  2.678  1.621  1.637  2.633  2.691  2.397  1.987  2.614  2.75  1.824  1.879  1.62  1.513  1.584  2.96  2.246  1.335  1.666  1.732  2.006  1.902  2.278  2.178  1.907  2.271  
 
 PAIP2B  400961  2  71409867  71454233  2p13.3  1.012  0.132  0.153  0  0.945  0.089  0.177  0.048  0.354  0.221  0.154  0.444  0.731  0.636  0.714  0.349  1.061  0.221  0.557  0.233  0.044  0.352  1.83  0.953  0.116  0.096  0.229  0.118  0.12  0.112  0.095  0.07  0.086  0.409  0.18  0.224  0.218  0.129  0.138  0.462  0.52  0.384  1.062  0.114  0.393  0.197  0.029  0.149  0.037  0.632  0.465  0.305  0.15  0.097  0.221  0.168  0.091  0.389  0.311  0.23  
 
 ZNF638  27332  2  71558884  71662191  2p13.1  2.135  2.745  0.966  1.199  1.903  1.084  1.723  1.818  1.948  1.992  2.425  1.433  1.992  2.884  2.417  1.819  1.852  3.692  2.853  1.667  1.829  1.766  1.127  1.131  1.977  2.157  1.15  1.731  1.937  1.4  2.031  2.072  1.984  1.277  1.792  2.324  1.822  1.645  1.875  2.491  2.198  1.705  2.361  2.298  1.413  1.685  1.034  1.797  2.233  2.805  1.92  1.354  1.381  1.296  2.28  1.792  1.837  1.748  1.727  2.136  
 
 DYSF  8291  2  71680752  71913893  2p13.3  0.07  0.599  0.391  0  0.142  0.06  0.327  1.707  0.111  0.76  0.147  0.045  0  0.103  0  0.039  0.078  0.092  0.071  0.557  0.193  0.158  0.2  0.071  0  1.55  0.334  0.504  0.67  0.341  0.042  0.17  0.087  0.199  0.098  0.243  1.156  0.507  0.793  0.169  0.467  0.638  0.238  0.073  0.091  0.855  0.419  0.224  0  1.628  0.114  0.845  0.603  0.127  3.37  0.322  1.745  1.905  1.234  4.111  
 
 CYP26B1  56603  2  72356366  72374991  2p13.2  0.13  0.487  0.325  0.202  0.057  0.76  0.908  1.258  0.348  1.513  0.461  0.066  0.367  1.34  0.323  0.02  0.073  0.121  0.028  0.076  0  0.172  0.397  0  0.108  0.508  0.063  1.096  0.077  0.092  0.038  0.056  0  0.201  0.688  2.532  0.109  0.692  0.49  1.253  0.778  0.515  0.526  0.337  0  0.062  0.352  0.63  0.076  1.233  0.059  1.263  0  1.332  0.819  0.071  0.716  0  1.215  0  
 
 EXOC6B  23233  2  72406443  73053177  2p13.2  0.616  1.368  0.54  0.157  0.711  1.064  0.965  0.871  0.422  1.19  1.262  0.247  0.964  1.724  0.982  0.352  0.65  0.512  0.288  0.142  0.19  0.545  0.153  0.358  0.961  1.34  0.606  1.321  1.28  0.543  1.041  1.018  1.032  0.274  0.753  1.889  1.282  1.29  0.65  1.838  1.916  0.823  1.062  1.422  0.703  0.81  0.507  1.198  2.432  0.533  1.625  0.823  0.792  0.736  0.877  0.628  1.419  1.016  0.531  1.69  
 
 SPR  6697  2  73114511  73119289  2p14-p12  5.032  2.405  2.014  1.64  3.431  1.438  4.602  2.625  3.573  2.469  3.633  3.529  4.453  5.096  4.151  3.547  4.135  0.693  1.789  1.814  2.664  1.458  1.333  1.598  4.401  3.875  4.04  3.849  4.254  5.638  4.424  4.541  4.063  3.102  4.219  4.751  3.512  3.879  3.462  5.34  3.948  3.974  5.437  2.372  2.112  2.47  4.443  2.859  2.333  4.476  4.07  2.819  3.383  3.538  4.462  3.284  2.791  4.078  3.357  3.911  
 
 EMX1  2016  2  73144603  73162020  2p13.2  0.125  0.129  0.168  0  0.043  0.168  0.038  0.106  0.196  0.104  0.088  0.128  0  0.231  0.118  0.07  0.223  0.238  0  0.13  0  0.255  0.276  0.067  0  0.054  0.329  0.071  0  0.24  0  0  0  0.267  0.082  0.051  0.049  0  0.238  0.139  0.047  0.298  0.081  0.078  0  0.073  0.044  0  0  0.087  0.07  0.861  0.047  0.206  0.074  0.068  0.088  0  0.299  0.343  
 
 SFXN5  94097  2  73169164  73298965  -  1.013  0.181  0.726  0.587  0.415  0.588  2.13  1.105  1.603  1.928  2.144  1.352  1.023  0.743  1.195  1.516  0.62  0.174  1.466  0.635  0.998  0.671  0.709  0.447  0.683  1.235  1.582  0.569  0.908  0.706  1.358  1.175  1.339  0.326  1.2  1.383  1.234  1.565  0.921  1.987  1.059  1.958  1.812  1.303  0.421  1.066  1.115  0.621  1.058  0.876  0.931  1.003  1.03  0.926  1.44  0.845  1.713  1.121  0.872  0.681  
 
 RAB11FIP5  26056  2  73300509  73340146  2p13  0.603  1.159  2.647  1.365  1.528  2.151  3.143  2.697  2.555  2.643  3.092  1.082  2.07  1.913  1.767  1.298  1.268  0.163  0.133  0.211  1.236  0.39  1.926  1.88  2.702  1.776  2.147  1.286  2.463  1.939  1.987  2.524  2.361  1.393  1.867  2.401  2.672  3.096  3.382  2.456  1.816  1.681  1.622  2.842  0.825  1.738  1.33  2.63  2.79  3.43  2.776  1.987  2.972  2.506  3.04  2.177  2.914  2.394  2.343  3.284  
 
 NOTO  344022  2  73429385  73438340  2p13.2  0  0  0.157  0  0.14  0  0  0.115  0.163  0.086  0  0.167  0  0.055  0  0.104  0  0.192  0  0  0  0.278  0.319  0  0.082  0.068  0.117  0.052  0  0.2  0  0  0  0.285  0.095  0  0.074  0  0.088  0.286  0  0.473  0.069  0  0.124  0.16  0.055  0.103  0  0  0.074  0.594  0.084  0.232  0.12  0.159  0  0  0  0.086  
 
 SMYD5  10322  2  73441365  73454355  2p13.2  3.147  0.748  2.095  1.981  2.292  2.469  3.992  2.252  2.865  2.552  3.117  2.115  3.387  3.49  3.047  2.81  2.344  0.16  4.422  2.268  2.974  2.82  1.569  2.358  3.589  2.753  2.996  2.962  3.178  3.35  3.58  3.717  3.336  1.325  3.092  3.143  3.461  3.189  2.409  4.026  3.236  3.006  3.971  2.792  2.269  2.245  2.536  2.968  2.966  4.278  3.244  2.424  2.874  2.387  3.395  2.876  2.512  3.216  2.789  3.165  
 
 PRADC1  84279  2  73455133  73460356  2p13.2  1.868  0.241  2.646  2.223  3.684  2.746  4.657  3.15  2.443  3.783  2.765  3.341  3.103  1.027  1.816  1.964  3.13  0  5.111  2.032  3.036  3.134  4.135  3.869  2.962  2.441  3.07  1.642  2.653  3.208  3.711  4.113  3.788  2.458  2.17  3.008  2.868  3.389  2.109  3.051  2.656  3.633  3.084  1.87  2.494  4.029  3.308  2.456  2.341  3.851  3.581  2.32  2.557  2.288  1.952  2.413  1.838  3.587  1.933  2.43  
 
 CCT7  10574  2  73461363  73480150  2p13.2  6.439  4.309  5.619  5.421  5.703  5.16  7.593  5.539  5.78  5.868  6.539  5.701  6.434  7.618  6.34  6.174  6.928  2.375  8.561  6.646  6.075  7.096  5.953  5.565  6.813  5.397  6.012  6.066  6.418  6.416  6.806  7.375  6.813  5.186  6.467  5.839  6.907  6.146  6.547  7.512  5.975  6.481  7.769  5.692  5.67  6.048  6.62  6.035  5.214  7.996  6.099  5.258  6.132  5.29  6.864  5.997  5.743  6.316  5.802  6.406  
 
 FBXO41  150726  2  73481809  73496758  2p13.2  1.501  0.293  0.775  0.407  1.202  0.38  0.764  0.914  0.867  1.188  0.761  0.595  2.102  2.146  2.532  2.332  1.21  0.225  3.346  1.491  1.005  1.707  1.279  1.959  1.54  1.672  1.157  1.481  1.555  0.942  1.973  1.523  2.755  1.139  0.971  1.392  1.494  1.991  0.417  1.981  0.652  1.049  1.36  0.553  0.861  0.798  0.952  1.436  0.232  2.188  0.611  1.486  1.482  0.995  1.21  0.739  1.701  1.376  1.534  1.114  
 
 EGR4  1961  2  73518056  73520829  2p13  0.131  0.074  0.214  0  0.111  0.086  0.08  0.065  0.225  0.103  0.078  0.285  0.15  0.137  0.197  0.247  0.644  0.357  0.045  0.408  0  0.501  0.44  0.077  0.08  0.088  0.315  0.145  0.118  0.281  0  0.069  0.085  0.328  0.148  0.081  0.072  0.086  0.171  0.369  0.142  0.944  0.135  0.057  0.134  0.137  0.14  0.078  0  0.428  0.081  1.001  0.107  0.285  0.24  0.161  0.065  0.098  0  0  
 
 ALMS1  7840  2  73612885  73837046  2p13  2.11  2.117  0.873  0.665  0.977  1.148  1.127  1.49  0.85  1.582  1.994  0.505  1.563  2.001  1.557  1.247  0.983  1.282  1.537  1.204  1.175  1.775  0.667  0.87  1.254  1.593  1.134  1.851  1.575  0.813  1.368  1.402  1.978  0.88  1.168  1.988  1.521  1.371  0.846  2.067  1.352  1.006  2.167  1.42  1.107  0.993  0.649  1.009  1.886  0.867  1.792  0.95  1.521  0.962  1.524  1.3  1.932  1.496  1.114  1.351  
 
 ALMS1-IT1  100874291  2  73684229  73686604  -  0.379  0.885  0.328  0  0.284  0.394  0.262  0.214  0.176  0.239  0.468  0.067  0.256  0.429  0.746  0.728  0.174  0.344  0.486  0.164  0.474  0.135  0.176  0.113  0.219  0.336  1.392  0.71  0.466  0.372  0.574  0.34  1.263  0.745  0.513  0.231  0.951  0.197  0.319  0.383  0.52  0.355  1.232  0.223  0.424  0.655  0  0.272  0.092  0.486  0.327  0.165  0.521  0.224  0.483  0.256  0.313  0.918  0.52  0.397  
 
 NAT8  9027  2  73867849  73869537  2p13.2  0  0  0.152  0  0  0  0.128  0  0.199  0  0  0  0  0.039  0  0.091  0  0  0  0  0  0.054  0.354  0  0  0.621  0.067  0.108  0.255  0.824  1.53  0.194  0  0.333  0  0  0  0  0.088  0.158  0  0.439  0  0  0  0  0  0  0  0  0  0.23  0  0  0  0  0  0  0  0  
 
 ALMS1P  200420  2  73872045  73912694  2p13.1  0  0  0.148  0  0.037  0  0  0  0.105  0.178  0.141  0  0  0.058  0  0  0  0  0  0.077  0  0.138  0.38  0  0  0.516  0.076  0  0.142  0.261  0.93  0.129  0  0.319  0  0.21  0  0.113  0.068  0.217  0  0.363  0.089  0  0  0  0  0  0.218  0.062  0  0.143  0.065  0  0.052  0.098  0  0  0  0  
 
 NAT8B  51471  2  73927635  73928467  2p13.1  0  0  0.17  0  0  0  0.14  0  0.239  0  0  0.155  0  0  0  0  0  0  0  0.231  0  0  0.46  0  0  0.293  0  0  0  0.237  0.318  0.192  0  0.556  0  0  0  0  0.125  0  0  0.142  0.082  0.197  0  0  0  0  0  0  0  0.401  0  0.09  0  0  0  0  0  0  
 
 TPRKB  51002  2  73956956  73964517  2p24.3-p24.1  2.251  6.219  1.835  1.627  3.293  3.757  3.151  1.878  1.934  2.835  3.449  2.742  2.283  3.838  1.502  3.106  2.992  7.249  5.253  4.021  3.334  3.797  2.293  2.472  3.639  2.325  1.175  1.714  2.114  0.935  3.139  2.96  2.54  2.704  3.801  2.718  2.04  2.133  4.833  2.809  2.977  3.583  3.618  3.062  2.5  3.017  2.134  1.532  2.796  4.514  2.997  1.764  2.088  2.755  3.048  2.539  2.494  3.918  2.276  2.263  
 
 DUSP11  8446  2  73989324  74007284  2p13.1  1.99  4.015  1.698  1.578  2.02  1.6  2.714  2.301  1.808  2.169  2.54  1.767  2.315  3.2  1.581  2.069  2.301  3.401  3.735  2.165  2.198  2.335  2.215  1.702  2.863  2.256  1.731  1.409  1.737  1.083  2.697  2.726  2.138  1.534  1.749  2.342  2.042  2.264  2.969  2.63  2.319  1.596  3.125  2.328  1.537  1.999  1.976  1.57  2.064  3.817  2.266  1.401  1.838  2.128  2.827  1.96  2.314  2.674  1.971  2.627  
 
 C2orf78  388960  2  74011315  74044274  2p13.1  0  0.067  0.187  0  0.044  0  0  0  0.133  0  0.124  0  0  0.3  0  0.033  0  0.059  0  0.088  0  0.09  0.43  0  0  0  0.082  0.055  0  0.08  0  0  0  0.315  0  0  0.035  0  0.035  0  0  0.15  0.033  0  0  0.031  0.194  0.048  0  0  0.027  0.292  0.029  0.116  0.085  0  0  0  0  0  
 
 STAMBP  10617  2  74056042  74094295  2p13.1  1.88  2.493  1.172  0.826  1.805  1.201  2.268  1.381  1.991  1.576  2.485  1.449  1.947  2.515  1.797  1.669  2.291  1.897  2.887  1.311  1.466  1.646  1.165  0.919  1.598  1.78  1.967  2.035  2.091  1.496  2.388  2.226  2.348  1.235  1.347  1.811  1.927  2.248  1.96  2.316  2.097  1.127  2.874  1.883  1.14  1.704  1.203  0.89  1.704  2.089  1.695  1.131  1.378  1.53  2.395  1.509  1.704  1.577  1.635  1.765  
 
 ACTG2  72  2  74120092  74146780  2p13.1  0.541  0  8.68  0  0.079  0.274  1.309  0.798  0.333  4.821  0.696  0  0  0.149  0  0.092  0  0  0.326  0.117  0  0.814  0.224  0.163  0.113  4.535  1.124  0.406  0.464  1.122  0.2  0.137  1.085  0.357  0.12  0.199  0.181  0.141  0.298  0.275  0.169  0.673  0.8  0.33  0  1.148  0.111  0  0  0.287  1.563  0.206  0.397  0.231  0.19  0.502  0.226  0  0.218  0  
 
 DGUOK  1716  2  74153952  74186088  2p13  3.931  5.969  3.659  3.307  3.815  3.774  4.858  3.327  3.847  4.089  4.502  3.793  3.864  5.259  2.899  4.482  5.153  5.712  6.101  5.287  3.921  5.387  3.787  3.159  4.254  4.151  3.58  3.544  3.88  2.457  4.972  4.698  4.016  3.47  4.311  4  3.833  4.041  5.54  4.274  4.016  4.253  4.47  3.945  3.147  3.608  4.929  2.689  3.445  4.844  3.994  3.294  3.41  3.971  5.242  3.554  3.457  4.721  3.931  4.191  
 
 DGUOK-AS1  100874048  2  74185094  74208566  -  1.77  3.08  0  0  2.124  0  0  0  0  0.3  0.653  1.847  0.587  3.26  0.649  1.289  0.933  4.65  2.524  1.433  2.204  2.619  1.603  1.189  0  0.283  0  0.366  0  0  1.508  0.366  0  0  1.764  1.073  0.322  1.267  0.571  0  0  0.744  2.776  0  0  0.517  0.915  0.488  0  1.285  0.476  1.257  0  0.17  1.731  0.765  0.742  0  0  0.413  
 
 TET3  200424  2  74213530  74335302  2p13.1  2.738  0.318  1.198  0.763  1.486  0.698  1.017  1.933  1.437  1.519  1.931  1.404  1.752  1.567  2.222  1.541  1.248  0.412  1.92  1.552  1.798  1.813  0.833  1.151  1.289  0.743  1.091  1.122  0.876  0.686  1.131  1.661  1.694  0.575  1.24  2.038  1.683  1.154  0.778  1.694  2.758  0.821  1.801  1.731  1.821  1.876  0.349  1.092  1.707  0.831  1.032  1.129  1.585  0.955  1.565  1.335  1.837  1.304  1.86  1.158  
 
 BOLA3  388962  2  74362527  74375039  2p13.1  4.884  7.453  3.609  2.393  4.954  5.143  5.194  3.465  3.298  3.954  4.298  4.141  3.679  5.368  2.913  4.431  5.371  8.186  6.434  7.194  6.042  5.912  3.107  4.913  5.5  4.456  4.064  3.229  4.458  5.158  6.085  6.045  4.406  3.45  5.825  5.171  4.246  4.211  6.576  4.834  4.922  5.064  5.292  4.792  3.544  5.217  6.532  3.362  4.762  6.06  4.314  3.858  4.312  3.865  5.129  4.475  3.658  5.53  4.409  4.202  
 
 BOLA3-AS1  100507171  2  74375107  74379516  -  0.472  0.324  0.742  0.357  0.298  0.148  0.506  0.325  0.391  0.441  0.3  0.159  0.132  0.307  0.223  0.123  0  0.563  0.566  0.195  1.341  0.333  0.453  0.318  0.332  0.875  0.865  0.202  0.593  0.587  1.221  1.795  1.224  0.845  0.281  1.072  0.492  0.378  0.301  0.863  0.211  0.874  2.532  0.473  0.829  0.665  0.297  0.468  0.188  1.135  0.32  0.76  0.124  0.088  0.966  0.46  0.444  1.237  0.723  0.202  
 
 MOB1A  55233  2  74383210  74405995  2p13.1  3.543  5.187  2.75  2.01  3.568  3.473  3.94  3.813  2.647  3.712  4.712  3.369  4.486  4.658  3.959  3.669  3.862  3.419  4.476  3.469  3.104  4.045  2.421  3.031  4.153  3.683  2.572  3.488  3.55  2.343  3.919  3.941  3.711  2.071  3.41  3.962  3.996  3.638  3.976  4.607  4.061  2.919  4.228  3.376  3.333  3.876  2.881  2.919  3.898  3.746  4.058  2.835  3.75  3.73  4.204  3.861  3.952  4.399  3.786  4.28  
 
 MTHFD2  10797  2  74425689  74442424  2p13.1  4.775  4.513  4.486  3.981  4.362  3.505  4.409  3.416  4.987  3.557  5.052  3.68  4.279  6.805  4.3  4.356  5.66  6.249  5.642  3.82  4.404  3.843  5.188  3.423  4.261  3.412  3.225  3.438  3.194  4.349  3.486  4.836  3.774  3.151  3.92  4.069  4.599  3.424  4.606  4.973  3.876  3.941  5.458  3.818  3.725  3.742  3.17  3.55  3.111  5.643  4.184  3.653  3.69  3.519  4.359  4.213  2.671  3.679  3.529  3.787  
 
 SLC4A5  57835  2  74443368  74570534  2p13  1.104  0.67  0.68  0.605  0.982  0.497  1.155  0.414  1.028  0.588  0.869  0.557  0.731  2.625  0.711  0.641  1.628  1.648  1.623  1.625  0.527  0.929  1.407  0.401  0.675  0.685  0.339  0.478  0.46  0.635  0.72  0.722  0.6  0.571  0.791  0.451  1.031  0.902  1.278  1.123  0.811  0.748  1.41  0.768  0.524  0.91  0.652  0.554  0.509  1.778  0.598  0.724  0.411  0.59  0.846  0.833  0.302  0.547  0.804  0.599  
 
 DCTN1  1639  2  74588280  74619214  2p13  3.634  1.165  3.733  2.859  2.885  3.04  4.52  3.661  3.274  3.839  3.654  2.568  3.458  3.398  3.642  2.824  3.272  0.211  4.866  2.317  2.777  3.465  2.341  2.727  3.985  3.881  4.048  4.502  4.103  3.944  3.306  4.222  4.246  2.319  3.156  3.966  3.981  3.579  3.034  5.443  3.596  3.248  3.946  3.556  3.009  3.4  2.88  3.784  3.8  5.2  3.881  3.248  3.743  3.09  4.06  3.513  4.042  3.949  3.455  4.268  
 
 DCTN1-AS1  100189589  2  74612844  74621008  2p13  0  0  0.073  0  0.03  0.076  0  0  0.124  0.025  0  0  0  0.078  0  0  0  0  0  0  0  0.138  0.203  0  0  0  0.042  0  0  0.036  0  0  0  0.144  0  0  0  0  0.036  0  0  0.061  0.022  0  0  0.062  0.021  0  0  0  0  0.315  0.044  0.047  0.029  0  0  0  0  0  
 
 C2orf81  388963  2  74641302  74644844  2p13.1  1.373  0.194  0.485  1.309  0.894  0.445  1.273  0.189  1.315  0.656  1.095  0.733  0.975  1.052  0.909  1.433  1.329  0.177  0.605  0.458  0.571  1.073  2.054  0.772  1.327  0.439  1.212  0.962  0.602  0.562  0.504  0.828  1.09  0.869  1.076  0.454  1.506  1.169  0.804  1.772  1.168  1.233  1.93  0.302  0.736  0.511  1.387  1.802  0.722  2.453  1.107  1.379  0.331  0.431  1.388  0.71  0.222  1.107  0.24  0.826  
 
 WDR54  84058  2  74648884  74652882  2p13.1  4.253  3.669  2.463  3.011  3.233  2.652  5.483  2.386  3.408  3.271  4.525  2.426  1.839  4.53  3.189  3.582  4.334  2.37  5.913  2.701  0.641  4.125  3.584  3.511  4.281  2.2  4.669  3.56  3.762  3.482  3.575  3.864  3.544  2.055  3.824  3.313  4.471  2.972  3.297  4.729  3.555  3.376  4.746  2.937  3.055  2.743  5.097  3.781  3.966  5.639  3.413  2.751  3.274  1.588  4.704  3.969  2.299  5.02  2.494  3.856  
 
 RTKN  6242  2  74652987  74669060  2p13.1  3.237  0.655  1.383  0.375  3.225  1.119  2.698  1.585  2.435  3.48  2.393  3.664  3.262  3.736  3.633  3.345  2.838  1.565  1.884  0.352  0.644  1.673  1.076  0.525  1.952  3.794  3.279  3.335  3.03  2.93  3.971  4.409  3.726  2.063  1.844  3.476  1.952  1.495  0.981  3.827  3.079  1.915  4.442  3.332  2.196  2.954  3.715  1.385  0.972  3.114  2.865  2.516  2.783  2.705  3.391  2.548  3.143  1.726  3.375  2.928  
 
 INO80B  83444  2  74682149  74685087  2p13.1  1.946  0.142  1.94  2.312  2.188  2.408  2.02  1.192  2.094  1.655  1.56  1.766  2.331  1.52  1.623  2.124  1.932  0  2.962  1.639  1.341  2.23  2.369  2.476  2.393  1.267  1.116  1.849  1.717  2.099  2.677  2.976  2.619  2.051  2.659  2.116  0.769  1.974  2.446  1.885  2.706  3.3  2.192  1.555  2.261  2.562  1.613  1.764  1.86  3.341  1.642  1.824  1.298  1.934  2.407  1.9  1.225  3.001  1.224  1.372  
 
 INO80B-WBP1  100532735  2  74682149  74688018  -  0.269  0  0  0  0.122  0  0  0  0.325  0  0.348  0  0  0  0.949  0  0  0.235  0  0  0.41  0  0  0.361  0  0.927  1.624  0.955  0.686  0.198  0  0  0  0  0  0  1.179  0  0  1.301  0  0  0  0  0  0  0  0  0  0.157  1.276  0  0.517  0  0.071  0  0.561  0  0  0.509  
 
 WBP1  23559  2  74685526  74688018  2p12  3.718  0.83  3.407  2.787  5.229  2.957  5.085  3.585  4.218  4.051  3.815  3.412  3.51  3.155  3.609  3.495  3.884  0  5.576  2.371  1.721  4.561  3.838  2.968  3.41  3.752  4.005  2.885  3.364  3.46  4.128  4.105  4.166  2.61  3.669  4.253  3.261  3.957  3.681  4.492  4.955  4.037  5.57  3.011  2.467  3.513  3.265  2.564  2.651  4.798  3.68  3.191  2.475  2.929  4.261  3.421  2.457  4.236  2.786  3.776  
 
 MOGS  7841  2  74688183  74692537  2p13.1  3.595  1.982  2.965  3.72  3.544  2.654  4.339  3.572  3.723  3.66  3.599  2.982  3.982  3.617  4.04  3.5  3.447  0.723  4.59  3.224  3.152  3.176  3.269  3.463  3.304  3.777  3.278  3.713  3.751  2.94  3.698  4.401  4.379  2.758  2.74  3.787  3.935  3.741  3.168  4.504  3.641  4.439  3.869  3.549  3.134  3.608  3.052  3.034  3.202  4.527  3.331  2.778  3.206  2.738  3.676  3.535  3.33  3.608  3.071  3.724  
 
 MRPL53  116540  2  74699084  74699942  2p13.1  5.004  5.107  3.976  3.696  5.192  4.501  5.851  3.737  4.454  4.368  4.121  3.871  3.611  4.656  3.509  4.138  4.846  4.536  6.253  5.52  3.385  6.036  4.756  4.382  4.608  3.574  4.896  3.93  4.497  4.684  5.614  5.039  4.376  4.173  5.043  4.819  3.669  3.866  5.573  4.039  5.277  5.548  5.575  3.171  4.14  4.401  5.317  3.724  3.206  5.85  3.099  4.063  3.963  3.747  5.602  3.892  2.349  5.755  2.86  3.325  
 
 CCDC142  84865  2  74699958  74710357  2p13.1  0.921  0.227  0.547  0.546  0.696  0.758  0.736  0.781  1.022  1.375  1.314  0.838  1.149  0.779  1.318  0.944  0.878  0.099  1.783  0.44  0.673  1.015  0.686  0.498  0.642  1.134  1.271  0.939  0.773  0.497  1.209  1.483  1.544  0.548  0.681  1.122  0.954  1.15  0.611  1.476  1.325  1.3  1.602  1.028  0.542  1.209  0.974  1.008  1.221  1.773  0.922  1.319  0.787  0.698  0.766  0.54  1.016  0.709  0.673  0.573  
 
 TTC31  64427  2  74710199  74721691  2p13.1  2.495  2.083  1.563  1.296  2.317  1.676  2.878  1.68  1.433  2.162  2.351  1.402  1.78  2.814  2.358  1.899  1.495  1.275  4.063  2.517  1.863  2.728  1.476  1.587  1.843  2.429  2.565  2.671  2.674  2.573  2.339  2.664  3.334  1.693  1.922  2.428  2.251  2.146  2.227  2.619  2.649  1.933  3.319  1.863  1.358  1.669  3.51  1.775  2.357  3.374  1.906  1.653  1.883  1.495  2.842  1.786  2.158  2.689  1.88  2.003  
 
 LBX2  85474  2  74724643  74730443  2p13.1  0.334  0.237  0.226  1.359  0.142  0.316  0.405  0.837  0.254  0.402  0.105  0.471  0.654  1.499  0.74  0.409  0.866  0.207  0.334  0.275  0.426  0.569  0.778  0  0.936  0.365  2.117  0.657  1.102  1.11  0.889  2.012  1.432  1.58  0.371  0.633  0.591  0.642  1.526  0  0.733  1.305  0.165  0.203  0  0.273  0.859  0.831  0.45  2.927  0.117  1.911  0.526  0.737  1.972  0.155  0.439  0.289  0.685  0.963  
 
 LBX2-AS1  151534  2  74729743  74732192  2p13.1  0.39  0.396  0.194  0.911  0.084  0.298  0.463  0.844  0.163  0.387  0.271  0.916  1.976  0.607  1.014  1.319  1.702  0.399  0.116  0.131  0.845  0.286  0.487  0.141  1.886  2.409  2.119  1.536  2.216  0.857  2.834  3.126  2.513  2.137  0.549  1.734  0.962  1.984  0.962  0.424  0.869  1.022  1.224  0.812  0.279  0.169  1.63  1.015  0.774  2.572  0.15  1.116  0.278  1.227  1.914  0.433  0.699  1.513  1.095  1.214  
 
 PCGF1  84759  2  74732169  74734821  2p13.1  3.012  2.899  1.703  1.584  2.466  1.506  4.111  2.383  1.489  2.617  2.992  2.184  3.001  4.167  2.615  2.407  3.409  1.375  4.021  3.529  2.06  2.831  1.804  2.05  2.81  2.43  2.797  2.8  2.879  3.06  3.021  3.596  3.233  1.723  2.278  2.329  2.893  3.165  3.048  3.677  2.96  1.704  4.081  2.008  1.91  2.446  2.666  2.148  2.11  3.636  2.589  1.755  2.13  2.633  3.077  2.582  2.589  2.807  2.054  2.677  
 
 TLX2  3196  2  74741595  74744275  2p13.1  0  0.107  0.547  0  0.085  0.341  0.34  0.158  0.979  0.247  0.192  0.272  0.135  0.096  0  0.093  0.099  0.128  1.734  0.207  0  1.173  0.504  0.056  0  0.049  0.817  0.06  0.133  0.537  0.05  0.135  0  0.306  0.208  0.081  0.092  0.084  0.242  0.257  0.069  0.89  0.135  0  0.243  0.263  0.738  0.439  0.09  0.159  0.069  1.164  0.036  0.327  0.222  0.123  0.065  0.076  0  0.431  
 
 DQX1  165545  2  74745257  74753408  2p13.1  0.291  0  0.147  0  0.469  0.073  0.218  0.024  0.155  0.098  0.078  0.918  1.089  0.124  0.219  0  0.285  0.163  0.257  0.086  0.105  0.261  0.201  0  0  0.056  0.305  0.037  0  0.166  0  0.15  0.141  0.185  0.09  0  0.058  0.481  0.195  0.09  0.04  0.092  1.65  0.131  0.142  0.076  1.285  0.162  0.22  0.26  0.055  0.558  0.107  0.183  0.033  0  0  0  0.087  0.111  
 
 AUP1  550  2  74753774  74757024  2p13  4.511  3.407  4.07  3.766  5.027  3.945  6.198  4.385  4.676  4.806  5.032  5.382  4.958  4.721  5.03  4.79  4.658  1.071  5.948  4.78  5.356  5.153  4.672  5.501  5.437  4.705  4.896  4.882  5.159  5.477  5.865  6.138  5.615  3.667  4.915  5.737  5.275  5.051  5.106  5.649  5.545  5.132  5.34  4.315  4.038  5.028  4.789  4.362  4.452  5.636  4.55  4.084  4.604  4.573  5.26  4.694  4.634  5.167  4.528  4.875  
 
 HTRA2  27429  2  74756531  74760683  2p12  2.021  1.836  2.235  1.921  1.857  1.571  2.929  2.009  2.451  2.145  2.307  1.739  2.498  2.292  2.05  1.748  2.283  0.634  4.421  2.623  2.546  3.069  1.932  1.6  2.508  1.643  2.933  2.139  2.306  3.084  2.739  2.626  3.359  2.404  1.599  2.333  2.288  1.677  3.159  2.53  1.849  2.9  3.312  1.892  1.461  2.233  2.418  2.122  1.032  4.322  2.217  1.913  1.796  1.562  2.289  1.737  1.789  2.886  1.89  2.338  
 
 LOXL3  84695  2  74759385  74781091  2p13  1.507  0.552  3.497  0.559  0.575  1.106  0.814  1.003  1.276  5.859  0.767  0.402  0.787  0.989  1.735  0.385  0.591  0.358  0.74  0.42  0.168  1.211  1.051  0.16  0.473  4.258  3.182  2.65  2.836  0.741  1.069  7.002  3.371  2.007  0.408  0.468  0.88  1.001  1.106  0.732  0.328  1.057  2.592  0.357  0.653  0.672  1.002  0.966  0.099  1.993  0.26  0.613  0.637  0.286  0.489  0.383  0.83  0.345  0.719  0.907  
 
 DOK1  1796  2  74776146  74784678  2p13  2.338  1.153  1.27  0.752  1.111  0.657  2.922  2.451  2.154  2.801  2.989  1.364  2.403  2.061  2.272  1.682  1.73  0.32  3.37  2.083  1.725  1.89  0.955  0.976  1.391  1.153  2.854  1.828  1.578  1.409  1.202  3.25  2.39  1.438  0.972  1.641  2.846  2.784  2.044  1.355  1.079  2.622  2.879  0.552  0.811  1.447  2.925  1.212  0.28  2.388  0.506  1.307  2.554  2.561  2.459  1.895  2.394  1.648  1.926  1.639  
 
 M1AP  130951  2  74785009  74875465  2p13.1  0  0  0.205  0  0.099  0  0.049  0.052  0.132  0.165  0.11  0.06  0  2.61  0  0.049  0  0  0  0.139  0  0.092  0.249  0  0  0  0.356  0.221  0.228  0.157  0.133  0.759  0.09  0.319  0  0  0.054  0  0.287  0  0  0.095  0.051  0.967  0.362  0.193  0.172  1.026  2.334  2.007  0.047  0.181  0  0.087  0.071  0  0.06  0.116  0.892  0  
 
 SEMA4F  10505  2  74881354  74910981  2p13.1  0.478  0.45  1.17  0.471  0.844  0.832  1.732  0.916  1.179  1.348  1.486  0.261  0.871  1.398  1.271  0.182  0.581  0.167  1.096  0.133  0.099  0.74  0.385  0.037  0.909  1.932  1.518  1.306  2.032  1.156  1.303  1.368  1.516  0.809  0.808  0.813  1.316  2.241  0.909  1.511  0.953  0.875  1.453  0.827  0.489  0.49  1.118  0.696  0.983  1.434  1.288  1.055  0.928  0.645  1.397  1.102  0.957  1.193  1.093  1.268  
 
 HK2  3099  2  75059781  75120481  2p13  2.034  2.342  1.877  2.606  0.871  2.132  1.609  1.377  1.999  2.018  2.099  1.283  2.263  1.492  2.525  1.34  2.977  3.528  2.577  1.863  2.043  1.749  1.453  1.605  2.213  2.869  2.987  2.858  4.729  1.971  2.648  3.579  3.699  1.933  0.272  1.014  1.838  1.468  1.532  1.681  2.369  1.272  2.275  2.712  1.655  2.068  1.455  0.674  1.157  1.265  3.27  1.254  1.559  1.387  1.627  1.341  1.901  2.371  1.031  1.13  
 
 LINC01291  102724515  2  75136257  75165545  2p12  0  0  0  0.997  0.663  0.567  0  0  0  0.269  0.216  0  0.337  0  0  0  0  0  0  0  0  0.314  0  0  0  0  0.344  2.248  1.187  0.425  1.027  1.659  0.997  0  0  0  0  0  0.911  0  0  0  0.451  0  0  0  0  0  0  0  0.345  0  0  0.294  0.213  0  0  0  0  0  
 
 POLE4  56655  2  75185774  75196859  2p12  4.301  6.254  4.596  4.168  3.892  5.027  6.339  4.148  3.793  4.781  5.004  3.71  3.912  3.538  3.116  3.803  3.1  4.309  5.33  5.987  3.489  5.151  3.657  3.981  4.976  3.789  4.534  3.676  6.252  4.314  4.955  5.172  4.683  3.728  4.418  4.08  4.06  3.866  5.134  4.79  4.789  4.446  5.901  3.726  3.51  3.918  6.532  4.368  4.133  6.51  4.385  3.619  1.927  3.941  5.155  4.09  2.957  5.366  3.891  3.925  
 
 TACR1  6869  2  75273589  75426645  2p12  0.256  0.048  0.231  0  0.033  0.102  1.313  0.187  0.268  0.329  1.81  0.095  0.094  0.047  0.073  0.077  0.064  0.096  0.154  0.116  0  0.191  0.354  0  0  0.091  0.245  0.158  0.34  0.096  0.061  0  0.154  0.314  0.051  0.244  0.154  0.161  0.287  0.082  0.122  0.27  0.054  0.094  0.102  0.989  0.086  0.282  0.746  0.038  0.113  0.306  0.038  0.124  0.133  0.121  0.084  0.22  0.133  0  
 
 EVA1A  84141  2  75719443  75796848  2p12  0  3.111  2.938  2.939  0  2.619  2.863  1.324  3.333  2.236  3.639  0.535  0  0.243  0.183  0.064  0.541  1.306  0.051  0.088  0  0.267  0.183  0  2.153  2.45  2.489  0.855  2.683  0.299  2.941  1.984  2.356  2.098  0.441  1.132  2.449  2.017  2.537  0  0.551  1.625  0.076  0.984  1.764  1.473  1.606  1.141  1.79  1.769  2.821  0.763  1.237  1.677  3.878  2.757  2.195  0.418  2.662  1.778  
 
 LOC101927884  101927884  2  75751193  75769832  2p12  0  0.151  0.201  0  0  0.347  0.38  0.55  0.179  0.296  0.718  0.416  0  0.083  0  0  0  0  0  0  0  0  0.407  0  0  0.737  0.883  0.245  0.552  0.074  0.668  0.14  0.467  0.228  0.468  0.38  0.452  0.579  0.178  0  0.096  0  0  0.208  0.241  0.279  0  0  0.303  0.07  0.441  0  0.182  0.213  0.925  0.237  0.477  0  0.568  0.208  
 
 MRPL19  9801  2  75873908  75889334  2p11.1-q11.2  2.016  3.243  1.35  1.057  1.975  1.393  2.041  1.458  1.761  1.59  2.367  1.28  2.122  2.853  1.948  1.768  1.78  3.692  2.88  2.386  1.801  1.76  1.102  0.884  2.08  2.08  1.398  1.26  1.525  1.242  2.326  2.131  1.735  1.323  1.86  2.168  1.815  1.802  2.348  2.164  2.098  1.819  2.778  1.942  1.387  1.447  0.902  1.197  1.874  2.609  2.051  1.216  1.583  1.222  2.416  1.762  1.759  2.156  1.568  2.245  
 
 GCFC2  6936  2  75889831  75938111  2p12  1.886  3.44  1.008  1.205  1.867  1.168  1.43  1.296  1.093  1.056  1.994  0.933  1.568  2.245  1.181  1.526  1.542  3.958  2.95  1.772  1.572  2.18  1.128  1.481  2.167  1.749  1.343  1.396  1.761  1.446  2.639  1.618  2.019  1.493  1.559  2.028  1.383  1.436  2.168  2.285  2.256  1.781  2.393  1.769  1.127  1.373  1.464  0.958  1.467  2.537  1.813  1.566  1.154  0.883  2.113  1.52  1.02  2.185  1.304  1.567  
 
 LRRTM4  80059  2  76974849  77749502  2p12  0  0  0.093  0  0.035  0  0  0.026  0.058  0.035  0  0  0  0.031  0  0  0  0.105  0  0  0  0.056  0.221  0  0  0.098  0.111  0.03  0  0.114  0.129  0.055  0.2  0.324  0  0.149  0  0  0.04  0  0  0.419  0.033  0  0.087  0.032  0.081  0  0  0  0  0.311  0  0.089  0.06  0  0  0.119  0  0  
 
 LOC101927907  101927907  2  77213090  77236914  2p12  0  0  0  0  0  0  0  0  0  0  0  0  0  0  0  0  0  0  0  0  0  0  0  0  0  0  0  0  0  0  0  0  0  0.182  0  0  0  0  0  0  0  0  0  0  0  0  0  0  0  0  0.115  0  0  0  0  0  0  0  0  0  
 
 LOC101927967  101927967  2  77970821  78517857  2p12  0  0  0  0  0  0  0  0  0  0  0  0  0  0  0  0  0  0  0  0  0  0  0  0  0  0  0.086  0.158  0  0.107  0  0  0  0  0  0  0  0  0  0  0  0.217  0  0  0  0  0.129  0  0  0  0  0.294  0  0  0  0  0  0  0  0  
 
 SNAR-H  100170221  2  78182032  78182152  2p12  5.338  0  0  0  0  0  0  0  0  0  0  0  0  0  0  0  0  0  0  0  0  1.907  0  0  0  0  0  0  0  0  0  0  0  0  0  0  0  0  0  0  0  0  0  0  0  0  0  0  0  0  0  0  0  0  0  0  0  0  0  0  
 
 LOC101927948  101927948  2  78315855  78354932  2p12  0  0  0  0  0  0  0  0  0  0  0  0  0  0  0  0  0  0.339  0  0  0  0  0.279  0  0  0  0  0.202  0  0.093  0  0  0  0.209  0  0  0  0  0  0  0  0.126  0  0  0  0  0  0  0  0  0  0.334  0  0  0  0  0  0.16  0  0  
 
 REG3G  130120  2  79252811  79255630  2p12  0  0  0  0  0  0  0  0  0  0  0  0  0  0  0  0  0  0  0  0  0  0.119  0  0  0  0  0.069  0  0  0  0  0  0  0  0  0  0  0  0  0  0  0.646  0  0  0  0  0  0  0  0  0  0  0  0  0  0  0  0  0  0  
 
 REG1A  5967  2  79347583  79350545  2p12  0  0  0  0  0  0  0  0  0  0  0  0  3.4  0  0  0  0  0  0  0  0  0  0  0  0  0  0.059  0  0  0.073  0  0.268  0  0  0  0.2  0  0  0  0  0  0  0  0  0  0  0  0  0  0  0  0.259  0  0  0  0  0  0  0  0  
 
 REG1CP  5969  2  79362628  79365553  2p12  0  0  0.096  0  0  0  0  0  0.123  0.018  0  0  0  0  0  0  0  0  0  0  0  0.033  0.232  0  0  0  0.046  0  0  0.033  0  0  0  0.227  0  0  0  0  0.019  0  0  0.124  0.033  0  0  0  0.027  0  0  0  0  0.087  0  0.029  0  0  0  0  0  0  
 
 REG3A  5068  2  79384131  79386880  2p12  0  0  0  0  0  0.187  0.149  0  0  0  0  0  0.542  0  0  0  0  0  0  0  0  0.072  0  0  0  0  0.058  0  0  0  0  0  0  0  0  0  0  0  0  0  0  0  0  0  0  0  0  0  0  0  0  0  0  0  0  0  0  0  0  0  
 
 LOC101927987  101927987  2  79720841  79727970  2p12  0  0  0  0  0  0  0  0  0  0  0  0  0  0  0  0  0  0  0  0.151  0  0  0  0  0  0  0  0  0  0.058  0  0  0  0  0  0  0  0  0  0  0  0  0  0  0  0  0  0  0  0  0  0  0  0  0  0  0  0  0  0  
 
 CTNNA2  1496  2  79740059  80875988  2p12-p11.1  0  0.052  0.144  0  0.023  0  0.183  0.408  0.106  0.952  0.027  0.177  0  0.028  0  0.035  0  0.296  0.061  0.047  0  0.162  0.17  0  0  0.076  0.196  0.148  0  0.125  0  0  0  0.305  0.055  0  0  0  0.06  0  0  0.413  0.055  0  0.075  0.032  0.091  0  0  0.137  0.038  0.521  0  0.161  0.063  0.06  0  0  0.084  0  
 
 LRRTM1  347730  2  80529002  80531487  2p12  0  0.039  0.133  0  0.051  0.067  0.067  0.088  0.231  0.29  0  0.469  0  0.041  0  0  0.057  0.381  2.953  0.113  0  0.355  0.375  0  0  0  0.261  0.044  0  0.127  0  0.153  0  0.317  0.071  0  0  0  0.106  0.121  0.048  0.443  0.066  0  0.119  0.059  1.004  0.075  0  0.123  0  1.269  0.039  0.136  0.152  0  0  0.09  0  0  
 
 LOC100507201  100507201  2  81688482  81694070  -  0  0  0  0  0  0  0  0  0  0  0  0  0  0  0  0  0  0  0  0  0  0  0  0  0  0  0  0  0  0  0  0  0  0  0  0  0  0  0.056  0  0  0  0  0  0  0  0  0  0  0  0  0.256  0  0  0  0  0  0  0  0  
 
 LOC1720  1720  2  83083926  83084893  2p12  0  0.162  0  0  0  0  0  0  0  0.118  0  0  0  0.165  0  0  0  0  0.109  0.112  0  0.216  0.372  0  0  0  0.078  0  0  0.116  0  0  0  0  0.136  0  0  0  0.074  0  0  0  0  0  0  0  0.863  0  0  0  0  0.43  0  0.098  0.133  0.145  0  0  0.191  0  
 
 FUNDC2P2  388965  2  84517805  84519324  2p11.2  0.629  0.532  0.427  0.774  0.561  0.436  0.598  0.085  0.863  0.608  0.359  0.404  0.227  0.42  0.252  0.745  0.415  0.443  0.471  1.426  0.252  1.096  0.847  0.507  0.209  0.101  0.419  0.314  0.211  0.147  0.285  0.149  0.248  0.511  0.37  0.126  0.226  0.202  0.795  0.179  0  0.733  0.859  0.223  0  0.159  0.373  0.166  0  0.364  0.301  0.258  0.476  0.604  0.316  0.455  0.193  0.632  0.301  0.081  
 
 SUCLG1  8802  2  84650646  84686586  2p11.2  4.745  5.145  3.228  3.227  4.564  2.926  5.127  3.578  3.694  3.918  4.377  4.401  4.358  4.595  3.594  3.924  4.967  5.312  6.062  5.601  4.315  4.923  3.69  3.456  4.644  3.814  4.158  3.297  4.127  4.475  5.323  5.217  4.512  3.761  4.048  4.289  3.588  3.672  4.991  4.376  3.694  3.989  4.727  3.773  3.165  4.111  4.973  3.473  3.481  5.64  4.127  2.288  3.384  3.301  4.566  3.795  3.332  4.211  3.232  4.069  
 
 DNAH6  1768  2  84743578  85046713  2p11.2  0.073  0.032  0.053  0.034  0.032  0.034  0.051  0.02  0.061  0.03  0.038  0.018  0.065  0.195  0.06  0.043  0.098  0.066  0.019  0.042  0  0.081  0.085  0  0.065  0.021  0.093  0.51  0.031  0.695  0.029  0.037  0.032  0.133  0.059  0.033  0.015  0  0.082  0.049  0.01  0.097  0.055  0.032  0.046  0.03  0.054  0.03  0.021  0.062  0.028  0.117  0.049  0.057  0.145  0.067  0.032  0.062  0.029  0.037  
 
 TRABD2A  129293  2  85048790  85108369  2p11.2  0.59  0.48  1.318  3.386  0.259  0.947  2.05  1.644  1.086  0.637  0.353  0.466  2.963  1.119  1.999  1.051  3.705  0.206  0.311  0.678  0  0.971  0.476  0.22  1.349  0.901  0.802  0.322  0.537  1.164  0.718  0.237  0  0.624  1.339  0.27  1.426  0.298  1.381  1.613  0.156  1.253  1.458  0.269  0.579  0.288  1.117  1.767  0.413  2.743  0.362  1.753  2.612  1.537  2.506  2.316  2.442  2.384  1.056  0.986  
 
 TMSB10  9168  2  85132762  85133799  2p11.2  8.631  10.238  10.281  10.893  8.423  10.636  10.793  8.755  9.831  9.559  9.021  8.986  8.362  10.212  7.846  10.668  11.775  6.558  9.624  9.17  6.881  10.252  7.551  7.297  10.72  8.14  9.157  6.854  7.868  7.699  9.026  8.857  7.607  7.56  10.459  9.853  8.376  9.034  11.223  9.243  8.786  10.492  10.899  10.048  9.015  9.527  11.153  9.882  9.736  12.011  9.239  8.144  9.831  10.156  12.089  10.261  9.2  11.265  9.76  9.465  
 
 KCMF1  56888  2  85198230  85286595  2p11.2  2.527  3.582  2.136  1.481  1.874  1.621  3.435  2.006  2.172  1.872  2.658  1.511  2.286  3.099  1.859  2.266  2.85  2.975  3.065  1.979  1.621  2.207  2.01  1.209  2.421  1.904  1.832  2.129  2.114  1.915  2.556  2.62  1.982  1.12  1.89  2.738  2.844  1.975  2.821  3.357  2.711  1.612  3.52  2.297  1.644  2.334  2.428  1.937  2.62  3.232  2.432  1.485  2.134  1.694  2.774  2.452  2.083  2.924  2.29  2.273  
 
 TCF7L1  83439  2  85360582  85537511  2p11.2  1.409  0.118  2.688  1.751  4.419  1.593  0.383  1.138  2.174  1.601  1.745  0.205  0.441  2.178  1.86  0.305  0.167  0.079  0.257  0.066  0.102  0.426  0.558  0.095  0.793  2.292  2.41  1.33  1.634  1.87  1.908  1.969  0.448  0.196  1.322  3.018  0.943  0.151  1.997  2.142  1.801  0.794  3.843  2.448  2.372  2.452  0.869  1.884  2.352  2.274  0.114  1.273  0.234  1.303  0.586  1.906  0.65  1.736  0.315  0.235  
 
 TGOLN2  10618  2  85545140  85555419  2p11.2  3.854  4.604  3.421  3.244  3.224  2.948  5.162  4.14  3.884  4.659  4.012  3.214  3.938  3.523  3.704  4.034  3.531  2.751  4.353  3.565  2.934  3.389  2.392  2.212  3.988  4.578  3.614  3.905  4.177  2.739  3.642  4.193  4.262  3.093  3.389  4.241  4.054  4.394  4.564  5.132  4.194  3.035  3.782  3.607  2.811  3.463  4.079  2.941  4.149  3.65  3.914  2.597  3.745  4.043  4.606  3.713  4.161  4.358  3.782  4.785  
 
 RETSAT  54884  2  85569077  85581821  2p11.2  2.242  2.152  1.953  2.264  2.199  1.487  3.317  1.795  2.645  1.897  2.344  2.34  3.189  3.172  2.203  2.206  2.969  1.525  2.528  1.242  1.298  1.528  1.481  1.321  2.135  4.489  4.019  3.039  3.233  3.141  3.351  2.817  2.741  1.532  2.192  2.447  2.382  2.66  2.434  2.37  1.525  1.806  2.636  1.981  1.98  2.184  2.82  1.854  2.034  3.2  3.451  1.491  1.929  2.148  2.992  2.677  2.061  1.882  2.16  3.041  
 
 ELMOD3  84173  2  85581842  85618875  2p11.2  1.512  0.519  0.918  1.012  1.348  0.642  2.15  0.766  1.819  1.704  1.644  0.896  1.324  0.91  1.218  0.886  0.707  0.14  2.634  0.682  0.928  1.324  0.937  0.289  1.25  1.492  1.527  0.823  1.512  0.791  1.371  1.604  1.449  0.779  1.041  1.262  1.4  1.593  1.605  1.655  1.218  1.377  1.651  0.694  0.862  1.16  1.067  1.057  0.975  1.91  1.577  0.726  0.977  1.35  1.482  0.825  1.47  1.225  0.874  0.771  
 
 CAPG  822  2  85621870  85641197  2p11.2  1.427  2.03  4.587  5.737  4.244  2.101  2.808  3.521  0.282  1.655  1.394  4.105  3.031  4.435  1.733  5.776  5.134  0.391  5.492  2.61  1.825  4.135  0.784  0.263  0.901  7.875  4.678  4.13  8.651  0.72  7.227  3.909  0.382  0.392  2.905  4.998  4.631  6.89  1.689  1.458  3.059  3.214  0.82  0.775  3.202  3.416  3.971  4.933  3.766  6.868  4.451  3.169  5.479  5.748  6.241  5.599  5.829  4.503  5.769  4.388  
 
 SH2D6  284948  2  85661917  85664152  2p11.2  0  0  0.157  0.4  0.193  0  0.364  0  0.319  0.182  0  0.27  0  0.373  0  0.161  0  0  0.14  0  0  0.184  0.38  0  0  0.204  0.188  0.31  0  0.08  0.493  0.287  0  0  0.156  0  0.11  0.277  0.151  0  0  0.313  0.055  0  0  0.198  0.628  0  0  0.205  0.223  0.222  0.106  0  0.158  0  0  0  0  0  
 
 PARTICL  100630918  2  85764589  85766009  -  1.299  0.707  0.465  0.548  0.97  0.838  0.934  0.175  0.531  0.536  0.399  0.194  0.678  1.101  0.347  1.044  0.468  0.632  1.931  0.645  0.369  1.127  0.752  0.152  0.672  0.503  0.613  0.775  0.688  0.273  0.926  0.812  0.681  1.015  0.759  1.314  0.591  0.488  0.709  1.171  1.338  0.834  1.811  0.758  0.528  0.484  0.652  0.525  0.341  1.126  0.753  0.762  0.384  0.483  1.024  0.45  0.526  1.286  0.523  0.504  
 
 MAT2A  4144  2  85766100  85772403  2p11.2  5.133  5.276  3.414  4.99  4.934  3.465  5.842  4.325  5.07  4.802  5.044  4.026  4.508  5.921  4.679  5.291  3.751  5.168  7.28  5.585  4.904  6.058  4.127  4.02  4.32  4.61  4.886  4.169  5.038  4.027  5.282  5.529  4.984  3.502  4.554  5.111  4.746  4.363  5.04  5.299  4.054  4.515  5.381  4.346  4.183  4.183  5.535  4.141  4.598  6.384  4.815  3.264  4.704  3.961  5.422  4.363  4.225  5.844  4.315  4.544  
 
 GGCX  2677  2  85771977  85788657  2p12  1.554  1.566  1.612  1.454  1.872  1.304  2.099  1.139  1.64  1.828  1.586  0.97  1.295  1.357  1.138  1.35  0.925  1.17  1.517  1.499  0.741  2.018  1.228  0.928  1.525  1.843  1.236  1.406  2.101  1.272  2.007  2.063  1.706  1.066  1.52  1.87  1.851  1.545  2.161  2.062  1.596  1.37  1.291  1.321  1.805  1.656  1.701  1.041  1.559  2.22  1.546  0.821  1.5  1.315  1.765  1.985  1.495  1.855  1.112  1.453  
 
 VAMP8  8673  2  85804613  85809156  2p12-p11.2  5.247  5.328  0.284  0  6.117  0.219  0.792  0  0.71  0.198  0.822  4.997  4.675  4.857  3.463  5.638  4.841  2.6  6.504  7.706  4.71  0.305  5.777  5.643  0.234  2.966  0.135  1.826  4.237  3.354  2.014  1.018  0  0  3.04  3.174  3.404  4.405  2.547  2.887  4.906  3.298  3.073  3.701  4.264  5.203  5.897  3.162  3.24  5.791  3.714  2.616  3.751  4.928  5.363  3.905  3.712  2.349  3.981  4.493  
 
 VAMP5  10791  2  85811530  85820511  2p11.2  1.908  0  2.017  0  1.18  4.223  6.969  0  1.54  3.049  2.045  1.223  0.957  0.726  1.765  3.887  0.927  0.263  6.392  2.823  0  4.608  0.877  0.764  0  5.02  6.508  2.955  2.848  0.251  0  2.346  5.02  4.751  0.269  2.23  3.218  5.509  0.338  0.505  0.859  0.42  0.771  0  0.358  1.944  6.069  0.703  0  1.164  0.253  0.962  2.896  1.926  5.727  3.26  3.895  0.345  2.558  4.799  
 
 RNF181  51255  2  85822836  85824831  2p11.2  5.662  7.222  4.577  4.012  5.916  5.206  6.708  4.587  4.159  4.738  5.137  4.481  4.034  5.193  3.47  4.798  5.312  5.714  6.537  6.504  4.717  5.59  4.351  4.279  5.475  3.997  4.853  3.96  4.824  4.311  5.226  5.514  4.163  3.143  5.078  4.678  4.531  4.607  7.108  4.862  5.126  4.361  5.876  4.083  5.049  5.101  6.592  4.342  3.731  6.534  4.857  3.546  4.677  4.614  6.154  4.889  3.979  6.364  4.477  4.682  
 
 TMEM150A  129303  2  85825669  85829822  2p11.2  2.032  0.264  1.554  1.711  2.547  1.458  2.721  1.009  2.626  1.313  1.214  0.676  2.005  2.023  1.193  2.225  1.215  0.274  1.013  0.969  1.944  0.817  3.025  2.269  1.42  2.709  2.388  1.59  2.359  2.157  3.128  2.863  2.16  1.83  1.348  3.239  2.035  2.266  1.148  2.499  1.56  1.547  3.247  1.817  1.369  2.186  1.731  1.269  1.328  3.393  1.318  0.941  0.892  1.639  2.293  1.733  1.486  2.204  1.053  1.5  
 
 USP39  10713  2  85829964  85876407  2p11.2  4.039  4.073  2.704  2.576  3.091  2.614  4.385  2.611  3.286  3.052  3.823  2.749  3.676  4.664  3.249  3.253  3.627  3.494  5.72  4.531  3.755  4.294  2.69  2.519  3.56  3.38  3.224  3.342  3.36  3.36  4.015  4.129  3.774  2.233  3.259  3.39  3.591  3.367  3.82  4.415  3.361  2.854  4.324  3.289  3.645  3.132  4.187  2.81  3.187  4.495  3.646  2.331  2.744  2.583  3.739  2.903  2.958  3.89  2.68  3.195  
 
 C2orf68  388969  2  85832375  85839179  2p11.2  2.233  1.579  1.223  1.362  2.465  1.674  1.606  1.298  1.904  1.784  1.881  1.908  1.572  1.767  1.748  2.528  1.856  1.197  3.244  1.873  1.555  1.989  1.834  1.467  1.484  0.889  1.345  1.514  1.207  0.852  1.758  1.608  1.961  1.374  1.869  2.084  1.438  1.35  1.532  1.949  2.383  1.74  2.187  1.353  2.176  1.508  1.758  0.807  1.581  1.639  1.54  1.085  1.12  1.018  2.166  1.782  1.568  1.979  1.365  1.212  
 
 SFTPB  6439  2  85884439  85895864  2p12-p11.2  0  0  0.103  0  0.059  0.042  0.052  0.031  0.145  0.063  0.034  0.048  0.057  0.092  0.064  0.045  0  0.099  0.063  0.082  0.084  0.142  0.407  0.04  0  0  0.106  0.029  0.053  0.088  0  0  0  0.257  0.123  0.083  0.043  0  0.131  0  0  0.312  0.058  0.042  0.114  0.045  0.173  0.052  0  0  0.054  0.607  0.026  0.097  0.055  0.052  0  0  0  0  
 
 GNLY  10578  2  85921413  85925974  2p11.2  0  0  0  0  0  0  0  0  0  0  0  0  0  0  0  0  0  0  0  0  0  0.289  0.31  0  0  0  0.092  0.324  0  0.092  1.041  0.268  0  0  0  0  0  0  0  0  0  0  0.053  0  0  0  0  0  0  0  0  0.383  0  0  0  0  0  0  0  0  
 
 ATOH8  84913  2  85980908  86018506  2p11.2  0.178  0.067  0.789  0.582  0.043  0.158  1.61  0.903  0.2  1.119  0.063  0.087  0.128  0.174  0.314  0.047  0.066  0.187  0  0.096  0  0.125  0.442  0.024  0.037  0.227  1.129  1.098  0.196  0.698  1.373  1.446  0.043  0.273  0.231  1.352  0.433  0.247  0.145  0.078  0.019  0.756  0.045  0.039  0.433  0.129  2.071  0.149  0  0.13  0.043  0.571  1.213  0.452  0.052  0.074  0.867  0.116  0  0.091  
 
 LOC284950  284950  2  86042252  86053911  2p11.2  0  0.051  0.124  0  0  0.077  0.095  0.112  0.069  0.038  0.038  0  0.067  0.022  0  0.027  0.041  0.058  0.027  0.063  0  0.092  0.228  0  0  0.045  0.079  0.038  0.042  0.099  0.105  0.146  0  0.165  0  0.209  0.06  0.073  0.062  0  0.09  0.149  0.04  0  0.099  0.143  0.155  0.065  0  0  0.053  0.063  0.095  0.04  0.029  0  0.051  0.071  0  0  
 
 ST3GAL5  8869  2  86066270  86116157  2p11.2  0.951  0.967  0.987  0.136  0.165  1.097  2.079  1.361  1.531  1.716  0.749  0.731  0.128  1.733  0.287  0.6  0.258  0.357  2.151  0.376  0.624  1.404  1.622  1.01  0.56  3.516  3.955  2.804  3.778  2.266  3.903  3.12  3.527  2.437  2.233  3.432  1.449  1.414  0.811  1.359  0.46  1.18  0.059  0.376  0.243  0.286  3.558  0.382  0  1.385  0.145  0.736  0.856  0.469  0.655  0.491  0.544  0.551  0.56  1.269  
 
 ST3GAL5-AS1  101928113  2  86116402  86119375  2p11.2  0  0  0  0  0  0  0  0  0.111  0  0  0  0  0  0  0  0  0  0  0  0  0.073  0.484  0  0  0  0.297  0.232  0.205  0.083  0.314  0.333  0.195  0.257  0  0.362  0  0  0.08  0  0  0.091  0  0  0  0  0  0  0  0  0  0.174  0  0  0  0  0  0  0  0  
 
 LOC90784  90784  2  86247338  86250991  2p11.2  1.875  1.393  0.68  1.13  1.198  1.172  2.478  1.026  1.49  1.583  2.12  1.444  1.947  1.917  1.681  1.125  1.189  0.904  2.47  2.129  1.679  2.236  1.484  1.66  1.402  1.955  2.176  2.053  2.339  2.074  2.102  2.673  2.215  1.148  2.255  2.581  1.148  1.983  2.057  3.278  1.859  1.47  2.681  2.477  1.163  1.851  2.374  1.744  1.448  2.153  1.018  1.164  1.462  1.642  1.554  1.009  1.119  1.125  1.171  1.146  
 
 POLR1A  25885  2  86253450  86333278  2p11.2  2.689  1.21  1.549  1.296  1.496  1.883  2.5  1.664  1.302  1.958  2.285  1.476  2.808  2.458  2.886  2.017  1.507  0.347  1.967  1.839  3.033  1.929  0.797  1.997  2.515  2.456  2.204  2.945  2.94  2.489  2.5  2.935  3.058  0.95  2.19  2.851  2.841  2.894  1.376  4.236  2.44  1.515  2.909  2.77  1.96  2.474  1.377  2.338  2.693  1.961  2.518  1.602  2.311  1.803  2.248  2.02  2.443  2.632  2  2.745  
 
 PTCD3  55037  2  86333304  86369280  2p11.2  3.092  3.72  1.148  1.632  2.145  1.21  3.007  1.415  1.968  1.861  2.33  2.136  2.407  3.64  2.707  2.409  2.915  3.112  4.069  3.378  2.467  3.371  2.057  1.521  1.988  2.626  2.696  2.211  2.584  2.709  2.828  2.81  2.605  1.531  1.971  2.504  2.301  2.646  2.414  3.201  1.903  1.919  3.733  2.185  1.287  1.902  3.8  2.004  1.856  3.79  2.298  1.18  1.801  1.251  3.185  1.902  1.96  1.665  1.719  2.197  
 
 SNORD94  692225  2  86362992  86363129  2p11.2  0  0  0  0  1.696  0  0  0  0  0  0  0  0  0  0  2.092  0  0  2.649  0  0  0  0  0  0  0  0  0  2.691  0.919  0  0  0  0  0  0  0  0  1.244  0  0  0  0  0  0  1.89  0  0  0  2.556  0  0  0  0  0  0  0  0  0  0  
 
 IMMT  10989  2  86371054  86422893  2p11.2|2  4.55  5.618  3.079  3.333  3.754  2.751  4.484  3.315  3.562  3.391  4.145  3.377  3.952  5.268  3.902  4.078  4.369  5.919  5.611  5.048  4.038  4.15  3.549  3.049  3.832  4.026  3.819  3.703  4.343  4.573  4.855  4.84  4.562  3.65  3.463  4.185  3.839  4.069  4.086  5.287  3.332  3.511  4.913  4.049  2.963  3.875  4.34  3.42  3.366  5.457  3.883  2.534  3.329  2.861  4.373  3.636  3.685  3.693  3.177  4.157  
 
 MRPL35  51318  2  86426555  86440477  2p11.2  3.337  4.377  1.827  1.396  2.404  2.087  3.066  1.494  2.08  1.686  2.768  2.502  2.447  3.434  2.041  2.506  3.117  3.534  3.981  3.76  2.887  2.787  2.404  2.273  2.983  2.524  2.461  2.106  2.588  2.856  3.689  3.248  3.002  2.037  2.721  2.551  2.029  2.306  3.069  2.521  2.211  2.272  3.845  2.291  1.965  2.207  3.44  1.758  1.723  3.722  2.222  0.893  2.363  1.824  3.118  2.028  2.043  2.116  2.206  2.265  
 
 REEP1  65055  2  86441119  86565206  2p11.2  0.673  0.266  0.11  0.234  0.531  0.313  0.073  1.43  0.485  0.868  1.429  0.042  0.156  1.84  0.493  0.036  0.046  0.177  0.037  0.137  0  0.256  0.775  0.976  0  0.1  0.375  0.09  0.099  0.634  0.037  0.065  0.081  0.214  0.183  0.3  0.064  0.095  0.168  0.207  0.591  0.119  0.827  0.151  0.247  0.99  0.427  0.814  0.981  1.075  0.043  0.379  0.154  0.156  0.096  0  0.103  0.112  0  0  
 
 KDM3A  55818  2  86668270  86719839  2p11.2  1.838  2.659  1.158  1.098  1.249  1.436  1.443  1.928  2.344  2.375  2.822  1.012  1.91  2.196  1.704  1.571  2.77  2.523  3.146  2.012  1.266  2.187  1.335  1.607  1.951  2.208  2.111  2.538  2.633  1.965  2.161  2.637  2.329  1.031  1.35  2.02  1.53  2.124  1.956  2.903  1.967  1.218  2.681  2.246  0.931  1.977  1.995  1.242  1.903  2.475  2.239  1.455  1.35  1.325  2.276  2.149  2.515  1.824  1.827  1.72  
 
 CHMP3  51652  2  86730552  86790620  2p11.2  3.987  5.437  3.255  2.276  3.949  3.016  4.685  3.383  4.02  3.988  3.847  3.475  3.538  4.228  2.995  3.604  3.685  3.766  4.296  3.547  2.374  2.781  2.373  1.514  3.888  3.877  3.525  2.703  2.834  3.089  3.91  3.424  3.671  3.125  3.332  4.13  3.269  4.359  4.346  4.461  3.969  2.955  4.641  3.123  2.648  3.795  3.975  2.765  4.084  4.381  4.106  2.233  3.505  3.352  4.162  3.164  4.022  3.36  3.487  3.788  
 
 RNF103-CHMP3  100526767  2  86730552  86948245  2p  0.581  0.1  0.447  0  0.208  0  0.152  0.252  0.138  0.232  0  0.145  0.82  0.322  0.306  0.293  0.39  0.89  0.531  0  0  0.686  0.315  0.159  0.093  0.057  0.712  0.03  0.44  1.352  0.409  0.294  0.419  0.253  0.109  0.16  0.156  0  0.083  0.433  0.455  0.117  0.064  0.153  0  0.378  0.305  0.131  0  0.1  0.25  0.682  0.143  0.081  0.445  0.602  0.182  0.642  0.355  0  
 
 RNF103  7844  2  86830515  86851000  2p11.2  2.548  3.524  1.156  1.177  2.485  0.93  2.165  1.861  2.737  2.044  1.984  1.103  2.394  2.533  1.696  1.504  1.609  2.653  2.486  0.762  0.537  1.247  1.881  0.641  1.56  1.79  1.578  1.372  1.749  1.063  1.583  2.033  1.384  1.55  1.418  2.331  1.206  1.683  2.162  2.049  2.332  0.973  2.223  1.1  0.715  1.68  1.606  0.748  1.894  2.674  2.122  0.97  1.28  1.396  1.842  1.771  1.874  1.092  1.203  2.004  
 
 RMND5A  64795  2  86947413  87005164  2p11.2  2.766  1.796  1.052  1.168  1.699  1.048  0.846  1.38  1.711  1.475  1.781  1.652  2.397  2.353  2.003  1.774  2.014  2.472  3.664  2.433  1.898  2.475  1.884  2.058  1.748  1.937  1.211  1.687  1.501  0.996  2.021  1.935  1.815  0.908  1.226  2.071  1.627  1.057  1.167  2.556  2.137  1.075  1.387  2.193  1.765  1.836  1.524  0.873  2.272  1.669  1.673  0.825  1.259  1.196  1.403  1.42  1.637  1.986  1.257  1.721  
 
 CD8A  925  2  87011727  87035519  2p12  0.624  0.165  0.185  0  0.149  0.142  0.099  0.041  0.099  0.048  0.046  0.043  0.236  0.206  0.314  0.083  0.071  0.17  3.492  0.065  0  1.571  0.667  0.306  0  0  0.14  0.03  0  0.219  0  0  0  0.21  0.242  0.102  0  0  0.135  0.407  0.136  0.523  0.462  0  0.172  0.078  0.735  0.121  0  0.566  0  0.607  0.062  0.132  0.126  0.097  0.041  0  0  0  
 
 CD8B  926  2  87042459  87089047  2p12  0  0  0.319  0  0  0  0  0  0.183  0  0  0  0  0  0  0  0  0.287  2.583  0  0  2.651  4.966  7.184  0  0  0.079  0  0  0.082  0  0  0  0.553  0  0  0  0  0  0  0  0.374  0.057  0  0  0  0.057  0  0  0  0  0  0  0  0  0  0  0  0  0  
 
 ANAPC1P1  100286979  2  87088947  87122165  2p11.2  0.582  0.341  0.109  0  0.155  0.186  0.244  0.16  0.112  0.202  0.357  0.232  0.705  0.389  0.82  0.309  0.441  0.158  0.125  0.433  0.157  0.523  0.451  0.586  0.47  0.404  0.343  0.447  0.692  0.766  0.3  0.534  0.632  0.205  0.435  0.408  0.602  0.395  0.187  0.795  0.322  0.49  0.793  0.135  0.216  0.22  0.359  0.493  0.091  0.196  0.342  0.334  0.675  0.357  0.721  0.583  0.565  0.5  0.369  0.254  
 
 RGPD2  729857  2  87140934  87239861  2p11.2  0  0.158  0  0.073  0.071  0.095  0.034  0.085  0.133  0.016  0.162  0.054  0.292  0.213  0.128  0.099  0.152  0.196  0.276  0  0.267  0.081  0  0  0.115  0.148  0.039  0.116  0.326  0.041  0.166  0.277  0.151  0.075  0.129  0.119  0.185  0  0.059  0.174  0.136  0.095  0.284  0.103  0.133  0.025  0  0  0  0.227  0.179  0.041  0.188  0.236  0.209  0.077  0.221  0.162  0  0.095  
 
 PLGLB1  5343  2  87237587  87248969  2p11.2  0.126  0.166  0.089  0  0  0.062  0.141  0.146  0.132  0.266  0.062  0  0  0.142  0.086  0  0  0.284  0.805  0.178  0  0.689  0.366  0.059  0  0  0.191  0  0  0  0  0  0.086  0.299  0  0.061  0  0  0.392  0  0  0.126  0  0  0  0.036  0.406  0  0  0.119  0  0  0  0.032  0  0  0  0  0.065  0  
 
 LOC285074  285074  2  87257797  87303536  2p11.2  2.046  2.844  1.697  1.821  1.311  1.052  2.108  1.533  2.069  1.696  1.977  1.343  2.065  2.401  1.975  1.95  1.884  2.679  3.95  2.9  1.623  3.049  1.201  1.276  1.67  1.601  1.842  1.84  2.059  1.901  2.167  1.889  2.436  1.091  1.717  2.003  2.451  1.776  2.685  2.378  1.806  1.564  1.785  1.568  1.355  1.175  1.991  2.038  1.376  3.15  1.909  1.288  1.577  1.606  2.252  1.48  1.936  2.15  1.832  1.695  
 
 LINC00152  112597  2  87754973  87821030  2p11.2  1.008  9.118  5.714  4.347  4.387  6.505  5.562  4.926  4.317  5.214  4.855  3.898  2.678  4.003  1.664  3.994  3.989  5.007  3.312  5.538  5.568  3.214  4.712  4.621  6.001  4.332  3.72  3.913  4.21  2.578  5.344  4.717  3.763  3.677  5.342  4.969  4.211  5.153  8.554  3.916  5.148  5.283  5.688  5.815  3.484  4.648  5.565  4.477  4.637  7.834  3.508  3.45  3.475  4.831  5.733  4.53  4.206  6.496  3.621  4.871  
 
 PLGLB2  5342  2  88047605  88058994  2p11.2  0  0  0.066  0  0  0  0  0  0.057  0  0  0  0  0  0.203  0  0  0  0  0  0  0  0.119  0  0  0  0  0  0  0.013  0  0  0.079  0  0  0  0  0  0  0  0  0  0  0  0  0.037  0  0.065  0  0  0  0  0  0.099  0.042  0  0  0  0  0  
 
 RGPD1  400966  2  88055478  88285309  2p11.2  0.388  0.07  0.1  0.318  0.019  0.135  0.11  0.102  0.146  0.206  0.052  0.124  0.173  0.107  0.172  0  0  0.388  1.539  0.163  0.087  1.954  0.286  0.229  0.141  0  0.168  0.03  0  0.08  0  0  0.192  0.27  0.027  0.125  0.08  0.041  0.113  0  0  0.052  0.213  0.028  0  0.11  0.044  0.036  0.126  0.178  0  0.052  0.058  0.317  0.038  0.066  0.048  0  0.106  0  
 
 KRCC1  51315  2  88326721  88355320  2p11.2  2.526  3.209  1.998  1.021  1.341  0.989  2.647  1.772  2.569  2.593  1.987  1.838  2.889  1.582  1.044  1.954  1.927  2.985  2.856  0.75  0.598  1.1  1.941  0.36  0.511  2.537  1.474  0.475  0.919  0.301  0.854  1.376  0.583  0.832  1.211  1.934  1.82  1.938  2.768  1.393  1.723  1.264  2.939  0.529  0.876  1.858  1.614  0.236  1.831  1.025  2.411  0.65  2.051  2.492  2.778  1.951  2.59  0  1.197  2.561  
 
 SMYD1  150572  2  88367298  88412902  2p11.2  0  0  0.142  0  0.055  0  0  0.035  0.13  0.051  0  0.04  0  0.019  0  0.021  0  0  0  0.044  0  0.053  0.245  0  0  0.041  0.155  0.068  0  0.092  0.031  0.118  0  0.266  0  0  0.023  0.06  0.05  0  0  0.103  0.04  0  0.063  0.032  0.016  0  0  0  0  0.326  0.024  0.06  0.041  0.06  0  0.087  0  0  
 
 FABP1  2168  2  88422507  88427650  2p11  0  0  0  0  0  0  0  0  0  0  0  4.978  0.787  0  0  0  0.763  1.424  0  0  0  0  0  0  0  0  0  0  0  0.108  0  0  0  0  0  0  0  0  0  0  0  0  0  0  0  0  0  0  0  0  0  0.651  0  0  0  0  0  0  0  0  
 
 THNSL2  55258  2  88469813  88486156  2p11.2  1.934  0  0.839  0.745  3.27  0  0  0.985  0.201  1.024  0.507  1.66  0.212  0  2.173  0.48  0  0.247  0  0  0  0.157  0.339  0  0  1.069  0.367  2.404  0.263  0.093  0.686  0.155  0  0.235  0  1.843  2.061  2.756  0.707  3.115  1.921  0.263  0.046  0.871  0.925  0.959  0.046  0.387  0.145  1.593  0.076  0.456  0  1.014  1.51  1.055  0.406  0  0  1.526  
 
 FOXI3  344167  2  88747725  88752053  2p11.2  0.243  0  0.193  0  0.548  0.421  0.175  0.426  0.166  0.173  0.121  0.21  0  0.127  0  0.251  0.289  0.096  0  0.2  0.192  0.475  0.543  0.19  0.163  0.109  0.233  0.22  0.19  0.307  0.217  0.244  0.252  0.295  0.15  0.134  0.111  0.136  0.161  0.386  0.577  0.664  0.128  0.135  0.768  0.623  0.115  0.204  1.188  0.103  0.188  1.351  0.104  0.798  0.265  0.208  0  0.316  0  0.123  
 
 TEX37  200523  2  88824168  88829103  2p11.2  0  0  0.367  0  0  0  0  0  0.162  0  0  0  0  0  0  0  0  0  0  0  0  0.096  0.319  0  0  0  0.121  0.373  0  0.063  0  0  0  0.296  0  0  0  0  0.162  0  0  0.114  0.318  0  0  0  0  0  0  0.175  0  0.246  0  0  0.117  0  0  0  0  0  
 
 LOC101928371  101928371  2  88838237  88875128  2p11.2  0.655  0.237  0.319  0  0.158  0  0.284  0.114  0.82  0.197  0.22  0  0  0.127  0.366  0  0.188  0  0  0.339  0  0.361  0.251  0.314  0  0.159  0.134  0.175  0.447  0.17  0  0.228  0.242  0.393  0  0  0.201  0.277  0.055  0  0  0.238  0  0  0.138  0.299  0.073  0.28  0  0  0.196  0.14  0.058  0.202  0.151  0.17  0.307  0  0  0  
 
 EIF2AK3  9451  2  88856258  88927094  2p12  1.121  1.04  0.578  0.523  1.027  0.395  1.074  1.311  1.396  1.273  1.462  0.937  0.928  1.151  1.122  0.632  1.254  0.78  1.909  0.922  0.496  0.917  1.095  0.858  0.511  1.52  0.855  0.718  1.258  0.981  1.239  1.157  1.393  0.573  0.468  1.449  1.042  1.442  1.26  1.315  1.151  0.859  0.83  1.1  0.685  1.205  0.435  0.49  1.019  1.346  1.494  0.527  0.759  1.223  1.225  1.03  1.373  0.736  0.605  1.382  
 
 RPIA  22934  2  88991175  89050452  2p11.2  2.642  4.045  1.294  1.737  1.519  1.706  2.125  1.145  1.485  1.552  2.115  1.991  2.961  3.365  1.845  2.5  2.621  4.147  5.114  4.217  2.433  3.861  2.398  1.497  2.884  1.665  1.957  1.902  1.402  1.503  2.372  2.113  2.628  1.812  1.799  1.677  2.539  1.99  2.559  3.156  2.683  2.056  2.832  2.459  1.115  1.455  2.403  1.283  1.611  2.98  2.199  1.179  1.877  0.928  2.926  2.163  1.362  2.645  1.8  2.273  
 
 ANKRD36BP2  645784  2  89065418  89106109  2p11.2  0  0  0  0.32  0  0  0.138  0  0  0.047  0  0.066  0  0.07  0  0.058  0  0.122  0  0.064  0  0.239  1.912  1.359  0  0  0.068  0  0.166  0.099  0  0  0  0.129  0  0  0.078  0  0.167  0.305  0  0.043  0  0  0  0  0.063  0.518  0  1.445  0.061  0.222  0.109  0.1  0.076  0.193  0.558  0  0  0.334  
 
 LOC654342  654342  2  91824708  91847975  2p11.1  3.118  4.135  3.791  0  1.181  2.282  2.889  3.054  0  3.2  1.777  3.233  2.295  2.777  2.938  2.263  0  4.533  2.284  0.248  2.201  3.39  1.736  2  5.983  2.002  3.455  1.079  3.859  2.66  4.178  4.578  3.566  2.384  3.367  2.883  4.731  3.247  5.041  4.286  3.073  1.691  4.82  0.352  1.719  1.779  4.898  4.794  2.665  6.847  4.806  2.682  3.631  3.581  2.633  4.799  3.495  5.971  1.661  4.757  
 
 GGT8P  645367  2  91963367  91970153  2p11.1  0  0  0.148  0  0.051  0.063  0.675  0.243  0.29  0.031  0.188  0.315  0.198  0.025  0  0.038  0  0  0  0  0  0.055  0.491  0.087  0.401  0.078  0.06  0.075  0  0.057  0  0  0  0.303  0.991  0.13  0.122  0.845  0.181  0  0  0.285  0.035  0  0  0  0.183  0.089  0  0.221  0  0.235  0.938  1.057  0.525  0.207  1.025  0.111  0.218  0.614  
 
 ACTR3BP2  440888  2  92129158  92130496  2p11.1  0  0  0.12  0  0  0  0  0  0  0  0  0  0  0  0  0  0  0  0  0  0  0  0.267  0  0  0  0  0  0  0.063  0  0  0  0.327  0  0  0  0  0  0  0  0.115  0.074  0  0  0  0  0  0  0  0  0  0  0  0  0  0  0  0  0  
 
 FAM95A  90499  2  95421070  95426929  2q11.1  0  0  0.164  0  0.114  0  0  0  0.175  0.087  0  0.057  0  0.024  0  0  0  0  0.075  0.065  0.208  0.112  0.489  0  0  0  0.087  0.859  0.143  0.055  0.357  0.095  0.06  0.406  0.062  0  0  0  0.034  0  0  0.522  0  0  0  0.037  0.023  0.419  0  1.435  0  0.153  0  0  0.141  0  0  0.175  0  0  
 
 ANKRD20A8P  729171  2  95426672  95522820  2q11.1  0  0.024  0.088  0  0.1  0  0.026  0  0.049  0.104  0.034  0.029  0  0.137  0.043  0  0  0.259  0.051  0  0.106  0.083  0  0  0  0  0.124  0.439  0.034  0.053  0  0.054  0.045  0.138  0  0  0  0  0.025  0  0.048  0.115  0.056  0.029  0  0  0.264  0.139  0  0.184  0.032  0.075  0.024  0.042  0.079  0  0  0  0  0  
 
 LOC442028  442028  2  95534429  95613087  2q11.1  0  0.021  0.154  0  0.051  0  0.033  0  0.128  0.166  0.031  0.037  0.052  0.075  0.047  0.045  0  0.046  0.041  0  0  0.052  0.366  0  0  0.044  0.111  0.566  0  0.075  0.038  0.037  0  0.201  0.05  0  0  0  0.06  0  0.032  0.055  0.039  0  0  0.022  0.034  0.049  0  0.026  0.033  0.289  0.027  0.094  0.031  0.046  0  0  0  0  
 
 TEKT4  150483  2  95537177  95542574  2q11.1  0.364  0.101  0.202  0  0.35  0.108  0.622  0.164  0.424  0.315  0.132  0.352  0.108  0.282  0  0.087  0.223  0.271  0.064  0.237  0.241  0.914  0.821  0.202  0.152  0.357  0.414  0.36  0.28  0.165  0.227  0.445  0.213  0.286  0.166  0.309  0.089  0  0.819  0.294  0.325  0.841  0.082  0.097  0.467  0.111  0.295  0.131  0  0.465  0.088  1.329  0  0.595  0.331  0.263  0.095  0.355  0  0.212  
 
 MAL  4118  2  95691399  95719737  2q11.1  0  0  0.409  0  0  0  1.109  0  0.506  2.788  0.435  0  0  0  0  0  0  0  2.963  0  0  7.932  7.046  4.993  0  3.05  0.198  2.605  0  0.136  0.206  0  0  0.223  0  0  0  0  0.084  0  0.221  0  0  3.221  3.649  2.272  0.089  0.312  6.12  0  0  0.412  0.119  0.206  5.507  5.611  0.596  0  3.141  3.242  
 
 MRPS5  64969  2  95752951  95787754  2p11.2-q11.2  4.455  3.667  2.966  3.361  3.41  3.832  4.354  3.723  3.66  3.254  4.025  4.139  3.836  4.444  3.839  3.907  4.367  2.842  5.571  4.982  4.19  4.833  3.712  3.555  4.558  3.383  4.02  3.152  3.824  4.488  4.814  4.772  4.352  3.046  4.097  3.988  4.258  3.791  4.475  4.712  3.769  3.608  5.224  4.142  3.108  3.449  4.9  4.493  3.805  5.823  3.945  2.508  3.533  2.752  4.974  4.307  4.209  4.678  4.041  3.493  
 
 ZNF514  84874  2  95813399  95825263  2q11.1  1.498  1.911  0.401  0.42  0.752  0.672  0.533  0.857  1.176  0.837  1.294  0.462  1.073  0.617  1.031  0.817  0.61  0.554  1.46  0.374  0.901  0.51  0.474  0.22  0.653  0.382  0.511  0.654  0.546  0.261  0.727  0.64  0.376  0.414  0.77  0.96  1.061  1.767  0.62  1.138  0.868  0.557  2.307  0.408  0.183  0.414  0.298  1.184  1.218  1.929  0.884  0.575  0.79  0.748  1.164  1.357  1.665  1.314  0.082  0.706  
 
 ZNF2  7549  2  95831161  95850064  2q11.2  1.273  1.05  0.634  0.548  0.819  0.45  0.903  0.84  0.878  0.868  0.734  0.621  0.488  0.832  0.645  0.492  0.572  1.244  1.031  0.942  0.555  0.9  0.782  0.343  0.554  0.749  0.865  0.424  0.447  0.35  0.691  0.791  0.739  0.6  0.657  0.686  0.675  0.826  0.896  1.378  0.469  0.523  1.33  0.593  0.563  0.532  0.526  0.846  0.746  1.501  0.827  0.396  0.644  0.396  0.912  0.771  0.766  0.595  0.461  0.508  
 
 PROM2  150696  2  95940200  95957055  2q11.1  3.57  0.343  0.133  0.117  3.858  0.181  0.08  0.041  0.138  0.366  0.131  3.511  0.22  0.351  1.193  2.345  0.09  0.135  0.23  0.147  0  0.292  0.341  0.047  0  0.037  0.134  0.05  0.034  0.101  0.086  0  0  0.151  0.147  0.284  0.14  1.544  0.397  0.153  2.557  0.078  0.466  0.061  0.304  1.731  0.318  0.524  1.258  0.887  0.158  0.527  0.838  0.203  0.155  0.085  1.192  0.949  0.09  0.151  
 
 KCNIP3  30818  2  95963071  96051825  2q21.1  1.082  0.649  0.762  1.064  0.088  1.168  1.039  1.44  1.178  1.457  0.678  0  0  0.265  0.29  0.051  0.056  0.093  0.553  0.052  0  0.114  0.448  0  0.098  1.911  1.158  0.602  0.982  2.517  3.138  1.724  1.741  0.851  0.297  0.978  2.034  3.732  0.86  1.127  0.075  1.864  0.686  0  1.156  1.396  0.046  1.662  2.079  2.447  0.118  1.085  2.829  0.511  3.41  0.477  3.798  1.966  0.682  3.223  
 
 FAHD2A  51011  2  96068447  96078879  2q11.2  2.732  1.561  1.91  2.084  2.306  1.611  4.013  2.436  2.864  3.041  2.805  1.641  2.246  2.925  2.658  1.862  2.894  0.759  4.382  3.124  2.443  2.655  2.452  2.501  2.559  3.095  3.307  2.989  3.044  2.407  4.199  4.918  3.903  2.126  2.008  2.598  2.702  2.63  2.488  2.474  2.276  2.278  3.54  2.102  1.942  2.288  4.107  3.427  2.307  5.19  3.044  2.056  2.48  1.252  3.383  2.129  2.581  2.698  2.109  3.306  
 
 TRIM43B  653192  2  96142714  96150479  2q11.1  0  0  0.099  0  0  0  0  0  0.102  0  0  0  0  0  0  0  0  0  0  0  0  0.03  0.186  0  0  0  0.096  0  0  0.057  0  0  0  0.261  0  0  0  0  0  0  0  0  0.034  0  0  0  0.028  0  0  0  0  0  0  0  0  0  0  0  0  0  
 
 TRIM43  129868  2  96257765  96265469  2q11.1  0  0  0  0  0  0  0  0  0.095  0  0  0  0  0.025  0  0  0  0  0  0  0  0.033  0.259  0  0  0  0.05  0  0  0.034  0  0  0  0.215  0  0  0  0  0  0  0  0  0.026  0  0  0  0  0  0  0  0  0  0  0  0  0  0  0  0  0  
 
 LINC00342  150759  2  96472799  96492729  2q11.2  1.465  1.362  0.718  0.57  0.821  0.879  0.958  1.811  0.766  1.697  1.042  0.808  0.81  1.236  1.349  0.883  0.805  1.656  2.216  1.706  0.85  2.444  0.771  1.116  0.458  1.029  0.908  0.743  1.303  0.486  1.178  1.275  1.56  0.693  1.111  1.319  0.787  2.234  1.505  0.955  1.536  1.393  2.527  0.947  0.599  1.281  1.771  2.948  1.672  2.185  1.562  1.508  1.241  0.403  1.719  1.02  2.141  1.281  1.777  0.564  
 
 ANKRD36C  400986  2  96515295  96658068  2q11.1  0.86  1.93  0.271  0.224  0.693  0.542  0.488  0.904  0.36  0.928  0.708  0.279  0.41  1.147  0.769  0.525  0.458  1.698  1.423  0.744  0.73  1.704  0.432  0.5  0.28  0.671  0.747  0.411  0.764  0.419  0.769  0.687  0.934  0.321  0.538  0.944  0.463  1.11  0.671  0.615  0.829  0.642  1.133  0.566  0.276  0.702  1.63  1.413  1.029  1.076  1.211  0.734  0.494  0.208  0.873  0.384  1.312  0.638  0.987  0.408  
 
 FAHD2CP  729234  2  96676298  96688884  2q11.1  1.035  0.317  0.6  0.7  0.894  0.947  1.293  0  1.491  1.382  0.566  0.426  1.269  2.061  0.999  0.537  1.061  0.163  1.808  0.206  1.18  0.506  1.122  0.943  0  1.191  0.664  0.852  0.464  1.165  1.649  2.012  0.76  0.826  0.809  1.658  1.144  0.92  0.272  1.229  1.202  1.655  2.687  0.381  0.389  0.325  1.369  0.507  0  2.164  1.243  1.287  0.822  0.374  0.829  0.499  0.265  0.748  0.443  0.455  
 
 GPAT2  150763  2  96687693  96700727  2q11.1  0.278  2.133  0.19  0.087  0.158  0.112  0.156  0.051  0.344  0.224  0.148  0.135  0.096  2.727  0.186  0.111  0.164  0.154  0.159  0.133  0.088  0.522  0.537  0.419  0  0.071  0.65  0.124  0.168  0.217  0.191  0.207  0.117  0.263  0.151  0.102  1.373  0.202  0.291  0.068  0.1  0.714  0.632  0  0.05  0.038  1.443  0.118  0.066  0.431  0.087  0.694  0.133  0.18  0.206  0  0.118  0.058  0  0.042  
 
 ADRA2B  151  2  96778622  96781984  2q11.1  0.51  0.042  0.101  0  0.055  0.085  0.038  0.098  0.256  0.124  0.03  0.167  0.084  0.131  0.045  0.085  0.056  0.16  0.023  0.082  0  0.243  0.543  0  0  0  0.302  0.057  0.044  0.153  0  0.063  0.138  0.208  0  0.112  0  0  0.114  0.692  0.068  0.259  0.102  0.079  0.062  0.118  0.593  0.103  0.073  0.434  0  0.747  0.022  0.136  0.123  0.053  0.027  0  0  0  
 
 ASTL  431705  2  96789588  96804175  2q11.1  0.595  0  0.144  0  0  0  0.11  0.218  0  0.051  0  0.239  0  0.096  0  0  0  0  0.089  0  0  0.184  0.292  0  0  0  0.088  0  0  0.08  0  0.09  0  0  0  0  0  0  0  0.246  0  0.163  0.064  0  0  0  0.878  0  0  0.139  0.06  0.229  0  0.096  0  0  0.085  0  0  0  
 
 DUSP2  1844  2  96808907  96811179  2q11  2.421  0.072  1.34  0.96  1.014  0.122  0.821  3.95  0.526  0.321  0.392  1.227  1.506  2.364  1.616  1.7  1.877  0.639  4.417  3.232  0  3.096  2.62  2.785  0  0.15  0.911  0.577  0.273  0.362  0.448  2.63  2.172  1.577  0.115  0.334  1.04  1.529  0.962  3.149  0  2.598  1.199  1.005  0.239  1.548  0.098  1.32  0.155  3.056  0.598  1.344  0.086  0.608  0.154  0.482  0.621  0  1.714  0.331  
 
 STARD7  56910  2  96850602  96874573  2q11.2  5.675  4.743  3.315  3.629  3.574  3.163  5.243  4.141  4.657  4.064  4.947  4.052  4.768  5.358  4.495  4.476  4.526  3.246  6.03  5.77  4.105  5.101  4.632  3.934  4.153  4.764  4.605  3.964  4.728  4.809  5.006  4.927  5.024  4.015  3.584  4.434  4.337  4.613  4.66  5.257  4.038  4.092  5.698  3.575  3.407  4.068  6.013  4.357  3.748  6.309  4.4  2.809  3.779  3.254  4.408  3.694  4.581  3.077  4.255  4.194  
 
 STARD7-AS1  285033  2  96874153  96908362  2q11.2  0.771  0.23  0.66  0.218  0.482  0.24  0.95  0.704  0.711  0.737  0.69  0.423  0.875  0.683  0.435  0.76  0.611  0.158  1.241  0.622  0.534  0.773  0.924  0.564  0.479  0.703  1.191  0.747  1.038  0.536  0.957  0.848  1.398  0.875  0.346  0.564  0.818  0.603  0.959  0.995  0.612  0.624  0.913  0.361  0.378  0.635  0.199  0.738  0.35  1.351  0.725  0.682  0.372  0.505  0.633  0.336  0.767  0.26  0.953  0.398  
 
 TMEM127  55654  2  96915945  96931751  2q11.2  3.322  1.785  2.602  2.055  2.852  1.894  4.097  3.641  3.435  3.003  3.321  2.46  2.757  2.461  2.561  2.487  2.138  0.477  2.496  2.2  1.824  2.203  1.954  1.848  2.973  3.048  2.837  3.005  2.938  2.809  2.711  3.118  3.774  2.375  2.231  3.638  3.099  3.562  3.213  4.023  3.251  2.183  3.041  2.2  2.414  2.62  3.237  2.946  2.887  3.3  3.289  1.767  2.854  3.079  3.125  2.8  3.517  2.173  3.409  3.271  
 
 CIAO1  9391  2  96931883  96939900  2q11.2  3.633  3.105  2.337  2.817  3.045  2.441  3.848  2.961  3.598  2.887  3.331  2.887  2.586  3.518  3.065  3.107  3.228  2.458  4.709  3.377  2.896  3.605  3.038  2.774  3.32  4.201  2.859  3.769  3.403  2.599  3.791  4.272  4.441  3.783  2.536  3.522  2.987  3.683  3.759  4.214  2.991  2.947  4.276  3.008  2.835  2.751  3.51  3.354  2.645  5.157  3.353  1.975  2.78  2.342  3.433  2.961  3.489  2.873  2.978  3.042  
 
 SNRNP200  23020  2  96940073  96971307  2q11.2  4.909  2.143  2.787  2.664  3.867  3.104  4.463  4.633  3.327  3.878  4.661  3.466  4.386  4.431  5  4.076  4.177  1.11  5.351  3.286  4.309  4.376  2.334  3.447  4.025  4.044  4.33  5.068  4.489  4.104  4.54  4.662  5.288  2.772  3.73  4.765  4.824  4.516  3.065  6.009  4.122  3.255  4.79  4.2  3.932  4.082  2.906  4.642  4.355  5.515  4.225  3.24  4.08  3.315  4.323  3.588  4.863  3.762  4.544  4.119  
 
 ITPRIPL1  150771  2  96991061  96994091  2q11.2  0.13  0  0.225  1.765  0.051  0.921  0.1  0.234  0.235  0.125  0.067  0.528  0  1.369  0.094  0.061  0  0.107  2.212  0  1.183  1.564  1.923  2.085  0  1.298  1.316  0.055  0  1.224  0.366  0  2.373  1.44  0  0.729  1.106  0  0.046  1.385  0.083  0.333  1.667  0  0.126  0.137  1.091  1.446  0  2.236  0  0.575  0  0.114  0.957  0  0.857  1.218  0.746  0.278  
 
 NCAPH  23397  2  97001478  97042833  2q11.2  2.406  1.971  1.241  1.047  1.793  2.392  3.237  2.373  0.58  2.093  2.806  1.993  1.827  3.166  2.246  2.269  2.365  0.912  4.06  2.366  2.273  2.681  1.37  1.841  2.222  2.075  2.182  2.131  2.198  1.868  2.873  2.76  2.94  1.281  2.254  2.291  2.656  2.763  2.014  3.738  2.73  1.798  2.959  2.244  1.517  2.018  2.746  2.896  2.084  3.941  2.178  1.263  2.32  1.471  2.738  1.544  2.7  2.518  2.406  2.209  
 
 NEURL3  93082  2  97163379  97173846  2q11.2  0  0  0.164  0.439  0.147  0  0.203  0.077  0.243  0.094  0.156  0.155  0.364  0.119  0  0.965  0.098  0.15  0  0  0  0.294  0.822  0.894  0  0  0.196  0.149  0  0.14  0  0  0.12  0.255  0.177  0.106  0  0.114  2.01  0  0.49  0.158  0.097  0.141  0.13  0.64  0.241  0  0  0.3  0.233  1.113  0.393  0.623  1.147  1.041  0.757  0  1.204  0.315  
 
 ARID5A  10865  2  97202463  97218371  2q11.2  1.863  0.247  1.569  1.67  0.312  1.296  2.624  3.067  2.656  2.01  2.374  1.109  1.614  1.431  0.805  1.014  1.036  0.114  4.043  2.153  1.462  2.294  4.723  5.288  1.973  1.199  1.624  2.008  1.639  1.055  1.424  2.332  2.064  1.345  1.029  1.354  1.965  1.845  1.429  2.192  1.529  1.105  1.871  1.036  0.838  1.925  0.303  1.762  0.523  2.785  0.38  1  1.417  1.851  1.601  1.164  2.439  1.258  1.722  1.639  
 
 KANSL3  55683  2  97258891  97304116  2q11.2  2.73  2.029  1.289  1.592  2.02  1.829  3.292  2.562  2.13  2.394  2.956  1.952  2.689  2.659  2.794  2.121  2.36  1.962  3.678  2.272  2.478  3.158  1.308  1.38  2.709  2.396  2.498  2.561  2.511  2.133  2.818  2.554  2.778  1.312  1.844  2.612  2.819  3.004  2.918  3.625  2.262  1.518  3.685  2.302  1.827  2.317  3.098  3.312  2.914  4.164  2.86  1.578  2.215  2.659  3.046  2.621  3.213  1.732  2.369  2.271  
 
 FER1L5  90342  2  97308473  97370622  2q11.2  0.135  0.031  0.086  0  0.07  0.023  0.017  0.05  0.084  0.056  0.074  0.025  0.049  0.11  0.035  0.055  0.036  0.064  0.025  0.044  0.058  0.106  0.176  0  0.031  0.056  0.183  0.038  0.031  0.088  0.036  0.029  0.028  0.109  0.036  0  0.029  0.055  0.065  0.067  0.03  0.276  0.101  0.033  0.036  0.053  0.045  0.08  0.034  0.062  0.05  0.398  0.037  0.13  0.116  0.047  0.056  0  0.025  0.034  
 
 LMAN2L  81562  2  97371666  97405813  2q11.2  2.623  2.356  2.088  1.973  2.479  1.403  4.153  3.313  2.96  2.739  2.889  1.915  1.825  2.916  1.974  1.702  2.155  0.481  2.998  2.493  1.481  2.219  1.781  1.424  1.928  1.613  1.623  1.66  2.646  1.809  2.018  3.049  2.023  1.387  2.002  2.391  2.907  2.698  3.235  3.363  2.47  1.711  3.378  1.607  1.643  1.901  4.259  2.644  1.955  4.45  2.717  1.25  2.709  2.433  2.429  2.47  3.084  1.965  2.367  2.291  
 
 CNNM4  26504  2  97426638  97477628  2q11  3.625  0.534  0.848  1.149  2.625  0.676  1.839  1.76  1.758  1.546  1.941  2.305  2.272  1.399  2.124  1.824  2.233  0.507  1.223  0.572  1.106  1.253  1.071  1.336  1.106  1.54  1.252  1.609  1.49  1.361  1.008  1.367  1.762  0.907  1.354  2.378  1.763  2.148  1.283  3.245  3.134  1.352  2.775  1.714  1.412  1.503  1.651  2.061  2.098  2.525  1.7  1.603  1.162  2.086  2.011  1.759  2.194  0.783  1.767  1.493  
 
 CNNM3  26505  2  97481990  97501121  2p12-p11.2  3.932  1.692  0.65  0.87  2.053  1.182  2.163  1.434  2.083  1.447  1.688  2.081  2.22  2.496  3.182  2.146  1.972  1.584  3.042  1.932  1.371  2.53  1.708  1.616  1.345  1.946  1.888  1.889  1.867  1.548  2.048  1.861  2.643  1.317  1.596  2.525  1.883  2.078  1.539  3.026  1.749  1.93  2.716  1.664  1.327  1.418  3.042  2.384  2.224  3.157  1.62  1.855  1.278  1.051  1.992  1.487  1.825  1.141  1.731  1.351  
 
 ANKRD23  200539  2  97503650  97509758  2q11.2  1.826  0.2  0.204  0.286  0.723  0.205  0.638  0.446  0.608  0.819  1.102  0.135  0.813  0.786  1.532  0.541  0.304  0.2  1.706  0.641  0.545  1.058  1.111  2.036  0.29  1.353  0.545  0.601  0.965  0.703  0.6  1.112  0.982  0.251  0.532  1.056  0.893  1.591  0.4  1.113  0.8  0.939  1.008  0.36  0.408  0.628  0.631  0.896  0.648  1.336  0.729  0.99  0.559  0.493  0.646  0.356  1.01  0.61  0.436  0.42  
 
 ANKRD39  51239  2  97513723  97523756  2q11.2  4.304  2.121  2.001  1.677  2.145  3.274  3.744  2.284  2.449  2.353  2.944  2.18  3.001  3.367  2.648  2.583  2.378  0.657  3.806  2.582  3  3.241  3.354  4.384  2.689  3.98  3.236  2.674  3.812  3.995  4.36  4.031  3.688  2.502  2.431  3.235  2.875  4.286  2.232  3.531  2.151  3.605  4.711  2.804  2.072  2.183  2.42  3.757  2.058  5.127  2.789  2.108  2.111  2.17  2.83  2.019  2.544  3.755  2.662  2.027  
 
 SEMA4C  54910  2  97525472  97535735  2q11.2  4.628  0.403  0.805  1.095  1.016  1.55  1.87  3.445  1.757  3.068  2.064  0.035  1.843  1.368  2.271  0.763  1.001  0.154  0.253  0.309  2.337  0.146  0.756  1.225  2.284  4.961  2.827  2.666  3.129  3.066  2.023  2.588  2.781  0.891  1.329  1.977  2.449  4.98  0.844  3.849  1.679  1.532  2.869  1.566  1.748  1.521  2.233  2.452  1.116  2.938  1.614  1.738  1.86  1.749  1.789  1.601  2.784  2.093  2.271  1.735  
 
 FAM178B  51252  2  97541618  97652301  2q11.2  1.685  0  0.164  0  0.141  0  0.108  0.394  0.258  1.896  0.092  0.304  0  0.11  0.214  0  0.16  0.174  0.413  0  3.84  0.27  2.979  4.34  0  1.699  0.306  2.147  2.579  3.983  0.162  0  0  0.219  0  0  0  2.65  0.127  0.303  0.325  0.487  0.222  0  0.226  0.196  0.197  0.139  0  0  0.099  0.834  0.107  0.187  0.173  0  0.106  0  0  0  
 
 LOC101927053  101927053  2  97584842  97591574  2q11.2  0.142  0.041  0.15  0  0.041  0  0  0  0.142  0.07  0  0  0  0.1  0  0.048  0  0.155  0.039  0.07  0  0.233  0.537  0  0  0.113  0.086  0.205  0.315  0.302  0  0  0  0.334  0  0  0  0  0.043  0  0  0.518  0.174  0  0  0.046  0.034  0  0  0  0  0.355  0.054  0  0.074  0  0  0  0  0  
 
 FAHD2B  151313  2  97749322  97760582  2q11.2  1.612  1.128  1.505  1.679  2.77  1.65  2.858  0.514  2.715  2.93  2.389  1.267  1.857  2.833  2.324  1.217  2.448  0.619  2.877  3.042  2.678  2.492  2.652  2.473  0.845  3.018  1.46  0.977  2.472  1.945  2.969  4.059  1.889  1.505  1.948  1.683  2.042  2.515  2.438  1.795  2.457  2.386  3.324  0.617  1.308  1.123  4.624  2.801  0.484  4.551  2.343  2.316  2.229  0.893  2.816  1.5  1.444  2.555  1.604  1.986  
 
 ANKRD36  375248  2  97779232  97930257  2q11.2  1.332  2.097  0.338  0.24  0.644  0.47  0.507  0.988  0.377  1.21  0.937  0.36  0.627  1.353  0.856  0.66  0.625  2.018  1.678  0.851  0.59  1.907  0.325  0.379  0.524  1.241  0.582  0.521  1.063  0.567  0.689  0.926  0.858  0.337  0.68  1.245  0.471  1.709  0.919  1.039  1.072  0.781  1.528  1.089  0.313  0.671  0.764  1.521  1.24  1.709  0.809  1.248  0.526  0.377  1.06  0.658  1.745  0.758  1.072  0.32  
 
 LOC100506076  100506076  2  98081675  98094824  2q11.2  0  0.886  0.224  0  0.236  0  0  0.259  0.37  0.501  0  0  0.256  0.293  0  0.202  0.686  0.554  1.706  2.01  0  2.056  0.773  0.265  0  0.284  0.135  0  0  0  0  0  0  0  0  0.331  0  0.442  2.356  0  0  0.719  2.283  0  0  0.282  1.939  0  0  1.999  0  0.877  0  0.49  1.384  0  0.235  0  0  0  
 
 LOC100506123  100506123  2  98081774  98089620  2q11  0.814  0.733  0  0  0.469  0  0.494  0.88  0  1.436  0.942  0.227  0.415  0.993  0.487  0.273  0.49  0  3.01  0.612  0  1.61  0.579  0.326  0  1.302  0.279  0  0.841  0.253  0.249  0.846  0.337  0  0.963  0.867  0  1.769  1.258  0.845  0.693  1.138  3.069  0.6  0.263  0.805  1.756  1.681  0.658  3.814  0.252  1.741  0.159  0.698  1.343  0.735  1.1  0  0.493  0  
 
 ANKRD36B  57730  2  98121260  98206428  2q11.2  1.106  1.967  0.398  0.211  0.653  0.383  0.572  0.86  0.398  1.034  0.794  0.33  0.658  1.277  0.887  0.649  0.637  1.67  1.736  0.822  0.556  1.862  0.522  0.461  0.4  0.799  0.73  0.508  1.162  0.44  0.703  0.884  1.147  0.458  0.73  0.998  0.5  1.363  0.791  0.875  0.891  0.592  1.571  0.73  0.197  0.763  1.242  1.562  1.169  1.564  0.825  0.917  0.588  0.289  1.016  0.578  1.691  0.773  0.935  0.39  
 
 COX5B  1329  2  98262520  98264657  2q11.2  8.008  8.053  5.433  5.525  7.156  6.734  7.788  6.958  6.619  6.526  6.476  7.717  7.003  8.062  5.887  7.144  8.251  7.279  8.43  9.621  7.265  7.88  5.919  7.411  7.708  6.362  7.028  5.899  7.559  6.629  7.848  8.131  8.289  6.955  7.303  7.053  5.641  7.015  8.235  7.491  6.763  6.688  7.564  6.786  6.489  7.42  7.632  7.053  6.079  9.08  6.437  5.045  6.208  6.621  7.577  6.487  6.381  7.738  7.263  6.287  
 
 ACTR1B  10120  2  98272401  98280561  2q11.1-q11.2  2.749  1.702  2.937  2.013  3.398  2.432  3.822  2.757  3.922  3.505  2.895  3.02  3.228  1.872  3.1  3.023  3.23  0.616  5.004  1.983  2.333  3.858  3.605  3.002  3.026  2.971  4.111  2.767  3.7  1.689  3.26  3.312  4.197  3.278  2.605  3.735  3.833  3.553  3.385  3.305  3.517  2.886  4.048  2.372  2.003  3.053  1.514  3.655  3.151  5.079  2.594  2.286  2.713  2.178  3.617  2.128  3.153  3.328  2.665  3.147  
 
 LINC01125  728537  2  98286205  98319529  2q11.2  0.368  0.278  0  0  0.197  0  0  0.131  0.268  0.256  0.157  0  0  0.101  0  0.092  0.154  0.156  0.434  0.108  0  0.418  0.307  0.117  0  0  0.438  0  0.225  0.061  0  0  0.518  0.249  0  0  0  0  0.853  0.243  0.097  0.376  0.453  0  0  0.127  0.393  0.215  0  0.341  0  0.3  0  0.151  0.323  0  0.246  0  0  0  
 
 ZAP70  7535  2  98330030  98356323  2q12  0.099  0.078  0.233  0  0.127  0.207  0.52  0.099  0.213  0.495  0.045  0.115  0  0.252  0  0.105  0.135  0.124  7.342  0.154  0  6.255  0.517  0.269  0  0.058  0.137  0.038  0.078  0.118  0  0.068  0.123  0.231  0  0  0.143  0  0.295  0.143  0  0.631  0.091  0  0  0.159  0.035  0.085  0.139  0.569  0.044  1.059  0.116  0.257  0.192  0.095  0.14  0  0  0  
 
 TMEM131  23505  2  98372800  98612354  2q11.2  2.603  3.299  1.556  1.705  2.247  1.44  2.572  2.904  2.189  2.394  2.259  1.587  2.239  3.609  1.681  1.602  2.044  1.71  2.841  2.357  1.74  2.55  1.203  1.45  2.382  2.785  2.384  2.531  3.171  1.939  2.291  3.394  2.905  1.781  1.557  3.071  2.367  2.769  2.856  3.357  2.94  1.511  2.401  2.017  1.147  1.617  1.705  2.335  2.078  3.117  2.973  1.706  1.986  2.223  2.598  1.775  3.048  1.523  2.17  2.35  
 
 VWA3B  200403  2  98703594  98929410  2q11.2  0  0  0.065  0  0.031  0.026  0.034  0.022  0.087  0.035  0.029  0.03  0.037  0.037  0  0  0  0.106  0  0.033  0  0.084  0.163  0  0  0  0.084  0  0  0.08  0  0  0  0.119  0  0  0  0  0.04  0  0.027  0.221  0.049  0  0  0.027  0.06  0  0  0  0.023  0.124  0.016  0.056  0.031  0.035  0  0  0  0  
 
 CNGA3  1261  2  98962617  99015064  2q11.2  0.069  0.051  0.145  0  0  0.09  0.029  0.029  0.241  0.051  0.037  0  0  0.036  0  0.032  0  0.159  0  0.071  0  0.15  0.493  0.043  0.048  0  0.083  0.031  0  0.071  0  0  0  0.278  0.063  0.031  0  0  0.063  0.061  0  0.104  0.084  0  0.067  0  0.037  0.048  0  0.021  0  0.455  0.065  0.063  0.063  0.05  0  0  0  0  
 
 INPP4A  3631  2  99061320  99207496  2q11.2  1.393  1.185  0.869  0.867  0.797  0.762  1.377  1.782  1.396  1.326  1.442  0.544  1.943  1.326  1.841  1.114  0.603  0.373  3.32  1.547  0.852  2.244  2.065  1.736  1.055  1.47  1.708  1.833  1.835  1.275  1.452  1.6  1.961  0.735  1.028  1.398  1.74  2.101  0.924  2.521  2.283  0.887  1.738  1.415  0.899  1.296  1.327  1.949  1.552  2.348  1.515  0.831  1.115  1.02  1.152  1.07  1.909  1.023  0.921  1.211  
 
 COA5  493753  2  99215785  99224981  2q11.2  2.698  3.446  1.019  1.477  2.453  1.029  2.526  1.657  3.05  1.943  1.865  1.864  1.572  2.609  1.558  2.049  2.255  4.248  3.979  2.521  1.98  1.889  2.261  1.537  1.847  1.668  1.549  1.233  1.575  1.377  2.092  2.223  2.078  1.973  1.862  2.21  1.298  2.321  2.465  2.153  1.765  1.771  3.045  1.683  1.289  1.435  1.188  1.763  1.151  4.136  1.642  0.907  1.121  1.408  2.394  1.835  1.691  1.7  1.758  1.34  
 
 UNC50  25972  2  99225041  99234977  2q11.2  3.299  4.984  1.868  2.085  3.599  1.75  3.999  3.202  3.55  3.192  3.433  2.465  2.614  4.359  2.441  3.113  3.02  4.989  4.914  3.383  2.796  2.669  3.064  2.294  2.749  3.319  2.057  2.107  2.898  2.016  3.499  3.822  3.115  2.402  2.473  3.492  2.609  3.356  4.014  3.387  3.131  2.42  3.439  2.49  1.633  2.349  3.054  2.586  2.348  5.144  2.489  1.333  2.159  2.696  3.569  3.167  3.278  3.016  3.094  2.954  
 
 MGAT4A  11320  2  99235568  99347589  2q12  1.264  0.857  0.076  0.31  0.933  0.077  0.052  0.148  0.5  0.254  0.476  1.41  0.668  0.704  1.219  0.185  0.934  0.245  2.674  1.267  0.068  1.662  0.432  0.286  0.025  0.609  0.073  0.166  0.163  0.309  0.511  0.441  0.217  0.309  0.106  0.731  0.67  0.179  0.287  0.446  0.517  0.183  0.5  0.644  0.176  0.796  0.105  0.202  0.832  1.169  0.036  0.396  0.015  0.098  0.092  0.29  0.192  0.05  0.209  0.109  
 
 LOC101927070  101927070  2  99377525  99388361  2q11.2  0  0  0.128  0  0  0  0.058  0.065  0.11  0.111  0  0  0  0  0.066  0  0  0  1.624  0.265  0  1.554  0.229  0.111  0  0.062  0.047  0.034  0  0.214  0.238  0.053  0  0.258  0  0  0  0  0  0  0  0  0.039  0  0  0.044  0  0  0  0  0  0.092  0  0.034  0  0  0.064  0  0  0  
 
 KIAA1211L  343990  2  99410308  99552684  2q11.2  0.237  0.214  0.098  0.381  1.006  0.109  0.042  0.89  0.111  0.079  0.219  0.774  1.052  0.106  0.387  0.073  0.582  0.153  0.088  0.123  0  0.251  0.389  0.31  0  0.035  0.166  0.077  0.032  0.169  0.073  0.064  0  0.177  0.078  0.211  0.027  0.053  0.5  0.263  0.893  0.422  0.168  1.273  0.29  0.895  0.112  0.062  0.678  0.122  0.043  0.768  0.042  0.148  0.294  0.101  0.043  0.042  0  0.103  
 
 TSGA10  80705  2  99613723  99771187  2q11.2  0.549  0.612  0  0  0.149  0.076  0.048  0.105  0.091  0.072  0.149  0.352  0.348  0.433  0.214  0.139  0.35  0.794  0.341  0.118  0  0.244  0.149  0.048  0.087  0.198  0.215  0.089  0.078  0.084  0.131  0.089  0.077  0.216  0.127  0.044  0.031  0.264  0.194  0.258  0.246  0.224  0.025  0.175  0.115  0.175  0.792  0.098  0.211  0.363  0.143  0.299  0.265  0.063  0.38  0.093  0.127  0.083  0.039  0.229  
 
 C2orf15  150590  2  99758184  99767928  2q11.2  1.578  0.114  0.168  0  1.132  0.13  0  0.057  0.308  0.059  0.525  0.599  1.386  0.354  1.003  1.366  0.863  0.74  0.673  0  0.179  0.455  1.13  0.24  0  0.192  0.123  0.131  0.133  0  0.113  0.152  0.211  0.196  0.176  0.397  0.067  0.108  0.207  0.394  1.078  0.406  0.225  0.292  0.322  1.039  0  0.312  0.37  1.06  0.271  0.279  0.133  0.105  0.798  0.154  0.172  0  0.303  0.521  
 
 LIPT1  51601  2  99771417  99779613  2q11.2  1.505  2.854  0.626  0.687  1.402  1.019  1.344  0.821  1.034  1.023  1.028  0.677  1.372  1.948  0.816  1.029  1.12  2.981  2.373  1.477  1.123  1.362  1.049  0.519  1.249  0.955  0.892  0.837  0.966  0.477  1.581  1.616  1.033  1.103  0.643  0.921  0.734  0.649  1.999  1.276  1.284  0.904  1.884  1.11  0.681  1.022  0.204  0.865  0.789  2.484  0.976  0.692  0.785  0.411  1.77  1.006  0.968  0.578  1.325  0.976  
 
 MITD1  129531  2  99785725  99797492  2q11.2  2.669  5.582  1.289  1.712  2.397  2.389  3  2.535  1.45  2.439  2.828  2.268  2.682  3.187  1.785  3.51  3.285  6.309  4.733  3.539  2.306  3.244  1.767  1.639  2.727  2.007  1.366  0.761  1.558  1.262  2.754  1.914  2.053  1.566  2.521  2.512  1.836  1.964  3.673  2.471  2.39  2.864  3.607  2.551  1.225  2.466  2.569  1.919  1.994  4.412  2.441  1.613  2.079  2.491  3.958  2.684  3.047  2.986  3.411  2.788  
 
 MRPL30  51263  2  99797541  99816020  2q11.2  2.301  3.306  1.242  1.298  2.27  1.284  2.245  1.914  2.168  1.774  2.293  1.794  2.267  2.652  1.865  2.196  2.637  4.572  3.17  3.54  2.569  2.806  1.658  1.206  2.047  2.405  1.666  2.094  2.352  1.689  2.914  2.675  2.345  2.023  1.92  2.096  1.76  1.852  2.983  2.185  1.713  1.99  2.35  2.129  1.814  1.934  2.993  2.05  1.721  4.205  2.463  1.055  1.816  1.678  2.728  2.097  2.408  1.528  2.11  2.239  
 
 LYG2  254773  2  99858710  99871570  2q11.2  0  0.132  0  0  0  0.194  0  0  0  0.125  0  0  0  0.299  0  0  0  0  0.196  1.78  0  0.271  0  0  0  0  0  0  0  0  0  0  0  0  0  0  0  0  0.1  0  0  0.181  0  0  0.367  0  0  0  0  0.248  0  0.312  0  0  0.111  0.199  0  0  0  0  
 
 LYG1  129530  2  99900700  99917639  2q11.2  0.295  0.775  0  0.627  0.185  0.344  0.649  0.313  0.351  0.7  0.712  0.217  0.458  1.82  0.837  0.36  0.847  1.119  1.809  0.658  0.269  1.395  0.544  0.267  0.185  0.112  0.152  0.089  0.141  0  0.307  0.263  0.234  0.184  0.253  0.76  0.26  0.313  0.704  0.586  0.216  0.428  0.962  0.532  0  0.21  1.07  0.244  0.488  0.923  0.423  0.292  0.318  0.321  0.418  0.245  0.586  0  0.305  0  
 
 TXNDC9  10190  2  99935486  99952860  2q11.2  2.864  5.017  2.005  2.002  3.348  2.479  3.134  3.235  2.247  2.567  3.218  2.37  2.78  2.593  1.433  3.221  1.728  4.098  4.25  3.463  2.258  2.259  2.266  1.703  3.661  2.873  2.042  1.926  2.856  2.089  3.281  3.304  2.808  2.376  2.297  3.034  2.154  2.166  4.574  3.205  3.117  2.889  3.506  2.127  2.244  2.384  2.472  2.663  2.162  5.232  2.913  1.675  2.459  2.464  3.652  3.046  3.594  3.025  3.419  3.307  
 
 EIF5B  9669  2  99953833  100016728  2q11.2  3.032  5.308  2.489  2.624  3.168  3.092  3.233  3.186  2.993  2.954  3.4  2.446  2.829  3.81  3.323  3.678  2.688  5.332  4.507  4.448  3.913  4.008  3.29  3.003  3.902  3.354  2.389  2.692  3.02  1.979  4.063  4.009  3.534  3.287  3.278  3.81  2.857  2.895  4.183  4  3.187  3.313  4.121  3.676  2.868  3.527  3.439  3.438  3.318  5.074  3.693  1.896  2.957  2.429  3.946  3.243  3.887  3.031  3.636  3.727  
 
 REV1  51455  2  100016937  100106480  2q11.1-q11.2  2.077  2.371  0.799  0.872  1.697  0.782  1.604  1.729  1.564  1.706  1.746  0.854  1.565  2.18  1.695  1.875  1.453  2.621  3.043  1.701  1.328  1.909  1.052  0.793  1.312  1.524  1.343  1.238  1.364  0.651  1.952  1.711  1.733  0.97  1.027  1.591  1.52  1.284  1.891  2.107  1.462  1.242  1.677  1.393  1.15  1.41  2.266  1.785  1.901  3.059  1.638  0.718  1.278  0.697  1.938  1.311  2  1.17  1.819  1.608  
 
 AFF3  3899  2  100163715  100759037  2q11.2-q12  0.952  0.026  1.293  1.214  2.357  0.936  1.403  1.984  0.552  1.476  0.642  0.053  0  0.035  0  0.076  0.014  0.056  1.897  0.212  0  1.271  0.353  0.034  0.094  1.725  1.119  0.648  1.037  0.633  1.512  0.584  0.577  0.308  0.018  0.1  0.144  0.038  0.813  0.612  0.017  0.25  0.099  0.045  0.047  0.05  0.072  0.754  0.52  0.724  0.63  0.372  0.77  0.442  1.746  1.174  2.32  0  1.833  1.12  
 
 LOC150577  150577  2  100824715  100867946  2q11.2  0  0  0.122  0  0  0  0  0  0.178  0.02  0  0  0  0  0  0  0  0.064  0  0.056  0  0.042  0.488  0  0  0  0.051  0  0  0.063  0  0  0  0.314  0.04  0  0  0  0.043  0  0  0.134  0.047  0  0  0  0  0  0  0  0  0.212  0  0.021  0  0  0  0  0  0  
 
 LONRF2  164832  2  100889752  100939195  2q11.2  1.438  0.037  0.111  0.028  0.654  0.075  0.289  0.057  0.603  0.104  0.854  0.044  0.012  0.533  0.024  0.092  0.055  0.074  0.02  0.066  0.658  0.127  0.245  0.01  0.022  0.425  1.059  0.349  0.068  1.407  1.486  2.139  0.153  0.317  0.545  1.586  0.026  0.063  0.246  2.635  0.568  0.489  0.215  0.024  0.374  0.502  0.25  0.073  0.924  0.069  1.349  0.72  0.061  0.356  0.135  0.233  0.036  0.017  0  0.209  
 
 CHST10  9486  2  101008321  101034130  2q11.2  0.901  0.398  0.992  1.48  1.246  0.858  1.788  1.537  1.91  1.606  1.659  0.04  0  0.042  0  0.076  0  0.07  2.581  1.475  1.788  2.05  0.237  0.059  2.244  2.304  2.564  1.871  2.391  2.552  2.934  2.857  2.914  1.508  1.484  2.263  1.449  1.683  1.069  2.717  0.767  1.355  2.568  1.122  1.152  0.962  0.41  2.024  1.139  3.37  1.619  1.285  1.164  1.022  1.786  1.698  1.642  1.093  1.023  1.409  
 
 NMS  129521  2  101086943  101099742  2q11.2  0  0  0  0  0  0  0  0  0  0  0  0  0  0  0  0  0  0  0  0.536  0  0  0  0  0  0  0.102  0  0  0.115  0  0  0  0  0  0  0  0  0  0  0  0  0  0  0  0  0  0  0  0  0  0  0  0  0  0  0  0  0  0  
 
 PDCL3  79031  2  101179417  101193201  2q11.2  3.467  4.882  2.153  1.804  3.161  2.862  3.531  2.99  2.89  2.429  3.521  2.128  2.388  3.82  1.739  2.709  2.656  3.979  4.894  2.708  2.656  3.327  1.55  1.691  2.693  2.844  1.996  2.246  2.649  1.971  4.111  3.631  3.1  2.125  2.425  3.405  2.408  2.39  3.019  3.416  2.623  2.83  3.602  2.935  2.256  2.902  2.321  3.128  2.707  5.379  2.827  2.136  2.345  2.17  2.916  2.389  3.473  2.512  2.662  3.058  
 
 NPAS2  4862  2  101436612  101613287  2q11.2  0.754  2.557  1.969  0.089  0.168  0.225  1.371  2.442  1.787  1.603  1.108  2.727  1.39  1.859  1.433  0.728  2.81  1.338  0.085  0.37  0.056  0.457  0.433  0.096  1.817  3.181  3.922  2.978  4.989  3.648  4.367  3.721  4.23  2.634  2.626  3.402  1.253  3.581  2.038  2.937  2.127  2.729  0.362  0.524  0.926  1.796  2.657  0.609  1.347  3.387  3.748  0.866  0.1  1.275  0.838  1.166  2.86  0.038  1.804  1.281  
 
 LOC101927142  101927142  2  101589109  101593623  2q11.2  0  0.152  0  0  0  0  0.349  0.098  0  0  0  0.424  0  0  0  0  0  0  0  0  0  0  0  0  0  0.219  0.77  0.269  0.526  1.169  1.021  0.261  0.351  0.409  0.271  0.227  0  0.376  0.23  0  0  0.518  0  0  0  0.409  1.578  0  0  0.323  0.257  0.313  0  0.251  0  0  0.32  0  0  0  
 
 RPL31  6160  2  101618690  101636155  2q11.2  9.401  11.938  6.698  6.026  9.01  8.926  9.366  8.147  7.064  8.253  8.243  8.564  8.35  10.097  7.706  9.059  9.526  13.476  10.611  12.412  8.613  10.002  7.75  8.926  9.229  7.826  8.078  7.856  8.455  7.84  9.267  9.184  8.585  6.7  9.165  8.071  7.956  8.483  10.605  8.065  8.204  8.141  9.972  8.948  8.203  8.303  10.323  8.888  7.875  11.64  7.115  6.612  8.11  8.092  9.974  8.233  8.423  9.323  8.887  8.283  
 
 TBC1D8  11138  2  101623689  101767846  2q11.2  1.293  1.111  0.679  1.655  0.798  0.529  1.608  0.539  1.445  1.721  1.167  1.45  0.957  2.686  1.704  1.774  2.242  2.593  0.252  1.5  0.13  0.636  0.651  0.333  0.542  1.206  2.129  1.277  1.059  0.916  1.113  1.868  2.751  1.646  1.965  2.377  1.744  1.413  0.689  2.265  2.614  1.374  0.848  1.888  1.473  1.183  2.646  0.41  1.863  0.993  2.6  1.431  0.709  0.69  1.017  1.103  1.584  0.375  1.323  0.681  
 
 CNOT11  55571  2  101869344  101886778  2q11.2  3.26  2.67  1.893  2.178  3.067  2.005  3.245  2.477  2.886  2.296  3.092  3.173  3.257  3.687  2.527  3.374  3.11  2.665  4.596  3.009  2.872  3.25  2.529  2.105  3.131  3.046  2.331  2.392  2.635  2.321  3.282  3.135  2.425  1.918  2.346  3.086  2.729  2.638  2.842  2.906  3.164  2.465  3.858  2.515  2.081  2.741  3.003  2.79  2.855  4.688  2.876  1.971  2.624  2.545  3.471  2.176  3.227  2.304  3.17  3.121  
 
 SNORD89  692205  2  101889397  101889511  2q11.2  0  0  0  0  0  0  0  0  0  0  0  0  0  0  0  3.458  0  0  0  0  0  0  0  0  0  0  0  0  0  0  0  0  0  0  0  0  0  0  0  0  0  0  0  0  0  0  0  0  0  0  0  0  0  0  0  0  0  0  0  0  
 
 RNF149  284996  2  101892062  101925178  2q11.2  2.108  2.945  1.2  1.546  1.676  0.047  1.129  1.341  1.883  1.117  1.853  2.79  2.323  2.892  1.516  2.641  2.278  2.294  2.753  1.148  1.186  1.469  1.764  1.247  2.213  1.814  1.547  1.417  1.719  1.353  1.862  2.437  1.421  1.279  1.292  1.956  1.676  2.032  2.723  1.811  2.518  1.796  1.936  1.808  0.839  1.409  1.478  1.825  1.786  3.902  2.067  1.308  1.875  1.746  2.481  2.027  2.846  1.718  2.585  2.055  
 
 CREG2  200407  2  101964815  102003965  2q11.2  0  0.03  0.118  0  0.016  0.065  0.054  0.036  0.147  0  0.803  0.733  0.733  0.044  0.051  0.038  0.591  0.248  0  0.047  0  0.121  0.247  0  0  0.12  0.069  0  0  0.065  0.057  0  0  0.235  0.099  0.457  0  0  0.074  0  1.459  0.325  0.043  0  0  0.105  0.296  0  0.222  0.033  1.712  0.479  0.065  0.209  0.044  0.06  0.043  0  0.078  0  
 
 RFX8  731220  2  102013822  102091165  2q11.2  0  0  0.292  0.914  0  0.144  0.334  0.402  0  0.059  0.072  0.604  0  0.024  0  0  0  0.889  2.15  0.598  0  0.637  0  0  0.998  0.096  0.08  0  0  0.058  0  0  0  0.215  0  0  0.867  1.113  0  0  0.182  0.162  0.029  0  0  0  0  0  0  0.093  0.178  0  0  0  0  0.077  0.238  0  0  0  
 
 MAP4K4  9448  2  102314164  102511152  2q11.2-q12  2.051  2.743  2.667  2.347  1.163  2.303  3.772  2.838  3.454  4.034  3.616  2.319  1.723  3.161  2.349  2.014  1.848  1.872  3.949  1.391  2.389  3.398  0.227  0.232  3.941  2.505  2.452  2.771  2.852  1.82  2.907  3.443  3.058  1.409  1.821  3.066  3.698  3.284  3.264  2.827  3.137  1.633  4.108  3.092  2.078  2.984  3.009  3.267  3.239  4.398  4.274  2.111  3.14  2.494  3.133  2.63  4.025  2.426  3.652  4.24  
 
 LOC100506328  100506328  2  102599926  102602007  2q12.1  0  0  0  0  0  0  0  0  0.173  0  0  0  0  0  0  0  0  0  0  0  0  0  0.296  0  0  0  0.044  0  0  0.044  0  0  0  0.182  0  0  0  0  0  0  0.139  0.075  0.049  0.23  0.579  1.825  0  0  0.52  0  0  0  0  0  0  0  0  0  0  0  
 
 IL1R2  7850  2  102608305  102644884  2q12  0  0  0  0  0  0  0  0  0.166  0.401  0.066  4.856  0  0.079  0  0.383  3.461  0.14  0.066  0  0  0.063  0.246  0  0  0.229  0.079  0.762  0  0.095  0  0.592  0  0.236  0.171  0.542  0  0.185  0.043  0  0.457  0.093  0  0.506  1.122  4.742  1.733  0  2.169  1.302  0.118  0.372  1.62  0.803  0  0  0.414  0  0.295  0  
 
 IL1R1  3554  2  102686835  102796334  2q12  0.154  0.609  1.387  1.584  0.972  0.587  1.585  1.195  0.137  2.834  0.414  0.143  0  0.786  0.151  0.07  0.103  0.062  0.021  0.087  0.204  0.067  0.206  0  0.377  0.461  0.189  0.666  0.094  0.053  0  0.918  0  0.22  1.154  1.679  0.798  0.836  0.869  1.095  1.058  0.744  0.929  0.776  2.035  3.408  0.042  0.272  1.882  0.179  0.061  0.67  1.716  1.605  0.661  0.939  2.284  0.915  1.482  0.836  
 
 IL1RL2  8808  2  102803432  102855811  2q12  0  0.044  0.093  0  0.037  0  0  0  0.086  0.033  0  0.119  0.148  0  0  0.151  0  0.062  0  0.057  0  0.082  0  0  0  0  0.074  0  0  0.059  0  0  0  0.127  0  0  0  0.19  0.031  0  0  0.321  0.042  0  0.102  0.63  0.026  0.864  0  2.044  0  0.222  0  0.073  0  0  0.521  0  0.446  0.085  
 
 IL1RL1  9173  2  102927961  102968497  2q12  0  0  0.294  0  0  0  0.05  0  0.082  0.023  0  0  0  0  0  0  0  0.1  0  0.074  0  0.075  0.249  0  0  0  0.07  0.028  0  0.048  0  0  0  0.341  0.061  0  0  0  0.075  0  0.553  0  0.058  0  0  0  0.034  0  0  0.065  0.206  0.516  0  0.047  0.036  0  0  0  0  0  
 
 IL18R1  8809  2  102972742  103015235  2q12  0  0  0.116  0.115  0  0  0  0.057  0.05  0.509  0.106  0.356  0  0.208  0  0.028  0.059  0  0.075  0.066  0.079  0.032  0.115  0  0.229  0  0.05  0.022  0  0.043  0  0.08  0  0.236  0.138  0.088  0  0.05  0.435  0  0.28  0.683  0.03  0  0.191  0.214  0.046  0  0.066  0.509  0.059  0.321  0.252  0.284  0.047  0.048  0.246  0  0.078  0  
 
 IL18RAP  8807  2  103035249  103069024  2q12  0  0  0  0  0  0  0  0  0.061  0.045  0  0  0  0.021  0  0  0  0  0  0.131  0.282  0.042  0.141  0  0  0  0.038  0  0  0.03  0  0  0  0.18  0.056  0  0  0  0.049  0  0  0.138  0.019  0  0  0.062  0.025  0  0  0.034  0  0.228  0  0.083  0.03  0  0  0  0  0  
 
 SLC9A4  389015  2  103089761  103150431  2q12.1  0  0  0.088  0  0.022  0  0  0  0.094  0.062  0.074  0  0  0.014  0  0.054  0.026  0.023  0  0.043  0  0.099  0.271  0  0  0  0.051  0  0  0.049  0  0  0  0.16  0  0  0  0  0.032  0  0.055  0.204  0.034  0  0.104  0.277  0.03  0  0.037  0  0  0.374  0  0.037  0.02  0  0  0  0  0  
 
 SLC9A2  6549  2  103236147  103327809  2q11.2  0.766  0.035  0.081  0  1.691  0  0  0.032  0.104  0.019  0.03  0.393  0  0.252  0.524  0.132  1.114  0.077  0.02  0.024  0.244  0.093  0.193  0  0  0.017  0.043  0.012  0  0.048  0  0.026  0  0.187  0.408  0.144  0  0  0.079  0.272  0.596  0.596  0.127  0.186  0.063  1.322  0.075  0.191  0.559  0.451  0.845  0.516  0  0.049  0  0.037  0.023  0.044  0  0  
 
 MFSD9  84804  2  103333665  103353337  2q12.1  1.549  0.284  0.443  0.428  0.779  0.623  1.307  1.09  0.857  0.713  0.911  1.428  1.808  1.149  1.262  1.525  1.73  0  1.583  0.562  0.752  0.89  0.723  0.298  0.948  1.222  1.226  1.087  1.204  1.054  1.124  1.095  1.179  0.691  0.753  1.041  1.185  0.95  0.508  1.408  1.123  0.768  1.722  0.82  0.87  0.976  1.39  1.579  1.032  2.281  0.984  0.749  1.104  0.826  1.241  0.637  1.15  0.785  1.336  1.225  
 
 TMEM182  130827  2  103378489  103434138  2q12.1  0.527  0.39  0.174  0.119  0.424  0.125  0.546  0.272  0.435  0.265  0.398  0.379  0.613  0.671  0.308  0.357  0.709  0.657  1.075  0.337  0.266  0.382  0.55  0.076  0.622  0.735  0.308  0.334  0.603  0.433  0.518  0.561  0.422  0.468  0.422  0.307  0.354  0.303  0.229  0.539  0.735  0.312  0.133  0.367  0.175  0.491  0.512  0.359  0.084  1.25  0.544  0.282  0.221  0.168  0.722  0.116  0.433  0.157  0.627  0.565  
 
 LOC100287010  100287010  2  104995307  105024790  2q12.1  0  0  0  0  0  0  0  0  0  0  0  0  0  0  0  0  0  0  0  0.1  0  0  0  0  0  0  0.059  0  0  0  0  0  0  0  0  0  0  0  0  0  0  0  0  0  0  0  0  0  0  0  0  0  0  0  0  0  0  0  0  0  
 
 LINC01102  150568  2  105050804  105129215  2q12.1  0  0  0  0  0  0  0  0  0.126  0  0  0  0  0  0  0  0  0  0  0  0  0  0.279  0  0  0  0.083  0.046  0.112  0.332  0.23  0.635  0  0.287  0  0  0  0  0  0  0  0.283  0  0  0  0  0.027  0  0  0  0  0.22  0  0.058  0  0  0  0  0  0  
 
 LINC01103  101927360  2  105104913  105126967  2q12.1  0  0  0  0  0  0  0  0  0.083  0  0  0  0  0  0  0  0  0.175  0  0  0  0  0  0  0  0  0.053  0  0  0.026  0  0  0  0.272  0  0  0  0  0  0  0  0  0.033  0  0  0  0  0  0  0  0  0  0  0.047  0  0  0  0  0  0  
 
 LINC01114  284998  2  105363094  105374177  2q12.1  0  0  0.119  0  0  0  0  0  0.127  0.031  0.057  0  0  0.021  0  0  0  0.092  0.029  0.056  0  0.081  0.234  0  0  0  0.071  0.045  0  0.081  0  0  0  0.277  0  0  0  0  0.026  0  0  0.04  0.036  0  0  0.048  0.058  0  0  0.564  0.031  0.264  0  0.07  0  0  0  0  0  0  
 
 LINC01158  100506421  2  105421882  105469641  2q12  0  0  0  0  0  0  0  0  2.393  0  0.353  0  0  0  0  0  0  0  0  0  0  0  0  0  0.298  0  0  0  0  0.974  0  1.963  0.645  0.347  0  0  0  0  0.156  0  0  0.658  0  0.807  0  0  0  0  0  0.523  0  0  0.795  1.163  1.082  1.27  0.805  0  0  0  
 
 POU3F3  5455  2  105470525  105475031  2q12.1  0.42  0.078  0.261  0.219  0.154  0.237  0.17  0.155  2.315  0.244  0.273  0.16  0.068  0.128  0.302  0.202  0.258  0.271  0.076  0.175  0.417  0.568  0.372  0.144  0.311  0.105  0.271  0.113  0.137  0.516  0.16  1.363  0.361  0.417  0.327  0.122  0.072  0  0.219  0.101  0.101  1.036  0.118  2.054  0.236  0.214  0.116  0.495  0.192  0.593  0.111  1.326  1.271  1.772  1.06  1.292  1.418  0.17  0.125  0.108  
 
 LINC01159  102682016  2  105481954  105489053  2q12.1  0  0  0.257  0  0.057  0  0  0.036  0.257  0.063  0  0  0  0.035  0  0  0  0.092  0  0  0  0.174  0.46  0  0  0  0.091  0  0  0.312  0  0  0.145  0.467  0  0  0  0  0.067  0  0  0.291  0.061  1.538  0  0.07  0  0  0  0.127  0  0.83  0.089  0.42  0.167  0.5  0.173  0  0  0  
 
 LOC102724691  102724691  2  105552698  105654954  2q12.1  0  0  0  0  0  0  0  0  0  0  0  0  0  0.304  0  0  0  0  0  0  0  0  0  0  0  0  0.109  0  0  0.086  0  0  0  0  0  0  0  0  0  0  0  0.335  0  0  0  0  0  0  0  0  0  0  0  0.338  0  0  0  0  0  0  
 
 MRPS9  64965  2  105654482  105716418  2q12.1  4.196  4.392  1.704  2.171  3.421  2.362  3.819  2.779  2.049  2.377  3.159  3.181  3.168  4.724  3.114  3.055  3.985  5.594  5.099  4.429  3.529  3.847  2.751  2.762  3.368  2.591  3.099  2.526  2.873  2.591  3.615  3.775  2.998  1.5  2.832  3.055  2.904  2.365  3.137  4.702  3.168  2.6  4.126  3.176  2.558  2.688  4.554  3.981  2.646  6.13  2.719  1.935  3.127  1.83  4.091  2.33  3.402  2.704  3.617  3.393  
 
 UTAT33  101927492  2  105713509  105719402  2q12.1  2.503  0.563  1.562  1.875  1.222  0.515  1.603  0.27  1.644  0.626  0.608  0.629  0.623  1.588  0.851  0.93  1.757  1.035  1.28  1.574  0  2.441  2.379  0.619  1.117  0.496  2.55  0.86  1.521  2.217  0.819  0.618  0.955  1.329  1.135  0.772  0.386  0.606  1.125  1.473  0.793  1.906  3.075  0  0  0.995  3.829  1.224  0  2.441  0.512  0.951  1.308  0.991  2.473  0.504  0.78  0.483  0.906  0.822  
 
 GPR45  11250  2  105858199  105859924  2q11.1-q12  0.151  0  0.171  0  0.067  0.074  0  0.079  0.223  0.079  0  0.081  0  0.117  0  0  0.174  0.207  0.051  0.098  0  0.474  0.615  0  0  0.18  0.11  0.119  0.306  0.774  0.142  0.136  0.074  0.292  0.061  0  0  0  0.214  0.319  0.13  0.423  0.067  0  0  0.091  0.042  0.436  0  0.224  0.044  1.391  0.312  0.895  1.129  0.12  0.595  0  0.543  0.624  
 
 TGFBRAP1  9392  2  105880846  105946171  2q12.1  2.721  1.843  1.133  1.421  2.314  1.941  2.423  2.36  2.228  1.99  2.338  1.858  2.634  2.309  2.615  2.167  1.677  1.101  3.531  2.464  1.804  2.54  1.674  1.993  2.229  3.146  2.905  3.12  3.4  2.527  3.108  2.76  3.556  2.211  1.493  2.199  2.492  1.694  2.191  3.329  1.945  1.795  2.663  1.894  1.609  1.71  2.582  2.677  1.965  3.304  1.719  1.576  1.848  1.783  3.171  1.698  2.418  1.741  2.925  3.042  
 
 C2orf49  79074  2  105953815  105965271  2q12.1  1.691  1.733  0.674  0.708  1.638  0.83  1.479  1.245  1.387  0.977  1.391  1.118  1.374  1.873  1.207  1.331  1.251  1.512  2.488  1.461  1.274  1.46  1.125  0.881  1.457  1.336  0.948  0.894  1.147  0.836  1.256  1.34  0.999  0.911  1.122  1.282  1.274  1.076  1.859  1.947  1.209  0.684  1.683  1.186  1.086  0.982  1.64  1.386  0.962  2.944  1.246  0.406  1.034  1.028  1.524  0.802  1.509  1.08  1.444  1.22  
 
 FHL2  2274  2  105977282  106055230  2q12.2  0.7  5.43  5.666  2.664  5.984  4.955  6.068  5.054  4.802  4.052  4.599  4.306  1.981  4.395  2.883  3.524  5.649  5.582  2.84  0.395  2.127  1.55  0.689  0.161  4.943  3.723  5.083  3.721  4.9  2.334  3.959  6.023  4.759  4.133  2.448  3.431  5.449  3.48  6.627  4.877  4.52  2.659  0.846  2.922  3.457  3.465  4.839  6.661  4.752  8.787  4.699  3.415  4.735  4.727  6.165  4.95  4.41  5.187  5.387  6.498  
 
 LOC285000  285000  2  106209553  106227016  2q12.2  0  0  0.158  0  0  0  0.149  0  0.102  0.117  0  0  0  0  0  0  0  0.196  0  0  0  0.079  0.172  0  0  1.501  0.827  2.193  3.23  0.117  2.014  0.504  0.118  0.125  0  0  0  0  0  0  0  0.135  0  0  0  0  0  0  0  0  0  0  0  0.055  0  0  0  0  0  0  
 
 NCK2  8440  2  106361519  106510730  2q12  3.032  3.295  2.559  2.392  2.358  2.614  3.098  3.56  4.35  3.397  3.031  2.403  3.162  3.131  2.695  2.639  3.442  1.402  4.146  2.717  1.663  2.904  2.831  3.033  4.036  4.84  4.816  3.66  4.415  3.963  4.776  3.85  3.9  2.599  2.008  2.697  3.299  3.545  2.522  3.685  3.051  2.591  4.518  2.608  2.4  2.838  4.268  3.579  3.107  4.351  1.932  2.043  3.649  2.28  3.05  2.125  4.234  2.47  3.508  3.393  
 
 C2orf40  84417  2  106682112  106694609  2q12.2  0  0  0  0  0  0  0  0  0  0  0.118  0  0  0  0  0  0  0  4.112  0.158  0  0.606  0  0  0  0  0.079  0.114  0  0.058  0  0  0  0  0  0  0  0  0  0  0  0.084  0  0  0  0.28  0  0  0  0.114  0  0.356  0  0.094  0  0  0  0  0  0  
 
 UXS1  80146  2  106709758  106810795  2q12.2  1.771  3.27  1.715  1.682  1.462  2.03  3.146  2.092  2.649  2.714  2.485  1.94  2.4  2.733  1.747  2.635  2.261  1.894  3.32  0.959  1.497  1.522  1.831  1.381  2.176  3.289  2.894  2.796  3.612  1.683  2.693  3.871  3.227  2.132  1.704  3.166  2.447  2.574  2.611  4.12  3.369  1.972  3.305  3.309  2.009  2.938  2.476  2.165  2.66  4.048  3.268  1.599  2.761  3.104  5.068  2.966  3.373  1.927  3.394  3.414  
 
 PLGLA  285189  2  106998569  107007851  2q12.2  0  0  0  0  0  0  0  0  0  0.115  0  0  0  0  0  0  0  0.202  0.243  0  0  0.462  0  0  0  0  0  0  0  0.152  0  0  0  0  0  0  0  0  0.137  0  0  0  0  0  0  0  0  0  0  0  0  0  0  0.122  0  0  0  0  0  0  
 
 RGPD3  653489  2  107021135  107084801  2q13  0.429  0.54  0.259  0.202  0.202  0.231  0.239  0.375  0.361  0.248  0.395  0.184  0.467  0.664  0.598  0.342  0.294  0.464  0.651  0.244  0.153  0.937  0.304  0.18  0.329  0.402  0.271  0.348  0.55  0.255  0.336  0.505  0.422  0.34  0.218  0.402  0.408  0.285  0.385  0.434  0.249  0.223  0.917  0.312  0.197  0.341  0.052  0.468  0.268  0.492  0.416  0.15  0.364  0.201  0.427  0.291  0.548  0.173  0.339  0.344  
 
 ST6GAL2  84620  2  107418055  107503563  2q11.2-q12.1  0  0.072  0.228  0  0  0.307  0  1.248  0.082  0.056  0.036  0  0  0.078  0  0  0.065  0.067  0  0.14  0  0.289  0.67  1.016  0  0  0.174  0  0  0.16  0  0.074  0  0.271  1.606  0  0  0  0.599  0  0  0.606  0.115  0.133  1.722  0.177  0.074  0.101  0  0.181  1.095  0.566  0  0.104  0.053  0  2.056  0  0  0.666  
 
 GACAT1  104326057  2  108370567  108438585  2q12.3  0  0  0  0  0  0  0  0  0  0  0  0  0  0.128  0  0  0  0  0  0  0  0  0  0  0  0  0.076  0  0  0  0  0  0  0  0  0  0  0  0  0  0  0  0  0  0  0  0.092  0  0  0  0  0  0  0  0  0  0  0  0  0  
 
 RGPD4-AS1  729121  2  108439519  108442583  2q12.3  0  0  0.141  0  0  0  0  0  0  0.035  0  0  0  0  0  0  0  0  0  0  0  0.094  0.252  0  0  0  0.044  0  0  0.062  0  0  0  0  0  0  0  0  0  0  0  0  0  0  0  0  0.042  0  0  0  0  0.137  0  0  0  0  0  0  0  0  
 
 RGPD4  285190  2  108443387  108509000  2q12.3  0.413  0.624  0.251  0.206  0.182  0.172  0.289  0.358  0.439  0.27  0.355  0.155  0.427  0.702  0.629  0.424  0.357  0.493  0.598  0.201  0.151  0.665  0.323  0.161  0.341  0.362  0.349  0.252  0.478  0.134  0.362  0.43  0.38  0.451  0.229  0.33  0.349  0.237  0.472  0.405  0.232  0.271  0.624  0.222  0.241  0.28  0.051  0.372  0.276  0.648  0.353  0.139  0.325  0.238  0.409  0.282  0.527  0.164  0.277  0.385  
 
 SLC5A7  60482  2  108602969  108630443  2q12  0  0.026  0.071  0  0.017  0  0  0  0.041  0  0  0  0  0.026  0  0  0  0.083  0  0.041  0  0.045  0.176  0  0  0  0.043  0  0  0.039  0  0  0  0.17  0  0  0  0  0.015  0  0  0.134  0.021  0  0  0  0.019  0  0  0  0  0.156  0  0.022  0  0  0  0  0  0  
 
 LINC01594  102724774  2  108784203  108834297  -  0  0  0  0  0  0  0  0  0  0  0  0  0  0  1.34  0  0  0.458  0  0  0  0  0  0  0  0  0  0  0  1.566  0.218  0  0  0  0  0  0  0  0  0  0  0  0  0  0  0  0  0  0  0  0  0  0.124  0.187  0  0  0  0  0  0  
 
 SULT1C3  442038  2  108863650  108881807  2q12.3  0  0  0  0  0  0  0  0  0  0  0  0  0  0  0.337  0  0  0  0  0  0  0  0  0  0  0  0  0  0  0  0  0  0  0  0  0  0  0  0.05  0  0  0  0  0  0  0  0  0  0  0  0  0  0  0  0  0  0  0  0  0  
 
 SULT1C2  6819  2  108905094  108926371  2q12.3  0  0  0.068  0  0  0  0.072  0.145  0.076  0  0  0.228  0.184  0.263  1.171  0  0.381  0.628  0  0  0  0.019  0.102  0  0  0.086  0.065  0.051  0.367  1.546  1.719  0.368  0  0.232  0.047  0  0  0.218  0.028  0  0  0.183  0.031  0.159  0.121  0.05  1.022  0  0.627  0.396  0.032  0.094  0.081  0.04  0.065  0  0.101  0  0.069  0.115  
 
 SULT1C2P1  151234  2  108938693  108970254  2q12.3  0  0  0.091  0  0  0  0  0  0.115  0  0  0  0  0  0.161  0  0  0  0  0  0  0.031  0.224  0  0  0  0.026  0  0  0.024  0  0  0  0.368  0  0  0  0  0  0  0  0.139  0.028  0  0  0  0.059  0  0  0  0  0.192  0  0  0.035  0  0  0  0  0  
 
 SULT1C4  27233  2  108994420  109004270  2q12.3  0  0  0.118  0  0  0  0  0.04  0  0  0  0  0  0  0  0  0  0.082  0  0  0  0  0  0  0  0  0.043  0  0  0.036  0  0  0  0.194  0.063  0  0  0  0  0  0  0.426  0  0  0  0  0.032  0  0  0  0  0  0  0  0  0  0.068  0  0  0.274  
 
 GCC2  9648  2  109065576  109125854  2q12.3  1.188  2.374  0.619  0.51  1.042  0.515  1.028  1.46  1.133  1.16  1.376  1.138  1.582  1.774  2.001  2.12  1.605  3.283  1.555  1.038  1.175  1.149  0.826  0.702  1.56  2.064  0.737  0.995  1.385  0.39  1.58  1.67  1.171  1.116  0.973  1.819  0.944  0.855  1.97  1.121  1.076  0.755  1.552  1.13  0.792  1.151  0.438  1.021  1.135  2.205  1.315  0.516  0.95  1.13  1.627  1.011  2.192  0.609  1.409  1.997  
 
 LIMS1  3987  2  109150810  109303702  2q12.3  2.472  3.896  3.047  1.837  2.319  2.532  3.662  3.706  3.7  3.047  4.317  2.053  2.465  3.5  3.141  2.068  2.544  3.876  3.868  2.731  3.331  2.82  1.861  1.802  4.866  2.471  1.753  2.853  2.113  2.056  2.976  4.894  2.868  2.35  2.147  3.595  2.908  2.035  3.846  2.9  2.777  2.378  5.399  2.815  2.074  3.645  2.365  2.531  3.567  4.215  3.131  1.468  2.844  2.138  3.293  2.822  4.427  2.408  3.504  4.852  
 
 RANBP2  5903  2  109335936  109402267  2q12.3  2.109  3.308  1.056  0.962  1.922  1.234  1.856  2.233  1.509  1.606  2.672  1.242  2.412  3.054  2.414  1.896  1.84  3.615  2.815  2.156  2.363  2.056  1.321  1.321  2.043  2.424  0.973  1.796  2.244  1.12  2.317  2.427  1.866  0.99  1.4  2.625  1.923  1.771  2.263  2.592  1.866  1.337  2.506  2.304  1.545  1.986  1.158  2.061  2.281  3.199  2.268  0.992  1.604  1.348  2.127  1.664  2.608  1.325  2.381  2.485  
 
 CCDC138  165055  2  109403205  109493047  2q12.3  0.894  1.761  0.3  0.277  0.646  0.821  0.569  0.563  0.375  0.632  1.266  0.528  0.453  1.482  1.09  1.087  0.973  1.853  2.61  0.7  1.451  1.312  0.646  0.689  0.713  0.573  0.404  0.406  0.548  0.448  1.359  1.064  0.888  0.39  0.499  1.054  0.82  0.459  0.806  1.085  0.527  0.97  1.231  1.186  0.491  0.819  0.986  0.671  1.311  2.298  0.693  0.418  0.649  0.308  0.916  0.764  1.048  0.555  1.078  1.038  
 
 EDAR  10913  2  109510926  109605828  2q13  0.07  0  0.105  0  0.016  0  0.025  0.02  0.085  0.077  0.077  1.167  0.663  0.227  0.314  0.059  0.149  0.218  0.04  0.101  0  0.19  0.285  0.06  0  0.046  0.121  0.027  0  0.066  0.033  0.084  0.042  0.259  0.037  0.146  0.092  0.081  0.086  0  0.106  0.075  0.042  0.222  0  0.126  0.062  0.109  0.248  0.062  0.027  0.265  0.089  0.031  0.047  0.092  0.051  0  0.04  0  
 
 SH3RF3-AS1  100287216  2  109743783  109746575  2q12.3  0.069  0.101  0.344  0.132  0.075  0.353  0.114  0.222  0.292  0.112  0.09  0.121  0  0.081  0.095  0.09  0.083  0.31  0.027  0.156  0  0.405  0.493  0.542  0  0.053  0.253  0.077  0.044  0.201  0.048  0.331  0  0.167  0.11  0  0.394  0.085  0.181  0.089  0.062  0.805  0.098  0.115  0.333  0.16  0.07  0.616  0.167  0.103  0.344  1.035  0.033  0.243  0.131  0.097  0.127  0.076  0.063  0  
 
 SH3RF3  344558  2  109745996  110262213  2q13  0  0.594  1.153  1.152  0.041  1.07  1.792  1.008  1.848  0.508  0.852  0.029  0.075  0.033  0.313  0.03  0.394  0.145  0.166  0.189  0  0.16  1.362  1.909  0.023  0.046  0.065  0.021  0  0.339  0.04  1.438  0  0.303  0.114  0.057  1.87  0.706  1.02  0.382  0.35  0.62  1.273  0.477  0.965  0.297  1.586  1.576  1.44  1.267  1.928  0.479  0  0.127  0.181  0.053  0.563  0.06  0.265  0  
 
 SEPT10  151011  2  110300373  110371783  2q13  0.741  5.155  1.878  1.766  1.658  2.443  1.753  2.422  1.711  2.775  2.879  1.535  2.239  4.096  2.184  2.534  3.147  2.574  0.229  0.12  0.107  0.16  0.348  0.587  3.23  2.491  1.799  1.897  2.198  1.128  2.421  2.473  2.357  2.084  1.568  3.456  2.445  2.09  3.044  2.843  2.292  2.409  4.114  2.931  1.011  2.453  1.897  2.645  2.808  5.135  3.248  1.866  2.011  2.126  3.193  2.354  3.227  2.862  2.908  3.492  
 
 SOWAHC  65124  2  110371910  110376564  2q13  0.673  2.064  0.998  1.189  1.43  0.891  1.019  1.663  0.848  0.653  0.134  1.482  1.929  1.437  0.965  2.099  1.92  0.842  0.045  0.08  0.054  0.28  0.233  0.073  2.558  1.13  0.7  1.394  1.191  0.67  0.911  1.254  1.151  0.87  0.819  2.453  0.882  0.992  1.629  2.144  1.732  1.834  3.487  1.414  1.661  2.022  1.337  2.188  1.649  3.502  2.149  1.422  0.87  1.004  1.556  1.399  1.805  1.425  1.494  1.06  
 
 RGPD6  729540  2  110550334  110615282  2q13  0.1  0.055  0.289  0.127  0.087  0.218  0.313  0.176  0.493  0.134  0.085  0.162  0.179  0.113  0.145  0.201  0.115  0.253  0.08  0.075  0  0.395  0.237  0  0.054  0  0.218  0.121  0.102  0.152  0.144  0.089  0.203  0.431  0.266  0.186  0.037  0.102  0.499  0  0.063  0.304  0.744  0.138  0  0.214  0.017  0.278  0.191  0.137  0.075  0.362  0.072  0.139  0.253  0.229  0.408  0.148  0  0.035  
 
 LIMS3  96626  2  110656008  110683202  2q13  0.729  0.897  1.312  0.361  0.381  0.895  1.265  1.29  1.201  0.892  1.613  0.44  0.709  1.201  0.909  0.503  0.609  1.412  1.051  0.793  0.683  0.841  0.639  0.481  3.824  0.5  0.602  0.705  0.606  0.647  0.801  3.031  0.886  0.683  0.624  1.071  1.215  0.486  1.308  0.87  0.819  0.643  2.571  0.583  0.57  2.555  0.426  0.735  0.881  1.974  0.809  0.282  1.173  0.691  1.181  0.734  1.442  0.675  0.897  2.001  
 
 LOC440895  440895  2  110705266  110726149  2q13  0  0  0  0  0  0  0  0  0  0  0  0  0  0  0  0  0  0  0  0  0  0  0  0  0  0  0.164  0  0  0.111  0  0  0  0  0  0  0  0  0  0  0  0.378  0  0  0  0.508  0.218  0  0  0.484  0  0  0  0  0  0  0.272  0  0  0  
 
 LINC01123  440894  2  110744556  110752858  2q13  1.634  0.869  0.507  0.264  0.051  0.078  0.15  0.59  0.233  0.071  0.102  1.342  2.786  1.874  2.251  2.061  1.642  0.671  0.063  0.598  0  0.22  0.588  0.132  0  0  0.325  0  0  0.481  0.319  1.9  0  0.5  1.479  1.791  1.347  1.296  0.634  1.134  0.844  1.13  0.121  1.404  0.936  2.403  1.505  1.028  0.564  1.547  1.279  1.184  0.914  1.087  0.387  1.092  0.919  0.154  0.855  1.903  
 
 MALL  7851  2  110841446  110874143  2q13  0.116  0.85  0.158  1.052  0.065  0  0.116  0.044  0.191  0.039  0.08  4.166  3.964  5.614  1.488  5.534  5.285  0.135  0.031  0.06  0  0.141  0.504  0  0.085  0.142  0.111  0.03  0.033  0.129  0  0.106  0  0.451  4.631  2.729  3.273  2.483  0.051  0.236  6.212  2.79  0.054  0.137  2.167  2.317  5.095  0  2.863  0.044  5.757  3.331  2.375  4.124  0.057  0.751  3.188  0.148  0  5.285  
 
 NPHP1  4867  2  110880913  110962639  2q13  0.236  0.706  0.151  0.364  0.491  0.185  0.721  0.183  0.562  0.438  0.588  0.132  0.148  0.945  0.288  0.296  0.371  0.819  0.133  0.061  0  0.138  0  0  0.28  0.302  0.442  0.162  0.312  0.088  0.131  1.348  0.194  0.162  0.466  0.318  0.317  0.433  0.375  0.468  0.45  0.444  0.401  0.17  0.313  0.199  1.355  0.521  0.555  1.919  0.376  0.215  0.354  0.201  0.851  0.468  0.235  0.192  0.512  0.401  
 
 LINC00116  205251  2  110969105  110980517  2q13  2.479  1.7  2.527  2.406  2.239  3.252  3.55  1.16  2.986  2.036  1.613  1.762  1.756  2.211  0.719  2.128  2.713  0.619  2.931  3.423  1.212  3.346  2.602  1.511  1.962  1.255  2.243  1.243  1.928  1.886  2.411  2.86  1.783  2.37  2.987  1.833  0.951  1.561  3.62  1.476  2.026  3.995  1.855  0  0.908  2.216  3.251  2.082  1.495  3.449  0.824  1.735  1.498  1.907  3.857  2.211  1.243  2.35  2.271  1.317  
 
 LOC100507334  100507334  2  111003214  111024135  -  0  0  0.189  0  0  0  0  0  0  0  0  0  0  0  0  0  0  0.228  0  0  0  0  0  0  0.168  0  0.088  0  0  0.141  0  0  0  0  0  0  0  0  0  0  0  0.363  0.114  0  0  0.289  0  0  0  0.258  0  0  0  0.095  0  0  0.184  0  0  0  
 
 LINC01106  151009  2  111132685  111142113  2q13  0.438  0.086  0.263  0  0  0  0  0.084  0.186  0.054  0  0.256  1.593  0.336  0.78  1.797  0.692  0  0  0.185  0  0.054  0.335  0  0  0  0.06  0  0  0  0  0.311  0  0.422  0.678  0.478  0.145  0.203  0.182  0.206  0.298  0.498  0  0.412  0.202  1.046  0.756  0.078  0.163  0.702  0.572  0.18  0.163  0.535  0.105  0.341  0.208  0  0.266  1.042  
 
 LIMS3-LOC440895  100271835  2  111160495  111230652  2q13  0  0  0.149  0  0  0  0.058  0.091  0.082  0  0.042  0.094  0  0.047  0  0  0  0.083  0  0  0  0.059  0.159  0  0.288  0  0.155  0  0  0.167  0  0  0  0.136  0.057  0  0.092  0  0  0  0  0.044  0.037  0  0  0.422  0.061  0.108  0  0.089  0  0.297  0.025  0.106  0  0  0  0  0  0.071  
 
 LIMS3L  100288695  2  111222627  111230652  2q13  0  0  0.663  0  0  0  0  0.393  0.148  0  0.525  0  0  0.066  0  0  0  0  0  0  0  0.043  0  0  3.586  0  0.192  0  0  0.073  0  2.503  0  0.225  0  0  0.402  0  0.085  0  0  0  0.532  0  0  2.07  0  0.348  0  1.325  0  0  0.219  0.068  0  0  0.178  0  0  0.12  
 
 RGPD5  84220  2  111271397  111334678  2q13  0.319  1.142  0.133  0.335  0.097  0.115  0.129  0.803  0.381  0.392  0.847  0  0.26  1.292  0.75  0.428  0.414  1.036  0.708  0  0.121  0.102  0  0  0.597  0.673  0.263  0.376  0.604  0.242  0.604  1.094  0.582  0  0.227  0.831  0.57  0.333  0.348  0.745  0.61  0  1.265  0.689  0.358  0.589  0.307  0.603  0.889  1.645  0.767  0.074  0.428  0.073  0.429  0.378  1.369  0.455  0.451  0.833  
 
 BUB1  699  2  111395274  111435684  2q14  3.352  5.382  1.701  2.017  1.97  2.803  2.934  2.483  1.039  2.899  3.817  2.095  3.197  4.117  3.016  2.852  3.093  3.577  4.991  2.89  3.076  3.172  1.681  3.129  3.117  2.935  2.297  2.6  2.997  2.778  3.182  3.266  3.064  1.59  2.446  3.099  3.246  3.972  2.737  4.045  2.943  2.32  4.038  3.018  2.167  3.105  2.641  3.765  2.333  5.239  3.542  2.342  3.047  2.256  3.645  3.281  4.322  3.024  3.416  3.302  
 
 ACOXL  55289  2  111490149  111875799  2q13  0  0.286  0.082  0  0.038  0  0  0.052  0.08  0.05  0  0.099  0  0.389  0.283  0.271  0.076  0.131  0.135  0  0  0.147  0.407  1.343  0  0  0.173  0.057  0  0.098  0.089  0  0  0.133  0  0  0.054  0.202  0.061  0  0  0.22  0.161  0  0.117  0.309  0.061  0  0  0  0  0.665  0.038  0.072  0.047  0.068  0.103  0.075  0  0  
 
 FLJ44006  400997  2  111855917  111873165  2q13  0  0.055  0.093  0  0  0  0  0  0.079  0.016  0  0  0  0.151  0.043  0.049  0  0  0  0  0  0.018  0.263  0.07  0  0  0.064  0.03  0  0.028  0  0  0  0.203  0  0  0  0  0.025  0  0  0.024  0.02  0  0  0.038  0.033  0  0  0  0  0.081  0  0.037  0  0  0  0  0  0  
 
 BCL2L11  10018  2  111878490  111926022  2q13  1.726  1.954  0.508  1.269  0.954  0.745  1.064  0.946  2.37  1.237  1.404  1.927  1.016  1.671  1.107  1.734  1.212  2.139  1.829  2.132  1.529  2.107  2.434  3.142  0.626  0.96  0.938  1.316  1.747  0.787  1.719  1.435  1.14  0.886  1.24  1.472  0.959  0.942  1.259  1.522  1.054  1.367  1.304  1.462  1.876  1.786  1.163  0.748  0.799  1.768  1.302  0.562  0.867  1.082  1.672  1.369  1.993  0.945  2.326  0.982  
 
 MIR4435-2HG  541471  2  112124590  112252692  2q13  2.055  8.206  5.466  4.378  3.614  6.191  5.074  5.295  4.48  5.446  4.887  3.986  3.223  3.235  2.308  3.892  4.521  4.408  3.768  5.499  4.763  3.468  5.621  5.698  5.776  5.141  4.19  4.849  4.604  4.025  5.32  4.844  4.595  4.29  4.971  4.237  4.595  5.283  8.024  4.051  5  5.448  5.8  5.281  4.104  4.217  6.063  4.788  4.436  7.149  4.203  3.417  3.779  5.13  5.588  5.834  4.664  6.121  3.988  4.296  
 
 ANAPC1  64682  2  112525213  112641741  2q12.1  2.644  2.677  1.1  0.987  1.567  1.306  2.376  1.432  1.234  1.46  2.295  1.401  2.65  2.867  2.573  2.052  2.086  2.477  3.443  2.939  2.606  2.672  1.301  1.681  1.959  2.233  1.478  2.169  2.395  2.265  2.474  2.677  2.618  1.14  1.682  2.596  2.704  2.195  2.009  3.032  2.195  1.426  2.746  2.063  1.686  1.544  2.019  2.408  1.685  3.485  2.239  1.296  2.049  1.594  2.84  2.244  2.839  1.866  2.523  2.613  
 
 MERTK  10461  2  112656190  112786945  2q14.1  0.393  0.981  0.081  0  0.065  0.325  0.085  0.184  0.456  0.077  0.158  0.059  0.588  1.01  0.568  0.474  0.192  0.319  0.197  0.07  0.288  0.127  0.456  0.481  0  2.276  0.249  0.112  1.269  4.542  2.686  0.979  0  0.222  0.306  0.543  1.322  0.18  0.111  1.823  1.467  0.304  1.065  1.632  0.402  0.198  1.383  0.881  0.175  1.251  0.489  0.541  0.505  0.191  0.603  1.015  0.709  0.708  1.046  0.697  
 
 TMEM87B  84910  2  112812799  112876895  2q13  1.799  2.112  0.997  0.752  1.895  0.557  1.672  1.326  0.839  1.35  1.484  1.204  1.971  1.605  1.43  1.866  2.051  1.785  1.885  1.055  0.692  0.979  1.184  0.721  1.853  2.552  1.759  1.155  1.798  1.18  1.82  2.446  1.176  0.96  0.672  1.631  1.99  1.206  2.751  1.597  1.983  0.776  2.33  0.757  0.537  0.689  1.233  0.951  0.7  2.414  1.779  0.77  1.162  1.277  1.621  1.206  2.475  0.72  1.702  1.979  
 
 FBLN7  129804  2  112895961  112945791  2q13  0  0.689  2.582  0  0.184  0.759  0.896  1.176  0.591  1.523  0  0.111  0  0.048  0.763  0.149  0  0.191  0.854  0.067  0  0.481  0.545  0.197  0  0.777  0.568  0.137  0.055  0.101  0.113  0.087  0.212  0.6  0.148  0  0.09  0.201  0.164  0.122  0.058  1.102  1.363  0.064  0.154  0.305  1.073  1.903  0  2.837  0.356  0.871  0.222  0.12  0.902  0.249  1.934  1.19  0  0.917  
 
 ZC3H8  84524  2  112973438  113012664  2q13  2.491  3.071  0.928  0.739  1.858  1.053  2.022  0.6  1.269  0.993  1.551  1.727  1.83  2.37  1.231  1.37  1.763  4.151  3.524  1.776  1.859  2.757  1.922  0.829  1.526  1.638  0.616  1.42  1.146  1.094  2.182  1.894  1.386  1.207  1.522  1.279  1.427  1.528  1.7  1.504  1.315  0.996  3.009  0.745  1.229  1.231  1.81  1.22  0.867  2.919  1.595  0.515  1.366  1.1  1.914  1.709  1.652  1.251  1.779  1.205  
 
 ZC3H6  376940  2  113033177  113097640  2q13  1.03  0.3  0.407  0.493  0.363  0.304  0.512  0.413  0.602  0.825  0.461  0.058  0.632  0.542  0.652  0.462  0.675  0.439  0.506  0.168  0.176  0.371  0.568  0.227  0.176  0.689  0.425  0.442  0.163  0.331  0.763  0.195  0.298  0.379  0.134  0.919  0.659  1.051  0.435  0.617  0.369  0.36  0.609  0.099  0.08  0.241  0.39  0.15  0.23  0.201  1.05  0.38  0.341  0.325  0.871  0.622  0.601  0.129  0.25  0.667  
 
 RGPD8  727851  2  113125945  113191222  2q13  0.173  0.189  0.371  0.231  0.055  0.373  0.508  0.225  0.606  0.229  0.093  0.289  0.157  0.299  0.068  0.432  0.194  0.818  0.158  0.04  0.058  0.531  0.274  0  0.16  0.258  0.351  0.174  0.145  0.191  0.328  0.125  0.377  0.425  0.512  0.41  0.065  0.072  0.989  0  0.146  0.39  0.617  0.251  0  0.24  0.414  0.523  0.437  0.296  0.137  0.359  0.112  0.262  0.432  0.561  0.204  0.445  0  0.083  
 
 TTL  150465  2  113239742  113290222  2q13  2.05  3.905  2.443  2.302  1.287  2.568  3.333  2.439  2.423  3.161  2.882  0.74  2.091  2.978  2.874  1.778  2.482  2.954  3.233  2.468  2.017  2.708  1.269  1.247  3.222  3.602  3.547  3.351  3.668  3.273  3.266  3.616  3.793  2.52  2.124  2.939  3.281  3.262  2.64  3.426  1.149  2.355  4.414  1.74  1.401  2.117  3.348  3.499  2.817  4.674  3.388  1.509  3.079  1.904  3.665  2.649  3.948  2.131  3.629  3.13  
 
 POLR1B  84172  2  113299491  113334729  2q13  2.637  2.241  1.013  1.708  1.624  1.35  2.054  1.285  1.681  1.054  2.042  1.572  2.789  2.817  2.551  2.185  1.95  2.175  3.54  2.991  2.182  1.533  2.066  2.1  2.422  2.168  1.646  2.973  2.303  2.305  2.258  3.08  3.537  2.278  1.555  2.306  2.551  1.981  1.946  2.887  1.805  1.357  3.149  2.256  1.856  1.545  2.358  2.346  1.601  3.734  2.176  1.083  1.525  1.349  1.788  2.434  2.034  1.72  2.173  2.116  
 
 CHCHD5  84269  2  113342013  113346617  2q13  5.735  2.883  3.322  3.613  4.991  4.002  6.08  3.765  3.888  3.546  3.921  4.088  4.123  5.696  3.862  4.515  4.234  1.056  5.553  5.144  4.239  4.741  4.011  4.099  5.038  4.283  5.156  4.578  4.579  4.963  4.942  5.251  4.748  3.563  4.976  4.699  4.027  3.994  4.178  5.048  3.566  4.902  0  4.248  2.213  3.585  6.259  5.126  4.65  6.814  2.581  3.767  4.17  4.018  6.079  5.534  3.897  5.376  4.814  3.941  
 
 FLJ42351  400999  2  113399406  113401757  2q13  0  1.216  0  0  0.218  0.323  0  0  0  0.855  0  0  0  0.122  0  0  0  0.358  0.434  0  0  0.359  0  0  0  0.906  1.481  0.32  0.451  0.089  0.662  0.347  0.611  0  0.236  0  0.365  0.407  1.416  0.729  0  0.287  0  0  0  0.425  0.141  0  0  0.465  0.391  0  0.19  0.211  1.128  0.405  0.472  0  0  0  
 
 SLC20A1  6574  2  113403433  113421400  2q13  2.648  4.979  2.565  3.5  2.638  2.499  4.612  4.267  3.412  2.937  3.681  2.09  2.419  4.786  2.735  2.266  3.229  3.069  4.94  2.22  3.482  2.832  1.782  1.692  5.602  5.05  4.366  4.838  5.84  2.248  3.879  4.903  3.746  2.599  2.423  3.002  5.202  4.506  4.101  3.836  3.545  3.058  3.268  2.014  1.775  2.016  3.378  3.199  4.081  4.69  4.585  1.924  3.783  2.701  4.15  3.76  3.729  3.336  4.102  3.444  
 
 CKAP2L  150468  2  113493926  113522254  2q13  1.365  4.209  1.905  1.265  1.434  2.242  3.03  1.608  0.66  2.259  2.464  0.777  1.377  2.494  1.792  1.405  2.115  2.28  3.146  1.664  1.262  2.06  0.959  0.701  2.637  2.028  1.597  1.689  1.96  1.119  1.779  2.239  2.077  1.397  1.727  1.777  2.265  2.574  2.727  2.175  2.054  1.837  1.705  1.963  1.086  1.344  2.382  2.653  2.333  3.126  2.875  1.209  1.961  1.395  2.249  1.947  2.964  2.376  2.253  2.119  
 
 IL1A  3552  2  113531491  113542971  2q14  0.167  0.59  0.146  0  0.044  0.428  0.787  0.041  0.105  0.159  0.29  0.079  0.128  0.162  0.2  0.036  0.09  0.072  0  0.076  0  0.064  0.26  0  4.993  0.146  0.116  0.114  0.131  0.044  0  0.088  0.242  0.237  0  0  1.208  0.327  3.379  0.098  0.284  0.397  0.234  0.055  0  0.284  0.032  0.279  0.534  0.468  0.413  0.354  0.173  0.137  0.054  0.058  0.389  1.054  0.06  0.983  
 
 IL1B  3553  2  113587336  113594356  2q14  0  0.334  2.553  0.218  0  0.451  3.852  0.188  0  0.139  0.411  0.107  0.268  0.127  0.137  0.077  0.225  0  0  0.764  0  0.143  0.252  0  8.45  0.145  0.413  0.147  0.9  0.205  0.247  0.416  0.114  0.459  0  0  3.246  1.388  6.77  0  0  0.447  0.043  0  0  0.239  0.096  0.344  0.135  0.624  6.301  0.254  0.179  0.232  0.065  0  0.481  0.172  0  0.221  
 
 IL37  27178  2  113670547  113676458  2q12-q14.1  0  0  0  0  0  0  0.505  0  0  0  0.226  0.373  0  0.112  0  0  0.84  0  0  0  0  0  0  0  0  0  0.271  0  0  0  0  0  0  0  0  0  0  0  0.453  0  0  0  0  0  0  0  0  0  0  0.223  0.876  0.363  0  0  0  0  0.356  0  0  0  
 
 IL36G  56300  2  113735595  113743249  2q12-q21  0  0.106  0  0  0  0  0.295  0  0  0.064  0  0  0  0.13  0  0  0.207  0.185  0  0  0  0  0  0  0  0  0.049  0  0  0.058  0  0  0  0  0  0  0  0.267  0.108  0  0.123  0.07  0.05  0  0  0  0  0  0  0.09  0  0.331  0.079  0.076  0  0  0.128  0  0  0  
 
 IL36A  27179  2  113763448  113765621  2q12-q14.1  0  0  0  0  0  0  0  0  0  0  0  0  0  0  0  0  0  0  0  0  0  0.3  0  0  0  0  0  0  0  0  0  0  0  0  0  0  0  0  0.107  0  0  0  0  0  0  0  0  0  0  0  0  0  0  0  0  0  0  0  0  0  
 
 IL36B  27177  2  113779667  113810444  2q14  0  0  0.206  0  0  0  0.106  0  0  0.054  0.081  0  0  0  0  0  0  0  0  0  0  0  0.287  0  0.313  0  0.085  0  0  0.085  0  0  0  0.558  0  0  0  0.245  0.098  0  0  0.106  0  0  0  0  0.047  0  0  0  0  0.344  0  0  0  0  0  0  0  0  
 
 IL36RN  26525  2  113816214  113822320  2q14  0  0  0.171  0  0.043  0  0.074  0  0.102  0  0  0  0  0  0.101  0.979  0  0  0  0.039  0  0.029  0.336  0  0.244  0  0.05  0  0  0.056  0  0  0  0.335  0  0  0  0  0.026  0  0.562  0.027  0.02  0  0  0.049  0  0  0  0  0  0.149  0  0  0  0  0  0  0  0  
 
 IL1F10  84639  2  113825546  113833427  2q13  0  0  0  0  0  0  0  0  0.132  0  0  0  0  0  0  0.079  0  0  0  0  0  0.067  0  0  0  0  0.06  0  0  0.065  0  0  0  0.294  0  0  0  0  0  0  0  0  0  0  0  0  0  0  0  0  0  0.334  0  0  0  0  0  0  0  0  
 
 IL1RN  3557  2  113875469  113891593  2q14.2  0.349  0  0.231  0  0.045  0  0  0.067  0.095  0.06  0  0  0.423  0.092  0.59  3.258  0.324  0  0  0.328  0.17  0.071  0.404  0  0  0  0.089  0.063  0  0.055  0  0  0  0.283  0  0  0  0.81  0.437  0  0.371  0.087  0  0.158  0.214  0.829  0  0  0  0  0.14  0.293  0  0.177  0  0  0.139  0  0  0  
 
 PSD4  23550  2  113931559  113960677  2q13  2.521  0.027  0.227  0  1.524  0.096  0.765  1.166  0.715  0.209  1.529  2.649  2.127  0.743  1.451  2.672  1.84  0.149  4.099  1.993  2.456  2.633  1.494  2.267  0.598  0.029  0.264  0.59  0.053  0.114  0.082  0.136  0.244  0.274  0.353  1.23  1.048  0.566  0.16  0.734  2.511  0.51  0.05  0.632  1.943  1.256  0.619  0.338  0.562  0.704  1.425  0.707  0.159  0.315  0.957  0.479  0.686  0.082  1.039  0.898  
 
 PAX8  7849  2  113973573  114036498  2q13  0.545  0.207  0.218  1.058  0.036  0.071  1.237  0.134  0.614  0.742  0.52  0.817  0.864  1.017  0.446  0.837  0.23  0.258  0.611  0.187  0.595  0.315  0.916  0.468  0.056  0.623  0.126  0.076  0.33  0.398  0  0.043  0  0.219  0.216  0.402  0.102  0.656  0.698  1.213  0.047  0.758  0.818  5.889  5.686  4.688  0.177  3.614  5.132  4.855  0.976  0.4  4.838  3.656  5.958  5.026  5.003  0.954  6.087  5.174  
 
 PAX8-AS1  654433  2  113993103  114024600  2q13  2.558  2.127  0.317  3.634  0  0  0.902  0.141  0.343  3.165  0.216  2.37  2.545  3.266  2.371  3.383  0.095  1.999  0.135  0.596  2.196  1.24  2.263  1.553  0.062  1.621  0.127  0.178  0.917  0.922  0  0.06  0  0.489  0.135  0.457  0.052  0.235  0.516  2.335  0.05  1.613  2.036  4.345  2.917  2.8  2.783  2.246  3.808  1.73  3.115  0.48  3.048  1.42  3.73  2.707  3.638  2.856  3.457  2.121  
 
 CBWD2  150472  2  114195267  114253781  2q13  2.888  3.516  0.998  0.845  2.946  1.865  2.72  1.992  1.539  2.122  2.682  1.636  1.599  3.474  1.97  2.34  2.367  5.189  4.128  3.651  1.896  2.659  2.099  2.332  1.612  1.693  1.302  1.293  1.339  0.983  2.175  2.371  1.999  0.953  1.529  2.574  2.108  2.102  2.223  2.188  1.99  1.724  2.489  2.359  1.556  2.415  3.03  1.454  2.164  3.803  2.139  1.23  1.844  1.27  2.503  1.883  1.885  1.278  2.249  1.68  
 
 FOXD4L1  200350  2  114256660  114258727  2q13  0.143  0.055  0.143  0  0.053  0  0.191  0.078  0.346  0.123  0.194  0  0  0.225  0  0.09  0.167  0.256  0.086  0.067  0.158  0.208  0.565  0  0  0.1  0.119  0.081  0  0.09  0.061  0.071  0.069  0.243  0.122  0.176  0.084  0.31  0.112  0.095  0  0.299  0.071  0.186  0.263  0.065  0.186  0.144  0.109  0.147  0.056  0.494  0.039  0.183  0.129  0.115  0  0  0.091  0  
 
 PGM5P3-AS1  101929127  2  114285144  114300250  9p24.3  0  0  0  0  0  0  0.908  0  0  0.453  0  0  0  0  0  0  0  0  0  0  0  0  0  0  0  0  0  0  0  0  0  0  0  0.567  0  0  0  0  0  0  0  0  0  0  0  0  0  0  0  0.503  0.342  0  0  0  0  0  0  0  0  0  
 
 PGM5P4-AS1  103344932  2  114286223  114300267  2q14.1  0  0  0  0  0  0  0.666  0  0.271  0.338  0  0  0  0.143  0  0  0  0  0  0  0  0.122  0.642  0  0  0  0.148  0  0  0.121  0  0  0  0.635  0  0  0  0  0.166  0  0  0  0  0  0  0  0.079  0  0  0.355  0.834  0.828  0  0.184  0.168  0  0  0  0  0  
 
 FAM138B  654412  2  114334958  114336429  2q14.1  0  0  0.202  0  0  0  0.312  0  0.175  0  0.108  0  0  0  0  0  0  0  0  0  0  0.041  0.548  0  0  0  0.076  0  0  0  0  0  0  0.38  0  0  0  0  0  0  0  0.159  0  0  0  0  0  0  0  0  0.168  0.191  0  0  0.163  0  0  0  0  0  
 
 WASH2P  375260  2  114341229  114356613  2q13  2.742  0.212  1.497  1.838  2.632  1.197  3.119  1.847  2.123  2.872  2.111  2.19  2.15  2.535  2.559  2.587  1.94  0.078  3.478  2.073  2.113  2.945  1.973  1.787  1.616  2.044  3.009  2.399  2.815  2.409  2.899  2.702  2.389  1.334  1.686  2.3  2.809  2.246  1.511  2.353  1.896  2.47  2.998  1.567  1.708  1.823  2.559  2.315  1.128  3.436  2.221  1.539  2.183  1.273  2.868  1.509  1.684  1.895  2.46  1.806  
 
 DDX11L2  84771  2  114356604  114361294  2q13  0.507  0  0.636  0.145  0.093  0.589  0.776  0.267  0.348  0.542  0.193  0.049  0.191  0.871  0.422  1.188  0.094  0.141  2.502  0.165  1.055  1.443  0.936  0.181  0.447  0.491  0.652  0.378  1.142  0.132  0.602  0.74  0.995  0.627  0.439  0.606  0.727  0.788  0.132  0.801  0.244  0.391  0.727  0.491  0.133  0.563  0.091  0.869  0.977  1.843  0.461  0.291  0.309  0.052  0.262  0.215  0.875  0.895  0.782  0  
 
 RPL23AP7  118433  2  114368815  114384715  2q14  2.074  3.729  1.824  1.661  2.094  1.586  2.943  1.885  1.307  1.972  1.999  1.082  1.133  2.327  1.206  2.027  2.081  3.7  2.979  2.91  1.725  2.446  1.693  1.042  2.108  1.662  2.015  1.331  1.862  1.251  2.44  2.766  2.296  2.249  1.828  1.767  1.747  2.064  2.746  1.544  1.633  1.543  1.973  1.938  1.167  1.804  0.988  2.224  1.575  4.058  2.457  1.254  1.92  1.142  2.823  2.079  1.781  2.306  2.407  2.093  
 
 RABL2A  11159  2  114384805  114400975  2q13  1.393  1.131  0.648  0.946  1.037  0.418  1.227  0.956  1.535  1.616  1.654  1.43  1.236  1.608  1.257  1.242  1.536  1.626  2.02  1.694  0.32  2.241  0.896  0.446  0.889  1.037  1.39  0.916  1.051  1.131  1.001  1.087  1.341  1.41  1.296  1.283  0.865  0.688  2.019  1.321  1.291  1.098  2.261  0.886  1.078  0.829  1.603  0.858  1.078  1.591  1.122  1.149  1.33  1.351  2.071  1.396  0.95  0.805  1.532  1.095  
 
 SLC35F5  80255  2  114470368  114514400  2q14.1  1.384  1.923  1.24  0.685  1.801  1.054  1.486  1.768  1.176  1.025  1.479  0.768  1.281  0.775  1.696  1.326  1.282  1.571  1.542  0.984  0.921  0.637  0.739  0.454  1.626  1.917  1.074  1.709  2.129  1.634  1.836  2.246  1.832  1.356  1.137  2.842  1.393  1.62  1.889  2.14  1.906  1.231  1.331  1.755  2.35  1.737  1.375  1.405  1.801  2.924  1.966  0.946  1.594  1.739  2.286  2.759  2.445  1.369  2.561  2.83  
 
 LOC101060091  101060091  2  114588759  114648569  -  0  0.43  0  0  0.167  0  0.853  0.221  0  0.453  0  0  0.412  0  0  0  0  0  0.593  0.577  0  0.752  0.625  0  0  0  0.651  0  0.197  0.214  0.269  0  0  0.327  0  0.356  0  0  0.77  0  0  0  0  0  0.525  0  0.552  0  0  0  0.568  0  0  0.614  0.212  0  0  0  0  0  
 
 ACTR3  10096  2  114647510  114719129  2q14.1  2.63  5.005  3.776  3.009  2.612  3.023  4.057  4.339  3.201  3.761  4.179  2.887  3.619  3.915  2.712  3.345  3.565  4.238  5.227  3.913  3.083  3.197  2.673  2.101  4.025  3.672  3.11  3.139  3.914  3.382  3.548  3.872  3.964  2.878  2.847  3.992  3.729  3.486  4.285  3.746  3.055  2.886  3.266  3.537  3.447  3.497  3.103  3.405  3.111  5.301  3.869  2.004  3.531  3.731  3.906  4.107  4.529  2.981  4.198  4.26  
 
 LOC100499194  100499194  2  114735245  114737561  2q14.1  0  0  0.136  0  0  0  0  0.085  0.124  0.157  0.155  0.059  0  0.037  0  0.089  0.044  0.075  0  0.104  0  0.155  0.449  0  0  0.097  0.149  0.227  0.194  0.272  0  0.127  0.19  0.301  0  0.2  0.197  0.24  0.034  0  0  0.108  0.05  0.39  0  0.144  0.036  0.109  0.209  0  0.046  0.293  0.057  0.032  0.065  0  0.228  0  0.186  0  
 
 LINC01191  440900  2  114737145  114764887  2q14.1  0  0  0.276  0  0  0  0.511  0  0  0.275  0.746  0  0  0.205  0  0  0.254  0.312  0.178  0  0  0.145  0.422  0  0  0  0.282  0.172  0.182  0.107  0.301  0.653  0.173  0.314  0  0  0.173  0.325  1.045  0  0  0.418  0  0.908  0  0  0  0.232  0.303  0.326  0  0.435  0  0  0  0  0  0  0.466  0  
 
 DPP10  57628  2  115199898  116602326  2q14.1  0  0  0.059  0  0.016  0  0  0.02  0.053  0.017  0.026  0  1.069  0.016  0  0.024  0  0.047  0  0  0  0.047  0.113  0  0  0  0.093  0  0  0.089  0  0  0  0.177  0  0  0.042  0  0.576  0  0  0.786  0.041  0  0  0  0.055  0  0  0  0  0.201  0  0.059  0.025  0.038  0  0  0  0  
 
 DPP10-AS3  101927591  2  115585917  115591125  2q14.1  0  0  0  0  0  0  0  0  0  0  0  0  0  0  0  0  0  0  0  0  0  0  0  0  0  0  0.057  0  0  0  0  0  0  0  0  0  0  0  0  0  0  0  0  0  0  0  0  0  0  0  0  0.426  0  0  0  0  0  0  0  0  
 
 DPP10-AS1  389023  2  115901624  115918920  2q14.1  0  0  0  0  0  0  0  0  0  0  0  0  3.256  0  0  0  0  0  0  0  0  0.09  0  0  0  0  0.176  0  0  0.104  0  0  0  0  0  0  0  0  1.219  0.29  0  2.716  1.058  0  0  0  0  0  0  0  0  0.43  0  0  0  0  0  0  0  0  
 
 DDX18  8886  2  118572254  118589953  2q14.1  3.495  5.063  2.044  2.504  2.742  2.608  3.418  2.714  2.944  2.565  3.418  2.459  3.152  4.432  2.882  3.273  3.525  5.019  5.045  4.32  3.586  3.645  3.071  2.57  3.427  2.978  2.452  2.894  2.616  2.222  3.124  3.614  3.559  2.943  2.99  3.34  3.095  2.572  3.369  3.509  2.915  2.845  3.829  3.875  2.719  2.071  2.856  2.915  2.529  5.108  2.779  1.836  2.609  2.277  4.288  3.797  3.532  2.935  3.285  3.691  
 
 CCDC93  54520  2  118673053  118771739  2q14.1  1.437  2.332  0.725  0.981  1.001  0.891  1.776  1.956  1.335  1.52  1.452  1.18  1.908  1.717  1.678  1.42  1.404  1.624  2.499  1.547  0.934  1.564  0.797  0.785  1.779  1.716  1.885  1.773  1.677  1.685  1.693  1.378  2.164  1.054  0.932  1.485  1.783  1.564  1.903  1.781  1.238  0.796  1.945  1.66  0.899  0.904  2.117  2.242  1.929  3.208  1.706  0.779  1.496  1.165  2.131  1.687  2.603  1.055  2.258  1.725  
 
 INSIG2  51141  2  118846049  118867597  2q14.2  1.577  3.337  1.829  0.949  1.539  1.23  2.169  1.564  1.963  2.071  2.349  1.542  1.994  2.016  1.743  2.39  3.186  3.282  3.089  1.46  0.726  1.642  1.399  0.72  1.387  1.929  0.707  1.228  1.814  1.072  1.415  1.815  1.627  1.152  0.794  1.876  1.823  1.75  1.48  1.309  2.142  1.304  1.415  2.198  0.485  1.119  2.768  1.502  1.332  3.926  2.215  0.865  1.345  1.464  1.512  2.126  3.021  0.811  1.489  1.991  
 
 LOC101927709  101927709  2  119357358  119360542  2q14.2  0  0  0  0  0  0  0  0  0.136  0  0  0  0  0.045  0  0  0  0  0  0  0  0.066  0.211  0  0  0  0.054  0  0  0.05  0  0  0  0.211  0  0  0  0  0  0  0  0  0  0  0  0  0  0  0  0  0  0  0  0  0  0  0  0  0  0  
 
 EN1  2019  2  119599746  119605759  2q14.2  0  0.079  1.423  1.755  0.05  1.923  0.474  1.406  1.535  1.613  0.636  0.061  0  0.061  0.089  0.103  0.05  0.356  0.027  0.073  0.152  0.277  0.45  0.041  0  0.045  0.998  0.03  0.902  0.177  0.515  0.982  0.699  0.755  0.424  1.361  0.039  0  0.132  0.146  0.055  0.968  0.071  0.058  0.124  0.096  0.121  0.232  0.058  0.387  1.082  1.141  0.044  0.728  0.139  0.114  0.039  0  0  0.08  
 
 MARCO  8685  2  119699744  119752236  2q14.2  0  0  0.072  0  0  0.235  0  0  0.09  0  0  0.843  0  0.032  0  0  0  0.082  0.05  0  0  0.119  0.193  0  0  0  0.085  0  0  0.044  0  0  0  0  0  0  0  0  0.26  0  0.173  0  0.054  0  0  0  0.026  0.271  0  0.293  0  0.367  0  0.054  0  0  0  0  0  0  
 
 C1QL2  165257  2  119913818  119916471  2q14.2  0  0.342  0.153  0  0.117  0.138  0.05  0.14  0.173  0.101  0.086  0.164  0  0.091  0  0.101  0.086  0.356  0  0.189  0  0.376  0.384  0.417  0  0.069  0.278  0.038  0.179  0.184  0.052  0  0  0.157  0.089  0.079  0  0  0.158  0.234  0  0.492  0.099  0  1.171  0.127  1.427  0  0.077  0  0  1.175  0.072  0.265  0.125  0.167  0.215  0  0  0  
 
 STEAP3  55240  2  119981383  120023227  2q14.2  2.3  1.792  2.837  1.683  2.069  2.319  4.112  5.423  3.864  3.905  3.321  1.781  2.627  2.784  2.487  1.783  1.216  0.505  0.106  2.114  2.527  0.332  1.255  1.609  4.085  2.383  2.796  1.977  0.591  2.499  1.721  2.879  3.604  2.42  2.487  3.418  3.175  4.46  2.544  3.151  2.733  2.228  1.933  1.974  1.075  0.792  1.841  2.591  1.051  3.94  3.197  2.142  3.502  2.735  4.113  2.025  4.414  1.452  3.83  3.698  
 
 STEAP3-AS1  100874111  2  120001997  120006647  -  0.213  0.07  0.268  0.114  0.077  0.146  0.199  0.462  0.228  0.284  0.233  0.041  0.247  0.155  0.26  0.079  0  0.087  0  0.054  0.151  0.072  0.455  0.097  0.219  0.204  0.166  0.192  0.066  0.041  0.064  0.177  0.384  0.351  0.178  0.184  0.288  0.626  0.08  0.24  0.167  0.176  0.26  0.121  0.202  0.058  0.227  0.302  0.122  0.146  0.21  0.367  0.521  0.295  0.353  0.102  0.698  0.093  0.494  0.127  
 
 C2orf76  130355  2  120060019  120124258  2q14.2  0.941  2.192  0.279  0.772  1.331  0.373  0.745  0.644  0.429  0.92  0.736  0.155  0.388  1.077  0.42  0.256  0.575  1.679  1.568  0.454  0  1.314  0.679  0.603  0.234  0.835  0.578  0.415  0.432  0.138  0.686  0.96  0.683  0.329  0.711  0.868  0.654  0.4  0.697  0.893  1.541  0.673  0.912  0.652  0.393  1.101  0  0.34  0  1.607  0.676  0.64  0.416  0.511  1.171  0.398  0.702  1.039  0.59  0.433  
 
 DBI  1622  2  120124499  120130127  2q12-q21  6.825  9.216  4.813  5.79  6.979  6.287  6.977  6.372  5.437  6.035  6.443  7.251  5.89  7.01  4.758  6.287  7.267  9.454  7.501  6.726  4.157  6.92  4.999  5.989  6.536  5.768  6.277  5.321  5.732  4.733  6.925  7.016  6.768  5.762  6.38  6.32  5.444  5.866  6.341  5.387  5.958  5.891  6.044  5.784  5.308  5.639  6.437  6.089  5.302  8.219  5.563  4.345  5.795  6.102  7.099  5.654  5.967  7.035  6.744  6.118  
 
 TMEM37  140738  2  120189445  120196096  2q14.2  1.072  0  0.161  0.39  0.151  0  0.308  0.988  0.398  0.226  0.685  0.284  2.053  1.06  0.104  0.587  0  0.229  0  0  0.177  0.133  0.647  0.084  0  0  0.091  0  0  0.103  0  0.148  0  0.31  2.099  1.011  0.639  0.14  0.838  0.828  0.462  2.85  0.31  0.796  0.727  0.249  0.609  0  0.817  0.779  0  0.987  0.557  2.446  1.971  0.188  0.951  0.078  0.314  2.568  
 
 SCTR  6344  2  120197418  120282028  2q14.1  0  0  0.116  0  0  0  0  0.052  0  0  0  0  0  0.056  0  0  0  0.106  0  0.078  0  0.081  0.288  0.082  0  0  0.085  0.054  0  0.062  0  0  0  0.181  0.069  0  0  0  0.125  0  0  0.274  0.075  0  0  0  0.041  0  0  0  0  0.505  0  0.154  0.145  0  0  0.088  0  0  
 
 CFAP221  200373  2  120302007  120414237  2q14.2  0  0.062  0.08  0  0.389  0  0  0.046  0  0  0.042  0.067  0  0.137  0  0  0  0  0  0  0.102  0.036  0.122  0  0  0  0.056  0  0  0.066  0  0  0  0.087  0.057  0.286  0  0  0.04  0  0  0.095  0.036  0.084  0  0.043  0.03  0  0  0  0  0.269  0.048  0.301  0.825  0.147  0.131  0  0.091  0  
 
 TMEM177  80775  2  120436742  120439694  2q14.2  2.455  2.879  1.587  2.482  2.45  1.915  2.921  2.03  2.829  2.357  2.969  2.615  3.325  3.619  2.993  2.789  2.516  3.531  3.87  2.802  3.326  2.264  1.796  2.274  2.595  2.964  2.73  3.445  3.369  2.889  3.879  3.926  4.038  2.715  3.04  2.881  2.693  2.525  1.866  3.525  2.809  2.856  3.895  2.987  2.225  1.969  2.268  3.345  1.96  5.312  2.221  1.884  2.738  2.06  3.642  2.724  2.434  2.899  2.901  2.836  
 
 PTPN4  5775  2  120517206  120742474  2q14.2  0.543  0.613  0.209  0.19  0.368  0.289  0.499  0.506  0.628  0.438  0.604  0.618  0.864  0.806  0.668  0.846  0.801  0.912  1.103  0.325  0.469  0.646  0.481  0.275  0.374  0.53  0.296  0.545  0.418  0.287  0.42  0.436  0.498  0.319  0.377  0.89  0.492  0.502  0.463  0.582  0.647  0.366  0.672  0.558  0.431  0.495  0.349  0.813  0.626  1.955  0.782  0.348  0.41  0.461  0.778  0.346  0.732  0.32  0.827  0.546  
 
 EPB41L5  57669  2  120770603  120936697  2q14.2  3.193  1.682  0.593  0.527  1.452  0.944  1.135  0.779  1.413  0.811  1.509  0.865  1.846  1.955  1.922  1.038  1.182  1.609  0.919  0.182  0.725  0.867  0.817  0.235  0.337  0.979  0.723  1.187  1.007  0.912  0.829  1.118  1.115  0.727  0.777  1.408  1.108  0.988  0.375  1.303  1.625  0.838  1.893  1.824  0.843  1.302  0.751  1.716  1.731  3  1.07  0.691  0.771  0.652  0.978  0.635  1.526  0.309  1.485  0.492  
 
 TMEM185B  79134  2  120975046  120980984  2q14.2  2.822  2.04  1.833  1.529  2.039  1.674  3.014  2.608  1.932  2.144  1.827  2.069  2.483  1.946  2.292  2.618  1.832  1.443  2.577  1.743  1.659  2.128  1.207  0.856  2.458  2.374  2.013  2.27  2.127  1.564  2.406  2.477  2.297  1.687  2.233  2.864  2.275  2.138  2.213  2.566  2.035  1.89  2.931  2.161  1.574  1.457  2.618  3.442  2.691  4.198  2.573  1.832  1.96  2.144  3.002  1.891  2.687  2.133  2.697  1.972  
 
 RALB  5899  2  121010413  121052286  2q14.2  1.47  1.314  2.483  2.39  1.907  1.348  3.608  2.593  2.564  2.578  2.279  2.509  3.478  1.743  2.474  3.312  2.878  0.945  0.59  1.015  1.693  0.32  1.545  0.985  2.824  3.64  2.573  2.703  3.576  2.248  3.356  2.835  2.893  2.308  1.978  2.983  2.435  2.588  2.091  2.132  2.38  1.751  3.106  2.089  1.772  1.652  1.981  3.207  3.288  4.445  3.785  1.742  2.808  2.802  3.04  2.8  3.561  2.964  3.238  2.778  
 
 INHBB  3625  2  121103718  121109383  2cen-q13  1.729  0.633  2.581  0  4.217  1.086  0.292  3.512  0.698  1.596  0.072  1.088  0.155  0.217  0.196  0.12  0.126  5.751  0.049  0.124  0  0.377  0.314  0.357  0.04  0.066  0.199  0.07  0.05  0.204  0.033  0  0  0.244  3.472  1.347  0.288  0.046  0.326  0.366  2.631  0.705  0.321  1.689  2.706  2.087  2.617  1.271  0.313  2.338  1.354  1.134  2.475  0.991  0.141  0.226  2.726  0.04  0  0.151  
 
 LINC01101  84931  2  121221910  121223925  2q14.2  0  0  0.158  0  0  0  0  0  0.148  0.109  0  0  0  0.055  0  0  0  0  0  0.061  0  0.065  0.428  0  0  0  0.098  0.046  0  0.078  0  0  0  0.28  0.051  0  0.102  0  0.037  0  0  0.199  0.061  0  0  0.1  0.027  0  0  0  0  0.329  0  0.054  0.049  0  0  0  0  0  
 
 GLI2  2736  2  121554866  121750229  2q14  0.047  0.714  1.397  0  0.078  1.318  2.629  0.872  0.234  0.475  0.815  0.152  0.052  1.273  1.468  0.444  0.374  0.12  0.055  0.08  0.081  0.232  0.413  0.5  0.056  0.37  0.178  1.357  0.161  0.417  0.149  1.179  0  0.259  2.48  2.626  1.895  1.567  1.048  0.316  0.288  1.086  0.743  0.417  0.775  0.545  1.724  2.361  1.931  1.737  1.076  1.762  1.583  0.846  2.575  1.333  1.124  0.087  0.023  1.573  
 
 TFCP2L1  29842  2  121974163  122042778  2q14  0.044  0.044  0.176  0.043  0.16  0.107  0.159  0.015  1.064  0.042  0.473  1.183  0.069  1.13  0.685  0.02  0.321  0.423  0  0.064  0.283  0.067  0.593  0.292  0.049  0.58  0.483  0.058  0.297  0.071  0  0.014  0  0.252  0.671  0.535  0.013  0.044  0.043  0.524  0.389  0.178  0.563  0.711  0.273  0.518  0.405  0.156  0.866  0.242  0.025  0.511  0.027  0.067  0.102  0.033  0.027  0.017  0.018  0.028  
 
 CLASP1  23332  2  122095351  122407052  2q14.2-q14.3  2.27  1.534  1.585  1.249  1.077  1.467  2.797  2.151  1.687  2.039  2.277  1.009  1.897  2.046  2.226  1.741  1.495  1.006  2.517  0.995  1.716  1.809  1.339  1.475  1.819  2.372  2.444  2.523  2.521  1.897  2.135  2.246  2.599  1.162  1.479  2.338  2.287  1.877  1.346  2.525  1.958  1.347  2.672  1.942  1.333  1.445  1.12  2.181  1.935  2.467  2.061  1.224  1.689  1.998  2.376  1.297  2.806  1.566  2.415  2.446  
 
 RNU4ATAC  100151683  2  122288455  122288585  2q14.2  0  2.717  5.603  0  0  4.719  0  0  0  0  0  3.515  0  2.807  0  1.976  2.51  0  0  0  0  0  0  2.575  0  0  0  0  0  0  0  0  0  0  3.879  0  0  0  0  0  0  8.057  3.008  0  0  0  0  0  0  0  0  0  1.851  2.067  0  3.136  0  0  0  0  
 
 NIFK-AS1  254128  2  122407229  122486136  2q14.2  1.717  2.539  1.4  1.356  1.213  0.612  1.532  1.08  1.716  1.314  1.408  0.894  1.3  1.691  1.148  1.34  1.243  2.979  2.955  2.202  1.807  1.586  2.466  0.944  1.436  1.22  1.66  1.696  1.65  1.027  1.84  1.936  1.721  2.033  1.3  1.162  1.49  1.278  1.521  1.409  0.873  1.687  2.168  1.139  0.711  0.625  1.305  1.271  1.185  2.35  1.333  0.722  1.147  0.96  1.833  0.902  1.361  1.35  1.521  1.34  
 
 NIFK  84365  2  122484520  122494503  2q14.3  3.165  5.802  2.38  2.797  3.069  2.68  3.715  3.052  2.901  2.659  3.578  2.569  2.975  4.213  2.553  2.971  3.222  6.017  5.095  4.101  3.62  3.553  3.476  2.89  3.827  3.059  2.194  2.692  2.607  1.641  3.318  3.528  3.156  2.968  2.572  3.07  3.11  2.639  4.019  3.287  2.404  3.055  3.8  3.804  2.634  2.522  2.856  2.612  3.036  4.993  3.172  1.675  2.776  2.596  3.218  2.537  3.519  2.604  3.718  3.231  
 
 TSN  7247  2  122513120  122525428  2q21.1  3.411  4.62  2.22  2.738  2.938  2.079  3.535  2.721  3.42  2.826  3.406  2.626  3.155  4.387  3.377  2.854  3.127  4.731  5.167  3.814  2.919  3.137  3.477  2.264  3.045  3.518  2.642  2.712  3.213  2.198  3.176  3.428  3.031  2.696  2.3  3.143  2.718  2.449  3.492  3.522  2.571  2.417  3.811  3.237  2.307  2.224  3.277  2.559  3.197  4.882  3.171  1.887  2.691  2.561  3.457  2.187  3.51  3.024  3.477  3.26  
 
 CNTNAP5  129684  2  124782863  125672954  2q14.3  0  0.021  0.09  0  0.021  0.02  0.029  0.013  0.158  0.024  0  0.035  0  0.027  0  0.026  0  0.057  0  0.06  0  0.086  0.289  0  0  0.016  0.101  0.013  0  0.096  0  0.02  0  0.226  0.041  0  0  0  0.032  0  0  0.142  0.045  0  0  0.029  0.062  0  0  0  0.028  0.269  0.016  0.047  0.021  0  0  0.032  0  0  
 
 GYPC  2995  2  127413510  127454251  2q14-q21  0  0  4.141  4.249  0  1.54  5.079  3.252  0.318  2.653  0.409  0  0  0.037  0  0  0  0  6.868  3.388  5.628  0.824  0.363  0  0  5.54  7.848  5.555  7.338  6.646  7.724  6.076  6.425  5.568  0  2.693  3.07  2.241  2.787  0.732  0  1.265  0.32  0  0  0.276  0.099  0  0  0.332  1.731  0.378  4.049  1.15  2.255  0.933  4.676  0  3.538  2.361  
 
 BIN1  274  2  127805598  127864903  2q14  0  0.915  2.067  2.629  0.241  2.48  3.641  3.825  2.541  2.967  2.576  3.717  2.802  2.065  2.728  2.817  1.998  1.137  3.212  0.25  0.278  1.655  1.382  0.449  1.002  1.308  2.435  1.095  0.849  0.171  0.424  2.937  3.299  2.309  2.289  4.185  2.819  2.535  2.412  2.118  3.097  3.518  0.616  3.665  1.166  2.519  2.872  2.802  3.192  4.758  2.196  1.765  2.995  3.135  3.745  3.409  3.516  2.555  2.695  3.05  
 
 CYP27C1  339761  2  127941411  127963343  2q14.3  0  0  0.429  0.774  0.033  0.247  0.925  1.027  0.11  0.524  0.078  0.046  0.033  0.019  0  0.033  0.048  0.136  0.015  0.036  0  0.08  0.27  0  0.1  0.065  0.399  0.048  0.083  0.059  0  0.068  0.056  0.286  0.06  0.526  0.218  0  0.042  0.78  0.666  0.223  1.159  0  0  0.038  0.025  0.038  0.838  0.413  0.591  0.234  0.104  0.08  0.063  0.125  0.086  0.113  0  0  
 
 ERCC3  2071  2  128014865  128051752  2q21  3.929  3.153  1.857  2.36  2.76  2.186  3.092  2.8  2.88  2.828  3.242  1.93  2.843  3.693  2.821  2.586  2.733  2.334  4.607  2.131  2.421  3.307  1.935  1.464  2.687  3.078  2.846  2.953  2.97  2.346  2.915  3.178  3.531  2.174  2.112  3.198  2.945  2.636  3.522  3.512  2.854  2.369  4.069  2.629  2.236  2.157  2.793  2.909  2.256  4.981  2.975  1.768  2.118  1.997  3.556  1.688  2.926  2.282  2.704  2.482  
 
 MAP3K2  10746  2  128056244  128100805  2q14.3  1.862  2.718  1.128  0.852  1.2  0.964  2.085  2.087  1.426  1.624  2.26  1.116  1.93  1.74  1.494  1.579  1.334  2.243  2.011  1.178  0.546  1.523  1.393  0.845  1.431  1.816  1.096  1.335  1.195  0.792  1.653  1.76  1.528  0.995  1.136  1.772  1.499  1.48  1.796  2.115  1.49  0.824  1.781  1.107  0.955  0.922  0.913  1.299  2.037  1.889  1.708  0.565  1.435  1.312  1.205  1.286  2.455  1.072  1.485  1.488  
 
 PROC  5624  2  128175995  128186822  2q13-q14  0.314  0  0.201  0  0.188  0.139  0.331  0.086  0.087  0.17  0.313  0.555  1.183  0.113  0.373  0.418  0.607  0.294  0.152  0.202  0  0.627  0.55  0.136  0.083  0.104  0.258  0.151  0.098  0.422  0.1  0  0  0.26  0.57  0.193  0.114  0  0.401  0.268  0.172  0.351  0.119  0  0.353  0.731  0.102  0.133  0.421  0.274  0.155  0.751  0.212  0.305  0.284  0.222  0.21  0  0.14  0  
 
 IWS1  55677  2  128238382  128284087  2q14.3  2.995  3.937  2.027  1.935  2.402  2.214  3.156  3.06  2.286  2.383  3.024  2.113  2.842  3.561  2.461  2.533  2.626  4.285  4.293  2.976  2.565  3.279  1.413  1.663  2.845  2.602  2.06  2.496  2.788  1.795  3.145  3.082  2.726  1.698  2.298  3.209  2.714  2.319  2.765  3.152  2.764  1.962  3.612  2.789  1.932  1.966  2.57  2.608  2.267  4.215  2.747  1.719  2.33  2.218  3.311  2.041  3.614  2.169  2.664  2.809  
 
 MYO7B  4648  2  128293377  128395303  2q21.1  0.049  0.032  0.23  0.063  0.042  0.467  0.219  0.121  0.137  1.132  0.045  1.608  1.819  0.07  0.162  0.166  0.109  0.13  0.286  0.083  0  3.796  0.177  0.036  2.584  0.033  0.159  0.271  0.058  0.102  0.043  0.051  0  0.181  0.052  0.05  0.027  0  0.102  0  0.068  0.353  0.082  0.072  0.084  0.084  0.049  1.884  0  2.497  0.534  0.611  0.077  0.122  0.137  0.041  0.274  0.056  0.021  0  
 
 LOC105373609  105373609  2  128317619  128328537  -  0  0  0.111  0  0  0  0  0.03  0.167  0  0  0.086  0.27  0.035  0  0.036  0  0  0  0  0  0.078  0.444  0  0  0  0.049  0  0  0.039  0  0  0  0.312  0  0  0  0  0  0  0  0.07  0.029  0  0  0  0.029  0  0  0  0  0.14  0  0.064  0  0  0  0  0  0  
 
 LIMS2  55679  2  128395995  128439360  2q14.3  0.216  0.677  5.576  1.814  0.415  0.945  0.539  1.141  0.342  2.395  0.157  0.085  0.12  1.021  0.13  0.848  0  0.262  6.138  0.097  0  5.964  0.672  0.248  0  1.63  0.275  0.587  0.696  0.187  0.835  0.295  0.115  0.215  0.571  3.183  0.455  0.289  1.989  0.818  0.116  0.892  2.592  0.938  0.651  2.3  0.124  3.694  0.676  3.943  0.719  1.227  0.435  0.592  1.091  0.305  4.163  1.076  1.539  0.851  
 
 GPR17  2840  2  128403438  128410213  2q21  0  0.048  0.642  0  0.075  0.098  0.045  0.271  0.251  0.304  0.096  0.081  0  0.097  0  0.085  0.107  0.161  0.092  0.066  0  0.499  0.642  0  0  0.109  0.173  0.384  0  0.129  0  0  0  0.177  0.138  0.327  0.05  0  0.083  0  0  0.231  0.087  0.076  0.09  0.359  0.092  0.623  0.225  0.295  0.056  0.708  0.06  0.2  0.181  0  0.756  0.069  0.152  0  
 
 SFT2D3  84826  2  128458596  128461407  2q14.3  1.695  1.347  1.28  1.126  1.781  0.869  1.487  1.475  2.498  1.524  1.517  1.013  1.333  1.112  0.975  1.708  1.58  1.482  2.807  1.71  1.277  2.624  1.961  0.537  0.821  1.177  1.581  1.046  1.03  0.969  1.232  1.314  1.237  1.184  1.012  1.709  0.998  0.974  1.553  1.435  1.563  1.885  2.128  1.234  0.779  0.548  1.6  1.104  0.892  2.443  0.735  1.519  0.56  0.971  1.825  1.019  1.276  0.734  0.995  0.845  
 
 WDR33  55339  2  128461807  128568761  2q14.3  2.75  3.759  1.451  1.516  2.078  1.876  2.452  2.513  2.4  2.255  2.826  1.906  2.837  3.309  2.884  2.588  2.309  3.494  3.915  2.933  2.574  3.037  1.787  2.081  2.273  2.484  1.701  2.292  2.522  1.591  2.555  2.843  2.35  1.474  1.822  2.611  2.425  2.498  2.591  3.119  2.579  1.84  3.629  2.888  1.911  1.946  2.725  2.526  2.491  3.838  2.352  1.572  2.225  1.739  2.584  1.63  3.119  2.136  2.57  2.277  
 
 POLR2D  5433  2  128603839  128615729  2q21  4.494  4.04  2.508  2.952  3.059  2.335  4.326  3.058  3.194  2.709  3.835  3.116  3.504  3.988  3.117  3.334  2.808  3.26  4.821  4.267  3.612  4.187  3.188  2.892  3.466  2.674  3.345  3.457  3.403  3.116  3.402  3.698  3.875  2.388  2.813  2.875  3.979  3.011  3.292  4.065  2.741  2.848  4.965  2.806  2.842  2.146  4.428  2.98  2.905  4.469  3.064  1.891  3.156  2.314  3.61  2.434  3.436  3.03  3.272  3.197  
 
 AMMECR1L  83607  2  128619206  128643514  2q21  2.209  1.54  1.675  1.63  1.468  1.25  1.947  2.376  1.897  1.593  1.806  0.921  1.764  1.803  1.807  1.712  1.541  1.182  2.413  1.469  1.637  1.804  1.256  0.9  2.028  2.557  2.488  2.152  2.496  1.907  2.551  2.308  2.545  1.396  1.171  2.096  2.128  1.624  1.762  2.37  1.534  1.13  2.961  1.683  1.535  1.227  1.108  2.154  1.797  3.007  1.998  0.936  1.591  1.2  1.765  1.332  2.437  1.366  2.062  1.646  
 
 SAP130  79595  2  128698790  128784869  2q14.3  4.469  3.635  2.085  1.662  3.006  2.619  3.703  3.156  1.738  2.929  3.302  2.169  2.92  3.223  3.034  3.077  2.356  2.317  3.423  2.351  2.881  3.463  1.432  2.288  2.835  3.144  3.251  3.824  3.474  2.719  3.049  3.528  3.944  1.837  2.338  2.707  3.764  3.715  2.731  3.783  2.708  1.805  4.241  2.646  2.556  2.63  2.237  3.869  3.43  3.799  3.144  2.254  3.44  2.289  3.542  2.393  3.859  2.966  3.161  3.139  
 
 UGGT1  56886  2  128848753  128953249  2q14.3  2.251  1.973  1.544  1.06  1.878  1.542  2.635  2.783  1.877  2.479  2.28  1.98  2.213  2.281  1.833  1.64  1.974  1.411  2.413  2.364  1.924  1.805  1.243  1.476  2.191  2.454  1.837  2.478  2.35  1.58  2.026  2.429  2.788  1.507  1.513  2.499  2.565  2.336  1.908  2.755  2.322  1.733  2.107  2.099  1.828  1.643  1.951  1.947  1.891  2.434  2.313  1.444  2.018  1.938  2.38  1.648  2.875  1.973  2.201  2.936  
 
 HS6ST1  9394  2  129023053  129076171  2q21  2.576  1.388  2.182  2.116  1.368  1.774  1.807  2.547  2.775  2.475  2.168  1.288  2.546  2.124  2.812  1.189  1.225  1.247  4.862  1.607  0.801  2.562  1.905  2.542  1.053  1.406  1.508  1.557  1.909  1.489  1.667  1.958  2.366  1.042  1.276  2.381  1.859  1.73  1.14  2.903  2.279  2.317  2.137  3.947  3.524  2.64  1.582  2.492  1.93  3.307  1.232  1.69  1.646  1.208  1.194  0.977  1.978  0.805  2.062  1.377  
 
 LOC101927881  101927881  2  129622173  129626301  2q14.3  0  0  0.162  0  0  0  0  0  0  0  0  0  0  0  0  0  0  0  0  0  0  0  0  0  0  0  0  0  0  0.07  0  0  0  0  0  0  0  0  0  0  0  0.386  0  0  0  0  0  0  0  0  0  0.793  0  0.054  0  0  0  0  0  0  
 
 LOC151121  151121  2  129999745  130031464  2q21.1  0  0  0.147  0  0  0  0  0  0.117  0  0  0  0  0.034  0  0  0  0.349  0  0  0  0.037  0.373  0  0  0  0.044  0  0.227  0.04  0  0  0  0.218  0  0  0  0  0.041  0.212  0  0.032  0.032  0.218  0  0  0  0  0  0  0  0.104  0  0  0  0  0  0  0  0  
 
 LOC389033  389033  2  130680434  130691890  2q21.1  0  0  0  0  2.638  1.382  0.478  1.72  0  0.764  0  0  0  0  0  0.244  0  0  0  0  0  0.139  0  0  0  0  0.138  0  0  0  0  0  0  0  0  0  0  0  0  0  0  1.203  0  0  0  0  0  0  0  0.508  0  1.267  0  0  0.596  0  0  0  0  0  
 
 LOC101927924  101927924  2  130680749  130704276  2q21.1  0  0  0  0  0.052  0  0  0.039  0  0  0  0  0  0  0  0  0  0  0  0  0  0.033  0.229  0  0  0  0.046  0  0  0.043  0  0  0  0.198  0  0  0  0  0.022  0  0  0.494  0.03  0  0  0.03  0.02  0  0  0  0  0  0  0  0  0  0  0  0  0  
 
 RAB6C-AS1  100131320  2  130724164  130738045  2q21.1  0.248  0  0.176  0  0.547  0  0  0.044  0.122  0.073  0.058  0  0  0.112  0  0.03  0  0  0.061  0.093  0  0.126  0.416  0  0  0  0.233  0.048  0  0.08  0  0  0  0.338  0.059  0.086  0  0  0.048  0.086  0  0.343  0.136  0.067  0.072  0.049  0.025  0  0  0.036  0.155  0.081  0.111  0.183  0.119  0  0  0  0  0  
 
 RAB6C  84084  2  130737234  130740311  2q21.1  1.509  0.556  0.954  1.129  0.941  0.355  0.724  0.334  1.007  0.791  0.475  0.97  1.03  1.232  0.578  0.873  1.153  0.641  0.595  0.882  0.276  0.831  1.09  0.511  1.823  0.951  0.942  0.226  0.551  0.576  0.629  0.452  0.613  0.936  0.4  1.296  0.52  0.789  2.197  0.589  0.506  0.365  2.142  0.363  0.564  0.552  0.856  0.575  1.038  0.468  0.821  0.476  0.966  0.439  0.702  0.67  0.392  0.42  0.83  0.551  
 
 FAR2P1  440905  2  130783571  130808704  2q21.1  1.765  0  0.494  0  0.213  0  0.033  0.02  0.118  0.024  0  0  0  0.546  0.043  0.029  0.278  0.397  0.025  0.042  0.209  0.028  0.579  0.039  0  0.031  0.108  0.046  0.797  0.074  0.032  0.027  0  0.255  0.071  0.477  0.034  0  0.021  0.436  0  0.15  2.253  0.556  0.276  0.232  0.572  0.423  0  0.848  0  0.142  0.015  0.042  0  0  0  0.26  0  0  
 
 POTEF  728378  2  130831107  130886795  2q21.1  2.849  0.423  2.817  2.603  1.439  0.951  2.789  2.194  2.308  1.984  2.636  0.816  1.621  1.315  1.497  1.228  1.64  0.319  2.25  1.368  0.889  1.115  0.789  0.769  1.185  1.11  3.165  1.613  2.209  2.513  1.007  1.066  1.423  0.961  1.318  2.165  1.632  1.386  1.704  2.437  1.289  1.831  2.765  1.473  1.376  1.175  1.58  1.981  0.537  2.229  1.908  1.321  2.661  1.954  1.982  1.117  1.8  1.282  1.562  1.766  
 
 MED15P9  285103  2  130887195  130896986  2q21.2  0.133  0  0.154  0  0  0  0.079  0  0.19  0.058  0  0.084  0  0.077  0  0.054  0  0.054  0.046  0  0.261  0.098  0.298  0  0  0  0.104  0.03  0.284  0.123  0  0  0  0.323  0  0  0  0  0.067  0  0  0.342  0.434  0  0  0  0.144  0  0  0.173  0  0.295  0.043  0.059  0.059  0.074  0.046  0  0  0  
 
 CCDC74B  91409  2  130896855  130902707  2q21.1  1.596  0.627  0.626  0.883  1.752  0.56  1.884  0.373  0.914  0.951  0.777  0  0  0.973  0.515  0.229  0.277  0.664  0.907  0  0  1.023  0.291  0.29  1.483  0.703  0.34  0.415  0.158  0.996  0.211  0.166  0.165  0.326  1.15  2.142  0.889  1.041  1.214  3.192  0.541  1.35  4.651  0  0.22  0  1.194  0.85  0.249  1.703  0.56  0.895  0.835  1.385  1.25  0.899  0.71  0.964  0.168  0.272  
 
 SMPD4  55627  2  130908964  130940323  2q21.1  4.004  2.605  2.445  2.36  3.112  2.453  3.947  4.175  2.725  3.649  3.966  3.017  4.091  3.806  4.365  3.641  3.795  2.033  4.918  2.938  4.058  3.631  2.311  3.013  3.679  3.34  3.859  2.808  4.111  3.098  3.688  4.246  4.421  2.134  2.926  3.791  4.22  4.179  2.766  4.648  3.972  3.646  4.275  3.483  3.133  3.218  3.036  4.688  3.652  5.721  3.576  2.892  3.893  3.434  3.855  2.893  4.658  3.557  4.407  3.884  
 
 MZT2B  80097  2  130939500  130948300  2q21.1  6.534  3.817  5.031  5.318  5.63  5.594  6.87  5.688  6.038  5.235  4.718  4.99  5.328  5.851  4.519  5.189  6.053  2.683  7.367  7.45  4.365  6.942  6.088  5.773  4.84  3.976  6.105  2.971  5.241  4.836  6.046  5.578  5.067  4.506  5.886  5.134  4.066  5.208  5.947  4.579  5.235  7.097  6.76  3.525  4.419  4.255  7.343  6.041  3.934  7.546  4.239  4.536  5.253  5.065  6.843  4.575  4.711  6.565  5.659  3.725  
 
 TUBA3E  112714  2  130949317  130956034  2q21.1  0.282  0.067  0.201  0  0.071  0  0.318  0.071  0.126  0.206  0.193  0  0.203  0.165  0  0.107  0.084  0  0.163  0.119  0  0.277  0.413  0  0  0  0.522  0.272  0.101  0.277  0.109  0.102  0.126  0.225  0  0  0.071  0  0.15  0  0  0.276  0.341  0  0  0.065  0.117  0  0  0.08  0.215  0.375  0.103  0.198  0.159  0.173  0.15  0.103  0  0  
 
 CCDC115  84317  2  131095505  131100254  2q21.1  3.232  2.435  1.604  1.465  2.937  0.914  2.991  1.919  2.395  1.875  2.405  1.607  2.971  2.865  1.943  2.398  2.44  3.148  4.153  2.362  2.772  2.833  2.192  1.491  2.228  3.044  3.024  1.573  2.817  1.968  2.804  2.678  2.914  2.273  1.527  2.418  1.754  2.23  2.345  2.152  2.686  1.893  3.431  1.752  1.453  1.939  1.887  1.417  1.3  3.563  2.176  1.681  1.831  2.089  2.973  1.044  2.609  2.477  1.957  2.269  
 
 IMP4  92856  2  131100488  131104197  2q21.1  5.784  3.417  3.371  3.331  3.417  3.653  6.43  4.175  3.524  3.78  4.556  4.422  5.022  4.744  4.701  3.949  3.697  1.982  4.981  4.208  4.616  3.909  3.506  4.342  5.015  4.184  5.69  4.355  5.351  5.994  5.065  6.095  5.636  3.078  4.511  4.25  5.021  4.465  3.966  5.068  4.725  4.298  5.778  3.765  3.996  3.696  5.865  4.938  3.406  6.476  4.373  3.304  4.936  4.217  4.865  4.118  4.288  4.73  4.833  4.314  
 
 PTPN18  26469  2  131113579  131132982  2q21.1  3.542  2.127  1.922  1.553  2.442  1.314  3.369  3.199  2.134  3.258  2.839  3.244  3.512  2.623  3.402  3.428  3.132  1.231  2.749  3.032  3.2  1.415  3.695  4.118  2.619  4.389  4.063  2.777  3.854  2.826  3.318  2.864  4.593  2.931  2.665  3.49  2.551  3.632  1.488  2.893  2.437  3.175  3.976  2.425  1.17  2.546  3.361  2.981  2.773  3.516  1.783  2.287  2.636  2.711  3.207  2.407  3.624  2.059  3.333  2.632  
 
 FAR2P2  100216479  2  131174325  131186119  2q21.1  0.624  0  0.247  0  0.042  0.113  0.123  0.042  0.142  0  0  0  0  0.059  0  0  0  0.228  0  0  0.261  0.042  1.402  0.43  0  0.452  0.074  0.542  0.073  0.047  0  0  0  0.41  0.155  0.365  0.152  0  0.046  0.904  0  0.338  0.816  0.13  0.136  0  0.034  0  0  0  0  0.622  0  0.041  0.23  0  0  0.141  0  0  
 
 CYP4F43P  646802  2  131193234  131199254  2q21.1  0.651  0  0.172  0  0  0  0  0  0.116  0  0  0  0  0.095  0  0  0  0  0  0  0.245  0  0.604  0  0  0  0.039  0  0.187  0.031  0  0  0  0.284  0  0  0  0  0  0.094  0  0.152  0.447  0.175  0.123  0.078  0  0.095  0  0.064  0  0.487  0  0  0  0  0  0  0  0  
 
 POTEI  653269  2  131220388  131266808  2q21.1  2.756  0.259  3.812  4.068  1.592  1.767  3.037  2.785  3.75  2.53  3.048  1.26  2.159  1.442  1.759  1.751  1.991  0.162  2.4  2.059  0.825  1.432  0.846  0.94  1.635  1.433  3.756  1.945  2.454  3.133  1.4  1.436  1.838  1.708  1.384  2.044  2.072  1.806  2.738  2.415  1.758  3.118  2.156  1.302  1.565  1.558  1.643  2.341  0.857  2.217  2.372  1.568  3.275  2.846  2.75  1.562  2.337  1.579  1.948  1.907  
 
 CFC1B  653275  2  131278666  131285565  2q21.1  0  0.103  0.213  0  0  0  0.202  0.196  0.158  0.169  0.129  0.224  0  0.197  0.204  0.205  0.167  0  0.169  0.248  0.188  0.304  1.184  0.469  0.148  0.105  0.238  0.081  0.201  0.146  0.173  0.243  0.31  0.222  0.129  0  0  0.323  0.209  0.181  0.097  0.166  0.495  0  0  0  0.095  0.23  0  0.191  0.096  0.468  0.226  0.322  0.292  0.207  0.149  0.331  0  0  
 
 TISP43  150527  2  131294142  131307471  2q21.1  0  0  0.161  0  0  0  0  0  0  0.025  0  0  0  0.021  0  0  0  0  0  0  0  0  0.2  0  0  0  0.083  0  0  0.026  0  0  0  0.22  0  0  0  0  0.038  0  0  0.039  0.037  0  0  0  0  0  0  0  0  0  0  0.04  0  0  0  0  0  0  
 
 LOC646743  646743  2  131328401  131341734  2q21.1  0  0  0  0  0  0  0  0  0  0  0  0  0  0  0  0  0  0  0  0  0  0  0  0  0  0  0  0  0  0  0  0  0  0.2  0  0  0  0  0  0  0  0  0  0  0  0  0  0  0  0  0  0  0  0  0  0  0  0  0  0  
 
 CFC1  55997  2  131349737  131357148  2q21.1  0  0  0.274  0  0  0  0  0.06  0.177  0  0  0  0  0  0  0  0  0  0  0  0  0.06  1.459  0.228  0  0  0.068  0  0  0.109  0  0  0  0.424  0  0  0  0  0.053  0  0  0.172  0  0  0  0  0  0  0  0  0  0.214  0  0.05  0  0  0  0  0  0  
 
 POTEJ  653781  2  131369105  131415610  2q21.1  2.962  0.524  3.586  3.465  1.792  1.551  3.192  2.756  3.171  2.473  2.988  1.272  2.051  1.586  1.583  1.946  1.98  0.161  2.357  1.973  0.744  1.845  1.182  0.918  1.495  1.446  3.663  1.848  2.513  3.285  1.413  1.51  1.919  1.489  1.626  2.058  1.976  1.803  2.593  2.276  1.669  2.594  2.709  1.25  1.514  1.455  1.447  2.3  0.834  2.196  2.184  1.46  3.176  2.634  2.882  1.78  2.218  1.456  1.917  1.915  
 
 CYP4F30P  100132708  2  131437622  131443435  2q21.1  0.629  0  0.149  0  0  0  0  0  0.131  0.027  0  0  0  0.099  0  0  0  0  0  0  0.327  0.027  0.513  0  0  0  0.046  0  0.147  0.035  0  0  0  0.215  0  0  0  0  0  0.132  0  0.162  0.359  0.148  0  0.043  0.052  0.102  0  0.096  0  0.066  0  0.037  0  0  0  0  0  0  
 
 GPR148  344561  2  131486642  131487909  2q21.1  0  0  0.3  0  0  0  0  0  0.133  0.071  0  0  0  0.049  0  0  0  0.177  0  0  0  0.123  0.488  0  0  0  0.106  0  0  0.114  0  0  0  0.219  0  0  0  0  0.035  0  0  0.074  0  0  0  0  0.058  0  0  0  0  0.806  0  0  0.061  0  0  0  0  0  
 
 AMER3  205147  2  131513076  131525707  2q21.1  0  0.032  0.113  0  0.024  0.022  0.039  0.034  0.149  0.038  0.025  0.042  0  0.044  0  0.025  0.037  0.12  0.022  0.023  0  0.096  0.449  0.028  0  0.033  0.119  0.029  0.029  0.112  0  0.026  0  0.263  0  0  0  0  0.058  0  0.025  0.162  0.055  0  0.037  0.035  0.079  0  0  0  0.018  0.519  0.025  0.109  0.021  0.034  0  0  0  0  
 
 ARHGEF4  50649  2  131674223  131804826  2q22  0.663  0.439  0.37  0.151  0.142  0.029  0.115  0.151  2.493  0.699  1.598  0.135  0.386  1.409  0.866  0.085  1.159  0.235  0.329  0.091  0  0.336  0.51  0.378  0  1.13  0.622  0.075  0.421  0.482  0.306  0.095  0.072  0.23  0.164  0.716  0.068  1.258  0.479  0.889  1.407  1.315  0.864  0.577  0.343  0.34  0.947  0.802  0.954  1.728  1.135  1.042  0.082  0.388  0.402  0.243  0.145  0.216  1.014  0.07  
 
 FAM168B  130074  2  131805448  131851004  2q21.1  3.508  3.165  2.797  2.527  3.087  2.369  4.354  3.901  3.731  3.419  4.014  2.947  3.603  3.444  3.731  2.879  3.428  3.615  4.655  2.763  2.844  3.228  2.567  2.663  3.764  3.456  4.324  2.997  3.935  3.85  3.865  4.174  3.969  2.222  2.982  3.873  4.155  3.254  2.995  4.411  3.639  2.635  4.743  3.119  2.933  2.666  3.526  3.558  3.51  4.874  3.613  2.036  3.554  3.063  3.567  2.689  3.875  3.151  3.569  3.604  
 
 PLEKHB2  55041  2  131862419  131907425  2q21.1  3.743  4.944  2.993  2.818  2.572  2.111  5.053  3.28  4.017  3.312  4.197  3.619  4.614  4.513  3.973  3.473  3.936  3.359  4.679  3.682  2.949  2.899  3.135  2.681  3.904  4.706  4.355  3.772  4.74  4.036  4.418  4.842  4.659  3.376  2.391  3.773  4.353  4.188  3.669  4.706  3.662  2.571  4.271  3.436  2.793  2.963  3.503  3.277  2.437  4.664  4.454  2.115  3.084  2.998  4.707  3.165  3.478  3.391  3.899  4.577  
 
 POTEE  445582  2  131975923  132022416  2q21.1  3.208  0.565  3.582  3.577  1.801  1.551  3.282  2.757  3.238  2.605  3.125  1.353  2.127  1.712  1.775  1.982  2.164  0.178  2.583  2.106  2.168  1.908  0.978  0.93  1.597  1.526  3.733  1.917  2.663  3.307  1.44  1.506  1.892  1.588  1.738  2.229  2.265  1.773  2.591  2.574  1.705  2.737  2.671  1.905  1.639  1.52  1.513  2.363  0.999  2.45  2.322  1.667  3.37  2.821  2.932  1.736  2.347  1.608  2.09  2.037  
 
 LOC440910  440910  2  132036862  132057392  2q21.1  1.374  0  0.201  0  0.07  0.128  0  0  0.174  0  0  0  0  0.124  0  0.052  0  0.161  0  0.102  1.389  0.153  0.522  0  0  0  0.074  0  0.197  0.098  0  0  0  0.305  0  0.076  0  0  0.093  0.218  0  0.329  1.044  0.324  0.148  0.076  0.032  0  0  0.086  0  0.399  0  0.067  0.043  0.09  0  0  0  0  
 
 WTH3DI  150786  2  132118064  132121731  2q21.1  1.556  0.7  0.896  0.916  0.81  0.172  0.782  0.322  0.84  0.862  0.537  0.752  0.905  1.141  0.76  1.039  1.123  0.658  0.74  1.183  0.184  1.402  0.811  0.473  1.523  0.791  1.018  0.354  0.786  0.792  0.563  0.426  0.535  0.801  0.621  0.863  0.578  0.645  2.074  0.593  0.463  0.221  2.273  0.461  0.245  0.498  0.565  0.662  0.7  0.82  0.786  0.527  1.1  0.493  1.111  0.706  0.615  0.394  0.831  0.494  
 
 LINC01120  389043  2  132160473  132165801  2q21.1  0  0.045  0.078  0  0  0  0.061  0  0.087  0.028  0  0  0  0.081  0  0  0  0.083  0.229  0  0  2.858  0.195  0  0  0  0.035  0  0  0.03  0  0  0  0.247  0  0  0  0  0.021  0  0  0.035  0.027  0  0  0  0  0  0  0  0  0.07  0  0.325  0  0  0  0  0  0  
 
 LOC401010  401010  2  132199733  132202467  2q21.1  0.747  0.127  0.704  1.454  0.31  0.504  0.511  0.4  1.209  0.761  0.826  0.453  0.705  0.39  0.783  0.699  0.851  0.228  0.579  0.503  0.709  0.51  0.783  0.583  0.458  0.361  0.84  1.13  1.389  0.683  0.706  0.835  1.108  0.879  0.366  0.365  1.165  0.369  0.368  1.19  0.38  1.065  0.684  0.312  0.623  0.343  0.099  0.498  0.324  0.547  0.62  0.794  1.218  0.404  0.468  0.454  0.547  0.418  0.217  0.348  
 
 TUBA3D  113457  2  132233579  132240507  2q21.1  0.721  0.113  0.307  0  0.17  0.145  0.609  0.269  0.3  0.234  0.269  0.06  0.426  0.303  0.314  0.223  0.185  0.106  0.317  0.199  0.234  0.42  0.363  0.144  0.171  0.223  0.547  0.416  0.126  0.349  0.098  0.109  0.178  0.295  0.167  0.217  0.24  0.381  0.197  0.274  0.173  0.465  0.814  0  0  0.219  0.165  0.317  0.295  0.192  0.234  0.529  0.339  0.364  0.268  0.138  0.345  0.237  0.378  0.139  
 
 MZT2A  653784  2  132241532  132250064  2q21.1  5.917  3.892  4.767  5.046  5.505  5.163  6.888  5.021  5.589  4.52  4.473  5.348  4.973  5.985  4.715  5.606  6.304  3.033  7.374  7.776  4.79  6.915  6.144  5.922  5.124  3.982  6.093  3.607  5.323  4.977  5.819  5.666  4.944  4.439  5.818  4.575  4.068  4.544  5.835  4.323  5.353  6.599  7.089  3.761  4.598  3.69  7.343  6.283  3.952  7.793  4.195  4.687  5.118  4.843  7.278  4.892  4.461  6.074  5.776  4.288  
 
 LOC150776  150776  2  132250385  132279149  2q21.1  2.791  1.665  1.536  1.69  2.35  1.179  2.503  2.372  1.658  2.417  2.347  1.465  2.328  2.624  2.584  2.743  2.606  1.422  3.789  2.188  2.144  3.177  1.796  1.573  1.946  1.985  2.741  1.144  2.467  1.89  2.13  2.471  2.6  1.152  1.724  2.019  2.588  2.178  2.459  2.483  2.249  2.588  2.77  2.047  1.335  1.691  2.448  2.813  2.198  4.239  1.814  1.811  2.469  2.046  3.029  1.383  3.09  2.018  2.869  1.823  
 
 CCDC74A  90557  2  132285406  132291239  2q21.1  2.207  1.614  0.903  1.927  2.042  0.976  2.913  0.596  0.82  1.428  1.663  0.296  0  2.043  0.92  0.826  0.544  1.504  2.953  0.138  1.535  2.067  0.497  0.816  2.267  1.45  0.445  0.545  0  0.633  0  0.139  0.465  0.634  1.418  2.246  1.651  2.751  1.54  3.457  0.416  1.536  4.625  0.294  0.655  0.07  1.648  1.759  0.304  3.35  1.088  1.588  1.34  2.442  2.526  1.022  1.278  1.615  0  0.823  
 
 POTEKP  440915  2  132354346  132366974  2q21.1  1.147  0  0  0  0  0  0  0  0  0  0  0  0  0  0  0  0  0  0  0  0  0  0  0  0  0  0.093  0  0  0  0  0  0  0  0  0  0  0  0  0  0  0  0  0  0  0  0  0  0  0  0  0  0  0  0  0  0  0  0  0  
 
 LINC01087  101927994  2  132394597  132407188  2q21.1  0.295  0  0.104  0  0.029  0  0  0  0.053  0  0  0  0  0.026  0  0.057  0  0.034  0  0  0.066  0.028  0.239  0.036  0  0  0.134  0.023  0.047  0.216  0  0  0  0.339  0  0  0  0  0.036  0  0  0.204  0.118  0  0.065  0  0.094  0  0  0  0  0.07  0  0.027  0.018  0  0  0  0  0  
 
 C2orf27A  29798  2  132480063  132524977  2q21.2  1.264  0.528  0.674  0  0.107  0.369  0.47  1.026  0.884  0.439  0.578  0  0.27  0.311  0.281  0.209  0.155  0.429  0.453  0.53  0  0.666  1.158  1.018  0.625  0.234  0.627  0.224  0.213  0.097  0.228  0.534  0.438  0.552  0.297  0.466  0.826  0.642  0.26  0.137  0.238  0.75  0.043  0  0  0.127  2.275  0.487  0.196  1.492  0.485  0.265  0.317  0.421  0.907  0.196  1.299  0  0.737  0.633  
 
 C2orf27B  408029  2  132552533  132559234  2q21  0.281  0  0.338  0  0  0  0.174  0.55  0.573  0.28  0.308  0  0  0.107  0  0  0  0  0.208  0.105  0  0.276  0.949  0  0.403  0  0.236  0  0  0.099  0  0.286  0  0.455  0  0.208  0.159  0.274  0.233  0  0.085  0.482  0  0  0  0.077  0.048  0.18  0  0.857  0.147  0.436  0.071  0.3  0.327  0  0.674  0  0.17  0.262  
 
 ANKRD30BL  554226  2  132905163  133015542  2q21.2  0.192  0  0.401  0  0.304  0.233  0.227  0.146  0.471  0.592  0.57  0.574  0.389  0.486  0.39  0.519  0.485  0  0  0.162  0.703  1.532  0.79  0.429  0.443  0.312  0.836  0.57  0.386  0.856  0.086  0  0  0.579  0.573  0.152  0.326  0  0.36  0.356  0.089  0.329  0.321  0.296  0.183  0.147  0.123  0  0.222  0.079  0.513  1.179  0.493  1.641  0.447  0.778  0.268  0.112  0.125  0.279  
 
 MIR663B  100313824  2  133014538  133014653  -  0  0  0  0  4.038  3.354  0  4.551  0  2.837  3.902  3.598  0  3.269  4.14  5.465  4.311  0  0  0  6.149  2.336  0  4.288  4.811  0  0  5.122  0  0  3.814  5.017  0  0  5.315  0  0  4.094  0  5.403  4.916  0  0  6.133  4.68  4.386  0  4.305  0  0  3.862  7.121  3.774  5.236  3.866  4.536  4.487  4.977  4.08  5.174  
 
 ZNF806  646915  2  133064716  133076320  2q21.2  0  0  0.19  0  0.036  0.061  0.054  0  0.206  0.225  0.185  0.145  0  0.115  0  0.091  0.116  0.088  0  0.067  0.138  0.083  0.205  0.074  0.08  0.121  0.355  0.108  0.072  0.389  0  0  0  0.551  0.119  0.068  0.083  0  0.079  0  0  0.064  0.144  0.162  0  0.044  0.04  0  0  0  0.186  0.215  0.054  0.246  0.187  0.193  0.108  0  0  0  
 
 GPR39  2863  2  133174146  133404169  2q21-q22  0.744  0.767  1.194  0.948  1.603  0.532  2.54  1.08  2.897  1.012  2.295  2.193  0.891  0.901  1.075  1.41  0.998  0.814  0.059  0.098  0.184  0.394  0.455  0.1  2.097  0.397  0.902  0.92  0.233  0.598  0.065  0.097  0.3  0.535  1.901  2.404  2.258  2.521  0.759  1.906  2.526  0.95  0.317  1.839  0.68  0.674  1.622  1.178  2.317  2.479  1.934  1.819  2.261  1.619  2.227  1.645  2.319  1.583  1.785  1.735  
 
 LYPD1  116372  2  133402336  133429070  2q21.2  0.359  0  0.24  0  0.083  0.372  4.671  0.207  5.69  4.514  5.608  0  0  0.083  0  0.08  0  0.047  0.035  0  0  1.064  0.316  0  0.917  2.021  3.956  2.754  1.757  0.077  1.586  1.382  2.786  1.654  0.877  1.518  0.587  0.226  1.207  0  1.466  3.384  0.038  0  2.723  0.161  1.91  0  0.733  0.415  0.181  0.466  1.332  1.823  1.837  0.474  2.809  0  0.288  4.242  
 
 NCKAP5  344148  2  133429371  134326031  2q21.2  0.213  0  0.355  0  0.281  0.028  0.108  0.021  0.156  1.418  0.121  1.116  0  0.023  0  0.028  0.037  0.204  0  0.034  0  0.057  0.311  0.154  0.148  0.38  0.095  0.983  0.479  0.047  1.13  1.055  1.211  0.417  0.483  0.53  0.07  0  0.038  0.045  0.51  0.853  0.03  0.022  1.039  0.021  0.427  0  0  0  0.282  0.186  0.15  0.276  0.123  0.113  0.941  0  0  0.995  
 
 MIR7853  102466866  2  133551020  134196212  -  0  0  0  0  0  0  0  0  0  0  0  0  0  0  0  0  0  0  0  0  0  0  0  0  0  0  4.861  0  0  4.451  0  0  0  0  0  0  0  0  0  0  0  0  0  0  0  0  0  0  0  0  0  0  0  0  0  0  0  0  0  0  
 
 LOC101928161  101928161  2  134023766  134042330  2q21.2  0  0  0  0  0  0  0  0  0  0.126  0  0  0  0  0  0  0  0  0  0  0  0  0  0  0  0  0  0  0  0  0  0  0  0  0.322  0  0  0  0  0  0  0.333  0  0  0  0  0  0  0  0  0  0.518  0  0  0  0  0  0  0  0  
 
 MGAT5  4249  2  135011829  135212192  2q21.3  2.431  1.692  1.422  1.165  1.376  0.964  1.709  2.358  1.522  2.178  1.919  2.211  2.901  2.006  2.278  2.134  2.892  3.261  2.176  2.138  0.994  1.52  1.616  1.973  2.292  3.213  3.514  2.704  4.117  2.238  2.757  3.191  3.916  2.507  1.013  1.545  2.629  1.887  2.033  2.377  1.8  1.339  3.311  1.705  2.063  2.014  2.492  1.911  1.378  2.484  2.214  1.415  1.753  1.804  1.341  1.345  2.507  1.815  2.067  1.773  
 
 TMEM163  81615  2  135213329  135476571  2q21.3  1.246  0.122  0.183  0  0.075  0.161  0.477  0.155  0.481  0.115  0.474  0.202  0.146  0.118  0  0.103  0.149  5.285  0.083  0.098  0.132  0.27  0.308  0.1  0.09  3.891  3.527  0.847  1.4  0.441  0.16  0  0.165  0.153  0.088  0.107  0.105  0.106  0.136  0.087  0.232  0.323  0.133  0.241  0.299  0.207  1.208  0.179  0.345  0.498  1.176  0.808  1.127  0.236  2.118  0.107  1.838  0.069  0.756  0.909  
 
 ACMSD  130013  2  135596185  135659602  2q21.3  0  0  0  0  0  0  0  0  0  0.06  0  0.204  0  0.042  0  0  0  0  0.096  0  0  0.077  0.24  0  0  0  0.11  0  0  0.038  0  0  0  0  0  0  0  0  0  0  0  0.118  0  0  0  0  0.06  0  0  0  0.113  0.173  0.137  0  0.063  0.421  0  0  0  0  
 
 CCNT2-AS1  100129961  2  135624202  135676176  2q21.3  0.395  0.905  0.219  0  0.18  0.119  0.472  0.312  0.655  0.387  0.344  0.171  0.158  0.443  0.158  0.172  0.169  0.828  0.766  0.562  0.388  0.66  0.338  0.198  0.217  0.303  0.125  0.153  0.245  0.413  0.134  0.279  0.439  0.419  0.226  0.13  0.158  0.322  0.312  0.211  0.307  0.076  0.262  0.171  0.303  0.256  0.638  0.348  0  1.341  0.183  0  0.444  0.378  0.54  0.199  0.374  0.131  0.374  0.308  
 
 CCNT2  905  2  135676391  135716915  2q21.3  1.318  1.343  0.639  0.437  1.132  0.54  1.067  1.201  1.053  1.06  1.408  0.771  1.405  1.905  1.556  1.242  1.176  1.441  2.218  1.403  1.082  1.831  0.67  0.726  1.061  1.472  1.289  0.828  1.375  0.885  1.3  1.447  1.219  0.666  0.836  1.342  1.495  0.997  1.415  1.495  1.115  0.913  1.601  1.417  0.901  1.034  1.167  1.096  1.096  2.355  1.283  0.541  1.174  0.84  1.571  0.689  1.975  0.667  1.465  1.131  
 
 MAP3K19  80122  2  135722060  135782248  2q21.3  0.203  0.041  0.16  0  0.136  0.094  0  0.125  0.065  0.088  0.062  0  0.11  0.123  0.114  0.242  0.168  0.089  0  0.107  0  0.135  0.247  0  0  0  0.661  0.075  0.096  0.1  0  0  0  0.201  0.091  0.068  0  0  0.234  0  0  0.264  0.059  0  0  0.115  0.065  0.117  0  0.057  0.058  0.337  0.118  0.066  0.239  0  0.154  0  0.104  0  
 
 RAB3GAP1  22930  2  135809834  135928279  2q21.3  2.163  3.049  1.961  1.452  1.879  1.352  2.345  2.279  2.279  1.905  2.273  1.53  2.062  2.224  1.906  2.226  2.371  2.622  2.319  2.222  1.919  1.771  1.825  1.055  2.084  2.159  1.768  1.153  1.816  1.61  2.167  2.316  1.783  1.076  1.409  1.897  1.945  1.881  3.498  2.354  2.16  1.444  2.502  1.795  1.369  1.746  2.382  2.199  1.907  3.752  2.443  0.991  1.736  1.644  2.703  1.484  2.69  1.727  1.995  2.38  
 
 ZRANB3  84083  2  135954538  136288806  2q21.3  0.747  0.45  0.244  0.292  0.297  0.148  0.336  0.426  0.18  0.301  0.469  0.27  0.66  0.945  0.526  0.407  0.375  0.959  0.973  0.456  0.558  0.561  0.228  0.111  0.256  0.412  0.185  0.095  0.213  0.119  0.289  0.314  0.162  0.276  0.218  0.389  0.289  0.494  0.185  0.69  0.299  0.391  0.495  0.259  0.2  0.37  0.213  0.251  0.442  0.765  0.625  0.326  0.435  0.323  0.788  0.249  0.869  0.162  0.591  0.524  
 
 R3HDM1  23518  2  136289024  136482840  2q21.3  3.479  3.595  2.135  1.644  2.845  1.632  3.764  3.378  2.268  3.094  4.03  2.452  3.04  3.934  3.424  2.786  3.123  2.738  4.014  3.529  3.078  4.367  1.959  2.585  2.953  3.03  2.596  2.259  3.043  1.945  3.143  3.726  2.773  1.356  2.425  3.661  3.325  3.561  3.297  4.317  2.755  1.934  4.229  3.101  2.309  3.159  2.79  3.288  3.165  3.414  3.515  2.341  2.786  2.168  3.11  2.574  3.675  2.878  3.116  3.357  
 
 UBXN4  23190  2  136499188  136542633  2q21.3  2.593  4.478  2.032  2.216  3.175  1.878  2.953  2.867  3.449  3.082  3.721  2.65  3.069  3.243  2.896  3.034  3.349  4.477  4.625  2.934  2.449  3.18  3.108  2.354  2.57  2.908  2.22  1.234  2.389  1.41  2.914  2.955  2.408  1.828  2.068  2.87  2.433  2.449  3.855  2.811  3.105  2.666  3.239  3.42  1.487  2.403  1.888  2.545  2.926  4.367  3.237  1.724  1.856  2.529  3.33  2.359  3.733  2.572  2.29  3.473  
 
 LCT  3938  2  136545414  136594750  2q21  0  0.026  0.135  0  0.038  0.04  0.041  0.03  0.122  0.035  0.059  0.073  0  0.093  0.095  0.052  0.085  0.091  1.795  0.081  0  1.2  1.22  1.117  0.031  0.019  0.076  0.012  0  0.059  0.06  0.025  0  0.251  0.026  0.082  0.022  0.033  0.061  0.035  0.047  0.204  0.042  0.036  0.035  0.045  0.06  0  0.202  0.053  0.019  0.525  0.03  0.048  0.045  0.026  0.02  0  0  0  
 
 LOC100507600  100507600  2  136577760  136580657  -  0  0.151  0  0  0  0  0.054  0.306  0.101  0.03  0  0  0  0.035  0  0  0  0  4.654  0  0  3.088  0.178  0  0.105  0.06  0.063  0  0  0.031  0  0  0  0.232  0  0.072  0.11  0.186  0  0  0  0  0  0  0  0  0  0  0.086  0.17  0  0  0.108  0  0  0  0  0.063  0  0  
 
 MCM6  4175  2  136597195  136634047  2q21  4.318  4.577  1.874  2.425  3.487  2.128  3.379  3.604  2.32  3.498  3.427  3.404  3.713  5.014  3.543  3.735  3.658  5.042  6.659  5.058  3.812  4.909  3.286  3.397  3.157  3.187  2.987  2.144  3.282  3.185  3.168  3.634  3.483  2.376  3.222  3.568  3.128  2.325  2.906  3.992  3.412  2.819  4.951  3.802  2.204  2.904  4.244  2.903  2.999  5.35  2.94  2.471  3.025  2.598  3.578  2.66  3.307  2.974  3.909  3.357  
 
 DARS  1615  2  136663450  136743254  2q21.3  3.339  5.518  2.145  1.864  3.364  2.226  3.115  3.383  2.783  3.278  4.028  3.093  3.678  4.667  2.841  3.295  4.029  5.32  5.482  4.623  3.353  3.777  2.555  2.353  3.819  3.44  2.347  2.051  2.971  2.774  3.839  4.255  3.127  2.292  2.497  3.35  3.386  3.307  3.706  3.883  3.4  2.263  3.893  3.929  2.348  2.976  3.798  2.84  2.733  5.36  3.482  2.389  3.034  2.72  3.923  2.64  4.369  2.502  3.168  4.027  
 
 DARS-AS1  101928243  2  136742745  136765112  2q21.3  0  0  0.522  0  0.228  0  0  0.409  0.469  0.377  0.94  0.387  0.238  0.2  0  0.302  0.388  0  0.394  0.359  0.427  0.299  0.795  0.452  0.275  0.189  0.214  0  0.556  0.078  0.708  0.443  0.363  0.478  0  0.27  0  0  0.283  0  0.301  0.56  0.351  0  0.367  0.181  0.053  0.276  0  0.554  0.522  0.703  0.284  0.283  0.433  0.232  0.53  0.32  0  0  
 
 CXCR4  7852  2  136871918  136875725  2q21  0.856  0.897  0.155  0.195  0.199  0.496  0.463  0  0.274  1.553  0.421  1.113  0  0.251  0  0  0  0.589  7.839  4.08  0  6.258  3.067  3.772  0  0  0.246  0  0  2.184  0.563  0  0  0.321  0.147  0  0  0  0.439  2.599  0  0.337  0.851  2.871  0.572  0.288  0.026  1.585  0.171  2.377  0.319  0.462  0.059  3.464  0.056  0.434  2.716  0  0  0  
 
 THSD7B  80731  2  137748461  138435287  2q22.1  0  0  0.1  0  0.019  0  0.017  0.017  0.092  0.014  0.017  0.039  0  0.017  0  0.017  0  0.06  0.438  0.022  0  0.049  0.206  0  0  0  0.058  0.013  0  0.057  0  0  0  0.165  0  0  0.047  0  0.021  0  0.02  0.187  0.023  0.056  0.381  0.036  0.068  0  0  0.032  0.018  0.197  0  0.025  0.014  0  0.015  0  0  0  
 
 LOC101928273  101928273  2  138636323  138685502  2q22.1  0  0  0  0  0  0  0  0  0  0  0  0  0  0  0  0  0  0.326  0  0  0  0  0.189  0  0  0  0  0  0  0.042  0  0  0  0  0  0  0  0  0  0  0  0  0.032  0  0  0  0.032  0  0  0  0  0  0  0  0  0  0  0  0  0  
 
 HNMT  3176  2  138721807  138773934  2q22.1  0  4.26  0.468  0  0.135  0.772  1.137  0.684  1.028  1.078  2.975  1.864  1.956  0  0  1.727  2.051  0.729  0  2.318  0  0.252  0  0  0  1.612  0.054  1.709  1.47  1.446  3.132  3.157  0  0.493  0.246  1.673  1.224  2.555  0.092  0  0.389  1.466  0  0  0  0.99  1.547  0  0.468  0  0.63  0.489  0.23  1.341  0  0.786  1.731  0  0  0.429  
 
 SPOPL  339745  2  139259349  139330805  2q22.1  1.713  1.607  0.672  0.986  1.437  0.69  1.159  1.207  0.836  1.045  1.533  0.903  1.134  2.022  1.074  1.204  1.189  0.836  1.076  0.882  0.208  0.214  0.554  0.27  1.19  1.027  0.497  0.879  0.851  0.383  1.138  1.104  0.917  0.584  0.54  1.286  0.967  0.916  1.356  1.573  1.534  0.691  1.25  0.878  0.883  1.227  0.747  0.708  1.041  1.348  1.681  0.55  0.825  0.681  0.902  0.786  1.375  0.905  1.21  1.234  
 
 NXPH2  11249  2  139426726  139537811  2q22.1  0  0  0.086  0.761  0.034  0  0  0.096  0.073  0.095  0.209  0  0  0.115  0  0  0  0  0  0  0  0.071  0.206  0  0  0  0.041  0  0  0.047  0  0  0  0.2  0  0  0.386  0  0.039  0  0  0.114  0.031  0  0.232  0.121  0.03  0.47  0.142  0.3  0  0.161  0  0  0.348  0.091  0  0.274  0  0.93  
 
 YY1P2  647012  2  139654893  139656744  2q22.1  0.231  0.085  0.12  0  0.099  0  0.12  0  0.118  0.047  0.09  0.143  0  0.106  0  0.095  0.138  0  0.15  0.341  0  0.453  0.273  0  0  0  0.102  0.042  0  0.111  0  0  0  0.296  0.148  0  0  0  0.309  0  0.057  0.22  0.111  0  0  0.039  0.355  0  0  0.059  0.09  0.417  0.097  0  0.13  0.099  0  0.084  0.065  0  
 
 LRP1B  53353  2  140988995  142889270  2q21.2  0  0.004  0.077  0.094  0.015  0.017  0.011  0.025  0.602  0.365  0.439  0.016  0.044  0.06  0  0.016  0.012  0.035  0  0.019  0  0.073  0.114  0.013  0.018  0.011  0.059  0.011  0.014  0.062  0  0.009  0  0.141  0.008  0.008  0.036  0.334  0.035  0.03  0.057  0.415  0.047  0.011  0.049  0.017  0.036  0.013  0.01  0.015  0.01  0.127  0.013  0.054  0.019  0.017  0.03  0.007  0  0.011  
 
 KYNU  8942  2  143635194  143799885  2q22.2  2.867  0.077  0  1.855  3.115  0  4.404  0  0.229  2.178  0.128  0.267  0.496  0.068  0  0.46  0  0  0  0.135  0.903  0.072  0  0  3.697  0.58  2.215  2.818  1.453  2.875  2.632  1.286  3.164  2.181  4.071  0.346  3.178  0.346  0.068  0  2.864  3.555  0.054  0  0  0  0.081  0  0.265  0  0.135  1.468  0  2.324  0.07  0.214  0.093  0  0  0  
 
 ARHGAP15  55843  2  143886898  144525921  2q22.2-q22.3  0  0  0  0  0.672  0  0.169  0  0  0.302  0  0  0  0.031  0  0  0  0  5.814  4.443  0.43  4.166  2.616  1.515  0  0.083  0.326  0.363  0  0.167  0.704  0.282  0.307  0.355  0  0  0  0  0  0  0  0  0  0  0  0  0.044  0  0  0.156  0  0.182  0  0.04  0.047  0  0.09  0  0  0  
 
 LOC101928386  101928386  2  144694633  144721722  2q22.3  0  0  0  0  0  0  0  0  0.217  0.128  0  0.146  0  0.092  0  0  0  0.478  0  0  0  0.337  0.401  0  0  0  0.134  0.08  0  0.165  0  0  0  0.235  0  0  0.092  0  0.081  0  0  0.861  0.634  0  0.176  0.082  0  0  0  0  0  0.968  0  0.213  0.113  0  0  0  0  0  
 
 GTDC1  79712  2  144703580  145090101  2q22.3  0.562  2.376  1.009  1.057  1.139  0.97  0.924  1.386  1.966  1.502  1.535  0.649  0.915  1.357  0.506  1.102  0.802  1.562  1.486  1.1  0.565  1.3  0.643  0.416  1.282  1.144  0.404  0.874  1.106  0.359  1.661  1.485  1.144  0.805  0.795  1.045  1.126  1.08  1.278  1.317  1.759  0.736  1.543  1.482  0.546  1.109  0.891  0.534  1.446  2.359  1.232  0.828  0.741  0.736  1.08  0.808  1.352  0.797  0.686  1.662  
 
 ZEB2  9839  2  145141941  145277958  2q22.3  0  0.799  1.457  1.205  0.015  1.376  1.077  1.936  0.805  3.236  1.578  0.024  0  0.065  0  0.024  0  0.083  0.021  2.342  2.156  0.139  0.318  0  1.815  3.477  2.33  3.129  2.961  2.314  2.813  2.7  2.966  1.509  0.208  0  1.672  0.898  1.511  0.355  0.027  0.622  0.12  0.033  0.035  0.028  0.061  1.205  0.075  1.124  0.135  0.22  0.968  0.933  1.93  0.778  2.618  0.935  0.78  1.442  
 
 TEX41  401014  2  145425533  145834291  2q22.3  0  0.02  0.096  0  0.029  0.038  0  0.065  0  0.064  0  0.023  0  0.012  0  0  0  0.079  0  0  0  0.023  0.13  0  0  0.814  0.807  0.779  0.653  0.695  1.278  0.599  0.981  0.513  0.092  0  0.037  0  0.017  0  0  0.079  0.015  0  0  0.018  0.053  0  0  0  0.021  0.069  0.041  0.059  0  0.106  0.281  0  0  0.038  
 
 PABPC1P2  728773  2  147344624  147348558  2q22.3  0.373  0.103  0.296  0.238  0.085  0.061  0.042  0.092  0.187  0.137  0.067  0.137  0.251  0.404  0.258  0.18  0.194  0.112  0.131  0.099  0  0.2  0.274  0.125  0.208  0.07  0.224  0.13  0.103  0.374  0.085  0.087  0.038  0.308  0.091  0.064  0.256  0.099  0.186  0.256  0.056  0.126  0.197  0.055  0  0.103  0.122  0.1  0  0.27  0.19  0.267  0.214  0.126  0.116  0.185  0.148  0.076  0.09  0.047  
 
 ACVR2A  92  2  148602085  148688396  2q22.3  0.804  1.226  0.642  0.26  0.326  0.391  0.776  0.811  1.819  1.184  0.91  0.569  0.883  1.179  0.825  0.607  0.999  1.022  0.34  0.222  0.224  0.192  0.278  0.296  0.71  0.488  0.753  0.725  0.271  0.484  0.715  0.649  0.731  0.874  0.443  0.63  0.7  0.308  0.545  0.581  0.604  0.519  0.728  0.904  0.747  0.43  1.226  0.643  0.468  1.73  0.721  0.255  0.388  0.422  0.507  0.272  0.926  0.268  0.509  0.57  
 
 ORC4  5000  2  148687965  148779173  2q22-q23  1.381  2.624  0.602  0.547  1.176  0.538  1.556  0.82  1.197  0.996  1.624  0.856  1.468  2.331  0.843  1.309  1.411  2.786  2.333  1.455  1.013  1.187  0.632  0.6  1.327  1.101  0.804  1.131  0.71  0.739  1.452  1.351  1.259  0.836  1.092  1.025  1.068  0.991  1.632  1.305  1.246  1.021  2.019  1.325  1.128  1.059  1.935  0.968  1.014  2.743  1.101  0.53  0.843  0.943  1.945  0.938  1.419  0.888  1.509  1.34  
 
 MBD5  55777  2  148778579  149271044  2q23.1  1.471  1.101  0.414  0.471  0.75  0.205  1.31  1.659  1.086  1.296  1.246  0.755  0.6  1.252  0.987  0.712  0.772  0.674  1.039  0.389  0.152  0.984  0.316  0.292  0.933  0.829  0.313  0.523  0.142  0.239  0.685  0.769  0.178  0.321  0.363  1.485  1.147  1.176  1.02  1.561  1.11  0.757  1.206  1.076  0.333  0.895  0.938  0.647  0.828  0.569  1.284  0.558  0.76  1.062  1.29  0.904  1.6  0.279  1.153  0.938  
 
 EPC2  26122  2  149402559  149545136  2q23.1  1.773  2.14  0.699  0.576  1.316  0.919  1.441  1.629  1.08  1.401  1.63  0.977  1.762  1.833  1.699  1.543  1.447  1.739  1.817  1.354  1.376  1.93  0.84  1.235  1.502  1.644  1.379  2.369  1.196  1.096  1.424  1.504  1.57  0.69  1.248  1.335  1.381  1.148  1.679  2.011  1.266  0.601  2.255  1.349  1.45  1.318  1.483  1.522  1.134  2.048  1.206  1.109  0.964  1.057  1.685  0.959  1.738  0.764  1.5  1.583  
 
 KIF5C  3800  2  149632791  149883273  2q23.1  0.18  0.03  0.082  1.446  0.109  0.858  0.74  0.159  1.843  0.604  1.53  0.052  0.072  1.001  0  0.03  0.087  0.067  0.021  0.073  0  0.166  0.194  0.026  0.229  0.039  0.187  0.091  0.047  0.099  0.058  0.515  0.051  0.154  0.159  0.05  0.202  0.06  0.09  0.478  0.057  0.386  1.137  0.176  0.826  0.099  0.18  0.622  0.054  0.528  0.781  0.578  0.031  0.051  0.055  0.032  0.041  0.89  0.024  0.054  
 
 LYPD6B  130576  2  149894980  150071772  2q23.2  1.289  0  0.101  0  1.871  2.115  4.296  0.203  0.147  0.516  0.327  0.443  0.294  1.371  0.488  0.536  1.262  0  0  0  0  0.085  1.076  0  0  0  0.066  0  0  0.071  0  0.09  0  0.182  0  0.13  3.51  0  0.115  0.17  2.121  0.485  0.502  0.979  1.186  1.699  1.247  0  0.329  0.29  0  0.372  0  0.065  0  0  0  0  0  0.264  
 
 LYPD6  130574  2  150186498  150330659  2q23.2  0.854  1.531  0.159  0.319  0.373  1.362  1.6  0.654  0.435  1.192  1.869  0.455  0.812  0.961  1.422  0.052  1.226  0.146  0.017  0  0.058  0.088  0.248  0  0.071  0.436  0.332  1.062  0.445  0.057  0.838  0.301  0  0.285  0.311  0  1.235  0  0.854  0.123  0.547  0.841  0.029  0.971  0.707  1.163  1.511  0.405  0.285  0.344  0.126  0.659  0.021  0.543  0.405  0.118  0.08  0.168  0.572  1.085  
 
 MMADHC  27249  2  150426146  150444330  2q23.2  4.438  6.746  3.169  2.861  4.683  2.804  5.089  4.304  3.358  3.901  5.216  3.627  4.002  5.927  3.723  4.712  4.658  7.474  5.887  4.25  4.958  3.573  3.466  3.196  5.123  4.157  3.803  3.773  3.561  2.421  5.006  5.627  4.605  3.676  3.313  4.237  4.579  3.696  4.935  4.138  4.63  3.698  4.87  3.929  3.846  4.392  4.453  2.663  3.576  5.343  4.55  2.634  3.783  3.801  4.336  3.886  4.736  3.882  4.202  4.844  
 
 LOC101929231  101929231  2  150443871  150704748  2q23  0  0.153  0.263  0  0.199  0  0  0.213  0.244  0.171  0.311  0.165  0.302  0  0  0.269  0.368  0.173  0.18  0  0.629  0.38  0.294  0  0.261  0.134  0.332  0.417  0.202  0.128  0  0.289  0.307  0.482  0.136  0.226  0.149  0  0.271  0  0.313  0.669  0.055  0.282  0  0.552  0.704  0  0  0  0.11  0.629  0  0.406  0.3  0  0.287  0  0.25  0.158  
 
 RND3  390  2  151324706  151344209  2q23.3  0.991  5.49  4.712  4.72  1.534  2.957  4.476  3.614  2.419  2.229  3.539  1.201  1.786  2.353  1.931  2.497  1.778  3.093  0.089  0.123  0  0.216  1.058  1.598  4.403  3.025  2.65  2.746  2.703  0.077  1.896  4.963  3.327  2.993  1.947  2.306  3.205  0.993  5.329  0.505  3.572  1.864  1.135  2.665  3.158  4.21  2.925  1.986  3.078  3.713  3.622  1.728  1.848  3.114  3.069  2.657  4.38  2.99  3.229  3.776  
 
 LOC101929260  101929260  2  151409045  151428735  2q23.3  0  0  0.121  0  0  0  0  0  0.087  0  0  0  0  0  0  0  0  0  0  0  0  0.027  0.251  0  0  0  0.024  0.733  0  0.042  0  0.094  0  0.256  0  0  0  0  0.044  0  0  0  0.029  0  0  0.047  0  0  0  0  0  0.07  0  0.03  0  0  0  0  0  0  
 
 LOC101929282  101929282  2  151485410  151491871  2q23.3  0  0  0  0  0  0  0  0  0  0  0  0  0  0  0  0  0  0  0  0  0  0  0  0  0  0  0  0  0  0.08  0  0  0  0  0  0  0  0  0.137  0  0  0  0  0  0  0  0  0  0  0  0  0  0  0  0  0  0  0  0  0  
 
 RBM43  375287  2  152104727  152118389  2q23.3  0.205  0.498  0.332  0.202  0.685  0.186  0.558  0.392  0.36  0.927  0.623  0.215  0.21  0.769  0.361  0.773  0.65  0.21  0.869  0.35  0.289  0.73  0.252  0.131  0.524  0.846  0.204  0.289  0.328  0.32  0.689  0.658  0.282  0.681  0.644  0.375  0.216  0.269  0.9  0.273  0.309  0.184  0.357  0.549  0.325  0.257  0.308  0  0.482  0.282  0.692  0.179  0.377  0.734  1.097  0.47  0.826  0.266  0.394  0.717  
 
 NMI  9111  2  152126981  152146430  2q23  0.799  4.171  0.891  2.977  1.116  0.502  2.619  1.765  0.847  1.542  2.207  2.299  2.38  2.079  1.357  3.618  2.539  1.339  4.853  3.225  2.298  3.685  1.933  2.053  0.97  0.888  0.878  0.854  0.517  0.056  0.515  1.146  1.192  1.35  0.972  1.49  1.27  1.468  2.728  1.872  1.928  0.791  0.075  1.261  1.04  2.26  1.732  0.495  1.675  2.379  2.138  0.886  1.394  2.553  3.36  1.523  3.468  1.795  2.604  2.632  
 
 LOC101929319  101929319  2  152194341  152235885  2q23.3  0  0  0.14  0  0  0  0.074  0  0.13  0  0  0  0  0.033  0  0  0  0  0  0  0  0.033  0.306  0  0  0  0.069  0  0  0.039  0.071  0.06  0  0.189  0  0  0  0  0.023  0  0  0  0.021  0  0  0  0  0  0  0  0  0.144  0.032  0.035  0.038  0  0  0  0  0  
 
 TNFAIP6  7130  2  152214105  152236562  2q23.3  0  0.062  0.797  0  0  0  2.289  0.556  0  0.604  1.588  0  0  0  0  0  0  0  0  0  0  0  0  0  0.15  0.098  0.283  0.966  0.642  0.03  0.837  3.098  1.244  0.96  0.089  0  0  0  0.145  0  0  0  0  0  0  0  0  0  0  0  3.002  0.101  0  1.357  3.681  1.793  0.949  0.147  0  0  
 
 RIF1  55183  2  152266396  152333860  2q23.3  1.744  3.426  1.005  0.925  1.525  1.121  1.597  2.092  1.073  1.654  2.528  1.048  2.022  2.475  2.087  2.006  1.785  2.943  2.259  1.754  2.351  1.917  0.917  1.182  1.759  1.973  1.074  1.721  1.199  1.112  1.985  2.085  2.347  1.321  1.54  1.905  1.922  1.565  1.801  2.528  1.982  1.276  1.833  2.134  1.304  1.524  1.041  0.891  1.873  1.108  2.23  1.208  1.535  1.519  2.234  1.556  2.707  1.463  2.203  2.308  
 
 NEB  4703  2  152341852  152591001  2q22  0.827  0.064  0.05  0.017  0.257  0.018  0.019  0.03  0.054  0.062  0.095  0.121  0.062  0.111  0.166  0.019  0.036  0.169  0.017  0.037  0.29  0.075  0.139  0.029  0.019  0.03  0.055  0.054  0.023  0.113  0.111  0.118  0.044  0.139  0.073  0.052  0.061  0.058  0.063  0.057  0.034  0.084  0.152  0.229  0.018  0.039  0.05  0.027  0.16  0.008  0.048  0.194  0.041  0.049  0.059  0.049  0.042  0.025  0.067  0.031  
 
 ARL5A  26225  2  152657479  152685009  2q23.3  2.22  4.145  1.228  1.159  2.141  1.241  2.41  2.291  2.202  1.691  2.617  1.834  2.209  3.47  2.158  2.106  2.379  3.917  3.775  3.734  2.829  2.907  1.537  1.315  2.666  2.46  1.251  1.997  1.65  0.841  2.608  2.629  2.081  1.597  1.608  2.063  2.318  1.958  2.923  2.617  1.872  1.982  3.063  2.044  2.004  1.984  2.814  0.935  1.887  2.271  2.227  1.171  1.701  1.597  2.556  1.795  2.324  1.593  2.598  2.477  
 
 CACNB4  785  2  152689285  152955593  2q22-q23  0  0.134  0.393  0  0.368  0  0.016  0.052  0.111  0.14  0.123  0.031  0  0.359  0.338  0.013  0  0.065  0.057  0.203  0  0.092  0.103  0  0.027  0  0.054  0.012  0.044  0.065  0.192  0.056  0  0.133  0.027  0  0  0.156  0.09  0.054  0.032  0.177  0.075  0.05  0.132  0.037  0.04  0.055  0  0.079  0  0.187  0  0.032  0.014  0.034  0.099  0.092  0.425  0  
 
 STAM2  10254  2  152973314  153032506  2q23.3  1.054  2.338  1.28  0.835  1.432  0.669  1.807  1.841  0.958  1.36  1.432  0.8  0.831  1.467  0.9  0.918  1.173  2.045  2.123  1.656  0.663  0.889  0.525  0.439  1.513  1.689  0.919  1.257  0.743  0.668  1.398  1.462  1.304  1  1.037  1.412  1.027  1.269  2.295  1.305  1.372  0.782  1.396  0.905  0.509  1.017  1.578  0.437  1.042  1.225  1.747  0.587  1.037  1.173  1.597  0.94  1.963  0.807  1.212  1.617  
 
 FMNL2  114793  2  153191750  153506348  2q23.3  0.432  1.357  1.263  1.802  0.123  0.912  1.915  0.958  4.047  2.894  2.664  0.032  1.447  1.368  1.488  0.19  1.963  1.097  0.364  0.279  0.312  0.322  0.881  1.278  2.293  2.821  2.642  3.133  2.774  2.097  2.441  2.851  3.863  2.575  1.145  1.884  1.386  1.753  1.572  2.175  1.588  1.445  2.987  2.281  1.618  1.64  0.546  0.938  2.555  1.803  2.286  1.146  1.423  1.906  3.442  1.785  2.945  1.438  3.197  2.409  
 
 PRPF40A  55660  2  153508106  153573975  2q23.3  2.646  4.67  1.46  1.831  2.495  1.525  2.991  2.671  1.986  2.305  3.221  1.991  3.013  4.044  2.694  2.919  2.952  4.597  4.057  3.289  3.364  2.393  1.878  2.064  3.012  2.617  1.715  2.16  1.691  1.724  2.869  2.859  2.443  1.587  2.211  2.75  2.589  2.181  3.204  3.149  2.638  2.153  3.649  2.751  1.924  2.453  2.11  1.444  2.439  2.535  2.794  1.587  2.418  2.24  3.186  2.162  3.327  2.178  2.973  3.13  
 
 ARL6IP6  151188  2  153574406  153617767  2q23.3  2.467  4.965  1.403  1.46  2.069  1.529  2.835  2.271  2.119  2.386  2.865  1.503  2.101  3.672  1.915  2.561  3.013  4.309  4.707  1.857  1.731  2.449  2.204  1.127  2.704  1.952  1.651  1.324  1.02  1.448  2.098  2.316  1.664  1.683  1.42  1.701  2.632  2.11  2.38  2.262  2.052  2.087  3.191  1.5  0.995  1.17  0.894  0.858  2.04  3.433  2.34  1.521  1.916  1.392  3.212  1.723  2.771  2.255  2.278  2.56  
 
 RPRM  56475  2  154333851  154335322  2q23.3  0.293  0.098  0.276  1.17  0.189  0  0.56  0.074  0.775  0.399  3.069  0.079  0.136  0.121  0  0.209  0  0.13  0  0.106  0  0.497  0.524  0.404  0  0.099  0.104  0.068  0  0.103  0  0  0  0.364  0  0  0.064  0.411  0.221  0.368  0.161  1.729  0.064  0  0  0.08  0.065  0.103  0.135  0.17  0.074  0.883  0.096  0.179  0.287  0  0  0  0  0  
 
 GALNT13  114805  2  154728410  155310489  2q24.1  0.04  1.024  0.094  0.583  0.038  0.24  0.084  0.197  0.255  0.464  0.66  0  0  0.05  0  0.022  0.045  0.079  0.016  0.023  0  0.039  0.267  0.102  0.839  0  0.069  0.018  0  0.078  0.024  0.033  0  0.127  0.511  0.372  0.36  0  0.929  0.17  1.288  0.889  0.034  0  0.259  0.027  0.044  0  0  0.022  1.025  0.145  0.025  0.033  0.021  0.29  0  0.053  1.07  0.023  
 
 LOC100144595  100144595  2  155292364  155313950  -  0  0  0.055  0  0  0.153  0  0.057  0.089  0.026  0  0  0  0  0  0  0  0  0  0  0  0  0.201  0  0.059  0  0.023  0  0  0.032  0  0  0  0.176  0  0  0  0  0.041  0  0.054  0.159  0.022  0  0  0  0.022  0  0  0  0  0  0  0  0  0  0  0  0  0  
 
 KCNJ3  3760  2  155555092  155714864  2q24.1  0.677  0.111  0.152  0  0.097  0  0  0  0.152  0.119  0  0.128  0.292  0.046  0  0.164  3.405  0.269  0  0  0  0.427  0.501  0  0  0  0.15  0.099  0  0.127  0  0  0  0.468  0  0  0  0  0.092  0  0  0.598  0.09  0  0  0.09  0.142  0  0  0  0  1.196  0  0.154  0.078  0  0  0  0  0  
 
 LOC101929378  101929378  2  156877046  157111443  2q24.1  0  0.087  0  0  0.648  0  0  0  0.099  0.066  0  0.178  0.344  1.03  0.415  0.477  0.258  1.026  0.401  0  0  0  0.221  0  0.145  0  0.09  0.107  0  0.039  0  0  0.087  0.205  0  0.09  0.167  0.182  0  0.207  0.186  0.037  0.066  0.142  0  0  0.031  0  0  0  0.238  0.257  0.05  0.073  0.347  0  0  0  0  0  
 
 NR4A2  4929  2  157180943  157189287  2q22-q23  0.613  0.387  0.131  0.692  0.203  0.084  0.159  0.676  0.182  1.248  0.444  0.458  1.163  2.28  0.764  0.435  1.726  1.88  0.062  0.288  0.66  0.138  0.641  0.3  0.603  1.298  2.272  1.237  0.645  1.7  0.997  2.793  2.065  1.328  0.361  2.708  0.18  0.158  0.148  3.232  1.033  1.155  0.1  0  0.333  0.467  0.506  0  0.156  0.113  0.281  0.593  0.062  0.378  0.175  0.085  0.223  0.491  0.183  0.062  
 
 GPD2  2820  2  157291964  157442915  2q24.1  2.332  2.591  1.036  1.301  1.25  1.003  2.593  2.259  1.727  2.219  4.028  1.326  3.432  3.39  1.796  2.035  2.719  1.745  1.843  1.673  1.133  0.275  0.549  0.759  2.042  3.105  2.424  2.264  2.477  2.388  3.304  3.222  3.455  2.353  1.86  2.37  2.691  2.213  0.825  2.645  3.041  2.365  0.734  1.753  1.775  2.375  2.025  0.592  1.354  0.927  2.673  1.192  1.165  1.929  1.447  1.16  2.448  1.831  1.605  2.255  
 
 GALNT5  11227  2  158114339  158167913  2q24.1  0  1.169  1.653  0  0  0.104  2.463  2.908  0.23  2.672  1.234  2.335  2.698  1.065  0.998  0.569  1.241  1.776  0  0  3.256  0.069  0.204  0  0  0.151  0.116  0.271  0  0.079  0  1.797  0  0.179  0.237  0.894  0.034  1.82  0.033  0  0.338  0.502  0.036  0  0.084  0.09  0.604  0.059  0.071  0.181  0.162  0.091  0.026  0.037  0  0.767  0.192  0.737  0  1.154  
 
 ERMN  57471  2  158175124  158184225  2q24.1  0  0  0  0  0  0.048  0.034  0.069  0.107  0.497  0.026  0.056  0  0.043  0  0  0  0.049  0  0  0  0.025  0.256  0  0  0  0.022  0  0  0.023  0  0  0  0.238  0  0  0  0  0.019  0  0  0.161  0  0  0  0  0.02  0  0  0  0  0.111  0  0  0  0  0  0  0  0  
 
 CYTIP  9595  2  158271130  158300604  2q11.2  0  0  0.141  0  0  0  0  0  0  0  0  0  0.181  0.023  0  0  0.073  0  0.211  0.954  0  0.097  3.207  1.929  0  0  0.053  0  0  0.096  0  0  0  0.198  0  0  0  0  0  0.119  0  0.159  0.084  0.082  0  0  0  0  0  0  0  0.198  0  0.035  0  0  0  0  0  0  
 
 ACVR1C  130399  2  158383278  158485399  2q24.1  0.043  0.056  0.096  0  0.026  0.085  0.073  0.115  0.06  0.066  0.054  0.082  0.076  0.126  0  0.015  0.17  0.229  0.027  0.019  0.042  0.064  0.134  0.071  0.04  0.424  0.278  0.19  0.354  0.229  0.442  0.284  0.305  0.329  0.02  0.029  0.014  0.047  0.121  0.057  0.1  0.141  0.038  0.057  0  0.033  0.185  0  0  0.068  0.036  0.149  0.011  0.103  0.035  0.022  0.018  0.019  0  0  
 
 ACVR1  90  2  158592957  158732374  2q23-q24  1.712  2.662  2.49  1.828  1.178  1.273  3.161  3.543  2.892  2.5  2.618  1.032  2.032  2.324  1.352  1.769  1.772  1.107  1.667  0.209  0.807  0.352  0.731  0.652  2.681  3.017  2.804  2.742  2.628  1.092  2.649  3.224  3.053  1.994  1.088  2.202  2.413  1.951  2.778  2.276  1.46  1.223  1.673  0.759  0.896  1.207  1.708  1.278  1.277  2.659  2.703  1.133  1.916  1.703  2.834  1.856  3.029  1.852  1.885  2.499  
 
 UPP2  151531  2  158851690  158992666  2q24.1  0  0  0  0  0.036  0  0  0  0.077  0  0  0  0  0.049  0  0  0  0.096  0  0  0  0.027  0.165  0  0  0  0.041  0.034  0  0.053  0  0  0  0.134  0  0  0  0.085  0.108  0  0  0.035  0.028  0  0  0  0.02  0  0  0  0  0.153  0  0  0.047  0  0  0  0  0  
 
 CCDC148-AS1  554201  2  159023161  159092681  2q24.1  0  0  0  0  0  0  0  0  0  0  0  0  0  0.059  0  0  0  0  0  0  0  0.076  0  0  0  0  0  0  0  0.057  0  0  0  0  0  0  0  0.21  0  0  0  0  0  0  0  0  0  0  0  0  0  0  0  0  0  0  0  0  0  0  
 
 CCDC148  130940  2  159027868  159313265  2q24.1  0.545  0.916  0.256  0  0.614  0.632  0.095  0.089  0.236  0.294  0.296  0.113  0.301  0.486  0.582  0.557  0.152  0.755  0  0.11  0.307  0.112  0.23  0  0.457  0.113  0.427  0.413  0.243  0.152  0.161  0.096  0.108  0.249  0.176  0.076  0.255  0.221  2.12  0.244  0.293  0.148  0.964  0.073  0.311  0.106  0.04  0.348  0.323  0.905  0.091  0.23  0.406  0.151  0.694  0.424  0.185  0.32  0.618  0.621  
 
 PKP4  8502  2  159313391  159537940  2q24.1  3.812  4.483  2.63  1.405  3.078  2.564  2.747  3.053  1.881  2.561  2.733  1.692  3.245  3.75  3.606  3.303  3.014  3.693  3.524  3.461  3.569  2.331  1.353  1.945  2.529  1.953  2.692  3.704  2.565  1.922  2.61  2.906  3.215  1.755  2.525  2.736  3.421  3.069  4.089  3.4  3.25  1.709  3.437  3.227  2.601  3.117  3.714  3.076  3.044  3.723  2.847  1.891  3.128  2.368  3.643  2.793  3.224  3.466  3.733  3.522  
 
 DAPL1  92196  2  159651828  159672496  2q24.1  0  0  0  0  0.296  0  0  0  0.254  0  0.184  0  0  0  0  0  0  0.603  2.014  0.426  0  0.208  0  0  0  0.302  0.069  0  0  0  0  0  0  0  0  0  0  0  0  0  0.375  0  0  0  0  0  0  0  0  0  0  0  0  0.127  0  0  0  0  0  0  
 
 TANC1  85461  2  159825145  160089170  2q24.2  1.494  2.104  1.422  0.575  1.464  0.993  2.223  2.496  2.016  2.726  2.492  1.822  2.174  1.812  1.893  1.669  1.745  1.153  1.342  0.3  0.055  0.987  0.708  0.621  1.59  3.486  2.799  2.924  3.259  3.05  2.474  2.931  2.862  1.529  0.888  1.763  2.592  1.674  3.057  1.674  2.04  1.201  0.662  2.094  1.966  2.263  0.986  1.24  1.817  1.416  1.651  1.191  1.862  1.786  1.51  1.409  2.42  0.8  1.82  2.374  
 
 WDSUB1  151525  2  160092303  160143310  2q24.2  1.435  2.885  0.663  1.23  1.004  0.631  1.23  0.718  1.323  0.965  1.13  0.838  1.046  2.093  1.155  1.243  1.475  3.305  2.95  1.054  1.151  1.097  0.876  0.357  1.089  1.803  1.35  1.22  1.412  1.254  1.831  1.278  1.577  1.198  0.868  1.492  1.006  1.152  1.053  1.519  1.659  0.819  1.522  0.886  0.555  1.12  0.498  0.569  1.039  1.902  1.106  0.788  0.729  1.107  1.465  0.774  1.489  1.371  1.555  1.534  
 
 LOC643072  643072  2  160471804  160474553  2q24.2  0.36  0.217  0.209  0  0.548  0.062  0.43  0.181  0.641  0.385  0.502  0.268  0.249  0.162  0.178  0.246  0.395  0.416  0.438  0.39  0.395  0.371  0.688  0.237  0.199  0.389  0.125  0.101  0.164  0.063  0.386  0.278  0.116  0.262  0.218  0.737  0.181  0.183  0.357  0.229  0.49  0.572  0.171  0.099  0.227  0.237  0.144  0  0.219  0.41  0.229  0.548  0.063  0.345  0.531  0.131  0.36  0.094  0.146  0.235  
 
 MARCH7  64844  2  160568967  160625094  2q24.2  1.906  3.536  1.681  1.532  2.503  1.45  2.809  2.311  2.131  2.402  2.979  1.463  2.72  3.004  2.364  2.386  2.501  2.678  4.228  3.408  2.332  2.526  1.172  1.346  2.817  2.571  1.612  2.003  1.978  1.765  2.658  2.826  2.071  1.352  1.976  2.575  2.533  2.06  3.423  3.004  2.78  1.729  3.312  2.91  1.801  2.172  3.403  1.771  2.54  3.547  2.633  1.185  2.125  1.977  2.862  1.831  3.2  2.371  2.967  2.653  
 
 CD302  9936  2  160625138  160654766  2q24.2  0.829  2.716  0.884  0.557  1.77  0.486  1.111  1.878  1.723  1.785  1.094  0.901  0.268  0.562  0.377  0.933  1.716  2.072  1.186  2.776  0.27  0.177  0.488  0.077  0.196  0.904  1.536  0.183  1.048  0.909  0.852  1.182  1.769  1.658  0.628  0.887  1.302  1.349  0.259  0.943  0.398  0.511  0.936  0.822  0.608  1.078  0.957  0.38  1.547  0.72  1.288  0.439  0.184  0.216  1.398  0.873  0.513  1.521  0.666  0.333  
 
 LY75-CD302  100526664  2  160625138  160761267  2q  0  0  0  0  0.015  0.026  0  0.054  0.029  0  0  0.018  0  0  0  0  0  0  0.097  0  0  0.026  0  0  0  0  0.017  0.013  0  0.009  0  0  0  0  0  0  0  0  0.013  0  0  0.044  0.015  0  0  0  0  0  0  0  0  0.07  0  0.056  0  0  0  0  0.046  0  
 
 LY75  4065  2  160659867  160761267  2q24  0  0.07  0.05  0  0.013  0  0.273  0.096  0.029  0.035  0.031  1.214  1.196  0.022  0.048  0.871  0.842  0.303  1.322  0.748  0.028  0.038  0.97  0.418  0  0.04  0.025  0.015  0.015  0.02  0  0  0  0.105  0  0.031  0.26  0.779  0.009  0.109  0  0.023  0.012  0.056  0.514  0.516  0.103  0.151  0.742  0.428  0.02  0.031  0.015  0.987  1.437  0.053  0.037  0.026  1.651  0  
 
 PLA2R1  22925  2  160797259  160919126  2q23-q24  0.105  0.278  0.317  0.152  0.689  0.04  0.029  0.269  0.09  0.44  0.025  0.396  0.534  0.105  0.113  0.714  0  0.037  0  0.035  0  0.082  0.095  0  0  0.407  0.05  0.012  0.047  0.043  0  0  0  0.247  0.148  1.248  0.186  0.649  0.264  0.426  0.034  0.467  0.447  0.233  0.059  0.136  0.257  0.181  0.219  0.428  0.571  0.237  0.048  0.322  0.512  0.26  0.566  0.053  0.293  0.137  
 
 ITGB6  3694  2  160956176  161056824  2q24.2  0  0.033  0.075  0  0.351  0  0  0  0.107  0  0.067  0.888  1.012  0.042  0.05  1.854  1.423  0.835  0.038  0.033  0  0.043  0.214  0  0.049  0  0.079  0  0  0.071  0  0  0  0.154  0.123  1.072  0  0  0.058  0  1.828  0.127  0.04  0  1.103  2.083  0.041  0  0.974  0.083  0.065  0.249  0.646  1.073  2.988  1.499  1.772  0  0.313  1.281  
 
 LOC100505984  100505984  2  161114229  161128406  -  0  0.792  0  0  0  0  0  0  0  0  0.319  0  0  0  0  0  0.543  0.501  0  0  0  0.162  0  0  0  0  0  0  0  0  0  0  0  0  0  0.288  0  0  1.205  0  0  0  0  0  0  0  0  0  0.459  0  0  0  0  0.166  1.256  0  0  0  0  0  
 
 RBMS1  5937  2  161128661  161350318  2q24.2  1.571  4.567  2.068  1.723  1.775  1.772  3.224  3.657  2.136  3.5  3.623  0.782  2.404  2.158  0.24  1.926  1.726  3.948  4.099  2.424  0.147  2.219  1.121  1.284  4.508  3.052  2.606  2.578  1.971  2.352  3.536  4.402  3.014  1.815  1.973  3.602  3.064  2.646  4.696  2.714  3.74  2.005  2.943  3.595  2.15  3.017  3.437  2.943  2.861  4.691  3.556  1.642  2.382  1.995  3.58  2.749  3.668  2.448  3.429  2.963  
 
 TANK  10010  2  161993465  162092683  2q24-q31  2.543  4.164  1.848  2.036  2.408  1.565  3.041  2.986  2.818  2.724  2.941  2.535  2.512  4.81  1.833  4.376  3.547  4.005  3.838  2.473  1.688  2.175  2.102  1.569  2.84  2.641  1.9  1.922  1.564  1.324  1.895  2.535  1.156  1.408  2.114  2.515  2.555  2.49  3.314  2.4  2.455  2.281  3.207  1.819  1.81  2.341  1.824  1.547  1.857  3.263  2.581  1.092  2.403  2.106  3.159  2.587  3.277  1.987  2.998  2.787  
 
 LOC101929512  101929512  2  162079768  162111154  2q24.2  0  0  0  0  0  0  0  0  0  0  0  0  0  0.195  0  0.286  0.308  0.459  0.797  0  0  0.228  0  0  0  0  0.079  0  0  0  0  0  0  0  0  0  0  0  0.16  0  0.27  0  0  0  0  0  0  0  0  0  0  0  0  0  0.178  0  0  0  0.309  0  
 
 LOC100996579  100996579  2  162101249  162105561  -  0.688  0.853  0  0  0.833  0  0  0  0.08  0  0  0.184  0.405  1.529  0.667  0.575  0.272  0.675  2.617  0  0  0.802  1.226  0.916  0  0  0.04  0  0  0.03  0  0  0  0.179  0.227  0.22  0.368  0.511  0.722  0  1.251  0.346  0.026  0.206  0  0  0.293  0  0  0.467  0.366  0.346  0  0  1.31  0  0  0.094  0.59  1.017  
 
 PSMD14  10213  2  162164785  162268228  2q24.2  3.993  7.154  3.292  3.299  4.209  2.865  5.795  4.627  3.432  4.209  5.619  3.789  4.536  5.924  3.697  4.332  4.27  7.297  6.252  5.046  4.664  4.043  3.781  3.567  4.699  4.205  4.204  3.883  3.697  2.96  5.012  5.378  4.68  3.013  3.835  4.437  4.585  4.251  5.249  4.969  4.371  3.954  5.145  4.12  3.636  4.179  5.252  3.5  4.194  5.857  5.012  2.91  3.72  3.508  4.893  3.757  5.213  3.992  4.256  4.89  
 
 TBR1  10716  2  162272619  162281573  2q24  0  0.09  0.162  0  0.042  0.056  0.073  0.078  0.115  0.082  0.078  0.066  0  0.141  0.096  0.052  0.069  0.204  0  0.133  0  0.428  0.411  0.075  0  0  0.098  0  0  0.105  0  0.053  0.065  0.19  0.092  0.064  0  0.062  0.112  0  0.069  0.72  0.083  0.049  0.056  0.053  0.04  0  0  0.044  0  1.176  0.021  0.153  0.161  0.094  0.038  0  0  0  
 
 AHCTF1P1  285116  2  162355577  162364413  2q24.2  0.807  1.179  0.509  0.95  0.516  0.494  0.681  0.597  0.592  0.736  0.722  0.396  0.637  1.127  1.002  0.752  0.627  0.703  0.648  0.713  0.698  0.887  0.439  0.487  0.592  0.818  0.833  0.817  0.772  0.496  0.612  0.771  0.635  0.555  0.607  0.747  0.816  0.757  0.861  1.095  0.762  0.655  0.887  0.749  0.425  0.672  0.468  0.838  0.468  0.845  0.823  0.375  0.951  0.471  1.029  0.938  0.518  0.508  0.832  0.538  
 
 SLC4A10  57282  2  162480844  162841786  2q24.2  0.126  0  0.048  0  0.013  0  0.02  0.016  0.044  0  0.028  0  0  0.012  0  0  0  0.029  0  0  0  0.038  0.118  0.034  0  0  0.037  0  0  0.032  0.023  0  0  0.205  0  0  0.021  0.071  0.021  0  0  0.023  0.018  0.032  0.242  0.03  0.021  0  0  0  0.027  0.121  0  0.057  0.035  0  0  0  0  0  
 
 DPP4  1803  2  162848754  162931052  2q24.3  0  0.088  0.084  0.116  0  0.042  0.936  1.279  0.186  0.445  0.023  1.345  2.376  0.265  0.359  0.394  3.095  0.034  0.047  0.042  0.172  0.344  0  0  0.052  0.361  0.081  0  0  0.044  0  0.048  0  0.207  0.098  0.038  0.051  0.888  2.142  0.048  0  0.221  0.033  3.931  0.161  0.091  2.128  0  0.713  0  3.127  0.443  1.771  2.913  2.666  0.299  3.205  0.046  2.669  0.278  
 
 LOC101929532  101929532  2  162970950  163029243  2q24.2  0  0  0.375  0  0  0  0.552  0.274  0.05  0.329  0  0  0  0.012  0  0  0  0.058  0  0  0  0.046  0.117  0  0.302  0  0.271  0.028  0  0.02  0.03  0.116  0.109  0.425  0  0  0  0  0.207  0  0  0.473  0.099  0  0  0  0.018  0  0  0.058  0  0.15  0  0.021  0  0  0  0  0  0  
 
 GCG  2641  2  162999378  163008914  2q36-q37  0  0  0  0  0  0  0  0  0  0  0  0  0  0  0  0  0  0  0  0  0  0.057  0  0.108  0  0  0.026  0  0  0.049  0  0  0  0  0  0  0  0  0  0  0  0.194  3.854  0  0  0  0  0  0  0.075  0  0.147  0  0  0  0  0  0  0  0  
 
 FAP  2191  2  163027193  163100045  2q23  0  0  1.223  0.239  0  0  2.891  2.454  0  2.249  0.098  0  0  0  0  0  0  0.065  0  0  0  0.047  0  0  2.59  0.117  2.006  0.469  0  0.091  0.516  0.932  0.843  0.334  0  0  0  0  0.78  0  0  0.067  0.757  0  0  0  0.028  0  0  1.633  0  0.066  0  0.048  0  0  0.043  0  0  0  
 
 IFIH1  64135  2  163123588  163175218  2q24  0.231  1.628  0.193  2.921  0.991  0  1.557  0.773  0.665  0.387  1.232  0.673  1.698  1.025  1.229  3.815  0.819  2.15  0.748  0.988  0.075  0.774  1.739  2.251  1.668  0.299  0.935  0.551  0.389  0.196  0.463  0.469  0.708  0.878  0.153  0.217  0.286  0.649  2.168  0.22  1.518  0.207  2.656  0.052  0.209  0.285  0.878  0.144  0.232  1.33  0.463  0.466  0.376  1.928  0.749  0.321  3.546  0.665  1.391  0.752  
 
 GCA  25801  2  163200582  163219148  2q24.2  1.106  2.574  0.049  0.487  1.444  0.56  0.892  0.535  1.014  0.736  1.728  1.048  1.54  1.724  0.828  1.851  2.076  2.326  0.098  2.266  0  0.113  0.807  0.677  0.69  0.588  0.602  0.697  0.44  0.25  0.664  0.681  0.94  0.781  0.84  0.334  0.706  0.218  0.738  0.386  1.172  0.553  0.59  1.017  0.829  0.919  0.493  0.712  0.699  2.534  1.072  0.676  0.711  0.37  2.056  0.746  1.062  1.03  1.393  1.956  
 
 KCNH7  90134  2  163227916  163695257  2q24.2  0  0.051  0.085  0  0.035  0  0.05  0.044  0.049  0.024  0.045  0.036  0  0.025  0  0.091  0  0.054  0  0.033  0  0.067  0.262  0  0  0  0.106  0.03  0  0.069  0  0  0  0.185  0.043  0  0  0  0.048  0  0  0.025  0.6  0  0.775  0.05  0.062  0  0  0  0  0.283  0  0.059  0.03  0.641  0.051  0  0  0  
 
 LOC101929570  101929570  2  163625445  163654482  2q24.2  0  0  0.06  0  0.049  0  0  0  0  0  0  0  0  0  0  0  0  0.066  0  0  0  0.05  0.115  0  0  0  0.045  0  0  0.027  0  0  0  0.282  0  0  0  0  0  0  0  0.19  0  0  0.07  0.033  0.028  0  0  0  0  0.133  0  0  0  0.119  0  0  0  0  
 
 FIGN  55137  2  164464117  164592513  2q24.3  0  1.458  0.971  0.523  0.68  0.602  1.036  1.106  1.704  1.111  1.559  0.033  0.041  0.02  0.049  0.025  0.035  0.069  0.046  0.061  0.08  0.157  0.262  0.035  1.174  0.68  0.989  1.405  0.927  0.924  1.444  1.037  1.138  0.561  0.73  1.282  1.448  1.448  0.287  2.202  1.89  0.704  0.058  1.762  2.261  2.194  0.05  1.567  3.334  1.516  0.514  0.729  2.395  1.308  2.443  1.779  3.257  1.404  2.926  1.7  
 
 GRB14  2888  2  165348915  165478360  2q22-q24  0.596  0.057  0.184  0  0.374  0.126  0.088  0.1  0.092  0.807  0.203  0.406  0.943  0.996  0.453  0.898  0.308  0.215  0  0.051  0  0.138  0.291  0.244  0.688  0.217  0.12  0.491  0.294  0.283  0.41  0.162  0.465  0.203  1.126  0.411  0.055  0  0.219  0.471  0.96  0.586  0.052  0.715  0.524  0.641  1.695  0.098  0.97  0.953  0.386  0.558  1.163  0.512  1.751  0.852  2.23  0.49  2.045  0.751  
 
 COBLL1  22837  2  165536694  165698678  2q24.3  0.797  0.864  1.309  0.899  1.352  0.668  1.202  0.932  0.641  0.803  1.122  0.596  1.071  0.622  1.469  1.479  1.099  0.454  0.915  0.042  0.194  0.119  0.734  0.515  0.948  0.533  1.158  1.05  0.861  0.64  0.964  1.017  1.684  0.895  0.339  0.976  0.491  0  2.385  0.5  1.347  0.538  0.575  3.722  0.484  1.098  0.578  0.463  0.875  0.449  1.624  0.371  1.721  0.934  1.036  0.676  2.323  0.741  1.791  1.175  
 
 LOC101929633  101929633  2  165697258  165705906  2q24.3  0  0  0  0  0  0  0  0.125  0  0  0  0  0  0  0  0  0.203  0  0.238  0  0  0.198  0  0  0  0  0.212  0  0  0.203  0  0  0  0.358  0  0  0  0  0.109  0  0  0.179  0  2.593  0  0.197  0.334  0  0  0  0.352  0  0  0  0  0  0  0  0  0  
 
 SLC38A11  151258  2  165754708  165812035  2q24.3  0  0  0  0  0  0.079  0.155  0  0.076  0  0.07  0.119  0  0.037  0  0.079  0  0.079  0  0  0  0.09  0.273  0  0  0  0.413  0.041  0  0.367  0  0.175  0  0.286  0  0  0  0  0.042  0  0  0.665  0.078  0.18  0.137  0.156  0.451  0  0  0  0.072  0.178  0  0.086  0.046  0  0  0  0  0  
 
 SCN3A  6328  2  165944029  166060577  2q24  0  0.05  1.274  0.113  0.008  0.018  0.371  0.017  0.083  0.043  0.047  0.015  0  0.013  0  0  0  0.033  0.962  0.024  0  0.918  0.121  0  0.098  0  0.039  0.013  0  0.03  0  0.029  0  0.172  0.034  0  0.012  0  0.056  0.086  0  0.152  0.016  0.36  0.035  0.015  0.044  0  0.137  0  0.982  0.101  0.046  0.254  0.139  0.019  0.132  0  0.041  0.026  
 
 SCN2A  6326  2  166095911  166248820  2q24.3  0  0.014  0.161  0  0.019  0.029  0.042  0.028  0.074  0.029  0.025  0  0  0.018  0  0.011  0.029  0.031  0.682  0.035  0  0.278  0.14  0.019  0.062  0.017  0.057  0.05  0  0.043  0  0.153  0.086  0.308  0.034  0  0.021  0.029  0.044  0.335  0.017  0.089  0.024  0.906  0.028  0.018  0.405  0  0.788  0.023  0.247  0.122  0.034  0.158  0.231  0.039  0.015  0  0.043  0.028  
 
 CSRNP3  80034  2  166326156  166545917  2q24.3  0.113  0.064  0.081  0  0.066  0.037  0.015  0.099  0.217  0.009  0.199  0.024  0.079  0.021  0  0.014  0.286  0.282  0.026  0.023  0.026  0.054  0.16  0.018  0.015  0.018  0.044  0.08  0.016  0.159  0.157  0.193  0.08  0.244  0.021  0  0.055  0.026  0.02  0.028  0.014  0.243  0.012  0.023  0.887  0.27  0.032  0.048  0.031  0.113  0.345  0.109  0.016  0.24  0.31  0.034  0.02  0.04  0  0.195  
 
 GALNT3  2591  2  166604312  166650803  2q24-q31  1.647  1.311  1.575  0  1.44  0.703  0.434  0.175  0  0.087  0.042  2.473  2.597  0.477  1.57  2.545  3.083  0.583  1.136  1.045  0  0.097  0.338  0.045  0.067  1.985  0.302  0.538  0.819  1.62  2.474  0.809  0.755  1.019  0  0.076  1.013  1.42  0.149  0  2.748  0.042  0.036  0.201  2.248  2.052  0.072  0.108  0.226  0.04  1.408  0.388  0.467  0.09  0.21  0  0.656  0  0.048  3.146  
 
 LOC100506124  100506124  2  166713985  166728451  -  0.181  0.386  0.14  0  0.108  0.159  0.076  0.208  0.112  0.333  0.709  0.112  0.205  0.499  0.508  0.104  0  0.13  0.986  0.462  0.18  0.815  0.264  0  0.189  0.676  0.572  0.279  0.247  0.575  0.94  0.74  0.431  0.223  0.172  0.14  0.319  0.429  0.192  0.356  0  0.249  0.274  0.799  0.14  0.099  0.373  0.184  0.489  0.355  0.22  0.315  0.367  0.076  0.786  0.081  0.333  0.457  0.624  0.092  
 
 TTC21B  79809  2  166729871  166810348  2q24.3  0.459  1.314  0.611  0.188  0.413  0.548  0.843  0.759  0.822  0.876  1.046  0.278  0.539  1.025  0.864  0.519  0.834  1.434  2.272  0.927  0.941  0.868  0.278  0.314  0.816  0.97  0.52  0.777  0.585  0.907  1.1  1.087  1.461  0.744  0.618  0.815  0.7  0.692  0.748  1.258  0.917  0.365  1.053  0.843  0.525  0.628  0.668  0.389  0.803  1.273  1.156  0.39  0.499  0.541  1.076  0.589  1.399  0.73  0.772  0.806  
 
 TTC21B-AS1  100506134  2  166790366  166804831  2q24  0  0  0  0  0  0.282  0  0.415  0  0.159  0  0  0  0  0.262  0  0  0  0  0.227  0  0.293  0  0  0  0  0  0  0.196  0  0  0  0  0  0.292  0  0  0.254  0.165  0  0  0.18  0  0  0  0.235  0  0.293  0  0  0  0  0.211  0  0.649  0  0.302  0  0  0  
 
 LOC102724058  102724058  2  166813927  166892910  2q24.3  0  0  0  0  0  0  0  0  0  0  0  0  0  0  0  0  0  0  0  0  0  0  0  0  0  0  0  0  0  0  0  0  0  0  0  0  0  0  0  0  0  0  0  0  0  1.111  0  0  0  0  0  0  0  0  0  0  0  0  0  0  
 
 SCN1A  6323  2  166845669  167005642  2q24.3  0  0.014  0.104  0  0.013  0.04  0.017  0.026  0.07  0.021  0  0.02  0  0.023  0  0  0  0.056  0.02  0  0  0.044  0.094  0  0  0  0.199  0  0  0.049  0.039  0.024  0  0.173  0.019  0  0  0  0.03  0  0  0.085  0.016  0.024  0.033  0.069  0.052  0  0  0  0  0.181  0.017  0.026  0.027  0  0  0  0  0  
 
 LOC101929680  101929680  2  166938040  167158293  2q24.3  0  0  0.095  0  0  0  0  0  0  0.027  0  0  0  0  0  0  0  0  0  0  0  0.026  0.181  0  0  0  0.134  0  0  0.084  0  0  0  0.086  0.082  0  0.046  0  0.213  0  0.192  0.117  0  0  0  0.077  0.052  0  0  0  0  0  0.054  0.031  0.199  0  0.074  0  0  0  
 
 SCN9A  6335  2  167051696  167232497  2q24  0  0.055  0.359  0.106  0.012  1.184  0.166  0.041  0.371  0.325  0.312  0.016  0.022  0.015  0  0.009  0.144  0.033  0.202  0.016  0.722  0.145  0.46  0.546  0  0  0.039  0.029  0  0.028  0  0.049  0  0.173  0.747  0  0.31  0.71  1.07  0.092  0.434  0.802  0.275  0.039  0  0.017  0.011  0.024  0  0.062  0.129  0.195  0.959  0.044  1.645  0.017  1.231  0.859  0  1.831  
 
 SCN7A  6332  2  167260082  167343481  2q21-q23  0  0  0.05  0  0  0.028  0  0  0.024  0  0  0.014  0  0  0  0  0  0.027  0.025  0  0  0.011  0.08  0  0  0  0.037  0.026  0  0.026  0  0  0  0.158  0  0  0.018  0  0.016  0  0  0.011  0.015  0.536  0  0  0.018  0  0  0  0  0.17  0.014  0.02  0.01  0  0  0  0  0  
 
 XIRP2  129446  2  167744996  168116261  2q24.3  0  0  0.111  0  0.018  0  0.041  0  0.112  0.022  0.124  0  0  0.014  0  0  0  0.055  0  0  0  0.035  0.187  0  0.094  0  0.046  0.573  0  0.057  0  0  0  0.319  0  0  0.37  0.147  0.025  0  0  0.082  0.036  0  0  0  0.099  0  0  0  0.114  0.16  0  0.032  0  0  0  0  0  0  
 
 XIRP2-AS1  100874011  2  167979318  167997465  -  0  0  0  0  0  0  0  0  0  0  0  0  0  0  0  0  0  0.056  0  0  0  0.027  0  0  0  0  0.026  0.04  0  0.02  0  0  0  0.129  0  0  0  0  0  0  0  0.051  0  0  0  0  0  0  0  0  0  0  0  0  0  0  0  0  0  0  
 
 LOC105616981  105616981  2  168671283  168797654  -  0  0  0.188  0  0  0  0  0  0.163  0  0.205  0  0  0.177  0  0  0  0  0  0  4.213  0  0.321  0  0  0  0.241  0.386  0  0.052  0  0  0  0.282  0  0  0  0  0.055  0  0  0  0  0.316  0  0  0.458  0  0  0  0  0.313  0.296  0.213  0.498  0  0.51  0  0.614  0  
 
 B3GALT1  8708  2  168675181  168727366  2q24.3  0  0.057  0.107  0  0  0  0  0  0.158  0.379  0.297  0  0.114  0.065  0  0  0  0.067  0  0  0.726  0.081  0.233  0  1.014  0.577  0.029  0.466  0.065  0.121  0.157  0.734  0.7  0.433  0  0  0.044  0  0  0  0.056  0  0.122  2.505  0.215  0  0.195  0  0  0.119  1.22  0.145  0.054  0.157  0.099  0  0.613  0.071  0.166  0.132  
 
 STK39  27347  2  168810529  169104105  2q24.3  0.944  4.02  1.771  0.654  0.483  1.659  2.441  2.175  1.615  2.261  2.122  2.708  2.557  4.907  1.614  4.561  4.138  3.17  4.1  1.014  0  2.824  0.319  0.685  2.377  2.268  1.114  0.671  1.003  0.78  2.315  2.119  2.054  1.272  1.487  2.421  2.236  2.604  2.207  1.801  2.487  1.426  2.559  2.132  0.939  0.842  2.963  1.248  1.876  2.343  3.182  1.689  1.747  1.582  2.952  1.746  3.007  1.792  2.219  2.421  
 
 CERS6  253782  2  169312758  169631644  2q24.3  2.339  2.377  1.266  1.06  2.367  1.126  0.634  2.193  1.393  2.137  2.11  1.898  3.285  3.101  2.41  2.2  2.697  2.679  1.365  2.665  1.372  2.81  1.337  1.497  2.351  1.72  0.534  0.029  0.975  1.247  1.347  1.786  0.795  0.349  1.351  2.025  2.147  1.218  1.911  2.731  2.172  2.008  2.649  2.401  1.341  1.779  1.961  1.815  1.659  2.26  2.328  1.139  0.748  1.682  1.598  1.608  0.125  1.565  2.356  2.003  
 
 CERS6-AS1  100861402  2  169628460  169642939  2q  2.177  0  0.685  1.454  1.5  0  0.672  1.263  1.981  1.53  1.036  1.388  1.475  2.542  1.445  1.873  1.931  0  1.948  1.186  0  1.57  1.094  0.42  1.378  0.69  0.762  0  0.271  0.677  0.643  0.721  0.571  0  0.643  0.882  1.55  1.03  0.289  2.021  0.772  1.795  2.513  0  0.444  0.838  1.008  1.114  0.439  2.551  1.357  0.128  0.463  0.412  1.475  1.079  0.049  0.723  1.383  0.995  
 
 NOSTRIN  115677  2  169643048  169721849  2q31.1  0.174  0.479  0.107  0  0.346  0  0.085  0.133  0.089  0.191  0.205  0.347  0.683  0.445  0.251  0.58  1.323  0.432  0.088  0.225  1.733  0.156  0  0.116  0  0.221  0.112  0.105  0.087  0.06  0.24  0  0.251  0  0.423  1.317  0.151  0.116  0.029  0.321  0.115  0.426  0.287  0.624  0.148  0.413  4.073  0.223  0.215  0.096  0.138  0.095  0.123  0.08  0.228  0  0.248  0.115  0.195  0.091  
 
 SPC25  57405  2  169727400  169746944  2q31.1  2.833  4.95  1.417  1.314  2.832  2.307  2.721  2.618  0.464  3.217  3.083  1.809  2.103  4.042  2.039  2.606  2.887  4.635  5.08  3.282  3.419  3.526  1.162  1.529  2.649  2.078  2.804  1.535  2.281  1.454  2.997  2.49  2.979  1.771  2.283  2.019  2.306  2.346  2.971  3.075  2.786  2.284  3.451  2.388  1.355  3.016  3.016  1.764  2.592  3.759  2.472  1.173  2.04  1.659  2.564  1.858  3.033  3.232  2.878  2.291  
 
 G6PC2  57818  2  169757749  169766510  2q24.3  0  0.054  0.154  0  0.081  0  0.08  0.036  0.096  0.034  0.05  0.047  0  0.033  0  0.038  0.058  0.115  0.081  0.052  0  0.089  0.243  0.076  0.056  0.046  0.096  0.061  0  0.103  0.095  0  0  0.297  0.059  0.069  0.076  0  0.021  0  0  0.416  0.055  0.105  0  0.04  0.114  0  0.087  0.056  0.082  0.152  0.076  0.05  0.037  0.096  0  0  0.069  0.049  
 
 ABCB11  8647  2  169779448  169887833  2q24  0  0.038  0.066  0  0  0.05  0.024  0.015  0.29  0  0.066  0  0  0.017  0  0  0  0.023  0  0.042  0  0.053  0.125  0  0  0  0.049  0  0  0.068  0  0  0  0.205  0  0  0  0  0.019  0  0.034  0.02  0.028  0  0  0.022  0.034  0  0  0.016  0.326  0.04  0  0.058  0  0  0  0  0  0  
 
 DHRS9  10170  2  169923544  169952677  2q31.1  0  0  0.106  0  0  0  0  0  0.106  0  0.552  0  0  0.088  0  1.569  1.401  0.183  0  1.64  0  0.036  0  0  0  0  0.066  0  0  0.049  0  0  0  0.25  0  0.147  0  0  0.034  0.143  0.347  0.278  0  0  0.433  0  0.971  0  0  0  4.017  0.272  0.579  0  0.078  0  0.159  0  0.177  0  
 
 LRP2  4036  2  169983618  170219122  2q31.1  0.071  0.016  0.101  0.023  0.014  0  0.017  0.015  0.114  0.016  0.025  0.029  0  0.016  0.016  0.014  0.015  0.06  0.007  0.029  0  0.076  0.145  0.016  0.015  0.041  2.027  0.024  0.05  0.211  0.012  1.123  0.071  0.186  0.014  0  0.006  0  0.032  0.748  0.011  0.237  0.03  0  0.189  0.029  0.216  0  0.01  0.01  0.018  0.143  0.012  0.043  0.024  0.029  0.017  0  0  0  
 
 BBS5  129880  2  170336005  170363165  2q31.1  0.505  0.393  0.538  0.622  0.646  0.296  0.367  0.583  0.698  0.856  0.537  0.063  0.207  0.354  0.354  0.089  0  0.131  1.134  0.2  0.909  0.64  0.463  0  0.891  1.397  0.573  0.991  1.587  1.048  2.429  1.949  0.876  0.632  0.572  1.178  0.552  0.935  0.485  0.924  0.319  0.771  0.771  0.654  0.529  0.36  0.342  0  0.723  0.034  0.778  0.507  0.501  0.471  0.796  0.604  0.707  0.552  0.322  0.713  
 
 KLHL41  10324  2  170366211  170382772  2q31.1  0  0.422  0.134  0  0.09  0  0  0.117  0.143  0.14  0.045  0  0  0.134  0  0  0  0  0.15  0.138  0  0.08  0.197  0  0  0.185  0.102  0.029  0.224  0.148  0.962  0.184  0.071  0.208  0  0.105  0  0  0.04  0.121  0  0.068  0  0.067  0  0.028  0.022  0  0.136  0  0.071  0.142  0.025  0.058  0.057  0  0.111  0.082  0  0  
 
 FASTKD1  79675  2  170386261  170430431  2q31  1.483  1.132  0.525  0.713  0.559  0.321  1.132  1.071  1.112  0.96  1.744  1.208  1.861  2.039  0.945  1.041  1.707  2.535  3.347  1.688  2.206  1.332  1.133  1.091  1.391  1.679  0.846  0.846  1.738  1.391  2.214  1.924  1.505  1.219  1.054  1.879  1.032  0.967  0.751  1.91  0.685  0.966  1.345  1.299  0.965  1.624  0.15  0.649  0.958  2.341  1.393  0.599  0.765  0.754  1.597  0.554  1.577  0.589  1.402  1.37  
 
 PPIG  9360  2  170440849  170494254  2q31.1  3.413  5.235  2.016  1.914  3.278  2.303  3.545  3.317  2.47  3.004  3.654  2.789  2.671  4.346  3.605  3.525  3.527  5.573  4.41  3.565  3.818  3.707  3.005  2.858  3.325  3.222  2.327  2.527  2.786  2.153  3.506  3.754  3.373  2.82  2.748  3.613  2.661  2.685  3.541  3.976  3.456  2.534  4.523  3.448  2.395  3.25  3.231  2.824  3.101  3.589  3.188  2.1  3.065  2.101  3.627  2.746  3.921  2.854  3.418  3.553  
 
 CCDC173  129881  2  170501934  170550931  2q31.1  0  0.194  0  0.15  0.169  0.085  0.127  0.067  0.076  0.092  0.057  0  0  0.138  0  0  0.179  0.278  0.079  0.048  0.591  0.089  0  0  0.113  0  0.073  0  0  0.027  0  0.065  0  0.148  0  0.12  0  0  0.157  0.231  0  0  0.032  0.192  0.129  0.107  0.096  0  0  0.159  0  0.165  0  0.122  0.06  0  0.043  0.087  0  0  
 
 PHOSPHO2  493911  2  170550963  170558218  2q31.1  0.621  1.322  0.303  0.32  0.882  0  0.51  0.26  0.433  0.478  0.655  0.417  0.664  0.859  0.541  0.613  0.707  0.977  1.341  0.939  1.146  0.512  0.859  0.252  0.304  0.51  0.078  0.491  0.607  0.057  0.488  0.68  0.809  0.761  0.239  0.255  0.284  0.715  1.134  0.642  0.782  0.639  0.065  0.353  0.354  1.052  0.556  0.267  0.705  1.054  0.218  0.367  0.263  0.546  0.691  0.205  0.648  0.714  0.801  0.492  
 
 PHOSPHO2-KLHL23  100526832  2  170550963  170608396  2q  0  0.203  0  0  0  0  0.399  0  0  0  0.508  0.116  0.441  0.183  0  0  0  0  0.389  0.249  0  0.057  0  0.244  0  0.201  0.546  0.363  0.26  0.186  0.27  0  0.554  0  0  0  0.238  0  0.039  0.646  0.145  0.272  1.795  0  0.326  0  0.093  0.143  0  0.41  0.087  0  0.419  0  0  0  0  0  0.245  0  
 
 KLHL23  151230  2  170590355  170608396  2q31.1  1.196  2.638  0.942  0.935  0.933  0.849  1.529  1.795  1.351  0.749  1.445  1.918  1.306  2.775  2.532  1.653  2.202  2.392  3.951  2.111  2.341  2.856  0.424  0.21  1.509  0.708  1.247  0.948  1.669  2.25  0.965  1.419  1.894  1.615  1.232  1.134  1.985  0.967  1.204  1.758  1.725  0.749  3.794  1.723  1.246  2.105  3.592  1.281  1.816  2.389  1.03  0.86  1.11  0.644  2.148  1.379  1.171  1.204  2.309  2.383  
 
 SSB  6741  2  170655321  170668571  2q31.1  3.349  6.606  3.022  2.924  4.177  2.909  3.2  3.549  3.429  3.397  4.479  3.1  3.977  4.585  3.434  4.463  4.365  7.995  5.248  3.853  5.297  3.713  3.505  3.293  4.275  3.662  2.23  2.839  3.242  1.394  4.289  4.689  3.394  2.67  3.572  4.009  3.363  3.242  4.18  4.305  4.11  3.974  3.994  4.5  3.222  4.003  2.942  2.587  3.838  5.49  3.947  2.529  2.917  2.675  4.097  3.28  4.309  4.108  3.294  4.568  
 
 METTL5  29081  2  170668266  170681441  2q31.1  4.02  7.355  2.324  2.068  4.158  2.605  4.935  3.688  2.949  3.315  4.286  3.823  4.348  5.693  3.374  4.164  4.306  8.356  5.853  3.457  5.044  3.47  2.749  3.999  4.621  3.182  3.171  3.254  3.733  3.164  4.454  4.658  3.866  2.682  3.702  3.67  4.088  4.4  3.454  4.518  4.263  3.303  5.671  3.614  3.66  4.233  4.947  2.945  3.593  5.359  4.047  2.583  3.481  3.495  4.307  3.495  3.939  4.495  4.312  4.344  
 
 UBR3  130507  2  170684017  170940639  2q31.1  1.199  2.096  0.746  0.482  1.295  0.626  1.525  1.387  1.149  1.282  1.989  0.766  1.09  1.845  1.403  1.087  0.958  1.994  1.784  0.772  1.709  0.96  0.673  0.694  0.731  1.53  0.884  1.053  1.317  0.731  1.195  1.429  1.352  0.629  0.649  1.679  1.121  1.239  1.345  1.822  1.315  0.911  1.299  1.142  0.655  1.443  0.73  0.705  1.222  1.198  1.746  0.702  0.767  0.882  0.888  0.731  1.528  0.394  0.734  1.241  
 
 MYO3B  140469  2  171034654  171511674  2q31.1-q31.2  0  0  0.054  0  0.079  0.032  0.035  0  0.049  0.023  0.032  0.046  0.033  0.023  0  0.027  0  0.077  0  0.022  0.154  0.046  0.116  0  0  0.113  0.169  0.047  0  0.074  0.03  0.339  0.043  0.154  0  0  0  0  0.035  0  0.088  0.05  0.031  0.078  0.197  1.037  0.12  0  0  0  0.022  0.159  0  0.059  0.028  0  0  0  0  0  
 
 LOC101929753  101929753  2  171197026  171207453  2q31.1  0  0  0  0  0  0  0  0  0  0  0  0.077  0  0  0  0  0  0  0  0  0  0.064  0  0  0  0  0.102  0  0  0.079  0  0.212  0  0.381  0  0  0  0  0  0  0  0  0.036  0  0  0.124  0.198  0  0  0  0  0  0  0  0  0  0  0  0  0  
 
 LOC101926913  101926913  2  171556877  171627276  2q31.1  0  0.306  0  0  0  0  0  0  0  0  0.132  0.689  0  0.127  0  0  0  4.113  0  0  0  0  0  0  0  0  0.113  0  0  0.108  0  0  0  0  0  0  0  0  0  0  0  0  0  0  0  0.301  0.089  0  0  0  0  0  0  0.244  0  0  0  0.279  0  0  
 
 LINC01124  440925  2  171568948  171571077  2q31.1  0  0.179  0.222  0  0.077  0  0.081  0.065  0.194  0.096  0.167  0.305  0.872  0.182  0.712  0.303  0.167  0.305  0  0.089  0  0.261  0.621  0  0  0  0.247  0.049  0.085  0.111  0.086  0.064  0  0.29  0.057  0.276  0.156  0  0.224  0.248  0.122  0.157  0.073  0  0  0.162  0.649  0  0.193  0.173  0.087  0.826  0.071  0.308  0.45  0  0.122  0.159  0  0.072  
 
 SP5  389058  2  171571856  171574498  2q31.1  0  0.741  0.188  0  0.096  0.264  0.067  0.185  0.157  0.236  0.387  2.003  1.804  0.413  2.021  0.525  0.158  3.399  0.044  0.2  0.14  0.438  0.524  0.064  0.155  0.053  0.208  0.04  0.11  0.196  0  0.075  0.065  0.222  0.187  0.939  0.143  0  0.251  0.39  0.073  0.918  0.106  0  0.395  0.291  0.234  0.316  0.153  1.097  0.488  1.398  0.156  0.17  0.983  0  0.418  0.307  0  0  
 
 ERICH2  285141  2  171627191  171655481  2q31.1  1.058  3.39  0  0.92  0.619  0.687  0  0.744  1.982  0.626  1.091  0.788  1.215  1.257  2.214  0.558  1.355  4.248  0  0.328  1.398  0.279  0.496  0  0.215  0  0.267  0.185  0.288  0.232  0.154  1.277  0.374  0.631  0  1.358  0  0  1.271  0.913  0.716  0.802  0.108  0.359  0.721  1.315  2.98  0.296  1.251  0.873  0.815  0.712  0.182  0.371  0.8  0  0.35  0.572  0  0.275  
 
 GAD1  2571  2  171673199  171717659  2q31  1.159  1.455  0.262  0.225  0.288  1.13  1.084  0.627  1.073  1.059  3.25  1.464  1.401  1.654  1.091  1.714  1.236  3.32  0.355  0.444  0.674  0.427  0.324  0.189  1.202  0.483  1.492  1.617  1.484  1.707  0.651  3.337  0.427  0.436  0.224  1.371  0.202  0.729  1.016  0.722  1.807  1.083  0.112  0.347  0.251  1.867  1.362  0.691  1.447  2.637  1.123  0.973  0.691  2.575  1.649  0.641  1.49  1.027  1.213  0.809  
 
 GORASP2  26003  2  171784947  171823643  2q31.1  4.009  5.18  3.543  3.351  4.318  3.556  5.338  5.177  4.081  4.337  5.529  4.664  4.809  4.726  4.495  4.291  4.599  5.193  5.102  4.593  4.265  4.414  4.216  3.961  4.31  4.468  4.138  4.484  4.768  3.177  4.464  4.677  4.592  3.223  3.736  4.17  4.555  4.197  4.539  4.897  4.843  3.549  4.732  4.133  3.535  4.217  3.737  3.672  4.221  5.125  4.607  2.994  4.105  4.855  4.897  4.223  5.28  4.152  4.56  4.684  
 
 TLK1  9874  2  171847332  172087824  2q31.1  1.286  2.471  0.891  0.714  1.596  0.822  1.322  1.775  1.551  1.257  2.021  1.359  2.016  2.437  1.845  1.828  1.894  2.369  3.095  2.154  2.179  2.452  1.058  1.199  1.653  1.618  0.696  1.333  1.413  0.566  1.633  1.851  1.56  1.086  1.176  1.824  1.548  1.56  2.986  2.338  1.926  1.152  1.755  1.698  1.297  1.499  1.121  1.172  1.651  2.074  1.568  1.37  1.182  1.679  2.214  1.135  2.169  1.689  1.77  1.656  
 
 METTL8  79828  2  172173912  172291312  2q31.1  1.168  1.757  0.569  0.618  0.832  0.138  0.681  0.678  0.718  0.658  0.685  0.551  1.292  1.485  0.981  0.904  0.752  1.458  1.378  1.328  1.277  1.144  0.934  0.758  1.224  1.196  0.824  0.845  1.028  0.451  1.261  1.614  1.228  0.864  0.84  0.755  0.821  1.103  0.837  1.654  0.773  0.706  1.569  0.943  0.527  0.8  0.936  0.373  0.478  1.133  1.259  0.508  0.736  0.462  1.761  0.597  1.143  0.726  1.258  1.302  
 
 DCAF17  80067  2  172290760  172341562  2q31.1  0.522  1.081  0.56  0.426  0.582  0.274  0.752  0.495  0.623  0.679  0.717  0.378  1.293  0.81  0.898  0.596  0.58  0.652  1.754  0.913  1.27  1.078  0.575  0.459  0.814  0.892  0.517  0.889  0.497  0.379  0.836  1.001  1.054  0.589  0.475  0.914  0.806  0.814  1.293  1.156  0.878  0.472  1.648  0.438  0.505  0.621  0.303  0.591  0.583  1.171  1.047  0.482  0.627  0.595  1.228  0.679  0.939  0.659  0.683  1.281  
 
 CYBRD1  79901  2  172378756  172414643  2q31.1  0  4.414  1.669  1.801  2.739  1.39  2.572  2.511  3.099  3.477  1.575  0.038  0.343  2.024  1.989  0.082  0  1.629  2.726  2.082  0.899  0.772  0.519  0.237  0.979  3.428  2.484  1.235  1.401  1.052  2.041  1.585  0.781  1.516  1.158  0.337  1.725  2.128  3.487  1.773  1.461  1.975  2.026  0  0.755  1.621  0.88  1.329  1.015  2.874  0.137  0.835  0  0.016  2.972  1.518  1.395  2.472  0  3.684  
 
 DYNC1I2  1781  2  172543918  172606668  2q31.1  2.082  4.44  2.153  1.882  2.658  2.055  3.499  2.853  2.36  2.726  2.878  1.983  2.831  2.591  2.045  2.373  2.121  4.193  3.519  3.046  2.497  2.803  1.492  1.358  3.421  2.422  2.314  2.024  2.887  2.124  2.707  3.031  2.641  1.745  2.251  2.645  2.565  2.735  3.657  2.991  2.217  1.874  3.744  2.24  1.773  2.38  3.038  2.081  2.717  4.135  2.876  0.83  2.556  2.203  3.783  2.467  3.605  2.404  2.959  3.593  
 
 SLC25A12  8604  2  172639914  172750816  2q24  0.929  2.225  0.731  0.8  1.11  0.611  1.984  1.07  1.238  1.117  1.668  0.944  1.057  1.945  1.337  1.271  1.526  2.738  2.841  1.734  1.418  1.88  1.138  0.978  1.509  1.345  1.168  0.836  1.596  1.353  1.708  2.021  1.959  1.349  1.063  1.627  1.177  1.449  1.685  2.018  1.078  1.313  2.103  1.372  1.103  1.3  1.658  0.723  0.925  2.325  1.229  0.696  1.035  1.011  1.511  0.564  1.269  1.224  1.36  1.442  
 
 HAT1  8520  2  172778934  172848600  2q31.2-q33.1  3.531  6.457  2.623  2.372  4.089  2.524  3.921  3.962  2.733  3.485  4.145  3.407  3.405  5.109  3.049  3.908  3.904  7.201  5.734  3.195  4.135  3.056  2.683  2.52  3.756  3.427  2.649  2.869  3.424  2.306  4.021  4.339  3.745  2.756  2.674  3.523  3.434  2.991  3.737  4.542  3.452  3.263  4.393  2.945  2.482  3.417  2.909  2.571  3.004  5.21  3.548  2.166  3.174  2.815  3.624  2.458  4.392  3.84  3.648  4.1  
 
 METAP1D  254042  2  172864803  172945587  2q31.1  0.827  0.832  0.385  0  0.822  0.615  1.252  0.967  0.676  0.778  0.979  0.913  0.903  1.249  0.796  1  0.621  0.792  1.501  0.623  1.327  0.962  0.753  0.508  0.983  1.119  0.546  0.76  1.169  0.367  1.271  1.379  1.12  0.386  0.575  0.79  1.408  0.526  0.47  0.968  1.108  1.121  1.056  0.688  1.047  0.28  0.198  0.726  0.684  1.271  0.788  0.823  0.528  0.486  1.314  0.368  0.976  0.904  0.766  0.827  
 
 DLX1  1745  2  172950207  172954401  2q32  0.394  0  1.392  0.97  1.504  1.442  3.077  1.969  0.639  1.261  1.082  0.074  0.338  0.378  0.691  0.169  0.423  1.309  0.256  0.297  1.342  0.219  0.313  0.052  1.892  1.714  2.207  0.33  1.388  1.052  1.297  3.22  2.928  1.729  0.4  0.434  0.945  0  0.662  1.23  0  1.533  2.673  0.822  0.9  0.101  0.368  1.377  0.107  2.331  1.877  0.437  0.887  0.192  1.674  0.85  0.656  0.896  1.221  1.269  
 
 DLX2  1746  2  172964165  172967478  2q32  0  0.077  0.916  0.737  0.783  0.946  1.842  3.308  0.445  0.454  0.385  0.127  0.149  0.643  0.185  0.176  0.243  0.271  0.064  0.132  0.684  0.428  0.38  0.154  2.417  0.976  1.209  0.184  0.467  0.661  0.526  2.766  2.086  1.058  0.313  0.724  0.363  0.188  0.826  1.589  0.495  0.793  2.892  0.377  0.821  0.204  0.082  0.666  0.132  0.935  0.489  0.886  0.436  0.326  0.781  0.283  1.384  0.874  0.515  0.866  
 
 DLX2-AS1  104326193  2  172967733  172974710  2q31.1  0  0  0  0  0.099  0  0  0  0  0.118  0  0  0  0.09  0  0  0  0  0  0  0  0.248  0  0  0  0  0.339  0  0.129  0.346  0  0.464  0.167  0.377  0  0  0  0  0.05  0.232  0  0  1.241  0  0.258  0  0  0  0  0  0  0.572  0  0  0  0  0  0  0  0  
 
 ITGA6  3655  2  173292313  173371181  2q31.1  0.2  3.263  0.245  1.901  1.016  0.94  2.309  1.163  2.171  4.011  2.446  5.212  3.935  3.155  2.278  3.835  5.141  2.993  2.486  0.965  0.062  0.81  0.186  0  4.108  3.656  3.189  4.261  4.033  1.73  2.428  4.344  3.922  2.849  1.524  2.892  2.199  3.136  3.039  2.295  3.193  1.854  1.684  1.99  0.88  0.401  4.517  2.725  3.226  3.397  4.41  2.042  1.468  0.825  1.657  1.424  2.065  5.05  1.977  1.448  
 
 PDK1  5163  2  173420100  173490351  2q31.1  0.832  2.381  1.957  1.127  1.172  1.24  0.822  2.048  1.786  1.652  2.361  0.917  0.971  1.216  1.274  1.057  2.518  2.511  2.524  2.089  0.956  1.226  1.693  1.434  1.655  0.998  2.338  1.253  2.707  1.717  1.544  2.078  2.432  1.307  0.694  0.881  0.981  1.405  1.511  1.114  1.327  1.743  0.757  1.476  0.65  1.873  1.084  0.398  1.234  0.968  1.994  1.005  1.154  0.369  1.898  0.993  3.39  1.134  0.631  1.483  
 
 RAPGEF4-AS1  91149  2  173587917  173600934  2q31.1  0  0.026  0.125  0  0  0  0.024  0.028  0.074  0.051  0.026  0  0  0.021  0  0  0.032  0.055  0.014  0  0  0.056  0.231  0  0  0  0.058  0  0  0.054  0  0  0  0.226  0  0  0  0.046  0.028  0  0  0.171  0.017  0.027  0  0.02  0.025  0  0.064  0  0.031  0.113  0  0.029  0.019  0  0.092  0  0  0  
 
 RAPGEF4  11069  2  173600524  173917620  2q31-q32  0.185  0.091  0.091  0  0.058  0  0.045  0.062  0.801  0.067  0.241  0.031  0.114  0.068  0  0.031  0.035  1.338  0.167  0.159  0  0.126  0.199  0.044  0.048  0  0.394  0.021  0.139  0.115  0.276  0.915  0.068  0.2  0  0.053  0.094  0.052  0.132  0.177  0.142  0.495  0.096  0.047  0  0.052  0.066  0  0.121  0.027  0.101  0.227  0.032  0.067  0.043  0  0.152  0  0  0  
 
 ZAK  51776  2  173940564  174132737  2q24.2  1.677  4.708  3.22  2.712  1.859  2.223  3.607  2.98  1.924  3.131  2.707  2.388  2.676  3.249  2.525  2.364  3.011  3.354  3.504  2.55  2.057  2  0.381  0.317  3.441  1.923  2.224  0.135  2.165  1.607  2.325  2.819  2.767  2.228  2.239  3.325  3.556  2.104  4.043  4.079  2.574  1.938  2.571  1.705  1.17  2.189  3.621  2.48  2.502  3.786  3.17  2.107  2.223  2.382  3.523  2.17  3.55  2.571  2.551  3.202  
 
 MLK7-AS1  339751  2  174062440  174146764  2q31.1  0.292  0.536  0.599  0.473  0.138  0.308  0.416  0.244  0.306  0.223  0.265  0.171  0.473  0.488  0.417  0.31  0.381  0.466  0.166  0.413  0  0.364  0.174  0.084  0.792  0.205  0.433  0.021  0.484  0.266  0.255  0.237  0.183  0.493  0.341  0.198  0.578  0.197  0.734  0.598  0.237  0.274  0.517  0.078  0  0.162  0.968  0.27  0.125  0.258  0.357  0.502  0.435  0.418  0.788  0.269  0.483  0.338  0.347  0.208  
 
 CDCA7  83879  2  174219560  174233718  2q31  1.981  3.547  0.586  2.402  0.03  1.696  2.224  2.388  1.922  1.798  2.485  3.027  2.707  3.642  2.722  3.282  4.64  4.419  6.111  4.498  3.351  4.754  1.308  2.163  2.899  2.054  2.076  1.162  2.535  2.04  2.525  2.652  3.407  2.773  2.813  2.632  2.479  2.315  2.096  3.697  1.957  2.869  3.547  3.733  1.002  2.271  3.184  2.437  2.81  4.725  2.037  1.989  2.588  2.154  3.733  2.351  3.273  2.422  3.302  2.819  
 
 SP3  6670  2  174771186  174830430  2q31  2.232  3.304  1.334  1.444  1.674  1.372  1.89  2.341  2.653  2.171  2.846  1.834  2.41  2.721  2.496  2.4  2.362  2.759  3.557  1.868  1.941  2.48  1.034  1.27  2.197  2.305  1.363  2.074  2.072  1.261  1.881  2.159  2.309  1.311  1.719  2.521  2.259  2.239  2.175  3.311  2.422  1.624  2.382  2.315  1.795  1.982  1.457  1.769  2.418  2.662  2.456  1.328  2.085  2.013  2.36  1.826  3.121  2.188  2.915  2.508  
 
 OLA1  29789  2  174937174  175113365  2q31.1  2.719  5.452  2.049  1.765  2.908  2.564  3.578  2.811  3.117  2.79  4.008  2.654  3.324  4.541  2.927  3.28  3.731  5.957  4.771  4.308  3.375  3.552  2.085  2.035  3.241  2.658  2.298  2.459  3.262  1.407  3.337  3.408  2.744  1.995  2.763  2.999  3.045  2.965  4.49  3.289  2.95  2.74  3.757  2.924  2.429  3.03  3.861  2.206  2.903  4.502  3.234  1.766  2.535  2.326  3.505  2.534  3.788  2.97  3.668  3.635  
 
 LINC01305  285084  2  175190754  175195370  2q31.1  0  0  0.121  0  0  0.121  0  0.088  0.12  0.033  0.028  0  0  0.024  0  0.05  0.042  0.103  0  0.032  0  0.141  0.264  0  0  0  0.098  0.03  0  0.072  0  0  0  0.287  0.087  0  0  0  0.049  0.074  0  0.412  0.025  0  0.098  0.075  0.064  0  0  0  0.029  0.363  0  0.104  0.047  0.074  0.351  0.041  0  0  
 
 SP9  100131390  2  175199820  175202268  2q31.1  0.167  0.162  0.166  0.293  0.146  0.431  0.208  0.149  0.708  0.184  0.282  0.18  0.154  0.116  0.41  0.198  0.156  0.598  0.107  0.308  0  0.928  0.513  0.077  0.171  0.062  0.349  0.058  0.245  0.253  0.099  0  0  0.323  0.588  0.215  0  0.129  0.304  0.278  0.186  0.854  0.161  0.236  0.194  0.153  1.343  0.121  0.152  0.066  0.101  1.644  0.19  0.375  0.295  0.128  0.171  0.132  0.087  0  
 
 CIR1  9541  2  175212877  175260443  2q31.1  2.15  4.052  1.39  1.716  2.576  1.218  2.897  2.487  2.235  2.311  2.39  1.94  1.247  2.866  1.849  2.557  2.485  4.422  3.748  2.404  1.94  2.957  2.568  1.679  2.289  2.276  1.658  1.668  1.935  0.885  2.133  2.458  2.067  2.223  1.741  1.767  1.538  1.931  3.463  1.832  2.084  1.774  2.652  1.365  1.405  1.878  1.129  1.365  1.637  3.283  2.117  0.877  1.533  1.689  2.889  1.578  2.626  1.95  2.479  2.352  
 
 SCRN3  79634  2  175260456  175294303  2q31.1  0.75  1.734  0.552  0.466  1.474  0.843  1.299  1.069  1.434  1.299  1.445  0.729  1.307  0.89  0.971  0.768  1.378  2.381  2.211  1.445  0.873  1.224  0.808  0.377  0.816  1.41  0.941  1.008  1.249  0.657  1.445  1.323  0.942  0.88  0.796  1.122  0.909  1.178  1.354  0.998  1.387  0.713  1.556  0.644  0.603  0.665  0.651  0.401  0.67  1.427  1.646  0.631  0.783  0.819  1.748  0.879  1.522  0.977  1.325  1.406  
 
 GPR155  151556  2  175296299  175351816  2q31.1  0.214  0.421  0.533  0.104  0.151  0.101  0.437  0.571  1.926  1.167  0.372  0.026  0.1  0.54  0.285  0.052  0.406  1.821  0.22  0.133  0.04  0.374  0.368  0.233  0.193  1.394  0.982  0.616  0.479  0.111  0.22  0.72  0.659  0.759  0.22  0.413  0.219  0.412  0.228  0.312  0.181  0.274  0.504  0.111  0.139  0.37  0.143  0.168  0.244  0.359  0.278  0.334  0.144  0.261  0.27  0.07  0.414  0.199  0.162  0.101  
 
 WIPF1  7456  2  175424301  175547627  2q31.1  0.259  2.032  2.23  0.148  0.05  1.319  2.346  1.861  3.308  2.723  1.904  0.052  0.042  0.029  0.032  0.046  0.044  2.061  3.832  3.053  0.353  3.553  3.023  2.369  3.117  3.63  3.147  3.154  4.309  0.51  3.011  3.326  3.402  2.067  1.393  2.183  1.98  1.547  0.307  0.491  0.12  1.038  0.784  0.146  0.077  1.049  0.068  1.49  1.664  1.836  0.113  0.829  1.49  0.318  2.048  1.132  2.21  0.957  0.037  1.267  
 
 CHRNA1  1134  2  175612322  175629200  2q31.1  0  0.172  0.123  0  0  0.22  0  0.324  1.389  0.077  0.109  0  0  0  0  0  0  0.756  0  0.053  0  0.122  0.212  0  0  0.538  0.076  0.121  0  0.098  0.307  0.249  0.253  0.291  0.058  0  1.181  0.196  0.085  0  0.202  0.348  0.029  0  0.249  0.257  0.077  0.143  0  0  0  0.482  0.046  0.1  0.116  0.152  0.105  0.269  0  0  
 
 CHN1  1123  2  175664041  175870107  2q31.1  1.687  1.976  1.224  1.319  0.324  0.964  2.394  2.073  2.233  3.288  3.034  0.347  0  0.996  0.957  0.091  0.659  3.886  1.964  0.183  0.14  2.594  0.333  0.286  1.21  0.807  0.376  0.387  0.273  0.92  1.103  2.192  2.938  1.973  0.913  1.286  2.04  0.909  1.639  1.556  0.528  2.003  2.67  0.074  1.315  0.326  0.898  0.966  0.702  3.051  1.821  0.908  1.054  0.588  2.282  0.342  2.822  0.667  2.386  1.274  
 
 ATF2  1386  2  175936977  176032934  2q32  1.874  2.799  1.592  1.331  1.607  0.901  1.93  2.284  1.987  2.064  2.259  1.485  1.833  2.239  2.059  1.793  2.156  2.416  3.294  2.307  2.107  2.37  1.404  1.481  2.329  2.111  2.077  1.96  2.144  1.176  2.102  2.164  1.886  1.142  1.545  1.755  2.349  2.363  2.233  2.321  1.975  1.142  2.947  2.147  1.525  1.988  1.76  1.951  2.182  2.73  2.128  1.02  1.755  2.122  2.47  1.693  3.05  1.836  2.698  2.622  
 
 ATP5G3  518  2  176040985  176046490  2q31.1  5.091  6.938  3.383  3.628  5.217  3.766  5.748  4.824  4.59  3.596  5.283  5.466  5.181  6.289  4.364  4.873  6.077  6.698  7.129  6.307  6.224  6.064  4.27  4.993  5.285  4.777  4.871  3.856  5.405  5.297  6.125  6.35  5.357  4.667  4.903  4.216  4.259  4.377  4.971  4.756  4.608  4.979  5.666  5.033  4.743  5.085  4.881  4.002  4.277  6.105  4.562  2.902  4.554  4.244  5.235  4.153  4.861  4.738  5.085  4.7  
 
 KIAA1715  80856  2  176788619  176867567  2q31  1.029  2.137  1.781  0.804  1.563  0.78  1.855  1.855  1.871  1.884  1.876  0.872  1.487  1.514  1.443  1.067  1.856  1.1  2.345  1.767  1.276  1.524  1.002  0.582  1.311  1.621  1.356  0.99  1.779  1.603  1.871  1.928  1.969  1.346  0.694  1.135  1.308  1.724  1.59  1.343  1.546  0.998  1.414  1.549  1.048  1.042  0.974  0.859  1.361  1.559  1.965  0.818  0.939  0.967  1.123  0.933  2.457  1.194  1.472  1.415  
 
 EVX2  344191  2  176944834  176948690  2q31.1  0.22  0  0.254  0  0.159  0.173  0.072  0.157  0.138  0.257  0.134  0.316  0  0.17  0.133  0.259  0.226  0.141  0.092  0.177  0.152  0.614  0.386  0.158  0.197  0.088  0.32  0.17  0.127  0.257  0.135  0.172  0  0.239  0.246  0.126  0.069  0.121  0.38  0  0  0.995  0.124  0.114  0  0.18  0.13  0.091  0.141  0.135  0.094  1.472  0.102  0.367  0.389  0.249  0.083  0  0  0.1  
 
 HOXD13  3239  2  176957531  176960666  2q31.1  0  0  0.236  0  0.068  0.246  1.805  1.004  0.585  0.43  0.051  0.048  0.106  0.074  0.507  0.068  0  0.265  0.067  0.557  0  1.311  0.103  0.053  2.031  0  1.248  0.205  1.148  0.832  1.453  2.788  3.553  2.405  0.127  0.454  0.608  0  0.1  0  0.06  0.72  0.065  0.123  0.451  0.068  0.202  0.809  0.531  1.854  0.078  0.862  0  0.545  0.276  0.074  1.301  0.657  0  0  
 
 HOXD12  3238  2  176964529  176965488  2q31.1  0  0.153  0  0  0.141  0  0  0  0.353  0.11  0.172  0  0  0  0  0  0  0.172  0  0  0  0.444  0.312  0  0  0  0.211  0.102  0  0.182  0  0  0  0.269  0  0  0  0  0.155  0  0  0.475  0.306  0  0  0  0.069  0  0  0  0  0.803  0  0.151  0.099  0  0.161  0  0  0  
 
 HOXD11  3237  2  176972083  176974316  2q31.1  0  0  0.13  0  0.181  0.283  0.881  2.087  3.564  2.168  2.841  0.147  0  0.168  0.493  0.164  0.181  0.292  0  0.133  0  0.606  0.28  0.09  0  0  0.215  0.134  0.196  0.207  0.184  0  0  0.254  0.202  1.286  0.409  0  0.174  0.711  0.091  1.384  0.097  0.516  0.153  0.147  0.055  0.127  0  0.592  0.072  1.251  1.931  1.861  1.867  0.203  2.683  0.721  0  0.16  
 
 HOXD10  3236  2  176981491  176984670  2q31.1  0  0  0.157  0  0  0.302  1.394  2.016  2.86  2.494  2.992  0.127  0  0  0.78  0  0  0  0  0.084  0  0.186  0.221  0  0  0  0.09  0.097  0  0.27  0  0  0.08  0.46  0.166  1.455  0.666  0  0.096  0.815  0  1.412  0  0.149  0  0  0.036  0.099  0  0.473  0  0.415  2.393  2.148  1.823  0  3.275  0.758  0  0.132  
 
 HOXD9  3235  2  176987412  176989645  2q31.1  0  0.073  0.333  0.256  0.131  0.201  0.49  1.144  1.761  1.698  1.557  0.266  0.115  0.945  0.878  0.284  1.691  0.463  0.154  0.43  0  0.666  0.58  0  0.948  0.137  0.5  0.303  0.25  0.205  1.502  0.21  0.799  0.845  0.203  0.886  0.187  0.115  0.499  0.722  0.144  1.102  0.926  2.198  0.165  0.154  0.11  0.45  0.9  1.351  0.066  1.323  1.537  1.293  1.727  0.856  2.519  0.141  0  0.12  
 
 HOXD8  3234  2  176994421  176997423  2q31.1  0  0.048  0.128  0  0.085  0.208  0.16  0.871  2.296  0.568  1.515  0.171  0.141  1.06  0.207  0.063  1.864  0.496  0.049  0.125  0.123  0.313  0.196  0.061  1.48  1.22  0.877  0.661  1.144  0.89  1.408  1.625  1.454  1.547  0.519  0.696  0.293  0  0.258  0.285  0.502  1.588  0.438  2.055  0.264  0.079  0.146  0.529  1.052  2.015  0.322  0.854  0.831  1.058  1.813  0.758  0.93  0.835  0  0.309  
 
 HOXD-AS2  100506783  2  176999568  177001826  2q31.1  0  0  0  0  0  1.282  0.991  1.132  1.207  1.368  0.863  0  0  0.804  0.332  0.186  1.907  0.243  0  0  0  0.344  0  0  0  0.998  0.458  0.599  1.335  0.968  2.349  1.915  1.397  1.028  0.719  1.102  0.365  0  1.1  0  0  1.415  1.237  1.514  1.249  0  0.085  0  1.155  0.623  0.735  1.316  1.222  1.5  2.991  1.467  1.154  1.851  0  0.901  
 
 HOXD4  3233  2  177016112  177017949  2q31.1  0  0.262  0  0  0  0.542  0.443  0.418  0.779  1.394  0.969  0  0  0.189  0.822  0.125  0.418  0.308  0  0.15  0  0.254  0.357  0  1.442  0.927  0.451  0.168  1.701  1.797  2.451  1.776  0.322  0.287  0.224  0.324  0.889  0.411  0.366  0  0  0.501  0.652  1.886  0.737  0.094  0.052  1.282  0.369  1.723  0.254  0.811  1.343  1.419  1.858  1.147  1.558  0.658  0  0.134  
 
 HOXD3  3232  2  177028804  177037826  2q31.1  0  0.335  0.217  0  0.104  0.225  0.184  0.318  0.827  0.744  0.465  0.096  0.105  0.207  0.474  0.141  0.384  0.101  0.08  0.161  0  0.426  0.483  0.094  0.585  0.287  0.251  0.106  0.771  0.925  0.773  0.502  0.149  0.382  0.127  0.165  0.666  0.129  0.285  0.154  0.056  0.354  0.105  0.901  0.199  0.086  0.071  0.309  0.385  0.539  0.138  0.908  0.43  0.81  0.647  0.403  0.883  0  0.083  0.066  
 
 HAGLR  401022  2  177037916  177053686  2q31.2  0.183  1.268  0.506  0  0.083  0.646  0.386  1.269  0.51  1.995  0.869  0.068  0.073  0.078  0.661  0.048  1.297  0.117  0.089  0.093  0  0.29  0.32  0  0.405  1.311  1.529  0.708  2.526  2.542  1.952  1.683  1.525  1.017  0.31  0  0.638  0.088  0.083  0.384  0.255  0.127  0.532  0.492  0.99  0.048  0.104  0.797  0.72  1.559  0.103  0.838  0.815  1.339  0.827  0.73  1.603  0.59  0  0  
 
 HAGLROS  102800310  2  177042893  177043737  2q31.2  0.391  0.203  0  0  0  0  0.246  0.518  0  0.194  0.134  0  0  0  0  0.172  1.591  0.422  0.742  0.654  0  1.792  0  0  0  0  0.164  0.795  0.584  0.939  0.495  0  0  0.281  0  0  0.601  0  0.173  0  0.42  0.814  0.139  0  1.497  0.193  0  0.486  0.803  1.711  0  1.042  0  1.126  1.908  0  0.743  0  0  0  
 
 HOXD1  3231  2  177053306  177055635  2q31.1  0.084  0.829  0.294  0  0.087  0.139  0.082  0.567  0.115  0.408  0.262  0.19  0  0.157  0  0.108  1.207  0.258  0  0.157  0  0.402  0.191  0  0.139  0.143  0.358  0.128  0.358  1.962  0.169  0.352  0.134  0.285  0.195  0  0.093  0  0.219  0  0.074  0.716  0.849  0.25  0.255  0.083  0.12  0.273  0.755  0.935  0.119  1.148  0.174  0.432  0.267  0.282  0.384  0.198  0  0  
 
 MTX2  10651  2  177134122  177202753  2q31.1  3.314  5.448  2.199  1.592  2.942  1.844  4.047  3.298  2.462  2.906  3.427  2.638  3.126  4.739  2.64  3.073  3.937  4.808  4.657  3.073  3.769  2.9  1.984  2.223  3.24  3.575  2.477  3.048  3.516  3.148  4.298  4.301  3.561  2.463  2.509  3.176  3.056  3.277  3.828  3.249  3.383  2.129  4.219  3.298  3.219  2.635  4.215  2.204  2.764  4.846  3.213  1.939  2.786  2.821  4.037  2.477  3.528  3.161  3.656  3.507  
 
 LINC01116  375295  2  177494308  177502302  2q31.1  0.569  4.156  2.708  0  1.564  1.883  3.975  2.277  1.735  1.75  2.448  0.158  0  0  0  0  0.57  0.89  0  0  0  0.135  0.305  0  3.28  0.188  0.894  1.125  1.987  1.118  2.488  2.736  1.97  1.733  0.658  0  1.099  0  0.326  1.589  0.73  1.799  0.699  0.806  0.906  0  0  1.211  2.043  3.402  3.377  0  2.47  3.538  3.336  2.684  3.601  2.083  0  2.207  
 
 LINC01117  102724224  2  177502481  177520686  2q31.1  0  1.14  0.586  0  0.283  0.878  0.502  0.405  0.507  0.363  0.175  0.315  0  0  0  0  0  0  0  0  0  0  0  0  0.999  0  0.227  0.404  0.741  0.229  0.807  1.159  0.686  0.718  0  0  0  0  0.084  0  0.463  0.25  0.129  0  0  0  0  0.337  0  0.903  0.647  0.784  0.732  1.386  1.412  0.256  0.757  0.389  0  0.664  
 
 HNRNPA3  220988  2  178077421  178088685  2q31.2  3.035  5.095  2.531  2.746  3.761  3.69  4.127  3.884  3.309  3.672  4.159  3.641  3.479  4.479  4.012  3.994  4.316  5.297  6.522  5.499  4.143  4.756  2.72  3.437  4.434  3.748  2.829  3.155  4.008  2.678  4.494  3.741  3.962  3.096  3.98  3.442  3.619  3.387  4.432  3.986  3.706  3.822  3.974  4.032  3.685  2.911  3.893  3.058  3.848  5.389  3.931  2.317  3.656  3.41  4.307  3.619  4.181  4.696  4.751  4.048  
 
 NFE2L2  4780  2  178095030  178129859  2q31  3.29  5.72  3.184  3.452  3.167  2.015  4.236  3.965  4.183  4.07  3.883  3.803  3.292  4.807  3.311  3.682  4.136  4.446  4.45  2.912  3.401  2.327  2.658  1.938  3.723  4.532  3.655  3.633  4.249  3.422  4.204  4.733  4.345  3.672  3.012  3.979  3.571  3.585  4.61  4.829  3.952  3.157  4.023  3.466  3.247  3.043  2.298  2.956  3.535  4.517  3.423  2.65  3.452  3.776  4.531  3.383  4.394  3.45  4.399  4.066  
 
 LOC100130691  100130691  2  178148235  178257419  2q31.2  0  0.11  0.124  0  0.016  0.04  0.21  0.109  0.187  0.111  0.111  0.558  0.097  0.254  0.08  0.072  0.04  0.143  0.211  0.13  0.066  0.157  0.312  0.073  0.074  0.264  0.044  0.141  0.033  0.043  0.108  0.048  0  0.237  0.101  0.133  0.23  0.212  0.13  0.192  0.052  0.08  0.061  0.026  0.052  0.055  0.05  0.079  0.09  0.07  0.079  0.29  0.185  0.075  0.347  0.078  0.131  0.186  0.254  0.106  
 
 AGPS  8540  2  178257470  178408564  2q31.2  0.028  2.647  1.077  0.688  0.013  0.863  2.461  2.024  1.944  1.592  3.474  1.611  1.457  2.395  1.598  1.598  1.352  2.639  3.338  1.809  1.833  1.799  1.004  0.931  1.944  1.902  0.767  1.458  1.79  1.009  2.413  1.964  1.438  0.961  1.312  2.039  1.947  1.677  1.783  1.878  1.579  1.721  2.264  1.432  1.489  1.186  1.752  1.221  2.416  2.918  2.173  0.922  1.701  1.566  2.111  1.306  2.493  1.533  2.367  2.727  
 
 TTC30B  150737  2  178414880  178417524  2q31.2  1.408  0.571  0.404  0.594  0.896  0  0.451  0.697  0.907  0.489  0.592  0.755  0.868  0.224  0.647  0.738  0.484  0.495  0.991  0.321  0.342  0.459  0.453  0.178  0.559  0.564  0.805  0.956  0.898  0.41  0.405  0.851  0.799  0.737  0.712  0.971  0.348  0.427  0.379  1.118  0.77  0.742  0.85  0.45  0.861  0.215  0.422  0.34  0.706  0.699  0.417  0.385  0.424  0.608  0.93  0.396  0.886  0.21  0.798  0.415  
 
 TTC30A  92104  2  178479025  178483694  2q31.2  1.014  0.708  0.411  0.371  0.939  0  0.507  0.829  0.966  0.69  0.861  0.695  0.471  0.345  0.435  0.44  0.334  0.817  0.97  0.437  0.291  0.545  0.367  0.044  0.42  0.513  0.5  1.022  0.872  0.481  0.356  0.853  0.606  0.577  0.587  0.9  0.267  0.316  0.406  0.945  0.674  0.56  0.936  0.389  0.806  0.184  0.456  0.384  0.719  0.656  0.347  0.632  0.447  0.763  0.969  0.424  1.039  0.583  0.622  0.45  
 
 PDE11A  50940  2  178487976  178973066  2q31.2  0  0.168  0.078  0  0.085  0  0  0.023  0.091  0.034  0.047  0.056  0.119  0.149  0  0  0.032  0.082  0  0.035  0.352  0.08  0.238  0  0.061  0.053  0.091  0.037  0.076  0.184  0.04  0.042  0  0.311  0.149  0  0.088  0.064  0.035  0  0.035  0.159  0.037  0  0  0.023  0.159  0.051  0.228  0.083  0  0.173  0.036  0.286  1.122  0.039  0.138  0  0.459  0.198  
 
 RBM45  129831  2  178977150  178994382  2q31.2  1.695  2.714  0.762  0.913  1.742  0.628  1.712  1.073  1.368  1.143  1.632  0.799  1.383  2.352  0.99  1.166  1.208  3.25  2.819  1.801  1.064  1.844  1.449  0.898  1.004  1.273  1.17  1.12  1.35  0.876  1.805  1.603  1.167  0.844  0.84  1.468  1.191  0.984  1.328  1.529  1.312  1.139  1.714  1.061  0.918  0.852  1.497  0.626  0.885  2.455  1.3  0.698  0.973  0.809  1.35  0.785  1.286  1.111  1.37  1.505  
 
 OSBPL6  114880  2  179059207  179264160  2q32.1  0.437  1.962  0.846  0.959  0.273  0.473  0.214  1.05  1.504  0.966  1.567  0.037  0.056  1.251  0.51  0  0  0.556  0.03  0.026  0.846  0.127  0.548  0.182  0.757  1.292  0.94  0.494  0.774  0.425  0.616  1.247  1.013  0.776  0.13  0.033  0.177  0.976  0.841  1.049  0.172  0.85  0.939  0.454  0.148  0.309  0.071  0.652  0.115  1.597  1.507  0.435  0.721  0.415  0.685  0.066  1.857  0.785  0.837  0.46  
 
 LOC101927027  101927027  2  179278385  179304970  2q31.2  0.572  1.406  0.784  0.928  1.491  0.77  1.177  1.502  0.822  0.887  1.503  0  0.18  1.649  0.256  0.175  0.108  2.232  3.045  1.647  1.184  2.307  1.757  1.675  1.714  1.566  1.411  1.139  1.626  0.507  1.579  1.926  1.655  0.961  0.821  0.923  1.245  2.055  0.761  1.327  1.018  0.857  1.222  1.114  0.566  0.857  0.579  0.586  0.916  1.292  1.435  0.713  0.968  1.67  1.866  0.612  2.79  1.242  1.918  1.714  
 
 PRKRA  8575  2  179296140  179315958  2q31.2  2.46  4.861  2.089  1.788  3.04  2.242  2.529  2.667  2.74  2.547  3.353  1.543  2.471  3.877  2.057  2.322  3.077  4.679  4.676  2.587  2.592  2.892  2.111  1.747  2.275  3.104  1.894  2.362  2.182  1.2  3.038  2.788  2.576  1.9  2.12  2.767  2.361  2.111  2.791  2.826  2.69  1.941  3.379  2.502  2.271  2.53  2.7  1.364  2.893  3.493  2.836  1.539  2.006  1.626  3.044  2.04  3.486  2.886  3.332  2.583  
 
 DFNB59  494513  2  179316162  179326110  2q31.2  0  0.483  0.105  0  0.293  0.074  0.193  0.297  0.239  0.177  0.188  0  0.248  0.467  0.094  0.124  0.434  0.988  1.531  0.076  0  0.581  0.383  0  0.13  0.287  0.301  0.169  0.227  0.053  0.22  0.221  0.141  0.351  0.239  0.444  0  0.119  0.214  0.169  0.15  0.103  1.146  0.253  0  0.147  0.558  0  0  0.645  0.118  0.254  0.079  0.17  0.719  0  0.586  0  0.099  0.217  
 
 FKBP7  51661  2  179328390  179343355  2q31.2  0.123  2.624  1.003  0.697  0.155  0.485  2.178  2.183  0.74  2.063  1.009  0.101  0.256  0.971  0.152  0.105  0.605  2.607  1.527  0.781  0.323  0.983  0.508  0.452  0.673  1.789  0.727  0.813  1.31  0.275  1.132  1.26  0.986  1.182  0.602  0.302  0.551  1.218  0.1  0.346  0.065  0.851  0.196  0.224  0.311  0.31  0.045  0  0.42  0.553  0.514  0.194  0.59  0.54  1.019  0.918  1.752  0.493  0.106  1.287  
 
 PLEKHA3  65977  2  179345198  179369782  2q31.2  1.317  3.242  1.4  1.045  1.397  1.233  2.471  2.427  1.894  1.832  2.647  0.82  1.624  2.318  1.381  1.316  1.741  2.157  2.531  1.532  1.1  1.798  1.243  0.762  2.052  2.127  1.283  2.059  1.799  1.114  1.974  2.347  1.733  1.415  1.261  1.902  2.146  1.731  2.383  1.877  1.377  1.51  2.053  1.343  1.668  1.214  0.968  0.82  1.756  1.925  2.057  1.121  1.623  1.006  1.768  1.368  2.165  1.817  2.119  1.965  
 
 TTN-AS1  100506866  2  179387553  179484944  -  0.333  0.48  0.473  0.144  0.647  0.233  0.563  0.866  0.336  0.951  0.044  0.297  0.849  0.666  0  0.05  0.051  1.427  0.828  0  1.032  1.199  0.418  0.29  0.704  0.04  0.376  0.568  1.007  0.554  0.418  0.803  0.915  0.477  0.575  0.258  0.603  1.004  0.214  0.842  0.648  0.657  0.276  0.615  0.664  0.5  0.314  0.602  0.951  0.889  0.624  0.342  0.072  0.052  0.868  0  1.631  0.537  0  0  
 
 TTN  7273  2  179390716  179672150  2q31  0.113  0.119  0.19  0.037  0.158  0.049  0.157  0.233  0.122  0.287  0.036  0.067  0.27  0.196  0.02  0.025  0.031  0.202  0.119  0.036  0.184  0.403  0.48  0.23  0.086  0.02  0.172  0.162  0.94  0.111  0.086  0.204  0.221  0.368  0.084  0.074  0.144  0.378  0.085  0.119  0.165  0.323  0.219  0.395  0.163  0.103  0.08  0.218  0.193  0.103  0.514  0.24  0.056  0.051  0.216  0.032  0.515  0.064  0.027  0.012  
 
 LOC101927055  101927055  2  179641652  179644690  -  0  0  0.116  0  0  0  0  0  0  0  0  0  0  0  0  0  0  0  0.04  0  0  0.046  0.145  0  0  0  0.023  0  0.091  0.02  0  0  0  0.276  0  0  0  0  0  0  0  0  0  0  0  0  0  0  0  0  0  0  0  0  0  0  0  0  0  0  
 
 CCDC141  285025  2  179694483  179914786  2q31.2  0  0.009  0.067  0  0.007  0  0.015  0.012  0.092  0.016  0  0.012  0  0.028  0  0.011  0  0.027  0.105  0.018  0  0.032  0.351  0.217  0  0.014  0.05  0  0.715  0.03  0  0.037  0  0.247  0.016  0  0  0.019  0.015  0  0  0.213  0.017  0.26  0  0.022  0.022  0  0  0.013  0.483  0.104  0.022  0.028  0.01  0.017  0  0  0  0  
 
 SESTD1  91404  2  179966418  180129350  2q31.2  0.559  1.431  0.716  0.653  0.226  0.361  0.886  1.314  1.247  1.2  1.841  0.632  0.652  1.341  0.207  0.599  1.05  1.214  1.98  0.706  0.131  0.943  0.343  0.272  0.823  1.26  1.032  0.479  0.724  0.864  1.52  1.03  1.766  1.093  0.55  0.601  0.829  0.495  0.802  0.33  1.747  1.039  1.009  2.128  0.528  1.046  0.838  0.208  1.518  0.475  1.081  0.319  0.609  0.438  0.926  0.915  1.037  0.471  0.721  0.769  
 
 ZNF385B  151126  2  180306710  180726232  2q31.2-q31.3  0.696  0.232  0.117  0.317  0.05  0.329  0  0.094  0.175  0.045  0.242  0  0  2.136  0  0.05  0  0.317  0.039  0  0  0.114  1.139  0.546  0  0.102  0.139  0.35  0  0.095  0  0  0  0.301  0  0.123  0.118  0.098  0.037  0  0.127  0.302  0.07  0.109  0.853  0.049  0.064  0.174  0.343  0.374  0.228  0.582  0.039  0.313  0.067  0.079  0  0.098  0  0  
 
 CWC22  57703  2  180809603  180871780  2q31.3  1.267  3.235  0.924  1.024  1.062  0.77  1.507  1.585  1.384  1.311  1.892  1.216  1.885  2.464  1.511  1.5  1.768  3.692  2.859  1.537  2.003  1.477  1.226  0.936  1.907  1.572  0.8  1.44  1.556  0.858  1.724  1.781  1.888  1.315  1.136  1.49  1.529  1.359  1.751  1.884  1.638  1.272  1.821  1.586  1.472  0.817  1.492  1.217  1.14  2.451  1.715  0.875  1.116  1.09  1.713  1.124  1.974  1.377  1.64  1.72  
 
 LINC00913  101669767  2  181556830  181781666  2q31.3  0  0  0  0  0  0  0.138  0  0  0  0  0  0  0  0  0  0  0  0  0  0.341  0  0.286  0  0  0  0.094  0  0  0.105  0  0  0  0.321  0  0  0  0.221  0.068  0  0.902  0.304  0  0  0.389  0  0.147  0  1.213  0  1.388  0  0  0.071  0  0  0  0  0  0  
 
 UBE2E3  10477  2  181845111  181928154  2q32.1  3.534  7.053  2.843  2.323  2.994  2.821  6.205  4.338  4.126  4.194  5.3  2.822  3.7  5.492  2.945  3.808  3.775  6.494  6.582  4.475  4  4.22  3.399  3.128  4.597  3.668  3.708  3.257  3.545  2.682  4.116  4.509  3.427  2.73  2.924  4.102  4.617  4.519  4.47  3.707  4.577  3.443  5.065  3.882  3.172  3.239  6.409  3.366  4.362  5.923  4.665  2.675  3.792  3.054  4.116  3.374  4.774  4.131  4.627  4.256  
 
 LOC101927156  101927156  2  181988563  182264280  2q31.3  0  0.131  0.27  0  0.039  0.27  0.078  0.068  0.383  0  0.059  0.106  0  0.063  0  0  0  0.253  2.349  0  0  1.198  0.277  0.11  0.111  0.063  0.08  0.038  0  0.19  0.193  0.438  0  0.447  0  0  0  0.155  0.089  0  0.083  0.619  0.05  0  0.147  0.205  0.531  0.11  0  0  0  0  0  0  0  0  0  0.085  0  0  
 
 ITGA4  3676  2  182321618  182402468  2q31.3  0  0.056  0.544  0.443  0.018  0.223  1.809  1.932  0.278  2.815  2.244  0.063  0  0.037  0  0.015  0  0.053  5.648  2.546  0  4.368  0.518  0.249  2.948  1.373  0.458  1.561  1.433  0.259  0.63  2.838  0.784  0.193  0.018  0  0.47  0  0.54  0.079  0.024  0.411  0.014  0.25  0.041  0.018  0.043  0.572  0.058  1.449  0.031  0.184  0.062  0.83  1.611  1.091  0.033  0.774  0  0  
 
 CERKL  375298  2  182401400  182521834  2q31.3  0.145  0.289  0.29  0.368  0  0.079  1.718  0.667  0.202  1.446  0.648  0.059  0  0.129  0  0  0  0  4.786  2.987  0  3.731  0.725  0  1.451  0.461  0.267  0.271  0.484  0.058  0.176  1.065  0.244  0.237  0  0  0.127  0  0.604  0  0  0.162  0.024  0.134  0  0  0.09  0.147  0  0.933  0.054  0.284  0  0.239  1.244  0.291  0  0.761  0.245  0  
 
 NEUROD1  4760  2  182540832  182545392  2q32  0  0.049  0.073  0  0.024  0  0  0.026  0.135  0.029  0.021  0  0  0.036  0  0  0  0.097  0  0.044  0  0.145  0.197  0  0  0  0.111  0  0  0.395  0  0  0  0.281  0  0  0  0  0.061  0  0  0.411  0.044  0  0  0.034  0.042  0  0  0  0  0.309  0.022  0.022  0.037  0  0  0  0  0  
 
 SSFA2  6744  2  182756442  182795464  2q31.3  1.624  3.777  1.726  1.338  1.67  1.787  3.507  2.268  3.174  3.283  3.418  2.08  3.409  6.344  2.75  3.7  3.912  4.463  3.787  1.836  2.037  2.106  1.435  1.231  3.237  2.757  1.987  2.821  2.996  1.665  3.008  3.797  3.324  2.443  2.019  2.701  3.158  2.487  2.55  2.524  3.681  1.655  0.305  2.717  1.38  1.486  3.098  1.907  2.532  3.179  3.395  1.664  1.929  1.713  2.305  2.338  2.549  2.245  2.629  3.324  
 
 PPP1R1C  151242  2  182818967  182996109  2q31.3  0.275  1.37  0  0  0.188  0  0.521  0  0.181  0.666  0  0.779  0.242  2.93  0.261  0.357  0.509  3.8  3.484  0.106  0  1.806  0.392  0.19  2.574  0  0.314  0.855  0  0.082  0  0.922  0  0.391  0.674  0.369  0.648  1.168  0.328  0  1.137  0.146  0  0  0  0.18  1.163  0  0  0  0.422  0.756  0  0.594  0.258  0.241  0  1.451  1.264  0  
 
 PDE1A  5136  2  183004761  183387572  2q32.1  0  0.062  0.121  0  0  0  0.058  0.097  0.132  0.116  0  0.076  0  0.217  0  0  0  0  0  0  0  0.024  0.176  0.071  0  0  0.12  0.032  0  0.162  0  0  0  0.297  0  0.487  0  0  0.065  0.102  0  1.327  0.039  0  0.138  0  0.138  0  0  0  0  0.141  0  0.112  0.034  0  0.077  0  0  0  
 
 DNAJC10  54431  2  183580767  183644750  2q32.1  1.912  3.459  1.94  1.719  2.063  1.048  2.909  2.294  2.386  2.319  2.647  1.364  2.426  2.988  1.855  2.235  2.783  3.081  3.106  1.865  1.753  1.499  2.174  2.127  2.526  2.564  1.62  2.088  2.441  1.446  2.473  2.763  2.248  1.338  1.633  2.966  2.74  2.806  2.395  2.685  2.44  1.722  2.591  2.056  1.341  1.492  1.707  1.35  1.954  3.177  2.837  1.438  1.928  2.254  3.08  2.389  3.548  2.215  3.272  3.401  
 
 FRZB  2487  2  183698004  183731498  2qter  0  0.061  0.093  0  0.023  0.053  0.062  0.023  0.075  0.055  1.087  0.042  0  0.543  0.06  0.034  0  0.177  0.613  0  0  0.068  0.218  0  0  3.948  0.054  0.08  0.047  0.037  0.104  0.11  0  0.229  0  0.145  4.537  0.345  0.118  0.129  0  0.143  0.26  0.05  0.148  0.043  0.019  0  0  0  0.068  0.417  0  0.064  0.081  0  0.065  0.168  0  0  
 
 NCKAP1  10787  2  183789578  183903586  2q32  2.951  5.437  2.838  1.79  3.015  2.127  3.718  3.628  2.951  3.288  4.225  2.383  3.906  4.264  3.484  3.45  3.984  3.894  2.926  0.095  2.717  0.511  0.187  0.039  3.523  3.865  2.803  3.152  3.876  2.745  3.795  4.008  3.336  1.995  2.556  4.278  3.667  3.605  4.172  4.023  3.892  2.231  3.852  3.242  2.633  2.934  2.74  2.485  3.682  4.519  3.537  2.336  2.826  2.916  3.755  2.997  3.901  2.917  4.005  4.13  
 
 DUSP19  142679  2  183943286  183964722  2q32.1  0.15  0.041  0.213  0.082  0.172  0.101  0.215  0.135  0.149  0.162  0.177  0.038  0.294  0.177  0.092  0.051  0.133  0.1  0.538  0.122  0.238  0.211  0.158  0  0.309  0.554  0.117  0.393  0.384  0.137  0.517  0.332  0.359  0.39  0.107  0.204  0.231  0.105  0.149  0.531  0.507  0.177  0.391  0.145  0.045  0.153  0.034  0.098  0.186  0.266  0.217  0.201  0.052  0.233  0.239  0.214  0.142  0.105  0.055  0.435  
 
 NUP35  129401  2  183982217  184026412  2q32.1  2.262  4.698  1.31  1.443  2.21  1.008  2.816  1.849  1.118  1.776  2.084  1.829  2.348  3.561  1.626  1.98  2.109  4.714  4.308  2.241  2.293  1.958  1.223  1.259  2.262  2.011  1.68  2.245  2.632  1.012  2.655  2.593  2.814  2.019  2.081  2.697  2.211  1.956  2.325  2.702  2.384  1.285  2.888  2.408  1.41  1.54  1.641  1.504  1.929  3.391  2.385  1.297  1.67  1.158  2.674  1.319  2.487  1.574  2.773  2.397  
 
 ZNF804A  91752  2  185463092  185804214  2q32.1  0  0.229  1.424  0  0.017  1.114  1.565  0.15  0.07  0.17  0.959  0  0  0.439  0  0  0.042  0.039  0.024  0.894  0.057  0.095  0.174  0.521  1.937  0.114  0.057  0.135  0.107  0.1  0  0  0  0.249  0.022  0  0.857  0  0.091  0.041  0  0.258  0.019  0  0.045  0.027  0.032  0.554  0  1.178  1.376  0.233  0  0.078  0.039  0.043  0  0.148  0  0.056  
 
 LOC101927196  101927196  2  186584600  186605204  2q32.1  0  0  0  0  0  0  0  0  0  0  0  0  0  0  0  0  0  0.512  0  0  0  0.147  0  0  0  0  0.056  0  0  0.064  0  0  0  0.276  0  0  0  0  0  0  0  0.184  0  0  0  0  0  0  0  0  0  0.483  0  0  0.088  0  0  0.318  0  0  
 
 FSIP2  401024  2  186603354  186698016  2q32.1  0.077  0.033  0.061  0  0.103  0.009  0.017  0.025  0.047  0.013  0.037  0.007  0.084  0.05  0.156  0.066  0.195  0.068  0.008  0.033  0.02  0.043  0.113  0.021  0.047  0.025  0.048  0.053  0.016  0.059  0.016  0.011  0.007  0.168  0.006  0.05  0.036  0.04  0.018  0.016  0.119  0.081  0.066  0.009  0.008  0.013  0.044  0  0.053  0.006  0.084  0.131  0.006  0.038  0.029  0.014  0.006  0.045  0.027  0  
 
 LINC01473  101927217  2  186898260  186951044  2q32.1  0  0  0  0  0  0  0  0  0  0  0  0  0  0  0  0  0  0.363  0.175  0  0  0  0  0  0  0  0  0.116  0  0  0  0  0  0  0  0  0  0  0  0  0  0  0  0  0  0  0  0  0  0  0  0  0  0  0  0  0  0  0  0  
 
 ZC3H15  55854  2  187350884  187374087  2q32.1  3.608  5.737  2.587  2.641  3.661  2.853  4.373  3.577  3.221  3.223  4.269  3.185  4.057  4.855  3.503  3.918  4.21  6.156  5.326  4.508  4.285  4.109  3.447  2.838  4.218  3.592  2.67  3.657  3.772  2.662  4.145  4.439  3.677  2.894  3.287  3.77  3.908  3.139  4.392  4.038  3.711  3.409  6.341  4.096  3.394  2.995  3.714  3.048  3.249  4.26  3.575  2.287  3.637  3.082  4.285  3.245  3.527  3.447  4.62  4.093  
 
 ITGAV  3685  2  187454789  187545629  2q31-q32  0.885  2.86  2.342  1.25  1.721  1.133  2.695  3.45  2.663  3.135  3.3  0.566  1.321  2.066  1.692  1.87  1.6  1.289  1.089  0.787  0.657  0.6  0.629  0.305  2.055  2.57  1.349  2.015  2.356  0.95  2.166  2.203  2.04  1.446  1.418  2.583  3.082  2.685  2.773  3.338  2.083  1.494  4.436  2.845  1.979  2.649  1.901  1.58  2.433  2.743  2.014  1.009  2.605  3.075  2.385  2.392  3.542  1.271  4.384  4.134  
 
 FAM171B  165215  2  187558788  187628512  2q32.1  0.517  1.272  0.647  0.203  0.286  1.211  0.963  1.033  1.134  0.308  1.112  0  0.347  1.544  2.483  0.034  0.614  0  0.032  0.058  0  0.217  0.245  0.23  0.81  1.077  0.431  1.167  1.187  0.465  0.237  0.797  0.811  0.612  0.513  0.592  1.091  0.968  0.368  1.214  1.427  0.528  4.54  2.037  0.612  0.32  0.147  0.336  0.219  0.209  0.625  0.657  0.391  0.785  0.963  0.358  0.288  0.676  0.855  0.612  
 
 ZSWIM2  151112  2  187692206  187713897  2q32.1  0  0  0  0  0  0  0  0  0.099  0  0  0  0  0  0  0  0  0  0  0  0  0.032  0  0  0  0  0.03  0  0  0.027  0  0  0  0.177  0  0  0  0  0  0  0  0.406  0  0  0  0  0.034  0  0  0.043  0  0  0  0  0  0  0  0  0  0  
 
 CALCRL  10203  2  188206689  188313021  2q32.1  0  0  0.075  0  0  0  0.07  0.045  0.758  0.022  0.288  0  0  0.154  0  0  0  0.05  0.08  0.025  0  0.017  0.917  0.334  0.063  0  0.028  0.033  0  0.036  0  0  0  0.156  0  0  0  0  0.013  0.184  0  0.172  0.087  1.461  0.031  0.03  0.101  0  0  0.057  0.027  0.035  0  0.224  0.321  0.159  0.383  0  0  0.045  
 
 TFPI  7035  2  188328957  188419219  2q32  0.147  4.556  2.881  0.937  0.045  0.839  3.891  1.67  1.438  1.199  1.268  1.505  0  2.617  0.989  3.795  3.265  0.376  3.68  0.045  3.011  0.145  0.122  0  3.422  0  0.137  1.561  0.32  0.578  0  0.878  1.922  1.407  1.757  3.182  3.784  3.682  0.219  3.832  0.646  2.616  0.305  1.983  0  0.071  3.564  0  2.383  0.83  3.853  2.418  3.035  3.966  4.538  3.284  3.077  4.152  2.911  3.987  
 
 LINC01090  104355152  2  188900322  189152418  2q32.1  0  0  0  0  0  0  0  0  0  0  0  0  0  0  0  0  0  0  0  0  0.945  0  0  0  0  0  0.131  0.413  0  0.094  0  0  0  0  0  0  0  0  0  0  0  0  0  0  0  0  0  0  0  0  0  0  0  0  0  0  0  0  0  0  
 
 GULP1  51454  2  189156395  189460652  2q32.3-q33  0.1  2.763  1.042  0.683  0.85  1.097  1.183  1.1  1.002  2.068  1.18  0.929  2.171  2.586  0.277  1.087  1.806  1.031  0.034  0.069  0.58  0.199  1.303  1.106  1.164  2.126  1.365  2.505  1.627  0.801  1.409  3.289  2.21  2.04  1.143  2.181  1.351  0.705  1.143  1.451  2.772  1.652  1.285  0.501  0.604  0.914  1.807  0.318  2.182  2.259  2.391  0.849  1.471  1.602  2.803  1.559  2.544  1.037  3.11  1.572  
 
 DIRC1  116093  2  189598464  189654831  2q33  0  0  0.145  0  0  0  0  0.046  0  0  0  0  0  0  0  0  0  0  0  0  0  0  0.275  0  0  0  0.035  0  0  0.036  0  0  0  0.394  0  0  0  0  0.041  0  0  0.287  0  0  0  0  0  0  0  0  0  0  0  0  0  0  0  0  0  0  
 
 COL3A1  1281  2  189839098  189877472  2q31  0.046  0.03  6.255  2.005  0  0.104  0.028  8.481  0.094  7.306  0  0.021  0  0.01  0  0  0  0.028  0  0.031  0  0.23  0.096  0.027  0.1  0  0.036  0.065  0  0.034  0  0.044  0  0.134  0  1.973  0.078  0  0.237  0.619  0  0.416  3.132  0  0  0  0.022  0  0  0.041  0.06  0.146  0.033  0.028  0  0.029  0.023  0.688  0  0  
 
 COL5A2  1290  2  189896640  190044605  2q14-q32  0.13  0.215  3.988  3.475  0.365  1.772  2.472  6.064  1.538  5.379  2.717  0.019  0.035  0.203  0.153  0.022  0.021  0.074  0.231  0  0  0.154  0.109  0.175  0.169  1.7  0.365  0.482  0.156  0.231  1.26  2.994  1.837  0.603  0.827  3.436  0.279  1.634  0.16  2.482  0.19  4.41  5.917  0.133  0.92  0.156  0.053  0.036  0.026  0.111  2.59  0.256  0.041  0.075  0.112  2.461  2.996  0.952  0.095  0.598  
 
 WDR75  84128  2  190306158  190340288  2q32.2  2.254  4.289  1.312  1.597  2.736  1.668  2.788  2.366  1.754  2.063  2.395  1.624  3.15  3.722  2.144  2.546  3.03  4.426  4.133  2.656  2.758  2.452  1.874  1.625  3.24  2.486  1.599  2.197  2.354  0.945  2.614  3.46  2.664  1.646  2.065  2.261  2.714  2.402  2.948  3.677  2.259  2.092  4.436  2.656  1.991  1.696  1.733  1.81  1.806  3.889  2.885  1.569  2.018  1.955  2.913  1.998  3.011  2.573  3.364  2.986  
 
 SLC40A1  30061  2  190425315  190445537  2q32  0  1.087  0.074  0  5.597  0.158  0.043  0.138  0.953  0.149  0.739  2.586  0.649  0.059  0.124  2.442  1.661  0.966  4.377  0.549  1.807  2.016  0.682  0.114  0  0  0.045  0  0.094  0.131  0.083  0.888  0  0.226  0.568  1.228  0.187  0  0.078  0.245  0.542  0.631  1.126  0  0.228  0.411  0.168  0  0  0.08  0.038  0.128  0  0.063  1.652  0.8  0.039  0.394  0  0.674  
 
 ASNSD1  54529  2  190526124  190535557  2p24.3-q21.3  2.469  4.25  1.822  1.964  2.987  1.668  2.841  2.131  2.644  2.461  2.276  1.677  2.243  3.175  1.845  2.192  2.291  3.45  3.933  2.264  1.601  2.641  2.335  1.408  2.126  2.533  1.63  1.455  2.207  1.234  2.585  2.894  1.953  1.795  1.635  1.97  2.201  2.086  3.359  2.51  1.733  2.426  3.436  2.395  1.549  1.721  1.065  1.425  1.693  3.666  2.583  1.252  1.559  1.68  2.43  1.679  2.232  1.58  2.96  2.227  
 
 ANKAR  150709  2  190540710  190611376  2q32.2  0.172  0.551  0.103  0.12  0.232  0.039  0.223  0.219  0.162  0.11  0.115  0.077  0.126  0.129  0.194  0.226  0  0.405  0.207  0.159  0.063  0.113  0.09  0.129  0.163  0.095  0.096  0.076  0.122  0.107  0.154  0.155  0.039  0.157  0.068  0.246  0.142  0.198  0.13  0.084  0.229  0.183  0.375  0.031  0.051  0.095  0.384  0.091  0.063  0.162  0.065  0.232  0.062  0.141  0.312  0.119  0.192  0.073  0.594  0.075  
 
 OSGEPL1  64172  2  190611385  190627924  2q32.2  1.14  2.176  0.783  0.718  1.422  0.562  1.562  0.733  0.894  1.007  0.869  0.759  0.753  1.752  0.87  0.677  1.061  1.858  2.224  1.012  0.421  1.073  0.589  0.541  0.704  1.579  0.864  1.08  1.539  1.149  1.75  1.472  1.149  1.384  0.755  0.944  1.032  1.111  1.203  1.418  1.122  0.715  1.894  1.42  0.369  0.439  1.025  0.317  0.711  1.334  1.191  0.546  0.492  0.485  1.296  0.64  1.089  0.895  0.99  0.962  
 
 OSGEPL1-AS1  101409258  2  190627505  190630282  2q32.2  0  0.43  0.159  0  0.271  0  0.17  0.232  0.332  0.203  0.091  0.243  0.171  0.257  0.297  0.23  0.136  1.023  0.83  0.415  0  0.33  0.886  0.185  0  0.221  0.185  0.076  0  0  0.353  0.218  0.228  0.46  0.379  0.304  0.128  0  0.446  0  0.155  0  0.121  0.148  0  0  0.232  0.178  0  0.29  0.088  0.341  0.201  0.43  0.299  0  0.204  0  0.21  0  
 
 ORMDL1  94101  2  190634992  190649097  2q32  2.291  4.896  2.133  2.591  2.873  2.673  3.8  3.223  3.111  2.837  3.261  2.201  2.439  3.311  2.179  2.722  2.798  3.738  4.868  3.264  2.121  3.272  3.285  2.536  2.718  2.741  2.353  2.184  3.091  1.621  3.472  3.44  2.991  2.353  2.28  2.488  2.622  2.624  3.773  3.007  2.378  2.229  3.489  2.13  1.86  2.048  3.278  1.65  2.183  4.029  2.659  1.483  2.134  2.331  3.419  2.264  3  2.767  3.186  2.592  
 
 PMS1  5378  2  190648810  190742355  2q31.1  0.697  2.509  0.802  1.678  0.798  0.699  0.855  0.908  1.118  1.126  0.951  0.67  1.159  2.283  1.014  1.309  1.551  2.112  3.118  1.397  1.234  1.51  1.288  1.031  0.921  1.252  0.56  0.719  1.083  0.293  1.572  1.089  1.041  0.822  0.999  1.309  1.141  1.105  1.527  1.404  1.127  0.875  1.224  1.312  0.664  0.698  0.566  0.838  0.789  2.021  1.189  0.834  0.791  0.981  1.692  0.876  1.6  1.178  1.669  1.488  
 
 MSTN  2660  2  190920425  190927455  2q32.2  0  0  0  0  0  0.069  0.071  0.105  0.134  0  0.032  0  0  0.183  0  0.031  0.05  0.041  0.052  0  0  0.128  0.157  0  0  0  0.035  0  0  0.02  0  0  0  0.254  0  0  0  0  0.028  0  0  0.026  0.081  0.153  0  0.025  0.04  0  0.066  0  0.035  0.163  0  0  0.043  0  0  0  0  0  
 
 C2orf88  84281  2  191002485  191068210  2q32.2  0.15  0.451  0.188  0  0.105  0.127  0.239  0.243  0.111  0.207  0.139  0.087  0.721  0.778  0.28  0.102  0.162  0.324  0.571  0.25  1.902  0.224  0.469  0.208  0.067  0.863  0.233  0.276  0.63  0.557  1.367  0.379  0.305  0.246  0.146  0.226  0.139  0.336  0.042  0.252  0.23  0.103  0.161  1.134  0.242  0.776  0.021  0.286  0.84  0.533  0.131  0.263  0.132  0.083  0.499  0.128  0.656  0.229  0.666  0.052  
 
 HIBCH  26275  2  191069359  191184771  2q32.2  1.657  3.108  0.714  0.631  2.492  1.025  2.084  1.358  1.291  1.288  1.55  1.227  1.819  3.318  1.234  1.726  2.096  4.382  3.737  1.338  1.702  1.57  1.63  2.215  1.373  1.586  1.015  0.978  2.589  0.677  2.556  2.106  2.31  1.973  1.45  1.933  1.32  1.389  1.301  1.783  1.692  1.484  2.21  1.756  0.889  1.844  1.172  0.923  1.34  3.163  1.596  0.637  1.07  1.327  1.792  1.407  1.82  1.255  2.334  2.084  
 
 INPP1  3628  2  191208195  191236391  2q32  0  2.903  1.701  0.499  0.061  1.077  3.204  2.352  2.023  1.7  1.314  2.014  2.996  2.621  1.205  2.389  3.962  1.102  2.614  0.905  0.215  0.601  0.605  0.25  0.888  1.719  0.625  0.899  1.378  0.179  1.932  2.095  1.193  0.733  0.662  1.758  2.145  2.286  2.228  0.803  1.376  1.159  1.333  1.481  0.84  1.132  2.312  1.461  1.581  2.731  1.897  0.99  0.726  0.537  2.509  0.436  1.785  1.129  1.193  1.737  
 
 MFSD6  54842  2  191273080  191367041  2q32.2  1.795  3.101  0.345  0.622  1.734  0.97  1.684  0.951  2.396  1.167  1.93  1.442  2.058  2.129  2.24  1.507  2.328  2.578  3.42  0.515  0.6  2.488  1.555  1.429  0.645  2.629  1.585  0.363  1.452  0.88  1.489  0.575  1.057  0.907  1.491  2.081  0.942  1.56  0.752  0.992  1.737  1.259  0.521  0.223  1.488  1.301  0.931  0.674  1.607  0.917  1.706  1.002  0.92  1.267  1.76  0.451  2.083  1.165  1.856  2.395  
 
 NEMP2  100131211  2  191371618  191399468  2q32.2  0.826  1.06  0.464  0.192  0.363  0.419  0.798  0.946  0.651  0.537  0.359  0.805  0.622  0.752  0.781  0.461  1.305  0.711  1.999  0.609  0.28  1.21  0.562  0.626  2.283  1.1  0.473  0.796  0.979  0.868  1.135  0.875  1.106  0.829  0.425  1.244  1.222  0.453  0.511  1.007  1.167  0.446  0.967  0.477  0.384  0.184  1.983  0.192  0.923  0.74  0.754  0.54  0.75  0.557  1.413  0.605  1.214  1.428  1.512  0.683  
 
 NAB1  4664  2  191513847  191557492  2q32.3-q33  1.039  2.949  0.949  0.717  0.979  0.836  1.72  2.6  1.573  1.394  1.221  1.702  1.809  2.562  1.489  1.518  3.637  3.936  3.032  1.366  1.002  1.465  0.79  0.575  1.958  1.713  1.194  1.62  1.939  1.003  2.057  1.58  1.613  1.066  1.167  2.979  1.966  1.669  1.644  2.643  1.732  0.943  1.947  1.2  2.262  1.133  1.268  1.145  2.279  2.58  2.161  0.835  1.227  0.911  1.667  1.729  2.471  1.555  2.262  1.609  
 
 GLS  2744  2  191745546  191830270  2q32-q34  0.904  3.726  3.567  1.757  1.672  3.102  2.632  3.607  2.53  3.037  3.4  0.983  3.948  2.651  2.061  2.035  2.94  3.344  4.287  2.772  1.727  2.786  2.566  1.989  2.913  2.965  2.048  2.652  3.249  1.536  3.336  3.7  3.544  3.509  2.203  3.359  2.52  2.751  4.953  2.029  2.113  2.23  2.149  3.738  1.654  2.57  2.508  1.607  4.372  3.67  3.933  3.793  4.335  4.21  6.649  4.384  5.57  1.677  5.932  5.042  
 
 STAT1  6772  2  191833761  191878976  2q32.2  2.074  3.848  4.126  4.656  2.57  2.448  2.915  3.461  2.048  3.879  2.122  1.867  3.047  3.472  2.483  5.933  1.875  2.471  3.303  2.547  1.781  2.208  1.692  3.357  4.004  3.022  2.15  2.591  4.2  1.664  2.819  2.78  2.738  2.132  2.882  3.203  2.156  2.396  5.321  3.679  2.922  2.015  2.747  2.385  1.85  2.436  2.256  1.653  2.955  3.448  3.054  1.976  4.079  3.384  6.388  2.628  6.823  2.41  5.313  3.576  
 
 STAT4  6775  2  191894301  192016322  2q32.2-q32.3  0  1.101  0.284  0  0  0.051  0.088  0.187  0  0.177  0.077  0.051  0  0.432  0.127  0.12  0  0.091  0.036  0  0  0.459  0.513  0.091  0.054  0  0.103  0  0  0.058  0  0  0  0.171  0.722  0.431  0  0.114  0.193  0.094  0.053  0.267  0.027  0  0  0.081  0.09  0  0.061  0.399  0.18  0.294  0.064  0.384  2.322  0.182  0.405  0  0.841  0  
 
 MYO1B  4430  2  192110106  192290115  2q12-q34  1.519  2.835  2.261  1.49  0.948  2.257  1.367  1.924  1.5  1.116  2.556  0.814  3.508  2.942  3.338  1.001  1.873  2.66  0.714  0.253  0  1.669  0.424  0.312  3.106  2.218  1.61  2.777  3  1.347  2.334  3.088  2.357  1.518  2.994  3.088  2.39  1.863  3.063  1.812  2.499  1.002  2.656  1.452  2.05  2.051  1.117  1.567  2.786  2.735  3.383  1.968  3.255  2.925  3.76  1.8  4.024  2.058  3.572  3.538  
 
 NABP1  64859  2  192542797  192553248  2q32.3  0.342  3.125  1.218  1.375  0.711  1.511  1.82  1.841  0.835  1.164  0.976  0.305  1.414  1.443  0.667  0.804  0.581  1.305  0.759  0.979  0.89  0.649  1.648  1.099  2.136  1.373  0.957  0.962  1.782  0.348  1.603  1.847  1.331  1.009  0.772  1.963  1.68  2.065  3.317  1.461  1.914  1.194  1.812  1.998  1.581  1.846  1.115  1.868  1.798  2.642  1.892  1.25  1.457  1.929  2.486  1.563  2.019  2.925  3.176  2.297  
 
 LOC105747689  105747689  2  192554906  192575195  -  0  0.532  0.154  0  0  0.34  0.35  0.292  0.129  0.061  0.178  0  0.089  0.608  0.096  0.04  0.07  0  0.055  0.128  0  0.137  0.444  0.08  0  0.098  0.212  0  0.365  0.127  0.121  0.162  0.083  0.397  0  0.105  0.261  0.154  0.502  0.11  0.36  0.069  0.041  0.252  0.205  0.307  0.343  0.24  0.118  0.323  0.116  0.227  0.257  0.261  0.645  0.14  0.079  0.191  0.729  0.084  
 
 SDPR  8436  2  192699031  192712006  2q32-q33  0  3.49  2.151  0.319  0.019  3.26  0.032  0.041  1.276  0.027  0.958  0.188  0  0.062  0  0.046  0.053  2.586  0.072  0.062  2.939  0.085  0.238  0  0.114  0  0.081  0.733  0.072  0.057  0  0  0  0.309  1.445  1.926  0.834  3.755  2.162  0.078  0  2.56  0.035  2.41  0.179  0.786  1.893  3.467  2.702  3.739  0.36  2.23  0.071  0.045  4.9  2.21  2.593  2.815  3.998  3.19  
 
 TMEFF2  23671  2  192813771  193059659  2q32.3  0  0.066  0.421  1.314  0.035  0.965  1.973  0.045  0.126  0.124  0  0.057  0  0.033  0  0  0.073  0  0.036  0  0  0.105  0  0  0.134  0  0.129  0.115  0.149  0.948  0  0  0  0.237  0.178  0  0.04  0.186  0.075  0.138  0  0.29  0.278  0  0  0.038  0.053  0.087  0  0.229  0.099  0.207  0  0.095  0.331  0.203  0.172  0.111  0.223  0  
 
 PCGEM1  64002  2  193614570  193641625  2q32  0  0.115  0  0  0  0  0  0  0  0  0  0  0  0  0  0  0  0  0  0  0  0  0  0  0  0  0.084  0  0  0.051  0  0  0  0.218  0  0  0  0  0  0  0  0.086  0.027  0  0  0  0.07  0  0  0  0  0  0  0  0  0  0  0  0  0  
 
 LOC101927406  101927406  2  195208992  195284355  2q32.3  0  0  0  0  0  0  0  0  0  0  0  0  0  0  0  0  0  0  0  0  0  0  0  0  0  0  0  0  0  0.038  0  0  0  0  0  0  0  0  0  0  0  0  0  0  0  0  0  0  0  0  0  0  0  0  0  0  0  0  0  0  
 
 LOC101927431  101927431  2  195595318  195626159  2q32.3  0  0  0.055  0  0  0  0  0  0.049  0  0  0  0  0  0  0  0  0  0.035  0  0  0.029  0  0  0  0  0.053  0  0  0.048  0  0.056  0  0.196  0  0  0  0  0  0  0  0.157  0  0  0  0.034  0  0  0  0  0  0  0  0  0  0  0  0  0  0  
 
 SLC39A10  57181  2  196521531  196602426  2q32.3  1.087  3.073  1.527  1.106  0.853  1.136  1.49  1.784  1.353  1.685  1.291  1.183  2.088  2.222  1.687  1.037  2.218  2.615  2.636  2.101  1.812  1.501  0.8  0.975  2.256  1.633  1.084  1.295  1.777  1.622  1.651  2.087  1.525  1.18  1.446  3.468  2.114  1.788  1.927  1.693  2.188  1.342  2.99  2.729  1.122  1.511  1.523  1.278  1.568  2.21  2.143  1.03  1.407  1.471  2.583  2.863  3.106  2.288  2.302  3.03  
 
 DNAH7  56171  2  196602426  196933536  2q32.3  0.02  0.011  0.068  0.026  0.059  0  0.034  0.029  0.047  0.027  0.035  0.048  0.023  0.025  0.024  0.015  0.037  0.07  0  0.038  0  0.055  0.112  0.013  0.024  0.011  0.038  0.048  0.017  0.058  0.014  0.024  0.025  0.144  0.013  0.073  0.014  0.02  0.027  0  0.03  0.1  0.097  0.032  0.034  0.027  0.025  0  0.074  0.041  0.044  0.142  0.014  0.041  0.04  0.05  0.158  0  0  0.011  
 
 STK17B  9262  2  196998306  197036336  2q32.3  0.434  1.914  1.536  1.132  0.554  1.078  1.257  2.215  0.54  1.145  0.89  1.056  1.199  1.074  1.853  1.408  2.066  0.516  3.844  1.167  0.722  2.156  1.834  1.226  1.746  2.368  0.571  0.95  2.059  0.071  0.869  1.343  0.657  0.703  0.589  0.991  1.279  1.379  1.045  0.609  2.108  0.693  0.453  1.789  1.397  1.087  1.044  0.795  1.457  1.536  1.582  0.581  0.89  0.537  1.133  0.76  2.072  1.261  2.021  0.954  
 
 HECW2  57520  2  197063974  197457335  2q32.3  0.548  0.107  0.949  0.232  0.022  0.296  0.717  0.476  0.084  1.025  0.052  0.04  0.043  0.327  0.024  0.013  0.027  0.075  0.012  0.048  0.957  0.056  0.225  0.158  0.476  0.058  0.069  0.046  0.058  0.103  0  0.132  0  0.193  0.203  1.096  0.557  0.647  0.556  0.153  0.408  0.248  0.496  0.036  0.092  0.021  0.387  0.302  0.242  0.361  0.396  0.259  0.455  0.353  0.061  0.132  2.013  0.333  0.606  0  
 
 LOC101927482  101927482  2  197124747  197128928  2q32.3  0  0.093  0.219  0  0  0.145  0.283  1.101  0  0.094  0  0  0.121  0  0.69  0.087  0  0.169  0.197  0  0  0.522  0  0  0.608  1.271  0  1.863  3.77  0.028  2.544  0.681  0  0.263  0  0  0.338  0  0.048  0  0  0.057  0.142  0  0  0  0  0  0  0  0.112  0  0.143  0  0.052  0  0.146  0  0  0  
 
 CCDC150  284992  2  197504355  197597530  2q33.1  0.458  1.101  0.169  0.129  0.347  0.395  0.321  0.373  0.068  0.448  0.468  0.178  0.308  1.303  0.54  0.531  0.434  1.28  1.543  0.788  0.53  0.738  0.51  0.362  0.299  0.23  0.452  0.242  0.524  0.105  0.466  0.455  0.56  0.24  0.406  0.43  0.536  0.512  0.474  0.518  0.507  0.389  0.998  0.727  0.469  0.985  0.91  0.368  0.655  1.064  0.345  0.312  0.305  0.516  0.704  0.187  1.15  0.451  0.691  0.198  
 
 GTF3C3  9330  2  197627755  197664492  2q33.1  1.821  2.624  1.128  1.058  1.67  1.02  2.496  1.829  1.444  1.472  1.436  1.189  2.125  3.035  2.198  1.792  2.335  3.367  3.585  2.775  1.8  2.506  1.261  1.14  1.815  1.951  1.749  1.631  2.382  1.422  2.214  2.59  2.206  1.415  1.48  1.87  2.291  2.012  2.469  2.547  2.123  1.28  2.644  1.798  1.511  1.496  2.151  1.213  1.293  3.01  2.32  1.207  1.596  1.551  2.547  1.61  2.421  1.825  2.376  2.293  
 
 C2orf66  401027  2  197669138  197675000  2q33.1  0  0.241  0  0.121  0  0  0  0.047  0.068  0.081  0.08  0.068  0  0.235  0  0  0.084  0.089  0.038  0.084  0  0.093  0.127  0  0.064  0.059  0.087  0.039  0.116  0.116  0.117  0.381  0  0.311  0.122  0.118  0  0.291  0.215  0  0  0.283  0.047  0.279  0  0.067  0.353  0  0  0.364  0.183  0.065  0  0.102  0  0.122  0  0.072  0  0  
 
 PGAP1  80055  2  197697727  197791454  2q33.1  0.457  0.406  0.296  0.33  0.382  0.375  0.443  0.422  0.439  0.577  0.309  0.153  0.229  0.576  0.616  0.193  0.916  0.868  0.943  0.292  0.049  0.549  0.203  0.104  0.244  1.156  0.366  0.384  0.972  0.159  0.714  0.967  0.845  0.542  0.22  0.285  0.358  0.566  0.386  0.834  0.455  0.217  0.317  0.404  0.229  0.357  0.29  0.274  0.189  0.565  0.751  0.179  0.209  0.233  0.494  0.176  0.718  0.375  0.393  0.471  
 
 ANKRD44  91526  2  197851385  198175521  2q33.1  0.492  0.916  0.486  0.135  0.713  0.313  0.991  1.2  0.593  0.931  0.679  0.158  0.275  0.517  0.39  0.078  0.497  0.315  1.781  1.54  0  1.088  1.074  1.34  0.436  2.127  2.741  1.957  3.707  1.986  1.982  1.839  2.838  1.176  0.188  0.801  0.686  0.183  0.558  0.44  0.32  0.785  0.34  0.276  0.133  0.522  1.702  0.488  0.746  0.254  0.548  0.624  0.662  0.823  0.326  0.144  0.704  0.475  0.813  0.477  
 
 ANKRD44-IT1  100874357  2  198115581  198167243  -  0  0  0.153  0  0.069  0  0  0.076  0  0.088  0  0  0  0.048  0  0  0  0  0  0.302  0  0.339  0  0.168  0  0.378  0.526  0.16  0.921  0.236  0.36  0.391  0.403  0.334  0  0  0  0  0.058  0  0  0.115  0.091  0  0  0  0.094  0.172  0.165  0  0  0.28  0.082  0  0.115  0  0.116  0  0  0  
 
 SF3B1  23451  2  198256697  198299817  2q33.1  4.515  5.574  2.699  2.804  4.024  3.304  4.759  4.455  3.521  3.973  4.72  3.625  4.385  5.864  3.69  4.157  4.495  5.664  6.099  4.777  3.716  4.461  3.012  3.405  4.036  4.23  3.553  4.123  4.414  3.381  4.481  4.674  4.337  2.625  3.392  4.405  4.338  4.104  4.075  4.949  4.055  3.043  5.378  4.555  3.422  3.981  4.104  3.351  3.917  5.247  4.272  2.856  3.812  3.593  4.573  3.597  4.86  3.926  5.14  4.465  
 
 COQ10B  80219  2  198318230  198339851  2q33.1  2.294  3.992  2.039  2.001  2.097  1.615  3.078  3.07  2.59  2.257  2.657  1.756  2.134  3.125  1.597  2.173  2.686  3.453  3.122  0.903  1.078  1.224  1.86  0.946  2.501  3.067  2.041  2.099  3.075  1.463  2.858  2.944  2.512  2.058  1.338  2.196  2.33  2.152  2.452  2.66  2.007  1.17  2.249  2.107  1.256  2.011  0.774  1.362  1.227  3.114  2.808  1.243  1.647  2.099  2.356  1.562  2.696  2.308  2.69  2.498  
 
 HSPD1  3329  2  198351307  198364998  2q33.1  6.053  7.589  4.395  4.636  5  4.752  5.848  5.368  5.304  4.773  6.662  5.53  6.336  8.049  6.068  6.329  7.5  8.043  7.744  6.854  6.33  5.723  6.008  5.791  6.125  5.729  4.745  5.701  6.071  5.463  6.751  6.94  6.391  5.32  5.956  5.619  6.539  6.003  6.202  6.579  5.271  6.614  6.639  7.041  5.338  5.681  5.481  5.203  5.654  7.716  6.374  4.038  5.936  5.491  6.557  5.814  6.594  4.957  7.2  6.751  
 
 HSPE1  3336  2  198364720  198368187  2q33.1  5.507  8.872  4.057  4.166  5.115  5.552  6.403  5.351  5.132  4.998  5.976  5.899  5.659  7.286  5.103  6.62  7.372  10.269  7.48  7.511  6.15  7.262  5.466  6.638  6.414  5.503  4.985  4.319  5.695  4.65  6.508  6.286  5.637  5.025  6.839  5.456  5.473  5.466  7.23  5.74  5.673  6.707  6.775  6.489  5.36  5.578  7.246  4.873  5.322  7.199  5.343  3.792  5.896  6.315  7.152  5.957  5.86  6.09  7.16  5.846  
 
 HSPE1-MOB4  100529241  2  198364720  198418423  2q33  0  0.195  0  0  0  0  0  0.087  0  0.026  0.027  0  0.068  0.054  0  0.035  0  0  0.051  0  0.219  0  0  0  0.055  0  0  0  0  0  0  0.054  0.054  0  0  0.076  0.045  0.071  0  0  0  0  0  0  0  0.078  0  0  0.179  0.116  0.068  0  0  0.026  0  0  0.037  0  0  0.058  
 
 MOB4  25843  2  198380294  198418423  2q33.1  2.118  4.026  1.572  1.044  2.363  2.15  2.988  2.203  2.061  1.938  2.866  1.717  2.815  2.827  1.752  2.319  1.844  3.822  3.752  2.222  1.127  1.741  1.25  0.965  2.522  2.506  1.728  1.711  2.226  1.126  2.679  2.855  2.179  1.428  1.669  2.124  2.19  2.075  3.163  2.451  2.431  2.044  2.467  2.078  1.813  2.177  2.062  1.187  1.752  3.084  2.272  1.012  1.713  1.889  2.072  1.426  2.444  2.017  2.68  2.588  
 
 RFTN2  130132  2  198435526  198540584  2q33.1  0.593  0.057  0.049  0  0.035  0.054  0.064  0.7  0.059  0.033  0.206  0  0  0.052  0  0.138  0  0.09  0.284  0.043  0  0.114  0  0  0.052  0.513  0.289  0.058  0.048  0.281  0.234  0.446  1.051  0.857  0.052  0.15  0.04  0  0.118  0.306  0.034  0.204  0.049  0.075  0  0.056  0.231  0.048  0.188  0  0.041  0.119  0.031  0.028  0.069  0.042  0.061  0  0  0  
 
 MARS2  92935  2  198570027  198573114  2q33.1  1.526  1.115  0.541  1.09  1.367  0.606  1.229  0.721  1.327  0.732  1.153  1.199  2.408  1.735  1.853  1.657  1.837  1.087  3.106  2.175  1.857  1.89  1.657  1.817  1.581  1.786  1.495  1.494  1.807  1.762  1.941  1.996  2.398  1.605  1.107  1.387  1.591  1.369  1.056  1.698  1.079  1.613  1.432  1.603  1.198  1.293  0.178  1.176  1.037  1.809  1.357  1.128  0.974  0.962  1.087  0.558  0.95  1.083  1.661  1.222  
 
 BOLL  66037  2  198591602  198651036  2q33  0.09  0.045  0  0  0  0.053  0.084  0.031  0.061  0  0  0  0  0.02  0  0.239  0  0.126  0.093  0  0  0.314  0.207  0  0  0.061  0.096  0  0.074  0.05  0  0  0  0.191  0  0.117  0  0.074  0.023  0  0  0.125  0.043  0.055  0  0  0.064  0  0.107  0  0  0.323  0  0.086  0  0  0  0  0  0  
 
 PLCL1  5334  2  198669425  199014608  2q33  0.044  0.026  0.065  0.08  0.057  0.023  0.032  0.028  0.086  0.098  0.035  0.021  0  0.026  0  0.05  0.022  0.046  1.191  0.035  0.684  0.887  0.444  0.35  0  0.862  0.164  0.019  0.49  0.059  0.374  0.079  0  0.232  0  0.092  0  0  0.025  0  0.037  0.124  0.014  0.674  0  0.03  0.036  0.028  0.025  0.025  0.023  0.219  0.01  0.052  0.031  0  0  0  0  0  
 
 LOC101927619  101927619  2  199164086  199239821  2q33.1  0  0.035  0  0  0.026  0.039  0  0.041  0.121  0.024  0  0  0  0  0  0  0  0.072  0  0  0  0  0.275  0  0  0  0.055  0.033  0  0.042  0.054  0.082  0.05  0.182  0  0  0  0  0  0  0.059  0.093  0.03  0  0.106  0.073  0.032  0  0  0  0  0  0  0  0  0  0  0.096  0  0  
 
 SATB2  23314  2  200134222  200335989  2q33  1.047  2.077  0.903  0.618  0.517  1.296  1.639  1.969  0.884  1.872  1.218  1.727  1.144  1.534  2.426  0.032  1.724  0.984  0.798  1.883  2.01  0.188  0.506  0.062  2.321  1.166  1.723  1.935  1.913  1.115  1.337  2.047  2.309  1.473  0.51  1.605  1.465  1.747  0.853  1.324  1.399  1.148  0.622  0.035  0.157  0.056  0.999  0.838  0.851  1.232  0.992  0.743  1.285  0.824  1.413  0.718  2.033  1.147  1.351  1.534  
 
 SATB2-AS1  150538  2  200332820  200337481  2q33.1  0  0.082  0.107  0  0.021  0.122  0  0.047  0.087  0.079  0.019  0.029  0.062  0.03  0.128  0.024  0.056  0.101  0  0.031  0.138  0.068  0.322  0  0  0  0.077  0  0  0.055  0  0  0.079  0.215  0.05  0  0  0  0.031  0  0.034  0.254  0.038  0  0  0  0.032  0  0  0  0  0.305  0.022  0.061  0.069  0  0  0  0  0.043  
 
 LOC101927641  101927641  2  200472790  200523855  2q33.1  0  0.033  0.078  0  0  0  0  0  0.06  0  0  0  0  0.023  0  0  0  0  0.03  0  0  0.057  0.234  0  0  0  0.025  0.031  0  0.017  0  0  0  0.211  0  0  0  0  0.06  0  0  0  0  0  0  0  0  0  0  0  0  0.108  0  0  0  0  0  0  0  0  
 
 FTCDNL1  348751  2  200625258  200715896  2q33.1  0.307  0.523  0.24  0  0.155  0.331  0  0  0.291  0.278  0  0  0.228  0.321  0  0.158  0  0.572  0  1.86  0  0.3  0  0  0.296  0.404  0.119  0.2  0  0.352  0.423  0.286  0  0.328  0.39  0  0.235  0  0.107  0.331  0.328  0.286  0  0  0  0.109  0.115  0  0.401  0.334  0  0.759  0.065  0.246  0.755  0  0.142  0.175  0  0.244  
 
 C2orf69  205327  2  200775978  200792996  2q33.1  1.229  2.42  0.991  1.437  1.272  1.009  1.289  1.125  1.413  1.245  1.403  0.52  1.503  1.61  1.32  1.388  1.271  1.61  1.888  1.881  0.724  1.464  0.879  0.473  1.257  1.687  1.126  0.893  1.395  0.841  1.995  1.622  0.98  1.03  0.823  0.96  1.073  1.177  1.857  1.543  1.025  1.188  1.452  1.016  0.95  0.976  1.578  0.907  1.308  2.528  1.693  0.822  0.966  0.663  1.533  0.538  1.572  1.09  1.756  1.186  
 
 TYW5  129450  2  200793633  200820459  2q33.1  0.839  1.246  0.434  0.476  0.794  0.318  0.757  0.766  0.682  0.583  0.957  0.557  0.768  1.123  0.782  1.016  0.869  1.343  1.488  0.899  0.28  0.901  0.578  0.27  0.727  1.288  0.675  0.444  1.167  0.428  0.964  0.875  0.668  0.597  0.578  0.768  0.724  0.713  0.908  0.719  0.935  0.489  1.247  0.459  0.393  0.628  0.732  0.43  0.551  1.298  1.048  0.559  0.492  0.434  1.296  0.417  1.04  0.673  1.518  0.61  
 
 C2orf47  79568  2  200820039  200828847  2q33.1  2.456  4.124  1.205  1.9  2.456  1.509  2.485  1.828  2.322  1.739  2.295  2.164  2.22  3.372  1.91  2.759  2.695  5.046  3.879  2.601  1.751  2.085  1.893  1.413  2.629  1.741  1.457  1.582  2.174  1.454  3.006  3.049  2  1.675  2.039  2.321  1.862  1.678  2.35  1.992  2.364  2.855  2.519  2.079  1.336  2.003  1.934  1.672  1.421  3.314  1.959  1.439  1.824  1.511  3.192  1.349  2.145  2.39  3.545  2.266  
 
 SPATS2L  26010  2  201170603  201346986  2q33.1  0.442  4.921  2.727  2.157  1.511  2.04  4.116  2.818  2.391  2.707  3.019  2.832  2.471  3.433  1.656  4.605  3.495  4.411  0.126  0.44  0.897  0.579  1.182  1.22  3.876  2.505  2.39  2.184  2.77  1.3  2.479  3.662  2.788  2.028  1.823  3.276  3.265  2.944  3.284  1.761  3.436  2.319  0.041  3.268  1.912  2.909  4.15  2.172  2.809  3.322  3.456  1.488  2.714  3.458  4.12  2.563  4.299  2.209  4.019  3.918  
 
 KCTD18  130535  2  201353683  201374792  2q33.1  1.586  1.488  0.98  0.837  1.446  0.74  1.235  0.988  2.082  1.151  1.406  0.704  1.336  1.856  0.826  0.946  1.17  1.468  2.659  1.027  0.306  1.219  1.147  0.251  0.986  1.223  0.765  0.851  1.185  1.025  1.459  1.135  0.824  0.734  0.637  1.576  1.044  0.927  0.889  1.344  1.432  1.243  0.987  0.628  0.582  0.727  0.35  0.272  0.703  1.445  1.081  0.695  0.757  0.855  1.21  0.582  1.307  1.001  1.161  1.166  
 
 SGOL2  151246  2  201390864  201448818  2q33.1  1.954  4.608  1.342  1.333  1.28  1.614  2.21  1.899  0.692  2.117  2.672  1.19  1.213  2.743  1.345  1.325  1.521  3.029  2.976  2.117  1.258  1.893  0.756  0.691  1.985  1.797  1.091  1.243  1.851  0.765  1.932  1.889  2.031  1.762  1.329  1.785  1.693  1.909  1.32  2.358  1.71  1.843  2.146  1.717  1.128  1.595  1.637  1.28  1.482  2.214  2.556  0.96  1.607  1.096  1.902  1.135  2.7  1.73  2.537  1.889  
 
 AOX1  316  2  201450730  201536217  2q33  0.086  2.24  0.958  0.672  0.017  1.154  1.064  0.909  0.051  0.674  0.158  0.03  0.028  0.055  0  0.063  0.06  0.087  0.019  0  0  0.069  0.108  0  2.667  0.02  0.04  0.207  0.095  0.055  0.066  0.1  0  0.17  0.036  0.698  1.825  0.71  1.154  0.483  0.19  0.215  0.265  0  0.054  0.063  0.048  0.327  0.1  1.377  2.207  0.188  0.672  0.505  0.535  0.383  1.144  2.65  0.255  1.31  
 
 AOX2P  344454  2  201560445  201658941  2q33.1  0.118  0.045  0.084  0  0.075  0.035  0.084  0  0.06  0.05  0.084  0.093  0.085  0.185  0.097  0.098  0.215  0.028  0.097  0.149  0  0.252  0.11  0.054  0.035  0.038  0.158  0.038  0.117  0.093  0  0.042  0.06  0.129  0.14  0.053  0.096  0  0.182  0.102  0.032  0.046  0.162  0.031  0  0.02  0.015  0  0  0.089  0.125  0.16  0.158  0.118  0.224  0.077  0.093  0  0.141  0  
 
 LOC100507140  100507140  2  201577027  201599900  2q33  0  0.325  0.083  0  0  0  0  0  0.091  0.03  0  0.068  0  0.05  0  0  0  0  0  0  0  0.098  0.267  0  0  0  0.081  0  0  0.059  0  0  0  0.138  0  0  0  0  0  0  0.126  0.084  0.023  0  0  0  0.093  0  0  0  0.163  0.175  0  0  0  0  0  0  0  0  
 
 LOC101927795  101927795  2  201645217  201676893  2q33.1  0  0.337  0.183  0.324  0  0.406  0  0.165  0.292  0.078  0  0.279  0  0.339  0  0.282  0.521  0  0.128  0  0.242  0.552  0.298  0  0.421  0  0.078  0  0  0.216  0.284  0.172  0  0  0.51  0.172  0  0  0.132  0  0.151  0.72  0.058  0.466  0  0.237  0.292  0  0.482  0  0  0.441  0.257  0.668  0.435  0.219  0  0  0  0  
 
 BZW1  9689  2  201676268  201688569  2q33  3.769  6.7  3.709  2.972  3.072  3.9  4.655  4.358  4.416  3.878  4.947  3.343  4.354  5.882  3.869  3.818  5.076  5.757  5.996  3.607  3.076  3.738  2.902  2.37  5.042  4.312  2.63  3.427  3.979  2.519  4.39  4.737  3.638  3.286  3.445  5.017  4.243  3.772  3.941  4.706  3.955  4.552  5.044  4.057  3.268  3.68  3.508  3.226  3.811  5.765  5.654  3.622  3.977  3.501  4.047  3.543  5.295  4.332  5.218  4.826  
 
 CLK1  1195  2  201717731  201729467  2q33  2.204  4.724  1.305  1.159  2.182  1.741  2.791  2.421  2.266  2.764  2.59  1.644  1.689  3.65  2.394  2.772  2.91  5.635  4.911  2.66  1.492  2.586  2.209  1.585  2.53  2.185  2.129  1.475  1.91  1.351  2.699  2.606  2.014  1.206  1.513  2.01  2.239  2.564  2.794  2.258  1.738  1.46  3.367  1.976  1.24  1.897  1.805  1.932  1.544  3.697  2.448  1.023  1.773  1.376  2.506  1.153  3.133  2.317  2.669  2.034  
 
 PPIL3  53938  2  201735678  201753849  2q33.1  3.09  4.94  1.545  1.168  2.808  1.989  3.706  2.393  1.826  2.165  3.25  1.991  2.947  4.296  1.788  2.327  3.216  5.682  4.871  3.389  1.611  3.027  1.737  1.369  2.535  2.174  2.144  2.101  2.22  1.209  2.622  2.71  2.675  2.137  1.892  2.018  2.255  2.372  2.423  1.707  1.765  1.729  3.59  2.175  1.48  1.881  3.528  2.096  1.21  4.353  2.175  1.465  2.218  2.011  2.897  2.072  3.34  2.672  3.155  2.28  
 
 NIF3L1  60491  2  201754049  201768655  2q33  3.462  4.7  2.239  2.3  3.095  2.364  4.128  2.672  2.411  2.454  3.403  2.715  3.066  4.032  3.255  3.169  3.522  4.568  5.133  3.593  2.442  3.476  2.919  2.678  3.228  2.764  3.326  2.59  3.551  2.817  3.633  3.429  3.845  2.67  2.899  3.141  3.414  3.047  2.368  3.557  3.27  2.526  3.99  2.743  2.685  2.635  3.22  2.304  2.313  4.496  2.996  1.763  2.866  2.534  3.721  2.33  3.459  3.321  3.587  2.983  
 
 ORC2  4999  2  201774893  201828424  2q33  1.918  1.739  0.665  0.524  0.954  1.001  1.709  1.3  0.849  1.001  1.625  1.145  2.068  2.224  1.677  1.656  0.789  1.412  2.536  1.397  1.736  1.419  0.939  0.911  1.717  1.219  0.965  1.205  1.59  0.58  1.177  1.471  1.436  0.882  1.281  1.453  1.456  1.182  1.018  1.977  0.853  1.129  2.199  1.64  1.117  1.162  0.927  1.145  1.587  2.523  1.798  0.968  1.208  1.227  1.639  1.027  1.74  1.529  1.923  1.305  
 
 FAM126B  285172  2  201838440  201936392  2q33.1  0.491  0.501  0.445  0.666  0.529  0.389  0.425  0.856  0.578  0.509  0.524  0.427  1.061  0.592  0.823  0.849  0.799  0.567  1.432  1.182  0.836  1.212  0.605  0.382  0.599  1.051  0.393  0.404  0.778  0.351  0.744  0.648  0.521  0.515  0.229  0.681  0.552  0.715  0.516  0.85  0.659  0.397  0.748  0.673  0.437  0.776  0.406  0.427  0.607  0.849  0.66  0.365  0.45  0.555  0.446  0.284  1.132  0.557  0.886  0.551  
 
 NDUFB3  4709  2  201936461  201950473  2q31.3  4.288  6.961  3.112  2.526  5.085  4.86  5.26  3.911  3.272  3.971  4.305  4.739  4.041  5.434  2.761  4.75  5.145  7.553  6.508  6.734  4.505  5.911  3.953  4.961  4.827  4.256  2.887  2.727  4.469  2.925  4.957  4.797  3.672  2.982  4.873  4.287  3.641  3.699  5.195  3.481  4.128  4.426  4.743  4.292  3.694  4.626  6.033  2.89  3.563  5.418  3.869  2.41  3.751  4.659  5.37  4.149  4.271  5.063  5.134  4.013  
 
 CFLAR  8837  2  201980826  202037411  2q33-q34  2.188  4.946  2.595  2.132  2.183  2.347  1.792  2.725  1.745  2.168  2.191  2.267  2.813  2.415  2.557  3.41  2.709  3.618  2.461  1.899  2.093  1.536  2.552  2.249  3.602  2.155  1.887  1.463  1.885  2.163  2.361  2.275  2.455  1.804  2.573  2.887  2.889  3.362  3.868  2.854  2.353  1.939  2.321  2.578  1.006  2.44  3.036  2.636  2.518  3.974  2.714  3.138  3.751  3.267  4.381  3.334  4.129  4.462  4.708  3.669  
 
 CFLAR-AS1  65072  2  202005011  202022515  2q33  0  0.137  0.159  0  0.063  0.166  0  0.132  0  0  0  0  0  0.065  0  0.174  0  0  0  0  0  0.072  0  0  0  0.08  0.047  0  0  0.126  0.2  0  0  0  0.243  0.116  0.111  0.376  0.145  0  0.244  0.294  0  0.185  0  0  0.06  0.304  0  0.176  0.084  0.273  0.21  0.242  0.468  0.196  0.438  0.587  0.545  0  
 
 CASP10  843  2  202047603  202094129  2q33-q34  0  0.823  0.504  0  0  0.103  0.653  0  0.219  0.093  0.212  0.669  1.313  0.455  1.088  1.165  0.426  0.11  2.064  1.456  1.221  1.777  1.517  1.121  0.084  0.614  0.154  0.127  0.533  0.62  0  0.653  0  0.565  0.386  0.075  0.409  0.656  0.359  0.301  0  0.585  0.033  0  0.505  0.955  0.599  0.233  0.58  0.667  0.445  0.709  0.623  1.12  1.171  0.612  1.013  0.821  1.112  1.024  
 
 CASP8  841  2  202098165  202152434  2q33-q34  0.813  1.866  0.664  1.375  0.858  0.327  1.246  1.608  0.749  1.003  1.671  1.891  2.321  1.543  1.938  1.963  1.463  1.336  3.335  1.515  0.902  1.662  1.622  1.697  2.39  1.779  1.196  1.559  2.09  0.901  1.758  1.982  1.885  1.237  1.453  1.625  1.834  1.814  1.323  1.67  1.684  1.238  0.682  1.209  1.27  1.716  1.349  1.078  1.764  2.282  1.669  0.964  1.394  1.926  2.253  1.288  2.912  2.197  2.564  2.441  
 
 ALS2CR12  130540  2  202152993  202222121  2q33.1  0  0  0.112  0  0.092  0.093  0  0.083  0.099  0.096  0.064  0.074  0  0.166  0.141  0.05  0  0.452  0.157  0.093  0  0.096  0.199  0  0  0.059  0.3  0.105  0.159  0.226  0.502  0.15  0.146  0.217  0  0.072  0.064  0.146  0.156  0  0  0.391  0  0  0  0.051  0.052  0  0.125  0.112  0.111  0.174  0.074  0.106  0.08  0  0.339  0  0.225  0  
 
 TRAK2  66008  2  202241929  202316319  2q33  2.541  1.572  2.199  1.368  1.328  1.744  2.878  2.343  2.254  2.346  2.022  0.979  1.28  1.921  1.619  1.154  1.284  0.428  1.487  1.59  0.856  1.045  0.91  0.772  1.886  2.49  2.68  2.105  2.955  2.719  3.406  2.694  3.094  1.997  1.139  1.988  1.812  1.938  1.949  1.965  1.332  1.179  2.085  1.373  1.406  1.626  2.099  1.128  1.611  1.6  2.365  0.924  1.674  1.665  1.711  1.561  2.772  1.506  2.334  2.012  
 
 STRADB  55437  2  202316391  202345574  2q33.1  2.094  3.926  1.544  0.799  1.155  1.591  2.228  1.626  2.753  2.061  2.785  0.805  1.748  2.767  0.569  1.176  1.557  2.556  3.428  1.266  1.285  1.106  1.023  0.599  1.34  2.897  2.12  2.081  2.752  2.119  3.953  3.629  3.34  2.358  1.327  2.623  2.184  2.928  1.654  2.058  1.118  1.718  2.948  1.634  0.515  1.473  1.419  0.577  2.181  2.08  2.081  0.834  1.72  1.467  2.476  1.569  2.88  1.007  3.03  3.194  
 
 ALS2CR11  151254  2  202352143  202483905  2q33.1  0  1.223  0.291  0  0  0  0  0.055  0.088  0  0  0  0  0.031  0  0  0  0  0  0  0  0.084  0.139  0  0  0.055  0.073  0.315  0.195  0.186  0.063  0.995  0  0.284  0  0.084  0  0.633  0.069  0.08  0  0.26  0.208  0  0.473  0.045  0.078  0.291  0  0.76  0.036  0.191  0  0.062  0  0  0.072  0.272  0.499  0  
 
 TMEM237  65062  2  202484906  202508252  2q33.2  1.029  2.056  1.115  0.637  1.193  1.047  2.15  1.595  0.72  1.497  1.336  0.468  0.847  2.068  0.893  0.68  1.258  1.972  3.226  0.814  0.475  1.674  0.451  0.275  0.951  1.4  1.009  0.495  1.324  0.806  1.564  1.323  1.362  0.802  0.69  1.032  1.058  1.298  1.548  1.4  1.15  1.057  2.037  0.953  0.745  1.351  0.907  0.505  1.137  1.761  1.205  0.622  0.855  0.952  1.657  0.533  1.901  1.276  1.351  2.143  
 
 MPP4  58538  2  202509596  202563417  2q33.2  0  0.202  0.263  0  0.023  0.525  0.727  0.21  0.094  0.883  0.188  0  0  0.069  0  0.031  0  0  0  0  0  0.054  0  0  2.589  0  0.134  0.125  0  0.036  0.097  0.332  0.341  0.448  0  0.196  1.393  1.456  0.819  0.102  0.071  0.027  0.024  0.087  0  0.059  0.078  0  0  0.104  0.805  0.109  0.109  0.24  0.143  0.196  0.296  0.471  0  0.87  
 
 ALS2  57679  2  202564985  202645895  2q33.1  1.179  2.395  1.232  1.066  1.27  1.599  1.901  1.494  1.117  1.523  1.76  1.63  1.538  1.918  1.452  1.275  1.27  1.671  1.754  1.397  0.913  0.943  0.589  0.278  1.53  1.932  1.076  1.484  1.932  1.183  1.404  1.575  1.601  0.95  0.893  1.482  1.947  1.856  1.299  1.841  1.562  0.885  2.135  2.038  0.928  1.452  0.978  1.108  2.01  2.101  1.66  1.025  1.716  1.344  2.644  1.099  2.624  1.679  2.788  2.298  
 
 CDK15  65061  2  202671151  202760273  2q33.2  0  2.334  1.045  0.564  0  0.686  1.702  3.109  0.066  1.542  0  1.848  0  0.029  0  0  0  0.352  0.034  0.061  0  0.074  0.336  0.052  0.124  0.254  0.122  0.042  0.118  0.076  0.18  0.059  0  0.364  0  0  0.71  2.399  0.194  0  0.277  0.401  0.052  0.044  0  0.054  0.178  0.056  0  0.308  0.031  0.194  3.018  1.676  0.174  0.161  2.309  0.735  0.332  0  
 
 FZD7  8324  2  202899309  202903160  2q33  0.319  3.536  2.614  1.668  0.713  1.499  3.371  2.28  2.719  1.9  1.739  0.413  2.745  1.643  1.442  1.341  0.917  0.265  0.097  0.229  0.248  0.337  0.76  0.141  0.614  1.442  2.015  1.363  1.911  1.28  1.93  2.072  2.439  1.766  0.761  1.075  2.175  1.604  0.48  0.796  3.842  1.572  1.244  1.327  0.404  1.306  1.294  1.28  1.465  2.302  0.088  1.413  1.702  1.428  1.746  1.006  2.273  1.634  2.312  1.441  
 
 KIAA2012  100652824  2  202937977  203061886  2q33.1  0.076  0.021  0.106  0  0.017  0.12  0.085  0.022  0.096  0.058  0.039  0.263  0.051  0.054  0  0.058  0.044  0.036  0  0.078  0  0.078  0.29  0  0.028  0  0.241  0.02  0.045  0.275  0.038  0.034  0  0.184  0.196  0  0  0  0.045  0.059  0  0.297  0.056  0  0  0.034  0.264  0  0  0  0.025  0.209  0.027  0.096  0.029  0.156  0.025  0  0.064  0  
 
 SUMO1  7341  2  203070902  203103322  2q33  3.992  6.681  3.388  3.58  4.132  3.466  4.348  3.89  4.225  3.858  4.282  3.305  3.723  5.539  3.108  4.664  4.531  7.418  6.169  3.823  2.97  3.764  3.783  2.767  4.436  3.451  2.44  3.061  3.634  1.914  3.895  4.119  3.865  3.647  3.294  3.795  3.545  3.325  3.563  4.133  3.946  3.566  4.879  3.782  3.635  3.881  4.166  3.074  3.71  5.961  3.754  2.633  2.957  3.161  4.353  3.083  4.101  4.572  4.494  4.26  
 
 NOP58  51602  2  203130514  203168384  2q33.1  3.789  5.807  2.426  2.752  3.97  3.106  3.983  3.993  2.393  3.455  4.592  3.625  4.287  5.356  3.853  4.32  4.058  6.845  6.142  3.735  4.254  4.352  3.462  3.983  4.732  3.693  2.721  3.972  4.097  2.143  4.518  5.506  4.331  3.136  3.832  4.211  4.006  3.39  3.324  4.693  4.262  3.9  4.389  4.44  4.072  4.215  3.632  3.331  3.734  5.638  3.821  2.79  3.753  3.073  4.196  3.506  4.487  3.716  4.78  4.489  
 
 BMPR2  659  2  203241049  203432474  2q33-q34  2.26  2.913  1.74  1.174  2.101  1.816  2.515  3.396  1.734  2.005  2.227  0.785  1.215  1.559  1.185  2.102  0.55  1.133  0.356  0.571  0.414  0.691  0.801  0.781  2.725  3.097  1.561  1.962  2.472  1.067  1.779  1.61  1.419  1.003  1.231  2.784  1.841  2.016  1.847  2.263  1.965  0.837  3.05  1.521  1.323  1.982  1.889  1.886  2.41  2.287  2.262  1.041  1.809  1.559  2.473  1.568  3.387  1.421  2.589  2.816  
 
 FAM117B  150864  2  203499900  203634480  2q33.2  1.518  0.566  0.391  0.286  0.648  0.254  2.087  0.423  0.917  0.713  0.924  1.349  1.378  1.599  1.168  1.507  1.299  1.193  2.576  1.279  0.762  2.345  0.931  0.855  1.189  0.951  1.167  1.146  1.068  0.963  1.025  1.656  1.449  0.897  0.759  1.032  0.696  0.567  0.305  1.117  0.741  0.486  2.233  1.45  1.097  1.121  0.853  0.458  1.027  1.129  1.394  0.572  0.833  0.49  1.519  0.948  0.67  0.851  1.393  0.973  
 
 ICA1L  130026  2  203637872  203736708  2q33.2  0.44  0.951  0.268  0.187  0.249  0.361  0.646  0.406  0.528  0.446  0.604  0  0.188  0.426  0.383  0.157  0.165  0.664  1.151  0.536  0.278  0.585  0.467  0.092  0.355  0.491  0.272  0.347  0.427  0.385  0.346  0.518  0.233  0.396  0.316  0.446  0.216  0.283  0.329  0.263  0.292  0.396  1.139  0.301  0.427  0.235  0.218  0.15  0.556  0.154  0.511  0.508  0.51  0.212  0.531  0.143  0.298  0.513  0.353  0.23  
 
 WDR12  55759  2  203745322  203776949  2q33.2  2.382  4.095  1.299  1.616  2.105  1.762  3.052  2.139  1.325  1.873  2.652  2.386  3.057  3.948  2.299  2.598  2.976  4.608  4.301  3.45  3.263  2.845  1.584  2.487  3.205  2.915  2.504  2.777  3.417  2.236  3.557  3.989  3.52  1.826  2.124  2.743  2.844  2.326  1.835  3.287  2.376  1.77  3.769  2.706  2.277  2.484  1.698  1.809  2.007  3.754  2.651  1.407  2.318  1.636  3.239  1.939  1.798  2.739  3.087  2.78  
 
 CARF  79800  2  203776940  203851208  2q33.2  0.44  0.188  0.148  0.079  0.349  0.092  0.214  0.248  0.498  0.476  0.204  0.136  0.27  0.336  0.247  0.274  0.179  0.346  1.077  0.313  0  0.507  0.304  0.1  0.1  0.408  0.182  0.158  0.227  0.145  0.177  0.105  0.112  0.243  0.137  0.321  0.178  0.179  0.152  0.26  0.299  0.122  0.239  0.14  0.078  0.134  0.161  0.044  0.189  0.149  0.324  0.271  0.152  0.196  0.385  0.081  0.23  0.057  0.189  0.276  
 
 NBEAL1  65065  2  203879601  204082717  2q33.2  0.497  0.68  0.388  0.182  0.749  0.363  0.396  0.908  0.233  0.683  0.766  0.256  0.815  0.628  0.868  0.711  0.379  0.377  0.14  0.09  0.221  0.117  0.153  0.109  0.593  0.914  0.376  0.545  0.811  0.483  0.643  0.585  0.607  0.258  0.387  1.24  0.589  0.739  0.213  0.996  0.855  0.236  1.021  0.598  0.37  0.791  0.186  0.681  0.674  0.456  0.883  0.458  0.617  0.417  0.839  0.535  1.373  0.628  1.144  1.007  
 
 CYP20A1  57404  2  204103163  204170563  2q33.2  0.503  1.473  0.585  0.442  0.61  0.878  0.921  1.008  1.187  0.97  0.691  0.539  0.634  0.513  0.791  0.481  0.534  0.927  1.481  0.715  0.599  1.218  0.809  0.399  0.784  1.089  0.626  0.567  0.872  0.386  1.036  0.933  0.913  0.721  0.606  0.809  0.728  1.029  0.661  0.917  0.787  0.684  0.893  0.486  0.428  0.615  0.689  0.629  0.907  1.327  0.916  0.433  0.512  0.419  0.859  0.449  1.247  0.752  0.803  0.815  
 
 ABI2  10152  2  204192961  204296896  2q33  2.334  3.394  1.585  1.394  1.907  2.484  3.344  2.905  3.086  2.652  3.018  1.34  1.808  3.071  2.794  2.483  2.506  2.78  3.973  0.904  1.076  2.525  0.855  0.64  2.67  2.325  1.69  2.23  2.47  1.714  2.787  2.602  2.561  1.431  1.822  2.756  2.926  2.1  2.386  3.107  2.102  1.715  4.226  2.087  2.259  1.924  1.805  2.187  2.787  3.101  3.024  1.44  1.995  1.555  2.849  1.919  2.452  2.556  2.371  2.761  
 
 RAPH1  65059  2  204298404  204400058  2q33  2.533  4.437  1.802  2.242  1.929  2.812  3.573  3.728  2.295  2.669  2.9  2.314  1.514  2.024  2.279  1.928  2.429  1.015  0.249  0.114  0.141  0.482  0.681  0.845  2.891  2.662  2.189  3.429  2.164  1.961  2.089  2.699  3.45  1.497  1.657  2.844  4.339  3.595  3.169  2.707  2.55  1.728  3.522  2.943  1.232  2.201  2.005  2.914  2.544  2.287  2.573  1.719  3.068  1.863  2.571  1.838  3.105  2.524  3.143  2.339  
 
 CD28  940  2  204571197  204603636  2q33  0  0  0.134  0  0  0  0.042  0.017  0.115  0.015  0  0  0  0.014  0  0  0  0.023  1.519  0.045  0  0.819  3.388  2.99  0  0  0.034  0.038  0  0.03  0  0  0  0.266  0  0  0.025  0  0.017  0  0  0.095  0.024  0  0  0.02  0.04  0  0  0  0  0.201  0.015  0.027  0.025  0  0  0  0  0  
 
 CTLA4  1493  2  204732510  204738683  2q33  0  0.067  0.152  0  0  0  0  0  0.101  0  0  0  0  0  0  0  0  0.112  0  0  0  0.046  0.315  0  0  0.168  0.33  0.27  0.526  0.112  1.032  0.33  0  0.257  0  0  0  0  0  0  0  0.186  0  0  0  0  0  0  0  0  0  0.136  0  0  0  0  0  0  0  0  
 
 ICOS  29851  2  204801470  204826298  2q33  0  0.059  0.155  0  0.081  0.565  0.83  0.195  0.092  0.076  0.188  1.025  0  0.144  0  0.082  0.068  0.082  0.94  0.36  0.105  1.635  0.183  0.17  0.082  0.044  1.961  0.447  0.35  1.521  0.475  0.904  0.19  0.242  0.129  0  0.033  0.114  0.196  0.059  0.159  1.163  0.303  0  0.67  0.936  2.015  0.148  0  0  0.064  0.277  0.058  0.279  0.107  0.162  0.094  0.373  0  0  
 
 PARD3B  117583  2  205410515  206484886  2q33.3  0.34  0.511  0.723  0.375  0.588  0.766  0.163  0.367  0.823  1.21  0.243  0.034  0.769  0.082  1.008  0.017  0.213  0.077  0.014  0.028  0.079  0.067  0.554  0.525  0  0.422  0.079  0.031  0.157  0.065  0.213  0.15  0.039  0.248  0.284  0.507  0.581  0.585  0.344  0.043  0.782  0.169  0.033  1.238  1.304  0.967  0.13  0.092  0.974  0.096  0.039  0.491  0.044  0.358  0.253  0.351  0.696  0.273  0.179  0.762  
 
 NRP2  8828  2  206547223  206662857  2q33.3  0  0.107  2.32  0.773  0.045  0.922  3.68  4.845  4.375  2.99  4.425  1.067  1.174  0.115  0.541  0.111  1.351  0.655  0.032  0.152  0.073  0.566  1.244  1.643  4.962  3.521  3.986  4.474  4.816  3.475  4.221  4.51  4.888  3.044  2.003  0.879  1.387  3.268  1.511  0.102  2.167  2.046  0.23  1.594  1.514  0.604  1.605  0.16  2.791  0.295  1.751  0.551  3.657  3.591  2.984  1.931  4.252  2.686  1.11  3.658  
 
 INO80D  54891  2  206858444  206950906  2q33.3  1.564  1.004  1.128  0.898  1.328  1.453  1.884  1.634  1.039  1.205  1.11  2.021  1.594  1.55  1.647  1.505  1.752  0.97  1.195  1.897  0.946  2.003  2.202  1.063  1.319  1.465  2.57  1.538  1.856  2.349  1.643  1.819  1.735  1.421  1.331  1.259  1.274  1.503  1.175  1.736  1.756  2.08  1.826  1.331  1.483  1.851  2.87  0.957  1.406  0.528  1.26  1.345  1.392  1.302  1.649  1.098  1.971  1.318  1.891  1.246  
 
 GCSHP3  100329109  2  206980296  206981296  -  1.602  1.893  0.748  1.164  1.132  0.587  1.979  1.53  1.869  1.576  1.269  1.724  1.848  2.411  1.617  0.839  2.055  1.834  2.864  2.889  0.781  1.219  0.705  0.708  1.116  1.916  1.695  0.857  1.167  1.333  1.661  1.837  1.169  1.385  1.198  1.804  0.822  1.61  0.913  1.352  1.065  1.669  0.791  0.868  0.702  1.269  0.734  0.534  1.038  1.794  1.9  0.788  1.176  1.044  1.313  0.717  1.537  1.552  0.999  1.319  
 
 NDUFS1  4719  2  206987802  207024243  2q33-q34  3.056  3.598  2.124  2.03  3.112  2.195  2.827  3.034  2.556  2.498  2.983  2.582  2.545  3.489  2.852  3.134  3.344  4.956  4.383  3.191  2.767  2.701  1.851  2.053  2.937  3.243  2.738  2.682  3.439  3.017  4.036  3.869  3.518  2.405  2.16  3.202  3.047  2.646  2.179  3.462  2.875  2.397  2.893  2.582  2.07  2.603  2.156  1.576  2.463  3.21  3.187  1.72  2.625  1.994  3.193  1.997  3.274  2.553  3.234  3.189  
 
 EEF1B2  1933  2  207024317  207027653  2q33.3  7.955  9.809  5.337  4.734  7.053  6.685  7.919  6.863  5.914  7.014  6.676  6.881  7.599  9.214  6.602  7.449  8.224  9.862  9.288  10.214  7.14  8.303  6.77  7.175  7.629  5.972  5.928  5.855  6.625  6.495  7.16  7.29  6.464  4.788  6.713  6.763  6.977  6.392  7.531  6.941  6.736  6.441  8.152  7.032  6.807  6.222  8.468  5.976  6.121  8.62  6.688  6.061  6.291  5.616  8.281  6.846  6.255  7.699  7.61  7.381  
 
 SNORA41  619569  2  207026951  207027083  2q33  0  2.624  0  0  0  0  0  0  0  0  0  0  0  0  0  2.418  0  0  3.263  0  0  1.919  0  0  0  0  1.28  0  0  1.147  2.571  0  0  0  2.482  0  0  0  0  0  0  0  0  0  0  3.144  1.29  0  0  0  0  4.423  0  0  0  0  0  0  0  0  
 
 GPR1  2825  2  207040041  207082771  2q33.3  0  3.268  2.403  1.335  0  1.966  1.104  0.81  2.049  0.278  1.019  0.078  0  0.027  0  0.05  0  1.045  0  0.074  0  0.144  0.238  0  0.462  0  0.179  0  0  0.229  0.154  0.173  0  0.192  0  0  0.782  0.731  1.655  0  0.126  0.369  0.218  0  0  0.382  0.628  0.291  0.417  0.838  0.854  0.12  0.627  1.857  0.078  0  0.71  0  0  0.851  
 
 GPR1-AS1  101669764  2  207068099  207130967  2q33.3  0.337  0.042  0.264  0  0.141  0.029  0.086  0  0.123  0.028  0.036  0.034  0  0.069  0  0.241  0.03  0.133  0.041  0.076  0  0.048  0.385  0.157  0.108  0  0.081  0.085  0.109  0.073  0  0  0  0.205  0  0  0  0  0.271  0  0  0.132  0.039  0  0.052  0.022  0.839  0.046  0.04  0.024  0  0.184  0  0.062  0.179  0.139  0.045  0  0  0  
 
 ZDBF2  57683  2  207139364  207179150  2q33.3  0.257  0.398  0.187  0  0.122  0.612  0.318  0.986  0.734  0.255  0.596  0.01  0.059  0.557  0  0.017  0.013  0.506  0.286  0.052  0  0.199  0.252  0.058  0.462  0.027  0.205  0.095  0.694  0.221  0.534  0.433  0.208  0.29  0.197  0.082  0.923  0.053  0.562  1.054  0.612  0.388  0.175  0  0.278  0.012  0.021  0.255  0.522  0.252  0.803  0.639  0.13  0.55  0.769  0.143  0.396  0.314  0.624  0.93  
 
 ADAM23  8745  2  207308367  207485854  2q33  0  0  0.394  0  0.02  0.348  0.044  0.26  0.364  2.459  0.608  0.034  0  0.041  0.128  0.028  0.025  0.04  0.017  0.036  0  0.106  1.822  2.477  0.709  0.095  0.162  0.071  2.271  1.494  1.406  2.174  0.896  0.44  0.496  0  0.275  0  0.312  0.753  2.025  1.251  0.541  0  0.033  0.018  0.062  0.134  0.152  0.393  0.145  0.452  0.015  0.467  0.062  0  0  0.492  0  0.159  
 
 LOC200726  200726  2  207507141  207514173  2q33.3  0  0  0  0  0  0  0  0  0  0.047  0  0  0  0  0  0  0  0  0  0  0  0.069  0.451  0  0  0  0.036  0  0  0.031  0  0.132  0  0.153  0  0  0  0  0  0  0  0  0  0  0  0  0  0  0  0  0  0  0  0  0  0  0  0  0  0  
 
 DYTN  391475  2  207516344  207583120  2q33.3  0  0  0.062  0  0  0  0  0  0  0  0  0  0  0.043  0  0  0  0  0  0  0  0  0.194  0  0  0  0.042  0  0  0.031  0  0  0  0  0  0  0  0  0.028  0  0  0  0.026  0  0  0  0.024  0  0  0  0  0.101  0  0.037  0  0  0  0  0  0  
 
 MDH1B  130752  2  207602488  207630273  2q33.3  0.332  0.08  0.071  0  0.352  0.073  0.278  0.156  0.163  0.084  0.132  0.155  0  0.851  0.1  0  0.145  0.134  0.18  0  0  0.313  0.199  0.051  0.207  0.097  0.247  0.054  0.206  0.291  0.149  0.181  0.187  0.261  0.258  0.171  0.202  0  0  0.607  0.19  0.3  0.02  0  0.198  0.058  0.02  0  0  0.243  0.091  0.373  0.191  0.102  0.277  0.093  0.108  0.2  0.124  0.187  
 
 FASTKD2  22868  2  207630111  207660911  2q33.3  1.607  2.019  0.733  1.045  1.328  0.668  1.811  1.288  1.055  1.095  1.299  1.067  1.921  2.152  1.048  1.632  1.295  2.296  2.533  2.033  1.191  1.522  0.949  0.96  1.835  2.149  1.32  1.265  1.839  1.929  2.149  1.84  1.809  1.104  1.134  1.747  1.401  1.303  1.27  1.61  1.423  1.089  1.818  1.267  1.095  1.019  1.229  0.493  0.868  1.48  1.647  0.928  1.296  1  2.261  1.024  1.906  1.107  1.752  1.716  
 
 CPO  130749  2  207804277  207834198  2q33.3  0  0  0  0  0  0  0  0  0  0  0  0.082  0  0.223  0  0  0  0.09  0  0  0  0  0  0  0  0  0.034  0  0  0.048  0  0  0  0.203  0  0  0  0.234  0  0  0  0  0  0  0  0  0  0  0.142  0  0  0  0  0  0  0  0  0  0  0  
 
 KLF7  8609  2  207938861  208031970  2q32  0.062  0.372  2.104  1.607  0.665  1.267  2.001  2.99  1.651  1.928  1.68  0.063  0.941  1.159  0.702  0.029  0.105  0.088  0.518  1.196  0.036  1.126  1.033  0.81  2.491  0.946  0.207  1.158  0.49  1.273  0.633  0.252  0.89  0.559  1.526  1.409  1.119  0.977  2.494  0.767  1.465  0.907  1.038  0.023  0.745  1.148  0.599  0.864  2.239  0.999  0.859  0.652  1.447  1.957  2.094  1.09  3.748  1.955  1.918  1.353  
 
 LOC101927865  101927865  2  208051413  208119040  2q33.3  0  0  0.496  0  0.115  0  0.303  0.838  0  0.127  0.61  0  0  0  0.264  0  0  0  0  0.203  0  0  0  0  0.543  0  0  0.15  0  0  0  0  0  0.483  0  0  0.628  0  1.033  0.376  0.717  1.315  0.698  0  0  0.225  0.234  0.274  0  0.53  0.4  0  0.22  0.235  1.268  0  0.978  0.625  0.774  0  
 
 CREB1  1385  2  208394615  208470284  2q34  1.418  1.983  1.067  0.719  1.197  0.934  1.729  1.43  1.461  1.481  1.354  0.776  1.097  1.731  1.328  1.268  1.19  1.578  2.604  1.658  0.867  1.89  0.878  0.712  1.221  1.355  1.105  0.994  0.92  0.763  1.368  1.305  1.17  0.843  0.979  1.347  1.439  1.285  1.236  1.292  1.387  1.23  1.416  1.514  0.873  1.189  1.375  0.674  1.446  1.341  1.477  0.649  1.4  0.816  1.421  1.125  1.963  1.234  1.64  1.672  
 
 METTL21A  151194  2  208445354  208490055  2q33.3  1.08  1.227  0.944  1.564  0.762  0.869  1.115  1.149  1.511  1.105  0.915  0.93  1.218  1.899  1.04  1.242  1.565  2.444  3.135  1.907  1.047  1.886  1.349  1.074  1.382  1.051  1.001  0.97  1.277  0.764  1.473  1.072  1.562  1.283  1.482  1.564  1.101  1.131  0.642  1.465  1.853  2.006  1.832  1.32  0.522  1.507  1.122  0.494  0.838  0.916  1.094  1.371  1.164  1.578  2.615  1.072  1.549  2.323  1.806  1.212  
 
 CCNYL1  151195  2  208576263  208620896  2q33.3  0.786  1.728  1.841  1.075  1.173  1.223  1.755  1.469  1.513  1.48  1.577  1.068  1.401  1.762  1.115  1.504  1.25  1.847  1.989  1.452  0.55  1.227  0.967  0.636  1.788  1.509  1.105  1.68  1.658  0.84  1.663  1.652  1.684  1.193  2.243  2.065  2.132  1.577  2.242  2.096  3.353  2.105  2.109  1.528  1.887  2.436  2.39  0.321  1.565  1.05  2.132  1.337  2.254  1.907  2.998  1.812  2.71  1.653  3.186  2.611  
 
 FZD5  7855  2  208627309  208634143  2q33.3  0.686  0.53  0.352  0.309  0.528  0.337  0.638  0.702  1.335  1.16  0.75  2.433  2.195  0.97  1.244  2.491  1.861  1.466  0.206  0.283  1.128  0.486  0.593  0.381  0.298  0.582  0.798  0.772  0.87  1.049  0.595  1.505  1.066  0.667  1.08  1.585  0.772  0.338  0.418  0.617  0.88  1.715  1.986  1.602  0.993  1.764  0.804  0.333  0.843  0.319  0.705  1.01  1.369  1.191  1.132  0.757  1.276  0.568  1.504  0.827  
 
 PLEKHM3  389072  2  208686011  208890284  2q33.3  0.523  0.295  0.536  0.337  0.285  0.352  0.838  0.989  0.979  1.056  0.476  0.483  0.553  0.811  0.681  0.774  0.549  0.336  0.741  0.529  0.155  0.905  0.558  0.388  0.677  1.062  0.693  0.756  0.909  0.493  0.666  0.983  0.782  0.573  0.447  0.601  0.63  0.61  0.452  0.773  0.507  0.524  1.143  0.703  0.505  0.532  0.235  0.227  0.463  0.195  0.725  0.535  0.469  0.595  0.644  0.331  0.976  0.544  0.675  0.731  
 
 LOC100507443  100507443  2  208983852  209021486  2q33-q35  0  0  0  0  0  0  0  0  0  0  0  0  0  0  0  0  0  0  0  0  0  0  0  0  0  0  0.208  0  0  0.135  0  0  0  0  0  0  0  0  0  0  0  0  0  0  0  0  0.198  0  0  0  0  0  0  0  0  0  0  0  0  0  
 
 CRYGD  1421  2  208986330  208989313  2q33.3  0  0  0  0  0.094  0  0  0  0  0  0  0.328  0  0  0  0  0  0.236  0  0  0  0.365  0  0  0  0  0.186  0  0  0.162  0  0  0  0.591  0  0  0  0  0  0  0  0.709  0.066  0  0  0  0.179  0  0  0  0  0.705  0  0  0  0  0  0  0  0  
 
 CRYGC  1420  2  208992860  208994554  2q33.3  0  0  0  0  0  0  0  0  0  0  0.162  0  0  0  0  0  0  0  0  0  0  0  0  0  0  0  0.073  0  0  0.067  0  0  0  0  0  0  0  0  0  0  0  0  0  0  0  0  0  0  0  0  0  0  0  0  0  0  0  0  0  0  
 
 CRYGB  1419  2  209007296  209010877  2q34  0  0  0  0  0  0  0  0  0  0  0  0  0  0  0  0  0  0  0  0  0  0.136  0  0  0  0  0  0  0  0.054  0  0  0  0  0  0  0  0  0  0  0  0  0  0  0  0  0  0  0  0  0  0.316  0  0  0  0  0  0  0  0  
 
 CRYGA  1418  2  209025463  209028297  2q34  0  0  0  0  0  0  0  0  0  0  0  0  0  0  0  0  0  0  0  0  0  0.115  0  0  0  0  0.091  0  0  0.108  0  0  0  0.478  0  0  0  0  0  0  0  0.429  0.105  0  0  0  0  0  0  0  0  0  0  0  0.178  0  0  0  0  0  
 
 C2orf80  389073  2  209030070  209054773  2q34  0  0  0  0  0  0  0  0  0  0  0  0  0  0  0  0  0  0.107  0  0  0  0.047  0  0  0  0  0.056  0  0  0.029  0  0  0  0  0  0  0  0  0.069  0  0  0.195  0.035  0  0  0  0.052  0  0  0  0  0  0  0  0  0  0  0  0  0  
 
 IDH1  3417  2  209100950  209119867  2q33.3  2.821  3.925  2.836  3.211  2.499  1.613  4.504  3.451  3.563  3.456  3.707  4.569  5.159  4.503  3.634  3.241  4.358  4.431  3.803  3.331  2.445  2.66  2.669  1.231  3.344  3.905  3.703  3.362  4.236  5.036  4.837  4.45  3.326  2.758  3.036  4.001  3.556  3.508  2.652  3.492  4.072  3.888  4.238  3.62  3.07  3.907  2.682  1.135  2.227  2.915  2.94  2.048  2.843  3.717  3.724  2.07  3.596  3.015  3.333  4.116  
 
 IDH1-AS1  100507475  2  209119956  209120918  -  0.694  1.285  0.423  1.003  1.706  0.631  0.7  0.377  0.693  0.349  0  0  0.339  0.792  0  0.356  0.876  1.012  1.461  0.545  0  1.118  1.08  0.414  0.554  0.433  0.123  0  0.333  0.155  1.429  1.114  0.356  0.831  1.203  0.675  0  0  0.468  0.469  0.403  0.748  1.006  0.936  0  0.983  0  0  0  0.586  0.382  0.774  0.402  0.232  1.519  0.48  0  0  0.585  0.492  
 
 PIKFYVE  200576  2  209130990  209223475  2q34  1.227  1.84  0.846  0.925  0.911  1.347  1.575  1.481  1.147  1.366  1.204  0.618  1.41  1.875  1.618  1.048  1.055  1.852  2.988  1.659  1.292  2.082  0.714  0.865  1.679  1.843  1.255  1.73  2.106  1.793  1.915  1.743  1.63  0.911  0.826  1.537  1.624  1.525  0.932  1.859  1.237  0.738  1.718  1.319  1.179  1.273  0.479  0.526  1.127  0.797  1.618  0.94  1.094  0.95  1.748  0.939  1.962  1.476  1.653  1.669  
 
 PTH2R  5746  2  209224568  209359231  2q33  0  0.057  0.13  0  0.043  0.054  0.043  0  0.106  0.042  0  0  0  0.02  0  0.035  0  0  0.084  0.78  0  0.129  0.242  0  0  0  0.054  0.074  0  0.066  0  0.042  0  0.212  0  0.064  0  0  0.076  0  0  0.063  0.129  0  0  0.964  0.119  0.305  0.102  0.698  0  0.258  0.035  0.787  0.1  0  0  0  0  0  
 
 MAP2  4133  2  210288770  210598834  2q34-q35  0.432  0.173  0.274  1.302  1.472  0.106  1.042  0.155  2.607  1.828  1.802  0.032  1.789  0.855  0.045  0.394  0  0.052  0.033  0.039  0  0.124  0.271  0.072  0.057  1.681  2.021  0.38  0.689  0.226  0.442  3.17  2.597  1.169  0.072  1.251  0.647  0.98  0.942  0.459  3.386  1.395  6.409  0.078  0.252  1.175  0.369  0  1.669  0.044  0.182  0.416  0.323  1.755  0.356  0.065  0.268  0.094  0.2  0.504  
 
 UNC80  285175  2  210636716  210864024  2q35  0  0.01  0.055  0  0.015  0.026  0.013  0.013  0.163  0.025  0.143  0.018  0  0.016  0  0.013  0.012  0.059  0.012  0.055  0.019  0.057  0.139  0.012  0  0.236  0.226  0.089  0.155  0.228  0.118  0.734  0.371  0.261  0.024  0.012  0.009  0.012  0.023  0.047  0.021  0.106  0.03  0  0.071  0.018  0.04  0  0.01  0.006  0.025  0.115  0.009  0.031  0.016  0.019  0.009  0.014  0  0  
 
 RPE  6120  2  210867288  210886984  2q32-q33.3  2.177  2.983  1.705  1.732  1.737  1.273  2.203  2.146  2.177  1.988  2.523  1.6  2.498  2.983  2.13  2.48  2.264  3.203  2.934  2.465  1.822  1.416  1.551  1.37  2.593  2.246  1.269  2.03  2.601  1.718  3.027  2.951  2.815  1.985  2.003  2.226  2.191  1.769  2.02  2.596  2.274  1.768  2.413  1.955  1.652  1.923  1.947  0.757  2.053  2.144  2.405  1.003  2.009  1.688  2.257  1.511  2.741  2.338  2.892  2.519  
 
 KANSL1L  151050  2  210886144  211036094  2q34  0.834  1.132  0.386  0.403  0.485  0.183  0.442  0.749  0.664  0.723  0.746  0.446  0.777  1.196  0.762  0.847  0.709  0.891  0.898  0.709  0.3  0.732  0.503  0.581  0.647  0.851  0.694  0.701  0.557  0.263  0.855  0.68  0.958  0.668  0.23  0.663  0.596  0.76  0.459  0.759  0.613  0.55  0.589  0.458  0.386  0.706  0.384  0.231  0.707  0.36  0.516  0.322  0.393  0.602  1.061  0.37  0.749  0.531  1.093  0.759  
 
 LOC101928020  101928020  2  210894645  210929080  2q34  0.231  0.476  0.104  0  0.2  0  0.149  0.317  0.237  0.335  0.389  0.099  0.36  0.551  0.246  0.388  0.148  0.387  0.754  0.89  0.283  0.488  0.34  0.408  0.212  0.554  0.713  0.398  0.789  0.108  0.495  0.445  0.716  0.241  0.381  0.173  0.272  0.48  0.178  0.147  0.413  0.049  0.488  0.281  0.329  0.218  0.892  0.136  0.318  0.214  0.138  0.194  0.232  0.247  0.581  0.145  0.257  0.234  0.465  0.134  
 
 ACADL  33  2  211052715  211090215  2q34  0.073  0  0  0  0  0  0  0.096  0.088  0  0.12  0  0.068  0.027  0  0  0  0.242  0  0  0  0.085  0  0  0  0  0.043  0  0  0.033  0  0.043  0  0.153  0.141  0.318  0  0  0.031  0  0.29  0.241  0.021  0  0.112  0.45  0.025  0  0.063  0.222  0  0.359  0  0.148  0.177  0  0  0  0  0  
 
 MYL1  4632  2  211154867  211179895  2q33-q34  0  0.123  0  0  0  0.205  0  0.094  0  0  0  0  0  0  0  0  0  0  0  0  0  0  0  0  0  0  0.129  0.188  0  0.069  0  0  0  0.235  0  0  0  0  0  0  0  0  0.099  0  0  0  0  0  0  0  0  0.362  0  0  0  0  0  0  0  0  
 
 LANCL1-AS1  102724820  2  211189435  211335057  2q34  0  0.053  0.092  0  0.035  0  0.122  0.069  0.087  0.145  0.104  0  0  0.153  0.139  0.054  0  0  0.11  0.179  0  0.148  0.313  0.097  0  0.11  0.093  0.096  0.166  0.051  0  0.076  0.076  0.203  0.066  0.204  0.059  0.201  0.041  0.113  0  0.048  0.274  0  0  0  0.028  0  0  0  0.077  0  0.064  0.04  0.193  0  0.106  0.114  0.122  0  
 
 LANCL1  10314  2  211295972  211341499  2q33-q35  2.042  2.148  1.804  0.754  2.297  1.561  2.221  1.908  2.442  1.81  1.858  1.386  1.962  3.195  1.929  1.263  2.136  2.097  3.9  2.429  1.582  2.15  1.333  0.975  2.781  2.618  1.902  2.224  2.467  1.211  2.419  2.164  2.705  2.097  1.921  3.124  1.924  2.379  1.569  3.347  1.448  2.261  2.643  1.495  1.598  1.394  1.317  0.633  1.986  1.676  2.353  1.18  1.976  1.26  2.704  1.657  1.835  2.184  2.391  2.706  
 
 CPS1  1373  2  211342405  211543832  2q35  0.321  0.485  0.908  0  0.049  0.513  0.851  0.7  1.031  0.136  0.714  0  0.064  2.006  0.065  0  0  0.079  0.531  0.996  0.256  0.078  0.187  0  1.027  1.216  0.792  1.666  1.686  0.043  1.094  0.452  1.524  0.77  2.17  3.536  0.038  1.04  0.03  4.995  0.053  3.972  0.059  0.042  0.286  0.043  0.026  0.135  0.452  0.106  0.562  0.115  1.256  0.16  2.003  0.156  1.145  0.279  0.433  1.136  
 
 CPS1-IT1  29034  2  211482294  211484599  2q34  0  0  0.121  0  0.031  0  0  0.031  0  0  0  0  0  0.096  0  0  0  0  0  0  0  0.042  0.124  0  0  0.06  0.053  0.033  0  0.031  0  0  0  0.15  0.112  0.099  0  0  0  0.155  0  0.493  0  0  0  0  0  0  0  0  0  0.087  0  0  0  0  0  0  0  0  
 
 ERBB4  2066  2  212240441  213403352  2q33.3-q34  1.311  0  0.08  0.025  0.733  0.029  0.024  0.013  0.068  0.164  0.031  0.046  0.074  0.018  0  0.009  0.013  0.054  0.006  0.043  0.04  0.034  0.127  0.035  0  0  0.12  0.054  0  0.142  0.017  0.183  0.014  0.169  0.018  0.261  0.02  0  0.014  0  0.034  0.078  3.191  1.861  0.913  1.254  0.138  0  0.446  0.007  0.029  0.099  0.01  0.047  0.012  0.109  0  0.019  0.073  0.035  
 
 LOC102725079  102725079  2  213660054  213683987  -  0  0  0  0  0  0  0  0  0  0  0  0  0  0  0  0  0  0  0  0  0  0.153  0  0  0  0  0  0  0  0  0  0  0  0  0  0  0  0  0  0  0  0  0  0  0  0  0  0  0  0  0  0  0  0  0  0  0  0  0  0  
 
 IKZF2  22807  2  213864410  214016333  2q34  0.201  0.29  0.154  0.274  0.411  0.101  0.323  0.13  0.176  0.107  0.061  0.104  0.095  0.131  0.019  0.917  0.32  0.066  2.507  0.422  0.061  1.86  0.117  0.055  0.198  0  0.06  0.017  0.015  0.046  0.017  0.042  0  0.188  0.013  0.242  0.122  0.314  0.04  0.058  1.274  0.245  0.622  0.198  0.704  0.677  0.135  0.036  0.28  0.097  0.224  0.109  0.009  0.051  0.034  0.154  0.02  0.184  0.044  0.204  
 
 LOC100130451  100130451  2  214141276  214148929  2q34  0  0  0.077  0  0.067  0  0  0  0.148  0.043  0  0  0  0  0  0  0  0  0  0  0  0.044  0  0  0  0  0  0  0  0.027  0  0  0  0.166  0  0  0  0  0  0  0  0.052  0  0  0  0  0  0  0  0  0  0  0.054  0  0  0  0  0  0  0  
 
 SPAG16  79582  2  214149102  215275225  2q34  0  3.456  0.779  1.049  2.657  1.642  1.911  1.776  1.974  1.614  1.623  0.772  0.516  0.077  0  2.439  0  3.123  2.876  0.813  0  1.431  1.201  0.17  1.538  1.047  0.123  1.18  1.74  0.801  1.19  1.883  0  0.194  1.449  1.588  1.527  1.461  0.032  0.835  2.306  1.656  2.182  1.053  0.886  1.084  2.099  0.657  1.015  1.988  1.541  0.829  1.24  1.189  2.144  1.307  1.114  1.556  1.985  1.923  
 
 VWC2L  402117  2  215276460  215440653  2q34-q35  0.235  0  0  0  0  0  0  0  0  0  0  0  0  0  0  0  0  0.151  0  0  0  0.132  0.465  0.248  0  0  0  0  0  0  0  0  0  0.362  0  0  0  0  0  0  0  0.226  0  0  0  0  0  0  0  0  0  0  0  0  0.077  0  0  0  0  0  
 
 BARD1  580  2  215590369  215674435  2q34-q35  2.663  3.1  1.276  1.345  2.199  1.555  2.349  1.817  1.224  2.212  2.516  1.017  1.782  2.275  1.957  1.929  1.892  2.181  3.416  2.466  0.889  2.516  1.15  0.903  1.776  1.463  1.468  1.509  1.259  1.099  1.701  1.38  1.742  1.066  1.26  1.421  1.371  1.518  1.281  1.939  2.346  1.365  2.642  1.344  1.018  1.886  3.085  0.776  1.799  1.333  1.573  1.069  1.579  0.981  1.865  0.994  1.413  1.555  2.306  1.878  
 
 LOC101928103  101928103  2  215674952  215828329  2q35  0.42  0.491  0.115  0.255  0.409  0.095  0  0.261  0.219  0.28  0.449  0  0.08  0.401  0.103  0  0.112  0.187  0.599  0.105  0  0.097  0.413  0.167  0.478  0  0.088  0.038  0  0.034  0.134  0  0.103  0.282  0  0.18  0.166  0.16  0.081  0.409  0.455  0.077  0.637  0.073  0.19  0.235  0.15  0  0.275  0.099  0.133  0.187  0.097  0.246  0.161  0.07  0.344  0  0.128  0.135  
 
 ABCA12  26154  2  215796265  216003151  2q34  1.43  0.041  0.044  0.035  1.284  0  0.046  0.038  0.178  0.091  0.076  1.017  0  0.16  0.043  0.038  0.077  0.048  0.041  0.026  0  0.046  0.097  0  0  0.022  0.082  0.014  0.138  0.046  0.018  0.014  0  0.152  0.032  0.053  0.024  0.057  0.056  0.067  0.221  0.116  0.08  0.046  0.049  0.086  0.027  0.035  0  0  0.016  0.125  0.363  0.597  0.293  0.378  1.353  0  0.229  0.178  
 
 ATIC  471  2  216176678  216214496  2q35  5.155  4.674  3.095  2.798  4.821  3.455  4.788  4.084  2.372  3.458  4.79  4.322  4.926  4.867  4.226  4.52  4.564  4.453  6.497  5.177  4.558  5.273  3.102  3.431  4.865  4.046  4.661  4.615  5.133  4.55  5.257  5.673  5.013  3.692  4.066  4.56  4.869  3.726  3.8  5.192  5.006  3.388  5.909  4.009  3.109  3.679  5.307  3.743  3.281  5.853  3.963  3.315  4.099  2.941  5.101  3.919  2.863  4.07  5.241  4.647  
 
 FN1  2335  2  216225162  216300793  2q34  0.734  3.52  9.183  4.481  1.253  6.347  4.951  7.915  2.954  8.399  3.997  0.092  0.195  0.973  0.188  0.41  0.383  0.261  1.292  0.187  0.968  0.583  5.904  7.797  7.404  6.525  6.964  6.582  8.989  3.738  7.5  5.873  2.982  2.87  2.719  4.712  5.406  6.737  3.25  1.046  1.646  5.056  0.159  1.2  1.313  1.206  2.71  6.844  2.104  8.628  4.231  0.893  7.823  1.452  7.384  8.782  8.877  0.76  9.153  7.218  
 
 LOC102724849  102724849  2  216318429  216328591  2q35  0  0  0  0  0  0.259  0  0  0  0  0  0  0  0  0  0  0  0  0  0  0  0.119  0  0  0  0  0  0  0  0  0  0  0  0  0  0  0  0  0  0  0  0  0  0  0  0  0  0  0  0  0  0  0  0  0  0.173  0  0  0  0  
 
 LINC00607  646324  2  216476285  216708259  2q35  0.049  0  0.16  0  0.118  0  0  0.04  0.107  0.065  0  0  0  0.016  0  0  0  0.027  0  0  0  0.059  0.275  0.071  0  0.077  0.186  0.034  0.042  0.06  0  0  0  0.268  0  0.044  0  0.111  0.032  0  0  0.107  0.029  0  0  0  0.053  0  0  0.02  0.027  0.157  0.135  0.055  0.052  0.046  0.079  0  0  0  
 
 MREG  55686  2  216807313  216878346  2q35  2.089  1.129  0.267  0.211  2.173  0.44  0.541  0.256  0.993  0.499  1.098  1.102  1.025  1.485  1.011  0.958  1.118  1.847  2.154  0.374  0.343  1.132  1.092  0.718  0.796  2.222  2.073  0.848  2.933  1.217  2.788  2.378  2.356  2.242  0.558  1.336  0.518  0.612  0.681  0.763  1.911  0.565  0.783  0.436  0.794  0.785  0.558  0.515  0.788  1.75  0.802  0.734  0.345  0.096  0.382  0.217  0.619  0.312  1.066  0.142  
 
 PECR  55825  2  216903110  216946539  2q35  1.633  1.553  0.899  0.684  1.756  0.827  1.697  0.773  1.726  1.475  1.827  1.182  0.831  1.624  1.204  1.82  1.402  1.925  2.147  0.116  0.511  0.576  1.037  0.12  0.885  2.093  1.125  0.998  2.002  1.397  2.401  2.126  1.897  1.535  1.976  1.524  1.185  1.699  0.149  1.608  1.201  1.346  2.332  1.703  1.114  1.408  2.086  0.748  1.387  1.765  0.968  1.004  1.436  1.011  1.908  1.723  2.198  0.759  1.989  1.606  
 
 TMEM169  92691  2  216946588  216967506  2q35  0.283  0.058  0.163  0.111  0.067  0  0.086  0.432  0.468  0.342  0.314  0.079  0  0.111  0  0.143  0.043  0.375  0.093  0  0  0.056  0.217  0  0  0.877  0.755  0.032  0.199  0.118  0.201  0.098  0.059  0.294  0.105  0.123  0.228  0.142  0.019  0.164  0.038  0.17  0.035  0.056  0.494  0.187  0.141  0  0.424  0.034  0  0.203  0.259  0.055  0.075  0.064  0.91  0  0.099  0.062  
 
 XRCC5  7520  2  216974019  217071016  2q35  4.562  5.843  3.537  3.652  4.844  4.191  5.259  4.979  4.323  4.647  5.969  4.407  4.885  6.163  4.6  5.2  4.977  6.504  6.909  5.52  4.5  5.593  3.12  3.188  5.185  4.681  3.814  4.575  4.81  4.055  5.401  5.191  4.883  3.812  4.527  4.874  4.722  4.388  4.225  5.121  4.977  4.339  5.656  5.065  4.063  4.33  4.977  3.884  4.615  6.127  4.888  3.622  4.432  3.584  5.48  4.142  5.487  4.84  5.093  5.288  
 
 PKI55  150967  2  217081611  217084915  2q35  1.54  0.228  0.417  0.233  0.847  0.636  2.201  0.881  0.797  1  1.83  0.337  0.494  2.322  0.925  0.444  0.503  0.637  1.655  0.713  0.224  0.734  0.305  0.393  0.904  0.778  0.909  0.861  0.519  0.113  1.081  0.63  1.393  0.598  1.513  1.904  0.705  1.715  0.609  0.952  0.723  0.858  1.41  1.695  0.408  1.488  2.2  1.56  1.343  1.572  0.912  0.804  0.611  0.732  0.918  0.252  1.574  0.35  1.709  1.531  
 
 MARCH4  57574  2  217122584  217236750  2q35  0.142  0.637  1.25  0.378  0.187  2.729  1.016  1.111  0.13  0.169  0.327  0.222  0.107  0.204  0.074  0.483  0.65  0.185  0.265  0.779  0  0.956  0.302  0.074  3.958  0.048  0.271  1.323  0.226  0.31  0.105  0.134  0.095  0.353  1.843  0.144  3.59  2.495  3.725  0.872  1.605  0.332  1.847  0.099  0.093  0.084  1.685  2.489  0.143  2.258  0.13  1.916  0.931  0.384  3.392  0.294  1.897  4.032  1.951  3.239  
 
 SMARCAL1  50485  2  217277136  217347774  2q35  2.001  1.691  1.525  1.354  1.489  0.974  2.122  1.831  1.459  1.887  1.86  1.008  1.859  1.989  1.82  1.526  1.581  1.284  3.019  1.61  1.892  1.821  0.658  0.742  1.901  2  1.78  1.754  2.291  1.428  2  2.381  2.427  1.326  1.231  1.752  2.051  2.072  1.04  2.734  1.871  1.211  1.721  1.639  1.155  1.566  1.422  1.632  1.582  2.731  1.774  1.387  1.575  1.371  2.662  1.616  2.729  1.785  2.423  2.298  
 
 RPL37A  6168  2  217363519  217366188  2q35  9.255  11.712  8.058  7.202  9.59  10.351  9.818  8.687  8.766  8.802  9.133  9.622  8.343  9.903  7.563  9.728  10.484  12.399  11.381  12.165  9.266  10.853  8.428  9.134  10.42  8.241  8.578  7.693  8.938  8.154  10.062  9.68  8.496  8.284  10.103  8.478  7.779  8.646  10.628  8.13  9.029  9.771  9.706  9.829  9.074  8.977  9.792  8.526  8.622  11.378  8.173  7.636  8.495  8.74  10.441  9.095  8.316  9.83  9.394  8.846  
 
 LINC01280  101928232  2  217454715  217471661  2q35  0  0  0.168  0  0  0  0.074  0  0.262  0.08  0.048  0  0  0.03  0  0  0  0.222  0  0  0  0.052  0.15  0  0  0  0.254  0  0  0.237  0  0  0  0.19  0  0  0  0  0.046  0  0  0  0.044  0  0  0  0.411  0  0  0  0  0.2  0  0  0  0  0  0  0  0  
 
 IGFBP2  3485  2  217498126  217529158  2q33-q34  4.433  0.076  0.319  0.502  6.476  0.94  3.277  1.724  9.092  8.38  7.094  5.5  0.116  5.185  3.391  0.444  6.269  0.074  6.106  1.763  0.203  4.974  0.462  0.108  0  0.511  0.92  2.312  1.133  5.253  4.396  5.024  0  0.842  3.718  0.494  0.873  0  2.043  4.987  4.807  6.441  5.246  4.149  2.683  6.213  1.831  0  0.438  0.192  0.514  1.709  0.157  0.183  0.259  0.095  0.4  0.368  0  0.361  
 
 IGFBP5  3488  2  217536827  217560272  2q33-q36  3.201  0.033  6.129  0.064  7.172  4.551  7.151  3.46  5.868  5.513  4.365  0.117  0  0.068  0  0.031  0.036  0.108  0.022  0.125  0.03  0.382  0.249  0.035  0.234  3.569  0.223  0.043  0.024  0.188  0.54  2.469  0  0.357  0.06  4.024  0.083  0  1.057  0.233  0.316  0.325  0.093  0.026  0.897  4.664  1.017  0  0.251  0.021  0.473  0.441  0.083  0.165  0.088  0.05  2.162  0.074  0.039  3.086  
 
 TNP1  7141  2  217724181  217724782  2q35-q36  0  0  0  0  0  0  0  0  0  0  0  0  0  0  0  0  0  0  0  0  0  0.27  0  0  0  0  0  0  0  0  0  0.719  0  0  0  0  0  0  0  0  0  0  0  0  0  0  0  0  0  0  0  0  0  0  0  0  0  0  0  0  
 
 DIRC3  729582  2  218148745  218621316  2q35  0  0.024  0.283  0  0.111  0.434  0.814  0.066  0.111  0.176  0.144  0  0  0.019  0  0  0  0.03  0.117  0  0.055  0.075  0.131  0  0  0.147  0.05  0.086  0.162  0.048  0  0  0  0.245  0  0.033  0  0  0.073  0  0.049  0  0.041  0  0  0.097  0.022  0  0  0  0.094  0.121  0.023  0.043  0.055  0  0.116  0  0  0  
 
 TNS1  7145  2  218664511  218808796  2q35-q36  0.85  0.026  2.502  0.038  0.309  0.975  0.617  1.295  0.174  3.042  1.758  0.036  0.128  0.717  0.192  0.116  0.05  0.048  0.037  0.088  0.291  0.113  0.274  0.16  2.531  3.663  0.254  0.639  4.572  0.523  3.535  0.952  0.065  0.332  0.454  0.393  0.362  2.063  1.124  0.21  0.206  0.722  0.109  0.332  1.431  2.195  0.287  2.216  0.111  1.665  0.228  0.415  1.518  1.522  2.809  0.081  3.599  1.637  2.806  1.976  
 
 RUFY4  285180  2  218899656  218955304  2q35  0.139  0.114  0.236  0  0.114  0.193  0.172  0.115  0.207  0.185  0.219  0.105  0.243  0.125  0.102  0.242  0.26  0.123  0.191  0.507  0  0.285  0.344  0.387  0.12  0.099  0.139  0.091  0.272  0.127  0.229  0.162  0.174  0.265  0.106  0.079  0.153  0.143  0.21  0.176  0.089  0.23  0.198  0  0.139  0.141  0.314  0.132  0.082  0.157  0.11  0.269  0.228  0.116  0.333  0.181  0.063  0.282  0.172  0  
 
 CXCR2P1  3580  2  218923877  218926013  2q35  0  0  0.126  0  0  0  0  0  0.15  0.05  0  0  0  0.026  0  0.061  0  0.07  0  0.217  0  0.097  0.295  0.073  0  0.092  0.076  0  0.137  0.063  0  0  0  0.274  0  0  0  0  0.048  0  0  0.172  0.05  0  0.108  0  0.077  0  0  0  0  0.441  0  0.066  0.055  0  0.051  0  0.058  0  
 
 CXCR2  3579  2  218990012  219001976  2q35  0  0  0.121  0  0  0  0.04  0  0.108  0.031  0.091  0  0  0.064  0  0  0  0.141  0.07  1.604  0  0.109  0.131  0  0  0  0.12  0  0  0.048  0  0  0  0.28  0  0  0  0  0.045  0  0.057  0.052  0.045  0  0  0  0.078  0  0  0  0  0.293  0  0  0.033  0  0  0  0  0  
 
 CXCR1  3577  2  219027567  219031716  2q35  0  0  0.133  0  0.045  0.06  0  0.032  0.189  0  0  1.243  0  0.024  0  0  0  0.083  0  0.048  0  0.125  0.275  0  0  0  0.099  0  0  0.063  0  0  0  0.366  0  0  0  0  0.032  0  0  0.184  0.054  0  0  0.044  0.058  0  0  0  0  0.29  0.029  0.081  0.026  0  0  0  0  0  
 
 ARPC2  10109  2  219081816  219119071  2q36.1  5.477  7.816  5.925  4.65  5.711  5.637  7.288  7.044  5.128  6.347  7.079  6.447  6.414  6.241  5.327  6.38  6.333  6.501  7.923  7.672  4.492  6.521  5.24  5.18  7.033  5.971  6.383  5.771  6.509  4.997  6.371  6.871  6.738  4.749  5.378  5.785  6.394  6.04  6.307  6.469  6.358  5.308  6.55  5.804  5.525  6.161  7.054  5.758  5.968  7.622  5.948  4.868  6.453  5.313  7.12  6.367  6.101  6.362  7.367  6.75  
 
 GPBAR1  151306  2  219125737  219128582  2q35  0  0  0.259  0.237  0.163  0  0.15  0.154  0.33  0.161  0.263  0.12  0.219  0.085  0.151  0.099  0  0.1  0.205  0.145  0  0.309  0.378  0.11  0.111  0.102  0.46  1.752  0.205  0.147  0.113  0.172  0.228  0.289  0  0  0  0.137  0.054  0.396  0.235  0.291  0.055  0.23  0.211  0.243  0.047  0.138  0  0.271  0.079  0.793  0.084  0.161  0.166  0.15  0.153  0.108  0.134  0  
 
 AAMP  14  2  219128851  219134932  2q35  4.791  2.645  3.48  3.204  4.131  3.275  4.61  4.178  4.179  3.705  4.789  4.019  4.042  5.072  4.084  4.589  4.198  1.689  5.801  3.688  3.883  3.867  2.908  3.184  4.238  4.147  3.987  4.696  4.89  3.579  4.76  5.35  5.193  3.472  3.79  3.838  4.394  4.461  3.4  5.203  4.373  4.171  4.244  4.025  4.229  4.527  3.125  3.785  3.315  5.771  3.968  2.776  3.65  3.283  4.151  3.278  3.173  4.559  4.518  4.06  
 
 PNKD  25953  2  219135114  219211516  2q35  4.764  2.511  3.758  4.203  5.122  4.857  5.926  4.964  4.553  4.721  5.135  4.804  4.245  5.406  4.734  4.918  5.984  1.376  6.594  6.093  4.013  5.396  3.764  4.132  4.735  4.979  4.941  4.251  5.887  4.488  5.385  5.361  5.617  4.447  4.536  5.129  4.313  4.688  4.191  5.273  4.849  5.016  6.002  4.685  4.993  5.788  5.687  4.451  3.644  5.915  3.972  3.252  4.358  4.093  5.091  3.963  4.011  4.137  5.203  3.825  
 
 TMBIM1  64114  2  219138916  219157280  2q35  0.412  2.211  3.931  3.85  3.471  3.244  4.453  3.228  3.656  4.096  3.504  4.429  5.06  3.362  3.925  5.304  5.05  0.476  1.58  1.488  3.642  0.496  2.142  2  4.422  4.315  4.509  4.132  4.853  3.633  4.438  4.911  4.603  2.874  3.124  4.063  4.843  5.325  2.647  4.855  5.69  3.061  4.864  3.276  3.287  4.459  3.165  3.627  4.034  4.9  4.213  2.585  3.299  3.424  3.778  3.393  3.911  4.59  4.212  4.443  
 
 CATIP-AS2  103689911  2  219190963  219222689  2q35  0.188  0  0.206  0  0.088  0  0  0.088  0  0.058  0  0  0.146  0.133  0.421  0.121  0.119  0.146  0  0.141  0  0.068  0.284  0  0  0.269  0.096  0.253  0.291  0.048  0.137  0.144  0.214  0.289  0  0.503  0.244  0  0.048  0.724  0.254  0.692  0  0  0  0.526  0.046  0.249  0  0  0  0  0.084  0  0.211  0.145  0.252  0.201  0.514  0  
 
 CATIP  375307  2  219221578  219232817  2q35  0  0  0  0  0.078  0  0  0  0.114  0  0  0.07  0  0.062  0.135  0.077  0  0  0  0  0  0.288  0.286  0  0  0  0.164  0.056  0.085  0.132  0.101  0  0  0.384  0.097  0  0  0  0.086  0.237  0  0.698  0.051  0  0  0.063  0.035  0  0  0  0  0.554  0.143  0.178  0.159  0  0  0  0  0  
 
 CATIP-AS1  101928513  2  219231387  219232625  2q35  0  0.374  0  0  0.534  0  0  0  0  0  0  0  0  0.509  0  0  0.308  0  0  0  0  0  0.731  0  0  0  0.413  0.48  0.443  0.085  0.699  0  0.531  0.801  0  0.423  0  0  0  0  0  0.664  0  0  0  0.349  0.161  0  0  0  0  0.621  0  0.676  0.415  0  0  0  0  0  
 
 SLC11A1  6556  2  219246751  219261617  2q35  0.057  0  0.176  0  0.247  0.118  0.094  0.081  0.114  0.12  0.064  0.263  0.179  0.123  0.086  0.125  0.273  0.107  0.42  0.19  0.289  0.221  0.338  0.045  0.106  0.04  0.237  0.102  0.182  0.103  0.032  0.036  0.254  0.398  0.092  0.327  0.066  0.107  0.082  0.513  0.162  0.388  0.528  0  0  0.091  0.045  0  0  0.168  0.078  0.461  0.037  0.151  0.127  0.076  0.043  0  0.042  0  
 
 CTDSP1  58190  2  219263060  219270664  2q35  3.908  0.729  2.965  2.992  3.762  2.842  3.978  3.507  3.956  3.934  3.544  3.655  4.135  3.99  4.494  4.371  4.132  0.185  5.42  3.539  3.47  3.325  2.688  2.738  3.654  3.172  4.044  3.459  4.047  3.078  2.913  3.818  4.558  2.843  3.206  3.934  3.851  3.684  1.921  5.041  4.386  4.148  4.801  3.667  2.999  3.117  3.246  3.005  3.729  4.314  3.432  2.549  2.836  3.063  3.805  3.13  3.396  3.841  4.184  2.85  
 
 VIL1  7429  2  219283837  219314248  2q35  0  0.036  0.092  0  0.055  0  0  0  0.073  0.043  0  4.667  5.188  0.037  3.448  2.209  2.134  2.118  0.033  0.089  0.313  0.072  0.147  0  0  0  0.091  0  0  0.064  0  0  0  0.17  0.151  0.155  0  0  0.019  0  0.093  0.212  0.029  0  0  0.206  0.027  0  0  0  0  0.228  0.021  0.086  0.028  0  0  0  0  0  
 
 USP37  57695  2  219314973  219433084  2q35  1.103  1.082  0.715  0.693  1.049  0.607  1.471  1.121  1.115  1.302  1.629  1.402  1.618  1.719  1.478  1.401  1.562  2.56  2.203  1.248  0.636  1.601  0.634  0.537  0.852  1.166  1.029  0.892  1.073  0.779  1.124  1.123  1.13  0.706  0.773  1.175  1.254  0.72  0.544  1.646  1.337  0.716  1.804  1.167  0.877  0.88  0.875  0.641  1.267  1.086  1.1  0.614  0.936  0.689  0.92  0.71  0.925  0.797  1.103  0.991  
 
 RQCD1  9125  2  219433302  219461158  2q35  3.176  3.765  2.675  2.745  2.962  3.406  4.083  3.638  2.737  3.334  4.212  3.155  3.583  4.021  3.501  3.644  3.555  4.368  5.078  3.962  2.793  4.365  2.372  2.841  3.601  3.379  2.86  3.147  3.63  2.298  3.63  3.79  3.593  2.239  3.285  2.915  3.416  2.812  2.86  3.725  3.138  2.63  3.843  3.244  3.111  2.758  2.983  2.473  3.223  3.637  3.675  2.314  3.236  2.866  3.581  2.517  2.794  3.675  3.931  3.17  
 
 PLCD4  84812  2  219472487  219501909  2q35  0.438  0.151  0.289  0.178  0.708  0.41  0.652  0.338  0.681  0.448  1.202  0.068  0.535  0.367  0.432  0.236  0.175  0.085  0.375  0.245  0.132  0.471  0.207  0.123  0.271  0.507  0.504  0.342  0.533  0.186  0.367  0.622  0.521  0.527  0.425  0.341  0.402  0.267  0.35  0.519  0.294  0.682  0.492  0.367  0.409  0.344  0.704  0.267  0.31  0.402  0.727  0.565  0.302  0.219  0.448  0.435  0.244  0.21  0.369  0.225  
 
 ZNF142  7701  2  219502639  219524355  2q35  3.294  0.84  1.623  1.562  2.099  1.772  2.956  2.79  1.635  2.293  3.137  1.905  3.075  2.765  3.524  2.171  1.761  0.554  2.751  2.155  2.335  2.754  1.081  2.043  2.436  2.317  2.669  3.07  3.02  2.277  2.445  2.965  3.409  1.475  2.077  2.576  3.121  2.861  1.529  3.429  2.631  2.072  3.472  2.804  2.377  2.266  2.052  2.706  2.347  2.304  2.81  2.053  2.313  1.87  2.491  1.797  2.321  2.059  3.144  2.377  
 
 BCS1L  617  2  219524378  219528166  2q33  3.688  2.191  2.558  2.424  3.291  2.267  4.237  2.595  2.547  3.052  3.746  3.031  3.43  4.048  3.506  3.258  3.428  1.342  4.971  4.117  2.922  3.327  2.707  2.91  3.462  3.167  3.594  2.746  4.132  3.046  3.97  4.855  4.336  2.656  2.747  3.353  3.198  3.393  2.708  4.009  3.363  3.377  4.649  3.266  2.29  3.012  3.984  2.792  2.325  4.647  3.314  2.299  2.834  1.744  3.737  2.561  2.456  2.956  3.901  3.319  
 
 RNF25  64320  2  219528586  219536781  2q35  3.097  0.857  2.033  2.467  3.169  2.278  4.013  3.746  2.792  2.995  3.738  2.062  2.596  3.377  2.478  2.594  2.586  0.515  4  1.609  1.862  2.551  1.635  1.686  3.215  2.711  3.26  3.313  3.537  2.187  3.376  3.821  3.715  1.839  2.246  2.672  2.854  2.493  2.316  3.507  2.64  2.586  3.823  2.372  2.851  2.765  1.52  3.012  2.177  4.417  2.828  1.655  2.549  2.17  3.318  1.833  1.9  3.047  3.204  2.666  
 
 STK36  27148  2  219536748  219567440  2q35  1.781  0.363  0.584  0.581  1.528  0.539  1.554  1.555  1.461  1.449  1.786  0.686  1.178  1.671  1.45  1.227  0.823  0.256  1.477  0.453  0.615  0.75  0.499  0.604  1.207  1.674  1.537  1.895  1.818  0.946  1.388  2.151  1.963  0.692  0.826  1.294  1.284  1.782  0.691  2.194  1.282  0.955  2.879  1.37  1.319  1.327  1.218  1.204  1.467  2.181  1.449  0.683  1.208  1.189  1.888  0.861  1.555  0.988  1.885  1.26  
 
 TTLL4  9654  2  219575567  219620138  2p24.3-p24.1  1.924  0.414  1.142  0.893  1.573  1.053  2.046  1.912  1.23  1.641  2.426  1.889  2.56  2.062  1.708  2.134  1.573  0.135  2.344  1.471  2.169  1.765  1.144  1.762  2.278  2.539  2.292  2.769  3.497  2.602  2.965  3.566  3.158  1.375  1.746  2.213  2.457  2.573  0.976  2.701  2.57  1.31  2.559  2.88  1.386  1.634  1.12  2.138  2.127  2.536  1.902  1.248  1.684  1.125  2.908  1.318  1.41  1.647  2.367  2.383  
 
 CYP27A1  1593  2  219646471  219680016  2q33-qter  0  0.575  0.304  0.337  0.515  0.495  0.323  0.196  2.668  2.672  2.345  4.563  0.091  0.314  0.17  0.12  0.108  0.419  0.077  0.063  0  0.294  0.267  0  0  3.659  3.707  3.131  4.895  3.739  4.378  5.133  3.713  3.323  0.064  0.743  1.806  1.591  0.288  0.1  0.245  0.777  0.054  0.123  0.127  0.08  0.111  0.143  0.08  0.335  0.287  1.003  0.073  0.218  2.723  0.095  0.119  0  0.66  0.366  
 
 PRKAG3  53632  2  219687105  219696512  2q35  0  0  0.107  0  0.042  0  0  0.035  0.115  0.048  0  0  0  0.03  0.114  0.046  0  0.054  0  0  0  0.132  0.27  0  0  0  0.163  0.066  0  0.066  0  0.061  0  0.3  0  0  0  0  0.06  0  0  0.291  0.068  0  0  0.091  0.03  0  0  0  0  0.33  0  0.057  0  0  0  0  0  0  
 
 WNT6  7475  2  219724545  219738954  2q35  0.126  0.179  0.195  0  0.16  0.424  0.1  0.942  0.697  0.157  0.167  0.127  0  0.471  0.128  0.237  0.149  0.592  0.048  1.06  0  0.617  1.315  0.619  0  0.101  0.303  0.085  0.073  0.191  0  0.076  0.192  0.2  0.218  0.179  0.2  0  0.214  0.199  0.294  0.907  0.162  0.124  0.29  0.278  0.076  0  0.112  0.128  0.075  1.304  0.067  0.338  0.241  0.177  0.08  0  0  0  
 
 WNT10A  80326  2  219745254  219758651  2q35  0  0.126  0.154  0  0.113  0.079  0.164  0.207  0.204  0.125  0.069  0.126  1.895  0.34  0.695  2.46  0.127  0.261  0.118  0.12  0  0.337  1.041  0.916  0.105  0  0.265  0.097  0.055  0.161  0.079  0.063  0.064  0.149  0.132  0.386  0.24  0.373  0.258  0.287  0.407  0.446  0.081  0.464  1.152  0.344  0.362  0  1.598  0.122  0.055  1.167  0.077  0.243  0.116  0.178  0.26  0.064  0  0.111  
 
 LINC01494  101928537  2  219765536  219795359  2q35  0  0  0  0  0  0  0  0  0.124  0  0  0  0  0.064  0  0  0  0  0  0  0  0.046  0  0  0  0  0.053  0  0  0.051  0  0  0  0  0  0  0  0  0  0  0  0  0.043  0  0  0  0  0  0  0  0  0.244  0  0.083  0  0  0  0  0  0  
 
 CDK5R2  8941  2  219824349  219826877  2q35  0.132  0.096  0.221  0  0.085  0.067  0.372  0.174  0.476  0.174  0.135  0.133  0.262  0.221  0  0.12  0.109  0.259  0.051  0.113  0.08  0.342  0.369  0  0.89  0.136  0.212  0.064  0.127  0.256  0.312  0.282  0.433  0.617  0.36  0.292  0.1  0.213  0.277  0.312  0.344  0.727  1.257  0.073  0.138  0.092  0.046  0.272  0  0.484  0.209  1.164  0.582  0.798  0.237  0.139  0.173  0.136  0.103  0  
 
 LINC00608  151300  2  219841005  219842644  2q35  0  0  0.119  0  0  0  0  0  0.148  0  0  0  0  0  0  0  0  0.152  0  0  0  0  0  0  0  0  0.047  0  0  0.065  0  0  0  0.373  0  0  0  0  0  0  0  0  0.031  0  0  0  0.036  0  0  0  0  0.133  0  0.056  0  0  0  0  0  0  
 
 FEV  54738  2  219845808  219850379  2q36  0  0.076  0.164  0  0.088  0.133  0.087  0.154  0.136  0.125  0.07  0.266  0  0.109  0  0.136  0.141  0.213  0.133  0.2  0.225  0.365  0.296  0.112  0.081  0  0.188  0.061  0  0.215  0  0.209  0  0.169  0.131  0  0  0  0.176  0  0  0.605  0.078  0.077  0.116  0.127  0.063  0  0  0  0.07  1.263  0.064  0.336  0.167  0.248  0  0  0  0.077  
 
 CRYBA2  1412  2  219854911  219858127  2q35  0  0.175  0.328  0  0.109  0  1.45  0.157  0.389  0.272  0.252  0  0  0.183  0  0.15  0  0.281  0  0  0  0.142  0  0  0  0.139  0.085  0  0  0.113  0  0  0  0  0  0  0.204  0  0.163  0  0  0.19  0  0  0.307  0  0  0.925  0  2.708  0.325  0.604  0.198  0.206  0.12  0  0  0  0  0  
 
 LOC100129175  100129175  2  219866936  219880444  2q35  0.481  0  0.166  0  0  0  0  0  0.148  0.026  0  0  0  0.152  0.161  0.041  0  0.047  0  0  0  0.093  0.211  0  0  0  0.077  0  0  0.05  0  0  0  0.352  0  0  0  0  0.064  0  0  0.551  0.044  0  0  0  0.034  0  0  0  0  0.18  0.028  0.066  0  0  0  0  0  0  
 
 CFAP65  255101  2  219867567  219906273  2q35  0.11  0.039  0.204  0  0.088  0.096  0.088  0.042  0.198  0.074  0.058  0.101  0.06  0.132  0.052  0.053  0.094  0.12  0.041  0.081  0  0.187  0.335  0  0  0.098  0.281  0.075  0.122  0.226  0.392  0.157  0.073  0.463  0.165  0  0  0  0.109  0.066  0  0.695  0.101  0  0.075  0.077  0.077  0  0  0.048  0.049  0.534  0.035  0.137  0.099  0.099  0  0  0  0  
 
 IHH  3549  2  219919141  219925238  2q33-q35  0.106  0.053  0.2  0  0.097  0.07  0.144  0.215  0.184  0.166  0.175  0.19  0.641  0.164  0.111  0.419  1.624  0.139  0.069  0.09  0  0.454  0.244  0  0  0.07  0.277  0.113  0.133  0.28  0.115  0.114  0.176  0.312  0.093  0  0  0.145  0.257  0.139  0  0.265  0.295  0.074  0.108  0.239  0.1  0.094  0.121  0.094  0.463  1.225  0.107  0.327  0.189  0.086  0.082  0  0  0  
 
 NHEJ1  79840  2  219940045  220025587  2q35  2.078  1.326  1.524  1.352  1.548  1.533  2.321  1.828  1.26  1.681  2.493  1.109  2.282  2.642  1.463  1.723  2.168  1.038  3.089  1.813  1.5  2.41  1.337  1.416  1.669  2.432  2.386  2.062  2.658  0.968  2.572  2.601  3.067  1.764  1.475  2.099  1.752  1.749  1.775  2.568  1.541  1.55  3.127  2.07  1.304  1.268  1.463  1.383  1.746  2.553  1.887  1.148  1.722  1.576  2.398  1.203  1.727  1.357  2.731  1.555  
 
 SLC23A3  151295  2  220026180  220034817  2q35  0.146  0.082  0.355  0  0.191  0  0.305  0.08  0.111  0.295  0.264  0  0.108  0.708  0.168  0.259  0.065  0.137  0.419  0.114  0  0.229  0.4  0.178  0.071  0.269  0.728  0.162  0.121  0.168  0.21  0.272  0.69  0.207  0.079  0.144  0.239  0.156  0.507  0.189  0.273  0.327  0.656  0.132  0.104  0.144  0.051  0.148  0.216  0.467  0.15  0.381  0.061  0.098  0.225  0.077  0.418  0  0.399  0  
 
 CNPPD1  27013  2  220036618  220041702  2q35  3.579  1.317  3.13  2.878  3.705  3.188  3.404  3.114  3.29  3.272  4.03  3.283  3.507  3.164  2.772  3.318  3.439  0.791  5.2  3.044  3.059  3.256  3.162  3.093  3.278  3.452  3.348  3.564  3.652  3.848  4.017  3.329  4.159  2.854  2.603  3.538  3.283  3.669  3.269  3.316  3.005  3.123  4.113  2.433  2.741  3.427  2.881  3.027  2.823  4.465  3.899  2.066  2.965  2.566  3.467  2.305  3.13  3.305  3.614  3.325  
 
 FAM134A  79137  2  220042938  220050197  2q35  3.349  1.831  2.771  2.766  3.056  2.3  3.999  3.209  3.298  3.329  3.555  2.686  2.959  3.074  3.07  2.933  2.823  0.929  3.759  2.452  1.528  2.352  1.86  1.92  2.93  3.619  3.48  3.466  3.892  4.294  3.885  3.451  4.277  2.693  2.077  3.027  2.845  3.982  3.028  3.931  2.862  2.762  3.886  2.136  2.1  2.806  3.89  2.619  2.991  3.811  3.395  2.148  2.92  2.167  3.264  2.891  2.914  3.095  3.729  2.774  
 
 ZFAND2B  130617  2  220071505  220074373  2q35  3.577  1.675  2.698  2.05  2.82  2.53  4.156  3.62  3.213  3.411  3.132  3.226  2.908  3.5  3.188  3.654  3.287  0.303  5.164  2.942  2.589  3.657  2.226  2.241  2.708  2.38  2.933  2.696  3.11  1.827  3.115  3.274  3.505  1.926  2.242  2.316  3.317  3.335  2.34  3.47  3.168  2.794  3.75  2.179  2.606  2.884  2.957  2.797  3.151  4.05  2.644  1.874  2.77  2.028  3.28  2.173  2.515  3.598  3.201  2.717  
 
 ABCB6  10058  2  220074487  220083712  2q36  2.728  0.726  1.874  1.072  1.959  1.268  3.659  2.765  2.66  2.61  4.035  1.474  3.956  1.976  0.452  1.3  1.122  0.461  1.258  0.182  1.646  0.925  0.564  0.467  1.378  1.948  3.131  3.006  3.302  3.599  3.669  3.785  3.441  0.976  2.412  3.026  2.456  1.212  2.677  2.508  0.435  3.111  0.871  2.301  1.681  2.224  1.263  1.721  0.752  2.865  1.424  1.805  1.282  2.085  1.553  1.386  1.692  1.496  1.576  1.411  
 
 ATG9A  79065  2  220084101  220094410  2q35  3.358  1.13  3.927  3.113  3.028  2.684  5.068  4.419  3.754  4.371  4.728  2.426  3.723  3.802  3.784  3.214  3.442  0.219  3.383  1.941  3.091  2.281  2.084  2.356  3.199  3.382  4.261  3.326  4.221  4.092  3.486  4.595  4.762  2.828  2.754  4.238  4.329  3.373  3.25  3.911  3.663  3.395  4.005  3.304  3  3.136  3.158  3.008  3.871  3.925  3.319  2.726  3.405  3.056  3.366  3.492  3.407  2.883  3.553  3.378  
 
 ANKZF1  55139  2  220094478  220101391  2q35  2.229  0.394  1.988  1.767  1.521  1.279  1.962  1.746  1.89  2.451  3.024  1.403  2.373  2.313  2.302  1.814  3.002  0.286  3.486  1.829  1.895  2.515  2.073  1.535  1.555  1.734  2.148  1.764  2.836  2.545  2.23  2.585  2.675  1.4  0.99  1.649  1.698  2.11  1.782  1.885  1.798  2.041  1.907  2.141  1.365  2.107  1.658  1.363  1.326  2.911  2.124  1.28  1.726  1.344  2.333  1.703  2.246  1.43  1.895  1.747  
 
 GLB1L  79411  2  220101319  220110151  2q35  0.479  0.654  0.486  0.664  1.237  0.697  1.015  0.713  0.807  0.88  1.294  0.417  0.406  0.819  0.581  0.693  0.546  0.511  0.983  0.541  0.253  0.701  0.423  0.324  0.569  2.391  1.056  1.605  1.323  0.22  2.324  1.299  1.215  0.892  0.92  0.886  0.652  0.674  0.927  1.014  1.206  0.845  0.816  0.599  0.49  0.489  2.168  0.403  1.044  0.954  0.717  0.566  0.829  0.394  1.563  0.44  2.147  0.593  0.708  0.749  
 
 STK16  8576  2  220110191  220115059  2q35  1.542  0.725  1.259  1.112  1.866  0.981  2.57  1.016  1.498  1.212  1.83  1.629  1.501  1.643  1.399  1.481  1.854  0.627  2.275  0.958  0.923  1.353  1.192  0.854  1.554  1.262  1.747  1.351  1.593  1.699  1.775  1.574  1.84  1.05  1.261  1.522  1.628  1.392  1.472  1.661  1.604  1.659  2.09  1.136  1.185  0.98  2.202  0.999  1.177  2.139  1.411  1.201  1.196  0.967  1.985  1.161  0.811  1.361  1.479  1.301  
 
 TUBA4A  7277  2  220114432  220119330  2q35  0.82  1.992  1.204  2.328  2.953  2.073  6.445  6.201  1.003  5.064  7.135  5.022  5.78  4.04  4.869  4.589  5.385  2.355  3.853  5.232  3.954  0.564  4.336  4.488  0.11  0.648  3.942  0.584  0.487  0.821  0.26  0.52  5.181  3.246  5.663  6.342  4.27  4.311  5.196  4.345  5.383  5.402  2.302  2.65  5.573  5.741  6.449  4.662  5.223  5.845  5.391  2.983  5.993  6.387  4.672  5.196  4.46  0.221  6.48  4.756  
 
 TUBA4B  80086  2  220117964  220136910  2q35  1.111  0.189  0.638  0.569  0.608  1.304  2.261  1.81  0.314  1.626  2.93  1.374  2.148  1.236  1.787  1.45  1.909  0  0.755  1.359  0.812  0.788  1.366  1.218  0.553  0.349  1.554  0.662  0.964  1.236  0.356  0.693  1.662  0.95  2.067  1.605  1.362  0.994  1.423  1.017  1.288  2.04  0.952  0.372  1.32  1.491  2.226  1.505  0.964  1.394  1.948  0.719  2.685  2.653  1.993  1.508  1.369  1.207  2.366  1.215  
 
 DNAJB2  3300  2  220144039  220151622  2q32-q34  1.781  0.216  2.28  1.507  3.358  2.144  3.633  3.388  3.397  4.165  3.999  2.052  2.144  1.886  1.94  1.971  2.377  0.102  3.23  0.217  0.212  1.87  2.377  0.985  1.882  3.141  3.49  2.911  3.372  2.171  3.227  2.85  3.614  2.112  1.634  3.925  2.36  2.851  2.747  3.005  3.132  2.092  3.848  1.884  2.804  3.467  0.603  2.515  1.911  4.015  3.045  1.38  2.395  2.364  2.65  2.161  2.412  2.173  2.768  3.001  
 
 PTPRN  5798  2  220154344  220174295  2q35-q36.1  0  0.064  0.24  0  0.059  0.046  0.472  2.401  0.114  0.312  0.206  0.043  0  0.1  0  0.06  0.096  0.131  0.075  0.058  0  0.153  0.24  0  1.228  0.059  0.288  0.081  0.089  0.404  0.179  0.106  0  0.172  0.115  2.073  1.689  2.109  0.943  4.26  0  0.954  0.921  0  0.083  0.096  0.06  0  0  0.067  0.099  0.538  0.04  2.234  1.257  0.114  0.297  0.574  0.075  0.055  
 
 RESP18  389075  2  220192130  220197899  2q35  0  0  0  0  0  0  0  0  0  0  0  0  0  0  0  0  0  0  0  0  0  0  0  0  0  0  0.058  0  0  0.128  0  0  0  0  0  0  0  0  0.066  0  0  0  0  0  0  0  0  0  0  0  0  0  0  0.148  0  0  0  0  0  0  
 
 DNPEP  23549  2  220238179  220252662  2q35  4.638  1.824  2.655  2.299  3.179  1.912  4.896  3.429  3.112  3.283  3.711  3.084  3.776  3.961  3.373  3.898  3.32  1.009  4.908  2.624  2.798  3.375  1.695  2.605  3.541  2.667  3.963  3.608  3.868  3.517  3.692  4.473  4.287  2.128  2.454  2.917  3.688  3.752  3.173  4.201  2.971  2.522  3.801  2.486  2.762  3.596  3.167  1.82  2.647  4.008  3.12  1.945  3.185  2.695  3.823  2.895  3.311  3.402  3.849  3.39  
 
 DES  1674  2  220283098  220291461  2q35  0.245  0.096  0.332  0.389  0.238  0.252  0.663  0.342  0.27  0.572  0.885  0.404  0.395  0.475  0.236  0.498  0.698  0.243  0.215  0.469  1.515  0.566  0.203  0.276  0.197  0.235  0.974  0.259  0.472  0.523  0.083  0.225  0.167  0.319  0.848  0.339  0.385  0.117  0.84  0.262  0.116  0.565  0.4  0.135  0.249  0.304  0.641  0.618  0.113  0.726  0.27  1.306  0.617  0.997  0.705  0.343  0.315  0  0.689  0.16  
 
 SPEG  10290  2  220299699  220358354  2q35  0.957  0.058  2.976  3.081  0.927  0.588  2.714  3.686  3.546  3.317  3.361  0.188  0.361  2.088  0.079  0.296  0.146  0.183  1.36  0.264  0.592  0.696  0.6  0.169  3.298  0.773  1.429  0.599  0.714  1.172  0.556  1.132  0.914  0.851  0.538  0.686  2.199  3.986  3.927  0.928  0.156  1.905  0.986  0.852  1.494  1.76  0.785  1.924  0.12  4.091  2.011  1.444  0.307  1.562  2.936  1.393  2.088  1.722  1.446  2.049  
 
 LOC100996693  100996693  2  220361076  220363009  -  0.444  0.256  0.645  0.48  0.242  0  0.913  0.578  0.57  0.81  0.375  0.669  0  0.694  0  0.309  0.375  0.385  0  0.336  0.507  0.741  0.311  0  0.478  0.22  1.672  0.246  0.277  0.724  0.201  0.265  0.559  0.644  0.762  0.3  0.192  0.407  1.052  0  0  0.645  0.164  0  0  0.202  0.153  0.248  0.236  0.559  0.246  2.274  0.177  0.709  0.666  0  0.333  0.256  0  0  
 
 GMPPA  29926  2  220363586  220371718  2q35  3.345  0.494  3.713  2.977  3.344  2.579  4.795  4.377  2.769  4.038  4.546  3.496  2.717  4.367  3.039  3.016  3.352  0.274  4.52  2.789  3.343  2.684  2.383  2.962  3.691  3.463  4.069  3.158  4.522  3.493  4.376  5.215  4.07  2.166  2.71  3.715  3.538  4.054  3.301  3.629  3.708  3.218  3.969  2.936  2.045  3.779  2.889  2.973  2.336  5.373  3.617  2.185  3.209  3.09  3.833  3.165  3.472  3.338  3.798  3.615  
 
 ASIC4  55515  2  220378891  220403494  2q35  0  0  0.144  0  0.075  0.132  0.245  0.217  0.285  0.174  0.219  0.092  0.06  0.298  0.055  0.077  0.076  0.182  0.045  0.047  0.93  0.159  0.286  0  0.051  0.122  0.236  0.16  0.082  0.176  0.078  0.186  0.108  0.18  0.079  0  0.043  0.099  0.26  0.364  0.098  0.398  0.08  0  0.128  0.059  0.065  0  0  0.035  0.047  0.668  0.05  0.28  0.178  0.069  0.079  0.054  0  0  
 
 CHPF  79586  2  220403668  220408487  2q35  3.143  0.33  4.698  3.972  2.961  2.587  5.224  5.599  5.963  5.526  4.243  3.043  4.41  5.265  3.806  2.112  3.568  0.427  2.034  0.701  2.053  1.03  3.438  2.927  3.46  4.046  5.202  5.849  5.029  3.237  4.867  5.994  6.016  5.286  1.923  3.675  3.635  5.75  3.867  3.6  3.943  4.899  3.383  2.201  2.619  2.742  2.111  2.564  1.441  3.317  4.04  3.348  4.024  3.737  3.667  3.459  3.469  2.644  2.663  2.739  
 
 TMEM198  130612  2  220408384  220415317  2q35  0.66  0.16  0.731  1.081  0.651  0.66  0.486  1.078  1.321  1.36  0.821  0.345  0.856  1.005  0.662  0.764  0.575  0.363  1.551  0.229  0.8  0.475  0.891  0.641  0.914  1.096  1.828  1.288  1.751  0.368  2.008  1.838  2.44  1.219  0.465  1.273  0.513  1.512  0.669  0.846  0.68  1.491  0.818  0.7  0.75  1.195  1.564  0.423  0.423  0.857  0.589  1.262  0.978  1.568  0.875  0.467  1.307  1.077  0.772  0.462  
 
 OBSL1  23363  2  220415449  220436268  2q35  3.672  0.328  2.769  2.591  2.005  1.213  3.018  3.29  3.056  3.789  3.043  0.886  0.88  1.782  0.059  0.223  0.171  0.779  0.596  0.5  1.082  0.667  0.34  0.073  2.057  2.628  3.621  1.153  3.426  3.239  3.06  3.311  4.018  2.344  1.35  2.447  3.136  2.557  2.189  2.395  3.104  2.687  3.347  1.918  2.132  2.48  2.033  1.696  1.367  3.195  2.562  2.288  2.204  0.84  3.233  1.815  2.352  1.824  1.61  2.176  
 
 INHA  3623  2  220436953  220440435  2q33-q36  1.916  0  0.563  0.895  0.917  0.361  0.423  0.242  0.646  0.83  0.846  0.117  0.1  0.043  0  0  0  0  0  0  0  0.093  0.352  0  0  0.231  1.212  0.051  0.441  0.2  0.228  0.689  0.711  0.686  0.138  2.174  0.188  0.428  0.63  0.351  1.004  0.723  1.532  0.285  0  0.629  0.355  0.149  0.182  0.458  0.462  0.766  0.105  0.107  0.54  0  0.424  0.206  0  0  
 
 STK11IP  114790  2  220462572  220481173  2q35  1.441  0.081  0.852  1.067  1.445  1.509  1.839  1.955  1.267  2.102  2.008  1.318  1.125  1.598  1.766  1.282  1.311  0.039  2.834  1.757  1.154  1.621  0.456  0.641  1.133  1.979  2.164  2.699  2.519  1.287  1.772  2.588  2.504  0.921  1.188  1.405  1.399  1.588  0.938  2.548  1.974  1.846  1.323  1.374  1.094  1.453  0.408  1.219  0.849  2.703  1.154  1.219  1.329  0.921  1.892  0.867  1.395  1.577  1.601  1.573  
 
 SLC4A3  6508  2  220492291  220506702  2q36  0.509  0.031  1.498  0.574  0.438  0.612  1.041  1.19  1.866  2.005  1.5  0.093  0  1.667  1.101  0.077  0.224  0.173  0.042  0.157  0.069  0.21  0.256  0  0.527  1.225  2.399  0.821  2.196  1.641  1.458  1.932  2.942  1.123  0.52  1.327  0.935  1.616  0.987  1.426  0.727  1.027  1.247  0.927  0.853  1.266  1.003  0.814  0.854  1.841  1.248  1.211  0.422  1.22  1.859  0.659  1.335  0.312  1.215  1.25  
 
 EPHA4  2043  2  222282746  222438922  2q36.1  0.222  0.316  0.822  2.53  1.207  0.473  2.96  0.106  1.257  1.824  1.932  1.552  0  0.458  0  0.025  0.648  1.206  0.017  0.06  0  0.117  0.128  0  0.625  1.158  0.116  0.92  1.712  1.785  1.975  0.984  0  0.229  0.133  0.665  0.294  0.246  1.618  0.058  0.55  0.616  0.068  0.826  0.705  0.197  0.436  0.388  0.722  0.244  0.533  0.333  0.46  0.351  0.669  0.708  0.203  0.74  0.225  0.131  
 
 PAX3  5077  2  223064605  223163715  2q35  0  0  0.208  0  0.085  2.227  0  0.057  0.225  0.089  0.264  0.115  0  0.074  0  0.111  0  0.378  0  0.191  0  0.302  0.205  0  2.258  1.804  3.27  2.732  3.259  0.945  2.83  2.694  3.049  1.762  0  0.223  0.764  0  0.131  0.518  0  0.46  0.12  0  0  0.116  0.121  0  0  0  0  0.956  0  0.201  0.15  1.209  0  0.355  0  0  
 
 CCDC140  151278  2  223162865  223169936  2q36.1  0  0  0.108  0  0.083  0.246  0  0.047  0.136  0  0  0  0  0.034  0  0  0  0  0  0.114  0  0.107  0.196  0  0.125  0  0.615  0.188  0.214  0.199  0.082  0.317  0.364  0.342  0  0  0  0  0.053  0  0  0.752  0.064  0  0  0  0.085  0  0  0  0  0.526  0  0.108  0.045  0.087  0  0  0  0  
 
 SGPP2  130367  2  223289321  223423617  2q36.1  0.937  0.35  0.113  0  0.172  0.142  0.455  0.082  0.223  0.185  0.705  2.056  2.809  1.115  1.148  1.621  3.013  0  0.904  0.183  0.181  0.573  0.545  0.122  0  0  0.21  0.064  0.4  0.537  0.387  0.555  0.24  0.205  0.226  0.191  0.113  0.236  0.059  0  0.611  0.845  0.053  1.408  1.552  0.477  0.162  0.172  2.194  0.736  0.112  0.935  1.23  0.273  0.131  0.23  0.669  0  1.086  1.15  
 
 FARSB  10056  2  223436161  223521074  2q36.1  2.659  2.664  2.325  2.12  3.641  3.176  4.188  2.698  2.741  3.189  4.369  2.421  3.184  4.296  2.935  3.45  3.731  2.181  5.178  4.409  3.704  3.86  3.22  2.989  3.654  3.254  2.768  2.834  3.613  2.455  4.179  4.563  2.81  1.88  3.177  3.357  3.625  3.571  4.009  3.141  3.494  3.055  3.645  3.555  2.14  2.836  3.696  2.277  2.759  4.9  3.715  2.147  2.983  1.583  3.69  2.556  2.679  2.999  2.97  3.524  
 
 MOGAT1  116255  2  223536456  223574649  2q36.1  0  0  0  0  0  0  0  0  0  0  0  0.114  0  0  0  0  0  0  0  0  0  0.193  0  0  0  0  0.09  0.107  0  0.071  0.108  0  0  0  0  0  0.088  0  0  0  0  0  0.04  0  0  0.08  0.113  0  0  0  0  0  0  0  0  0  0  0  0  0  
 
 ACSL3  2181  2  223725731  223808119  2q34-q35  2.499  3.802  2.398  2.477  2.672  1.912  3.014  2.623  3.563  2.973  4.051  2.834  2.912  4.143  2.292  3.304  3.396  3.691  3.403  2.78  2.416  3.143  1.351  1.103  3.367  4.782  2.748  3.61  4.023  4.868  4.793  4.443  3.56  2.91  2.286  3.555  2.968  3.067  2.937  3.179  3.661  2.483  1.529  2.437  2.034  2.18  2.615  2.342  2.914  4.471  3.195  1.577  2.43  1.761  3.032  2.21  2.153  2.804  2.926  3.12  
 
 KCNE4  23704  2  223916647  223920357  2q36.1  0  0  1.31  0  0.048  0.068  0.958  0.376  2.102  2.197  2.217  0.071  0  0.047  0  0.04  0.094  0.155  0.133  0.096  0  0.239  0.24  0.042  0.037  0.123  0.219  0.098  0  0.718  0.129  1.06  0.222  0.419  0.096  0.251  0.074  0  0.045  0.111  0  3.434  0.043  0  0  0.068  0.042  0  0.055  0  0.082  0.265  0.035  0.6  0.036  0.047  0.648  0  0.053  0.048  
 
 SCG2  7857  2  224461657  224467217  2q35-q36  0  0.558  1.706  3.219  0  0.716  1.009  0.959  0.962  1.687  4.564  0  0.054  2.332  0.075  0  1.349  1.126  1.417  0  0.122  0.112  0.197  0  1.239  0.466  0.051  1.382  1.54  0.121  1.468  1.812  0.347  0.98  0  0.86  0.402  0.77  0.196  3.852  0.246  1.009  0.022  0.273  0  0.516  0.907  0.058  0.25  0.059  0.18  0.177  0  0.09  0  0  0.215  0  0.066  0  
 
 AP1S3  130340  2  224620046  224702319  2q36.1  0.456  1.006  0.465  0.223  0.71  0.596  0.442  0.355  0.398  0.584  1.996  1.118  1.261  3.665  1.405  1.925  1.803  1.084  0.536  0.681  0.12  0.193  0.326  0.314  0.911  1.397  0.36  0.859  1.475  0.451  1.097  1.197  0.844  0.524  1.546  1.237  0.943  0.948  2.077  2.05  2.187  0.438  1.2  1.367  0.632  1.168  1.678  0.604  1.939  1.455  2.865  0.682  1.4  0.531  1.075  0.931  0.449  0.427  1.747  2.428  
 
 WDFY1  57590  2  224740059  224810104  2q36.1  1.407  2.184  1.959  1.254  1.416  2.162  1.665  2.555  1.786  2.392  2.564  1.667  2.429  2.875  1.96  2.334  2.521  1.864  1.996  1.482  1.214  0.884  0.445  0.433  2.387  3.628  2.764  2.271  4.257  2.832  3.633  2.889  3.541  2.372  1.4  1.88  2.922  2.188  3.061  2.377  2.153  0.791  2.077  1.803  1.488  1.408  0.861  1.301  2.01  1.928  2.902  0.856  2.526  1.6  2.496  1.947  2.154  2.287  2.943  2.709  
 
 MRPL44  65080  2  224822120  224832431  2q36.1  2.582  3.235  1.902  2.84  3.133  1.747  3  2.091  2.646  2.309  3.379  2.798  2.917  4.039  2.41  3.201  3.458  4.304  4.387  3.462  2.192  2.719  2.019  1.05  3.039  3.401  2.468  2.125  3.451  2.72  3.908  3.41  3.402  3.194  2.305  2.654  2.096  2.019  3.273  2.857  2.427  2.564  2.845  2.636  1.914  1.714  2.706  2.151  1.869  4.088  2.545  1.404  2.211  1.915  3.286  1.906  1.639  2.244  3.17  2.886  
 
 SERPINE2  5270  2  224839764  224904036  2q36.1  0.379  0.273  4.105  6.211  0.088  2.424  5.821  2.366  5.321  6.93  4.255  0.616  2.261  4.214  0.442  2.251  0.058  3.047  0.881  1.47  0  1.383  3.878  1.475  0.934  7.582  7.967  7.919  5.852  2.61  5.418  9.348  7.488  7.254  2  1.554  1.867  0.549  6.011  3.034  2.038  5.375  2.134  0.135  0.915  0.115  3.337  1.482  4.764  3.553  4.378  1.146  2.757  3.103  3.142  4.996  2.664  0.604  3.958  0.93  
 
 FAM124B  79843  2  225243414  225266711  2q36.2  0  0  0.192  0  0  0.191  0.051  0  0.13  0  0  0  0  0.021  0  0  0  0.092  0.224  0.084  0.901  0.049  0.21  0  0  0  0.042  0  0  0.052  0  0.258  0  0.355  0  0  0  0  0.053  0  0  0.047  0.029  0  0  0  0.021  0  0  0  0  0.196  0  0.062  0  0  0  0  0  0  
 
 CUL3  8452  2  225334866  225450114  2q36.2  2.088  2.88  1.313  1.277  2.308  1.702  2.725  2.086  2.131  1.964  2.595  1.672  2.513  3.307  2.132  2.152  2.219  3.143  3.709  2.133  1.605  2.081  0.859  1.108  2.393  2.284  1.537  1.954  2.197  1.347  2.415  2.777  2.26  1.404  1.646  2.38  2.369  1.93  2.38  2.935  1.763  1.619  2.959  1.554  1.684  1.653  2.3  1.572  1.931  3.252  2.24  1.31  1.756  1.178  2.26  1.697  1.595  1.934  2.006  2.38  
 
 DOCK10  55619  2  225629806  225907330  2q36.2  0.093  0  0.798  0.524  0.361  0.425  2.051  1.436  0.973  1.262  0.756  0.015  0  0.016  0.035  0.025  0  0.056  2.547  0.559  0  0.776  0.791  0.483  1.179  2.375  1.091  1.42  2.427  0.461  1.388  2.812  2.153  0.89  0.81  0.05  0.945  0.163  1.26  0.073  0.547  0.815  0.069  0  0.109  0.073  0.039  0.235  0.372  0.385  0.779  0.762  0.977  1.175  1.223  1.001  1.021  1.04  0  0.792  
 
 NYAP2  57624  2  226265601  226518734  2q36.3  0  0.054  0.147  0  0.041  0  0.984  0.072  0.076  0.031  0.024  0.062  0  0.044  0  0.033  0.024  0.096  0  0.058  0  0.115  0.149  0  0  0  0.179  0.016  0  0.136  0  0  0  0.219  0.037  0.099  0.314  0  0.044  0  0.023  0.108  0.035  0  0.738  1.739  0.08  0  0  0  0  0.462  0.206  0.412  0.049  0  0.023  0  0  0.368  
 
 LOC646736  646736  2  227007509  227044778  2q36.3  0  0  0  0  0  0  0.224  0  0  0  0  0  0  0.597  0  0  0  0  0  0  0  0  0  0  0  0  0.062  0.069  0  0.068  0  0  0  0  0  0  0.132  0  0  0  0  0  0  0  0  0  0.06  0  0  0  0  0  0  0  0  0  0  0  0  0  
 
 IRS1  3667  2  227596032  227663506  2q36  3.43  2.467  1.44  1.532  0.855  1.901  2.725  2.334  1.512  2.221  2.69  0.17  1.612  3.348  1.692  3.049  0.983  0.437  0.592  0.43  0.304  1.167  0.303  0.315  1.255  1.552  1.152  2.342  1.325  1.065  0.561  2.158  1.983  0.851  2.314  3.522  2.782  2.984  0.532  3.149  2.159  1.323  0.188  1.4  1.227  1.548  2.36  1.88  1.057  0.981  2.605  1.984  2.686  1.359  1.704  2.138  2.032  1.375  2.241  1.738  
 
 RHBDD1  84236  2  227700670  227863923  2q36.3  1.261  1.508  1.015  1.068  1.447  0.666  2.162  1.515  1.459  1.184  1.145  1.075  1.58  2.694  1.443  1.283  0.852  1.48  2.067  1.559  1.534  2.295  0.917  0.589  1.725  1.418  1.132  1.24  1.286  1.143  1.204  1.677  0.613  0.51  1.26  1.308  1.623  1.384  1.481  1.782  1.622  0.949  2.127  1.236  0.82  1.196  1.338  0.768  1.36  1.714  1.724  0.86  1.216  1.146  1.804  1.317  1.13  1.334  1.192  1.548  
 
 COL4A4  1286  2  227867426  228029275  2q35-q37  0.028  0.092  0.092  0  0.036  0.044  0.046  0.054  0.167  0.56  0.199  0.033  0.019  0.695  0.057  0.048  0.021  0.061  0.04  0.033  0.018  0.134  0.327  0.241  0.02  0.011  0.068  0.359  0.013  0.052  0  0.055  0  0.2  0.43  0.643  0.012  0.065  0.724  0.06  0.25  0.357  0.026  0.39  0.071  0.672  0.039  0.197  1.299  0.167  0.025  0.429  0.024  0.106  0.29  0.026  0.03  0.033  0.368  0.023  
 
 COL4A3  1285  2  228029280  228179508  2q36-q37  0.046  0.022  0.046  0.032  0.01  0.019  0.024  0.027  0.165  0.109  0.134  0.023  0.017  0.378  0  0.019  0.015  0.031  0.011  0.025  0  0.058  0.451  0.173  0.019  0.017  0.042  0.199  0.025  0.056  0.018  0.017  0.019  0.116  0.215  0.345  0.014  0.032  0.11  0  0.066  0.318  0.028  0.048  0  0.232  0.03  0.03  1.645  0.044  0.028  0.173  0.026  0.037  0.205  0.018  0  0.045  0.101  0.02  
 
 LOC654841  654841  2  228085767  228189880  2q36-q37  0  0.083  0.103  0  0.027  0  0.11  0.093  0.204  0.072  0.104  0.053  0.072  0.221  0  0  0.044  0  0.041  0.054  0.095  0.032  0.132  0  0.058  0.054  0.101  0.12  0.079  0.044  0.099  0  0.072  0.186  0.071  0.074  0.054  0.163  0.055  0  0.059  0.101  0.227  0  0.063  0.065  0  0.061  0.159  0.105  0.031  0.226  0.024  0.056  0.097  0.049  0.139  0  0  0.077  
 
 MFF  56947  2  228189866  228222552  2q36.3  2.638  4.75  2.346  2.622  3.375  2.993  3.904  3.383  3.412  3.623  3.637  2.351  3.057  4.23  2.323  3.009  3.853  5.685  5.204  4.308  2.585  3.801  2.685  2.065  3.466  3.364  3.249  2.941  4.025  2.371  3.815  4.204  4.136  3.713  3.452  3.872  2.806  3.209  4.845  3.502  3.299  4.846  4.111  3.435  2.587  3.412  3.264  2.136  4.179  4.675  3.211  3.761  2.788  3.195  3.988  2.707  2.333  3.564  3.044  3.459  
 
 TM4SF20  79853  2  228226873  228244022  2q36.3  0  0  0.118  0  0.072  0  0  0.047  0.071  0.055  0.052  0  1.172  0.066  0  0  0.059  0.059  0.037  0  0  0.062  0  0  0  0  0.068  0  0  0.024  0  0.088  0  0.202  2.162  0.086  0  0  0.111  0  0.065  0.277  0  0.052  0  0.055  0  0  0.069  0  0.06  0.113  0  0.072  0.039  0  0  0  0  0  
 
 AGFG1  3267  2  228336887  228425938  2q36.3  1.65  2.672  1.447  2.192  1.596  1.452  2.583  2.454  1.697  2.029  2.272  1.144  2.017  2.7  1.775  1.752  1.954  2.75  3.016  1.918  1.49  1.954  0.777  0.669  2.098  2.404  1.591  2.485  2.417  1.939  2.22  2.575  1.94  1.169  2.202  2.808  2.68  1.808  2.872  3.331  2.129  2.297  2.76  1.953  1.857  1.746  2.234  1.818  2.596  2.905  2.337  1.904  2.171  1.775  2.709  1.669  1.933  2.347  2.528  2.575  
 
 C2orf83  56918  2  228474805  228498036  2q36.3  0  0  0.098  0  0  0  0  0  0  0  0  0  0  0  0  0  0  0  0  0.052  0  0.038  0.167  0.058  0  0  0.049  0  0  0.044  0  0  0  0.143  0  0  0  0  0.034  0  0  0  0  0  0  0  0.048  0  0  0  0  0.082  0  0.053  0  0  0  0  0  0  
 
 SLC19A3  80704  2  228549925  228582745  2q37  0  0.025  0.145  0.493  0.034  0.064  0.035  0.023  0.125  0.032  0.154  0.044  0  0.013  0  0  0  0.058  0  0.024  0  0.058  0.215  0  0.05  0.486  0.072  0.948  0.322  0.055  0.292  0.898  0  0.247  0.507  0.26  0.048  0.059  0.057  1.295  0.081  0.587  0.019  0  0.154  0.281  0.06  0  0.048  0.114  0  0.288  0.147  0.586  0.202  0.09  0.047  0.076  0.053  0.058  
 
 CCL20  6364  2  228678557  228682280  2q36.3  0  0.28  0  0  0  0  0  0.464  0  0  0.126  0.282  0.778  0  0  0.233  0.327  4.375  0  0  0  0  0.548  0  5.519  0.696  0.065  0.42  1.219  0  1.205  0.282  0  0  0.208  0  0.731  0  3.312  3.872  0.435  0.504  0  0  0  0.141  0.132  0  0.265  0.507  0.635  0  0  0.135  0.279  0  0.304  0.461  0.916  0  
 
 DAW1  164781  2  228736326  228789026  2q36.3  0  1.185  0.11  0  0  0.431  0  0  0  0  0.207  0  0  0.038  0  0  0  0  0  0  0  0  0  0  0.127  0  0.029  0.044  0  0.021  0  0  0  0  0.114  0.522  0.844  0.589  3.208  2.069  0.153  0.368  0  0  0  0.327  0.044  0.376  0.478  1.521  1.345  0.744  0.262  0.698  3.273  0.577  0.079  2.846  2.564  3.015  
 
 SPHKAP  80309  2  228844669  229046361  2q36  0  0  0.1  0  0.021  0.018  0  0.018  0.11  0.011  0  0.043  0  0.014  0  0.024  0.018  0.079  0  0.018  0  0.054  0.14  0  0.035  0  0.047  0.024  0  0.042  0  0  0  0.263  0.023  0  0  0  0.038  0.055  0  0.156  0.032  0  0.028  0.029  0.108  0  0  0  0.016  0.205  0.02  0.045  0.027  0.031  0  0.019  0  0  
 
 PID1  55022  2  229888688  230136057  2q36.3  0.088  0.86  1.068  0.996  0.143  1.359  1.746  1.94  0.919  1.675  1.253  0.081  0.901  0.093  0.137  0.048  0.107  0.135  0  0.066  0.087  0.339  0  0.053  1.567  0  0.545  1.745  0.27  0.578  0  0.402  1.366  1.118  0.206  2.51  1.609  0.51  0.462  0.193  0.104  1.493  0.06  0.629  0  0.142  0.316  0.215  0.168  0.945  0.493  0.979  0.718  1.074  0.097  0.32  0.734  0.26  0  0.139  
 
 DNER  92737  2  230222344  230579286  2q36.3  0  0.12  0.797  1.072  0.043  0.563  2.491  0.321  0.51  0.09  1.422  0.07  0  0.482  0  0.058  0.119  0  0.062  0.091  0  0.203  0.128  0.032  2.501  0.025  0.219  3.223  0.391  5.137  1.552  1.98  2.729  2.53  0.11  2.899  2.109  0.844  3.674  1.295  0.94  2.958  0.106  0.051  0  0.042  0.048  0.583  0.067  1.288  1.163  0.523  0.103  0.246  0.095  0.218  0.335  0.157  0  0.14  
 
 TRIP12  9320  2  230628552  230786725  2q36.3  2.189  2.988  1.812  1.779  2.09  2.102  2.995  3.272  2.178  2.574  2.678  1.697  3.138  3.068  2.724  2.697  2.469  2.633  3.589  2.337  1.985  2.307  0.923  1.119  3.275  3.148  2.303  3.274  3.209  2.163  2.805  3.176  3.268  1.79  2.016  3.06  3.116  2.798  2.898  3.692  2.826  1.726  3.512  2.281  2.511  2.005  1.611  2.141  2.629  3.232  3.038  1.992  2.648  2.206  3.22  2.263  2.617  2.752  2.937  3.45  
 
 FBXO36  130888  2  230787206  230877825  2q36.3  0.537  0.279  0.365  0.716  1.182  0.45  0.472  0.937  0.532  0.653  0.567  0.288  1.154  1.154  0.763  0.654  1.139  0.418  0.991  0.529  0.195  0.431  0.275  0.123  0.851  0.056  0.224  0.096  0.154  0.104  0.1  0.443  0  0.303  0.613  1.164  0.394  0.665  0.497  0.377  0.951  0.328  1.426  0.18  0.113  0.202  0.887  0.209  0.478  0.563  1.36  0.587  0.899  0.136  1.772  0.096  0.419  0.269  0.777  0.853  
 
 SLC16A14  151473  2  230899689  230933715  2q36.3  1.006  0.033  0.128  0.645  0.512  0.076  1.928  0.817  0.251  0.064  0.508  0.063  0.364  1.798  1.896  0.176  0.032  0.484  0.835  0.91  0  0.302  0.644  0.534  0.068  0  0.07  0.022  0.041  0.056  0.044  0.049  0  0.252  0.268  0.946  0.186  0.043  0.09  0.723  2.542  1.613  2.349  0  0.475  0  0.558  0.106  0  0.413  0.721  0.293  0.044  0.041  0.124  0  0  0.128  0.743  0.084  
 
 SP110  3431  2  231033644  231090444  2q37.1  0.543  1.621  0.592  2.047  1.909  0.794  2.535  2.523  0.753  1.701  1.348  1.303  1.307  1.711  1.093  4.385  0.655  0.778  3.099  2.375  1.131  2.687  1.234  2.125  3.577  2.055  1.697  1.832  2.117  1.051  1.971  2.356  1.986  1.609  0.985  0.575  1.113  0.606  4.315  1.262  2.218  1.292  2.188  0.73  1.059  0.312  2.455  0.636  0.901  1.871  1.039  0.641  1.6  2.024  2.953  1.13  2.327  1.88  1.695  2.257  
 
 SP140  11262  2  231090444  231177930  2q37.1  0  0.79  0.27  0  0.124  0.55  0.766  0.905  0.228  0.285  0.184  0.086  0  0.199  0  0.398  0.095  0.153  2.238  0.535  0  0.195  2.291  1.575  3.334  0.241  0.268  0.41  0.549  0.04  0.2  0.228  0.229  0.437  0  0.088  0.693  0.138  0.525  0  0.253  0.369  0.03  0  0.168  0  2.683  0  0  0.362  0.128  0.152  0.612  0.3  1.12  0.257  0.237  0.243  0.568  1.049  
 
 SP140L  93349  2  231191893  231268445  2q37.1  0.945  1.375  1.072  1.012  1.642  1.295  2.487  1.835  1.193  1.899  1.261  1.201  0.563  1.885  0.992  2.766  1.482  0.746  3.121  1.355  1.142  2.48  1.173  0.931  1.96  1.524  0.923  1.659  1.896  0.358  0.673  1.751  1.607  1.358  0.255  1.458  0.759  1.126  2.901  0.274  1.744  1.368  0.021  0.755  1.081  0.488  2.885  0.931  1.385  2.539  1.227  1.085  1.364  1.106  2.295  0.735  1.512  1.68  1.262  1.789  
 
 SP100  6672  2  231280870  231410317  2q37.1  0.516  4.483  2.491  3.049  1.481  2.029  3.334  3.105  1.746  2.414  1.933  1.923  2.495  3.376  1.611  4.962  2.004  3.79  3.515  2.914  1.792  2.603  1.502  1.871  4.203  2.318  1.776  2.009  2.588  1.103  2.649  2.869  2.407  2.141  1.459  1.592  2.244  2.28  4.761  0.466  2.707  1.775  0.284  1.526  1.096  1.318  2.732  1.448  2.041  3.941  2.585  1.379  1.885  2.292  3.627  2.198  3.444  2.402  2.612  3.364  
 
 LOC151475  151475  2  231555635  231565244  2q37.1  0  0  0.18  0  0  0  0  0  0.202  0.035  0  0.06  0  0.022  0  0.052  0  0  0  0  0  0.066  0.251  0  0  0  0.044  0  0  0.043  0  0.047  0  0.299  0  0  0  0  0.031  0  0.282  0.268  0.027  0  0  0.043  0.022  0  0  0  0  0.117  0.032  0.027  0  0  0  0  0  0  
 
 CAB39  51719  2  231577556  231685790  2q37.1  1.954  4.344  2.156  2.086  1.728  1.937  3.988  2.877  3  2.383  2.179  2.406  3.233  3.666  2.559  3.265  2.775  4.648  4.718  3.22  2.776  2.801  1.866  1.344  2.737  2.78  2.612  2.292  2.9  1.949  2.639  3.07  2.866  2.405  1.575  2.782  2.902  3.139  3.321  2.939  3.724  2.104  3.128  2.091  1.606  1.803  2.924  1.779  2.994  3.732  3.544  1.931  2.906  1.854  2.968  1.791  2.394  2.279  3.093  3.099  
 
 ITM2C  81618  2  231729312  231743969  2q37  2.873  2.881  4.074  3.824  3.111  3.431  5.491  5.362  5.544  4.847  3.514  4.087  3.546  5.098  4.124  3.17  5.082  2.406  6.508  4.872  2.111  3.509  4.798  5.084  3.398  5.529  5.929  5.302  5.224  4.636  5.278  5.598  6.141  5.09  3.26  3.835  5.295  4.262  2.929  4.801  3.739  3.843  4.208  3.921  3.447  3.186  4.976  4.705  2.457  6.334  4.218  3.396  2.146  2.88  4.104  3.162  2.154  4.384  3.571  3.098  
 
 LOC151484  151484  2  231751260  231769232  2q37.1  0  0  0.104  0  0.067  0  0.249  0.035  0.147  0.152  0  0.193  0.17  0  0.099  0.138  0.167  0.096  0.129  0.09  0  0.113  0.438  0.129  0  0.474  0.146  0.123  0.157  0.087  0.074  0.107  0  0.229  0.123  0.094  0.092  0.079  0.096  0  0  0  0.053  0  0  0.09  0.983  0  0  0.055  0.177  0.37  0.039  0.07  0.084  0  0.05  0  0.199  0  
 
 GPR55  9290  2  231772042  231789941  2q37  0  0.028  0.106  0  0.04  0.047  0.192  0.065  0.151  0.106  0.066  0.116  0.111  0.029  0  0  0  0.092  0.068  0.028  0.102  0.215  0.731  0.443  0.058  1.165  0.728  0.552  0.886  0.138  0.432  0.617  0.311  0.449  0  0  0.106  0.075  0.064  0  0  0.138  0.04  0  0  0  0.368  0.057  0  0.058  0.329  0.343  0.055  0.193  0.103  0  0  0  0  0  
 
 SPATA3-AS1  348761  2  231849082  231860747  2q37.1  0  0  0.144  0  0.056  0  0.088  0.033  0.128  0.024  0.03  0.101  0.114  0.053  0.209  0  0.071  0.076  0  0.076  0.063  0.12  0.271  0  0  0.047  0.098  0.161  0.035  0.064  0  0.079  0.093  0.267  0.151  0  0.039  0  0.048  0.102  0.03  0.198  0.054  0.049  0.114  0  0.041  0  0  0.069  0.081  0.298  0  0.1  0.054  0  0.026  0.054  0  0  
 
 SPATA3  130560  2  231860838  231871999  2q37.1  0  0  0  0  0  0  0  0  0  0  0  0  0  0  0  0  0  0.167  0  0  0  0.066  0.29  0  0  0  0.132  0  0  0.087  0  0  0  0  0  0  0  0  0  0  0  0.26  0  0  0  0.093  0  0  0  0  0  0.285  0  0.15  0  0  0  0  0  0  
 
 C2orf72  257407  2  231902280  231914427  2q37.1  0.177  0.032  0.156  0  1.666  0.24  0.196  0.277  1.05  0.758  0.44  1.724  0.189  0.677  0.884  0.203  0.815  0.402  0.034  0.049  0  0.259  0.4  0.301  0  0.573  0.254  1.05  0.679  0.331  0.19  0.605  0  0.302  0.724  0.248  0.122  0.198  0.159  0.708  0.421  0.737  1.699  0.382  0.164  0.218  0.179  0.417  0.686  0.867  0.154  0.93  0.018  0.412  0.232  0.061  0.107  0.093  0  0  
 
 PSMD1  5707  2  231921577  232037540  2q37.1  3.749  5.111  3.029  3.063  4.242  3.274  6.255  4.823  3.243  4.349  4.844  3.639  4.525  5.533  4.096  4.313  4.029  4.59  5.493  4.669  3.815  4.293  2.645  3.125  4.482  4.52  4.624  4.18  4.325  4.418  4.873  5.316  4.372  2.427  4.198  4.774  4.993  4.648  4.934  5.514  4.578  3.419  5.467  4.015  3.893  3.726  5.506  4.076  4.395  5.364  4.928  3.193  4.548  3.055  4.783  3.771  4.375  4.221  4.596  5.073  
 
 HTR2B  3357  2  231972949  231989824  2q36.3-q37.1  0.103  0.271  0.194  0  0.139  0.223  0.177  0.252  0.097  0.256  0.196  0.062  0.319  0.36  0.311  0.293  0  0.176  0.182  0.173  0.125  0.255  0.178  0.166  0  1.052  0.49  0.322  2.446  0.133  1.823  1.681  0.347  0.549  0.266  0.254  0.24  0.361  0.224  0.113  0.225  0.292  0.143  0.191  0.132  0.066  0.071  0.196  0.221  0.148  0.247  0.156  0.145  0.075  0.283  0.147  0.223  0.058  0.179  0.076  
 
 ARMC9  80210  2  232063259  232240590  2q37.1  1.647  0.783  1.227  1.13  0.796  0.67  2.467  2.287  0.852  2.043  1.923  0.624  0.553  1.352  1.376  1.281  1.051  0.379  1.279  0.326  0.18  0.689  0.21  0  1.273  3.532  2.436  2.932  4.869  1.882  2.515  3.594  2.416  1.532  1.067  1.024  1.6  2.063  1.403  1.741  0.994  1.092  2.412  1.063  0.52  0.663  0.745  1.127  0.972  2.327  1.373  0.735  1.637  1.624  2.201  1.194  1.447  0.994  1.458  1.996  
 
 B3GNT7  93010  2  232260334  232265875  2q37.1  0.068  0.207  0.248  0  0.085  0.056  0.158  0.131  0.358  1.845  0.374  0.244  0.847  0.868  0.11  0.203  3.26  0.187  0.067  2.561  0  0.419  0.381  0.049  0.057  1.919  1.051  1.624  0.167  0.339  0.152  1.931  4.097  2.993  0.343  0.994  2  0.769  0.228  0.435  2.103  0.081  0.271  0.587  0.231  0.504  0.505  0.06  0.349  0.132  2.016  0.839  1.608  0.286  1.47  0.143  0.113  0.095  1.183  1.002  
 
 NCL  4691  2  232319458  232329205  2q37.1  5.768  6.302  5.083  5.167  5.259  6.253  6.284  6.024  5.139  5.351  6.85  5.54  6.403  6.574  6.857  6.306  6.087  6.29  7.082  6.541  5.834  6.754  4.982  5.756  6.358  5.786  5.647  6.198  6.519  5.751  6.552  6.844  6.554  4.659  6.35  5.985  6.343  5.591  6.05  6.835  6.271  5.813  6.666  6.501  5.861  5.357  5.994  5.876  5.693  6.929  6.045  5.158  6.368  5.175  6.577  5.714  5.455  6.406  6.263  6.628  
 
 SNORA75  654321  2  232320510  232320647  2q37.1  0  0  0  0  0  0  0  0  0  0  0  0  0  1.716  0  0  0  0  0  0  0  0  0  0  0  0  0  0  0  0  0  0  0  0  0  0  0  0  0  0  0  5.163  0  0  0  0  0  0  0  2.828  0  0  0  0  0  0  0  0  0  0  
 
 LINC00471  151477  2  232373136  232379050  2q37.1  0.2  0.174  0.169  0.216  0.163  0  0.187  0.287  0.233  0.212  0.337  0.129  0.256  0.289  0.282  0.079  0  0.101  0.301  0.072  0  0.318  0.554  0.324  0.168  0.069  0.088  0.085  0.197  0.074  0.347  0.437  0.233  0.459  0.443  0  0.224  0  0.131  0.316  0.335  0.173  0.043  0  0.152  0.281  0.526  0.15  0  0.412  0.074  0.309  0.129  0.187  0.348  0  0  0  0  0  
 
 NMUR1  10316  2  232387870  232395182  2q37.1  0  0.061  0.175  0  0.043  0  0  0.026  0.147  0.058  0.048  0  0  0.059  0  0.041  0  0.07  0.035  0.086  0.095  0.209  0.304  0  0  0.046  0.115  0.047  0  0.207  0  0.063  0.048  0.304  0.044  0.128  0  0  0.095  0.098  0.069  0.326  0.084  0.086  0.254  0.068  0.054  0  0  0.054  0.026  0.607  0.063  0.1  0.06  0  0.044  0.096  0  0  
 
 C2orf57  165100  2  232457574  232458994  2q37.1  0  0  0.149  0  0  0  0  0  0.231  0.079  0  0  0  0.057  0  0.098  0  0.175  0  0  0  0.137  0.383  0  0  0.08  0.2  0  0  0.121  0  0  0  0.325  0  0  0  0  0.073  0  0  0.113  0.126  0  0  0  0.061  0  0  0  0  0.762  0  0.095  0.085  0  0  0  0  0  
 
 PTMA  5757  2  232573234  232578250  2q37.1  7.538  9.186  6.429  7.85  8.13  7.397  8.006  7.135  7.441  7.151  8.818  8.638  7.83  9.287  8.467  8.727  8.88  10.121  10.367  9.501  7.517  9.355  7.291  6.824  8.114  7.351  6.966  6.927  6.637  5.869  7.604  7.76  7.677  6.429  7.449  7.428  7.28  6.9  7.358  7.581  7.375  8.398  7.884  8.288  7.241  7.458  7.604  7.332  7.912  9.489  7.782  6.371  6.818  5.944  8.556  7.171  6.856  8.365  8.112  8.216  
 
 PDE6D  5147  2  232597134  232646037  2q35-q36  3.095  4.446  3.057  2.692  2.788  2.346  4  3.391  3.141  3.337  3.625  2.763  2.297  3.818  2.557  2.324  3.57  3.403  5.061  3.81  1.258  4.332  2.918  2.035  3.364  2.918  3.701  2.734  3.461  1.897  3.783  3.211  3.671  3.116  2.413  2.913  2.287  3.642  4.704  2.794  2.752  2.456  4.03  2.273  0.723  2.545  4.439  2.827  2.491  4.454  3.566  2.173  2.735  2.171  3.917  2.328  2.032  2.572  3.427  3.044  
 
 COPS7B  64708  2  232646380  232673963  2q37.1  2.447  1.574  2.002  2.331  2.742  2.564  3.73  2.978  2.826  2.705  3.581  2.355  2.6  3.021  3.138  3.366  2.899  1.695  4.6  2.81  2.798  3.904  2.013  2.033  3.224  2.646  3.086  2.68  3.322  1.659  2.894  3.155  3.401  1.824  2.57  2.705  3.459  2.962  3.071  3.263  2.666  2.731  4.16  2.36  2.422  1.999  2.464  3.061  2.762  3.925  3.097  2.075  2.637  2.124  3.401  2.533  2.313  3.348  2.816  2.719  
 
 NPPC  4880  2  232786804  232791038  2q24-qter  0  0  0  0  0.421  0  0  0  0.353  0.132  0  0.332  0  0.098  0  0  0  0  0.296  0  0  0.522  0.43  0.286  0  0.324  0.167  1.322  2.001  0.425  0.183  0.184  0  0.26  0  0  0  0  0.131  0  0.217  0  0.336  0  0  0  0.092  0  0  0.637  0  0.833  0  0.15  0.278  0.24  0  0  0  0  
 
 DIS3L2  129563  2  232826292  233208678  2q37.1  1.692  1.033  1.047  1.048  1.36  1.302  1.909  1.613  1.464  1.781  2.142  1.258  2.161  2.134  2.223  1.56  1.14  0.982  2.58  1.257  1.334  1.768  0.713  1.043  1.692  1.947  1.492  2.741  2.481  1.602  1.81  2.001  2.307  1.08  1.505  2.11  2.126  1.915  1.353  2.474  1.971  1.655  1.614  1.461  1.382  1.135  1.065  1.594  1.779  2.047  1.755  1.521  1.482  0.901  2.225  1.305  1.345  1.658  1.984  2.189  
 
 ALPP  250  2  233243243  233247599  2q37  0  0.083  0.151  0  0.041  0.14  0.034  0.066  0.189  0.063  0.148  0.032  0.129  0.055  0.231  0.097  0.037  0.119  0.029  0.062  0  0.168  0.281  0  0  0  0.113  0.047  0  0.105  0.055  0  0  0.224  0.139  1.206  0.292  0.123  0.098  0.131  2.947  0.412  0.065  0.359  0  0.241  0.05  0.364  0.407  1.176  0.042  0.59  0.05  0.101  0.088  0.055  0  0.107  0  0.322  
 
 ECEL1P2  347694  2  233250459  233251754  2q37.1  0  0  0.371  0  0.166  0  0.075  0  0.266  0  0.073  0  0  0.069  0  0.075  0  0  0.079  0.114  0  0.295  0.259  0  0  0  0.169  0.064  0  0.113  0  0  0  0.384  0  0.101  0.638  0.207  0.09  0  0.144  0.311  0.069  0  0  0.114  0.173  0  0  0  0  0.936  0  0.137  0.104  0  0  0  0  0.197  
 
 ALPPL2  251  2  233271551  233275424  2q37  0  0  0.091  0  0.046  0.244  0.057  0.048  0.114  0.054  0.066  0.077  0.438  0.068  0.067  0.184  0.066  0.133  0.062  0.069  0  0.179  0.363  0.05  0.054  0  0.139  0.066  0.059  0.116  0  0  0  0.174  0.098  0.22  0.069  0.079  0.074  0.132  0.591  0.261  0.227  0.139  0  0.161  0.051  0.074  0  0.171  0.031  0.782  0.045  0.123  0.209  0.051  0  0  0.048  0.076  
 
 ALPI  248  2  233320821  233325455  2q37.1  0  0.058  0.139  0  0.028  0  0.05  0.04  0.16  0.125  0.088  0.084  0.202  0.089  0  0.097  0.083  0.071  0  0.119  0  0.299  0.315  0.048  0  0  0.166  0.029  0.075  0.143  0  0  0  0.129  0.093  0.043  0  0  0.171  0  0  0.52  0.202  0  0  0.082  0.09  0  0  0  0  0.746  0  0.131  0.228  0.126  0.031  0  0  0  
 
 ECEL1  9427  2  233344536  233352569  2q37.1  0.116  0.092  0.133  0  0.226  0.164  0.077  0.1  0.152  0.121  0.106  0.187  0  0.162  0.056  0.186  0.123  0.17  0.057  0.235  0.084  0.527  0.176  0.099  0.072  0.048  0.221  0.118  0.09  0.163  0.045  0.048  0  0.088  0.24  0.148  0.082  0.06  0.228  3.075  0  1.092  1.5  0.605  0.104  0.226  0.245  0.096  0.249  0.08  0.116  1.27  0.098  0.233  0.245  0.176  0.067  0  0  0  
 
 PRSS56  646960  2  233385172  233390425  2q37.1  0  0.035  0.114  0  0.051  0  0  0.053  0.121  0.057  0.037  0.073  0  0.097  0  0.058  0  0.119  0  0  0  0.208  0.205  0.058  0  0.053  0.192  0.047  0.047  0.135  0  0  0  0.104  0.149  0.093  0  0  0.088  0.157  0.057  0.446  0.06  0  0  0.084  0.092  0  0  0  0.043  1.029  0.045  0.203  0.149  0  0  0  0  0  
 
 CHRND  1144  2  233390869  233401375  2q37.1  0.318  0  0.134  0  0.076  0  0  0.049  0.1  0.062  0.062  0  0.078  0.088  0  0.052  0.076  0.516  0.034  0.067  0  0.12  0.218  0  0  0  0.087  0.055  0.068  0.095  0  0  0  0.235  0  0.053  0  0.109  0.108  0.244  0.239  0.386  0.05  0  0.073  0.031  0.04  0  0  0.044  0  0.399  0.032  0.083  0.076  0  0  0  0.076  0  
 
 CHRNG  1146  2  233404436  233411038  2q37.1  0.461  0.036  0  0  0.149  0.142  0.07  0.155  0.171  0.2  0.118  0  0.254  0.168  0.303  0.178  0.128  0.137  0  0.068  0  0.372  0.152  0.138  0.098  0.177  0.295  0.259  0.363  0.123  0.087  0.092  0.243  0.205  0.121  0.137  0.154  0.164  0.088  0.416  0.295  0.365  0.065  0.216  0  0.075  0.033  0.084  0.157  0.091  0.169  0.508  0.209  0.194  0.208  0  0  0.186  0.276  0  
 
 TIGD1  200765  2  233412679  233415300  2q37.1  1.234  1.236  0.436  0.711  1.373  0.473  0.819  0.793  1.184  0.803  1.124  0.276  0.951  1.239  0.799  1.159  0.792  2.628  2.991  1.543  0.85  1.743  0.357  0.194  0.636  0.759  0.44  1.493  0.938  0.552  1.099  1.536  1.057  1.016  0.904  1.185  0.763  0.604  1.191  1.173  1.186  0.998  0.902  0.89  0.712  0.586  0.431  0.433  0.498  1.763  0.909  0.789  0.538  0.846  1.399  0.459  0.763  0.82  1.099  0.814  
 
 EIF4E2  9470  2  233415296  233448355  2q37.1  3.931  3.832  3.502  3.019  4.205  3.531  4.815  4.773  3.475  4.02  4.917  3.916  3.93  4.468  3.442  4.575  4.343  3.937  5.526  4.75  3.646  4.242  2.625  2.671  4.285  3.213  3.543  3.455  3.796  2.719  4.009  4.811  3.993  2.824  3.779  3.988  3.51  3.268  4.574  4.037  4.496  3.784  4.916  4.679  4.162  3.634  4.078  3.264  3.805  5.038  4.069  2.673  4.078  3.561  4.729  3.876  3.491  3.854  4.112  4.062  
 
 EFHD1  80303  2  233470766  233547491  2q37.1  2.232  1.64  2.829  1.052  6.692  0.127  1.668  0.461  1.575  2.355  1.805  0.103  0  0.433  1.808  0.062  0  0.144  0.057  0.078  0.949  0.309  0.236  0  0  2.521  0.299  0.922  0.117  1.095  0.869  0.183  0  0.239  0.528  1.7  2.421  0  0.181  2.41  5.199  0.693  3.918  0.424  3.744  3.338  0.282  1.332  0.452  1.694  0.111  0.78  0.301  0.15  0.08  1.077  0.154  0  0.144  0.563  
 
 GIGYF2  26058  2  233562014  233725289  2q37.1  1.861  2.017  1.307  1.28  1.779  1.114  2.063  1.929  1.942  1.878  2.589  1.458  2.229  2.553  2.533  1.908  1.81  1.973  2.383  1.563  1.895  1.857  1.194  1.208  1.822  2.283  1.662  1.886  2.473  1.762  2.288  2.233  2.101  1.315  1.647  1.984  2.062  1.961  1.965  2.566  2.163  1.361  2.488  2.264  1.477  1.495  1.215  1.658  2.031  2.343  2.191  1.282  1.657  1.282  2.177  1.434  1.637  1.76  1.997  2.141  
 
 KCNJ13  3769  2  233630511  233641275  2q37  0.252  0.145  0.085  0  0.19  0.213  0.114  0.19  0.088  0.219  0.302  0.113  0.318  0.387  0.446  0.297  0.141  0.056  0.359  0.144  0.077  0.181  0.177  0.051  0.048  0.598  0.105  0.159  0.234  0.223  0.533  0.237  0.215  0.305  0.097  0.151  0.219  0.278  0.136  0.065  0.202  0.367  0.325  0.305  0.252  0.198  0.04  0.159  0.22  0.247  0.152  0.234  0.162  0.063  0.236  0.084  0.228  0.26  0.268  0.067  
 
 C2orf82  389084  2  233734993  233741107  2q37.1  0  0  0  0  0  0  0.468  0.298  0.388  0.182  0.378  2.102  0  0.542  0  0.32  0.45  0  0  0.229  0  0.766  0  0  0  0  0.295  0  0  0.121  0.472  0  0  0  1.694  0  0  0.557  0.476  0  0.458  1.121  0.136  0  0.405  0.237  0.937  0  0  0.54  0.312  2.194  0  0.231  0.605  0  0  0.473  0  0  
 
 NGEF  25791  2  233743395  233877951  2q37  0.336  0.277  0.088  2.123  1.75  0.058  2.919  1.177  1.239  1.135  0.671  0.318  1.146  1.35  0.794  0.91  1.464  0.381  0.042  0.101  0  0.247  0.249  0  0.214  0.05  0.121  0.086  0.073  0.123  0.054  0.147  0.047  0.268  1.417  0.205  0.712  1.898  0.468  1.499  1.883  0.638  0.26  0.803  0.489  0.523  1.181  0.179  2.549  0.512  4.319  0.864  0.223  0.291  0.706  1.075  0.181  0.316  0.083  0.114  
 
 NEU2  4759  2  233897381  233899767  2q37  0  0.071  0  0  0  0  0  0  0.129  0.101  0  0  0  0.06  0  0  0  0  0  0  0  0.204  0.332  0  0  0  0.076  0  0  0.054  0  0  0  0  0  0  0  0.224  0.109  0  0.215  0.29  0.072  0  0  0  0  0  0  0  0.315  0.97  0  0.071  0.077  0  0  0  0  0  
 
 INPP5D  3635  2  233924676  234116549  2q37.1  0  0.034  0.077  0.074  0.041  0.1  0.031  0.033  0.111  0.044  0.022  2.059  0  0.293  0.03  2.518  0.048  0.986  3.471  3.004  0.392  2.047  0.824  0.993  0.042  0.049  0.082  0.193  0.029  0.081  0.027  0.061  0  0.124  0.027  0  0  0.066  0.425  0.09  0.024  0.171  0.052  0.195  0.038  0.044  0.751  0  0.045  0.036  0.105  0.441  0.024  0.196  0.182  0.075  0  0  0  0.139  
 
 ATG16L1  55054  2  234160216  234204320  2q37.1  1.453  1.316  1.707  1.953  1.857  0.94  2.833  1.559  2.395  1.977  2.399  1.901  2.28  2.11  1.888  2.157  1.826  1.157  2.538  1.653  0.959  1.713  1.146  0.599  1.481  1.979  2.011  2.193  2.057  1.946  2.13  2.578  2.438  1.646  1.849  1.991  2.054  2.335  1.935  2.643  2.283  1.955  2.393  1.863  1.362  1.752  1.675  1.271  2.381  2.63  3.409  1.25  2.367  1.783  2.943  1.726  1.877  1.301  2.511  1.966  
 
 SCARNA5  677775  2  234184371  234184649  2q37.1  0  0.645  0.674  0  0.637  1.949  0  0.894  0.727  0.515  0.675  1.921  1.139  1.09  0  1.647  0.685  0  0  0.943  0  0.852  0  0  0.697  0  0  0.914  0  0  0  0  0  1.69  1.436  0  0  0  0.691  0  1.245  1.176  0  1.231  0  0  0  0  0  0  0.798  0.944  0.586  0  1.498  1.437  0.607  0  1.533  0  
 
 SCARNA6  677772  2  234197321  234197587  2q37.1  0  0  0.721  0  0  0  0  0.551  0  0  0.577  1.055  0  0.486  0  1.247  0  0  0  0  1.806  0  0  0  0  0  0  0  0.554  0  0  0  0  0  0.907  0  0  0  0  0  0  0  0  1.087  0  0.574  0  0  0  0  0.745  0  0  0.354  0  0.949  0  1.319  0  0  
 
 SAG  6295  2  234216308  234255701  2q37.1  0  0.055  0.101  0  0.057  0.11  0.082  0.081  0  0.049  0.096  0.082  0  0.093  0.105  0.063  0  0  0.142  0.081  0  0.135  0.186  0  0  0  0.192  0.073  0.072  0.108  0  0.065  0  0.131  0.172  0.082  0.06  0.086  0.101  0  0  0.176  0.066  0  0  0  0.071  0  0.111  0.088  0  0.529  0.133  0.139  0.119  0  0  0  0  0  
 
 DGKD  8527  2  234263152  234380743  2q37.1  1.319  0.803  1.626  1.495  0.954  1.052  2.452  3.231  1.862  1.671  2.967  1.1  2.251  2.305  2.212  1.377  1.971  0.65  3.025  1.581  1.252  1.577  0.861  0.894  1.883  1.205  1.546  2.353  1.547  1.336  1.471  2.177  1.894  0.785  2.532  3.352  2.935  2.543  1.642  2.767  1.74  3.379  1.672  1.592  0.789  1.233  1.571  2.189  1.687  3.525  2.062  2.451  2.302  1.809  2.785  1.457  2.397  1.825  2.383  2.331  
 
 USP40  55230  2  234384164  234474236  2q37.1  1.241  1.536  0.894  1.425  1.589  0.868  2.061  2.534  2.378  2.46  3.117  1.143  1.823  2.394  1.677  1.721  1.784  0.774  1.299  0.698  0.921  0.828  0.853  0.418  1.429  1.566  1.562  1.396  1.845  1.853  1.764  1.922  1.365  0.827  1.806  2.187  3.364  1.801  2.954  2.348  2.403  1.884  0.035  1.278  0.967  1.243  1.597  1.164  1.886  2.29  2.014  1.501  1.82  1.129  2.64  2.215  1.56  0.982  1.971  2.704  
 
 UGT1A8  54576  2  234526290  234681945  2q37  0  0  0.095  0  0  0  0.743  0  0  0.032  0.073  0.262  0.249  0  0  3.115  0  0.079  0  0  0  0.136  0  0  0  0  0.052  0  0.083  0.042  0  0  0  0.074  0  0  0  0  0.025  0  0  0.058  0  0  0  0  0  0  0  0  0.296  0  0.096  0.047  0  0.65  0.09  0  0  0  
 
 UGT1A10  54575  2  234545122  234681951  2q37  0  0  0.148  0  0  0  0.342  0  0  0  0.11  2.444  1.596  0.099  0  4.372  0  0.102  0  0  0  0.086  0  0  0  0.065  0.211  0  0  0.072  0  0  0  0.102  0  0  0  0  0.057  0  0  0.465  0.025  0  0  0  0  0  0  0  0.471  0  0.087  0  0.16  0.09  0.635  0  0  0  
 
 UGT1A9  54600  2  234580543  234681951  2q37  0  0  0.103  0  0  0  0.137  0  0  0  0.043  0.08  0.125  0  0  1.858  0  0  0  0  0  0.058  0.136  0  0  0  0.026  0  0  0.031  0  0  0  0.149  0.302  0  0  0  0  0  0  0.451  0  0  0  0  0  0  0.151  0  0.062  0.081  0.038  0.059  0  2.141  0.129  0  0  0  
 
 UGT1A7  54577  2  234590583  234681945  2q37  0  0.056  0.13  0  0  0  0.178  0  0  0  0.998  0.148  0.174  0  0  1.483  0  0.09  0  0  0  0.128  0.126  0  0  0  0.042  0  0  0.033  0  0  0  0.169  0.653  0.913  0  0  0.131  0  0  0  0  0  0  0  0.144  0  0  0  0.105  0  0.109  0.051  0  0.105  0.169  0  0  0  
 
 UGT1A6  54578  2  234600320  234681951  2q37  1.403  0  0.104  0  0  0  1.753  0  0.087  0  0.741  3.386  5.593  0  0.293  3.484  2.906  1.25  0  0  0  0.061  0  0  0  0  0.375  0  0.1  0.177  0  0  0.144  0.116  0.609  0.345  1.023  0  0.129  0  2.795  1.239  0.024  1.01  0  0.262  0  0  3.498  0  0.045  0.461  3.407  2.165  0  3.181  0.218  0.073  0.434  0  
 
 UGT1A5  54579  2  234621637  234681945  2q37  0  0  0  0  0  0  0  0  0.08  0  0.063  0.181  0.715  0  0  1.248  0.327  0.099  0  0.051  0  0.075  0  0  0  0  0.049  0  0  0.035  0  0  0  0.083  0.177  0  0.274  0  0.03  0  0.582  0.13  0  0  0  0.061  0  0  0.393  0  0  0.1  0.689  0.329  0  0.222  0.057  0  0  0  
 
 UGT1A4  54657  2  234627437  234681945  2q37  0  0  0  0  0  0  0.112  0  0  0  0  0.154  0.797  0  0  0.841  0  0.077  0  0  0  0.048  0.117  0  0  0  0  0  0  0.031  0  0  0  0  0.086  0  0.147  0  0.064  0  0.435  0.037  0  0  0  0  0  0  0.157  0  0  0.109  0.596  0.51  0  0.139  0.099  0  0  0  
 
 UGT1A3  54659  2  234637772  234681945  2q37  0  0  0  0  0  0  0  0  0  0  0.083  1.109  0.966  0  0  0.976  0.261  0  0  0  0  0.026  0.132  0  0  0  0  0  0  0.021  0  0  0  0.13  0.158  0  0.167  0  0  0  0.314  0  0  0  0  0  0  0  0.334  0  0  0  0.23  0  0  0.197  0  0  0  0  
 
 DNAJB3  414061  2  234651395  234652661  2q37  0  0.285  0  0  0  0.702  0  0.409  0  0.056  0  0  0.568  0  0  0.55  0.13  0.723  0  0.097  0  0.318  0  0  0  0  0.061  0  0  0.06  0.561  0.698  0.514  0.168  0.118  0  0.168  0.498  0  0  0.732  0.808  0.06  0  0.538  0.575  0  0.296  0.285  0  0  0.969  0.273  0.105  0.099  0  0  0.627  0  0  
 
 LOC100286922  100286922  2  234662961  234663991  2q37.1  0.436  0.16  0.401  0  0.163  0.216  0  0.14  0  0.099  0.377  0  0.621  0  0  0.809  0  0.396  0  0.176  0  0.248  0  0  0  0.174  0.112  0  0.83  0.854  0  0  0  0  0  0  0  0  0.638  0  0.318  0.367  0.072  0  0  0.211  0  0  0.855  0  0  0.785  0.316  0.119  0.675  0  0.336  0  0  0  
 
 UGT1A1  54658  2  234668918  234681945  2q37  0  0.034  0.072  0  0  0  0  0  0.069  0.043  0.047  4.358  2.398  0  0  1.241  1.36  0  0  0  0  0.053  0  0  0  0  0.028  0  0  0.021  0  0  0  0.145  0.797  0.088  0.354  0  0.044  0  0.355  0.08  0  0.14  0  0  0  0  0.489  0  4.652  0.121  0.278  0.129  0  0.213  0.067  0  0  0  
 
 MROH2A  339766  2  234684324  234742069  2q37.1  0  0.037  0.029  0  0.03  0  0  0.02  0.089  0.028  0.014  0.035  0.052  0.02  0  0.073  0.049  0.042  0.014  0.03  0  0.068  0.097  0  0  0.022  0.05  0.055  0.031  0.053  0  0.031  0  0.127  0.079  0  0.038  0  0.038  0  0.019  0.075  0.022  0  0  0.058  0.013  0  0  0  0.085  0.245  0.022  0.074  0.038  0.04  0.02  0  0.038  0  
 
 HJURP  55355  2  234745346  234763212  2q37.1  2.832  3.975  2.858  2.908  2.33  2.511  4.173  2.708  1.505  3.834  4.634  2.204  2.494  3.789  3.384  3.168  3.738  3.071  4.942  2.544  2.533  3.585  1.801  1.593  3.253  3.165  3.624  3.622  4.002  2.88  3.785  4.072  4.246  3.001  4.137  2.726  4.314  3.369  3.54  4.057  3.344  3.723  3.244  2.822  2.299  2.855  3.075  2.787  2.988  3.904  3.894  2.456  3.718  3.058  4.598  2.697  3.749  3.505  3.837  3.552  
 
 MSL3P1  151507  2  234774089  234777055  2q37.1  2.231  2.884  0.847  0.677  0.08  0  0.109  0.637  0.126  0.089  0.164  1.782  1.486  2.963  1.409  2.451  2.501  3.008  2.942  1.233  1.822  2.499  0.859  0.301  1.568  0.153  1.8  2.412  2.099  0.406  2.394  1.961  1.895  1.681  1.892  2.931  1.253  0.76  0.252  1.256  2.511  0.586  1.552  1.44  0.465  0.947  1.105  1.722  0.975  3.561  2.184  0.216  0.072  1.719  1.819  1.609  1.583  0.817  2.456  1.517  
 
 TRPM8  79054  2  234826042  234928166  2q37.1  0  0.021  0.129  0  0.115  0.045  0.057  0.111  0.365  0.328  0.3  0.024  0  0.042  0  0.036  0.05  0.052  0.014  0.028  0  0.076  1.047  0.137  0.026  0.213  0.246  1.069  0.572  0.364  0.608  1.967  1.123  1.079  0  0.036  0.028  0.105  0.05  0  0.033  0.259  0.042  0  0.045  0.09  0.038  0  0  0.027  0.029  0.257  0.139  0.153  0.12  0.043  0.09  0.056  0.047  0.289  
 
 SPP2  6694  2  234959345  234985776  2q37.1  0  0  0  0  0  0  0  0  0  0  0  0  0  0  0  0  0  0  0  0  0  0  0  0  0  0  0.051  0  0  0.063  0  0  0  0  0  0  0  0  0  0  0  0.08  0  0  0  0  0  0  0  0  0  0  0  0  0  0  0  0  0  0  
 
 ARL4C  10123  2  235401685  235405697  2q37.1  0.389  2.351  3.035  3.265  0.358  1.361  3.345  2.076  6.82  2.961  5.116  5.531  0.809  1.124  2.663  2.606  2.313  0.645  6.777  0.35  0  4.599  1.168  1.239  4.087  2.158  1.319  1.502  1.035  0.8  0.721  1.729  1.492  1.738  3.473  4.105  1.424  3.482  2.333  2.171  3.02  2.522  0.217  2.147  1.924  1.639  3.353  1.033  2.532  2.797  2.891  1.148  4.669  3.378  5.788  4.389  4.494  2.045  5.116  5.109  
 
 SH3BP4  23677  2  235860627  235964358  2q37.1-q37.2  1.945  3.431  3.358  1.923  2.265  3.359  4.309  4.775  4.872  3.693  4.937  2.906  2.507  2.884  4.532  2.812  3.541  2.953  0.039  0.069  0.085  0.54  0.888  0.943  2.068  2.982  4.099  3.072  4.868  3.604  4.239  3.188  4.155  2.316  2.486  3.342  4.196  3.961  3.045  2.003  3.477  1.829  0.601  3.007  3.346  3.577  2.673  2.29  3.025  2.738  2.392  2.124  4.03  3.204  3.79  2.214  3.651  2.21  3.866  4.692  
 
 AGAP1  116987  2  236402732  237040444  2q37  1.97  1.393  1.097  1.16  1.965  1.539  2.056  2.356  2.221  2.252  2.485  2.236  2.107  2.328  2.551  1.618  2.081  1.661  0.091  0.254  1.679  0.506  1.454  1.673  1.632  1.72  1.959  2.492  2.482  2.11  1.957  2.226  2.391  1.323  1.592  2.921  2.16  1.738  1.222  2.405  3.041  1.441  2.145  2.24  2.029  1.707  2.237  1.757  2.991  1.514  2.191  2.168  2.229  1.295  2.153  1.499  1.998  1.921  2.461  1.893  
 
 AGAP1-IT1  100506749  2  236414394  236416210  2q37.2  0.859  0.176  0.513  0  0.729  0.273  1.422  1.011  0.455  0.781  0.834  1.522  0.817  1.276  1.398  0.901  0.663  0.444  0  0  0.735  0.123  1.124  0.758  0  0.555  1.042  0.697  1.084  0.549  0.772  0.98  0.889  0.524  0.387  0.969  0.902  0.62  0.155  0.372  1.129  0.808  1.434  0.849  0.446  0.488  2.737  0.542  0.784  0.472  0.65  1.217  0.984  0.594  1.23  0.71  0.835  0  1.515  0  
 
 GBX2  2637  2  237073878  237076652  2q37.2  0.331  0.063  1.505  0.185  0.127  1.323  0  0.537  0.942  0.119  0.527  0.113  0.113  1.352  0  0.102  0.104  0.992  0  0.136  0  0.421  0.201  0.144  0.528  0.12  0.267  0.651  0.667  0.385  0.731  0.809  0  0  0.56  0.596  0.669  0  0.856  0.845  0.502  1.152  5.444  0.686  0  0.221  0.076  1.252  0.372  2.185  0.431  1.371  0.3  0.317  1.291  0.276  0.19  0.802  0  0.448  
 
 ASB18  401036  2  237103514  237172988  2q37.2  0.192  0.097  0.312  0  0.189  0.127  0.067  0.105  0  0.122  0.087  0.313  0  0.302  0  0.117  0.081  0.345  0  0.18  0  0.566  0  0  0.141  0  0.231  0.108  0.11  0.226  0.093  0  0  0.186  0  0  0.12  0  0.17  0  0.26  0.677  0.211  0.108  0.171  0.154  0.081  0.322  0  0.101  0.103  1.544  0.12  0.281  0.855  0  0.089  0.141  0  0  
 
 IQCA1  79781  2  237232789  237416178  2q37.3  0  0  0.116  0  0.028  0  0.05  0.036  0  0.038  0.036  0  0  0.24  0  0.044  0  0.184  0.067  0.05  0  0.087  0.116  0  0  0  0.091  0.025  0  0.091  0  0  0  0.156  0.049  0  0.771  0  0.437  0  0  0.236  2.279  0.059  0.978  0.099  0.053  1.301  0  2.453  0.069  0.675  0.078  0.111  0.371  0  0.383  0  0  1.721  
 
 ACKR3  57007  2  237478379  237490994  2q37.3  6.213  0.036  0.726  2.904  0.101  0.794  0.379  6.202  2.929  2.811  2.766  0.198  0  0.045  0  0.102  0  0  2.382  0  0  0.429  0.2  0.11  0  0  0.112  0.069  0  0.091  0  0  0  0.334  0.348  0.339  0.07  0  1.805  0.368  0  1.109  0.411  0.533  0.368  0.103  0.246  0.479  0  0.722  0.073  0.857  1.981  3.373  0.031  0  4.292  0  0  0.597  
 
 COPS8  10920  2  237994083  238007489  2q37.3  2.155  4.299  2.585  3.777  3.229  2.908  5.041  4.059  3.257  3.913  4.109  2.418  2.926  3.731  2.462  2.54  3.122  4.831  4.985  3.25  1.973  3.151  2.351  2.31  3.464  4.562  3.246  2.74  4.362  2.479  4.641  4.124  3.944  3.356  2.777  3.464  3.199  3.237  3.955  3.255  2.906  2.605  4.003  2.837  2.114  2.355  3.557  2.605  2.261  4.514  3.834  1.696  2.738  2.547  4.2  2.637  2.789  3.037  3.494  3.454  
 
 COL6A3  1293  2  238232654  238322850  2q37  0.155  0.521  6.298  0.567  0.154  2.646  5.782  6.039  0.164  4.348  0.527  0.086  0  0.1  0.043  0.16  0.076  0.183  0.029  0.079  0.086  0.284  0.19  0  1.236  1.355  0.335  0.51  0.474  0.22  0.149  0.051  0.036  0.41  0.185  0.027  3.984  0.826  0.139  0  0.055  0.499  0.061  0  0.057  0.055  0.852  0.053  0  0.064  2.879  0.853  3.69  0.163  0.301  3.417  1.479  0.034  0  0.067  
 
 MLPH  79083  2  238395052  238463961  2q37.3  3.685  2.619  1.068  2.34  4.215  1.767  5.262  3.128  0.231  3.899  0.033  3.099  0.124  3.446  0.789  2.895  2.316  0.076  0.082  0.088  0  0.209  0.14  0  0  5.419  4.528  2.945  5.483  3.915  5.461  3.374  3.912  3.233  3.803  3.073  4.274  3.072  2.106  0.229  3.478  2.98  0.138  0.085  0.43  0.06  7.576  1.504  1.665  3.525  3.746  0.904  1.492  1.63  3.196  2.422  1.513  1.961  0  3.282  
 
 PRLH  51052  2  238475216  238475818  2q37.3  0  0  0  0  0.578  0  0  0  0  0  0  0  0  0  0  0  0  0  0  0  0  0  0  0  0  0  0.238  0  0  0.199  0  0  0  0  0  0  0  0  0  0  0  0  0  0  0  0  0  0  0  0  0  1.619  0  0.45  0  0  0  0  0  0  
 
 RAB17  64284  2  238482964  238499769  2q37.3  2.244  0  0.11  0  3.546  0  0.126  0.047  1.046  0.206  1.351  1.482  2.24  2.387  2.729  2.103  1.493  0.245  0  0.059  0.18  0.166  0.555  0.274  0  3.623  2.469  2.87  3.764  3.827  4.153  2.518  0.086  0.299  0  0.422  0.531  0.282  0.258  0.606  2.14  0.675  0.104  3.149  1.758  2.757  0.652  0  0.104  0.158  0.497  1.116  0  0.098  1.915  0.076  0.089  0  0.234  2.07  
 
 LRRFIP1  9208  2  238536223  238690290  2q37.3  3.833  5.26  3.808  3.042  3.92  3.031  5.317  4.988  2.943  3.206  4.894  3.662  3.708  4.744  3.815  3.835  4.541  4.791  5.021  4.951  2.751  4.011  3.516  3.338  4.234  3.671  4.465  3.582  4.518  3.801  4.839  4.624  4.775  3.579  3.405  4.194  4.451  4.167  5.215  3.711  3.358  3.049  4.275  5.127  2.53  3.676  5.657  3.315  3.697  4.222  4.401  3.012  4.748  3.162  4.942  3.398  3.69  3.34  4.838  5.352  
 
 RBM44  375316  2  238707387  238751451  2q37.3  0  0.046  0.036  0  0.048  0.03  0.034  0.077  0.06  0.031  0.34  0  0.072  0.099  0  0.04  0.101  0.156  0.108  0.061  0  0.083  0.082  0.058  0.055  0.06  0.045  0  0  0.032  0.055  0.052  0  0.139  0.053  0.207  0.021  0.067  0.324  0  0.063  0.124  0.02  0.201  0  0.03  0.023  0  0.11  0.095  0.148  0.073  0.018  0.016  0.023  0  0.059  0  0  0.054  
 
 RAMP1  10267  2  238768186  238820759  2q36-q37.1  3.171  0.666  3.004  5.709  0  0.885  2.534  0.877  0.457  4.166  2.82  0  4.404  3.916  3.406  3.034  4.806  0.537  0  0.412  0  0.241  0.462  0  0  5.414  0.133  0.144  0  0.231  0.154  0  0  0  0  0.24  1.105  4.672  2.633  0  0  0.87  0.062  0  0  0.47  0.704  1.843  0  2.077  4.713  1.004  0  0.132  0.166  0.162  0.702  5.378  0  0.201  
 
 UBE2F  140739  2  238875586  238951423  2q37.3  2.648  3.89  2.885  2.956  2.342  2.035  3.993  3.587  2.921  2.675  3.623  1.978  2.52  3.109  1.897  2.061  2.242  4.113  3.858  2.788  2.447  2.936  2.167  1.796  2.633  2.734  2.494  2.431  2.828  3.073  3.267  3.183  2.938  1.945  1.988  2.677  2.755  3.199  4.484  2.592  2.475  1.518  4.16  2.377  2.044  1.794  3.603  1.816  1.868  3.194  3.447  1.253  2.257  1.748  2.732  1.799  2.024  2.924  2.12  2.286  
 
 UBE2F-SCLY  100533179  2  238875586  239008054  -  0  0  0  0  0  0  0.212  0.127  0.22  0.244  0  0.101  0.159  0.077  0.088  0  0  0  0.19  0  0.377  0  0  0  0  0.085  0.075  0  0  0.137  0.072  0  0  0  0  0.187  0  0  0.043  0  0  0  0.075  0  0  0  0  0  0  0  0  0.373  0  0.078  0  0  0  0  0  0  
 
 SCLY  51540  2  238969564  239008054  2q37.3  1.692  1.27  1.353  1.846  0.89  1.374  2.898  1.986  1.999  1.612  2.471  1.86  2.969  2.469  2.568  1.447  2.324  1.917  3.461  1.845  1.431  2.164  1.545  1.969  1.062  2.385  3.169  2.462  2.95  3.627  2.496  2.805  3.634  1.794  1.882  1.84  2.335  2.396  1.356  2.123  1.794  2.576  2.315  1.358  1.191  1.313  1.925  1.534  0.736  2.044  1.306  1.078  1.763  1.139  2.183  1.486  1.128  2.282  1.911  1.657  
 
 ESPNL  339768  2  239008950  239041928  2q37.3  0.068  0.07  0.183  0  0.047  0.064  0.029  0.091  0.175  0.078  0.079  0.094  0  0.06  0  0.112  0.071  0.237  0.393  0.08  0  0.213  0.387  0.082  0.066  0.084  0.194  0.065  0  0.165  0.055  0.031  0  0.173  0.049  0.042  0  0.076  0.159  0.344  0.051  0.499  0.098  0.068  0.103  0.103  0.07  0.98  0  2.303  0.062  0.858  0.095  0.877  0.132  0.149  0.058  0.09  0  0  
 
 KLHL30  377007  2  239047362  239061547  2q37.3  0  0.038  0.225  0  0.023  0.034  0.027  0.046  0.177  0.082  0.054  0.071  0.038  0.147  0.061  0.381  0.067  0.131  0.029  0.047  0.067  0.323  0.507  0.109  0  0.47  0.167  0.042  0.041  0.143  0  0  0  0.224  0.11  0.38  0.039  0  0.116  0.05  0.268  0.296  0.069  0.397  0.099  0.423  0.117  0.091  0  0.502  0.057  0.634  0.043  0.173  0.348  0.104  0.043  0  0.049  0.063  
 
 FAM132B  151176  2  239067648  239077532  2q37.3  1.139  1.229  1.957  2.604  0.103  0.676  1.001  2.162  1.292  2.121  2.325  0.165  0.157  1.811  0.843  0.749  0.825  0.429  0.244  0.047  0.506  0.285  0.433  0.138  0.569  1.333  0.363  0.345  1.229  2.095  3.293  1.035  0.948  0.719  0.666  1.27  0.663  0.415  1.719  1.83  1.097  0.896  2.26  1.403  0.77  1.732  2.48  0.948  0.816  2.503  0.645  0.823  1.468  1.697  1.871  0.463  0.852  0.787  1.473  1.107  
 
 ILKAP  80895  2  239079042  239112324  2q37.3  2.645  2.559  2.27  2.486  2.607  2.515  4.156  3.542  3.022  2.94  3.561  2.324  3.095  3.97  3.035  2.603  2.587  1.877  5.117  3.453  2.464  3.909  2.062  1.706  2.264  3.07  3.487  3.008  3.687  3.693  4.323  3.804  3.329  1.764  2.598  2.734  3.121  2.532  3.398  3.351  2.616  3.163  4.014  2.455  2.474  2.031  3.651  2.57  2.525  4.261  1.954  1.872  2.869  1.713  3.329  1.814  1.9  3.011  2.679  2.52  
 
 LOC151174  151174  2  239133753  239140318  2q37.3  0  0  0.167  0  0  0  0  0  0  0.095  0.15  0  0  0.114  0  0.182  0.104  0  0.315  0.863  1.026  0.231  0.545  0  0  0.387  0.601  0.198  0.249  0.361  3.224  0.807  0.394  0.252  0  0  0  0.521  0.258  0.695  0  0.07  0.175  0.131  0  0.082  0.034  0  0  0  0  0.665  0  0.181  0.233  0.244  0.127  0.799  0  0  
 
 LOC643387  643387  2  239140326  239142985  2q37.3  1.783  2.031  1.111  1.238  1.074  0.853  1.99  0.973  1.054  1.136  1.35  0.853  1.24  1.393  1.472  1.3  1.249  2.45  2.081  2.343  0.567  2.401  1.184  0.745  0.928  0.832  1.765  1.146  1.139  1.034  1.084  1.074  1.115  0.92  0.993  0.609  1.622  0.731  1.838  0.985  0.876  1.033  1.848  0.665  0.525  0.632  2.96  0.95  0.468  1.579  1.068  0.492  1.768  0.656  1.612  1.071  0.814  0.962  1.131  0.782  
 
 HES6  55502  2  239146907  239148765  2q37.3  2.208  1.987  2.066  5.759  2.282  1.992  2.34  2.67  4.819  2.692  4.819  1.483  3.308  3.942  2.227  1.438  2.64  5.816  2.824  5.67  4.005  2.399  1.506  1.973  1.002  4.032  3.062  2.406  4.338  5.278  6.432  4.174  3.713  2.909  1.966  2.994  0.882  1.125  2.911  2.104  1.028  3.25  3.521  2.761  0.763  2.214  3.757  1.606  1.208  3.351  1.167  1.798  1.647  1.17  1.975  1.212  0.947  2.212  1.927  1.061  
 
 PER2  8864  2  239152678  239197207  2q37.3  1.297  0.561  0.429  0.2  1.863  0.761  1.224  2.913  0.843  1.323  1.324  1.306  1.077  1.733  0.82  0.8  1.611  1.84  2.07  1.768  1.168  2.219  0.459  0.469  0.806  0.496  0.694  0.449  0.746  0.74  0.78  0.665  0.685  0.379  1.079  2.115  1.156  0.776  0.754  1.276  1.601  0.887  1.416  1.171  0.724  0.589  0.345  0.274  1.113  0.206  0.983  0.484  0.813  0.245  0.428  0.659  0.716  0.272  0.846  0.433  
 
 TRAF3IP1  26146  2  239229184  239309541  2q37.3  0.945  1.04  0.632  0.761  1.099  1.126  1.613  1.798  1.198  1.451  2.078  0.796  0.908  1.552  1.674  0.73  1.161  1.234  1.708  0.768  0.622  0.977  0.451  0.426  1  1.265  0.977  0.996  1.476  0.933  1.256  1.303  1.337  0.66  1.42  1.569  1.427  1.302  1.344  1.576  1.115  0.972  1.802  1.22  1.103  0.899  1.342  1.188  1.324  1.557  1.452  0.774  1.37  1.062  1.562  0.863  1.098  1.416  1.581  1.593  
 
 ASB1  51665  2  239335625  239360891  2q37  1.168  1.255  1.98  1.112  0.99  1.992  2.069  2.358  2.166  1.434  2.921  0.815  1.226  1.501  1.437  1.156  1.561  0.475  2.693  0.844  1.278  1.364  0.861  0.425  1.771  1.488  1.221  2.126  1.715  1.531  1.565  1.701  1.862  1.187  1.29  1.74  2.931  1.649  2.456  1.883  1.44  1.414  1.688  1.091  1.133  0.866  2.065  1.313  1.441  1.971  2.153  0.972  1.572  1.093  1.756  1.033  1.154  1.694  1.584  1.142  
 
 LINC01107  151171  2  239419330  239464140  2q37.3  0  0  0.135  0  0.032  0.063  0  0.04  0.132  0.057  0.165  0  0  0.058  0  0  0  0  0.028  0.091  0  0.144  0.361  0  0  0  0.076  0.049  0  0.078  0  0  0  0.406  0  0.183  0  0  0.165  0  0.196  0.213  0.04  0  0  0  0.036  0  0.103  0  0.077  0.228  0.031  0.092  0.092  0.07  0  0  0  0  
 
 TWIST2  117581  2  239756672  239832244  2q37.3  0  0.192  3.028  3.64  0.178  0.881  2.669  2.381  0.147  1.777  0.069  0.171  0  0.096  0  0.231  0  0.684  0.078  0.444  0  0.723  0.304  0.142  0  0  0.36  0.093  0  0.699  1.121  2.181  0  0.38  1.657  0.186  1.68  0  3.691  1.867  0.505  2.849  0.18  0  0  0.114  0.144  1.786  1.872  3.306  0  2.625  0  0.461  2.065  0.197  0  1.647  0  0.63  
 
 FLJ43879  401039  2  239840997  239847965  2q37.3  0  0.026  0.16  0  0.04  0.121  0.037  0  0.075  0.196  0  0.046  0  0.033  0  0.039  0.042  0.095  0  0.041  0  0.078  0.158  0  0  0  0.107  0  0  0.097  0  0  0  0.174  0.028  0  0  0  0.048  0  0  0.068  0.038  0  0  0.022  0.026  0  0  0.03  0.359  0.376  0  0.082  0.027  0  0  0  0  0  
 
 HDAC4  9759  2  239969863  240322643  2q37.3  0.289  0.79  0.591  0.809  0.329  0.406  0.991  1.387  1.678  1.746  1.232  2.523  1.337  1.2  1.107  0.693  1.076  3.617  2.482  1.415  0.612  3.356  0.624  0.589  0.265  1.586  1.929  1.45  2.159  1.035  1.82  1.422  2.013  0.92  0.902  1.934  1.106  1.027  0.721  1.572  0.764  1.672  1.04  0.566  0.265  0.483  1.211  0.368  0.951  0.193  0.977  1.129  0.582  0.764  0.735  0.633  0.904  0.6  0.93  0.663  
 
 MGC16025  85009  2  240115026  240117153  2q37.3  0  0.054  0.167  0  0.052  0  0.043  0.22  0.309  0.341  0.205  0.108  0.223  0.194  0.221  0.102  0.088  0.203  0.194  0.112  0  0.728  0.325  0.051  0  0.821  0.471  1.868  1.663  1.449  1.8  0.913  0.887  0.529  0.135  0.213  0.089  0.162  0.053  0  0.065  0.267  0.071  0  0  0.064  0.053  0.07  0  0  0.133  0.576  0.042  0.101  0.073  0.071  0.161  0.121  0.065  0  
 
 LOC150935  150935  2  240684553  240722356  2q37.3  0  0  0.136  0  0  0  0  0  0  0.072  0  0  0  0  0  0  0  0  0  0  0  0  0.289  0  0  0  0.061  0.089  0  0.072  0  0  0  0.337  0  0  0.095  0  0  0  0  0  0  0  0  0.091  0  0  0  0  0  0.201  0  0  0  0  0  0  0  0  
 
 NDUFA10  4705  2  240896788  240964819  2q37.3  2.331  2.41  1.739  1.899  3.275  2.35  3.322  3.022  2.727  2.376  3.127  2.758  3.035  3.403  3.036  2.689  2.659  1.824  3.823  3.845  2.271  3.118  1.897  1.83  2.153  2.704  2.269  2.535  3.129  2.507  3.13  3.479  3.096  2.001  2.757  2.761  2.68  2.516  3.131  3.109  2.587  2.507  3.228  2.499  2.115  2.377  3.524  2.395  2.455  3.92  3.104  1.614  2.67  1.816  3.521  1.978  1.892  2.789  2.847  2.818  
 
 OR6B2  389090  2  240968907  240969846  2q37.3  0  0.129  0.176  0  0  0  0  0  0.155  0.08  0  0.13  0  0  0  0  0  0  0  0  0  0.116  0.384  0  0  0  0.167  0  0  0.148  0  0  0  0.272  0  0  0  0  0  0  0  0.301  0  0  0  0  0.238  0  0  0  0  0.441  0  0.102  0  0  0  0  0  0  
 
 PRR21  643905  2  240981229  240982399  2q37.3  0  0  0.245  0  0  0  0  0  0  0  0  0  0  0  0  0  0  0  0  0  0  0  0  0  0  0  0.033  0  0  0.034  0  0  0  0.192  0  0  0  0  0  0  0  0  0  0  0  0  0  0  0  0  0  0  0  0  0  0  0  0  0  0  
 
 OR6B3  150681  2  240984493  240985489  2q37.3  0  0  0.241  0  0.509  0  0  0.113  0.157  0.408  0.264  0.17  0.266  0.203  0  0.258  0.158  0  0.201  0.145  0  0.521  0  0  0  0  0.191  0.323  0.294  0.165  0  0  0  0  0.43  0.12  0  0  0.32  0.175  0  0.288  0.076  0  0  0  0.274  0.156  0  0.113  0.136  0.724  0.255  0.196  0.411  0  0  0  0.176  0  
 
 MYEOV2  150678  2  241065979  241075764  2q37.3  5.203  8.016  4.591  4.33  6.263  6.957  6.896  6.31  5.485  5.939  6.283  5.941  4.644  6.359  4.155  5.597  5.094  8.257  7.792  7.893  5.133  7.332  4.53  5.264  6.044  4.749  5.34  4.749  5.575  4.564  6.123  6.45  5.589  4.935  6.684  5.79  4.967  5.169  8.014  5.252  5.674  6.238  6.849  6.047  5.94  5.621  7.559  5.088  5.829  7.524  5.224  4.176  5.385  5.304  6.471  5.431  4.017  6.361  5.868  5.322  
 
 OTOS  150677  2  241078445  241080073  2q37.3  0  0  0  0  0  0  0  0  0  0  0  0  0  0  0  0  0  0  0  0  0  0.108  0.582  0  0  0  0.188  0  0  0.221  0  0  0  0  0  0  0.225  0  0  0  0  0.286  0  0  0  0  0.404  0  0  0  0  0  0  0  0  0  0  0  0  0  
 
 GPC1  2817  2  241375114  241407495  2q35-q37  1.061  0.357  3.787  2.916  2.302  3.535  5.096  5.575  5.376  6.903  4.241  1.126  4.261  3.628  3.8  3.239  1.748  0.208  0.736  0.306  0.225  1.045  1.163  1.947  1.887  3.233  3.943  3.723  3.731  2.481  2.75  4.139  4.503  2.891  3.888  4.55  4.443  4.656  4.496  4.174  4.335  6.052  2.974  3.385  2.222  2.441  4.108  3.546  2.33  4.225  3.293  4.483  3.147  4.514  3.356  1.275  4.01  4.781  3.674  4.165  
 
 PP14571  100130449  2  241388835  241396117  2q37.3  0.249  0  0.265  0  2.93  0.596  0.107  0.25  0.17  0.681  0.29  0.084  0.215  0.3  0.424  0.164  0.065  0.101  0  0.101  0  0.131  0.47  0.063  0  0.151  0.398  0.144  0.166  0.245  0.087  0.071  0.169  0.361  0.406  0.201  0.214  0.375  0.354  0.148  3.305  0.496  0.116  1.152  0.41  0.367  0.076  0.326  0.309  0.108  0.197  0.461  0.137  0.325  0.204  0.127  0.289  0.307  0.146  0  
 
 ANKMY1  51281  2  241418838  241500527  2q37.3  0.942  0.316  0.529  0.654  0.817  0.613  1.086  1.113  1.07  1.436  0.917  0.986  1.544  1.168  1.436  1.294  1.149  0.502  1.982  1.091  0.822  1.526  0.863  0.801  0.549  1.088  1.081  1.274  1.097  0.938  1.238  0.925  1.183  0.506  1.051  0.774  1.447  0.886  0.68  0.694  1.168  1.465  0.872  0.21  0.514  0.652  1.592  0.538  0.568  0.505  0.804  1.124  1.108  0.641  1.352  0.491  0.73  0.5  1.088  0.521  
 
 DUSP28  285193  2  241499470  241503431  2q37.3  0.474  1.043  0.528  0.851  1.44  1.276  0.929  1.017  1.003  1.037  1.032  0.955  0.732  1.342  0.899  1.031  1.042  2.326  2.303  1.732  0.509  1.71  1.045  0.649  0.737  0.693  1.082  0.601  1.258  0.639  1.228  0.976  1.029  0.977  1.203  0.761  0.6  0.735  1.822  0.752  1.344  1.527  2.071  0.337  0.374  0.903  1.057  0.425  0.624  1.258  0.563  1.42  0.678  0.682  1.435  0.418  0.211  0.853  0.867  0.603  
 
 RNPEPL1  57140  2  241508003  241518149  2q37.3  1.905  0.158  3.219  3.289  2.816  2.351  4.17  4.253  4.796  3.749  4.291  3.548  3.881  2.985  3.956  3.666  3.43  0.16  4.987  2.371  2.347  2.616  2.909  3.228  2.72  3.714  4.223  3.927  4.549  4.61  4.559  3.458  4.407  2.977  3.269  4.592  3.811  3.329  2.494  3.429  4.221  4.405  3.882  1.886  2.249  2.759  2.896  3.436  2.951  4.381  3.413  3.221  3.345  2.777  3.996  3.309  2.558  4.141  3.915  3.108  
 
 CAPN10-AS1  101752400  2  241522166  241525733  2q37.3  0.445  0.453  0.283  0.175  0.604  0.154  0.758  0.589  0.379  0.704  0.634  0.336  0.707  0.488  0.712  0.645  0.293  0.709  1.074  0.459  0.452  0.55  0.69  0.421  0.179  0.634  0.414  0.781  0.65  0.585  0.497  0.676  0.947  0.402  0.471  0.746  0.567  0.698  0.68  0.736  0.538  0.669  1.155  0.467  0.388  0.3  0.701  0.666  0.527  0.82  0.654  0.635  0.533  0.376  0.843  0.484  0.491  0.522  0.675  0.431  
 
 CAPN10  11132  2  241526132  241538526  2q37.3  0.671  0.284  1.149  1.538  1.234  1.456  1.971  1.783  2.124  2.017  2.154  1.564  1.781  1.784  1.67  1.806  1.646  0.319  3.654  1.903  0.809  2.474  1.938  1.566  1.19  1.648  1.802  1.82  2.297  1.22  2.2  2.009  2.327  0.982  1.66  2.162  2.161  1.88  1.844  2.134  1.825  2.658  2.048  1.027  1.031  1.145  1.444  1.692  1.061  2.682  1.292  1.475  1.421  1.053  2.362  1.141  1.056  1.81  1.896  1.57  
 
 GPR35  2859  2  241544824  241570676  2q37.3  0.122  0  0.24  0.603  0.175  0.358  0.352  0.346  0.513  1.054  0.41  3.178  3.702  0.27  2.497  2.634  2.734  0.386  1.156  0.304  0  0.43  0.268  0.087  0.128  0.061  0.467  0.196  0.129  0.416  0.149  0.226  0.113  0.213  0.277  0.385  0.255  0.168  0.299  0.458  0.251  0.528  0.395  0.104  0  0.254  0.086  0.334  0  0.756  0.349  0.832  0.128  0.523  0.428  0.163  0.297  0  0.157  0.082  
 
 AQP12B  653437  2  241615834  241622317  2q37.3  0  0  0.209  0  0  0  0  0  0.132  0.076  0  0  0  0.053  0  0  0  0  0  0  0  0.194  0.302  0  0  0  0.178  0.08  0  0.131  0  0  0  0.231  0  0  0  0  0.066  0  0  0.38  0.119  0  0  0  0  0  0  0  0  0.54  0  0.103  0.081  0  0  0  0  0  
 
 AQP12A  375318  2  241631261  241637900  2q37.3  0  0  0.182  0  0  0  0  0  0.152  0.054  0  0  0  0  0  0  0  0  0  0  0  0.128  0  0  0  0  0.176  0  0  0.132  0  0  0  0.208  0  0  0  0  0.061  0  0  0.084  0.068  0  0  0  0  0  0  0  0  0.58  0  0.147  0.074  0  0  0  0  0  
 
 KIF1A  547  2  241653180  241759725  2q37.3  1.037  0.03  0.117  0.605  0.074  0.069  0.056  0.057  0.204  0.144  0.072  0.065  0.041  0.074  0.057  0.064  0.085  0.139  0.034  0.07  0.073  0.133  0.32  0.262  0.018  0.138  0.285  0.144  0.045  0.478  0.409  0.212  0.021  0.163  0.136  0.052  0.087  0  0.092  3.945  0.196  0.327  5.497  0.528  0.239  1.358  0.087  0.038  0.159  0.022  0.059  0.627  0.036  0.155  0.087  0.089  0.047  0.284  0.021  0.018  
 
 AGXT  189  2  241808161  241818536  2q37.3  0  0  0.131  0  0.104  0.102  0.121  0  0.149  0.076  0.065  0.2  0  0.055  0  0.076  0.1  0.075  0.064  0.104  0  0.281  0.315  0  0  0  0.206  0  0  0.241  0  0.12  0  0  0  0  0  0  0.097  0  0  0.213  0.132  0  0  0.115  0.388  0  0  0  0.063  0.75  0  0.178  0.089  0  0  0  0  0  
 
 C2orf54  79919  2  241825464  241835573  2q37.3  0.164  0.046  0.156  0  0.608  0.096  0.156  0.05  0.218  0.116  0.045  0.845  0.615  0.056  3.116  1.45  0.092  0.483  0.083  0.056  0  0.252  0.437  0.173  0  0.076  0.312  0.047  0  0.21  0  0.107  0  0.25  0  0.223  0  0  0.074  0  3.789  0.448  0.135  0.252  0.395  1.187  0.419  0  0  0.143  0.058  1.033  0.04  0.184  0.095  0.071  0  0  0.182  0  
 
 LOC200772  200772  2  241894035  241906868  2q37.3  0.243  0  0.186  0  0  0.048  0.074  0.043  0.107  0.352  0  0  0  0.107  0  0.051  0  0.092  0  0.097  0  0.137  0.472  0.937  0  0.06  0.352  0.033  0.123  0.13  0  0  0  0.163  0  0  0  0  0.06  0  0  0.184  0.103  0  0  0.049  0.024  0  0  0  0.046  0.383  0  0.065  0.07  0  0  0  0  0  
 
 SNED1  25992  2  241938254  242033643  2q37.3  0.118  0.069  0.375  1.066  0.282  0.093  0.788  1.293  0.855  3.128  0.179  0.122  0.148  0.448  0.215  0.143  0.106  0.161  0.16  0.132  0.042  0.383  0.18  0.081  0.057  1.703  2.078  0.286  0.629  0.202  0.067  0.796  0.283  0.537  0.081  0.296  0.205  0.168  0.195  0.493  0.215  0.451  0.134  0.135  0.077  0.118  0.611  0.154  0.525  0.185  0.11  0.674  0.093  0.231  0.194  0.05  0.098  0.188  0.102  0.087  
 
 MTERF4  130916  2  242026508  242041747  2q37.3  2.999  2.54  1.523  1.739  2.498  1.456  2.613  2.486  1.975  2.057  2.723  2.156  2.526  3.087  2.707  2.182  2.189  2.81  4.083  2.693  1.647  3.107  1.518  1.224  2.192  1.993  1.822  2.223  2.422  2.497  2.385  2.734  2.948  1.55  1.866  2.713  2.487  2.122  2.47  2.533  2.338  2.258  3.71  2.314  1.67  1.428  2.859  1.952  1.674  3.59  2.074  1.699  2.339  1.579  3.184  1.911  1.603  2.323  2.89  2.339  
 
 PASK  23178  2  242045513  242089394  2q37.3  1.252  0.447  0.543  0.768  0.982  0.578  0.912  1.144  0.286  0.841  1.289  0.654  1.2  1.332  1.85  1.175  1.024  0.933  2.74  1.274  1.252  1.56  0.451  0.643  0.603  1.304  1.065  1.433  1.459  1.336  1.347  0.859  1.607  0.684  0.796  1.608  1.377  0.748  0.679  1.557  1.289  1.152  1.935  1.048  0.485  0.768  0.515  0.954  1.077  1.205  0.843  0.995  1.043  0.557  1.182  0.479  0.599  0.861  1.228  1.066  
 
 PPP1R7  5510  2  242088987  242123065  2q37.3  3.019  2.436  2.927  3.036  4.119  3.129  4.796  4.57  3.161  3.92  4.502  3.608  2.858  3.698  2.675  3.432  4.088  2.201  5.819  5.289  2.85  4.61  3.023  2.837  3.381  3.602  3.793  2.603  4.156  2.075  3.901  4.291  3.862  2.83  3.102  3.986  3.174  3.338  4.686  2.835  3.856  3.549  4.179  3.461  2.902  3.421  4.572  2.802  2.768  4.644  3.182  2.328  3.114  3.008  4.369  2.785  2.777  3.451  3.389  3.774  
 
 ANO7  50636  2  242127923  242164791  2q37.3  0.381  0.092  0.535  0.32  0.264  0.459  0.35  0.774  0.51  0.427  0.841  0.146  0.745  0.278  0.444  0.731  0.16  0.238  0  1.813  0.478  0.291  0.714  0.115  0.412  0.71  0.545  0.544  0.611  0.619  0.565  0.897  0.739  0.616  0.307  0.675  0.3  0.307  0.57  0.654  0.492  1.095  1.023  0.343  0.51  0.459  0.069  0.287  0.457  0.705  0.58  1.126  0.5  0.516  0.689  0.246  0.384  0.239  0.292  0.214  
 
 HDLBP  3069  2  242166681  242255115  2q37.3  4.448  3.308  4.791  3.748  4.246  4.108  5.689  6.513  4.863  5.361  5.332  4.016  4.817  4.897  5.137  3.929  4.631  1.823  4.126  3.659  3.409  3.38  3.063  3.234  4.044  4.923  4.755  4.965  5.625  4.779  4.945  5.61  5.159  3.126  3.822  5.668  5.469  5.355  4.268  5.628  4.89  3.668  5.184  4.824  4.161  4.155  4.474  4.322  4.595  5.283  4.983  3.845  4.574  3.865  4.985  4.861  4.833  4.281  4.868  5.329  
 
 SEPT2  4735  2  242254601  242293441  2q37  4.066  5.908  4.626  4.497  3.991  4.121  5.442  5.445  5.699  5.327  5.649  3.924  4.974  5.784  4.777  4.191  4.907  5.96  5.89  5.232  3.532  4.143  3.872  2.906  4.406  4.852  4.211  4.428  4.935  3.811  4.843  5.401  4.52  3.517  4.26  5.875  4.86  4.47  5.546  5.594  4.309  4.34  5.597  4.806  3.378  4.027  5.734  3.88  4.729  6.107  5.065  3.592  4.619  3.912  5.544  4.168  4.576  4.469  5.158  5.427  
 
 FARP2  9855  2  242295663  242434257  2q37.3  0.851  0.628  0.795  0.397  0.972  1.051  1.56  2.158  1.022  1.681  2.066  1.623  1.656  1.416  2.094  1.648  1.722  0.251  1.781  0.49  1.078  0.65  1.136  1.304  0.954  3.405  2.797  2.639  3.67  2.911  3.633  3.662  3.742  1.839  1.193  2.147  1.252  2.076  0.928  2.362  1.384  0.965  1.653  1.786  1.301  1.198  0.776  1.345  2.192  1.413  1.816  1.253  1.641  0.975  1.523  1.278  1.622  1.535  1.769  1.515  
 
 STK25  10494  2  242434121  242448987  2q37.3  3.797  2.058  4.153  3.695  2.82  3.518  4.717  4.889  4.276  4.232  4.565  3.016  4.547  4.642  4.523  3.782  3.523  1.37  5.459  3.5  3.509  4.151  2.381  2.41  3.478  3.703  5.056  3.681  4.896  3.798  4.196  4.926  5.035  3.002  3.332  4.853  4.627  4.515  4.116  5.173  3.569  4.249  5.383  3.881  2.979  3.475  4.58  4.883  3.458  6.389  4.219  3.069  4.499  2.703  5.176  3.905  4.088  4.845  5.144  4.351  
 
 BOK-AS1  100379249  2  242483800  242498558  2q37.3  0.475  0.696  0.613  0  0.302  0.336  1.11  0.725  0.316  0.853  0  0.319  0.777  0.242  0  0.343  0.886  0.332  0.49  0.264  0.404  0.72  0  0.166  0  0  0.476  0.722  0  0.539  1.018  0  0  0.679  0.238  0.212  0.609  0  0.488  0.671  0  0.842  0.273  0  0  0.371  0.403  0.345  0.259  0.184  0  1.478  0.306  0.555  0.545  0.239  0.155  0.529  0.493  0  
 
 BOK  666  2  242498145  242513553  2q37.3  1.442  0.28  3.564  2.898  2.636  2.724  0.444  3.675  1.241  2.209  0.421  1.5  2.613  2.239  2.804  2.999  1.849  0.053  0.213  0.121  0.519  0.293  0.286  0.179  2.41  0.869  1.15  2.15  2.582  2.137  2.465  0.636  0.285  0.325  1.85  3.209  3.022  2.402  2.212  3.502  2.727  1.5  3.536  1.673  1.802  2.694  1.405  3.537  2.513  4.894  2.078  2.362  3.038  1.89  3.814  2.405  2.425  2.746  3.694  3.529  
 
 THAP4  51078  2  242523819  242576725  2q37.3  4.03  2.538  3.326  3.606  3.996  3.585  5.149  4.527  3.875  3.716  4.4  3.632  4.236  4.37  3.944  3.937  4.013  2.321  5.5  4.328  3.429  4.407  2.911  3.402  3.649  3.476  4.255  3.427  4.433  4.137  4.425  5.135  4.475  2.575  3.897  4.46  4.422  4.043  4.39  4.606  3.921  4.21  5.106  3.545  3.49  3.349  4.816  3.486  3.637  5.082  3.663  2.928  3.574  2.678  4.723  3.744  2.772  3.817  4.245  3.478  
 
 ATG4B  23192  2  242577026  242613271  2q37.3  2.231  0.704  1.993  2.644  2.463  1.946  2.971  3.23  3.079  2.941  2.933  2.05  2.604  2.633  2.605  2.587  2.72  0.676  4.511  2.31  1.914  3.241  2.023  1.592  1.38  2.538  3.156  2.473  4.027  1.883  2.757  2.888  3.005  1.794  2.092  3.384  2.975  2.445  2.418  3.391  2.538  2.886  3.259  2.117  2.189  2.367  2.599  2.714  2.334  4.39  2.757  2.001  2.039  1.399  3.344  1.59  1.769  2.451  2.671  2.052  
 
 DTYMK  1841  2  242615156  242626383  2q37.3  4.53  4.574  4.158  4.095  4.753  4.987  6.037  5.146  4.017  5.065  5.634  4.803  4.116  5.497  4.149  5.069  5.258  3.834  7.064  5.569  4.066  5.993  3.87  3.451  4.375  4.464  4.947  3.661  5.077  3.958  5.395  5.071  4.963  3.636  5.19  5.139  4.875  4.52  4.944  5.395  5.133  5.914  5.485  3.951  3.924  3.988  6.753  4.447  4.383  6.261  4.343  3.844  4.819  3.7  5.978  4.077  3.688  6.143  5.372  4.444  
 
 ING5  84289  2  242641455  242668896  2q37.3  0.846  1.241  0.596  0.611  0.862  0.722  0.938  1.258  1.522  1.081  1.329  0.58  1.046  1.389  1.903  1.349  1.015  2.064  3.644  1.467  0.991  1.784  0.698  0.504  0.734  1.195  1.133  0.885  1.214  0.878  1.277  1.192  1.484  0.777  0.822  1.49  1.341  1.081  0.939  1.845  1.052  1.581  1.572  1.312  1.293  0.757  1.059  1.364  1.115  2.4  0.728  0.898  0.78  0.565  1.403  0.646  0.963  0.99  1.349  0.876  
 
 D2HGDH  728294  2  242673993  242708231  2q37.3  2.503  0.369  0.923  0.842  1.561  1.265  1.89  2.007  1.694  1.892  1.855  1.835  2.584  1.829  2.625  2.473  2.815  0.308  3.965  2.096  1.635  2.563  1.417  1.555  1.076  2.144  2.406  2.582  2.617  2.214  2.645  2.665  2.369  1.303  2.024  2.87  2.047  1.587  1.588  2.09  1.976  2.987  1.558  1.23  1.582  1.326  2.346  1.813  1.409  3.25  1.843  2.275  1.239  0.979  3.567  1.392  0.77  1.637  1.809  1.296  
 
 GAL3ST2  64090  2  242716239  242743702  2q37.3  0  0.084  0.197  0  0.259  0  0  0.187  0.183  0.242  0.155  0.125  1.349  0.176  2.09  0.157  0.245  0.364  0.069  0.277  0.185  0.814  0.481  0  0.082  0.168  0.337  0.075  0  0.276  0  0  0  0.157  0.268  0.143  0.179  0.171  0.345  0.169  0.446  1.211  0.493  0.161  0.211  0.112  0.076  0.164  0  0  0.156  1.857  0.103  0.386  0.419  0.218  0.153  0  0  0  
 
 NEU4  129807  2  242750159  242758739  2q37.3  0  0  0.151  0  0.162  0  0.097  0.086  0.238  0.142  0.06  0.217  0.411  0.072  0.918  0.1  0.099  0.113  0.294  0.124  0  0.341  0.348  0.066  0  0.096  0.301  0.193  0  0.167  0  0  0  0.275  0.17  0  0  0  0.146  0.184  0.072  0.369  0.384  0  0  0.102  0.077  0.099  0  0.055  0.089  0.794  0.05  0.242  0.12  0.108  0  0  0  0  
 
 PDCD1  5133  2  242792032  242801058  2q37.3  0  0  0.121  0  0.031  0  0  0.186  0.253  0.075  0.075  0.064  0.082  0.064  0.286  0.047  0.066  0.128  0.714  0.048  0.186  1.65  1.082  1.441  0  0.058  0.118  0.043  0  0.141  0  0  0  0.227  0.074  0  0.101  0  0.083  0  0  0.2  0.081  0  0.112  0.145  0.081  0  0  0.13  0.049  0.632  0.036  0.107  0.053  0  0.066  0  0.1  0  
 
 RTP5  285093  2  242811885  242815482  2q37.3  0  0  0.217  0  0.045  0  0.142  0.071  0.272  0.091  0.068  0.085  0  0.091  0  0  0.076  0.296  0.564  0.138  0  1.561  0.349  0  0  0  0.152  0.041  0  0.103  0  0  0  0.296  0  0  0  0  0.086  0  0.061  0.197  0.091  0  0  0.058  0.03  0  0  0  0.049  1.03  0.034  0.128  0.123  0.09  0  0  0  0  
 
 LINC01237  101927289  2  242823513  243020873  2q37.3  0  0  0  0  0  0  0  0  0  0  0  0  0  0.227  0  0.148  0  0  1.786  0  0  0.826  0  0.621  0  0.181  0.265  0  0  0.17  0  0  0  0  0  0  0  0  0  0  0  0  0  0  0  0  0  0  0  0.276  0  0.368  0  0.163  0  0  0  0  0  0  
 
 LOC102723927  102723927  2  242912833  242919427  2q37.3  0.318  0  0.113  0  0  0  0  0.077  0.138  0  0.136  0  0.108  0.403  0.553  0.103  0  0.074  2.103  0.123  0  1.333  0.233  0.155  0  0.269  0.079  0.04  0.086  0.193  0.097  0.528  0.304  0.249  0  0  0.099  0  0.078  0.131  0  0.192  0.083  0  0.104  0.106  0.093  0.207  0.073  0.125  0.106  0.314  0.066  0.08  0.22  0  0.077  0.06  0.215  0  
 
 LOC728323  728323  2  243030783  243102476  2q37.3  0.192  0.427  0.23  0.297  0.541  0.454  0.226  0.504  0.663  0.26  0.495  0.38  0.676  0.324  0.385  0.481  0.687  0.658  0.823  0.471  1.514  0.981  0.432  0.464  0  0.65  0.265  0.358  0.433  0.385  0.49  0.19  0.294  0.375  0.554  0.256  0.162  0.385  0.602  0.296  0.233  0.647  0  0.344  0.374  0.458  0  0.325  0.287  0.132  0.302  0.307  0.108  0.21  0.207  0.456  0.241  0.336  0.496  0.299  
 
 CHL1  10752  3  238278  451097  3p26.1  0  0.017  0.206  0  0.029  0.071  0.489  0.03  0.085  0.278  0.023  0.081  0  0.03  0  0.044  0  0.046  0.031  0.077  0  0.125  0.332  0  0  3.193  2.163  1.241  1.236  1.597  1.166  1.25  1.512  0.918  0.052  0.4  0  0  0.04  0  0  0.855  0.044  0.032  0  0.077  0.255  0  0  0  0.063  0.537  0  0.052  0.05  0.034  0  0  0  0  
 
 CHL1-AS1  101927193  3  405055  427478  -  0  0  0  0  0  0  0.124  0  0.126  0  0  0  0  0  0  0  0  0  0  0  0  0  0  0  0  0.57  0.425  0.159  0.148  0.052  0  0  0.266  0.201  0  0  0  0  0  0  0  0  0  0  0  0  0  0  0  0  0  0  0  0  0  0  0  0  0  0  
 
 LINC01266  101927215  3  633787  887698  -  0  0  0.117  0  0  0  0  0  0.06  0  0  0  0  0.016  0  0  0  0  0  0  0  0.015  0.121  0  0  0.039  0.039  0.03  0  0.032  0  0  0  0.154  0  0  0  0  0  0  0  0.115  0.028  0  0  0  0.016  0  0  0  0  0.062  0  0  0  0  0  0  0  0  
 
 CNTN6  27255  3  1134341  1445292  3p26-p25  0  0.025  0.061  0  0.018  0  0  0  0.038  1.63  0  0  0  0  0  0  0  0.04  0  0  0  0.033  0  0  0  0.039  0.038  0.249  0  0.055  0  0.089  0  0.121  0  0.036  0.031  0  0.019  0  0  0  0.026  0  0  0  0.019  0  0  0  0.03  0.128  0.094  0.287  0.105  0.118  0.16  0  0  0.119  
 
 CNTN4  152330  3  2140549  3099645  3p26.3  0  0  0.069  0.714  0  0  0  0  0  0  0  0.039  0  0.015  0  0.03  0  0.033  0  0.043  0  0.032  0.178  0.032  0  0  0.144  0.111  0  0.057  0  0  0  0.118  0  0.04  0  0  0.061  0  0  0.122  0.045  0  0  0  0.048  0  0  0  0  0.057  0  0.031  0  0  0  0  0  0  
 
 CNTN4-AS2  100873976  3  2152092  2185925  -  0  0  0  0  0  0  0  0  0  0  0  0  0  0  0  0  0  0  0  0  0  0  0  0  0  0  0.161  0  0  0  0  0  0  0  0  0  0  0  0  0  0  0  0  0  0  0  0  0  0  0  0  0  0  0  0  0  0  0  0  0  
 
 CNTN4-AS1  100873975  3  3081344  3102829  -  0  0.093  0  0.475  0  0  0  0  0  0  0  0  0  0  0  0  0  0  0  0.127  0  0  0.298  0  0  0  0.094  0  0  0.043  0  0  0  0  0.216  0  0  0  0.058  0  0  0  0  0  0  0  0  0  0  0  0  0  0  0  0  0  0  0  0  0  
 
 IL5RA  3568  3  3108007  3152058  3p26-p24  0  0.047  0.449  0.381  0.1  0.299  0.736  0.064  0.543  0.044  0.139  0.951  0  0.08  0  0.102  0.069  0.174  0  0.444  0.106  0.172  1.041  0.103  0.066  0.05  1.534  0.038  0  1.243  0.226  0.33  0.087  0.972  0.15  0  0  0  0.134  0  0  0.755  0.207  0  0.178  0.182  2.061  0.101  0  0  0  0.295  0.047  0.16  0.069  0.099  0.068  0.082  0  0  
 
 TRNT1  51095  3  3168599  3190706  3p25.1  2.105  2.596  0.985  1.86  1.646  1.138  1.517  0.991  1.256  1.729  1.4  1.269  1.799  2.256  1.625  1.508  1.478  2.736  3.211  2.158  1.466  1.857  1.277  0.925  1.706  1.492  1.152  1.148  1.217  1.17  1.644  1.492  1.332  1.019  1.111  1.56  2.036  1.066  1.637  1.381  1.116  1.353  1.143  1.306  0.985  1.221  0.221  1.64  1.276  2.485  1.646  0.619  1.213  0.657  1.704  1.236  1.107  1.482  1.643  2.047  
 
 CRBN  51185  3  3191316  3221401  3p26.2  2.142  2.606  0.977  1.752  2.617  1.167  1.867  1.502  2.006  1.993  1.576  1.014  1.396  1.961  1.432  1.247  1.671  1.808  3.758  2.495  1.567  2.262  1.641  0.879  1.366  1.472  1.309  1.415  1.092  1.249  1.564  1.682  1.552  1.095  0.938  1.487  1.586  1.61  2.25  1.104  1.521  1.053  1.969  1.166  1.102  1.415  1.229  1.6  1.332  2.953  2.008  0.608  1.434  0.602  1.507  1.091  1.361  1.615  1.611  2.248  
 
 LRRN1  57633  3  3841120  3889387  3p26.2  0.214  0.037  0.143  3.531  1.411  0  0.057  0.488  0.174  3.335  0.196  0  0  0.029  0.806  0.15  0  0.047  1.914  0.092  0  0.683  0.223  0.027  0  0.036  0.082  0.038  0  0.07  0  0.039  0  0.155  0.056  0  0  0  0.374  0  0.769  0.183  0.042  0.037  3.404  0.881  0.516  0  2.364  0.029  0.026  0.453  0.024  0.1  0.035  0  0  0  0  0.287  
 
 SETMAR  6419  3  4344987  4358949  3p26.1  2.284  1.584  1.123  2.201  2.149  0.564  1.83  1.588  1.734  2.102  1.991  0.888  1.717  1.992  1.421  1.113  1.188  3.212  3.797  2.592  1.616  2.662  1.016  0.496  1.219  2.117  1.751  1.412  1.608  1.53  2.181  1.634  1.469  1.28  0.888  0.892  1.537  0.924  1.87  1.253  1.162  1.76  2.617  1.417  1.212  1.206  0.699  1.144  1.512  2.186  1.671  1.138  1.471  0.615  1.831  0.798  1.236  1.178  1.173  2.178  
 
 SUMF1  285362  3  4402828  4508966  3p26.1  2.75  3.01  1.877  1.954  4.069  1.675  2.639  2.042  2.062  2.827  1.827  1.59  2.55  2.976  1.837  1.57  2.317  2.949  1.712  1.966  0.903  1.024  1.458  0.913  1.79  3.088  1.391  2.355  2.455  2.355  2.526  2.222  0.714  0.511  1.226  2.201  2.453  3.049  2.732  2.146  2.418  1.51  1.851  1.432  1.296  1.977  2.943  1.787  2.134  3.075  2.706  1.507  2.372  1.186  2.942  1.704  1.995  2.458  2.622  3.41  
 
 ITPR1-AS1  100996539  3  4532000  4534847  -  0.249  0.141  0.112  0  0.431  0.081  0  0.09  0.248  0.084  0  0  0  0.064  0.185  0  0  0  0.196  0.17  0.148  0.116  0.259  0  0  0.205  0.097  0.404  0.127  0.042  0.242  0.138  0  0.238  0  0.081  0.116  0  0.17  0  0  0.469  0.577  0  0  0  0.032  0  0  0.256  0.553  0.202  0  0.057  0.103  0  0  0  0  0.201  
 
 ITPR1  3708  3  4535031  4889524  3p26.1  0.389  0.125  0.24  0.522  0.797  0.221  0.653  0.706  0.361  0.427  1.204  0.22  0.289  0.415  0.286  0.067  0.118  0.159  0.714  0.884  0.81  0.544  0.186  0.241  0.538  1.491  1.32  1.449  0.976  0.375  0.77  0.787  0.709  0.324  0.26  0.691  0.581  0.541  0.186  0.765  0.141  0.661  0.697  0.387  0.207  0.127  0.093  0.365  0.147  0.563  1.805  0.432  0.422  0.738  0.431  0.221  1.295  0.285  0.389  1.105  
 
 EGOT  100126791  3  4790877  4793274  3p26.1  0  0  0.273  0  0.297  0  0  0.104  0.206  0.114  0.187  0  0  0.056  0  0  0  0  0  0.258  0  0.203  0.321  0.163  0  0.275  0.881  0.148  0.103  0.208  0.209  0  0.159  0.397  0.099  0.189  0.083  0  0.155  0  0.116  0.167  0.09  0.243  0.228  0.299  0.094  0.143  0  0  0.221  0.212  0.434  0.185  0.414  0.162  0.973  0  0.576  0.157  
 
 BHLHE40-AS1  100507582  3  4938492  5021646  3p26  0  0  0.242  0  0.172  0  0.092  0.173  0.214  0.22  0.795  0.147  0  0.046  0  0  0.076  0.082  0  0.058  0  0.042  0.294  0  0  0.134  0.234  0  0.098  0.192  0  0  0  0.151  0.123  0.131  0.175  0  0.145  0.155  0.139  0.541  0.3  0  0  0.188  0.052  0.111  0.133  0  0.127  0.108  0.309  0.118  0.115  0  0.179  0  0.102  0.11  
 
 BHLHE40  8553  3  5021096  5026865  3p26  2.354  2.574  3.747  2.727  4.126  2.797  3.791  5.38  2.953  3.979  4.712  3.458  2.614  3.035  0.999  0.481  3.533  3.207  0.324  1.285  3.027  1.057  1.146  1.243  3.274  5.258  4.071  2.894  3.717  3.605  3.196  3.58  2.722  1.809  3.659  4.261  4.145  2.176  4.349  4.143  3.134  3.399  3.503  3.191  3.308  3.859  0.878  2.684  4.244  3.393  5.668  2.142  4.49  4.274  3.753  3.239  5.248  3.785  4.518  3.983  
 
 ARL8B  55207  3  5163929  5222601  3p26.1  3.639  5.193  2.747  3.253  3.797  2.219  4.105  3.245  3.36  3.41  3.589  2.516  3.372  4.215  2.465  2.38  2.881  5.043  4.581  3.682  3.191  2.641  2.496  1.569  3.566  4.16  3.293  3.468  3.59  3.512  3.802  3.511  2.909  2.283  2.537  3.027  3.467  3.545  4.089  3.249  3.001  2.033  4.325  2.529  1.214  2.952  3.671  2.945  2.7  4.411  3.885  1.604  3.412  2.321  2.975  2.417  3.019  3.187  3.597  4.381  
 
 EDEM1  9695  3  5229358  5261650  3p26.1  1.585  1.719  1.852  2.183  1.877  1.53  2.912  2.537  2.871  2.411  2.763  1.181  2.097  1.392  1.904  1.741  2.27  1.044  4.154  2.173  1.329  2.41  3.522  2.006  2.383  3.471  2.547  3.245  2.758  1.975  2.624  2.98  2.398  1.454  1.138  2.14  3.264  2.331  2.695  2.216  2.623  1.277  1.225  1.452  0.69  1.622  1.129  1.317  2.019  1.813  2.54  1.193  1.728  1.229  1.934  1.461  2.059  1.919  1.433  2.996  
 
 GRM7-AS3  100507601  3  6674044  6847136  -  0  0  0  0  0.351  0  0  0  0  0  0  0  0  0  0  0  0  0  0  0  0  0  0  0  0  0  0.12  0  0  0.097  0  0  0  0  0  0  0  0  0  0  0  0  0  0  0  0  0  0  0  0  0  0  0  0  0  0  0  0  0  0  
 
 GRM7  2917  3  6902801  7783218  3p26.1-p25.1  0  0.024  0.145  0  0.028  0  0.022  0  0.071  0.042  0.129  0.067  0  0.044  0  0.021  0.055  0.068  0.022  0.054  0  0.183  0.31  0  0  0.02  0.095  0.158  0  0.085  0  0.034  0  0.12  0  0  0.061  0  0.043  0  0.046  0.102  0.049  0  0.057  0.033  0.072  0.04  0.041  0  0  0.51  0  0.07  0.056  0.077  0  0  0  0  
 
 GRM7-AS2  100873936  3  6934323  6935704  -  0  0  0  0  0  0  0  0  0  0  0  0  0  0  0  0  0  0  0  0  0  0.147  0  0  0  0  0  0  0  0  0  0  0  0  0  0  0  0  0  0  0  0  0  0  0  0  0  0  0  0  0  0  0  0  0  0  0  0  0  0  
 
 GRM7-AS1  100873937  3  7561427  7576971  -  0  0  0  0  0  0  0  0  0  0  0  0  0  0.172  0  0  0  0  0  0  0  0  0  0  0  0  0  0  0  0  0  0  0  0  0  0  0.27  0  0  0  0  0  0  0  0  0  0  0  0  0  0  0  0  0  0  0  0  0  0  0  
 
 LOC101927394  101927394  3  7994491  8057994  -  0  0  0.086  0  0  0  0  0.024  0.094  0.035  0.028  0  0  0  0  0  0  0  0  0  0  0.024  0.14  0  0  0  0.04  0  0  0.021  0  0  0  0.089  0  0  0  0  0  0  0  0  0  0  0  0  0  0  0  0  0  0.15  0  0  0  0  0  0.075  0  0  
 
 LMCD1-AS1  100288428  3  8262833  8543344  3p26.1  0  0.042  0.16  0  0.183  0  0.107  0.149  0.229  0.136  0.101  0  0  0.074  0  0.065  0  0.094  0  0  0  0.054  0.226  0  0.079  0.065  0.214  0.318  0.076  0.192  0.159  0.209  0.137  0.223  0.24  0.153  0.334  0  0.043  0.075  0.34  0.089  0.034  0  0.373  0.163  0.096  0  0  0.164  0.082  0.187  0.255  0.049  0.222  0.122  0.052  0.11  0.132  0.231  
 
 LMCD1  29995  3  8543492  8609811  3p26-p24  0.838  0.926  3.66  0.615  4.631  1.476  1.643  4.14  4.968  1.803  2.652  1.6  0  0.82  0.811  1.043  0.48  2.917  0.149  0.087  0  0.226  0.596  0.103  0.787  1.024  2.812  3.113  2.351  1.285  2.574  2.798  2.002  1.576  2.527  1.967  1.617  0.842  0.622  0.911  1.414  1.286  1.526  0.701  4.121  2.837  3.13  2.002  0.583  4.576  0.79  1.202  3.142  1.804  3.229  1.801  2.767  1.438  3.63  4.33  
 
 LINC00312  29931  3  8613467  8616354  3p25.3  0.099  0.066  0.369  0  1.197  0.085  0.096  0.969  0.968  0.214  0.523  0.098  0  0.128  0.058  0.11  0  1.433  0  0  0  0.038  0.206  0  0  0.029  0.234  0.31  0.074  0.065  0.188  0.099  0  0.139  0.309  0.163  0.098  0.051  0.057  0  0.051  0.268  0.227  0  0.624  0.447  0.258  0.186  0  0.718  0  0.124  0.498  0.131  0.432  0  0.365  0  0.996  0.629  
 
 SSUH2  51066  3  8661085  8693764  3p26.1  0  0  0.309  0.503  0.053  0  0.163  0.125  0.123  0.52  0.299  0.598  0.347  0.533  0  0.1  0.294  0.145  0.389  0.116  0  0.139  0.605  0.134  0.184  0  0.102  0.928  0.099  0.065  0.148  1.232  0  0.166  0.076  0.216  0  0.154  0.564  0  0  0.213  0.042  0  0.303  0.144  0.123  0  0.163  0.082  0.171  0.343  0.062  0.108  0  0  0.096  0.301  0  0  
 
 CAV3  859  3  8775485  8788451  3p25  0.178  0.072  0.293  0.264  0.135  0  0.138  0  0.188  0.069  0  0.128  0  0.051  0  0.144  0  0  0.067  0  0  0.168  0.366  0.148  0  0  0.068  0  0.087  0.198  0  0  0  0.29  0.096  0  0  0.137  0.225  0  0  0.615  0.138  0  0  0.06  0.311  0  0  0.115  0.08  0.584  0.05  0  0.1  0  0.142  0  0  0  
 
 OXTR  5021  3  8792094  8811300  3p25  0.434  2.878  4.809  3.828  0.185  3.32  0.502  0.21  0.248  1.385  0.314  0.228  0.256  0.473  0.313  0.124  0.237  0.496  0.866  0.27  0.261  0.511  1.535  1.699  0.181  0.18  0.306  0.202  0.068  0.121  0.194  0.246  0.236  0.31  0.637  0.638  0.515  0.441  4.299  0.759  0.668  0.562  0.168  0.193  1.25  0.632  2.091  4.735  0.918  5.128  2.033  1.036  1.357  0.268  1.601  0.965  0.376  3.71  1.154  3.231  
 
 RAD18  56852  3  8918879  9005159  3p25-p24  1.861  3.174  1.241  1.803  1.798  1.728  2.047  1.284  0.535  1.622  1.508  0.74  1.493  2.131  1.578  1.383  1.982  1.816  2.99  1.827  1.913  2.428  0.989  0.995  1.294  1.898  1.437  1.663  1.309  1.303  1.605  1.597  1.629  0.888  1.519  1.267  2.071  1.516  2.174  1.327  1.058  1.106  2.111  1.133  1.119  1.417  1.42  1.758  1.87  2.692  2.462  1.016  1.815  0.734  1.636  1.043  1.378  1.789  1.658  2.2  
 
 SRGAP3  9901  3  9022275  9291369  3p25.3  0.636  0.016  0.165  0.235  1.253  0.023  0.231  0.065  0.368  0.556  0.673  0.041  0.058  0.477  0.129  0.017  0.169  0.11  0.068  0.067  0.04  0.294  0.28  0.017  0  0.38  0.109  0.049  0.099  0.301  0.073  0.156  0.104  0.294  0.1  0.343  0.066  0.713  0.078  0.186  0.376  0.394  0.248  0.768  1.059  0.941  0.13  0.079  0.125  0.103  0.1  0.507  0.038  0.096  0.251  0.119  0.053  0  0  0.226  
 
 SRGAP3-AS3  100288831  3  9258578  9261192  3p25.3  0.153  0  0  0  0.177  0  0  0  0.12  0.193  0.078  0  0  0.05  0  0  0  0  0  0  0  0.132  0.238  0  0  0.116  0.043  0  0  0.034  0  0  0  0  0  0  0  0.15  0  0  0  0.186  0  0.2  0  0.38  0  0  0  0  0  0.177  0  0  0  0  0  0  0  0  
 
 THUMPD3  25917  3  9404716  9428475  3p25.3  2.394  3.302  1.616  2.589  2.529  1.426  2.616  1.525  1.955  2.304  2.402  1.294  2.24  3.391  1.413  1.593  2.617  3.391  3.809  2.042  2.702  2.713  2.132  1.399  2.021  2.319  2.066  1.925  1.891  1.96  2.398  2.227  1.686  1.265  1.92  2.113  2.145  1.933  2.587  2.268  1.997  1.377  2.359  2.2  1.463  1.841  1.386  1.376  2.109  2.86  2.288  1.087  1.904  1.012  2.112  1.403  1.436  1.824  1.825  2.442  
 
 THUMPD3-AS1  440944  3  9430536  9439174  3p25.3  2.109  2.795  1.016  1.109  2.311  1.348  1.239  1.684  1.085  2.252  2.15  0.771  1.714  1.812  1.483  1.851  1.316  4.24  3.332  2.22  1.607  2.18  1.786  1.115  1.054  1.369  1.451  1.19  1.3  0.957  1.963  1.9  1.401  0.741  1.497  1.371  2.222  1.656  2.649  0.875  1.634  1.764  1.362  0.822  1.218  1.794  1.967  1.524  1.641  2.821  1.632  0.859  1.968  0.356  2.096  1.374  1.476  1.671  2.23  1.651  
 
 SETD5  55209  3  9439383  9519838  3p25.3  3.956  3.111  2.53  3.236  3.268  2.167  3.088  3.316  2.702  3.595  3.439  1.942  3.325  3.734  3.838  2.83  3.084  2.247  3.266  2.732  3.408  3.086  1.608  1.864  3.063  2.953  2.701  2.982  2.573  2.44  3.005  2.922  2.51  0.919  2.488  2.931  3.767  3.115  3.245  3.058  3.165  1.964  3.297  3.246  2.643  2.78  2  3.231  3.427  3.008  3.365  1.745  3.327  1.69  3.267  2.644  3.298  3.127  3.332  3.656  
 
 LHFPL4  375323  3  9540044  9595486  3p25.3  0.085  0.146  0.186  0.055  0.09  0.247  0.12  1.602  0.789  1.768  0.177  0.046  0.063  0.098  0.282  0.046  0.046  0.133  0.073  0.085  0  0.217  0.46  0.066  0  0.071  0.142  0.043  0.108  0.737  0.102  0.052  0.11  0.228  0.078  0.16  0.053  0.213  0.12  1.138  0.068  0.312  1.291  0.166  0.04  0.14  0.052  0.764  0.169  0.863  0.049  1.123  0.105  0.069  0.083  0.071  0.074  1.413  0.105  0  
 
 MTMR14  64419  3  9691116  9744078  3p26  2.915  2.038  1.651  2.634  2.996  1.305  2.838  2.287  2.465  2.519  2.354  1.578  2.448  2.695  2.294  2.176  2.589  1.273  3.85  3.318  2.179  2.825  2.638  1.835  2.294  2.686  2.517  3.208  2.652  2.334  2.363  2.751  3.097  2.015  1.832  2.259  3.041  2.789  3.314  2.571  2.134  1.909  3  1.638  1.856  1.95  1.869  2.371  1.542  3.771  2.166  1.296  2.001  1.478  2.414  1.448  1.896  2.467  3.006  2.756  
 
 CPNE9  151835  3  9745490  9771592  3p25.3  0  0  0.093  0.17  0.247  0.098  0.093  0.064  0.108  0.088  0.066  0.165  0.171  0.535  0  0  0.101  0  0.087  0.328  0  0.192  0.388  0  0  0.151  0.2  0.064  0  0.131  0.117  0.097  0  0.15  0  0  0.107  0.098  0.133  0  0  0.322  0.392  0  0  0.135  0.103  0  0  0.251  0.059  0.545  0  0.19  0.14  0.095  0.104  0.109  0.07  0  
 
 BRPF1  7862  3  9773433  9789699  3p26-p25  3.209  1.125  1.554  2.339  1.977  1.665  3.015  2.611  1.598  2.518  2.287  1.291  2.275  2.621  2.84  1.715  1.815  0.514  3.447  2.672  2.594  2.642  1.201  1.671  1.981  2.103  2.834  2.759  2.2  2.204  2.069  2.422  2.334  0.914  1.549  1.892  3.258  2.369  1.756  2.995  1.981  1.243  2.76  1.991  1.537  1.939  2.014  2.602  2.371  2.595  2.39  1.446  2.909  1.292  2.113  1.432  2.047  2.35  2.675  2.516  
 
 OGG1  4968  3  9791627  9808353  3p26.2  3.131  1.723  2.433  2.956  3.033  2.464  3.191  2.689  2.597  3.149  2.826  1.86  2.751  2.422  3.025  2.16  2.06  0.813  2.595  2.639  2.35  1.972  2.056  2.339  2.696  2.706  3.174  2.85  2.638  2.684  2.728  2.956  3.558  2.136  2.389  2.326  3.403  3.507  1.696  2.846  2.022  2.435  3.571  2.042  1.808  2.352  2.067  2.906  1.913  4.094  2.842  2.262  3.157  1.723  2.573  2.123  2.398  3.479  3.074  2.412  
 
 CAMK1  8536  3  9799028  9811668  3p25.3  2.188  0.523  2.248  2.496  1.008  2.163  1.382  1.238  2.102  1.942  1.986  0.273  1.7  2.427  2.338  0.507  0.145  0  0.162  3.498  0.45  0.456  0.296  0.159  0.153  1.901  0.6  0.982  0.46  0.632  1.493  1.575  0.785  0.529  1.476  1.774  1.746  2.673  3.034  0.456  0.349  2.028  3.756  1.743  0.769  0.914  2.234  2.567  1.599  3.689  1.999  1.806  2.525  0.167  3.154  1.906  1.856  3.458  2.759  2.757  
 
 TADA3  10474  3  9821647  9834695  3p25.3  5.264  2.173  4.461  4.607  5.134  3.119  5.288  4.549  4.651  4.783  4.728  3.091  4.163  5.107  3.868  3.884  4.772  0.45  5.286  4.225  4.318  3.878  3.68  3.024  3.786  4.109  4.247  4.178  4.025  3.656  4.055  4.341  4.536  3.44  3.395  4.384  4.852  4.942  3.978  5.138  3.315  4.006  4.96  3.458  3.33  3.911  3.56  3.889  3.862  5.212  3.822  3.336  4.191  3.166  4.45  3.101  3.849  4.08  4.13  4.526  
 
 ARPC4  10093  3  9834178  9848789  3p25.3  3.871  1.989  4.934  5.591  4.179  4.632  5.258  4.818  5.151  5.224  5.003  4.508  4.771  4.245  3.831  4.623  5.067  0.605  5.359  3.654  4.307  3.925  4.155  3.702  5.182  4.848  4.121  4.542  4.175  3.927  4.94  4.893  4.499  3.485  4.118  4.386  5.143  4.534  3.864  4.659  4.443  4.68  3.184  4.041  4.248  4.931  1.698  4.21  4.304  5.302  4.782  3.342  4.118  3.732  4.467  3.709  4.034  4.926  4.951  5.747  
 
 ARPC4-TTLL3  100526693  3  9834231  9878040  3p  0.302  0  0.228  0.839  0.45  0  0.494  0.5  0.639  0.776  0.654  0.298  0.623  0.308  0.997  0  0.321  0  2.201  0.061  1.016  0.439  0.197  0  0.224  0.574  1.066  0.612  0.424  0.169  0.434  0.474  0.508  0.319  0.149  0.142  1.085  0.532  0.203  0.26  0.14  0.484  1.094  0.228  0.234  0.542  0.024  0.174  0.645  1.276  0.484  0.179  1.227  0.327  0.382  0.155  0.372  0.712  0.175  0.372  
 
 TTLL3  26140  3  9851643  9878040  3p25.3  1.309  0.2  0.508  1.289  1.466  0.529  1.009  1.115  0.639  1.753  0.977  0.355  0.968  1.548  1.316  0.418  0.871  0.072  2.052  0.999  1.488  1.843  0.741  0.376  0.127  1.264  2.596  0.826  0.663  1.091  0.838  0.666  0.976  0.291  0.771  0.597  1.324  1.204  1.339  0.731  0.611  0.831  2.987  0.325  0.182  0.756  0.336  0.851  1.313  1.009  0.731  0.7  2.36  0.517  1.391  0.555  0.822  1.97  0.792  0.655  
 
 RPUSD3  285367  3  9879532  9885702  3p25.3  4.08  2.255  2.722  3.796  4.714  2.676  4.503  2.505  2.604  3.221  3.563  3.279  3.805  4.125  2.64  2.915  4.024  1.895  5.018  4.427  4.186  3.95  3.244  3.609  3.728  3.994  4.926  4.762  4.213  4.991  4.953  4.537  4.558  2.592  3.616  2.805  4.7  3.886  3.732  3.265  4.08  3.274  4.69  3.252  2.332  3.066  3.274  3.691  3.095  5.505  3.609  2.394  3.358  1.987  3.852  3.177  2.245  4.68  4.126  4.02  
 
 CIDEC  63924  3  9908393  9921938  3p25.3  0.354  0  0.78  0.866  0.588  0  1.063  0.158  0.797  0.431  0.229  0.77  0.576  0.12  0.255  0.388  0.453  0.228  0.534  0.285  0  0.427  0.703  0.169  0  0.215  0.688  0.235  0.408  0.279  0.195  0.337  0.208  0.361  1.193  0.13  0.469  0.21  1.106  0  0.189  1.16  0.756  0  0.356  0.312  1.091  0.495  0  0.458  0.974  0.41  0.169  0.182  0.351  0.197  0  0.209  0  0  
 
 JAGN1  84522  3  9932270  9936031  3p25.2  4.47  3.951  2.864  4.466  4.191  2.602  4.678  3.189  3.949  3.79  3.396  2.743  3.349  4.474  3.229  3.461  4.054  3.917  5.068  4.125  3.476  3.476  4.108  2.923  3.264  3.63  3.865  3.681  3.56  3.512  3.297  3.98  3.679  3.131  3.041  3.003  4.153  2.895  4.287  3.39  3.148  3.629  4.216  2.615  2.751  2.577  3.224  2.992  2.621  4.777  3.524  2.336  3.185  1.949  3.601  2.996  2.682  3.752  3.47  3.777  
 
 IL17RE  132014  3  9944295  9958084  3p25.3  0.991  0.171  0.103  0.757  2.063  0.309  0.262  0.169  0.146  0.296  0.163  1.45  1.889  0.88  1.491  1.295  1.151  0.112  1.567  0.373  0  0.582  0.398  0.157  0.113  0.366  0.695  0.225  0.11  0.125  0.119  0.091  0.101  0.211  0.158  0.203  0.417  0.553  0.174  0  1.105  1.401  0.078  0.232  0.24  0.805  0.051  0.464  0.141  0.869  0.456  1.177  0.068  0.317  0.941  0.145  0.117  0.223  0.07  0.323  
 
 IL17RC  84818  3  9958757  9975305  3p25.3|3p25.3-p24.1  1.632  0.136  1.299  2.675  2.454  0.704  1.601  1.9  3.036  2.27  1.862  0.603  1.428  1.031  1.465  1.177  1.28  0.104  0.367  0.407  0.678  0.368  0.559  0.136  1.308  1.889  1.811  2.402  2.116  1.992  1.535  1.791  2.714  1.505  1.073  1.338  1.809  1.499  0.985  1.564  1.442  2.582  0.436  0.735  1.31  1.807  0.402  2.046  0.59  3.238  3.141  1.472  1.12  1.109  1.314  1.243  1.5  2.786  1.761  2.248  
 
 CRELD1  78987  3  9975523  9987097  3p25.3  1.074  0.718  1.65  2.341  2.817  1.287  2.078  2.678  2.926  3.138  1.399  1.502  1.513  1.426  2.395  1.244  1.926  0.589  1.887  1.153  1.366  1.427  1.697  1.191  0.76  2.486  2.564  1.822  2.311  1.925  1.737  1.761  2.61  1.561  1.083  1.315  2.536  2.02  2.213  1.501  1.744  1.703  1.496  1.14  1.146  2.146  0.958  1.767  0.92  2.992  1.878  0.81  1.154  0.851  1.879  1.984  1.331  2.044  1.736  2.522  
 
 PRRT3  285368  3  9987225  9994078  3p25.3  2.081  0.187  0.704  1.613  2.032  0.394  1.155  0.604  0.954  1.309  0.865  0.141  0.405  0.666  0.642  0.317  0.327  0.206  0.841  0.25  0.414  1.218  0.845  0.464  0.268  0.482  0.888  0.543  0.866  0.469  0.445  0.163  0.72  0.479  0.539  0.848  0.781  0.805  1.007  0.886  0.836  1.374  1.497  0.565  2.221  0.741  0.565  0.819  0.461  1.45  0.631  1.226  0.321  0.263  0.426  0.465  0.361  1.028  0.294  0.785  
 
 PRRT3-AS1  100874032  3  9989087  9996471  -  0  1.453  2.701  1.091  0.314  1.555  0  0.75  0.437  0.973  0.27  0.986  1.581  2.334  1.7  1.497  1.659  1.653  0.296  0.621  0.563  0.726  0  0  0.94  1.677  1.74  0.523  1.32  1.061  1.971  0.252  2.488  0.992  2.201  1.823  1.266  1.36  0.331  1.285  1.996  1.197  0.125  0.78  0  1.868  2.571  1.287  0  2.856  2.481  1.559  0.457  0.867  0  0.314  0.83  1.036  1.986  0.468  
 
 EMC3  55831  3  10005635  10028522  3p25.3  3.856  4.333  2.683  4.628  4.551  2.587  5.378  3.564  3.731  4.322  3.807  3.134  3.145  2.658  2.508  2.682  2.813  4.404  4.626  4.291  3.906  2.997  3.51  2.975  3.619  3.33  3.249  3.404  3.495  3.67  3.806  4.086  2.96  1.986  2.986  3.597  3.738  3.287  5.148  3.159  3.375  3.338  3.361  2.738  3.034  3.7  3.308  2.953  3.134  4.806  3.675  2.07  3.332  2.63  3.249  2.737  2.798  3.483  3.167  4.143  
 
 LOC442075  442075  3  10028576  10048687  3p25.3  2.406  1.092  0.744  1.754  1.949  1.109  1.917  1.39  0.542  1.831  1.647  0.577  1.878  1.602  2.129  1.312  0.832  0.501  2.03  1.772  1.986  2.007  1.165  0.894  0.715  1.45  1.906  2.185  0.873  1.474  1.178  1.177  2.112  0.849  1.255  0.976  2.604  2.043  1.575  1.589  1.85  1.36  2.721  0.977  1.412  1.784  2.598  1.241  1.548  1.661  1.5  1.343  2.161  0.78  1.482  1.069  1.339  1.288  1.738  1.043  
 
 LOC401052  401052  3  10048101  10052779  3p25.3  0.713  0.075  0.453  0.879  1.237  0.146  0.202  0.233  0.362  0.453  0.382  0.207  0.299  0.341  0.316  0.318  0.317  0  0.385  0.315  0.218  0.562  0.579  0.112  0.32  0.387  0.177  0.259  0.513  0.36  0.403  0.591  0.249  0.577  0.133  0.861  0.48  0.756  0.8  0.51  0.25  0.648  1.319  0.305  0  0.284  0.03  0.166  0.127  0.291  0.416  0.467  0.312  0.394  0.37  0.232  0.286  0.194  0.176  0.321  
 
 CIDECP  152302  3  10059236  10067820  3p25.3  2.268  1.242  2.746  3.272  2.917  1.741  4.402  2.154  2.653  2.26  2.279  1.04  1.854  1.679  1.519  1.179  1.869  0  3.088  1.769  2.187  1.963  2.324  0.961  1.64  2.092  3.234  1.854  1.86  2.343  2.206  2.76  2.755  1.066  1.601  1.903  2.873  2.471  3.261  1.808  2.377  1.8  2.845  1.042  1.525  2.276  2.673  2.429  1.516  3.723  2.902  1.29  2.053  1.105  2.162  1.199  1.397  3.17  1.663  1.979  
 
 FANCD2  2177  3  10068112  10143614  3p26  2.577  2.04  1.141  1.312  2.314  1.215  2.107  1.238  0.393  1.917  1.982  1.158  1.449  2.98  2.351  1.551  2.122  1.87  3.697  2.468  2.285  2.451  1.227  1.53  1.579  2.276  1.827  1.825  1.631  1.505  1.93  1.708  2.209  0.777  1.642  1.587  2.95  1.795  1.276  2.035  2.287  1.263  1.526  1.629  1.252  2.038  1.882  1.326  2.531  1.876  1.757  1.403  2.041  0.829  1.982  1.225  1.589  2.449  1.956  2.597  
 
 FANCD2OS  115795  3  10123003  10149915  3p25.3  0.264  0  0.389  0  0.122  0.19  0.106  0  0.208  0  0  0  0  0.066  0  0  0.16  0  0.13  0.136  0  0.245  0.445  0  0.206  0  0.163  0.132  0.149  0.094  0  0  0  0.211  0  0  0.161  0  0.096  0  0  0.125  0.074  0  0  0.235  0.059  0  0  0  0  0.217  0.148  0.141  0.111  0  0.136  0  0  0  
 
 BRK1  55845  3  10157332  10168874  3p25.3  5.653  6.125  5.145  5.965  6.337  4.98  5.594  4.356  5.633  5.683  5.096  3.889  4.362  5.347  3.814  4.581  5.429  4.575  6.535  5.994  4.655  5.368  5.246  3.821  4.676  4.55  4.489  3.803  4.545  3.964  4.802  4.805  4.221  4.04  4.377  4.391  4.654  5.007  6.278  4.163  4.094  4.749  5.709  4.059  3.923  5.171  4.527  4.494  4.195  6.72  4.916  3.376  4.277  3.371  4.324  3.777  4.429  5.041  4.55  5.215  
 
 VHL  7428  3  10183318  10195354  3p25.3  2.561  1.766  1.568  3.161  2.332  1.655  1.966  2.194  2.213  2.152  2.464  1.418  2.448  2.084  2.612  2.033  2.081  2.106  3.233  2.183  2.782  2.34  2.013  1.562  1.668  1.774  1.453  1.594  1.154  1.137  1.991  1.88  1.661  1.216  1.807  2.121  2.301  1.972  2.463  1.734  1.937  1.8  2.315  1.97  1.47  1.61  1.262  1.633  2.28  2.813  2.335  1.441  1.777  0.743  1.67  1.48  0.152  2.338  2.148  2.604  
 
 IRAK2  3656  3  10206562  10285427  3p25.3  0.066  1.481  0.706  2.239  0.252  0.405  1.969  1.175  0.672  1.107  0.565  0.661  0.416  0.722  0.345  2.069  0.307  1.313  0.027  0.067  0.649  0.084  0.533  0.041  1.152  0.235  0.217  0.245  0.133  0.07  0  0.511  0.051  0.409  0.83  1.357  1.245  1.644  2.187  0.521  0.965  0.34  0.956  0.285  0.423  0.099  0.519  0.186  0.376  0  1.676  0.93  1.117  0.626  1.17  1.336  0.791  0.809  1.697  2.153  
 
 TATDN2  9797  3  10290176  10322906  3p25.3  3.026  1.782  1.98  2.977  2.273  1.884  3.042  2.64  2.31  2.651  2.583  1.327  2.715  2.219  2.668  2.025  1.74  0.808  3.824  2.473  2.312  3.168  2.125  1.886  2.423  2.205  2.457  2.366  2.137  2.228  2.089  2.42  2.593  1.327  1.877  2.509  3.259  2.737  2.667  2.707  1.897  1.737  2.408  2.126  1.714  2.266  2.201  1.833  2.486  2.126  2.726  1.505  2.74  1.395  1.79  1.649  1.927  2.429  2.403  2.905  
 
 GHRLOS  100126793  3  10322635  10335133  3p25.3  0.43  0.34  0.674  1.125  0.52  0.417  0.779  0.77  0.333  0.939  0.506  0  0.798  0.84  1.233  0.561  0.171  0.158  1.271  1.1  0.584  0.954  0.608  0.408  0.292  0.331  1.238  0.619  0.761  0.244  0.366  0.449  0.858  0.363  0.378  0.52  0.951  1.67  0.604  0.448  0.404  0.473  0.478  0.608  0.4  0.562  0.465  0.563  0.981  0.174  0.709  0.397  0.963  0.267  0.255  0.406  0.629  0.918  1.687  0.417  
 
 LINC00852  84657  3  10326102  10327430  3p25.3  0.901  0.841  0.704  1.216  0.944  0.989  1.218  0.931  0.479  0.917  0.837  0.248  0.929  0.963  1.875  0.732  0.272  0.518  2.051  0.796  0.36  1.786  0.841  0.632  0.356  0.553  1.791  0.988  1.02  1.085  0.5  0.369  1.052  0.412  0.415  0.59  1.235  1.495  0.709  0.803  0.457  0.281  0.488  0.951  0.784  0.914  0.774  0.681  0.565  0.784  0.973  0.695  1.154  0.231  0.394  0.35  0.899  0.899  1.382  0.628  
 
 GHRL  51738  3  10327433  10334631  3p26-p25  0  0  0  0  0.476  1.226  0  0.246  0  0  0  0  0  0.414  0  0  0  0  0  1.23  0  0.205  0  0  0  0  0.424  0  0  0  0  0  0  0  0  0  0  0  0  0  0  0.769  0  0  0  0  0  0  0  0  0  0  0  0  0  0  0  0  0  0  
 
 SEC13  6396  3  10342612  10362872  3p25-p24  5.538  5.126  5.135  6.015  5.202  4.489  7.007  5.832  4.734  5.999  5.588  4.15  5.051  5.808  4.583  4.016  4.898  4.133  6.642  5.496  5.14  4.734  4.656  4.01  4.345  5.217  5.299  4.974  4.97  4.557  5.285  5.54  5.003  3.453  4.502  4.704  6.132  4.907  5.455  4.981  4.605  4.393  5.413  4.541  4.158  5.044  4.775  4.954  4.477  6.744  5.941  3.396  5.28  4.253  5.034  5.025  4.891  5.551  5.047  5.636  
 
 ATP2B2  491  3  10365706  10547268  3p25.3  0.251  0.073  0.275  0.648  0.161  0.095  0.299  0.19  0.268  0.261  0.139  0.144  0.116  0.212  0.087  0.105  0.149  0.152  0.171  0.225  0.023  0.397  0.529  0.138  0.093  0.111  0.221  0.114  0.161  0.276  0.076  0.091  0.045  0.256  0.176  0.139  0.149  0.059  0.308  0.096  0.054  0.399  0.207  0.05  1.164  1.193  0.304  0.104  0.056  0.14  0.158  0.646  0.194  0.17  0.24  0.127  0.104  0.114  0.094  0.057  
 
 ATP2B2-IT2  100874325  3  10667700  10668492  -  0  0  0  0  0  0  0  0  0  0  0  0  0  0  0  0  0  0  0  0  0  0  0  0  0  0  0  0  0  0.098  0  0  0  0  0  0  0  0  0  0  0  0  0  0  0  0  0  0  0  0  0  0  0  0  0  0  0  0  0  0  
 
 LINC00606  285370  3  10801168  10805877  3p25.3  0  0  0.119  0  0  0  0  0.028  0.049  0  0  0.048  0  0  0  0  0  0.07  0  0  0  0.036  0.361  0  0  0  0.031  0  0  0.053  0  0  0  0.189  0  0  0  0  0.037  0  0  0.166  0.024  0  0  0  0.018  0  0  0  0.038  0.257  0  0.052  0.027  0.07  0  0  0  0  
 
 SLC6A11  6538  3  10857916  10980146  3p25.3  0  0.065  0  0  0.229  0  0  0.043  0.071  0.062  0  0.12  0  0.066  0  0.056  0.065  0.161  0  0.139  0  0.267  0.222  0  0  0  0.166  0.042  0  0.209  0  0  0  0.121  0.1  0  0  0  0.081  0.806  0  0.493  0.68  0  0.487  0.053  0.033  0  0  0  0  0.847  0.03  0.136  0.117  0  0  0  0  0  
 
 SLC6A1  6529  3  11034419  11080935  3p25.3  0  0  0.103  0  0.028  0  0.024  0.032  0.073  0.045  0.03  0  0  0.036  0  0.03  0  0.103  0  0.054  0  0.09  0.195  0.028  0  0.018  0.088  0.022  0  0.069  0  0  0  0.157  0  0.028  0  0  0.065  0  0  0.397  0.04  0  0  0.022  0.052  0  0  0.018  0.032  0.461  0.021  0.106  0.051  0  0  0  0  0  
 
 SLC6A1-AS1  100874090  3  11047783  11060910  -  0  0  0  0  0  0  0  0  0  0  0  0  0  0  0  0  0  0  0  0  0  0  0  0  0  0  0  0  0  0  0  0  0  0  0  0  0  0  0  0  0  0  0  0  0  0  0  0  0  0  0  0  0  0.237  0  0  0  0  0  0  
 
 HRH1  3269  3  11178778  11304939  3p25  0  2.513  1.334  2.725  0.033  0.422  2.312  2.657  1.068  2.033  1.279  0.815  1.234  1.117  0.513  0.608  1.237  0.449  0  0.295  0  0.116  0.371  0.152  1.729  0.081  0.173  0.95  0  0.052  0  0.149  0.108  0.221  0.392  1.227  2.596  1.924  2.214  1.273  0.807  0.711  0.029  0.287  0.616  0.808  1.22  1.279  0.925  1.691  1.637  1.164  1.458  0.6  1.273  1.648  1.563  1.609  1.199  1.871  
 
 ATG7  10533  3  11314009  11599139  3p25.3  1.59  0.643  1.264  2.92  1.531  1.135  2.593  1.657  1.242  1.947  1.637  1.048  1.458  1.504  1.445  1.18  1.595  0.399  2.534  1.767  1.074  1.154  0.939  0.582  1.896  1.48  1.397  1.5  1.494  0.945  1.497  2.136  1.235  0.663  0.992  1.59  2.654  2.307  2.091  2.4  1.552  1.216  1.916  1.088  0.779  1.302  0.981  1.645  1.713  3.094  2.337  0.841  1.529  0.741  1.347  1.12  0.916  2.483  1.537  2.6  
 
 VGLL4  9686  3  11597540  11762242  3p25.3  2.522  1.498  1.912  2.107  2.22  2.058  2.517  2.945  4.206  2.918  3.072  2.518  2.827  1.729  2.139  1.784  2.64  1.988  3.213  1.391  0.995  2.305  2.638  1.957  2.68  3.043  2.827  2.485  2.216  3.509  2.641  3.086  2.366  1.633  1.251  2.04  2.107  2.266  3.194  1.512  2.167  2.147  2.732  2.78  2.334  2.243  1.998  1.532  1.716  2.04  2.32  1.522  2.028  1.458  2.257  2.219  1.434  1.9  1.759  2.998  
 
 TAMM41  132001  3  11831915  11888393  3p25.2  2.532  3.24  1.127  2.001  2.305  1.841  1.987  1.598  1.477  1.359  1.869  1.481  1.685  2.169  1.694  1.364  2.275  3.58  3.636  2.998  2.648  2.402  1.352  1.671  2.135  1.733  1.338  1.755  1.434  1.312  2.262  2.307  1.489  1.069  1.823  1.735  2.195  1.937  2.402  1.382  1.483  1.812  2.795  1.806  1.668  2.06  1.331  1.717  1.583  3.104  2.445  1.278  1.855  1.236  2.03  0.947  1.181  1.314  1.714  2.269  
 
 SYN2  6854  3  12045833  12233532  3p25  0  0.051  0.194  0.272  0.045  0.097  0.049  0.044  0.168  0.062  0.053  0.038  0  1.061  0  0.057  0.121  0.096  0.039  0.08  0  0.128  0.445  0.049  0  0.044  0.152  0.061  0  0.127  0.041  0.06  0  0.154  0.055  0  0.152  0  0.084  0.259  0  0.279  0.314  0  0.067  0.096  0.036  0.177  1.335  0.057  0.067  0.588  0.045  0.12  0.212  0.133  0.03  0  0  0.091  
 
 TIMP4  7079  3  12194567  12200851  3p25  0  0.222  0.129  0  0  0  2.022  0.16  0.735  0.51  1.829  1.217  0.98  0.082  0  0.502  0.929  0.083  0  0  0  0.089  0.383  0.556  0  0.176  0.055  1.199  0  0.03  0  0  0  0.133  1.07  0.095  2.155  1.538  0.317  0  0  1.559  0.054  0  0.194  0  0.148  0.204  0  0.249  0.891  0.359  0.184  0.905  0.461  0  0.547  0  0  0.267  
 
 PPARG  5468  3  12329348  12475855  3p25  0.588  3.713  0.905  3.437  0  0.123  0.125  0.717  0  0.057  0  3.033  2.521  2.491  0.242  4.023  5.165  3.886  0  0.705  0  0.193  0.306  0  0  0.474  0.117  0  0.262  0.829  0.147  0.096  0  0.168  2.113  0.39  3.638  2.664  1.478  0.481  1.842  1.262  0.043  0.225  0.183  0.076  4.114  0.471  1.9  1.433  4.592  1.242  2.132  1.395  3.263  1.747  1.363  2.121  2.814  3.04  
 
 TSEN2  80746  3  12525930  12574820  3p25.2  2.26  1.662  0.975  1.921  1.762  0.671  1.56  0.68  0.918  1.084  1.276  0.884  1.989  2.585  1.446  1.748  1.337  2.05  3.057  2.493  1.915  2.128  1.691  1.074  1.381  1.818  0.993  1.582  1.367  1.481  1.558  1.943  1.016  0.721  1.413  1.341  2.042  1.749  1.233  1.799  1.18  0.865  2.475  1.014  0.85  1.483  2.577  1.5  1.228  2.525  2.153  0.894  1.406  0.658  1.497  0.866  0.85  1.427  1.311  1.752  
 
 MKRN2OS  100129480  3  12581279  12586963  3p25.2  0.62  0.078  0.151  0  1.686  0  0.175  0.083  0.129  0.099  0.121  0.473  1.416  0.32  1.055  0.607  0.81  0.122  0.085  0.187  0  0.277  0.363  0  0  0.195  0.428  0.068  0.119  0.126  0  0  0  0  0  0  0.317  0.337  0  0  0.408  0.642  0  0  0.413  0.439  0  0.235  0  0.179  0.324  0.297  0.178  0  0  0  0  0.156  0.338  0.337  
 
 MKRN2  23609  3  12598512  12625210  3p25  2.629  3.168  2.106  2.712  2.662  2.131  3.014  2.225  2.892  2.299  2.797  1.291  2.467  3.572  2.392  1.823  2.921  3.88  4.097  2.565  2.706  2.833  2.201  1.259  2.179  2.401  2.241  2.626  2.355  1.979  2.955  3.021  1.954  1.339  1.543  2.311  3.287  2.374  2.357  2.547  1.674  1.69  2.807  2.281  1.907  2.215  1.255  2.865  2.474  4.613  3.282  1.255  2.41  1.045  2.165  1.533  1.954  2.718  2.347  3.248  
 
 RAF1  5894  3  12625099  12705700  3p25  4.291  4.074  3.318  4.016  4.405  3.067  4.149  3.428  3.491  3.766  3.943  2.064  3.641  3.849  3.623  2.904  3.245  2.924  5.237  3.128  3.146  4.163  3.27  2.206  3.133  3.346  3.202  3.541  3.171  3.141  3.108  3.423  2.894  1.744  2.582  3.167  4.408  3.448  3.826  3.745  2.939  2.589  3.967  3.307  2.84  3.409  2.395  3.673  3.472  5.276  3.904  2.46  3.344  2  3.285  2.409  2.864  3.679  3.208  4.001  
 
 TMEM40  55287  3  12775028  12810956  3p25.2  0.739  0  0.39  0.555  2.907  1.541  0.318  0.082  0.623  0.208  0.338  0.238  0.132  1.249  0.112  0.808  0.184  0.18  0.143  0.252  0  0.24  0.74  0  0.391  0.053  0.504  0.312  0  0.318  0  0.106  0  0.537  0.163  0  0.407  0  5.021  1.446  3.048  0.379  0.085  0  0  0.255  1.084  1.917  0  3.988  0.365  0.164  0.082  0.211  0.174  0.301  0  4.212  0  0.37  
 
 CAND2  23066  3  12838170  12876313  3p25.2  0.354  0.099  0.261  1.004  0.679  0.339  0.219  0.646  0.194  0.362  0.326  0.12  0.25  0.334  0.212  0.177  0.174  0.175  0.242  0.374  1.404  0.774  0.485  0.162  0.073  0.809  0.296  0.781  0.109  0.312  0.11  0.136  0.159  0.253  0.197  0.62  0.573  0.38  0.775  1.413  0.222  0.73  2.051  0.172  0.492  0.599  0.058  1.803  0.269  2.164  0.367  0.964  0.212  0.19  0.158  0.224  0.125  0.672  0.137  0.463  
 
 RPL32  6161  3  12876443  12883081  3p25-p24  7.428  9.343  6.144  7.059  8.608  7.345  7.434  6.216  6.897  6.962  6.609  6.663  6.209  7.585  5.682  7.166  8.437  9.332  9.146  10.043  7.677  8.992  7.414  6.777  7.454  5.731  6.16  5.919  5.967  6.134  6.814  6.775  6.042  5.977  7.311  6.097  6.762  6.287  9.268  6.005  6.354  7.324  7.194  7.169  6.787  6.75  7.445  6.92  6.426  9.375  6.77  5.786  6.707  5.628  7.77  6.457  5.172  7.69  6.526  7.278  
 
 IQSEC1  9922  3  12938541  13114617  3p25.2  3.314  1.854  1.91  2.277  2.314  1.532  1.945  2.383  1.908  2.177  1.548  0.926  1.501  2.42  2.519  1.291  0.655  1.247  1.823  0.424  2.646  2.319  1.931  2.353  0.867  2.709  1.825  2.523  1.779  2.894  1.961  2.204  2.092  1.123  1.833  2.35  3.025  2.746  1.896  2.458  2.066  2.077  2.803  1.981  1.73  1.987  1.784  3.12  2.034  2.917  3.128  1.976  2.5  1.133  2.013  1.479  1.96  2.689  1.97  1.944  
 
 NUP210  23225  3  13357729  13461819  3p25.1  4.609  0.516  0.174  2.724  3.209  0.387  1.005  0.08  2.849  1.819  3.138  2.097  2.185  3.705  5.04  3.259  3.74  1.437  5.046  4.438  4.469  3.707  2.885  4.005  0  3.168  0.351  2.395  2.23  2.806  2.217  0.456  0  0.21  2.967  2.886  4.135  0.032  2.379  3.311  3.168  3.484  3.032  3.38  2.393  2.957  3.492  3.717  2.971  3.531  0.845  2.747  0.112  1.165  3.312  1.108  0.033  3.201  3.21  4.338  
 
 HDAC11-AS1  100874101  3  13518487  13521553  -  0  0  0  0  0  0  0  0  0.324  0.274  0  0  0  0.156  0  0.211  0  0  0  0  0  0.12  0  0  0  0.214  0.114  0.172  0  0.243  0.431  0  0  0.455  0  0  0  0  0  0  0  0  0  0  0.456  0.558  0.813  0  0.6  0  0  0.62  0  0.178  0  0  0  0.503  0  0  
 
 HDAC11  79885  3  13521714  13547924  3p25.1  1.329  0.978  0.916  1.263  2.085  0.945  1.304  0.917  1.403  1.912  1.058  0.475  1.366  1.457  1.456  0.989  1.644  0.353  0.646  0.087  0.728  0.538  0.766  0.145  0.08  1.554  1.285  1.787  1.007  1.375  1.797  1.374  1.651  0.695  1.045  0.853  1.954  2.053  1.113  1.375  1.092  1.25  1.363  0.799  1.085  1.362  0.98  1.319  2.2  2.435  1.904  0.965  1.25  0.899  1.246  1.319  1.164  1.834  1.448  1.548  
 
 FBLN2  2199  3  13590624  13679922  3p25.1  0  0.028  0.154  5.877  0.045  0.074  2.244  0.071  0.197  0.124  0.028  0.082  0  0.074  0.043  0.073  0.049  0.144  3.908  0.094  0.103  0.204  0.336  0  0  0.197  0.168  0.062  0.031  0.125  0.101  0.047  0  0.108  0.034  0.742  1.209  3.078  0.651  2.236  0  0.481  0.171  1.924  0.65  0.981  0.062  1.914  1.835  3.052  0.129  0.78  0.037  0.107  0.078  0.088  0.25  3.186  0.051  0.082  
 
 LINC00620  285375  3  13692220  13788132  3p25.1  0  0  0  0  0  0  0  0  0  0  0  0.124  0  0.122  0  0  0  0.139  0  0  0  0  0  0  0  0  0.08  0  0  0.107  0  0  0  0  0  0  0  0  0  0  0  0.089  0.064  0  0  0.111  0.08  0  0.279  0  0  0.292  0  0  0  0  0  0  0  0  
 
 WNT7A  7476  3  13860081  13921618  3p25  0  0.062  0.146  0  0.101  0  0.251  0.05  0.117  0.079  0.089  0.11  0  0.181  0  0.089  0  0.21  0  0.209  0.274  0.395  0.33  0.306  0  0  0.15  0.046  0  0.154  0  0  0  0.16  0  0.13  0  0  0.234  0  2.427  0.379  0.117  3.58  0.946  5.136  0.386  0  4.112  0  3.784  1.209  0.047  0.26  0.155  0.165  0  1.87  0  0.941  
 
 FGD5P1  100132526  3  13974552  13978444  3p25.1  0  0.035  0.221  0  0.019  0.111  0  0  0.189  0.031  0.033  0.09  0  0.022  0  0.046  0  0.056  0.031  0.081  0.058  0.084  0.514  0  0  0  0.115  0.019  0.044  0.08  0  0.032  0  0.172  0.043  0  0  0  0.063  0  0  0.465  0.059  0  0  0.074  0.072  0  0.103  0  0.037  0.461  0.021  0.118  0.055  0  0  0  0  0  
 
 TPRXL  348825  3  13978806  14107481  3p25.1  0  0  0.12  0  0.11  0.069  0.321  0.046  0.128  0.148  0.052  2.438  2.115  0.057  0  0.059  0.076  0.14  0.042  0  0  0.153  1.275  0.978  0  0.044  0.173  0.035  0.139  0.129  0.234  0.068  0  0.106  0  0.077  0  0  0.103  0  0  0.734  0.046  0.062  0  0.086  0.039  0  0.126  0  0.051  0.43  0  0.113  0.12  0.058  0.059  0  0  0  
 
 CHCHD4  131474  3  14153576  14166371  3p25.1  3.155  2.641  2.176  3.674  3.198  1.656  3.11  1.888  2.033  2.094  2.028  1.563  3.267  3.198  2.453  1.963  2.724  1.326  4.072  3.162  3.323  3.309  3.294  2.689  2.758  2.383  2.404  2.251  2.282  3.055  3.329  3.003  2.595  1.859  2.578  2.402  3.058  2.154  3.124  2.468  1.838  1.89  3.205  2.366  1.762  2.515  2.118  2.072  1.877  3.24  2.891  1.402  1.999  1.435  2.306  1.935  1.28  2.803  2.213  2.551  
 
 TMEM43  79188  3  14166439  14185180  3p25.1  2.919  3.75  4.004  4.674  3.017  2.333  3.818  3.681  3.208  3.986  3.426  1.376  3.311  2.768  3.102  1.995  2.302  3.288  3.117  2.382  2.672  2.169  3.04  2.603  3.1  3.165  3.425  3.487  3.459  2.335  3.341  3.89  3.457  2.184  1.911  3.351  3.873  3.936  4.191  3.959  2.36  2.007  3.113  3.081  1.987  2.55  2.671  3.326  2.717  4.712  4.147  1.676  3.008  2.276  3.497  3.189  3.277  3.386  3.459  4.16  
 
 XPC  7508  3  14186647  14220172  3p25  2.503  1.399  1.528  2.806  2.692  1.29  1.916  1.445  1.921  2.204  1.89  0.884  2.465  2.819  2.513  1.468  2.003  2.443  2.691  1.782  2.739  1.948  2.398  1.456  1.997  2.551  2.112  2.271  1.621  1.999  2.332  2.657  1.7  1.119  1.929  2.292  2.435  2.206  3.529  2.603  1.465  1.889  2.74  3.04  0.784  1.933  1.826  1.576  1.639  2.708  2.143  1.324  1.517  1.29  3.444  2.344  1.493  1.422  1.809  4.139  
 
 LSM3  27258  3  14220227  14239869  3p25.1  5.265  7.897  3.902  4.453  6.793  5.354  5.613  4.227  3.616  4.834  5.371  4.921  4.415  6.085  3.841  4.939  5.609  8.636  7.192  6.567  6.011  6.212  4.661  4.64  5.196  4.337  3.733  3.448  4.157  3.052  5.366  4.726  4.139  3.275  5.11  4.027  4.854  4.529  6.741  3.731  5.107  5.055  5.208  4.976  4.496  5.109  5.441  4.348  4.228  6.513  5.135  3.02  4.966  4.219  5.831  4.883  4.016  6.23  5.319  5.472  
 
 LINC01267  101927565  3  14389950  14394068  -  0  0.09  0.166  0  0  0  0  0  0.111  0.056  0.039  0  0  0.048  0.077  0  0  0.131  0.031  0.072  0  0.095  0.33  0  0  0  0.081  0.035  0  0.054  0  0  0  0.23  0  0  0.04  0  0.048  0  0  0.496  0.038  0  0  0  0.532  0  0  0.038  0  0.267  0  0.085  0  0.085  0  0  0  0  
 
 SLC6A6  6533  3  14444075  14530857  3p25.1  2.736  1.014  2.097  1.417  2.725  1.124  3.187  2.291  1.958  4.232  2.344  3.352  4.212  1.786  3.286  2.303  1.993  0.249  2.855  1.974  3.664  2.389  2.049  2.136  2.815  2.297  2.794  2.617  2.028  1.813  2.072  2.221  1.914  0.942  3.052  2.026  4.076  2.07  2.847  2.279  2.618  3.005  1.732  2.868  0.627  1.632  1.814  1.809  0.379  1.982  3.114  2.873  3.16  2.452  4.167  3.616  3.05  3.452  2.802  4.527  
 
 GRIP2  80852  3  14530618  14581850  3p24-p23  0.115  0.031  0.202  0.079  0.185  0.027  0.105  0.051  0.091  0.249  0.052  0.1  0.101  0.09  0.079  0.08  0.054  0.082  0.037  0.177  0.103  0.192  0.42  0.067  0.045  0.09  0.203  0.044  0.066  0.098  0.526  0.116  0.03  0.186  0.232  0.094  0.168  0.118  0.362  0.075  0.095  0.184  0.067  0.074  0  0.101  0.028  0.188  0.031  0.168  0.466  0.487  0.087  0.128  0.328  0.085  0.137  0.179  0.106  0.108  
 
 CCDC174  51244  3  14693252  14714166  3p25.1  2.107  1.61  1.081  1.176  1.935  1.17  2.165  1.017  1.343  1.402  1.434  0.724  1.432  2.235  1.323  1.1  1.818  1.195  2.709  2.506  1.6  2.042  1.373  0.861  1.181  1.294  1.658  1.084  1.207  1.478  1.663  1.572  1.152  0.715  1.087  1.149  1.632  1.395  2.156  1.325  1.365  0.906  1.716  1.467  0.963  1.229  2.23  1.368  0.896  2.404  1.892  0.888  1.343  0.737  1.711  0.941  1.258  1.639  1.476  1.736  
 
 C3orf20  84077  3  14716605  14814543  3p25.1  0  0.045  0.099  0  0.035  0.079  0  0.027  0.054  0.036  0.043  0  0  0.094  0  0.061  0.07  0  0.031  0  0  0.165  0.198  0.103  0  0  0.068  0.055  0  0.067  0  0.058  0  0.089  0  0  0.042  0  0.085  0  0.047  0.074  0.044  0  0  0.075  0.022  0  0  0.033  0.057  0.325  0.029  0.046  0  0.087  0.045  0  0  0  
 
 FGD5  152273  3  14860468  14976072  3p25.1  0.04  0.03  0.15  0  0.301  0.03  0.157  0.041  0.127  0.116  0.033  2.094  0.111  0.098  0.031  0.083  0.096  0.456  0.54  0.117  0.063  0.611  0.593  1.614  0.049  0.071  0.228  0.07  0.071  0.181  0.03  0  0.051  0.144  0.06  0.027  0.092  0  0.104  0  0  0.194  0.173  0.028  0  0.104  0.166  0  0.037  0.017  0.067  0.297  0.041  0.134  0.065  0.065  0.048  0.026  0.056  0.02  
 
 FGD5-AS1  100505641  3  14984285  14989948  -  3.597  4.43  2.839  2.736  4.119  2.295  4.22  3.416  3.381  3.717  3.442  2.111  3.711  3.304  3.023  3.03  3.268  3.61  5.239  2.617  2.659  3.227  2.743  2.041  3.49  3.713  3.528  3.43  3.13  2.875  3.297  3.158  3.113  2.256  2.764  3.077  3.687  3.498  3.896  3.476  3.587  2.533  4.118  2.616  2.066  2.779  2.435  3.067  2.333  4.272  4.148  2.264  3.119  1.557  3.378  2.413  2.679  3.235  3.467  4.175  
 
 NR2C2  7182  3  14989090  15090786  3p25  2.223  1.916  1.333  1.408  1.699  1.354  1.513  1.993  1.633  1.848  2.095  0.811  1.97  2.101  2.066  1.549  1.313  0.957  2.561  1.478  1.751  2.002  0.72  0.931  1.415  2.123  1.921  2.419  1.981  1.848  1.805  1.632  2.099  0.88  1.263  1.785  2.437  1.694  1.63  2.203  1.551  1.063  1.822  1.479  1.388  1.579  0.574  1.768  1.166  1.708  2.197  1.11  1.987  0.775  1.607  1.056  1.883  1.681  1.922  1.928  
 
 MRPS25  64432  3  15090018  15106816  3p25  3.372  2.213  1.458  1.837  2.939  1.54  2.473  1.298  1.722  1.794  1.93  2.097  2.737  2.859  2.089  2.159  2.393  3.022  3.785  3.406  2.962  2.641  2.21  2.376  2.338  2.827  2.699  2.462  2.847  3.319  3.274  2.892  2.84  1.976  2.291  1.922  2.509  2.302  2.879  1.899  1.512  2.03  2.659  1.942  1.655  2.03  2.441  2.27  1.111  3.397  2.265  1.396  2.354  1.079  2.239  1.771  1.491  2.249  2.188  2.436  
 
 RBSN  64145  3  15111575  15140664  3p25.1  2.175  2.201  1.539  1.592  2.32  1.711  2.006  1.441  1.536  1.615  1.847  0.443  1.778  1.833  1.759  0.942  1.983  1.534  2.029  1.534  1.783  1.235  1.088  0.769  1.591  1.652  1.205  1.922  1.414  1.719  1.943  1.851  1.94  1.108  1.108  1.468  1.973  1.725  1.874  1.696  1.493  0.98  1.952  1.559  1.483  1.138  1.451  1.819  1.191  2.227  2.093  0.973  1.65  0.737  1.508  1.148  1.327  2.488  1.647  2.072  
 
 COL6A4P1  344875  3  15206868  15247466  3p25.1  0.143  0  0.125  0  0.096  0  0  0  0.066  0.038  0.03  0.099  0.074  0.353  0.147  0.114  0.069  0.098  0.068  0.066  0  0.18  0.352  0  0  0  0.046  0  0  0.061  0  0  0  0.267  0  0  0  0.091  0.046  0.085  0  0.084  0.028  0  0  0  0.022  0  0  0  0  0.395  0  0.032  0  0  0  0  0  0  
 
 CAPN7  23473  3  15247732  15294423  3p24  1.358  1.412  0.941  0.908  1.85  0.933  1.228  1.525  1.357  1.41  1.445  0.601  1.924  1.397  1.847  0.893  1.05  0.969  2.033  0.918  1.563  1.177  0.878  0.609  0.91  1.72  0.885  1.164  1.341  0.977  1.864  1.524  1.216  0.606  0.926  1.562  1.627  1.36  1.518  1.451  1.379  1.034  0.881  1.508  0.657  1.408  0.072  1.253  1.343  1.416  1.537  0.602  1.069  0.638  0.951  0.969  1.229  1.195  1.148  1.785  
 
 SH3BP5-AS1  100505696  3  15295690  15306005  3p25.1  1.003  0.863  0.693  0.282  1.188  0.613  0.916  0.699  0.594  1.256  1.257  0.261  0.835  1.253  1.162  0.619  0.431  0.972  0.948  0.326  0.491  1.896  1.183  0.611  0.208  1.192  1.708  1.129  1.296  0.706  1.294  1.135  1.196  0.685  0.437  0.745  0.905  1.075  1.138  0.457  0.583  0.742  1.278  1.106  0.385  0.683  0.82  0.767  0.811  0.771  0.627  0.442  0.872  0.32  0.862  0.197  0.805  0.341  0.803  0.444  
 
 SH3BP5  9467  3  15295862  15382901  3p24.3  2.425  1.05  1.382  0.594  1.822  0.9  2.36  2.573  0.867  2.307  3.474  1.709  1.071  2.042  2.087  1.045  1.289  0.555  0.548  0.295  1.792  3.665  2.743  3.191  1.851  2.968  1.622  3.779  2.966  2.121  1.534  3.447  3.454  1.923  1.265  2.519  1.162  0.531  3.079  1.041  1.455  1.64  1.776  0.731  0.874  1.569  1.533  1.308  0.75  1.702  2.212  1.019  1.41  1.287  1.053  0.765  1.361  1.886  1.116  2.119  
 
 METTL6  131965  3  15451266  15469054  3p25.1  2.358  2.418  1.368  1.722  2.422  1.071  2.317  1.699  1.727  1.775  1.955  0.897  1.349  1.834  1.291  1.344  1.506  2.341  3.063  1.912  1.485  1.926  1.672  0.913  1.585  1.909  1.263  1.844  1.482  1.516  1.933  2.212  0.959  0.783  1.511  1.673  1.88  1.789  2.561  1.616  1.437  1.287  3.009  1.209  1.204  2.025  1.793  1.332  0.604  2.948  2.072  0.972  1.656  0.899  1.662  0.977  1.121  1.485  1.797  2.041  
 
 EAF1  85403  3  15469063  15484120  3p25.1  2.149  1.226  1.048  1.651  1.942  1.298  1.806  1.648  1.57  1.948  1.788  1.18  2.306  2.223  2.056  1.222  2.115  1.457  2.531  1.11  1.995  1.585  1.324  0.921  1.44  2.015  0.953  2.513  1.466  1.033  1.66  1.746  1.141  0.785  0.962  1.499  2.29  1.494  1.867  1.626  1.624  0.99  1.354  1.511  1.626  1.465  0.365  1.464  1.157  1.889  2.203  0.853  1.261  0.83  1.025  0.914  1.072  1.613  1.446  1.957  
 
 COLQ  8292  3  15491639  15563258  3p25  0.196  0  0.098  0.147  0.479  0.081  0.1  0.118  0.305  0.654  0.297  0.039  0.202  0.615  0.349  0  0.208  0.084  0.428  0.355  0  0.339  0.371  0.05  0.048  0.07  0.135  0.062  0  0.119  0.063  0.204  0  0.21  0.115  0.058  0.106  0.3  0.57  0  0.045  0.242  0.167  0.193  0.111  0.069  0.044  0.235  0.202  0.45  0.147  0.37  0.03  0.058  0.079  0.074  0  0.737  0  0.08  
 
 HACL1  26061  3  15602210  15643359  3p25.1  2.045  2.193  1.137  1.46  1.585  0.854  2.168  1.508  1.242  1.965  1.464  0.947  1.963  2.15  1.815  1.694  2.13  3.254  3.528  2.478  1.616  2.065  1.175  0.736  1.946  2.15  2.008  2.193  2.335  2.623  1.685  2.423  1.896  1.226  1.227  1.191  1.917  1.579  2.531  2.028  1.548  1.296  1.828  1.534  1.433  1.136  0.829  1.386  1.296  3.323  2.174  1.415  1.518  0.711  2.393  1.281  1.434  3.487  1.938  2.869  
 
 BTD  686  3  15642858  15687328  3p25  1.956  3.444  1.585  2.803  3.081  1.302  2.869  2.313  2.428  3.046  1.38  2.048  1.667  1.718  1.489  1.628  1.781  3.278  1.925  2.546  0.895  2.081  1.857  1.887  1.06  2.52  2.442  2.659  2.916  2.754  1.965  1.686  1.954  1.403  1.433  1.409  2.279  1.922  2.778  2.175  1.386  2.551  1.739  1.342  1.037  1.008  1.354  2.309  1.238  3.342  2.042  0.956  1.945  1.285  2.576  1.819  1.097  3.655  1.954  2.121  
 
 ANKRD28  23243  3  15708743  15901053  3p25.1  1.973  3.086  1.617  1.964  1.353  1.476  2.391  2.005  1.675  2.391  2.434  1.087  2.02  2.245  1.952  1.669  1.852  1.835  2.994  1.932  1.988  1.713  1.492  1.305  1.329  4.471  2.201  3.763  3.402  3.294  2.393  2.404  2.554  1.504  1.417  1.226  3.049  1.753  3.154  2.355  1.9  1.086  2.56  1.533  1.46  1.735  1.248  1.764  1.52  2.328  2.395  1.491  1.927  1.063  2.46  1.616  1.649  3.02  2.326  2.748  
 
 GALNT15  117248  3  16216183  16271253  3p25.1  0  0.056  0.279  0  0.027  0  0.211  1.102  0.095  0.93  0.026  0  0  0.016  0.033  0.022  0  0  0.021  0  0  0.083  0.272  0.106  0.029  0.035  0.323  0.093  0  0.051  0.034  0  0.072  0.221  0  0  0.023  0.109  0.274  0.067  0  0.134  0.034  0  0  0.017  0.11  0  0  0  0  0.494  0.022  0.022  0  0  0.042  0.104  0  0  
 
 DPH3  285381  3  16298567  16306496  3p25.1  1.293  3.382  1.783  2.13  1.845  1.614  2.445  1.655  2.222  1.813  1.645  0.654  1.047  1.846  0.776  1.05  1.216  3.085  3.06  1.902  2.151  1.47  1.297  0.809  1.914  1.606  1.544  1.717  1.293  1.051  2.302  1.605  1.644  1.542  1.391  1.405  1.979  1.715  3.038  1.529  1.301  1.276  1.59  1.333  1.393  1.336  1.782  2.42  0.734  4.468  1.779  0.66  1.379  0.727  1.557  0.833  1.393  1.978  1.393  2.052  
 
 OXNAD1  92106  3  16306666  16347594  3p25-p24  1.626  1.888  0.859  0.883  1.252  0.549  1.612  0.86  0.895  0.905  1.012  0.736  1.729  1.134  0.959  0.835  1.48  1.734  2.367  1.479  1.857  1.252  1.069  1.021  0.829  1.486  1.033  1.224  1.213  1.449  1.823  1.392  1.5  0.906  0.766  1.2  1.851  1.047  0.892  0.867  0.811  0.477  1.463  0.758  0.932  1.198  0.371  1.511  0.42  2.554  1.37  0.612  0.981  0.418  1.037  0.54  0.63  1.241  0.737  1.231  
 
 RFTN1  23180  3  16357351  16555222  3p24.3  0.747  4.088  4.288  1.431  0.261  2.13  4.387  3.185  0.362  2.631  0.562  0.052  0.545  1.352  0.528  0.134  0.222  0.14  4.048  2.98  0.465  2.691  2.178  2.243  3.35  0.661  2.231  0.861  0.819  0.726  2.491  0.945  1.653  0.552  0.773  0.761  3.141  1.823  4.487  0.355  0.461  0.359  0.442  0.925  0.416  0.925  0.073  3.636  0.183  4.281  0.359  0.8  2.444  1.204  3.002  2.124  2.723  2.108  1.98  2.986  
 
 LINC00690  100996597  3  16577824  16582880  3p24.3  0  0  0  0  0.051  0  0.237  0  0  0  0  0.491  0  0  0  0  0  0.124  0  0.1  0  0.063  0  0  0  0  0.551  0  0  0.45  0  0  0  0  0  0  0  0  0  0  0  0  0.055  0  0  0.06  1.572  0  0  0  0  0  0  0.075  0  0  0  0  0  0  
 
 DAZL  1618  3  16628300  16647006  3p24.3  0  0  0.225  0  0.02  0  0  0  0.04  0  0  0.048  0  0.023  0  0  0  0.074  0  0  0  0.03  0.137  0  0  0  0.045  0  0  0.048  0  0  0  0.11  0  0  0  0  0.023  0  0  0.414  0.016  0  0  0  0.067  0  0  0  0  0.162  0  0.062  0.029  0  0  0  0  0  
 
 PLCL2  23228  3  16926451  17132098  3p24.3  0.077  0.759  0.347  0.296  0.057  0.814  1.002  0.547  0.675  0.286  0.916  0.031  0.844  0.9  0.304  0.339  0.943  0.144  2.283  0.845  0.104  1.188  1.145  1.148  0.597  0.937  0.649  0.457  0.51  0.627  0.037  0.421  0.262  0.42  0.298  0.034  0.829  0.922  0.695  0.092  0.383  0.573  0.07  0.043  0.096  0.035  0.537  0.516  0.221  0.997  0.849  0.344  0.021  0.291  0.502  0  0.441  0.573  0  0.938  
 
 TBC1D5  9779  3  17198653  17782399  3p24.3  2.496  1.644  1.688  1.46  2.636  1.394  3.091  2.296  2.226  2.568  2.211  1.296  1.4  2.39  2.139  1.143  1.95  2.044  3.358  2.577  2.066  2.467  1.582  1.479  2.205  2.684  2.285  2.379  1.911  2.529  2.293  2.342  2.248  1.458  1.871  2.939  3.728  2.713  1.963  2.709  2.625  1.453  2.38  1.944  1.534  1.921  1.223  1.589  2.254  1.795  3.27  1.558  2.2  1.232  2.584  1.993  1.49  2.856  1.676  3.496  
 
 LOC339862  339862  3  18004063  18310410  3p24.3  0.417  0  0.124  0  0  0  0  0  0.075  0.29  0  0  0  0.312  0  0.047  0  0.221  0.323  0.113  0.103  1.399  0.236  0  0  0  0.099  0  0  0.075  0  0  0  0.239  0  0  0  0  0.023  0  0  0.025  0.028  0  0  0.119  0.073  0  0  0  0  0.088  0.045  0.037  0  0  0  0  0  0  
 
 SATB1  6304  3  18389132  18480265  3p23  0  1.074  0.545  0.478  5.056  1.225  0.327  0.331  1.311  1.743  0.791  0.516  1.154  0.684  0.61  1.852  2.977  1.538  6.143  2.727  0  4.484  1.074  0.807  0.571  0.424  0.448  0.914  0.072  0.634  0.592  1.047  0.377  0.393  0.085  0.298  1.1  0.672  0.286  0.098  1.263  0.448  0.059  0  0.178  0.094  0.922  0.319  0.761  1.445  1.034  0.167  1.345  0.806  0.034  0.081  0.031  0.231  0.031  1.272  
 
 SATB1-AS1  101927777  3  18486728  18571606  3p24.3  0  0  0  0  0.421  0  0  0  0  0  0  0  0  0.065  0  0.37  0.722  0  1.234  0.219  0  0.408  0  0.167  0  0  0.165  0  0  0.138  0  0  0  0  0  0  0  0  0  0  0  0  0  0  0  0  0.068  0  0  0  0  0.327  0.125  0.1  0  0  0  0  0  0  
 
 KCNH8  131096  3  19190016  19577135  3p24.3  0  0  0.104  0  0.035  0  0  0.012  0.073  0.113  0.015  0.209  0.358  0.016  0.154  0.023  0.707  1.174  0.414  0.032  0  0.131  0.207  0  0  0.634  0.039  0.135  0.136  0.275  0.063  0  0  0.13  0  0  0.062  0  0.023  0  0  0.081  0.025  0  0  0  0.015  0  0  0.162  0  0.176  0.014  0.054  0.03  0.036  0  0  0  0  
 
 EFHB  151651  3  19920965  19975706  3p24.3  0.076  0.061  0.046  0  0.089  0  0  0  0.076  0.034  0.051  0  0  0.07  0  0  0  0.042  0  0.041  0  0.051  0.112  0  0  0  0.034  0.156  0  0.02  0  0  0  0  0.237  0.077  0  0  0.018  0  0.053  0.285  0.107  0  0  0.142  0  0  0  0  0  0.12  0.23  0.07  1.752  0.538  0  0  0.124  0.097  
 
 RAB5A  5868  3  19988571  20026667  3p24-p22  2.903  3.638  2.134  2.331  2.489  2.396  2.542  2.609  2.739  2.725  3.015  1.629  2.882  3.365  2.187  2.078  3.031  2.939  4.003  2.723  2.449  2.277  1.704  1.044  2.621  2.728  2.207  2.509  2.183  1.573  2.541  2.93  2.513  1.877  1.997  2.474  2.93  2.307  3.807  2.024  2.775  1.755  2.439  2.308  1.929  2.674  1.46  2.239  2.093  3.829  3.29  1.42  2.375  1.692  2.432  2.196  2.428  2.621  2.757  3.81  
 
 PP2D1  151649  3  20021452  20053765  3p24.3  0.171  0.055  0.11  0  0.077  0.064  0  0.109  0.073  0.044  0.077  0.088  0  0.105  0.186  0.067  0.129  0  0.085  0  0  0.113  0.277  0  0  0.131  0.124  0.057  0  0.056  0  0.086  0.194  0.188  0.073  0.133  0.106  0  0.078  0  0.096  0.056  0.022  0  0  0.06  0.053  0  0  0  0.06  0.097  0.069  0  0.045  0  0  0.166  0.124  0  
 
 KAT2B  8850  3  20081523  20195896  3p24  0.563  1.673  0.621  0.732  0.764  0.995  0.668  0.939  0.81  1.398  1.642  0.131  0.224  0.713  0.715  0.125  0.717  0.569  1.54  0.526  0.714  0.558  0.736  0.556  0.363  2.434  2.017  2.081  1.595  1.185  1.81  2.477  2.478  1.845  0.578  1.134  0.973  0.5  1.578  0.856  0.673  0.447  0.3  0.314  0.326  0.756  0.111  0.509  0.72  0.878  1.25  0.638  0.516  0.718  0.521  0.429  0.467  1.182  0.52  1.532  
 
 SGOL1  151648  3  20202084  20227725  3p24.3  2.931  3.956  1.313  1.59  2.042  1.873  2.215  1.877  0.356  2.118  2.54  1.335  1.759  2.592  2.005  1.652  2.4  4.078  3.726  1.858  2.34  2.497  1.132  1.154  2.003  1.862  1.568  1.567  1.479  1.085  2.234  1.687  1.938  1.27  1.88  1.801  2.572  2.046  1.949  1.862  2.075  1.192  2.392  1.769  1.095  2.163  1.777  1.769  1.437  2.424  2.119  1.201  1.785  1.261  1.853  1.273  1.663  2.548  2.08  2.601  
 
 SGOL1-AS1  100874028  3  20215735  20227919  -  0.314  0.15  0.358  0.946  0  0.527  0.349  0  0.212  0.132  0  0.344  0  0.076  0  0.305  0.425  0  0.279  0  0.476  0.643  0.347  0  0  0.177  0.56  0  0.509  1.043  0  0  0.253  0.402  1.032  0  0  0  0.135  0  0.254  0.658  0  0  0.737  0.357  0.064  0.665  0  0  0  0.816  0.245  0.161  0.472  0  0  0  0  0  
 
 LOC101927829  101927829  3  20383959  20392420  -  0  0  0.075  0  0  0  0.058  0  0.086  0  0  0.207  0  0  0  0  0  0  0  0  0  0  0  0  0  0  0.282  0  0  0.17  0.054  0.066  0  0  0  0  0  0  0  0  0  0.126  0.033  0  0  0.051  0.259  0  0  0  0  0  0  0  0  0  0  0  0  0  
 
 VENTXP7  391518  3  21447217  21448177  3p24.3  0  0  0.145  0  0  0  0  0  0.164  0.073  0  0.108  0  0  0  0  0  0  0  0  0  0.167  0.481  0  0  0  0.077  0  0  0.092  0  0  0  0.259  0  0  0  0  0.065  0  0  0.305  0  0  0  0  0  0  0  0  0  0.49  0  0.081  0  0  0  0  0  0  
 
 ZNF385D  79750  3  21462489  21792816  3p24.3  0  0  0  1.641  0  0.362  1.289  0.629  0.12  2.167  0.197  0  0  0  0  0  0  0  0.278  0.098  0.149  0.058  0.133  0  0  0  0.092  0  0  0.085  0  0  0  0.152  0  0  0.787  1.483  0.142  0  0.283  0.296  0.146  0  0  0  0.034  0  0.666  0.097  2.449  0.526  0.415  0.09  0.644  0  0.771  1.048  0  0.247  
 
 ZNF385D-AS1  100874216  3  21584280  21621451  -  0  0  0  0  0  0  0  0  0  0.172  0  0  0  0  0  0  0  0  0.199  0.437  1.524  0.094  0  0  0  0  0  0  0  0.045  0  0  0  0  0  0  0  0  0  0  0  0  0  0  0  0  0  0  0  0  0  0  0  0  0  0  0  0  0  0  
 
 ZNF385D-AS2  100874221  3  21998630  22021320  -  0  0  0  0  0  0  0  0  0  0  0  0  0  0  0  0  0  0.355  0  0  0  0  0  0  0  0  0  0  0  0  0  0  0  0  0  0  0  0  0  0  0  0  0  0  0  0  0  0  0  0  0  0  0  0  0  0  0  0  0  0  
 
 UBE2E2  7325  3  23244783  23632296  3p24.2  2.628  4.131  2.849  3.253  3.371  2.465  0.252  2.986  2.644  2.45  2.631  0.061  2.177  2.383  2.534  0.454  0  3.449  4.21  2.533  0.449  1.563  2.69  2.281  2.969  0.347  1.738  1.762  1.667  2.667  0.24  2.281  0  0.119  2.247  2.089  3.365  3.103  2.923  2.522  2.689  1.629  2.204  2.965  2.355  3.173  2.493  2.604  2.286  4.319  2.628  1.838  3.112  1.333  2.454  2.288  2.652  3.966  3.152  3.18  
 
 UBE2E1-AS1  100874092  3  23845514  23848396  -  0  0  0  0  0  0  0  0  0  0.09  0  0  0  0.154  0  0  0  0  0  0  0  0.096  0  0  0  0  0.052  0  0  0.065  0  0  0  0  0  0  0  0  0  0  0  0.085  0  0  0  0  0.074  0  0  0.148  0  3.762  0  0  0  0  0  0  0  0  
 
 UBE2E1  7324  3  23847383  23933131  3p24.2  1.775  2.995  2.065  1.965  2.776  3.303  2.148  2.644  2.027  2.152  2.584  2.26  2.461  3.84  2.465  2.579  3.297  3.517  4.359  5.87  3.659  3.506  2.268  3.205  3.57  1.989  1.487  1.949  2.15  1.264  2.678  2.893  1.839  1.647  3.317  2.048  2.99  2.163  4.797  1.643  2.982  2.592  2.28  3.765  2.587  2.784  3.596  2.264  2.232  4.446  3.127  1.913  3.052  2.128  3.186  2.867  2.158  3.603  2.949  3.225  
 
 NKIRAS1  28512  3  23933571  23958537  3p24.2  0.834  2.332  0.87  0.866  1.559  0.927  1.781  1.284  1.484  1.17  1.662  0.557  0.937  2.204  0.891  0.509  0.941  1.123  2.235  0.753  0.486  0.499  0.798  0.375  1.219  1.643  0.865  2.032  1.645  0.557  1.605  1.637  1.473  1.103  0.78  1.36  1.612  1.546  1.176  1.146  0.956  1.179  1.891  0.874  1.152  1.308  0.767  1.025  0.736  2.038  1.475  0.467  1.483  0.715  1.109  0.887  1.184  1.239  1.774  1.474  
 
 RPL15  6138  3  23958294  23965187  3p24.2  7.978  7.567  6.995  6.929  8.38  7.036  8.333  7.06  7.742  7.231  7.447  6.739  7.095  8.568  6.867  6.895  8.416  7.637  9.648  9.548  7.464  8.522  8.108  6.919  7.699  6.7  7.355  6.902  7.144  7.164  7.494  7.455  6.981  6.379  7.169  6.467  7.868  7.334  8.847  6.532  6.715  7.494  8.137  7.388  6.795  6.939  7.764  7.423  5.769  9.44  7.204  5.637  7.439  5.629  7.763  6.802  6.112  8.404  7.307  7.784  
 
 NR1D2  9975  3  23986750  24022109  3p24.2  1.583  2.536  1.144  0.951  1.725  1.443  2.069  2.197  2.373  1.95  1.601  0.771  1.766  2.976  1.774  1.825  1.913  0.962  0.818  0.896  0.444  1.106  0.941  0.385  1.171  1.099  1.403  1.764  1.241  1.183  1.145  1.288  1.776  0.983  0.897  0.918  2.464  1.771  2.651  0.87  1.884  1.196  0.603  0.8  1.662  1.156  0.369  1.343  1.346  1.353  2.794  0.884  1.963  0.799  1.693  1.474  1.525  2.135  2.476  2.418  
 
 LINC00691  152024  3  24141464  24144738  3p24.2  0.143  0.213  0.214  0  0.038  0  0.409  0.048  0.102  0.139  0.029  0.047  0  0  0  0  0  0  0  0.107  0  0.141  0.282  0.289  0  0  0.057  0  0.342  0.062  0  0  0  0.228  0.122  0.05  0.148  0  0.107  0  0  0.342  0.042  0  0.378  0.041  0.646  0.201  0  0  0  0.156  0.093  0.056  0.069  0.092  0.116  0  0  0  
 
 THRB  7068  3  24158644  24536313  3p24.2  0.149  0.067  0.143  0.092  1.096  0.13  0.129  0.787  0.6  0.682  0.319  0.436  0.225  0.241  0.035  0.056  0.999  0.089  0.02  0.023  0.398  0.095  0.267  0  0  0  0.194  0.078  0.025  0.081  0.041  0.578  0.033  0.158  0.285  1.018  0.441  0.065  0.299  0.059  1.322  0.367  0.065  0.162  0.863  1.318  0.775  0.081  1.164  0.115  0.592  0.233  1.025  0.744  0.876  1.15  1.608  0.308  2.032  1.003  
 
 LOC101927854  101927854  3  24192814  24231447  3p24.2  0  0.178  0  0  0  0.262  0.217  0  0  0.135  0.216  0  0  0.171  0  0  0.241  0  0  0  0.52  0.174  0  0  0  0  0.119  0  0  0.134  0  0  0  0  0  0  0.362  0  0  0  0.198  0  0.103  0.411  0  0  0.13  0  0  0  0  0  0.167  0.145  0  0  0.206  0  0.546  0  
 
 THRB-AS1  644990  3  24535577  24541502  3p24.2  0  0  0  0  0.585  0  0  0  0  0.235  0  0  0  0  0  0  0  0.307  0  0  0  0  0  0  0  0  0.065  0  0  0.051  0  0  0  0  0  0.218  0  0  0.106  0  0.134  0  0  0  0.328  0.269  0  0  0  0  0.194  0.396  0.182  0.18  0.188  0  0.286  0  0.42  0  
 
 RARB  5915  3  25215822  25639422  3p24.2  0.197  0.171  1.511  0  0.151  0.62  2.542  0.543  1.464  1.591  2.671  0.065  0  0.174  0.124  0.126  0.131  0.63  0.171  0.289  0  0.391  0.162  0  1.789  0.846  1.204  0.919  0  0.074  0.106  1.131  1.076  0.773  0.925  1.157  0.729  0.196  0.186  1.549  0.24  1.451  2.782  0.099  0  0.261  0.421  0.151  0.341  0.711  0.154  0.226  0.045  0.356  0.202  0.869  0  0.184  0  1.793  
 
 TOP2B  7155  3  25639395  25705863  3p24  2.961  3.628  2.04  2.115  3.011  1.688  2.129  2.588  2.143  2.714  2.953  1.116  2.537  3.257  2.681  1.873  3.302  3.848  5.395  4.233  2.737  3.734  2.203  1.527  2.457  2.913  1.66  2.372  2.249  1.745  2.576  2.65  1.796  1.203  1.964  3.322  2.914  2.208  3.066  2.656  2.415  1.764  2.881  2.558  2.024  2.477  1.994  1.732  2.24  3.412  3.04  1.408  2.206  1.211  2.466  1.911  2.502  2.432  2.023  3.718  
 
 NGLY1  55768  3  25760434  25831530  3p24.2  1.514  3.691  1.8  1.837  2.562  1.413  2.363  1.788  1.853  2.04  2.626  1.353  2.416  2.699  1.769  1.44  1.843  3.884  4.806  3.215  3.069  2.871  2.133  1.719  1.826  1.957  1.52  2.167  1.797  1.409  2.345  2.163  1.721  1.402  1.553  2.431  2.213  2.23  3.069  1.874  1.924  1.58  2.038  1.57  1.14  1.558  1.852  1.701  1.033  3.32  2.858  1.403  2.136  0.817  2.068  1.715  1.674  2.122  1.752  2.629  
 
 OXSM  54995  3  25831562  25836025  3p24.2  2.914  3.033  1.731  2.316  3.017  0.778  2.519  1.185  1.86  1.969  2.475  1.984  2.704  3.412  2.046  1.891  3.031  2.94  3.814  2.819  2.619  2.675  2.717  1.851  1.9  2.425  2.227  1.921  2.09  2.339  2.829  2.807  1.955  1.384  1.696  2.446  2.323  1.842  2.436  2.214  1.748  1.794  2.079  2.199  1.412  1.53  1.289  1.837  1.037  3.574  2.251  1.352  1.786  0.973  2.414  1.661  1.221  1.753  2.005  2.588  
 
 LINC00692  285326  3  25900022  25915186  3p24.2  0  0  0  0  0  0  0  0  0  0  0  0  0  0  0  0  0  0  0  0  0  0  0  0  0  0  0.066  0  0  0.058  0  0  0  0  0  0  0  0  0  0  0  0  0  0  0  0  0.064  0  0  0  0  0  0  0  0  0  0  0  0  0  
 
 LRRC3B  116135  3  26664299  26752265  3p24  0  0.077  0.148  0.162  0.034  0  0  0  0.098  0.054  0  0  0  0  0  0  0  0.092  0.075  0  0  0.211  0.405  0  0  0  0.095  0  0  0.131  0  0  0  0.238  0  0  0  0  0.089  0  0  0.277  0  0  0  0  0.08  0  0  0  0  0.863  0  0.13  0.066  0  0  0  0  0  
 
 NEK10  152110  3  27152393  27410951  3p24.1  0  0.059  0.204  0  0.152  0.196  0.365  0.09  0.165  0.509  0.273  0  0  0.112  0.128  0.04  0  0  0.097  0.055  0  0.137  0.195  0  0.184  0.054  0.101  0.257  0.108  0.051  0  0.148  0.179  0.181  0.079  0.068  0.09  0  0.137  0.271  0.07  0.131  0.095  0.581  0.103  0.078  0.03  0.072  0.095  0.102  0.147  0.272  0.121  0.255  0.042  0  0.24  0  0.161  0.061  
 
 SLC4A7  9497  3  27414211  27525911  3p22  0.571  1.702  1.682  2.138  1.52  1.266  2.825  1.371  1.598  2.046  2.431  0.47  1.364  1.621  1.141  0.907  1.627  2.67  1.794  1.76  0.529  1.854  0.402  0.426  1.491  1.945  1.059  1.412  1.477  0.436  1.438  1.932  1.525  0.877  1.23  1.243  1.843  0.829  1.975  2.202  0.93  0.877  1.382  1.831  1.074  1.092  1.324  0.93  1.286  1.355  2.876  0.768  1.382  0.919  1.074  1.436  1.231  1.388  1.207  2.295  
 
 EOMES  8320  3  27757439  27764206  3p24.1  0.607  0.037  0.147  0.502  0.025  0  0.119  0.045  0.166  0.053  0.088  0.033  0  0.672  0.153  0.029  0  0.475  0.773  0.1  0  0.864  0.279  0.041  0.14  0.093  0.151  0.267  0.116  0.623  0.117  0.137  0  0.17  0.202  0.145  0  0  0.059  0.211  0.198  0.265  0.212  0  0.098  0.058  0.061  0.238  0.082  0.626  0.048  0.597  0  0.104  0.037  0  0  0  0  0  
 
 CMC1  152100  3  28283123  28361263  3p24.1  2.861  6.174  2.05  2.969  3.953  3.449  3.524  2.731  3.147  2.917  3.512  3.439  3.202  4.213  1.614  2.996  4.509  7.504  4.923  3.947  4.44  3.755  2.177  2.185  3.372  3.282  1.764  2.152  2.249  1.115  3.011  3.253  2.353  2.739  3.674  3.344  2.915  2.822  4.391  2.228  2.788  3.389  2.891  3.271  2.701  3.319  2.328  2.192  2.794  4.783  3.507  1.841  2.465  2.135  3.785  2.954  2.222  3.659  3.076  3.801  
 
 AZI2  64343  3  28363843  28390618  3p24.1  2.047  4.174  2.073  2.06  2.637  2.044  2.199  2.219  2.552  2.158  2.518  1.198  1.95  3.151  1.648  2.228  2.435  4.713  3.778  2.574  2.365  2.452  1.12  0.957  2.507  2.117  1.345  1.76  1.798  1.152  2.322  2.397  1.964  1.541  1.74  2.398  2.174  2.167  3.002  1.956  2.172  1.92  2.489  1.999  1.122  1.666  2.42  2.058  1.65  4.273  2.672  1.621  1.861  1.219  2.11  1.559  1.84  2.561  1.741  2.704  
 
 ZCWPW2  152098  3  28390639  28566632  3p24.1  0.108  0.403  0.13  0  0.112  0.08  0  0.09  0  0.141  0  0  0  0.274  0  0  0.067  0.093  0.17  0  0  0.106  0  0  0  0.166  0.04  0  0  0.031  0  0  0  0.103  0.082  0.142  0.088  0.155  0.095  0  0.064  0.208  0.313  0.125  0  0  0  0  0  1.387  0.156  0.09  0  0.052  0.043  0  0.102  0  0.072  0  
 
 LINC00693  645206  3  28616768  28799828  3p24.1  0  0  0.121  0  0.041  0  0  0.326  0.214  0.097  0.261  0  0  0.085  0  0  0  0.223  0  0  0  0.104  0  0  0  0  0.119  0  0  0.112  0  0  0  0  0  0  0.115  0  0.039  0  0  0.065  1.569  0  0  0.129  0.068  0.135  0  0.219  0.07  0.173  0  0.069  0  0  0  0  0  0  
 
 RBMS3-AS3  100873979  3  29305684  29322430  -  0  2.965  0  0  0  0.902  0.121  0.196  0.463  0.083  0.331  0  0  0  0  0  0  0  0  0  0  0.117  0  0  0  0  0  0.088  0.173  0.064  0.667  0.267  0  0  0.243  0  0  0.249  0.776  0  0  0.687  0  0  0.228  0.601  0  0  0  0.497  0  0.227  1.854  0.442  2.087  1.306  0.404  0.876  0  1.166  
 
 RBMS3  27303  3  29322802  30051886  3p24-p23  0  2.371  2.313  1.485  0  2.679  2.298  2.55  1.897  2.323  2.282  0.079  0  0.069  0.598  0  0  0  0  0.091  0  0.232  0.206  0  1.097  1.227  0.951  1.667  0.219  0.554  1.018  1.356  1.481  0.699  0.095  1.261  1.713  2.547  1.851  0  0.708  1.18  0.319  0  1.334  2.42  0.24  1.886  1.758  1.472  0.56  1.045  2.822  1.492  2.539  2.298  2.916  2.9  1.53  4.191  
 
 RBMS3-AS1  100873977  3  29968301  29975647  -  0  0  0  0  0  0  0  0  0  0  0  0  0  0  0  0  0  0  0  0  0  0  0  0  0  0  0.096  0  0  0  0  0  0  0  0  0  0  0  0  0  0  0  0  0  0  0  0  0  0  0  0  0  0  0  0  0  0  0  0  0  
 
 TGFBR2  7048  3  30647993  30735633  3p22  0.192  3.743  2.382  1.761  0.021  1.504  2.952  2.467  3.227  4.31  3.479  2.402  4.9  3.276  1.836  2.598  3.441  3.595  2.423  2.387  0.222  1.826  0.934  0.395  5.371  3.426  2.716  2.845  2.392  1.53  1.938  3.177  2.331  1.246  2.554  3.595  4.311  4.003  3.045  0.466  3.824  1.321  0.793  3.807  2.648  3.594  3.093  1.996  2.836  2.662  3.615  2.393  3.822  2.483  2.945  3.344  3.363  2.401  2.397  4.077  
 
 GADL1  339896  3  30767691  30936153  3p24.1-p23  0  0  0  0  0  0  0  0  0.032  0.016  0.031  0  0  0  0  0  0  0  0.021  0  0  0.036  0.109  0  0  0.026  0.06  0  0  0.049  0.07  0  0  0.139  0  0  0  0  0  0  0  0.103  0.024  0.038  0.068  0.085  0.018  0  0  0.02  0  0.058  0.019  0.029  0  0  0.029  0  0  0  
 
 STT3B  201595  3  31573992  31679114  3p23  2.853  3.92  2.715  3.102  2.964  2.227  2.64  2.925  3.609  3.149  3.157  2.842  3.677  3.96  3.061  2.832  3.633  3.548  5.301  3.437  2.693  3.268  3.389  2.707  3.125  3.535  1.791  3.025  2.842  2.212  3.322  2.942  2.454  1.791  2.168  3.429  3.892  2.606  3.034  2.967  2.923  2.649  2.706  2.773  2.442  2.953  1.759  2.477  2.339  4.021  3.424  1.995  3.088  1.687  2.515  2.473  2.809  3.151  2.873  3.79  
 
 OSBPL10  114884  3  31702316  32023342  3p22.3  2.205  2.623  0.852  0.373  1.381  1.025  1.68  1.678  3.333  1.71  3.252  2.068  2.499  2.154  2.175  1.666  3.126  2.495  0.044  0.073  0.078  0.247  0.617  0.31  1.122  2.789  0.96  2.895  0.428  3.173  0.209  3.506  2.367  1.524  0.721  1.348  3.491  2.515  1.026  0.925  1.726  1.368  5.019  1.654  3.022  2.977  1.91  1.303  2.46  1.965  2.515  1.462  2.601  1.343  2.164  1.657  1.848  0.167  2.947  2.742  
 
 OSBPL10-AS1  100874206  3  31745704  31763078  -  0  0  0  0  0  0  0  0  0  0  0  0  0  0  0  0  0  0  0  0  0  0  0  0  0  0  0  0.132  0  0  0  0  0  0  0  0  0  0  0  0  0  0  0  0  0  0  0  0  0  0  0  0  0  0  0  0  0  0  0  0  
 
 ZNF860  344787  3  32023265  32033228  3p23  0.788  0.656  0.118  0  0.977  0.037  0.039  0.065  0.24  0.108  0.605  0.632  0.732  0.63  0.415  0.776  0.626  0.964  0.024  0  0  0.071  0.212  0.197  0.113  0.767  0.066  0.245  0  0.882  0  1.034  0.079  0.23  0.052  0.283  0.698  0.686  0.161  0.201  0.596  0.148  3.449  0.261  0.956  0.931  1.007  0.139  0.285  0.158  0.754  0.567  0.892  0.389  0.761  0.257  0.616  0  0.898  0.759  
 
 GPD1L  23171  3  32148002  32210207  3p22.3  2.258  2.148  0.696  1.039  2.289  1.029  1.553  0.744  2.46  1.383  1.517  0.853  1.913  1.602  1.799  0.66  1.925  1.884  0  2.927  0.798  1.243  1.939  1.258  0.892  1.523  1.396  1.641  1.153  2.046  1.286  1.48  1.769  1.332  0.877  1.778  1.026  1.183  0.954  0.709  1.008  1.072  2.132  0.984  1.192  1.807  1.375  0.746  0.669  1.086  2.498  0.859  0.951  0.39  1.025  0.628  0.404  1.007  0.858  1.346  
 
 CMTM8  152189  3  32280170  32411813  3p22.3  2.545  1.935  0.48  1.008  2.986  0.133  0.707  1.249  1.562  1.382  2.927  2.139  3.377  4.129  2.279  2.81  3.179  4.388  1.082  1.644  0  1.909  2.122  1.807  0  1.406  1.309  1.961  2.131  2.034  2.388  3.683  1.439  1.113  1.654  2.283  2.259  1.435  2.471  1.924  3.861  1.736  3.622  2.548  2.314  1.676  3.45  1.51  0.731  2.618  1.167  1.662  0.762  0.903  1.829  1.429  0.322  2.434  2.19  2.271  
 
 CMTM7  112616  3  32433162  32496333  3p22.3  0.51  5.182  2.537  1.458  3.603  2.734  3.031  2.375  3.712  3.13  4.931  2.389  3.153  5.81  3.33  4.76  4.814  3.932  4.476  5.026  1.012  0.419  3.851  4.352  0  3.061  4.804  4.929  4.617  3.42  3.824  4.933  5.23  4.015  3.098  2.415  5.09  4.685  4.092  2.365  4.715  2.425  4.235  3.287  5.025  4.761  5.884  2.953  2.131  3.925  4.587  2.332  3.804  1.712  3.612  3.361  2.675  3.957  4.022  3.969  
 
 CMTM6  54918  3  32522803  32544403  3p22.3  3.135  5.208  3.115  2.558  3.327  2.461  4.433  3.003  4.312  3.436  3.639  3.141  4.053  4.322  2.683  2.984  3.694  3.937  5.023  3.858  1.976  3.11  3.293  1.659  3.359  3.408  3.311  3.051  2.791  2.552  3.475  3.479  3.497  3.236  2.187  3.351  4.212  2.921  4.283  2.862  3.961  2.421  4.035  2.314  3.911  3.593  3.211  2.194  2.283  3.7  3.949  1.789  2.965  1.641  2.875  2.571  2.665  3.248  3.324  4.133  
 
 DYNC1LI1  51143  3  32567462  32612366  3p22.3  2.358  3.702  2.623  2.5  2.894  2.429  2.781  2.248  2.455  2.098  2.998  1.206  2.319  3.378  1.679  1.599  2.172  2.605  4.218  2.659  3.071  2.804  2.067  1.562  2.758  2.129  1.974  2.021  1.997  1.798  2.578  2.627  2.04  1.609  2.299  2.608  2.896  2.197  3.083  2.176  2.293  2.458  3.221  2.335  2.405  2.456  1.36  2.328  1.814  3.877  2.7  1.567  2.309  1.291  2.094  1.612  2.083  2.519  2.334  2.998  
 
 CNOT10  25904  3  32726636  32815367  3p22.3  2.221  2.459  1.085  1.818  2.724  1.504  1.896  1.827  1.731  1.867  2.352  1.36  2.735  2.95  2.148  1.856  2.378  2.095  3.912  2.543  3.025  2.685  1.788  1.487  2.031  1.833  1.514  2.246  1.702  1.417  2.066  2.179  1.943  1.036  1.675  1.642  2.472  1.777  2.147  2.222  1.902  1.273  2.877  1.725  1.757  2.226  1.793  1.918  1.542  3.506  2.152  1.123  1.611  0.781  1.774  1.186  1.668  2.209  2.272  2.525  
 
 TRIM71  131405  3  32859509  32933771  3p22.3  0.067  0.08  0.19  0.12  0.159  0.216  0.104  0.106  0.19  0.135  0.13  0.121  0.095  0.143  0.085  0.169  0.161  0.441  0.159  0.477  0.093  0.867  0.4  0.217  0.077  0.06  0.193  0.049  0.091  0.223  0.068  0.064  0.104  0.261  0.189  0.072  0.094  0  0.229  0.085  0.09  0.618  0.514  0.098  0.211  0.16  0.406  0.495  0  0.29  0.118  1.075  0.12  0.259  0.3  0.101  0.094  0.055  0  0  
 
 CCR4  1233  3  32993065  32996403  3p24  0  0  0.163  0  0  0  0  0  0.141  0  0.056  0  0  0.025  0  0  0  0  1.606  0  0  0.254  0.401  0  0  0  0.054  0.049  0  0.031  0  0  0  0.146  0  0  0  0  0  0  0  0.188  0.03  0  0.192  0.043  0.031  0  0  0  0  0.271  0  0  0  0  0  0  0  0  
 
 GLB1  2720  3  33038099  33138694  3p21.33  3.738  3.902  2.82  4.023  4.48  2.554  4.801  3.839  4.063  4.028  3.334  2.499  3.219  3.128  3.615  3.049  4.598  3.009  4.219  5.005  2.869  3.258  3.532  2.99  3.089  4.021  3.981  3.437  3.98  3.33  3.643  3.937  3.536  2.322  2.531  4.106  4.242  4.414  4.691  3.169  3.415  2.777  3.94  2.947  2.916  3.876  4.59  3.372  2.066  4.653  2.984  2.065  3.47  2.689  3.985  3.312  3.287  3.235  3.95  4.634  
 
 TMPPE  643853  3  33131907  33138293  3p22.3  0.967  0.526  0.74  0.427  0.561  0.485  0.519  0.734  0.731  0.704  0.778  0.309  0.91  0.776  0.819  0.385  0.368  0.09  0.474  0.828  1.084  0.665  0.604  0.481  0.828  1.036  0.613  1.468  0.995  0.691  0.884  0.76  1.095  0.387  0.41  1.182  1.069  1.161  0.616  1.2  0.754  0.423  0.625  1.177  0.758  1.061  0.355  0.704  0.782  0.132  1.073  0.721  0.898  0.363  0.753  0.631  0.899  0.849  1.019  1.992  
 
 CRTAP  10491  3  33155449  33189265  3p22.3  3.09  3.407  3.48  3.07  3.448  2.506  4.668  3.965  4.769  4.207  3.8  1.369  2.911  2.799  2.597  1.505  2.296  1.09  2.552  2.74  1.709  1.547  2.41  1.907  3.243  4.76  4.548  4.397  4.282  3.84  4.448  4.643  4.309  3.595  3.191  3.203  3.742  4.24  4.318  2.737  1.312  3.519  3.729  2.259  2.185  2.554  2.4  2.969  2.61  3.978  4.069  2.47  3.611  2.92  3.946  3.541  3.533  3.898  3.31  3.958  
 
 SUSD5  26032  3  33191536  33260707  3p22.3  0.123  0.136  0.992  1.118  0.295  0.131  0.109  2.175  0.379  0.409  0.28  0  0.03  0.041  0  0  0.035  0.082  0.024  0.03  0  0.131  0.391  0  0.03  0.079  0.494  0.549  1.186  0.082  0.334  0.967  0.055  0.226  0.113  0.033  0.525  0.143  0.399  1.618  1.81  0.527  0.418  0.106  0.48  0.66  0.022  0.468  0.545  0.324  0.049  0.467  0.017  0.114  0.09  0.22  0.03  0.819  0.024  0.684  
 
 FBXL2  25827  3  33318933  33428757  3p22.3  0.576  1.352  0.663  0.584  1.132  0.481  0.668  0.504  1.111  0.703  0.959  0  0.409  1.528  0.703  0.122  0.246  1.585  0.098  0.148  0.155  0.367  0.21  0.119  0.66  0.68  0.719  0.547  0.698  0.308  0.559  0.445  0.577  0.404  0.622  0.691  1.029  1.037  1.364  0.507  0.856  0.817  1.518  1.032  0.725  0.646  0.87  1.022  1.129  1.823  0.969  0.541  0.786  0.34  1.476  1.054  0.727  0.912  0.942  1.498  
 
 UBP1  7342  3  33429828  33481897  3p22.3  3.205  3.269  2.477  2.726  2.71  1.851  3.309  2.556  3.059  2.441  3.261  1.572  3.229  3.461  2.482  2.499  2.694  2.857  3.924  2.965  2.828  2.751  2.378  2.21  2.938  2.693  2.422  3.149  2.697  2.821  2.677  2.865  2.852  1.795  1.965  2.397  3.033  2.808  3.56  2.958  2.314  2.294  3.428  2.583  2.543  2.566  1.998  2.453  2.023  3.633  3.394  1.809  2.759  1.504  2.199  1.987  2.27  3.141  2.842  3.498  
 
 CLASP2  23122  3  33537738  33759705  3p22.3  1.432  1.887  0.857  0.83  1.281  0.872  1.298  1.159  1.316  1.023  1.491  0.631  1.222  1.402  1.365  0.77  1.367  1.507  2.763  1.645  1.715  1.596  0.828  0.625  1.347  1.642  1.13  1.177  1.393  1.494  1.602  1.364  1.398  0.794  0.947  1.585  1.807  1.191  1.465  1.667  1.149  0.895  1.426  1.476  1.15  1.298  0.773  1.145  1.044  1.895  1.723  0.764  1.143  0.508  1.355  0.725  0.943  1.006  1.136  1.965  
 
 PDCD6IP  10015  3  33840062  33911199  3p22.3  3.331  4.352  2.71  2.6  4.098  2.52  3.919  3.281  2.601  3.076  3.439  2.348  3.742  4.213  3.154  2.562  3.538  3.979  4.089  3.194  3.202  2.837  2.007  1.978  3.01  3.372  3.039  3.186  2.951  2.981  3.592  3.307  3.122  1.957  2.581  3.381  3.289  3.7  3.766  3.128  3.092  2.202  4.1  2.635  2.354  3.089  3.098  3.047  2.423  4.294  3.978  1.865  3.037  1.999  3.011  2.429  2.915  3.316  3.156  4.119  
 
 LOC101928135  101928135  3  34917288  35435515  -  0  0  0  0  0  0  0  0  0  0.055  0  0  0  0  0  0  0  0  0  0  0  0  0  0  0  0  0.046  0  0  0.051  0  0  0  0  0  0  0  0  0  0  0  0  0  0  0  0  0.051  0  0  0  0  0  0  0  0  0  0  0  0  0  
 
 ARPP21  10777  3  35680665  35835988  3p22.3  0  0.059  0.216  0  0.05  0  0.239  0.053  0.238  0.277  0.066  0.068  0  0.045  0  0  0  0.113  4.656  0.069  0  4.146  0.462  0  0  0.975  0.204  0.041  0  0.115  0  1.623  0  0.406  0.068  0  0.196  0  0.049  0  0  0.509  0.047  0  0  0  0.143  0  0  0.077  0.545  0.334  0.081  0.108  0.068  0  0.203  0  0  0  
 
 STAC  6769  3  36421978  36589498  3p22.3  0  2.079  2.63  2.247  0.042  0.958  2.162  2.089  1.718  2.071  2.808  0.033  0  0.039  0  0  0  0.1  0.387  0  0  1.245  0.232  0  0  0  0.068  1.105  0.051  0.079  0.379  0.078  0.77  0.414  0.255  0.498  2.904  3.068  1.628  1.171  0.879  1.268  0.021  0  0.18  0.338  0.069  0.648  1.639  0.493  0.234  0.168  3.745  1.053  2.133  1.863  3.2  2.926  3.224  2.43  
 
 DCLK3  85443  3  36753912  36781352  3p22.2  0  0.025  0.122  0  0.014  0  0  0.012  0.127  0.023  0.017  0  0  0.02  0  0  0  0.063  0  0  0  0.145  0.262  0  0  0  0.107  0  0.018  0.049  0  0  0  0.19  0.025  0  0  0  0.023  0.04  0.04  0.279  0.037  0  0.04  0.021  0.014  0  0  0  0  0.194  0  0.04  0.022  0  0  0  0  0  
 
 TRANK1  9881  3  36868307  36986548  3p22.2  0.082  0.107  0.187  0.778  0.297  0.028  1.028  0.436  0.362  0.658  0.591  0.303  0.373  0.185  0.586  1.255  0.14  0.075  0.202  0.495  0.187  1.026  0.665  1.016  0.027  0.163  0.703  0.58  0.354  0.733  0.274  0.089  0.574  0.404  0.126  0.634  0.195  0.359  0.748  0.502  0.054  0.442  0.136  0.093  0.11  0.196  0.098  0.09  0.842  0.264  0.209  0.459  0.368  0.805  0.59  0.268  0.796  0.388  0.369  1.188  
 
 EPM2AIP1  9852  3  37027356  37034795  3p22.1  2.244  2.233  0.778  0.197  2.896  0.759  1.623  1.215  1.425  1.662  1.385  0.437  1.573  1.816  1.712  0.667  0  2.181  2.82  1.585  1.706  1.717  1.021  0.733  1.043  1.522  1.296  1.488  0.823  1.048  1.499  1.393  1.201  0.708  1.045  1.479  1.151  1.502  1.853  1.017  1.166  1.276  1.753  1.466  0.701  1.015  1.388  1.202  1.182  1.547  1.228  0.578  1.151  0.394  0.983  0.682  1.155  1.241  1.189  1.601  
 
 MLH1  4292  3  37034840  37092337  3p21.3  3.082  3.985  1.887  1.771  3.965  2.044  3.853  2.747  2.085  2.759  3.247  2.075  2.727  1.813  2.865  2.187  0  3.498  4.001  3.973  3.383  3.355  1.534  2.008  2.555  3.077  3.071  2.492  2.761  2.873  3.532  3.475  2.827  1.422  2.528  2.596  3.161  2.982  2.887  3.212  2.913  1.933  3.223  0.887  1.53  2.097  3.308  2.736  0.113  4.497  3.109  0.935  3.124  1.544  3.114  2.21  2.719  2.876  3.147  4.008  
 
 LRRFIP2  9209  3  37094116  37217851  3p22.2  1.599  3.644  2.106  2.136  2.372  2.619  2.794  2.26  1.989  2.818  3.179  0.946  1.91  2.369  1.507  1.354  2.092  1.985  3.302  1.673  1.735  1.822  1.643  1.084  2.182  2.326  2.676  2.259  2.043  1.735  2.595  2.666  2.49  1.556  1.831  1.981  2.34  2.138  3.477  1.921  2.293  2.05  2.039  2.232  1.817  2.249  1.638  2.257  0.283  3.389  2.92  1.267  2.296  1.255  3.355  1.468  2.428  1.638  3.183  2.912  
 
 GOLGA4  2803  3  37284681  37408370  3p22-p21.3  2.558  4.297  2.07  1.94  3.788  2.24  2.71  2.586  2.449  2.223  2.765  1.033  2.792  3.274  2.418  1.954  2.427  3.528  2.153  1.553  1.803  2.012  1.655  1.072  1.996  3.051  1.775  2.846  2.352  1.921  2.938  2.483  2.103  1.816  1.88  2.415  2.499  2.159  3.195  2.032  2.549  1.935  1.97  2.382  1.892  2.388  1.443  2.048  2.129  2.638  3.323  1.354  2.109  1.509  2.33  2.062  2.3  1.996  2.201  3.512  
 
 C3orf35  339883  3  37427759  37476988  3p22.2  0.392  0.402  0.321  0  0.765  0  0.302  0.288  0.427  0.198  0.31  0  0.258  1.29  0.628  0.393  0.347  0.205  0.996  0.623  0  0.477  0.507  0.227  0  0.379  0.424  0.45  0.35  0.163  0.805  0.397  0.178  0.437  0.521  0.372  0.349  0.586  0.671  0  0.302  0.569  0.473  0.42  0  0.205  1.752  0  0  0.566  0.411  0.234  0.207  0.129  0.339  0.259  0.387  0  0.492  0  
 
 ITGA9  3680  3  37493812  37861281  3p21.3  0.081  0.031  0.083  0  0.08  0.04  0.063  0.13  0.382  0.792  0.06  0.06  0  0.11  0.207  0.057  0.053  0.074  0.054  0.063  0  0.173  0.094  0  0  1.313  2.352  0.121  1.698  1.44  1.757  1.853  2.215  0.721  0.064  0.067  0.038  0.062  0.114  0.088  0.069  0.349  0.06  0.079  0.357  0.303  0.102  0.097  0.069  0.072  0.036  0.437  0.053  0.051  0.083  0.064  0.049  0.042  0.033  0  
 
 ITGA9-AS1  101928153  3  37795179  37903271  3p22.2  0  0  0  0  0.333  0  0.638  0  0  0.224  0.169  0.244  0  0  0.345  0.319  0  0.313  0.614  0  0  0.206  0  0  0  0.459  0.846  0.249  0.562  0.238  0.455  0.281  0.312  0  0.396  0  0.313  0.381  0.24  0  0.37  0  0.164  0.368  0  0.227  1.871  0  0  0  0  0  0  0  0.3  0  0.382  0.763  0  0  
 
 CTDSPL  10217  3  37903668  38025960  3p21.3  2.699  3.58  1.798  1.906  3.688  1.459  2.907  1.672  3.098  3.092  2.118  2.546  2.409  2.953  3.443  3.636  2.978  3.438  2.989  0.064  1.85  2.3  0.362  0  0.758  3.212  3.679  3.302  3.021  2.458  2.676  2.211  2.964  1.818  1.611  2.624  3.237  2.633  2.659  2.032  3.486  1.725  2.962  3.039  1.501  2.876  3.408  2.697  2.077  2.905  0.199  1.256  2.05  1.222  2.559  2.414  2.355  2.598  3.05  3.411  
 
 VILL  50853  3  38035077  38048676  3p21.3  0  0.04  0.252  0.097  0.158  0.265  0.045  0.063  0.13  0.049  0.035  2.024  1.838  0.089  0.277  0.155  2.16  0.125  2.786  0.751  1.033  2.133  0.407  0.754  0.639  1.048  0.351  0.963  0.595  0.781  0.82  0.384  0.067  0  0  0.219  0.518  0  0.579  0.407  3.096  0.43  0.16  0.288  0.158  0.126  0.037  0.957  0.112  1.427  0.033  0.828  0.099  0.342  0.814  0.358  0.452  0.116  0.211  0.384  
 
 PLCD1  5333  3  38048986  38071154  3p22-p21.3  0.715  0.052  0.528  2.401  0.954  0.336  0.983  0.409  1.685  1.539  1.049  0.512  0.516  1.098  1.073  0.734  1.227  0.28  1.094  0.25  3.298  0.946  0.685  1.061  0.6  1.702  1.078  1.205  0.972  1.159  1.041  1.171  1.06  0.615  0.668  0.787  1.169  0.519  0.242  0.972  0.376  0.711  0.739  0.426  0.612  0.654  0.764  0.721  0.098  1.388  0.973  1.135  0.521  0.328  1.303  0.397  0.669  1.461  0.481  1.18  
 
 DLEC1  9940  3  38080695  38164228  3p21.3  0.106  0.022  0.084  0.078  0.152  0.028  0.044  0.049  0.15  0.082  0.115  0.03  0.154  0.14  0.183  0.133  0.172  0.088  0.115  0.076  0.042  0.165  0.176  0.121  0.034  0.124  0.268  0.142  0.247  0.075  0.056  0.072  0.069  0.109  0.083  0.055  0.071  0.047  0.121  0.063  0.071  0.259  0.048  0.029  0  0.1  0.034  0.046  0  0.056  0.056  0.344  0.076  0.097  0.119  0.095  0.059  0.048  0.053  0  
 
 ACAA1  30  3  38164200  38178733  3p22.2  3.711  2.634  2.646  2.646  3.569  2.348  4.317  2.387  3.683  3.508  3.245  3.265  3.875  3.961  3.775  4.01  3.984  1.834  5.598  4.408  2.723  4.29  2.229  2.65  2.716  3.486  4.269  3.139  3.822  3.669  3.948  4.351  3.24  1.804  2.819  2.985  3.568  3.961  3.477  2.832  2.965  3.113  3.52  2.651  2.76  3.692  4.28  2.899  2.22  4.025  3.584  1.952  3.259  1.828  2.97  1.809  2.442  2.537  2.998  3.518  
 
 MYD88  4615  3  38179968  38184512  3p22  3.555  2.87  2.36  3.942  3.823  2.075  4.703  2.58  3.212  3.146  3.97  2.741  3.873  3.277  3.757  4.48  3.548  1.978  5.498  3.659  2.999  4.758  1.858  3.101  2.066  3.724  3.614  3.634  3.225  2.085  2.894  2.162  3.457  2.308  2.457  2.724  3.389  3.413  4.026  2.482  2.529  2.399  4.892  0.706  2.399  2.853  3.243  2.456  0.979  3.065  3.782  2.069  2.807  2.881  3.033  2.201  3.573  2.273  3.245  4.018  
 
 OXSR1  9943  3  38207025  38296979  3p22.2  2.727  3.848  2.828  3.117  2.522  2.52  3.275  2.755  2.347  2.671  3.221  1.425  2.064  2.67  2.408  2.13  2.912  2.541  4.594  2.676  2.806  3.053  1.811  2.056  2.63  2.471  2.787  2.949  2.562  1.932  2.408  2.3  2.308  1.56  1.732  2.237  3.071  2.586  3.291  2.361  2.257  1.79  2.546  2.367  1.681  2.348  2.76  2.813  1.696  4.041  2.861  1.587  2.446  0.886  2.431  1.787  2.302  2.432  2.547  3.235  
 
 SLC22A13  9390  3  38307297  38319806  3p21.3  0.133  0.045  0.225  0  0.09  0.133  0.102  0.09  0.236  0.178  0.187  0.07  0.122  0.239  0.26  0.062  0  0.098  0.126  0.063  0.104  0.208  0.311  0.069  0.044  0.067  0.216  0.289  0.111  0.094  0.08  0.113  0.142  0.166  0.069  0.169  0.085  0.237  0.221  0  0.264  0.242  0.125  0  0  0.196  0.022  0.625  0.108  0.15  0.237  0.551  0  0.083  0.074  0.062  0.16  0  0.118  0.055  
 
 SLC22A14  9389  3  38347444  38359859  3p21.3  0  0  0.198  0  0.048  0  0.115  0  0.168  0.043  0.127  0  0  0.093  0  0  0  0  0.043  0  0  0.117  0.277  0  0  0  0.133  0.242  0  0.053  0  0.1  0  0.171  0  0.097  0.074  0.166  0.154  0  0.055  0  0.043  0.06  0  0.1  0  0.148  0  0.291  0.107  0.444  0  0.054  0  0  0  0  0  0  
 
 XYLB  9942  3  38388250  38456467  3p22-p21.3  0.895  0.63  0.514  0.242  1.61  0.474  0.835  0.887  0.253  0.706  0.393  0.623  1.49  1.278  1.345  0.496  1.109  0.323  1.406  1.606  1.938  1.98  0.468  0.963  0.743  2.678  1.83  1.775  1.733  1.728  2.243  1.86  2.344  0.737  1.169  0.947  0.999  1.657  1.34  1.358  1.37  0.649  1.647  0.992  0.366  0.797  0.785  1.275  0.646  1.348  1.51  0.706  0.823  0.483  0.667  0.53  0.739  1.274  1.219  0.922  
 
 ACVR2B-AS1  100128640  3  38492517  38496311  3p22.2  0.231  0.215  0.22  0.139  0.283  0.172  0.087  0.092  0.243  0.121  0.085  0.12  0.324  0.127  0.236  0.111  0.242  0.06  0.594  0.124  0.132  0.693  0.284  0.073  0.066  0.742  0.216  0.807  0.382  0.346  0.233  0.118  0.287  0.374  0.315  0.097  0.259  0.242  0.133  0.151  0.22  0.215  0.611  0.163  0.101  0.333  0.019  0.104  0.126  0.555  0.146  0.121  0.053  0.177  0.076  0.122  0.08  0.112  0.123  0.334  
 
 ACVR2B  93  3  38495789  38534633  3p22  1.243  0.704  0.289  0.305  0.483  0.356  0.351  0.3  1.129  0.343  0.43  0.338  0.564  1.346  1.324  0.377  0.473  0.989  1.473  0.804  1.327  1.526  0.695  0.474  0.825  1.317  0.797  1.15  0.433  1.012  1.005  0.673  0.638  0.323  0.403  0.456  0.931  0.718  0.485  0.844  0.884  0.475  1.211  0.876  0.463  0.541  0.498  0.651  0.194  0.888  0.339  0.372  0.555  0.162  0.474  0.464  0.368  0.548  0.693  0.741  
 
 EXOG  9941  3  38537762  38567796  3p21.3  0.56  1.814  0.559  0.731  0.676  1.051  1.577  1.059  1.083  1.582  1.384  0.453  1.273  1.077  1.256  0.834  0.904  1.783  2.943  1.289  1.203  1.943  0.878  0.717  0.896  1.504  0.941  1.096  0.946  0.337  1.007  1.007  1.003  0.594  0.747  1.129  1.34  1.053  1.286  0.798  0.79  1.212  0.935  0.749  0.79  1.326  1.519  0.913  0.475  2.082  1.303  0.631  1.159  0.433  1.412  0.694  0.784  0.909  1.174  1.532  
 
 SCN5A  6331  3  38589552  38691164  3p21  0.092  0.877  0.106  0  0.034  0.143  0.041  0.042  0.115  0.044  0.021  0.026  0.145  0.891  0.029  0.04  0.042  0.61  0.029  0.053  0  0.181  0.413  0.029  0  0.021  0.102  0.471  0.018  0.49  0.032  0.03  0  0.149  0.117  0.027  0.678  0.044  0.117  0.181  0.026  0.42  0.122  0.05  0.039  0.136  0.417  0.558  0  0.593  0.028  0.481  0.017  0.069  0.097  0.032  0.021  1.608  0.682  2.502  
 
 SCN10A  6336  3  38738836  38835501  3p22.2  0  0.039  0.098  0  0  0.025  0.026  0.024  0.105  0.029  0.014  0.019  0  0.056  0  0  0.032  0.058  0  0.087  0  0.051  0.298  0  0  0  0.054  0.023  0  0.061  0  0  0  0.163  0.03  0  0.032  0  0.034  0  0  0.098  0.029  0  0.05  0.038  0.012  0  0  0  0.02  0.236  0.033  0.045  0.016  0  0.019  0  0.03  0.069  
 
 SCN11A  11280  3  38887259  38995142  3p22.2  0  0.022  0.109  0  0  0  0  0.01  0.066  0.015  0.011  0.032  0  0.017  0  0.013  0  0.031  0  0  0  0.063  0.225  0  0  0.014  0.047  0.022  0  0.044  0  0  0  0.131  0.026  0  0  0  0.024  0  0  0.071  0.054  0  0  0.035  0.016  0  0  0.027  0.021  0.133  0  0.02  0.032  0  0  0  0  0  
 
 WDR48  57599  3  39093476  39138161  3p21.33  2.23  2.624  1.843  2.21  2.102  0.956  2.432  1.915  2.964  1.974  1.739  1.089  1.833  2.974  1.946  1.595  2.386  2.613  3.38  2.726  1.964  2.561  2.001  1.448  1.936  2.124  2.135  2.351  1.961  1.676  1.768  1.846  1.878  1.428  1.56  1.588  1.983  1.892  2.476  1.847  1.11  1.134  2.308  1.517  1.7  1.539  1.622  2.069  1.19  3.065  2.177  1.142  1.88  1.05  2.151  1.627  1.829  1.5  2.138  2.533  
 
 GORASP1  64689  3  39138089  39149854  3p22-p21.33  2.608  1.209  2.025  2.244  3.215  1.69  3.25  2.418  2.459  2.748  2.396  2.194  3.004  2.126  2.334  2.083  2.169  0.945  1.718  2.073  2.701  1.184  1.999  2.417  2.463  2.477  3.171  3.185  2.624  2.886  3.077  2.871  2.855  1.649  1.544  2.29  2.648  2.266  2.703  2.116  2.375  1.815  3.188  2.038  1.817  2.624  1.895  2.747  1.914  3.565  2.908  1.315  2.643  1.443  2.149  2.083  2.158  2.154  2.493  3.058  
 
 TTC21A  199223  3  39149151  39180394  3p22.2  0.241  0.052  0.175  0.329  0.504  0.11  0.368  0.2  0.217  0.305  0.165  0.143  0.165  0.279  0.164  0.154  0.093  0.103  0.068  0.069  0.094  0.116  0.321  0.445  0.078  0.29  0.457  0.548  0.267  0.134  0.28  0.325  0.136  0.129  0.176  0.159  0.22  0.332  0.124  0.216  0.441  0.2  0.451  0.11  0.227  0.236  0.416  0.255  0.094  0.546  0.197  0.462  0.143  0.093  0.228  0.199  0.274  0.308  0.189  0.208  
 
 CSRNP1  64651  3  39183341  39195102  3p22  1.117  0.304  1.382  2.456  0.994  1.565  1.744  2.394  1.367  1.168  1.712  0.536  0.889  1.249  1.369  1.283  1.605  0.178  0.989  0.483  2.188  0.285  1.305  2.028  2.596  1.141  0.762  1.934  0.54  1.056  0.483  0.712  0.677  0.413  1.102  2.029  2.054  1.161  1.517  2.297  2.054  1.093  0.731  0.645  1.147  1.848  0.501  1.631  0.95  2.564  1.528  1.004  1.022  1.302  1.204  0.965  1.875  1.632  1.146  2.104  
 
 XIRP1  165904  3  39224705  39234087  3p22.2  0.032  0  0.18  0  0.029  0.022  0.038  0.666  0.246  0.03  0.019  0.052  0  0.031  0  0.046  0.059  0.062  0.025  0.059  0.093  0.107  0.499  0.061  0  0.031  0.166  0.092  0.043  0.088  0  0.033  0.021  0.2  0.034  0.042  0  0.046  0.058  0.042  0  0.538  0.13  0.043  0  0.06  0.063  0.039  0.036  0.041  0.018  0.378  0.059  0.079  0.036  0.081  0.056  0.281  0  0.025  
 
 CX3CR1  1524  3  39304984  39323226  3p21.3  0.081  0.038  0.098  0  0.045  0  0  0.026  0.195  0.042  0.05  0  0  0.065  0  0  0  0.064  0.059  0.128  0  0.085  4.294  6.068  0  0  0.08  0  0  0.08  0  0  0  0.177  0  0  0  0  0.048  0  0  0.071  0.13  0  0  0  0.03  0  0  0.037  0  0.294  0  0.052  0  0  0  0  0  0  
 
 CCR8  1237  3  39371196  39375171  3p22  0  0.092  0  0  0.052  0  0  0  0.081  0  0  0  0  0  0  0  0  0  0.208  0  0  0.094  0.318  0  0  0  0.05  0  0  0.077  0  0  0  0.141  0  0  0  0  0.051  0  0  0.314  0.033  0  0  0  0.047  0  0  0  0  0.118  0  0.061  0  0  0  0  0  0  
 
 SLC25A38  54977  3  39424814  39438819  3p22.1  3.312  3.13  2.075  2.146  3.02  1.888  3.745  2.4  3.742  2.439  3.196  1.561  2.752  3.303  2.505  2.037  2.662  2.558  4.742  2.993  2.573  3.516  2.901  2.325  2.325  2.154  2.349  3.079  2.505  1.588  2.573  3.245  2.577  1.532  2.118  1.78  2.647  2.8  3.662  2.3  2.24  2.336  3.378  1.815  2.207  2.349  2.093  2.108  1.629  3.541  2.933  1.334  2.43  1.622  2.133  1.712  1.925  2.506  2.078  2.68  
 
 RPSA  3921  3  39448179  39454032  3p22.2  8.975  7.726  7.438  6.825  8.748  7.72  9.924  7.792  8.106  7.985  8.817  7.645  8.107  10.343  8.095  7.468  9.207  6.295  10.488  9.941  8.637  8.957  7.866  8.171  8.593  7.248  8.114  7.407  7.488  8.308  8.356  8.854  7.792  5.479  7.79  7.562  8.9  8.442  8.858  8.275  7.847  8.158  9.029  7.97  7.681  7.67  8.706  8.664  6.94  10.724  8.286  7.032  8.15  5.965  8.714  7.682  7.366  9.423  8.75  9.214  
 
 SNORA6  574040  3  39449881  39450030  3p22.2  3.271  2.539  0  0  1.516  2.545  2.722  0  0  1.245  2.373  0  2.361  3.571  3.283  0  1.821  0  3.762  2.909  0  2.281  0  2.727  0  0  1.879  2.783  1.752  1.427  2.562  0  3.018  0  2.383  0  1.916  0  0.927  0  0  0  0  0  0  0  1.235  1.97  0  3.003  2.261  0  1.704  0  1.261  0  2.337  3.461  2.263  0  
 
 SNORA62  6044  3  39452544  39452698  3p22.1  1.99  0  0  0  0  2.19  0  0  0  1.425  2.014  0  2.017  1.54  2.522  1.715  1.881  0  3.506  2.477  0  1.667  0  0  0  0  1.455  2.16  1.462  0.978  0  2.744  0  0  1.999  0  2.449  2.178  0.905  0  0  6.004  0  0  0  2.159  1.351  2.511  0  3.289  0  2.312  1.683  0  1.759  0  0  2.71  0  0  
 
 MOBP  4336  3  39509063  39570988  3p22.1  0  0  0.267  0  0  0  0  0  0.267  0  0  0  0  0.067  0  0  0.151  0.144  0  0  0  0.232  0.587  0  0  0  0.11  0  0  0.141  0  0  0  0.275  0  0  0  0  0.146  0  0  0.184  0.095  0  0  0.131  0.284  0  0  0  0  0.513  0.056  0.069  0  0  0  0  0  0  
 
 MYRIP  25924  3  39850404  40301811  3p22.1  0.925  0  0.133  0  0.44  0.064  0  0.022  0.171  0.316  0.028  0.031  0.73  0.108  0.432  0  1.002  0.533  0  0.066  0  0.102  0.245  0  0  0.047  0.086  0.897  0.041  0.077  0.032  0.035  0  0.17  0  0.033  0.031  0.05  0.208  0  0.038  0.17  0.033  0  0  0.037  0.034  0.265  0  0  0.032  0.543  0.022  0.056  0.062  0.17  0  0  0  0  
 
 EIF1B-AS1  440952  3  40214637  40351189  3p22.1  0.253  0  0.235  0  0.069  0  0.044  0.074  0.264  0.082  0.076  0  0.079  0.168  0  0  0.085  0.142  0.104  0.085  0.303  0.232  0.355  0.162  0  0.077  0.092  0.139  0.049  0.091  0.103  0.053  0  0.234  0.054  0.124  0.045  0.101  0.073  0  0.145  0.493  0.039  0  0  0.102  0.06  0.112  0.075  0.165  0  0.225  0.146  0.108  0.132  0.146  0.045  0.066  0  0.068  
 
 EIF1B  10289  3  40351172  40353915  3p22.1  3.358  5.723  2.796  3.414  3.797  3.018  3.323  3.071  3.722  3.354  3.414  1.991  2.834  3.933  1.474  2.591  4.3  6.233  5.166  3.922  4.563  3.594  3.386  2.57  3.456  3.375  2.618  2.902  2.818  2.744  3.739  3.442  2.831  2.934  2.572  3.181  3.018  2.808  4.783  2.821  3.331  3.342  3.486  3.241  2.833  2.988  1.936  2.838  2.514  5.26  3.662  2.074  2.865  1.848  2.856  2.389  2.375  3.941  2.797  3.519  
 
 ENTPD3  956  3  40428646  40470117  3p21.3  0.139  0  0.083  0  0.675  0  0  0.077  0.103  0.048  0  0.106  0  0.033  0  0.044  0.11  0.071  0  0  0.147  0.092  0.232  0  0.118  0  0.091  0  0  0.093  0.084  0  0  0.123  0  0.062  0.11  0.81  0.122  0  0.317  0.216  0.06  0.09  0.451  0.077  0.306  0  0  0  0.184  0.315  0.127  0.051  0.057  0  0.073  0  0  0.523  
 
 ENTPD3-AS1  285266  3  40432441  40494799  3p21.33  0.325  0.065  0.226  0  0.086  0  0.099  0.048  0.169  0.098  0.043  0.146  0  0  0  0.095  0.095  0.055  0.117  0  0  0.069  0.377  0.081  0  0.048  0.034  0  0  0.047  0  0  0  0.189  0.075  0  0  0  0.142  0  0.136  0.055  0.027  0  0.081  0.08  0.094  0  0  0.076  0  0.176  0.133  0.032  0.114  0.099  0.057  0.074  0  0  
 
 RPL14  9045  3  40498782  40503863  3p22-p21.2  7.934  9.473  7.057  7.129  8.615  7.083  8.215  6.919  7.976  7.338  7.5  6.787  7.313  8.512  6.662  7.579  8.758  9.195  9.39  9.302  8.618  8.71  8.077  7.599  7.538  6.801  7.212  6.858  7.131  7.213  7.87  7.791  6.77  6.111  6.799  6.747  7.24  7.125  9.136  6.562  6.872  8.221  7.675  7.757  6.878  7.352  6.811  7.49  6.15  9.71  7.396  5.998  7.364  5.852  8.153  6.761  6.341  8.202  7.417  8.104  
 
 ZNF619  285267  3  40518603  40531728  3p22.1  1.183  0.51  0.394  0.442  0.695  0.385  0.771  0.42  0.41  0.445  0.611  0.408  0.984  1.015  0.672  0.395  0.616  0.3  0.057  0.567  0.838  0.082  0.55  0.538  0.502  0.564  0.5  0.724  0.622  0.48  0.596  0.455  0.429  0.243  0.297  0.366  0.596  0.762  0.649  0.339  0.257  0.166  0.027  0.472  0.48  0.361  0.149  0.503  0.259  0.585  0.677  0.458  0.451  0.223  0.47  0.366  0.502  0.733  0.681  1.141  
 
 ZNF620  253639  3  40547529  40559712  3p22.1  1.142  0.46  0.208  0.266  1.215  0.502  0.174  0.185  0.792  0.179  0.839  0.352  0.716  1.188  1.206  0.731  0.956  0.933  0.234  0.375  0.468  0.519  0.476  0.31  0.069  0.712  0.08  0.125  0.556  0.485  0.514  0.193  0.071  0.247  0.362  0.556  0.779  0.399  0.271  0.598  0.81  0.457  1.56  0.984  0.484  0.429  0.856  0.787  0.103  1.163  0.113  0.564  0.12  0.203  0.368  0.305  0.349  0.972  0.282  0.867  
 
 ZNF621  285268  3  40566368  40581285  3p22.1  1.28  2.798  1.003  1.273  1.801  1.449  1.51  1.268  1.515  1.398  1.653  0.496  0.994  1.775  1.25  1.291  1.291  3.176  2.257  1.793  1.389  1.535  1.324  0.535  0.792  1.595  1.506  1.465  1.083  0.865  1.766  1.587  1.116  0.896  0.596  1.114  1.218  1.172  2.191  0.969  1.055  1.294  2.27  0.931  0.965  1.249  2.186  1.313  0.859  2.68  1.638  1.081  1.233  0.642  1.383  0.836  1.392  1.79  1.523  1.436  
 
 CTNNB1  1499  3  41240941  41281939  3p21  4.316  2.708  3.399  3.754  4.067  3.448  3.655  4.598  4.903  4.614  4.865  4.165  5.021  5.477  5.008  3.306  5.137  2.694  3.895  2.795  3.565  2.848  0.933  2.281  4.244  5.788  4.314  5.611  5.303  5.972  6.285  5.3  4.833  2.722  2.888  4.658  5.32  4.193  4.566  4.431  4.364  2.806  3.484  4.743  4.554  4.183  1.755  4.343  4.019  4.766  4.55  3.079  4.841  2.977  4.016  3.442  4.255  4.285  4.857  5.61  
 
 ULK4  54986  3  41288089  42003660  3p22.1  0.356  0.374  0.175  0.167  0.396  0.308  0.738  0.265  0.184  0.355  0.41  0.315  0.302  0.529  0.408  0.175  0.303  0.456  0.667  0.511  0.16  0.503  0.217  0.109  0.186  0.499  0.364  1.088  0.604  0.335  0.211  0.357  0.35  0.198  0.129  0.482  0.172  0.348  0.341  0.411  0.191  0.454  0.829  0.468  0.777  0.927  0.187  0.288  0.501  0.348  0.53  0.313  0.38  0.218  1.197  0.346  0.557  0.481  0.688  0.536  
 
 TRAK1  22906  3  42132745  42267382  3p22.1  1.792  2.761  2.178  1.936  2.106  1.743  2.059  2.604  2.939  2.468  2.274  2.699  2.946  2.523  2.541  2.816  3.314  2.888  2.073  2.23  2.1  1.349  2.139  2.331  1.347  3.162  2.317  2.385  2.255  2.503  2.892  2.589  2.204  1.465  1.285  2.26  2.509  2.579  2.272  1.817  2.355  1.659  2.866  2.204  2.791  2.835  1.966  2.604  2.098  2.745  2.628  1.806  1.944  0.903  2.436  1.397  1.83  2.68  2.14  2.966  
 
 CCK  885  3  42299317  42307662  3p22.1  0  0  0  0  0.06  0.195  0.247  0.174  0.158  0.11  0.087  0.722  0  0.717  0  0.173  0  0  0  0.148  0  0.27  0.311  0  0  0.155  0.341  0  0  0.284  0  0  0  0.265  0.475  0.22  0  0  0.112  0.553  0  0.674  0.069  0  0.263  0.76  0.226  0.742  0  2.887  0.173  0.801  0.06  0.174  0.097  0.141  0  0  0  0  
 
 LYZL4  131375  3  42438569  42452094  3p22.1  0  0  0  0  0  0.299  0.149  0  0.157  0.149  0  0.529  0  0  0  0  0  0  0  0.215  0  0.071  0  0  0  0  0.089  0  0  0.051  0  0  0  0  0  0  0  0.386  0  0  0  0.941  0.081  0  0  0  0  0  0  0  0  0.29  0  0.091  0  0  0  0  0  0  
 
 VIPR1  7433  3  42530790  42579065  3p22  0.993  1.036  0.213  0  3.738  0.146  0.549  0.213  0.179  0.378  0.053  1.841  1.811  1.253  1.881  2.961  1.059  0.314  0.038  0.077  0  0.143  0.709  0.414  0.118  0  0.217  0.092  0  0.251  0  0.073  0  0.18  0.269  0.149  1.733  1.814  0.156  0.085  2.666  0.666  0.288  0.204  0.248  0.217  1.724  0.383  0  1.712  0.053  0.742  0.052  0.084  0.088  0.12  0.063  0.209  0  3.209  
 
 SEC22C  9117  3  42589458  42642572  3p22.1  1.796  4.278  2.233  2.584  3.544  2.035  3.211  2.023  3.465  2.84  3.19  0.778  2.146  3.707  1.96  1.689  3.044  3.643  4.07  1.837  2.539  2.658  2.154  1.231  2.155  2.966  2.229  1.945  2.093  1.838  2.708  2.669  1.863  1.61  1.506  2.482  2.595  2.771  3.596  2.209  2.197  2.481  2.494  2.184  1.21  1.334  2.284  2.115  2.033  4.118  3.067  1.825  2.287  1.236  2.779  1.73  1.745  2.82  2.516  3.046  
 
 SS18L2  51188  3  42632297  42636490  3p21  3.273  5.978  2.042  3.009  4.312  2.186  3.642  2.523  2.759  2.984  3.383  2.45  2.936  4.197  2.448  3.428  3.662  7.322  5.271  4.789  3.536  4.588  4.129  3.434  2.846  2.789  2.426  2.013  2.467  1.917  3.12  2.789  2.397  2.324  1.959  2.231  2.721  2.473  4.434  2.088  2.128  2.976  3.763  3.127  2.505  2.622  2.338  2.568  1.549  4.537  2.889  1.686  2.801  2.107  3.774  2.373  2.416  3.217  3.403  3.158  
 
 NKTR  4820  3  42642146  42690233  3p23-p21  1.919  3.654  1.27  1.287  2.736  1.358  2.078  1.768  1.396  2.278  2.364  0.887  2.52  3.216  2.821  2.016  1.578  3.801  4.123  2.582  2.32  3.041  1.382  1.57  1.254  2.093  1.732  2.105  1.72  1.252  2.18  1.912  1.739  0.765  1.439  2.1  2.274  2.187  2.38  1.639  1.8  1.457  2.453  2.409  1.27  1.602  2.153  2.047  1.991  2.5  2.328  1.142  2.173  0.964  2.492  1.52  2.288  1.912  2.499  2.26  
 
 LOC101928323  101928323  3  42653801  42695880  3p22.1  0  0  0.882  1.018  0.416  0.444  0  0.507  0.715  0.944  0.281  0.273  0  0.329  0  0  0  0  0  0  0  0.309  0  0  0.486  0.394  0.507  0.147  0  0.184  0.332  0  0  0.527  0.371  0.45  0  0.419  0.806  0  0  0  0.211  0  0  0.317  0.179  0.397  0  0.456  0  0.661  0.416  0.33  1.024  0  0  0.322  0  0.775  
 
 ZBTB47  92999  3  42695175  42709072  3p22.1  0.364  0.359  1.654  1.651  1.505  0.683  1.434  1.501  1.612  2.311  0.691  0.271  1.299  0.808  1.214  0.257  0.667  0.079  0.086  0.834  0.45  0.226  0.658  0.349  1.46  1.377  1.657  0.778  0.73  0.144  1.097  1.565  1.634  0.882  0.412  1.536  1.187  1.658  1.892  0.18  0.198  0.676  1.337  0.933  0.64  0.526  0.331  1.317  0.978  1.663  0.812  1.01  1.706  1.01  1.702  1.416  1.26  1.366  1.224  1.914  
 
 KLHL40  131377  3  42727010  42733938  3p22.1  0  0.054  0.219  0  0.082  0.105  0.077  0.048  0.194  0.057  0.033  0.09  0.056  0.046  0  0.074  0.155  0.136  0.046  0.124  0  0.421  0.438  0  0  0  0.143  0  0  0.17  0  0.058  0  0.151  0.143  0  0.038  0  0.161  0  0  0.664  0.082  0  0.092  0.161  0.108  0  0  0  0.05  1.208  0  0.18  0.16  0.13  0  0  0  0  
 
 HHATL  57467  3  42734154  42744319  3p22.1  0  0.081  0  0  0.053  0  0  0.065  0.165  0.051  0  0.076  0  0.085  0.127  0.077  0  0.09  0  0.096  0  0.165  0.289  0.128  0  0  0.157  0  0  0.101  0  0  0.095  0.166  0  0  0  1.114  0.096  0  0.089  0.362  0.057  0  0  0.058  0.064  0  0  0.05  0  0.545  0.127  0.1  0.065  0.11  0.058  0  0  0  
 
 CCDC13  152206  3  42749873  42814745  3p22.1  0  0  0.11  0  0.046  0  0.042  0  0.274  0.074  0.105  0  0.054  0.111  0.082  0  0  0.054  0.062  0.061  0  0.167  0.297  0  0  0.045  0.053  0.072  0  0.045  0  0.274  0.056  0.153  0.054  0  0  0.07  0.09  0.106  0  0.502  0.031  0  0  0.033  0.045  0  0  0.09  0.055  0.297  0.033  0.04  0.044  0  0  0  0  0.076  
 
 CCDC13-AS1  100874114  3  42774066  42786650  -  0  0  0  0  0  0  0  0  0  0  0  0  0  0  0  0  0  0  0  0  0  0  0  0  0  0  0  0  0  0  0  0  0  0  0  0  0  0  0  0  0  0.539  0  0  0  0  0  0  0  0  0  0  0  0  0  0  0  0  0  0  
 
 LOC729083  729083  3  42812103  42815126  3p22.1  0  0  0.206  0  0.072  0  0  0.109  0.128  0  0.104  0  0  0.096  0  0  0  0  0.106  0  0  0.358  0.494  0  0  0  0.131  0.067  0  0.062  0  0  0  0.214  0  0  0  0  0.154  0  0.258  0.63  0.067  0  0  0.223  0.054  0  0  0  0.087  0.763  0  0  0  0  0  0  0  0  
 
 HIGD1A  25994  3  42824399  42846027  3p22.1  1.851  5.504  2.605  2.555  4.069  2.993  4.146  2.942  3.369  3.219  4.332  2.745  3.687  4.233  3.038  2.712  4.185  4.872  4.986  3.495  3.511  3.391  2.269  1.806  3.203  3.7  2.498  2.715  2.945  2.427  4.029  3.246  2.768  2.257  2.18  3.144  3.05  3.146  4.787  3.176  3.429  2.887  3.959  3.797  3.041  3.675  3.308  3  2.499  5.097  4.117  2.011  3.258  1.755  3.502  2.276  2.556  3.376  3.156  4.399  
 
 ACKR2  1238  3  42850963  42908775  3p21.3  0  0.031  0.136  0  1.971  0.086  0.085  0.032  0.219  0.115  0.064  0.053  0.057  0.057  0.109  0.043  0.109  0  0.078  0.072  0.155  0.12  0.392  0  0  0.128  0.039  0.026  0  0.138  0  0  0  0.147  0.069  0  0  0  0.056  0.247  0.487  0.062  0.184  0.325  0.493  2.392  0.052  0  0.098  0.045  0.115  0.41  0.066  0.086  0.052  0  0  0  0  0.057  
 
 CYP8B1  1582  3  42913683  42917633  3p22.1  0  0  0.138  0  0.132  0  0  0.018  0.197  0.034  0.024  0  0  0.028  0  0.029  0  0.073  0  0.046  0  0.098  0.327  0  0  0  0.058  0  0  0.054  0  0  0  0.372  0  0  0  0  0.034  0  0  0.148  0.03  0.353  0.047  0.48  0.035  0  0  0  0.025  0.212  0  0.037  0.022  0.055  0  0  0  0  
 
 ZNF662  389114  3  42947401  42960825  3p22.1  0  0.345  0.203  0  0.031  0.069  0.016  0.206  0.107  0.039  0.027  0.047  0  0.027  0  0  0  0.09  0.021  0.036  0.077  0.043  0.23  0  0  0.022  0.057  0.042  0  0.042  0  0.021  0  0.183  0  0  0  0  0.019  0.035  0  0.09  0.027  0  0.083  0.44  0.404  0.671  0  1.308  0  0.114  0.033  0.037  0.017  0.027  0  0.04  0  0  
 
 KRBOX1-AS1  100506275  3  42975735  42978277  -  0  0.199  0.177  0  0  0.05  0  0  0.19  0.048  0  0  0  0.026  0  0  0  0  0  0  0  0.121  0.256  0  0  0  0.072  0  0  0.073  0  0  0  0.22  0  0  0  0  0  0  0  0.44  0.032  0  0  0.069  0.04  0  0  0  0  0.284  0.032  0.063  0  0  0  0  0  0  
 
 KRBOX1  100506243  3  42977833  42984283  3p22.1  0  6.076  1.485  0  0.115  0  0.234  0  0  1.956  0  0  0  0  0  0  0  0  0  0  0.734  0  0  0  0  0.287  0  1.093  0  0  0  0  0  0  0  0  0  0  0  0  0  0  0  0  0  0.317  3.872  0  0  0.158  0  0.658  0  0  0  0  0  0  0  0  
 
 FAM198A  729085  3  43020758  43099207  3p22.1  0.143  0.124  0.217  0  1.19  0.069  0  0.037  0.15  0.34  0.043  0.033  0  0.377  0  0.031  0  0.166  0.104  0.327  0  1.095  0.655  0.305  0  0  0.088  0.107  0  0.066  0  0  0  0.177  0  0  0  0  0.031  0.068  0  0.145  0.227  0.788  0  0.143  0.212  0.06  0  0.066  0.042  0.427  0.034  0.052  0.05  0.042  0  0  0  0  
 
 POMGNT2  84892  3  43120720  43147575  3p22.1  2.243  2.224  2.058  2.78  3.452  1.573  3.463  1.929  3.445  3.543  2.721  1.933  2.549  3.455  3.453  1.765  2.737  1.357  4.319  2.54  2.282  3.354  1.387  1.765  2.329  2.553  3.202  3.475  3.341  3.154  2.665  2.971  2.882  1.732  1.536  1.967  2.811  2.842  1.851  3.585  2.564  2.339  3.505  2.857  2.284  1.758  2.387  3.144  2.064  3.632  3.167  2.413  3.169  1.552  2.235  1.627  1.694  2.829  2.583  2.7  
 
 SNRK  54861  3  43328003  43392634  3p22.1  0.955  1.853  0.595  0.771  1.121  0.607  1.563  1.211  1.505  1.29  1.317  0.601  1.691  1.795  1.303  0.877  1.684  1.24  2.887  1.445  0.756  1.452  1.247  1.049  1.108  1.943  1.386  1.451  1.664  1.219  1.681  1.267  1.296  0.561  0.576  1.122  1.335  1.32  1.116  1.419  1.004  0.692  1.328  1.215  0.909  0.622  1.052  1.124  0.844  1.633  2.793  0.796  1.324  0.41  0.896  0.744  1.071  1.15  1.498  1.813  
 
 SNRK-AS1  100873954  3  43388410  43393454  -  0.099  0.225  0.159  0.34  0.373  0.149  0.252  0.154  0.269  0.388  0.188  0  0.053  0.118  0.215  0.052  0.066  0.18  0.033  0.28  0  0.279  0  0  0  0.114  0.079  0.165  0.12  0.056  0.07  0.076  0.054  0.152  0.066  0.06  0.119  0.167  0.337  0.158  0.073  0.517  0.042  0.049  0  0.05  0  0.301  0.075  0.047  0.305  0.286  0.115  0.188  0.091  0  0.264  0.123  0.161  0  
 
 ANO10  55129  3  43407817  43663560  3p22.1  1.461  1.52  3.193  2.427  3.203  1.558  4.22  2.445  2.539  2.93  2.142  1.001  2.097  1.537  1.978  0.746  1.095  0.339  1.581  0.736  2.111  1.451  1.669  1.009  2.35  2.233  2.454  2.312  2.3  2.048  3.213  2.722  2.316  1.542  1.662  1.963  2.801  2.713  2.307  1.836  2.142  2.135  2.187  1.555  1.327  1.856  3.058  2.256  1.773  2.889  3.306  1.287  2.848  1.9  2.469  2.269  2.834  2.801  3.161  3.229  
 
 ABHD5  51099  3  43732374  43764217  3p21  0.964  2.922  0.934  0.785  2.084  0.91  2.314  1.227  1.204  1.575  1.667  0.501  1.628  1.756  1.135  0.718  1.133  1.143  1.901  1.111  1.426  1.028  0.914  0.561  0.547  1.39  1.062  0.965  0.977  0.6  1.407  1.05  1.146  0.752  0.546  1.094  1.235  1.379  1.565  1.078  1.434  0.876  1.626  0.797  0.646  1.115  0.995  0.851  0.868  1.505  2.123  0.398  1.493  0.539  1.581  0.873  1.509  1.424  2.287  2.09  
 
 TOPAZ1  375337  3  44283377  44373590  3p21.31  0  0.033  0.048  0  0  0.028  0  0.016  0.073  0.062  0  0  0  0.013  0  0  0  0  0  0.042  0  0.058  0.127  0  0  0  0.053  0.022  0  0.044  0  0  0  0.141  0.024  0.019  0  0  0.028  0  0  0.136  0.027  0  0  0.021  0.04  0.057  0  0  0.013  0.111  0.011  0.043  0  0.032  0  0  0  0  
 
 TCAIM  285343  3  44379610  44450940  3p21.31  2.124  3.385  1.192  1.719  2.494  1.385  2.06  1.506  2.449  1.609  2.454  1.446  2.224  2.363  1.57  1.826  2.214  2.186  3.8  2.451  2.089  2.545  2.069  1.271  1.853  2.361  1.564  1.683  1.397  1.489  2.066  1.316  1.577  1.38  1.344  1.72  1.898  2.242  2.34  1.71  2.086  1.969  2.39  2.022  1.558  1.669  2.441  1.721  0.923  2.701  2.316  1.206  1.889  1.197  2.437  2.04  1.593  2.019  1.952  2.985  
 
 ZNF445  353274  3  44481261  44519162  3p21.32  0.743  0.644  0.839  0.475  0.984  0.812  0.87  1.048  0.847  1.067  1.057  0.491  1.062  1.265  1.371  0.55  0.607  0.406  2.122  1.08  1.404  1.578  0.428  0.467  0.581  0.818  0.55  0.834  0.735  0.61  0.738  0.777  0.774  0.3  0.571  0.871  1.186  0.966  0.61  0.875  0.732  0.535  0.933  1.116  0.741  0.658  0.739  1.176  1.026  0.953  1.32  0.523  1.105  0.276  0.647  0.582  0.856  0.883  0.89  1.383  
 
 ZNF852  285346  3  44540461  44552132  3p21.31  0.363  0.818  0.388  0.139  0.5  0.273  0.279  0.299  0.26  0.323  0.249  0.093  0.458  0.523  0.771  0.277  0.472  0.431  1.045  0.588  0.855  0.668  0.488  0.42  0.198  0.184  0.11  0.287  0.295  0.139  0.277  0.263  0.262  0.22  0.252  0.46  0.562  0.506  0.291  0.414  0.352  0.545  0.64  0.564  0.094  0.167  0.111  0.329  0.569  0.308  0.387  0.23  0.179  0.241  0.293  0.166  0.422  0.236  0.477  0.448  
 
 ZKSCAN7  55888  3  44596666  44624956  3p21.32  0  0.496  0.394  0.348  0.058  0.174  0.109  0.292  0.425  0.514  0.207  0  0  0.133  0  0.073  0.125  0.213  1.825  0.153  0  0.234  0.281  0  0  0.144  0.111  0.098  0  0.08  0  0  0  0.245  0.13  0.684  0.817  0.101  0.709  0.256  0.092  0.956  0.782  0.209  0  0.123  0.045  0.179  0  0.054  0  0.715  0.069  0.1  0.098  0.132  0.34  0.252  0  0.479  
 
 ZNF660  285349  3  44626455  44637557  3p21.31  0  0.532  0.145  0  0  0  0  0.19  0.06  0.096  0.109  0.056  0  0.11  0  0  0  0  0.336  0  0.091  0  0.154  0  0  0  0.039  0  0  0.034  0  0  0  0.155  0  0.06  0.54  0.229  0.072  0  0  0  0.048  0  0  0.089  0.047  0.253  0  0  0  0.237  0  0  0  0.555  0.407  0.19  0.547  0.981  
 
 ZNF197-AS1  100874094  3  44658619  44666289  -  0  0.157  0  0  0  0  0  0.244  0  0  0  0  0  0.201  0  0  0  0  0.557  0  0  0  0  0  0  0  0  0  0  0  0.335  0  0.349  0.361  0  0  0.169  0  0.103  0  0  0  0  0  0  0  0  0  0  0  0  0  0.192  0  0  0  0  0  0.236  0  
 
 ZNF197  10168  3  44666510  44689963  3p21  0.821  1.658  0.999  0.946  1.489  0.987  1.382  1.189  0.877  1.164  1.341  0.397  0.55  1.937  0.973  0.626  1.173  1.57  2.25  1.055  1.289  1.09  0.887  0.388  0.869  1.025  0.586  0.624  1.152  0.084  2.495  1.126  1.604  1.255  0.265  1.021  1.225  1.217  1.302  0.786  1.036  0.434  0.894  1.201  0.872  0.496  0.839  0.944  0.88  1.511  1.278  0.581  1.036  0.409  0.739  0.841  0.795  0.676  1.157  1.365  
 
 ZNF35  7584  3  44690232  44702283  3p21.32  0.864  1.093  1.02  1.16  2.189  0.868  1.67  1.358  0.922  1.146  1.743  0.659  0.561  1.417  1.286  0.936  1.087  0.689  0.604  1.341  1.229  0.133  0.949  0.856  0  0.507  0.864  0.06  1.013  0.112  1.533  0.3  1.295  0.814  0.587  1.311  1.525  1.033  1.565  0.147  1.304  0.562  1.85  0.067  1.358  0.962  0.868  1.188  0.918  1.414  1.491  0.616  1.55  0.541  1.111  0.679  1.14  1.681  1.52  1.665  
 
 ZNF502  91392  3  44754134  44765323  3p21.31  0  1.536  0.245  0.653  0  0.077  0.937  0.669  0.089  0.791  0.068  0.339  0.308  0.842  0  0.184  0  1.058  0.038  0.306  0.136  0.061  0.194  0  0  0.285  0.144  0.061  0  0.513  0  0.737  0  0.165  0.273  0.159  0.167  0.699  1.119  0.519  1.114  0.186  0.045  0  0.223  0.097  0.284  0.589  0  0.583  0  0.476  0  0.321  0.62  0.346  0.835  0  0  0.396  
 
 ZNF501  115560  3  44771097  44778575  3p21.31  0  0.788  0.291  0.555  0.07  0.552  0.401  0.11  0.715  0.302  0.56  0.095  0  0.022  0  0.141  0.321  0.279  1.37  0.053  0.319  0.296  0.464  0.102  0  0.089  0.115  0.223  0.068  0.058  0.063  0.257  0  0.216  0  0.183  0.448  0.366  0.231  0  0.488  0.093  0.015  0  0  0.165  0.4  0.139  0.418  0.026  0  0.251  0  0.241  0.073  0  0.461  0.467  0  0.341  
 
 KIAA1143  57456  3  44790235  44803173  3p21.31  1.293  2.702  1.115  1.495  2.449  1.396  1.683  1.577  1.225  1.901  1.861  1.178  1.652  1.93  1.962  1.542  2.373  2.996  3.163  2.49  2.227  2.039  1.132  1.308  1.608  1.863  1.131  1.393  0.898  0.429  1.382  1.27  1.327  1.108  1.37  1.808  1.537  2.267  2.397  1.811  1.494  1.678  1.728  1.549  1.272  0.965  0.335  1.707  1.29  2.543  2.193  1.193  1.383  0.914  1.767  1.026  1.534  2.104  1.933  2.539  
 
 KIF15  56992  3  44803208  44894748  3p21.31  1.235  3.126  0.766  0.573  1.255  1.223  1.352  1.135  0.367  1.781  1.727  0.726  1.069  1.846  1.184  1.063  1.65  2.483  3.655  1.521  1.503  2.1  0.605  0.838  0.969  1.152  0.961  0.976  0.971  0.637  1.453  1.001  1.342  0.649  0.868  1.221  1.167  1.359  1.307  1.833  1.271  0.951  1.91  1.629  0.615  0.979  1.92  1.173  1.311  1.868  1.684  0.745  1.246  0.423  1.263  0.749  1.413  1.686  1.508  1.822  
 
 TMEM42  131616  3  44903400  44907159  3p21.31  1.591  2.743  2.378  2.65  3.016  2.245  3.325  2.647  3.222  2.867  2.582  1.922  2.329  3.083  2.343  2.269  1.914  1.652  3.071  2.838  0.428  2.804  1.283  0.216  2.336  1.207  1.561  1.706  2.096  0.623  2.177  2.074  1.004  1.635  1.992  2.466  3.317  2.704  3.725  1.529  1.323  2.323  2.964  1.858  0.969  1.182  3.335  2.061  1.803  4.138  2.074  2.237  2.947  1.596  4.069  2.938  1.726  3.9  3.103  3.208  
 
 TGM4  7047  3  44916097  44956088  3p22-p21.33  0  0  0.11  0  0  0.053  0  0.035  0.114  0.03  0.222  0  0  0.021  0  0.025  0  0.038  0.051  0.056  0  0.081  0.238  0  0  0  0.084  0.024  0  0.055  0  0  0  0.197  0  0  0.152  0  0.394  0  0  0.029  0.036  0.087  0  0.117  0.028  0  0.109  0.111  0.229  0.247  0.12  0.049  0.08  0.107  0.06  0  0.097  0.185  
 
 ZDHHC3  51304  3  44956752  45017674  3p21.31  1.308  1.6  1.204  1.191  1.767  1.136  2.235  1.308  2.031  1.546  1.752  1.913  2.116  1.926  1.611  2.033  2.049  1.222  2.257  2.167  1.603  1.476  1.137  1.095  1.413  1.239  1.422  1.386  1.299  1.355  1.208  1.215  1.157  0.605  0.879  1.457  2.154  1.57  1.196  1.448  1.976  1.294  1.689  1.116  1.506  1.281  1.912  1.674  1.221  2.126  2.292  1.041  1.771  0.861  1.552  0.938  1.158  1.934  2.094  1.968  
 
 EXOSC7  23016  3  45017740  45054158  3p21.31  3.512  4.215  2.728  3.325  4.142  3.317  4.687  3.137  3.411  2.887  3.506  3.081  3.585  4.726  3.113  3.678  4.123  3.852  5.915  4.985  4.318  4.583  3.741  3.742  3.176  3.066  3.381  3.266  3.158  3.219  3.629  3.793  2.844  2.016  2.892  2.889  4.325  3.429  3.387  3.334  3.482  3.198  3.969  3.229  2.911  3.022  4.237  3.344  2.587  5.121  3.877  2.72  3.854  2.071  4.281  2.899  2.477  3.953  3.778  4.066  
 
 CLEC3B  7123  3  45067758  45077565  3p22-p21.3  0  0.152  1.328  0  0.244  0  0.39  0.313  0.479  0.704  0.215  0.675  0.239  0.404  0  0.233  0.233  0  0.428  0.544  0  0.757  0.877  0.714  0.24  0.146  0.195  0.127  0.22  0.136  0  0.446  0  0.275  0  0  0.285  0  0.427  0  0  1.174  0.185  0  0.364  0.419  0.547  0.264  0  0.477  0.309  1.604  0.167  0.326  0.307  0  0.218  0  0.253  0  
 
 CDCP1  64866  3  45123765  45187914  3p21.31  0  3.975  0.278  5.824  3.052  0.109  4.241  0.057  0.262  0.065  0.062  3.439  4.204  5.224  3.484  3.157  3.762  1.977  0.073  0.165  1.883  0.175  0.903  0.508  0.053  1.017  4.409  0.229  0.476  0.148  0.078  0.065  0  0.252  0.705  3.113  4.771  5.853  4.806  4.035  4.514  0.997  0.062  0.556  2.403  3.21  4.579  0  2.725  0  6.16  3.982  5.376  4.99  3.83  3.869  4.062  5.22  4.394  4.898  
 
 TMEM158  25907  3  45265955  45267814  3p21.3  0.217  3.915  4.805  6.026  0.113  2.572  5.157  1.159  4.533  3.459  2.902  0.185  0.168  1.757  0.25  0.527  0.547  0.992  0.062  0.969  1.627  0.628  0.461  0.239  3.417  1.397  1.913  1.282  2.794  0.416  1.214  3.365  1.823  3.994  0.46  2.509  2.066  4.211  3.766  1.718  0.563  3.406  1.193  0.133  0.259  1.06  0.949  0.262  0.321  0.221  4.284  2.07  1.992  1.238  0.982  0.292  1.312  1.343  1.295  1.152  
 
 LARS2  23395  3  45430074  45590328  3p21.3  1.707  1.313  1.11  1.74  1.633  1.201  2.362  1.285  1.798  1.701  1.987  1.31  2.257  2.224  2.64  1.557  2.05  1.285  3.124  1.782  2.443  1.728  1.086  1.633  1.786  1.953  1.832  2.079  2.03  2.544  2.337  2.336  2.095  0.874  1.159  1.424  2.105  1.775  0.74  1.754  1.437  1.509  2.182  1.587  1.319  1.176  1.28  2.115  1.334  2.477  2.512  1.148  1.918  0.639  1.688  1.091  1.403  1.861  1.703  2.747  
 
 LARS2-AS1  100885795  3  45524186  45551037  3p21.31  0.337  0.117  0.267  0  0.283  0.102  0.377  0.149  0.162  0.253  0.229  0  0.277  0.552  0.491  0.208  0.122  0  0.555  0.4  0  0.397  0.443  0.108  0  0.231  0.219  0.183  0.175  1.075  0.532  0.309  0.233  0.25  0.111  0.175  0.194  0.216  0.099  0.137  0.266  0.382  0.958  0.108  0  0.101  0.076  0.57  0.153  0.302  0.3  0.215  0.432  0.064  0.215  0  0.283  0  0.292  0.188  
 
 LIMD1  8994  3  45636322  45722755  3p21.3  1.389  0.793  1.485  2.518  1.586  1.266  1.333  1.957  3.575  1.681  2.595  1.299  2.4  1.573  2.15  1.283  1.269  0.602  2.907  1.097  2.038  1.707  1.447  2.063  1.661  2.374  1.556  2.238  3.703  1.091  2.669  2.552  0.035  0.173  1.788  2.506  2  1.856  1.505  2.299  1.745  1.94  1.21  1.962  1.131  1.127  0.976  1.43  0.863  1.275  3.049  1.078  1.486  1.081  1.284  1.531  1.855  2.307  1.823  2.798  
 
 LIMD1-AS1  644714  3  45719656  45730374  3p21.31  0.124  0.075  0.117  0.352  0.077  0  0  0  0.349  0  0.041  0  0  0.166  0  0  0  0  0.112  0  0  0.195  0  0  0  0.078  0.026  0  0  0.025  0.101  0  0  0.155  0  0.119  0  0  0  0.138  0.069  0.363  0  0  0  0  0  0  0  0  0.048  0.092  0.073  0.049  0  0  0  0  0  0  
 
 SACM1L  22908  3  45730753  45786900  3p21.3  1.109  2.549  1.383  1.525  1.965  0.971  2.057  1.343  2.28  1.794  2.002  0.784  2.281  1.983  2.007  1.367  1.844  1.352  4.653  2.838  1.861  2.261  1.567  1.166  1.718  2.531  1.549  1.529  2.048  2.031  2.355  2.252  1.437  1.247  0.906  1.984  1.963  1.804  1.904  1.777  1.547  1.445  1.314  1.566  1.144  1.361  0.675  1.176  1.197  2.88  2.548  0.759  1.568  0.996  1.629  1.133  1.453  1.547  1.495  2.621  
 
 SLC6A20  54716  3  45796940  45838035  3p21.3  0.087  0.058  0.137  0  0.033  0  0.061  0.025  0.177  0.029  0.071  0  2.932  0.631  0.614  1.611  2.117  0.193  0.708  0.06  0  0.242  0.318  0  0  0.028  0.097  0.022  0.035  0.079  0  0.024  0  0.176  0.038  0.063  0.036  0.053  0.08  0.059  0  0.633  0.05  0.469  0  0.203  0.124  0  0  0.02  0.043  0.321  0.017  0.107  0.242  0.124  0.033  0  0.029  0.084  
 
 LZTFL1  54585  3  45864809  45957216  3p21.3  0.938  2.49  0.677  1.258  1.554  0.706  0.974  1.189  1.515  1.008  1.009  0.888  0.658  1.801  0.991  1.089  1.315  3.056  4.303  1.748  0.828  2.681  1.123  0.903  1.103  0.748  0.875  0.837  0.773  0.689  0.982  0.822  0.759  0.699  0.521  0.979  0.806  1.183  0.68  0.793  0.733  0.695  0.903  0.556  0.931  0.516  0.362  0.814  0.413  1.985  0.934  0.357  1.154  0.555  1.134  0.797  0.845  0.926  1.157  1.813  
 
 CCR9  10803  3  45927995  45944667  3p21.3  0  0  0.183  0  0  0.056  0.041  0  0.187  0.058  0  0  0  0.07  0  0.036  0.107  0  1.134  0  0  3.351  0.397  0  0  0.117  0.053  0  0.048  0.049  0.117  0  0  0.203  0.066  0  0.059  0  0.026  0  0  0.042  0.032  0.055  0  0  0.022  0.095  0  0  0.044  0.376  0  0  0.034  0.086  0  0.09  0  0  
 
 FYCO1  79443  3  45959390  46037316  3p21.31  1.129  0.699  0.926  1.126  1.754  0.864  1.812  0.433  1.383  0.976  1.284  0.404  1.773  1.438  2.027  1.105  1.021  0.24  1.544  1.074  1.284  1.24  0.673  0.844  1.045  3.031  2.54  2.372  3.006  2.153  2.151  1.625  1.662  0.9  0.767  1.541  1.948  1.838  0.652  1.855  1.899  0.816  1.371  1.752  1.23  0.987  0.327  1.591  1.24  1.407  1.933  1.877  1.256  0.924  1.724  1.172  0.853  1.714  1.356  2.62  
 
 CXCR6  10663  3  45984972  45989845  3p21  0.318  0.04  0.197  0.14  0.255  0.126  0.27  0.063  0.213  0.118  0.163  0  0.339  0.397  0.728  0.18  0  0  0.209  0.188  0.257  0.271  0.342  0.214  0.076  0.752  0.37  0.758  0.77  0.208  0.514  0.227  0.266  0.089  0.217  0.083  1.045  0.284  0  0.171  0.393  0.08  0.363  0.355  0.159  0.082  0.047  0.444  0.268  0  0.184  0.392  0.288  0.071  0.181  0  0.267  0.143  0.162  0.286  
 
 XCR1  2829  3  46062290  46068979  3p21.3  0  0  0.19  0  0  0  0.086  0  0.135  0.051  0  0  0  0  0  0  0.112  0.223  0  0  0  0.179  0.766  0  0  0  0.074  0  0  0.079  0  0  0  0.235  0  0  0  0  0.1  0  0  0.142  0.451  0  0  0  0.988  0  0  0  0  0.753  0  0.08  0.102  0  0  0  0  0  
 
 CCR1  1230  3  46243199  46249832  3p21  0  0.086  0.123  0.433  0.057  0  0.422  0.149  0.207  0.077  0  2.078  0  0  0  0.06  0  0  0  1.329  0  0.132  3.272  4.605  0  1.538  0.206  0.074  0.36  0.093  0.437  0.085  0  0.166  0  0.073  0  0.111  0.054  0  0.09  0.254  0.022  0  0  0.055  0  0  0  0  0  0.201  0  0.326  0.054  0  0.049  0  0  0  
 
 CCR3  1232  3  46283871  46308197  3p21.3  0  0  0.165  0  0  0  0.154  0  0.162  0  0  0  0  0  0  0  0  0.137  0  0.319  0  0.059  0.68  0.416  0  0  0.054  0  0  0.066  0  0  0  0.188  0  0  0  0  0.035  0  0.154  0.586  0  0  0  0  0  0  0  0  0  0.485  0  0.079  0  0  0  0  0  0  
 
 CCR2  729230  3  46395234  46402413  3p21.31  0  0.067  0.143  0  0.042  0  0.066  0.029  0.122  0  0  0  0  0.022  0  0  0  0.244  0  0.31  0  0.132  3.097  0.651  0  0  0.114  0  0  0.069  0  0  0  0.394  0  0.09  0.053  0  0.056  0  0  0.295  0.049  0  0  0.065  0.069  0  0  0  0.044  0.329  0  0.048  0.064  0  0  0  0  0  
 
 LOC102724297  102724297  3  46406150  46448550  3p21.31  0  1.349  0  0  0.104  0  0  0  0  0  0  0  0  0  0  0.561  0  0  0  0  0  0.08  0  0  0  0  0.067  0  0  0.074  0  0  0  0  0  0  0.811  0.198  0  0  0  0  0  0  0  0  0  0  0  0  1.319  0.211  0  0  0  0  0  0  0  0.221  
 
 CCR5  1234  3  46411632  46417697  3p21.31  0  0  0.103  0  0.032  0  0  0.019  0.141  0  0  0  0  0.019  0  0.022  0  0  0.039  0.031  0  0.039  0.393  0  0  0  0.055  0  0  0.041  0  0  0  0.236  0  0.054  0.053  0  0  0  0  0.081  0.018  0  0  0  0.027  0  0  0  0  0.166  0  0.026  0.034  0  0  0  0  0  
 
 CCRL2  9034  3  46448720  46451014  3p21  0  4.398  0.132  0  0  0  1.205  0  0.15  0  0  0.544  0.698  0  0  1.071  0  0  1.671  0.179  0.464  1.399  0.264  0.127  0  0.102  0.06  0  0  0.079  0  0  0  0.191  0  0  1.825  1.079  0.357  0  0  0.089  0  0  0  0  0.369  0  0  0.06  2.345  0.163  0.244  0.048  0  0  0.081  0.621  0  1.436  
 
 LTF  4057  3  46477495  46506632  3p21.31  0  0.04  0.315  1.179  0.026  0  0.828  0.038  0.117  0.034  0.032  0.084  0  0.018  0  0  0.058  0  0.066  0.043  0  0.112  3.119  0.769  0  0  0.067  0.033  0  0.073  0  0  0  0.1  0  0  0.108  0  0.036  0  0  0.116  0.061  0.078  0  0.131  0.034  0  0.354  0  0.053  0.3  0.033  0.056  0.059  0  0  0.084  0  0.102  
 
 RTP3  83597  3  46539484  46542439  3p21.3  0  0.11  0  0  0  0  0.454  0  0.157  0  0  0  0  0  0  0  0  0  0  0  0  0.276  0  0.183  0  0  0.124  0  0  0.098  0  0  0  0  0  0  0  0  0.154  0  0  0.222  0  0  0.297  0.311  0  0  0  0  0  0  0  0  0  0  0  0.787  0  0  
 
 LRRC2  79442  3  46556877  46608040  3p21.31  0  0.023  1.582  0.204  0.032  0.059  0.12  0.056  0.182  0.029  0.198  0.202  0  0.023  0  0.031  0  0.477  0.5  0.047  0  0.096  0.391  0.078  0  0.516  0.177  0.047  0  0.139  0.101  0.032  0  0.181  0.032  0  0.307  0.047  0.149  0  0.043  0.094  0.033  0  0  0.037  0.339  0.073  0  0.212  0.025  0.229  0.017  0.066  0.017  0.038  0.017  0.374  0.024  0.123  
 
 LRRC2-AS1  83598  3  46598887  46601178  3p21.3  0  0  0.232  0  0  0  0  0  0  0  0  0  0  0  0  0  0  0  0  0  0  0.09  0  0  0  0  0.118  0.181  0  0.122  0  0  0  0  0  0  0  0  0  0  0  0  0  0  0  0  0  0  0  0  0  0  0  0  0  0  0  0  0  0  
 
 TDGF1  6997  3  46616044  46623952  3p21.31  0  0.057  0  0  0  0  0.066  0  0  0  0  1.189  0  0.028  0  0.083  0  3.078  0  0.078  0  0.088  0.175  0  0  0  0.117  0  0  0.128  0  0  0  0.175  0  0  0  0  0  0  0  0  0.033  0  0  0  0.13  0  0  0  0  0.105  0  0.044  0  0  0  0  0  0  
 
 LOC100132146  100132146  3  46653924  46668033  3p21.31  0  0  0  0  0  0  0  0  0  0  0  0  0  0.029  0  0  0  0.199  0.049  0  0  0.038  0.322  0  0  0  0.437  0  0  0.288  0  0  0  0.096  0  0  0  0  0  0  0  0.249  0.038  0  0.088  0  0.185  0  0  0  0  0.084  0  0.081  0  0  0  0  0  0  
 
 ALS2CL  259173  3  46710484  46735194  3p21.31  0.986  0.453  1.559  0  1.742  0.161  0.243  0.163  0.196  0.227  0.139  0.361  1.842  0.843  1.306  1.428  0.8  0.095  0.043  0.19  0  0.12  0.664  0.578  0.254  0.188  0.091  0.294  0.08  0.199  0.132  0.057  0  0.089  0.537  2.5  0.726  1.635  1.896  0.215  3.413  0.413  0.603  1.923  0.701  1.842  1.205  1.611  2.38  2.198  2.194  1.74  1.059  0.736  0.923  0.095  1.151  1.27  0.647  1.515  
 
 TMIE  259236  3  46742822  46752413  3p21  0.155  0.106  0.554  0  0.086  0  0  0  0.184  0.05  0.049  0  0  0.16  0  0.133  0  0.284  0  0  0  0.17  0.5  0  0  0  0.075  0.102  0  0.059  0  0  0  0.094  0  0.321  0.091  0  0.183  0.431  0.695  0  0.433  0.318  0  0.08  0.727  0.162  0  0.349  0.087  0.586  0  0.094  0.084  0  0.078  0.222  0.11  0.105  
 
 PRSS50  29122  3  46753605  46759373  3p21.31  0  0.097  0.1  0  0  0  0  0.136  0.159  0.048  0  0  0  0  0  0  0  0  0.07  0.083  0  0.059  0.452  0  0  0  0.139  0.055  0  0.118  0  0  0  0  0.089  0.422  0  0.157  0.073  0.601  0.096  0.055  0.93  0  0  0.226  0.296  0  0  1.088  0  0.563  0.054  0.145  0.135  0  0  0  0.445  0  
 
 PRSS46  100287362  3  46761072  46777921  3p21.31  0  0  0  0  0  0  0  0  0.127  0  0  0  0  0  0  0  0  0.137  0  0  0  0.058  0.449  0  0  0  0.058  0  0  0.062  0  0  0  0  0  0  0.099  0  0.046  0  0  0.598  0  0  0  0  0  0  0  0.181  0  0.161  0  0  0  0  0  0  0.157  0  
 
 PRSS45  377047  3  46783580  46786245  3p21.31  0  0.092  0  0  0  0  0  0  0.171  0  0  0  0  0  0  0  0  0  0  0  0  0.09  0  0  0  0  0  0  0  0.048  0  0  0  0  0  0.13  0.255  0  0  0  0  0  0  0  0  0  0  0  0  0.826  0  0.607  0  0  0  0  0  0.333  0.142  0  
 
 PRSS42  339906  3  46871893  46875585  3p21.31  0  0  0  0  0  0  0.257  0  0  0  0  0  0  0.128  0  0  0  0  0.154  0  0  0.153  0.567  0  0  0  0.125  0  0  0.096  0  0  0  0  0  0  0  0  0  0  0  0.26  0.118  0  0  0.099  0  0  0  0  0  0.818  0  0.083  0.196  0  0  0  0.247  0  
 
 MYL3  4634  3  46899356  46904973  3p21.3-p21.2  0  0  0.15  0  0  0  0  0  0.162  0  0  0  0  0.054  0  0  0  0.328  0  0  0  0.216  0  0  0  0  0.062  0  0  0.078  0  0  0  0  0  0.182  0  0  0.051  0  0  0.287  0.083  0.819  0  0.231  0  0  0  0.128  0  0.64  0  0.141  0.14  0  0  0  0  0  
 
 PTH1R  5745  3  46919235  46945289  3p22-p21.1  0.429  0.078  0.14  0  0.475  0.077  0.087  0.046  0.209  0.167  0.128  0.193  0.295  0.334  0.201  0.473  0.286  0.113  0.328  0.327  0  0.64  0.222  0.109  0.06  0.217  0.353  0.181  0.058  0.667  0.145  0.08  0  0  0.234  0.928  0.21  0.306  0.172  0.169  0  0.563  0.225  1.672  0.092  0.092  0.05  0.73  0  1.225  0.069  0.921  0.209  0.188  0.981  0.358  0.165  0.17  0.325  0.073  
 
 CCDC12  151903  3  46963219  47023500  3p21.31  3.793  3.664  3.008  3.445  4.438  3.506  5.264  3.387  3.721  3.532  3.901  3.636  3.406  4.547  3.135  3.744  4.45  3.469  6.087  4.755  4.247  4.948  3.421  3.817  3.259  3.26  3.789  3.064  3.153  4.258  3.438  3.464  2.933  1.66  3.205  3.832  3.724  3.855  4.245  3.75  3.839  3.833  5.126  3.495  2.711  2.567  5.098  3.937  2.507  5.362  3.481  2.298  3.944  2.142  4.322  3.254  3.037  4.571  3.847  4.053  
 
 NBEAL2  23218  3  47021172  47051194  3p21.31  2.996  0.136  0.317  1.132  3.225  0.737  0.839  0.623  1.215  0.897  1.982  3.005  3.379  1.933  2.734  3.049  3.09  0.098  3.278  1.703  2.87  2.526  1.017  2.365  1.331  1.992  1.136  2.478  1.387  1.474  1.231  1.001  1.447  0.394  1.485  3.107  2.402  2.906  1.248  2.265  3.379  2.154  1.613  2.306  1.561  1.701  0.921  2.275  0.655  2.165  1.724  2.17  1.815  0.768  2.151  1.369  1.635  1.773  2.889  2.236  
 
 NRADDP  100129354  3  47053031  47054957  3p21.31  0.37  0  0.2  0  0.71  0  0  0.104  0.215  0.143  0.207  0.415  0  0.389  0.376  0.423  0.406  0  0.714  0  0.385  0.457  0.355  0.309  0  0.155  0.449  0.234  0.23  0.083  0  0  0  0  0  0.275  0.195  0.336  0.184  0  0.291  0.115  0.159  0.202  0.294  0.13  0  0.211  0.292  0.429  0  0  0.117  0.096  0.106  0  0.138  0  0.532  0  
 
 SETD2  29072  3  47057897  47205467  3p21.31  2.585  2.901  1.789  1.599  2.298  1.645  2.715  2.536  2.053  2.35  2.666  1.57  2.538  2.827  2.976  1.987  2.059  2.281  2.975  2.698  2.405  3.007  1.533  1.974  2.057  2.078  1.941  2.231  1.862  2.063  1.954  2.056  1.981  0.881  1.675  2.568  2.7  2.457  2.977  2.485  2.136  1.539  2.504  2.221  1.923  1.894  1.992  2.59  1.953  2.449  2.564  1.399  2.621  1.56  2.395  2.034  2.284  2.412  2.674  2.892  
 
 KIF9-AS1  285352  3  47205859  47285606  3p21.31  0.263  0.117  0.346  0.291  0.245  0.044  0.259  0.24  0.384  0.249  0.226  0.077  0.318  0.287  0.549  0.123  0.094  0.107  0.111  0.175  0.131  0.159  0.398  0.084  0.105  0.07  0.138  0.177  0.11  0.083  0.083  0.11  0.072  0.223  0.111  0.329  0.347  0.346  0.473  0.272  0.085  0.302  0.283  0.074  0.208  0.115  0.038  0.385  0.191  0.518  0.74  0.436  0.39  0.18  0.179  0.069  0.258  0.174  0.292  0.245  
 
 KIF9  64147  3  47269515  47324337  3p21.31  0.554  1.005  0.333  0.473  0.876  0.225  0.944  0.195  0.836  0.38  0.341  0.829  0.559  0.868  0.385  1.144  1.182  2.647  1.408  1.34  0.635  0.953  0.381  0.574  0.546  0.324  0.592  0.494  0.587  0.587  0.729  0.748  0.557  0.505  0.681  0.627  0.38  0.233  0.703  0.391  0.431  0.605  1.052  0.219  0.433  0.36  0.568  0.551  0.215  1.178  0.447  0.431  0.475  0.298  0.896  0.26  0.129  0.346  0.599  0.686  
 
 KLHL18  23276  3  47324329  47388306  3p21.31  1.265  1.043  1.505  1.879  1.324  0.951  2.406  1.697  1.751  1.436  2.023  0.968  1.958  1.988  1.638  1.055  1.352  0.564  2.415  1.532  2.527  1.573  1.162  1.358  1.689  1.385  1.548  1.89  1.482  1.429  1.575  2.244  1.943  0.933  1.013  1.663  2.19  1.693  1.557  1.878  1.272  1.149  2.168  1.479  1.447  1.215  1.248  1.773  1.007  2.3  2.382  0.979  1.935  0.801  1.276  1.001  1.207  1.32  1.931  2.148  
 
 PTPN23  25930  3  47422471  47454931  3p21.3  2.761  0.396  2.863  3.661  3.372  2.412  3.988  3.46  3.281  3.417  3.717  2.05  3.746  3.181  3.888  2.808  2.95  0.139  2.969  2.027  3.209  2.777  1.808  2.987  2.979  4.008  4.017  4.528  4.358  4.886  4.323  4.281  4.39  2.038  2.536  3.55  4.285  4.194  3.17  4.133  3.716  2.914  3.785  2.72  3.448  3.411  1.31  4.298  2.149  3.784  3.638  2.881  3.687  2.383  3.343  3.008  3.325  4.18  3.912  4.185  
 
 SCAP  22937  3  47455183  47517445  3p21.31  3.18  0.708  2.466  2.501  2.89  1.911  4.392  3.36  3.819  3.216  2.936  2.68  3.404  2.548  3.8  2.359  2.573  0.092  4.386  2.627  3.267  3.336  2.207  3.049  3.047  2.914  3.184  3.864  3.087  3.593  3.005  3.451  3.167  1.335  2.137  3.121  3.934  3.403  2.425  3.743  2.969  3.02  3.059  2.771  3.353  2.355  1.933  3.427  2.603  3.654  3.223  2.218  3.345  1.518  2.915  2.555  2.359  3.522  3.087  3.745  
 
 ELP6  54859  3  47537129  47555199  3p21.31  3.152  2.885  2.623  2.691  3.088  2.614  4.416  2.88  2.738  3.245  3.072  2.42  3.06  3.636  3.242  2.751  3.35  1.339  5.168  2.801  3.597  3.933  2.436  2.468  2.757  2.939  3.761  2.966  3.094  3.394  3.603  3.785  3.288  2.188  1.995  2.459  3.861  3.367  2.93  3.007  2.746  2.688  4.05  2.421  2.629  2.248  3.133  3.482  2.294  4.982  3.186  2.398  3.314  1.318  3.46  2.594  2.151  3.453  3.089  3.628  
 
 CSPG5  10675  3  47603727  47621730  3p21.3  0.695  0.18  0.313  0.962  0.619  0.359  0.166  0.251  1.226  0.321  0.48  0.123  0.452  0.733  0.809  0.356  0.573  0.109  0.208  0.088  0.384  0.309  0.397  0.375  0.117  0.308  0.489  0.599  0.25  0.785  0.267  0.333  0.216  0.165  0.202  0.477  0.185  0.583  0.646  1.737  0.613  0.754  0.26  1.486  0.901  1.235  0.275  0.801  1.141  0.471  0.253  0.969  0.25  0.315  0.571  0.369  0.165  1.664  0.303  0.456  
 
 SMARCC1  6599  3  47627377  47823405  3p21.31  3.066  2.177  2.994  3.421  3.584  2.801  3.498  3.52  3.519  3.167  3.966  3.008  3.952  3.998  4.258  3.573  4.078  1.982  4.729  3.718  4.32  4.17  2.898  3.663  3.653  3.534  3.277  3.591  3.179  3.304  3.866  3.704  3.707  2.494  2.606  3.515  4.135  3.531  2.8  3.776  3.344  2.664  4.369  4  4.065  3.725  2.728  3.902  0.808  3.897  3.641  2.456  3.691  2.175  3.436  3.285  3.455  3.816  3.784  5.069  
 
 DHX30  22907  3  47844398  47891686  3p21.31  3.604  2.259  2.697  3.176  3.125  3.176  4.117  3.224  3.521  3.272  3.706  2.368  3.977  3.991  4.288  2.76  3.346  1.964  5.311  3.143  4.193  4.311  2.588  3.132  3.373  3.615  3.573  4.221  3.776  4.03  3.979  4.276  3.861  1.957  2.765  3.57  4.467  3.58  4.022  4.519  3.492  3.759  3.795  3.354  3.222  2.747  3.228  3.792  2.422  4.605  3.608  2.9  3.527  1.825  3.372  2.675  2.434  4.005  3.305  3.771  
 
 MAP4  4134  3  47892179  48130769  3p21  3.512  3.641  4.728  4.491  2.991  3.843  5.41  5.149  4.542  5.665  4.902  2.316  4.339  4.695  4.561  3.756  3.92  1.376  3.481  2.578  4.056  3.224  3.261  3.862  4.68  4.869  4.766  4.876  4.699  4.572  3.992  4.679  4.821  2.929  3.767  4.4  4.954  5.375  4.785  4.752  3.239  3.718  4.664  4.164  4.044  3.884  3.403  5.178  4.103  4.848  5.789  3.919  5.268  3.965  4.849  4.452  5.071  4.857  5.071  5.747  
 
 CDC25A  993  3  48198667  48229801  3p21  1.291  2.151  0.858  1.35  1.557  1.716  1.881  1.375  0.777  1.192  1.634  1.394  2.386  2.041  2.017  1.671  1.729  2.04  3.804  2.366  2.587  2.63  1.448  1.872  1.825  1.813  1.637  1.622  1.428  1.596  2.498  2.173  2.264  0.963  1.125  1.587  2.452  1.3  1.045  2.12  1.642  1.444  2.474  1.845  1.534  1.427  1.639  1.647  1.39  2.359  1.688  0.905  1.876  0.637  1.405  1.084  1.142  1.619  1.743  2.143  
 
 CAMP  820  3  48264836  48266981  3p21.3  0  0  0  0  0  0  0  0  0.254  0  0  0  0  0  0  0  0  0  0  0  0  0  0.62  0  0  0.282  0.07  0.143  0  0.07  0  0  0  0  0  0  0  0  0  0  0.34  0  0  0  0  0  0.163  0  0  0  0  0  0  0  0  0  0  0  0  0  
 
 ZNF589  51385  3  48282595  48312479  3p21  1.722  0.436  0.538  0.765  1.357  0.475  1.049  0.439  0.869  1.278  1.032  0.703  1.81  2.246  2.441  0.922  1.542  0.816  2.505  1.925  1.837  1.597  1.023  1.396  1.181  1.888  2.173  1.178  1.821  1.783  1.803  1.355  2.1  0.739  0.686  0.682  1.314  1.327  0.735  1.241  0.959  0.733  3.4  1.408  0.693  0.957  1.474  1.684  0.738  2.161  1.251  0.726  1.053  0.292  1.007  0.587  0.608  1.209  1.095  0.837  
 
 NME6  10201  3  48333806  48343175  3p21  1.65  1.734  1.578  1.885  2.252  1.544  2.739  1.484  1.798  1.588  2.191  1.362  2.231  1.801  1.667  1.438  1.545  0.605  2.738  1.939  1.909  2.244  1.813  1.976  2.1  1.25  1.674  1.735  1.446  1.869  2.296  1.954  2.141  1.217  1.387  1.96  2.347  2.491  2.47  2.151  1.763  1.396  2.465  1.876  1.273  1.388  1.134  1.974  1.411  2.969  2.225  1.258  1.973  0.903  2.037  1.731  1.659  2.584  2.363  2.176  
 
 SPINK8  646424  3  48348335  48369831  3p21.31  0  0  0.516  0  1.3  0.513  1.046  0  0  0.72  0.845  0.417  0.961  0.527  0.965  1.61  1.562  0  1.394  1.55  0  2.487  0  1.068  0.5  0.384  0.795  0.535  0.68  1.304  0.627  0  0  0  1.435  0.409  0.305  0  2.635  0.636  0  1.097  0.948  0.579  0  0  0.78  0.512  0  1.062  0.581  0  1.098  0.984  1.48  0.762  0.629  0  0.593  0.805  
 
 FBXW12  285231  3  48413708  48436190  3p21.31  0  0  0  0  0  0  0  0  0  0.066  0  0  0  0.043  0  0  0  0  0.103  0  0  0  0  0  0  0  0.083  0  0  0.071  0  0  0  0  0  0  0  0  0  0  0  0.209  0.05  0  0  0  0  0  0  0  0  0.168  0  0.063  0  0  0  0  0  0  
 
 PLXNB1  5364  3  48445260  48471460  3p21.31  2.477  0.027  0.897  1.088  2.749  0.592  1.194  1.65  1.798  2.54  2.093  1.72  3.006  1.771  2.882  2.911  2.068  0.146  1.94  0.207  0.64  0.974  0.484  0.328  0.706  1.017  1.881  1.619  0.967  1.044  1.204  1.675  1.335  0.601  1.236  2.363  1.55  1.086  1.181  0.781  1.566  0.988  2.639  2.28  2.969  2.296  1.431  1.446  1.246  1.347  1.217  0.832  1.473  1.219  2.114  1.772  1.438  0.419  2.198  2.544  
 
 CCDC51  79714  3  48473579  48481529  3p21.31  3.046  3.187  2.04  3.203  2.846  1.419  3.428  2.156  2.289  2.288  2.865  2.608  4.3  4.116  3.38  2.521  3.458  3.996  4.181  3.421  2.341  2.948  2.695  2.848  2.653  3.221  3.388  3.166  3.238  3.109  3.61  3.859  3.326  2.347  2.505  2.81  2.957  2.482  2.901  3.243  2.66  3.084  2.887  3.732  3.063  2.632  3.484  3.338  1.542  4.794  2.962  1.975  3.334  2.127  3.566  2.381  2.071  2.87  3.118  3.142  
 
 TMA7  51372  3  48481685  48485537  3p21.31  5.195  9.122  4.689  5.049  6.615  7.056  6.094  5.359  5.662  5.885  6.111  5.331  4.722  7.411  4.477  5.772  6.594  9.149  8.319  7.805  6.09  7.624  5.629  6.176  5.92  5.624  3.861  4.227  4.537  3.508  6.103  6.041  4.962  4.54  6.317  5.753  4.575  5.186  8.203  4.736  5.901  6.944  6.142  6.61  6.286  6.001  5.802  5.608  5.237  7.844  5.45  4.639  5.806  5.605  6.454  5.525  5.011  7.03  5.534  6.394  
 
 ATRIP  84126  3  48488113  48507054  3p21.31  2.094  0.701  1.2  1.75  1.5  1.087  2.842  1.186  1.269  1.874  1.836  0.984  2.158  1.932  2.404  1.246  1.708  0.137  3.481  1.339  1.74  1.573  1.209  1.57  1.133  1.807  2.152  1.689  1.656  2.014  2.107  2.42  1.856  0.86  0.97  1.883  2.393  2.112  0.978  2.24  1.48  1.683  1.698  1.627  1.587  1.553  1.448  1.82  1.293  2.903  1.872  1.378  1.94  0.692  1.461  1.019  1.289  1.179  1.714  1.96  
 
 TREX1  11277  3  48506918  48509044  3p21.31  2.153  0.184  2.05  4.672  1.957  2.555  4.546  2.849  3.026  2.977  3.883  1.697  3.573  3.216  2.671  3.86  2.617  0.156  4.548  2.697  0.597  2.946  3.371  3.839  2.624  3.004  4.018  2.719  3.339  4.046  2.995  3.446  3.51  1.961  1.797  1.291  1.763  2.263  3.29  0.885  0.98  4.082  2.116  2.825  1.464  1.869  3.252  3.322  1.45  4.799  2.31  2.063  3.186  2.537  3.055  1.964  2.37  3.157  2.585  2.634  
 
 SHISA5  51246  3  48509196  48542259  3p21.31  4.356  2.853  5.185  6.474  5.027  4.15  6.347  5.061  5.377  5.273  5.102  2.903  5.837  4.123  5.244  5.624  4.944  0.693  5.246  3.207  3.912  4.156  4.157  5.598  5.292  4.854  5.44  5.318  4.644  5.506  4.599  5.457  4.957  3.705  4.497  5.056  5.541  6.434  4.916  5.713  4.648  5.366  5.697  4.611  4.646  3.962  4.448  4.851  3.654  5.763  5.289  4.184  5.275  3.882  4.923  5.046  4.945  6.142  5.019  5.865  
 
 PFKFB4  5210  3  48555116  48594227  3p22-p21  1.26  0.493  1.949  1.498  1.448  0.688  2.802  2.463  2.297  2.1  3.999  0.223  1.967  2.016  2.094  1.111  3.302  0.146  0.921  0.538  1.265  0.788  0.637  0.617  1.131  4.22  3.394  4.917  3.722  2.754  4.947  3.731  2.69  1.399  0.725  1.456  2.316  3.038  1.207  1.084  1.312  1.897  1.026  2.085  0.696  1.248  1.836  1.14  0.48  2.108  2.309  1.209  3.754  0.916  1.412  1.361  3.512  1.029  1.146  1.504  
 
 UCN2  90226  3  48599150  48601201  3p21.3  0.505  0.347  2.374  0  0.497  0.342  2.094  3.098  0.321  1.987  3.55  0  0.26  0.646  0.15  0.316  0.234  0  0.208  0.07  0  0.248  0.575  0.247  0.497  5.83  3.591  6.562  7.233  1.832  6.832  6.027  3.372  1.444  1.327  2.309  3.988  2.602  0.995  0.616  0.184  1.555  0.962  0.404  0.394  0.502  3.271  1.902  1.156  3.025  0.492  0.489  5.59  0.728  2.068  0.37  4.369  0.261  0.59  1.783  
 
 COL7A1  1294  3  48601505  48632593  3p21.1  0.128  0.052  2.488  0.071  0.646  0.259  2.343  3.91  0.289  3.286  3.532  0.052  0.1  0.304  0.192  0.058  0.113  0.099  0.144  0.046  0  0.09  0.134  0.027  0.093  0.566  0.486  0.584  0.279  0.22  0.171  0.349  0.197  0.122  2.061  3.431  2.817  1.555  1.986  0.624  0.502  0.759  0.484  0.347  0.58  0.508  1.526  2.004  1.398  3.049  0.177  0.309  4.63  1.469  2.416  0.564  4.376  0.029  0.278  1.696  
 
 UQCRC1  7384  3  48636431  48647098  3p21.3  5.423  3.218  4.858  4.988  5.648  4.668  7.35  5.722  5.513  5.456  6.502  5.593  6.08  6.311  5.887  4.934  6.399  2.057  7.385  6.028  5.307  4.903  4.136  5.175  5.409  5.671  6.867  5.937  5.967  7.446  6.512  6.653  6.241  3.95  4.518  6.003  6.46  5.992  5.362  5.987  5.699  5.428  6.479  5.262  6.325  5.541  5.157  6.331  5.135  7.721  6.135  4.463  5.961  3.648  5.406  4.914  5.015  5.865  5.834  6.448  
 
 TMEM89  440955  3  48658274  48659189  3p21.31  0  0  0  0  0.42  0  0.462  0.26  0.373  0.376  0.426  0  0.349  0.455  0  0.24  0  0.579  0.202  0  0  0.569  0  0  0  0.492  0.655  0.191  0  0.676  0  0  0  0  0  0  0.365  0  0.25  0  0  0.475  0.539  0  0  0.196  0.172  0  0  0.66  0  1.17  0.398  0  0  0  0  0  0.332  0  
 
 SLC26A6  65010  3  48663155  48672926  3p21.3  2.259  1.174  2.081  2.013  2.275  1.377  3.587  2.869  2.074  3.112  2.979  1.032  2.893  3.242  2.196  1.65  1.975  0.58  3.719  2.232  1.836  2.811  1.191  1.624  1.109  3.227  3.248  2.53  2.643  2.537  2.975  3.051  2.75  0.956  1.324  2.9  3.09  3.163  2.684  2.219  1.706  2.324  2.879  2.39  1.613  1.303  3.517  2.707  1.343  3.836  2.224  2.092  2.801  1.16  2.047  1.511  2.582  2.399  2.463  2.221  
 
 CELSR3  1951  3  48673895  48700348  3p21.31  0.987  0.066  0.855  1.229  1.82  0.677  1.913  1.001  0.476  2.325  0.432  0.287  1.658  0.518  0.95  1.026  0.535  0.074  2.142  0.155  0.192  0.381  0.711  0.718  1.434  0.449  1.113  1.58  0.483  1.073  0.369  0.256  1.996  0.581  0.492  1.006  1.075  0.918  0.188  2.07  1.222  0.516  1.676  0.309  0.288  0.343  0.116  2.389  0.056  1.763  0.615  1.245  0.337  0.205  1.234  0.225  0.296  1.201  1.843  0.289  
 
 CELSR3-AS1  102724368  3  48701200  48706603  3p21.31  0  0  0  0  0  0  0  0  0  0  0  0  0  1.684  0  0  0  0  0  0  0  0  0  0  0  0  1.089  0  0  0  0  0  0  0  0  0  0  0  0  0  0  0  0  0  0  0  0  0  0  0  0  0  0  0.892  0  0  0  0  0  0  
 
 NCKIPSD  51517  3  48711271  48723366  3p21  2.769  1.638  2.455  2.882  2.506  2.162  3.404  2.887  2.715  3.188  3.504  2.025  3.343  3.173  3.665  2.731  2.977  1.052  3.71  2.183  1.876  2.519  1.659  2.485  2.207  2.774  3.278  3.546  3.379  3.126  3.105  3.326  3.339  1.527  1.76  2.856  3.488  3.149  2.255  3.466  2.971  2.626  3.265  2.782  2.6  2.003  2.659  3.682  2.187  4.079  3.127  2.727  2.991  1.424  2.743  2.37  2.407  3.165  3.02  3.095  
 
 IP6K2  51447  3  48725435  48754711  3p21.31  4.228  3.75  3.387  3.954  4.593  3.263  4.587  3.586  4.529  3.718  4.246  2.308  3.856  4.748  4.157  3.851  4.759  4.644  5.958  3.701  2.953  5.217  3.323  2.903  3.567  3.347  3.858  3.069  3.361  3.79  3.515  3.732  2.918  2.223  3.146  4.08  3.885  3.447  4.518  4.212  3.463  4.035  4.801  3.993  3.917  3.272  4.502  3.954  2.174  5.652  4.245  2.85  3.285  2.329  4.238  3.003  2.976  3.711  3.756  4.612  
 
 PRKAR2A  5576  3  48788092  48885270  3p21.3-p21.2  1.023  1.889  1.453  1.353  1.732  1.922  1.664  2.1  1.52  1.658  2.171  1.214  2.439  1.713  1.579  1.277  1.732  0.788  0.19  1.174  2.094  0.296  0.703  1.686  1.925  1.354  1.269  2.573  2.033  0.928  1.879  2.007  1.162  0.498  0.992  2.132  2.386  2.168  1.07  2.776  1.834  1.346  1.215  2.335  2.02  1.644  0.185  2.319  1.914  1.415  2.483  1.278  2.245  1.147  0.627  1.284  1.854  1.986  0.754  2.582  
 
 PRKAR2A-AS1  100506637  3  48885369  48889415  -  0.108  0.241  0.275  0.352  0.507  0.181  0.392  0.126  0.4  0.256  0.251  0.142  0.267  0.326  0.09  0.225  0.363  0  0  0.089  0.063  0.098  0.849  0.205  0.1  0.19  0.057  0.349  0.242  0.273  0.148  0.238  0  0.312  0.036  0.256  0.317  0.469  0.091  0.412  0.472  0.305  0.666  0.196  0.237  0.11  0.459  0.144  0  0.347  0.26  0.189  0.202  0.107  0.103  0.284  0.122  0.249  0.045  0.38  
 
 SLC25A20  788  3  48894355  48936426  3p21.31  1.712  1.428  1.061  1.832  2.718  1.272  3.047  1.382  2.385  1.762  1.695  1.83  2.67  2.105  1.203  1.121  1.636  1.009  3.969  2.176  1.603  2.439  2.453  2.681  1.164  1.159  2.465  1.452  1.409  1.828  1.765  2.183  1.693  1.016  1.026  1.679  1.627  0.983  0.844  1.982  0.926  1.802  2.69  0.767  1.077  1.39  2.553  1.03  0.744  2.165  1.929  1.047  1.372  0.75  1.587  0.893  0.878  1.646  0.761  2.097  
 
 ARIH2OS  646450  3  48955220  48956818  3p21.31  0.171  0.135  0.518  0.719  0.42  0.592  0.311  0.249  1.064  0.374  0.208  0.236  0.396  0.379  0.225  0.223  0.507  0.145  1.088  0.266  0.413  0.511  0.957  0.471  0.204  0.302  0.362  0.461  0.24  0.219  0.36  0.618  0.464  0.525  0.124  0.332  0.205  0.201  0.282  0.244  0.4  1.152  0.14  0.327  0.408  0.224  0.148  0.206  0.124  0.651  0.172  0.858  0.141  0.283  0.315  0.094  0.088  0.176  0.148  0.194  
 
 ARIH2  10425  3  48956280  49022971  3p21  2.844  2.919  2.466  3.47  2.601  2.283  4.06  3.148  3.809  3.161  3.64  2.184  3.58  3.751  3.511  2.579  2.75  1.943  4.659  2.565  2.825  2.905  2.91  2.859  3.372  3.324  3.156  4.158  2.962  3.704  3.609  3.963  3.42  1.962  2.291  3.108  3.997  3.571  3.326  3.697  2.683  2.707  4.243  2.459  2.726  2.675  2.045  3.165  2.145  4.09  3.503  1.915  3.321  1.593  2.666  2.065  2.683  3.104  3.044  3.602  
 
 P4HTM  54681  3  49027340  49044581  3p21.31|3p21.3  2.92  0.621  1.263  2.278  2.886  1.343  4.237  1.934  4.043  2.828  2.082  1.845  3.671  3.303  3.679  2.45  3.303  0.472  2.287  1.333  0  1.114  2.957  2.77  0.253  0.958  0.497  0.6  0.645  0.265  0.116  0  0.066  0.37  1.452  2.904  2.319  3.082  3.322  2.11  2.723  3.772  3.229  1.17  1.77  1.551  2.537  2.363  0.931  3.652  2.579  2.128  2.001  1.299  2.767  1.2  1.299  2.643  1.595  2.007  
 
 WDR6  11180  3  49044636  49053386  3p21.31  4.473  2.626  3.031  3.566  3.589  2.883  4.368  4.112  3.826  3.675  4.235  2.488  4.166  4.177  5.118  3.351  3.364  1.358  5.37  3.368  3.05  4.1  2.502  3.297  2.856  3.752  4.04  4.585  3.807  3.061  3.558  1.493  3.73  1.574  2.763  3.728  4.74  4.077  2.885  3.93  3.693  3.353  4.791  3.488  3.094  3.25  4.291  4.384  2.454  5.148  4.185  3.415  4.232  1.876  3.709  3.414  3.091  3.71  4.242  4.647  
 
 DALRD3  55152  3  49052920  49058504  3p21.31  2.532  0.904  1.946  2.556  3.088  1.319  3.077  1.718  3.131  2.071  2.497  1.542  3.194  3.125  2.856  2.231  2.126  0.509  3.619  1.413  1.432  1.532  1.873  2.368  2.063  2.524  2.653  2.492  2.236  3.125  3.094  2.851  2.288  1.493  1.903  2.961  2.53  2.666  2.733  2.436  2.546  2.652  3.791  1.637  2.185  1.795  1.411  2.482  2.022  4.192  2.654  1.947  2.359  1.29  2.957  1.99  2.075  3.391  3.079  2.589  
 
 NDUFAF3  25915  3  49057907  49060926  3p21.31  4.618  4.7  5.197  5.361  5.097  4.733  5.613  4.902  5.079  4.676  4.7  4.455  4.891  5.534  4.637  4.518  4.672  3.236  7.313  6.587  3.44  6.349  5.006  5.346  4.803  4.687  4.546  4.456  4.815  5.305  5.59  5.331  4.455  3.753  4.242  4.875  4.532  4.481  6.169  4.468  5.291  5.407  5.374  3.814  3.712  4.899  5.293  4.477  3.854  6.389  5.165  4.252  5.321  3.779  5.597  4.368  3.701  6.407  4.556  5.283  
 
 IMPDH2  3615  3  49061761  49066875  3p21.2  5.98  5.384  4.72  4.747  5.46  4.921  6.405  5.744  4.778  4.896  5.601  4.784  6.413  7.069  5.356  4.926  6.258  4.716  7.583  6.246  5.522  5.327  5.058  5.358  5.457  4.836  5.712  5.438  4.879  6.085  6.028  6.347  5.49  4.247  4.495  5.311  6.117  5.945  5.246  5.609  5.451  5.511  6.256  5.668  3.94  4.709  5.587  5.868  4.165  7.941  5.911  4.118  5.537  2.993  5.239  5.033  4.549  6.114  5.342  6.358  
 
 QRICH1  54870  3  49067141  49131504  3p21.31  3.884  3.797  2.91  3.286  3.433  3.085  3.963  4.246  3.075  3.5  4.1  2.681  4.009  4.659  4.362  3.094  3.308  2.608  4.147  3.371  3.272  4.211  2.284  3.375  3.523  3.486  3.383  3.817  3.602  3.188  3.302  3.486  3.326  1.412  2.874  3.485  4.268  3.753  3.524  4.437  3.785  3.004  4.221  3.765  3.107  3.03  2.448  3.931  3.356  3.959  3.754  2.974  4.291  2.7  3.766  3.162  3.506  4.037  3.884  4.437  
 
 QARS  5859  3  49133364  49142562  3p21.31  4.014  4.147  2.844  3.166  4.772  2.914  4.627  4.771  3.437  4.049  4.627  4.166  4.77  4.836  4.413  3.638  4.445  2.764  6.02  5.129  4.136  4.147  2.938  4.187  4.411  4.385  4.856  4.895  4.381  5.743  4.96  4.976  5.109  2.855  3.079  4.344  4.721  4.283  4.062  4.083  4.193  3.227  4.735  4.157  3.881  3.964  3.44  4.326  2.987  5.772  4.521  3.05  4.106  2.604  4.282  3.654  3.77  4.464  4.316  4.882  
 
 USP19  10869  3  49145478  49158371  3p21.31  2.382  0.786  1.656  1.96  2.048  1.477  2.74  2.474  2.613  2.234  2.53  1.242  2.882  2.763  2.922  1.81  2.016  0.564  3.438  2.211  2.225  2.386  1.588  2.229  2.051  2.504  2.644  3.594  2.666  3.345  2.618  3.199  3.361  1.407  1.495  2.679  3.1  2.52  2.146  3.187  2.07  2.137  2.44  2.092  2.247  1.823  0.966  2.371  1.346  2.955  2.587  1.589  2.091  1.248  2.068  1.578  1.905  2.421  2.165  2.751  
 
 LAMB2  3913  3  49158546  49170599  3p21  2.705  1.221  3.827  3.499  4.204  2.33  3.498  5.016  4.304  4.995  3.265  0.592  2.541  2.491  3.629  1.565  1.987  0.124  0.317  0.45  0.74  0.482  0.52  0.148  1.667  4.228  4.301  4.27  4.052  1.884  3.553  3.105  3.631  1.201  1.637  3.925  3.543  3.395  2.55  4.111  3.549  3.172  4.414  2.857  2.383  3.008  3.461  3.866  2.193  4.548  3.413  2.148  2.953  2.427  2.81  2.596  3.685  4.147  2.864  4.113  
 
 LAMB2P1  22973  3  49190291  49191834  3p21.3-p21.2  0.612  0  0.246  0.635  0.433  0.315  0.571  0.343  0.689  0.599  0.196  0  0.411  0.756  0.458  0.107  0.512  0.197  0.545  0.36  0  0.724  0.636  0  0  0.28  0.599  0.152  0.08  0.586  0.088  0.084  0.142  0.28  0.127  0.176  0.297  0.619  0.299  0.298  0.476  0.545  0.542  0  0.105  0.155  0.147  0.123  0.141  0.217  0.426  0.526  0.067  0.113  0.371  0.175  0.077  0.167  0.116  0.185  
 
 CCDC71  64925  3  49199967  49203785  3p21.31  2.607  0.395  1.744  2.361  2.051  1.836  3.544  2.568  1.965  2.357  2.571  1.271  2.037  2.759  2.866  1.568  1.635  0.241  3.377  2.002  2.211  2.708  0.838  1.412  1.912  2.049  3.179  2.519  2.402  2.845  1.857  1.928  2.415  0.655  1.418  2.055  2.827  1.831  2.074  3.006  2.311  2.273  2.991  2.434  1.853  1.281  2.496  2.296  1.9  2.995  2.35  1.715  2.606  1.067  2.585  1.929  1.616  2.542  1.988  3.157  
 
 KLHDC8B  200942  3  49209017  49213919  3p21.31  1.437  0.728  1.642  1.598  1.592  0.297  2.014  1.537  2.117  1.883  1.706  0.191  1.205  2.206  0.375  0.5  0.204  0.208  0.543  0.409  2.64  0.742  0.589  0.202  0.658  2.249  2.002  1.696  1.762  1.651  2.469  1.881  2.275  1.093  0.653  1.79  2.287  2.54  1.422  1.447  0.29  1.421  1.919  1.142  1.205  1.293  1.117  1.761  1.108  3.283  2.438  0.87  0.529  1.373  0.905  0.703  1.315  1.493  1.506  2.029  
 
 C3orf84  646498  3  49215068  49229291  3p21.31  0  0  0  0  0  0  0  0  0  0.106  0  0  0  0  0  0  0  0  0  0  0  0.098  0.366  0  0  0  0  0  0  0.077  0  0  0  0.268  0  0  0  0  0  0  0  0.096  0  0  0  0  0  0  0  0  0  0.317  0  0  0  0  0  0  0  0  
 
 CCDC36  339834  3  49235860  49295537  3p21.31  0  0  0.153  0  0.038  0  0.048  0.055  0.186  0.078  0.06  0.04  0  0.063  0  0.045  0  0.043  0.034  0.077  0  0.099  0.386  0  0.142  0.701  0.942  0.811  1.462  0.07  1.336  0.169  0.076  0.155  0  0  0  0.095  0.093  0  0  0.427  0.039  0  0  0  0.037  0.093  0.231  0.043  0.046  0.198  0  0.736  0.043  0  0  0.149  0  0  
 
 C3orf62  375341  3  49306029  49314508  3p21.31  0.558  0.601  0.31  0.448  0.666  0.237  0.684  0.3  1.157  0.745  0.622  0.225  0.764  1.003  0.682  0.586  0.671  0.56  1.804  0.621  0.469  1.169  1.035  0.473  0.218  1.009  1.278  0.457  0.767  0.366  0.653  0.446  0.839  0.648  0.286  0.36  0.484  0.393  1.1  0.657  0.353  0.591  1.021  0.502  0.612  0.359  0.091  0.717  0.286  1.203  0.402  0.553  0.631  0.524  0.585  0.384  0.229  0.687  0.763  0.559  
 
 USP4  7375  3  49314576  49377536  3p21.3  1.748  2.357  2.089  2.271  1.778  2.064  2.594  2.655  2.449  2.195  2.222  1.566  2.437  2.087  2.292  1.926  2.211  1.247  3.603  3.469  1.737  2.482  1.417  1.885  2.366  2.376  2.373  2.708  2.396  2.067  2.816  2.885  2.54  1.311  1.205  2.304  2.698  2.641  2.714  2.144  1.783  1.727  2.024  1.763  1.746  2.239  2.15  2.153  1.482  2.665  3.038  1.302  1.882  0.965  2.037  1.584  2.276  2.817  1.855  2.832  
 
 GPX1  2876  3  49394608  49395791  3p21.3  0.33  6.669  7.144  7.354  0.188  7.418  8.042  6.686  7.381  6.926  6.711  5.903  5.626  7.79  5.33  6.741  7.358  4.995  7.502  7.242  0.63  5.724  6.457  4.953  6.18  7.229  7.253  5.27  7.211  3.561  7.365  7.524  6.385  5.842  5.419  6.591  6.929  7.546  8.565  0.184  3.192  7.826  7.602  5.883  0.181  5.78  5.568  7.134  5.535  8.92  6.385  5.294  6.957  6.277  7.914  6.705  5.323  6.527  7.033  7.496  
 
 RHOA  387  3  49396578  49449526  3p21.3  5.74  7.383  6.57  6.698  6.05  5.866  6.947  6.703  6.836  6.79  6.696  5.334  6.349  7.831  6.115  6.033  6.802  5.983  8.17  7.669  5.266  6.465  6.166  5.379  6.204  6.549  5.763  5.665  5.691  5.61  6.086  6.021  5.616  4.847  5.49  5.954  6.603  6.171  7.736  6.013  5.286  6.563  6.418  5.808  5.426  4.973  5.858  6.523  5.677  8.367  6.713  4.92  6.409  5.153  6.632  6.057  6.107  6.977  6.716  7.287  
 
 TCTA  6988  3  49449638  49453909  3p21  1.719  1.871  2.736  2.147  3.384  2.262  3.318  2.491  2.63  3.517  2.75  2.387  2.207  2.547  2.035  2.307  1.892  0.379  3.378  2.282  1.264  1.958  3.195  2.643  1.805  3.373  2.92  2.091  3.291  1.914  3.497  3.691  4.009  3.077  1.199  2.704  2.039  3.962  2.611  2.454  2.291  2.899  3.774  1.8  1.613  2.766  2.108  1.937  1.741  3.651  2.634  1.9  1.645  1.863  1.468  1.983  2.217  1.335  2.066  2.733  
 
 AMT  275  3  49454210  49460111  3p21.2-p21.1  1.229  0.462  0.879  0.79  1.594  0.286  1.976  1.258  0.68  2.068  1.552  1.039  0.793  2.286  1.335  1.53  2.105  0.165  2.625  2.225  0.381  2.228  1.021  0.441  0.192  1.439  2.564  0.741  1.5  0.913  0.841  0.549  1.155  0.515  1.163  0.756  1.353  2.14  3.071  0.804  0.722  0.881  2.841  0.501  0.581  0.739  1.993  1.281  0.487  1.875  0.823  1.034  0.982  1.036  2.596  1.321  1.198  0.791  1.039  0.563  
 
 NICN1  84276  3  49459765  49466757  3p21.31  1.003  1.13  0.893  0.857  1.435  0.62  0.716  0.925  1.296  1.047  0.892  0.468  0.496  0.841  0.805  0.663  0.537  0.343  2.667  1.251  0.56  1.52  1.271  1.008  0.824  1.199  1.142  0.947  1.182  0.846  1.141  0.506  1.36  1.11  0.794  1.438  1.253  1.66  0.684  0.636  0.53  1.179  1.853  0.528  0.466  0.472  1.012  0.866  0.499  1.02  0.64  0.589  1.162  0.552  1.256  0.776  0.507  1.161  0.738  1.54  
 
 DAG1  1605  3  49506135  49573051  3p21  4.847  3.259  4.171  4.714  5.785  3.738  5.211  4.442  5.397  6.156  5.475  3.241  4.809  4.902  5.436  4.129  4.182  1.365  2.413  1.998  2.08  2.548  1.923  2.703  3.37  6.094  5.624  5.309  5.515  4.657  3.884  4.891  5.3  3.268  3.637  5.324  5.459  5.246  4.507  5.389  5.414  3.975  4.757  5.068  4.224  4.169  3.725  4.771  4.4  4.117  4.571  3.549  4.206  3.644  4.318  4.553  4.039  5.368  4.58  5.329  
 
 BSN-AS2  100132677  3  49586738  49591799  3p21.31  0  0  0.257  0.16  0.051  0  0  0  0.14  0.034  0.031  0  0  0.068  0  0.028  0  0  0.162  0.061  0  0.116  0.3  0  0  0  0.08  0  0.059  0.059  0.071  0.122  0  0.316  0.077  0.071  0  0.073  0.106  0  0  0.15  0.742  0  0  0.084  0.043  0  0  0.082  0.071  0.196  0.034  0.059  0.043  0.094  0  0  0  0.045  
 
 BSN  8927  3  49591921  49708982  3p21.31  0.353  0.035  0.229  0.151  0.102  0.109  0.098  0.218  0.278  0.259  0.105  0.068  0.157  0.386  0.269  0.064  0.073  0.175  0.646  0.135  0.536  0.443  0.517  0.057  0.395  0.217  0.295  0.375  0.609  0.203  0.412  0.626  0.244  0.276  0.143  0.227  0.139  0.248  0.302  0.903  0.238  0.371  0.929  0.105  0.145  0.189  0.108  0.218  0.223  0.087  0.305  0.762  0.187  0.189  0.226  0.112  0.208  0.136  0.204  0.184  
 
 APEH  327  3  49711434  49720934  3p21.31  3.904  0.471  2.636  3.038  4.141  2.193  4.417  3.696  3.242  3.582  3.672  3.772  4.495  4.886  4.267  3.727  4.224  0.072  5.579  4.424  3.402  3.748  2.664  3.914  3.858  4.134  4.391  4.881  4.142  5.268  4.511  4.745  4.861  2.689  2.788  4.253  4.412  4.148  3.547  4.065  3.905  3.159  4.264  3.488  3.585  3.715  4.428  3.934  3.35  5.569  4.137  2.885  3.527  2.274  4.071  3.272  3.248  3.967  3.981  4.973  
 
 MST1  4485  3  49721379  49726196  3p21  0.827  0.085  0.265  0.271  0.961  0.289  0.36  0.467  0.636  0.505  0.419  0.085  0.748  0.514  0.883  0.245  0.191  0.072  1.813  0.239  0.15  1.067  0.841  0.302  0.13  0.913  0.788  0.789  0.496  0.245  0.793  0.311  0.697  0.31  0.232  0.675  0.359  0.506  0.579  0.569  0.466  0.34  0.566  0.372  0  0.476  0.355  0.487  0.1  1.19  0.554  0.511  0.321  0.193  0.859  0.271  0.521  0.268  0.287  0.431  
 
 RNF123  63891  3  49726949  49758962  3p24.3  1.599  0.63  1.222  1.124  1.98  1.257  2.676  2.419  2.024  2.17  1.968  1.437  2.404  2.162  2.831  1.465  2.249  0.172  3.215  2.131  2.213  2.361  1.114  1.931  1.78  2.365  2.499  3.128  2.481  2.92  2.415  2.752  3.042  0.937  1.188  1.71  2.823  2.328  1.911  2.525  2.228  1.369  1.989  1.516  1.989  1.883  2.266  2.523  1.279  3.342  2.412  1.378  1.849  0.798  1.869  1.274  1.543  2.63  2.077  2.647  
 
 AMIGO3  386724  3  49754266  49757238  3p21  0.847  0.147  1.074  0.701  1.595  0.861  1.984  2.567  1.951  2.042  2.273  0.415  1.095  0.638  1.541  0.668  0.794  0.246  2.64  0.49  0.882  1.674  1.27  1.151  0.234  0.972  2.13  1.275  1.221  0.61  0.824  1.03  1.78  0.794  0.443  1.517  2.268  0.844  0.689  1.204  1.318  1.445  1.026  0.657  1.068  0.777  0.24  1.426  0.353  1.53  0.945  1.775  2.068  0.868  0.647  0.339  1.301  1.099  0.989  1.052  
 
 GMPPB  29925  3  49758908  49761407  3p21.31  2.809  0.65  2.716  3.001  2.48  2.831  3.921  3.12  2.463  2.61  2.944  2.412  3.543  2.798  3.198  2.432  2.819  0.198  4.546  1.961  1.357  3.014  2.474  2.748  2.596  3.06  2.731  3.116  3.627  3.523  3.977  3.784  3.076  0.981  1.765  3.105  3.514  2.637  2.414  2.928  2.849  3.237  2.212  2.36  2.47  2.83  3.125  3.078  1.945  4.439  3.342  2.11  2.873  1.475  2.763  2.266  1.978  3.435  2.243  3.172  
 
 IP6K1  9807  3  49761727  49823973  3p21.31  1.834  0.607  2.037  2.955  1.835  1.915  3.083  2.924  3.572  2.742  3.102  1.481  2.781  1.78  2.998  2.042  1.83  0.256  3.422  1.472  1.891  2.257  2.231  2.41  2.383  2.874  3.024  2.911  2.713  3.043  2.792  2.939  3.216  1.572  1.387  2.568  3.46  2.884  2.175  3.114  2.309  2.4  2.813  1.823  2.327  1.935  0.976  3.016  1.767  3.547  2.923  1.816  2.585  1.28  1.895  1.856  2.283  2.429  2.537  3.113  
 
 CDHR4  389118  3  49828166  49837268  3p21.31  0  0  0.063  0  0.142  0  0  0.035  0.105  0.053  0.048  0  0  0.083  0  0.062  0  0  0  0  0  0.089  0.279  0.049  0  0  0.114  0.041  0  0.09  0  0  0  0.07  0.213  0.072  0  0.059  0.059  0  0  0.31  0.044  0  0  0.055  0.028  0.112  0  0.114  0.042  0.339  0  0.094  0.091  0.062  0  0  0.048  0  
 
 FAM212A  389119  3  49840686  49842463  3p21.31  0  0.205  1.71  0.497  0.279  0.211  1.585  1.627  0.965  1.284  0.638  0.114  0.175  0.951  0.319  0.311  0  0  2.551  2.121  1.366  0.534  1.007  0.648  0.346  1.813  1.271  1.225  1.132  0.918  3.019  2.489  1.679  1.543  0.247  0.603  0.433  1.043  0.477  0.539  0.174  0.824  1.234  0  0.322  0.152  0  0.89  0.203  1.373  1.442  0.706  0.347  0.531  0.248  0.236  0.516  0.836  0.486  0.223  
 
 UBA7  7318  3  49842637  49851391  3p21  0.114  0.409  0.882  0.848  0.364  0.155  2.958  2.262  0.248  2.662  0.748  0.241  1.187  0.261  0.55  1.931  0.086  0.072  3.964  1.908  0  3.335  0.385  0.872  0.251  1.705  2.286  1.964  1.492  0.501  0.281  1.411  2.489  0.995  0.101  0.07  0.706  1.245  2.742  0.215  0.098  0.597  0.05  0.052  0.282  0.51  0.891  0.199  0.312  0.916  0.658  0.277  1.271  1.572  1.708  1.728  2.578  1.965  0.257  2.055  
 
 TRAIP  10293  3  49866027  49893992  3p21.31  2.601  1.436  1.501  1.855  2.194  1.728  3.256  1.737  0.933  2.25  2.706  1.535  2.014  2.939  2.458  1.757  2.455  0.795  4.331  2.813  1.544  3.12  1.88  1.83  1.42  1.889  2.828  2.693  2.146  2.555  2.154  2.288  3.29  1.531  1.609  1.741  2.385  2.665  2.318  2.748  1.859  1.915  3.626  1.607  1.982  2.057  3.512  2.441  1.292  3.471  2.415  1.263  2.358  1.102  2.75  1.072  2.219  2.369  2.636  2.349  
 
 CAMKV  79012  3  49895421  49907369  3p21.31  0  0.025  0.202  0  0.031  0  0.046  0.044  0.237  0.062  0.044  0.039  0.086  0.021  0  0  0.057  0.142  2.714  0.238  0.157  1.454  0.406  0.286  0  0  0.097  0.02  0.075  0.746  0.058  0.198  0  0.24  0.074  0.182  0.05  0  0.053  0.392  0  0.363  0.703  0.121  0.098  0.057  0.034  0  0  0  0.073  0.395  0  0.07  0.068  0  0  0.076  0  0  
 
 MST1R  4486  3  49924435  49941306  3p21.3  0.279  0.402  0.132  0.754  2.274  0.031  0.161  0.056  0.161  0.051  0.026  2.165  3.719  2.264  3.219  2.975  2.491  0.8  0.344  0.089  0.569  0.215  0.533  0.13  0.037  0.074  0.157  0.113  0.046  0.097  0  0  0.077  0.245  0.916  2.543  0.347  1.26  0.498  0.439  3.81  0.237  0.039  1.921  0.616  1.133  3.07  1.038  2.35  2.905  3.057  2.069  0.025  0.865  0.681  0.11  0.069  0  0.94  1.29  
 
 MON1A  84315  3  49946301  49967445  3p21.31  1.705  0.467  1.867  2.337  1.93  1.339  3.178  1.418  2.19  1.903  1.777  1.786  2.71  2.041  2.008  1.496  1.325  0.132  3.049  1.669  1.93  2.016  1.783  2.262  2.022  1.776  2.707  2.152  1.805  2.529  1.819  2.251  2.706  1.338  0.881  1.478  2.085  2.126  1.488  1.577  1.496  2.022  2.469  1.235  1.719  1.65  1.618  2.274  0.612  2.643  2.048  1.557  1.461  0.89  1.196  1.205  1.101  1.559  1.496  1.785  
 
 RBM6  10180  3  49977476  50114685  3p21.3  2.328  3.215  1.852  1.963  2.689  1.934  2.748  2.321  2.777  2.622  2.877  1.436  2.683  3.404  2.788  2.066  2.263  2.746  4.382  2.624  2.352  3.154  1.826  2.358  2.336  2.425  1.952  2.184  2.054  1.978  2.564  2.727  2.771  1.358  1.679  2.411  2.809  2.571  2.149  2.454  2.022  2.154  2.78  2.104  2.182  1.529  1.692  2.776  2.008  3.872  3.038  1.464  2.433  1.108  2.737  1.749  2.081  2.544  2.43  2.822  
 
 RBM5  10181  3  50126340  50156397  3p21.3  2.908  2.969  2.226  2.182  2.698  2.343  3.686  2.587  3.003  3.328  3.141  1.741  3.027  3.508  3.273  2.373  2.633  2.166  4.748  3.27  2.35  3.641  2.134  2.23  2.272  3.117  3.248  2.706  2.959  3.123  3.217  3.259  2.79  1.339  1.98  2.806  3.294  3.031  2.34  2.878  2.207  2.379  3.663  2.719  2.407  2.08  4.201  3.292  2.192  4.535  3.168  1.697  3.198  1.602  3.234  2.009  2.849  2.873  3.013  3.14  
 
 RBM5-AS1  100775107  3  50137035  50138421  3p21.3  1.221  0.445  0.82  0.801  0.782  0.948  0.413  0.759  1.305  1.426  1.039  0.151  0.954  0.872  1.742  0.695  0.756  0.182  0.835  0.773  0.847  0.899  0.476  0.656  0.395  1.488  0.604  0.813  0.953  1.183  0.851  0.757  0.846  0.409  0.453  1.138  1.093  1.318  0.502  0.804  0.822  0.506  0.633  1.079  0.46  0.837  0.14  1.316  0.77  0.504  1.553  0.492  1.353  0.389  0.982  0.514  1.298  1.088  0.92  1.041  
 
 SEMA3F  6405  3  50192847  50226508  3p21.3  3.918  0  0.48  3.703  3.42  0.178  1.289  2.026  0.48  2.703  2.414  0.059  1.704  1.399  3.001  1.848  0.241  0.114  0.133  0.163  0.546  0.299  0.37  0.115  0.108  0.328  0.384  0.607  0.11  0.91  0.309  0.473  0.043  0.241  0.59  3.002  3.343  1.461  0.997  0.694  1.499  1.353  1.624  1.443  3.302  1.859  0.833  1.361  1.869  1.581  3.646  0.84  1.578  1.344  2.41  1.804  2.167  2.011  3.657  2.159  
 
 GNAT1  2779  3  50229042  50235129  3p21  0  0  0.167  0.138  0.083  0.04  0.075  0.059  0.279  0.129  0.104  0.122  0  0.219  0.069  0.067  0.051  0.193  0.053  0.138  0  0.292  0.357  0.06  0.053  0.071  0.246  0.031  0.075  0.132  0  0.073  0  0.242  0.067  0  0.033  0  0.088  0  0  0.815  0.083  0  0  0  0.055  0  0  0.037  0.068  0.688  0.063  0.172  0.151  0.082  0.057  0  0.055  0  
 
 SLC38A3  10991  3  50242678  50258411  3p21.3  0.681  0  0.079  0  1.325  0.158  0.161  0.835  4.825  1.33  3.079  0  0.066  1.208  0  0  0.067  0.185  0.102  0.061  0  0.348  0.392  0.051  0  0.155  0.108  0.075  0  0.1  0.194  0.053  0  0.151  0  0  0.249  0  0.056  0.265  0  1.3  0.662  0.247  0.703  0.345  0.058  0.731  0  1.181  0.057  0.552  0  0.128  0.074  0  0  0.62  0  0  
 
 GNAI2  2771  3  50264119  50296786  3p21.31  4.84  4.686  6.37  5.733  4.854  4.878  6.962  6.615  6.985  6.845  6.627  3.652  4.689  5.675  4.674  5.155  4.195  2.601  7.187  6.116  3.549  6.114  4.827  5.213  6.211  6.122  6.219  6.502  5.762  4.692  5.404  5.966  6.388  4.741  3.473  4.815  6.93  6.627  5.168  5.2  3.783  5.789  5.183  3.939  4.826  4.852  4.7  6.447  4.729  6.955  6.829  5.241  6.588  4.741  5.784  5.371  6.005  6.813  5.634  6.961  
 
 SEMA3B-AS1  101928931  3  50304072  50304803  -  0  0  0  0  0.95  0.538  0  0  0  0.293  0  0  0  0.265  0  0  0  0.661  0  0  0  0  0  0  0  0  0  0.542  0  0  0  0  0  0  0.474  0  0  0.608  0  0  0  0  0  1.303  0  0.33  0  0  0  0  0  1.039  0  0.315  0  0  0  0  0  0  
 
 SEMA3B  7869  3  50304989  50314602  3p21.3  1.51  0.792  1.498  0.355  1.314  0.218  1.616  1.375  2.455  5.427  1.397  0.086  3.915  2.072  3.148  3.036  2.304  0.442  0.113  0.225  0.137  0.469  0.542  0.221  0.137  4.722  1.209  2.251  0.566  0.649  2.248  0.954  0.595  0.389  1.881  1.536  1.858  3.103  1.267  0.333  0.349  3.654  2.281  2.007  0.881  1.951  3.317  1.346  1.19  2.946  1.744  1.455  0.393  0.41  1.589  1.303  0.56  0.882  0.46  1.006  
 
 LSMEM2  132228  3  50316457  50325545  3p21.31  0.491  2.213  0.512  0.652  0.61  0  0.499  0.384  0.432  0.782  0.405  0.347  0.52  0.743  0.626  0.44  0.456  1.436  0.176  4.397  0.487  2.825  1.265  0.904  0.21  0.377  0.524  0.448  0.431  0.235  0.355  0.385  0.633  0.55  0.236  0.185  0.487  0.491  3.265  0.278  0.586  0.834  0.109  0.225  0.551  0.532  3.555  0.687  0.198  0.17  0.286  0.666  0.601  0.162  0.371  0.243  0.403  0.879  0.779  0.329  
 
 IFRD2  7866  3  50325162  50330026  3p21.3  3.884  1.599  3.594  4.126  3.765  3.387  5.332  3.539  4.022  3.905  4.301  3.473  4.917  4.651  4.304  3.885  3.953  0.459  5.618  3.139  3.616  3.978  3.932  4.53  4.277  3.274  4.59  4.127  3.667  4.667  4.13  5.071  5.108  3.134  3.359  4.327  5.263  4.487  2.311  4.506  4.427  4.916  4.79  3.915  3.9  3.475  2.043  4.573  3.757  5.833  4.199  3.762  4.391  2.464  3.838  3.677  3.286  5.281  4.438  4.419  
 
 HYAL3  8372  3  50330258  50336899  3p21.3  0  0.705  1.994  1.346  1.445  0.999  1.914  0.82  1.606  2.012  1.237  1.422  1.589  1.709  1.419  1.151  1.944  0.249  1.704  1.677  1.565  0.97  2.089  1.338  0.816  0.634  2.488  1.358  0.893  0.67  0.297  1.702  2.997  2.011  1.247  2.382  1.315  2.085  1.319  2.324  1.115  1.895  2.615  0.852  0.965  1.256  2.006  1.164  1.102  1.327  1.508  2.1  1.545  1.239  1.179  1.506  2.073  0.985  1.674  1.256  
 
 NAT6  24142  3  50333832  50336852  3p21.3  1.68  0.48  2.239  2.723  2.27  1.904  2.369  1.485  1.912  2.205  1.957  0.936  2.469  2.433  2.368  2.049  3.818  0  3.042  2.691  1.66  2.482  2.376  2.84  2.042  2.301  1.847  1.969  2.124  2.165  2.519  3.127  2.984  2.031  1.43  2.367  1.664  2.152  0.841  2.035  1.387  2.652  2.21  1.98  0.66  1.366  2.003  1.885  2.209  3.244  2.849  1.652  2.171  1.124  2.238  1.468  1.773  1.907  2.502  2.396  
 
 HYAL1  3373  3  50337319  50349812  3p21.3-p21.2  0.463  0.515  0.622  0.387  1.373  0.177  0.274  0.287  0.376  0.594  0.273  1.53  3.449  1.969  0.476  0.176  1.559  0.2  0.114  0.174  1.062  0.205  0.734  0.164  0  1.795  0.528  0.637  0.161  3.539  0.442  0.364  1.009  0.691  0.6  2.044  0.355  0.918  0.23  0.229  0.229  1.093  0.95  2.267  0.141  0.168  4.776  0.164  0.181  0.097  0.454  0.515  0.247  0.839  1.221  0.273  0.15  0.27  0.196  0.126  
 
 HYAL2  8692  3  50355220  50360281  3p21.3  3.057  0.598  3  4.232  3.47  2.298  4.519  4.104  4.149  4.04  3.54  2.209  4.245  4.207  4.078  2.897  3.417  0.309  3.671  2.015  1.492  2.579  1.717  2.322  3.055  4.476  4.013  3.997  3.98  4.36  3.898  3.942  4.158  2.565  2.087  4.378  3.873  4.034  2.23  4.979  3.404  3.454  4.569  3.689  3.34  2.837  3.004  3.689  2.849  4.969  4.143  2.898  3.628  2.095  3.963  3.075  2.529  3.072  3.648  4.4  
 
 TUSC2  11334  3  50362339  50365669  3p21.3  2.884  2.52  2.245  2.974  3.452  2.946  3.705  2.26  3.621  2.475  3.413  2.58  3.449  3.961  2.559  2.856  3.092  1.51  4.77  3.101  2.26  3.501  2.742  3.191  3.013  2.792  2.071  3.127  2.527  2.72  3.141  3.045  3.12  2.205  2.484  2.774  3.192  3.229  3.242  2.769  2.589  2.72  3.884  2.284  3.181  2.616  2.361  2.715  2.155  4.046  2.88  2.063  2.798  1.905  3.297  2.369  2.152  3.705  3.445  3.457  
 
 RASSF1  11186  3  50367216  50378367  3p21.3  1.841  0.97  3.034  3.281  2.463  2.791  3.851  3.082  1.89  3.203  3.368  1.711  2.798  2.892  2.682  2.318  3.549  0.22  5.026  3.276  2.48  2.698  2.899  2.769  2.43  2.407  4.444  2.402  2.205  2.58  2.271  2.604  2.954  1.401  1.423  2.676  3.325  3.846  1.84  3.104  2.111  2.497  3.786  1.735  1.922  2.232  3.404  3.613  2.168  4.62  3.282  2.26  3.331  1.753  2.351  2.335  2.767  2.94  2.643  3.058  
 
 RASSF1-AS1  102060282  3  50374941  50375727  3p21.3  0.289  0.197  0.369  0.423  0.392  0.849  0.163  0.096  0.43  0.531  0.165  0.153  0.473  0.367  0  0.226  0.362  0.147  1.341  0.547  0.698  0.565  1.228  0.325  0.23  0.33  0.993  0.308  0.793  0.856  1.212  0.368  0.684  0.791  0.322  0.585  0.294  0.49  0.182  0.246  0.338  0.831  2.309  0.738  0.218  0.25  0  0.717  0  1.143  0  0.972  0.281  0.345  0.399  0.224  0  0.661  0.192  0  
 
 ZMYND10  51364  3  50378536  50383177  3p21.3  0.36  0  0.365  1.291  1.533  0.126  0.654  0.074  0.352  0.616  0.356  0.112  0.208  0.622  0.514  0.138  0.301  0.099  1.1  0.187  0  0.54  0.491  0  0  0.171  0.553  0.278  0.469  0.135  0.125  0.16  0.36  0.296  0.14  0.543  0.191  0.3  0.06  0.522  0.146  0.376  1.146  0.106  0.301  0.188  0.043  0.569  0.228  0.741  0.416  0.827  0.153  0.225  0.489  0.442  0.065  0.426  0.124  0.156  
 
 NPRL2  10641  3  50384918  50388486  3p21.3  2.631  2.331  1.598  2.125  3.341  1.412  3.886  2.13  2.411  2.402  2.692  2.228  2.93  3.728  2.378  2.141  2.763  1.219  4.61  3.708  1.863  3.471  2.47  3.012  2.126  2.253  2.842  2.368  2.215  2.807  2.527  2.816  3.185  1.548  1.738  2.218  2.411  2.676  0.861  2.163  2.389  2.125  3.547  2.173  2.105  2.151  3.801  2.511  1.75  3.971  2.936  1.663  1.885  1.519  2.598  2.265  1.594  2.539  2.385  2.514  
 
 CYB561D2  11068  3  50388125  50405628  3p21.3  2.784  0.952  2.5  3.735  3.184  2.522  5.041  3.272  3.447  3.335  3.528  2.19  3.341  2.604  2.624  2.126  1.992  0.696  4.256  2.831  1.241  2.714  3.278  3.481  2.68  3.304  4.088  3.252  3.265  3.552  3.324  3.742  4.147  3.275  2.085  2.655  2.632  3.372  2.113  2.692  2.224  3.447  3.482  2.18  1.997  2.803  3.176  3.176  2.362  4.464  3.363  2.129  3.323  2.912  2.397  3.094  2.387  2.836  2.529  3.179  
 
 TMEM115  11070  3  50392179  50396939  3p21.3  2.636  2.056  3.322  3.859  3.367  2.758  4.191  3.292  4.559  3.719  3.496  1.745  3.078  3.056  3.162  2.533  2.879  0.903  3.696  2.559  2.343  2.474  2.708  2.589  3.076  3.3  3.135  3.593  3.338  3.579  3.335  4.013  4.021  3.144  2.473  3.289  3.826  3.874  2.92  3.308  3.376  3.858  2.855  2.795  3.073  2.394  1.949  3.309  2.58  4.326  3.667  2.64  3.172  2.147  3.213  2.499  2.562  3.1  3.13  3.737  
 
 CACNA2D2  9254  3  50400043  50541675  3p21.3  0.449  0.157  0.142  0.157  0.427  0.048  0.229  0.214  0.207  0.281  0.132  0.748  0.354  1.274  0.332  0.265  0.27  0.083  0.272  0.415  0.16  0.414  0.451  0.235  0.111  0.685  0.477  0.146  0.153  0.551  0.212  0.082  0.204  0.169  0.277  0.26  0.139  0.08  0.267  0.66  0.084  0.168  1.624  0.661  0.226  0.278  0.167  0.09  0.362  0.153  0.251  0.832  0.286  0.389  0.286  0.133  0.134  0.176  0.158  0.105  
 
 C3orf18  51161  3  50595455  50608458  3p21.3  0.133  0.665  1.204  0  0.693  1.13  1.832  0.148  2.411  0.825  1.063  0.305  0.44  1.658  1.373  0.086  0.092  0  0.566  0.059  0.161  0.471  0.834  0.51  0.712  1.91  0.677  0.217  0.759  0.57  0.118  0.096  0.327  0.668  0.678  1.51  1.234  2.048  0.18  1.52  0.133  0.716  2.689  0.066  0.421  0.224  1.577  1.128  0.291  1.798  2.091  1.083  0.072  0.739  0.774  0.439  1.044  1.26  0.401  0.842  
 
 HEMK1  51409  3  50606908  50622421  3p21.3  0.751  0.265  0.954  0.778  1.036  0.761  1.454  1.094  1.007  1.638  1.067  0.814  0.811  1.146  1.385  0.623  0.918  0.121  2.146  0.987  1.013  1.619  0.828  1.141  0.684  1.287  1.605  1.848  1.53  0.843  1.453  1.282  1.719  0.643  0.638  0.899  1.589  1.438  0.667  0.803  0.868  1.165  0.494  1.185  0.638  0.672  0.603  0.911  0.864  1.669  1.655  0.713  1.477  0.644  0.949  0.701  1.435  1.063  0.754  1.32  
 
 CISH  1154  3  50643884  50649262  3p21.3  1.706  0.145  0.601  0.479  3.248  0  1.708  0.311  0.944  1.018  0.665  0.984  1.586  1.367  1.438  0.707  0.576  0.179  2.26  1.994  4.33  1.066  1.754  2.195  0.141  1.622  0.965  0.664  0.354  1.043  1.596  0.894  1.227  0.84  0.484  1.771  0.495  1.224  0.088  0.424  0.185  0.559  2.021  1.066  0.572  0.992  0.265  1.074  0.881  1.831  1.224  0.437  1.238  1.298  0.769  1.218  0.827  0.16  1.097  0.554  
 
 MAPKAPK3  7867  3  50649292  50686728  3p21.3  3.1  3.087  1.775  2.279  3.314  1.201  4.888  1.842  2.647  2.091  2.31  2.558  3.839  4.487  3.711  2.636  3.512  1.382  5.624  4.747  3.315  3.57  2.225  3.293  3.498  3.376  3.858  3.315  2.505  4.135  3.593  3.668  3.985  2.347  2.449  3.706  3.875  5.106  2.055  2.131  3.448  2.398  4.019  3.171  2.28  2.478  4.974  3.868  3.895  4.982  4.888  3.454  4.865  1.641  3.694  2.942  3.04  4.224  4.602  4.544  
 
 DOCK3  1795  3  50712671  51421629  3p21.2  0.115  0.274  0.122  0.063  0.16  0.024  0.409  0.097  0.691  0.209  0.406  0.063  0.1  0.833  0.175  0.106  0.107  0.047  0.205  0.121  0.03  0.207  0.263  0.044  0.063  0.275  0.148  1.122  0.088  0.213  0.095  0.137  0.222  0.235  0.234  0.544  0.326  0.36  0.372  0.63  1.043  0.602  0.628  0.119  0.336  0.089  0.239  0.695  1.064  1.096  0.533  0.479  0.202  0.217  0.125  0.071  0.095  0.695  0.115  0.738  
 
 MANF  7873  3  51422667  51426828  3p21.1  3.852  5.925  4.83  5.158  4.91  5.346  5.771  5.28  6.753  5.038  5.101  5.562  4.461  6.127  4.27  4.529  6.331  4.238  6.514  6.274  2.81  5.706  6.192  5.548  5.043  4.815  4.17  3.41  4.169  3.302  5.399  5.116  4.622  4.228  3.954  4.275  4.831  4.563  5.886  4.081  4.627  5.637  5.491  4.755  3.748  3.822  5.18  4.163  3.691  6.042  5.136  3.267  4.582  3.429  5.157  4.551  3.436  5.781  4.329  5.443  
 
 RBM15B  29890  3  51428698  51435336  3p21.2  2.897  1.417  2.338  3.05  2.676  1.741  3.454  2.774  3.739  2.786  3.237  1.93  3.416  2.991  3.464  2.636  2.598  0.825  4.218  2.01  2.052  3.496  2.29  2.537  2.536  2.689  2.875  2.788  2.439  3.311  2.74  2.792  3.273  1.851  1.715  2.806  3.112  2.651  2.119  3.142  2.507  2.787  3.781  2.722  2.36  2.607  2.059  2.737  2.718  3.091  2.967  2.26  3.028  1.564  2.682  2.438  2.357  2.806  2.953  2.749  
 
 VPRBP  9730  3  51433297  51534018  3p21.2  1.696  0.852  1.069  1.055  1.088  0.977  1.673  1.358  1.248  1.385  1.878  0.977  2.154  2.026  2.771  1.334  1.284  0.481  1.914  1.394  1.631  1.902  0.501  1.678  1.353  1.78  1.642  2.578  1.745  1.921  1.315  1.451  2.496  0.676  0.975  1.748  2.419  1.9  1.049  2.423  1.717  0.825  1.629  1.585  1.507  1.541  1.401  2  1.726  1.508  2.229  1.231  1.864  0.82  1.512  1.358  1.5  1.812  1.818  2.529  
 
 RAD54L2  23132  3  51575595  51697612  3p21.2  1.708  0.857  1.415  1.664  1.556  1.658  2.264  2.376  2.04  2.414  2.398  1.249  2.186  1.463  2.512  1.422  1.148  0.212  2.297  1.708  1.403  2.429  0.877  0.737  1.546  3.401  2.012  2.874  2.144  1.968  2.476  2.114  1.812  0.63  1.026  1.835  2.793  2.322  1.471  2.545  1.97  1.309  2.552  1.787  1.817  1.87  1.27  2.714  1.893  2.002  2.362  1.337  2.153  0.877  1.567  1.431  1.804  1.98  2.037  2.804  
 
 TEX264  51368  3  51705190  51738339  3p21.31  3.903  1.642  3.138  3.671  4.413  2.622  5.342  3.394  5.147  3.979  4.01  3.649  4.178  4.169  3.444  3.505  4.608  0.372  5.923  3.109  2.824  3.896  4.442  4.16  4.183  3.925  4.988  3.924  4.291  4.902  4.8  4.785  5.1  4.221  3.28  4.35  4.028  4.459  3.728  4.162  3.722  4.294  4.559  3.428  2.35  3.638  1.461  3.674  3.215  5.347  4.229  2.737  3.933  2.591  4.016  3.551  2.869  3.814  3.52  4.436  
 
 GRM2  2912  3  51741080  51752625  3p21.2  0  0.05  0.159  0.246  0.353  0.072  0.036  0.028  0.26  0.129  0.05  0  0  0.097  0  0.049  0  0.128  0.115  0.096  0  0.225  0.483  0.081  0.053  0.073  0.121  0.085  0.044  0.158  0  0.147  0  0.294  0  0.109  0  0  0.15  0.089  0.046  0.168  0.646  0.158  0.13  0.091  0.042  0  0  0.121  0.038  0.746  0.058  0.068  0.094  0  0  0  0.06  0  
 
 IQCF6  440956  3  51812576  51813203  3p21.2  0  0  0  0  0  0  0  0  0  0  0  0  0  0  0  0  0  0  0  0  0  0.249  0.616  0  0  0  0.456  0  0  0.213  0  0  0  0  0  0  0  0  0  0  0  0.317  0  0  0  0.218  0  0  0  0  0  0  0  0  0  0  0  0  0  0  
 
 IQCF4  100506840  3  51851618  51853651  -  0  0  0  0  0  0  0  0  0  0  0  0  0  0  0  0  0  0  0  0  0  0.216  0  0  0  0  0.096  0  0  0.121  0  0  0  0  0  0  0  0  0  0  0  0  0  0  0  0  0  0  0  0  0  0  0  0  0  0  0  0  0  0  
 
 IQCF3  401067  3  51860898  51864874  3p21.2  0  0  0  0  0  0  0  0  0  0  0  0  0  0  0  0  0  0.127  0  0  0  0.049  0.402  0  0  0  0.112  0  0  0.084  0  0  0  0.27  0  0  0  0  0  0  0  0.457  0.042  0  0  0  0.039  0  0  0  0  0.368  0  0.057  0  0  0  0  0  0  
 
 IQCF2  389123  3  51895644  51897440  3p21.2  0  0  0  0  0  0  0  0  0.231  0  0  0  0  0  0  0  0  0  0  0  0  0.363  0  0  0  0  0.165  0  0  0.123  0  0  0  0  0  0  0  0  0  0  0  0.153  0  0  0  0  0  0  0  0  0.16  0.603  0  0.131  0  0  0  0  0  0  
 
 IQCF5-AS1  100996916  3  51907611  51909783  3p21.2  0  0  0.162  0  0  0  0  0  0.201  0  0  0  0  0.041  0  0  0  0  0  0  0  0.029  0.174  0  0  0  0.073  0  0  0.068  0  0  0  0.238  0  0  0  0  0  0  0  0.296  0.047  0  0  0.041  0.036  0  0  0  0  0  0  0.037  0  0  0  0  0  0  
 
 IQCF5  389124  3  51907736  51909600  3p21.2  0  0  0  0  0  0  0  0  0  0  0  0  0  0  0  0  0  0  0  0  0  0.223  0  0  0  0  0  0  0  0  0  0  0  0  0  0  0  0  0  0  0  0  0  0  0  0  0  0  0  0  0  0.603  0  0  0  0  0  0  0  0  
 
 IQCF1  132141  3  51928891  51937386  3p21.2  0  0  0.233  0  0  0  0  0  0  0  0  0  0  0.173  0  0  0  0  0  0  0  0.209  0.351  0  0  0  0.089  0  0  0.138  0  0  0  0  0  0  0  0  0.095  0  0  0  0.063  0  0  0  0  0  0  0  0.221  0.345  0  0.085  0  0  0  0  0  0  
 
 RRP9  9136  3  51967441  51975957  3p21.2  2.111  0.571  1.971  2.081  1.514  2.47  4.115  2.367  2.197  2.188  2.739  1.56  3.335  2.823  3.011  2.566  2.442  0  4.379  2.439  2.93  2.374  1.561  2.983  3.166  2.299  3.153  3.355  2.729  3.107  3.462  4.029  3.629  1.429  2.161  2.4  4.172  3.103  2.17  3.918  2.524  2.476  3.552  3.156  2.045  2.895  1.004  3.031  1.709  4.287  3.633  1.588  3.012  1.716  2.11  1.935  1.752  3.011  3.07  3.542  
 
 PARP3  10039  3  51976320  51982883  3p21.31-p21.1  1.147  1.765  2.07  1.093  1.432  0.893  1.939  1.597  1.668  2.043  1.214  0.898  2.081  2.107  1.581  1.251  1.309  1.302  2.227  0.647  0.626  2.04  1.53  1.365  1.399  2.123  2.543  2.412  1.846  1.395  2.4  2.049  3.099  1.85  0.981  1.947  1.717  2.439  2.392  2.108  0.826  1.594  1.363  1.282  0.209  1.573  2.037  2.091  1.359  3.37  2.502  1.57  1.874  2.064  3.151  1.161  1.954  2.683  3.029  3.248  
 
 GPR62  118442  3  51989329  51991520  3p21.1  0  0.084  0.283  0.124  0.094  0.124  0.128  0.18  0.146  0.362  0.129  0.101  0  0.102  0  0.193  0.182  0.219  0.047  0.123  0.086  0.326  0.488  0  0.083  0.166  0.212  0.161  0.118  0.171  0.064  0.092  0  0.217  0.143  0.093  0.065  0  0.163  0.092  0  1.032  0.07  0.066  0.136  0.157  0.038  0.136  0  0.297  0.134  1.336  0.071  0.278  0.172  0.173  0  0  0  0  
 
 PCBP4  57060  3  51991469  52001482  3p21  1.394  0.434  3.703  3.951  3.065  2.645  3.511  4.003  3.606  5.204  2.916  0.928  2.278  2.52  2.727  1.829  2.366  0.111  2.602  0.169  0.854  0.696  0.875  1.166  3  4.959  2.897  4.117  4.194  2.517  4.247  5.037  4.207  2.773  1.935  3.533  2.498  3.897  2.73  3.636  2.028  2.423  4.499  3.229  2.346  4.009  1.23  3.738  2.513  5.039  3.403  1.865  2.375  1.916  2.522  1.917  3.129  3.659  3.384  4.083  
 
 ABHD14B  84836  3  52002525  52008646  3p21.2  3.032  1.407  2.58  3.106  4.624  1.78  4.431  2.707  2.795  2.99  2.91  3.512  4.355  4.409  3.525  3.432  3.995  1.001  4.28  3.847  3.651  2.114  4.685  4.914  3.899  4.486  4.749  4.621  4.421  4.883  4.307  4.501  5.203  4.384  2.946  3.6  3.271  4.414  3.229  3.1  2.974  3.986  4.818  2.626  2.249  3.632  2.819  3.73  3.2  5.018  3.989  3.366  3.901  3.126  4.505  3.718  2.898  3.691  4.053  3.832  
 
 ABHD14A  25864  3  52009041  52015216  3p21.1  0.896  0.431  2.178  3.069  1.895  2.316  3.627  2.989  3  3.21  1.796  1.535  2.844  1.884  3.109  2.28  3.42  0.143  3.822  3.159  1.595  3.262  2.92  4.534  2.637  2.528  2.954  3.664  3.063  4.284  3.14  2.594  3.903  2.468  2.483  2.847  2.518  3.792  1.967  2.435  1.762  2.888  4.153  2.669  1.665  3.033  3.037  2.579  1.478  3.903  3.1  2.185  3.12  0.858  2.831  2.56  1.758  2.806  2.647  2.488  
 
 ABHD14A-ACY1  100526760  3  52009041  52023218  3p  0  0  0  0  0  0.194  0  0  0  0.197  0  1.27  0.384  0  0  0.273  0  0  0  0  0  0  0  2.035  0  0.357  0  0  0  0.023  0  0  0  0  0.285  0  0.497  0  0  0  0  0  0  0  0  0  0  0  0  0  0  0  0  0  0  0  0.163  0  0  0.268  
 
 ACY1  95  3  52017299  52023218  3p21.1  3.673  1.533  1.728  1.673  3.687  1.727  3.581  2.128  2.049  2.136  1.592  3.166  4.944  3.35  2.794  3.406  3.958  0.704  3.572  3.052  3.529  1.016  2.705  2.877  3.398  3.77  4.036  4.004  3.446  5.254  4.581  4.063  4.418  2.795  2.187  2.708  3.035  3.047  1.465  3.113  3.31  2.561  4.02  2.746  2.582  3.059  4.705  2.574  2.128  4.263  2.884  2.042  2.87  1.434  3.51  3.045  1.96  2.926  3.094  3.944  
 
 RPL29  6159  3  52027643  52029958  3p21.3-p21.2  9.139  5.831  7.975  8.747  9.149  7.907  9.905  8.297  8.84  8.533  8.459  8.111  8.731  10.258  8.53  8.766  9.724  3.093  10.667  10.203  8.19  9.531  9.673  9.315  9.325  7.844  9.016  8.191  8.158  9.47  9.03  9.275  9.399  8.377  8.306  8.172  8.691  8.855  9.348  8.622  8.619  9.331  9.778  8.842  8.289  8.419  9.191  9.042  7.43  10.505  8.473  7.55  8.714  6.988  9.141  8.364  7.282  9.96  8.59  9.221  
 
 DUSP7  1849  3  52082936  52090461  3p21  1.94  3.416  2.889  3.627  1.768  2.525  3.524  3.644  2.846  3.596  3.311  1.181  2.708  2.817  2.465  1.349  1.844  1.043  3.977  3.583  2.354  3.503  1.762  2.665  3.562  2.206  2.876  3.049  2.258  2.728  2.809  3.32  3.935  2.671  1.53  2.043  3.339  3.587  2.933  2.593  1.851  2.819  2.495  2.88  1.743  2.613  2.128  3.377  2.812  3.971  3.917  1.646  3.296  1.625  2.312  2.207  2.563  3.849  3.604  3.672  
 
 LINC00696  100128378  3  52096109  52099128  3p21.2  0  0  0.374  0  0.025  0  0  0.028  0.226  0.044  0.064  0.04  0  0.086  0  0.03  0  0.061  0  0.042  0  0.03  0.507  0.044  0  0  0.13  0.062  0  0.063  0  0  0.049  0.349  0.034  0  0.03  0.078  0.018  0  0  0.046  0.137  0  0  0.082  0.036  0  0  0  0.086  0.24  0.05  0.043  0.032  0.064  0  0  0  0  
 
 POC1A  25886  3  52109248  52188706  3p21.2  2.076  2.49  1.922  2.395  2.36  1.692  3.22  1.742  1.352  2.408  2.557  2.061  2.089  2.651  2.409  2.03  2.795  0.643  4.419  2.353  1.683  2.939  1.983  1.802  1.782  2.011  2.54  2.384  2.257  2.269  2.034  2.21  2.821  1.564  1.911  2.058  3.203  2.953  2.804  3.185  2.035  2.372  2.981  1.926  1.603  2.797  2.553  2.749  2.281  3.923  2.859  1.76  2.628  1.142  2.571  1.673  1.95  3.038  2.478  2.925  
 
 ALAS1  211  3  52232098  52248343  3p21.1  2.846  2.955  2.711  3.947  3.3  2.263  4.758  2.653  3.339  2.701  4.033  2.977  4.554  4.213  3.494  3.396  4.54  2.279  4.255  4.883  2.212  2.629  2.502  2.448  3.712  4.113  3.912  3.288  4.015  4.886  4.488  4.165  3.941  2.371  2.87  3.551  4.264  3.488  3.791  4.081  3.427  3.127  4.292  2.777  2.576  3.584  2.856  3.789  2.227  5.021  4.305  2.629  3.163  2.346  3.25  2.991  2.519  3.889  3.482  3.893  
 
 TLR9  54106  3  52255095  52260179  3p21.3  0.045  0.02  0.264  0.119  0.091  0.05  0.086  0.043  0.333  0.129  0.083  0.119  0.062  0.051  0.068  0.04  0.069  0.066  3.322  0.859  0  1.482  3.158  4.188  0.048  0.046  0.141  0.036  0.035  0.126  0.027  0  0.037  0.401  0.04  0.039  0.072  0.161  0.122  0.123  0.031  0.33  0.145  0  0.074  0.152  0.298  0.123  0.039  0.098  0.092  0.94  0.026  0.129  0.104  0.067  0.076  0  0.042  0.034  
 
 TWF2  11344  3  52262625  52273183  3p21.1  2.576  0.78  4.384  5.206  2.763  2.491  4.908  3.326  4.547  4.179  4.643  3.019  4.213  4.723  3.603  3.649  3.981  0.241  5.815  3.797  2.869  3.677  4.138  4.162  4.93  4.147  5.21  3.526  3.847  4.584  4.179  4.636  5.542  4.199  2.911  3.533  3.936  3.585  3.889  3.222  2.102  4.288  3.936  3.629  3.665  3.906  2.333  4.273  2.637  5.704  4.589  2.766  3.694  2.822  4.56  3.827  3.351  4.371  3.907  5.007  
 
 PPM1M  132160  3  52279808  52284615  3p21.2  1.132  0.301  2.17  1.515  1.749  0.692  1.699  1.17  2.016  1.489  2.084  0.457  1.407  2.03  1.741  0.906  1.276  0.182  4.156  0.489  0.642  2.193  2.444  2.439  1.778  1.734  1.65  0.967  0.737  0.584  0.601  1.151  1.496  0.827  0.723  1.335  1.733  1.062  1.99  1.232  0.588  1.057  2.353  1.215  0.683  0.715  1.031  1.853  0.269  2.884  1.74  1.007  0.583  0.877  2.098  0.844  1.315  0.983  1.738  1.798  
 
 WDR82  80335  3  52288437  52312659  3p21.2  2.501  2.501  2.758  3.381  2.645  2.356  3.631  2.834  3.633  3.157  3.607  2.441  3.725  3.021  3.99  2.619  2.902  2.178  4.291  3.194  2.658  3.576  2.579  3.041  3.26  3.104  2.969  2.85  2.367  3.301  3.007  3.224  3.717  2.206  2.368  3.063  3.201  2.824  3.257  3.428  2.396  2.761  3.23  3.644  2.902  3.162  2.379  3.139  3.218  3.664  3.721  1.978  3.42  2.106  2.965  2.889  2.659  3.067  2.819  4.204  
 
 GLYCTK  132158  3  52321835  52329272  3p21.1  0.512  0.36  0.657  1.198  1.086  0.758  1.627  0.945  0.536  1.008  1.024  2.272  2.625  1.008  3.343  1.057  1.264  0.236  2.691  0.763  0.626  1.211  0.913  1.291  1.024  1.649  2.267  1.775  1.348  1.934  1.362  1.469  2.654  0.806  0.824  0.934  1.577  1.612  0.573  0.628  0.606  1.484  0.867  0.945  0.986  0.897  1.021  1.532  0.647  2.345  0.906  1.539  1.462  0.461  1.209  0.99  0.864  1.205  1.044  1.627  
 
 GLYCTK-AS1  100874110  3  52322595  52323749  -  0  0.176  0  0  0.09  0  0.18  0.112  0.161  0.141  0.24  0.192  0.242  0.227  0.952  0.137  0  0  0.205  0.213  0.223  0.475  0.485  0.164  0  0.153  0.586  0.409  0  0.571  0.127  0.196  0.436  0  0  0  0.297  0.268  0.185  0  0.124  0.325  0.619  0.137  0  0.28  0.464  0.294  0.206  0  0.193  0.393  0.443  0.205  0.109  0.294  0.233  0.346  0  0.198  
 
 DNAH1  25981  3  52350334  52434513  3p21.1  0.365  0.069  0.18  0.232  0.252  0.345  0.39  0.432  0.271  0.594  0.389  0.4  0.712  0.673  1.248  0.202  0.389  0.207  0.58  0.711  0.141  0.661  0.378  0.312  0.076  0.26  1.213  0.161  0.521  0.773  0.3  0.47  0.317  0.236  0.181  0.218  0.24  0.288  0.399  0.624  0.443  0.834  0.421  0.237  0.184  0.694  0.617  0.388  0.376  0.245  0.394  0.799  0.214  0.411  0.357  0.215  0.371  0.341  0.134  0.222  
 
 BAP1  8314  3  52435019  52444121  3p21.31-p21.2  2.525  1.303  2.689  3.588  2.898  2.17  4.473  3.454  4.093  3.619  3.937  2.569  3.821  3.032  3.88  2.258  2.946  0.296  5.065  2.891  2.768  3.654  2.639  3.168  3.103  3.679  3.779  3.886  3.479  3.958  3.617  3.928  4.35  2.231  2.231  3.189  4.035  4.016  0.136  3.72  3.283  3.16  3.889  2.941  2.463  3.383  3.24  3.836  3.013  4.629  3.596  2.321  3.595  2.172  3.303  2.839  3.106  3.992  3.777  3.72  
 
 PHF7  51533  3  52444576  52457657  3p21.1  0.449  2.355  0.438  1.044  0.6  0.505  1.111  0.417  0.636  0.797  0.529  0.718  0.404  0.745  0.532  0.206  0.733  0.386  2.267  1.335  0.821  1.496  0.711  0.638  0.254  0.561  0.44  0.421  0.435  1.101  0.636  0.54  0.525  0.59  0.273  0.504  0.742  1.124  0.278  1.255  0.272  0.723  1.106  0.346  0.215  0.325  0.42  0.712  0.892  1.22  0.785  1.113  0.512  0.241  0.471  0.199  0.286  0.747  0.346  0.34  
 
 SEMA3G  56920  3  52467267  52479043  3p21.1  0.138  0  0.199  0  0.437  0.049  0.102  0.053  0.189  0.076  0.091  0.12  0.077  0.501  0.173  0.062  0.086  0.567  0.461  0.361  0  0.436  0.544  0.545  0.035  1.081  0.269  0.182  0.028  0.219  0.098  0.157  0.101  0.199  0.1  0.066  0  0.054  0.06  0.283  0.054  0.186  0.066  0.29  0.07  0.316  0.788  0.326  0  0.59  0.109  0.406  0.034  0.143  0.076  0.104  0.026  0.368  0  0.036  
 
 TNNC1  7134  3  52485106  52488057  3p21.1  0  0  0  0  3.536  0  0.436  0.144  0.299  0.136  0.255  0.154  2.081  1.822  4.943  1.196  0.529  0  1.326  0.249  0  0.38  0.816  0.416  0  0  0.516  0  0  0.327  0  0.225  0  0  1.329  3.767  0  0  0.24  0.307  4.444  0.188  5.555  4.689  1.877  8.365  1.811  3.899  1.291  5.298  0.366  1.413  0  0.197  1.336  0.8  0  2.764  0  0  
 
 NISCH  11188  3  52489523  52527088  3p21.1  2.622  1.042  2.337  2.526  2.582  1.622  2.837  2.789  3.205  3.683  2.838  1.635  2.435  2.886  3.715  2.168  2.499  0.551  4.406  1.955  2.21  3.401  1.946  2.162  2.192  2.739  3.482  2.735  2.822  2.4  2.358  2.777  3.052  1.606  1.466  2.642  3.238  3.149  1.747  3.118  2.266  2.496  3.35  2.914  1.907  2.786  2.57  3.232  2.219  3.795  2.741  2.23  2.688  1.317  3.143  2.238  2.366  2.517  2.854  3.351  
 
 STAB1  23166  3  52529355  52558511  3p21.1  0.109  0.091  0.098  0  0.116  0.08  0.142  0.093  0.113  0.271  0.076  0.049  0.077  0.168  0.06  0.088  0.096  0.185  0.191  0.212  0.082  0.235  0.221  0.069  0.085  0.098  0.268  0.125  0.092  0.244  0.086  0.042  0.086  0.089  0.087  0.078  0.054  0.143  0.104  0.047  0.062  0.327  0.149  0.047  0.048  0.089  0.346  0.068  0.023  0.072  0.095  0.465  0.096  0.135  0.118  0.061  0.049  0.06  0.042  0.041  
 
 NT5DC2  64943  3  52558384  52569093  3p21.1  5.621  1.734  5.678  4.149  4.432  3.943  6.124  6.098  5.228  7.024  6.586  4.061  4.408  5.013  4.9  3.786  4.143  0.43  6.332  3.227  4.612  4.818  5.105  5.442  4.706  4.042  5.666  4.521  4.705  5.181  5.507  3.179  5.277  2.769  4.182  5.061  6.751  6.126  2.207  5.252  3.808  5.028  5.991  3.746  4.841  5.319  5.579  4.595  1.409  5.522  5.958  3.512  6.034  2.451  4.768  4.43  4.701  5.57  5.287  5.602  
 
 SMIM4  440957  3  52570620  52574586  3p21.1  3.654  5.54  3.462  3.961  3.476  4.722  4.237  3.023  4.148  3.988  3.497  3.123  3.412  3.632  1.612  4.306  3.768  4.789  5.02  4.467  3.38  4.731  3.784  4.161  3.491  3.221  3.067  2.692  4.376  2.346  5.812  4.872  3.739  4.181  4.084  3.371  2.319  2.557  4.319  2.151  3.351  5.182  3.673  4.322  3.404  4.794  1.583  2.372  2.38  4.42  2.363  2.768  2.877  3.967  4.21  2.831  2.121  2.534  2.932  2.573  
 
 PBRM1  55193  3  52579367  52719866  3p21  2.017  3.696  1.674  1.626  2.031  1.846  2.236  2.256  1.701  2.284  2.681  1.559  1.719  2.832  2.587  1.677  1.724  3.13  3.8  2.334  1.566  3.259  1.304  1.756  1.667  2.603  1.911  2  2.233  1.86  3.189  2.119  2.415  1.33  1.131  2.09  2.555  1.887  0.787  2.243  2.212  1.319  2.459  1.965  1.853  2.001  1.383  1.815  1.817  2.265  2.469  1.185  2.134  0.873  1.085  1.033  2.028  2.56  1.469  2.942  
 
 GNL3  26354  3  52719935  52728510  3p21.1  3.577  6.161  2.949  3.285  3.594  2.777  4.524  3.406  3.239  3.246  4.539  2.826  4.345  5.907  4.071  3.68  4.075  5.544  6.128  4.247  4.018  3.729  3.693  4.068  3.618  3.727  3.204  3.451  3.392  3.412  4.303  4.615  3.909  2.513  2.93  3.897  4.235  3.477  2.926  3.62  3.802  3.122  4.707  4.075  3.051  3.352  2.655  3.613  2.894  5.86  4.261  2.369  3.861  2.083  3.615  3.257  3.339  4.499  3.752  4.584  
 
 GLT8D1  55830  3  52728499  52740099  3p21.1  2.728  4.475  2.581  3.148  3.548  1.481  4.093  3.05  3.205  3.572  3.183  1.614  2.464  3.766  2.368  2.252  3.237  3.715  4.123  2.96  1.664  3.035  3.385  2.5  2.659  2.986  2.745  2.741  2.358  2.278  2.654  3.199  3.116  2.551  1.882  2.873  2.793  2.764  3.294  3.071  2.212  2.34  3.862  2.599  1.537  1.916  3.092  2.129  2.323  4.438  3.377  1.891  2.414  1.385  2.977  2.315  2.231  2.354  2.558  3.768  
 
 SPCS1  28972  3  52739856  52742197  3p21.1  4.15  6.621  4.049  4.208  5.79  3.697  5.785  4.269  5.027  4.491  4.83  4.128  4.478  5.894  3.451  4.508  5.338  7.439  6.294  5.591  4.249  4.785  6.357  5.784  4.928  4.836  3.846  4.4  4.073  4.15  5.309  5.29  4.839  4.852  3.913  4.757  4.055  4.739  4.917  3.896  4.561  4.552  4.544  4.349  3.836  4.558  4.861  3.856  3.619  6.357  4.776  3.429  4.688  3.218  4.823  4.087  3.456  5.738  4.856  5.289  
 
 NEK4  6787  3  52744795  52804965  3p21.1  1.571  2.025  0.821  0.866  1.615  0.895  1.406  1.482  1.711  1.373  1.95  0.994  1.894  2.018  1.901  1.204  1.428  1.397  2.398  1.774  0.998  1.296  0.886  1.106  1.273  1.658  1.05  1.59  1.348  1.137  1.689  1.551  1.685  0.741  0.968  1.863  2.21  1.78  1.109  1.867  1.652  0.849  1.753  1.297  1.452  1.384  0.847  1.67  1.705  1.97  1.973  0.843  1.732  0.69  1.325  0.911  1.024  1.732  1.437  2.266  
 
 ITIH1  3697  3  52811601  52826084  3p21.1  0  0  0.089  0  0.046  0  0.046  0  0.109  0  0  0.046  0  0.031  0  0.062  0  0.134  0  0.064  0  0.168  0.3  0  0  0  0.12  0  0  0.07  0  0  0  0.192  0  0  0  0  0.045  0  0  0.046  0.04  0  0  0.048  0.31  0  0  0  0.051  0.302  0  0.046  0.067  0  0  0  0  0  
 
 ITIH3  3699  3  52828783  52843025  3p21.1  0  0.035  0.189  0  0.019  0  0  0  0.066  0.088  0.031  0  0  0.044  0  0.04  0  0.205  0.033  0.059  0  0.085  0.197  0  0  0.113  0.073  0.334  0  0.096  0.08  0.055  0.089  0.199  0.039  0  0  0  0.058  0  0  0.241  0.049  0  0  0  0.035  0.071  0  0.189  0.039  0.326  0  0.058  0.086  0  0  0  0  0  
 
 ITIH4  3700  3  52847005  52864717  3p21.1  0  0.088  0.166  0  0.375  0.111  0.479  0.124  0.275  0.427  0.229  0.046  0  0.417  0.258  0.051  0.078  0.14  0.519  0.08  0  0.31  0.4  0.044  0.054  0.053  0.492  0.09  0.13  0.12  0.128  0.258  0.146  0.259  0.155  0.28  0.188  0.193  0.256  0.275  0.11  0.228  0.066  0.129  0.107  0.147  0.04  0.095  0.269  0.349  0.1  0.43  0.074  0.097  0.093  0.058  0.194  0.087  0.098  0.065  
 
 ITIH4-AS1  100873993  3  52857950  52859330  -  0  0  0  0  0  0  0  0  0  0.246  0  0  0  0  0  0  0  0  0  0  0  0  0  0  0  0  0.159  0  0  0  0  0  0  0  0  0  0  0  0  0  0  1.193  1.015  0  0  0  0  0  0  0  0  0  0  0  0  0  0  0  0  0  
 
 MUSTN1  389125  3  52867130  52869235  3p21.1  0  0.492  0.713  0.732  0.633  0  0.989  0.218  1.04  1.009  1.08  0  0.231  1.239  0.656  0.351  0.615  0  3.249  0.866  0  1.337  0.848  0.552  0.296  0.603  1.137  0.718  0.846  0.348  1.064  0.424  0.656  0.342  0.249  0.511  0.632  1.219  0.711  0.587  0.403  0.515  0.161  0.201  0  0.615  0.267  0.337  0.657  0.894  0.858  0.781  0.469  0.369  0.469  0  0.703  0.634  0.865  0.3  
 
 TMEM110-MUSTN1  100526772  3  52867130  52931597  -  0  0  0  0  0  0.388  0  0  0  0  0.308  0  0  0.059  0  0  0  0  0  0  0.202  0  0  0  0  0  0  0  0.334  0  0  0.861  0  0  0.193  0.224  0  0  0  0  0  0.454  0  0  0.175  0  0  0.16  0  0.102  0  0.488  0.257  0.156  0.134  0  0  0.423  0  0  
 
 TMEM110  375346  3  52870771  52931597  3p21.1  0.789  0.765  1.397  1.146  1.065  0.615  2  1.273  2.047  1.512  1.568  0.512  1.386  1.379  1.559  0.58  0.895  0.477  2.353  1.058  0.549  1.276  0.921  0.711  1.397  1.629  2.016  1.6  1.512  1.726  1.55  1.441  1.974  0.908  0.635  1.017  1.796  1.763  0.716  1.447  1.072  0.835  0.886  0.808  0.946  1.306  0.427  1.295  1.277  1.779  1.942  0.667  1.45  0.655  0.876  0.811  1.276  1.249  1.217  1.517  
 
 SFMBT1  51460  3  52937582  53080089  3p21.1  1.221  2.236  0.822  1.101  1.046  1.078  1.402  1.5  0.98  1.279  1.259  1.147  1.839  1.828  1.724  1.577  1.454  1.757  2.581  1.397  0.488  1.484  0.735  0.714  1.013  1.666  1.574  1.456  1.304  1.203  1.613  1.204  1.388  0.622  0.695  1.901  1.521  1.115  1.118  1.64  1.682  1.012  3.138  1.754  1.722  1.344  1.419  1.567  2.011  2.116  1.311  0.879  1.514  0.441  0.861  0.515  0.984  1.543  1.858  1.714  
 
 RFT1  91869  3  53122500  53164470  3p21.1  1.621  1.024  1.351  1.727  1.284  0.637  3.062  1.521  1.993  1.825  1.94  1.201  1.994  2.293  1.778  1.386  1.655  0.917  2.896  2.091  0.905  1.388  1.809  1.923  1.713  1.748  1.501  1.292  1.338  1.402  1.501  1.949  1.767  0.871  1.121  1.685  2.875  1.874  1.125  2.203  1.804  1.713  1.65  0.999  0.977  1.506  2.57  1.49  1.279  2.366  2.126  1.137  1.985  0.756  1.584  1.44  1.173  2.128  1.855  2.493  
 
 PRKCD  5580  3  53195222  53226733  3p21.31  2.645  1.597  1.492  0.653  2.194  0.407  1.656  2.054  1.089  1.691  2.016  1.803  2.324  2.435  1.835  2.481  1.66  0.515  1.138  2.738  1.61  0.653  1.087  1.202  1.672  3.621  3.397  3.588  3.167  2.866  3.184  4.717  4.379  2.448  1.078  1.744  2.259  2.789  0.885  1.51  2.015  1.402  1.305  1.778  1.52  2.31  0.554  2.346  1.568  2.82  2.922  1.922  2.451  1.302  2.213  1.101  2.196  2.129  2.71  3.373  
 
 TKT  7086  3  53258722  53290130  3p14.3  5.805  3.039  5.709  5.985  5.274  4.168  7.742  6.164  7.821  7.833  7.732  5.615  7.36  6.31  6.556  6.144  6.766  0.99  7.331  6.093  5.738  5.654  4.755  6.388  5.447  5.719  6.555  7.198  6.374  6.851  6.813  7.114  7.4  5.317  7.331  6.404  7.65  6.287  5.291  6.723  7.291  7.857  5.575  6.964  5.656  6.302  5.534  5.626  6.372  6.861  6.086  5.842  6.856  6.055  6.4  6.944  5.976  7.04  6.245  7.819  
 
 DCP1A  55802  3  53317444  53381654  3p21.1  2.28  2.655  1.646  2.208  1.72  1.029  2.691  2.219  2.504  1.996  2.481  1.687  3.332  3.266  2.92  2.553  3.139  3.237  3.532  2.323  1.812  2.918  1.893  1.63  2.36  2.473  2.404  1.921  1.739  2.166  2.326  2.35  2.486  1.563  1.178  1.969  3.086  2.076  1.628  2.467  1.815  1.7  2.696  2.153  1.525  1.229  1.93  2.024  1.993  2.744  2.663  1.215  2.15  1.164  2.437  1.779  1.887  2.152  2.073  2.677  
 
 CACNA1D  776  3  53529075  53846492  3p14.3  1.062  0.011  0.111  0  1.389  0.019  0.012  0.015  0.205  0.609  0.06  0.27  0  0.126  0.637  0.423  0.503  0.156  0.021  0.056  0  0.118  0.5  0.876  0  0.116  0.782  0.026  0.436  0.094  0.768  0.103  0  0.195  0.173  1.175  0.014  0  0.064  0.362  0.279  0.185  0.052  0.071  0.085  0.258  0.273  0.177  0.041  0.168  0.034  0.359  0.023  0.126  0.045  0.085  0.018  0  0.028  0.923  
 
 CHDH  55349  3  53850323  53880420  3p21.1  2.051  0.038  0.169  0.156  1.994  0.057  0.163  0.037  1.415  0.145  0.537  2.404  1.546  1.63  2.147  2.548  2.19  2.791  0.104  0.057  0.238  0.26  0.806  1.483  0  0.497  0.21  0.123  0.061  0.129  0.05  0  0  0.231  0.682  1.716  0.498  0  0.109  1.208  0.761  1.264  0.131  0.992  0.667  1.037  2.568  0.54  0.492  1.765  0.647  1.199  0.121  0.106  1.501  0.125  0.137  0.042  1.726  1.297  
 
 IL17RB  55540  3  53880576  53899827  3p21.1  1.775  0.244  0.108  0  2.375  0  0.172  0.141  0.951  0.214  0.53  2.088  0.804  1.958  1.88  1.507  2.126  2.783  0.127  0.686  0.394  0.345  0.797  0.865  0.083  0.629  0.163  0.229  0.197  0.056  0  0.258  0.11  0.247  0.248  1.505  0.463  0.285  0.559  1.12  0.68  0.579  0.038  0.383  0.348  0.751  2.965  0.6  0.429  1.619  0.448  0.411  0.2  0.155  0.782  0.103  0.249  0.179  1.445  0.649  
 
 ACTR8  93973  3  53901093  53916229  -  1.497  2.108  1.232  1.14  1.034  0.592  1.896  1.229  1.472  1.464  1.703  0.734  1.531  2.002  1.526  1.178  1.309  1.706  2.14  1.278  1.078  1.263  0.974  0.897  0.948  1.905  1.004  1.377  1.448  1.474  1.611  1.919  1.129  0.548  0.868  1.613  1.407  1.317  1.443  1.241  1.045  1.12  1.53  1.312  1.408  1.404  0.648  0.905  0.976  1.913  1.92  0.796  1.623  0.743  1.472  0.714  1.294  1.328  1.451  2.354  
 
 SELK  58515  3  53919225  53925989  3p21.31  3.119  5.995  3.65  3.179  4.246  3.584  4.806  3.462  4.476  4.085  4.067  3.47  3.046  4.194  2.888  3.591  4.892  6.447  5.407  5.659  2.999  4.521  4.696  4.384  3.802  3.608  3.806  3.453  3.579  2.899  4.177  3.923  3.895  2.843  2.585  3.383  3.728  3.823  5.413  3.025  3.227  3.275  4.116  3.352  3.339  3.939  5.358  3.661  2.766  5.405  4.276  2.232  3.826  2.779  4.098  3.181  3.176  5.092  3.862  4.201  
 
 CACNA2D3  55799  3  54156692  55108584  3p21.1  0.144  0  0.441  0  0.021  0  0.242  0.016  0.081  1.528  0.119  0.029  0  0.037  0  0.023  0  0.06  0  0.929  0  0.086  0.088  0.147  0.056  0  0.093  0.067  0  0.094  0  0.033  0  0.14  0.037  0  0.066  0  0.164  0  0  0.646  0.044  0.556  0.497  0.345  0.088  0  0.872  0  0.037  0.247  0.262  0.141  0.56  0.163  0.289  0  0.358  0.585  
 
 ESRG  790952  3  54666150  54673884  3p14.3|3p14.3  0  0.031  0.219  0  0  0.07  0.079  0.025  0.247  0.096  0  0.45  0  0.067  0  0  1.002  0.035  0  0  0  0.029  0.495  0.095  0  0.036  0.245  0.022  0  0.042  0.034  0  0  0.342  0.088  0  0.028  0  0.04  0  0.053  0.037  0.365  0.071  0  0.086  0.072  0.593  1.309  0.246  0.492  0.084  0.023  0.035  0.064  0  0  0.04  0  0  
 
 CACNA2D3-AS1  100874237  3  54908631  54935282  -  0  0  0.137  0  0.033  0  0  0  0.112  0.107  0  0  0  0.021  0  0  0  0  0  0  0  0.085  0.134  0  0  0  0.035  0  0  0.039  0  0  0  0.159  0  0  0  0  0.032  0  0  0.033  0  0.057  0  0.062  0  0  0.088  0  0  0.195  0  0  0.09  0  0  0  0  0  
 
 LRTM1  57408  3  54952380  55001115  3p14.3  0  0  0.192  0  0  0  0  0  0.224  0.23  0  0  0  0  0  0  0  0.161  0  0  0  0.096  0.388  0  0  0  0.097  0  0  0.066  0  0  0  0.367  0  0  0  0  0  0  0  0.096  0.064  0.183  0  0  0.041  0  0.159  0  0  0.409  0.074  0.109  0.145  0  0  0  0  0  
 
 WNT5A  7474  3  55499742  55521670  3p21-p14  0  0.014  3.511  0.524  0.039  0.891  3.615  1.138  0.559  3.055  2.089  0.115  0.531  0.028  0  0.018  0.028  0.1  0.029  0.084  0.067  0.222  0.394  0.082  2.796  0.033  0.698  0.03  0.102  0.281  0.082  0.494  0  0.216  0.033  0.661  1.803  0.026  2.761  0.139  1.551  1.331  0.054  1.268  2.141  1.397  0.104  0.459  0.33  1.505  1.996  0.605  0.403  0.222  0.176  0.069  1.032  0.037  0.023  1.952  
 
 ERC2  26059  3  55542335  56502391  3p14.3  0.213  0.015  0.132  0  0.017  0.032  0.173  0.023  0.154  0.025  0.07  0.04  0.028  0.019  0  0.031  0.027  0.102  0  0.028  0  0.092  0.227  0.066  0.027  0.013  0.134  0.013  0  0.105  0.026  0  0  0.222  0.025  0.024  0.289  0  0.214  0.057  0.038  0.238  0.162  0.425  0.229  0.233  0.133  0.519  0.03  0.471  0.144  0.222  0.011  0.055  0.327  0  0  0.035  0.202  0.429  
 
 ERC2-IT1  711  3  55691242  55693497  3p14.3  0  0  0.179  0  0  0  0  0  0.105  0  0  0  0  0.018  0  0  0  0  0  0  0  0  0.377  0.18  0  0  0.037  0  0  0.021  0  0  0  0.305  0  0  0.072  0  0.129  0  0  0.263  0.032  0.072  0  0.101  0  0.248  0  0.041  0  0.22  0  0  0.061  0  0  0  0  0  
 
 CCDC66  285331  3  56591183  56655848  3p14.3  0.938  2.952  0.47  0.524  0.666  0.589  1.053  0.391  1.091  0.758  1.213  0.467  0.902  1.604  1.046  0.613  0.897  3.221  2.533  1.315  0.896  1.573  0.807  0.715  0.715  1.056  0.647  0.887  0.617  0.476  0.972  0.948  0.74  0.555  0.585  0.705  1.033  0.904  1.072  1.247  0.772  0.726  1.262  0.859  0.588  0.736  0.302  0.971  0.915  2.215  1.136  0.389  0.689  0.321  0.902  0.513  0.84  0.635  1.099  1.152  
 
 FAM208A  23272  3  56654159  56717135  3p14.3  1.663  2.552  1.249  1.875  1.659  0.86  1.803  1.771  1.663  1.95  2.118  0.918  1.77  2.107  1.771  1.459  1.186  2.122  2.749  1.393  1.309  1.558  1.116  1.058  1.865  1.434  1.116  1.691  1.308  1.201  1.601  1.739  1.716  1.14  1.079  2.289  1.646  1.725  2.069  1.944  2.044  1.219  2.269  1.334  0.946  1.479  1.004  2.139  2.293  3.691  2.568  1.268  1.783  1.111  2.001  1.556  1.618  1.943  2.054  2.312  
 
 ARHGEF3  50650  3  56761445  57113336  3p14.3  0.702  1.83  0.375  0.663  0.729  0.436  1.265  0.813  1.2  1.47  2.008  1.463  1.466  2.016  0.819  1.181  1.047  2.996  2.478  0.502  0.267  2.573  2.075  2.571  0.487  1.534  1.54  0.808  1.175  1.022  0.642  1.26  1.788  2.058  0.382  1.602  0.821  1.462  1.695  0.851  1.362  0.742  1.312  1.568  0.85  1.382  0.572  0.509  1.209  1.454  1.962  0.715  0.802  0.864  0.729  0.597  0.878  0.499  1.659  1.407  
 
 SPATA12  353324  3  57094468  57109460  3p14.3  0.076  0.039  0.157  0  0.043  0  0.5  0.075  0.29  0.13  0.503  0.203  0.511  0.444  0.222  0.243  0.526  0.541  0  0  0.13  0.045  0.52  0  0  0.435  0.084  0.204  0.111  0.49  0.429  0.142  0.281  0.328  0.122  0.354  0.199  0.484  0.147  0  0.568  0.169  0.019  0.249  0.18  0.301  0.061  0.086  0.485  0.136  0.376  0.191  0.173  0.077  0.281  0.108  0.182  0.181  0.255  0.457  
 
 IL17RD  54756  3  57124009  57199403  3p14.3  0.165  1.348  0.265  0.209  0.093  0.254  0.259  0.107  0.906  0.272  1.137  0.267  0.148  1.188  0.431  0.202  0.369  0.957  0.008  0.038  0  0.077  0.245  0.018  0.174  0.298  0.44  0.152  0.254  0.293  0.067  0.209  0.166  0.302  0.168  0.146  0.219  0.692  0.21  0.492  0.719  0.245  0.687  0.349  0.427  0.73  0.295  0.099  0.496  0.079  0.68  0.341  0.271  0.197  0.153  0.137  0.137  0.208  0.415  0.731  
 
 HESX1  8820  3  57231943  57234280  3p14.3  0  0.486  0  0  0.261  0  0.074  0.152  0.231  0.157  0.383  0  0.216  0.722  0  0.107  0.208  1.631  0.382  0.548  0  0.21  0.461  0  0.261  0.157  0.149  0  0.127  0.057  0.187  0.218  0.195  0.326  0  0.267  0.162  0  0  0.17  0.369  0.456  0.32  0.15  0  0.147  0  0.205  0.175  0.495  0.321  0.332  0.095  0.121  0.383  0  0.082  0  0  0.13  
 
 APPL1  26060  3  57261764  57307498  3p21.1-p14.3  1.453  3.047  1.362  1.352  1.769  1.336  2.199  2.048  1.796  2.069  2.411  1.391  2.222  2.558  2.017  1.02  2.204  2.812  3.138  2.772  0.745  2.239  1.538  1.323  2.063  1.886  1.671  1.694  1.64  1.238  2.049  1.98  2.309  1.385  1.162  2.072  1.881  2.182  1.769  2.101  1.916  1.352  2.068  1.498  1.555  1.945  0.934  1.752  2.444  2.422  2.893  1.205  2.287  0.97  1.768  1.837  2.027  1.955  1.923  2.969  
 
 ASB14  142686  3  57302378  57326710  3p21.1  0.147  0.271  0.156  0  0.224  0  0.128  0.155  0.171  0.195  0.242  0  0.198  0.344  0.184  0.037  0.118  0.361  0.328  0.262  0  0.438  0.268  0.125  0.115  0.116  0.25  0.135  0  0.082  0.147  0.093  0.176  0.326  0.122  0.136  0.167  0.28  0.182  0  0.106  0.46  0.498  0.216  0.086  0.193  0.451  0.287  0  0.103  0.316  0.208  0.143  0.087  0.197  0.14  0.162  0.207  0.11  0.065  
 
 DNAH12  201625  3  57327726  57530071  3p14.3  0  0.06  0.052  0  0.029  0.029  0.071  0.046  0.034  0.04  0.039  0.085  0.073  0.054  0.054  0  0.136  0.319  0.019  0.049  0.048  0.074  0.144  0  0  0  0.133  0.047  0  0.072  0.041  0.077  0.033  0.214  0.028  0  0.035  0  0.032  0  0.037  0.074  0.042  0  0.042  0.058  0.101  0  0  0.066  0.098  0.099  0.05  0.062  0.026  0  0  0.032  0.032  0  
 
 PDE12  201626  3  57541980  57547768  3p14.3  1.611  2.51  1.454  1.563  2.102  1.202  2.223  1.625  1.679  1.631  2.252  1.669  2.607  2.34  2.589  1.355  2.186  1.665  3.03  2.389  1.769  2.3  1.283  1.824  2.16  2.32  2.031  2.629  2.147  2.197  2.158  2.259  2.724  1.559  1.52  2.078  2.071  2.427  2.401  2.1  2.005  1.595  1.433  2.075  1.634  1.869  0.867  2.358  1.699  2.384  2.636  1.54  2.329  1.264  1.749  1.485  1.461  1.806  1.628  3.022  
 
 ARF4  378  3  57557089  57583215  3p21.2-p21.1  3.804  7.673  5.747  5.407  5.386  5.115  6.546  5.891  5.148  6.029  6.058  4.167  4.864  5.825  4.17  4.744  5.532  6.313  6.138  5.16  3.657  4.176  4.616  3.678  5.408  5.596  4.279  4.893  4.958  3.581  5.276  5.561  5.186  4.838  3.899  5.138  5.792  5.107  6.906  4.523  4.973  4.589  5.115  4.594  4.415  5.586  4.489  4.463  4.233  6.911  6.137  3.55  5.144  4.976  5.138  5.706  5.197  4.758  5.283  6.488  
 
 DENND6A  201627  3  57611180  57678816  3p14.3  1.317  2.485  0.56  0.766  0.932  0.548  1.241  1.141  1.008  1.12  1.515  0.731  1.633  1.807  1.173  1.124  0.604  2.194  2.804  1.046  0.461  1.493  0.787  0.624  1.259  1.133  0.983  0.905  0.908  0.902  1.287  0.903  1.211  0.731  0.571  1.415  1.145  1.388  1.021  1.399  1.313  0.74  1.539  1.034  0.53  1.059  1.276  0.848  1.244  1.91  1.511  0.674  0.867  0.556  0.835  0.822  0.704  0.891  1.226  1.435  
 
 SLMAP  7871  3  57742982  57915741  3p21.2-p14.3  1.537  3.618  2.13  1.812  1.566  0.973  2.104  1.963  1.992  1.633  2.404  1.249  1.748  3.116  1.497  2.03  2.285  3.832  2.987  1.843  1.508  1.708  1.458  1.053  1.897  1.732  1.266  1.492  1.488  1.258  1.604  1.643  1.965  1.562  1.026  2.152  2.261  2.199  2.646  1.765  2.432  1.777  2.051  1.96  1.437  1.918  2.022  1.933  1.9  3.32  2.796  1.468  1.946  1.224  1.848  1.552  1.065  1.749  2.045  2.977  
 
 FLNB  2317  3  57994126  58157982  3p14.3  3.639  3.576  2.906  3.284  3.544  2.632  4.294  3.298  2.937  3.589  4.281  2.227  4.6  3.033  3.738  4.207  4.928  0.906  2.393  0.947  2.189  2.336  0.779  1.151  4.181  2.296  1.209  3.876  1.573  2.355  1.57  2.185  2.833  0.786  4.171  4.712  5.362  4.814  4.691  3.614  4.885  3.343  2.863  4.189  3.105  4.308  4.613  4.984  4.282  5.15  6.072  4.435  4.887  3.381  5.222  3.674  4.072  4.658  4.864  5.586  
 
 DNASE1L3  1776  3  58178352  58196730  3p14.3  0  0  0.227  0  0.103  0  0  0  0.135  0  0.106  0  0  0.117  0  0  0  0.137  0  0.119  0  0.129  0.59  0.24  0  0.109  0.067  0.143  0.132  0.067  0.134  0  0  0.22  0  0  0  0  0.157  0.245  0  0.318  0.074  0  0  0  0  0  0  0.102  0.151  0.372  0.24  0.131  0  0  0  0  0  0  
 
 ABHD6  57406  3  58223258  58280461  3p14.3  0.359  1.198  0.874  1.284  0.639  0.327  1.938  1.126  1.703  1.531  1.035  0.758  1.561  1.711  1.362  0.573  1.958  1.037  1.569  0.315  0.416  1.286  1.427  1.148  0.671  2.153  1.913  2.032  2.147  1.83  2.357  1.87  2.612  1.614  0.74  1.557  1.038  1.286  0.814  1.441  0.407  1.151  1.651  0.595  0.476  0.415  2.401  1.076  0.614  1.723  2.158  1.072  1.031  0.485  0.882  0.268  0.592  0.941  0.907  1.301  
 
 RPP14  11102  3  58291971  58305920  3p14.3  1.599  2.702  1.53  2.253  2.584  1.148  2.485  1.83  1.883  1.835  2.312  1.498  2.351  2.731  1.905  1.615  2.259  2.196  2.756  2.424  1.955  2.468  2.19  1.502  2.061  2.256  1.363  1.987  1.452  1.979  2.488  2.43  2.297  1.721  1.029  1.873  2.036  2.117  2.575  1.989  1.69  1.49  2.491  1.842  1.669  2.117  2.001  2.01  1.701  2.985  2.546  1.301  2.131  0.88  1.545  1.283  1.782  1.556  1.782  2.233  
 
 PXK  54899  3  58318616  58411854  3p14.3  0.39  2.918  2.181  1.567  3.582  0.829  2.909  2.166  1.91  2.755  1.861  0.282  0.896  1.75  0.972  0.444  0.801  1.009  3.178  3.941  1.251  1.663  1.688  0.875  1.642  1.727  1.509  1.969  1.789  0.659  1.573  2.373  2.389  1.858  0.44  1.512  1.811  3.34  2.787  0.983  0.576  1.093  0.89  1.69  0.973  2.067  0.275  0.96  1.798  1.841  2.088  0.743  2.947  1.043  0.828  0.72  2.059  0.867  2.588  2.998  
 
 PDHB  5162  3  58413356  58419579  3p21.1-p14.2  3.819  5.337  3.715  3.195  5.064  2.574  4.781  4.033  3.526  3.9  4.406  2.834  3.871  4.909  3.42  3.322  4.14  5.083  5.995  4.6  3.31  3.891  3.165  3.278  3.803  3.558  4.28  3.544  3.905  4.325  4.298  4.689  4.34  2.904  3.396  4.416  4.34  4.165  4.546  4.086  3.403  3.148  4.493  4.601  3.17  4.185  3.401  3.98  3.192  5.887  3.916  2.193  3.804  2.001  3.812  3.294  3.187  4.141  3.857  4.856  
 
 KCTD6  200845  3  58477822  58488087  3p14.3  1.246  3.343  1.354  1.011  3.732  1.028  1.984  1.507  1.986  1.448  2.135  0.368  1.474  2.508  1.273  0.933  1.474  2.237  2.77  1.113  0.56  1.161  1.377  0.993  1.428  0.913  1.414  0.978  0.7  0.67  1.209  1.25  1.485  1.409  1.006  1.705  1.72  1.884  1.505  1.346  0.966  1.706  1.239  1.87  0.671  1.058  1.113  1.299  1.213  2.91  1.983  0.916  1.245  0.334  0.955  0.967  1.175  1.786  1.213  1.41  
 
 ACOX2  8309  3  58490862  58522929  3p14.3  0.139  1.464  0.14  0  3.291  0  0.718  0.074  0.533  0.388  1.254  0.08  0.247  2.399  0  0.07  0.059  0.341  0.042  0.205  0  0.149  0.229  0  1.246  0.195  0.181  1.727  0.1  0.616  0.121  1.716  1.414  1.264  0.423  1.9  0.367  2.392  0.168  0.944  0.131  1.525  0.082  1.526  0  0.101  2.957  0.158  0.125  0.624  2.152  0.614  0.116  0.124  0.125  0.132  0.188  0.148  0  0.075  
 
 FAM107A  11170  3  58549838  58613337  3p21.1  0  0.345  0.152  0.15  0.085  0  0.124  0.057  0.145  1.147  0.028  0.084  0  0.064  0  0.054  0  0.12  0.042  0.055  0.149  0.132  0.422  0  0  0  0.1  0.104  0  0.089  0  0  0  0.28  0.045  0  0.038  0.158  0.149  0  0  0.785  0.096  3.67  4.676  2.715  0.035  0.098  0  0.074  0.153  0.497  0.15  0.107  0.079  0.089  0.42  0.515  0  0  
 
 FAM3D  131177  3  58619669  58652561  3p14.2  0  0  0.172  0  0.14  0  0  0  0  0  0  0.869  0  0.062  0  1.58  0.174  0  0  0  0  0.097  0.322  0  0  0  0.034  0  0  0.064  0  0  0  0.261  0  0  0  0  0.079  0  0.622  0.079  0  0  0  0  0  0  0  0  0  0.256  0  0.087  0  0  0  0  0  0  
 
 C3orf67  200844  3  58727736  59035715  3p14.2  0.645  0.657  0.176  0.457  0.799  0.112  0.155  0.119  0.104  0  0.313  0.542  0.336  0.582  0  0  0.275  0.868  0  0.044  0.296  0.271  0.72  0.399  0.641  0.884  0.112  0.747  0.678  0.097  1.283  1.521  0.482  0.283  0.222  0.191  0.584  1.084  0.316  0.61  0.307  0.323  0.724  0.332  0.198  0.257  0.137  0.403  0.328  0.937  1.746  0.661  0.701  0.408  1.107  0.386  1.292  0.415  1.195  0.835  
 
 C3orf67-AS1  101929238  3  58810196  59004819  -  0  0  0.071  0  0.063  0  0  0.048  0  0  0.053  0.052  0  0.065  0  0  0  0  0  0  0  0.049  0.133  0  0  0.208  0.107  0.075  0  0.037  0.147  0.09  0  0.286  0  0  0.209  0.287  0  0  0  0.211  0.03  0  0  0  0.04  0  0  0  0.129  0.112  0.167  0  0.064  0  0.153  0  0.111  0  
 
 FHIT  2272  3  59735035  61237133  3p14.2  0.753  0.703  0.215  1.049  0.524  0  0.363  0.15  0.359  0.53  0.307  0.218  1.383  1.465  0.435  0.309  0.925  0.407  1.17  2.046  1.198  2.443  1.722  1.587  0.163  0.811  0.995  0.776  0.332  0.906  1.601  0.558  0.595  0.519  0  0.314  0.87  1.234  0.095  0  0  0.268  0.669  0.894  0.388  0.81  0.94  1.132  0  2.353  0  0.428  0.162  0.277  1.207  0.406  0.384  0.709  0.41  0.327  
 
 PTPRG  5793  3  61547242  62280573  3p21-p14  1.327  3.007  1.684  1.213  0.115  0.823  1.609  1.894  2.53  2.173  2.195  0.335  2.196  2.149  1.912  0.097  1.024  0.13  0.12  0.46  0.087  0.217  0.542  0.339  2.214  0.131  1.444  2.57  3.469  1.769  2.333  2.537  2.542  1.138  0.668  1.76  2.865  2.899  0.973  0.17  0.035  0.908  1.007  1.489  1.823  1.705  0.9  1.139  1.969  0.552  0.063  1.87  2.578  0.712  1.849  1.537  2.405  1.776  2.279  2.114  
 
 PTPRG-AS1  100506994  3  62247493  62304622  -  1.714  1.24  0.316  0.33  0.416  0.052  0.639  0.13  0.485  0.419  0.668  0  0.241  0.161  0.157  0.106  0.051  1.953  0  0  0  0.039  0.247  0  0.792  0.81  0.783  0.549  1.318  0.823  1.699  0.773  0.976  0.446  0.279  0.276  0.782  0.739  0.048  0.459  0.093  0.284  1.353  0.478  0.182  0.379  0.587  0.739  0.143  1.43  0.209  0.516  0.687  0.096  0.833  0.274  0.839  0.684  0.981  1.188  
 
 C3orf14  57415  3  62304647  62321888  3p14.2  3.732  3.909  0.659  0.781  2.381  0  1.547  0.821  0.203  1.182  2.009  0  0  0.045  0  0.235  0  2.645  0  0  0  0  0  0.152  2.426  1.429  1.922  0.909  2.68  0.447  3.224  2.033  1.689  1.333  1.278  1.198  0.838  1.068  0.95  1.317  0.615  0.815  1.761  0.6  0.398  1.432  1.194  0.746  0.554  1.855  0.92  0.727  1.85  0.703  2.055  0.698  1.234  1.31  1.953  2.143  
 
 FEZF2  55079  3  62355346  62359190  3p14.2  0  0.056  0.195  0  0.109  0.12  0.05  0.161  0.177  0.132  0.142  0.088  0  0.094  0  0.228  0.168  0.33  0  0.139  0.098  0.369  0.26  0.122  0  0.051  0.183  0.052  0.061  0.215  0.114  0.111  0.097  0.143  0.072  0  0  0  0.198  0  0  0.277  0.074  0.074  0.085  0.445  0.057  0  0  0  0.052  1.256  0.082  0.199  0.15  0  0  0.107  0.069  0.068  
 
 CADPS  8618  3  62384020  62861064  3p14.2  0.058  0  0.117  0  0.036  0.027  0.037  0.033  0.087  0.039  0.215  0.053  1.113  0.027  0.353  0.033  0.037  0.178  0.02  0.065  0  0.105  0.22  0.04  0.559  1.328  0.356  0.033  0.878  0.144  1.041  0.429  0  0.091  0.031  0.044  0.036  0  0.06  0  0  0.065  0.057  0.03  0.034  0.033  0.055  0  0  0.053  0.075  0.388  0.063  0.093  0.061  0.055  0  0.031  0.045  0.026  
 
 LINC00698  285401  3  63088363  63110737  3p14.2  0  0  0  0  0  0  0  0  0.145  0.03  0  0  0  0.101  0  0  0  0  0  0  0  0.068  0.241  0  0  0.859  0.101  0.345  2.027  1.748  3.914  1.126  0  0.185  0  0  0  0  0.043  0  0  0.25  0.037  0  0  0  0  0  0  0  0  0.108  0  0.059  0  0  0  0  0  0  
 
 SYNPR  132204  3  63263913  63602597  3p14.2  0  0.07  0.086  0  0  0  0  0  0.131  0  0  0  0  0.02  0  0  1.06  0.096  0  0  0  0.093  0.11  0  0  0  0.063  0  0  0.04  0.119  0  0  0.226  0  0  0  0  0  0  0  0.226  0.036  0  0  0  0.027  0  0  0  0  0.134  0.038  0.029  0.039  0  0  0  0  0  
 
 SYNPR-AS1  100874016  3  63409271  63535727  -  0  0  0  0  0  0  0  0  0  0  0  0  0  0  0  0  0  0.236  0  0  0  0  0  0  0  0  0  0  0  0  0  0  0  0  0  0  0  0  0  0  0  0  0  0  0  0  0  0  0  0  0  0.311  0  0  0  0  0  0  0  0  
 
 SNTN  132203  3  63638343  63650891  3p14.2  0.161  0  0.084  0  0.114  0  0  0  0  0  0  0  0  0  0  0  0.1  0  0  0  0  0  0  0  0  0  0.053  0  0  0.03  0  0  0  0  0  0  0  0  0  0  0  0  0  0  0  0  0.073  0  0  0  0  0  0  0  0  0  0  0  0  0  
 
 C3orf49  132200  3  63805040  63834312  3p14.1  0.402  0.173  0  0  0  0  0.135  0  0  0.116  0  0.106  0.195  0.265  0.176  0  0  0  0.092  0  0  0.194  0.294  0  0  0  0.224  0.076  0  0.078  0  0.108  0.139  0  0  0  0.102  0  0.123  0  0  1.709  0  0  0  0  0  0  0  0.063  0.073  0  0.058  0.052  0.062  0  0  0  0  0  
 
 THOC7  80145  3  63819545  63849599  3p14.1  5.9  7.189  3.013  2.518  4.416  3.517  4.803  3.641  2.957  3.812  4.416  3.494  3.867  5.244  2.958  3.718  4.207  8.287  6.544  5.561  3.602  4.643  3.292  3.7  4.24  3.628  3.513  3.001  4.522  2.916  5.601  5.07  3.526  2.779  3.532  3.76  3.817  3.851  5.513  3.147  3.934  3.348  4.267  3.666  4.079  3.999  5.082  3.239  4.159  5.629  3.018  2.588  4.064  2.529  4.338  3.661  3.342  4.484  4.375  5.158  
 
 THOC7-AS1  100874039  3  63846320  63847495  -  1.481  0.171  1.859  0.744  0.504  2.3  1.108  0.896  1.241  0.419  0.552  1.32  0.439  0.367  0.579  1.027  0.562  0.367  0.348  0.488  0.553  0.623  1.48  0.828  1.111  0.74  1.79  0.396  0.747  1.683  1.884  2.453  1.07  1.485  0.427  0.513  0.625  0.931  0.721  0  0.875  3.377  0.369  0.479  2.027  2.22  3.056  1.372  0.98  0.155  0.174  0.4  0.763  0.322  0.563  0.634  0.408  1.943  0.757  0.438  
 
 ATXN7  6314  3  63850232  63989136  3p21.1-p12  2.24  1.636  1.187  1.193  1.425  0.847  1.76  2.06  1.126  1.793  1.775  0.7  1.655  2.098  1.893  1.435  1.581  0.752  2.331  1.259  1.043  1.869  1.101  1.079  1.491  1.985  2.337  1.886  2.597  1.398  2.352  2.028  2.091  1.087  0.973  1.933  2.072  1.856  2.205  2.014  1.618  1.111  2.008  1.266  2.009  1.719  1.328  1.672  2.147  1.66  1.079  1.267  1.895  0.722  1.59  1.168  1.728  1.536  1.898  2.143  
 
 PSMD6-AS2  100507062  3  63989697  63997917  3p14  1.002  0.429  0.292  0.244  0.321  0.169  0.249  0.35  0.205  0.577  0.405  0.058  0.389  0.525  0.447  0.335  0.239  0.658  0.545  0.183  0.152  0.5  0.165  0.2  0.188  0.54  0.83  0.297  0.807  0.217  0.89  0.438  0.551  0.317  0.088  0.336  0.604  0.56  0.661  0.169  0.309  0.195  0.542  0.239  0.327  0.528  0.533  0.457  0.344  0.329  0.098  0.348  0.351  0.174  0.385  0.121  0.522  0.278  0.715  0.298  
 
 PSMD6  9861  3  63996224  64009686  3p14.1  7.593  7.095  3.248  3.668  4.418  3.424  5.402  3.935  3.662  4.39  5.191  2.686  4.176  5.651  3.702  3.467  4.247  6.809  6.402  4.128  3.777  3.989  3.499  3.406  4.225  4.31  4.599  3.847  5.091  3.257  5.722  5.673  4.556  3.588  3.268  4.058  4.279  3.956  4.565  3.711  3.948  3.774  4.043  3.794  4.704  3.917  3.942  3.649  3.78  6.097  3.475  2.587  4.159  2.563  3.957  3.01  3.755  4.239  4.156  5.106  
 
 PRICKLE2-AS1  100652759  3  64053639  64088807  -  0.098  0.041  0.13  0  0.045  0  0.082  0.118  0.056  0.158  0.164  0.024  0  0.024  0.053  0  0  0.036  0.04  0.034  0  0.034  0.087  0.024  0.067  0.087  0.057  0.041  0.106  0.034  0.066  0.061  0.037  0.206  0.107  0.11  0.043  0.112  0.055  0  0.038  0.019  0.016  0.051  0.058  0.043  0.019  0  0.063  0.013  0.03  0.139  0.222  0.056  0.135  0  0.18  0  0.147  0.037  
 
 LINC00994  100287879  3  64064036  64073039  3p14.1  0.126  0  0.104  0  0  0  0  0.04  0.079  0.051  0.038  0  0  0  0  0  0  0  0  0  0  0  0.257  0  0  0  0.15  0  0  0.097  0  0  0  0.261  0  0  0  0  0.035  0  0  0  0.076  0  0  0  0.027  0  0  0  0  0.164  0.035  0  0.07  0  0  0  0  0  
 
 PRICKLE2  166336  3  64079525  64211131  3p14.1  0.61  1.197  1.436  1.059  0.479  0.314  1.353  1.818  1.295  2.065  1.945  0.051  0  0.027  0.124  0.028  0  0.128  0.012  0.021  0  0.165  0.209  0  0.76  1.084  0.298  0.436  1.185  0.044  1.064  1.593  0.308  0.443  0.79  1.682  0.413  1.415  1.112  0  0.149  0.572  0.026  0.583  0.586  0.92  0.35  0.208  0.338  0.315  0.01  0.235  1.986  1.278  1.601  0.654  1.867  0.037  1.554  1.758  
 
 PRICKLE2-AS2  100874242  3  64089145  64092034  -  0  0  0  0  0  0  0  0  0  0.134  0.097  0  0  0  0  0  0  0  0  0  0  0  0  0  0  0  0.051  0  0  0.049  0  0  0  0  0  0  0  0  0  0  0  0  0  0  0  0  0.196  0  0  0  0  0  0.129  0  0.127  0  0.254  0  0  0  
 
 PRICKLE2-AS3  100874243  3  64173219  64187236  -  0  0.039  0.288  0.085  0.029  0  0.212  0.143  0.079  0.188  0.115  0.064  0  0  0  0  0  0  0  0.031  0  0.046  0.314  0  0  0.065  0.265  0.03  0.067  0.049  0.062  0.063  0  0.195  0.138  0.079  0  0.084  0.077  0  0  0.046  0  0  0.052  0.108  0.804  0  0  0.025  0  0.109  0.283  0.017  0.16  0  0.158  0  0.081  0.069  
 
 ADAMTS9  56999  3  64501330  64673365  3p14.1  0  0.423  0.085  0  0.015  0  0.044  0.069  0.904  1.619  0.175  0.279  0  0.023  0  0.029  0.023  0.164  0.011  0.05  0.109  0.063  0.197  0.024  0.435  0.013  0.992  1.525  1.407  0.159  0.433  1.49  0.532  0.24  0.761  1.71  0.399  0  0.045  0.024  0.069  0.15  0.032  2.924  0.666  0.255  0.553  0.042  1.558  0.049  0.015  0.266  2.271  0.454  1.38  0.71  1.558  0.016  1.945  2.148  
 
 ADAMTS9-AS1  100507082  3  64547021  64575760  -  0  0.039  0.072  0  0  0  0  0  0.059  0.115  0.026  0  0  0  0  0  0  0  0  0  0  0.074  0.152  0  0  0  0.168  0.067  0.139  0.078  0.061  0.035  0.081  0.259  0.073  0.049  0  0  0.022  0  0  0.219  0.018  0.406  0  0.039  0.059  0  0.109  0  0  0.116  0.258  0.041  0.129  0  0.166  0  0.251  0  
 
 ADAMTS9-AS2  100507098  3  64670545  64997143  -  0  0.05  0.159  0  0  0  0  0  0.147  0.19  0.066  0.067  0  0.036  0  0  0  0.064  0  0  0  0.039  0.208  0  0  0  0.157  0.035  0.083  0.105  0  0  0  0.222  0  0.098  0  0  0.028  0  0  0.327  0.046  0.067  0  0  0.038  0  0.179  0  0  0.145  0.308  0.171  0.546  0.123  0.435  0  0.219  0.133  
 
 MAGI1  9223  3  65339905  66024509  3p14.1  1.905  1.582  1.441  0.362  1.178  0.679  0.23  1.117  1.076  1.094  0.944  0.607  1.177  2.698  1.828  1.24  1.177  1.773  0.107  0.091  1.617  0.356  0.643  0.534  0.695  0.122  0.443  1.214  1.306  0.491  0.782  1.871  0.805  0.89  0.413  1.347  1.915  1.031  0.9  1.15  1.273  0.676  1.409  1.203  0.884  1.651  1.709  1.13  0.278  1.261  0.59  0.834  0.732  0.392  0.858  0.39  0.602  0.627  1.077  1.706  
 
 MAGI1-AS1  100873983  3  65879490  65910972  -  0  0  0  0  0.165  0  0  0  0  0.131  0  0  0  0.454  0.317  0.261  0  0  0  0  0  0  0  0  0  0  0.09  0  0  0  0  0.369  0  0  0  0  0.246  0  0  0  0  0.645  0  0  0  0.203  0.094  0  0  0.163  0  0  0  0  0  0  0  0  0  0  
 
 SLC25A26  115286  3  66119284  66429351  3p14.1  1.28  2.167  1.38  1.271  1.726  0.883  2  1.283  2.02  1.62  1.831  1.527  1.284  2.516  1.128  1.462  1.737  2.199  3.03  2.47  1.001  2.226  2.078  1.161  1.572  1.588  1.364  1.346  2.471  1  2.647  2.209  2.205  2.205  1.264  1.322  1.398  1.251  1.475  1.232  1.308  1.655  1.675  1.137  1.429  1.397  2.145  1.232  1.495  2.3  0.961  0.936  1.464  0.784  1.938  1.123  1.108  1.395  1.864  2.035  
 
 LRIG1  26018  3  66429220  66550845  3p14  2.513  1.329  2.618  0.716  3.344  1.114  1.431  3.239  5.673  3.094  3.099  1.807  2.152  0.805  0.395  2.064  2.846  1.122  1.193  1.689  0.088  0.987  0.34  0.641  0.343  0.233  0.827  2.164  1.878  0.894  1.324  2.478  2.352  1.514  0.485  1.743  2.587  0.841  1.422  1.285  2.946  1.337  0.551  1.163  1.68  2.074  2.352  1.201  0.911  1.246  0.065  0.723  1.983  0.319  0.703  0.761  2.57  0.867  1.094  1.952  
 
 KBTBD8  84541  3  67048726  67061632  3p14  0.039  0  0.168  0.107  0.129  0.181  0.249  0.114  0.134  0.07  0.202  0.187  0.32  0.211  0.188  0.044  0.108  0.128  1.498  0.722  0.616  0.688  0.368  0.231  0.408  0.103  0.184  0.376  0.429  0.082  0.335  0.304  0.356  0.436  0.051  0  0.145  0  0.391  0.129  0.057  0.109  0.116  0.326  0.148  0.21  0.528  0.124  0  0.164  0.102  0.076  0.228  0.061  0.038  0.036  0.107  0.047  0.09  0.28  
 
 SUCLG2  8801  3  67410883  67705038  3p14.1  2.001  3.625  1.833  1.668  2.681  1.624  2.196  2.331  2.113  2.707  2.833  2.143  1.723  3.392  2.085  2.455  3.145  2.857  3.552  2.862  1.985  1.988  1.355  1.652  2.41  3.438  2.633  2.422  2.444  1.713  3.419  3.01  3.389  3.341  2.08  2.873  2.712  2.844  3.052  2.069  2.53  2.099  2.7  2.496  1.266  2.205  2.028  1.54  2.063  2.871  2.236  1.349  2.281  1.568  3.041  2.44  1.554  2.296  3.095  3.857  
 
 SUCLG2-AS1  101927111  3  67705120  67998136  -  0.233  0.938  1.081  0.404  0.162  0.677  0.795  0.751  0.463  1.109  0.625  0.25  0.479  0.178  0  0.125  0.138  0.219  0.169  0  0  0.115  0.184  0  0.27  1.539  0.625  1.695  0.992  0.384  1.513  0.695  2.685  1.387  0.801  0.75  1.088  0.817  0.575  0.881  0.463  0.239  0.068  0.13  0  0.777  1.208  0.683  1.189  0.217  0.811  0.957  1.432  0.464  1.099  0.332  0.636  0.367  1.273  1.574  
 
 FAM19A1  407738  3  68040733  68594771  3p14.1  0  0  0.376  0.188  0  0  0.073  0.04  0.089  0  0  0.106  0  0.034  0  0  0  0.072  0  0  0  0.033  0  0  0  0  0.227  0  0  0.219  0  0  0  0.341  0  0  0  0  0.043  0  0  0  0.06  0  0  0  0.237  0  0  0  0.044  0.445  0  0.059  0  0.088  0  0  0  0  
 
 FAM19A4  151647  3  68780914  68981761  3p14.1  0  0  0.134  0  0  0  0  0.04  0.109  0.048  0  0.051  0  0.05  0  0  0  0.082  0  0.143  0  0.07  0.365  0  0  0  0.113  0  0  0.083  0  0.055  0  0.368  0  0  0  0  0.063  0  0  0.202  0.152  0  0  0.055  0.052  0  0  0  0  0.223  0  0.047  0.037  0  0  0  0  0  
 
 EOGT  285203  3  69024362  69063045  3p14.1  0.532  1.922  1.446  0.784  0.933  0.68  1.222  2.141  0.591  1.524  1.145  0.791  0.973  1.884  1.299  0.658  0.775  1.007  1.669  0.61  0.768  0.202  0.54  0.576  1.745  2.369  2.084  1.491  2.435  1.141  2.392  2.944  2.703  1.774  0.515  1.693  1.374  1.498  1.053  1.587  1.018  0.529  1.52  0.545  0.403  0.729  1.856  0.975  1.604  0.94  0.676  0.924  1.464  0.743  1.148  1.159  1.764  0.904  1.677  1.884  
 
 TMF1  7110  3  69068977  69101484  3p21-p12  1.081  2.707  1.04  1.342  1.451  0.726  1.511  2.07  1.189  1.738  1.637  0.835  1.402  1.743  1.468  1.107  1.543  2.147  1.836  1.205  0.709  0.869  0.745  0.59  1.227  2.104  1.113  1.061  2.044  0.575  2.15  2.094  1.592  1.31  0.708  1.434  1.261  1.163  1.26  1.131  1.332  0.942  0.859  1.479  0.95  1.197  0.229  0.863  1.125  1.047  0.986  0.708  1.034  0.958  1.193  1.192  1.224  1.004  1.109  2.181  
 
 UBA3  9039  3  69103880  69129524  3p24.3-p13  1.608  4.387  1.743  1.855  2.643  1.76  3.318  3.091  2.449  3.216  3.886  1.697  3.059  3.491  2.406  2.888  3.096  3.997  4.782  3.692  1.705  3.055  1.821  1.553  2.769  3.44  2.468  1.999  3.689  1.305  4.041  3.914  3.147  2.037  1.678  2.749  2.829  2.904  2.946  2.427  3.115  1.862  2.403  2.442  2.07  2.547  2.304  1.986  2.363  3.376  2.425  1.71  2.576  1.543  3.217  2.172  2.751  2.638  3.113  3.759  
 
 ARL6IP5  10550  3  69134089  69155239  3p14  3.745  4.772  3.443  3.683  3.92  1.803  4.827  4.091  4.957  4.173  4.832  2.923  3.943  4.252  3.613  3.805  4.417  4.074  4.945  4.184  1.474  3.159  3.711  3.327  4.11  4.515  4.824  3.449  4.978  3.249  4.891  5.272  4.777  4.612  3.245  4.576  3.638  4.798  4.102  2.334  4.015  3.731  2.942  3.216  2.706  3.166  3.753  2.962  2.804  3.713  3.805  3.411  4.315  2.989  4.084  3.744  4.067  3.102  3.918  4.687  
 
 LMOD3  56203  3  69157822  69171746  3p14.1  0  0  0.086  0  0  0  0.044  0.035  0  0  0  0.155  0  0.025  0  0.111  0  0  0  0.05  0  0.161  0.209  0  0  0.061  0.115  0.078  0  0.104  0  0.142  0  0.394  0  0  0.055  0.079  0.06  0  0  0.366  0.09  0  0  0  0.038  0  0  0  0.042  0.159  0.028  0.076  0  0  0  0  0  0  
 
 FRMD4B  23150  3  69217933  69435455  3p14.1  0.424  0.195  0.086  0  0.602  0  0.151  0.032  0.167  0.163  0.502  0.853  0.755  0.184  1.054  1.175  0.691  0.355  0.129  0.715  0  0.196  0.625  0.216  0.06  1.455  0.897  0.553  2.569  1.187  1.931  1.615  1.593  0.989  0.159  0.074  0.505  0.558  0.154  0.446  0.691  0.414  0.037  0.904  0.177  0.537  0.59  0.256  0.051  0.128  0.918  0.228  0.845  0.436  0.878  0.593  0.46  0.208  0.834  1.184  
 
 MITF  4286  3  69788585  70017488  3p14.2-p14.1  0.138  1.348  0.714  1.354  0.203  0.518  1.965  1.136  1.1  2.498  1.085  0.094  0.183  0.309  0.448  0  0.099  0.344  0.046  0.488  1.396  0.22  1.338  1.188  0.502  5.855  5.425  3.799  5.425  5.913  6.5  4.922  5.182  4.39  1.575  0.783  2.053  2.313  0.448  0.872  0.709  0.753  0.472  3.097  0.539  1.854  1.324  0.687  2.217  0.346  1.266  1.63  2.376  1.937  2.377  1.839  1.202  0.783  1.753  3.355  
 
 LINC01212  101927152  3  70048894  70064451  -  0  0  0.132  0  0  0  0  0  0.081  0.037  0  0  0  0  0  0  0  0.051  0  0  0.203  0.024  0.26  0  0.086  0.689  0.322  0.698  0.767  0.582  1.396  1.211  1.186  0.783  0  0  0  0  0  0  0  0.046  0.032  0.129  0.124  0.064  0  0  0  0  0  0.075  0  0.035  0.032  0  0  0  0  0  
 
 FOXP1  27086  3  71003864  71633140  3p14.1  1.781  3.117  2.462  2.168  1.244  2.216  2.889  3.528  2.171  2.285  2.716  1.646  0.447  0.718  1.478  2.276  2.427  3.367  2.113  2.567  1.718  1.746  0.578  0.88  2.296  1.734  0.997  0.961  1.005  1.132  1.808  1.354  0.578  0.823  1.443  2.115  2.07  1.741  3.404  1.03  1.746  1.106  3.278  0.73  1.309  1.67  3.071  1.177  2.405  1.082  3.333  1.237  2.821  1.544  2.918  1.734  2.671  1.811  3.429  3.684  
 
 FOXP1-AS1  104502416  3  71338908  71355005  3p13  0  0  0  0  0.12  0  0  0  0  0.107  0  0  0  0  0  0  0  0  0  0  0  0  0  0  0  0  0  0  0  0.072  0  0  0  0  0  0  0  0  0  0  0  0  0.313  0  0.394  0  0.099  0  0  0  0  0  0.279  0  0.163  0  0.209  0  0  0  
 
 MIR1284  100302112  3  71591120  71591240  -  0  0  0  0  0  0  0  0  0  0  0  0  0  0  0  0  0  0  0  0  0  1.568  0  0  0  0  0  0  0  0  0  0  0  0  0  0  0  0  0  0  0  0  0  0  0  0  0  0  0  0  0  0  0  0  0  0  0  0  0  0  
 
 EIF4E3  317649  3  71728439  71803924  3p14  0.572  0.207  0.374  0  1.382  0.426  0.431  0.322  1.007  0.858  1.047  0.259  0.167  0.74  0.279  0.025  0.65  0.117  0.047  0.407  0.188  0.982  0.921  0.422  0.038  0.731  0.235  0.134  0.256  0.516  0.682  0.233  0  0.33  0.538  0.76  0.137  0.183  0.596  0.478  0.191  0.697  4.111  0.452  0.122  0.499  0.332  0.163  0.793  0.262  0.606  0.697  0.659  0.328  0.216  0.179  0.306  0.062  0.15  0.677  
 
 GPR27  2850  3  71803200  71804328  3p21-p14  0.803  0.141  0.302  0  0.936  0.254  0.093  0.286  0.192  0.356  0.105  0.282  0  0.131  0  0.21  0.257  0.376  0  0.626  0  1.133  0.474  0.133  0  0.073  0.238  0.125  0  0.276  0.153  0.245  0  0.303  0.412  0  0  0  0.377  0.45  0  1.751  0.936  0.32  0.785  0.882  0.158  0.163  0.439  0.16  0  2.635  0.08  0.485  0.513  0  0  0.199  0  0  
 
 PROK2  60675  3  71820805  71834357  3p13  0  0  0.133  0  0  0  0.085  0  0  0  0.057  0  0  0  0  0  0  0  0  2.631  0  0.509  0.186  0  0  0  0.103  0  0  0.05  0  0  0  0.298  0  0  0  0  0.066  0  0  0  0.406  0.084  0  0  0.084  0  0  0  0  0.303  0  0  0  0  0  0  0  0  
 
 LINC00877  285286  3  72084669  72149606  3p14.2  0  0  0.068  0  0  0  0  0  0.1  0  0  0  0  0  0  0  0  0.078  0.096  0  0  0.055  0.219  0  0  0  0.05  0  0  0.045  0  0  0  0.355  0  0  0  0  0.021  0  0  0.033  0.042  0  0  0  0.035  0  0  0  0  0.204  0  0.034  0  0  0  0  0  0  
 
 LINC00870  201617  3  72200407  72223490  3p14.2  0  0  0.146  0.142  0.07  0.048  0.049  0.085  0.105  0.086  0.044  0.082  0  0.144  0  0  0  0.063  0  0  0  0.045  0.247  0  0  0.038  0.142  0.049  0.089  0.259  0.219  0  0  0.403  0.081  0  0  0  0.041  0  0.066  0  0.072  0  0  0.082  0.025  0  0  0  0.053  0.221  0  0.053  0.028  0.058  0  0  0  0  
 
 RYBP  23429  3  72423743  72495774  3p13  2.152  3.083  1.86  2.142  2.495  1.37  2.823  2.94  1.946  2.629  2.529  1.503  1.952  2.675  1.787  1.802  2.72  2.023  2.878  2.402  1.712  2.561  1.51  0.926  1.888  2.129  2.436  2.083  2.685  1.902  3.091  2.839  2.448  1.971  1.361  1.86  2.637  1.672  3.059  2.031  2.215  1.527  2.855  1.751  1.712  2.405  1.708  1.579  2.084  1.962  2.3  1.286  2.641  1.137  2.28  1.639  2.088  1.763  2.752  2.504  
 
 SHQ1  55164  3  72798427  72897598  3p13  1.799  2.744  1.204  1.712  1.739  0.472  2.564  1.949  1.096  2.002  2.064  1.142  1.896  2.93  1.421  1.209  1.631  3.483  4.627  2.918  1.376  3.347  1.136  1.027  1.741  1.87  2.123  2.116  2.863  1.72  2.804  2.993  3.221  2.644  1.168  1.681  1.895  1.488  1.549  1.899  0.882  0.954  2.535  1.449  1.37  1.563  1.488  1.108  1.452  1.901  1.968  0.886  1.801  0.991  1.35  1.142  1.333  1.375  1.508  2.293  
 
 GXYLT2  727936  3  72937384  73024522  3p13  0.426  1.311  1.709  1.695  0.871  1.13  1.517  3.198  2.065  2.996  2.856  0.077  0  0.65  1.203  0.081  1.633  0.889  3.4  1.776  0.74  3.816  0.153  0  1.518  1.127  2.339  2.058  2.311  1.753  1.38  3.114  3.223  2.055  0.618  1.032  1.843  1.602  1.647  1.253  0.577  1.452  1.63  0  1.409  2.278  0.391  0.479  1.07  0.638  0.095  0.762  2.077  1.344  0.705  0.88  1.485  1.642  0.41  2.65  
 
 PPP4R2  151987  3  73046118  73115011  3p13  1.683  3.498  1.781  1.685  2.65  1.248  2.237  2.956  2.031  2.69  3.26  1.542  2.651  3.084  2.264  2.116  2.618  3.061  3.74  2.645  2.274  3.264  1.695  1.725  2.609  2.804  1.445  1.805  2.908  0.794  3.882  3.915  2.836  1.903  1.661  2.937  2.694  1.862  3.14  2.364  2.554  1.457  1.956  2.636  1.985  2.615  1.155  1.72  2.538  2.257  2.798  1.576  2.042  0.711  1.429  1.729  2.076  2.74  2.443  3.457  
 
 EBLN2  55096  3  73110809  73112471  3p13  1.158  1.031  0.473  0.523  0.734  0.205  0.655  0.865  0.597  1.008  0.985  0.184  0.785  1.782  1.01  0.778  0.416  0.854  2.137  1.13  0.52  1.471  0.512  0.352  0.333  0.864  1.508  0.62  1.326  0.936  1.931  1.245  1.259  0.542  0.294  0.534  1.01  0.831  0.745  0.564  0.556  0.489  1.351  0.708  0.415  0.875  0.716  0.406  0.632  0.641  0.649  0.524  0.877  0.061  0.634  0.319  0.776  0.666  0.762  0.516  
 
 PDZRN3  23024  3  73431580  73674072  3p13  0.5  0.076  1.276  1.322  1.103  0.302  0.43  1.889  2.333  2.989  1.496  0.161  0  0.059  0.094  0.105  1.403  0.388  0.027  0.175  0  0.494  0.373  0.048  0.054  1.802  1.922  3.254  2.048  0.317  1.522  3.226  2.489  1.416  0  0  0  0  0.184  0.148  0  0.988  0.086  0.042  0.145  0.151  0.072  0  0  0.088  0.039  1.211  0.045  0.217  0.176  0.099  0  0.1  0  0.178  
 
 LOC101927296  101927296  3  73570472  73618925  3p13  0  0  0  0  0.198  0.148  0  0.453  0.234  0.793  0.232  0  0  0  0  0  0  0.186  0  0  0  0  0  0  0  0.391  0.784  0.706  0.256  0.105  0.428  0.63  0.648  0.319  0  0  0  0  0  0  0  0  0  0  0  0  0  0  0  0  0  0.185  0  0  0  0  0  0  0  0  
 
 PDZRN3-AS1  100874099  3  73672718  73677050  -  0  0  0.231  0  0.132  0  0  0.104  0.221  0.237  0.055  0  0  0  0  0  0.045  0.115  0  0  0  0.035  0.161  0  0  0.128  0.117  0.172  0.084  0.035  0.078  0.32  0.056  0.266  0  0  0  0  0.017  0  0  0.396  0.031  0  0  0  0.025  0  0  0  0  0.138  0  0.02  0  0  0  0  0  0  
 
 CNTN3  5067  3  74311719  74570291  3p12.3  0  0  0.044  0  0.086  0  0  0.013  0.044  0.012  0  0  0  0.018  0  0  0  0  0  0.021  0  0.02  0.057  0  0  0  0.03  0  0  0.038  0.419  0.102  0  0.164  0  0  0  0  0.011  0  0  0.016  0.023  0  0  0.014  0.023  0  0  0  0  0.084  0  0.025  0.013  0  0  0  0  0  
 
 FAM86DP  692099  3  75470702  75484266  3p12.3  1.459  1.587  1.26  1.457  1.251  1.069  2.2  1.316  1.581  1.353  1.499  0.529  1.818  1.552  1.577  1.527  1.536  1.409  2.014  1.483  1.178  1.342  1.618  1.865  1.385  3.032  4.28  3.142  4.228  2.529  3.223  3.924  4.745  3.204  1.487  0.958  2.459  3.094  1.388  1.973  1.477  1.366  1.937  0.643  0.587  0.962  2.77  1.076  1.232  1.691  2.484  1.229  1.905  0.703  2.158  1.15  1.023  1.313  2.334  2.558  
 
 FRG2C  100288801  3  75713486  75716368  3p12.3  0  0  0.193  0  0  0  0  0  0.136  0  0  0  0.107  0  0  0  0  0  0.348  0  0  0  0.288  0  0  0  0.174  0  0  0  0  0  0  0.488  0  0  0  0  0  0  0  0.036  0  0  0  0  0.072  0  0  0  0  0.09  0  0  0  0  0  0  0  0  
 
 LINC00960  401074  3  75721431  75728454  3p12.3  0.358  1.261  1.278  1.311  0.213  0.193  0.695  0.068  0.359  0.176  0.129  0.131  0.553  0.271  0.644  0  0.938  0.174  0  0.264  0  0.994  0.779  0  0  0.403  0.443  0.648  0.351  0.138  0.303  1.075  1.507  1.546  0.098  0.535  1.15  0.294  0.079  0.431  0.125  0.301  1.181  0.457  0.472  0.107  0.186  0.534  0.42  0.604  0.574  0.594  0.086  0.112  1.082  0.434  0.617  0.828  1.012  0.149  
 
 ZNF717  100131827  3  75779111  75834734  3p12.3  0  2.058  0.562  0.908  1.465  0  1.39  0.765  0.093  1.282  0.466  1.669  0.877  0.064  1.352  1.174  0.84  2.715  0.234  1.368  0.882  0.343  1.98  1.15  1.352  1.205  0.661  0.978  1.88  0.575  2.176  2.445  0.938  1.042  0.427  0.817  1.396  1.185  0.277  0.958  0.519  0.527  0.16  0.607  0.904  0.912  0.628  1.102  1.112  1.578  1.239  0.679  0.177  0.642  1.57  0.756  0.896  0.848  0.804  1.485  
 
 ROBO2  6092  3  77089293  77699114  3p12.3  0  0.025  0.318  0.086  0.678  0.042  0.275  0.011  0.07  0.024  0.022  0.044  0  0.02  0  0.013  0.018  0.17  0  0.033  0.237  0.058  0.147  0  0.072  1.573  0.104  0.058  0.926  0.916  0.856  1.895  0  0.291  0.017  0  0.018  0  0.037  0.114  0  0.186  0.092  0.157  0.172  0.018  0.045  0.03  0  0.066  0.019  0.192  0  0.043  0.017  0.044  0  0  0.059  0  
 
 ROBO1  6091  3  78646387  79817059  3p12  0.032  0.024  2.827  2.539  1.892  0.76  2.737  1.885  1.772  3.293  2.575  0.03  1.064  0.027  0.026  0.024  1.205  2.645  0.033  0.567  0.05  0.134  0.24  0.055  2.416  2.244  2.941  1.815  2.273  2.058  2.716  4.449  3.404  1.917  0.963  3.076  2.152  1.949  1.056  3.22  1.497  0.895  0.89  0.308  1.456  0.065  0.207  1.913  0.548  2.337  2.069  0.403  0.977  0.448  1.203  1.262  1.934  1.341  0.9  2.814  
 
 LOC101927374  101927374  3  79460876  79484377  -  0  0  0.136  0  0  0  0  0  0  0  0.069  0  0  0  0  0  0  0  0  0  0  0  0.161  0  0  0  0.017  0.189  0  0  0  0  0  0.392  0  0  0  0  0  0  0  0  0.028  0  0  0  0  0  0  0  0  0  0  0  0  0  0  0  0  0  
 
 GBE1  2632  3  81538849  81810950  3p12.3  1.175  3.911  3.715  1.758  1.342  1.85  2.853  3.068  3.484  3.2  3.631  0.689  1.29  2.278  1.346  0.675  2.391  2.964  2.916  3.221  1.116  1.401  0.991  1.379  2.439  1.767  2  1.957  2.108  1.981  2.994  3.005  2.717  2.184  1.365  1.864  2.574  2.388  4.599  1.626  1.746  2.623  0.808  1.554  1.125  2.982  0.672  2.049  1.996  3.651  3.819  1.382  2.338  1.905  1.971  1.819  2.751  1.818  2  2.822  
 
 LINC00971  440970  3  84687555  84918726  3p12.1  0  0  0.074  0  0.007  0  0.014  0  0.036  0.014  0.013  0.034  0  0.012  0  0  0.025  0.028  0  0  0  0.019  0.139  0  0  0  0.029  0  0  0.029  0  0  0  0.297  0  0  0.019  0  0.029  0  0  0.146  0.027  0  0.036  0.022  0.023  0  0  0  0.015  0.021  0  0.021  0  0  0  0  0  0  
 
 CADM2  253559  3  85008132  86123579  3p12.1  0  0.016  0.06  0.075  0.021  0.018  0  0.015  0.046  0.012  0.01  0.028  0  0.023  0  0  0.018  0.062  0  0.019  0.03  0.056  0.156  0  0.021  0  0.073  0.028  0  0.117  0  0  0  0.291  0.048  0  0  0  0.016  0.034  0.056  0.588  0.098  0  0  0.022  0.035  0  0  0.059  0.263  0.148  0  0.034  0.019  0  0  0  0  0  
 
 CADM2-AS2  100874037  3  85849136  85877200  -  0  0  0  0  0  0  0  0  0  0  0  0  0  0  0  0  0  0  0  0  0  0  0  0  0  0  0  0  0  0  0  0  0  0.579  0  0  0  0  0  0  0  0.13  0  0  0  0  0  0  0  0  0  0  0  0  0  0  0  0  0  0  
 
 VGLL3  389136  3  86987122  87040257  3p12.1  0.029  0.428  2.726  1.484  0.027  1.748  0.695  2.765  0.087  2.288  0.367  0.033  0.483  0.03  0.27  0.079  0.013  0.038  0.032  0.04  0  0.105  0.183  0.017  3.166  0.018  0.088  0.953  0.011  0.107  0.033  0.02  0.315  0.38  0.399  1.084  2.072  0.38  2.197  0.075  0.754  0.14  0.04  0.017  0.088  0.11  0.106  1.322  0.107  1.791  0.209  0.145  0.023  0.05  0.024  0.076  0.039  0.151  0  0.363  
 
 LINC00506  100846978  3  87138429  87206219  3p12.1  0  0  0.17  0  0  0.116  0  0.373  0.211  0.13  0.083  0  0  0.036  0  0  0  0.206  0.105  0  0  0.073  0.312  0  0  0  0.07  0  0  0.07  0  0  0  0.44  0  0  0.107  0  0.069  0  0  0.249  0.078  0  0  0  0  0  0  0  0  0.271  0  0.099  0  0  0  0.503  0  0  
 
 CHMP2B  25978  3  87276412  87304698  3p11.2  2.342  3.307  2.065  2.627  2.692  1.993  2.27  2.389  2.108  2.391  3.042  2.14  2.87  2.896  2.27  2.348  4.144  4.402  3.104  2.501  1.432  2.488  1.464  0.874  2.204  2.202  1.584  1.864  1.922  1.308  3.234  2.688  2.76  2.741  2.312  2.244  2.125  1.475  3.581  1.922  2.4  1.977  1.87  1.463  1.704  1.929  1.695  1.763  2.106  4.039  3.382  1.235  1.625  1.269  1.981  1.947  1.464  1.887  1.672  2.834  
 
 POU1F1  5449  3  87308782  87325737  3p11  0  0  0  0  0  0  0  0  0  0  0  0  0  0  0  0  0  0.108  0  0  0  0  0  0  0  0  0.037  0  0  0.053  0  0  0  0  0  0  0  0  0  0  0  0  0  0  0  0  0  0  0  0  0  0  0  0  0  0  0  0  0  0  
 
 HTR1F  3355  3  88031725  88042919  3p12  0  0.045  0.119  0  0  0  0  0  0  0  0  0  0  0  0  0  0  0  0  0  0.524  0.02  0.132  0.047  0  0.041  0.035  0.045  0.106  0.022  0.076  0.093  0.039  0.373  0  0.139  0.193  0  0  0  0  0  0.016  0  0.075  0.022  0.264  0  0  0  0  0.137  0  0  0.022  0  0  0  0  0  
 
 CGGBP1  8545  3  88101099  88199016  3p12-p11.1  3.213  4.134  2.281  2.23  2.62  2.142  3.338  2.798  2.978  3.142  3.237  1.632  2.578  3.346  2.457  1.34  2.762  4.212  4.688  2.996  1.896  3.621  2.276  1.685  2.639  3.54  3.236  3.034  2.522  2.415  3.407  3.866  3.636  3.529  2.072  2.533  2.614  2.476  3.557  2.685  2.204  1.837  2.862  2.008  1.938  2.199  2.307  2.318  3.096  3.631  2.997  1.907  2.441  1.481  2.25  1.975  2.12  2.299  2.374  2.849  
 
 ZNF654  55279  3  88188261  88193814  3p11.1  0.795  1.288  0.692  0.744  0.975  0.257  0.89  0.872  1.037  1.127  1.212  0.205  0.814  0.843  0.849  0.288  0.972  0.957  1.609  0.804  0.539  0.814  0.374  0.324  0.817  1.434  0.766  1.059  0.937  0.398  1.558  1.895  1.526  1.538  0.277  0.656  0.674  0.715  1.405  1.014  0.79  0.487  0.436  0.857  0.396  0.869  0.114  0.561  1.064  1.194  1.199  0.363  0.565  0.265  0.476  0.324  0.824  0.443  0.747  1.328  
 
 C3orf38  285237  3  88198892  88207115  3p11.1  1.692  2.553  1.156  1.795  1.671  0.515  1.57  1.315  1.707  1.681  1.785  1.002  1.708  2.139  1.358  0.735  1.535  1.771  3.136  1.526  1.079  1.828  1.216  0.925  1.588  1.876  1.073  1.189  1.354  0.701  1.777  1.71  1.696  1.62  1.053  1.391  1.561  1.2  2.354  1.807  1.397  1.098  2.101  1.294  1.323  1.309  1.125  1.386  1.74  2.617  1.748  0.684  1.491  0.84  1.41  0.949  1.204  0.987  1.335  2.209  
 
 EPHA3  2042  3  89156673  89531284  3p11.2  0  0  0.18  0.087  0.015  0.051  2.081  2.416  4.312  1.709  3.269  0.093  0  0.022  0  0.028  0  0.198  0.11  0.027  0  1.713  0.278  0  0  0.181  1.521  0.526  0.588  1.998  1.744  3.175  3.257  1.578  0  0  0  0.684  0.034  0.053  0  1.6  0.256  0.327  0.093  0  0.177  0  0  0.06  0.408  0.246  0  0.044  0  0  0.063  0  0  0  
 
 PROS1  5627  3  93591880  93692934  3q11.2  0  0.959  0.082  0.296  0.164  0.478  0.282  0.662  1.187  2.32  0.716  2.318  0.604  1.374  0.94  1.095  0.945  2.246  0.033  0.21  1.213  0.113  0.154  0  0.516  3.195  2.246  1.43  2.616  2.28  2.741  2.252  2.364  2.457  0.561  1.995  0.144  2.14  0.16  1.64  0.26  0.935  1.536  0.429  0.133  0.133  0.357  0.622  0.395  2.196  0.551  0.929  0.349  0.959  2.356  0.078  0.777  0.823  0.811  1.234  
 
 ARL13B  200894  3  93698982  93774522  3q11.1  0.701  2.565  0.801  0.641  0.841  0.592  1.001  1.293  0.965  1.53  1.377  0.859  0.755  1.157  0.891  1.275  1.094  3.054  1.692  0.761  1.004  1.049  0.359  0.216  1.139  1.107  0.863  0.875  0.538  0.355  1.145  1.378  1.279  0.997  0.955  0.905  1.033  0.798  1.185  1.38  0.684  0.806  1.28  1.165  0.63  0.663  0.594  1.274  1.447  2.446  1.276  0.67  1.014  0.588  1.228  0.584  0.89  1.4  1.245  1.7  
 
 STX19  415117  3  93733214  93747454  3q11  0  0.169  0  0  0.213  0.253  0  0.06  0  0  0  0.512  0  0.188  0  0.624  0.842  0.285  0  0.082  0  0.104  0.346  0  0  0.078  0.059  0.106  0  0.18  0  0  0  0.269  0.121  0  0  0  0.181  0  0.783  0  0  0  0.321  0.789  0  0.159  0.206  0  0.088  0  0  0  0.081  0  0.121  0  0  0  
 
 DHFRL1  200895  3  93776765  93782067  3q11.1  0.822  0.506  0.573  0.439  0.86  0.389  0.851  0.839  0.815  1.113  0.783  0.853  0.858  0.6  0.663  0.782  0.766  0.689  1.778  0.443  0.198  1.275  0.603  0.463  0.664  0.857  0.868  0.567  0.558  0.719  0.874  0.581  1.197  1.024  0.898  0.765  0.527  0.561  0.632  1.154  1.05  0.487  0.197  0.292  0.436  0.199  0.76  0.353  0.448  0.533  0.914  0.459  0.554  0.312  0.47  0.316  0.288  0.283  0.399  0.707  
 
 NSUN3  63899  3  93781854  93845630  3q11.1  1.906  1.828  0.581  0.836  1.587  0.71  1.875  1.172  0.67  1.421  1.912  1.291  1.399  2.019  1.008  1.592  1.694  1.514  2.261  1.108  0.76  1.272  0.585  0.571  0.851  1.728  1.254  1.56  0.963  0.702  1.82  1.608  1.733  0.884  0.884  0.996  1.489  0.984  1.26  1.384  1.084  0.964  2.146  0.906  0.81  0.845  1.111  0.65  0.739  1.706  1.51  0.594  1.134  0.79  0.88  0.601  0.823  1.008  1.126  1.692  
 
 LINC00879  255025  3  94657106  94710169  3q11.2  0  0  0  0  0  0  0  0  0  0  0  0  0  0  0  0  0  0  0  0  0  0.058  0  0  0  0  0  0.176  0  0.036  0  0  0  0  0  0  0  0  0  0  0  0  0  0  0  0  0  0  0  0  0  0.211  0  0  0  0  0  0  0  0  
 
 MTHFD2P1  100287639  3  95373266  95402037  3q11.2  0  0  0.175  0.177  0.03  0.07  0.097  0  0.099  0  0.252  0.113  0  0.047  0  0  0  0  0  0.047  0  0.051  0.34  0  0.12  0  0.193  0.054  0  0.284  0  0.093  0  0.421  0.066  0  0  0  0.027  0.147  0  0.182  0.082  0  0.061  0.066  0.332  0  0  0  0  0.153  0.043  0.025  0.557  0  0  0  0  0  
 
 EPHA6  285220  3  96533424  97467786  3q11.2  5.166  4.923  2.557  1.071  4.674  4.444  2.229  3.148  0.764  2.742  2.849  2.437  4.046  2.991  2.872  3.476  2.35  4.421  1.47  4.218  1.619  4.275  0.937  4.864  2.881  4.707  2.295  2.595  2.666  2.93  4.812  3.807  3.837  1.112  3.589  2.997  3.702  4.221  4.772  3.02  4.322  3.563  2.864  2.533  2.974  3.554  2.312  3.525  2.172  1.339  3.291  2.052  4.19  2.638  3.91  2.679  2.802  4.305  4.796  3.147  
 
 ARL6  84100  3  97483364  97520086  3q11.2  0.569  1.447  0.766  0.604  0.544  0.148  0.947  0.634  0.528  0.696  0.685  0.395  0.482  0.611  0.408  0.623  0.722  1.653  1.136  0.553  0  0.666  0.361  0.155  0.599  0.551  0.568  0.702  0.468  0.101  0.789  0.675  0.954  0.833  0.563  0.547  0.517  0.612  0.779  0.896  0.421  0.815  0.571  0.491  0.673  0.546  0.114  0.494  0.716  1.335  0.908  0.493  0.339  0.469  0.542  0.256  0.285  0.436  0.341  0.745  
 
 CRYBG3  131544  3  97540883  97663830  3q11.2  0.045  0.869  0.569  0.383  0.839  0.373  0.665  1.249  0.476  1.351  1.11  0.431  0.895  0.761  1.161  0.797  0.723  0.264  0.426  0.263  0.039  0.181  0.335  0.237  0.75  1.043  0.592  1.011  0.858  0.579  0.81  0.739  1.362  0.783  0.463  1.051  0.717  1.029  0.559  0.909  0.914  0.304  0.112  0.427  0.542  0.722  0.177  0.492  1.133  0.49  1.478  0.483  1.044  0.412  0.873  0.372  1.004  0.622  1.119  1.339  
 
 MINA  84864  3  97660660  97691295  3q11.2  1.035  3.145  1.537  1.352  1.948  0.81  2.035  1.622  1.578  1.851  1.809  1.258  1.766  2.643  1.588  1.953  1.478  2.703  2.896  1.427  1.491  1.647  1.726  1.306  1.973  1.694  1.686  1.802  1.351  1.307  2.038  2.119  2.253  1.429  1.275  1.735  1.972  1.897  1.867  2.342  1.747  1.06  1.425  1.742  1.467  1.678  0.667  1.091  1.88  2.517  2.051  0.972  1.812  0.969  1.543  1.073  1.264  1.487  1.776  2.328  
 
 GABRR3  200959  3  97705526  97754148  3q11.2  0  0  0  0  0  0  0  0  0  0  0  0  0  0  0  0  0  0.237  0  0  0  0.066  0  0  0  0  0.056  0  0  0.032  0  0  0  0  0  0  0  0  0  0  0  0  0  0  0  0  0  0  0  0  0  0.261  0  0  0  0  0  0  0  0  
 
 OR5AC2  81050  3  97806016  97806946  3q12.1  0  0  0.178  0  0  0  0  0  0  0  0  0  0  0.072  0  0  0  0  0  0  0  0  0  0  0  0  0.1  0  0  0.114  0  0  0  0.443  0  0  0  0  0  0  0  0  0  0  0  0  0  0  0  0  0  0  0  0  0  0  0  0  0  0  
 
 OR5H1  26341  3  97851541  97852483  3q11.2  0  0  0.189  0  0  0  0  0  0  0  0  0  0  0  0  0  0  0  0  0  0  0  0  0  0  0  0.11  0  0  0.061  0  0  0  0.224  0  0  0  0  0  0  0  0.285  0  0  0  0  0.246  0  0  0  0  0  0  0  0  0  0  0  0  0  
 
 OR5H14  403273  3  97868229  97869162  3q12.1  0  0  0  0  0  0  0  0  0  0  0  0  0  0  0  0  0  0  0  0  0  0  0  0  0  0  0  0  0  0  0  0  0  0.596  0  0  0  0  0  0  0  0.322  0  0  0  0  0  0  0  0  0  0  0  0  0  0  0  0  0  0  
 
 OR5H15  403274  3  97887543  97888485  3q12.1  0  0  0  0  0  0  0  0  0  0  0  0  0  0  0  0  0  0  0  0  0  0  0  0  0  0  0  0  0  0  0  0  0  0.284  0  0  0  0  0  0  0  0.103  0  0  0  0  0.343  0  0  0  0  0  0  0  0  0  0  0  0  0  
 
 OR5H6  79295  3  97983128  97984106  3q12.1  0  0  0  0  0  0  0  0  0  0  0  0  0  0  0  0  0  0  0  0  0  0.11  0.255  0  0  0  0.065  0  0  0.04  0  0  0  0.254  0  0  0  0  0  0  0  0  0  0  0  0  0  0  0  0  0  0  0  0  0  0  0  0  0  0  
 
 OR5H2  79310  3  98001731  98002676  3q12.1  0  0  0  0  0  0  0  0.086  0  0.061  0  0  0  0  0  0  0  0  0  0  0  0  0  0  0  0  0.164  0  0  0.15  0  0  0  0.486  0  0  0  0  0.066  0  0  0  0  0  0  0  0.069  0  0  0  0  0  0  0.099  0  0  0  0  0  0  
 
 OR5K4  403278  3  98072697  98073663  3q12.1  0  0  0.144  0  0  0  0  0  0  0  0  0  0  0  0  0  0  0  0  0  0  0  0  0  0  0  0  0  0  0.042  0  0  0  0.223  0  0  0  0  0  0  0  0  0  0  0  0  0  0  0  0  0  0  0  0  0  0  0  0  0  0  
 
 OR5K3  403277  3  98109509  98110475  3q12.1  0  0  0.18  0  0  0  0  0  0  0  0  0  0  0  0  0  0  0  0  0  0  0  0  0  0  0  0.035  0  0  0  0  0  0  0.447  0  0  0  0  0  0  0  0  0  0  0  0  0  0  0  0  0  0.19  0  0  0  0  0  0  0  0  
 
 OR5K1  26339  3  98188323  98189420  3q11.2  0  0  0.149  0  0  0  0  0  0  0  0  0  0  0.04  0  0  0  0  0  0  0  0.063  0  0  0  0  0  0  0  0.044  0  0  0  0.269  0  0  0  0  0  0  0  0  0  0  0  0  0  0  0  0  0  0  0  0  0  0  0  0  0  0  
 
 OR5K2  402135  3  98216524  98217475  3q11.2  0  0  0.201  0  0  0  0.181  0  0  0  0  0  0  0.07  0  0  0  0  0  0  0  0  0.263  0  0  0  0.092  0  0  0  0  0  0  0.408  0  0  0  0  0  0  0  0  0  0  0  0  0  0  0  0  0  0  0  0  0  0  0  0  0  0  
 
 CLDND1  56650  3  98234316  98241910  3q12.1  2.312  5.412  3.224  2.839  2.151  2.392  5.442  3.957  4.195  4.151  3.844  1.919  2.712  5.694  2.71  2.418  3.213  2.171  4.316  2.436  1.776  3.093  3.111  2.044  3.533  3.973  3.518  3.487  3.016  2.025  3.838  3.313  3.989  3.274  2.107  3.207  3.643  4.991  4.056  3.538  3.367  2.369  2.919  2.67  2.039  2.475  3.383  2.568  2.989  4.223  4.221  1.948  3.199  2.43  3.502  2.977  3.241  3.362  2.912  3.755  
 
 GPR15  2838  3  98250742  98251994  3q11.2-q13.1  0  0.391  0.357  0  0  0  1.692  0  0.172  0  0  0.808  0  1.288  0  0  0  0  0  0  0  0.05  0  0  0  0  0.031  1.157  0  0.039  0  0  0  0.368  0  0  0.176  0.147  0  0  0  0  0  0  0  0  0  0  0.376  0  0  0.268  3.393  0.085  0.546  0.683  2.228  0  0.135  2.461  
 
 CPOX  1371  3  98298289  98312455  3q12  1.467  4.002  2.038  0.925  1.716  1.192  3.895  1.56  1.599  2.491  2.234  1.571  2.275  6.252  2.282  1.762  3.124  2.206  3.749  2.026  1.59  1.875  1.472  1.053  2.1  3.063  3.026  2.917  2.229  3.101  3.368  2.871  3.498  2.649  1.202  2.033  2.272  3.898  2.128  2.193  1.276  1.661  1.566  1.959  1.193  1.594  0.576  1.441  2.183  3.226  2.867  1.358  2.804  1.31  2.021  1.607  2.725  1.546  1.881  2.855  
 
 ST3GAL6-AS1  100874207  3  98433176  98451495  -  0  0.654  0.539  0  0.259  0.274  0  0.175  0  0.426  1.072  0  0  0.219  0  1.793  0  0.572  0.318  0  0  0.197  0.48  0.454  0.472  1.3  0.644  0.555  1.35  1.29  1.504  0.726  1.201  0.842  0  0.228  0.471  0.895  1.85  0  0  0.51  0  0  0  1.235  2.455  0  0  0  0.66  0.257  0.186  0.153  1.683  1.135  0.73  0  0  0.701  
 
 ST3GAL6  10402  3  98451079  98514689  3q12.1  0  1.039  0.446  0  0.09  0.63  0.379  0.421  0.231  0.782  0.495  0.066  0.121  1.442  0.103  0.488  0.057  0.263  0.835  0.667  0  0.236  2.949  2.368  0.799  2.204  2.26  2.308  2.241  1.999  4.123  2.823  2.921  2.426  0.465  0.235  0.65  0.826  0.946  0.386  0.169  0.222  0.098  0.154  0.166  1.037  0.522  0.126  0.349  0.271  1.36  0.304  0.512  0.169  0.758  0.271  0.633  0.367  0.407  0.645  
 
 DCBLD2  131566  3  98514813  98620533  3q12.1|3  1.368  5.053  3.013  2.999  2.445  2.589  5.946  3.429  4.37  4.194  6.136  1.063  3.403  6.493  2.074  5.181  2.063  5.229  1.583  0.748  0.775  1.392  0.581  0.726  3.424  4.715  3.552  3.983  4.624  1.93  3.392  4.375  3.479  2.598  4.842  3.959  4.207  5.744  5.421  1.852  2.963  3.214  1.192  1.687  3.702  2.896  5.463  1.678  3.7  3.572  5.393  4.014  5.479  3.765  5.496  5.435  4.1  3.695  5.77  7.638  
 
 COL8A1  1295  3  99357439  99515577  3q12.3  0  2.398  5.63  0.61  0.027  2.232  3.235  3.476  1.092  6.077  4.278  0  0  0.04  0  0.048  0  0.085  0  0.07  0  0.188  0.362  0  0.084  0  0.235  3.645  0.065  0.062  0  0.4  2.241  2.646  0  0.047  0.881  2.747  3.338  0  0.085  4.579  0.022  0  0.529  3.284  0.045  0.463  1.677  1.013  0.62  0.23  3.327  2.934  4.633  3.794  2.803  1.175  0  5.516  
 
 HP09053  101929357  3  99535475  99542709  -  0.505  0.758  1.243  1.294  0.614  0.637  0.962  0.391  0.616  0.677  0.358  0.504  0.314  0.477  0.276  0.898  0.433  1.06  0.626  1.605  0.387  1.885  1.237  0.12  0.118  0.224  0.631  0.364  0.198  0.182  0.444  0.405  0.522  0.949  0.544  0.184  0.411  0.419  1.314  0.278  0.197  0.904  0.974  0.294  0.217  0.392  1.991  0.221  0.385  0.283  0.339  0.637  0.57  0.141  0.921  0.364  0.278  0.353  0.241  0.209  
 
 CMSS1  84319  3  99536677  99897476  3q12.1  2.512  6.436  3.217  2.839  3.395  2.564  4.119  3.077  2.212  3.328  3.658  2.638  2.605  3.921  2.201  3.61  3.047  6.73  5.298  4.726  3.162  4.347  3.596  2.776  3.605  2.486  2.569  2.766  1.732  1.877  3.959  4.033  3.755  2.85  2.868  2.819  3.216  3.401  4.157  3.379  2.641  2.995  3.527  2.972  2.902  3.339  3.132  1.554  3.711  4.073  4.028  1.683  3.243  2.073  3.388  3.07  2.515  3.944  3.09  3.917  
 
 FILIP1L  11259  3  99551987  99833357  3q12.1  1.41  2.453  1.158  0.242  1.574  2.087  2.109  3.304  3.484  1.969  3.361  1.237  1.958  0.749  2.071  1.919  1.798  1.209  2.019  1.252  2.309  1.649  0.428  1.934  2.049  2.139  1.359  1.755  0.908  1.241  1.554  1.608  1.364  0.42  1.34  1.757  2.325  1.977  1.849  2.015  1.783  1.581  1.144  1.967  1.901  2.304  1.396  1.777  1.935  2.633  2.139  1.307  2.11  2.28  1.571  1.959  2.932  1.473  2.512  4.361  
 
 TMEM30C  644444  3  99904667  99913030  3q12.1  0  0  0  0  0  0  0  0  0  0  0  0  0  0  0  0  0  0  0  0  0  0  0  0  0  0  0  0  0  0  0  0  0  0  0  0  0  0  0  0  0  0  0  0  0  0  0.487  0  0  0  0  0  0  0  0  0  0  0  0  0  
 
 TBC1D23  55773  3  99979660  100044096  3q12.2  0.902  2.696  1.805  1.145  1.589  1.225  1.705  2.208  1.469  2.153  1.917  0.964  1.426  1.895  1.066  1.425  1.496  1.497  2.202  1.558  1.731  0.965  0.82  0.739  1.47  1.956  1.172  1.491  1.068  0.316  1.346  1.378  1.835  1.348  0.777  1.18  1.358  1.64  3.058  1.672  1.055  0.771  0.934  0.965  1.042  1.37  0.193  0.725  1.448  1.819  2.491  0.839  1.241  1.28  1.047  1.515  1.497  1.45  1.377  2.378  
 
 NIT2  56954  3  100053561  100074478  3q12.2  2.84  4.915  2.193  2.626  3.982  1.718  3.617  3.05  1.82  3.164  3.389  3.178  3.042  4.06  1.924  3.589  2.971  3.205  4.31  4.843  1.983  3.174  2.38  2.256  2.723  3.391  2.812  2.719  2.677  2.217  4.468  4.042  3.511  2.174  3.169  3.155  2.909  3.395  4.545  3.666  2.954  2.49  3.524  3.122  2.353  3.466  2.382  0.806  3.071  2.911  3.838  1.697  2.532  2.493  3.928  3.145  2.609  3.337  3.075  4.118  
 
 TOMM70A  9868  3  100082302  100120242  3q12.2  2.602  3.706  2.252  2.551  3.151  1.565  2.941  2.716  2.107  2.623  2.613  2.638  3.365  3.451  2.086  2.849  2.655  3.193  3.931  2.96  2.688  2.616  2.374  2.254  2.591  2.772  2.39  3.13  2.092  2.409  3.179  2.98  3.762  2.923  2.192  2.408  2.749  2.547  2.957  3.186  2.191  2.184  2.419  2.599  2.464  2.359  1.918  1.213  2.128  2.673  3.699  1.252  2.706  2.08  2.166  2.59  2.149  2.329  2.51  2.991  
 
 LNP1  348801  3  100120036  100175170  3q12.2  0.433  0.301  0.49  0.381  0.406  0.372  0.729  0.789  0.644  1.122  0.94  0.835  0.582  0.332  0.151  0.382  0.203  1.374  1.619  0.326  0.728  0.89  0.773  0.29  0.332  0.505  0.441  1.315  0.38  0.688  0.266  0.425  0.92  0.793  0.309  0.26  0.541  0.42  0.535  0.673  0.453  1.04  0.058  0.166  0.732  0.261  0.038  0.174  0.636  0.775  2.529  0.795  0.726  0.391  0.732  0.847  0.244  0.228  0.351  0.456  
 
 TMEM45A  55076  3  100211462  100296285  3q12.2  0  1.576  3.593  1.412  0.176  1.037  1.208  2.545  2.427  3.134  3.517  0  0.214  1.483  0.177  0  0  0.404  0  0  0  0.293  0.976  0  0.448  1.429  1.2  1.478  1.372  0.335  1.084  1.851  2.069  1.428  0  1.15  0.91  0.306  0.149  0.967  0.553  1.593  1.519  0.689  0.236  2.02  0.035  0.115  0.246  1.445  3.137  0.413  0.503  2.224  0.454  1.558  2.892  0.42  0.127  1.525  
 
 ADGRG7  84873  3  100328432  100414325  3q12.2  0  0.044  0.079  0  0  0  3.277  0  0  0  0  0.087  0  0.038  0  0.038  0  0  0  0  0  0.065  0  0  0.051  0  0.029  1.686  0  0.035  0  0  0  0.178  0  0  0  0  0.019  0  0  0.139  0  0  0  0  0  0  0  0  0  0.188  0  0  0.023  0  0  0  0  0  
 
 TFG  10342  3  100428133  100467811  3q12.2  4.949  5.703  5.014  4.871  5.736  3.435  5.976  5.784  4.42  5.798  5.868  4.574  4.79  5.925  4.286  5.155  5.032  3.756  5.473  4.744  3.95  4.672  3.883  3.582  5.12  4.937  5.35  5.1  4.841  4.563  5.572  5.59  5.801  4.298  4.408  5.031  5.316  5.026  5.921  5.863  5.245  4.175  5.176  4.846  4.919  4.689  4.438  3.654  4.657  5.565  5.694  3.628  5.235  4.726  5.241  5.644  4.725  5.319  5.257  5.4  
 
 ABI3BP  25890  3  100468178  100712334  3q12  0.056  0.299  1.912  2.927  0.066  0.066  0.234  0.867  3.02  3.42  0.487  0.065  0  0.119  0.064  0.056  0.054  0.041  0.046  0.082  0  0.189  0  0  0.21  0.097  0.073  0.088  0.034  0.06  0  0.134  0.028  0.171  0.051  0.041  0.145  0.279  3.076  0  0.032  0.893  0.027  0.073  0.082  0.054  0.16  0  0  0.098  3.508  0.064  0.109  0.237  0.128  3.71  0.832  0.549  0.048  0.278  
 
 IMPG2  50939  3  100941389  101039419  3q12.2-q12.3  0.03  0.023  0.117  0  0.043  0.033  0.076  0.049  0.064  0.043  0.043  0.063  0  0.077  0.028  0.015  0.026  0.055  0.07  0.03  0.027  0.037  0.19  0  0  0.017  0.085  0.076  0  0.085  0.029  0.045  0.017  0.222  0.035  0.025  0  0.041  0.053  0.029  0.029  0.161  0.044  0.154  0.037  0.047  0.061  0.055  0.037  0.045  0.049  0.102  0.013  0.038  0.023  0.042  0.03  0.021  0.014  0  
 
 SENP7  57337  3  101043032  101232085  3q12  0.579  1.507  0.74  0.667  1  0.823  1.097  1.014  0.749  1.349  0.754  0.404  0.55  2.298  0.494  0.298  1.143  1.906  2.002  0.566  1.417  0.909  0.437  0.086  0.236  0.697  0.587  0.581  0.37  0.282  0.555  0.302  0.941  0.716  0.607  0.811  0.374  0.604  0.806  0.694  0.804  0.318  1.249  1.276  0.207  0.683  0.27  0.418  0.606  0.657  1.099  0.504  0.593  0.762  0.561  0.661  0.547  0.819  0.316  0.909  
 
 FAM172BP  131909  3  101237710  101242731  3q12.3  0  0.069  0.199  0  0  0  0  0  0.073  0  0  0  0  0  0  0.042  0  0  0  0  0  0.04  0.428  0  0  0  0.043  0.025  0  0.043  0  0  0  0.421  0  0  0  0  0.079  0  0  0.053  0.026  0  0  0.081  0.05  0  0  0.272  0.223  0.129  0.03  0  0  0  0  0  0  0  
 
 TRMT10C  54931  3  101280679  101285290  3q12.3  3.281  4.957  2.086  3.537  4.319  1.874  2.897  2.735  2.46  2.431  2.912  3.255  3.166  4.597  2.615  3.661  3.193  4.885  4.363  4.622  3.282  3.704  2.746  2.224  3.514  2.762  2.394  2.725  2.43  2.256  3.179  3.276  3.857  3.666  2.804  2.345  2.16  1.975  3.942  2.956  2.368  2.705  2.924  3.331  2.922  2.695  2.184  1.448  2.41  3.088  3.007  1.566  2.423  1.725  2.819  2.754  1.728  2.761  2.63  3.283  
 
 PCNP  57092  3  101293041  101313281  3q12.3  1.215  3.534  2.878  2.788  3.12  2.337  2.37  2.706  3.129  2.786  3.362  2.573  2.853  3.528  2.192  3.441  3.278  3.878  4.771  4.068  2.793  3.407  2.43  1.886  3.159  2.845  1.562  1.887  2.179  1.393  3.224  2.76  2.897  2.71  2.378  2.545  2.59  3.041  4.49  2.963  2.507  2.496  2.161  3.167  2.346  2.378  1.53  1.236  3.129  3.467  3.689  1.738  2.265  2.176  3.051  2.72  2.56  3.472  3.096  3.943  
 
 ZBTB11  27107  3  101368282  101395988  3q12.3  1.282  1.284  0.974  1.072  1.917  0.819  1.427  1.628  1.163  1.463  1.371  0.915  1.194  1.528  1.648  1.511  1.628  0.916  2.841  1.934  1.509  1.897  0.699  0.793  1.393  1.389  0.838  1.792  1.178  0.807  1.544  1.683  1.679  1.199  0.918  1.487  1.514  1.244  1.994  2.067  1.118  0.966  1.17  1.429  1.277  1.095  1.088  0.73  1.147  1.466  2.265  0.844  1.48  0.959  1.312  1.303  1.425  1.503  1.566  1.904  
 
 ZBTB11-AS1  100009676  3  101395273  101398057  3q12.3  0.665  0.706  0.793  0.587  0.86  0.25  0.909  0.634  0.905  0.651  0.647  0.244  0.702  0.689  0.497  0.708  0.687  0.523  1.051  0.646  1.319  0.769  1.188  0.808  0.59  0.806  0.637  1.223  0.496  0.273  1.298  1.116  1.21  1.008  0.68  0.702  0.603  0.79  1.148  1.152  0.564  0.778  0.619  0.834  0.943  0.626  0.595  0.299  0.639  0.716  0.663  0.857  0.664  0.316  0.504  0.56  0.315  0.721  0.453  0.685  
 
 RPL24  6152  3  101399933  101405563  3q12  7.187  10.561  6.402  6.045  8.683  7.08  7.564  7.363  6.611  7.549  7.454  7.63  6.836  8.836  5.948  8.308  8.551  10.977  9.821  9.04  7.849  8.375  7.533  7.125  8.256  6.218  5.725  5.666  6.073  5.11  8.066  7.489  7.164  6.097  7.229  6.551  6.544  6.597  9.255  6.661  6.892  7.458  7.054  8.748  7.411  7.642  6.101  5.318  7.57  8.33  7.319  5.562  6.616  6.548  8.076  7.414  6.115  8.021  7.106  8.244  
 
 PDCL3P4  285359  3  101431277  101432260  3q12.3  1.445  1.011  0.99  0.685  1.034  0.793  0.668  0.407  1.292  0.621  0.766  0.275  0.74  1.126  1.432  0.63  0.522  0.684  1.522  0.248  0.313  0.918  0.759  0.513  0.481  0.504  0.915  0.564  1.067  0.462  0.889  0.944  0.625  1.038  0.663  0.531  0.517  0.57  0.603  0.536  0.562  0.835  1.121  0.58  0.451  0.827  1.962  0.723  0.34  1.194  0.844  0.273  0.703  0.923  0.865  0.697  0.718  0.233  0.538  0.245  
 
 CEP97  79598  3  101443436  101489406  3q12.3  0.533  1.535  0.74  0.81  0.797  0.448  0.89  1.053  0.515  1.251  1.153  0.334  0.641  0.851  1.059  0.474  0.548  0.645  1.909  0.479  0.433  0.868  0.593  0.312  0.728  1.196  0.787  1.227  0.714  0.608  0.844  0.677  1.651  0.995  0.454  0.476  0.713  0.666  0.758  1.101  0.338  0.375  0.715  1.051  0.523  0.508  0.218  0.403  1.013  0.77  1.413  0.605  0.881  0.463  0.534  0.693  0.624  0.774  0.507  0.911  
 
 NXPE3  91775  3  101498028  101547075  3q12.3  0.39  1.574  0.806  0.556  0.424  0.57  1.695  1.467  1.169  1.537  1.873  0.139  0.921  0.632  0.474  0.144  0.221  0.453  1.964  0.353  0.148  1.222  1.014  0.714  0.995  0.938  0.574  0.738  0.955  0.382  0.869  0.677  1.071  0.869  1.083  0.283  1.302  1.475  0.471  0.88  0.223  1.061  0.575  0.357  0.513  0.375  0.396  0.373  1.02  0.969  1.281  0.526  1.313  1.535  1.29  1.215  1.436  1.592  0.875  1.818  
 
 NFKBIZ  64332  3  101546833  101579869  3p12-q12  1.023  2.369  1.371  2.662  1.123  0.434  1.582  2.386  0.458  1.571  1.034  1.179  1.114  1.05  0.761  2.551  1.108  0.342  0.686  0.165  0.651  0.511  0.431  0.519  2.596  2.321  2.861  1.895  2.286  3.737  3.318  1.981  2.488  1.392  0.448  1.493  2.16  1.471  2.124  2.057  1.088  0.571  0.617  3.188  0.791  1.547  2.131  0.48  1.341  0.891  2.358  0.647  3.069  1.687  4.638  3.495  3.849  1.622  4.075  3.805  
 
 LOC152225  152225  3  101659702  101716770  3q12.3  0  2.248  0.202  0.165  0  0.097  0.084  0.214  0.108  0.038  0.134  0.077  0  0.029  0  0.046  0  0  0  0  0  0.024  0.253  0  1.048  0.098  0.994  0.762  0.588  0.227  1.252  0.226  0.556  0.618  0  0  1.34  0.175  0.047  0  0.073  0.318  0.023  0  0  0  0.336  0  0  0.178  0.77  0  0.291  0  0.16  0.36  0.238  0.098  0  1.532  
 
 ZPLD1  131368  3  102153858  102198685  3q12.3  0.049  0.038  0.103  0  0.098  0  0.138  0  0.093  0.144  0.064  0.039  0.095  0.054  0.108  0.038  0.03  0.028  0.088  0.055  0.122  0.145  0.188  0.102  0.131  0.119  0.071  0.191  0  0.087  0  0  0  0.262  0.053  0.106  0.129  0  0.034  0.126  0  0.295  0.576  0.223  0  0.03  0.057  0  0.151  0.101  0.163  0.134  0.074  0.051  1.212  0.19  0  0  0.125  1.493  
 
 ALCAM  214  3  105085556  105295757  3q13.1  0.966  5.623  4.334  4.667  2.137  2.789  3.352  3.839  2.601  2.584  4.204  2.9  2.047  3.558  1.405  0.272  4.094  3.208  1.24  2.888  0.15  1.223  0.351  0.153  4.845  1.204  1.462  3.563  1.409  1.437  0.202  1.527  0.4  0.605  2.651  3.27  3.762  3.514  4.916  1.038  3.809  2.261  2.752  4.242  2.205  3.999  3.148  2.796  4.747  4.125  5.183  2.571  4.662  3.498  3.559  4.451  3.559  3.585  1.504  5.523  
 
 CBLB  868  3  105377108  105587887  3q13.11  1.026  1.408  2.337  1.13  0.929  0.453  1.848  2.041  0.932  2.989  1.404  0.561  0.938  1.047  0.729  0.789  1.862  1.37  1.423  0.729  0.536  1.347  0.148  0.196  1.604  1.875  1.687  1.791  1.857  1.773  1.535  2.957  1.549  0.985  0.542  1.519  2.17  0.922  1.15  1.422  1.375  0.658  1.137  2.153  2.611  2.018  0.168  0.18  1.337  0.438  2.265  0.454  0.765  0.741  1.003  1.284  1.223  0.494  0.84  2.892  
 
 LINC00882  100302640  3  106828636  106959485  3q13.12  0  0  0.586  0  0  0.367  0.401  0.399  0.264  0.501  0.194  0  0  0.361  0  0  0  0  0.354  0  0  0.503  0  0  0  0  0.392  0.779  0  3.839  0.676  0.267  0  0  0  0.226  0.578  0  0.233  0.364  0  0  0  0  0  0.922  0  0  0  0  0.171  0  0  0.128  0  0  0  0  0  0.246  
 
 DUBR  344595  3  106959538  107045811  3q13.12  0.714  1.566  1.456  1.222  0.921  0.638  1.551  0.799  0.848  1.016  0.922  0.502  0.662  0.944  0.541  0.315  0.575  0.276  1.038  1.103  0  1.313  0.572  0.278  0.963  1.185  0.774  1.242  0.715  4.145  1.522  1.083  1.307  1.195  0.655  0.789  1.586  1.249  1.216  0.866  0.292  0.405  0.367  0.536  0.656  0.713  0.405  0.859  0.733  1.996  0.861  0.562  0.662  0.494  1.06  0.823  0.621  1.261  0.593  0.939  
 
 CCDC54  84692  3  107096187  107097481  3q13.12  0  0  0.169  0  0.059  0.092  0  0.165  0  0.157  0.084  0.117  0  0  0  0  0  0  0.073  0  0  0.077  0.449  0  0  0.115  0.084  0.131  0  0.091  0.102  0.112  0  0.357  0  0  0.094  0  0.054  0  0  0.098  0.053  0  0.173  0.207  0.119  0  0  0  0.09  0  0.102  0.085  0  0  0  0.17  0  0.12  
 
 LOC101929579  101929579  3  107099322  107101006  3q13.12  0  0  0  0  0  0.344  0  0  0  0  0  0  0  0  0  0  0  0  0  0  0  0  0  0  0  0  0  0  0  0  0  0  0  0  0  0  0  0  0  0  0  0  0  0  0  0  0  0  0  0  0  0  0  0  0  0  0  0  0  0  
 
 LOC101929607  101929607  3  107149776  107182759  -  0  0.086  0.09  0  0  0  0  0  0  0  0  0  0  0  0  0  0  0  0  0  0  0  0  0  0  0  0.057  0  0  0.044  0  0  0  0.229  0  0  0  0  0.041  0  0  0  0  0  0  0.079  0.07  0  0  0  0  0  0  0  0  0  0  0  0  0  
 
 BBX  56987  3  107241782  107530176  3q13.1  1.569  3.674  2.434  2.165  2.449  1.485  3.122  2.712  2.909  2.887  3.192  1.082  2.433  3.361  2.293  2.065  2.779  2.314  2.491  2.1  0.744  2.265  1.587  1.457  2.7  2.558  2.324  2.479  2.05  2.364  2.454  3.406  3.032  2.283  1.798  1.996  2.943  2.428  2.803  3.034  2.519  1.832  0.586  2.453  2.044  2.858  1.462  1.826  2.629  3.026  2.938  1.666  2.265  1.309  2.215  2.26  2.38  2.285  1.596  3.276  
 
 LINC00635  151658  3  107560508  107596915  3q13.12  0  0  0  0  0  0  0  0  0  0  0  0  0  0  0  0  0  0  0  0  0  0.05  0  0  0  0  0.051  0  0  0.042  0  0  0  0.287  0  0  0  0  0  0  0  0  0  0  0  0.069  0.038  0  0  0  0  0  0  0  0  0  0  0  0  0  
 
 LINC00636  285205  3  107602051  107647753  3q13.12  0  0  0.104  0  0  0  0  0.08  0  0.049  0  0.077  0  0.061  0  0  0  0  0  0  0  0.16  0.415  0  0  0  0.073  0  0  0.167  0  0  0  0.194  0  0  0  0  0.064  0  0  0.452  0  0  0.574  0.134  0.146  0  0  0  0  0.222  0  0.063  0  0  0.087  0  0  0  
 
 CD47  961  3  107761940  107809935  3q13.1-q13.2  3.274  3.668  1.388  2.697  2.656  1.365  4.063  2.431  1.885  3.232  2.154  1.614  2.106  2.257  2.151  1.503  2.074  2.598  5.781  3.432  1.33  3.916  2.364  1.987  1.904  1.673  1.606  1.552  1.357  1.715  2.291  2.525  2.128  1.706  1.143  1.004  1.724  2.475  2.907  1.878  1.499  1.099  0.32  1.906  4.36  3.742  2.028  1.813  2.344  3.825  3.469  1.074  2.398  2.14  1.661  2.311  2.738  1.811  1.915  2.135  
 
 LINC01215  101929623  3  107843910  107857457  -  0  0  0.091  0  0.053  0  0.059  0.039  0.083  0.051  0.056  0.054  0  0.041  0  0.08  0.081  0  4.511  1.39  0.24  2.505  1.311  0.242  0  0  0.033  0  0  0.048  0  0  0  0.143  0.076  0  0  0  0.067  0  0  0.035  0.019  0.068  0  0.103  0.045  0  0  0.059  0.046  0.302  0  0.096  0.032  0  0.082  0  0  0  
 
 IFT57  55081  3  107879658  107941417  3q13.13  1.814  3.902  1.393  1.765  2.112  0.947  2.911  2.164  1.977  2.295  2.242  0.862  1.244  2.877  1.54  0.95  1.141  3.727  2.827  2.937  0.614  2.002  0.996  0.909  1.492  1.08  1.078  1.277  1.124  0.837  1.587  2.798  1.593  1.201  1.357  1.041  1.448  2.172  2.897  1.8  1.278  1.344  1.501  1.59  3.548  2.104  1.659  0.872  2.066  3.131  2.897  0.89  1.924  1.557  2.093  1.734  1.39  1.586  1.067  2.332  
 
 HHLA2  11148  3  108015336  108097131  3q13.13  0  0  0.123  0  0  0  0  0  0  0  0  0  0.297  0.024  0  0.062  0.131  0  0.32  0.079  0  0.09  0.125  0  0  0  0.057  0  0  0.03  0  0.061  0  0.18  0  0  0  0  0.035  0  0  0.193  0.031  0  0  0  0.029  0  0  0  0.205  0.09  0  0.132  0  0  0  0  0  0  
 
 MYH15  22989  3  108099215  108248169  3q13.13  0.034  0.047  0.12  0.149  0.01  0  0.153  0.032  0.04  0.025  0.194  0.045  0.091  0.961  0  0.024  0.046  0.092  0.1  0.186  0  0.08  0.181  0  0.219  0.016  0.085  0.075  0.028  0.057  0  0.108  0.028  0.266  0.017  0.034  0.051  0.306  0.098  0.695  0.074  0.163  0.064  0.313  0.04  0.032  0.257  0.041  0.061  0.088  1.554  0.261  0.176  0.069  0.075  0.073  0.039  0.156  0.025  0.061  
 
 KIAA1524  57650  3  108268717  108308491  3q13.13  1.33  3.568  0.951  0.663  1.133  1.064  1.597  1.663  0.203  1.669  2.159  0.925  1.38  2.281  1.337  1.369  1.27  2.606  2.071  0.966  1.196  1.301  0.376  0.601  1.64  1.396  0.951  1.315  1.128  0.654  1.705  1.607  2.169  1.085  1.287  1.372  1.481  1.466  1.507  2.622  1.3  1.126  1.336  1.579  1.367  1.728  0.344  1.574  2.017  1.771  2.199  0.946  1.726  1.102  1.394  1.166  1.584  1.547  1.767  2.177  
 
 DZIP3  9666  3  108308336  108413693  3q13.13  1.1  1.384  0.59  0.62  1.43  0.458  0.899  1.098  0.297  1  1.067  0.224  0.741  2.046  0.307  0.442  0.87  1.26  1.477  0.106  0.358  0.782  0.253  0.11  0.589  1.231  0.554  1.236  0.936  0.717  1.607  1.573  1.116  0.678  0.494  0.948  1.06  0.871  0.692  2.058  1.37  0.289  0.917  0.894  0.852  1.182  0.603  0.468  0.875  1.467  1.146  0.686  0.921  0.402  1.182  0.637  0.615  0.305  0.499  1.529  
 
 RETNLB  84666  3  108474485  108476130  3q13.1  0  0  0.261  0  0  0  0  0  0  0  0  0  0  0  0  0  0  0  0  0  0  0  0  0  0  0  0  0  0  0  0  0  0  0.454  0  0  0  0  0  0  0  0.923  0  0  0  0  0  0  0  0  0  0.346  0  0  0  0  0  0  0  0  
 
 TRAT1  50852  3  108541630  108573714  3q13  0  0  0  0  0  0  0.069  0  0  0.065  0  0  0  0  0  0  0  0  5.037  0  0  3.18  0.143  0  0  0  0.033  0  0  0.028  0  0  0  0.2  0  0  0  0  0.035  0  0  0  0  0  0  0  0  0  0  0.074  0  0  0  0  0  0  0  0  0  0  
 
 GUCA1C  9626  3  108626641  108672677  3q13.1  0  0  0  0  0  0  0  0  0  0  0  0  0  0  0  0  0  0  0  0  0  0  0  0  0  0  0.075  0  0  0.064  0  0  0  0.234  0  0  0  0  0.078  0  0  0  0  0.509  0  0  0.067  0  0  0  0  0  0  0  0  0  0  0  0  0  
 
 MORC1  27136  3  108677086  108836993  3q13  0  0  0.074  0  0  0  0  0  0  0  0.017  0.025  0  0.017  0  0  0  0  0  0  0  0.091  0.652  0.35  0  0  0.078  0.034  0.869  0.055  0.802  0.099  0  0.102  0  0  0  0  0  0  0  0.078  0.018  0  0  0  0.042  0  0  0  0  0.112  0  0.021  0  0  0  0  0  0  
 
 FLJ22763  401081  3  108855560  108868951  3q13.13  0  0  0  0  0  0  0  0.039  0  0  0  0.483  0  0.03  0  0  0  0.09  0  0  0  0.063  0  0  0  0  0.061  0.102  0.599  0.044  0.512  0.143  0  0.159  0  0  0  0  0  0  0  0.03  0  0  0  0  0  0  0  0  0  0  0  0  0  0  0  0  0  0  
 
 LINC00488  677779  3  108897011  108904108  3q13.13  0  0  0.126  0  0  0  0.419  0  0.1  0  0  0  0  0  0  0  0  0  0  0  0  0.022  0.169  0  0  0.215  0.079  0.984  1.902  0.021  1.983  0.269  0.371  0.358  0.089  0  0  0  0  0  0  0.254  0  0.201  0  0  0.025  0  0  0  0  0  0  0.1  0  0.189  0  0  0  0  
 
 DPPA2  151871  3  109012634  109035364  3q13.13  0  0  0  0  0  0  0  0  0  0  0  0  0  0  0  0  0  0  0  0  0  0  0  0  0  0  0  0  0.135  0.039  0  0  0  0.269  0  0  0  0  0  0  0  0  0.036  0.239  0  0  0  0  0  0  0  0  0  0  0  0  0  0  0  0  
 
 DPPA4  55211  3  109044987  109056419  3q13.13  0  0.027  0.124  0  0  0.137  0  0  0.078  0  0  0  0  0  0  0  0.045  0.082  0  0  0  0.049  0.211  0  0  0  0.029  0.063  0  0.037  0  0  0  0.171  0  0  0  0  0.015  0  0  0  0  0  0  0  0.034  0  0  0  0  0.07  0  0  0  0  0  0  0  0  
 
 LINC01205  401082  3  109128836  109214014  3q13.13  0  0  0.272  0  0.121  0  0.059  0  0.187  0  0  0  0  0.037  0  0  0  0  0  0  0  0.076  0.513  0  0  0  0.077  0.72  0.226  0.049  0  0  0  0.44  0.095  0  0  0  0.055  0  0  0.048  0.054  0  0  0  0.049  0  0  0  0  0.143  0  0.045  0  0  0  0  0  0  
 
 PVRL3-AS1  100506555  3  110764162  110788806  -  0  0.307  0.121  0  0  0  0  0  0  0.125  0  0  0.129  0.193  0  0  0  0  0  0  0  0.053  0  0  0  0  0  0  0.095  0.033  0  0  0  0.275  0  0  0  0  0  0  0.159  0  0  0  0  0.114  0  0  0  0  0  0  0.151  0  0.257  0  0  0.337  0  0.136  
 
 PVRL3  25945  3  110790605  110913016  3q13  0  3.2  1.708  1.576  0.179  1.279  2.756  2.038  1.382  2.746  2.337  0  2.275  3.745  0.818  0.325  1.072  0.104  1.399  0.058  0.082  0.769  0.188  0  2.381  0.121  0.968  1.857  1.085  0.71  0.877  1.379  1.96  1  1.498  1.818  2.47  1.845  1.785  0.503  1.577  1.384  0.058  2.47  2.058  1.33  1.577  1.33  2.821  2.178  1.717  0.912  2.433  1.283  2.278  1.726  2.256  2.18  1.848  2.882  
 
 CD96  10225  3  111260925  111371206  3q13.13-q13.2  0  0  0.117  0  0  0  0.543  0  0.089  0.165  0.049  0.023  0  0.075  0  0.021  0  0  2.547  0.047  0.069  3.209  0.256  0  0  0.07  0.034  2.299  0  0.138  0.029  0.389  0.16  0.321  0  0  1.102  0  0.018  0  0  0.022  0.01  0.067  0.044  0.017  0.024  0  0.074  0.028  0.044  0.186  0  0.015  0.109  0.061  0  0  0.073  0.112  
 
 ZBED2  79413  3  111311746  111314182  3q13.2  0  0  0.204  0  0  0  0.482  0  0.15  0.036  0.039  0  0  1.857  0  0.428  0  0  0.137  0  0  1.466  0.445  0  0  0  0.056  0.35  0  0.102  0  0  0  0.478  0.15  0  0.139  0  0.064  0  0.45  0  0.043  2.179  2.327  1.408  0.308  0  3.374  0.123  0.147  0.505  0.764  0.156  3.615  1.508  0  0.232  2.701  4.108  
 
 PLCXD2  257068  3  111393522  111565294  3q13.2  0  0  0.183  0  0.079  0.267  0.254  0.095  0.124  0.036  0.197  0.146  0.48  0.478  0.148  0.276  0.254  0  0.111  0.07  0  0.155  0.44  0  0.246  0.069  0.055  0.433  0.067  0.163  0.205  0.098  0.273  0.413  0.261  0.267  0.658  0.216  0.484  0.125  0.566  0.226  0.046  0.222  0.236  0.591  0  0.413  0.585  0.239  1.334  0.487  0.433  0.181  0.657  0.288  0.152  0.297  0.669  0.816  
 
 PLCXD2-AS1  100874115  3  111395112  111396280  -  0  0.108  0  0  0  0  0  0  0  0  0  0.127  0.212  0.391  0  0.133  0.172  0  0  0  0  0  0.307  0  0  0  0  0.134  0  0  0.155  0  0  0.357  0  0  0.126  0  0.258  0  0.145  0  0  0  0  0.146  0  0  0  0.089  0.319  0  0  0.064  0.276  0  0  0  0  0.138  
 
 PHLDB2  90102  3  111451326  111695364  3q13.2  0.07  2.979  2.036  1.835  0.346  1.81  2.02  2.171  0.964  2.272  1.755  0.033  0.203  4.125  0.537  0.058  0.288  0.058  0.09  0.074  0  0.337  0.196  0  2.819  0.347  0.238  0.469  0.185  0.421  0.99  1.385  1.342  0.658  1.308  2.898  2.583  2.868  3.817  1.367  1.599  0.308  0.087  1.228  1.067  3.466  0.599  1.797  1.836  2.128  2.466  1.387  2.478  0.676  2.862  1.707  2.344  2.456  3.261  3.8  
 
 ABHD10  55347  3  111697722  111712215  3q13.2  2.074  3.58  2.222  2.018  2.565  1.001  2.803  1.769  1.894  2.294  2.42  1.963  2.421  5.582  2.334  2.619  2.795  3.373  4.134  3.678  1.29  3.094  1.106  0.636  2.025  2.556  2.571  1.763  2.311  2.057  3.031  2.976  2.783  1.993  1.656  1.762  2.058  2.056  2.913  2.558  1.664  1.798  1.796  1.92  1.384  1.979  2.71  1.89  1.773  3.993  3.279  1.273  2.029  1.26  2.485  2.115  1.365  1.371  1.935  2.902  
 
 TAGLN3  29114  3  111717585  111732735  3q13.2  0  0.179  0.254  0  0.065  0  1.245  0  0  0.057  1.396  0  0  0.119  0  0  0  0  0.138  0  0  0.192  0.29  0  0  0  0.081  0  0  0.058  0  0  0  0.51  0  0  1.241  0.401  0.16  0  0  0.09  0.054  0  0.432  0.168  0.041  0  0  0  2.432  0.534  0.54  0.238  3.163  0.571  0.167  0  1.673  1.761  
 
 TMPRSS7  344805  3  111758464  111800116  3q13.2  0  0  0.135  0  0.065  0  0  0  0.056  0  0  0  0  0.087  0  0.089  0  0  0  0.068  0  0  0.219  0  0  0  0.05  0  0  0.026  0  0  0  0.154  0.059  0  0  0  0.043  0  0  0.162  0.023  0  0  0  0.019  0  0  0  0.096  0.259  0  0.057  0.082  0  0  0  0  0.127  
 
 C3orf52  79669  3  111805181  111837073  3q13.2  0.268  1.556  1.061  0.568  1.204  0.524  0.612  2.054  0.069  0.266  0  1.225  1.165  1.771  0.835  1.438  1.024  0.983  0.38  0  0.801  0.128  0.204  0.052  1.96  0.41  0.817  0.875  0.489  0.881  1.375  1.043  0.907  0.72  0.756  1.16  1.596  1.505  1.934  0.513  0.972  0.469  0.046  0.539  1.195  0.999  1.728  0.847  0.691  2.059  1.547  0.576  1.746  0.65  1.868  1.019  1.062  0.284  2.018  2.132  
 
 GCSAM  257144  3  111839687  111852152  3q13.2  0  0.044  0.187  0  0.193  0.05  0.062  0.283  0.097  0.082  0  0.117  0.106  0.362  0.119  0.083  0.082  0  0.246  0.233  0  0.528  0.128  0.064  0.148  0  0.119  0.094  0.082  0.189  0.062  0.068  0.166  0.314  0.073  0.2  0.161  0.357  0.149  0.085  0.135  0.027  0.014  0  0  0.345  0.159  0.242  0.083  0.29  0.376  0.159  0.218  0.059  0.18  0  0.293  0  0.576  0.226  
 
 SLC9C1  285335  3  111859751  112013074  3q13.2  0  0  0.044  0  0  0  0  0  0  0  0  0  0  0.061  0  0  0  0.062  0  0  0  0.012  0  0  0  0  0.046  0  0  0.035  0.039  0  0  0.094  0  0  0  0  0.063  0  0  0.166  0.018  0  0  0.02  0.031  0  0  0  0  0.06  0.034  0.032  0.019  0  0  0  0  0.035  
 
 CD200  4345  3  112051915  112081658  3q12-q13  0  0  0.171  0.154  0  0  0  0  0.1  0.191  0.036  0  0  0.16  0  0  0  0  0.115  0.045  0  0.194  0.219  0  0  2.426  1.786  0.556  3.034  0.023  3.178  2.683  1.765  0.539  0  0.444  0.143  0  2.464  0  0  0.039  0  0  2.188  0.663  0  0  2.08  0  0  0.071  0  0  0  0.319  0.127  2.149  0  3.089  
 
 BTLA  151888  3  112182812  112218408  3q13.2  0  0  0.123  0  0  0  0  0  0  0  0  0  0  0.015  0  0  0  0  0  0  0  0.07  0.093  0  0  0  0.089  0  0  0.032  0  0  0  0.308  0  0  0  0  0.077  0  0  0.037  0.021  0  0  0  0.025  0  0  0.09  0  0.104  0.141  0.019  0  0  0  0  0  0  
 
 ATG3  64422  3  112251353  112280810  3q13.2  3.728  6.213  2.813  3.199  4.474  2.338  4.872  4.036  2.705  3.88  3.714  3.619  3.848  4.91  3.067  3.765  3.942  5.745  5.678  4.73  3.39  3.949  2.756  3.217  3.885  3.47  3.847  3.653  3.382  2.755  4.64  4.784  4.751  2.955  2.852  3.344  3.604  3.823  4.116  3.915  3.231  2.778  3.692  2.972  3.32  4.05  3.765  3.149  3.831  5.217  4.419  2.057  3.341  2.694  3.464  3.307  2.997  4.141  3.413  4.424  
 
 SLC35A5  55032  3  112280856  112303284  3q13.2  1.239  3.253  1.593  1.279  1.641  0.803  2.283  1.965  2.024  2.314  1.709  1.596  1.661  2.297  1.071  1.649  1.399  3.343  2.697  1.773  0.674  1.408  1.264  1.081  1.541  2.204  1.865  1.729  1.812  0.899  2.31  2.257  2.309  1.609  1.255  1.65  1.887  1.733  2.088  1.71  1.382  1.034  1.339  1.262  1.536  1.524  1.65  1.056  1.503  2.421  1.978  0.65  1.333  0.951  1.324  1.522  1.198  1.416  1.275  2.28  
 
 LOC100506621  100506621  3  112315640  112320816  -  0.043  0.579  1.651  0.415  0.01  0.238  0.413  2.427  0.088  2.599  0.689  0  0.033  0.037  0.052  0  0  0  0.022  0.035  0  0.075  0.231  0.026  0.646  0  0.044  0.023  0.035  0.056  0.038  0.059  0  0.252  0.755  0.347  0.175  0.542  3.567  0.062  0.356  0.18  0.015  0  0.518  0.5  0.051  1.523  0.301  2.138  0.36  0.119  0.49  0.267  1.029  0.483  0.473  0.808  0  2.388  
 
 CCDC80  151887  3  112323232  112359990  3q13.2  0  3.083  6.41  4.696  0.251  2.515  2.902  6.041  0.59  6.081  3.477  0.059  0.131  0.056  0.101  0.055  0  0.099  0.156  0.356  0  0.203  0.384  0.052  3.863  0.194  0.332  0.554  0.175  0.116  0.538  1.265  0.244  0.492  3.099  2.503  1.998  2.469  6.972  0.184  3.082  1.944  0.299  0.408  2.97  2.909  0.82  5.027  1.919  4.92  3.635  1.385  4.082  2.529  5.144  4.189  3.079  4.427  0.383  5.926  
 
 LOC101929694  101929694  3  112455295  112468166  -  0  0  0.118  0  0.059  0  0  0  0  0.027  0  0  0  0  0  0.103  0  0  0  0  0  0  0.25  0  0  0  0.027  0.042  0  0.024  0  0  0  0.394  0  0  0  0  0  0  0  0.047  0  0  0  0  0  0  0  0  0  0  0  0  0  0  0  0  0  0  
 
 CD200R1L  344807  3  112534555  112564797  3q13.2  0  0  0  0  0  0  0  0  0  0  0  0  0  0  0  0.136  0  0.178  0.069  0  0  0  0  0  0  0  0.037  0  0  0.038  0  0  0  0.252  0  0  0  0  0  0  0  0.08  0  0  0  0  0  0  0  0  0  0  0  0  0  0  0  0  0  0  
 
 CD200R1  131450  3  112641531  112693937  3q13.2  0  0  0.16  0  0.073  0  0  0  0  0  0  0  0  0  0  0  0  0  0.366  0  0  0  0  0  0  0  0.037  0  0  0.04  0  0  0  0.283  0  0  0  0  0.056  0  0.122  0.117  0.042  0  0  0.193  0.751  0  0.242  0  0.094  0  0  0  0.063  0  0  0  0  0  
 
 GTPBP8  29083  3  112709799  112720221  3q13.2  1.539  2.91  1.861  1.58  1.586  1.272  1.562  1.735  1.229  1.707  2.004  1.855  1.319  2.49  1.095  1.974  1.824  2.362  3.207  2.004  1.891  1.983  1.053  1.177  1.708  0.954  1.318  1.215  1.331  1.327  2.013  1.737  1.925  1.379  2.028  1.529  1.438  0.985  2.314  1.634  1.885  1.095  1.991  1.736  1.893  2.014  1.482  1.855  1.591  3.763  2.029  0.967  1.835  0.639  1.94  1.475  1.065  1.311  1.716  2.174  
 
 C3orf17  25871  3  112721291  112738555  3q13.2  1.837  2.053  1.458  1.554  1.913  0.611  2.019  1.854  1.891  1.739  2.014  1.389  1.418  2.573  1.392  1.886  1.789  2.281  3.257  1.762  1.017  1.736  1.47  0.875  1.868  1.961  2.162  1.603  1.569  1.817  2.247  2.392  2.234  1.764  1.339  1.56  1.733  1.635  2.259  2.143  1.462  0.928  2.683  1.411  1.391  1.784  1.612  1.479  1.359  3.398  2.152  0.835  1.527  0.866  1.764  1.288  1.436  1.459  1.629  2.071  
 
 LOC101929717  101929717  3  112861196  112886578  -  0  0.043  0.177  0  0  0.052  0.058  0.026  0.096  0.079  0.032  0  0  0.03  0  0  0  0  0.064  0  0  0.105  0.313  0  0  0  0.041  0  0  0.037  0.081  0  0  0.333  0  0  0  0  0.108  0  0  0.046  0.024  0  0  0.036  0  0  0  0.04  0.045  0.062  0.024  0.028  0.062  0  0  0  0  0  
 
 BOC  91653  3  112930311  113006310  3q13.2  0.643  0.051  2.201  0.488  0.459  1.223  1.135  1.699  2.629  2.534  1.658  0.061  0  0.514  0.746  0.076  0  0.054  0.062  0.154  0  0.311  0.6  0.09  0.137  0.134  2.93  0.058  2.089  0.885  1.524  2.095  0  0.453  0.705  0.721  1.758  0.36  0.407  0.19  0.227  0.555  0.373  0.544  2.097  0.665  1.407  0.33  0.528  0.742  0.24  0.709  0.939  0.54  0.786  0.116  1.191  0.616  0  0.157  
 
 CFAP44  55779  3  113005776  113160361  3q13.2  0.298  0.273  0.245  0.127  0.609  0.037  0.206  0.243  0.239  0.514  0.302  0.258  0.177  0.474  0.385  0.564  0.333  0.463  0.696  0.368  0  0.565  0.254  0.12  0.11  0.264  0.138  0.252  0.21  0.206  0.311  0.286  0.238  0.314  0.137  0.356  0.212  0.185  0.225  0.187  0.233  0.393  0.028  0.306  0.222  0.162  0.437  0.294  0.284  0.658  0.478  0.167  0.157  0.069  0.025  0.051  0.144  0.157  0.136  0.03  
 
 CFAP44-AS1  100874029  3  113122844  113152839  -  0  0  0  0  0.097  0  0  0  0  0.095  0  0  0  0  0  0.208  0  0  0  0.155  0  0  0  0  0  0  0.061  0  0  0  0  0  0.26  0  0  0  0  0  0.096  0  0  0  0  0  0  0  0  0  0  0  0  0  0  0  0  0  0  0  0  0  
 
 SPICE1  152185  3  113161564  113234034  3q13.2  1.095  0.431  0.466  0.624  1.134  0.295  0.693  0.838  0.308  0.877  0.941  0.822  1.07  0.898  1.057  1.254  1.48  0.603  1.593  0.581  0.364  0.763  0.555  0.42  0.72  0.676  0.594  1.117  0.673  0.405  1.041  0.805  1.306  0.657  0.515  0.816  0.62  0.71  0.69  1.18  0.869  0.189  0.921  0.625  0.807  1.183  0.094  0.75  0.568  1.607  1.564  0.359  0.499  0.495  0.625  0.602  0.519  0.64  0.647  1.01  
 
 SIDT1  54847  3  113251217  113348422  3q13.2  0.678  0.028  0.135  0  2.502  0  0.039  0.038  0.077  0.137  0.039  0.37  0.093  0.028  0.053  1.007  0.67  0.061  1.728  0.146  0  1.248  0.416  0.281  0.032  0.093  0.1  0.04  0.034  0.078  0.12  0.03  0  0.323  0.049  0.283  0.041  0  0.074  0.082  0.664  0.097  0.044  0.025  0.053  0.018  0.047  0  0  0.089  2.459  0.298  0.069  0.064  0.045  0.052  0.021  0  0  0.031  
 
 USF3  205717  3  113367232  113415493  3q13.2  2.498  1.69  0.957  0.665  1.956  0.708  1.816  1.891  1.233  1.722  1.495  1.282  1.654  2.153  1.856  1.922  1.567  1.506  2.956  2.534  0.604  2.291  1.011  0.694  1.182  1.893  1.88  1.753  1.388  1.656  1.808  1.551  2.333  1.22  0.86  1.074  1.64  1.381  1.95  2.095  1.322  0.865  2.162  1.224  1.669  1.647  2.343  1.362  1.363  2.085  1.871  0.863  1.559  0.83  1.238  1.286  1.036  1.228  1.147  1.961  
 
 NAA50  80218  3  113435306  113465146  3q13.2  3.339  3.212  2.311  2.502  2.801  2.153  3  3.092  2.33  2.925  3.003  3.171  3.646  4.388  2.907  3.627  3.306  3.116  4.453  2.949  2.674  2.866  2.487  2.29  3.076  2.431  1.816  2.909  2.489  2.029  3.291  3.661  3.101  2.216  2.032  2.591  3.191  2.632  3.655  3.397  2.75  1.957  2.537  2.858  3.175  3.304  1.395  2.635  2.861  4.419  3.943  1.649  2.918  2.166  2.249  2.093  2.107  2.908  3.002  3.513  
 
 ATP6V1A  523  3  113465865  113530905  3q13.31  3.112  3.155  2.003  1.758  3.259  1.391  2.631  3.213  2.571  3.014  2.646  2.457  3.466  3.33  2.687  2.943  3.143  3.068  3.542  2.637  2.454  1.885  2.493  2.405  2.153  3.103  2.277  2.589  2.625  1.91  3.577  3.366  3.24  2.793  1.603  2.524  2.595  2.553  3.704  3.003  2.947  1.729  1.933  2.813  2.292  3.167  1.79  1.596  2.009  3.497  3.115  1.321  2.411  2.481  1.655  1.663  1.94  1.683  2.304  3.365  
 
 GRAMD1C  54762  3  113557680  113666021  3q13.31  0.211  1.19  0.198  0.115  0.367  0  0  0.289  0.211  0.561  0.178  0.637  0.605  0.947  0.128  0.485  0.401  0.113  0.744  0  0  0.102  0.243  0.219  0.286  0.498  0.179  0.138  0.186  0.099  0.417  0.312  0.235  0.342  0.075  0  0.178  0.344  0.107  0.342  0.442  0.347  0.126  0.217  0.163  0.121  0.109  0.05  0.044  0.392  0.074  0.188  0.068  0.161  0.189  0.154  0  0.174  0.193  0.055  
 
 ZDHHC23  254887  3  113666747  113681827  3q13.31  1.42  0.603  0.244  0.332  1.17  0  0.257  0.337  0.756  0.169  0.64  0.731  1.093  1.592  1.391  1.313  0.829  0.682  1.847  0.531  0.072  0.714  0.769  0.578  0.494  0.298  0.579  0.7  0.376  0.792  0.139  0.455  1.16  0.868  0.454  0.524  0.293  0.718  0.315  0.764  0.602  0.124  1.102  0.255  0.926  1.2  0.256  0.267  0.686  0.778  0.251  0.578  0.61  0.316  0.811  0.4  0.071  0.519  1.51  0.317  
 
 CCDC191  57577  3  113682983  113775460  3q13.31  0.584  0.162  0.213  0.59  1.119  0.04  0.49  0.355  0.221  0.69  0.246  0.519  0.353  0.831  0.245  0.56  0.41  0.817  0.591  0.169  0  0.429  0.378  0.059  0.167  0.1  0.109  0.262  0.103  0.087  0.182  0.299  0.06  0.222  0.252  0.186  0.298  0.26  0.439  0.581  0.347  0.232  0.022  0.219  0.135  0.083  0.238  0.154  0.368  0.87  0.334  0.427  0.594  0.238  0.637  0.315  0.11  0.071  0.41  0.306  
 
 QTRTD1  79691  3  113775581  113807268  3q13.31  1.775  1.501  0.992  1.657  1.585  0.994  2.157  1.535  1.237  1.529  1.579  1.699  2.107  2.466  1.866  1.949  1.609  1.685  2.989  1.643  1.403  1.635  1.204  1.061  1.92  1.577  1.496  1.754  1.634  1.268  1.9  2.45  1.946  1.303  1.179  1.387  1.873  1.336  1.684  2.229  1.356  1.033  1.57  1.72  1.397  1.689  0.954  1.606  1.233  2.887  2.215  1.122  1.874  1.119  1.45  1.553  1.089  1.288  1.907  2.124  
 
 DRD3  1814  3  113847498  113918254  3q13.3  0  0  0.122  0  0  0.101  0  0  0  0  0  0.126  0  0.443  0  0  0  0  0  0.145  0  0.141  0.392  0  0  0  0.212  0  0  0.034  0  0  0  0.256  0  0  0  0  0.062  0  0  0.319  0.046  0  0  0.073  0  0  0  0  0  0.37  0  0.22  0  0  0  0  0  0  
 
 ZNF80  7634  3  113953479  113956425  3q13.3  0  0  0.159  0  0.144  0  0  0  0.067  0  0  0  0  0.163  0  0  0  0  0.524  0  0  0.09  0.23  0  0  0  0.05  0.04  0  0.058  0  0  0  0.283  0  0  0  0  0.02  0  0  0.17  0.05  0  0  0  0.146  0  0  0  0.025  0.076  0  0.037  0  0  0  0  0  0  
 
 TIGIT  201633  3  114012832  114029135  3q13.31  0  0.036  0.107  0  0  0  0.113  0.071  0.174  0.099  0.11  0  0  0.857  0  0  0  0.076  0.043  0.036  0  0.065  0.156  0  0  0  0.035  0.041  0  0.03  0  0  0  0.284  0.062  0.073  0  0.061  0.079  0  0  0.453  0.022  0.08  0  0  0  0  0  0.035  0  0.13  0  0.022  0.053  0.05  0.047  0.06  0  0  
 
 ZBTB20  26137  3  114033346  114866127  3q13.2  0.212  0.612  1.118  0.282  0.085  0.557  2.212  1.832  2.774  2.555  1.804  0.213  0.21  3.048  0.143  0.216  0.356  0.9  0.299  0.304  0.047  0.435  0.234  0.039  0.276  1.057  1.316  1.831  0.56  0.583  0.737  1.657  0.835  0.717  1.049  1.228  1.204  1.331  0.712  0.274  0.174  1.077  0.037  0.971  0.513  0.642  0.497  0.46  1.165  0.515  0.484  0.494  1.534  1.075  2.024  1.357  1.329  0.67  1.151  2.561  
 
 MIR568  693153  3  114035321  114035416  -  0  0  0  0  0  6.936  0  0  0  0  0  0  0  0  0  0  0  0  0  0  0  0  0  0  0  0  0  0  0  0  0  0  0  0  0  0  0  0  0  0  0  0  0  0  0  0  0  0  0  0  0  0  0  0  0  0  0  0  0  0  
 
 ZBTB20-AS1  100131117  3  114070657  114107825  3q13.31  0  0  0.153  0  0.033  0  0.134  0.169  0.375  0.424  0.143  0  0  0.303  0  0  0  0.131  0  0.091  0  0.158  0  0  0  0.117  0.172  0.176  0.119  0.024  0.076  0.117  0.074  0.261  0.105  0.219  0.251  0.351  0.036  0  0  0.055  0  0.104  0  0.074  0  0.138  0  0  0.045  0.198  0.211  0.156  0.425  0.093  0.118  0.076  0  0.272  
 
 LOC101929754  101929754  3  114172439  114238979  3q13.31  0  0  0  0  0  0  0  0  0  0.166  0  0  0  0.783  0  0  0  0  0  0  0  0  0  0  0  0  0.418  0  0  0.061  0  0  0  0  0.365  0  0  0  0  0  0  0.749  0  0  0  0  0  0  0  0  0  0  0.149  0  0.132  0  0  0  0  0  
 
 ZBTB20-AS3  104413890  3  114591879  114595361  3q13.31  0  0  0  0  0  0.451  0  0  0  0.129  0  0  0  0.131  0  0.167  0  0  0  0  0  0  0  0  0  0  0.118  0  0  0  0  0  0  0.358  0  0  0  0  0.156  0  0  0  0  0  0  0  0  0  0  0  0  0  0  0  0  0  0  0  0  0  
 
 ZBTB20-AS4  100874131  3  114819269  114821908  -  0  0  0.255  0  0  0  0  0.115  0  0  0  0  0  0.138  0  0.174  0  0  0.287  0  0  0.073  0.383  0  0  0  0.262  0  0  0.185  0  0  0  0  0  0  0  0  0.122  0  0  0  0.092  0  0  0  0.195  0  0  0  0  0  0  0  0.187  0  0  0  0  0  
 
 GAP43  2596  3  115342150  115440334  3q13.1-q13.2  0  0  0.194  0  0  0  0.089  0.29  1.228  1.737  2.665  0.121  0  0.032  0  0  0.187  1.743  0.096  0  0  0.181  0.367  0  0  0  0.145  0.123  0  0.149  0.085  0.124  0  0.406  0  0  0.169  4.134  0.029  0  0  1.476  0.043  0  0  0.076  0.173  0.711  0  1.598  0.05  0.256  0.088  0.042  0  0  0.594  0  0  0  
 
 LSAMP  4045  3  115521209  116164385  3q13.2-q21  0  0.021  0.284  0.099  0.01  0  0.023  0.406  1.226  1.739  0.221  0.239  0.022  0.028  0  0.06  1.038  0.246  0.202  0.017  0  0.091  0.153  0  0.64  1.636  0.174  1.394  0.784  0.268  1.428  1.346  0.635  0.741  0.03  0.054  0.417  0.205  0.049  0  0.066  0.857  0.177  0.208  0  0.022  0.322  0.058  0.202  0.021  0.5  0.374  0.138  0.038  0.03  0.04  0.126  0.019  0  0.017  
 
 LSAMP-AS1  100506708  3  116078870  116088932  -  0  0  0  0  0  0  0  0.34  0  0.441  0.157  0  0  0  0  0  0.444  0.851  0  0  0.496  0  0  0  0  0.396  0  0.188  0.42  0  0.701  0.341  0  0  0  0  0  0  0  0  0.371  0.159  0  0  0  0  0.084  0  0  0  0.199  0  0  0  0  0  0  0  0  0  
 
 TUSC7  285194  3  116428634  116435887  3q13.31  0  0  0.134  0  0  0  0  0  0  0.191  0  0  0  0  0  0  0  0  0.034  0  0  0  0  0  0  0  0.024  0  0  0.052  0  0  0  0.299  0.069  0  0  0  0  0  0  0  0.026  0  0  0  0  0  0  0  0  0.112  0  0  0.042  0  0  0  0  0  
 
 LINC00901  100506724  3  116640277  116651085  -  0  0  0.128  0  0  0  0  0  0  0.054  0  0  0  0  0  0  0  0.141  0  0  0  0.042  0  0  0  0  0.062  0  0  0.027  0  0  0  0.454  0.116  0  0  0  0  0  0  0.05  0.038  0  0  0  0  0  0  0  0  0.128  0  0  0  0  0  0  0  0  
 
 IGSF11  152404  3  118619478  118864898  3q13.32  0.153  0.051  0.188  0  0.101  0  0.037  0.031  0.268  0.104  0.037  0.057  0  0.054  0  0  0  0.046  0.029  0.028  0.195  0.114  0.3  0  0  2.377  0.941  0.831  2.198  2.823  2.725  1.779  3.525  1.862  1.052  0  0  0  0.032  0  0.657  0.165  0.04  0.042  0  0.052  0.04  0  0  0.06  0.12  0.19  0  0.047  0.042  0  0  0  0  0  
 
 IGSF11-AS1  100506765  3  118661922  118667088  3q13.32  0  0  0  0  0  0  0  0  0  0  0  0  0  0  0  0  0  0  0  0  0  0  0  0  0  0.069  0.026  0  0  0.086  0.118  0  0.096  0.172  0  0  0  0  0  0  0  0  0  0  0  0  0  0  0  0  0  0  0  0  0  0  0  0  0  0  
 
 C3orf30  152405  3  118864996  118870302  3q13.32  0  0  0.151  0  0  0  0  0  0.091  0  0  0.073  0  0  0  0  0  0  0  0  0  0.059  0.482  0  0  0  0.024  0  0  0.022  0  0  0  0.272  0.057  0  0  0  0  0  0  0.051  0  0  0  0  0  0  0  0  0  0.115  0  0  0  0  0  0  0  0  
 
 UPK1B  7348  3  118892424  118924000  3q13.32  0  0  0.083  0  0  0  0  0  0.069  0.031  0  0.056  0  0.046  0  0  0  0.094  0  0  0.099  0  0.246  0  0  0  0.067  0  0  0.09  0  0.065  0  0.312  2.184  0.937  0  0  3.274  0  0.193  0.225  0  0.922  3.998  1.79  0.097  0  0  0  0.098  0.116  0.047  0.042  0  2.604  0  0  0  1.679  
 
 B4GALT4  8702  3  118930588  118959752  3q13.3  1.588  1.78  2.705  1.526  2.59  1.333  2.491  3.094  1.919  2.044  2.203  2.327  2.084  2.126  1.799  1.618  1.757  1.569  1.803  0.969  0.792  0.999  1.219  0.379  1.553  1.973  2.428  2.443  2.546  1.787  2.545  3.113  2.388  1.488  3.49  1.695  1.732  1.503  2.586  2  3.647  0.897  1.855  2.214  1.918  2.519  3.707  1.835  2.117  3.726  3.561  0.889  1.944  1.379  1.914  2.256  1.639  1.545  1.954  2.771  
 
 B4GALT4-AS1  100874201  3  118945332  119009513  -  0  0  0.9  0  0.17  0  0.451  0.544  0  1.171  0.621  0  0  0.116  0.311  0  0  0  0  0  0  0.167  0  0  0  0.669  1.577  0.774  1.367  0.422  0.269  0  0.933  0.599  0.58  0.251  0  0  0.143  0  0  0.169  0  0  0  0.346  0  0  0.424  0.321  0.68  0  0.853  0.537  0.335  0.387  0.51  0  0.427  0.599  
 
 ARHGAP31  57514  3  119013219  119138323  3q13.33  0.117  0.386  1.627  0.329  0.35  0.678  1.596  3.432  0.441  2.366  1.68  0.06  0.185  0.38  0.656  0.071  0.084  0.047  0.35  0.102  0.049  0.27  0.608  0.03  1.399  2.541  2.786  2.665  2.706  1.445  2.039  2.533  3.93  2.034  0.105  1.631  0.733  0.96  0.415  0.936  0.088  0.604  0.058  0.119  1.988  1.062  0.203  0.818  0.125  0.397  2.568  0.366  1.922  1.553  0.538  0.73  1.775  0.177  0.968  2.288  
 
 ARHGAP31-AS1  100874246  3  119033139  119041607  -  0  0.52  0.476  0  0.211  0  0  1.053  0  0.609  0.31  0  0  0.168  0.447  0  0  0  0  0  0  0.091  0  0  0  0.71  0.644  0.81  0.578  0.662  0.289  0.697  1.67  0.438  0  0.294  0.26  0.415  0  0.912  0.205  0.107  0.741  0  0.655  0.38  0.231  0.356  0  0.208  0.529  0  0.888  0.346  0.447  0.622  0.642  0.331  0.743  1.046  
 
 TMEM39A  55254  3  119147806  119182529  3q13.33  1.683  2.033  1.485  1.289  1.597  1.059  2.637  3.276  1.443  2.408  1.862  1.458  1.684  2.075  1.715  1.587  1.75  1.828  2.389  1.928  1.006  1.424  1.675  1.087  1.57  1.956  1.826  1.565  1.627  1.251  1.806  2.23  2.281  1.372  0.904  1.378  2.285  1.835  1.071  2.225  1.493  0.774  1.34  1.336  1.54  1.866  1.63  1.572  1.201  2.767  2.587  0.778  1.805  1.361  1.655  2.103  1.634  1.21  1.769  2.116  
 
 POGLUT1  56983  3  119187784  119213554  3q13.33  1.677  2.25  0.802  1.492  1.869  0.691  2.426  1.861  1.593  1.477  1.295  1.189  1.431  2.205  1.395  1.549  1.267  2.829  2.487  2.256  0.988  1.681  1.462  1.329  1.011  1.181  1.7  1.524  1.245  0.77  1.521  1.727  2.361  1.816  0.878  1.336  1.728  1.235  1.011  1.654  1.397  0.738  1.812  1.046  1.175  1.259  2.219  1.178  0.717  2.595  1.569  0.557  1.294  0.491  1.032  1.015  0.938  1.284  1.347  1.469  
 
 TIMMDC1  51300  3  119217367  119243125  3q13.33  4.061  4.523  3.428  3.346  4.397  1.969  4.277  4.202  3.773  4.155  3.733  3.649  3.864  4.694  2.745  3.959  3.938  4.631  5.277  5.031  3.363  4.132  3.918  2.927  4.038  3.964  3.708  4.298  4.435  4.218  4.927  5.536  5.023  4.354  3.096  3.383  3.716  3.533  4.042  4.243  3.39  3.016  3.953  2.964  3.891  3.818  3.905  3.027  3.364  5.743  4.588  2.724  3.758  2.932  4.391  3.624  3.254  3.423  3.98  4.677  
 
 CD80  941  3  119243139  119278481  3q13.3-q21  0  0  0.105  0  0  0.061  0.202  0.084  0.042  0.093  0.04  0  0  0.074  0  0  0.145  0.127  0.051  0  0.473  0.084  0.144  0  0  0  0.104  0.03  0  0.039  0.071  0  0  0.355  0  0  0  0  0.038  0  0  0.199  0.03  0  0  0  0.018  0  0  0  0.054  0.131  0.044  0  0  0  0.048  0  0  0  
 
 ADPRH  141  3  119298279  119308792  3q13.31-q13.33  0.099  0.126  0.457  0  0.348  0.279  0  0.111  0.092  0.276  0.341  0  0  0.266  0.086  0.303  0.046  0.525  0.06  0.164  0.157  0.157  0.711  0.309  0  0.821  0.189  0.079  0.699  0.175  0.347  0.686  0  0.392  0.237  0.063  0.205  0.321  0.071  0.469  0.261  0.251  0.02  0  0.466  0.442  0.14  0.206  0  0.442  1.314  0.345  0.346  0.177  0.359  0.106  0.443  0  0.545  0.469  
 
 PLA1A  51365  3  119316694  119348658  3q13.13-q13.2  0  0  0.145  0  0  0  0  0  0  0  0  0  0  0.068  0  0.065  0.13  0.137  0.075  0  0  0  0.345  0  0  3.452  0.121  1.39  6.078  0.124  4.791  2.413  0  0.293  0.123  0  0  0  0  0  0  0  0  0.435  0  0  0.215  0  0  0  0.462  0  0  0.373  0.115  0  0.359  0  0  0  
 
 POPDC2  64091  3  119360898  119379437  3q13.33  0.171  0  0.332  0  0.217  0  0.41  0.147  0.382  0.231  0.384  0  0  0.38  0.179  0.065  0.163  0  0.834  0.538  0  0.7  0.593  0  0.165  0.178  0.592  0.101  0.095  0.05  0.752  0.458  0.393  0.594  0  0  0.331  0.372  0.234  0.118  0.077  0.339  0.026  0.144  0.167  0.276  0  0  0.139  0.496  0.432  0.38  0.223  0.09  0.082  0  0.067  0.108  0.103  0.109  
 
 COX17  10063  3  119388371  119396243  3q13.33  4.662  7.1  3.822  2.673  6.117  4.081  5.018  4.814  3.939  4.237  4.333  5.127  3.175  5.057  2.684  5.771  5.645  8.468  6.926  5.744  4.538  5.87  4.575  4.412  4.079  4.069  4.235  3.18  3.98  3.298  6.099  5.357  3.806  3.695  5.542  4.166  3.441  3.856  5.123  4.095  4.577  5.087  4.624  4.092  5.373  6.343  4.429  3.365  4.807  6.346  4.559  2.72  3.451  4.362  4.637  4.312  2.318  4.551  3.912  4.213  
 
 MAATS1  89876  3  119421868  119485949  3q12-q13.3  0.053  0  0.113  0.102  0.206  0  0.103  0  0.079  0.019  0.033  0  0  0.064  0  0.036  0  0  0  0.023  0  0.042  0.179  0.027  0.038  0.037  0.047  0.016  0.061  0.07  0  0  0  0.193  0  0  0  0  0.058  0.209  0  0.103  0.026  0  0.169  0.053  0.016  0.046  0.187  0.077  0.054  0.322  0  0.16  0.264  0.111  0.035  0  0  0.047  
 
 NR1I2  8856  3  119499330  119537332  3q12-q13.3  0  0  0.169  0  0.03  0.093  0  0.032  0.092  0.031  0  0.109  0.227  0.02  0  0.052  0  0.187  0.192  0.125  0  0.085  0.38  0  0  0.037  0.068  0  0  0.099  0  0  0  0.325  0.064  0  0  0.045  0.025  0  0  0.044  0.029  0  0.066  0.067  0.034  0  0  0.023  0.16  0.307  0  0.051  0.053  0.092  0  0  0  0  
 
 GSK3B  2932  3  119540801  119813264  3q13.3  2.212  2.296  2.262  2.441  2.968  1.425  2.841  3.164  2.398  2.582  2.495  2.057  2.604  2.923  2.429  2.325  2.551  1.959  2.764  1.791  1.482  2.706  1.339  1.425  2.401  3.01  2.443  2.725  2.616  2.069  2.828  3.389  3.06  1.922  1.76  2.52  2.485  2.413  2.3  3.023  2.508  1.444  2.326  2.349  2.495  2.553  1.259  2.548  2.608  3.231  3.324  1.448  2.055  1.51  1.913  2.275  1.838  2.103  2.543  3.217  
 
 GPR156  165829  3  119884327  119963142  3q13.33  0.135  0.031  0.383  0.13  0.332  0.144  0.156  0.137  0.219  0.166  0.33  0.036  0  0.032  0.039  0.052  0  0.034  0.066  0.028  0  0.071  0.359  0  0  0.028  0.108  0.079  0.038  0.052  0.058  0.034  0  0.352  0  0.173  0.083  0.19  0.035  0.206  0.057  0.059  0.033  0.057  0.434  0.452  0.037  0.793  0.326  0.75  0.322  0.163  0.026  0.074  0.053  0.181  0.057  0.189  0.035  0.073  
 
 LRRC58  116064  3  120043575  120068186  3q13.33  2.626  2.69  1.791  2.035  2.595  1.114  2.777  2.859  2.523  2.477  2.324  2.092  2.645  3.512  2.197  2.178  2.106  1.692  3.535  2.625  1.838  2.806  1.526  1.373  2.678  2.86  2.502  2.648  2.354  2.065  3.086  3.536  3.77  2.411  1.521  2.138  2.088  2.116  1.636  2.944  2.148  1.663  2.202  2.307  2.053  2.33  1.306  1.944  2.459  2.632  2.901  1.008  2.165  1.162  1.62  1.932  1.859  1.837  2.142  2.575  
 
 FSTL1  11167  3  120113060  120169918  3q13.33  0.069  5.778  7.562  6.94  3.023  5.153  5.464  7.372  2.829  6.531  4.708  0  0.048  2.445  0  0.051  0.048  0.072  3.414  0.283  0.403  2.262  0.27  0  3.797  2.946  1.317  2.972  3.663  2.123  3.848  3.962  1.582  0.599  1.797  3.394  3.767  0.267  6.359  1.933  2.968  2.491  4.423  5.063  5.229  5.352  3.676  5.315  5.795  6.514  6.495  0.395  5.125  4.622  2.336  6.619  5.184  4.712  0  6.099  
 
 NDUFB4  4710  3  120315127  120321258  3q13.33  5.576  7.514  4.924  4.874  7.26  5.013  6.405  5.909  5.708  5.934  5.886  6.411  4.97  6.463  3.835  6.45  6.453  8.696  8.033  7.221  5.943  7.058  4.778  5.086  6.056  5.068  5.256  4.38  5.434  4.849  6.699  6.125  4.894  5.052  5.975  5.05  4.288  4.75  6.351  5.123  5.799  5.801  5.849  6.098  5.763  6.457  5.483  4.713  5.831  7.259  5.681  3.776  5.044  5.55  5.845  5.572  4.036  6.146  5.428  5.44  
 
 HGD  3081  3  120347014  120401418  3q13.33  0  0  0.116  0  0  0  0  0.049  0.154  0  0.042  3.109  2.627  0.114  0.097  0.487  1.19  1.154  0.057  0  0  0.079  0  0  0  0  0.021  0  0  0.034  0  0  0  0.153  1.413  0.088  0  0  0.067  0  0  0.997  0.031  1.202  0.257  1.806  0  0  0.896  0.047  0.044  0.364  0.546  1.889  1.592  1.984  0.336  0.083  1.478  1.115  
 
 RABL3  285282  3  120405527  120461384  3q13.33  1.702  1.95  1.581  1.376  2.215  0.655  2.496  1.937  1.898  1.75  1.807  1.681  1.468  2.112  1.496  1.44  1.629  2.363  2.846  2.581  1.198  2.025  0.672  0.767  1.239  1.349  0.942  0.855  0.804  0.342  0.873  1.059  0.95  0.653  1.084  0.848  1.095  1.538  1.729  1.334  0.651  1.212  0.829  1.425  1.171  1.27  0.945  1.439  1.113  2.007  2.201  0.593  1.658  1.019  1.438  1.206  1.356  1.103  1.539  2.085  
 
 GTF2E1  2960  3  120461557  120501916  3q21-q24  1.908  1.757  1.367  1.575  1.613  0.603  2.139  1.633  1.352  1.32  1.678  0.946  1.986  2.295  1.254  1.42  1.502  2.026  2.875  1.934  1.41  1.33  1.472  1.129  1.926  1.61  1.053  1.564  1.512  1.36  1.719  2.4  2.057  1.221  1.162  1.275  1.614  1.454  0.899  2.28  1.036  1.031  1.601  1.526  1.46  1.708  0.759  1.121  1.401  2.536  2.094  0.78  1.722  1.24  1.693  1.527  1.206  1.448  2.003  2.414  
 
 STXBP5L  9515  3  120627049  121143608  3q13.33  0.11  0.057  0.128  0  0.148  0.029  0.054  0.092  0.051  0.103  0.129  0.064  0.118  0.258  0.195  0.128  0.096  0.083  1.236  0.215  0.078  0.529  0.162  0.033  0.025  0.055  0.17  0.049  0.09  0.379  0.214  0.114  0.09  0.22  0.043  0.059  0.161  0.096  0.049  0.038  0.037  0.146  0.333  0.11  0.02  0.12  0.17  0.13  0.132  0.054  0.13  0.076  0.098  0.052  0.062  0.026  0.056  0.034  0.107  0.04  
 
 POLQ  10721  3  121150272  121264853  3q13.33  1.004  0.862  0.501  0.684  0.768  0.548  0.887  1.145  0.161  1.017  1.113  0.616  1.266  1.331  1.453  1.451  0.775  1.14  1.959  1.101  0.635  1.09  0.568  0.561  0.571  1.024  0.648  0.689  1.123  0.694  1.167  0.991  1.614  0.636  0.858  0.728  1.258  0.892  0.528  1.583  0.899  0.573  0.723  0.845  0.776  1.098  0.347  0.946  1.073  1.332  1.205  0.612  1.104  0.592  1.076  0.514  0.953  0.897  1.35  1.428  
 
 ARGFX  503582  3  121286777  121309469  3q13.33  0  0  0.134  0  0.016  0  0  0  0.067  0.027  0  0.029  0  0  0  0.049  0  0.118  0  0.026  0  0.048  0.317  0  0  0  0.05  0  0  0.033  0  0  0  0.363  0  0  0  0  0.018  0  0  0.04  0.079  0  0  0  0.022  0  0  0  0.015  0.08  0.019  0  0.024  0  0  0  0  0  
 
 FBXO40  51725  3  121312169  121349139  3q13.33  0  0  0.106  0  0.058  0  0.052  0.035  0.081  0.022  0.025  0.046  0.047  0.058  0.057  0.031  0.052  0.055  0.085  0.171  0  0.15  0.292  0  0.023  0.033  0.039  0.013  0  0.032  0.058  0.046  0.04  0.361  0.04  0  0.034  0  0.1  0.036  0  0.084  0.023  0  0  0.028  0.023  0.034  0  0.041  0.019  0.275  0.027  0.052  0.021  0  0  0  0.034  0  
 
 HCLS1  3059  3  121350244  121379795  3q13  0  0.049  0.195  0  0  0  0.266  0.042  0  0.107  0.155  0  0  0.043  0  0  0  0  5.871  4.522  1.699  4.6  4.531  3.779  0.456  0  0.056  0.054  0  0.101  0  0.51  0  0.333  0  0  0.162  0.268  0.109  0  0.203  1.381  0  0  0  0.604  0.04  0  0.752  0.383  0.17  0.11  0  0  0  0.114  0.063  0  0  0  
 
 GOLGB1  2804  3  121382045  121468614  3q13  2.359  2.455  1.533  1.262  2.918  0.935  2.154  2.983  1.723  2.185  2.252  1.618  1.918  2.354  2.073  2.17  1.897  2.294  1.847  1.427  0.789  1.495  1.341  1.032  1.147  2.087  1.593  2.21  2.201  1.075  1.897  1.976  2.224  1.507  1.264  1.698  1.634  1.996  1.821  2.055  1.698  1.038  1.813  1.64  1.547  1.982  1.047  1.642  2.071  2.574  2.919  0.839  1.693  1.492  1.925  2.044  2.796  1.205  1.875  2.522  
 
 IQCB1  9657  3  121488609  121553926  3q13.33|3q21.1  1.684  1.501  1.012  1.78  1.274  0.961  2.066  1.746  0.989  1.942  1.931  1.807  1.918  2.241  1.626  2.09  2.107  1.631  4.365  2.41  1.393  2.374  1.503  0.966  1.205  1.571  1.315  1.938  1.755  1.388  2.324  2.082  2.004  1.367  0.998  0.912  1.748  1.441  1.13  2.271  1.809  1.247  1.892  1.511  1.292  1.667  0.567  1.925  1.55  3.939  2.036  0.707  1.508  0.827  1.847  1.304  2.046  1.597  1.864  2.162  
 
 EAF2  55840  3  121554033  121605373  3q13.33  0.38  0.63  0.247  0  0.739  0.288  0.6  0.747  0  0.657  0.823  1.155  0.62  1.397  0.276  0.868  1.286  3.501  2.871  3.368  0.547  1.561  1.049  0.258  0.703  0.324  0.247  0.337  0.497  0.081  1.029  0.659  0.521  0.478  0.735  0.249  0.433  0.267  0.169  1.157  1.052  0.104  1.11  0.964  0.469  0.481  0.206  0.532  0  2.11  0.501  0  0.641  0.308  0.51  0.547  0.353  0.598  0.463  0.877  
 
 SLC15A2  6565  3  121613170  121663034  3q13.33  0.372  0  0.083  0.064  0.018  0.025  0.021  0.039  0.222  0.071  0.035  0.175  0.132  0.094  0.07  0.21  0.095  0.159  0.372  0.35  0  0.255  0.231  0  0  0.049  0.038  0.043  0  0.025  0.046  0.074  0  0.277  0.062  0.058  0.051  0.035  0.022  0.109  0.042  0.04  0.127  0.028  0.074  0.134  0.13  0.054  0  0.077  0.053  0.137  0.029  0.022  0.023  0  0.024  0  0.036  0  
 
 ILDR1  286676  3  121706169  121741127  3q13.33  1.271  0  0.152  0  1.32  0  0  0  0.105  0  0.029  1.318  1.123  0.577  1.186  0.878  0.209  0  0  0.083  0  0.095  0.952  0.328  0  0  0.051  0.03  0  0.04  0  0  0  0.31  0  0.104  0  0  0.019  0.102  1.094  0.149  0  0.089  0.781  0.382  0.019  0  0  0  0.061  0.209  0  0.029  0.096  0  0.044  0  0  0  
 
 CD86  942  3  121774208  121839988  3q21  0  0.058  0.105  0  0.036  0  0  0  0.182  0  0  0  0  0.043  0  0  0  0.064  0  0.602  0  0  1.849  0.69  0  0  0.078  0  0  0.043  0  0  0  0.233  0  0  0  0  0  0  0  0  0.025  0.065  0  0  0.079  0  0  0  0.084  0  0  0  0  0  0  0  0  0  
 
 CASR  846  3  121902529  122005344  3q13  0  0.043  0.19  0.065  0.021  0  0.018  0.021  0.075  0.028  0.02  0.059  0  0.057  0  0.025  0  0.147  0  0.068  0  0.12  0.348  0  0  0  0.121  0  0  0.057  0  0.042  0  0.409  0.023  0  0  0  0.073  0  0.034  0.252  0.042  0.031  0  0.042  0.035  0  0  0  0  0.476  0.026  0.084  0.046  0  0  0  0  0  
 
 CSTA  1475  3  122044010  122060815  3q21  1.611  0  2.274  0  0.269  0.595  0.109  0  0  0  0.093  0  0  2.302  0  0  0  0.964  0  6.214  0  0  0.306  0  0  0  0.202  0.506  0  0  0  0  0  0.362  0.489  0  0  0  3.067  0  0.226  0.794  0  0  0  0.299  0  0  0  0  0  0.309  0  0.748  0  0  0.803  0  0  0  
 
 CCDC58  131076  3  122078437  122102078  3q21.1  2.592  3.713  2.098  1.679  2.445  3.152  2.54  2.741  1.64  1.944  2.98  3.451  2.87  4.292  1.747  2.953  3.641  5.38  4.535  2.125  3.718  2.143  1.721  2.545  3.353  2.14  1.098  1.536  2.341  1.265  3.63  3.646  2.355  1.604  3.087  1.825  1.706  1.759  1.507  2.331  2.412  3.093  1.335  3.578  3.663  2.415  3.421  1.749  3.082  4.243  2.853  1.618  2.686  1.919  2.946  2.841  1.71  3.451  3.046  2.813  
 
 FAM162A  26355  3  122103022  122128961  3q21.1  4.281  5.535  4.347  3.838  4.706  3.429  3.577  4.473  3.469  4.514  4.762  5.28  4.195  5.097  3.53  4.884  6.557  7.626  5.789  5.774  4.515  5.3  3.694  4.126  3.98  3.426  3.624  1.394  4.339  3.929  4.636  4.642  4.403  3.662  3.734  3.076  2.473  3.613  4.289  3.818  3.696  3.666  4.36  5.292  4.89  5.035  3.558  2.887  3.467  5.682  5.106  2.99  3.373  1.373  3.863  4.207  3.864  3.963  3.274  4.089  
 
 WDR5B  54554  3  122130699  122134882  3q21.1  0.954  0.921  0.572  0.73  1.141  0.291  0.912  0.783  0.851  0.803  0.686  0.334  0.71  0.913  0.569  0.914  0.413  1.782  1.253  0.79  0.179  0.744  0.392  0.226  0.41  0.843  0.715  0.862  0.636  0.141  0.885  0.978  0.905  0.848  0.667  0.602  0.664  0.667  0.58  0.809  0.572  0.485  0.337  0.328  1.004  0.566  0.828  0.475  0.627  0.876  1.111  0.214  0.568  0.344  0.672  0.551  0.438  0.382  0.418  0.934  
 
 LOC102723582  102723582  3  122135052  122162094  3q21.1  0  0  0.28  0  0.318  0  0  0.553  0  0.32  0.207  0  0  0  0.45  0.323  0.317  0  0  0  0  0.413  0.42  0  0  0.201  0.749  0.3  0.351  0.079  0.511  0  0.497  0.652  0  0  0.377  0.324  0  0  0  0.181  0  0  0  0.448  0  0.264  0  0.33  0.242  0.45  0.428  0.104  0.231  0.443  0.204  0  0  0  
 
 KPNA1  3836  3  122140747  122233786  3q21  2.475  2.654  2.066  2.136  2.441  1.267  2.527  3.029  2.057  2.314  2.61  2.031  2.591  2.933  1.907  2.346  1.662  2.485  3.464  1.966  1.682  1.974  1.421  1.199  2.1  2.399  1.983  2.196  2.596  1.776  2.644  2.867  2.819  1.838  1.438  2.144  2.248  2.077  2.341  2.933  1.926  1.68  2.328  1.923  2.886  2.444  1.554  2.335  1.888  3.632  3.187  1.245  2.303  1.432  1.848  1.782  1.814  1.998  2.237  2.559  
 
 PARP9  83666  3  122246759  122283523  3q21  0.759  1.147  0.673  3.58  1.915  0.309  2.209  2.397  1.633  2.319  2.609  1.552  2.051  2.02  1.963  5.436  1.048  0.906  3.136  0.727  0.519  2.298  0.764  2.389  2.208  2.212  3.129  1.766  3.095  1.662  1.778  2.852  3.186  2.877  0.738  1.439  1.21  1.76  2.612  1.375  2.05  1.353  0.482  0.509  1.762  1.64  1.499  0.403  1.226  1.624  2.162  0.67  1.59  2.082  1.782  1.237  3.002  1.46  1.715  1.863  
 
 DTX3L  151636  3  122283184  122294049  3q21.1  1.031  1.294  1.011  4.047  1.385  0.381  2.282  2.535  1.322  2.456  1.895  2.246  2.802  1.414  2.006  4.367  1.552  0.68  2.446  1.218  0.739  2.586  1.041  1.982  2.202  2.163  2.703  2.491  2.958  1.579  1.854  2.041  3.409  2.58  0.882  1.449  1.58  2.046  2.686  1.748  1.637  1.005  0.355  0.718  2.339  1.962  1.399  0.96  1.517  1.451  2.416  0.661  1.97  2.67  1.964  1.887  3.204  1.686  2.782  2.988  
 
 PARP15  165631  3  122296448  122357894  3q21.1  0  0  0.139  0  0  0  0.028  0.037  0.063  0  0.197  0  0  0.012  0  0.044  0  0  2.108  0  0  0.393  0.208  0  0  1.221  0.049  0.017  0  0.059  0  0  0  0.219  0  0  0  0.506  0.013  0  0  0.338  0.029  0  0  0.02  0.046  0  0  0  0.03  0.078  0  0.019  0.028  0  0  0  0  0  
 
 PARP14  54625  3  122399671  122449687  3q21.1  0.233  1.393  0.588  3.604  1.153  0.251  2.04  2.382  1.403  1.643  2.38  1.636  2.924  1.547  2.847  5.374  1.342  0.997  2.531  0.698  0.635  2.242  1.244  1.699  1.43  1.736  2.231  1.75  1.877  1.41  1.266  1.116  2.559  1.583  0.908  1.273  1.71  1.464  2.975  1.248  1.231  1.39  0.919  1.184  1.381  1.502  0.644  0.473  1.73  1.881  2.126  0.729  2.281  2.288  2.123  2.051  3.566  1.588  2.222  1.961  
 
 HSPBAP1  79663  3  122458843  122512666  3q21.1  0.899  1.24  0.596  1.172  0.756  0.566  1.37  1.11  1.046  1.288  0.659  0.834  0.885  1.189  1.086  1.269  1.043  1.148  2.813  2.308  0.368  1.862  1.797  0.569  1.17  1.521  1.951  1.134  1.549  1.587  1.793  1.538  2.111  1.671  0.584  0.603  1.043  0.989  2.006  1.268  0.672  0.779  1.707  0.183  1.748  1.164  1.17  0.547  0.479  1.947  1.279  0.463  0.562  0.208  1.403  0.698  0.324  0.777  1.201  1.088  
 
 DIRC2  84925  3  122513900  122599986  3q21.1  1.244  2.23  0.776  0.846  1.718  0.637  1.657  1.62  1.578  1.751  1.403  0.543  1.437  1.971  1.386  1.25  1.261  1.035  1.394  0.786  0.18  0.737  0.842  0.347  1.339  1.717  1.734  1.525  1.424  1.317  2.177  1.738  2.039  1.633  0.962  1.549  1.491  1.216  1.822  1.924  1.653  0.951  1.873  0.605  1.842  1.666  0.673  0.523  1.521  1.497  2.02  1.278  1.273  0.882  1.051  1.038  0.991  1.026  1.353  2.01  
 
 LOC100129550  100129550  3  122605359  122611263  3q21.1  0.313  0.529  0.309  0.336  0.472  0.202  0.555  1.031  1.119  1.294  0.803  0.104  0.322  1.237  0.48  0.268  0.259  0.333  0.638  0.564  0  0.444  0.526  0.1  0.755  0.838  0.818  0.44  0.175  0.364  0.748  0.69  0.345  0.515  0.474  0.908  0.532  0.899  1.104  1.066  0.53  0.423  0.906  0.415  0.584  0.616  0.488  0.148  0.683  0.515  1.078  0.409  0.372  0.388  0.477  0.258  0.194  0.447  0.412  1  
 
 SEMA5B  54437  3  122628039  122747452  3q21.1  0  0.022  0.205  0.428  0.034  0.102  0  0.197  0.39  0.956  0.454  0.075  0  0.092  0.079  0.085  0.064  0.144  0.02  0.073  0  0.207  0.398  0  0.049  0.056  0.222  0.031  0.033  0.139  0.039  0.057  0.045  0.349  0.031  0.141  0.043  0.323  0.098  0.046  0.042  0.435  0.068  0  1.637  0.322  0.123  0.084  0  0.025  0.078  0.668  0.552  0.436  0.085  0.075  2.509  0.065  0.035  0  
 
 PDIA5  10954  3  122785855  122880953  3q21.1  1.822  2.837  2.234  3.038  1.795  1.247  1.1  3.571  1.751  2.947  2.232  2.094  2.151  3.222  1.258  2.085  1.969  2.823  2.37  3.033  1.945  1.473  1.959  2.064  1.917  2.572  2.191  1.779  2.645  1.716  2.659  3.198  2.307  1.138  1.421  1.462  1.946  2.74  2.384  2.98  1.136  1.698  1.622  2.829  1.903  2.32  1.486  1.648  1.536  3.987  2.805  1.119  2.028  1.397  2.081  2.173  3.152  1.114  2.514  2.802  
 
 MIR7110  102465667  3  122880640  122880726  -  0  0  0  0  0  0  0  0  0  0  0  0  0  0  0  0  0  0  0  0  0  18.248  0  0  0  0  0  0  0  0  0  0  0  0  0  0  0  0  0  0  0  0  0  0  0  0  0  0  0  0  0  0  0  0  0  0  0  0  0  0  
 
 SEC22A  26984  3  122920773  122992982  3q21.1  1.757  2.031  1.356  1.397  2.106  0.671  2.243  1.586  1.305  1.731  1.436  1.155  1.122  1.768  0.966  1.394  1.063  1.618  2.505  1.781  0.61  1.328  1.129  0.851  1.045  1.213  1.517  0.783  1.127  0.775  1.449  1.656  1.395  1.063  0.944  0.958  1.474  1.252  2.043  1.328  0.962  0.877  2.081  0.959  0.983  1.032  2.141  0.899  0.947  2.474  1.885  0.48  1.237  0.784  1.28  1.184  1.027  1.129  1.194  1.765  
 
 ADCY5  111  3  123001142  123167392  3q21.1  0.093  0.046  0.171  0.199  0.121  0.162  0.106  0.167  0.247  0.238  0.092  0.116  0.065  0.242  0.097  0.251  0.125  0.184  0.089  0.165  0.041  0.382  0.589  0.384  0  0.064  0.324  0.074  0.088  0.144  0.216  0.107  0.078  0.324  0.187  0.322  0.07  0.159  0.232  0.362  0.078  0.349  0.124  0.778  0.118  0.162  0.751  0.835  0.062  1.936  0.098  1.043  0.055  0.17  0.15  0.136  0.087  0.075  0.068  0.056  
 
 HACD2  201562  3  123213362  123303924  3q21.1  3.345  3.436  2.701  1.239  3.755  2.855  3.643  4.25  1.898  3.531  4.485  2.523  2.873  3.857  3.373  2.309  2.595  2.773  1.558  2.621  0.819  1.94  0.971  2.279  2.248  2.59  2.023  3.352  2.081  1.755  2.546  2.898  3.591  1.573  2.886  3.594  2.894  2.52  3.572  3.419  3.604  2.109  2.486  3.496  3.148  3.535  1.671  3.282  3.657  2.303  3.513  2.359  3.46  1.894  2.265  2.629  2.55  2.468  3.489  2.634  
 
 MYLK-AS1  100506826  3  123304359  123349666  3q21  2.142  1.5  1.44  0.847  1.663  0.929  2.18  1.281  0.942  1.681  1.002  0.493  0.408  1.438  0.465  0.596  1.18  1.059  1.928  0.97  0.35  1.413  1.281  0.844  0.24  0.42  0.817  0.913  0.495  0.07  0.252  0.813  0.562  0.383  0.688  0.422  0.924  1.212  0.659  1.04  1.117  1  0.78  0.301  0  1.091  2.141  0.704  0  1.037  1.281  0.889  1.44  0.569  1.41  0.815  0.585  0.87  0.791  1.358  
 
 MYLK  4638  3  123331142  123603149  3q21  0.559  3.365  3.671  0.721  0.762  1.025  6  4.888  2.692  5.82  2.175  0.055  0.314  0.735  0.274  0.343  0.147  0.399  0.167  0.325  0.143  0.564  1.123  2.024  0.467  1.553  0.761  1.796  0.756  0.898  1.222  2.069  1.596  1.735  1.741  1.47  3.976  3.948  4.436  1.252  0.344  1.558  0.718  3.031  1.327  2.084  2.307  1.196  1.784  1.598  1.001  1.486  3.015  2.347  4.333  3.709  2.78  1.571  3.484  6.339  
 
 MYLK-AS2  100873940  3  123408490  123411254  -  0  0  0  0  0  0  0.29  0.266  0  0  0  0  0  0.12  0  0  0  0.275  0  0  0  0  0  0  0  0  0  0  0  0  0  0  0  0  0  0  0  0  0.104  0  0  0  0  0  0  0  0  0  0  0  0  0  0.24  0  0.21  0  0.33  0  0  0  
 
 CCDC14  64770  3  123632273  123680255  3q21.1  1.966  3.176  1.648  1.168  2.236  1.169  2.735  2.755  1.492  2.887  2.421  1.389  1.754  2.82  1.872  2.226  1.994  2.396  3.847  2.462  1.407  2.684  1.103  0.859  1.019  2.205  2.585  2.315  2.607  1.497  2.626  2.387  2.739  1.782  1.383  1.866  2.301  1.96  2.02  1.769  1.062  1.563  2.389  2.231  1.619  2.279  2.706  1.767  2.263  3.426  2.325  1.152  1.949  1.509  2.704  1.775  2.087  1.406  2.511  2.489  
 
 ROPN1  54763  3  123687878  123710199  3q21.1  0  0  0  0  0  0  0  0  0  1.605  0.204  0  0  0.096  0  0  0  0  0  0  0  0.154  0  0  0  2.098  2.404  1.454  2.665  1.688  3.891  2.91  2.527  2.201  0  0  0  0  0  0  0  0  0  0  0  0  0  0  0  0  0  0.239  0  0  0  0  0  0  0  0  
 
 KALRN  8997  3  123813557  124440036  3q21.2  0.373  0.123  0.748  0  0.197  0.224  1.067  0.857  0.479  1.56  0.322  1.579  0.574  0.212  0.227  0.279  1.243  0.278  0.025  0.096  0.187  0.16  0.485  0.036  0.048  0.594  0.373  0.251  0.082  0.172  0.045  1.599  0.236  0.698  0.122  0.117  0.078  0.289  0.098  0.347  0.218  0.369  0.45  0.452  0.415  0.878  0.114  0.475  0.216  0.66  0.821  0.632  0.972  0.503  0.212  0.166  1.202  0  1.128  0.162  
 
 UMPS  7372  3  124449212  124468119  3q13  1.994  2.303  1.163  1.423  2.065  0.703  2.119  2.103  1.252  1.571  1.69  1.779  1.906  2.283  1.569  2.304  1.704  2.529  3.208  2.716  1.436  2.292  1.419  1.131  1.686  1.718  1.479  1.691  1.632  1.456  1.933  2.411  2.407  1.611  1.421  1.482  1.758  1.377  1.739  2.171  1.504  1.345  1.792  1.865  2.321  1.984  1.761  1.52  1.476  3.071  1.386  0.897  1.847  1.176  1.734  1.356  1.157  1.231  2.06  2.14  
 
 ITGB5  3693  3  124480794  124606152  3q21.2  3.482  3.043  4.427  3.036  3.497  2.342  4.554  5.018  3.739  4.621  3.509  2.287  2.428  3.047  2.652  4.843  3.209  2.363  0.1  0.115  1.071  0.395  2.493  2.146  2.943  3.477  3.947  5.479  4.911  2.306  3.246  5.093  4.22  3.114  3.275  4.343  4.369  4.639  3.809  3.013  3.451  3.812  2.208  2.276  2.512  2.628  3.658  2.234  3.527  4.352  3.253  1.947  3.946  3.262  3.337  3.069  3.574  1.023  3.923  4.584  
 
 MUC13  56667  3  124624288  124653595  3q21.2  0  0.037  0.129  0  0  0  0.351  0.041  0  0  0  5.088  5.51  0.053  0.623  2.752  4.13  1.589  0.032  0  0  0.074  0.154  0  0  0  0.048  0  0  0.036  0  0.243  0  0.31  0.8  0.083  0  0.081  0.211  0  0  1.247  0.027  0  0  0.053  0.059  0  0  0  0.426  0.073  0.04  0.514  0  0  0  0  0  0  
 
 HEG1  57493  3  124684553  124774802  3q21.2  0.16  3.443  4.254  2.247  2.711  2.642  4.509  4.282  1.686  4.439  2.647  0.039  0.123  0.653  0.705  0.073  0.591  0.388  0.254  0.166  0  1.137  0.321  0.229  3.51  2.168  1.815  3.51  1.541  1.97  1.887  3.507  3.393  1.707  0.675  2.069  4.496  2.744  4.982  1.44  0.196  1.596  1.63  1.578  2.47  0.964  2.308  3.004  0.797  3.396  1.947  0.992  2.925  1.933  2.568  1.479  2.904  2.715  2.064  3.913  
 
 SLC12A8  84561  3  124801479  124931609  3q21.2  0.835  0.941  2.93  0.787  1.866  1.411  3.352  1.251  0.121  0.91  0.963  1.209  1.806  1.881  2.05  1.562  1.029  1.317  0.033  0.161  0  0.132  0.277  0.111  1.4  0.517  0.085  0.7  0.106  0.068  0.08  0.215  0.09  0.366  0.149  1.189  3.831  1.73  4.502  0.209  0.548  0.95  0.025  0.781  1.305  1.567  0.879  1.144  0  2.411  0.574  0.9  0.868  1.59  1.171  0.867  1.806  0.04  2.321  2.339  
 
 ZNF148  7707  3  124944512  125094198  3q21  1.926  2.327  1.699  1.597  1.987  0.911  2.262  2.468  1.863  2.119  2.138  1.479  1.769  2.274  1.701  2.104  1.716  2.403  2.432  1.88  0.715  1.897  1.139  0.739  1.467  2.052  1.72  1.762  1.761  1.502  1.811  1.992  1.908  1.526  0.785  1.587  1.875  1.628  2.602  2.176  1.596  0.939  1.679  1.44  1.752  1.728  1.255  1.545  1.768  2.328  1.706  0.819  1.686  1.046  1.617  1.615  1.6  1.348  2.031  2.452  
 
 SNX4  8723  3  125165487  125239058  3q21.2  1.536  2.535  1.254  1.437  2.657  1.285  1.886  2.757  1.736  1.984  2.368  1.808  1.671  2.36  1.602  2.271  2.009  2.775  4.19  2.235  1.674  2.299  1.382  0.787  1.666  2.542  1.096  1.588  2.136  0.972  2.675  2.851  2.137  1.263  0.994  1.955  1.808  1.188  2.663  2.411  1.632  1.352  2.478  2.191  1.536  2.132  1.177  1.391  1.502  3.774  1.746  0.636  1.668  1.187  1.885  1.558  1.455  1.233  1.864  2.542  
 
 OSBPL11  114885  3  125247701  125314381  3q21  1.163  1.389  0.938  1.304  1.089  0.994  1.301  1.526  1.123  1.39  1.578  0.866  1.516  1.662  1.348  1.062  0.825  1.181  2.453  0.31  0.563  1.464  1.045  1.213  1.235  1.158  1.07  1.562  1.346  0.862  1.743  1.464  2.055  1.218  0.716  1.496  1.608  1.071  1.37  1.931  1.089  0.877  1.484  1.146  2.141  2.153  0.236  1.464  2.142  2.563  1.487  0.81  1.144  0.992  0.919  1.949  0.968  0.906  1.522  1.898  
 
 LOC101927056  101927056  3  125546079  125604920  3q21.2  0  1.44  0.73  0  0.194  0  0.28  0.568  0  0.631  0  0  0  0.138  0.624  0  0  0.271  0  0  0  0.273  0.594  0.562  0  0.387  0.421  0.274  0  0.069  0  0.25  0  0  0  0  0  0.671  0.929  0  0  0.114  0.927  0  0  0.525  0  0.536  0  1.26  0.23  0.439  0.758  0  0.494  0  0.281  0  0  0  
 
 FAM86JP  100125556  3  125635443  125648867  3q21.2  1.43  0.299  0.511  1.221  1.111  0.582  0.778  0.488  0.737  0.471  0.751  0.885  1.052  1.345  1.241  1.7  1.487  0.273  1.006  0.875  0.345  0.824  1.51  1.195  1.399  0.736  1.55  1.259  1.781  1.134  1.213  1.383  1.732  1.224  1.192  1.34  1.866  0.379  0.978  2.006  1.026  1.216  1.826  0.742  0.317  0.538  1.806  0.637  0.566  1.541  1.93  0.98  0.817  0.387  1.56  0.796  0.177  0.646  0.775  0.955  
 
 ALG1L  200810  3  125648117  125655887  3q21.2  2.73  0  0  0  1.53  0  0.274  0  0  0.104  0  1.119  0.704  0.225  1.931  2.423  0.251  0  1.494  0  0  0.335  0.759  0.33  0  0.259  0.674  0.224  0  0.13  0.771  0.254  0.356  0.32  0  0.656  0.368  0.554  0.073  0.603  0.664  0.718  0.218  0  0  0.343  0.218  0  0  0  0.369  0.381  0  0  0.168  0  0  0  0  0  
 
 ROPN1B  152015  3  125687986  125702296  3q21.2  0  0.274  0.212  0  0.088  0  0.184  0.076  0.131  1.779  0.082  0.375  0  0.287  0  0.267  0.121  0  0.28  0  0  0.315  0.335  0  0  2.034  2.591  2.244  2.793  1.36  4.269  2.684  3.182  2.939  0  0  0  0  0.112  0.228  0.18  0.218  0.074  0  0  0.233  0.562  0  0  0.183  0  0.37  0.076  0.125  0.103  0.177  0  0  0  0  
 
 SLC41A3  54946  3  125725199  125820391  3q21.2  2.515  2.569  2.505  2.252  2.091  1.804  4.163  3.139  3.467  3.429  2.618  3.089  2.907  3.551  3.356  3.565  3.025  2.05  4.693  3.595  1.465  3.417  2.619  2.183  2.729  2.934  4.293  3.275  3.75  2.649  3.705  3.774  3.572  2.651  1.455  2.685  4.271  3.359  2.334  3.067  2.614  2.483  3.37  2.373  1.95  2.142  3.316  2.36  2.449  4.127  2.81  1.595  2.799  1.693  2.111  2.662  2.082  2.311  2.018  3.108  
 
 ALDH1L1  10840  3  125822403  125900029  3q21.3  0  0  0.128  0  0  0.219  0.074  0.051  0.116  0.037  0.046  0.099  0  0.053  1.84  0.161  0.061  0.061  0.053  0.062  0  0.147  0.854  0  0.058  0.047  0.158  0.931  0.049  0.327  0  0  0.223  0.158  0.136  0  0.052  0  0.069  0.228  0.053  0.147  0.55  0  0  0.227  1.565  0.078  0.065  0.622  0  0.428  0.029  0.103  0.062  0  0  0.053  0  0  
 
 ALDH1L1-AS2  100862662  3  125898907  125929011  3q21.3  0  0.129  0.217  0  0  0.052  0  0.069  0.128  0.037  0.032  0.056  0  0.04  0.234  0  0.099  0.074  0  0.064  0  0.119  0.254  0  0  0.035  0.154  0.047  0  0.114  0  0  0  0.342  0.062  0  0  0  0.107  0  0  0.334  0.069  0.051  0.07  0.069  0.062  0  0  0.07  0.044  0.541  0  0.099  0.057  0.074  0  0  0  0  
 
 KLF15  28999  3  126061477  126076236  3q21.3  0.136  0.599  0.219  0.221  0.181  0.548  0.064  0.078  1.766  0.13  0.085  0.141  0.166  0.404  1.659  0.398  0.132  0.43  0  0.592  0.746  0.241  2.216  2.029  0.083  0.477  1.266  1.819  1.019  1.675  1.863  1.534  2.217  1.833  0.129  0.121  0.228  0.074  0.194  0.323  0.225  0.219  0.223  0.494  0.233  0.261  0.238  0.119  0  0.253  0.062  0.731  0.029  0.114  0.145  0.093  0.067  0.639  0.098  0.059  
 
 LOC100506907  100506907  3  126111874  126113641  -  0  0  0  0  0  0  0  0  0  0  0  0  0  0  0  0  0  0  0  0  0  0.135  0.477  0  0  0  0.08  0  0  0.1  0  0  0  0.336  0  0  0  0  0  0  0  0  0  0  0  0  0  0  0  0  0  0.491  0  0  0  0  0  0  0  0  
 
 CFAP100  348807  3  126113781  126155398  3q21.3  0  0.051  0.159  0  0.12  0  0.124  0.062  0.111  0.131  0.048  0.163  0.065  0.105  0  0.099  0.098  0.189  0.066  0.209  0  0.145  0.273  0  0  0.069  0.273  0.042  0.076  0.25  0  0  0  0.193  0  0  0.083  0  0.095  0  0.061  0.089  0.27  0  0  0.114  0.102  0  0  0  0  0.587  0  0.141  0.137  0  0  0  0  0  
 
 ZXDC  79364  3  126156443  126194762  3q21.3  2.72  1.286  1.638  1.922  2.627  1.187  2.285  2.687  2.302  2.606  2.378  2.02  2.511  2.44  2.74  2.654  1.989  1.293  3.483  2.258  1.393  2.689  1.814  1.622  1.558  2.401  2.67  2.443  2.485  2.746  2.883  2.783  2.557  1.612  1.288  2.09  2.775  2.436  1.989  2.712  2.197  1.634  2.239  2.086  1.989  2.387  2.04  2.492  2.318  3.352  1.92  1.704  1.92  1.297  1.867  1.71  1.799  1.909  1.605  2.1  
 
 UROC1  131669  3  126200007  126236616  3q21.3  0  0.045  0.147  0  0.022  0  0.035  0  0.095  0.051  0.023  0.031  0  0.037  0  0.025  0  0.078  0.14  0.346  0  0.078  0.305  0.126  0  0  0.115  0.069  0  0.176  0  1.173  0  0.186  0  0  0  0  0.039  0  0  0.192  0.039  0  0  0.067  0.034  0  0  0.067  0.038  0.216  0  0.077  0.021  0  0  0.043  0  0  
 
 CHST13  166012  3  126243130  126262134  3q21.3  0  0.054  0.255  0  0.155  0.197  0.165  0.153  0.255  0.333  0.157  0.188  0.182  0.233  0  0.226  0.136  0.357  0.123  0.986  0  0.575  0.597  0.234  0  0.076  0.286  0.15  0.135  0.646  0.085  0  0  0.251  0.492  0.846  0.217  0  0.744  0.945  0.073  1.155  0.769  0.082  0.226  0.238  0.085  0.841  0.091  2.013  0.173  1.721  0.113  0.458  1.94  0.252  0.062  1.031  0.87  0.136  
 
 C3orf22  152065  3  126245841  126277808  3q21.3  0  0  0  0  0  0  0  0  0.246  0.089  0  0  0  0.042  0  0  0  0  0  0  0  0.102  0.58  0  0  0  0.11  0  0  0.142  0  0  0  0.29  0  0  0  0  0.072  0  0  0.382  0.095  0  0  0  0.058  0  0  0.06  0  0.414  0.055  0.078  0  0  0  0.143  0  0  
 
 TXNRD3NB  645840  3  126290621  126327398  3q21.3  1.179  0.225  0.276  0.379  0.102  0  0.597  0  0  0.322  0  0  0  0.684  0.205  0.146  0  0  0  0.22  0  0.111  0.651  0  0  0  0.348  0  0  0.465  0  0  0  0.652  0  0.28  0.274  0.24  0.208  0.239  0  0.453  0.143  0  0  0  1.345  0  0  0  0  0  0  0  0.131  0  0  0.255  0  0  
 
 TXNRD3  114112  3  126325894  126373967  3q21.3  1.268  1.246  0.517  0.575  1.542  0.362  1.001  1.188  0.649  1.436  0.802  0.154  1.386  1.802  1.296  1.002  1.472  0.934  1.241  0.881  0.22  0.944  0.539  0.307  0.169  1.212  1.485  0.933  1.373  1.373  1.422  1.76  1.404  0.867  0.405  1.181  1.088  0.865  2.056  1.263  0.941  0.616  1.705  0.636  0.499  0.934  1.684  0.42  0.723  2.082  1.07  0.854  0.718  0.365  1.544  0.91  0.654  0.832  0.851  1.777  
 
 NUP210P1  255330  3  126380923  126390782  3q21.3  0  0  0  0  0  0  0  0  0  0  0  0  0  0  0  0  0  0  0  0  0  0.063  0  0  0  0  0  0  0  0.052  0  0  0  0.248  0  0  0  0  0  0  0  0  0.141  0  0  0  0  0  0  0  0  0.185  0  0  0  0  0  0  0  0  
 
 CHCHD6  84303  3  126423062  126679263  3q21.3  2.641  0.86  2.172  1.825  2.612  2.186  3.909  2.795  2.055  2.423  2.364  2.475  2.761  3.397  2.553  3.068  3.022  0.193  4.347  1.951  2.049  2.988  1.661  2.442  2.374  3.786  4.52  3.098  3.434  4.166  6.595  4.608  4.369  3.423  2.323  2.262  3.032  3.429  2.283  2.942  2.986  2.03  3.23  2.83  3.278  3.312  1.606  3.757  2.453  5.161  2.991  1.758  2.41  1.699  2.809  2.936  1.518  3.364  2.323  3.071  
 
 PLXNA1  5361  3  126707436  126756235  3q21.3  2.279  1.496  3.365  1.921  2.121  1.047  2.942  4.176  2.688  3.382  2.573  2.027  2.939  1.72  3.147  3.611  1.423  0.46  2.155  0.679  2.493  1.546  0.925  1.778  2.241  3.935  3.658  3.911  3.458  3.512  4.025  4.099  3.552  1.585  1.373  2.459  3.89  4.433  1.9  4.04  2.273  1.993  2.124  2.38  2.385  2.413  1.612  3.243  2.486  3.235  2.562  2.014  2.66  2.047  1.592  3.099  2.568  1.9  1.693  2.64  
 
 C3orf56  285311  3  126911973  126917027  3q21.3  0  0  0.17  0  0  0  0  0  0.134  0.051  0  0  0  0.033  0  0  0  0.072  0.063  0.08  0  0.227  0.36  0  0  0  0.083  0.046  0  0.104  0  0  0  0.334  0  0  0  0  0.064  0  0  0.526  0.047  0  0  0  0  0  0  0  0.055  0.258  0  0.11  0  0  0  0  0  0  
 
 LOC101927123  101927123  3  127041149  127109513  -  0  0.095  0  0  0  0  0  0  0  0  0  0  0  0  0  0  0  0.2  0  0  0  0.144  0  0  0  0  0.061  0.067  0  0.085  0  0  0  0.313  0  0  0  0  0.153  0  0  0  0  0  0  0  0.053  0  0  0  0  0  0  0.101  0  0  0  0  0  0  
 
 LINC01471  101927149  3  127216443  127256623  3q21.3  0  0  0.223  0  0.087  0  0.087  0  0.138  0.128  0.112  0.284  0  0.199  0  0.193  0.3  0  0.152  0  0  0.277  0.291  0  0  0  0.141  0.06  0.142  0.095  0  0  0  0.239  0  0  0  0  0.127  0  0  0.332  0.042  0  0  0  0.084  0  0.217  0  0.074  0.435  0.066  0.514  0.151  0.175  0.136  0  0  0  
 
 TPRA1  131601  3  127291906  127309602  3q21.2  2.919  0.659  3.302  3.501  3.066  2.311  5.064  4.477  3.619  3.662  2.996  2.617  3.518  3.113  3.202  3.408  2.849  0.074  4.346  3.418  1.954  2.961  3.14  2.706  2.814  2.815  4.145  3.802  3.212  4.091  3.447  4.051  4.285  2.988  2.525  2.878  3.646  3.555  4.282  3.642  2.997  3.178  3.121  2.893  3.017  3.581  4.05  3.792  3.851  5.123  2.776  3.016  3.911  3.055  4.228  3.503  2.955  3.45  4.586  4.067  
 
 MCM2  4171  3  127317199  127341278  3q21  4.806  1.614  2.641  3.112  4.11  2.846  4.642  4.471  2.584  4.13  4.291  4.157  4.588  4.754  5.148  4.853  4.237  1.32  6.146  3.893  3.986  4.904  3.541  3.947  2.749  2.37  3.908  4.182  3.643  3.242  2.894  3.766  5.015  2.906  3.082  3.772  4.662  3.313  2.322  5.508  3.88  3.745  4.1  4.155  3.251  3.565  3.407  4.31  3.667  5.87  3.513  3.572  4.344  2.908  3.715  3.444  2.821  3.85  4.506  4.597  
 
 PODXL2  50512  3  127348001  127391653  3q21.3  1.642  0.073  0.5  4.744  1.678  1.154  2.103  2.378  3.26  1.381  1.932  1.1  3.039  0.072  3.01  1.908  2.985  0.228  0.398  1.743  2.636  0.352  2.692  3.569  0  2.041  3.589  1.592  2.905  3.379  3.922  4.223  3.112  3.085  1.33  2.407  1.135  2.9  0.613  2.878  0.082  3.581  2.228  3.041  2.552  2.392  0.05  3.177  2.657  4.268  2.992  2.664  0.106  1.675  1.501  1.909  1.032  2.024  3.083  0.801  
 
 ABTB1  80325  3  127391780  127399769  3q21  0.835  0.582  2.195  1.019  0.964  0.768  1.397  1.847  2.647  1.629  0.67  1.039  1.249  1.896  1.517  1.339  1.941  0.164  2.787  0.746  0  1.47  1.833  1.029  1.265  2.31  2.427  2.438  2.408  1.627  1.703  1.87  2.713  1.737  1.235  1.076  1.49  1.88  1.538  2.178  0.591  1.038  1.113  1.075  0.782  0.764  0.834  2.003  1.439  3.293  1.905  1.337  0.875  1.042  1.529  1.286  1.138  1.43  2.023  1.875  
 
 MGLL  11343  3  127407904  127542093  3q21.3  0.754  1.043  4.368  0.224  1.887  1.338  3.561  4.927  3.172  1.429  0.397  2.281  2.49  0.652  0.111  3.462  1.711  0.102  0.368  0.167  0.066  0.268  2.654  3.025  1.15  1.015  0.727  3.062  1.618  0.274  1.542  2.81  1.967  2.39  3.094  3.517  1.664  2.93  4.063  0.493  0.887  0.927  0.058  3.146  0.332  3.376  3.247  4.408  4.504  4.973  5.316  3.711  3.414  4.283  4.662  3.48  3.888  5.077  4.731  4.686  
 
 KBTBD12  166348  3  127641901  127706514  3q21.3  0.647  0.045  0.669  0.132  0.456  0.193  0.926  0.191  0.234  0.54  0.356  0.268  0.461  0.303  0.373  0.532  0.404  0.059  0.365  0.359  0.045  0.511  0.418  0.334  0.269  0.216  0.903  0.33  0.598  0.409  0.115  0.198  0.23  0.355  0.183  0.16  0.365  0.145  0.738  0.465  0.066  0.157  0.091  0.08  0.075  0.147  0.34  0.583  0.047  1.466  0.256  0.323  0.567  0.241  0.416  0.308  0.618  0.066  0.237  0.208  
 
 SEC61A1  29927  3  127771211  127790526  3q21.3  4.713  2.554  6.086  5.19  4.907  4.173  6.873  6.464  6.019  5.97  5.563  5.081  5.385  4.942  5.089  5.161  4.466  0.927  5.839  4.028  4.066  4.188  5.261  5.139  4.872  4.994  6.156  5.597  5.64  5.511  5.611  6.764  6.479  4.692  3.559  5.309  5.641  5.386  4.949  6.105  4.496  4.647  5.012  4.694  4.633  5.4  2.96  4.911  4.498  6.534  5.401  3.244  5.348  4.86  4.526  5.323  5.995  4.486  4.999  6.252  
 
 RUVBL1-AS1  100874089  3  127794652  127797899  -  0  0  0  0  0.317  0  0  0.326  0  0.142  0  0  0  0.177  0  0.167  0  0  0  0  0  0.171  0  0  0  0  0.105  0.158  0  0.119  0  0  0  0  0  0  0  0  0.136  0  0  0  0.432  0  0  0.211  0  0.34  0  0  0  0.503  0.184  0.129  0  0  0  0  0  0  
 
 RUVBL1  8607  3  127799799  127842671  3q21  4.773  2.713  3.625  4.645  4.511  2.964  5.02  4.401  3.423  3.81  4.382  3.892  4.102  5.219  4.125  4.762  4.285  1.661  6.036  4.223  4.262  4.733  3.593  3.766  4.128  3.276  3.934  4.578  3.864  4.192  4.468  6.258  4.75  3.588  3.809  4.02  5.344  4.516  4.026  5.86  3.881  4.353  5.082  3.95  4.57  3.822  2.76  4.018  3.396  6.112  4.184  3.383  4.438  2.648  3.901  3.947  4.041  4.589  4.025  4.869  
 
 EEFSEC  60678  3  127872299  128127489  3q21.3  2.966  0.494  1.927  2.649  2.661  1.78  3.223  2.925  2.846  2.808  2.644  2.693  2.414  2.534  2.641  2.938  2.658  0.48  3.173  2.629  1.967  2.704  2.121  2.484  1.443  2.312  3.001  3.22  2.161  2.513  2.73  2.96  3.46  2.135  1.833  2.577  2.656  2.847  2.36  3.434  2.098  2.789  2.542  1.998  2.252  2.711  1.912  2.336  2.23  3.419  2.551  1.785  2.604  1.284  2.012  1.442  2.719  2.585  2.892  2.623  
 
 DNAJB8  165721  3  128181274  128186091  3q21.3  0  0  0.208  0  0.067  0  0  0  0.192  0.049  0.043  0  0  0.034  0  0.072  0  0.156  0  0.05  0  0.203  0.465  0  0  0  0.17  0  0  0.107  0  0.061  0  0.298  0  0  0  0  0.042  0  0  0.204  0.037  0  0  0.035  0.048  0  0  0  0  0.646  0  0.127  0.093  0.175  0.041  0  0  0.078  
 
 DNAJB8-AS1  285224  3  128182436  128191160  3q21.3  0  0  0.177  0  0.068  0  0  0  0  0.03  0  0  0  0.023  0  0  0  0  0  0  0  0.061  0.298  0  0  0  0.154  0  0  0.037  0  0  0  0.4  0  0  0  0  0  0  0  0  0.033  0  0  0.053  0.022  0  0  0  0  0.2  0  0  0  0  0  0  0  0  
 
 GATA2  2624  3  128198264  128212030  3q21.3  1.374  0.73  1.916  0.805  2.453  2.388  0.932  1.084  0.491  0.764  1.624  0.372  1.997  1.805  2.038  3.96  0.399  0.447  0.157  2.034  4.739  0.278  0.389  0.441  1.052  0.152  0.315  0.275  0.433  1.364  0.087  2.616  1.179  0.775  0.53  1.779  2.61  2.073  2.29  1.919  1.029  2.501  2.413  2.031  0.406  0.984  0.964  2.156  0.479  3.075  0.081  1.792  1.11  0.398  0.137  0.185  1.643  2.151  0.845  0.928  
 
 GATA2-AS1  101927167  3  128208045  128222050  3q21.3  0  0.845  0.445  0.115  1.219  0.559  0.203  0.091  0.346  0.321  0.623  0.248  1.469  0.678  0.967  2.093  0  0.678  0  0.47  0.922  0.125  0.337  0  0.192  0  0.122  0.051  0.048  0.131  0  0.045  0  0.347  0  0.786  0.455  0.552  1.438  0.278  1.273  0.844  1.506  0.573  0  0.237  1.394  0.199  0  0.715  0.062  0.728  0.416  0.154  0.083  0.084  0.638  0.348  0  0.19  
 
 LOC90246  90246  3  128226677  128229429  3q21.3  0  0.345  0.216  0  0.768  0.081  0.039  0  0.224  0.096  0.369  0  0.693  0.3  0.291  0.351  0  0.09  0  0.086  0.231  0.05  0.305  0  0  0  0.09  0  0  0.065  0  0  0  0.503  0  0.197  0.162  0.233  0.633  0.076  0.577  0.205  1.059  0.163  0.087  0.085  1.122  0.054  0.121  0.077  0  0.609  0.173  0.054  0  0  0.203  0  0  0  
 
 LINC01565  23434  3  128290842  128294929  3q21  0  0  0.265  0  0  0.086  0.031  0.031  0.109  0.074  0.039  0.044  0  0.053  0  0.093  0  0.071  0  0.098  0.137  0.091  0.409  0  0  0  0.103  0  0  0.099  0  0.056  0  0.461  0  0  0  0  0.081  0  0.099  0.602  0.214  0  0  0.072  0.047  0  0  0  0.036  0.275  0  0.093  0  0  0.04  0  0  0  
 
 RPN1  6184  3  128338812  128369719  3q21.3  4.723  5.074  5.493  6.466  5.449  4.135  6.48  6.703  6.402  6.183  5.506  5.66  5.545  5.6  5.812  5.71  5.721  4.798  6.368  5.047  4.262  3.992  5.712  5.505  5.099  5.176  5.575  5.649  5.683  5.749  5.945  6.96  5.663  4.129  4.025  5.236  6.186  5.551  5.137  6.427  4.775  4.969  5.149  5.166  5.4  5.653  2.776  5.313  5.241  7.027  5.389  3.882  5.662  4.588  4.718  5.57  6.232  5.03  5.425  6.24  
 
 RAB7A  7879  3  128444978  128533641  3q21.3  5.659  6.101  5.2  5.244  6.072  4.293  6.625  6.203  5.761  5.781  5.398  5.804  5.726  6.276  5.04  6.106  5.899  6.216  6.247  6.577  4.471  5.616  4.657  4.564  5.81  6.815  6.955  6.711  6.965  6.454  7.235  7.808  7.235  6.022  4.438  4.928  5.641  5.891  6.786  6.115  5.184  4.449  6.235  4.569  5.601  5.571  5.394  5.455  5.162  6.678  5.74  4.219  5.353  4.924  5.82  5.578  5.933  5.524  5.624  6.276  
 
 LOC653712  653712  3  128580350  128590384  3q21.3  0.358  0  0.19  0  0.189  0  0.076  0.347  0.282  0.294  0.275  0.147  0.355  0.245  0.384  0.143  0.218  0.207  0.213  0  0  0.205  0.344  0  0  0.202  0.681  0.416  0.245  0.456  0.138  0.31  0.362  0.31  0  0.199  0.177  0  0.174  0.739  0  0  0  0.306  0.215  0.341  0.081  0.472  0.183  0.18  0.187  0.501  0.163  0.206  0.15  0.166  0  0  0.164  0.117  
 
 ACAD9  28976  3  128598332  128631957  3q21.3  2.902  1.104  2.011  2.66  3.191  1.822  3.076  3.298  2.106  2.849  2.554  2.901  2.927  2.936  3.266  3.3  2.191  0.803  4.245  3.003  1.97  2.853  1.595  2.071  2.19  2.599  3.069  2.659  2.958  2.6  3.502  3.699  3.364  1.743  1.699  2.611  3.001  2.518  2.651  3.473  2.428  2.378  2.922  2.369  2.301  3.121  2.474  2.841  2.35  4.512  2.789  1.74  2.589  1.86  2.833  2.455  2.131  2.382  2.916  3.298  
 
 KIAA1257  57501  3  128689781  128712986  3q21.3  0.193  0.092  0.1  0  0.801  0  0  0  0.221  0  0.133  0.251  0.311  0.76  0.887  0.147  0.528  0.486  0.11  0.475  0  0.232  0.477  0  0  0  0.1  0.053  0  0.096  0  0  0  0.217  0  0  0  0  0.066  0.095  0  0.078  0.073  0  0  0.073  0.039  0  0  0.084  0.052  0.55  0  0.115  0  0  0  0  0  0  
 
 EFCC1  79825  3  128720471  128759585  3q21.3  0.145  0.084  0.301  0.412  0.179  0.166  0.135  0.113  0.42  0.154  0.118  0.194  0.268  0.429  0.376  0.256  0.208  0.225  0.174  0.337  0  0.408  0.746  0.182  0.069  0.161  0.335  0.167  0.127  0.236  0.248  0.18  0.122  0.448  0.06  0  0.168  0.095  0.271  0.228  0.098  0.825  0.083  0  0.262  0.221  0.083  0.146  0  0.224  0.154  1.207  0.145  0.2  0.168  0.118  0.105  0.078  0.143  0.086  
 
 GP9  2815  3  128779609  128781254  3q21.3  0.271  0  0.215  0  0  0  0  0.092  0  0.214  0.127  0.137  0  0.1  0  0.166  0.27  0  0.119  0.212  0  0.335  0.651  0  0  0  0.364  0  0  0.227  0  0  0  0.243  0  0  0  0  0.164  0  0  0.442  0.097  0  0  0.131  0.091  0  0  0  0  1.243  0  0.235  0.11  0.216  0  0  0  0  
 
 RAB43  339122  3  128806411  128840993  3q21.3  1.281  0.369  1.808  1.86  1.608  1.183  1.577  3.757  2.196  2.589  2.226  2.095  1.683  1.061  2.232  2.38  1.364  0.4  3.197  0.641  0.689  1.409  1.157  1.145  2.2  2.176  1.942  2.894  3.316  1.474  2.285  3.466  2.905  1.573  0.731  1.46  3.137  2.345  1.786  2.944  2.168  1.508  1.338  1.752  1.968  2.816  0.957  1.787  2.206  2.29  2.294  1.562  2.361  2.201  1.644  2.323  2.142  1.565  2.411  2.844  
 
 ISY1-RAB43  100534599  3  128806411  128880073  3q  0  0  0  0  0  0  0  0.023  0  0  0  0  0.906  0.038  0.826  0  0  0  0.073  0  0.296  0  0  0.18  0  0.242  0.038  0.68  0.27  0.12  0  0  0.883  0  0  0  0.742  0  0  0  0  0  0  0  0  0  0  0  0.913  0.556  0.825  0  0  0  0  0.188  0.336  0.718  0  0  
 
 ISY1  57461  3  128846258  128880073  3q21.3  2.051  1.586  1.844  2.18  2.074  1.341  2.513  2.687  2.031  2.419  2.225  1.903  1.746  2.757  1.819  2.505  2.744  0.87  3.795  3.174  1.687  3.111  2.205  1.449  1.976  1.902  1.769  1.661  1.526  1.248  1.937  2.784  2.971  2.22  1.044  1.385  1.919  1.761  2.964  2.26  1.476  1.54  1.866  1.713  2.062  2.127  1.66  2.011  1.744  3.717  1.996  1.215  1.928  1.213  2.23  1.689  1.527  1.769  2.152  2.548  
 
 CNBP  7555  3  128886657  128902810  3q21  4.903  5.901  3.894  5.05  4.655  3.28  5.384  5.24  4.887  4.468  5.043  4.622  5.158  6.207  4.807  5.217  5.303  6.943  6.857  5.11  4.334  5.126  4.938  4.25  4.77  4.569  4.606  4.955  4.979  4.677  5.405  6.308  5.14  4.201  3.605  4.22  4.962  4.477  5.128  5.191  4.577  4.026  5.188  4.419  4.616  4.65  4.556  4.604  4.119  7.183  5.038  3.559  4.585  3.27  4.572  4.38  4.091  5.08  4.874  5.243  
 
 COPG1  22820  3  128968452  128996616  3q21.3  4.378  2.899  4.084  3.72  4.502  2.767  5.125  6.114  3.387  5.032  4.808  4.204  4.524  4.721  4.593  3.783  4.171  1.212  4.42  2.568  3.249  2.681  3.099  3.7  3.363  5.252  4.995  5.353  5.333  4.712  5.348  6.133  5.728  3.192  2.513  4.28  5.082  4.673  3.208  5.304  4.105  2.941  3.877  3.837  3.776  4.872  2.238  4.071  3.791  5.173  4.978  2.726  4.352  3.717  3.591  4.587  4.107  4.064  4.24  5.251  
 
 HMCES  56941  3  128997683  129024135  3q21.3  4.025  1.862  2.788  3.123  4.434  2.193  3.716  3.407  2.892  3.251  3.79  3.837  4.167  4.485  3.518  4.173  3.675  0.91  5.332  1.764  3.026  3.889  2.191  1.779  2.757  3.486  3.902  3.529  3.583  2.931  4.261  4.741  4.203  3.119  2.57  3.689  3.913  3.071  3.696  4.734  3.533  3.042  4.555  3.489  2.899  3.323  2.076  3.51  3.917  5.516  3.203  2.128  2.684  2.186  3.6  3.235  2.65  3.431  3.662  3.673  
 
 H1FX  8971  3  129033613  129035120  3q21.3  3.964  1.371  4.447  6.745  4.918  3.883  4.575  5.13  5.89  4.525  4.651  4.024  3.412  3.618  3.671  5.333  5.03  1.027  7.045  3.584  3.503  5.157  4.65  4.279  3.582  3.246  4.602  3.165  3.845  3.224  4.33  4.04  4.904  4.96  4.619  4.099  4.431  4.183  4.038  4.665  4.379  6.877  3.459  2.976  4.412  3.661  3.114  4.723  2.975  6.877  3.584  4.181  2.908  1.796  4.758  3.375  2.317  5.201  3.921  3.76  
 
 H1FX-AS1  339942  3  129035113  129043412  3q21.3  0.556  0.23  0.426  0.545  0.499  0.274  0.574  0.34  0.441  0.951  0.846  0.066  0  0.215  0.521  0.179  0.048  0.08  0.918  0.092  0  0.4  0.469  0.288  0.204  0.567  0.944  0.463  0.585  0.119  0.345  0.609  0.597  0.526  0.282  0.612  0.556  0.242  0.297  0.54  0.227  1.102  0.447  0.402  0.537  0.342  0.369  0.929  0.177  1.231  0.32  0.627  0.634  0.178  0.733  0.391  0.525  0.154  0.442  0.176  
 
 RPL32P3  132241  3  129101676  129118282  3q21.3  0.815  0.37  0.517  0.705  1.177  0.407  0.87  0.844  0.814  1.153  1.187  0.61  0.657  0.959  0.782  1.04  0.944  0.321  2.118  1.184  0.488  1.152  1.108  0.579  0.58  0.851  1.343  0.949  0.875  1.184  1.189  1.223  1.787  0.976  0.589  0.638  0.647  0.472  1.198  0.792  0.764  0.613  0.564  0.588  0.855  0.981  0.704  0.802  0.97  1.439  0.593  0.465  0.552  0.539  0.928  0.424  0.464  0.804  0.782  0.555  
 
 SNORA7B  677797  3  129116052  129116191  3q21.3  0  0  0  0  0  0  0  0  0  0  0  0  0  1.66  0  0  0  0  0  0  0  2.317  0  0  0  0  0  2.068  0  0  0  0  0  0  0  0  0  0  0  0  0  0  0  0  0  0  0  0  0  0  0  0  0  1.748  0  0  0  0  0  0  
 
 EFCAB12  90288  3  129120163  129147494  3q21.3  0  0.051  0.221  0.22  0.436  0  0.054  0.096  0.128  0.107  0.242  0.165  0.09  0.155  0.323  0.074  0  0.226  0.143  0.179  0.16  0.255  0.42  0.272  0  0.049  0.136  0.153  0  0.112  0.103  0.134  0  0.332  0.329  0.806  0.052  0  0.097  0.189  0.125  0.128  0.16  0.079  0.115  0.166  0.068  0  0  0.242  0.083  0.335  0  0.16  0.106  0  0  0.09  0.079  0.056  
 
 MBD4  8930  3  129149786  129159022  3q21.3  2.444  3.428  1.862  2.541  2.87  1.079  2.871  2.667  1.738  2.484  2.826  1.935  2.371  2.383  2.044  2.956  2.332  4.322  4.071  3.339  2.142  2.952  2.502  1.249  1.624  2.53  1.933  1.7  1.944  0.904  2.434  2.741  2.473  2.07  1.365  2.407  2.024  1.341  2.923  2.691  1.586  1.925  2.513  2.106  1.651  2.436  2.282  1.673  2.086  3.51  2.25  1.42  1.42  1.48  1.985  1.928  1.51  1.748  2.052  2.417  
 
 IFT122  55764  3  129158878  129239350  3q21  1.477  0.426  0.768  1.379  2.5  0.496  1.727  1.997  0.846  2.242  1.865  1.353  0.755  1.397  1.224  1.515  0.854  0.362  3.021  1.841  0.761  1.893  0.6  0.7  0.848  1.454  1.864  1.753  1.606  1.459  1.532  2.109  1.543  0.659  1.175  1.972  1.544  1.375  0.97  2.897  1.33  1.192  1.496  0.979  1.688  1.765  1.703  1.643  1.633  2.655  1.254  1.297  1.284  0.715  1.139  1.023  1.092  1.161  1.669  1.579  
 
 RHO  6010  3  129247481  129254187  3q21-q24  0  0.034  0.166  0  0.066  0  0  0.033  0.095  0.024  0.036  0  0  0.025  0  0  0.063  0.079  0  0.049  0  0.14  0.511  0  0  0  0.09  0.041  0  0.057  0  0  0  0.395  0.055  0  0  0  0.046  0  0  0.098  0.034  0  0  0.085  0.039  0  0  0  0.038  0.539  0.027  0.088  0.056  0.057  0  0  0  0  
 
 H1FOO  132243  3  129262056  129270310  3q22.1  0  0  0.231  0  0.078  0  0  0.098  0  0.129  0  0  0  0  0  0  0  0  0  0  0  0.203  0.494  0  0  0  0.104  0  0  0.046  0  0  0  0  0  0  0  0  0  0  0  0.276  0.049  0  0  0  0  0  0  0  0  0.734  0  0  0  0  0  0  0  0  
 
 PLXND1  23129  3  129274055  129325582  3q22.1  1.011  0.191  2.471  0.131  2.525  0.33  1.189  4.413  2.528  4.329  2.899  0.143  0.881  1.353  1.288  0.176  0.446  0.292  1.389  1.62  1.195  1.012  1.099  2.08  0.335  3.737  0.555  4.364  0.985  1.363  3.217  1.298  1.092  0.42  1.385  3.39  3.034  3.16  1.563  2.707  1.034  1.906  3.235  2.866  1.797  2.027  1.075  1.904  1.615  1.566  0.514  1.497  1.169  1.865  1.553  2.124  2.174  0.876  1.243  2.249  
 
 TMCC1  23023  3  129366634  129612419  3q22.1  1.241  0.691  0.892  0.859  1.546  0.347  0.968  1.813  1.78  1.381  2.153  1.027  1.344  1.153  1.312  1.232  0.993  1.439  1.399  0.635  0.317  1.045  0.774  0.582  0.658  1.101  0.589  1.01  0.743  0.696  0.995  1.105  0.846  0.778  0.849  1.56  1.022  1.493  1.91  1.354  1.396  0.531  2.741  0.875  1.462  1.921  0.531  0.949  1.99  1.265  1.763  0.795  0.88  1.607  1.035  0.945  1.591  0.834  1.1  1.986  
 
 TMCC1-AS1  100507032  3  129612713  129627755  3q21-q22  0.227  0.942  0.389  0.717  0.248  0.071  0.219  0.167  0.118  0.592  0.148  0.157  0.129  0.294  0.123  0.168  0.046  0.219  1.123  1.141  0.279  0.43  0.571  0.068  0.592  0.626  0.458  0.548  0.693  0.188  1.171  1.537  0.725  0.91  0.503  0.403  0.546  1.157  0.174  0.849  0.171  0.532  1.222  0.28  0.149  0.153  0.348  0.154  0.353  0.389  0.21  0.19  0.621  0.288  0.969  0.607  0.753  0.236  0.382  0.564  
 
 TRH  7200  3  129693235  129696781  3q13.3-q21  0  0.069  0.198  0  0.113  0  0.054  0  0.215  0.091  0  0.127  0  0.081  0  0  0.073  0.076  0.075  0.065  0  0.154  0.384  0.056  0  0.08  0.25  0  0  0.147  0.112  0  0.08  0.229  0.085  0  0  0  0.062  0  0  0.107  0.054  0  0  0.121  0.103  0  0  0  0  0.505  0  0.198  0.082  0  0  0  0  0  
 
 ALG1L2  644974  3  129800673  129817233  3q22.1  0  0.108  0.252  0  0.132  0  0  0  0.519  0  0  0  0  0  0  0.114  0.165  0.124  0.088  0  0  0.177  0.855  0  0  0.163  0.179  0.155  0  0.123  0  0  0  0.37  0  0  0.129  0  0  0  0  0.284  0.049  0  0  0.095  0.106  0  0  0  0  0.315  0.095  0.108  0  0  0  0  0  0  
 
 FAM86HP  729375  3  129816624  129830276  3q22.1  0.859  0.559  0.385  0.603  1.244  0.479  0.833  0.722  0.57  0.619  0.607  0.662  0.941  0.875  1.144  1.047  1.166  1.093  0.773  0.57  0.174  0.855  1.139  1.235  0.262  1.014  1.643  1.233  1.284  0.821  0.86  1.473  1.265  1.166  0.452  0.354  0.66  0.802  1.134  1.053  0.751  0.623  1.643  0.18  0.395  1.007  1.546  0.472  0.307  0.815  0.544  0.528  0.638  0.394  1.053  0.324  0.237  0  0.837  0.506  
 
 COL6A4P2  646300  3  129931662  129992649  3q22.1  0  0  0.13  0  0.009  0  0.014  0.01  0.067  0.014  0.016  0.013  0  0.01  0  0.02  0.014  0.051  0.01  0.025  0  0.057  0.249  0.041  0  0.01  0.039  0.012  0  0.037  0  0  0  0.24  0.036  0  0  0  0.025  0  0  0.033  0.029  0  0  0.018  0.023  0  0  0.009  0.009  0.326  0  0.025  0.023  0.017  0.014  0  0  0  
 
 COL6A5  256076  3  130064358  130203690  3q22.1  0  0.03  0.088  0  0.037  0.038  0.011  0.031  0.077  0.023  0.014  0.043  0  0.024  0  0.028  0  0.046  0  0.046  0.479  0.072  0.206  0.023  0.02  0.021  0.103  0.013  0.026  0.081  0.016  0.014  0  0.192  0.036  0  0.016  0  0.05  0.021  0.02  0.154  0.061  0.018  0.032  0.047  0.063  0  0.024  0  0.024  0.189  0.017  0.049  0.043  0.039  0  0.027  0  0  
 
 COL6A6  131873  3  130279177  130395888  3q22.1  0.043  0  0.247  0  0.04  0.022  0.038  0.028  0.081  0.036  0.047  0.063  0.058  0.05  0.057  0.046  0.048  0.052  0.053  0.063  0.081  0.076  0.17  0.039  0.034  0.07  0.071  0.052  0.043  0.046  0.026  0.025  0.037  0.274  0.035  0.064  0.062  0.086  0.051  0.053  0.026  0.142  0.033  0.08  0.024  0.02  0.033  0.049  0.078  0.034  0.055  0.293  0.034  0.068  0.045  0.062  0.023  0  0.066  0.051  
 
 PIK3R4  30849  3  130397777  130465696  3q22.1  2.043  1.244  1.072  1.141  2.028  0.658  1.906  1.962  1.233  1.712  1.936  1.512  2.014  2.306  1.973  2.133  1.795  1.682  2.946  1.614  1.408  1.772  0.808  1.086  1.734  2.254  1.519  2.274  1.966  1.476  1.88  2.78  2.752  1.544  0.983  1.678  1.857  1.957  1.691  2.505  1.496  0.987  1.821  1.597  1.938  1.834  0.678  1.705  1.531  2.918  1.759  0.986  1.729  1.099  1.865  1.58  1.379  1.712  2.023  2.572  
 
 ATP2C1  27032  3  130569368  130735555  3q22.1  2.026  3.18  3.231  2.19  2.572  1.355  3.464  2.906  2.271  3.126  2.723  1.423  3.271  3.11  3.054  2.536  3.17  2.687  3.966  2.103  1.571  2.028  2.231  1.809  2.524  2.845  2.108  2.763  2.643  2.144  3.059  3.439  3.144  1.888  1.222  2.705  3.156  3.47  3.423  3.606  2.584  1.541  2.649  2.489  2.251  3.271  1.447  2.69  2.579  4.456  3.337  1.667  2.661  2.161  2.658  2.759  2.713  1.887  3.183  4.472  
 
 ASTE1  28990  3  130732716  130745698  3q22.1  1.175  1.129  0.926  1.195  1.504  0.446  1.567  1.516  0.816  1.441  1.292  0.949  1.158  1.037  0.862  0.789  0.908  1.728  2.673  1.158  0.815  1.258  0.908  0.494  0.781  1.168  1.124  0.902  0.881  1.026  1.211  1.958  1.638  1.051  0.533  0.982  1.114  1.237  1.124  1.409  0.941  0.421  0.55  0.987  0.861  1.338  1.325  0.848  0.676  1.687  1.096  0.572  1.137  0.348  1.193  0.625  0.651  0.722  1.281  1.275  
 
 NEK11  79858  3  130745693  131069309  3q22.1  0.826  0.989  0.46  0.647  1.224  0.231  0.416  0.764  0.349  0.58  0.566  0.379  0.17  0.564  0.25  0.293  0.366  0.152  0.819  0.09  0.107  0.302  0.25  0.224  0.446  0.073  0.126  0.488  0.176  0.069  0.104  0.711  0.096  0.236  0.421  0.682  1.27  0.734  0.583  0.969  0.618  0.416  0.067  0.267  0.602  0.427  1.02  0.294  0.54  1.334  0.409  0.526  0.677  0.253  1.061  0.476  0.304  0.43  1.048  1  
 
 LOC339874  339874  3  131043935  131100319  3q22.1  0  0.284  0.181  0  0.16  0.236  0.12  0.147  0.083  0.092  0.078  0  0.138  0.256  0  0.079  0  0.153  0.073  0.079  0  0.026  0  0  0.081  0  0.048  0.054  0  0.059  0  0  0  0.206  0  0.088  0.258  0.715  0.187  0  0  0.191  0.047  0  0  0.128  0.026  0.237  0  0.161  0  0.204  0.117  0.065  0.266  0.083  0.21  0.366  0.233  0  
 
 NUDT16P1  152195  3  131080688  131083966  3q22.1  0  1.46  0.169  0  0.726  0  0.084  0.136  0.137  0.054  0.093  0.707  2.313  1.837  1.199  1.681  0.054  0.704  0.094  0  1.033  0.073  0.395  0  0  0  0.04  0.059  0  0.058  0  0.059  0  0.312  0  0.379  1.432  2.598  0.693  0.158  0  1.063  0.029  0  0  0.522  1.506  0.96  0  1.798  0.054  1.034  1.432  1.1  1.847  0.995  1.012  1.391  2.237  1.002  
 
 NUDT16  131870  3  131100514  131107674  3q22.1  1.21  0.88  1.693  1.529  1.628  1.626  1.841  1.619  1.404  1.62  1.697  0.982  1.422  1.476  1.574  1.095  1.206  1.421  0  1.165  0.905  0.365  1.585  1.337  0.75  1.131  1.662  1.722  1.555  0.953  1.398  2.353  1.585  1.336  0.894  0.701  1.352  1.704  1.77  1.304  1.116  1.724  0.519  1.202  1.939  1.639  0.98  1.684  1.222  2.263  1.11  1.396  1.483  0.909  1.167  1.181  0.676  1.872  1.349  1.364  
 
 MRPL3  11222  3  131181044  131221860  3q21-q23  4.28  5.239  3.556  3.522  4.62  3.432  4.876  4.942  3.514  4.137  4.983  4.522  4.594  5.432  4.164  4.607  4.692  5.98  6.567  5.601  4.041  4.444  3.87  3.867  4.598  4.178  3.797  4.082  4.268  3.668  4.982  5.986  4.728  3.685  3.585  4.331  4.74  4.69  4.978  4.84  4.407  3.989  4.419  4.117  4.372  4.545  3.658  4.133  3.987  6.28  4.406  3.349  4.665  3.512  4.472  4.52  3.785  4.505  4.708  5.138  
 
 SNORA58  677836  3  131197841  131198077  3q21.2  0  0  0  0  0  0  0  0.935  0  0.594  0  1.048  0  0.676  0  0.714  0  0  0  1.2  0  0.817  0  0  0  0  0.57  0  0  0.642  0  1.021  0  0  0  0.988  0  0  0.94  0  0  0  0  0  0  0  0  0.911  0  0  0  0  0  0  0  0  0  0  0  0  
 
 CPNE4  131034  3  131252403  131758450  3q22.1  0.061  0.049  0.105  0  0.037  0  0.027  0.031  0.154  0.031  0.35  0.048  0  0.02  0  0  0  0  1.147  0.049  0  0.099  0.347  0.096  0  0  0.07  0.019  0  0.037  0  0  0  0.293  0.249  0.153  0  0.081  0.061  0  0.085  0.197  0.041  0  0.707  0.138  0.034  0  0  0  0.027  0.265  0.021  0.021  0.06  0.228  0  0  0  0  
 
 ACPP  55  3  132036210  132087146  3q22.1  0.763  0.052  0.174  0  0.113  0  0.624  0  0.129  0  0.169  1.888  0.94  0.05  0.39  0.319  0.059  0.111  0  1.489  0  0  2.537  0.791  0  0  0.06  0.632  0  0.054  0.15  1.849  0  0.604  0  0  0  0  0.038  0  0.308  0.2  0.033  0.187  0.242  0.325  0.03  0  0  0  0.136  0.157  0  0.031  0  0  0  0  0  0  
 
 DNAJC13  23317  3  132136552  132257876  3q22.1  1.853  1.826  1.353  1.242  2.125  0.884  1.948  2.381  1.172  1.956  1.937  1.1  1.751  2.058  1.663  1.664  1.701  1.642  2.314  1.403  0.684  1.513  0.619  0.573  1.904  2.337  2.439  2.221  2.284  1.95  2.35  2.754  2.79  1.424  1.012  2.051  1.768  2.229  1.28  2.593  1.666  0.947  1.205  1.983  1.563  1.781  1.311  1.743  1.947  3.018  1.96  1.18  1.715  1.129  2.2  1.696  1.701  1.501  2.09  2.965  
 
 ACAD11  84129  3  132276981  132378975  3q22.1  0.593  0.773  1.118  1.8  1.43  0.505  0.964  1.157  1.106  1.43  0.944  0.509  0.469  1.418  0.678  0.544  1.683  1.491  2.016  0.814  0.452  1.052  1.375  0.523  0.96  1.124  1.266  0.967  1.173  1.154  1.514  2.291  0.957  0.678  0.448  0.759  0.927  0.885  0.978  1.924  0.708  0.749  1.308  1.954  0.723  0.661  0.801  1.023  1.452  2.704  0.907  0.638  0.859  0.394  1.535  0.832  1.381  0.452  0.781  1.578  
 
 NPHP3-ACAD11  100532724  3  132276981  132441303  3q  0.045  0.03  0.103  0.164  0.06  0  0.042  0.132  0.125  0.219  0.166  0.056  0.175  0.116  0.137  0.074  0.087  0  0.318  0.038  0  0.058  0.076  0.03  0.092  0.373  0.08  0.201  0.195  0.216  0.346  0.362  0.167  0.142  0.095  0.147  0.136  0.137  0.05  0.228  0.066  0.036  0.2  0.386  0.075  0.11  0.007  0.073  0.209  0.486  0.222  0.132  0.182  0.101  0.106  0  0.375  0  0.103  0.199  
 
 ACKR4  51554  3  132316080  132321485  3q22  0.083  0.126  0.099  0  0.111  0  0.078  0.164  0  0.099  0.11  0.468  0.111  0.347  0.112  0.217  0.152  0  0.04  0  0.098  0.151  0.208  0.082  0.057  0.078  0.143  0.107  0.128  0.052  0.086  0.587  0.812  0.501  0  0.119  0.048  0.065  0.037  0  0.078  0.094  0.027  0.267  0  0.107  0.051  0.07  0.205  0.033  0.08  0.123  0.099  0.256  0.308  0.204  0.149  0  0.137  0.061  
 
 UBA5  79876  3  132373289  132396944  3q22.1  1.752  2.57  1.576  1.061  2.207  0.921  2.145  2.348  1.485  1.916  1.958  1.826  1.757  3.075  2.061  1.922  1.948  3.649  3.862  1.926  1.488  1.696  2.513  1.884  1.703  2.028  1.342  1.881  1.729  0.908  2.324  3.114  2.218  1.507  1.04  1.906  1.501  1.902  1.473  2.367  1.575  1.622  1.658  2.424  1.666  1.21  0.77  1.295  1.777  3.519  1.841  1.048  1.56  0.923  1.502  1.54  2.441  1.491  1.817  2.505  
 
 NPHP3  27031  3  132399452  132441303  3q22.1  0.564  1.158  0.636  0.597  1.148  0.511  0.943  1.521  0.921  1.285  1.156  0.173  0.384  0.861  0.782  0.593  0.816  0.965  1.494  0.537  0.299  0.317  0.713  0.282  1.163  1.398  1.055  0.629  1.067  0.42  1.375  1.485  1.064  0.715  0.359  1.202  0.711  1.15  1.124  1.412  0.529  0.463  0.913  1.333  0.45  0.876  0.939  0.567  1.28  1.355  1.061  0.349  0.841  0.563  1.483  1.043  1.366  0.447  0.895  1.418  
 
 NPHP3-AS1  348808  3  132441185  132593050  3q22.1  0  0  0.13  0  0  0  0  0.039  0.072  0.034  0.041  0  0  0.042  0.056  0  0  0.064  0.034  0.05  0  0.076  0.229  0  0  0  0.073  0.049  0.052  0.07  0  0  0  0.218  0.06  0  0  0  0.02  0  0  0.273  0.022  0  0  0  0.038  0  0  0  0.029  0.139  0.035  0.026  0  0  0  0  0.064  0  
 
 TMEM108  66000  3  132757131  133116619  3q21  0  0  0.199  1.811  0.032  0.244  0  1.394  1.959  1.759  1.802  0  0  0.021  0  0.034  0  0.048  0  0  0.695  0.17  0.498  0  0.083  0  0.308  0.058  0.344  0.084  0.057  0.584  0.298  0.475  0  0.042  0.049  0  0.052  0.253  0  0.369  0.043  0.493  0.059  0.296  0.129  0.669  0  1.489  0  0.779  0.024  0.079  0  0.073  0.043  0  0  0  
 
 TMEM108-AS1  101927455  3  132965291  132975965  -  0  0  0.234  0  0  0  0  0  0.192  0.222  0.171  0  0  0  0  0  0  0  0  0  0  0.036  0.322  0  0  0  0.07  0  0  0.028  0  0  0.054  0.313  0  0  0  0  0.027  0  0  0  0  0  0  0  0.032  0.101  0  0.125  0.074  0.098  0  0  0  0  0  0  0  0  
 
 BFSP2  8419  3  133118838  133194056  3q22.1  0  0  0.125  0  1.933  0  0  0.056  0  0  0  0  0  0.037  0  0  0  0.068  0.066  0.078  0  0.186  0.396  0.193  0  0  0.041  0.095  0  0.057  0.2  0  0  0.397  0  0  0  0  0.053  0  0  0.145  0.031  0  0  0.072  0.048  0  0  0  0  0.42  0  0  0  0  0  0  0  0  
 
 CDV3  55573  3  133292433  133309118  3q22.1  3.666  4.036  3.805  4.521  6.272  3.27  4.395  5.086  3.924  3.852  4.447  4.01  4.259  4.821  3.746  4.017  4.182  3.346  5.496  4.133  3.603  3.935  5.264  4.503  4.832  3.619  3.623  3.733  3.735  3.214  4.484  5.009  4.22  3.638  3.004  3.907  4.64  4.447  3.935  4.356  3.758  3.261  4.741  4.528  4.153  4.394  3.016  4.31  4.39  6.35  4.134  3.312  4.693  3.817  4.464  4.272  5.168  4.468  5.178  5.385  
 
 TOPBP1  11073  3  133319448  133380737  3q22.1  2.464  2.653  1.319  1.742  2.47  1.346  2.609  3.111  1.13  2.408  2.699  2.171  2.717  2.92  2.433  2.322  2.223  2.598  4.125  2.8  2.131  2.878  1.809  1.818  2.173  2.431  2.106  2.795  2.344  2.045  2.461  2.909  3.343  1.955  1.718  2.303  2.611  2.63  1.663  3.399  2.264  1.541  2.663  2.429  1.979  2.422  1.096  1.939  2.241  3.229  2.003  1.391  2.155  1.772  1.827  2.112  2.957  2.087  2.244  2.837  
 
 TF  7018  3  133464976  133497850  3q22.1  0  0  0.087  0  0.032  0.108  0  0.033  0.083  3.355  0.042  0  0  0.076  0  0  0  0  0  0  0.115  0.11  0.257  0  0  0  1.931  0.141  0.144  0.065  0.57  0.936  0  0.222  0  0.158  0  0  0.172  0  0  0.109  0.037  0  0  0.058  0.033  0  0  0.032  0.03  0.166  0.03  0.12  0  0  0.059  0  0  0  
 
 SRPRB  58477  3  133502876  133540336  3q22.1  2.999  2.189  3.262  3.177  4.077  1.849  4.648  4.196  3.184  3.669  3.35  3.544  3.525  4.004  2.783  2.613  2.313  1.7  4.381  4.331  2.315  3.29  4.532  3.746  2.918  2.842  3.512  2.706  3.404  2.722  3.826  5.226  3.962  2.907  2.289  3.409  2.937  2.914  3.841  3.64  2.763  2.433  2.996  2.529  2.696  3.146  3.594  2.74  2.071  4.728  3.4  1.596  3.316  2.649  2.451  3.171  3.639  2.527  2.922  3.455  
 
 RAB6B  51560  3  133543079  133614691  3q22.1  0.463  0.089  1.066  0.519  0.975  0.564  0.561  0.843  0.781  1.187  0.673  0.151  0.188  0.883  0.474  0.04  0.12  0.033  0.271  0.464  0.689  0.362  0.725  0.177  0.236  0.108  1.66  0.877  1.136  2.329  1.518  1.717  1.424  1.032  0.186  1.214  0.714  0.716  1.077  1.686  0.125  0.33  1.324  0.77  1.28  0.56  0.569  1.033  0.361  2.506  0.151  0.766  0.527  0.494  0.554  0.129  1.759  0.759  0.46  0.31  
 
 C3orf36  80111  3  133646989  133648656  3q22.1  0  0  0.376  0  0  0  0  0  0.154  0.161  0.07  0.1  0  0.044  0  0  0  0.072  0  0.088  0  0.091  0.444  0  0  0  0.105  0  0  0.107  0  0  0  0.365  0  0.068  0  0.361  0.088  0  0  0.342  0.052  0  0  0.043  0.05  0  0  0  0  0.203  0  0.09  0.061  0  0.336  0  0  0  
 
 SLCO2A1  6578  3  133651539  133748920  3q21  0.305  0.041  0.775  0.7  0.506  0.055  1.755  0.337  0.147  2.039  0.473  0  0  0.083  0.036  0.024  0.037  0.094  0.048  0.068  0  0.21  0.381  0.037  0.027  0.022  0.12  0.037  0.035  0.103  0  0.052  0  0.3  0  1.962  1.319  2.544  0.432  0.315  0.035  0.135  0.146  0  0.435  0.343  0.356  0.077  0  1.306  0.025  1.138  1.332  0.125  1.97  0.039  3.171  0.076  0  0  
 
 RYK  6259  3  133875977  133969586  3q22  2.014  2.958  2.784  2.275  2.982  1.808  2.792  3.598  2.625  3.125  3.087  2.334  2.558  3.28  2.432  2.689  2.758  2.488  3.115  2.516  1.3  2.06  1.205  1.223  2.761  2.695  2.63  2.731  3.516  2.079  3.291  3.764  3.545  2.172  1.608  2.815  3.012  3.158  2.568  3.363  2.77  1.712  2.448  2.496  3.734  2.985  1.61  2.408  3.013  3.996  2.718  1.578  2.47  1.498  2.442  2.746  3.264  2.551  2.657  3.676  
 
 AMOTL2  51421  3  134074186  134094259  3q21-q22  1.92  2.633  3.439  3.458  2.997  3.431  2.405  4.617  3.344  3.163  3.952  0.536  1.519  2.759  3.759  2.48  1.349  0.835  0.36  0.104  0.137  0.436  0.494  0.103  3.528  1.691  2.001  4.322  1.535  0.874  0.607  1.522  3.289  1.765  2.569  3.292  3.915  3.691  3.234  3.988  3.322  1.993  3.183  4.035  5.201  4.73  1.8  5.505  3.89  6.125  2.039  3.619  3.403  3.651  4.35  4.112  4.296  4.787  4.447  5.059  
 
 ANAPC13  25847  3  134196545  134204865  3q22.2  3.522  5.584  2.752  2.931  4.348  1.908  4.277  4.352  3.314  4.211  3.592  3.105  3.176  4.524  2.441  3.331  3.428  6.103  4.892  3.834  3.238  3.412  3.528  2.75  4.375  3.027  3.578  3.378  3.187  1.723  3.816  5.137  4.552  4.468  2.483  3.489  3.477  4.544  3.876  3.311  3.212  2.812  4.596  3.48  2.372  4.034  2.773  2.749  2.804  5.604  3.414  1.71  2.338  3.026  4  3.513  3.614  3.064  3.359  4.591  
 
 CEP63  80254  3  134204574  134293855  3q22.2  1.681  2.73  1.138  0.634  1.75  0.618  1.854  1.637  1.094  1.643  1.591  1.096  1.364  1.852  0.844  0.862  0.958  2.926  3.461  1.695  0.651  1.957  1.473  1.197  1.178  1.622  1.288  1.711  1.294  1.175  2.104  1.901  2.149  1.367  1.195  1.577  1.522  1.798  1.165  1.543  1.245  0.761  1.118  0.992  0.727  1.24  1.01  1.113  1.309  2.276  1.655  0.712  1.601  0.913  1.64  1.109  2.214  1.111  1.343  2.251  
 
 KY  339855  3  134318764  134369864  3q22.2  0.042  0.029  0.274  0  0.042  0  0.118  0.046  0.097  0.707  0.034  0.064  0  0.029  0  0.044  0.078  0.145  0.022  0.087  0  0.158  0.481  0  0.035  0.016  0.098  0.066  0  0.071  0.033  0.024  0  0.313  0.044  0  0.042  0.068  0.069  0  0.028  0.424  0.06  0  0  0.075  0.02  0  0  0.015  0.025  0.443  0.878  0.067  0.067  0.049  0.732  0  0  0.024  
 
 EPHB1  2047  3  134514098  134979307  3q21-q23  0.221  0  0.293  0.058  0.031  0.24  0.448  0.051  0.154  0.395  0.044  0.212  0  0.061  0  0.024  0  0.173  0.021  0.08  0  0.147  0.345  0  0.118  0  0.108  0.296  0.803  0.084  0.195  1.658  0  0.303  0.063  0.034  0  0  0.095  0.117  0.025  0.257  0.79  0  0.472  0.371  0.84  1.149  0.333  1.173  0.033  0.383  1.035  0.128  2.448  0.042  1.872  0  0  0.428  
 
 PPP2R3A  5523  3  135684514  135866752  3q22.1  1.196  2.429  1.169  1.076  1.611  0.775  1.452  2.606  1.122  1.828  2.144  0.107  0.198  1.31  0.565  0.723  0.189  0.235  0.481  0.188  0.097  0.492  0.228  0.047  0.956  1.889  1.482  1.972  1.471  1.092  1.028  1.677  1.656  1.19  0.338  0.501  0.85  2.235  0.967  0.925  0.684  0.612  1.671  1.046  1.486  2.508  1.075  1.165  1.937  2.001  1.926  0.757  2.4  1.374  1.906  1.947  3.144  1.392  2.648  2.54  
 
 MSL2  55167  3  135867759  135914688  3q22.3  2.089  1.546  1.02  1.378  1.626  0.662  1.14  1.897  1.283  1.493  1.699  1.353  1.568  1.863  1.919  1.485  1.551  1.381  3.243  2.205  1.361  3.06  1.269  1.123  1.303  1.353  1.145  1.557  1.368  0.789  1.232  1.572  1.895  1.022  0.68  1.152  1.552  1.704  1.615  2.183  1.025  0.874  1.634  1.422  1.484  1.602  0.199  1.424  1.87  1.52  1.607  1.102  1.388  1.03  1.294  1.417  1.943  1.308  1.428  1.853  
 
 PCCB  5096  3  135969166  136049013  3q21-q22  4.335  2.459  2.428  2.155  4.117  1.603  3.656  3.012  1.951  2.964  3.259  2.853  3.695  3.997  3.434  3.076  3.48  2.306  5.294  3.926  3.744  4.212  2.396  2.259  2.139  3.116  2.982  3.38  3.566  3.511  3.426  4.803  3.419  1.857  2.072  2.36  3.086  3.052  2.552  4.081  2.593  2.109  3.576  2.873  2.764  3.318  2.998  3.356  2.668  5.825  2.903  2.406  3.266  1.583  3.463  2.909  3.237  2.824  2.839  3.722  
 
 STAG1  10274  3  136055998  136471245  3q22.3  2.229  2.519  1.099  0.752  1.681  1.209  1.642  2.196  1.309  1.78  2.409  1.399  2.039  2.377  1.914  1.616  1.264  2.14  3.118  1.129  0.971  1.591  0.972  1.169  1.601  1.78  1.304  1.92  1.45  0.914  1.489  1.655  2.108  0.943  0.844  1.629  2.353  1.848  1.36  1.76  1.249  1.129  2.107  1.896  1.376  1.535  1.028  1.336  1.598  2.607  1.458  0.887  1.369  1.168  1.164  1.081  2.608  1.579  1.651  1.945  
 
 SLC35G2  80723  3  136537860  136574734  3q22.3  0.28  3.014  1.754  1.191  0.167  0.699  3.742  3.71  1.161  1.904  2.193  0  0.103  1.091  0.184  0.723  0.154  2.56  3.192  0.098  0.409  0.856  0.455  0  0  0.265  0.212  1.304  0.149  1.55  0  3.647  0.152  0.395  0.97  0.733  2.616  3.052  2.553  1.387  0.769  1.217  3.257  0.345  0.461  0.869  1.823  1.007  1.288  3.147  1.237  1.245  2.014  0.128  2.332  1.763  2.38  1.041  1.99  1.52  
 
 NCK1-AS1  101927597  3  136560817  136580905  -  1.23  0.482  0.525  0.487  2.071  0.803  1.49  0.503  0.489  0.927  1.352  0.919  0.802  1.037  0.533  1.172  1.103  1.369  1.958  0.955  0.755  1.157  1.875  0.984  0.864  0.75  0.611  0.574  0.597  0.204  1.279  1.435  0.768  0.977  0.431  0.623  1.225  1.012  0.735  0.86  1.571  0.98  1.211  0.563  1.038  1.307  0.323  0.779  0.575  1.782  1.223  0.435  0.708  0.876  0.75  0.384  0.881  0.61  0.82  0.859  
 
 NCK1  4690  3  136581049  136670452  3q21  1.434  2.196  1.305  1.827  1.65  0.544  2.019  1.451  1.443  1.676  1.863  1.033  1.713  2.003  1.096  1.22  1.807  2.174  3.294  1.32  1.017  1.8  1.345  0.878  1.289  1.584  1.459  1.227  1.403  1.278  1.702  2.01  1.532  1.305  0.723  1.237  1.451  1.6  2.262  1.728  1.011  1.004  2.303  1.177  1.081  1.292  1.515  1.164  1.43  2.936  1.593  0.942  1.421  1.601  1.285  1.678  1.91  0.885  1.609  1.898  
 
 IL20RB  53833  3  136676706  136729926  3q22.3  0.703  0.301  0.39  1.583  0.554  0.067  1.676  0.535  0.618  0.348  0.949  0.417  0.325  1.12  0.257  0.47  0.134  1.43  1.488  0.296  0.393  1.113  0.493  0.321  0.169  0.072  0.924  0.431  0.211  0.164  0.55  1.004  0.438  0.342  0.105  0.443  1.503  0.751  2.946  0.43  0.408  0.653  0.921  0.177  0.256  0.458  0.537  0.391  0.719  0.956  0.412  0.298  0.717  2.041  0.312  0.72  0.736  0.216  0.264  0.159  
 
 SOX14  8403  3  137483133  137485172  3q22-q23  0  0.093  0.192  0  0.055  0  0  0.056  0.108  0.057  0  0.101  0  0.065  0  0.1  0  0.103  0  0.064  0  0.314  0.612  0  0  0  0.198  0.039  0  0.115  0  0  0  0.394  0.05  0.064  0  0  0.097  0  0  0.341  0.102  0  0.122  0.091  0.107  0  0  0  0.037  0.588  0.037  0.149  0.164  0  0  0  0  0  
 
 LOC100507274  100507274  3  137490752  137499719  -  0  0  0  0  0  0  0  0  0.154  0  0  0  0  0  0  0  0  0.082  0  0  0  0.052  0.186  0  0  0  0.058  0  0  0.086  0  0  0  0.281  0  0  0  0  0  0  0  0  0.036  0  0  0  0  0  0  0  0  0.145  0  0.052  0  0  0  0  0  0  
 
 CLDN18  51208  3  137717657  137752494  3q22.3  0  0  0.251  0  0.019  0.044  0.027  0.322  0.163  0.308  0.166  0.086  0  0.128  0.097  0.044  0.047  0.088  0.031  0.039  0  0.09  0.418  0  0  0.226  0.195  0.041  0.068  0.075  0.175  0.113  0.146  0.266  0.062  0.15  0.09  0.247  0.126  0  0  0.571  0.05  0.052  0  0.052  0.065  0.115  0.109  0  0.047  0.271  0.151  0.157  0.092  0.051  0.436  0  0.052  0  
 
 DZIP1L  199221  3  137780826  137834451  3q22.3  0  0.11  0.749  0.22  0.522  0.627  0.839  1.947  0.25  1.651  1.268  0  0  0.718  0.554  0.048  0.141  0.046  0.033  0.234  0.122  0.15  0.365  0.051  0.267  1.247  1.202  1.042  1.556  0.961  1.296  1.648  1.5  0.473  0.365  0.764  0.905  1.416  0.541  0.643  0.181  0.611  1.085  0.623  0.282  0.349  0.181  1.107  0.913  1.505  0.069  1.112  1.116  0.686  1.442  0.665  2.406  0.489  1.454  0.918  
 
 A4GNT  51146  3  137842559  137851229  3p14.3  0  0  0.375  0  0  0.127  0  0  0.302  0.094  0.046  0  0  0.037  0  0  0  0  0  0  0  0.061  0.318  0  0  0  0.069  0  0  0.043  0  0  0  0.432  0.164  0  0  0  0.06  0  0  0.047  0.042  0  0  0.041  0.031  0  0  0  0  0.132  0  0.042  0.177  0  0.095  0  0  0  
 
 DBR1  51163  3  137879829  137893791  3q22.3  1.857  1.415  1.265  1.485  2.612  0.921  2.196  1.904  1.441  1.869  2.645  1.932  2.047  2.208  1.341  1.616  1.795  1.887  3.626  2.481  1.246  2.458  1.323  1.036  1.625  2.155  2.018  1.663  1.96  1.473  2.037  2.949  2.624  1.928  0.969  1.697  1.964  1.812  1.98  2.314  1.398  1.084  2.543  1.661  1.579  1.832  1.456  1.562  1.441  3.311  1.567  1.387  1.701  1.07  1.494  1.602  2.12  1.399  2.054  2.484  
 
 ARMC8  25852  3  137906089  138017228  3q22.3  1.94  2.963  1.286  0.86  2.344  0.939  2.154  2.014  1.588  1.657  2.192  1.159  1.673  2.591  1.351  1.803  1.657  3.049  3.649  1.907  1.43  2.178  1.236  0.965  1.608  1.987  1.539  1.857  1.822  1.125  1.827  2.405  1.909  1.253  0.842  1.843  2.039  2.14  2.168  2.535  1.498  1.082  2.686  1.542  1.551  1.749  1.396  1.491  1.801  3.117  1.69  1.239  2.084  0.911  1.689  1.236  2.16  1.148  1.961  2.31  
 
 NME9  347736  3  137980278  138048728  3q22.3  0.151  0.081  0.224  0  0.21  0  0.165  0.222  0.102  0.243  0.235  0  0.228  0.17  0.255  0.183  0.081  0.213  0.267  0.097  0.153  0.123  0.406  0  0.054  0.254  0.367  0.258  0.19  0.413  0.296  0.17  0.198  0.448  0.192  0.154  0.16  0.408  0.103  0  0.089  0.321  0.448  0.18  0.074  0.201  0.041  0.333  0.233  0.229  0.133  0.343  0.176  0.03  0.127  0  0.439  0.191  0.145  0.093  
 
 MRAS  22808  3  138066489  138124377  3q22.3  0.301  0.59  2.149  0.669  1.214  0.909  1.511  3.497  1.519  1.914  0.674  0  0  1.462  0.182  0.028  0.059  0.245  0.026  0.072  0.683  0.145  0.75  0.116  0.118  2.609  3.255  2.054  1.52  0.615  0.713  2.135  0.521  0.709  0.447  2.791  0.922  2.285  0.689  1.507  0.219  0.299  4.002  1.206  1.143  1.762  0.042  1.398  0.815  2.414  0.05  0.681  2.052  1.271  1.315  1.091  3.384  0.743  1.91  2.075  
 
 ESYT3  83850  3  138153414  138197256  3q22.3  0.067  0.063  0.15  0  0.257  0.025  0.113  0.261  0.106  0.252  0.091  0.035  0.387  0.175  0.175  0.042  0.039  0.368  0.085  0.045  0  0.214  0.462  0.061  0  0.373  0.473  0.178  0.121  0.059  0.09  0.173  0.233  0.223  0.14  0.497  0.097  0.307  0.095  0.424  0.084  0.212  0.289  0.037  0.21  0.395  0.042  0.557  0.172  0.823  0.241  0.426  0.117  0.145  0.182  0.041  0.068  0.273  0.723  0.24  
 
 CEP70  80321  3  138213180  138313225  3q22.3  1.188  2.431  0.676  0.884  2.533  0.429  1.494  1.539  0.864  1.617  2.396  0.54  0.739  2.897  0.796  0.888  1.393  3.917  3.304  0.087  0.99  1.87  0.995  0.375  0.641  0.933  0.93  1.094  1.33  0.371  1.13  2.64  2.207  1.446  0.726  1.732  0.934  1.4  1.477  1.876  1.399  0.969  1.909  0.965  0.979  1.755  1.457  0.976  1.267  2.827  1.733  0.954  1.052  0.858  1.452  1.151  1.791  1.303  1.72  1.637  
 
 FAIM  55179  3  138327541  138352213  3q22.3  2.072  3.998  1.53  0.978  3.399  1.774  2.117  2.408  1.304  1.851  2.09  1.803  1.817  3.532  1.549  1.2  1.764  6.149  3.426  0.542  0.827  0.348  0.33  0  1.452  1.888  0.833  0.578  1.517  1.031  1.446  2.68  1.338  0.794  1.685  2.432  1.553  2.51  2.35  2.205  2.537  1.915  2.953  1.503  1.523  1.734  1.829  0.998  2.45  3.514  1.675  1.412  2.494  1.054  2.233  1.428  3.634  1.015  3.181  2.607  
 
 PIK3CB  5291  3  138371539  138478201  3q22.3  1.329  1.573  0.854  1.185  2.007  0.583  1.566  1.352  1.354  1.234  2.061  1.536  1.477  1.803  1.174  1.451  1.696  1.575  1.287  1.675  1.656  0.882  0.873  0.351  0.988  1.984  1.435  1.378  2.09  1.696  2.18  1.948  2.258  1.373  0.684  1.687  1.481  1.978  1.574  1.734  1.244  1.145  2.054  0.799  1.095  1.452  0.909  1.529  1.599  2.737  1.826  1.003  1.606  1.154  1.411  1.564  2.02  1.415  1.736  2.077  
 
 LINC01391  103344930  3  138654030  138662862  3q22.3  0.074  0  0.244  0  0.066  0.109  0.084  0.235  0.159  0.417  0.035  0.064  0  0.117  0.074  0.066  0  0.114  0.037  0.093  0.146  0.182  0.507  0  0  0.028  0.163  0.033  0.035  0.112  0  0.051  0  0.348  0.069  0.069  0.082  0.363  0.079  0.088  0.092  0.258  0.057  0.067  0.159  0.097  0.164  0.082  0  0.065  0  0.442  0.044  0.11  0.218  0.05  0.026  0.046  0.067  0.13  
 
 FOXL2  668  3  138663065  138665982  3q23  0.503  0.261  0.287  0.325  0.329  0.888  0.675  1.548  0.18  1.595  0.318  0.143  0.356  0.133  0.272  0.132  0.16  1.048  0.672  0.312  1.513  0.506  0.636  0.427  0.258  0.133  0.319  0.232  0.054  0.264  0.201  0.18  0.287  0.484  0.316  0.15  0.739  1.123  0.552  0.75  0.427  1.347  1.137  0.175  1.193  0.388  0.229  0.994  0  1.86  0.203  1.176  0.19  0.222  1.611  0.271  0.099  0.391  0.212  1.716  
 
 FOXL2NB  401089  3  138666075  138672830  3q22.3  0.104  0.023  0.217  0  0.029  0.363  0.426  0.695  0.154  1.109  0.256  0.029  0.066  0.287  0.076  0.031  0  1.583  0  0.034  0.98  0.055  0.42  0  0  0  0.098  0  0.03  0.074  0  0.026  0  0.417  0.126  0.054  0.254  1.223  0.033  0.409  0.096  0.365  2.199  0.146  0.393  0.154  0.097  0.241  0  1.043  0  0.233  0  0.051  0.868  0.108  0.096  0.15  0.13  1.775  
 
 PRR23A  729627  3  138722803  138725110  3q23  0  0  0.099  0  0.048  0.099  0.047  0.043  0.11  0.059  0  0.137  0  0.074  0  0.048  0  0.115  0  0.082  0  0.202  0.559  0  0  0  0.143  0.03  0  0.114  0  0  0  0.403  0.137  0  0  0  0.113  0  0.057  0.139  0.053  0  0  0.05  0.169  0  0  0  0  0.548  0.056  0.131  0.066  0.139  0  0  0  0  
 
 PRR23B  389151  3  138737872  138739768  3q23  0  0  0.104  0  0.076  0.134  0  0.082  0.144  0.106  0.036  0.085  0  0.105  0  0.122  0.13  0.152  0  0.098  0  0.447  0.582  0  0  0  0.179  0.045  0  0.152  0.079  0  0  0.212  0.077  0  0  0  0.082  0  0  0.515  0.084  0.08  0  0.085  0.059  0  0  0  0.046  0.8  0  0.201  0.175  0.07  0  0  0  0  
 
 PRR23C  389152  3  138760943  138763734  3q23  0  0.084  0.173  0  0.027  0.047  0.053  0.036  0.157  0.06  0  0  0  0.041  0  0.047  0.08  0.062  0.029  0.071  0  0.19  0.431  0  0  0.035  0.107  0.024  0  0.109  0  0  0  0.269  0  0  0  0  0.097  0  0  0.48  0.059  0  0  0.045  0.044  0  0.064  0  0.051  0.406  0  0.109  0.041  0.092  0  0.094  0  0  
 
 BPESC1  60467  3  138823026  138844005  3q23  0  0.028  0.111  0  0  0  0.029  0  0.103  0.028  0  0.026  0  0.052  0  0  0  0.041  0  0.029  0  0.045  0.381  0  0  0  0.074  0  0  0.054  0  0  0  0.404  0  0  0  0  0.032  0  0  0.05  0.043  0  0  0  0.068  0  0  0  0  0.37  0  0.033  0  0.037  0  0  0  0  
 
 PISRT1  140464  3  138951833  138952364  3q23  0  0  0  0  0  0  0  0  0  0  0  0  0  0.126  0  0  0  0  0  0  0  0  0  0  0  0  0  0  0  0  0  0  0  0  0  0  0  0  0  0  0  0  0  0  0  0  0  0  0  0  0  0.33  0  0  0  0  0  0  0  0  
 
 MRPS22  56945  3  139062860  139075887  3q23  3.441  5.463  2.478  2.754  4.536  2.394  3.796  3.711  2.42  3.288  3.907  3.868  3.317  5.544  2.579  3.684  3.918  6.159  5.22  4.517  4.436  3.122  2.93  2.114  3.382  3  2.89  3.011  2.993  2.796  4.129  4.764  4.336  3.227  2.498  2.711  3.089  3.927  3.904  3.73  3.025  2.763  3.847  3.087  3.309  3.812  2.926  3.092  2.711  5.708  3.342  2.663  3.24  1.867  3.6  3.127  3.676  3.539  3.312  3.786  
 
 COPB2  9276  3  139076432  139108522  3q23  3.729  4.508  3.673  3.257  4.85  2.463  4.878  5.27  3.347  4.575  4.92  3.738  4.031  4.659  3.483  3.419  3.577  4.21  4.893  4.2  3.421  2.801  3.035  2.894  3.363  4.294  3.543  3.934  4.411  3.474  4.632  5.66  4.555  3.059  2.431  3.943  4.134  4.718  4.309  4.64  3.594  2.423  4.224  3.57  3.33  4.54  2.402  3.159  3.731  5.177  4.352  2.543  3.841  3.289  3.375  4.282  4.976  3.336  3.817  4.952  
 
 LOC100507291  100507291  3  139108644  139302161  -  1.176  0.525  0  0  1.523  0.306  0.416  0.364  0.472  0.653  0.484  0.603  0.437  0.89  0.634  0.45  0.377  1.228  0.783  0.334  0  0.306  0.734  0.328  0  0  0.239  0.286  0  0.117  0.335  0.314  0  0.446  0.242  0  0.621  0  0.735  0.979  0.881  0.478  3.166  0  0  1.471  0.1  0  0.361  1.339  0.416  0.429  0.327  0.198  0.805  0.749  0.415  0  0  0.777  
 
 RBP2  5948  3  139171725  139195352  3q23  0  0  0  0  0.116  0  0  0  0  0.143  0  2.258  0  0  0  0  0  0  0  0  0  0  0  0  0  0  0.11  0  0  0  0  0  0  0  0  0  0  0  0  0  0  0  0  0  0  0  0  0  0  0  0  0  0  0  0  0  0  0  0  0  
 
 RBP1  5947  3  139236275  139258671  3q23  0  0  2.192  1.853  0.366  0.597  4.99  0.302  0.21  0.914  0  0  0  6.056  0  0  0  5.327  0.158  0.229  0  0.175  0.608  0  0  0  0.257  0.101  0  0.229  0  0.373  0  0.435  0.809  2.73  0.397  0.502  0.222  1.428  0  0.739  6.604  0.62  2.598  4.751  6.202  0.607  1.631  0.61  0  1.425  0  0.116  0.129  0.347  0.141  0  0  0.362  
 
 NMNAT3  349565  3  139279022  139396885  3q23  0.143  0.139  0.406  0  1.234  0.819  0.124  0.054  1.694  0.177  0.523  2.463  1.099  1.268  0.793  0.392  1.141  3.318  2.729  0.522  0.627  0.96  0.756  0.337  0.083  0  0.15  0  0  0.365  0.187  0.926  0  0.378  0.977  0.345  0.06  0.198  1.035  0.611  0.091  3.51  0.303  0.374  1.066  0.908  0.322  0.155  0  0.727  0.174  0.962  0.047  1.082  1.158  1.361  0.057  0.101  0.353  0.899  
 
 CLSTN2  64084  3  139654026  140286919  3q23  0  0  0.5  0  1.931  0.14  0.31  0.025  0.212  0.583  0.04  0.06  0  0.035  0  0.044  0.049  0.147  0  0.321  0  0.148  0.407  0.028  0  0  0.108  0.136  0.088  0.099  0.081  0.045  0.04  0.302  0.026  0  0.036  0  0.604  0.076  0.032  0.053  0.334  0.034  1.619  0.636  0.05  0.279  0  0.015  0.028  0.37  0.02  0.11  0.166  3.27  0.033  0  0  3.014  
 
 CLSTN2-AS1  101927808  3  140224459  140227631  3q23  0  0  0.144  0  0  0.122  0  0  0.088  0.02  0  0  0  0  0  0  0  0  0  0.04  0  0.059  0.331  0  0  0  0.078  0  0  0.033  0  0  0  0.213  0  0  0  0  0  0  0  0.152  0.019  0  0.095  0.061  0.021  0.312  0  0  0  0  0  0  0.03  0  0  0  0  0  
 
 TRIM42  287015  3  140396865  140419992  3q23  0  0.043  0.187  0  0.081  0  0  0  0.082  0.041  0  0.083  0  0.026  0  0  0  0.166  0  0.147  0  0.211  0.417  0  0  0  0.078  0  0  0.06  0  0  0  0.333  0  0  0  0  0.069  0  0  0.244  0.042  0  0  0.048  0  0.385  0  0  0.028  0.727  0  0.058  0.054  0  0  0  0  0  
 
 SLC25A36  55186  3  140660661  140698785  3q23  2.23  2.757  1.796  1.795  2.293  1.361  2.227  2.478  2.218  2.278  2.629  1.362  2.49  3.078  2.196  1.978  2.615  3.336  3.334  3.407  1.864  2.781  2.256  1.765  2.129  2.301  1.855  2.212  2.237  2.126  2.577  3.214  2.784  2.196  1.137  1.419  1.985  2.565  3.431  2.225  1.824  1.441  2.37  2.137  2.097  2.842  1.304  2.022  2.084  3.379  2.453  1.686  2.211  0.998  2.092  1.968  2.871  2.016  2.701  2.429  
 
 SPSB4  92369  3  140770243  140867453  3q23  0.271  0.079  0.229  0  0.188  0.215  0.073  0.13  0.475  0.161  0.052  0.091  0  0.142  0  0.077  0.057  0.247  1.128  0.096  0  1.298  0.536  0.09  0  0.033  0.205  0.036  0  0.237  0.064  0.124  0  0.422  0.049  0  0  0  0.121  0.28  0.064  0.591  1.965  0.159  0.155  0.108  0.073  1.444  0.187  1.455  0.032  1.192  0.025  0.164  0.092  0.073  0  0.074  0  0  
 
 PXYLP1  92370  3  140950666  141013745  3q23  0.846  0.286  0.635  1.183  1.09  0.243  0.58  1.311  1.139  1.069  0.831  1.399  0.876  1.714  0.839  0.53  0.725  0.455  3.143  0.109  0.136  1.615  0.313  0.224  0.309  1.014  0.8  1.519  0.991  0.137  0.468  1.821  0.847  0.68  0.45  0.909  0.62  1.644  0.676  0.84  0.554  0.374  0.772  0.901  0.695  1.525  0.073  0.524  1.271  1.463  0.116  0.723  0.034  0.291  0.202  0.565  0.561  0.421  0.703  0.681  
 
 ZBTB38  253461  3  141043054  141168632  3q23  1.28  3.268  2.666  1.528  2.288  1.219  2.779  3.96  1.994  2.335  2.999  1.343  2.087  1.887  1.323  1.401  1.98  1.954  0.1  0.324  1.256  0.149  3.159  4.003  2.901  2.993  2.315  2.664  2.112  2.57  2.763  3.402  3.072  2.091  2.264  2.663  2.5  3.908  4.347  1.882  1.59  1.964  1.79  1.26  0.907  2.175  1.144  1.872  1.954  2.459  1.266  2.327  3.026  1.948  3.49  2.048  4.549  2.76  1.955  3.231  
 
 RASA2  5922  3  141205888  141334205  3q22-q23  0.403  1.632  0.571  0.482  0.934  0.477  0.421  1.158  0.543  0.849  0.78  0.328  0.608  1.138  0.489  0.824  0.787  1.276  2.418  1.011  0.534  1.115  0.979  0.299  0.878  1.417  0.424  0.8  0.811  0.316  0.658  0.865  0.803  0.501  0.406  0.836  0.557  0.874  1.421  1.245  0.575  0.703  0.95  1.05  0.581  1.307  0.849  0.44  1.21  0.988  0.754  0.583  0.539  0.291  0.584  0.718  1.175  0.63  1.043  1.08  
 
 RNF7  9616  3  141457050  141465645  3q22-q24  3.325  5.279  3.413  2.887  4.592  2.823  4.652  4.272  3.288  3.431  4.567  3.128  3.088  4.659  1.9  3.581  3.95  5.059  4.996  3.733  3.312  3.374  3.305  2.705  3.518  3.036  3.498  3.165  3.421  2.432  4.001  4.967  3.765  3.149  3.079  3.164  3.108  4.223  4.654  3.516  3.445  2.945  4.632  3.316  3.224  4.319  4.731  2.688  2.966  5.344  3.434  2.631  3.312  2.463  3.546  3.213  3.704  4.02  3.406  3.794  
 
 GRK7  131890  3  141497042  141535892  3q24  0.136  0  0.28  0  0.087  0  0  0  0.128  0.072  0.067  0  0  0.087  0  0.06  0  0.272  0  0.081  0  0.268  0.332  0  0  0  0.109  0  0  0.149  0  0  0  0.322  0  0  0.063  0  0.056  0  0  0.085  0.044  0  0  0.141  0.04  0  0  0  0.057  0.793  0  0.044  0.101  0  0  0  0  0  
 
 ATP1B3  483  3  141595469  141645382  3q23  4.119  6.445  4.17  5.431  5.213  3.354  6.042  4.915  4.578  5.223  5.406  4.083  5.013  6.084  4.045  4.162  5.141  5.984  6.413  5.693  4.117  5.228  4.607  4.131  4.372  4.843  5.621  5.603  5.567  4.161  6.11  7.005  5.633  4.464  3.594  4.525  5.407  5.595  4.794  6.688  4.029  4.268  5.638  4.713  3.373  5.183  5.58  3.798  4.304  5.887  4.28  3.438  5.062  3.003  4.421  4.475  4.978  4.371  4.958  5.219  
 
 TFDP2  7029  3  141663269  141868386  3q23  1.056  1.923  0.988  1.092  1.763  0.785  1.26  1.907  1.146  1.833  1.394  0.549  1.386  1.888  1.139  0.587  0.833  1.751  4.868  2.112  1.844  4.304  1.009  0.659  0.907  0.792  1.259  1.141  1.251  0.735  1.144  1.836  1.581  1.071  0.865  0.539  0.869  1.57  1.563  1.788  0.577  0.832  2.279  2.198  1.667  1.922  0.652  1.062  1.381  1.985  1.16  0.866  1.032  0.952  1.037  0.962  1.474  1.13  0.916  2.037  
 
 GK5  256356  3  141876368  141944449  3q23  0.649  0.573  0.716  0.519  2.022  0.459  0.804  1.321  0.565  1.104  1.198  0.687  1.25  0.798  0.845  1.018  0.967  0.614  1.887  0.594  0.806  0.955  0.695  0.374  0.374  0.91  0.651  0.502  0.532  0.455  1.255  1.249  1.047  0.584  0.472  0.838  0.873  0.876  0.627  1.201  1.014  0.465  1.117  1.017  0.668  1.546  0.248  0.324  1.084  0.633  0.715  0.531  0.898  0.41  0.584  0.635  0.955  0.665  0.919  0.993
[truncated: 7,693,721 more chars]
